# Conservation of ciliary proteins in plants with no cilia

**DOI:** 10.1186/1471-2229-11-185

**Published:** 2011-12-30

**Authors:** Matthew E Hodges, Bill Wickstead, Keith Gull, Jane A Langdale

**Affiliations:** 1 Department of Plant Sciences, University of Oxford, South Parks Rd, Oxford OX1 3RB, UK; 2 Centre for Genetics and Genomics, University of Nottingham, Nottingham NG7 2UH, UK; 3 Sir William Dunn School of Pathology, University of Oxford, South Parks Rd, Oxford OX1 3RE, UK

**Keywords:** Evolution, Cilia, Flagella, Basal Body, Centriole, Land Plants

## Abstract

**Background:**

Eukaryotic cilia are complex, highly conserved microtubule-based organelles with a broad phylogenetic distribution. Cilia were present in the last eukaryotic common ancestor and many proteins involved in cilia function have been conserved through eukaryotic diversification. However, cilia have also been lost multiple times in different lineages, with at least two losses occurring within the land plants. Whereas all non-seed plants produce cilia for motility of male gametes, some gymnosperms and all angiosperms lack cilia. During these evolutionary losses, proteins with ancestral ciliary functions may be lost or co-opted into different functions.

**Results:**

Here we identify a core set of proteins with an inferred ciliary function that are conserved in ciliated eukaryotic species. We interrogate this genomic dataset to identify proteins with a predicted ancestral ciliary role that have been maintained in non-ciliated land plants. In support of our prediction, we demonstrate that several of these proteins have a flagellar localisation in protozoan trypanosomes. The phylogenetic distribution of these genes within the land plants indicates evolutionary scenarios of either sub- or neo-functionalisation and expression data analysis shows that these genes are highly expressed in *Arabidopsis thaliana *pollen cells.

**Conclusions:**

A large number of proteins possess a phylogenetic ciliary profile indicative of ciliary function. Remarkably, many genes with an ancestral ciliary role are maintained in non-ciliated land plants. These proteins have been co-opted to perform novel functions, most likely before the loss of cilia, some of which appear related to the formation of the male gametes.

## Background

Centrioles and cilia/flagella are microtubule-based organelles involved in cellular motility and signalling (for review see [1]). The ultrastructural morphology of these ancient organelles is remarkably conserved in extant eukaryotes and their phylogenetic distribution pattern suggests that the last eukaryotic common ancestor (LECA) possessed the ability to produce a centriole plus cilia with both sensory and motility functions [2-4]. Despite this widespread phylogenetic distribution, lineage specific modifications have been shown to occur and numerous instances of independent cilia loss have been reported [5-9].

In recent years, multiple high-throughput proteomic studies and bioinformatic analyses in disparate species have identified a cohort of proteins associated with centriole and ciliary functions [2,3,10-16]. These proteins include both those with ciliary-specific roles (such as intraflagellar transport proteins, outer- and inner-dynein arms and radial spoke proteins) as well as those such as tubulins that are also involved in other cellular functions (for an extensive database see Cildb [17]). To date, however, little is known about what happens to genes involved in ciliary function when an evolutionary transition to cilia loss occurs.

The land plant lineage is a good model for studying the transition to ciliary loss for several reasons. First, this ancient monophyletic group was ancestrally ciliated, but there have been at least two independent loss events within the group, once in gymnosperms and once at the base of the angiosperms [18-20]. Second, sufficient genomic information exists for an in-depth analysis of protein compositional changes during the process of ciliary loss. Third, the land plants are a sister lineage to the Chlorophyta, a group that includes the well-studied ciliary model species *Chlamydomonas reinhardtii*. The Chlorophytes thus provide a good outgroup for identification and analysis of genes with ciliary function.

Within the ciliated basal land plants, cilia are produced only in specialised sperm cells [4]. Ciliogenesis in these cells occurs *de novo*, as opposed to the canonical template pathway seen in animal lineages [21]. It can thus be assumed that there are regulatory mechanisms that ensure correct spatial and temporal expression of genes required for ciliary function within this restricted phase of the plant life cycle. On losing the ability to produce cilia, it is unclear whether these regulatory mechanisms are also lost.

Here, we describe an approach that uses phylogenetic profiling to identify a core set of ciliary function proteins. By applying a scoring system to orthologous sets of proteins within 45 eukaryotic species we aimed to identify proteins with an ancestral ciliary function that are maintained in non-ciliated land plants. We then tested this ancestral ciliary function in the flagellated protozoan *Trypanosoma brucei*, and investigated potential plant roles through analysis of gene expression data and phylogenetic inference. We conclude that at least some of the genes identified in cilia formation in non-seed plants retain a role in male gametophyte development in seed plants.

## Results

### Identification of ciliary proteins by phylogenetic distribution

To gain a deeper understanding of the key proteins involved in ciliary function across eukaryotes, we analysed the distribution of orthologuos proteins encoded in the genomes of 45 species (Additional file 1). The species were selected to represent a wide evolutionary spread across six major groups of eukaryotes (namely: Archaeplastida, Excavata, Chromalveolata, Holozoa, Fungi and Amoebozoa). For each species, a complete or near-complete genome sequence is publically available. The species analysed include organisms that produce cilia/flagella at some point in their life cycle (which we refer to here as "ciliated species") and those that do not (referred to here as "non-ciliated species"). Proteins which are phylogenetically conserved in ciliated, but not in non-ciliated species, are considered to possess a "ciliary profile" and are predicted to perform a ciliary function.

To identify proteins with a ciliary profile, we performed a reciprocal best BLAST (RBB) analysis using the predicted proteome of *Chlamydomonas reinhardtii *to identify putative orthologues encoded in the genomes of 44 other eukaryotic species. The green alga *Chlamydomonas reinhardtii *was chosen as the query genome due to its phylogenetic position in the sister lineage to (at the base of) the land plants. The relatively short phylogenetic distance to the land plants, when compared to the spread of the eukaryotic phylogeny, reduces the chance that sequence divergence would prevent accurate identification of orthologues. Furthermore, *Chlamydomonas *is a well-studied ciliary model species.

The predicted proteome of *Chlamydomonas *has 15,143 proteins. Of these, 6,982 proteins have more than one putative orthologue. To reduce the noise of false positives in the dataset, only proteins with identifiable hits in at least 5 species within the analysis were retained. This cut-off resulted in a final defined dataset of 4,802 proteins (31.7% of the proteins in the *Chlamydomonas *predicted proteome) (Additional file 2). Within this dataset, 772 proteins (16.1%) have a putative orthologue in each of the major eukaryotic groups used in this analysis. The most parsimonious explanation for the phylogenetic distribution of these 772 proteins is that these proteins were present in the last eukaryotic common ancestor (LECA), and that they have subsequently been lost in certain lineages through evolutionary divergence.

**Figure 1:**
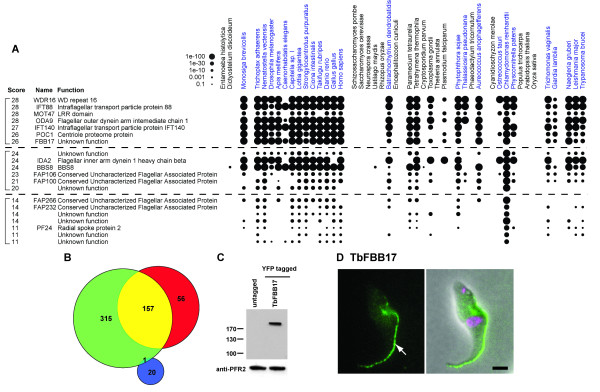
**A) Phylogenetic distribution of representative examples of the filtered ciliary profile proteins from the reciprocal best BLAST analysis with different scores**. Ciliated species are shown in blue. **B) **Venn diagram depicting the results of the bioinformatic analysis. A total of 4,802 proteins have five or more identifiable orthologues in 45 queried genomes. 472 of these proteins are detected in ciliary proteomic screens (green). 213 proteins are identified with the ciliary profile (red), of which 157 are detected in proteomic screens (yellow). 21 proteins have been identified as conserved in ciliated species and in the land plants (CCP, blue), of which one has been identified in proteomic screens. **C) **Western blot of whole cell lysates with anti-TY BB2 antibody (above) and loading control anti-PFR2 antibody (below). **D) **Direct fluorescence of whole cell *Trypanosoma brucei *showing cellular localisation of TbFBB17 (Tb10.406.0130) N-terminal YPF chimera (left panel) and an overlay of signal from YFP (green), DAPI (magenta) and phase-contrast (right panel). Tb10.406.0130 localises to the flagellum (white arrow). Scale bar = 2 μm.

We found that a simple presence or absence analysis of protein phylogenetic distribution in ciliated and non-ciliated species was too stringent to identify proteins with ciliary function because of false negatives. Therefore, we allowed for both false positives and negatives in the dataset by applying a positive score for a hit in a ciliated species, and a negative score for a hit in a non-ciliated species (see Methods). By using this scoring system we found that out of the 4,802 proteins in the dataset, 213 (4.4%, Additional file 3) proteins possess a score indicative of a ciliary profile (examples in Figure 1). As an indication that the method to identify ciliary proteins is accurate, 157 of these 213 (73.7%) proteins have been previously detected within cilia in proteomic analyses. This equates to 33.3% of all proteins (472) found in proteomic analyses of cilia. The remaining 56 proteins with a ciliary profile currently have no known function. We hypothesise that these represent a novel set of proteins with an as yet uncharacterised ciliary function.

### Ciliary profile proteins localise to the flagellum of trypanosomes

Most of the ciliary profile proteins have been detected in one or more proteomic studies of cilia [10,12,14,22]. To test whether the 56 proteins which have not been previously identified in proteomic studies are likely to play a role in cilia function, we investigated the cellular localisation of one of these proteins in the single-celled excavate *Trypanosoma brucei. T. brucei *is a notable model organism for cilia studies, with many examples of transgene introduction for localisation studies. Many of the ciliary profile proteins with known function have already been localised in *T. brucei *(for review see [23]).

We selected a candidate protein, FBB17, which has a putative trypanosome orthologue (herein called TbFBB17) encoded by the gene Tb10.406.0130, for endogenous locus *TY-YFP*-tagging in *T. brucei*. TbFBB17 is a putative zinc carboxypeptidase which has not been detected in any cilia proteomic study to date [10,12,14,17,22]. The tagging strategy ensured that the YFP-TbFBB17 chimera is transcribed by the endogenous gene promoter. This strategy has previously been shown to produce protein levels similar to wild-type for other loci [24]. The correct incorporation of the *YFP*-tag at the 5' end of the targeted Tb10.406.0130 gene was checked by PCR (data not shown) and by immunoblotting of cell lysates with monoclonal antibodies directed against the short epitope TY-tag [25](Figure 1).

To determine the intracellular localisation of TbFBB17, transformed cells were examined by fluorescence microscopy. Figure 1 shows that TbFBB17 is found along the length of the flagellum (white arrow). This observation supports the prediction that the identified ciliary profile proteins do indeed play a role in ciliary function. To determine whether TbFBB17 is stably associated with the cytoskeleton, we assessed the localisation of the tagged protein in cells which had been detergent-extracted to remove non-cytoskeletal components. In such cells the TbFBB17 flagellum signal is removed (data not shown). This explains the lack of identification of TbFBB17 in the trypanosome cilia proteome as the proteomes are derived from cytoskeletal cell fractions [5]. A similar explanation may apply to the other 55 proteins that have been identified in this study but not in ciliary proteomic screens.

### Identification of ciliary proteins in non-ciliated land plants and fungi

**Figure 2:**
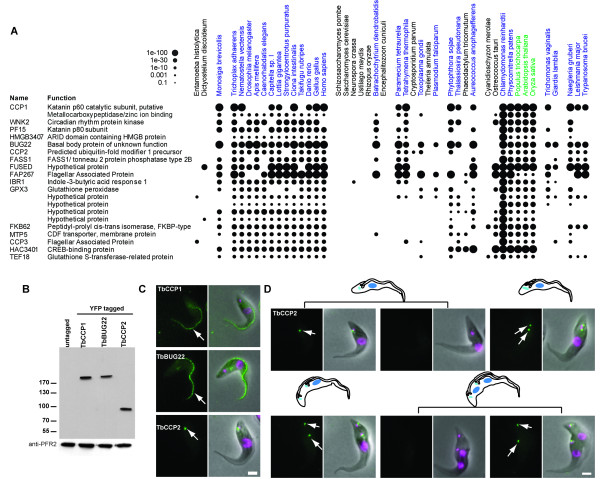
**A) Phylogenetic distribution of the filtered conserved in ciliated species and non-ciliated land plants, but absent in other non-ciliated species (CCP) proteins from the reciprocal best BLAST analysis**. Ciliated species are shown in blue and non-ciliated land plants in green. **B) **Western blot of whole cell lysates with anti-TY antibody (above) and loading control anti-PFR2 antibody (below). **C) **Direct fluorescence of whole cell *Trypanosoma brucei *showing cellular localisation of TbCCP1 (Tb11.02.1370), TbBUG22 (Tb10.70.5560) and TbCCP2 (Tb927.8.5380). Left panel showing fluorescence signal from YFP and an overlay in right panel of signal from YFP (green), DAPI (magenta) and phase-contrast. All three possess a ciliary localisation (white arrows point to flagella (CCP1 and BUG22) and basal bodies (CCP2)). **D) **Direct fluorescence of whole cell *Trypanosoma brucei *showing cellular localisation of TbCCP2 (Tb927.8.5380). Expression is cell-cycle regulated (white arrows depict basal bodies). Scale bar = 2 μm.

In land plants cilia have been lost at least twice, once in the gymnosperms and once at the base of the angiosperms [18-20]. Such evolutionary transitions pose the question of what happens to ancestral ciliary proteins when the ability to produce cilia is lost. To investigate this phenomenon, a scoring system was defined that identified proteins with putative orthologues conserved in both ciliated species and also land plants, but not in other non-ciliated species (CCP, for conserved in ciliated and plants, see Methods). This analysis identified 21 CCP proteins, out of the dataset of 4,802 proteins (0.44%), that have been retained in land plants which do not possess the ability to produce a cilium (Figure 2, Additional file 4). In agreement with our predictions, one of these proteins (BUG22) has previously been detected in ciliary proteomic studies [12,26]. The remaining proteins have no characterised ciliary function. A similar analysis within the non-ciliated fungal lineages (present in ciliated species and non-ciliated fungi) identifies two proteins (0.04% of the 4,802 proteins in the dataset). Neither of these two proteins are present in the CCP set, and neither have been detected in any proteomic study. The lower number of proteins identified in the fungal analysis may reflect the high level of genomic streamlining in this lineage [27].

### CCP proteins localise to the trypanosome flagellum

To test the hypothesis that CCP proteins possess an ancestral ciliary function, localisation of three proteins from this subset was examined in *T. brucei *cells. These three proteins, named TbCCP1 (the putative p60 subunit of katanin), TbBUG22 (the trypanosome orthologue of BUG22), and TbCCP2 (a proteins of hypothetical function) are encoded by genes Tb11.02.1370, Tb10.70.5560 and Tb927.8.5380, respectively. Orthologues of CCP1 and CCP2 have not been detected in any previous ciliary proteomic study, whereas BUG22 has been identified in a proteomic analysis of *Chlamydomonas *cilia [12]. The localisation of each protein was investigated using endogenous locus N-terminal *TY-YFP *tagging as for TbFBB17. Correct insertion of the tag was checked by PCR (data not shown) and immunoblotting of cell lysates with monoclonal antibodies directed against the TY tag (Figure 2).

Fluorescence microscopy of transformed cells demonstrated that all three proteins have a localisation pattern consistent with a role in ciliary function. Both TbCCP1 and TbBUG22 localise to the flagellum of *T. brucei *throughout the cell cycle (Figure 2, white arrows). Interestingly, TbCCP2 was found to localise to a position consistent with the basal body of the flagellum in a cell-cycle regulated fashion (Figure 2, white arrows). Trypanosomes undergo morphological changes which can be used to position cells within the cell cycle (see cartoons in Figure 2). Early in the cell cycle, the pro-basal body matures to form a second basal body from which a new flagellum will extend. Shortly thereafter, the kinetoplast (an organelle containing all the mitochondrial DNA) divides, coupled to the separation of the two basal bodies and extension of a new flagellum from the posterior basal body. Once the new flagellum is fully extended, nuclear mitosis occurs and finally cytokinesis. Examination of TbCCP2 fluorescence in the light of this positional information reveals that TbCCP2 is not detected just before kinetoplast division, nor during nuclear division, but it is present on or near the basal bodies throughout the rest of the cell cycle (Figure 2). In conclusion, these data demonstrate that the identified CCP proteins do indeed perform a ciliary function in a species which can produce flagella.

### Evolutionary history of CCP proteins

**Figure 3:**
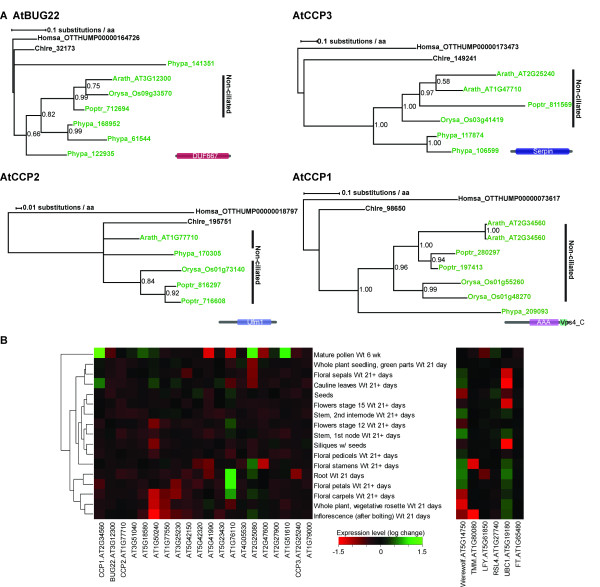
**A) Phylogenetic Bayesian inferences for four of the CCP proteins, demonstrating different evolutionary scenarios**. Land plants in green. For each protein, Pfam domain structure of human orthologue is shown. **B) **Heatmap illustration of microarray expression data for the CCP proteins (left) and controls (right) in different developmental stages scored by fold change from median.

To investigate possible functions of the CCP proteins in non-ciliated land plants we performed phylogenetic analysis of the identified protein sets. Understanding the evolutionary relationships of the CCP proteins provides insight into possible mechanisms of neo- or subfunctionalisation. Bayesian phylogenetic inference of the identified homologous proteins of BUG22 (Figure 3) suggest that the gene duplicated at the base of the land plants because *Physcomitrella patens *a basal land plant with the ability to produce cilia, possesses four copies of the gene that resolve in 2 clades. Subfunctionalisation may then have occurred with one copy retaining the ancestral ciliary role and the other being co-opted for alternative roles. Subsequently the ancestral copy was lost within the non-ciliated land plants (represented by *Arabidopsis thaliana, Oryza sativa *and *Populus trichocarpa*). In contrast, the Bayesian phylogenies of the other CCP orthologues are consistent with the hypothesis of neofunctionalisation/co-option of an ancestral ciliary protein upon the evolutionary transition to loss of ability to produce cilia because the genes resolve into a single land plant clade (Figure 3).

### CCP proteins are highly expressed in male floral organs and mature pollen

While the CCP proteins have a ciliary role in ciliated species, the question of what their function is within the non-ciliated land plants remains. To this end, we analysed microarray data mined from a public database of the non-ciliated flowering plant *Arabidopsis thaliana *[28]. The expression pattern of each of the genes at different developmental stages is shown in Figure 3, (expression data in Additional file 5). Clustering of the CCP expression data by fold change shows that the genes are highly expressed in mature pollen, with an average fold increase of 2.8. In contrast, the expression pattern of a number of control genes shows a more ubiquitous expression pattern. We performed a Monte Carlo estimation analysis to calculate the sum fold change of pollen expression data for 22,640 random sets of 21 genes within the *Arabidopsis *genome to determine how likely such a fold increase in expression is. This showed that pollen expression levels of the CCP proteins was very highly significant (*p *= 0.0006).

## Discussion

Over 300 proteins have previously been associated with ciliary function (for example see [17]) with most being identified through proteomic analysis of isolated cilia (e.g. [10,12,14,15]). However, such studies fail to detect proteins which are not stably associated with the protein preparation used. Here, we have identified a novel set of 56 proteins possessing a ciliary profile, through phylogenetic distribution analysis and experimental validation in *T. brucei *(Figure 1). In addition we identified 21 proteins with a predicted ancestral ciliary role that have been maintained in non-ciliated land plants (Figures 2, 3).

### Flowering plants have retained CCP proteins despite loss of ability to produce cilia

The phylogenetic distribution of cilia in land plants shows that cilia loss occurred at least twice: once in gymnosperms at the divergence of Ginkgo and the Cycads from the Conifers and Gnetals and once at the base of the flowering plants [18,19]. All other land plants produce cilia only within specialised sperm cells ([29-32], reviewed in [20]). During the evolutionary transition to loss of cilia, many ciliary function proteins were also lost (Additional file 3). However, we have identified 21 proteins with identifiable orthologues that are conserved in ciliated species and in the land plants (CCP proteins). At least a subset of these proteins possess a ciliary function in the flagellated *Trypanosoma brucei*, but the function of orthologues within the non-ciliated land plants has presumably diverged. Interestingly, a similar analysis for those proteins conserved during the evolutionary loss of cilia in fungal lineages identifies only two proteins, neither of which is present in the CCP set. Such low (21 and 2) numbers suggest that while loss of proteins is widespread during evolutionary transitions, it is relatively rare for a protein to be co-opted to a different function without undergoing significant sequence divergence.

The flagella localisation of all three tested CCP proteins in trypanosomes (Figure 2) supports their hypothesised ancestral ciliary role and validates the scoring system that we used for CCP identification. While there is limited data on the putative roles of the 21 CCP proteins in non-ciliated plants, it is of note that where information is available they are generally associated with cytoskeletal/microtubule roles. For example, CCP1 is a putative katanin, a class of known AAA+ microtubule-severing proteins [33] and when mutated the CCP gene *tonneau2/fass1 *markedly affects cell shape and arrangement in *Arabidopsis thaliana*, due to cytoskeletal abnormalities [34]. Another CCP protein, FUSED, has been shown to be involved in the hedgehog signalling pathway in *Drosophila*, and hedgehog receptors localise to the cilium in many species [35]. Together, these observations suggest that CCP proteins play regulatory roles that were ancestrally associated with cilia, but are now maintained for cytoskeletal functions in non-ciliated plants. Examination of the CCP proteins for Pfam domains [36] which include ubiquitanases, AAA+ domain containing proteins, protein kinases, tubulin-tryosine ligases and phosphatidic acid phosphatases, supports the suggestion that ancestral ciliary function proteins have been co-opted in land plants to perform cytoskeletal roles. Picket-Heaps [37] proposed an autogenous origin of cilia from a microtubule-organising centre ancestrally involved in the regulation of cytoplasmic and mitotic microtubules [4]. If this was the case, it is equally possible that upon loss of cilia evolutionary pressures drove the co-option of cytoskeletal functions in the opposite direction.

CCP protein function can also be inferred from the observation that CCP genes are highly expressed within mature pollen [28] (Figure 3, Additional file 5). This expression pattern is curious given the ancestral role of these proteins in ciliary formation in the sperm cells of basal land plants and the fact that sperm cells in pollen are not ciliated. It is conceivable that genes involved in the cell-specific formation of cilia in basal land plants may have acquired additional (new) functions in gametes before the loss of cilia, and are thus maintained in pollen on loss of cilia to perform similar cell-specific (possibly cytoskeletal) roles in a non-ciliary context. Such specificity would explain why only a small number of ciliary function genes were maintained or co-opted on the evolutionary transition to loss of cilia in seed plants, whereas the majority were lost or diverged.

## Conclusions

The work presented here demonstrates the existence of a set of ancestral ciliary proteins within non-ciliated land plants, and suggests that the process of cilia loss can be accompanied by the co-option of ciliary function proteins into novel contexts.

## Methods

### Bioinformatic definition of protein sets

Every predicted protein encoded in the *Chlamydomonas *genome was used as a query in a reciprocal best BLAST (RBB--see below) search with an e-value threshold of < 10^-5 ^against each of the predicted proteomes of each of 45 eukaryotic organisms. During RBB, a BLAST search is performed using a query sequence against a target genome. If the top hit from the target genome identifies the query sequence in the reciprocal BLAST, the sequence identified is a RBB hit. The species used in the analysis have a broad phylogenetic distribution and include 29 organisms which produce centriole/cilia/flagella at some point in their life-cycle (termed "ciliated") and 16 organisms which do not (termed "non-ciliated"). Importantly, each of these species possess a complete or near-complete genome sequence which is publically available. If the top hit from the RBB was the initial query sequence from *Chlamydomonas*, the identified target sequence was inferred to be an orthologue and was thus included in the dataset. Non-reciprocating sequences were discarded. The ciliary profile proteins were determined as those proteins whose evolutionary distribution of orthologues within the 45 species scored above 10 for a metric of *β - 10α*, (where *α *was the number of non-ciliated species possessing an orthologue and *β *was the number of ciliated species possessing an orthologue). The conserved in ciliated species and plants (CCP) protein set was defined as proteins with plant orthologues where *β > 18 *and *μ < 2*, (where *μ *was the number of non-ciliated species, excluding plant species, possessing an orthologue).

### Phylogenetic inference

For phylogenetic analysis, the sequences of each protein set were aligned using MAFFT6.24 [38] adopting the L-INS-I strategy, and trimmed by eye to well aligned blocks. A Bayesian phylogeny was inferred from the protein alignment using the metropolis-coupled Markov chain Monte Carlo (MCMCMC) method as implemented in the program MrBayes3.1.2, with 2 independent runs from random start trees using the WAG substitution matrix with a gamma-distributed variation in substitution rate approximated to 4 discrete categories [39].

### Endogenous-locus tagging

Endogenous-locus tagging was performed using procyclic-form *Trypanosoma brucei *Lister 427 cells [40]. Cells were maintained as axenic cultures in SDM79 supplemented with 10% v/v foetal calf serum (FCS) at 28°C, as described by Brun and Schӧnenberger [41]. Sequences were integrated at endogenous gene loci following the strategy outlined in [42]. Briefly, genomic DNA for PCR was isolated from strain Lister 427 *T. brucei *procyclic cells as in [43]. PCR amplicons containing ~200 bp from the 5'end of the target cDNA sequence (CDS) with a linearisation site added to the 5' end of the 3' primer, and ~200 from the 3'end of the 5' intergenic region for the target CDS with the same linearization site incorporated into the 5' primer for this fragment, were cloned together into the XbaI-BamHI sites downstream of *TY-YFP *in the pEnT6P vector, such that the N-terminal end of the CDS was in frame with *YFP*. The vector was then linearised at the site between the 5'end of the CDS and intergenic region fragments prior to transfection into procyclic-form Lister 427 *T. brucei *cells as described previously [44]. Post-electroporation, cells were allowed to recover in normal growth media for 14 h, after which time a stable transformant population was selected by the addition of 1 μg ml^-1 ^puromycin.

### Fluorescence microscopy

For analysis of YFP-tagged proteins in fixed whole cells, procyclic trypanosomes were harvested from mid-log phase cultures by centrifugation, washed once in phosphate buffered saline (PBS) and allowed to adhere to ethanol-washed plain glass slides for 5 min in PBS at ~2 × 10^7 ^cells ml^-1^. Cells were then fixed for 5 min in 2% w/v formaldehyde in PBS, followed by permeabilisation in -20°C methanol for 10 min and rehydrated in PBS. Samples were mounted in 1% w/v 1,4-Diazabicyclo[2.2.2]octane, 90% glycerol, 50 mM sodium phosphate pH 8.0 containing 0.25 μg ml^-1 ^4',6-diamidino-2-phenylindole.

### Western blot analysis

For whole-cell protein samples, cells were harvested, washed in PBS buffer, pelleted and immediately resuspended in boiling Laemmli buffer (2% sodium dodecyl sulphate (SDS), 10% glycerol, 400 mM β-mercaptoethanol, 50 mM Tris-HCl pH 7.2). SDS-polyacrylamide gel electrophoresis and immersion transfer to nitrocellulose membrane were performed using standard techniques described elsewhere [43]. For immunoblotting, the membranes were blocked with 5% (w/v) skimmed milk powder in TBS-T (20 mM Tris-HCl, 500 mM NaCl, 0.05% Tween 20), labelled with anti-TY BB2 monoclonal antibody [25] in TBS-T/1% skimmed milk powder, and developed with horseradish peroxidase-conjugated anti-mouse immunoglobulins (Sigma). As a loading control, membranes were re-probed with anti-PFR2 (L8C4) monoclonal antibody and developed as above.

## Abbreviations

LECA: Last Eukaryotic Common Ancestor; BLAST: Basic Local Alignment Search Tool; RBB: Reciprocal Best BLAST; CCP: Conserved in Ciliated and Plants.

## Authors' contributions

MEH carried out the bioinformatic analysis and molecular experiments. MEH, BW, JAL and KG conceived the study and participated in its design and coordination and helped to draft the manuscript. All authors read and approved the final manuscript.

## Supplementary Material

Additional file 1 **Source and version of the genomes used in the analysis**. Click here for file

Additional file 2 **Protein information and sequence identification for orthologous sets of each protein identified in the bioinformatic screen**. Protein information shown is the annotation from the JGI database for Chlamydomonas reinhardtii. Apime = *Apis mellifera*, Cioin = *Ciona intestinalis*, Caeel = *Caenorhabditis elegans*, Cyame = *Cyanidioschyzon merolae*, Capsp = *Capitella sp.*, Dicdi = *Dictyostelium discoideum*, Drome = *Drosophila melanogaster*, Danre = *Danio rerio*, Enccu = *Encephalitozoon cunculi*, Galga = *Gallus gallus*, Homsa = *Homo sapiens*, Lotgi = *Lottia gigantea*, Leima = *Leishmania major*, Monbr = *Monosiga brevicollis*, Neucr = *Neurospora crassa*, Naegr = *Naegleria gruberi*, Nemve = *Nematostella vectensis*, Ostta = *Ostreococcus tauri*, Parte = *Paramecium tetraurelia*, Sacce = *Saccharomyces cerevisiae*, Schpo = *Schizosaccharomyces pombe*, Strpu = *Strongylocentrotus purparatus*, Triad = *Trichoplax adhaerens*, Trybr = *Trypanosoma brucei*, Tetth = *Tetrahymena thermophila*, Takru = *Takifugu rubripes*, Ustma = *Ustilago maydis*. Click here for file

Additional file 3 **Protein information and sequence identification for ciliary profile proteins**. Click here for file

Additional file 4 **Protein information and sequence identification for CCP proteins**. Click here for file

Additional file 5 **mRNA Expression data for Arabidopsis thaliana mined from and used in analysis of CCP protein functions**. Click here for file
