# Supplementary material for: Conservation of ciliary proteins in plants with no cilia
Source: BMC Plant Biol. 2011 Dec 30;11:185. doi: 10.1186/1471-2229-11-185 (PMC3268115; doi:10.1186/1471-2229-11-185)
Supplement: Additional file 1 — Source and version of the genomes used in the analysis. [file 1471-2229-11-185-S1.PDF]

# Additional file 1 - genome source

| Species | Source | Version | Web reference |
|---|---|---|---|
| *Apis mellifera* | beebase | v2 (prerelease) | racerx00.tamu.edu |
| *Arabidopsis thaliana* | TAIR | TAIR7 | www.arabidopsis.org/ |
| *Aspergillus nidulans* | Broad Institute | v1.0 | www.broad.mit.edu/annotation/fgi/ |
| *Aureococcus anophagefferens* | JGI | v1.0 | www.jgi.doe.gov/ |
| *Batrachochytrium dendrobatidis* | Broad Institute | v1.0 | www.broad.mit.edu/annotation/fgi/ |
| *Caenorhabdatis elegans* | WormBase | WS170 | www.wormbase.org/ |
| *Capitella sp.* | JGI | v1.0 | www.jgi.doe.gov/ |
| *Chlamydomonas reinhardtii* | JGI | v3.1 | www.jgi.doe.gov/ |
| *Ciona intestinalis* | JGI | v2.0 | www.jgi.doe.gov/ |
| *Cryptosporidium parvum* | CryptoDB | v3.4 | www.cryptodb.org/ |
| *Cyanidioschyzon merolae* | C.merolae genome project | v1 | merolae.biol.s.u-tokyo.ac.jp/ |
| *Danio rerio* | VEGA | May 2007 | vega.sanger.ac.uk/ |
| *Dictyostelium discoideum* | dictyBase | 05-20-2007 | dictybase.org/ |
| *Drosophila melanogaster* | ENSEMBL | BDGP4.3, 44.43b | www.ebi.ac.uk/ensembl/ |
| *Encephalitozoon cuniculi* | NCBI | 1 | www.ncbi.nlm.nih.gov |
| *Entamoeba histolytica* | geneDB | 17102005 | www.genedb.org/ |
| *Gallus gallus* | ENSEMBL | WASHUC2, 44.2b | www.ebi.ac.uk/ensembl/ |
| *Giardia lamblia* | GiardiaDB | v1.0 | www.giardiadb.org/ |
| *Homo sapiens* | VEGA | Jun-07 | vega.sanger.ac.uk/ |
| *Leishmania major* | geneDB | v5.2 | www.genedb.org/ |
| *Lottia gigantea* | JGI | v1.0 | www.jgi.doe.gov/ |
| *Monosiga brevicollis* | JGI | v1.0 | www.jgi.doe.gov/ |
| *Naegleria gruberi* | JGI | v1.0 | www.jgi.doe.gov/ |
| *Nematostella vectensis* | JGI | v1.0 | www.jgi.doe.gov/ |
| *Neurospora crassa* | Broad Institute | v7.0 | www.broad.mit.edu/annotation/fgi/ |
| *Oryza sativa* | TIGR | v5.0 | www.tigr.org/tdb/e2k1/osa1/ |
| *Ostreococcus tauri* | JGI | v2.0 | www.jgi.doe.gov/ |
