# Supplementary material for: Conservation of ciliary proteins in plants with no cilia
Source: BMC Plant Biol. 2011 Dec 30;11:185. doi: 10.1186/1471-2229-11-185 (PMC3268115; doi:10.1186/1471-2229-11-185)
Supplement: Additional file 2 — Protein information and sequence identification for orthologous sets of each protein identified in the bioinformatic screen. Protein information shown is the annotation from the JGI database for Chlamydomonas reinhardtii. Apime = Apis mellifera, Cioin = Ciona intestinalis, Caeel = Caenorhabditis elegans, Cyame = Cyanidioschyzon merolae, Capsp = Capitella sp., Dicdi = Dictyostelium discoideum, Drome = Drosophila melanogaster, Danre = Danio rerio, Enccu = Encephalitozoon cunculi, Galga = Gallus gallus, Homsa = Homo sapiens, Lotgi = Lottia gigantea, Leima = Leishmania major, Monbr = Monosiga brevicollis, Neucr = Neurospora crassa, Naegr = Naegleria gruberi, Nemve = Nematostella vectensis, Ostta = Ostreococcus tauri, Parte = Paramecium tetraurelia, Sacce = Saccharomyces cerevisiae, Schpo = Schizosaccharomyces pombe, Strpu = Strongylocentrotus purparatus, Triad = Trichoplax adhaerens, Trybr = Trypanosoma brucei, Tetth = Tetrahymena thermophila, Takru = Takifugu rubripes, Ustma = Ustilago maydis. [file 1471-2229-11-185-S2.PDF]

Additional file 2 - protein information

Short	Description	Ciliary signature	Eukaryotic > 5	LECA	In proteomics	CCP	Fungal CCF equivlant	Apime
GOX10	putative glyoxal or galactose oxidase	-	-	-	-	-	-	-
GOX11	putative glyoxal or galactose oxidase	-	-	-	-	-	-	-
GOX12	putative glyoxal or galactose oxidase	-	-	-	-	-	-	-
GOX6	putative glyoxal or galactose oxidase	-	-	-	-	-	-	-
GOX9	putative glyoxal or galactose oxidase	-	-	-	-	-	-	-
GOX7	putative glyoxal or galactose oxidase	-	-	-	-	-	-	-
GOX8	putative glyoxal or galactose oxidase	-	+	-	-	-	-	-
FAP130	Flagellar Associated Protein	-	-	-	-	-	-	-
GOX4	putative glyoxal or galactose oxidase	-	-	-	-	-	-	-
GOX1	putative glyoxal or galactose oxidase	-	-	-	-	-	-	-
SOT2-pseudo	putative di or tri-carboxylate translocator	-	-	-	-	-	-	-
YEE1	conserved membrane protein	-	-	-	-	-	-	-
KIR1	putative ketoacid isomerase-related protein	-	-	-	-	-	-	-
		-	-	-	-	-	-	-
APG5	Autophagy protein	-	+	-	-	-	-	GB16402-PA
INDA	putative indigoidine synthase	-	-	-	-	-	-	-
ELG34	exostosin-like glycosyltransferase	-	-	-	-	-	-	-
		-	-	-	-	-	-	-
	protein kinase	-	-	-	-	-	-	-
	similar to monooxygenase, DBH-like 1	-	-	-	-	-	-	-
FLU	FLU chloroplast precursor	-	-	-	-	-	-	-
FAP45	Flagellar Associated Protein Weakly Similar to Nasopharyngeal Epithelium Specific Protein 1	+	+	-	+	-	-	GB14447-PA
FAP53	Flagellar Associated Protein	+	+	-	+	-	-	-
		-	-	-	-	-	-	-
SUFA	iron-sulfur cluster assembly protein	-	+	-	-	-	-	-
FAP147	Flagellar Associated Protein Weakly Similar to Myc-Binding Protein-Associated Protein	-	-	-	+	-	-	-
	Ser/Thr protein kinase	-	-	-	-	-	-	-
	eukaryotic signal peptide peptidase-like protein	-	+	-	-	-	-	-
NADK	ATP-NAD kinase	-	+	-	-	-	-	GB16468-PA
FTX1	frataxin	-	+	-	-	-	-	GB15590-PA
SUFS1	cysteine desulfurase (selenocysteine lyase)	-	+	-	-	-	-	-
SUFD	iron-sulfur cluster assembly protein	-	+	-	-	-	-	-
SUFB	iron-sulfur cluster assembly protein	-	+	-	-	-	-	-
ISCA	iron-sulfur cluster assembly protein	-	+	-	-	-	-	GB10611-PA
CLR23	Coxiella-Like Repeat protein	-	-	-	-	-	-	-
CLR17	Coxiella-Like Repeat protein	-	-	-	-	-	-	-
	highly expressed pseudogene	-	-	-	-	-	-	-
CLR16	Coxiella-Like Repeat protein	-	-	-	-	-	-	-
CHR3410	SNF2 superfamily protein	-	+	-	-	-	-	GB17968-PA
CHR3417	SNF2 superfamily protein	-	-	-	-	-	-	-
ZRT5	zinc-nutrition responsive transporter	-	+	-	-	-	-	-
ZIP4	ZIP family transporter	-	-	-	-	-	-	-
THB2	truncated hemoglobin, plastid-located	-	-	-	-	-	-	-
CYP739A2	cytochrome P450, unkown function, in CYP85 clan	-	-	-	-	-	-	-
CYP97A6	cytochrome P450, putative carotenoid hydroxylase	-	-	-	-	-	-	-
CYP97A5	cytochrome P450, putative carotenoid hydroxylase	-	-	-	-	-	-	-
FAP42	Flagellar Associated Protein Similar to Adenylate/Guanylate Kinases	-	-	-	+	-	-	-
	cytochrome P450, pseudogene	-	-	-	-	-	-	-
CYP771A1	cytochrome P450, unkown function	-	-	-	-	-	-	-
CYP769A1	cytochrome P450, unkown function	-	-	-	-	-	-	-
PURA	adenylosuccinate synthase	-	+	-	+	-	-	GB14705-PA
PUR6	Phosphoribosylaminoimidazole carboxylase, eukaryotic-type	-	+	-	-	-	-	-
YEE3	conserved membrane protein	-	-	-	-	-	-	-
CYP770A1	cytochrome P450, unkown function	-	-	-	-	-	-	-
CYP768A1	cytochrome P450, unkown function	-	-	-	-	-	-	-
CYP767A1	cytochrome P450, unkown function	-	-	-	-	-	-	-
CYP748A1	cytochrome P450, unkown function	-	-	-	-	-	-	-
CYP747A1	cytochrome P450, unkown function	-	+	-	-	-	-	-
CYP746A1	cytochrome P450, unkown function	-	+	-	-	-	-	GB11973-PA
CYP745A1	cytochrome P450, unkown function	-	+	-	-	-	-	-
CYP744C1	cytochrome P450, unkown function, in CYP711 clan	-	+	-	-	-	-	-
CYP744B1	cytochrome P450, unkown function, in CYP711 clan	-	+	-	-	-	-	GB18052-PA
	cytochrome P450, pseudogene	-	-	-	-	-	-	-
MPC1	Phosphate Carrier	-	+	-	-	-	-	-
CYP744A4	cytochrome P450, unkown function, in CYP711 clan	-	-	-	-	-	-	GB13748-PA
CYP744A3	cytochrome P450, unkown function, in CYP711 clan	-	-	-	-	-	-	-
CYP744A2	cytochrome P450, unkown function, in CYP711 clan	-	-	-	-	-	-	-
CYP744A1	cytochrome P450, unkown function, in CYP711 clan	-	-	-	-	-	-	-
FDX3	Apoferredoxin, chloroplast precursor	-	-	-	-	-	-	-
FDX4	Apoferredoxin, chloroplast precursor	-	+	-	-	-	-	-
FDX6	Apoferredoxin, chloroplast precursor	-	+	-	-	-	-	-
ACA1	calcium-transporting ATPase/ calmodulin binding	-	-	-	+	-	-	-
CYP743C1	cytochrome P450, unkown function, in CYP711 clan	-	-	-	-	-	-	-
CYP743B3	cytochrome P450, unkown function, in CYP711 clan	-	+	-	-	-	-	-
CYP743B2	cytochrome P450, unkown function, in CYP711 clan	-	-	-	-	-	-	-
DMC1	Rad51 homologue involved in meiotic recombination.	-	+	-	-	-	-	-
FCL1	Putative 5-Formyltetrahydrofolate cycloligase (5,10-methenytetrahydrofolate synthetase)	-	+	-	-	-	-	GB16714-PA
CYP743B1	cytochrome P450, unkown function, in CYP711 clan	-	-	-	-	-	-	GB17784-PA
CYP743A2	cytochrome P450, unkown function, in CYP711 clan	-	-	-	-	-	-	-
CYP743A1	cytochrome P450, unkown function, in CYP711 clan	-	-	-	-	-	-	-
CYP742A1	cytochrome P450, unkown function	-	-	-	-	-	-	-
CYP741A1	cytochrome P450, unkown function	-	-	-	-	-	-	-
CYP740A1	cytochrome P450, unkown function, in CYP85 clan	-	-	-	-	-	-	-
CYP739A6	cytochrome P450, unkown function, in CYP85 clan	-	-	-	-	-	-	-
CYP739A5	cytochrome P450, unkown function, in CYP85 clan	-	-	-	-	-	-	-
CYP739A4	cytochrome P450, unkown function, in CYP85 clan	-	-	-	-	-	-	-
CYP739A3	cytochrome P450, unkown function, in CYP85 clan	-	-	-	-	-	-	-
CYP739A1	cytochrome P450, unkown function, in CYP85 clan	-	-	-	-	-	-	-
FKB15-2	peptidyl-prolyl cis-trans isomerase, FKBP-type	-	-	-	-	-	-	-
CYP738A1	cytochrome P450, unkown function, in CYP85 clan	-	+	-	-	-	-	-
OHP1	low CO2 and stress-induced one-helix protein	-	-	-	-	-	-	-
CGK	cGMP-dependent protein kinase	-	-	-	-	-	-	-
CYP737A1	cytochrome P450, unkown function, in CYP85 clan	-	-	-	-	-	-	-
CYP710B1	cytochrome P450, putative C-22 desaturase	-	+	-	-	-	-	-
CYP97C3	cytochrome P450, putative carotenoid hydroxylase	-	-	-	-	-	-	-
CYP97B6	cytochrome P450, putative carotenoid hydroxylase	-	-	-	-	-	-	-
CYP97A6	cytochrome P450, putative carotenoid hydroxylase	-	-	-	-	-	-	-
CYP97A5	cytochrome P450, putative carotenoid hydroxylase	-	+	-	-	-	-	GB30317-PA
CYP55B1	cytochrome P450, putative nitric oxide reductase	-	-	-	-	-	-	-
	Putative thioredoxin-like protein containing Erv1/Alr domain	-	+	-	-	-	-	GB11873-PA
FUM2	Fumarase (fumarate hydratase)	-	+	-	-	-	-	GB15172-PA
Ycf39-2	Putative pyridine nucleotide binding protein	-	+	-	-	-	-	-
MSD3B	superoxide dismutase [Mn]	-	-	-	-	-	-	-
MSD3A	superoxide dismutase [Mn]	-	-	-	-	-	-	-
IRT1	zinc transporter, chloroplast precursor	-	-	-	-	-	-	-
ZRT4	zinc-nutrition responsive transporter	-	+	-	-	-	-	GB12714-PA
PRX3	Peroxiredoxin, type II, probably cytosolic	-	-	-	-	-	-	-
ZIL2	ZIP family transporter-like	-	+	-	-	-	-	-
ZIP2	ZIP family transporter	-	-	-	-	-	-	-
ZIP3	ZIP family transporter	-	+	-	-	-	-	-
MLK	Mixed lineage protein kinase	-	-	-	-	-	-	-
PFK1P	Phosphofructokinase family protein, plastid form	-	+	-	-	-	-	-
FKB16-6	peptidyl-prolyl cis-trans isomerase, FKBP-type	-	-	-	-	-	-	-
PPD2	Pyruvate,phosphate dikinase (PPDK)	-	+	-	-	-	-	-
PCK1b	phosphoenolpyruvate carboxykinase; PEP carboxykinase	-	-	-	-	-	-	-
CYN16	Peptidyl-prolyl cis-trans isomerase, cyclophilin-type	-	-	-	-	-	-	-
LEU2	isopropylmalate synthase, putative	-	+	-	-	-	-	-
DXR	1-deoxy-D-xylulose 5-phosphate reductoisomerase, putative chloroplast precursor	-	+	-	-	-	-	-
CMS	4-diphosphocytidyl-2C-methyl-D-erythritol synthase, putative chloroplast precursor	-	+	-	-	-	-	-
	Amidase	-	+	-	-	-	-	-
CRTISO	carotenoid isomerase, putative chloroplast precursor	-	+	-	-	-	-	-
THD1	threonine deaminase	-	+	-	-	-	-	GB12293-PA
SHKF1	possible shikimate kinase	-	+	-	-	-	-	-
SBP1	Sedoheptulose-1,7-bisphosphatase - like protein	-	-	-	-	-	-	-
PGM	phosphoglycerate mutase-like protein	-	-	-	-	-	-	-
RHE	RNA helicase;	-	+	-	-	-	-	-
ASPS1	aspartyl-tRNA synthetase, possibly mitochondrial	-	+	-	-	-	-	GB11285-PA
PTP	uncharacterized transport protein	-	-	-	-	-	-	-
PKD1	protein kinase domain containing protein	-	-	-	-	-	-	-
MPI	mannose-6-phosphate isomerase like	-	-	-	-	-	-	-
SCLA1	Succinyl-CoA ligase alpha-chain, mitochondrial precursor	-	-	-	-	-	-	-
SCLB1b	Succinate-CoA ligase beta chain, possibly cytosolic	-	-	-	-	-	-	-
DXS,DXS1	1-deoxy-D-xylulose 5-phosphate synthase, putative chloroplast precursor	-	+	-	-	-	-	GB19641-PA
IDH3	isocitrate dehydrogenase, NADP-dependent, mitochondrial	-	+	-	-	-	-	GB14517-PA
CYN65	Peptidyl-prolyl cis-trans isomerase, cyclophilin-type	-	+	-	-	-	-	GB18535-PA
CYN52	Peptidyl-prolyl cis-trans isomerase with two cyclophilin domains	-	-	-	-	-	-	-
CYN53	Peptidyl-prolyl cis-trans isomerase with two cyclophilin domains	-	-	-	-	-	-	-
TIM8	Mitochondrial import inner membrane translocase subunit	-	+	-	-	-	-	GB14140-PA
CYN38	Peptidyl-prolyl cis-trans isomerase, cyclophilin-type	-	+	-	-	-	-	-
DGD1	galactolipid galactosyltransferase	-	+	-	-	-	-	-
DHQS	3-dehydroquinate synthase	-	+	-	-	-	-	-
ClpP2	catalytic subunit of mitochondrial ClpXP protease	-	+	-	-	-	-	GB10807-PA
CLPY1	ATP-dependent subunit of the HslUV protease	-	+	-	-	-	-	-
ACLA1	ATP Citrate Lyase, subunit A	-	+	-	-	-	-	-
CLPX	ClpX, ATP-dependent Clp protease ATP-binding subunit	-	+	-	-	-	-	GB13515-PA
NAE	anion transporter	-	-	-	-	-	-	-
CAH7	carbonic anhydrase 7	-	-	-	-	-	-	-
ANT1	ANT1 Adenine Nucleotide Translocator 1, mitochondrial	-	-	-	-	-	-	-
DLD2	dihydrolipoamide dehydrogenase, plastid precursor, putative	-	+	-	-	-	-	-
ERG4/ERG24	sterol redcutase	-	-	-	-	-	-	-
PHOD	PHOD Putative alkaline phosphatase	-	-	-	-	-	-	-
DEGO	DegP-type protease, inactive	-	-	-	-	-	-	-
DLA2	dihydrolipoamide acetyltransferase, possibly plastidic	-	+	-	-	-	-	-
LSP1	putative signal transducer for phototaxis	-	-	-	-	-	-	-
SULTR1	sulfate transporter	-	+	-	-	-	-	GB30556-PA

OASTL2	cysteine synthase	-	+	-	-	-	-	-	AT1G55880.1	[illegible]
PHOA	PHOA Putative alkaline phosphatase	-	-	-	-	-	-	-	-	[illegible]
METC	cystathionine beta-lyase	-	+	+	-	-	-	GB15881-PA	AT3G57050.2	[illegible]
DUR2	allophanate hydrolase	-	-	-	-	-	-	-	-	[illegible]
DUR3A	urea active transporter, isoform A	-	+	-	-	-	-	GB13996-PA	AT5G45380.1	[illegible]
LPB1	LPB1 Low Photochemical Bleaching 1 protein	-	-	-	-	-	-	-	AT3G56040.1	[illegible]
PTB6	PTB6 Putative sodium/phosphate symporter	-	-	-	-	-	-	-	-	[illegible]
PDH1b	pyruvate dehydrogenase, E1 beta subunit, mitochondrial precursor	-	-	-	+	-	-	-	-	[illegible]
PTA2	PTA2 Putative proton/phosphate symporter	-	+	-	-	-	-	-	AT5G43340.1	[illegible]
LCI5	low-CO2-inducible protein	-	-	-	-	-	-	-	-	[illegible]
PTB3	PTB3 Putative sodium/phosphate symporter	-	-	-	-	-	-	-	-	[illegible]
PTB2	PTB2 Putative sodium/phosphate symporter	-	-	-	-	-	-	-	-	[illegible]
GAP1b	glyceraldehyde 3-phosphate dehydrogenase, cytosolic	-	-	-	-	-	-	-	-	[illegible]
GAP2	glyceraldehyde 3-phosphate dehydrogenase, probably cytosolic	-	-	-	-	-	-	-	-	[illegible]
PTB4	PTB4 Putative sodium/phosphate symporter	-	+	-	-	-	-	GB13603-PA	-	[illegible]
PTB5	PTB5 Putative sodium/phosphate symporter	-	-	-	-	-	-	-	AT3G26570.2	[illegible]
TAL2	Transaldolase, putative	-	-	-	-	-	-	-	-	[illegible]
PFK2	Phosphofructokinase family protein	-	-	-	-	-	-	-	-	[illegible]
	amino acid permease	-	+	-	-	-	-	GB10226-PA	AT5G02180.1	[illegible]
MRS2E	Mg2+ transporter protein, CorA-like	-	+	-	-	-	-	-	AT5G22830.1	[illegible]
ERG5	Sterol C-22 desaturase	-	-	-	-	-	-	-	-	[illegible]
PHOX	PHOX Alkaline phosphatase, secreted, phosphate-repressible	-	-	-	-	-	-	-	-	[illegible]
RSP1	Radial spoke protein 1	-	+	-	+	-	-	-	-	[illegible]
ERG11	sterol 14 desaturase	-	-	-	-	-	-	-	-	[illegible]
CAS	cycloartenol synthase	-	-	-	-	-	-	-	-	[illegible]
PHC6	cell wall protein pherophorin-C6	-	-	-	-	-	-	-	-	[illegible]
PHC5	cell wall protein pherophorin-C5	-	-	-	-	-	-	-	-	[illegible]
PHC4	cell wall protein pherophorin-C4	-	-	-	-	-	-	-	-	[illegible]
PHC3	cell wall protein pherophorin-C3	-	-	-	+	-	-	-	-	[illegible]
PHC2	cell wall protein pherophorin-C2	-	-	-	+	-	-	-	-	[illegible]
SHMT3	serine hydroxymethyltransferase 3	-	+	-	+	-	-	GB14485-PA	AT4G32520.1	[illegible]
PHC1	cell wall protein pherophorin-C1	-	-	-	-	-	-	-	-	[illegible]
RSP17	Radial spoke protein 17	-	-	-	+	-	-	-	-	[illegible]
DEG10	DegP-type protease, mitochondrial	-	+	-	-	-	-	-	AT5G36950.1	[illegible]
PGK2	Phosphoglycerate kinase, cytosolic form	-	-	-	-	-	-	-	-	[illegible]
MFT8	general substrate transporter:major facilitator superfamily	-	-	-	-	-	-	-	-	[illegible]
MITC8	putative mitochondrial substrate carrier protein	-	+	-	-	-	-	-	AT2G46320.3	[illegible]
	hypothetical protein	-	+	-	-	-	-	-	AT5G02240.1	[illegible]
GLYK	glycerate kinase	-	-	-	-	-	-	-	-	[illegible]
PTB7	PTB7 Putative sodium/phosphate symporter	-	-	-	-	-	-	-	-	[illegible]
DEG5A	DegP-type protease	-	+	-	-	-	-	GB16003-PA	-	[illegible]
DEG1	DegP-type protease, thylakoid lumen	-	+	-	-	-	-	-	AT3G27925.1	[illegible]
SHMT2	serine hydroxymethyltranferase 2	-	+	-	-	-	-	-	AT4G13930.1	[illegible]
MME5	NADP malic enzyme	-	-	-	-	-	-	-	-	[illegible]
THI4	thiazole biosynthetic enzyme regulatory fragment	-	-	-	-	-	-	-	-	[illegible]
PANB	Ketopantoate hydroxymethyltransferase	-	+	-	-	-	-	-	AT3G61530.2	[illegible]
PSBS	chloroplast PsbS	-	-	-	-	-	-	-	-	[illegible]
PTB12	PTB12 Putative sodium/phosphate symporter	-	-	-	-	-	-	-	-	[illegible]
PTB8	PTB8 Putative sodium/phosphate symporter	-	+	-	-	-	-	-	-	[illegible]
PRD1	prephenate dehydratase, putative	-	+	-	-	-	-	-	AT1G08250.1	[illegible]
MAS1	malate synthase	-	+	-	+	-	-	-	AT5G03860.1	[illegible]
HXT1	putative hexose transporter, plastid	-	+	-	-	-	-	GB13688-PA	AT5G16150.3	[illegible]
HSD1	homoserine dehydrogenase, putative	-	+	-	-	-	-	-	-	[illegible]
FER2	putative ferritin subunit	-	-	-	-	-	-	-	-	[illegible]
CHM1	chorismate mutase, putative	-	+	-	-	-	-	-	AT3G29200.1	[illegible]
AHD1	bifunctional aspartate kinase/homoserine dehydrogenase, putative	-	+	-	+	-	-	-	AT4G19710.2	[illegible]
ACS3	Acetyl CoA synthetase	-	-	-	+	-	-	-	-	[illegible]
PFK1b	Phosphofructokinase family protein, cytosolic form	-	+	-	-	-	-	-	-	[illegible]
PTB1	PTB1 Putative sodium/phosphate symporter	-	-	-	-	-	-	-	-	[illegible]
PGM1a	Phosphoglyceromutase	-	-	-	+	-	-	-	-	[illegible]
FBA4	Fructose-1,6-bisphosphate aldolase	-	-	-	-	-	-	-	-	[illegible]
SMP6b	U6 snRNA-associated Sm-like protein LSm4, putative	-	+	+	-	-	-	GB12398-PA	AT5G27720.1	[illegible]
RET1	related to EIN2	-	-	-	-	-	-	-	-	[illegible]
PGP1	phosphoglycolate phosphatase 1	-	+	-	-	-	-	GB12982-PA	AT5G36700.1	[illegible]
HDH1	possible hydrolase	-	-	-	-	-	-	-	-	[illegible]
	Expressed Protein of unknown function	-	-	-	-	-	-	-	-	[illegible]
PSRP-6	Chloroplast ribosomal protein Plastid-Specific Ribosomal Protein 6; Chloroplast large ribosomal subunit protein Plastid-Specific Ribosomal Protein 6;	-	-	-	-	-	-	-	-	[illegible]
CYN19-2	Peptidyl-prolyl cis-trans isomerase, cyclophilin-type	-	+	-	+	-	-	GB17056-PA	AT2G21130.1	[illegible]
		-	+	-	-	-	-	-	AT5G08400.2	[illegible]
PRPL28	Chloroplast ribosomal protein L28; Chloroplast large ribosomal subunit protein L28;	-	+	-	-	-	-	-	AT2G33450.1	[illegible]
	acyl carrier protein thioesterase, putative	-	+	-	-	-	-	-	AT3G25110.1	[illegible]
	protein of unknown function	-	-	-	-	-	-	-	-	[illegible]
BCA3	branched chain amino acid aminotransferase	-	+	-	-	-	-	-	AT3G05190.1	[illegible]
LCI3	Low-CO2 inducible protein	-	-	-	-	-	-	-	-	[illegible]
LCI2	low CO2 inducible gene 2	-	-	-	-	-	-	-	-	[illegible]
PDK2	pyruvate dehydrogenase kinase, putative	-	-	-	-	-	-	-	-	[illegible]
PDK1	pyruvate dehydrogenase kinase, putative	-	+	-	-	-	-	-	-	[illegible]
	haloacid dehalogenase-like hydrolase	-	+	-	-	-	-	-	AT2G25870.1	[illegible]
	protein of unknown function	-	-	-	-	-	-	-	-	[illegible]
PRPL18	Plastid ribosomal protein L18, imported to chloroplast, large ribosomal subunit	-	+	-	-	-	-	-	AT1G48350.1	[illegible]
PYK2	Pyruvate kinase, cytosolic	-	-	-	-	-	-	-	-	[illegible]
PYK3	Pyruvate kinase	-	-	-	+	-	-	-	-	[illegible]
ELG1	exostosin-like glycosyltransferase	-	-	-	-	-	-	-	-	[illegible]
	expressed protein of unknown function	-	-	-	-	-	-	-	-	[illegible]
PCK1a	phosphoenolpyruvate carboxykinase; PEP carboxykinase	-	+	-	-	-	-	-	AT4G37870.1	[illegible]
HPT2	putative hexose-phosphate transporter	-	-	-	-	-	-	-	-	[illegible]
	putative NADH/cytochrome b5 reductase	-	-	-	-	-	-	-	-	[illegible]
SSD3	sterol sensing domain protein	-	+	-	-	-	-	-	AT4G38350.1	[illegible]
CAH8	carbonic anhydrase 8	-	+	-	-	-	-	GB19829-PA	-	[illegible]
GCST	Glycine cleavage system, T protein	-	+	-	-	-	-	GB12854-PA	AT1G11860.2	[illegible]
FAP96	protein of unknown function	-	+	-	+	-	-	-	-	[illegible]
PTB9	PTB9 Putative sodium/phosphate symporter	-	-	-	-	-	-	-	-	[illegible]
UBI1	ubiquitin, minor isoform UBI1b	-	-	-	-	-	-	-	-	[illegible]
	possible regulation of calmodulin expression	-	-	-	-	-	-	-	-	[illegible]
HYDG	Hydrogenase assembly factor	-	-	-	-	-	-	-	-	[illegible]
GUN4	GUN4 chloroplast precursor	-	+	-	-	-	-	-	AT3G59400.1	[illegible]
DEG9	DegP-type protease	-	-	-	-	-	-	-	-	[illegible]
		-	-	-	-	-	-	-	-	[illegible]
PHGPx1	Glutathione peroxidase, selenoprotein	-	-	-	-	-	-	-	-	[illegible]
SelW1	Selenoprotein W1	-	-	-	-	-	-	-	-	[illegible]
SelK1	Selenoprotein K1	-	+	-	-	-	-	-	-	[illegible]
SelM2	Selenoprotein M2	-	-	-	-	-	-	-	-	[illegible]
SelW2	Selenoprotein W2	-	-	-	-	-	-	-	-	[illegible]
SelT	Selenoprotein T	-	+	-	-	-	-	GB15757-PA	AT5G58640.2	[illegible]
SelU	Selenoprotein U	-	+	-	-	-	-	-	-	[illegible]
PHGPx2	Glutathione peroxidase, selenoprotein	-	-	-	-	-	-	-	-	[illegible]
MKP2	MAP Kinase Phosphatase Homolog 2	-	-	-	-	-	-	GB19901-PA	-	[illegible]
	Thioredoxin-like protein, unusual active site WCNAC	-	+	-	-	-	-	-	AT4G29670.1	[illegible]
	Thioredoxin-like protein	-	-	-	-	-	-	-	-	[illegible]
	Thioredoxin-like protein	-	-	-	-	-	-	-	-	[illegible]
NTR2	NADPH dependent thioredoxin reductase 2	-	-	-	-	-	-	-	-	[illegible]
TRXh2	thioredoxin h2, cytosolic, splicing variant c	-	-	-	-	-	-	-	-	[illegible]
TRXh2	thioredoxin h2, cytosolic, splicing variant a	-	-	-	-	-	-	-	-	[illegible]
TRXh2	thioredoxin h2, cytosolic, splicing variant b	-	+	-	-	-	-	-	-	[illegible]
CTR3	copper transport related	-	-	-	-	-	-	-	-	[illegible]
SPP1C	signal peptide peptidase	-	-	-	-	-	-	-	-	[illegible]
MSRA1	Peptide methionine-S-sulfoxide reductase	-	+	-	-	-	-	-	-	[illegible]
MSRA2	Peptide methionine-S-sulfoxide reductase, putative	-	+	-	-	-	-	-	-	[illegible]
COPT1	CTR type copper ion transporter	-	+	-	-	-	-	-	AT3G46900.1	[illegible]
CTR1	CTR type copper ion transporter	-	+	-	-	-	-	GB19681-PA	-	[illegible]
CTR2	CTR type copper ion transporter	-	-	-	-	-	-	-	-	[illegible]
LIPA2	lipoic acid synthetase	-	+	-	-	-	-	-	AT5G08415.1	[illegible]
FRE1	ferric-chelate reductase/ oxidoreductase	-	+	-	-	-	-	-	AT1G01580.1	[illegible]
	putative carnitine/acylcarnitine translocase	-	-	-	-	-	-	-	AT1G79900.1	[illegible]
IMP	mitochondrial signal peptidase	-	-	-	-	-	-	-	-	[illegible]
VPE	vacuolar processing enzyme	-	+	-	-	-	-	-	AT1G62710.1	[illegible]
	Roadblock/LC7 family protein	-	+	-	-	-	-	-	-	[illegible]
	Unknown Protein	-	-	-	-	-	-	-	-	[illegible]
	OST3/OST6 family protein	-	+	-	-	-	-	GB12640-PA	AT1G61790.1	[illegible]
TRS85	Component of TRAPP complex, TRS85-like	-	+	-	-	-	-	GB12801-PA	AT5G16280.1	[illegible]
	RING and FYVE domain containing protein	-	+	-	-	-	-	GB14697-PA	AT3G43230.1	[illegible]
	unknown protein	-	+	-	-	-	-	-	AT5G47560.1	[illegible]
	AAA-ATPase domain family protein	-	-	-	-	-	-	-	-	[illegible]
SF3b4	Nuclear pre-mRNA splicing factor, component of splicing factor 3b	-	+	+	-	-	-	GB12347-PA	AT2G18510.1	[illegible]
SPL2	similar to splicing factor 3a, subunit 2	-	+	+	-	-	-	GB15090-PA	AT2G32600.1	[illegible]
		-	-	-	-	-	-	-	-	[illegible]
TPR6	Tetratrichopsptide-repeat protein	-	-	-	-	-	-	-	AT1G04190.1	[illegible]
GCSH	glycine cleavage system, H-protein	-	+	-	-	-	-	-	AT2G35120.1	[illegible]
	unknown protein	-	-	-	-	-	-	-	-	[illegible]
ALK3	Aurora Like Kinase Homolog 3	-	-	-	-	-	-	-	-	[illegible]
	BPI/LBP/CETP N-terminal domain protein	-	-	-	-	-	-	-	-	[illegible]
MSP	Membrane selenoprotein	-	-	-	-	-	-	-	-	[illegible]
SelH	Selenoprotein H	-	+	-	-	-	-	-	AT4G31360.1	[illegible]
ESU1	putative Endosulfine, cAMP-regulated phosphoprotein	-	-	-	-	-	-	-	-	[illegible]
ESU1	putative Endosulfine, cAMP-regulated phosphoprotein	-	-	-	-	-	-	-	-	[illegible]
IDH2	isocitrate dehydrogenase, NAD-dependent	-	+	-	-	-	-	GB19422-PA	AT3G09810.1	[illegible]
IDH1	isocitrate dehydrogenase, NAD-dependent, mitochondrial	-	-	-	-	-	-	-	-	[illegible]
MMP5	metalloproteinase, cell wall protein, homolog of Volvox VMPs	-	-	-	-	-	-	-	-	[illegible]
MMP4	metalloproteinase, cell wall protein, homolog of Volvox VMPs	-	-	-	-	-	-	-	-	[illegible]
PHC18	cell wall protein pherophorin-C18	-	-	-	+	-	-	-	-	[illegible]
PHC13	cell wall protein pherophorin-C13	-	-	-	+	-	-	-	-	[illegible]
PHC11	cell wall protein pherophorin-C11	-	-	-	-	-	-	-	-	[illegible]
PHC24	cell wall protein pherophorin-C24	-	-	-	-	-	-	-	-	[illegible]
PHC10	cell wall protein pherophorin-C10	-	-	-	-	-	-	-	-	[illegible]
PHC20	cell wall protein pherophorin-C20	-	-	-	-	-	-	-	-	[illegible]
PHC9	cell wall protein pherophorin-C9	-	-	-	-	-	-	-	-	[illegible]

PHC23	cell wall protein pherophorin-C23	[illegible]
CAX3	CAX family of Cation antiporters, membrane protein	[illegible]
CTP2	heavy metal transporting ATPase (HMA), P-type ATPase superfamily, membrane protein	[illegible]
PHC8	cell wall protein pherophorin-C8	[illegible]
HMA1	heavy metal transporting ATPase (HMA), P-type ATPase superfamily, membrane protein	[illegible]
PHC7	cell wall protein pherophorin-C7	[illegible]
PTN1	PTEN PI-3 phosphatase	[illegible]
SDH1	succinate dehydrogenase subunit A; mitochondrial	[illegible]
	SET domain containing protein; putative histone methyltransferase	[illegible]
	Hypothetical WD40 domain protein	[illegible]
SYP1	Qa-SNARE, PM-type	[illegible]
CTP3	heavy metal transporting ATPase (HMA), P-type ATPase superfamily, membrane protein	[illegible]
MTP3	CDF transporter, membrane protein	[illegible]
UTP1	Nucleolar protein, Component of the U3 processome	[illegible]
FUM1	fumarate hydratase	[illegible]
DVR	3,8-divinyl protochlorophyllide a 8-vinyl reductase, chloroplast precursor	[illegible]
CAO	Chlorophyll a oxygenase, chloroplast precursor	[illegible]
TIC55	Rieske iron-sulfur cluster 55 kDa protein of the chloroplast inner membrane translocon	[illegible]
HMOX1	Heme oxygenase	[illegible]
	Nickel chaperone for hydrogenase or urease, putative	[illegible]
CTH1	Copper target homolog 1, chloroplast precursor, functional alternative spliced variant	[illegible]
UROS	Uroporphyrinogen-III synthase, putative chloroplast precursor	[illegible]
		[illegible]
	putative hydroxyproline-rich glycoprotein	[illegible]
FUS1	plasma membrane protein involved in gamete fusion	[illegible]
CDPKK1	CDPKK1; Calcium/Calmodulin Dependent Protein Kinase Kinase Homolog 1	[illegible]
RWP13	transcription factor, RWP-RK domain containing; putative	[illegible]
RWP12	hypothetical protein	[illegible]
RWP10	hypothetical protein	[illegible]
RWP1	hypothetical protein	[illegible]
NTR1	NADPH-dependent thioredoxin reductase 1, cytosolic selenoprotein	[illegible]
PRK	Phosphoribulokinase, chloroplast precursor	[illegible]
LEU3	3-Isopropylmalate dehydrogenase, splicing variant b	[illegible]
LEU3	3-Isopropylmalate dehydrogenase, splicing variant a	[illegible]
DNJ3	DnaJ-like protein 3, DnaJ/PDI fusion protein, similar to human ERdj5	[illegible]
RB60	RB60 (PDI1), protein disulfide isomerase 1, located in the chloroplast and the ER	[illegible]
RWP11	transcription factor, RWP-RK domain containing; putative	[illegible]
CITRX	Thioredoxin CITRX	[illegible]
TRXh1	Thioredoxin h1, cytosolic	[illegible]
TRXf2	Thioredoxin f2, chloroplast precursor	[illegible]
PK1	Protein kinase homolog 1 putative	[illegible]
	Putative adaptin-interacting protein	[illegible]
FAP9	probable flagellar ATPase, related to Ras GTPase	[illegible]
		[illegible]
ARL16	ARF-like 16 GTPase	[illegible]
RWP7	transcription factor, RWP-RK domain containing; putative	[illegible]
	hypothetical protein of unknown function	[illegible]
	RNA helicase involved in non-sense mediated decay	[illegible]
	hypothetical protein of unknown function	[illegible]
RWP6	transcription factor, RWP-RK domain containing; putative	[illegible]
PKY1	Protein Tyrosine Kinase Homolog 1, putative	[illegible]
SRP35	putative SR protein factor involved in mRNA splicing	[illegible]
SRP36	putative SR protein factor involved in mRNA splicing	[illegible]
RSZ22	putative SR protein factor involved in mRNA splicing	[illegible]
SCL26	putative SR protein factor involved in mRNA splicing	[illegible]
LZY1A	unknown protein; pseudogene	[illegible]
RWP9	hypothetical protein	[illegible]
RWP8	transcription factor, RWP-RK domain containing; putative	[illegible]
RWP4	transcription factor, RWP-RK domain containing; putative	[illegible]
RFC4	DNA replication factor C complex subunit 4	[illegible]
CNK9	Chlamydomonas NIMA-related Kinase Homolog 9	[illegible]
GAS30	hydroxyproline-rich glycoprotein; stress-induced	[illegible]
VSP4	hydroxyproline-rich glycoprotein; cell wall protein	[illegible]
MTA3a	protein of unknown function; possibly a pseudogene	[illegible]
MUT9	kinase involved in transcriptional gene silencing	[illegible]
UROD3	Uroporphyrinogen decarboxylase	[illegible]
NIT2	transcription factor regulating nitrogen metabolism	[illegible]
PI3K	Phosphoinositide 3-kinase	[illegible]
CLPS3	ClpS-like protein	[illegible]
PI4K	Phosphatidylinositol 4-kinase	[illegible]
VSP3	extracellular matrix protein	[illegible]
MKP3	MAP Kinase Phosphatase Homolog 3	[illegible]
ZSP2-1	lectin-like protein; component of the zygote cell wall	[illegible]
ZSP1	hydroxyproline-rich glycoprotein; zygote-specific	[illegible]
E2FR1	related to E2F and DP transcription factors	[illegible]
DP1	DP transcription factor homologue	[illegible]
E2F1	E2F family homologue	[illegible]
CYCA1	A-type cyclin homologue	[illegible]
CDKI1	cyclin dependent kinase, Chlamydomonas specific	[illegible]
CYCD1	D-type cyclin homologue	[illegible]
GP2	hydroxyproline-rich glycoprotein component of the outer cell wall	[illegible]
GRX5	Glutaredoxin 5, CGFS type, probably mitochondrial	[illegible]
GRX4	Glutaredoxin 4, CGFS type, probably cytosolic, contains 2 glutaredoxin modules	[illegible]
	Thioredoxin-like protein	[illegible]
PR46a	Probable ubiquitin-fold modifier 1 precursor	[illegible]
GRX1	Glutaredoxin 1, CPYC type, probably cytosolic	[illegible]
SRP23	putative nuclear SR-like RNA binding protein	[illegible]
NDA6	putative type-II NADH dehydrogenase	[illegible]
NDA7	putative type-II NADH dehydrogenase	[illegible]
	putative protein kinase	[illegible]
SEC8	Component of the Exocyst Complex	[illegible]
TATC	TatC-like sec-independent protein translocon subunit	[illegible]
ATP9B	F1Fo ATP synthase subunit 9 (subunit c), isoform B	[illegible]
ATP9A	F1Fo ATP synthase subunit 9 (subunit c), mitochondrial	[illegible]
SECY2	Preprotein translocase secY subunit, chloroplast precursor	[illegible]
NDA1	putative mitochondrial type-II NADH dehydrogenase.	[illegible]
COX16	putative cytochrome c oxidase assembly factor 16	[illegible]
COX5c	Cytochrome c oxidase 7kD subunit, mitochondrial	[illegible]
COX12	cytochrome c oxidase 16 kD subunit, mitochondrial	[illegible]
"NAD	Putative mitochondrial NADH dehydrogenase (ubiquinone oxidoreductase)' '	[illegible]
COX11	putative protein involved in assembly of copper in mitochondrial cytochrome c oxidase	[illegible]
REX1B	Conserved Protein of Unknown Function	[illegible]
ODC1.4	Ornithine Decarboxylase	[illegible]
ODC1.3	Ornithine Decarboxylase	[illegible]
ODC1.2	Ornithine Decarboxylase	[illegible]
ODC2	Ornithine Decarboxylase 2	[illegible]
SEC24C	putative COP-II coat subunit	[illegible]
	DUF962-domain containing membrane protein	[illegible]
	Hep_59-domain containing protein	[illegible]
	LIM-domain contating metalloprotease	[illegible]
MTA1	protein of unknown function; plus gamete specific	[illegible]
MTA2	hydroxyproline-rich glycoprotein, putative component of the zygote ECM	[illegible]
	Trehalase-like protein	[illegible]
REX1S	Probable DNA Repair Enzyme	[illegible]
	EH-domain, GTPase-like protein	[illegible]
	Inositol-polyphosphate phosphatase-like	[illegible]
	EMP/nonaspanin domain family	[illegible]
	EMP/nonaspanin domain family	[illegible]
	VID72-domain protein	[illegible]
	emp24/gp25L/p24 family protein	[illegible]
ERO1	endoplasmic reticulum oxidoreductin 1 (ERO1) family protein	[illegible]
ZYS1a	putative transcription factor; zygote-specific	[illegible]
SAG1	plus agglutinin protein	[illegible]
COG1	Component of oligomeric golgi complex 1	[illegible]
COG5	Component of oligomeric golgi complex 5	[illegible]
COG6	Component of oligomeric golgi complex 6	[illegible]
COG4	Component of oligomeric golgi complex 4	[illegible]
COG3	Component of oligomeric golgi complex 3	[illegible]
APG6	Autophagy protein	[illegible]
	Apg1/Unc-51-like Serine-Threonine Kinase	[illegible]
SDH3	Succinate dehydrogenase subunit b560, mitochondrial	[illegible]
PRPS17	Plastid ribosomal protein S17, imported to chloroplast, small ribosomal subunit	[illegible]
PRPS10	Plastid ribosomal protein S10, imported to chloroplast, small ribosomal subunit	[illegible]
PRPL27	Plastid ribosomal protein L27, imported to chloroplast, large ribosomal subunit	[illegible]
PRPL15	Plastid ribosomal protein L15, imported to chloroplast, large ribosomal subunit	[illegible]
PRPL11	Plastid ribosomal protein L11, imported to chloroplast, large ribosomal subunit	[illegible]
PSRP-3	Plastid-specific ribosomal protein 3, imported to chloroplast	[illegible]
PRPS6	Plastid ribosomal protein S6, imported to chloroplast, small ribosomal subunit	[illegible]
PRPS21	Plastid ribosomal protein S21, imported to chloroplast, small ribosomal subunit	[illegible]
PRPS20	Plastid ribosomal protein S20, imported to chloroplast, small ribosomal subunit	[illegible]
PRPS16	Plastid ribosomal protein S16, imported to chloroplast, small ribosomal subunit	[illegible]
PRPS15	Plastid ribosomal protein S15, imported to chloroplast, small ribosomal subunit	[illegible]
PRPS13	Plastid ribosomal protein S13, imported to chloroplast, small ribosomal subunit	[illegible]
PRPL33	Plastid ribosomal protein L33, imported to chloroplast, large ribosomal subunit	[illegible]
PRPL31	Plastid ribosomal protein L31, imported to chloroplast, large ribosomal subunit	[illegible]
PRPL3	Plastid ribosomal protein L3, imported to chloroplast, large ribosomal subunit	[illegible]
PRPL24	Plastid ribosomal protein L24, imported to chloroplast, large ribosomal subunit	[illegible]
PRPL21	Plastid ribosomal protein L21, imported to chloroplast, large ribosomal subunit	[illegible]
PRPL17	Plastid ribosomal protein L17, imported to chloroplast, large ribosomal subunit	[illegible]
PRPL13	Plastid ribosomal protein L13, imported to chloroplast, large ribosomal subunit	[illegible]
	Polyprotein of PSRP-7 and EF-Ts, alternative splicing	[illegible]
	Plastid-specific ribosomal protein 7, imported to chloroplast	[illegible]
PETs	Polyprotein of PSRP-7 and EF-Ts, imported to chloroplast	[illegible]
GRX6	Glutaredoxin 6, CGFS type, probably chloroplastic	[illegible]
TRXo	Thioredoxin o, probably mitochondrial	[illegible]
GRX2	Glutaredoxin 2, CPYC type glutaredoxin, probably cytosolic	[illegible]

RPL41	Ribosomal protein L41, component of cytosolic 80S ribosome and 60S large subunit	-	-	-	-	-	-	-	[illegible]
RPL24	Ribosomal protein L24, component of cytosolic 80S ribosome and 60S large subunit	-	+	+	-	-	-	GB12361-PA	[illegible]
RPS29	Ribosomal protein S29, component of cytosolic 80S ribosome and 40S small subunit	-	+	+	-	-	-	GB12771-PA	[illegible]
RPS16	Ribosomal protein S16, component of cytosolic 80S ribosome and 40S small subunit	-	+	+	-	-	-	GB10669-PA	[illegible]
RPS8	Ribosomal protein S8, component of cytosolic 80S ribosome and 40S small subunit	-	+	+	-	-	-	GB13741-PA	[illegible]
RPS2	Ribosomal protein S2, component of cytosolic 80S ribosome and 40S small subunit	-	+	+	+	-	-	GB10861-PA	[illegible]
RPS17	Ribosomal protein S17, component of cytosolic 80S ribosome and 40S small subunit	-	+	+	-	-	-	GB10913-PA	[illegible]
RPP2	Acidic ribosomal protein P2, component of cytosolic 80S ribosome and 60S large subunit	-	+	-	-	-	-	-	[illegible]
RPL7a	Ribosomal protein L7a, component of cytosolic 80S ribosome and 60S large subunit	-	+	+	-	-	-	GB11643-PA	[illegible]
RPL4	Ribosomal protein L4, component of cytosolic 80S ribosome and 60S large subunit	-	+	+	-	-	-	GB15503-PA	[illegible]
RPL36	Ribosomal protein L36, component of cytosolic 80S ribosome and 60S large subunit	-	+	+	-	-	-	GB18771-PA	[illegible]
RPL34	Ribosomal protein L34, component of cytosolic 80S ribosome and 60S large subunit	-	+	+	-	-	-	GB14114-PA	[illegible]
RPL40	Ribosomal protein L40, component of cytosolic 80S ribosome and 60S large subunit	-	+	-	-	-	-	GB18108-PA	[illegible]
RPS9	Ribosomal protein S9, component of cytosolic 80S ribosome and 40S small subunit	-	+	+	-	-	-	GB12637-PA	[illegible]
RPS20	Ribosomal protein S20, component of cytosolic 80S ribosome and 40S small subunit	-	+	+	-	-	-	GB17488-PA	[illegible]
RPL8	Ribosomal protein L8, component of cytosolic 80S ribosome and 60S large subunit	-	+	+	-	-	-	GB17629-PA	[illegible]
RPL19	Ribosomal protein L19, component of cytosolic 80S ribosome and 60S large subunit	-	+	+	-	-	-	GB11299-PC	[illegible]
RPL18	Ribosomal protein L18, component of cytosolic 80S ribosome and 60S large subunit	-	+	+	-	-	-	GB11029-PA	[illegible]
	RabGAP/TBC Domain Protein	-	-	-	-	-	-	-	[illegible]
RPL11	Ribosomal protein L11, component of cytosolic 80S ribosome and 60S large subunit	-	+	+	-	-	-	GB30393-PA	[illegible]
	RabGAP/TBC Domain Protein	-	+	-	-	-	-	GB18429-PA	[illegible]
RPL10a	Ribosomal protein L10a, component of cytosolic 80S ribosome and 60S large subunit	-	+	+	-	-	-	GB19558-PA	[illegible]
RPL10	Ribosomal protein L10, component of cytosolic 80S ribosome and 60S large subunit	-	+	+	-	-	-	GB14595-PA	[illegible]
	EMB30/GNOM-like ARF-GEF	-	+	-	-	-	-	GB12561-PA	[illegible]
	RabGAP/TBC Domain Protein	-	+	+	-	-	-	GB17762-PA	[illegible]
	RabGAP/TBC Domain Protein	-	+	-	-	-	-	-	[illegible]
FABL1	FAB-like, FYVE-domain PI-3,4-kinase	-	+	+	-	-	-	GB10702-PA	[illegible]
VPS53	subunit of GARP complex	-	+	+	-	-	-	GB14279-PA	[illegible]
CHLM	Magnesium protoporphrin IX S-adenosyl methionine O-methyl transferase, chloroplast precursor	-	+	-	-	-	-	-	[illegible]
GTS2	non descriminatory Gln-Glu-tRNA synthetase, putative	-	+	-	-	-	-	GB16504-PA	[illegible]
VDR1	violaxanthin de-epoxidase related, chloroplast	-	+	-	-	-	-	-	[illegible]
VPS39	subunit of VPS-C complex	-	+	-	-	-	-	GB13254-PA	[illegible]
VPS23	subunit of the ESCRT-I complex	-	+	-	-	-	-	GB17037-PA	[illegible]
VPS22	subunit of ESCRT-II complex	-	+	+	-	-	-	GB14733-PA	[illegible]
VPS2B	subunit of the ESCRT-III complex	-	+	-	-	-	-	-	[illegible]
Cds1	mitochondrial half-size ABC transporter, membrane protein	-	+	-	-	-	-	GB14017-PA	[illegible]
FNR	Ferredoxin-NADP reductase, chloroplast	-	-	-	-	-	-	-	[illegible]
FNR	Ferredoxin-NADP reductase, chloroplast	-	-	-	-	-	-	-	[illegible]
FNR	Ferredoxin-NADP reductase, chloroplast	-	-	-	-	-	-	-	[illegible]
FNR	Ferredoxin-NADP reductase, chloroplast	-	+	-	-	-	-	-	[illegible]
GMT1	GDP-Mannose transporter, Golgi apparatus	-	-	-	-	-	-	-	[illegible]
GMT1	GDP-Mannose transporter, Golgi apparatus	-	+	-	-	-	-	-	[illegible]
PEX5	peroxisomal targeting signal 1 receptor	-	+	-	-	-	-	GB19731-PA	[illegible]
	putative lysine N-methylase, probably chloroplastic	-	+	-	-	-	-	-	[illegible]
SCR1	Scramblase	-	-	-	-	-	-	-	[illegible]
	possible interference with METM	-	-	-	-	-	-	-	[illegible]
	possible interference with estExt_fgenesh2_pg.C_70372	-	-	-	-	-	-	-	[illegible]
VPS29	subunit of Retromer complex	-	+	+	-	-	-	GB11437-PA	[illegible]
GTS1	Gln-Glu (Glx) non discriminatory tRNA synthetase, chloroplast precursor	-	+	+	-	-	-	GB12123-PA	[illegible]
VPS35	subunit of Retromer complex	-	+	+	-	-	-	GB15658-PA	[illegible]
CLH	Chlorophyllase I, chloroplast precursor putative	-	-	-	-	-	-	-	[illegible]
VPS18	subunit of the VPS-C complex	-	+	-	-	-	-	GB10262-PA	[illegible]
ARLP3	ARF-like GTPase	-	-	-	-	-	-	-	[illegible]
ERG1	ERA-like, small Ras-type GTPase	-	+	-	-	-	-	-	[illegible]
BBSL1	BBS3-like, ARF-like small GTPase	-	-	-	-	-	-	-	[illegible]
ARLC1	ARLC1, small Arf-like-realted GTPase	-	+	-	-	-	-	GB18495-PA	[illegible]
ARLB1	ARLB1, small Arf-like-related GTPase	-	-	-	-	-	-	-	[illegible]
ARLP1	ARF-like GTPase	-	+	-	-	-	-	GB13258-PA	[illegible]
ARFC1	ARFC1, small Arf-related GTPase	-	-	-	-	-	-	-	[illegible]
	putative Fructose 2-6 bisphosphatase or Phosphoglycerate mutase	-	+	-	-	-	-	-	[illegible]
CHLH1	CHLH Magnesium chelatase subunit H, chloroplast precursor	-	+	-	-	-	-	-	[illegible]
RAB28	RAB28, small Rab-related GTPase	-	-	-	-	-	-	-	[illegible]
RABH1	RABH1, small Rab-related GTPase	-	+	-	-	-	-	GB11363-PA	[illegible]
RABG1	RABG1, small Rab-related GTPase	-	+	+	-	-	-	GB13451-PA	[illegible]
RABE1	RABE1, small Rab-related GTPase	-	+	-	-	-	-	GB17826-PA	[illegible]
RABA1	RABA1, small Rab-related GTPase	-	+	+	-	-	-	GB17764-PA	[illegible]
RABC1	RABC1, small Rab-related GTPase	-	-	-	-	-	-	-	[illegible]
RAB23	RAB23, small Rab-related GTPase	+	+	-	-	-	-	-	[illegible]
SEC6	Component of the Exocyst Complex	-	+	-	-	-	-	-	[illegible]
TOM40	40 kDa Translocon at the outer-envelope-membrane of mitochondria	-	-	-	-	-	-	-	[illegible]
TOC75	75 kDa Translocon at the outer-envelope-membrane of chloroplasts	-	+	-	-	-	-	-	[illegible]
VPS41	vacuolar assembly protein	-	+	-	-	-	-	GB16155-PA	[illegible]
ERD2C	KDEL Receptor-like	-	-	-	-	-	-	-	[illegible]
	ARF GTPase-activating domain-containing protein	-	+	-	-	-	-	GB13475-PA	[illegible]
FKB16-2	FKBP-type peptidyl-prolyl cis-trans isomerase	-	-	-	-	-	-	-	[illegible]
SEC12	regulator of COP-II vesicle coat	-	-	-	-	-	-	-	[illegible]
FKB16-2	FKBP-type peptidyl-prolyl cis-trans isomerase	-	-	-	-	-	-	-	[illegible]
OEP80	chloroplast outer envelope protein	-	+	-	-	-	-	-	[illegible]
	expressed protein of unknown funtion	-	-	-	-	-	-	-	[illegible]
	partially conserved protein of unknown function	-	-	-	-	-	-	-	[illegible]
	partially conserved protein of unknown function	-	-	-	-	-	-	-	[illegible]
SGA1	Putative serine glyoxylate aminotransferase, variant SGA1b	-	-	-	-	-	-	-	[illegible]
AP1B1	C-term fragment of AP1B1, Beta-Adaptin	-	-	-	-	-	-	-	[illegible]
	Zn-finger protein	-	-	-	-	-	-	-	[illegible]
	Zn-finger protein	-	+	-	-	-	-	GB11704-PA	[illegible]
SRP9	Subunit of the Signal Recognition Particle	-	+	-	-	-	-	GB17230-PA	[illegible]
SRRA1	Alpha Subunit of the SRP Receptor	-	+	+	-	-	-	GB13324-PA	[illegible]
	conserved protein of unknown function	-	-	-	-	-	-	-	[illegible]
VCL1	vacuoleless, VPS16-homologue	-	+	-	-	-	-	GB10207-PA	[illegible]
VSR1	Putative Vacuolar Sorting Receptor	-	+	-	-	-	-	-	[illegible]
VPS45	SM/Sec1-family protein	-	+	+	-	-	-	GB30528-PA	[illegible]
VPS33	SM/Sec1-family protein	-	+	-	-	-	-	GB15752-PA	[illegible]
SEC1	SM/Sec1-family protein	-	+	-	-	-	-	-	[illegible]
TML1	R-SNARE, Tomsyn-like family	-	+	-	-	-	-	-	[illegible]
VMPL2	R-SNARE, VAMP-like family	-	-	-	-	-	-	-	[illegible]
VMPL1	R-SNARE, VAMP-like family	-	-	-	-	-	-	-	[illegible]
YKT6	R-SNARE, YKT6-family	-	+	+	-	-	-	GB12321-PA	[illegible]
GOS1	Qb-SNARE, Gos1/GS28-family	-	+	-	-	-	-	GB11495-PA	[illegible]
MEMB1	Qb-SNARE, Bos1/Membrin family	-	+	-	-	-	-	GB14211-PA	[illegible]
SEC20	Qb-SNARE, Sec20-family	-	+	-	-	-	-	-	[illegible]
AP4E1	Epsilon-Adaptin	-	+	-	-	-	-	-	[illegible]
AP4S4	Sigma4-Adaptin	-	+	-	-	-	-	-	[illegible]
AP4M4	Mu4-Adaptin	-	+	-	-	-	-	-	[illegible]
CYN1	cyclophilin, bacterial type	-	-	-	-	-	-	-	[illegible]
CYN1	cyclophilin, bacterial type	-	-	-	-	-	-	-	[illegible]
AP4B4	Beta-4-Adaptin	-	+	+	-	-	-	GB16141-PA	[illegible]
AP2S2	Sigma2-Adaptin	-	+	+	-	-	-	GB20047-PA	[illegible]
AP2A1	Alpha-Adaptin	-	+	+	-	-	-	GB16637-PA	[illegible]
SEC31	COP-II coat subunit	-	+	+	-	-	-	GB12808-PA	[illegible]
SEC24B	putative COP-II coat subunit	-	-	-	-	-	-	-	[illegible]
SEC24A	COP-II coat subunit	-	+	+	-	-	-	GB19737-PA	[illegible]
COPZ2	putative Zeta2-COP	-	-	-	-	-	-	-	[illegible]
COPD1	Delta-COP	-	+	+	-	-	-	GB17619-PA	[illegible]
COPB1	Beta-COP	-	+	+	+	-	-	GB13119-PA	[illegible]
COPA1	alpha-COP	-	+	+	-	-	-	GB14108-PA	[illegible]
DRP4C	Dynamin-related GTPase	-	-	-	-	-	-	-	[illegible]
DRP4A	DRP4A, Dynamin-related GTPase	-	-	-	-	-	-	-	[illegible]
CYCB1	B-type cyclin	-	-	-	-	-	-	-	[illegible]
CYCB1	B-type cyclin	-	+	+	-	-	-	GB14560-PA	[illegible]
DRP4B	Dynamin-related GTPase	-	-	-	-	-	-	-	[illegible]
DRP1	Dynamin-related GTPase	-	-	-	-	-	-	-	[illegible]
DRP3	Dynamin-related GTPase	-	+	+	-	-	-	GB15728-PA	[illegible]
DRP5B	Dynamin-related GTPase	-	+	-	-	-	-	-	[illegible]
DRP2	Dynamin-related GTPase	-	-	-	-	-	-	-	[illegible]
CLPC-like	chaperone, Hsp100 family	-	-	-	-	-	-	-	[illegible]
HSP101	ClpB chaperone, Hsp100 family	-	+	-	-	-	-	-	[illegible]
CLPB1	ClpB chaperone, Hsp100 family	-	+	-	-	-	-	GB10557-PA	[illegible]
CLPC1	ClpC chaperone, Hsp100 family	-	-	-	-	-	-	-	[illegible]
CLPD	ClpD chaperone, Hsp100 family	-	+	-	-	-	-	-	[illegible]
CHC	Clathrin Heavy Chain	-	+	+	-	-	-	GB19359-PA	[illegible]
SYP3	Qa-SNARE, Sed5/Syntaxin5-family	-	+	+	-	-	-	GB18673-PA	[illegible]
SYP8	Qa-SNARE, Ufe1/Syntaxin 18 family	-	+	-	-	-	-	GB15087-PA	[illegible]
VAMP75	R-SNARE, VAMP7-family	-	+	-	-	-	-	GB13035-PA	[illegible]
SNAP34	Qb+c-SNARE, SNAP25-family	-	+	-	-	-	-	-	[illegible]
SYP2	Qa-SNARE, Pep12/Syntaxin 7-family	-	-	-	-	-	-	-	[illegible]
SYP72	Qc-SNARE, SYP7-family	-	+	-	-	-	-	-	[illegible]
SYP71	Qc-SNARE, SYP7-family	-	+	-	-	-	-	-	[illegible]
VAMP73	R-SNARE, VAMP7-family	-	-	-	-	-	-	-	[illegible]
VAMP72	R-SNARE, VAMP7-family	-	+	-	-	-	-	-	[illegible]
SYP4	Qa-SNARE, Tlg2/Syntaxin16-family	-	+	+	-	-	-	GB14922-PA	[illegible]
		-	-	-	-	-	-	-	[illegible]
	unknwon function	-	-	-	-	-	-	-	[illegible]
ATP12	Assembly factor 2 for mitochondrial ATP synthase F1 complex	-	-	-	-	-	-	-	[illegible]
		-	-	-	-	-	-	-	[illegible]
HTB30	Histone H2B	-	-	-	-	-	-	-	[illegible]
		-	-	-	-	-	-	-	[illegible]
		-	-	-	-	-	-	-	[illegible]
		-	-	-	-	-	-	-	[illegible]
ADCY3	adenylate cyclase	-	-	-	-	-	-	-	[illegible]
HTB29	Histone H2B	-	-	-	-	-	-	-	[illegible]
HFO29	Histone H4	-	-	-	-	-	-	-	[illegible]
		-	-	-	-	-	-	-	[illegible]
HTB28	Histone H2B	-	-	-	-	-	-	-	[illegible]
		-	-	-	-	-	-	-	[illegible]
		-	-	-	-	-	-	-	[illegible]
		-	-	-	-	-	-	-	[illegible]

		-	-	-	-	-	-	-	-	-	-	-	104541	124335	397109	-	-	-	-	-	-	-	-	-	-	-	-	-	-	-	-	-	-	-	-	-	-	-	-	-	-	-	-	-	-	-	-	-	-	-	-	-
		-	-	-	-	-	-	-	-	-	-	-	-	124237	-	-	-	-	-	-	-	-	-	-	-	-	-	-	-	-	-	-	-	-	-	-	-	-	-	-	-	-	-	-	-	-	-	-	-	-	-	-
		-	-	-	-	-	-	-	-	-	-	-	-	124221	-	-	-	-	-	-	-	-	-	-	-	-	-	-	-	-	-	-	-	-	-	-	-	-	-	-	-	-	-	-	-	-	-	-	-	-	-	-
		-	-	-	-	-	-	-	-	-	-	-	-	124220	-	-	-	-	-	-	-	-	-	-	-	-	-	-	-	-	-	-	-	-	-	-	-	-	-	-	-	-	-	-	-	-	-	-	-	-	-	-
HFO25	Histone H4	-	-	-	-	-	-	-	-	-	-	-	-	-	-	-	-	-	-	-	-	-	-	-	-	-	-	-	-	-	-	-	-	-	-	-	-	-	-	-	-	-	-	-	-	-	-	-	-	-	-	-
		-	-	-	-	-	-	-	-	-	-	-	-	124134	-	-	-	-	-	-	-	-	-	-	-	-	-	-	-	-	-	-	-	-	-	-	-	-	-	-	-	-	-	-	-	-	-	-	-	-	-	-
		-	-	-	-	-	-	-	-	-	-	-	-	124110	-	-	-	-	-	-	-	-	-	-	-	-	-	-	-	-	-	-	-	-	-	-	-	-	-	-	-	-	-	-	-	-	-	-	-	-	-	-
		-	-	-	-	-	-	-	-	-	-	-	-	124081	-	-	-	-	-	-	-	-	-	-	-	-	-	-	-	-	-	-	-	-	-	-	-	-	-	-	-	-	-	-	-	-	-	-	-	-	-	-
		-	-	-	-	-	-	-	-	-	-	-	-	124010	-	-	-	-	-	-	-	-	-	-	-	-	-	-	-	-	-	-	-	-	-	-	-	-	-	-	-	-	-	-	-	-	-	-	-	-	-	-
		-	-	-	-	-	-	-	-	-	-	-	-	123901	-	-	-	-	-	-	-	-	-	-	-	-	-	-	-	-	-	-	-	-	-	-	-	-	-	-	-	-	-	-	-	-	-	-	-	-	-	-
		-	-	-	-	-	-	-	-	-	-	-	-	123900	-	-	-	-	-	-	-	-	-	-	-	-	-	58375	-	-	75746	-	-	-	-	-	-	-	-	-	-	-	-	-	SINFRUP00000137853	-	-	-	-	-	-	-	-
		-	-	-	-	-	-	-	-	-	-	-	-	123879	-	-	-	-	-	-	-	-	-	-	-	-	-	-	-	-	-	-	-	-	-	-	-	-	-	-	-	-	-	-	-	-	-	-	-	-	-	-
		-	-	-	-	-	-	-	-	-	-	CE03157	-	123824	-	-	-	-	-	-	-	-	-	-	-	-	-	-	-	-	-	-	-	-	-	-	-	-	-	-	-	-	-	-	-	-	-	-	-	-	-	-
		-	-	-	-	-	-	-	-	-	-	-	-	123820	-	-	-	-	-	-	-	-	-	-	-	-	-	-	-	-	-	-	-	-	-	-	-	-	-	-	-	-	-	-	-	-	-	-	-	-	-	-
		-	-	-	-	-	-	-	-	-	-	-	-	123806	-	-	-	-	-	-	-	-	-	-	-	-	-	-	-	-	-	-	-	-	-	-	-	-	-	-	-	-	-	-	-	-	-	-	-	-	-	-
		-	-	-	-	-	-	-	-	-	-	-	-	123804	-	-	-	-	-	-	-	-	-	-	-	-	-	-	-	-	-	-	-	-	-	-	-	-	-	-	-	-	-	-	-	-	-	-	-	-	-	-
		-	-	-	-	-	-	-	-	-	-	-	-	123768	-	-	-	-	-	-	-	-	-	-	-	-	-	-	-	-	-	-	-	-	-	-	-	-	-	-	-	-	-	-	-	-	-	-	-	-	-	-
		-	-	-	-	-	-	-	-	-	-	-	-	123759	-	-	-	-	-	-	-	-	-	-	-	-	-	-	-	-	-	-	-	-	-	-	-	-	-	-	-	-	-	-	-	-	-	-	-	-	-	-
		-	-	-	-	-	-	-	-	-	-	-	-	123720	-	-	-	-	-	-	-	-	-	-	-	-	-	-	-	-	-	-	-	-	-	-	-	-	-	-	-	-	-	-	-	-	-	-	-	-	-	-
		-	-	-	-	-	-	-	-	-	-	-	-	123703	-	-	-	-	-	-	-	-	-	-	-	-	-	-	-	-	-	-	-	-	-	-	-	-	-	-	-	-	-	-	-	-	-	-	-	-	-	-
		-	+	-	+	-	-	GB16437-PA	AT1G29900.1	20552	BDEG_05044	CE03105	177358	123700	251474	-	CML055C	OTTDARP00000005939	DDB0167010	CG18572-PA	-	-	ENSGALP00000026615	-	OTTHUMP00000122585	LmjF16.0590	190512	17284	59636	189113	NCU08287	Os01g38970.1	269	-	24195	180534	109206	PF13_0044	229687	RO3G_15064.1	YJL130C	SPAC22G7.06c	GLEAN3_21404	SINFRUP00000168120	-	40323	TA02545	33.m00007	29177	-	Tb927.5.3800	-
		-	-	-	-	-	-	-	-	-	-	-	-	123698	-	-	-	-	-	-	-	-	-	-	-	-	-	-	-	26571	-	-	-	-	-	-	-	-	-	-	-	-	-	-	-	-	-	-	-	-	-	-
HTR23	Histone H3	-	-	-	-	-	-	-	-	-	-	-	-	-	-	-	-	-	-	-	-	-	-	-	-	-	-	-	-	-	-	-	-	-	-	-	-	-	-	-	-	-	-	-	-	-	-	-	-	-	-	-
		-	+	-	-	-	-	GB17377-PA	AT5G39410.1	11729	-	CE32871	155386	123659	214091	cgd4_700	-	OTTDARP00000024112	-	CG2604-PC	19069400	-	ENSGALP00000017293	-	OTTHUMP00000038085	LmjF28.0380	222130	37312	46157	179729	NCU07563	Os07g40620.1	36499	-	32292	61612	158117	PFB0880w	556357	RO3G_05771.1	-	-	-	SINFRUP00000160381	-	23081	-	33.m01355	63283	-	-	UM04426.1
HFO24	Histone H4	-	-	-	-	-	-	-	-	-	-	-	-	-	-	-	-	-	-	-	-	-	-	-	-	-	-	-	-	-	-	-	-	-	-	-	-	-	-	-	-	-	-	-	-	-	-	-	-	-	-	-
HTB23	Histone H2B	-	-	-	-	-	-	-	-	-	-	-	-	-	-	-	-	-	-	-	-	-	-	-	-	-	-	-	-	-	-	-	-	-	-	-	-	-	-	-	-	-	-	-	-	-	-	-	-	-	-	-
		-	+	-	-	-	-	-	-	-	-	CE19681	-	123624	276020	-	-	-	-	-	-	-	-	-	-	-	135743	-	-	-	-	-	33529	GSPATP00010793001	-	-	-	-	-	-	-	-	GLEAN3_00652	SINFRUP00000134202	-	-	-	-	-	-	Tb11.02.5220	-
		-	-	-	-	-	-	-	-	-	-	-	-	123610	-	-	-	-	-	-	-	-	-	-	-	-	-	-	-	-	-	-	-	-	-	-	-	-	-	-	-	-	-	-	-	-	-	-	-	-	-	-
		-	-	-	-	-	-	-	-	-	-	-	-	123594	-	-	-	-	-	-	-	-	-	-	-	-	-	-	-	-	-	-	-	-	-	-	-	-	-	-	-	-	-	-	-	-	-	-	-	-	-	-
		-	-	-	-	-	-	-	-	-	-	-	-	123592	-	-	-	-	-	-	-	-	-	-	-	-	-	-	-	-	-	-	-	-	-	-	-	-	-	-	-	-	-	-	-	-	-	-	-	-	-	-
		-	-	-	-	-	-	-	-	-	-	-	-	123590	-	-	-	-	-	-	-	-	-	-	-	-	-	-	-	-	-	-	-	-	-	-	-	-	-	-	-	-	-	-	-	-	-	-	-	-	-	-
		-	+	-	-	-	-	-	-	-	-	-	-	123583	-	-	-	-	-	-	-	-	-	-	-	LmjF36.3430	-	-	-	-	NCU02263	-	-	-	-	-	-	-	-	RO3G_02126.1	-	-	GLEAN3_12255	-	-	-	-	-	-	-	-	UM01385.1
		-	-	-	-	-	-	-	-	-	-	-	-	123582	-	-	-	-	-	-	-	-	-	-	-	-	-	-	-	-	-	-	-	-	-	-	-	-	-	-	-	-	-	-	-	-	-	-	-	-	-	-
		-	+	-	-	-	-	GB12346-PA	AT5G22920.1	-	BDEG_07738	-	144246	123572	298819	cgd1_2590	-	OTTDARP00000015983	DDB0204548	CG16947-PA	19069559	-	ENSGALP00000037629	GL50803_103659	OTTHUMP00000160598	-	194257	18535	3530	237629	NCU06754	Os03g05270.2	-	GSPATP00027809001	12877	199440	132206	-	558319	-	-	SPAC2F3.16	-	-	87.m00163	33603	-	44.m02596	-	-	-	-
		-	+	-	-	-	-	GB11604-PA	AT1G09280.1	-	-	CE05303	223070	123571	-	-	-	OTTDARP00000023411	DDB0216713	-	-	-	-	-	OTTHUMP00000163303	-	224587	-	-	210684	NCU06012	Os12g37630.1	-	-	-	153012	127094	-	240340	RO3G_03746.1	YOR280C	SPAC22A12.06c	GLEAN3_01130	SINFRUP00000130270	-	-	-	-	52561	-	-	UM06281.1
		-	-	-	-	-	-	-	-	-	-	-	-	123568	-	-	-	-	-	-	-	-	-	-	-	-	-	-	-	-	-	-	-	-	-	-	-	-	-	-	-	-	-	-	-	-	-	-	-	-	-	-
RPA70a	Replication protein A 70 kDa DNA-binding subunit, single-strand DNA binding protein (SSB)	-	+	+	-	-	-	GB15735-PA	AT2G06510.1	71962	BDEG_04869	CE04405	172916	123566	293956	cgd7_4620	CMC123C	-	DDB0188462	CG9633-PA	19069311	234.m00044	ENSGALP00000004840	-	OTTHUMP00000115691	LmjF28.1820	208727	451	45814	171280	NCU03606	Os05g02040.1	26905	GSPATP00011502001	14457	11886	108438	PFD0470c	646356	RO3G_12935.1	YAR007C	SPBC660.13c	GLEAN3_01822	SINFRUP00000148081	7.m00532	263415	TA15495	46.m02906	50671	87866.m00024	Tb11.01.0870	UM05156.1
		-	+	-	-	-	-	-	AT4G00560.1	-	-	-	100199	123563	-	-	CMC049C	OTTDARP00000023901	DDB0206398	-	-	-	ENSGALP00000002680	-	OTTHUMP00000160917	-	171179	-	-	224653	NCU01177	Os02g57990.1	37604	-	-	159664	141102	-	244358	RO3G_01016.1	-	-	GLEAN3_03827	SINFRUP00000150935	-	4064	-	-	25402	92700.m00074	-	-
		-	+	-	-	-	-	-	AT1G73110.1	-	-	-	-	123555	-	-	-	-	-	-	-	-	-	-	-	-	-	-	-	-	-	Os04g56320.1	3096	-	-	152870	-	-	178252	-	-	-	-	-	-	-	-	-	-	-	-	-
		-	-	-	-	-	-	-	-	-	-	-	-	123538	-	-	-	-	-	-	-	-	-	-	-	-	-	-	-	-	-	-	-	-	-	-	-	-	-	-	-	-	-	-	-	-	-	-	-	-	-	-
		-	+	-	-	-	-	-	AT4G12910.1	-	-	CE19621	-	123507	-	-	-	-	-	-	-	-	ENSGALP00000011124	-	OTTHUMP00000031778	-	159616	33274	55413	-	-	Os12g15470.3	35835	-	-	136699	-	-	835003	-	-	-	GLEAN3_15939	SINFRUP00000135907	-	-	-	-	-	-	-	-
PUF2	pumilio family (Puf) protein	-	+	+	-	-	-	GB10504-PB	AT2G29140.1	52010	BDEG_01837	CE31253	180493	123505	263768	-	CMR410C	-	DDB0218189	CG9755-PE	19069698	142.m00167	ENSGALP00000026539	-	OTTHUMP00000151186	LmjF33.1150	220913	28935	59906	99756	NCU06511	Os01g62650.1	-	GSPATP00003882001	11337	206830	108468	-	752121	RO3G_04980.1	YLL013C	SPAC1687.22c	GLEAN3_06847	SINFRUP00000160217	73.m00189	32366	-	641.m01474	28798	87778.m00067	Tb10.389.0940	-
		-	+	-	-	-	-	-	AT1G51760.1	52138	-	-	172156	123463	-	-	-	-	DDB0204719	-	-	-	-	-	-	LmjF20.1570	-	1658	-	-	-	Os01g37960.1	-	GSPATP00017078001	13725	158291	-	-	-	-	-	-	-	-	57.m00264	-	-	-	-	82012.m00162	Tb927.1.3000	UM01992.1
		-	-	-	-	-	-	-	-	-	-	-	-	-	-	-	-	-	-	-	-	-	-	-	-	-	-	-	-	-	-	-	-	-	-	-	-	-	-	-	-	-	-	-	-	-	-	-	-	-	-	-
		-	-	-	-	-	-	-	-	-	-	-	-	123451	-	-	-	-	-	-	-	-	-	-	-	-	-	-	-	-	-	-	-	-	-	-	-	-	-	-	-	-	-	-	-	-	-	-	-	-	-	-
		-	-	-	-	-	-	-	-	-	-	-	-	123446	-	-	-	-	-	-	-	-	-	-	-	-	-	-	-	-	-	-	-	-	-	57200	-	-	-	-	-	-	-	-	-	-	-	-	-	-	-	-
PRL2		-	+	-	-	-	-	-	-	-	BDEG_02340	CE29046	-	123440	-	-	-	-	-	-	-	-	-	-	OTTHUMP00000177614	-	-	-	-	177893	NCU05618	Os07g03499.1	-	-	-	-	-	-	-	-	-	-	-	SINFRUP00000179057	-	-	-	-	26844	-	-	-
		-	-	-	-	-	-	-	-	-	-	-	-	123436	-	-	-	-	-	-	-	-	-	-	-	-	-	-	-	-	-	-	-	-	44901	-	-	-	-	-	-	-	-	-	-	23979	-	-	-	-	-	-
ASC200		-	+	+	-	-	-	GB15157-PA	AT1G20960.1	37524	BDEG_03657	CE21971	222545	123427	238542	cgd2_2770	CMQ149C	-	DDB0190760	CG5931-PA	19069147	3.m00609	ENSGALP00000024889	GL50803_9352	OTTHUMP00000161012	LmjF13.0500	189896	36978	37179	158557	NCU02685	Os02g01740.1	1	GSPATP00030936001	21388	205535	117362	PF14_0370	776276	RO3G_02202.1	YER172C	SPAC9.03c	GLEAN3_03407	SINFRUP00000181201	28.m00357	35726	TA18045	42.m03275	51100	87912.m00137	Tb11.02.1930	UM03738.1
		-	+	-	-	-	-	-	AT1G62640.2	18929	-	-	-	123419	-	-	-	-	-	-	-	-	-	-	-	-	-	-	-	-	-	Os04g55060.1	21374	-	-	114893	-	PFB0505c	267168	-	-	-	-	-	-	270141	-	44.m00012	-	-	-	-
		-	-	-	-	-	-	-	-	-	-	-	-	123407	-	-	CMF073C	-	-	-	-	-	-	-	-	-	-	-	-	-	NCU00790	Os06g45940.2	-	-	-	-	-	-	-	-	-	-	-	-	-	-	-	-	-	-	-	-
ANK9	protein of unknown function with ankyrin repeats	-	+	-	-	-	-	GB19788-PA	-	-	BDEG_00463	-	190552	123395	-	cgd6_4480	CMS172C	-	-	-	-	207.m00068	-	GL50803_114671	-	-	134675	-	-	-	-	-	-	-	-	147186	-	-	-	-	-	-	-	-	336.m00019	3580	-	-	-	-	Tb11.02.2520	-
		-	+	-	-	-	-	-	-	17100	-	-	-	123392	-	-	CML117C	-	-	-	-	-	-	-	-	-	-	-	-	-	-	-	-	-	34675	-	-	-	-	-	-	-	-	-	132.m00104	32261	-	-	-	-	-	UM01760.1
		-	+	-	-	-	-	-	AT2G29540.2	-	BDEG_05799	CE00225	127647	123383	233537	-	-	-	DDB0183844	CG10685-PA	19069584	-	ENSGALP00000031703	GL50803_10840	OTTHUMP00000018171	-	125219	-	80573	112100	NCU00419	Os12g09280.1	10132	GSPATP00020004001	12779	129266	142725	-	641125	RO3G_15306.1	YNL113W	SPAC1687.01	-	-	1.m00830	-	TA08625	55.m00281	30263	-	-	UM01184.1
		-	+	-	-	-	-	GB16943-PA	AT4G15880.1	6220	-	CE33694	224463	123346	376368	cgd5_2810	CMM297C	OTTDARP00000011458	DDB0184304	CG11023-PA	-	-	ENSGALP00000010482	-	OTTHUMP00000173885	-	201054	25282	-	158777	-	Os01g25370.1	-	GSPATP00022327001	-	143972	136437	PFL1635w	-	-	YPL020C	SPBC19G7.09	-	SINFRUP00000158748	-	18071	TA07090	33.m01285	-	83472.m00492	-	UM00354.1
COX10	cytochrome c oxidase assembly protein, heme A: farnesyltransferase	-	+	-	-	-	-	GB17609-PA	AT2G44520.1	3278	BDEG_06331	CE35917	19609	123320	248398	-	CMT232C	-	DDB0187813	CG5037-PA	-	-	ENSGALP00000002093	-	OTTHUMP00000064705	LmjF23.1520	64495	16299	63810	238754	NCU06141	Os01g34390.1	17071	GSPATP00032241001	32550	44233	129369	PFE0970w	755787	RO3G_08531.1	YPL172C	SPBC365.02c	GLEAN3_20391	SINFRUP00000140985	39.m00170	15142	TA07535	20.m03941	34046	-	Tb927.5.1310	UM05692.1
		-	+	-	-	-	-	-	AT4G26180.1	-	-	CE10358	187133	123309	-	-	CMO303C	OTTDARP00000021347	DDB0184176	-	-	-	ENSGALP00000006457	-	OTTHUMP00000019707	LmjF36.0510	-	-	-	-	-	Os01g38990.1	4113	-	-	152223	-	-	417761	RO3G_08316.1	-	-	-	SINFRUP00000173926	57.m00206	33941	-	59.m03676	-	-	-	-
MRE11		-	+	+	-	-	-	GB17160-PA	AT5G54260.1	72789	BDEG_02875	CE37459	93010	123286	266253	cgd1_1420	CMB035C	-	DDB0191996	CG16928-PA	19170874	290.m00057	ENSGALP00000035634	-	NP_005582.1	LmjF27.1890	108504	25241	58924	245550	NCU08730	Os04g54340.1	11562	GSPATP00023641001	54699	30474	127889	PFA0390w	582090	RO3G_11820.1	YMR224C	SPAC13C5.07	GLEAN3_28109	SINFRUP00000135805	115.m00109	34332	TA10845	65.m01956	24372	83641.m00179	Tb927.2.4390	UM04704.1
		-	-	-	-	-	-	-	-	-	-	-	-	-	-	-	-	-	-	-	-	-	-	-	-	-	-	-	-	-	-	-	-	-	-	-	-	-	-	-	-	-	-	-	-	-	-	-	-	-	-	-
PDE13	3',5'-cyclic-nucleotide phosphodiesterase	-	-	-	-	-	-	-	-	-	-	-	-	123270	-	-	-	-	-	-	-	-	-	-	-	-	-	-	-	-	-	-	-	-	-	-	-	-	-	-	-	-	-	-	-	-	-	-	-	-	-	-
		-	-	-	-	-	-	-	-	-	-	-	-	123261	-	-	-	-	-	-	-	-	-	-	-	-	-	-	-	-	-	-	-	-	-	-	-	-	-	-	-	-	-	-	-	-	-	-	-	-	-	-
PDE12	3',5'-cyclic-nucleotide phosphodiesterase	-	-	-	-	-	-	-	-	-	-	-	-	123257	-	-	-	-	-	-	-	-	-	-	-	-	-	-	-	-	-	-	-	-	-	-	-	-	-	RO3G_14383.1	-	-	-	-	-	-	-	-	-	-	-	-
		-	+	-	-	-	-	GB30219-PA	-	23536	BDEG_00563	CE24201	82908	123240	-	-	-	-	DDB0189537	-	-	-	ENSGALP00000020947	-	NP_443085.2	-	-	32879	58815	-	NCU05600	-	21437	GSPATP00001374001	14688	-	158003	-	-	-	YPL109C	SPBC21C3.03	-	SINFRUP00000134017	-	2580	-	-	-	-	-	UM00676.1
		-	+	-	-	-	-	GB13335-PA	AT5G37830.1	-	BDEG_02758	CE40315	176070	123233	250090	-	CMP112C	-	DDB0186276	CG4752-PA	-	-	-	-	NP_060040.1	LmjF18.1040	170168	15677	82976	86728	NCU04569	Os01g73514.2	8507	GSPATP00014285001	42276	173479	108268	-	759026	RO3G_09746.1	YKL215C	SPAC11D3.15	GLEAN3_04892	SINFRUP00000156146	1.m00671	21538	-	-	30232	-	-	UM00080.1
		-	+	-	-	-	-	-	AT4G21210.1	-	-	-	57666	123209	-	-	-	-	-	-	-	-	-	-	-	-	-	-	-	-	-	Os07g34640.2	34453	-	49027	216074	-	-	196775	-	-	-	-	-	-	-	-	-	-	-	-	-
SF3b2	Nuclear pre-mRNA splicing factor, component of splicing factor 3b	-	+	+	-	-	-	GB12513-PA	AT4G21660.1	21560	BDEG_04853	CE14540	151106	123204	262175	cgd2_3510	CMT357C	-	DDB0218611	CG3605-PA	19069674	194.m00112	-	GL50803_17126	NP_006833.2	LmjF30.0570	178463	37714	29790	186750	NCU05452	Os02g58090.1	8195	GSPATP00035567001	49469	201031	143586	PF14_0587	817772	RO3G_00658.1	YMR240C	SPAC22F8.10c	GLEAN3_03478	SINFRUP00000168009	1.m00933	269780	TA04540	583.m05633	20192	82952.m00142	Tb927.6.2000	UM05454.1
		-	+	+	-	-	-	GB11156-PA	AT1G77050.1	72308	BDEG_01735	CE37303	148777	123188	270140	cgd3_2330	CMO107C	-	DDB0191735	CG32344-PA	-	32.m00207	ENSGALP00000012094	GL50803_90950	NP_076977.3	LmjF34.2050	127166	20053	29296	92056	NCU07712	Os08g32090.1	740	GSPATP00027460001	26853	30506	138741	-	204718	RO3G_10586.1	YDL031W	SPAC31A2.07c	GLEAN3_07386	SINFRUP00000155902	52.m00254	14109	TA03295	25.m01816	58231	83992.m00178	Tb927.4.2630	-
		-	+	-	-	-	-	-	AT5G42770.1	6675	BDEG_02451	-	-	123186	-	cgd2_4300	CMB088C	-	DDB0218334	-	-	-	-	GL50803_13889	-	LmjF32.1030	-	13421	-	-	-	Os03g12810.1	31283	GSPATP00024135001	5720	181169	-	PFI0310w	243473	-	-	-	-	-	51.m00212	-	-	49.m03212	-	86498.m00515	Tb11.01.5890	-
		-	+	-	-	-	-	-	AT5G13800.1	61225	-	-	-	123169	-	cgd4_4130	-	-	-	-	-	-	-	-	-	-	-	-	-	-	-	Os06g24730.1	15213	-	50641	124591	-	-	821437	-	-	-	-	-	-	-	-	-	-	-	-	-
		-	+	-	-	-	-	-	AT3G09600.2	9668	-	-	-	123164	-	cgd2_3980	CMK043C	-	DDB0188105	-	-	33.m00259	-	-	-	-	-	-	80538	-	-	-	25878	GSPATP00026886001	-	-	138847	-	-	-	-	-	-	-	22.m00220	-	-	-	-	-	-	-
		-	+	-	-	-	-	GB19793-PA	-	11045	-	CE27290	181876	123154	225919	-	-	-	DDB0216780	CG10645-PA	-	189.m00094	ENSGALP00000019276	GL50803_93548	NP_079105.4	-	186156	20946	32896	161656	-	-	-	GSPATP00020915001	-	-	-	-	-	-	-	-	-	SINFRUP00000161315	68.m00164	-	-	-	63473	86603.m00106	Tb927.5.1830	-
		-	+	+	-	-	-	GB11391-PA	AT4G23850.1	37371	BDEG_00974	CE24550	224649	123147	386595	cgd3_640	CME186C	OTTDARP00000019782	DDB0190288	CG3961-PC	19069340	112.m00118	ENSGALP00000014349	GL50803_9062	NP_001986.2	LmjF01.0490	216255	33146	32809	239941	NCU04380	Os01g48910.2	35810	GSPATP00005296001	17720	181177	108547	PFE1250w	817412	RO3G_12701.1	YOR317W	SPBP4H10.11c	GLEAN3_12875	SINFRUP00000165455	39.m00284	29867	TA18970	50.m00056	50825	92066.m00124	Tb09.160.2780	UM03737.1
		-	+	-	-	-	-	-	AT4G31530.1	-	-	-	-	123134	-	-	-	-	-	-	-	-	-	-	-	-	-	-	-	-	-	Os03g23980.1	5700	-	49437	24317	-	-	172209	-	-	-	-	-	-	11970	-	-	-	-	-	-
		-	+	-	-	-	-	-	AT3G05170.1	13634	-	-	-	123133	-	-	-	-	-	-	-	-	-	-	-	LmjF33.2100	-	-	3927	-	NCU01845	Os06g01950.1	5035	-	45200	115780	130100	-	295062	-	YDR051C	-	-	-	12.m00427	17193	-	-	-	-	Tb11.02.0440	-
GDPD4	glycerophosphoryl diester phosphodiesterase family protein	-	-	-	-	-	-	-	-	-	-	-	-	123120	-	-	-	-	-	-	-	-	-	-	-	-	-	-	-	-	-	-	-	-	-	-	-	-	-	-	-	-	-	-	-	-	-	-	-	-	-	-
GTR10	glycosyl transferase	-	-	-	-	-	-	-	-	-	-	-	-	123118	-	-	-	-	-	-	-	-	-	-	-	-	-	-	-	-	-	-	-	-	-	-	-	-	-	-	-	-	-	-	-	-	-	-	-	-	-	-
ADC1	aminodeoxychorismate (ADC) synthase, putative	-	+	-	-	-	-	-	AT2G28880.1	10437	BDEG_01195	-	-	123116	-	-	CMO324C	-	-	-	-	-	-	-	-	-	-	39378	-	-	NCU01210	Os06g48620.1	22317	-	-	190627	123952	PFI1100w	770528	RO3G_06010.1	YNR033W	SPBP8B7.29	-	-	-	261908	-	20.m03753	-	-	-	UM03729.1
		-	-	-	-	-	-	-	-	-	-	-	-	123085	-	-	-	-	-	-	-	-	-	-	-	-	-	-	-	-	-	-	-	-	-	-	-	-	-	-	-	-	-	-	-	3837	-	-	-	-	-	-
		-	+	-	-	-	-	-	-	-	-	-	120927	123083	248570	cgd5_3460	-	OTTDARP00000003855	-	CG11883-PB	-	-	ENSGALP00000025482	-	-	-	197614	-	-	-	NCU07590	-	-	-	-	-	-	-	-	RO3G_12782.1	-	-	GLEAN3_22186	SINFRUP00000157219	7.m00513	-	-	-	-	-	-	UM01485.1
		-	+	-	-	-	-	GB13734-PA	AT1G12370.2	8694	-	-	152478	123069	-	-	CMH170C	-	-	CG11205-PB	-	-	ENSGALP00000012174	-	-	-	125833	37396	-	163561	-	Os10g08580.1	36201	GSPATP00003465001	51952	184349	142011	PFE0675c	581193	-	-	-	-	SINFRUP00000156958	-	40995	-	20.m03950	-	-	-	-
C_660082		-	+	+	-	-	-	GB20125-PA	AT5G41970.1	-	BDEG_03449	CE18880	225005	123048	291392	-	CMS088C	-	DDB0189530	CG11980-PA	19168760	219.m00108	ENSGALP00000022078	GL50803_10858	NP_067653.3	LmjF09.0840	217311	20236	80897	105041	NCU03859	Os02g46150.1	22966	GSPATP00021296001	41745	128158	108501	PFF1295w	830885	RO3G_03268.1	YER156C	SPAC694.04c	-	SINFRUP00000141293	143.m00076	832	TA11240	583.m05666	52107	82414.m00110	Tb11.52.0013	UM06421.1
C_60084		-	+	-	-	-	-	-	AT5G62190.1	895	-	-	52524	123042	-	-	-	-	-	-	-	-	-	-	-	-	-	-	-	-	-	Os09g34910.1	16941	GSPATP00003441001	-	127777	108896	-	-	RO3G_12022.1	-	-	-	-	-	-	-	-	55752	-	Tb927.5.4420	-
MTR4	putative Mtr4 also called DOB1	-	+	+	-	-	-	GB13548-PA	AT1G59760.1	268	BDEG_06845	CE06562	155216	123024	289546	cgd8_2520	CMA072C	OTTDARP00000015060	DDB0167152	CG4152-PA	19068810	131.m00124	ENSGALP00000023673	GL50803_17146	OTTHUMP00000122510	LmjF36.3000	162536	35219	81560	234599	NCU03363	Os11g07500.1	17888	GSPATP00016170001	50326	183907	135412	PFF0100w	297279	RO3G_00987.1	YJL050W	SPAC6F12.16c	GLEAN3_09441	SINFRUP00000143734	31.m00231	34661	TA13610	31.m00908	21387	94472.m00065	Tb10.6k15.3220	UM01484.1
		-	-	-	-	-	-	-	-	-	-	-	-	123022	-	-	-	-	-	-	-	-	-	-	-	-	-	-	-	-	-	-	-	-	-	-	-	-	-	-	-	-	-	-	-	-	-	-	-	-	-	-
HXK2	hexokinase, putative	-	-	-	-	-	-	-	-	-	-	-	-	-	-	-	-	-	-	-	-	-	-	-	-	-	-	-	-	-	-	-	-	-	-	-	-	-	-	-	-	-	-	-	-	-	-	-	-	-	-	-
		-	+	-	-	-	-	-	-	58602	-	-	-	123019	-	-	-	-	DDB0167745	-	-	-	-	-	-	-	-	11190	69138	-	-	-	-	GSPATP00011181001	-	-	131128	-	-	-	-	-	-	-	38.m00288	14555	-	-	-	-	-	UM00497.1
		-	-	-	-	-	-	-	-	-	-	-	-	123015	-	-	-	-	-	-	-	-	-	-	-	-	-	-	-	-	-	-	-	-	-	-	-	-	-	-	-	-	-	-	-	-	-	-	-	-	-	-
ACC	biotin carboxylase	-	-	-	-	-	-	-	AT5G35360.1	-	-	-	-	122970	-	-	CMS299C	-	-	-	-	-	-	-	-	-	-	-	-	-	-	-	-	-	-	116924	-	-	831870	-	-	-	-	-	-	-	-	-	-	-	-	-
		-	-	-	-	-	-	-	-	-	-	-	-	-	-	-	-	-	-	-	-	-	-	-	-	-	-	-	-	-	-	-	-	-	-	-	-	-	-	-	-	-	-	-	-	-	-	-	-	-	-	-
		-	+	-	-	-	-	-	AT5G24330.1	30296	BDEG_00415	-	-	122939	294598	cgd4_4410	-	-	DDB0204941	CG8677-PA	-	-	-	-	NP_065952.1	-	-	-	-	116372	-	Os02g03030.1	-	-	42822	194183	158172	-	422428	-	-	SPBP19A11.06	GLEAN3_05714	SINFRUP00000182328	-	-	TA07395	-	-	-	-	-
		-	+	-	-	-	-	GB17652-PA	AT2G21280.1	-	-	-	223709	122924	212042	-	CML183C	-	DDB0205896	CG8768-PA	-	-	-	-	OTTHUMP00000066750	-	219736	-	49407	161857	-	Os02g58790.1	17435	-	9246	131169	-	-	821134	-	-	-	GLEAN3_01333	SINFRUP00000141805	-	-	-	50.m03099	-	-	-	-
		-	-	-	-	-	-	-	-	-	-	-	-	122922	-	-	-	-	-	-	-	-	-	-	-	-	-	-	-	-	-	-	-	-	50155	-	-	-	-	-	-	-	-	-	-	12594	-	-	-	-	-	-
		-	+	-	-	-	-	-	AT4G01150.1	-	-	-	-	122918	-	-	-	-	-	-	-	-	-	-	-	-	-	-	-	-	-	Os02g49870.2	30062	-	-	114083	-	-	409886	-	-	-	-	-	-	-	-	-	-	-	-	-
		-	+	+	-	-	-	GB11200-PA	AT4G31790.2	29030	BDEG_07361	CE02109	166705	122908	289673	cgd6_1200	CMJ181C	OTTDARP00000009813	DDB0169516	-	19069668	129.m00136	ENSGALP00000008387	GL50803_28666	OTTHUMP00000012524	LmjF31.1610	213693	33663	-	93189	NCU07266	Os03g23970.2	21860	GSPATP00028621001	5236	198807	109732	PF10_0087	410812	RO3G_02815.1	YLR172C	SPCC576.14	-	SINFRUP00000166840	144.m00131	20788	TA03050	50.m03346	-	-	Tb927.4.4650	UM04406.1
		-	-	-	-	-	-	-	-	-	-	-	-	122896	-	-	-	-	-	-	-	-	-	-	-	-	-	-	-	-	-	-	-	-	-	-	-	-	-	-	-	-	-	-	-	-	-	-	-	-	-	-
		-	+	-	-	-	-	-	AT5G65110.2	62072	-	-	222560	122887	-	-	CMK115C	-	DDB0187085	-	-	-	-	-	OTTHUMP00000161929	-	-	-	33147	-	-	Os11g39220.1	-	GSPATP00012079001	-	159691	-	-	832274	-	-	-	-	-	20.m00427	-	-	583.m05685	57233	-	-	-
		-	+	-	-	-	-	GB12099-PA	AT5G63860.1	71888	-	CE38062	172844	122886	392870	cgd6_2770	CMH019C	OTTDARP00000019836	DDB0185474	CG11734-PB	-	44.m00154	ENSGALP00000005334	-	OTTHUMP00000176367	LmjF17.0240	218809	-	34255	170394	-	Os02g34860.1	-	GSPATP00024537001	3182	195710	127329	PF11_0385	718641	-	-	-	GLEAN3_01208	SINFRUP00000142720	116.m00105	38047	TA05090	50.m05637	21647	121196.m00013	Tb927.7.6320	-
ARSA	Putative ArsA	-	+	-	-	-	-	-	AT3G10350.1	30751	-	-	-	122880	-	-	CMP225C	-	-	-	-	-	-	-	-	-	-	-	-	-	-	Os02g51100.1	12285	-	32803	128576	-	-	822576	-	-	-	-	-	-	1704	-	-	-	-	-	-
LIPG2	triacylglycerol lipase (gastric ligase) precursor	-	+	-	-	-	-	GB10537-PA	AT5G14180.1	19589	BDEG_00253	CE18732	5448	122836	211925	-	-	OTTDARP00000021165	DDB0202316	CG6113-PA	-	-	ENSGALP00000010290	-	OTTHUMP00000020038	-	161944	34184	67907	221778	NCU02148	Os08g41780.1	-	GSPATP00018168001	-	121274	108318	PF11_0276	246811	RO3G_06706.1	YKL140W	SPCC1672.09	GLEAN3_00676	-	1.m00926	-	-	83.m01199	54597	-	-	UM03436.1
CPSF1		-	+	+	-	-	-	GB15626-PA	AT1G61010.3	71470	BDEG_00634	CE32993	184406	122831	237575	cgd8_460	CMH020C	OTTDARP00000005726	DDB0217481	CG5222-PA	19069341	33.m00214	ENSGALP00000026443	GL50803_4498	OTTHUMP00000115483	LmjF34.3430	188747	26966	72801	243769	NCU03479	Os03g63590.1	28857	GSPATP00028787001	28482	177452	109560	PF14_0364	577561	RO3G_14511.1	YLR277C	SPAC17G6.16c	-	SINFRUP00000133946	6.m00500	33033	TA05230	76.m01591	19764	95400.m00087	Tb927.4.1340	UM01470.1
		-	+	-	-	-	+	GB13427-PA	-	29643	BDEG_04240	CE12770	226124	122809	296059	-	CMT298C	OTTDARP00000014628	-	CG17337-PA	-	-	ENSGALP00000022230	-	OTTHUMP00000073036	-	224093	37652	60072	183701	NCU07153	-	-	-	18404	-	-	-	-	RO3G_05381.1	YFR044C	SPBC1198.08	GLEAN3_22172	SINFRUP00000133963	-	39315	-	31.m00872	-	-	-	UM06330.1
FAP23	Flagellar Associated Protein Similar to cob(I)alamin adenosyltransferase	-	+	-	-	-	-	GB13846-PA	-	60211	-	CE39476	175326	122771	285683	-	-	OTTDARP00000016283	DDB0218202	-	-	-	ENSGALP00000006146	-	OTTHUMP00000169142	LmjF24.2240	214269	38259	31057	86221	-	-	-	-	45992	-	-	-	-	-	-	-	-	-	-	263198	-	-	32318	88271.m00550	-	-
		-	-	-	-	-	-	-	-	-	-	-	-	-	-	-	-	-	-	-	-	-	-	-	-	-	-	-	-	-	-	-	-	-	-	-	-	-	-	-	-	-	-	-	-	-	-	-	-	-	-	-
CYP743B1	cytochrome P450, unkown function, in CYP711 clan	-	-	-	-	-	-	-	-	-	-	-	-	122749	-	-	-	-	-	-	-	-	-	-	-	-	-	-	-	-	-	-	-	-	-	-	-	-	-	-	-	-	-	-	-	-	-	-	-	-	-	-
HYP2		-	+	+	-	-	-	GB14158-PA	AT5G11900.1	6922	-	CE22051	152798	122741	268060	cgd8_2890	CMH011C	-	DDB0205981	CG9099-PA	-	76.m00160	ENSGALP00000033977	-	OTTHUMP00000169680	LmjF25.0410	222001	-	81951	185620	NCU02984	Os01g09890.1	20006	GSPATP00003519001	31987	211491	133183	PF08_0079	835761	RO3G_14076.1	YJR014W	SPBC16C6.05	GLEAN3_08407	SINFRUP00000136431	50.m00193	10731	TA13120	55.m04672	52695	81350.m00084	Tb11.03.0700	-
	ammonium transporter	-	-	-	-	-	-	-	-	-	-	-	-	122737	-	-	-	-	-	-	-	-	-	-	-	-	-	-	-	-	-	-	-	-	-	-	-	-	-	-	-	-	-	-	-	-	-	-	-	-	-	-
		-	+	+	-	-	-	-	AT1G29630.2	29882	BDEG_00638	CE28728	95129	122732	-	cgd6_1580	CME067C	OTTDARP00000024168	DDB0183988	CG10387-PA	19069601	513.m00039	ENSGALP00000017465	GL50803_9922	NP_006018.3	LmjF23.1270	127400	29551	30993	12162	NCU06089	Os01g56940.1	15130	GSPATP00018289001	48206	114516	140571	PF07_0105	234673	RO3G_00344.1	YOR033C	SPBC29A10.05	GLEAN3_10287	SINFRUP00000162770	265.m00045	269871	TA09295	44.m04661	26121	87363.m00196	Tb927.8.3220	UM03141.1
CYP743B3	cytochrome P450, unkown function, in CYP711 clan	-	-	-	-	-	-	-	-	-	-	-	-	122730	-	-	-	-	-	-	-	-	-	-	-	-	-	-	-	-	-	-	-	-	-	-	-	-	-	-	-	-	-	-	-	-	-	-	-	-	-	-
HSF2	Heat shock transcription factor 2	-	+	-	-	-	-	-	-	71265	-	-	130381	122706	-	-	CMO187C	-	DDB0204052	CG5748-PA	-	-	ENSGALP00000023947	-	-	-	214431	37599	-	-	-	-	-	-	45112	-	-	-	-	-	-	SPAC8C9.14	-	-	-	-	-	-	-	-	-	-
		-	+	-	-	-	-	-	-	-	BDEG_03997	-	-	122702	-	-	-	-	-	-	-	-	-	-	-	-	67491	-	-	-	-	Os01g34700.2	30316	PTETP8500001001	-	-	-	-	732264	-	-	-	-	-	82.m00155	-	-	-	-	-	-	-
		-	-	-	-	-	-	-	-	-	-	-	-	122698	-	-	-	-	-	-	-	-	-	-	-	-	-	-	-	-	-	-	-	-	-	-	-	-	-	-	-	-	-	-	-	-	-	-	-	-	-	-
		-	+	-	-	-	-	-	AT2G05990.2	19845	-	-	136903	122688	-	-	CMT381C	-	-	-	-	-	-	-	-	-	-	-	-	-	-	Os09g10600.1	28591	-	10068	119545	137179	PFF0730c	639726	-	-	-	-	-	-	32860	-	50.m00011	-	-	-	-
		-	+	+	-	-	-	GB14053-PA	AT1G67320.1	29345	BDEG_03986	CE24028	219114	122684	296640	cgd8_1410	CMK284C	-	DDB0191064	CG5553-PA	19170867	51.m00142	ENSGALP00000036225	GL50803_6980	NP_000938.2	LmjF03.0090	128625	32834	70379	179624	NCU04646	Os07g22400.2	29082	GSPATP00034409001	46806	227680	139888	PFI0530c	712440	RO3G_14812.1	YKL045W	SPAC17D11.06	-	SINFRUP00000154785	21.m00198	37328	TA04485	113.m00800	57927	83412.m00019	Tb10.70.4390	UM03852.1
		-	+	-	-	-	-	-	AT1G78620.1	13936	-	-	-	122683	-	-	CMS030C	-	-	-	-	-	-	-	-	-	-	-	-	-	-	Os01g52864.2	16498	-	10054	139646	-	-	728254	-	-	-	-	-	-	269392	-	-	-	-	-	-
AMT3	ammonia channel	-	-	-	-	-	-	-	-	-	-	-	-	122681	-	-	-	-	-	-	-	-	-	-	-	-	-	-	-	-	-	-	-	-	-	-	-	-	-	-	-	-	-	-	-	-	-	-	-	-	-	-
		-	+	-	-	-	-	-	AT1G06690.1	-	-	-	-	122660	-	-	CMT102C	-	-	-	-	-	-	-	-	-	-	-	-	-	NCU11289	Os07g05000.1	-	-	-	-	-	-	714930	-	-	-	-	-	-	-	-	-	-	-	-	-
	protease-inhibitor-like protein	-	+	-	-	-	-	-	-	-	-	-	103110	122624	281118	cgd4_3550	-	OTTDARP00000010275	-	-	-	-	-	-	-	-	-	-	-	-	-	-	-	-	-	-	-	-	-	-	-	-	-	-	-	-	-	-	-	7530	-	-	-
	galactose kinase	-	+	-	-	-	-	GB12997-PA	AT3G06580.1	-	BDEG_06138	CE36705	220087	122621	248311	-	CMN198C	OTTDARP00000023522	DDB0184231	CG5288-PC	-	349.m00050	ENSGALP00000022349	-	NP_001001556.1	-	206567	33741	-	197607	NCU08687	Os03g61710.1	16820	GSPATP00015163001	-	186065	113901	-	723945	RO3G_06381.1	YBR020W	SPBPB2B2.13	GLEAN3_05226	SINFRUP00000137581	11.m00431	260829	-	44.m02561	51952	97007.m00136	-	UM01962.1
		-	-	-	-	-	-	-	-	-	-	-	-	122617	-	-	-	-	-	-	-	-	-	-	-	-	-	-	-	-	-	-	-	-	-	-	-	-	-	-	-	-	-	-	-	-	-	-	-	-	-	-
		-	+	-	-	-	-	GB13982-PA	AT1G12050.1	11473	BDEG_02184	CE04750	180564	122609	247309	-	CMQ154C	-	DDB0190487	CG14993-PA	-	-	ENSGALP00000010465	-	OTTHUMP00000185572	-	207382	34985	-	241460	NCU05537	Os02g10310.1	-	GSPATP00029655001	50281	137922	108641	-	652386	RO3G_09357.1	-	-	GLEAN3_20609	SINFRUP00000178115	3.m02000	33724	-	-	49733	-	-	UM01423.1
		-	-	-	-	-	-	-	-	-	-	-	-	122591	-	-	-	-	-	-	-	-	-	-	-	-	-	-	-	-	-	-	-	-	-	-	-	-	-	-	-	-	-	-	-	-	-	-	-	-	-	-
		-	-	-	-	-	-	-	AT4G18240.1	-	-	-	-	122579	-	-	-	-	-	-	-	-	-	-	-	-	-	-	-	-	-	Os01g52250.4	-	-	-	111318	-	-	173714	-	-	-	-	-	-	-	-	-	-	-	-	-
MCM8	minichromosome maintenance protein 8	-	+	-	-	-	-	GB17620-PA	AT3G09660.1	-	BDEG_00708	-	101979	122561	288765	-	CMT087C	-	DDB0185320	-	19069605	60.m00145	ENSGALP00000014958	-	OTTHUMP00000030217	LmjF05.0330	207337	9453	60270	40200	-	Os05g38850.1	20481	GSPATP00029613001	52561	20647	116205	-	809823	-	-	-	-	SINFRUP00000150393	205.m00041	261512	-	-	59235	-	Tb10.406.0500	-
		-	+	-	-	-	-	-	AT5G38520.1	-	-	-	-	122551	-	-	-	-	-	-	-	-	-	-	-	-	-	-	-	-	-	Os05g34630.1	35866	-	15323	186114	-	-	266807	-	-	-	-	-	-	-	-	-	-	92258.m00089	-	-
		-	+	-	-	-	-	-	AT5G17630.1	-	-	-	-	122546	-	-	-	-	-	-	-	-	-	-	-	-	-	-	-	-	-	Os08g08840.2	31613	-	-	128449	-	-	242137	-	-	-	-	-	-	-	TA05100	55.m10326	-	-	-	-
SMP9		-	+	+	-	-	-	GB10686-PA	AT4G02840.1	22657	BDEG_05445	CE02065	162674	122530	216977	cgd5_2220	-	-	DDB0204374	CG10753-PA	19068456	31.m00228	ENSGALP00000019299	GL50803_12119	OTTHUMP00000073086	LmjF22.1060	187321	-	33608	235585	NCU00505	Os04g39444.1	32286	GSPATP00023511001	51100	157208	158358	PF11_0266	714948	RO3G_01482.1	YGR074W	SPAC27D7.07c	GLEAN3_12607	SINFRUP00000163287	310.m00032	6600	TA06565	57.m01848	55621	85781.m00219	Tb927.7.3120	UM03142.1
		-	+	-	-	-	-	-	AT5G20250.3	61137	-	-	-	122516	-	-	-	-	-	-	-	-	-	-	-	-	-	-	-	-	NCU02550	Os08g38710.2	17963	-	-	202553	-	-	582552	RO3G_08273.1	-	-	-	-	-	31661	-	-	-	-	-	UM00375.1
	carbohydrate transporter, putative	-	+	-	-	-	-	GB17692-PA	-	-	-	-	183805	122510	-	-	-	OTTDARP00000014933	-	CG8791-PA	-	-	ENSGALP00000025604	-	-	-	-	26357	-	-	-	-	17389	-	21441	-	-	-	-	RO3G_15123.1	-	-	-	SINFRUP00000143039	-	-	-	-	36003	-	-	-
		-	+	-	-	-	-	GB16341-PA	AT4G37880.1	-	BDEG_06102	CE29467	159757	122469	295310	-	CMI164C	-	DDB0217500	CG3295-PA	19068864	-	ENSGALP00000036340	-	OTTHUMP00000161498	-	207069	29612	69056	183725	NCU00898	Os06g38940.2	-	GSPATP00004684001	-	203246	135917	MAL7P1.155	833140	-	YDR255C	SPBC29A3.03c	-	SINFRUP00000137246	298.m00021	-	TA05905	42.m03316	61773	-	-	-
3PIDK1	3-Phosphoinositide Dependent Protein Kinase Homolog 1	-	+	-	-	-	-	GB15780-PA	AT5G04510.1	-	-	-	131028	122463	297924	-	-	-	DDB0186031	-	-	-	ENSGALP00000010345	-	OTTHUMP00000081361	-	111786	-	80266	84740	NCU03571	Os01g65230.1	4818	GSPATP00012278001	-	144576	127180	-	725163	-	YDR490C	SPBC1778.10c	GLEAN3_23401	SINFRUP00000146153	-	-	-	-	31577	81529.m00430	-	-
		-	+	-	-	-	-	-	-	-	-	-	-	122448	-	-	-	-	-	-	-	-	-	-	-	-	-	32345	29467	-	-	-	5838	GSPATP00012822001	-	-	-	-	-	-	-	-	-	-	-	36798	-	-	-	80920.m00074	-	-
		-	+	-	-	-	-	-	AT5G64840.1	28694	-	-	-	122447	-	-	CMI117C	-	-	-	-	-	-	-	-	-	-	-	-	-	-	Os11g39020.1	28165	-	17043	30640	-	-	217877	-	-	-	-	-	-	40554	-	-	-	-	-	-
		-	+	-	-	-	-	GB17684-PA	AT1G36160.1	65524	BDEG_01453	CE36026	150877	122441	238665	cgd8_3680	CMM188C	OTTDARP00000018957	DDB0187917	CG11198-PB	-	-	ENSGALP00000034072	-	NP_942135.1	LmjF31.2970	199489	10823	59984	20079	NCU08535	Os05g22940.1	30336	-	55209	115301	108918	-	830215	RO3G_04977.1	YNR016C	SPAC56E4.04c	GLEAN3_13473	SINFRUP00000155052	-	12234	-	41.m00004	22916	-	Tb927.8.7100	UM04629.1
		-	+	-	-	-	-	GB16978-PA	AT4G36400.2	54174	BDEG_08395	CE26814	166406	122423	286093	-	CMR349C	-	DDB0201710	CG3835-PA	-	-	ENSGALP00000010244	-	NP_689996.4	LmjF23.0810	182587	25135	69307	112243	NCU06441	Os07g08950.2	15080	GSPATP00036992001	20192	113231	108284	-	219447	RO3G_15216.1	YDL178W	SPBC713.03	-	SINFRUP00000129731	33.m00339	42965	-	-	60696	-	Tb927.6.1500	UM01314.1
		-	-	-	-	-	-	-	-	-	-	-	-	122396	-	-	-	-	-	-	-	-	-	-	-	-	-	-	-	-	-	-	-	-	-	-	-	-	-	-	-	-	-	-	-	-	-	-	-	-	-	-
		-	-	-	-	-	-	-	-	-	-	-	-	122385	-	-	-	-	DDB0188116	-	-	-	ENSGALP00000039331	-	OTTHUMP00000020279	-	-	-	-	-	-	-	-	-	-	-	-	-	-	-	-	-	-	-	-	-	-	-	33358	-	-	-
		-	+	+	-	-	-	GB14313-PA	AT3G16090.1	1818	BDEG_01639	CE05945	174092	122373	246686	cgd8_2560	CMG190C	OTTDARP00000016923	DDB0187676	CG5140-PA	19068477	125.m00093	ENSGALP00000004897	-	NP_115807.1	LmjF15.1410	119031	24008	65623	32018	NCU04633	Os06g19680.2	29041	GSPATP00010782001	49801	2973	144577	PF14_0215	774007	RO3G_14809.1	YOL013C	SPBC17D11.02c	GLEAN3_28739	SINFRUP00000175611	101.m00130	24902	TA02935	540.m00334	30090	85745.m00095	Tb09.160.3780	UM00542.1
		-	-	-	-	-	-	-	-	-	-	-	-	122372	-	-	-	-	-	-	-	-	-	-	-	-	-	-	-	-	-	-	-	-	-	-	-	-	-	-	-	-	-	-	-	-	-	-	-	-	-	-
		-	+	-	-	-	-	GB10006-PA	AT1G04690.1	-	BDEG_00637	-	159286	122352	285095	-	-	OTTDARP00000014012	-	CG32688-PA	-	-	ENSGALP00000001339	-	OTTHUMP00000000875	-	153606	-	73544	166341	NCU02887	Os02g57240.1	-	GSPATP00034004001	-	185360	108826	-	814875	RO3G_06898.1	YPL088W	SPCC965.06	-	SINFRUP00000167043	178.m00076	-	-	-	27180	-	-	UM06301.1
		-	+	-	-	-	-	-	AT2G47490.1	-	BDEG_05724	CE02342	-	122344	-	-	CMK164C	OTTDARP00000022874	DDB0219583	-	-	-	-	-	NP_001098117.1	LmjF35.3330	-	39198	30038	196580	NCU08941	Os05g28870.1	35550	GSPATP00011480001	13538	110150	129848	-	572659	RO3G_10839.1	YEL006W	SPAC227.03c	-	SINFRUP00000133695	94.m00153	38111	-	-	-	-	-	UM01495.1
		-	-	-	-	-	-	-	-	-	-	-	-	122343	-	-	-	-	-	-	-	-	-	-	-	-	-	-	-	-	-	-	-	-	-	-	-	-	-	-	-	-	-	-	-	-	-	-	-	-	-	-
		-	-	-	-	-	-	-	-	-	-	-	-	122334	-	-	-	-	-	-	-	-	-	-	-	-	-	-	-	-	-	-	-	-	-	-	-	-	-	-	-	-	-	-	-	-	-	-	-	-	-	-
		-	+	-	-	-	-	GB16566-PA	AT2G16570.1	-	BDEG_05649	CE01074	18069	122298	-	-	CMR240C	OTTDARP00000011171	DDB0167867	CG2867-PA	-	-	ENSGALP00000022273	-	OTTHUMP00000159062	-	228583	29823	-	248582	NCU04216	Os01g65260.1	35723	-	1840	182238	108750	-	195747	RO3G_13068.1	YMR300C	SPAC4D7.08c	GLEAN3_03803	SINFRUP00000166233	-	39075	-	-	25641	-	-	UM06035.1
		-	-	-	-	-	-	-	-	-	-	-	-	122295	221053	-	-	-	-	-	-	-	-	-	-	-	-	-	-	-	-	-	-	-	-	-	-	-	-	-	-	-	-	-	-	-	-	-	-	-	-	-
		-	+	-	-	-	-	GB16891-PA	AT4G03020.1	-	-	CE23620	225127	122289	-	-	CMR161C	-	DDB0184092	CG9945-PA	-	-	-	-	OTTHUMP00000164696	-	124623	1381	1490	89550	NCU02151	Os05g05210.2	-	-	-	124836	125661	-	572777	-	-	-	-	SINFRUP00000132783	20.m00409	-	-	-	59895	-	-	UM00687.1
		-	+	-	-	-	-	-	AT2G04360.1	-	-	-	-	122264	-	-	-	-	-	-	-	-	-	-	-	-	-	-	-	-	-	Os08g25700.1	-	-	33845	5635	-	-	732486	-	-	-	-	-	-	1022	-	-	-	-	-	-
MRPL30	Putative mitochondrial ribosomal protein L30	-	+	-	-	-	-	-	AT5G55140.1	-	-	-	-	122263	-	-	-	-	-	-	-	-	-	-	-	-	-	-	-	131067	-	Os08g01859.1	34023	-	-	190638	-	-	552748	-	-	-	-	-	-	-	-	-	-	-	-	-
		-	+	-	-	-	-	GB20143-PA	AT1G80480.1	64741	-	-	-	122261	-	cgd8_890	-	-	DDB0204544	-	-	-	ENSGALP00000016478	-	-	-	-	-	69859	-	NCU03781	Os02g55630.1	17877	-	41508	31956	137005	PF14_0052	-	-	-	-	-	-	16.m00355	3054	TA18160	-	-	-	-	-
PYK5	pyruvate kinase	-	-	-	-	-	-	-	-	-	-	-	-	122254	-	-	-	-	-	-	-	-	-	-	-	-	-	-	-	-	-	-	-	-	-	-	-	-	-	-	-	-	-	-	-	-	-	-	-	-	-	-
		-	-	-	-	-	-	-	-	-	-	-	-	122243	-	-	-	-	-	-	-	-	-	-	-	-	-	-	-	-	-	-	-	-	-	-	-	-	-	-	-	-	-	-	-	-	-	-	-	-	-	-
		-	-	-	-	-	-	-	AT1G16270.1	-	-	-	-	122213	-	-	-	-	-	-	-	-	-	-	-	-	-	-	-	-	-	Os02g54510.3	-	-	-	106226	-	-	262334	-	-	-	-	-	-	-	-	-	-	-	-	-
		-	-	-	-	-	-	-	-	-	-	-	-	122211	-	-	-	-	-	-	-	-	-	-	-	-	-	-	-	-	-	-	-	-	-	-	-	-	-	-	-	-	-	-	-	-	-	-	-	-	-	-
		-	+	-	-	-	-	-	AT2G40840.1	-	-	-	44581	122203	-	-	CMP352C	-	DDB0185931	-	-	1.m00695	-	GL50803_8978	-	-	-	-	-	-	-	Os07g46790.1	940	-	-	147034	-	-	255128	-	-	-	-	-	-	-	-	-	-	87873.m00084	-	-
PRF1	pyruvate-ferredoxin oxidoreductase	-	+	-	-	-	-	-	-	-	-	-	154655	122198	-	cgd4_690	-	-	-	-	-	5.m00419	-	GL50803_17063	-	-	-	-	-	-	-	-	-	-	-	-	-	-	-	-	-	-	-	-	-	31316	-	-	-	86827.m00425	-	-
GTR5	glycosyl transferase	-	+	+	-	-	-	GB17208-PA	AT1G78800.1	29980	BDEG_00980	CE32364	229017	122195	298994	cgd1_230	CMT168C	-	DDB0168248	CG1291-PA	-	90.m00172	ENSGALP00000022055	-	OTTHUMP00000123474	LmjF34.2420	224255	37048	32499	93273	NCU03503	Os04g49960.1	15723	GSPATP00017608001	22554	222991	108450	-	568797	RO3G_14829.1	YGL065C	SPBC11B10.01	-	SINFRUP00000157154	34.m00276	263193	-	42.m00096	-	93205.m00051	Tb927.4.2230	UM02653.1
		-	+	-	-	-	-	-	AT5G52970.1	6353	-	-	-	122132	-	-	CMP233C	-	-	-	-	-	-	-	-	-	-	-	-	-	-	Os07g34950.1	6354	-	43556	112930	-	-	576713	-	-	-	-	-	-	262367	-	-	-	-	-	-

		-	+	+	-	-	-	GB18087-PA	AT3G12390.1	-	BDEG_07667	CE22740	177200	122124	370864	cgd5_3450	CMT271C	-	DDB0205559	CG8759-PC	19068666	38.m00218	ENSGALP00000000669	-	OTTHUMP00000096203	LmjF04.0770	197780	34333	57099	240229	NCU00635	Os01g71230.2	10373	GSPATP00025183001	48728	114623	108927	PFF1050w	547766	RO3G_03711.1	YHR193C	SPBC25H2.05	GLEAN3_15675	SINFRUP00000127819	26.m00243	39622	TA11185	20.m03918	38286	95537.m00120	Tb09.211.0130	UM05472.1
		-	-	-	-	-	-	-	-	-	-	-	-	122089	221737	-	-	-	-	-	-	-	-	-	-	-	-	-	-	-	-	-	-	-	-	-	-	-	-	-	-	-	-	-	-	-	-	-	-	-	-	-
SMC4	Structural Maintenance of Chromosomes Protein 4 homolog.	-	+	+	-	-	-	GB15940-PA	AT5G48600.1	72033	BDEG_05200	CE03287	224718	122088	259758	cgd7_1850	CME029C	OTTDARP00000021313	DDB0186952	CG11397-PA	19069132	45.m00158	ENSGALP00000032358	GL50803_17465	OTTHUMP00000174800	LmjF21.1330	176437	25081	-	106520	NCU09063	Os05g41750.1	17727	GSPATP00014322001	44165	111974	142411	PFE0450w	577636	RO3G_09980.1	YLR086W	SPBC146.03c	GLEAN3_13617	SINFRUP00000152497	51.m00273	42365	TA11440	44.m02645	50834	95305.m00302	Tb10.70.7470	UM03133.1
PDE25	3',5'-cyclic-nucleotide phosphodiesterase	-	-	-	-	-	-	-	-	-	-	-	-	122025	-	-	-	-	-	CG32498-PL	-	-	-	-	-	-	-	-	-	-	-	-	-	-	-	-	-	-	-	-	-	-	-	-	-	-	-	-	-	-	-	-
		-	+	-	-	-	-	-	AT5G47860.1	-	-	-	-	121991	-	-	CMR396C	-	-	-	-	-	-	-	-	-	-	-	-	-	-	Os04g33280.2	5511	-	3527	173029	-	-	179487	-	-	-	-	-	-	263321	-	-	-	-	-	-
		-	-	-	-	-	-	-	-	-	-	-	-	121983	-	-	-	-	-	-	-	-	-	-	-	-	-	-	-	-	-	-	-	-	-	-	-	-	-	-	-	-	-	-	-	-	-	-	-	-	-	-
		-	+	-	-	-	-	GB12291-PA	AT4G24160.1	22544	BDEG_01149	CE32824	162120	121979	265936	-	CML191C	OTTDARP00000018361	-	CG1882-PB	-	-	ENSGALP00000030511	-	OTTHUMP00000164495	LmjF23.0140	219704	29634	-	125604	NCU06332	Os09g34860.1	-	GSPATP00017732001	54974	131728	157051	PF14_0015	550934	RO3G_02430.1	YGR110W	SPAC6G10.03c	-	SINFRUP00000155355	58.m00149	264297	TA14360	55.m04964	37541	-	Tb927.8.2110	UM06261.1
HAG3406		-	+	-	-	-	-	-	AT1G26220.1	-	-	-	-	121974	-	-	CMV200C	-	-	-	-	-	-	-	-	-	-	-	-	-	-	Os08g01170.1	19015	-	-	72140	-	-	421506	-	-	-	-	-	-	-	-	-	-	-	-	-
		-	-	-	-	-	-	-	-	-	-	-	-	121971	-	-	-	-	-	-	-	-	-	-	-	-	-	-	-	-	-	-	-	-	-	-	-	-	-	-	-	-	-	-	-	-	-	-	-	-	-	-
		-	+	-	-	-	-	-	AT3G17930.1	8479	-	-	-	121963	-	-	CMQ319C	-	-	-	-	-	-	-	-	-	-	-	-	-	-	Os01g59080.1	36206	-	46071	118254	-	-	575028	-	-	-	-	-	-	31402	-	-	-	-	-	-
		-	+	-	-	-	-	-	AT4G19670.2	60444	-	-	-	121942	-	-	-	-	-	-	-	-	-	-	-	-	138098	-	-	-	-	Os09g11170.1	-	-	-	17898	-	-	828666	-	-	-	-	-	-	22784	-	42.m03475	-	85479.m00199	-	-
		-	-	-	-	-	-	-	-	-	-	-	-	121939	-	-	-	-	-	-	-	-	-	-	-	-	-	-	-	-	-	-	-	-	-	-	-	-	-	-	-	-	-	-	-	-	-	-	-	-	-	-
	GTP-binding protein	-	+	-	-	-	-	GB19395-PA	AT1G72660.3	28954	BDEG_00389	CE36094	149543	121923	-	cgd3_130	CMN324C	OTTDARP00000020768	DDB0205772	-	-	-	ENSGALP00000007928	-	OTTHUMP00000065704	-	184295	35181	36239	179334	NCU01183	Os05g28940.1	-	GSPATP00017286001	50671	165484	108433	-	813711	-	-	SPBC354.01	GLEAN3_16886	SINFRUP00000156741	31.m00249	42652	-	-	20516	84316.m00056	-	UM01687.1
		-	-	-	-	-	-	-	-	-	-	-	-	121911	-	-	-	-	-	-	-	-	-	-	-	-	-	-	-	-	-	-	-	-	-	231229	-	-	-	-	-	-	-	-	-	-	-	-	-	-	-	-
HBV1	Histone H2B variant	-	+	-	-	-	-	-	-	-	-	-	-	121902	-	cgd7_1700	-	-	-	-	-	-	ENSGALP00000021743	-	-	-	-	38595	83327	118965	-	-	-	-	-	59905	-	PF07_0054	-	-	-	-	-	-	-	-	TA07290	25.m00008	27729	96280.m00144	-	-
		-	-	-	-	-	-	-	-	-	-	-	-	121889	-	-	-	-	-	-	-	-	-	-	-	-	-	-	-	-	-	-	-	-	-	-	-	-	-	-	-	-	-	-	-	-	-	-	-	-	-	-
		-	+	-	-	-	-	-	AT2G40860.1	-	-	-	-	121874	-	-	CMG113C	-	-	-	-	-	-	-	-	-	-	-	-	-	-	Os01g36080.2	20474	-	-	106079	-	-	835373	-	-	-	-	-	-	-	-	-	-	-	-	-
		-	-	-	-	-	-	-	-	-	-	-	-	121864	-	-	-	-	-	-	-	-	-	-	-	-	-	-	-	-	-	-	-	-	-	-	-	-	-	-	-	-	-	-	-	-	-	-	-	-	-	-
		-	+	-	-	-	-	-	AT5G12130.1	37330	-	-	-	121861	-	-	CMM279C	-	-	-	-	-	-	-	-	-	-	38690	-	-	-	Os05g03000.2	3870	-	12759	180945	-	-	281777	-	-	-	-	-	-	33500	-	-	35034	-	-	-
HTB5	Histone H2B	-	-	-	-	-	-	-	-	-	-	-	-	121856	-	-	-	-	-	-	-	-	-	-	-	-	-	-	-	-	-	-	-	-	-	-	-	-	-	-	-	-	-	-	-	-	-	-	-	-	-	-
		-	-	-	-	-	-	-	-	-	-	-	-	121846	-	-	-	-	-	-	-	-	-	-	-	-	-	-	-	-	-	-	-	-	-	-	-	-	-	-	-	-	-	-	-	-	-	-	-	-	-	-
HFO1	Histone H4	-	-	-	-	-	-	-	-	-	-	-	-	-	-	-	-	-	-	-	-	-	-	-	-	-	-	-	-	-	-	-	-	-	-	-	-	-	-	-	-	-	-	-	-	-	-	-	-	-	-	-
		-	+	-	-	-	-	-	AT5G15530.1	-	-	-	-	121834	-	-	CMV134C	-	-	-	-	-	-	-	-	-	-	-	-	148718	-	-	-	-	-	115965	-	-	665593	-	-	-	-	-	-	-	-	-	-	-	-	-
		-	+	-	-	-	-	-	AT3G02050.1	-	-	-	-	121823	-	-	-	-	DDB0219297	-	-	-	-	-	-	-	-	-	-	-	-	Os02g31940.1	-	-	-	215671	-	-	816880	-	-	-	-	-	-	-	-	-	-	-	-	-
HFO5	Histone H4	-	-	-	-	-	-	-	-	-	-	-	-	-	-	-	-	-	-	-	-	-	-	-	-	-	-	-	-	-	-	-	-	-	-	-	-	-	-	-	-	-	-	-	-	-	-	-	-	-	-	-
		-	+	-	-	-	-	GB10902-PA	AT2G05760.1	-	-	CE13395	177506	121791	288747	-	-	OTTDARP00000022901	DDB0203378	CG6293-PA	-	-	ENSGALP00000000260	-	OTTHUMP00000030174	-	168657	-	-	80445	-	Os01g55500.1	-	-	31650	131303	-	-	411672	RO3G_01794.1	-	-	GLEAN3_08192	SINFRUP00000128512	-	-	-	-	26265	-	-	-
		-	-	-	-	-	-	-	-	-	-	-	-	121784	-	-	-	-	-	-	-	-	-	-	-	-	-	-	-	-	-	-	-	-	-	-	-	-	-	-	-	-	-	-	-	-	-	-	-	-	-	-
		-	+	-	-	-	-	GB11065-PA	AT3G20475.1	-	BDEG_08014	CE26678	-	121781	295937	-	CMN192C	-	DDB0186169	-	-	-	ENSGALP00000004306	-	NP_079535.3	-	233005	26766	75725	238590	NCU09384	-	-	-	52173	-	-	-	568546	-	YDL154W	-	-	SINFRUP00000161087	-	-	-	-	58009	-	-	-
		-	+	-	-	-	-	GB14768-PA	AT2G17640.1	-	-	-	-	121778	-	-	CMI299C	-	-	-	-	13.m00319	-	-	-	LmjF34.2850	-	-	81554	-	-	Os03g04140.1	4140	-	-	15820	-	-	818359	-	-	-	-	-	-	-	-	-	62996	-	-	-
HFO21	Histone H4	-	-	-	-	-	-	-	-	-	-	-	-	-	-	-	-	-	-	-	-	-	-	-	-	-	-	-	-	-	-	-	-	-	-	-	-	-	-	-	-	-	-	-	-	-	-	-	-	-	-	-
		-	+	-	-	-	-	-	AT3G19900.1	-	-	-	-	121745	-	-	CMG187C	-	-	-	-	-	-	-	-	-	-	-	-	-	-	Os02g58310.1	18934	-	41545	138352	-	-	822311	-	-	-	-	-	-	-	-	-	-	-	-	-
		-	-	-	-	-	-	-	-	-	-	-	-	-	-	-	-	-	-	-	-	-	-	-	-	-	-	-	-	-	-	-	-	-	-	-	-	-	-	-	-	-	-	-	-	-	-	-	-	-	-	-
		-	-	-	-	-	-	-	-	-	-	-	-	121724	-	-	-	-	-	-	-	-	-	-	-	-	-	-	-	-	-	-	-	-	-	-	-	-	-	-	-	-	-	-	-	-	-	-	-	-	-	-
		-	+	-	-	-	-	-	AT5G08170.1	-	-	-	-	121719	-	-	CML290C	-	-	-	-	-	-	-	-	-	-	-	49517	246873	-	Os04g39210.1	-	-	36398	161931	-	-	836805	-	-	-	-	-	19.m00291	-	-	-	-	-	-	-
		-	-	-	-	-	-	-	-	-	-	-	-	121707	-	-	-	-	-	-	-	-	-	-	-	-	-	-	-	-	-	-	-	-	-	-	-	-	-	-	-	-	-	-	-	-	-	-	-	-	-	-
DNJ22	DnaJ-like protein 22, rather cytosolic	-	-	-	-	-	-	-	-	-	-	-	-	121702	281935	-	-	-	-	-	-	-	-	-	-	-	-	-	-	-	-	-	33226	-	-	-	-	-	-	-	-	-	-	-	-	-	-	-	-	-	-	-
		-	-	-	-	-	-	-	-	-	-	-	-	121700	-	-	-	-	-	-	-	-	-	-	-	-	-	-	-	-	-	-	-	-	-	-	-	-	-	-	-	-	-	-	-	-	-	-	-	-	-	-
		-	-	-	-	-	-	-	-	-	-	-	-	121699	-	-	-	-	-	-	-	-	-	-	-	-	146942	-	-	-	-	-	-	GSPATP00016019001	-	-	-	-	-	-	-	-	-	-	-	-	-	-	-	-	-	-	-
		-	+	-	-	-	-	-	AT5G58770.1	16553	-	-	-	121674	-	-	CMR474C	OTTDARP00000007068	-	-	-	-	-	GL50803_15256	-	LmjF13.0020	-	-	-	-	-	Os01g63830.1	5970	-	36359	190474	133259	MAL8P1.22	804506	-	-	-	-	-	-	35541	-	-	-	-	Tb927.7.5880	-
		-	+	-	-	-	-	GB14238-PA	-	-	BDEG_01033	-	-	121660	271338	-	CMS384C	-	-	-	-	234.m00046	-	-	-	LmjF05.0390	-	-	-	-	-	-	1716	-	-	-	-	-	240760	-	-	-	-	-	427.m00006	-	-	-	-	95540.m00051	Tb10.406.0580	-
		-	-	-	-	-	-	-	-	-	-	-	-	121658	-	-	CML092C	-	-	-	-	-	-	-	-	-	-	-	-	-	-	-	-	-	-	-	-	-	-	-	-	-	-	-	-	-	-	-	-	-	-	-
		-	+	+	-	-	-	GB11117-PA	AT2G40600.1	61490	BDEG_00428	CE05161	95133	121629	255065	-	CMD127C	OTTDARP00000022432	DDB0204524	-	-	30.m00220	ENSGALP00000023837	-	NP_054786.2	-	101291	-	77152	35922	NCU07925	Os03g21830.1	-	GSPATP00001530001	45202	182881	142064	PF14_0466	242926	-	-	-	-	SINFRUP00000137113	28.m00359	-	-	49.m03196	27195	94962.m00302	Tb927.5.2590	UM06033.1
		-	+	-	-	-	-	GB17081-PA	AT5G06600.2	-	BDEG_08282	CE36180	149911	121621	269931	cgd8_1530	CMR384C	-	DDB0167082	CG1490-PB	19168685	-	ENSGALP00000039009	-	OTTHUMP00000081656	LmjF35.1740	184079	33210	81745	172482	NCU03797	Os12g30540.1	15481	GSPATP00021878001	40154	216093	133568	MAL7P1.147	820184	RO3G_03379.1	YMR304W	SPBC713.02c	GLEAN3_10737	SINFRUP00000143602	30.m00363	24035	TA04875	80.m00082	21658	96726.m00085	Tb09.211.4910	UM02639.1
		-	-	-	-	-	-	-	-	-	-	-	-	121619	-	-	-	-	-	-	-	-	-	-	-	-	-	-	-	-	-	-	-	-	-	103945	-	-	-	-	-	-	-	-	-	-	-	-	-	-	-	-
		-	-	-	-	-	-	-	-	-	-	-	-	121606	-	-	-	-	-	-	-	-	-	-	-	-	-	-	-	-	-	-	-	-	-	-	-	-	-	-	-	-	-	-	-	-	-	-	-	-	-	-
		-	+	-	-	-	-	-	AT4G16510.1	14079	-	-	-	121601	-	-	-	-	DDB0190044	-	-	3.m00652	-	-	-	LmjF21.0910	-	-	71240	-	-	Os02g44330.1	-	GSPATP00014662001	23833	117069	137780	-	799860	RO3G_08468.1	-	-	-	-	12.m00354	1979	-	-	-	84963.m00167	Tb10.70.6810	-
DYRK2	Dual-Specificity Tyrosine Regulated Protein Kinase Homolog 2	-	+	-	-	-	-	-	AT1G73460.1	24517	-	-	-	121593	-	-	-	-	-	-	-	-	-	GL50803_137695	-	LmjF14.1070	-	-	1301	-	-	Os03g51020.1	16877	GSPATP00007044001	-	181271	132207	-	569399	-	-	-	-	-	92.m00122	31069	TA11120	20.m03853	-	-	Tb10.100.0230	-
		-	+	-	-	-	-	GB11583-PA	AT3G20810.1	27076	BDEG_02431	CE05235	225438	121559	289756	-	-	OTTDARP00000021457	DDB0216678	CG13902-PA	-	-	ENSGALP00000010168	-	NP_079049.1	-	142327	2560	68331	163066	NCU10760	Os09g31380.1	11636	-	37877	133637	108145	-	178731	RO3G_13118.1	-	-	GLEAN3_11059	SINFRUP00000171383	6.m00647	5286	-	28.m00292	21598	-	-	UM05658.1
		-	-	-	-	-	-	-	-	-	-	-	-	121540	-	-	-	-	-	-	-	-	-	-	-	-	-	-	-	-	-	-	-	-	-	-	-	-	-	-	-	-	-	-	-	-	-	-	-	-	-	-
		-	-	-	-	-	-	-	-	-	-	-	-	121528	-	-	-	-	-	-	-	-	-	-	-	-	-	-	-	-	-	-	-	-	-	-	-	-	-	-	-	-	-	-	-	-	-	-	-	-	-	-
HDA3415	Histone deacetylase	-	-	-	-	-	-	-	-	-	-	-	-	121526	-	-	-	-	-	-	-	-	-	-	-	-	-	-	-	-	-	-	-	-	-	-	-	-	-	-	-	-	-	-	-	-	-	-	-	-	-	-
		-	-	-	-	-	-	-	-	-	-	-	-	121518	-	-	-	-	-	-	-	-	-	-	-	-	-	-	-	-	-	-	-	-	-	-	-	-	-	-	-	-	-	-	-	-	-	-	-	-	-	-
		-	+	-	-	-	-	-	AT1G70610.1	10595	-	CE07240	-	121501	-	-	-	OTTDARP00000016717	-	-	-	-	-	-	-	LmjF25.0530	-	-	80315	-	-	Os01g68330.1	-	-	-	224391	-	-	-	RO3G_04594.1	-	-	GLEAN3_26178	SINFRUP00000148199	-	26553	-	-	-	-	-	-
		-	-	-	-	-	-	-	-	-	-	-	-	121490	-	-	-	-	-	-	-	-	-	-	-	-	-	-	-	-	-	-	-	-	-	-	-	-	-	-	-	-	-	-	-	-	-	-	-	-	-	-
		-	+	-	-	-	-	GB16890-PA	AT1G07030.1	19467	BDEG_00640	CE03769	156775	121489	225163	cgd2_1030	CMR309C	OTTDARP00000021630	DDB0190286	-	-	-	ENSGALP00000000517	-	OTTHUMP00000020256	LmjF29.2780	140739	37999	73440	238240	NCU02423	Os03g18550.1	5106	GSPATP00011589001	4342	61978	143688	PFI0255c	577051	RO3G_09640.1	YKR052C	SPAC8C9.12c	GLEAN3_19095	SINFRUP00000128913	5.m00405	16184	TA14105	65.m00009	32519	-	Tb927.3.2980	UM01059.1
PAFB3401	Paf1 complex component	-	+	-	-	-	-	GB16641-PA	AT5G61150.2	-	BDEG_02273	CE05166	221877	121477	277946	-	-	OTTDARP00000015196	DDB0186792	CG1433-PA	-	-	ENSGALP00000007469	-	OTTHUMP00000176285	-	-	6772	-	182256	-	Os12g38970.1	37987	-	44561	97917	-	-	569930	RO3G_10712.1	YOR123C	-	-	SINFRUP00000165308	-	4757	-	-	-	-	-	UM04830.1
	N-acetylglutamate synthase, putative	-	+	-	-	-	-	GB12826-PA	AT2G37500.1	-	-	-	-	121469	-	-	CMS424C	-	DDB0184137	-	-	-	-	-	-	-	-	-	-	-	NCU10468	Os03g17120.1	28741	-	12762	213576	142184	-	746969	RO3G_03761.1	YMR062C	SPBC1271.14	-	-	-	2779	-	-	-	-	-	UM02671.1
		-	-	-	-	-	-	-	-	-	-	-	-	-	-	-	-	-	-	-	-	-	-	-	-	-	-	-	-	-	-	-	-	-	-	-	-	-	-	-	-	-	-	-	-	-	-	-	-	-	-	-
CTP1	heavy metal transporting ATPase (HMA), P-type ATPase superfamily, membrane protein	-	+	-	-	-	-	GB10214-PA	AT1G63440.1	-	BDEG_05089	CE26305	32189	121438	389056	cgd3_3740	CMP215C	OTTDARP00000021675	DDB0217251	CG1886-PA	-	-	ENSGALP00000035689	-	OTTHUMP00000023593	LmjF33.2090	136210	27752	272	87416	NCU04076	Os02g10290.1	28276	-	10677	81365	108217	PFI0240c	554346	RO3G_13980.1	YDR270W	SPBC29A3.01	GLEAN3_28504	SINFRUP00000148707	67.m00176	263051	-	20.m03647	56324	-	Tb11.47.0023	UM00227.1
FAP73	Conserved Uncharacterized Flagellar Associated Protein	+	+	-	+	-	-	GB16531-PA	-	68012	BDEG_06843	-	93836	121413	291597	-	-	OTTDARP00000019348	-	-	-	-	ENSGALP00000001819	GL50803_10924	XP_373547.3	LmjF13.0240	208832	30998	46913	187067	-	-	32401	GSPATP00037040001	-	90079	137187	-	-	-	-	-	-	-	119.m00076	9727	-	-	51517	83711.m00156	Tb11.02.2130	-
		-	-	-	-	-	-	-	-	-	-	-	-	121409	-	-	-	-	-	-	-	-	-	-	-	-	-	-	-	-	-	-	-	-	19659	-	-	-	-	-	-	-	-	SINFRUP00000181441	-	-	-	-	-	-	-	-
		-	+	-	-	-	-	GB30529-PA	AT2G35690.1	-	BDEG_06346	CE11546	167362	121379	237579	-	-	-	DDB0188674	CG5009-PA	-	-	ENSGALP00000003376	-	OTTHUMP00000182484	-	212832	29702	-	170851	-	Os06g01390.1	8072	GSPATP00003865001	19979	221246	109131	-	836303	RO3G_10134.1	YGL205W	-	GLEAN3_12644	SINFRUP00000160496	5.m00569	263878	-	50.m03176	28074	-	-	UM02208.1
TIP1		-	+	+	-	-	-	GB17959-PA	AT3G20390.1	28780	BDEG_01816	CE37748	178589	121371	296601	-	CMO242C	OTTDARP00000016339	DDB0169400	CG15261-PA	-	169.m00122	ENSGALP00000028747	GL50803_480	OTTHUMP00000178126	-	208507	35357	30249	242360	NCU10226	Os07g33240.1	28579	GSPATP00038189001	-	121716	-	-	569140	RO3G_06061.1	YER057C	SPBC2G2.04c	GLEAN3_15701	SINFRUP00000171495	166.m00060	-	-	-	64080	81529.m00530	-	UM00065.1
TRM5	tRNA-(N1G37) methyltransferase	-	+	+	-	-	-	GB14510-PA	AT3G56120.1	12241	BDEG_01497	CE17599	218789	121365	239717	cgd6_5130	CMJ080C	-	DDB0206004	CG32281-PA	19068781	247.m00080	ENSGALP00000019401	GL50803_4164	OTTHUMP00000028073	LmjF16.0310	110690	1458	31272	118817	NCU09311	Os01g29409.1	25699	GSPATP00037147001	44373	41777	108672	PFI0700c	261765	RO3G_09009.1	YHR070W	SPAPB18E9.01	-	SINFRUP00000168129	104.m00127	268027	TA17855	49.m00041	2329	81617.m00200	Tb927.8.5720	UM00301.1
		-	+	-	-	-	-	GB17175-PA	-	-	BDEG_00678	CE31765	-	121355	206451	cgd3_3040	-	-	-	-	19069116	304.m00063	-	-	-	-	190501	-	-	-	NCU06240	-	-	GSPATP00023383001	-	126373	-	PFI1685w	-	RO3G_17007.1	YPL203W	SPCC24B10.07	-	SINFRUP00000158340	-	-	TA12860	42.m00079	27989	81877.m00054	Tb09.211.2360	UM01124.1
		-	-	-	-	-	-	-	-	-	-	-	-	121353	-	-	-	-	-	-	-	-	-	-	-	-	-	-	-	-	-	-	-	-	-	-	-	-	-	-	-	-	-	-	-	-	-	-	-	-	-	-
HCS2	cytochrome c heme lyase (holocytochrome c synthase)	-	+	-	-	-	-	GB18121-PA	-	5253	BDEG_02651	CE02327	3770	121344	297693	-	-	OTTDARP00000019974	DDB0217711	-	-	-	ENSGALP00000008923	-	NP_005324.2	-	-	27974	-	-	NCU08138	-	5743	-	-	-	-	PFL1185c	-	RO3G_03410.1	YKL087C	SPAC24C9.02c	GLEAN3_25686	SINFRUP00000133442	-	28667	TA15105	583.m05596	28549	-	-	UM04702.1
		-	+	-	-	-	-	-	-	14206	-	-	21692	121343	-	-	-	-	-	-	-	-	-	-	-	LmjF34.1410	161339	-	-	-	NCU09783	-	35540	-	48946	157730	128690	-	280845	-	-	-	-	-	-	24619	-	33.m01302	53759	-	-	-
		-	+	-	-	-	-	-	AT5G14460.1	5154	-	-	-	121340	-	-	CMF022C	-	-	-	-	-	ENSGALP00000014785	-	OTTHUMP00000020554	-	-	13301	-	5335	-	Os05g01750.1	5148	-	11965	168093	123541	PF10_0175	795537	RO3G_09003.1	YNL292W	-	-	-	-	37374	-	-	-	-	-	-
		+	+	-	-	-	-	-	-	-	BDEG_05157	-	228976	121332	274040	-	-	-	-	-	-	-	-	-	NP_689800.3	-	65972	-	52840	119683	-	-	-	GSPATP00008663001	-	-	129634	-	-	-	-	-	-	-	-	-	-	-	57008	80671.m00454	-	-
SQD2b		-	-	-	-	-	-	-	-	-	BDEG_02231	-	-	121326	-	cgd5_3140	-	-	-	-	-	-	-	-	-	-	-	-	-	-	-	-	3203	-	-	-	-	-	-	-	-	-	-	-	-	-	-	-	-	-	-	-
		-	-	-	-	-	-	-	-	32297	-	-	-	121322	-	-	-	-	-	-	-	-	-	-	-	-	-	30730	-	-	-	-	-	-	-	-	-	-	584333	-	-	-	-	-	-	-	-	-	-	-	-	UM02235.1
PP2C-3c	Protein Phosphatase 2C catalytic subunit Homolog 3	-	+	-	-	-	-	GB17739-PA	AT3G11410.1	-	-	-	-	121320	-	-	CMS179C	-	-	-	-	109.m00098	-	GL50803_11740	-	-	-	-	80475	-	-	Os01g46760.1	-	-	12510	13662	-	-	279694	-	YDL006W	-	-	-	-	262261	-	-	-	-	-	-
		-	+	-	-	-	-	-	AT5G04520.1	-	-	-	-	121292	-	-	-	-	-	-	-	-	-	-	-	-	-	16153	36730	79879	-	Os03g24830.1	21865	-	-	176817	135789	-	833890	-	-	-	-	-	-	-	-	-	-	-	-	UM03905.1
		-	-	-	-	-	-	-	-	-	-	-	-	121275	-	-	-	-	-	-	-	-	-	-	-	-	-	-	-	-	-	-	-	-	-	-	-	-	-	-	-	-	-	-	-	-	-	-	-	-	-	-
		-	-	-	-	-	-	-	AT1G76690.1	-	-	-	-	121264	-	-	-	-	-	-	-	-	-	-	-	-	-	-	-	-	-	Os06g11240.1	-	-	-	-	-	-	810138	-	-	-	-	-	4.m00643	-	-	-	-	-	-	-
		-	-	-	-	-	-	-	-	-	-	-	-	-	-	-	-	-	-	-	-	-	-	-	-	-	-	-	-	-	-	-	-	-	-	-	-	-	-	-	-	-	-	-	-	-	-	-	-	-	-	-
ADCY38	adenylate cyclase	-	-	-	-	-	-	-	-	-	-	-	-	121205	-	-	-	-	-	-	-	-	-	-	-	-	-	-	-	-	-	-	-	-	-	-	-	-	-	-	-	-	-	-	-	-	-	-	-	-	-	-
		-	+	-	-	-	-	GB13058-PA	AT3G17770.1	-	-	CE21240	218793	121201	205629	-	-	-	DDB0190144	-	-	-	ENSGALP00000008385	-	NP_056348.2	LmjF10.0420	119250	-	72653	83220	NCU04400	Os03g51000.2	8836	-	42962	127848	108786	-	823154	RO3G_04434.1	YML070W	SPAC977.16c	GLEAN3_00487	-	2.m02276	35684	-	-	-	-	-	UM00109.1
		-	+	-	-	-	-	-	AT2G39080.1	-	-	-	-	121199	-	-	CMO263C	-	-	-	-	-	-	-	-	-	-	-	-	-	-	Os01g26920.1	18198	-	44401	234923	-	-	721040	-	-	-	-	-	-	5487	-	-	-	-	-	-
PDE19	3',5'-cyclic-nucleotide phosphodiesterase	-	+	-	-	-	-	-	-	-	-	-	-	121190	-	-	-	-	-	-	-	-	-	GL50803_14058	-	-	-	-	-	-	-	-	-	GSPATP00011621001	-	-	-	-	-	-	YOR360C	-	-	-	95.m00117	262976	-	-	-	-	-	-
		-	+	-	-	-	-	-	-	64193	-	-	-	121188	220238	-	-	-	DDB0188580	-	-	-	ENSGALP00000002641	-	OTTHUMP00000033539	-	-	-	82761	185021	NCU00784	-	-	-	-	143497	-	-	-	-	-	-	GLEAN3_25958	SINFRUP00000138479	-	21176	-	-	-	-	-	-
		-	-	-	-	-	-	-	-	-	-	-	-	121185	-	-	-	-	-	-	-	-	-	-	-	-	-	-	-	-	-	-	-	-	-	-	-	-	-	-	-	-	-	-	-	-	-	-	-	-	-	-
		-	+	+	-	-	-	GB12235-PA	AT2G45270.1	2360	BDEG_06456	CE20480	106307	121180	207731	cgd6_3550	CMS344C	OTTDARP00000003651	DDB0183804	CG14231-PA	19171260	124.m00124	ENSGALP00000003745	GL50803_17420	NP_071748.2	LmjF31.0100	227060	17603	48339	152754	NCU03836	Os12g29990.1	17544	GSPATP00023505001	9987	186936	144475	PF10_0299	243990	RO3G_14294.1	YDL104C	SPCC1259.10	GLEAN3_21022	SINFRUP00000150388	-	2332	TA11230	20.m03710	21983	86498.m00500	Tb927.7.6470	UM00617.1
		-	-	-	-	-	-	-	-	-	-	-	-	121178	-	-	-	-	DDB0218524	-	-	-	-	-	-	-	-	-	-	-	-	-	34305	-	-	-	-	-	-	-	-	-	-	-	-	-	-	80.m00091	-	-	-	-
		-	-	-	-	-	-	-	-	-	-	-	140938	121177	-	-	-	-	-	-	-	-	-	-	-	-	75118	-	-	-	-	-	-	-	-	-	-	-	-	-	-	-	-	-	-	-	-	-	-	-	-	-
ADCY10	adenylate cyclase	-	-	-	-	-	-	-	-	-	-	-	-	121176	-	-	-	-	-	-	-	-	-	-	-	-	-	-	-	-	-	-	-	-	-	-	-	-	-	-	-	-	-	-	-	-	-	-	-	-	-	-
		-	-	-	-	-	-	-	-	-	-	-	-	121167	-	-	-	-	-	-	-	-	-	-	-	-	-	-	-	-	-	-	-	-	-	-	-	-	-	-	-	-	-	-	-	-	-	-	-	-	-	-
ADCY9	adenylate cyclase	-	-	-	-	-	-	-	-	-	-	-	-	121159	-	-	-	-	-	-	-	-	-	-	-	-	-	-	-	-	-	-	-	-	-	-	-	-	-	-	-	-	-	-	-	-	-	-	-	-	-	-
GTR26	dolichyl-diphosphooligosaccharide-protein glycosyltransferase (DDOST)	-	+	+	-	-	-	GB18042-PA	AT5G66680.1	-	BDEG_02709	CE01081	181863	121156	272502	cgd2_1650	CMK108C	-	DDB0184059	CG9022-PA	-	364.m00051	ENSGALP00000016354	-	NP_005207.2	-	197848	-	80524	186036	NCU00669	Os07g10830.1	-	GSPATP00004648001	-	234790	140631	PFI0960w	820006	RO3G_02802.1	YEL002C	SPCC338.15	GLEAN3_27730	SINFRUP00000131698	127.m00105	-	-	20.m03837	37905	82234.m00094	-	UM04994.1
		-	-	-	-	-	-	-	AT4G22240.1	-	-	-	-	121152	-	-	-	-	-	-	-	-	-	-	-	-	-	-	-	-	-	Os09g04790.1	-	-	-	130613	-	-	828527	-	-	-	-	-	-	-	-	-	-	-	-	-
		-	-	-	-	-	-	-	-	-	-	-	-	121148	-	-	-	-	-	-	-	-	-	-	-	-	-	-	-	-	-	-	-	-	-	-	-	-	-	-	-	-	-	-	-	-	-	-	-	-	-	-
GTR14	glycosyl transferase	-	+	+	-	-	-	-	AT5G22130.1	-	BDEG_07733	CE02103	223413	121145	-	cgd1_3720	-	-	DDB0188161	CG9865-PB	-	248.m00069	ENSGALP00000037081	GL50803_17502	OTTHUMP00000025755	LmjF30.2030	217696	15575	1788	174137	NCU06057	Os03g46750.1	30298	GSPATP00021651001	18303	140814	-	PFL0540w	639801	RO3G_06892.1	YJR013W	SPBC13E7.05	GLEAN3_20788	SINFRUP00000175180	287.m00036	42624	TA08865	583.m05330	49771	-	Tb927.6.3300	UM00351.1
		-	-	-	-	-	-	-	-	-	-	-	-	121118	-	-	-	-	-	-	-	-	-	-	-	-	-	-	-	-	-	-	-	-	-	-	-	-	-	-	-	-	-	-	-	-	-	-	-	-	-	-
		-	+	-	-	-	-	GB16275-PA	AT3G47730.1	-	BDEG_05952	CE20234	226736	121110	292370	-	CMJ039C	OTTDARP00000016397	DDB0184187	-	-	-	ENSGALP00000003050	GL50803_8227	-	-	110141	22693	69484	87844	NCU04021	Os08g30740.1	-	GSPATP00017828001	-	190702	143446	-	581361	-	-	-	GLEAN3_23638	SINFRUP00000177341	15.m00305	264740	-	-	10137	96145.m00158	-	UM03454.1
		-	+	-	-	-	-	GB14449-PA	AT1G78010.1	59431	-	CE19834	-	121098	211691	-	CMV025C	OTTDARP00000023606	DDB0190890	-	-	-	-	-	OTTHUMP00000076787	-	103257	15612	56870	-	NCU04149	Os08g31460.1	15928	-	-	65194	108375	-	551541	RO3G_09583.1	YMR023C	SPAC222.05c	GLEAN3_20224	SINFRUP00000158019	105.m00146	38757	TA17200	20.m03859	53723	-	-	UM01358.1
		-	-	-	-	-	-	-	-	-	-	-	-	121076	-	-	-	-	-	-	-	-	-	-	NP_076433.2	-	-	-	-	-	-	-	-	-	-	-	-	-	-	-	-	-	GLEAN3_12080	-	-	-	-	-	-	-	-	-
ADCY28	adenylate cyclase	-	-	-	-	-	-	-	-	-	-	-	-	121064	-	-	-	-	-	-	-	-	-	GL50803_16599	-	-	-	-	-	-	-	-	-	-	-	-	-	-	-	-	-	-	-	-	-	-	-	-	-	-	-	-
DNJ21	DnaJ-like protein 21, rather cytosolic	-	-	-	-	-	-	-	-	-	-	-	-	121057	-	-	-	-	-	-	-	-	-	-	-	-	-	-	-	-	-	-	-	-	-	-	-	-	-	-	-	-	-	-	-	-	-	-	-	-	-	-
		-	+	-	-	-	-	GB10352-PA	AT1G07110.1	2314	BDEG_02760	CE30082	161796	121054	262091	-	CMN085C	OTTDARP00000023756	DDB0217769	CG3400-PE	-	-	ENSGALP00000010211	-	OTTHUMP00000019039	LmjF03.0800	186853	27797	38553	234745	NCU09347	Os05g07130.2	14708	GSPATP00020537001	17495	146321	131873	-	815458	RO3G_15926.1	YJL155C	SPAC732.02c	GLEAN3_02649	SINFRUP00000143959	-	14563	-	-	37504	84270.m00676	Tb927.3.2710	UM02633.1
		-	-	-	-	-	-	-	-	-	-	-	-	121029	-	-	-	-	-	-	-	-	-	-	-	-	-	-	-	-	-	-	-	-	-	-	-	-	-	-	-	-	-	SINFRUP00000183023	-	-	-	-	-	-	-	-
		-	+	-	-	-	-	-	-	-	-	-	-	121025	-	-	-	-	DDB0217267	-	-	-	-	-	-	-	-	-	-	-	NCU09160	-	-	-	-	-	-	-	582496	RO3G_10365.1	-	-	-	-	-	20829	-	-	-	-	-	UM02197.1
		-	-	-	-	-	-	-	-	-	-	-	-	121020	-	-	-	-	-	-	-	-	-	-	-	-	-	-	-	-	-	-	-	-	-	-	-	-	-	-	-	-	-	-	-	-	-	-	-	-	-	-
PDE24	3' 5' cyclic phosphodiesterase	-	-	-	-	-	-	-	-	-	-	-	-	121006	-	-	-	-	-	-	-	-	-	-	-	-	-	-	-	-	-	-	-	-	-	-	-	-	-	-	-	-	-	-	-	-	-	-	-	-	-	-
		-	-	-	-	-	-	-	-	-	-	-	-	121004	-	-	-	-	-	-	-	-	-	-	-	-	-	-	-	-	-	-	-	-	-	-	-	-	-	-	-	-	-	-	-	-	-	-	-	-	-	-
		-	-	-	-	-	-	-	-	-	-	-	-	120987	-	-	-	-	-	-	-	-	-	-	-	-	-	-	-	-	-	-	-	-	-	-	-	-	-	-	-	-	-	-	-	-	-	-	-	-	-	-
		-	-	-	-	-	-	-	-	-	-	-	-	120979	-	-	-	-	-	-	-	-	-	-	-	-	-	-	-	-	-	-	-	GSPATP00030689001	-	-	-	-	-	-	-	-	-	-	20.m00312	-	-	83.m01257	-	-	-	-
		-	+	-	-	-	-	-	AT1G60710.1	-	-	-	-	120977	-	-	-	-	-	-	-	-	-	-	-	-	-	-	-	-	NCU07402	Os04g26910.1	-	-	44546	197877	-	-	411480	-	YPR127W	SPAC1F7.12	-	-	-	-	-	-	-	-	-	UM03006.1
		-	-	-	-	-	-	-	-	-	-	-	-	120971	-	-	-	-	-	-	-	-	-	-	-	-	-	-	-	-	-	-	-	-	-	-	-	-	-	-	-	-	-	-	-	-	-	-	-	-	-	-
		-	+	-	-	-	-	GB19688-PA	AT4G33730.1	-	BDEG_00174	-	221658	120954	214556	-	-	OTTDARP00000018261	-	-	-	-	-	-	OTTHUMP00000168529	-	233201	30348	-	-	-	Os02g54530.1	-	-	-	27775	-	-	649729	-	-	-	-	-	95.m00110	-	-	-	-	-	-	-
		-	-	-	-	-	-	-	-	-	-	-	-	120933	-	-	-	-	-	-	-	-	-	-	-	-	-	-	-	-	-	-	-	-	-	-	-	-	-	-	-	-	-	-	-	-	-	-	-	-	-	-
		-	-	-	-	-	-	-	-	-	-	-	-	120931	-	-	-	-	-	-	-	-	-	-	-	-	-	-	-	-	-	-	-	-	-	-	-	-	-	-	-	-	-	-	-	-	-	-	-	-	-	-
		-	+	-	-	-	-	-	AT5G38360.1	-	-	-	-	120927	-	-	-	-	-	-	-	-	-	-	-	-	-	-	-	-	-	Os04g31240.1	35770	-	43025	7020	-	-	198166	-	-	-	-	-	-	2657	-	-	-	-	-	-
		-	+	-	-	-	-	GB16823-PA	AT4G20140.1	71506	-	-	224341	120925	-	-	-	-	DDB0204296	-	-	-	ENSGALP00000038622	-	-	LmjF12.0860	-	25375	-	-	-	Os07g31500.1	20190	-	107	215125	136635	-	817473	RO3G_11619.1	-	-	GLEAN3_07065	SINFRUP00000167775	-	268761	-	-	54150	-	-	-
		-	+	-	-	-	-	-	AT1G50170.1	32321	-	-	-	120910	-	-	CMH050C	-	-	-	-	-	-	-	-	-	-	-	-	-	-	Os02g19440.1	21976	-	49508	129073	131724	-	181024	-	-	-	-	-	-	24431	-	-	-	-	-	-
CDKE1	cyclin dependent kinase	-	+	-	-	-	-	-	AT5G63610.1	-	-	CE32409	224421	120881	-	-	-	OTTDARP00000022236	DDB0189823	CG10572-PA	-	-	ENSGALP00000027576	-	OTTHUMP00000017005	-	210630	-	70082	11543	-	Os10g42950.4	-	-	-	184162	119398	-	757657	RO3G_14610.1	YPL042C	SPAC23H4.17c	GLEAN3_01690	SINFRUP00000158484	-	-	-	-	32638	-	-	UM03131.1
		-	-	-	-	-	-	-	-	-	-	-	-	120875	-	-	-	-	-	-	-	-	-	-	-	-	-	-	-	-	-	-	-	-	-	-	-	-	-	-	-	-	-	-	-	-	-	-	-	-	-	-
		-	-	-	-	-	-	-	-	-	-	-	-	120852	-	-	-	-	-	-	-	-	-	-	-	-	-	-	-	-	-	-	-	-	-	-	-	-	-	-	-	-	-	-	-	-	-	-	-	-	-	-
IF3F		-	+	-	-	-	-	GB17508-PA	AT2G39990.1	13838	-	CE00932	177448	120842	297483	cgd7_1080	CMP265C	-	DDB0191852	CG9769-PA	-	-	ENSGALP00000008530	-	XP_290345.1	-	200059	18528	-	230299	NCU01021	Os05g01450.1	23389	-	11458	120144	109294	PFI0895c	659552	RO3G_03247.1	-	SPBC4C3.07	GLEAN3_23686	SINFRUP00000170198	-	38225	TA08465	42.m03611	51319	-	-	UM00175.1
		-	+	-	-	-	-	GB13140-PA	-	-	-	-	-	120829	-	-	-	OTTDARP00000024312	-	CG8745-PA	-	-	ENSGALP00000017115	-	NP_112569.2	-	-	30473	-	-	-	-	-	-	52110	-	-	-	-	-	-	-	-	SINFRUP00000155980	-	2775	-	-	-	-	-	-
		-	-	-	-	-	-	-	-	-	-	-	-	120803	-	-	-	-	-	-	-	-	-	-	-	-	-	-	-	-	-	-	5996	-	-	-	-	-	-	-	-	-	-	-	-	-	-	-	-	-	-	-
		-	+	-	-	-	-	-	AT2G39630.1	-	-	-	-	120765	-	-	-	-	-	-	-	-	-	-	-	-	-	-	-	-	NCU06386	Os03g60700.1	-	-	-	-	-	-	822479	-	-	-	-	-	-	-	-	-	-	97114.m00211	-	-
		-	-	-	-	-	-	-	-	-	-	-	-	120760	-	-	-	-	-	-	-	-	-	-	-	-	-	-	-	-	-	-	-	-	-	-	-	-	-	-	-	-	-	-	-	-	-	-	-	-	-	-
		-	+	-	-	-	-	-	-	-	-	-	-	120756	273477	-	CMF144C	-	DDB0218369	CG12162-PB	-	-	ENSGALP00000010670	-	-	LmjF26.0580	131282	-	82074	241548	-	Os05g07860.1	35322	-	-	142413	-	-	662013	-	-	-	-	-	-	22763	-	-	-	-	Tb927.7.1200	-
		-	+	-	-	-	-	-	AT2G27940.1	36689	BDEG_04216	CE18510	180387	120741	274137	cgd8_3470	-	OTTDARP00000019944	-	CG32850-PA	-	408.m00043	-	-	OTTHUMP00000023575	-	114185	7692	5673	-	NCU05314	Os07g27950.2	33582	-	48034	-	158458	-	563889	-	YBR062C	-	GLEAN3_25460	-	-	21152	-	44.m02707	-	-	-	UM01244.1
HFO41	Histone H4	-	-	-	-	-	-	-	-	-	-	-	-	120734	-	-	-	-	-	-	-	-	-	-	-	-	-	-	-	-	-	-	-	-	-	-	-	-	-	-	-	-	-	-	-	-	-	-	-	-	-	-
		-	+	-	-	-	-	-	AT5G13030.1	-	BDEG_05492	-	-	120727	-	-	CMO340C	-	-	-	-	-	-	-	-	-	122035	-	81114	132366	NCU01758	Os06g21600.1	22489	-	29423	220952	-	-	750527	-	YPL222W	SPAC20G4.05c	-	-	-	37944	-	-	-	-	-	-
		-	+	+	-	-	-	GB13458-PA	AT3G59990.3	71349	BDEG_05250	CE24146	173347	120712	298079	cgd2_2480	CMP247C	-	DDB0190923	CG4008-PA	19069326	159.m00092	ENSGALP00000018537	GL50803_86600	OTTHUMP00000168760	LmjF21.0840	179055	37121	44726	181577	NCU04306	Os12g43370.2	27405	GSPATP00037051001	17304	203982	109386	PF14_0327	198596	RO3G_03853.1	YBL091C	SPBC14C8.03	GLEAN3_00075	SINFRUP00000129935	87.m00211	24414	TA18485	541.m00143	61709	91003.m00121	Tb10.70.6620	UM06034.1
		-	-	-	-	-	-	-	-	-	-	-	-	120709	-	-	-	-	-	-	-	-	-	-	-	-	-	-	-	-	-	-	-	-	-	-	-	-	-	-	-	-	-	-	-	-	-	-	-	-	-	-
		-	+	-	-	-	-	GB17153-PA	AT2G15910.1	29128	BDEG_06813	CE03453	30109	120707	-	-	CMS025C	-	DDB0217888	CG14701-PA	19171349	-	ENSGALP00000018305	-	OTTHUMP00000170418	LmjF26.2630	-	3774	31070	246077	NCU09465	Os06g45070.2	21927	GSPATP00002921001	8694	19840	123359	PFL0860c	649489	-	YBL071W-A	SPAC8F11.02c	GLEAN3_05314	SINFRUP00000154495	17.m00402	32788	TA04920	-	25301	-	Tb09.160.0380	UM03601.1
		-	-	-	-	-	-	-	-	-	-	-	-	-	-	-	-	-	-	-	-	-	-	-	-	-	-	-	-	-	-	-	-	-	-	-	-	-	-	-	-	-	-	-	-	-	-	-	-	-	-	-
		-	-	-	-	-	-	-	-	-	-	-	-	120672	-	-	-	-	-	-	-	-	-	-	-	-	-	-	-	-	-	-	7887	-	-	-	-	-	-	-	-	-	-	-	-	-	-	-	-	-	-	-
		-	+	+	-	-	-	GB13977-PA	AT4G05190.1	-	BDEG_07539	-	2580	120667	-	cgd3_2590	CMR497C	OTTDARP00000017239	DDB0189377	-	-	172.m00081	-	GL50803_8886	NP_002254.1	-	140562	14231	32240	124130	NCU04581	Os04g53760.2	19771	GSPATP00031428001	-	130120	-	-	822824	-	YPR141C	SPAC664.10	-	-	75.m00179	35310	-	-	28367	-	-	UM04507.1
		-	+	-	-	-	-	GB13592-PA	AT3G52140.1	-	-	-	-	120664	-	-	-	OTTDARP00000021421	-	CG5433-PA	-	-	-	GL50803_11177	-	LmjF34.3490	132171	-	61341	164652	NCU00021	Os02g48620.2	-	GSPATP00016369001	49558	203627	135510	-	173323	RO3G_05230.1	-	SPBC530.06c	-	-	34.m00237	6886	-	-	-	-	Tb927.4.1280	UM03013.1
		-	+	-	-	-	-	-	AT3G12080.1	37344	-	-	134007	120661	-	-	CMC059C	-	DDB0205682	-	-	-	-	-	-	-	-	-	56634	153067	-	Os11g41910.1	35581	-	17172	135839	-	PFL0835w	203056	-	-	-	-	-	20.m00420	32414	TA04910	27.m00865	-	-	-	-
PRP4		-	+	-	-	-	-	GB19235-PA	AT2G41500.1	-	BDEG_03988	CE05372	226300	120649	243767	cgd5_1130	-	-	DDB0187564	CG6322-PA	-	144.m00085	ENSGALP00000014372	-	OTTHUMP00000021946	-	216477	37807	-	241701	NCU11251	Os03g23929.1	5194	GSPATP00037851001	21983	119600	155637	MAL13P1.385	801796	RO3G_02077.1	YPR178W	SPAC227.12	GLEAN3_14361	SINFRUP00000153743	109.m00112	260891	TA20625	49.m03354	21736	-	-	UM05432.1
		-	-	-	-	-	-	-	-	-	-	-	-	120606	251785	-	-	-	-	-	-	-	-	-	-	-	-	-	-	-	-	-	-	-	-	-	-	-	-	-	-	-	-	-	-	-	-	-	-	-	-	-
		-	-	-	-	-	-	-	AT1G74710.2	-	-	-	-	120595	-	-	-	-	-	-	-	-	-	-	-	-	-	-	-	-	-	-	-	-	-	118465	-	-	422972	-	-	-	-	-	-	263409	-	-	-	-	-	-
		-	+	-	-	-	-	-	AT3G26710.1	21109	-	-	-	120574	-	-	CMM306C	-	-	-	-	-	-	-	-	-	-	-	-	-	-	Os11g32320.1	33436	-	42329	130718	-	-	177406	-	-	-	-	-	-	-	-	-	-	-	-	-
		-	+	-	-	-	-	GB19801-PA	AT1G17650.1	21710	BDEG_02337	CE18481	-	120516	238041	-	CMJ086C	OTTDARP00000022870	DDB0184496	CG4747-PA	-	-	ENSGALP00000002822	-	NP_115958.2	LmjF30.0180	105065	33474	81719	178116	NCU10110	Os01g39270.1	17117	GSPATP00031619001	17487	200413	120688	-	810195	RO3G_07836.1	-	-	-	SINFRUP00000137091	96.m00123	2669	-	55.m10231	50898	-	Tb927.6.1570	-
MRPL1	Putative mitochondrial ribosomal protein L1, imported to mitochondria	-	-	-	-	-	-	-	-	-	-	-	-	120508	-	-	-	-	-	-	-	-	-	-	-	-	-	32902	-	-	-	-	-	-	-	-	-	-	-	-	-	SPAC1610.02c	GLEAN3_14005	-	-	-	-	-	-	-	-	-
		-	-	-	-	-	-	-	-	-	-	-	-	-	-	-	-	-	-	-	-	-	-	-	-	-	-	-	-	-	-	-	-	-	-	-	-	-	-	-	-	-	-	-	-	-	-	-	-	-	-	-
		-	-	-	-	-	-	-	-	-	-	-	-	120503	-	-	CMQ410C	-	-	-	-	-	-	-	-	-	-	-	-	225281	-	-	29196	-	-	-	-	-	587858	-	-	-	-	-	-	-	-	-	-	-	-	-
		-	-	-	-	-	-	-	-	-	-	-	-	120498	-	-	-	-	-	-	-	-	-	-	-	-	-	-	-	-	-	-	-	-	-	-	-	-	-	-	-	-	-	-	-	-	-	-	-	-	-	-
		-	+	-	-	-	-	GB13599-PA	AT3G02870.1	5328	BDEG_00870	CE29761	159956	120462	204987	-	CMQ201C	OTTDARP00000020171	DDB0204100	CG9391-PA	-	208.m00082	ENSGALP00000025398	-	OTTHUMP00000177805	-	178401	-	50823	166138	NCU06348	Os03g39000.2	17504	-	17226	205419	141411	-	716898	RO3G_06496.1	YDR287W	-	GLEAN3_15691	SINFRUP00000144417	-	17443	-	41.m01381	52515	-	Tb927.5.2690	UM02750.1
		-	-	-	-	-	-	-	-	-	-	-	-	120433	-	-	-	-	-	-	-	-	-	-	-	-	-	-	-	-	-	-	-	-	-	-	-	-	-	-	-	-	-	-	-	-	-	-	-	-	-	-
GCY	membrane guanylyl cyclase	-	-	-	-	-	-	-	-	-	-	CE18923	-	120423	-	-	-	-	-	-	-	-	-	-	-	-	-	-	-	-	-	-	-	-	-	-	-	-	-	-	-	-	-	SINFRUP00000170230	-	-	-	-	-	-	-	-
		-	+	-	-	-	-	GB15719-PA	AT5G20930.1	30088	-	CE39220	174294	120417	295529	-	CMQ173C	OTTDARP00000022601	-	CG32782-PC	-	-	ENSGALP00000000558	-	OTTHUMP00000163077	LmjF31.2860	54679	31879	74916	117131	-	Os03g53880.1	20733	GSPATP00000017001	10499	223492	-	-	721419	-	-	-	GLEAN3_22809	SINFRUP00000140996	68.m00147	37523	-	-	23353	-	Tb927.8.7220	-
		-	+	-	-	-	-	GB14437-PA	AT3G07370.1	-	-	CE29991	156491	120414	297354	-	-	-	-	CG5203-PA	-	-	ENSGALP00000001527	-	OTTHUMP00000149944	-	127906	-	-	169527	NCU10270	Os05g01460.1	30282	GSPATP00028388001	-	151528	136120	PF07_0026	830590	-	-	-	GLEAN3_21620	SINFRUP00000169909	4.m00627	-	TA21335	-	23038	-	-	-
		-	+	+	-	-	-	GB13044-PA	AT1G68310.1	24619	BDEG_07382	CE16053	113866	120412	296907	cgd7_5070	CML160C	OTTDARP00000023253	DDB0184491	CG7949-PA	19069380	14.m00303	ENSGALP00000008227	GL50803_8819	OTTHUMP00000080295	LmjF07.1010	187336	14898	70708	180659	NCU02573	Os04g50864.1	14785	GSPATP00018996001	11954	229872	-	PF07_0011	813730	RO3G_01974.1	YHR122W	SPAC144.16	-	SINFRUP00000178450	125.m00083	33601	TA09900	541.m01246	50205	86827.m00474	Tb927.8.720	UM01420.1
		-	-	-	-	-	-	-	-	-	-	-	-	120391	-	-	-	-	-	-	-	-	-	-	-	-	-	-	-	-	-	-	-	-	-	-	-	-	-	-	-	-	-	-	-	-	-	-	-	-	-	-
		-	+	-	-	-	-	-	AT1G21070.1	3854	-	-	-	120386	-	-	-	-	-	-	-	-	-	-	-	-	-	-	-	-	-	Os07g39280.1	27683	-	-	83692	-	-	753827	-	-	-	-	-	-	-	-	-	-	85473.m00024	-	-
		-	-	-	-	-	-	-	-	-	-	-	-	120385	-	-	-	-	-	-	-	-	-	-	-	-	-	-	-	-	-	-	-	-	-	-	-	-	-	-	-	-	-	-	-	-	-	-	-	-	-	-
		-	+	-	-	-	-	GB16285-PA	AT3G15080.1	4515	BDEG_07350	CE19046	25656	120367	372043	cgd7_1030	CMT575C	-	DDB0218293	CG6833-PA	19069102	-	ENSGALP00000017739	-	OTTHUMP00000022484	LmjF10.0270	124106	14000	59694	43493	NCU05217	Os08g28980.1	5562	GSPATP00005519001	5546	143178	138287	-	815795	RO3G_04877.1	YOL080C	SPBC1604.09c	GLEAN3_16653	SINFRUP00000159011	1.m00738	7123	-	25.m00206	19790	82761.m00526	Tb927.8.3710	UM01397.1
CHR3406		-	-	-	-	-	-	-	-	-	-	-	-	120362	-	-	-	-	-	-	-	-	-	-	-	-	-	-	-	-	-	-	-	-	-	-	-	-	824295	-	-	-	-	-	-	-	-	-	-	-	-	-
HDA3408	Histone deacetylase	-	-	-	-	-	-	-	-	-	-	-	-	120355	-	-	-	-	-	-	-	-	-	-	-	-	-	-	-	-	-	-	-	-	-	-	-	-	-	-	-	-	-	-	-	-	-	-	-	-	-	-

FKB15-4	peptidyl-prolyl cis-trans isomerase, FKBP-type	-	-	-	-	-	-	-	-	-	-	[illegible]
	ABC1 family ser/thr kinase	-	+	-	-	-	-	-	AT5G05200.1	1637	-	[illegible]
		-	+	-	-	-	-	GB14389-PA	AT1G18180.1	31445	BDEG_03738	[illegible]
		-	-	-	-	-	-	-	-	-	-	[illegible]
		-	-	-	-	-	-	-	-	-	-	[illegible]
		-	+	+	-	-	-	GB15068-PA	AT5G55130.1	55300	BDEG_01402	[illegible]
		-	+	-	-	-	-	GB18738-PA	AT5G57440.1	-	BDEG_00810	[illegible]
		-	+	-	-	-	-	GB12316-PA	AT1G12650.4	-	BDEG_01236	[illegible]
		-	-	-	-	-	-	-	-	-	-	[illegible]
		-	-	-	-	-	-	-	-	-	-	[illegible]
		-	+	+	-	-	-	GB13779-PA	AT1G77740.1	23649	BDEG_00322	[illegible]
CEP	Cysteine protease	-	+	-	-	-	-	GB19923-PA	AT1G47128.1	2413	-	[illegible]
		-	+	-	-	-	-	-	AT5G14740.4	-	-	[illegible]
		-	+	-	-	-	-	GB15204-PA	-	3043	BDEG_06066	[illegible]
		-	-	-	-	-	-	-	-	-	-	[illegible]
		-	-	-	-	-	-	-	-	-	-	[illegible]
		-	+	+	-	-	-	GB10970-PA	AT4G02070.1	71064	BDEG_04079	[illegible]
		-	-	-	-	-	-	-	-	-	-	[illegible]
		-	-	-	-	-	-	-	-	-	-	[illegible]
		-	+	-	-	-	-	-	-	-	-	[illegible]
		-	-	-	-	-	-	-	-	-	-	[illegible]
		-	-	-	-	-	-	-	-	-	-	[illegible]
PSAD		-	+	-	-	-	-	-	AT4G02770.1	-	-	[illegible]
		-	+	-	-	-	-	GB12800-PA	AT2G38010.2	53967	-	[illegible]
LSD1		-	+	-	-	-	-	-	AT4G20380.7	-	-	[illegible]
		-	-	-	-	-	-	-	AT3G06370.1	-	-	[illegible]
		-	-	-	-	-	-	-	-	-	-	[illegible]
ADCY12	adenylate cyclase	-	-	-	-	-	-	-	-	-	-	[illegible]
		-	+	-	-	-	-	GB14584-PA	AT4G34910.1	52462	BDEG_02440	[illegible]
SPLH3	DEAH-box nuclear pre-mRNA splicing factor	-	-	-	-	-	-	-	AT1G27900.1	-	-	[illegible]
	soluble starch synthase, ADP-glucose alpha-1,4 glucane alpha-4-glucanotransferase	-	-	-	-	-	-	-	-	-	-	[illegible]
SRT3401	Histone deacetylase (SIR2 family)	-	+	+	-	-	-	GB11699-PA	AT5G09230.7	12619	BDEG_06909	[illegible]
PTP3	Protein Tyrosine Phosphatase Homolog 3, hypothetical	-	+	+	-	-	-	GB18971-PA	AT1G71860.3	-	BDEG_05381	[illegible]
		-	-	-	-	-	-	-	-	-	-	[illegible]
		-	+	-	-	-	-	GB16392-PA	AT2G47090.1	-	BDEG_00170	[illegible]
MRPL4	Putative mitochondrial ribosomal protein L4, imported to mitochondria	-	+	-	-	-	-	GB11146-PA	AT2G20060.1	14958	BDEG_02108	[illegible]
ATPvL2		-	+	+	-	-	-	GB17836-PA	AT4G32530.1	30346	BDEG_01526	[illegible]
		-	+	-	-	-	-	-	AT2G34140.1	-	-	[illegible]
TRN1	Similar to transportin	-	+	+	-	-	-	GB18272-PA	AT2G16950.2	-	BDEG_03855	[illegible]
		-	-	-	-	-	-	-	-	-	-	[illegible]
		-	+	-	-	-	-	-	AT2G38050.1	-	BDEG_02008	[illegible]
		-	-	-	-	-	-	-	-	68633	-	[illegible]
		-	+	-	-	-	-	-	AT3G30841.1	-	-	[illegible]
		-	-	-	-	-	-	-	-	-	-	[illegible]
		-	-	-	+	-	-	-	AT2G38040.2	-	-	[illegible]
		-	-	-	-	-	-	-	-	-	-	[illegible]
		-	+	-	-	-	-	-	-	-	BDEG_00761	[illegible]
		-	+	-	-	-	-	GB14693-PA	AT5G67320.1	38888	BDEG_05459	[illegible]
		-	+	-	-	-	-	GB18611-PA	-	52027	-	[illegible]
		-	+	-	-	-	-	-	AT1G58280.1	5157	-	[illegible]
		-	+	-	-	-	-	-	AT2G21660.2	8994	-	[illegible]
		-	+	-	-	-	-	GB10090-PA	AT4G22285.1	-	BDEG_02266	[illegible]
		-	-	-	-	-	-	-	AT5G23050.1	-	-	[illegible]
		-	-	-	-	-	-	-	-	-	-	[illegible]
		-	+	+	-	-	-	GB10637-PA	AT4G07390.1	28225	-	[illegible]
GLD1	putative glucose-6-phosphate-1-dehydrogenase, cytosolic	-	+	-	-	-	-	GB15779-PA	AT3G27300.3	51995	BDEG_05810	[illegible]
		-	+	-	-	-	-	GB16347-PA	AT4G39120.1	4399	-	[illegible]
		-	+	-	-	-	-	-	AT3G09310.1	9306	-	[illegible]
		-	+	-	-	-	-	GB15689-PA	AT2G39260.1	-	BDEG_08415	[illegible]
		-	-	-	-	-	-	-	AT3G22310.1	-	-	[illegible]
		-	+	-	-	-	-	-	AT5G58200.1	-	-	[illegible]
SPP	Stromal Processing Peptidase	-	+	-	-	-	-	-	AT5G42390.1	22177	-	[illegible]
		-	+	-	-	-	-	-	AT4G23470.3	6131	-	[illegible]
		-	+	-	-	-	-	-	AT3G47390.1	-	BDEG_06656	[illegible]
		-	+	-	-	-	-	GB16840-PA	AT5G18420.1	39088	BDEG_08479	[illegible]
		-	+	-	-	-	-	-	AT2G17695.1	-	-	[illegible]
TNT	tRNA-nucleotidyltransferase, mitochondrial	-	+	+	-	-	-	-	AT1G22660.2	27394	BDEG_01473	[illegible]
ANK7	protein of unknown function with ankyrin repeats	-	-	-	-	-	-	-	-	-	-	[illegible]
		-	+	-	-	-	-	-	AT1G13820.1	-	-	[illegible]
HDA3407	Histone deacetylase	-	+	-	-	-	-	-	AT5G26040.2	-	-	[illegible]
		-	-	-	-	-	-	-	-	-	-	[illegible]
		-	+	-	-	-	-	-	AT2G30600.4	-	-	[illegible]
		-	-	-	-	-	-	-	-	-	-	[illegible]
		-	+	+	-	-	-	GB18615-PA	AT3G56990.1	2213	BDEG_03534	[illegible]
		-	+	-	-	-	-	-	AT3G63190.1	58615	BDEG_07628	[illegible]
VPS52	subunit of GARP complex	-	+	-	-	-	-	GB16280-PA	AT1G71270.1	71762	BDEG_05241	[illegible]
		-	+	-	-	-	-	GB13104-PB	-	-	-	[illegible]
		-	+	-	-	-	-	GB19283-PA	AT1G64480.1	-	BDEG_03653	[illegible]
		-	+	-	-	-	-	-	AT5G22800.1	-	-	[illegible]
		-	-	-	-	-	-	-	-	-	-	[illegible]
		-	-	-	-	-	-	-	-	-	-	[illegible]
		-	-	-	-	-	-	-	-	-	-	[illegible]
		-	-	-	-	-	-	-	-	-	-	[illegible]
		-	-	-	-	-	-	-	-	-	-	[illegible]
		-	-	-	-	-	-	-	-	-	-	[illegible]
		-	+	-	-	-	-	-	-	61956	-	[illegible]
STPK22	Serine/Threonine Protein Kinase Homolog 22 (hypothetical)	-	-	-	-	-	-	-	-	-	-	[illegible]
		-	-	-	-	-	-	-	-	-	-	[illegible]
		-	+	-	-	-	-	-	-	59334	-	[illegible]
		-	+	-	-	-	-	GB20067-PA	AT1G50670.1	-	BDEG_03318	[illegible]
		-	+	-	-	-	-	-	AT3G02110.1	2295	-	[illegible]
		-	-	-	-	-	-	-	-	-	-	[illegible]
		-	-	-	-	-	-	-	-	-	-	[illegible]
		-	+	-	-	-	-	-	-	71164	-	[illegible]
		-	+	+	-	-	-	GB14799-PA	AT5G62030.1	2324	BDEG_03816	[illegible]
		-	+	-	-	-	-	GB11485-PA	AT4G19210.1	-	BDEG_02577	[illegible]
		-	-	-	-	-	-	-	-	-	-	[illegible]
CAX2	CAX family of Cation antiporters, membrane protein	-	+	-	-	-	-	-	AT3G13320.1	-	-	[illegible]
		-	-	-	-	-	-	-	-	-	-	[illegible]
		-	+	-	-	-	-	-	AT3G19960.1	-	-	[illegible]
		-	-	-	-	-	-	-	-	-	-	[illegible]
		-	-	-	-	-	-	-	-	-	-	[illegible]
MTP1	CDF transporter, membrane protein	-	+	+	-	-	-	GB10664-PA	AT2G46800.2	18887	BDEG_04205	[illegible]
		-	-	-	-	-	-	-	-	-	-	[illegible]
SPT2		-	+	-	-	-	-	-	AT4G36480.2	70661	BDEG_03561	[illegible]
		-	+	+	+	-	-	GB17602-PA	AT4G26300.1	18800	BDEG_00145	[illegible]
		-	-	-	-	-	-	-	-	-	-	[illegible]
PYK2,PYK3	pyruvate kinase	-	-	-	-	-	-	-	-	-	-	[illegible]
TRM7	tRNA-2'-O-ribose methyltransferase	-	+	+	-	-	-	GB17707-PA	AT5G01230.1	5019	-	[illegible]
		-	+	-	-	-	-	-	-	-	-	[illegible]
		-	+	-	-	-	-	-	AT2G22610.1	1243	-	[illegible]
		-	+	-	-	-	-	GB20131-PA	-	29648	-	[illegible]
		-	+	-	-	-	-	-	AT4G11800.1	-	-	[illegible]
		-	+	+	-	-	-	GB16533-PA	AT2G38710.2	-	BDEG_07119	[illegible]
		-	+	-	-	-	-	-	AT5G17530.2	-	-	[illegible]
		-	-	-	-	-	-	-	-	-	-	[illegible]
		-	+	-	-	-	-	-	AT5G12850.1	-	-	[illegible]
		-	+	-	-	-	-	GB11347-PA	AT3G55070.1	-	BDEG_00659	[illegible]
		-	+	-	-	-	-	GB12476-PA	AT1G72090.1	1140	BDEG_01511	[illegible]
		-	-	-	-	-	-	-	-	-	-	[illegible]
gerer		-	+	-	-	-	-	GB10960-PA	AT3G07720.1	-	BDEG_03959	[illegible]
		-	+	-	-	-	-	-	AT2G31985.1	-	-	[illegible]
		-	-	-	-	-	-	-	-	-	-	[illegible]
		-	-	-	-	-	-	-	-	-	-	[illegible]
		-	+	-	-	-	-	-	AT4G33110.1	-	BDEG_03739	[illegible]
FBB3		-	-	-	-	-	-	-	-	-	-	[illegible]
		-	+	-	-	-	-	GB17041-PA	AT2G41250.1	-	-	[illegible]
		-	-	-	-	-	-	-	-	-	-	[illegible]
		-	+	-	-	-	-	-	-	-	BDEG_01031	[illegible]
		-	+	-	-	-	-	-	AT5G14420.2	-	-	[illegible]
		-	-	-	-	-	-	-	-	-	-	[illegible]
FAP198	Conserved Uncharacterized Flagellar Associated Protein	+	+	-	+	-	-	-	-	33534	-	[illegible]
ATPvA2		-	+	-	-	-	-	GB11840-PA	AT2G21410.1	53354	BDEG_04661	[illegible]
		-	-	-	-	-	-	-	-	-	-	[illegible]
		-	+	-	-	-	-	GB19484-PA	AT2G41790.1	71803	BDEG_06023	[illegible]
MRPS11	Putative mitochondrial ribosomal protein S11, imported to mitochondria	-	+	-	-	-	-	GB19903-PA	ATCG00750.1	-	BDEG_08340	[illegible]
		-	+	+	-	-	-	GB19812-PA	AT5G05450.1	26008	BDEG_03602	[illegible]
	Starch Branching Enzyme	-	+	-	-	-	-	-	-	-	-	[illegible]
EFG3		-	+	+	-	-	-	GB10073-PA	AT1G18070.2	62149	BDEG_08741	[illegible]
FBB12		-	-	-	-	-	-	-	-	-	-	[illegible]
MSCP1		-	+	-	-	-	-	GB10464-PA	AT4G39460.1	25372	BDEG_00588	[illegible]
		-	-	-	-	-	-	-	-	-	-	[illegible]
		-	+	-	-	-	-	GB12902-PA	AT1G43690.1	20352	BDEG_06974	[illegible]
		-	-	-	-	-	-	-	-	-	-	[illegible]
		-	-	-	-	-	-	-	-	64931	-	[illegible]
possibleHST1		-	+	-	-	-	-	-	AT3G11945.1	-	-	[illegible]
	RNA binding protein	-	+	+	-	-	-	GB12485-PA	AT3G47120.1	19053	BDEG_04309	[illegible]
		-	+	+	-	-	-	GB19960-PA	AT1G79450.1	25382	BDEG_08171	[illegible]
HDA3413	Histone deacetylase	-	-	-	-	-	-	-	-	-	-	[illegible]
		-	+	-	-	-	-	-	AT4G32320.1	-	-	[illegible]
CBP20	putative CBP20	-	+	-	-	-	-	GB16622-PA	AT1G13690.1	17250	-	[illegible]
	protease	-	+	-	-	-	-	GB15408-PA	AT5G26860.1	37292	BDEG_00889	[illegible]
ETK1		-	+	+	-	-	-	GB15348-PA	AT2G26830.1	-	BDEG_02673	[illegible]
		-	+	-	-	-	-	-	AT2G42070.1	15915	-	[illegible]
		-	-	-	-	-	-	-	-	-	-	[illegible]
		-	-	-	-	-	-	-	-	-	-	[illegible]

		-	+	-	-	-	-	-	AT3G06810.1	19300	[illegible]
ARP3	Actin-related protein	-	+	+	-	-	-	GB13344-PA	AT1G13180.1	53141	[illegible]
		-	+	+	-	-	-	GB12622-PA	AT2G46230.1	24178	[illegible]
		-	+	-	-	-	-	-	AT1G49820.1	65901	[illegible]
		-	-	-	-	-	-	-	AT3G08640.1	-	[illegible]
ATPK	Adenosine 5'-phosphosulfate kinase	-	+	-	-	-	-	GB20024-PA	AT4G39940.1	72468	[illegible]
		-	+	-	-	-	-	-	AT2G43945.1	24094	[illegible]
		-	+	-	-	-	-	GB11984-PA	AT1G08780.1	26407	[illegible]
		-	-	-	-	-	-	-	-	-	[illegible]
		-	+	-	-	-	-	-	AT3G10420.2	-	[illegible]
TEF19	Upregulated under low CO2 conditions, IMP dehydrogenase / GMP reductase domain, chloroplast location proposed	-	+	-	-	-	-	GB10104-PA	AT4G27030.1	-	[illegible]
ANK30	protein of unknown function with ankyrin repeats	-	-	-	-	-	-	-	-	-	[illegible]
		-	+	-	-	-	-	-	-	19929	[illegible]
		-	+	-	-	-	-	GB18486-PA	AT4G27630.2	-	[illegible]
		-	-	-	-	-	-	-	-	-	[illegible]
		-	+	+	-	-	-	GB15485-PA	AT1G20920.2	53132	[illegible]
		-	-	-	-	-	-	-	-	-	[illegible]
		-	+	-	-	-	-	GB12497-PA	AT4G11820.2	19038	[illegible]
		-	+	-	-	-	-	-	-	61431	[illegible]
ADCY30	adenylate cyclase	-	-	-	-	-	-	-	-	-	[illegible]
		-	+	-	+	-	-	GB11023-PA	AT3G56460.1	12964	[illegible]
SPL8	U2 snRNP auxiliary factor, large subunit; U2AF	-	+	-	-	-	-	-	AT4G36690.1	64670	[illegible]
CYP744A2	cytochrome P450, unkown function, in CYP711 clan	-	-	-	-	-	-	-	-	-	[illegible]
		-	-	-	-	-	-	-	-	-	[illegible]
		-	-	-	-	-	-	-	-	-	[illegible]
		-	-	-	-	-	-	-	-	-	[illegible]
		-	+	-	-	-	-	-	AT1G36070.1	-	[illegible]
CYP744A3	cytochrome P450, unkown function, in CYP711 clan	-	-	-	-	-	-	-	-	-	[illegible]
		-	-	-	-	-	-	-	-	-	[illegible]
		-	-	-	-	-	-	-	-	-	[illegible]
CYP744B1	cytochrome P450, unkown function, in CYP711 clan	-	-	-	-	-	-	-	-	-	[illegible]
		-	+	+	-	-	-	GB13406-PA	AT5G57280.1	33432	[illegible]
		-	+	-	-	-	-	GB14843-PA	AT5G11860.4	23845	[illegible]
		-	-	-	-	-	-	-	-	-	[illegible]
		-	-	-	-	-	-	-	-	-	[illegible]
		-	+	-	-	-	-	GB13947-PA	AT1G75440.1	-	[illegible]
		-	+	-	-	-	-	GB14728-PA	AT2G22250.3	21970	[illegible]
		-	+	-	-	-	-	GB14451-PA	-	10854	[illegible]
		-	-	-	-	-	-	-	-	-	[illegible]
DOM34	putative Dom34	-	+	+	-	-	-	GB10750-PA	AT4G27650.1	22353	[illegible]
		-	-	-	-	-	-	-	-	-	[illegible]
		-	-	-	-	-	-	-	-	-	[illegible]
		-	+	-	-	-	-	-	-	-	[illegible]
FAP39	Flagellar Associated Protein	-	-	-	-	-	-	-	-	-	[illegible]
MDH3	malate dehydrogenase	-	-	-	-	-	-	-	-	-	[illegible]
ADCY19	adenylate cyclase	-	-	-	-	-	-	-	-	-	[illegible]
		-	-	-	-	-	-	-	-	-	[illegible]
		-	-	-	-	-	-	-	-	-	[illegible]
		-	+	-	+	-	-	GB18952-PA	AT5G47720.1	-	[illegible]
PYK9	pyruvate kinase	-	-	-	-	-	-	-	AT3G52990.1	-	[illegible]
		-	-	-	-	-	-	-	-	-	[illegible]
		-	+	-	-	-	-	-	AT2G35810.1	-	[illegible]
		-	-	-	-	-	-	-	-	-	[illegible]
		-	+	-	-	-	-	GB18610-PA	-	-	[illegible]
		-	+	-	-	-	-	GB13662-PA	AT5G25220.2	-	[illegible]
	alpha-amylase	-	+	-	-	-	-	-	-	-	[illegible]
		-	+	-	-	-	-	-	AT4G09750.1	13138	[illegible]
		-	+	-	-	-	-	-	AT4G00231.1	-	[illegible]
		-	-	-	-	-	-	-	-	-	[illegible]
		-	+	-	-	-	-	-	AT3G18480.1	-	[illegible]
		-	+	-	-	-	-	-	AT1G59840.2	-	[illegible]
		-	+	-	-	-	-	-	AT1G60200.1	35861	[illegible]
AK6	Adenylate Kinase Homolog 6	-	+	-	-	-	-	-	AT2G39270.1	-	[illegible]
		-	+	-	-	-	-	-	AT1G64355.1	67808	[illegible]
SHKD1	dehydroquinate dehydratase/shikimate:NADP oxidoreductase, putative	-	+	-	-	-	-	-	AT3G06350.1	-	[illegible]
		-	-	-	-	-	-	-	-	-	[illegible]
		-	+	-	-	-	-	GB13519-PA	AT1G26110.1	-	[illegible]
MRPS19	Putative mitochondrial ribosomal protein S19, imported to mitochondria	-	+	-	-	-	-	-	ATCG00820.1	-	[illegible]
		-	+	-	-	-	-	-	-	61268	[illegible]
		-	+	-	-	-	-	-	AT4G10030.1	65077	[illegible]
ATF1	glucosamine--fructose-6-phosphate aminotransferase	-	+	+	-	-	-	GB18357-PA	AT3G24090.1	28862	[illegible]
		-	-	-	-	-	-	-	-	-	[illegible]
		-	+	-	-	-	-	-	AT2G14260.2	25330	[illegible]
		-	-	-	-	-	-	-	-	-	[illegible]
		-	+	-	-	-	-	GB14269-PA	AT3G54440.1	3538	[illegible]
		-	+	-	-	-	-	GB13045-PA	AT1G79440.1	34350	[illegible]
		-	+	-	-	-	-	GB15298-PA	AT4G28830.1	31452	[illegible]
		-	+	-	-	-	-	-	AT4G28370.1	71361	[illegible]
CHK	carbohydrate kinase	-	+	-	-	-	-	-	AT2G16790.1	-	[illegible]
		-	+	-	-	-	-	-	-	-	[illegible]
		-	+	+	-	-	-	GB14908-PA	AT1G60430.2	69782	[illegible]
		-	-	-	-	-	-	-	-	-	[illegible]
		-	-	-	-	-	-	-	-	-	[illegible]
CNX5	molybdopterin cofactor sulfurase family protein	-	+	-	-	-	-	GB19617-PA	AT5G44720.1	5555	[illegible]
		-	+	-	-	-	-	-	AT5G20380.1	-	[illegible]
		-	+	-	-	-	-	-	-	20477	[illegible]
		-	-	-	-	-	-	-	-	-	[illegible]
HSF1	Heat shock transcription factor 1	-	+	-	-	-	-	GB13400-PA	AT1G32330.1	-	[illegible]
		-	+	-	-	-	-	-	AT5G17560.1	-	[illegible]
FAD5		-	+	-	-	-	-	GB11969-PA	AT3G15850.1	33153	[illegible]
		-	+	-	-	-	-	-	-	-	[illegible]
		-	+	-	-	-	-	-	AT5G24690.1	-	[illegible]
		-	+	-	-	-	-	-	-	-	[illegible]
POLZ1	DNA polymerase zeta	-	+	+	-	-	-	GB18804-PA	AT1G67500.1	-	[illegible]
		-	+	-	-	-	-	-	AT5G20070.1	5532	[illegible]
		-	-	-	-	-	-	-	-	-	[illegible]
		-	+	+	-	-	-	GB10746-PA	AT2G41835.1	-	[illegible]
OGT1		-	+	-	-	-	-	GB13441-PA	AT3G11540.1	359	[illegible]
		-	+	-	-	-	-	-	AT1G50900.1	-	[illegible]
		-	+	-	-	-	-	GB16832-PA	AT3G06530.1	-	[illegible]
TIC32	Short-chain dehydrogenase 32 kDa translocon at the inner membrane of chloroplasts	-	+	-	-	-	-	GB11795-PA	AT4G23420.2	-	[illegible]
DNJ14	DnaJ-like protein 14, probably cytosolic	-	-	-	-	-	-	-	-	-	[illegible]
		-	+	-	-	-	-	-	-	35322	[illegible]
PFP2	phosphofructokinase	-	+	-	-	-	-	GB17113-PA	-	-	[illegible]
		-	+	-	-	-	-	-	AT1G31220.1	-	[illegible]
		-	-	-	-	-	-	-	-	-	[illegible]
XDH1	xanthine dehydrogenase/oxidase	-	+	-	-	-	-	GB17045-PA	AT4G34890.1	36810	[illegible]
		-	-	-	-	-	-	-	-	-	[illegible]
	hypothetical transcription factor Hap5a-like protein	-	+	-	-	-	-	GB30092-PA	AT5G63470.2	26154	[illegible]
		-	-	-	-	-	-	-	-	-	[illegible]
		-	+	-	-	-	-	-	AT1G47750.1	-	[illegible]
		-	+	-	-	-	-	GB10166-PA	-	53975	[illegible]
		-	+	-	-	-	-	-	AT5G03330.2	6451	[illegible]
		-	+	-	-	-	-	-	AT4G14305.1	25325	[illegible]
		-	-	-	-	-	-	-	-	-	[illegible]
		-	+	-	-	-	-	-	-	1353	[illegible]
DHC11	Flagellar inner dynein arm heavy chain 11	-	-	-	+	-	-	-	-	-	[illegible]
		-	-	-	-	-	-	-	-	-	[illegible]
		-	-	-	-	-	-	-	-	-	[illegible]
		-	+	-	-	-	-	GB19370-PA	AT5G51300.3	26367	[illegible]
		-	+	-	-	-	-	-	AT3G57030.1	-	[illegible]
		-	-	-	-	-	-	-	-	-	[illegible]
		-	+	-	-	-	-	-	-	33417	[illegible]
FtsH11	membrane AAA-metalloprotease, mitochondrial	-	-	-	-	-	-	-	-	-	[illegible]
		-	+	+	-	-	-	GB17389-PA	AT2G38130.2	-	[illegible]
		-	-	-	-	-	-	-	-	-	[illegible]
		-	+	-	-	-	-	GB18317-PA	AT1G48520.1	34352	[illegible]
		-	-	-	-	-	-	-	-	22870	[illegible]
MME3	NADP malic enzyme	-	-	-	-	-	-	-	-	-	[illegible]
CYG4	guanylate cyclase	-	-	-	-	-	-	-	-	-	[illegible]
CGE2	Chloroplast GrpE homolog 2	-	-	-	-	-	-	-	-	-	[illegible]
		-	+	-	-	-	-	-	AT4G04670.1	26173	[illegible]
		-	-	-	-	-	-	-	-	-	[illegible]
		-	+	-	-	-	-	GB15231-PA	AT1G17710.1	22824	[illegible]
		-	+	-	-	-	-	GB16933-PA	AT3G44620.1	-	[illegible]
	sulfhydryl oxidase	-	+	-	-	-	+	GB13302-PA	-	15676	[illegible]
		-	+	-	-	-	-	-	-	-	[illegible]
HDA3409	Histone deacetylase	-	+	-	-	-	-	-	-	28875	[illegible]
ADCY20	adenylate cyclase	-	-	-	-	-	-	-	-	-	[illegible]
		-	-	-	-	-	-	-	-	-	[illegible]
		-	+	-	-	-	-	-	AT1G52590.1	-	[illegible]
ADCY21	adenylate cyclase	-	-	-	-	-	-	-	-	-	[illegible]
		-	-	-	-	-	-	-	-	-	[illegible]
		-	-	-	-	-	-	-	-	-	[illegible]
DHC7	Dynein heavy chain 7	-	+	-	+	-	-	GB14230-PA	-	61597	[illegible]
		-	-	-	-	-	-	-	-	-	[illegible]
		-	+	+	-	-	-	GB19625-PA	AT5G57990.1	2843	[illegible]
		-	+	-	-	-	-	-	AT2G35610.1	-	[illegible]
CYG3	putative guanylate cyclase	-	-	-	-	-	-	-	-	-	[illegible]
		-	-	-	-	-	-	-	-	-	[illegible]
		-	+	-	-	-	-	GB10938-PA	AT2G34750.1	-	[illegible]
		-	+	-	-	-	-	-	AT3G61540.1	-	[illegible]
		-	+	-	-	-	-	GB11933-PA	AT5G11720.1	-	[illegible]
CHR3412		-	-	-	-	-	-	-	-	-	[illegible]
		-	+	-	-	-	-	GB12768-PA	AT5G60930.1	-	[illegible]
PIMT1		-	+	-	-	-	-	GB10882-PA	AT5G50240.2	35150	[illegible]

		-	-	-	-	-	-	-	-	-	-	-	-	117309	-	-	-	-	-	-	-	-	-	-	-	-	-	-	-	-	-	-	-	-	-	-	-	-	-	-	-	-	-	-	-	-	-	-	-	-	-	-
		-	+	+	-	-	-	GB14411-PA	AT5G56680.1	71352	BDEG_07461	CE05684	163999	117306	211412	cgd8_350	CMR205C	-	DDB0189775	CG6796-PA	19171415	159.m00096	ENSGALP00000027851	-	NP_078954.3	LmjF34.2340	125127	27602	68452	184858	NCU06866	Os01g27520.1	19977	GSPATP00013875001	8755	57185	108671	PFB0525w	745198	RO3G_00195.1	YCR024C	SPBC1198.10c	GLEAN3_09446	SINFRUP00000143471	106.m00167	26191	TA14880	59.m03518	53048	85661.m00062	Tb927.4.2310	UM04442.1
		-	+	-	-	-	-	-	AT5G11050.1	39143	-	-	-	117291	-	cgd6_2250	-	-	-	CG9045-PA	-	-	-	-	-	-	-	-	-	-	-	-	6990	-	-	49319	127211	-	172369	-	-	-	-	-	-	-	-	8.m00188	-	-	-	-
		-	+	+	-	-	-	GB12723-PA	AT2G47990.1	38045	BDEG_07429	CE25232	172738	117285	286747	cgd3_2270	CMQ382C	-	DDB0185580	CG3071-PA	-	19.m00311	ENSGALP00000029076	GL50803_15026	NP_115551.2	LmjF23.0030	226017	32803	79190	231042	NCU09843	Os07g12320.1	21638	GSPATP00017221001	43713	163919	108976	-	644959	RO3G_12718.1	YMR093W	SPBC428.19c	GLEAN3_28036	SINFRUP00000149253	67.m00197	269030	TA16915	49.m03158	30805	96252.m00270	Tb927.8.1980	UM04260.1
		-	+	-	-	-	-	-	AT4G11570.2	26283	-	-	-	117282	-	-	CMF057C	-	-	-	-	-	-	-	-	-	-	-	-	-	-	Os09g39560.1	-	-	-	45351	-	-	548466	-	-	-	-	-	-	22075	-	-	-	-	-	-
		-	+	-	-	-	-	-	AT3G48420.1	52151	-	-	-	117277	-	-	CMJ188C	-	-	-	-	-	-	-	-	-	-	-	-	-	-	Os03g36750.1	15089	-	12969	146609	-	-	824722	-	-	-	-	-	-	23357	-	-	-	-	-	-
		-	-	-	-	-	-	-	-	3421	-	-	-	117250	-	-	-	-	-	-	-	-	-	-	-	-	-	-	-	-	-	-	-	-	11563	-	-	-	-	-	-	-	-	-	-	-	-	-	-	84394.m00079	-	-
HYD3	iron hydrogenase/prelamin A binding protein homology	-	+	+	-	-	-	GB14880-PA	AT4G16440.1	-	BDEG_00187	CE29883	150962	117241	298953	cgd1_190	CML104C	OTTDARP00000022848	DDB0189262	CG17683-PA	19170843	233.m00102	ENSGALP00000008600	GL50803_33030	OTTHUMP00000151179	LmjF05.0230	111940	22967	47235	210509	NCU03204	Os03g53750.1	10324	GSPATP00022987001	48846	146182	129368	PFF0685c	808286	RO3G_11045.1	YNL240C	SPCC1450.10c	-	SINFRUP00000168560	17.m00284	262126	TA05450	49.m03293	61804	-	Tb10.406.0260	UM02802.1
		-	+	-	-	-	-	-	-	-	-	-	118723	117237	369895	-	-	-	-	-	-	-	ENSGALP00000034743	-	-	-	-	-	-	105257	NCU05512	-	-	-	-	-	-	-	-	-	-	-	-	SINFRUP00000178583	-	-	-	-	-	-	-	-
HTV2	Histone H3 variant	-	-	-	-	-	-	-	-	-	-	-	-	117234	-	-	-	-	-	-	-	-	-	-	-	-	-	-	-	-	-	-	-	-	-	-	-	-	-	-	-	-	-	-	-	-	-	-	-	-	-	-
		-	+	+	-	-	-	GB15178-PA	AT3G04680.2	27837	BDEG_01395	CE17941	222774	117224	254004	cgd5_2880	CMO312C	-	DDB0186537	CG5970-PA	19170859	55.m00185	ENSGALP00000011907	-	OTTHUMP00000096149	LmjF30.2410	226039	32061	30302	236308	NCU06314	Os02g12570.1	16034	-	15508	119032	141114	-	736228	RO3G_01049.1	YOR250C	SPAC22H10.05c	-	SINFRUP00000143850	-	-	TA06270	83.m01280	54844	85859.m00407	Tb927.6.3690	UM03070.1
		-	-	-	-	-	-	-	-	-	-	-	-	117219	-	-	-	-	-	-	-	-	-	-	-	-	-	-	-	-	-	-	1251	-	-	-	-	-	-	-	-	-	-	-	-	-	-	-	-	-	-	-
RML	protein of unknown function, RimM-like	-	+	-	-	-	-	-	AT5G46420.1	-	-	-	-	117217	-	-	CMS193C	-	-	-	-	-	-	-	-	-	-	-	-	-	-	Os05g49130.2	33018	-	43626	133550	-	-	278191	-	-	-	-	-	-	262862	-	-	-	-	-	-
		-	-	-	-	-	-	-	-	20630	-	-	-	117208	-	-	-	-	-	-	-	-	-	GL50803_5180	OTTHUMP00000168365	-	-	-	-	-	-	-	-	-	-	-	-	-	587179	-	-	-	-	-	-	-	-	-	-	-	-	-
		-	+	-	-	-	-	-	AT5G10730.1	-	-	-	-	117171	-	-	-	-	-	-	-	-	-	-	-	LmjF14.0290	-	-	-	-	-	Os02g35039.1	32032	GSPATP00000381001	45515	6130	144098	-	763631	-	YLR290C	SPCC1840.09	-	-	4.m00394	36709	-	-	-	-	Tb927.7.4440	-
		-	+	-	-	-	-	-	AT2G27450.1	19125	-	-	-	117164	-	-	-	-	-	-	-	-	-	-	-	-	-	-	-	77053	-	Os02g33080.1	-	-	38930	204180	-	-	594963	-	-	-	-	-	-	-	-	-	-	-	-	-
		-	-	-	-	-	-	-	-	-	-	-	-	117116	-	-	-	-	-	-	-	-	-	-	-	-	-	-	-	-	-	-	-	-	-	-	-	-	-	-	-	-	-	-	-	-	-	-	-	-	-	-
BIOF	7-keto-8-aminopelargonic acid synthase	-	+	-	-	-	-	-	AT5G04620.1	-	-	-	-	117112	-	-	-	-	-	-	-	-	-	-	-	-	-	-	-	-	-	Os10g41150.1	5827	-	-	188417	-	-	423768	-	YDR232W	-	-	-	-	-	-	-	-	-	-	-
		-	-	-	-	-	-	-	-	-	-	-	-	117106	-	-	-	-	-	-	-	-	-	-	-	-	-	-	-	-	-	-	-	-	-	-	-	-	-	-	-	-	-	-	-	-	-	-	-	-	-	-
PUS2	tRNA pseudouridine synthase, putative	-	+	-	-	-	-	-	AT3G06950.1	58785	-	-	109251	117104	-	-	CMQ041C	-	-	-	-	-	-	-	-	-	-	-	-	-	-	Os05g19954.1	22599	-	46408	56067	142435	-	721191	-	-	-	-	-	-	264420	TA07595	-	-	-	-	-
HTA37	Histone H2A	-	-	-	-	-	-	-	-	-	-	-	-	-	-	-	-	-	-	-	-	-	-	-	-	-	-	-	-	-	-	-	-	-	-	-	-	-	-	-	-	-	-	-	-	-	-	-	-	-	-	-
APC11	Anaphase promoting complex subunit 11	-	+	+	-	-	-	GB17329-PA	AT3G05870.1	9374	BDEG_08012	CE00978	198714	117084	-	cgd1_2640	CMS386C	OTTDARP00000011547	DDB0187183	-	-	153.m00094	-	GL50803_8241	OTTHUMP00000182653	LmjF35.4500	178662	-	-	235548	NCU11300	Os03g19059.1	20179	GSPATP00029441001	10271	56804	108356	PFF1180w	292476	-	YDL008W	SPAC343.03	-	SINFRUP00000132911	65.m00210	37040	-	-	30014	93835.m00202	Tb09.211.1655	UM02288.1
		-	-	-	-	-	-	-	-	-	-	-	-	117083	-	-	-	-	-	-	-	-	-	-	-	-	-	-	-	-	-	-	-	-	-	-	-	-	-	-	-	-	-	-	-	-	-	-	-	-	-	-
		-	+	+	-	-	-	-	AT2G18250.1	-	BDEG_07740	CE31383	149109	117035	-	cgd7_3480	CMT176C	-	DDB0219342	CG10575-PA	-	53.m00191	-	GL50803_7139	OTTHUMP00000181214	LmjF32.2070	-	-	70370	-	-	Os07g08210.3	37130	GSPATP00028848001	48302	158602	127190	-	593919	RO3G_14912.1	YGR277C	SPAC1F12.08	GLEAN3_25758	SINFRUP00000164239	-	6416	-	-	-	96258.m00145	Tb11.01.6920	UM05745.1
		-	+	-	-	-	-	GB14677-PA	AT2G35040.1	20345	BDEG_04115	CE37714	20325	116979	298907	-	CML194C	OTTDARP00000019854	DDB0217901	-	-	-	ENSGALP00000005643	-	OTTHUMP00000163981	-	217098	18908	-	60966	NCU02629	Os08g10570.2	23261	-	23429	139786	109486	-	833594	RO3G_16533.1	YMR120C	SPCPB16A4.03c	-	SINFRUP00000139899	-	28330	-	-	60868	-	-	UM01023.1
		-	-	-	-	-	-	-	-	-	-	-	-	116962	-	-	-	-	-	-	-	-	-	-	-	-	-	-	-	-	-	-	-	-	-	-	-	-	-	-	-	-	-	-	-	-	-	-	-	-	-	-
		-	+	-	-	-	-	-	AT5G51270.1	3218	-	CE31033	53959	116959	280934	-	-	-	-	-	-	-	ENSGALP00000015600	-	OTTHUMP00000115867	-	228846	-	-	-	-	Os06g04880.2	-	-	-	161314	-	-	830380	-	-	-	GLEAN3_00073	SINFRUP00000145494	-	-	-	42.m03447	51565	-	-	-
		-	+	-	-	-	-	-	AT3G21580.2	-	-	-	-	116945	-	-	-	-	-	-	-	-	-	-	-	-	-	-	-	-	-	Os05g33230.1	3934	-	-	203642	-	-	418672	-	-	-	-	-	-	-	-	-	-	-	-	-
CUL3		-	+	-	-	-	-	GB18897-PA	AT4G02570.3	52007	-	-	-	116937	-	cgd4_3150	-	OTTDARP00000022286	-	-	19069150	1.m00738	-	-	-	LmjF24.2290	-	34736	81152	-	-	Os01g27150.1	18216	GSPATP00037059001	29317	107536	109188	PF08_0094	828007	RO3G_05393.1	YDL132W	-	GLEAN3_02933	-	6.m00351	267964	TA10985	80.m02207	-	80222.m00144	Tb927.8.5970	UM01087.1
		-	+	-	-	-	-	GB11454-PA	AT3G07050.1	11767	BDEG_04232	CE02270	221992	116921	271417	cgd4_4040	CMT043C	OTTDARP00000001975	DDB0187301	-	-	-	ENSGALP00000002452	GL50803_16498	OTTHUMP00000023375	LmjF33.2400	145774	27175	1547	88115	NCU03066	Os01g27730.1	11354	GSPATP00036057001	2182	138131	140130	-	-	RO3G_06983.1	YER006W	SPBC26H8.08c	GLEAN3_10114	SINFRUP00000164824	53.m00196	40985	-	-	23415	80746.m00312	Tb11.02.0240	UM05112.1
FtsH3	membrane AAA-metalloprotease, mitochondrial	-	+	-	-	-	-	GB16186-PA	AT2G29080.1	72112	BDEG_01338	CE34400	177529	116904	287805	cgd1_3360	CME055C	-	DDB0185924	CG6512-PB	-	-	ENSGALP00000016620	-	NP_006787.1	LmjF34.1060	127065	16146	30699	107005	NCU01479	Os05g38400.3	21043	GSPATP00023836001	23646	227865	142856	PF11_0203	808632	RO3G_04828.1	YMR089C	SPBC543.09	GLEAN3_10966	SINFRUP00000155357	45.m00242	41306	TA13125	20.m00332	30182	-	Tb927.4.3300	UM00898.1
		-	+	+	-	-	-	GB17281-PA	AT1G11930.2	14200	BDEG_06611	CE02615	138840	116897	228283	cgd5_620	CMS406C	-	DDB0218117	-	-	35.m00231	ENSGALP00000004889	GL50803_15041	OTTHUMP00000177353	LmjF23.1480	231455	34574	-	178404	NCU03579	Os05g05740.1	9553	GSPATP00008789001	5512	105198	108136	PFI0965w	811711	RO3G_03526.1	YBL036C	SPAC644.09	-	SINFRUP00000154212	12.m00521	31757	TA13895	641.m00171	61518	81839.m00104	Tb927.5.1280	UM02631.1
AGA	alpha-galactosidase	-	+	-	-	-	-	GB10294-PA	AT3G56310.1	29988	-	CE06273	154474	116873	-	-	-	OTTDARP00000023945	DDB0215062	CG5731-PA	-	-	ENSGALP00000019396	-	OTTHUMP00000028744	-	209340	25259	-	50257	-	Os07g48160.1	-	GSPATP00014638001	-	68636	-	-	249183	RO3G_03726.1	-	SPAC869.07c	GLEAN3_19040	SINFRUP00000156777	266.m00024	-	-	583.m05316	23253	85228.m00078	-	UM04503.1
DDC1	aromatic-aminoacid decarboxylase	-	+	-	-	-	-	GB18462-PA	AT2G20340.1	16213	BDEG_03335	CE28344	158583	116869	230165	-	-	OTTDARP00000021565	-	CG10697-PA	-	-	ENSGALP00000021345	-	NP_001076440.1	LmjF16.0420	139922	19231	70090	98001	NCU08275	Os07g25590.1	15785	GSPATP00035189001	-	187205	138485	-	816969	RO3G_00030.1	-	-	GLEAN3_21931	SINFRUP00000158344	167.m00070	14772	-	-	21756	-	Tb927.8.5680	UM06083.1
		-	+	-	-	-	-	-	AT4G10300.1	67075	-	-	-	116860	-	-	-	-	-	-	-	-	-	-	-	-	-	-	-	-	-	Os04g36760.1	-	-	-	144325	-	-	816966	-	-	-	-	-	-	37584	-	-	-	-	-	-
		-	+	-	-	-	-	GB19032-PA	AT2G21070.2	28636	-	CE31860	204861	116855	387982	cgd6_2010	-	OTTDARP00000021426	DDB0203108	CG7544-PA	-	-	ENSGALP00000023989	-	OTTHUMP00000183007	-	121844	27301	2339	121407	NCU11362	Os02g02880.2	20321	GSPATP00035406001	-	152340	157247	-	195974	RO3G_00407.1	-	SPAC27D7.08c	-	SINFRUP00000144250	48.m00202	11744	-	44.m02747	28751	-	-	UM03926.1
		-	-	-	-	-	-	-	-	-	-	-	-	116839	-	-	-	-	-	CG4402-PA	-	-	-	-	-	-	-	-	-	-	-	-	-	-	-	-	-	-	-	-	-	-	-	-	-	-	-	-	-	-	-	-
E2F1	E2F family homologue	-	+	-	-	-	-	GB18882-PA	-	7640	-	CE20390	92886	116834	224251	cgd6_1430	CMT067C	OTTDARP00000022548	-	CG1071-PA	19068696	-	-	-	-	-	171492	-	54558	-	-	-	-	GSPATP00023524001	47264	-	155323	-	-	-	-	-	GLEAN3_06753	SINFRUP00000173807	232.m00052	-	-	-	50818	82360.m00291	-	-
		-	+	-	-	-	-	GB18097-PA	AT1G71800.1	23269	BDEG_00121	CE16126	160825	116831	392922	cgd1_1310	CMF108C	OTTDARP00000023972	DDB0218243	CG11266-PE	19173364	-	ENSGALP00000038998	-	OTTHUMP00000019616	LmjF25.0500	-	-	30472	132864	NCU03311	Os11g07490.1	6187	-	8451	9554	137794	PFI1600w	754396	RO3G_14170.1	YGL044C	SPAC644.16	GLEAN3_17830	SINFRUP00000133041	-	20421	-	76.m01552	56724	-	Tb927.7.3730	UM02881.1
		-	+	-	-	-	-	-	AT3G20780.1	66743	-	-	-	116827	-	-	CMT273C	-	-	-	-	-	-	-	-	-	-	15735	-	-	-	Os09g10770.1	11079	-	15042	146513	-	-	-	-	-	-	-	-	-	40335	-	-	-	-	-	-
		-	+	-	-	-	-	-	AT1G53670.1	16484	-	-	104871	116803	-	-	CMP201C	-	-	-	-	-	-	-	-	-	-	-	55441	144955	-	Os06g27760.1	20711	-	7718	150487	-	-	568844	-	-	-	-	SINFRUP00000127900	-	33576	-	-	-	-	-	-
		-	-	-	-	-	-	-	-	-	-	-	-	116793	-	-	-	-	-	-	-	-	-	-	-	-	-	-	-	-	-	-	-	-	-	-	-	-	-	-	-	-	-	-	-	-	-	-	-	-	-	-
		-	+	-	-	-	-	GB14583-PA	AT5G02080.1	54169	BDEG_04920	CE22938	135719	116788	254327	cgd4_2250	CMQ096C	OTTDARP00000023704	DDB0204867	-	-	-	ENSGALP00000007780	-	OTTHUMP00000008431	LmjF25.1900	110784	30065	81838	163564	NCU09090	Os02g36550.1	30052	GSPATP00015070001	10005	162138	158649	PFD0610w	710235	RO3G_14618.1	YIL083C	SPCC4B3.18	GLEAN3_00456	SINFRUP00000147336	319.m00014	15545	TA12810	641.m01494	21703	-	Tb927.3.1970	UM03699.1
		-	+	-	-	-	-	GB10680-PA	AT2G32295.1	-	BDEG_00242	CE19142	-	116773	-	cgd8_3000	-	OTTDARP00000010014	DDB0220588	CG10483-PA	-	50.m00205	ENSGALP00000006180	-	-	-	-	33138	-	-	NCU03278	Os12g40340.1	36846	-	46016	216630	-	PFF0365c	216767	RO3G_01075.1	-	SPAC227.01c	GLEAN3_19111	SINFRUP00000164180	72.m00116	23201	TA11745	-	-	-	-	UM00073.1
		-	+	-	-	-	-	-	AT1G50575.1	-	-	-	108314	116762	-	-	-	-	-	-	-	-	-	-	-	LmjF15.0040	-	-	-	224968	-	Os03g39010.1	5215	-	-	128217	-	PFD0670c	419160	-	-	-	-	-	-	-	-	46.m01701	-	-	Tb927.5.1460	-
		-	+	+	-	-	-	GB17415-PA	AT1G64790.1	52278	BDEG_05638	CE30024	172201	116760	-	cgd8_1930	CMH194C	OTTDARP00000002110	DDB0205796	CG17514-PA	-	137.m00089	ENSGALP00000011775	GL50803_93275	NP_006827.1	LmjF18.0820	223326	8718	58726	168356	NCU05803	Os03g51140.1	18507	GSPATP00017081001	49748	159348	129738	MAL13P1.26	573768	RO3G_09454.1	YGL195W	SPAC18G6.05c	-	SINFRUP00000180570	51.m00184	270116	-	44.m02663	58366	94687.m00115	Tb10.389.0150	UM04443.1
PDF1B		-	+	-	-	-	-	-	AT5G14660.2	33186	-	-	-	116759	-	-	CME111C	-	-	-	-	-	-	-	-	-	-	-	52822	-	-	Os01g45070.1	5618	-	-	147838	-	PFI0380c	173925	-	-	-	-	-	-	30977	-	35.m00903	-	-	-	-
CCT7	T-complex protein , eta subunit	-	+	+	-	-	-	GB10561-PA	AT3G11830.1	59786	BDEG_02850	CE28255	150692	116746	250547	cgd4_3860	CMT558C	-	DDB0183853	CG8351-PA	19069314	3.m00636	ENSGALP00000036304	GL50803_16124	OTTHUMP00000041016	LmjF35.3860	213954	19218	83001	238513	NCU09700	Os06g47320.1	9055	GSPATP00003544001	37131	178205	117988	PFC0350c	827209	RO3G_04764.1	YJL111W	SPBC25H2.12c	GLEAN3_04700	SINFRUP00000128352	62.m00245	13653	TA03370	113.m00012	51102	94090.m00038	Tb09.211.2570	UM00565.1
		-	+	-	-	-	-	-	AT3G10600.1	-	BDEG_04749	-	119893	116724	211085	-	-	-	DDB0205912	-	-	-	-	-	-	-	-	-	-	-	-	Os11g05690.1	21573	-	15324	229662	156002	-	808200	RO3G_15118.1	YHL036W	-	GLEAN3_20211	-	-	13922	-	-	-	-	Tb927.6.4660	-
		-	-	-	-	-	-	-	-	-	-	-	-	116723	-	-	-	-	-	-	-	-	-	-	-	-	-	-	-	-	-	-	-	-	-	-	-	-	-	-	-	-	-	-	-	-	-	-	-	-	-	-
		-	+	-	-	-	-	-	AT1G63610.2	70357	-	-	-	116701	-	-	CMQ124C	-	-	-	-	-	-	-	-	-	-	-	-	-	-	Os02g58650.1	-	-	42457	193876	-	PFE1330c	206656	-	-	-	-	-	-	4296	-	-	-	-	-	-
		-	-	-	-	-	-	-	-	-	-	-	-	116696	-	-	-	-	-	-	-	-	-	-	-	-	-	-	-	-	-	Os03g12790.1	-	-	-	-	-	-	-	-	-	-	-	-	-	-	-	-	-	-	-	-
		-	+	-	-	-	-	-	AT1G70070.1	28826	-	-	-	116679	-	-	CMO352C	-	-	-	-	-	-	-	-	-	-	-	-	-	-	Os02g50560.2	19658	-	-	141731	-	-	-	-	-	-	-	-	-	262824	-	-	-	-	-	-
		-	+	-	-	-	-	-	AT1G44575.1	-	-	-	-	116665	-	-	-	-	-	-	-	-	-	-	-	-	-	-	-	-	-	Os01g64960.1	33644	-	-	146248	-	-	816277	-	-	-	-	-	-	-	-	-	-	-	-	-
		+	+	-	-	-	-	GB18995-PA	-	-	BDEG_06663	-	219244	116664	-	-	-	OTTDARP00000020651	-	CG31623-PA	-	-	ENSGALP00000005150	GL50803_11885	OTTHUMP00000080945	LmjF36.4890	115136	37938	77107	131384	-	-	-	GSPATP00022750001	-	-	135046	MAL13P1.238	-	-	-	-	-	SINFRUP00000149902	72.m00164	38735	-	44.m04652	19315	97029.m00082	Tb11.01.5550	-
		-	-	-	-	-	-	-	-	-	-	-	-	116658	-	-	-	-	-	-	-	-	-	-	-	-	-	-	-	-	-	-	-	-	-	-	-	-	-	-	-	-	-	-	-	-	-	-	-	-	-	-
		-	-	-	-	-	-	-	-	27283	-	-	-	116652	-	-	-	-	-	-	-	-	-	-	-	-	-	-	-	-	-	-	36024	-	-	-	-	-	-	-	-	-	-	-	-	269113	-	-	-	-	-	-
		-	+	+	-	-	-	GB12341-PA	AT1G77180.2	52680	BDEG_03964	CE06519	153812	116646	299270	cgd8_4380	-	-	DDB0190349	CG8264-PA	-	45.m00148	ENSGALP00000017080	-	NP_036377.1	-	233248	24891	69261	243279	NCU03799	Os06g11420.1	-	GSPATP00038119001	26061	182359	115734	PFB0875c	787442	RO3G_13467.1	YAL032C	SPCC188.11	-	SINFRUP00000128826	172.m00083	268084	TA13585	44.m02774	51016	94179.m00294	-	UM02618.1
		-	-	-	-	-	-	-	-	-	-	-	-	116637	-	-	-	-	-	-	-	-	-	-	-	-	-	-	-	-	-	-	-	-	-	-	-	-	-	-	-	-	-	-	-	-	-	-	-	-	-	-
		-	-	-	-	-	-	-	-	-	-	-	-	116601	-	-	-	-	-	-	-	-	-	-	-	-	-	-	-	-	NCU11306	-	-	-	-	-	-	-	-	-	-	-	-	-	-	-	-	-	-	-	-	-
	calcium-transporting ATPase	-	-	-	-	-	-	-	-	-	-	-	-	116583	-	-	-	-	-	-	-	-	-	-	-	-	-	-	-	-	-	-	-	-	-	-	-	-	-	-	-	-	-	-	-	-	-	-	-	-	-	-
		-	+	-	-	+	-	GB10687-PA	AT5G18580.1	31049	BDEG_05703	-	161182	116578	203725	cgd6_4380	-	-	-	-	-	-	ENSGALP00000016315	GL50803_17157	OTTHUMP00000027933	LmjF29.1930	110627	1300	64738	161077	-	Os05g05710.1	-	GSPATP00007128001	-	89210	-	-	811180	-	-	-	-	SINFRUP00000139586	86.m00165	-	-	-	57146	81869.m00184	Tb927.3.4470	-
		-	+	-	-	-	-	-	AT2G34090.1	-	-	-	-	116572	-	-	-	-	-	-	-	-	-	-	-	-	-	1225	-	-	NCU11098	Os01g42800.1	35204	-	-	116344	-	-	568731	RO3G_10144.1	YNL011C	-	-	-	-	270110	-	-	-	-	-	UM01874.1
		-	-	-	-	-	-	-	-	-	-	-	-	116571	-	-	CMN155C	-	-	-	-	-	-	-	-	-	-	-	64943	-	-	-	-	-	-	-	158732	-	-	-	-	SPCC965.09	-	-	-	-	-	-	-	-	-	-
		-	-	-	-	-	-	-	-	-	-	-	-	116565	-	-	-	-	-	-	-	-	-	-	-	-	-	-	-	-	-	-	-	-	-	-	-	-	-	-	-	-	-	-	-	-	-	-	-	-	-	-
		-	+	-	-	-	-	-	AT2G07050.1	19883	BDEG_05772	-	173562	116558	-	-	CMJ009C	-	DDB0190523	-	-	-	ENSGALP00000009988	-	OTTHUMP00000115785	LmjF06.0650	198742	18210	29622	-	NCU01119	Os02g04710.1	25264	GSPATP00022597001	21046	206381	-	-	717351	RO3G_15243.1	YHR072W	SPAC13G7.01c	GLEAN3_12600	SINFRUP00000142634	194.m00023	277	-	-	22852	-	Tb927.7.5230	UM02240.1
		-	+	-	-	-	-	GB19533-PA	AT3G27120.1	12251	BDEG_02940	CE09865	159570	116557	271066	-	CMN138C	-	DDB0202133	CG3326-PA	19069628	283.m00077	ENSGALP00000017259	-	OTTHUMP00000024484	LmjF15.0500	212112	16309	3410	-	-	Os06g03940.1	5255	GSPATP00018989001	2208	203033	123421	PFD0385c	174445	-	-	-	-	SINFRUP00000179539	6.m00525	35108	TA15825	-	31346	92022.m00055	Tb927.5.1870	-
		-	-	-	-	-	-	-	-	-	-	-	-	116553	-	-	-	-	-	-	-	-	-	-	-	-	-	-	-	-	-	-	-	-	-	-	-	-	-	-	-	-	-	-	-	-	-	-	-	-	-	-
SulP2	transmembrane component of sulfate-transporting ABC transporter in chloroplast enveloppe	-	-	-	-	-	-	-	-	-	-	-	121961	116547	-	-	CMV042C	-	-	-	-	-	-	-	-	-	-	-	-	-	-	-	-	-	-	-	-	-	673938	-	-	-	-	-	-	-	-	-	-	-	-	-
		-	+	-	-	-	-	-	AT5G46110.4	-	-	-	-	116544	-	-	-	-	-	-	-	-	-	-	-	-	-	-	-	-	-	Os01g13770.3	26943	-	-	154933	-	-	647933	-	YJL193W	-	-	-	-	-	-	-	-	-	-	-
CYP743A1	cytochrome P450, unkown function, in CYP711 clan	-	-	-	-	-	-	-	-	-	-	-	-	116541	-	-	-	-	-	-	-	-	-	-	-	-	-	-	-	-	-	-	-	-	-	-	-	-	557726	-	-	-	-	-	-	-	-	-	-	-	-	-
CK2B	CK2-beta chain	-	+	+	-	-	-	GB12504-PA	AT5G47080.1	54649	BDEG_06590	CE06343	223599	116537	296560	cgd3_3670	CMM005C	OTTDARP00000014948	DDB0186091	CG15224-PF	19069371	6.m00407	-	GL50803_7421	OTTHUMP00000195479	LmjF36.5090	225993	13118	71259	245041	NCU02754	Os07g31280.1	6069	GSPATP00034461001	11285	59578	158138	PF11_0048	826719	RO3G_14078.1	YOR039W	SPAC1851.03	GLEAN3_16763	SINFRUP00000137002	130.m00075	17208	TA06185	59.m00021	6279	89667.m00110	Tb11.01.2590	UM06107.1
CYP746A1	cytochrome P450, unkown function	-	-	-	-	-	-	-	-	-	-	-	-	116510	-	-	-	-	-	-	-	-	-	-	-	-	-	-	-	-	-	-	-	-	-	-	-	-	-	-	-	-	-	-	-	-	-	-	-	-	-	-
	ubiquitin conjugating enzyme	-	+	-	-	-	-	GB18000-PA	AT5G50870.1	69594	BDEG_06658	CE19850	150271	116503	298937	cgd4_210	-	OTTDARP00000022676	-	CG8284-PA	-	-	ENSGALP00000036618	-	NP_005330.1	-	223907	39004	-	235155	NCU01225	Os01g70140.1	25784	GSPATP00022887001	30389	104845	142734	PF13_0301	662201	RO3G_16320.1	YDR177W	SPBC2D10.20	-	SINFRUP00000142439	70.m00134	10985	TA06755	46.m00017	24883	-	-	UM00525.1
PDE3	3',5'-cyclic-nucleotide phosphodiesterase	-	-	-	-	-	-	-	-	10851	-	-	-	116500	-	-	-	-	-	-	-	-	-	-	-	-	-	-	-	-	-	-	-	GSPATP00024350001	-	-	-	-	-	-	-	-	-	-	-	-	-	-	-	-	-	-
RABH1	RABH1	-	+	+	-	-	-	-	-	35265	-	CE07541	169557	116488	-	cgd2_1940	CMQ122C	-	DDB0189756	-	-	37.m00226	ENSGALP00000030639	-	-	LmjF02.0260	-	-	35987	176370	NCU05234	Os07g31370.1	6501	GSPATP00037634001	21535	155254	-	-	800974	-	YLR262C	-	GLEAN3_28209	-	55.m00233	3582	-	-	-	85473.m00033	Tb927.2.2130	UM06104.1
		-	+	-	-	-	-	-	-	-	-	-	-	116475	-	-	-	-	-	-	-	-	-	GL50803_12942	-	-	-	-	-	-	-	-	-	-	-	135395	-	-	-	-	-	SPAC4A8.02c	-	-	118.m00110	-	-	-	-	82181.m00138	-	-
		-	-	-	-	-	-	-	-	-	-	-	-	116461	-	-	CMQ253C	-	-	-	-	-	-	-	-	-	-	-	-	-	-	-	-	GSPATP00001360001	-	-	-	-	-	-	-	-	-	-	-	-	-	-	-	83584.m00120	-	-
		-	+	-	-	-	-	-	AT3G29010.1	25163	-	-	-	116449	-	-	-	-	DDB0190217	-	-	-	-	-	-	-	-	-	3696	-	-	Os08g33810.1	19448	-	12459	196421	138165	PFI1160w	577664	-	-	-	-	-	-	36995	TA03985	83.m01296	-	-	-	-
		-	-	-	-	-	-	-	-	-	-	-	-	116444	-	-	-	-	-	-	-	-	-	-	-	-	-	-	-	-	-	-	-	-	-	-	-	-	-	RO3G_02065.1	-	-	-	-	-	-	-	-	-	-	-	UM02753.1
		-	-	-	-	-	-	-	-	-	-	-	-	116435	-	-	-	-	-	-	-	-	-	-	-	-	-	-	-	-	-	-	-	-	-	-	-	-	-	-	-	-	-	-	-	-	-	-	-	-	-	-
		-	+	-	-	-	-	-	AT3G27110.2	3999	-	-	-	116429	-	-	CMS394C	-	-	-	-	-	-	-	-	-	-	-	-	-	-	Os01g73910.2	-	-	-	-	-	-	174459	-	-	-	-	-	-	21591	-	-	-	-	-	-
PRFB	peptide chain release factor RF-2	-	+	-	-	-	-	GB13490-PA	AT5G36170.2	35500	BDEG_00392	-	-	116426	-	-	CME148C	-	DDB0188136	-	-	-	-	-	-	-	-	-	-	-	-	Os07g36250.2	11334	-	21873	184553	108484	MAL7P1.20	421358	-	-	-	-	-	-	34785	TA07415	-	-	-	-	-
		-	-	-	-	-	-	-	-	-	-	-	-	116423	-	-	-	-	-	-	-	-	-	-	-	-	-	-	-	-	-	-	-	-	-	-	-	-	-	RO3G_03856.1	YLR239C	SPAC4F10.05c	-	-	-	-	-	-	-	-	-	-
		-	+	-	+	-	-	-	AT5G27930.2	25355	BDEG_04471	-	-	116421	-	cgd5_4170	CMG203C	-	-	-	-	-	-	-	-	-	-	34068	-	-	-	Os07g37890.1	-	GSPATP00021612001	9890	13857	-	MAL8P1.109	827706	-	-	-	-	-	287.m00025	31862	TA08300	83.m01213	-	-	-	-
STPK19	Serine/Threonine Protein Kinase Homolog 19	-	+	+	-	-	-	GB19502-PA	AT1G30640.1	12818	BDEG_01490	CE39616	164045	116419	216897	cgd2_1830	CMP312C	OTTDARP00000008562	DDB0219376	CG8637-PA	19068537	5.m00460	ENSGALP00000036572	GL50803_8587	OTTHUMP00000166990	LmjF06.1180	169498	33947	35993	100066	NCU07296	Os05g43570.3	34621	GSPATP00017820001	16032	165372	140772	-	761359	RO3G_14382.1	YNL161W	SPAC821.12	GLEAN3_12503	SINFRUP00000134689	25.m00332	35135	-	-	49578	81529.m00394	Tb927.7.5770	UM04956.1
		-	+	-	-	-	-	GB14956-PA	AT1G14520.1	58690	-	CE37495	152876	116415	230894	-	-	-	DDB0188751	CG6910-PA	-	-	-	-	OTTHUMP00000196606	-	123081	21355	-	171813	NCU08856	Os06g36560.1	-	-	45657	58210	108635	-	418574	RO3G_16516.1	-	-	-	SINFRUP00000129972	-	269590	-	-	-	-	-	UM00029.1
		-	+	-	-	-	-	-	AT5G01930.1	-	-	-	-	116397	-	-	-	-	-	-	-	-	-	-	-	-	-	-	-	-	-	Os05g25480.1	3718	-	-	122605	-	-	672273	RO3G_08893.1	-	-	-	-	-	-	-	-	-	-	-	-
		-	+	-	-	-	-	GB11537-PA	AT5G53850.3	-	BDEG_02364	CE37460	206371	116383	285557	-	CMM212C	OTTDARP00000023142	DDB0186256	CG11134-PA	-	-	ENSGALP00000012691	-	NP_057041.1	LmjF28.1840	210645	29994	70372	197093	NCU09264	Os11g29370.1	-	-	31147	111231	128671	-	827604	RO3G_05404.1	YJR024C	SPAC20H4.05c	GLEAN3_04875	SINFRUP00000164623	55.m00207	36007	-	-	64275	-	-	-
		-	+	-	-	-	-	GB30493-PA_2	AT5G52250.1	-	BDEG_06581	-	134783	116381	371639	-	CMK039C	-	DDB0219336	-	-	-	ENSGALP00000007158	-	NP_001001740.1	-	238291	26379	-	167	-	Os02g53140.1	2347	-	-	24385	-	-	807645	RO3G_04832.1	-	-	-	SINFRUP00000133886	-	-	-	-	21397	-	-	-
		-	+	+	-	-	-	GB17468-PA	AT4G09140.1	55239	BDEG_04376	CE35190	136607	116380	295838	cgd5_2560	CMQ290C	OTTDARP00000021644	DDB0187465	CG11482-PA	19170773	146.m00127	ENSGALP00000019676	GL50803_16149	OTTHUMP00000170616	LmjF24.1420	141265	25323	81389	167864	NCU08309	Os01g72880.1	29370	GSPATP00010239001	54331	126125	119559	PF11_0184	249544	RO3G_08797.1	YMR167W	SPBC1703.04	GLEAN3_15213	SINFRUP00000135651	9.m00384	263509	TA06860	57.m01789	50100	90417.m00143	Tb927.8.6840	UM05208.1
NCR2	NADPH-cytochrome P450 reductase	-	+	-	-	-	-	GB19444-PA	AT4G24520.1	32584	BDEG_01117	CE02018	173459	116375	294831	-	-	OTTDARP00000018819	DDB0190667	CG11567-PA	-	-	ENSGALP00000003187	GL50803_15897	NP_000932.3	LmjF28.1240	187717	16143	77765	187451	NCU09741	Os04g55960.1	14922	GSPATP00027717001	9283	120835	142875	PFI1140w	818445	RO3G_14252.1	YHR042W	SPBC29A10.01	-	SINFRUP00000162470	196.m00061	264678	TA04090	-	63169	84843.m00225	Tb11.02.5420	UM06273.1
		-	+	+	-	-	-	GB15949-PA	AT5G41770.1	26550	BDEG_07138	CE28622	207252	116356	295004	cgd7_3690	-	-	DDB0206216	CG3193-PA	-	208.m00091	ENSGALP00000013717	-	OTTHUMP00000030376	LmjF36.4280	210722	33965	701	162041	NCU02717	Os05g22260.1	8591	GSPATP00009181001	20135	158739	108879	PFD0180c	797324	RO3G_05759.1	YLR117C	SPBC31F10.11c	GLEAN3_18535	SINFRUP00000161445	220.m00037	41106	TA13380	59.m03439	33203	84950.m00117	Tb10.6k15.0680	UM02676.1
		-	+	+	-	-	-	GB11700-PA	AT4G18905.1	2155	BDEG_01811	CE37529	174087	116342	250326	cgd4_2750	CMT215C	-	DDB0167442	CG6751-PA	-	341.m00046	ENSGALP00000036924	GL50803_15531	OTTHUMP00000169078	LmjF24.1490	201468	13313	53681	109727	NCU02729	Os01g69970.1	32681	GSPATP00023154001	8842	43439	140998	PFL1820w	747757	RO3G_12131.1	YLR196W	SPBC1711.16	-	SINFRUP00000133614	81.m00175	38143	TA18660	80.m02222	61806	82490.m00335	Tb927.8.6770	UM00292.1
		-	+	-	-	-	-	GB12178-PA	AT4G28070.2	21262	BDEG_00919	CE16882	168008	116326	289905	-	CMJ050C	OTTDARP00000006900	DDB0191646	CG8520-PA	-	-	ENSGALP00000024652	-	OTTHUMP00000016943	LmjF05.1060	183409	28553	32540	128230	NCU04481	Os08g16480.1	30075	GSPATP00032545001	2542	114973	114824	PFE1090w	262512	RO3G_11627.1	YEL052W	SPBC115.02c	-	SINFRUP00000175538	69.m00275	38813	TA02690	35.m00933	25679	-	Tb927.7.6930	UM00002.1
		-	-	-	-	-	-	-	-	-	-	-	-	116311	-	-	-	-	-	-	-	-	-	-	-	-	-	-	-	-	-	-	-	-	-	-	-	-	-	-	-	-	-	-	-	-	-	-	-	-	-	-
TEF30	Unknown function, chloroplast location proposed	-	+	-	-	-	-	-	AT1G55480.1	-	-	-	-	116298	-	-	CMJ146C	-	-	-	-	-	-	-	-	-	-	-	-	-	-	Os07g07540.1	9992	-	-	117443	-	-	414772	-	-	-	-	-	-	-	-	-	-	-	-	-
RFK2	Putative riboflavin kinase	-	-	-	-	-	-	-	-	-	-	-	-	-	-	-	-	-	-	-	-	-	-	-	-	-	-	-	-	-	-	-	-	-	-	-	-	-	-	-	-	-	-	-	-	-	-	-	-	-	-	-
		-	-	-	-	-	-	-	-	26011	-	-	-	116281	-	-	-	-	-	-	-	-	-	-	-	-	-	-	-	49632	-	-	36110	-	-	-	-	-	-	-	-	-	-	-	-	-	-	-	-	-	-	-
SQD2a		-	+	-	-	-	-	-	AT5G01220.1	52616	-	-	-	116277	-	-	CMR015C	-	-	-	-	-	-	-	-	-	-	11530	-	-	-	Os07g01030.1	-	-	50356	118377	-	-	417124	RO3G_05324.1	-	-	-	-	-	38775	-	-	-	-	-	-
PDE2	3',5'-cyclic-nucleotide phosphodiesterase	-	+	-	-	-	-	-	-	-	BDEG_05838	-	-	116276	-	-	-	-	-	-	-	-	ENSGALP00000019935	-	NP_001003683.1	-	-	-	-	-	-	-	-	-	-	-	-	-	-	-	-	-	-	SINFRUP00000163402	-	261018	-	57.m03117	-	-	-	-
PYR5		-	+	-	-	-	-	GB19369-PA	AT3G54470.1	15088	BDEG_04963	CE00638	222872	116267	297735	-	CMK046C	-	DDB0206404	-	-	-	ENSGALP00000019192	-	OTTHUMP00000172523	LmjF16.0550	217260	32885	82737	81583	NCU03488	Os01g72240.1	27952	-	11740	109596	135354	-	708505	RO3G_14508.1	YEL021W	SPCC330.05c	GLEAN3_03135	SINFRUP00000155998	-	37071	TA19940	55.m04838	51535	-	Tb927.5.3810	UM04214.1
		-	-	-	-	-	-	-	-	-	-	-	-	116259	-	-	-	-	-	-	-	-	-	-	-	-	-	-	-	-	-	-	-	-	-	-	-	-	-	-	-	-	-	-	-	-	-	-	-	-	-	-
		-	-	-	-	-	-	-	AT1G55310.2	-	-	-	-	116256	-	cgd6_2740	-	-	-	-	-	-	-	-	-	-	-	-	-	-	-	-	-	-	-	-	-	-	-	-	-	-	-	-	-	-	-	-	-	96267.m00175	-	-
HY3	Similar to mouse hydrocephaly protein hydin	+	+	-	+	-	-	GB12690-PA	-	62593	BDEG_07678	-	222082	116240	260992	-	-	OTTDARP00000013990	-	-	-	-	ENSGALP00000003781	GL50803_137712	NP_116210.2	LmjF30.1810	122343	18473	78704	30005	-	-	-	GSPATP00035607001	-	69823	155290	PF14_0419	-	-	-	-	-	SINFRUP00000143351	72.m00131	-	-	20.m03960	30117	84511.m00322	Tb927.6.3150	-
		-	-	-	-	-	-	-	-	-	-	-	-	116235	-	-	-	-	-	-	-	-	-	-	-	-	-	-	-	-	-	-	-	-	-	-	-	-	-	-	-	-	-	-	-	-	-	-	-	-	-	-
		-	+	+	-	-	-	GB11055-PA	AT1G49760.1	55048	BDEG_00480	CE03230	164502	116226	250845	cgd6_3010	CMJ286C	-	DDB0192007	CG5119-PG	19069362	193.m00064	ENSGALP00000006018	GL50803_5942	OTTHUMP00000178156	LmjF35.5040	137449	6550	80977	230864	NCU04799	Os04g42600.2	1233	-	23959	216291	108322	PFL1170w	287102	RO3G_11747.1	YER165W	SPAC57A7.04c	GLEAN3_15317	SINFRUP00000136945	-	13224	TA06545	42.m03384	27786	93199.m00037	Tb09.211.0930	UM03494.1
		-	+	-	-	-	-	-	-	-	-	-	-	116197	-	-	-	-	-	CG1417-PC	-	-	-	-	NP_057419.2	-	-	-	-	-	-	-	-	GSPATP00025595001	-	-	-	-	-	-	-	-	-	-	27.m00309	-	-	-	-	-	-	UM00030.1
		-	+	+	-	-	-	GB13895-PA	AT3G04480.1	22120	BDEG_08234	CE39095	141560	116196	240105	cgd4_610	CMS447C	-	DDB0205409	CG1578-PA	19173508	86.m00165	ENSGALP00000015931	-	OTTHUMP00000160179	LmjF25.0300	138598	14389	31983	238043	NCU02670	-	19064	GSPATP00008350001	5702	20916	127038	PFL1080c	573724	RO3G_13066.1	YLR143W	SPBC577.12	-	SINFRUP00000146274	15.m00547	36273	TA13205	20.m03750	49818	-	Tb11.03.0780	UM04996.1
		-	+	-	-	-	-	GB15902-PA	AT1G32340.1	-	-	CE11232	228754	116192	286945	-	-	OTTDARP00000004065	-	-	-	-	ENSGALP00000011795	-	OTTHUMP00000160065	-	73005	-	-	34058	NCU03719	Os04g50760.1	31082	-	-	115789	-	-	590037	-	YML068W	SPAC1002.14	Triad	SINFRUP00000162293	54.m00241	-	-	-	60996	-	-	UM02525.1
		-	-	-	-	-	-	-	-	-	-	-	-	116145	-	-	-	-	-	-	-	-	-	-	-	-	-	-	-	-	-	-	-	-	-	-	-	-	-	-	-	-	-	-	-	-	-	-	-	-	-	-
		-	-	-	-	-	-	-	-	-	-	-	-	116132	-	-	-	-	-	-	-	-	-	-	-	-	-	-	-	-	-	-	-	-	-	-	-	-	-	-	-	-	-	-	-	-	-	-	-	-	-	-
		-	+	-	-	-	-	GB14691-PA	AT1G08370.1	17384	-	CE22543	65950	116128	257928	cgd5_3890	CMM070C	OTTDARP00000008437	DDB0187387	CG11183-PA	-	-	ENSGALP00000008524	-	NP_060873.3	-	77631	-	81215	120311	-	Os12g06020.1	35538	GSPATP00023521001	46257	175733	136630	PF10_0314	825199	-	-	SPBC3B9.21	-	SINFRUP00000157984	-	4253	TA10140	25.m01847	-	-	-	UM02949.1
		-	-	-	-	-	-	-	-	-	-	-	-	116116	-	-	-	-	-	-	-	-	-	-	-	-	-	-	-	-	-	-	-	-	-	-	-	-	-	-	-	-	-	-	-	-	-	-	-	-	-	-
		-	+	-	-	-	-	GB14776-PA	AT4G17650.1	-	-	CE39270	153456	116115	389411	-	CMQ192C	OTTDARP00000022495	DDB0217315	CG9410-PB	-	-	ENSGALP00000013042	-	OTTHUMP00000163623	-	201780	-	4375	183953	-	Os01g56560.1	-	-	-	152681	128404	-	410920	-	YOL008W	SPCC16A11.07	-	SINFRUP00000128886	110.m00084	269976	TA18425	44.m02806	22596	-	Tb927.8.5310	UM05395.1
		-	+	-	-	-	-	-	AT5G06300.1	35169	-	-	-	116096	-	-	CMQ414C	-	DDB0218284	-	-	-	-	-	-	LmjF17.0735	-	-	78760	162910	NCU06711	Os02g41770.1	25182	-	6277	145049	-	-	595075	RO3G_08957.1	YJL055W	-	-	-	-	6418	-	-	61856	-	-	UM03153.1
EIF2B	eukaryotic translation initiation factor 2 alpha-subunit-like	-	+	-	-	-	-	-	AT1G17220.1	-	-	-	-	116089	-	-	-	-	-	-	-	-	-	-	-	-	-	-	-	-	-	Os05g49970.2	-	-	-	182684	-	-	232591	-	-	-	-	-	-	-	TA07605	-	-	-	-	-
		-	+	-	-	-	-	-	-	-	-	-	-	116066	-	-	-	-	DDB0189864	-	-	-	-	-	OTTHUMP00000076153	-	145414	-	-	-	-	-	-	-	-	-	-	-	-	-	-	-	-	-	79.m00151	-	-	-	-	92145.m00166	-	-
		-	-	-	-	-	-	-	-	-	-	-	-	116056	-	-	CMJ282C	-	-	-	-	511.m00032	-	-	-	-	-	-	-	-	-	Os06g43090.1	-	-	-	-	-	-	644073	-	-	-	-	-	-	-	-	-	-	-	-	-
		-	-	-	-	-	-	-	-	-	-	-	-	116043	-	-	-	-	-	-	-	-	-	-	-	-	-	-	-	-	-	-	-	-	-	-	-	-	-	-	-	-	-	-	-	-	-	-	-	-	-	-
MRPL2	Putative mitochondrial ribosomal protein L2, imported to mitochondria	-	+	-	-	-	-	-	ATCG01310.1	-	BDEG_05995	-	-	116036	290713	-	CMP277C	-	-	-	-	-	-	-	NP_057034.2	-	213398	-	-	35830	NCU02757	Os04g16771.1	30408	-	-	-	-	PF11_0337	747143	RO3G_15972.1	YEL050C	SPCC16C4.15	-	SINFRUP00000147401	-	-	TA03645	52.m01650	34775	-	-	UM04511.1
		-	-	-	-	-	-	-	-	-	-	-	-	116033	-	-	-	-	-	-	-	-	-	-	-	-	-	-	-	-	-	-	-	-	-	-	-	-	-	-	-	-	-	-	-	-	-	-	-	-	-	-
		-	+	-	-	-	-	-	-	-	-	CE11123	225477	116006	-	-	-	OTTDARP00000021757	-	-	-	-	ENSGALP00000008766	-	OTTHUMP00000174189	-	-	-	-	127195	NCU10572	-	-	-	45830	-	-	-	-	-	-	-	-	SINFRUP00000173647	-	-	-	-	-	-	-	-
		-	-	-	-	-	-	-	-	-	-	-	-	116001	-	-	-	-	-	-	-	-	-	-	-	-	-	-	-	-	-	-	-	-	-	-	-	-	-	-	-	-	-	-	-	-	-	-	-	-	-	-
		-	-	-	-	-	-	-	-	-	-	-	-	115988	-	-	-	-	-	-	-	-	-	-	-	-	-	-	-	-	-	-	-	-	-	-	-	-	-	-	-	-	-	-	-	-	-	-	-	-	-	-
		-	-	-	-	-	-	-	-	-	-	-	-	115983	-	-	-	-	-	-	-	-	-	-	-	-	-	-	-	-	-	-	-	-	-	-	-	-	-	-	-	-	-	-	-	-	-	-	-	-	-	-
PFD2	Molecular chaperone Prefoldin, subunit 2	-	+	+	-	-	-	GB15399-PA	AT3G22480.1	20181	BDEG_05877	CE23815	167007	115978	372170	cgd7_4630	CMD037C	OTTDARP00000022651	DDB0189393	CG6302-PA	-	298.m00083	-	GL50803_11713	OTTHUMP00000029707	LmjF09.0670	218864	36976	31313	148111	NCU09249	Os11g36050.1	19076	GSPATP00012500001	13931	55333	141655	PF14_0167	641197	RO3G_16859.1	YEL003W	SPAC227.10	-	SINFRUP00000135218	5.m00475	21686	TA08615	83.m00020	19142	83057.m00092	Tb11.01.4520	UM04767.1
		-	+	+	-	-	-	GB11221-PA	AT4G01560.1	-	BDEG_03326	CE28332	20161	115941	382881	cgd2_160	CML272C	OTTDARP00000023658	DDB0186705	CG6712-PA	19069719	221.m00094	ENSGALP00000014284	GL50803_17112	OTTHUMP00000011472	LmjF27.0450	216676	38546	2758	182962	-	Os06g04600.2	4144	GSPATP00004969001	12679	137685	109169	PFI1070c	644687	RO3G_03491.1	YHR088W	SPAC4F8.04	GLEAN3_01164	SINFRUP00000148590	52.m00296	31465	TA04340	50.m00083	-	97354.m00225	Tb11.03.0050	UM00184.1
		-	-	-	-	-	-	-	-	-	-	-	-	115938	-	-	-	-	-	-	-	-	-	-	-	-	-	-	-	-	-	-	-	-	-	-	-	-	-	-	-	-	-	-	-	-	-	-	-	-	-	-
ATPvA3	putative vacuolar proton ATPase subunit A	-	+	-	-	-	-	-	-	-	-	-	-	115936	-	-	-	OTTDARP00000020055	DDB0184117	-	19171426	-	ENSGALP00000005144	GL50803_18470	-	-	-	-	-	-	-	-	-	-	28794	-	-	-	-	-	-	-	-	SINFRUP00000128898	-	40728	-	44.m02753	-	-	-	-
		-	+	-	-	-	-	-	AT3G10690.1	53761	-	-	50156	115934	-	-	CMS243C	-	-	-	-	-	-	-	-	-	-	-	-	-	-	-	29886	-	33633	135715	-	PFL1120c	805680	-	-	-	-	-	-	39845	TA14985	41.m00003	-	-	-	-
LCID	low-CO2-inducible protein	-	-	-	-	-	-	-	-	-	-	-	-	115931	-	-	-	-	-	-	-	-	-	-	-	-	-	-	-	-	-	-	-	-	-	-	-	-	-	-	-	-	-	-	-	-	-	-	-	-	-	-
		-	-	-	-	-	-	-	-	-	-	-	-	115925	-	-	-	-	-	-	-	-	-	-	-	-	-	-	-	-	-	-	-	-	-	-	-	-	-	-	-	-	-	-	-	-	-	-	-	-	-	-
		-	-	-	-	-	-	-	-	-	-	-	-	115917	-	-	-	-	-	-	-	-	-	-	-	-	-	-	-	-	-	-	-	-	-	-	-	-	-	-	-	-	-	-	-	-	-	-	-	-	-	-
		-	-	-	-	-	-	-	-	-	-	-	-	115915	-	-	-	-	-	-	-	-	-	-	-	-	-	-	-	-	-	-	-	-	-	-	-	-	-	-	-	-	-	-	-	-	-	-	-	-	-	-
		-	-	-	-	-	-	-	-	-	-	-	-	115900	-	-	-	-	-	-	-	-	-	-	-	-	-	-	-	-	-	-	-	-	-	-	-	-	-	-	-	-	-	-	-	-	-	-	-	-	-	-
SPT1		-	+	+	-	-	-	GB10698-PA	AT5G23670.2	20733	BDEG_07928	CE27380	165279	115899	369345	-	CMJ240C	OTTDARP00000021174	DDB0183793	CG4162-PA	19170818	6.m00501	ENSGALP00000017050	GL50803_14374	OTTHUMP00000028486	LmjF35.0320	212374	20161	82916	241814	NCU00447	Os01g70380.1	16411	GSPATP00009596001	10378	152834	108183	PFL2210w	834365	RO3G_09773.1	YDR062W	SPAC21E11.08	GLEAN3_14244	SINFRUP00000128825	197.m00071	268266	-	80.m00093	30228	93793.m00171	Tb10.70.3220	UM00941.1
		-	-	-	-	-	-	-	-	-	-	-	-	115896	-	-	-	-	-	-	-	-	-	-	-	-	-	-	-	-	-	-	-	-	-	-	-	-	-	-	-	-	GLEAN3_14949	-	-	-	-	-	-	-	-	-
	Serine/threonine protein kinase	-	-	-	-	-	-	-	-	-	-	-	-	115879	-	-	-	-	-	-	-	-	-	-	-	-	-	-	-	-	-	-	-	-	-	-	-	-	-	-	-	-	-	-	-	-	-	-	-	-	-	-
		-	-	-	-	-	-	-	-	-	-	-	-	115878	-	-	-	-	-	-	-	-	-	-	-	-	-	-	-	-	-	-	-	-	-	-	-	-	-	-	-	-	-	-	-	-	-	-	-	-	-	-
		-	+	-	-	-	-	-	AT5G07100.2	-	-	-	-	115858	-	-	-	-	DDB0217613	-	-	-	-	GL50803_9237	-	-	-	-	-	-	-	Os05g27730.1	12928	-	-	26902	-	-	665964	-	-	-	-	-	-	-	-	-	-	-	-	-
	PDR-like ABC transporter	-	+	-	-	-	-	-	-	21877	-	-	-	115842	-	-	-	-	-	-	-	-	-	-	-	-	-	-	-	-	-	-	-	-	83	-	127859	-	-	-	YDR011W	SPCC18B5.01c	-	-	-	264773	-	-	-	-	-	-
		-	+	+	-	-	-	GB15826-PA	AT5G57950.1	-	BDEG_05780	CE25810	171080	115832	387067	cgd5_2360	CMR331C	-	DDB0202482	-	-	288.m00065	ENSGALP00000006932	-	OTTHUMP00000169606	LmjF16.0290	182313	-	68057	183435	NCU11053	Os09g25310.1	36996	-	-	115990	108853	PFC0785c	653576	RO3G_04649.1	YIL007C	SPAC2H10.02c	-	SINFRUP00000127838	-	34219	TA12400	42.m00053	24455	96423.m00188	Tb927.8.5740	UM02794.1
PRP17	Nuclear pre-mRNA splicing factor, component of the spliceosome	-	+	+	-	-	-	GB11047-PA	AT1G10580.1	23948	BDEG_04795	CE28405	169920	115829	294948	cgd1_1800	-	-	DDB0188449	-	-	117.m00179	ENSGALP00000024253	-	OTTHUMP00000016997	LmjF25.1850	205766	34436	980	187918	NCU06921	Os03g27970.2	1487	GSPATP00010403001	54743	99420	132966	PFL0970w	229948	RO3G_13777.1	YDR364C	SPBC6B1.10	GLEAN3_24485	SINFRUP00000134988	61.m00272	13794	TA13035	57.m01744	26041	95721.m00139	Tb927.3.1930	UM04752.1
		-	+	-	-	-	-	GB10739-PA	AT4G06599.1	-	BDEG_07538	-	159759	115803	283846	-	-	-	DDB0217344	-	-	-	ENSGALP00000005808	-	NP_659486.1	-	117394	-	-	23801	-	Os01g65450.2	14795	-	43439	204566	-	-	830623	-	-	-	-	SINFRUP00000128840	-	24158	-	41.m02948	51063	-	-	UM04219.1
		-	-	-	-	-	-	-	-	-	-	-	-	115786	-	-	-	-	-	-	-	-	-	-	-	-	-	-	-	-	-	-	-	-	-	-	-	-	-	-	-	-	-	-	-	-	-	-	-	-	-	-
		-	-	-	-	-	-	-	AT5G14100.1	-	-	-	-	115782	-	-	-	-	-	-	-	-	-	-	-	-	-	-	-	-	-	Os11g29850.2	-	-	-	149024	-	-	597142	-	-	-	-	-	-	-	-	-	-	-	-	-
		-	+	-	-	-	-	-	AT2G27680.1	-	-	-	-	115779	-	-	-	-	DDB0184494	-	-	-	-	-	-	-	159401	-	-	220742	-	Os09g39390.1	17549	-	42373	113976	-	-	649116	-	-	-	-	-	-	-	-	-	-	-	-	-
	ADP-glucose pyrophosphorylase large subunit	-	-	-	-	-	-	-	-	-	-	-	-	115751	-	-	-	-	-	-	-	-	-	-	-	-	-	-	-	-	-	-	-	-	-	-	-	-	-	-	-	-	-	-	-	-	-	-	-	-	-	-
		-	+	-	-	-	-	GB19495-PA	AT2G40700.1	-	BDEG_00291	-	160521	115746	261079	-	-	OTTDARP00000018141	-	CG8611-PB	-	127.m00131	ENSGALP00000005578	GL50803_16042	OTTHUMP00000022436	-	120211	-	29381	213101	NCU06520	Os05g01990.1	-	-	-	132701	128286	-	826211	RO3G_13531.1	YKR024C	SPBC21H7.04	GLEAN3_16671	SINFRUP00000133643	-	24712	TA05845	-	-	-	-	UM06228.1
		-	+	-	-	-	-	-	AT5G63420.1	-	-	-	-	115745	-	-	-	-	-	-	-	-	-	-	-	-	-	33080	-	-	-	Os02g33610.2	34892	-	-	10739	-	-	773164	-	-	-	-	-	-	-	-	-	62993	-	-	-
		-	+	-	-	-	-	GB14666-PA	-	-	-	CE07985	148455	115716	245470	-	-	-	DDB0185616	CG1104-PA	-	-	ENSGALP00000025077	-	OTTHUMP00000016878	LmjF36.6800	229220	-	72758	184952	-	Os05g02650.1	30296	GSPATP00023533001	-	224354	141157	-	-	-	-	-	-	SINFRUP00000175256	37.m00174	-	TA18735	41.m01359	25797	-	Tb10.6k15.2450	-
		-	+	-	-	-	-	-	AT2G44920.2	23610	-	-	-	115715	-	-	CMO201C	-	DDB0183824	-	-	-	-	-	-	-	-	-	82104	-	-	Os01g05080.1	22391	-	-	126894	-	-	567387	-	-	-	-	-	-	-	-	-	-	-	-	-
GTR9	glycosyl transferase	-	-	-	-	-	-	-	-	-	-	-	-	-	-	-	-	-	-	-	-	-	-	-	-	-	-	-	-	-	-	-	-	-	-	-	-	-	-	-	-	-	-	-	-	-	-	-	-	-	-	-
		+	+	-	-	-	-	GB11035-PA	-	71663	-	-	167439	115671	291593	-	-	OTTDARP00000020259	-	CG4598-PA	-	-	ENSGALP00000012529	-	OTTHUMP00000080405	LmjF31.2250	-	13701	33361	15022	-	-	9443	-	18064	-	134968	-	-	-	-	-	GLEAN3_26928	SINFRUP00000138616	-	268835	-	-	60851	-	Tb927.8.7530	-
		-	+	-	-	-	-	-	AT4G04850.1	70862	BDEG_00265	-	-	115665	-	cgd1_2190	-	-	DDB0184575	-	-	-	-	-	-	-	-	-	-	-	-	Os12g42300.1	36643	-	-	20698	-	-	202307	RO3G_03883.1	-	-	-	-	-	38289	-	-	30483	-	-	-

PRMT3403	Protein arginine N-methyltransferase
FAP160	acid phosphatase
	PfkB-type carbohydrate kinase family protein, ribokinase
ADCY32	adenylate cyclase
ARS1	
GDPD2	glycerophosphoryl diester phosphodiesterase family protein
	SET domain containing protein, putative histone methyltransferase
HFO19	Histone H4
HTA20	Histone H2A
RPN12	26S proteasome regulatory subunit RPN12
LIPG3	gastric triacylglycerol lipase
MSD5	superoxide dismutase [Mn]
CDPKK2b	Calcium/Calmodulin-Dependent Protein Kinase Kinase Homolog 2, isoform beta putative
MDJ1	Mdj1, chaperone associated with the inner membrane, mitochondrial.
HTB20	Histone H2B
POA6	20S proteasome alpha subunit F
COX6B	Mitochondrial cytochrome c oxidase subunit 6b
MCM3	minichromosome maintenance protein 3
CPN60C	Chaperonin 60C
beta-amylase	beta-amylase
ANS1	anthranilate synthase, alpha subunit, putative
SPL1	
STPK15	Serine/Threonine Protein Kinase Homolog 15
ARLD2,ARL6b	ARL6b, ARF-like 6 GTPase
HTR17	Histone H3
EIF2A	eukaryotic translation initiation factor 2 alpha-subunit-like
TEF20	notchless-like WD40 repeat-containing protein, chloroplast location proposed
MFT3	putative MATE efflux family protein
MSD4	superoxide dismutase [Mn]
PDE5	Cyclic nucleotide phosphodiesterase
HFO16	Histone H4
GSK3	Glycogen Synthase Kinase 3
UBC1	Ubiquitin activating enzyme E1
	Uncharacterised ARF-like GTPase domain protein
TOP3	
C_150148	
BOR1	probable borate transporter
KAS-like	
	DNA topoisomerase like-protein
AK2	Adenylate Kinase Homolog 2
PRMT3404	Protein arginine N-methyltransferase
CYP748A1	cytochrome P450, unkown function
LPA1	elongation factor-like protein
ANK22	protein of unknown function with ankyrin repeats
RNPH3	3'-5' Exoribonuclease PH component of the Exosome
PIMT2	
TRM4	tRNA-(m5C) methyltransferase
MTDH/MTCH3	Putative 5,10-methylenetetrahydrofolate dehydrogenase/5,10-methenyltetrahydrofolate cyclohydrolase
CYCC1	C-type cyclin homologue
TOP1	
APG7	Autophagy protein
EFG7	elongation factor-like protein
FAP215	Conserved Uncharacterized Flagellar Associated Protein
FPGS1	Putative folylpolyglutamate synthase
PRP18	putative pre-mRNA splicing factor 18

		-	-	-	-	-	-	-	-	-	-	[illegible]
		-	-	-	-	-	-	-	-	-	-	[illegible]
		-	+	-	-	-	-	-	AT1G16720.1	-	-	[illegible]
		-	-	-	-	-	-	-	-	-	-	[illegible]
		-	+	-	-	-	-	GB15086-PA	-	-	-	[illegible]
		-	+	-	-	-	-	-	-	18992	-	[illegible]
		-	+	-	-	-	-	GB15392-PA	AT1G22800.1	-	BDEG_05878	[illegible]
		-	-	-	-	-	-	-	-	59947	-	[illegible]
	ABC1 family ser/thr kinase	-	+	-	-	-	-	-	AT4G31390.1	-	-	[illegible]
LKHA4	Leukotriene A-4 hydrolase (LTA-4 hydrolase)	-	+	-	-	-	-	-	AT5G13520.1	60785	BDEG_02729	[illegible]
		-	-	-	-	-	-	-	-	-	-	[illegible]
		-	-	-	-	-	-	-	-	-	-	[illegible]
		-	+	-	-	-	-	GB14299-PA	-	-	BDEG_04968	[illegible]
		-	+	-	-	-	-	-	AT5G48020.1	21353	BDEG_06941	[illegible]
		-	-	-	-	-	-	-	AT2G43570.1	-	BDEG_06104	[illegible]
		-	+	-	-	-	-	GB10830-PA	AT1G05350.1	-	-	[illegible]
	soluble starch synthase, ADP-glucose alpha-1,4 glucane alpha-4-glucanotransferase	-	-	-	-	-	-	-	-	-	-	[illegible]
		-	+	-	-	-	-	-	-	-	-	[illegible]
		-	+	+	-	-	-	GB11647-PA	AT2G40730.1	63692	BDEG_07410	[illegible]
		-	+	-	-	-	-	GB13289-PA	AT3G16170.1	-	BDEG_02417	[illegible]
MYO3	myosin heavy chain	-	-	-	-	-	-	-	-	-	-	[illegible]
		-	-	-	-	-	-	-	-	-	-	[illegible]
		-	+	-	-	-	-	GB18418-PA	AT5G55120.1	-	-	[illegible]
PDE16	3',5'-cyclic-nucleotide phosphodiesterase	-	-	-	-	-	-	-	-	-	-	[illegible]
THG1	Histidine tRNA 5'-guanylyltransferase	-	+	-	-	-	-	GB12356-PA	AT2G32330.1	-	BDEG_04230	[illegible]
		-	+	-	-	-	-	-	AT5G19500.1	58694	-	[illegible]
		-	+	-	-	-	-	GB12872-PA	AT1G67490.1	-	BDEG_05261	[illegible]
		-	+	-	-	-	-	-	AT5G64940.2	29234	-	[illegible]
		-	-	-	-	-	-	-	-	-	-	[illegible]
		-	-	-	-	-	-	-	-	-	-	[illegible]
		-	+	-	-	-	-	-	AT2G11810.1	1716	-	[illegible]
		-	+	-	-	-	-	-	-	25715	-	[illegible]
		-	-	-	-	-	-	-	-	-	-	[illegible]
		-	+	-	-	-	-	-	AT5G57850.1	-	-	[illegible]
		-	+	-	-	-	-	-	AT2G20890.1	-	-	[illegible]
SYP1	Qa-SNARE, PM-type	-	+	-	-	-	-	-	AT5G08080.1	13713	-	[illegible]
		-	+	-	-	-	-	-	AT1G45110.1	64242	-	[illegible]
		-	+	-	-	-	-	-	-	-	BDEG_01403	[illegible]
		-	+	+	-	-	-	GB17676-PA	AT1G06720.1	283	BDEG_05248	[illegible]
		-	-	-	-	-	-	-	-	-	-	[illegible]
		-	-	-	-	-	-	-	-	-	-	[illegible]
		-	-	-	-	-	-	-	-	-	-	[illegible]
PAO	Putative Pheophorbide A oxygenase, chloroplast precursor	-	-	-	-	-	-	-	AT3G44880.1	11520	-	[illegible]
		-	-	-	-	-	-	-	-	-	-	[illegible]
		-	-	-	-	-	-	-	-	-	-	[illegible]
		-	+	-	-	-	-	GB14072-PA	AT2G26000.2	62719	BDEG_06153	[illegible]
		-	-	-	-	-	-	-	-	-	-	[illegible]
		-	+	+	-	-	-	GB16233-PA	AT1G72440.1	-	BDEG_03399	[illegible]
		-	+	-	-	-	-	GB12198-PA	-	-	-	[illegible]
		-	+	-	-	-	-	-	AT5G02050.1	-	-	[illegible]
		-	-	-	-	-	-	-	-	-	-	[illegible]
		-	+	-	-	-	-	-	AT1G35220.1	-	-	[illegible]
SRT3402	Histone deacetylase (SIR2 family)	-	+	-	-	-	-	GB17426-PA	AT5G55760.1	4190	-	[illegible]
		-	+	-	-	-	-	GB15096-PA	-	3405	BDEG_08228	[illegible]
		-	-	-	-	-	-	-	-	-	-	[illegible]
		-	-	-	-	-	-	-	-	-	-	[illegible]
		-	-	-	-	-	-	-	-	-	-	[illegible]
		-	+	-	-	-	-	-	-	-	-	[illegible]
ANK5	protein of unknown function with ankyrin repeats	-	-	-	-	-	-	-	-	-	-	[illegible]
		-	+	-	-	-	-	GB19428-PA	AT1G35190.1	61245	BDEG_08257	[illegible]
		-	+	-	-	-	-	-	AT4G33950.1	-	-	[illegible]
		-	-	-	-	-	-	-	-	-	-	[illegible]
		-	-	-	-	-	-	-	-	-	-	[illegible]
CNX1B	molybdenum cofactor biosynthesis protein	-	+	-	-	-	-	GB11831-PA	AT5G20990.1	19802	BDEG_02560	[illegible]
		-	-	-	-	-	-	-	-	-	-	[illegible]
		-	-	-	-	-	-	-	-	-	-	[illegible]
MCM4	minichromosome maintenance protein 4	-	+	+	-	-	-	GB20008-PA	AT2G16440.1	33815	BDEG_07032	[illegible]
		-	+	-	-	-	-	GB15151-PA	AT1G79890.1	-	BDEG_04964	[illegible]
		-	-	-	-	-	-	-	-	-	-	[illegible]
PUS8	Pseudouridine synthase, mitochondrial precursor, putative	-	-	-	-	-	-	-	-	-	-	[illegible]
		-	+	+	-	-	-	GB17825-PA	AT1G29940.1	55255	BDEG_04902	[illegible]
		-	+	-	-	-	-	-	-	71110	-	[illegible]
		-	+	-	-	-	-	GB18589-PA	AT1G49340.1	-	BDEG_06202	[illegible]
		-	-	-	-	-	-	-	-	-	-	[illegible]
		-	-	-	-	-	-	-	-	-	-	[illegible]
		-	-	-	-	-	-	-	AT5G46090.1	-	-	[illegible]
		-	-	-	-	-	-	-	-	-	-	[illegible]
		-	+	-	-	-	-	-	AT3G16857.2	-	-	[illegible]
		-	-	-	-	-	-	-	-	-	-	[illegible]
		-	+	-	-	-	-	GB12788-PA	AT4G38600.1	-	-	[illegible]
		-	+	-	-	-	-	-	-	-	-	[illegible]
		-	-	-	-	-	-	-	-	-	-	[illegible]
		-	-	-	-	-	-	-	-	-	-	[illegible]
		-	+	-	-	-	-	-	AT2G46890.1	69760	-	[illegible]
		-	+	-	-	-	-	GB17733-PA	AT4G19810.1	-	BDEG_06156	[illegible]
		-	-	-	-	-	-	-	-	-	-	[illegible]
		-	-	-	-	-	-	-	-	-	-	[illegible]
		-	+	+	-	-	-	GB19311-PA	AT1G27970.1	18389	BDEG_07790	[illegible]
		-	+	+	-	-	-	GB14226-PA	AT3G18600.1	10453	BDEG_00386	[illegible]
		-	+	-	-	-	-	-	-	38878	-	[illegible]
VPS24	subunit of the ESCRT-III complex	-	+	-	-	-	-	GB15107-PA	AT5G22950.1	7138	BDEG_02992	[illegible]
NRAMP2	natural resistance-associated macrophage protein	-	+	-	-	-	-	GB15139-PA	-	-	BDEG_00070	[illegible]
		-	-	-	-	-	-	-	-	-	-	[illegible]
KDG3	diacylglycerol kinase	-	+	-	-	-	-	-	AT5G57690.1	22252	-	[illegible]
DP1	DP transcription factor homologue	-	+	-	-	-	-	-	AT5G03415.1	23476	-	[illegible]
COQ5A	ubiquinone/menaquinone biosynthesis methyltransferase COQ5	-	+	-	-	-	-	GB18574-PA	AT5G57300.2	59050	BDEG_06534	[illegible]
		-	-	-	-	-	-	-	-	-	-	[illegible]
		-	+	-	-	-	-	GB17051-PC	AT1G66510.2	-	-	[illegible]
		-	-	-	-	-	-	-	-	-	-	[illegible]
POLA4		-	+	-	-	-	-	-	-	-	-	[illegible]
		-	-	-	-	-	-	-	-	-	-	[illegible]
		+	+	-	-	-	-	GB18745-PA	-	-	-	[illegible]
R1protein	R1 Protein, alpha-glucan water dikinase	-	+	-	-	-	-	-	AT1G10760.1	-	-	[illegible]
		-	+	+	-	-	-	GB15154-PA	AT2G23820.2	-	BDEG_07691	[illegible]
EZY2	protein of unknown function	-	-	-	-	-	-	-	-	-	-	[illegible]
	Granule Bound starch synthase	-	-	-	-	-	-	-	-	-	-	[illegible]
EZY1	chloroplast nucleoid localized protein, zygote-specific	-	-	-	-	-	-	-	-	-	-	[illegible]
		-	+	-	-	-	-	-	AT2G20830.1	4928	-	[illegible]
EF-Sec		-	+	-	-	-	-	-	-	1204	-	[illegible]
HFO6	Histone H4	-	-	-	-	-	-	-	-	-	-	[illegible]
HCF136		-	+	-	-	-	-	-	AT5G23120.1	29089	-	[illegible]
		-	+	+	-	-	-	GB14903-PA	AT2G01280.1	71825	BDEG_05265	[illegible]
		-	-	-	-	-	-	-	-	-	-	[illegible]
HTB10	Histone H2B	-	-	-	-	-	-	-	-	-	-	[illegible]
AMPK1	putative AMP-activated protein kinase catalytic subunit homolog 1	-	-	-	-	-	-	-	-	-	-	[illegible]
HTA10	Histone H2A	-	-	-	-	-	-	-	-	-	-	[illegible]
PYC	pyruvate carboxylase	-	+	-	-	-	-	GB19885-PA	-	65502	BDEG_03750	[illegible]
		-	-	-	-	-	-	-	-	69248	-	[illegible]
		-	-	-	-	-	-	-	-	-	-	[illegible]
HTB13	Histone H2B	-	-	-	-	-	-	-	-	-	-	[illegible]
		-	-	-	-	-	-	-	-	-	-	[illegible]
NIC7	unknown protein; quinolinate synthetase A-related	-	+	-	-	-	-	-	AT5G50210.1	-	-	[illegible]
HTR33	Histone H3	-	-	-	-	-	-	-	-	-	-	[illegible]
		-	+	-	-	-	-	-	AT2G46830.1	-	-	[illegible]
IF3H	eukaryotic translation initiation factor 3	-	+	-	-	-	-	GB10272-PA	AT3G11400.1	-	BDEG_04900	[illegible]
PABPN1	putative PABPNI. poly(A) binding protein 2. similar to human accession NP_004634	-	+	+	-	-	-	GB13003-PA	AT5G51120.1	23087	BDEG_07650	[illegible]
		-	-	-	-	-	-	-	-	-	-	[illegible]
		-	+	-	-	-	-	GB11980-PA	AT2G43770.1	22631	BDEG_03578	[illegible]
HFO9	Histone H4	-	-	-	-	-	-	-	-	-	-	[illegible]
HTR7	Histone H3	-	-	-	-	-	-	-	-	-	-	[illegible]
beta-amylase	beta-amylase	-	-	-	-	-	-	-	-	-	-	[illegible]
HFO12	Histone H4	-	-	-	-	-	-	-	-	-	-	[illegible]
HTB11	Histone H2B	-	-	-	-	-	-	-	-	-	-	[illegible]
HTA13	Histone H2A	-	-	-	-	-	-	-	-	-	-	[illegible]
		-	+	-	-	-	-	GB14405-PA	AT2G17570.1	-	BDEG_02194	[illegible]
ARP3401	Actin related protein	-	+	-	-	-	-	GB14337-PA	AT1G18450.1	-	BDEG_05750	[illegible]
		-	-	-	-	-	-	-	-	-	-	[illegible]
		-	-	-	-	-	-	-	-	-	-	[illegible]
		-	+	-	-	-	-	-	AT5G03880.1	28134	-	[illegible]
PP2A-1r	Protein Phosphatase 2A Regulatory Subunit	-	+	-	-	-	-	GB15363-PA	AT5G44090.1	3205	BDEG_00441	[illegible]
		-	+	+	-	-	-	GB12568-PA	AT3G56510.2	61077	BDEG_06491	[illegible]
ADCY16	adenylate cyclase	-	-	-	-	-	-	-	-	-	-	[illegible]
DMT3403	Dnmt1 class (cytosine-5-)DNA methyltransferase	-	-	-	-	-	-	-	-	-	-	[illegible]
HTR34	Histone H3	-	-	-	-	-	-	-	-	-	-	[illegible]
		-	+	-	-	-	-	GB17290-PA	AT1G19140.1	-	BDEG_01460	[illegible]
		-	+	+	-	-	-	GB12626-PA	AT3G15460.1	4706	BDEG_02751	[illegible]
POC1	centriole proteome protein	+	+	-	+	-	-	GB15587-PA	-	27325	BDEG_01586	[illegible]
		-	-	-	-	-	-	-	-	-	-	[illegible]
		-	-	-	-	-	-	-	AT2G02820.2	-	-	[illegible]
		-	-	-	-	-	-	-	-	-	-	[illegible]
		-	-	-	-	-	-	-	-	-	-	[illegible]
TRM2B	tRNA (uracil-5-)-methyltransferase	-	+	-	-	-	-	GB14708-PA	AT2G28450.1	64214	BDEG_01510	[illegible]
		-	+	+	-	-	-	GB19522-PA	AT3G46100.1	30004	BDEG_05049	[illegible]
CYN18-1	Peptidyl-prolyl cis-trans isomerase, cyclophilin-type	-	+	-	-	-	-	GB18452-PA	AT1G01940.1	35677	BDEG_05126	[illegible]
		-	-	-	-	-	-	-	-	-	-	[illegible]
CDC14	Cell Division Cycle 14 Homolog	-	+	+	-	-	-	GB10224-PA	-	19103	BDEG_03666	[illegible]

PDE22	3',5'-cyclic-nucleotide phosphodiesterase	-	-	-	-	-	-	-	112177	-	-	-
		-	-	AT4G01650.2	16631	-	-	-	112157	-	-	CMA013C
		-	GB11413-PA	AT3G12600.1	-	-	CE29935	149402	112153	-	-	CMM026C
		-	-	-	-	-	-	-	112129	-	-	-
		-	-	-	-	-	-	-	112116	-	-	-
PDE23	3' 5' cyclic phosphodiesterase	-	-	-	-	-	-	-	112108	-	-	-
	Serine/threonine protein kinase	-	-	-	-	-	-	-	112095	-	-	-
ADCY15	adenylate cyclase	-	-	-	-	-	-	-	112056	-	-	-
MEN2	Putative naphthoate synthase.	-	-	AT1G60550.1	-	-	-	102781	112040	-	-	CMV113C
		-	-	-	-	-	-	-	112030	-	-	-
		-	-	-	-	-	-	-	112011	-	-	-
		-	GB11545-PA	AT4G24710.1	-	BDEG_00686	CE01547	163622	111999	293013	cgd6_550	-
		-	-	AT1G67600.1	-	-	-	-	111993	-	-	-
ADCY13	adenylate cyclase	-	-	-	-	-	-	-	111992	-	-	-
		-	-	AT3G51670.1	-	-	-	-	111979	-	-	-
		-	GB18600-PA	AT4G00090.1	-	-	CE10584	149780	111975	-	-	-
		-	-	AT1G21370.2	13322	-	-	-	111971	-	-	CMK175C
		-	-	AT4G13400.1	27564	BDEG_07012	-	-	111970	-	-	-
		-	-	AT1G13640.1	53540	-	CE40500	-	111968	-	cgd2_1340	CMM224C
		-	-	-	-	-	-	-	111945	-	-	-
		-	-	-	70776	-	-	95444	111943	379488	-	-
		-	-	-	-	-	-	-	111942	-	-	-
		-	-	AT3G55960.1	-	-	-	-	111940	-	-	-
		-	GB17750-PA	AT2G23940.1	-	BDEG_02405	CE27195	-	111935	227666	-	-
		-	GB15327-PA	-	22001	BDEG_07993	CE28131	214836	111927	298715	cgd5_3070	-
FAP133	Flagellar Associated Protein	-	-	-	54930	-	-	-	111920	-	-	-
		-	-	-	-	-	-	-	111903	-	-	-
POLK1	DNA polymerase kappa	-	-	AT1G49980.1	30647	-	CE00159	151315	111898	-	-	-
		-	-	-	-	-	-	-	-	-	-	-
		-	GB14886-PA	AT3G20630.1	23104	BDEG_04325	CE30696	182887	111890	286184	cgd6_4550	CMT098C
		-	GB12432-PA	AT4G36080.1	-	BDEG_05729	CE38054	225574	111873	-	-	-
		-	-	AT5G59570.1	-	-	-	-	111844	-	-	-
HSK1	possible homoserine kinase	-	-	AT2G17265.1	60300	-	-	-	111837	-	-	-
		-	-	-	27956	-	-	-	111806	-	-	-
		-	-	-	-	-	-	-	111796	-	-	-
		-	GB15936-PA	AT3G04500.1	7855	BDEG_08260	CE24459	170084	111793	287314	cgd1_1070	-
CBF1		-	GB19404-PA	AT4G14540.1	55371	BDEG_04026	CE14792	159684	111791	392696	cgd4_1720	CMR350C
		-	GB11559-PA	AT2G01730.1	-	BDEG_08136	CE32859	151615	111783	206303	cgd5_1170	-
		-	-	AT4G27990.1	-	-	-	-	111779	-	-	CMV083C
		-	GB10527-PA	AT1G78570.1	23143	BDEG_01096	CE04644	223439	111778	-	-	CMQ006C
		-	-	-	-	-	-	-	111746	-	-	-
		-	-	AT3G06200.1	-	-	-	-	111725	-	-	-
FUO1	Putative electron transfer flavoprotein-ubiquinone oxidoreductase.	-	GB19888-PA	AT2G43400.1	71690	BDEG_08255	CE29662	177493	111715	293823	-	CMT089C
		-	GB12688-PA	AT1G10390.2	-	BDEG_03601	CE34448	222010	111714	230079	cgd3_2360	CMB112C
		-	-	-	-	-	-	-	111701	-	-	-
		-	GB18025-PA	AT3G02710.1	7421	BDEG_01408	CE12290	149947	111690	246797	cgd5_3800	-
		-	-	-	-	-	-	-	111637	-	-	-
	similar to U2 snRNP auxiliary factor, large subunit	-	GB12876-PA	-	8852	-	CE30403	155651	111628	295877	cgd2_1480	-
		-	GB18144-PA	AT1G07360.1	-	BDEG_05482	CE33830	123077	111627	252331	cgd7_5320	-
		-	GB13534-PA	AT5G15700.1	20280	BDEG_06055	CE34220	156688	111616	272974	-	CMJ257C
CPH-like-1		-	-	AT5G24850.1	1648	-	-	-	111597	-	-	CMJ130C
		-	GB10206-PA	AT1G16560.3	-	BDEG_03362	-	219496	111593	214344	-	-
		-	-	-	54327	BDEG_04326	-	-	111581	-	-	CMA046C
		-	-	-	-	-	-	110471	111578	379116	-	-
		-	GB15051-PA	AT2G29900.1	-	BDEG_02383	CE24946	159308	111567	246693	-	-
COX15	Putative cytochrome c oxidase assembly factor COX15.	-	GB20115-PA	AT5G56090.1	2874	BDEG_08667	CE02326	180739	111563	202150	-	-
		-	-	-	9884	-	-	181697	111555	-	-	-
		-	-	AT5G06210.1	-	-	-	-	111544	381470	-	-
		-	-	-	-	-	CE30570	170160	111541	264320	cgd7_2570	-
		-	GB11747-PA	AT2G45695.1	-	BDEG_06707	-	203305	111518	218486	cgd7_3310	CMJ177C
		-	-	AT5G43260.1	-	-	-	-	111509	-	-	-
		-	GB17066-PA	AT2G40090.1	1717	BDEG_08057	CE09076	172331	111503	-	-	CMS164C
		-	-	-	-	-	-	-	111483	-	-	-
		-	GB18326-PA	AT4G38890.1	5440	BDEG_02437	-	165435	111482	212631	cgd5_2440	-
		-	-	-	-	-	-	-	111463	-	-	-
		-	-	AT4G01995.1	64975	-	-	-	111461	-	-	-
		-	-	AT2G04842.1	55515	-	-	-	111453	-	-	CMO098C
		-	-	AT5G47435.1	70450	-	-	-	111421	-	-	-
		-	-	-	-	-	-	-	111413	-	-	-
		-	GB10215-PA	AT5G22110.1	72694	BDEG_02644	CE29749	182971	111410	272197	-	-
		-	-	AT4G33910.1	-	-	-	-	111407	-	-	-
		-	-	AT3G52155.1	-	-	-	-	111403	-	-	-
		-	GB19433-PA	AT4G05590.1	54427	BDEG_01975	CE28447	174885	111398	207144	-	CMB099C
		-	-	-	-	-	-	-	111376	-	-	-
	PfkB-type carbohydrate kinase family protein, ribokinase	-	GB14732-PA	AT1G17160.1	28224	-	CE03147	20019	111372	299057	-	-
		-	GB10234-PA	AT5G05080.2	5740	BDEG_02986	-	152679	111359	238987	-	CMS304C
TOM40	Translocase of outer membrane TOM40, mitochondrial	-	GB18251-PA	AT3G20000.1	69596	-	CE05298	174847	111351	288611	cgd1_2560	CMF062C
		-	-	-	-	-	-	-	111345	-	-	-
		-	-	AT5G26880.1	-	-	-	-	111338	-	-	CMR425C
MTHFR	Putative 5,10-methylenetetrahydrofolate reductase	-	-	-	-	-	-	-	-	-	-	-
RPB373aa		-	GB16593-PA	AT2G15430.1	22116	BDEG_06967	CE39091	155833	111305	205097	cgd1_2710	CMP043C
		-	-	-	-	-	-	-	111301	-	-	-
		-	GB17060-PA	AT1G24050.1	-	BDEG_00676	-	176694	111290	-	cgd4_2290	CMN146C
		-	-	-	-	-	-	-	111278	-	-	-
CHR3414		-	GB16633-PA	AT3G19210.1	71986	BDEG_05747	CE25143	-	111277	250186	cgd1_1180	-
		-	GB14718-PA	AT2G40360.1	59370	BDEG_07630	CE22116	130363	111274	289886	cgd2_3680	CMP117C
		-	GB19873-PA	AT3G58510.3	59462	BDEG_06252	CE38657	224610	111269	260795	cgd4_3000	CMT173C
CDPKK1a	Calcium/Calmodulin-Dependent Protein Kinase Kinase Homolog 1isoform alpha	-	-	-	-	-	-	-	111266	-	-	-
		-	-	-	-	-	-	-	111258	-	-	-
		-	-	-	-	-	-	-	111255	-	-	-
		-	-	-	-	-	-	-	111185	-	-	-
		-	-	-	-	-	-	-	-	-	-	-
		-	-	-	-	-	-	-	111160	-	-	-
		-	-	-	-	-	-	-	111122	-	-	-
		-	-	-	-	-	-	-	111092	-	-	-
		-	-	-	-	-	-	-	111086	-	-	-
		-	-	-	-	-	-	-	111068	-	-	-
		-	-	-	-	-	-	-	111066	-	-	-
		-	-	-	-	-	-	-	111044	-	-	-
		-	-	-	-	-	-	-	111014	-	-	-
		-	-	-	-	-	-	-	111008	-	-	-
		-	-	-	-	-	-	-	111002	-	-	-
		-	-	-	-	-	-	-	110992	-	-	-
		-	-	-	-	-	-	-	110991	-	-	-
HFO47	Histone H4	-	-	-	-	-	-	-	-	-	-	-
		-	-	-	-	-	-	-	110956	-	-	-
		-	-	-	-	-	-	-	110955	-	-	-
		-	GB14322-PA	-	-	-	-	-	110819	-	-	-
HFO31	Histone H4	-	-	-	-	-	-	-	-	-	-	-
HTA31	Histone H2A	-	-	-	-	-	-	-	-	-	-	-
HFO46	Histone H4	-	-	-	-	-	-	-	-	-	-	-
HTR46	Histone H3	-	-	-	-	-	-	-	-	-	-	-
		-	-	-	-	-	-	-	110360	-	-	-
HFO30	Histone H4	-	-	-	-	-	-	-	-	-	-	-
		-	-	-	-	-	-	-	110256	-	-	-
		-	-	-	64979	-	-	-	110216	-	-	-
		-	-	-	-	-	-	-	-	-	-	-
		-	-	-	-	-	-	-	110032	-	-	-
		-	-	-	-	-	-	-	110007	-	-	-
		-	-	-	-	-	-	-	109976	-	-	-
		-	GB14339-PA	AT5G26667.3	-	-	-	176353	109953	-	-	-
HTB44	Histone H2B	-	-	-	-	-	-	-	-	-	-	-
		-	-	-	-	-	-	-	109828	-	-	-
		-	-	-	-	-	-	-	109818	-	-	-
		-	-	-	17538	-	-	60885	109759	396708	-	-
		-	-	-	-	-	-	-	109602	-	-	-
		-	-	-	-	-	-	-	109545	-	-	-
		-	-	-	-	-	-	-	109540	-	-	-
		-	-	-	-	-	-	-	109509	-	-	-
HTA26	Histone H2A	-	-	-	-	-	-	-	-	-	-	-
HTB26	Histone H2B	-	-	-	-	-	-	-	-	-	-	-
		-	-	-	-	-	-	-	109432	-	-	-
		-	-	-	-	-	-	-	-	-	-	-
		-	-	AT4G31770.1	70538	BDEG_08102	-	-	109425	262089	cgd3_4330	CMK205C
HTR25	Histone H3	-	-	-	-	-	-	-	-	-	-	-
HTA25	Histone H2A	-	-	-	-	-	-	-	-	-	-	-
		-	-	-	-	-	-	-	-	-	-	-
		-	-	-	-	-	-	-	-	-	-	-
		-	-	AT4G10100.3	-	BDEG_08111	CE02019	120233	109356	-	-	-
		-	-	-	-	-	-	-	109352	-	-	-
		-	-	-	-	-	-	-	109351	-	-	-
		-	-	-	-	-	-	-	109269	-	-	-
		-	-	-	-	-	-	-	109252	-	-	-
		-	-	-	-	-	-	-	109250	-	-	-
		-	-	-	-	-	-	-	109243	-	-	-
		-	-	-	-	-	CE15680	228220	109222	285533	cgd7_1670	-
	speract/scavenger receptor cystein-rich (SRCR) family protein, transmembrane glycoprotein	-	-	-	-	-	-	-	109212	-	-	-
		-	GB13508-PA	-	-	BDEG_07697	CE17071	224546	109194	213831	-	CMN203C
		-	-	-	-	-	-	-	109119	-	-	-
		-	-	-	-	-	-	-	109029	-	-	-
		-	-	-	-	-	-	-	109028	-	-	-
		-	-	-	-	-	-	-	108984	-	-	-
		-	-	-	-	-	-	-	108983	-	-	-
FAP32	Conserved Uncharacterized Flagellar Associated Protein	-	GB17268-PA	-	31144	BDEG_08408	CE40390	181738	108954	380898	-	-
PRDX3	peroxiredoxin	-	-	-	-	-	-	-	108953	-	cgd4_740	-
		-	-	-	-	-	-	-	108940	-	-	-

HTB24	Histone H2B	[illegible]
HDA3414	Histone deacetylase	[illegible]
BIOA	Diaminopelargonic acid synthase	[illegible]
CYP747A1	cytochrome P450, unkown function	[illegible]
UNG1	uracil DNA-glycosylase (UDG)	[illegible]
	putative Peptidyl-tRNA hydrolase 2/Bcl-2 inhibitor of transcription 1	[illegible]
MAPK4	Hypothetical Mitogen-Activated Protein Kinase Homolog 4	[illegible]
GTR16	glycosyl transferase	[illegible]
LIPG1	putative carboxylic ester hydrolase/ lipase	[illegible]
IF2Bd,EIF2Bd		[illegible]
MRPL17	Putative mitochondrial ribosomal protein L17	[illegible]
CYCU1	U-type cyclin	[illegible]
TEF28	Unknown function, mitochondrial localization assumed	[illegible]
C_660080		[illegible]
PDE9	3' 5' cyclic phosphodiesterase	[illegible]
SRP19	Subunit of the Signal Recognition Particle	[illegible]
MFT10	related to major facilitator superfamily	[illegible]
HAG3401	Histone acetyltransferase	[illegible]
CLPB2		[illegible]
HTB42	Histone H2B	[illegible]
MGE1	Mge1, chaperone that assists matrix Hsp70, mitochondrial.' '	[illegible]
RPT1	Raptor, KOG1, Mip1p; TOR binding partner	[illegible]
ANK4	protein of unknown function with ankyrin repeats	[illegible]
PYK4	pyruvate kinase	[illegible]
CPN4,CCT4		[illegible]
	putative ATP-dependent RNA helicase	[illegible]
MNP2	ATP-binding protein, MRP/NBP35 family, N-terminal iron-sulfur binding sequence	[illegible]
BLP	bystin-like family protein	[illegible]
HTA5	Histone H2A	[illegible]
NUDC		[illegible]
HTB1	Histone H2B	[illegible]
EFP1	organellar elongation factor P	[illegible]
PUS6	Pseudouridine synthase, putative	[illegible]
FAP266	Conserved Uncharacterized Flagellar Associated Protein	[illegible]
PDE21	3',5'-cyclic-nucleotide phosphodiesterase	[illegible]
MITC5	putative mitochondrial substrate carrier	[illegible]

EFG8	elongation factor-like protein	-	+	-	-	-	-	-	AT2G31060.2	-	BDEG_01798	-	-	106481	-
		-	-	-	-	-	-	-	-	-	-	-	-	106469	-
		-	-	-	-	-	-	-	-	-	-	-	-	106461	-
FAP250	Flagellar Associated Protein	+	+	-	+	-	-	GB12512-PA	-	23262	BDEG_02772	-	167685	106450	216480
		-	-	-	-	-	-	-	-	-	-	-	-	106436	-
CNX2	hypothetical molybdopterin biosynthesis protein, MoaA family	-	+	-	-	-	-	GB11214-PA	AT2G31955.2	37684	BDEG_06408	CE27759	180583	106419	401154
		-	-	-	-	-	-	-	-	-	-	-	-	106402	-
		-	+	+	-	-	-	GB14360-PA	AT2G21270.3	60034	BDEG_06980	CE23674	175414	106366	372848
FKB53	peptidyl-prolyl cis-trans isomerase, FKBP-type	-	+	-	-	-	-	GB16520-PA	AT4G25340.1	-	BDEG_02133	-	-	106360	-
		-	+	-	-	-	-	-	-	18592	-	-	148050	106339	-
SYI	isoleucyl-tRNA synthetase	-	+	-	-	-	-	GB13728-PA	AT5G49030.1	10097	-	CE35957	214074	106327	280519
		-	-	-	-	-	-	-	-	-	-	-	-	106306	-
		-	-	-	-	-	-	-	-	-	-	-	-	106213	-
		-	-	-	-	-	-	-	-	-	-	-	-	106211	-
		-	+	-	-	-	-	GB19321-PA	AT4G26430.1	-	BDEG_04584	-	163068	106208	293268
		-	-	-	-	-	-	-	-	-	-	-	-	106207	-
		-	+	-	-	-	-	GB15108-PA	AT3G01090.2	-	BDEG_07083	CE07458	227168	106184	212676
		-	+	-	-	-	-	GB12402-PA	AT1G08110.4	35548	-	-	169681	106176	386156
		-	+	-	-	-	-	-	AT5G24300.1	-	-	-	-	106158	-
		-	-	-	-	-	-	-	-	-	-	-	-	106109	-
		-	-	-	-	-	-	-	-	-	-	-	-	106083	-
	Hypothetical zinc-dependent DNA-binding protein	-	+	-	-	-	-	-	AT1G21000.1	-	-	-	-	106059	-
HFO39	Histone H4	-	-	-	-	-	-	-	-	-	-	-	-	-	-
ADCY34	adenylate cyclase	-	-	-	-	-	-	GB16030-PA	-	-	-	-	-	106046	-
		-	+	-	-	-	-	-	AT2G21620.1	-	BDEG_02300	-	-	105992	-
		-	-	-	-	-	-	-	-	-	-	-	-	105965	-
		-	-	-	-	-	-	-	-	-	-	-	-	105941	-
		-	+	-	-	-	-	GB11301-PA	AT4G35780.1	-	-	CE31572	223355	105918	262769
		-	+	-	-	-	-	-	AT2G44040.1	25872	-	-	115186	105908	-
		-	-	-	-	-	-	-	AT5G51840.1	-	-	-	-	105899	-
		-	-	-	-	-	-	-	-	-	-	-	-	105896	-
		-	+	-	-	-	-	-	AT4G13590.1	-	-	-	-	105873	-
ANK3	protein of unknown function with ankyrin repeats	-	-	-	-	-	-	-	-	-	-	-	-	105860	-
		-	-	-	-	-	-	-	-	-	-	-	-	105845	-
		-	-	-	-	-	-	-	-	-	-	-	-	105836	-
		-	-	-	-	-	-	-	-	-	-	-	-	105835	-
		-	+	-	-	-	-	-	AT3G61320.1	-	-	-	-	105828	-
		-	+	-	-	-	-	-	AT4G36960.1	6078	BDEG_02604	-	175992	105806	-
VAMP75	VAMP75 R-SNARE	-	-	-	-	-	-	-	-	-	-	-	-	105787	-
		-	+	-	-	-	-	-	AT2G45990.2	-	-	-	-	105761	-
RACK1	Receptor of activated protein kinase C 1, component of 40S small ribosomal subunit	-	+	+	-	-	-	GB11076-PA	AT3G18130.1	37768	BDEG_04723	CE06090	227676	105734	201339
		-	+	-	-	-	-	-	AT5G65930.2	14287	-	-	167295	105722	373630
		-	+	+	-	-	-	GB12981-PA	AT3G53030.1	1293	BDEG_06479	CE36373	173738	105706	205223
		-	+	+	-	-	-	GB17360-PA	AT3G08505.1	-	BDEG_03510	CE25488	174661	105687	206244
		-	-	-	-	-	-	-	-	-	-	-	-	105686	-
RABC2	RABC2 Rab GTPase	-	-	-	-	-	-	-	-	-	-	-	-	105683	-
		-	-	-	-	-	-	-	-	-	-	-	-	105679	-
		-	+	-	-	-	-	GB10684-PA	AT2G20110.1	-	-	CE17990	-	105644	264943
ATPG		-	+	-	-	-	-	GB11218-PA	AT4G32260.1	-	-	-	-	105641	-
CHLH2	Putative CHLH Magnesium chelatase subunit H	-	-	-	-	-	-	-	-	-	-	-	-	105632	-
FBB9		+	+	-	+	-	-	GB15430-PA	-	23309	BDEG_07243	-	147290	105624	298701
		-	+	-	-	-	-	-	AT3G50880.1	61681	-	-	-	105620	-
		-	-	-	-	-	-	-	-	-	-	-	-	105568	-
SPC18	Signal Peptidase, catalytic subunit	-	+	+	-	-	-	GB16282-PA	AT1G52600.1	27033	BDEG_01225	CE22466	152214	105562	-
		-	-	-	-	-	-	-	-	-	-	-	-	105539	-
		-	+	-	-	-	-	-	AT5G52520.1	18779	-	-	-	105530	-
		-	-	-	-	-	-	-	-	-	-	-	-	105488	-
FTSY	Chloroplast SRP Receptor	-	+	-	-	-	-	-	AT2G45770.1	32555	-	-	-	105469	-
		-	+	-	-	-	-	-	AT4G15520.1	6321	-	-	-	105431	-
		-	+	-	-	-	-	-	-	-	-	-	-	105419	-
		-	+	+	-	-	-	GB12712-PA	AT4G25730.1	52052	BDEG_00046	CE38201	103272	105410	-
		-	+	-	-	-	-	-	AT3G21140.1	-	-	-	-	105401	-
		-	-	-	-	-	-	-	-	-	-	-	-	105391	-
TEF9	Unknown function, chloroplast location proposed	-	+	-	-	-	-	-	AT3G61870.1	-	-	-	-	105340	-
TBY1	Tubby protein	-	+	-	-	-	-	GB19892-PA	AT2G18280.2	20811	BDEG_07594	CE29091	19568	105335	237654
GTR7	glycosyl transferase	-	-	-	-	-	-	-	AT5G20280.1	-	-	-	-	105328	-
		-	+	-	-	-	-	-	AT3G26670.3	-	-	-	-	105319	-
		-	+	-	-	-	-	-	-	12165	BDEG_04182	-	150138	105305	299037
		-	+	-	-	-	-	GB20019-PA	AT4G10050.1	-	BDEG_05247	CE26705	173271	105291	262546
		-	+	+	-	-	-	GB14121-PA	AT5G23290.1	28705	-	CE00827	18513	105267	-
		-	+	-	-	-	-	-	AT4G13250.1	24309	-	-	-	105237	-
		-	+	-	-	-	-	-	-	64442	-	-	113940	105231	-
CAX1	CAX family of Cation antiporters, membrane protein	-	+	-	-	-	-	-	AT1G55730.2	6661	BDEG_06016	-	-	105167	-
		-	-	-	-	-	-	-	-	-	-	-	-	105128	-
		-	+	-	-	-	-	-	AT3G02875.1	-	-	-	-	105119	-
		-	+	-	-	-	-	-	AT1G21920.1	-	-	-	-	105117	-
ATM2	half-size ABC transporter, membrane protein	-	+	-	-	-	-	GB11034-PA	AT5G58270.1	29015	BDEG_02240	CE37072	149358	105113	-
		-	-	-	-	-	-	-	-	-	-	-	-	105104	-
		-	+	-	-	-	-	-	-	3319	BDEG_04684	-	-	105088	-
		-	+	-	-	-	-	GB18192-PA	AT5G56360.1	-	BDEG_07470	CE15547	173651	105079	291203
		-	+	-	-	-	-	-	-	59332	BDEG_05942	-	-	105035	-
		-	+	-	-	-	-	-	-	64756	-	-	-	104999	277969
	ABC1 family ser/thr kinase	-	-	-	-	-	-	-	-	-	-	-	-	104934	-
		-	-	-	-	-	-	-	-	-	-	-	-	104900	-
		-	+	-	-	-	-	GB19054-PA	-	-	BDEG_05762	CE00419	38304	104871	294875
HDA3402	Histone deacetylase	-	+	-	-	-	-	GB10010-PA	AT3G18520.2	3079	BDEG_07924	CE37192	126869	104855	262330
		-	+	-	-	-	-	-	AT3G07700.2	-	BDEG_06944	-	-	104846	-
LhcSR3		-	-	-	-	-	-	-	-	-	-	-	-	-	-
		-	+	-	-	-	-	-	AT3G04850.1	60657	-	-	229042	104804	-
		-	+	-	-	-	-	-	AT3G60910.1	5839	BDEG_02744	CE38274	-	104798	-
		-	-	-	-	-	-	-	-	-	-	-	-	104788	-
		-	+	-	-	-	-	-	AT3G21215.1	-	BDEG_06836	CE08260	112394	104778	295508
		-	+	-	-	-	-	-	AT5G47650.1	66734	-	-	219640	104774	-
		-	-	-	-	-	-	-	-	-	-	-	-	104732	-
PSBP4		-	+	-	-	-	-	-	AT4G15510.3	-	-	-	-	104731	-
LhcSR3*		-	-	-	-	-	-	-	-	-	-	-	-	104720	-
		-	+	-	-	-	-	-	-	-	-	-	46118	104719	-
	cation transporting ATPase	-	+	-	-	-	-	-	-	-	BDEG_00733	-	-	104709	-
		-	+	-	-	+	-	GB10754-PA	AT1G50240.2	5353	-	-	150354	104702	-
		-	+	+	-	-	-	GB13175-PA	AT3G05350.1	824	BDEG_01177	CE14560	169985	104657	261881
		-	+	-	-	-	-	-	AT2G18950.1	67156	-	-	-	104647	-
GTR20	glycosyl transferase	-	+	-	-	-	-	GB14100-PA	AT2G44660.1	27313	BDEG_07699	CE36914	4725	104640	293849
		-	+	-	-	-	-	GB19076-PA	-	-	BDEG_01434	CE25503	105424	104611	391194
		-	+	-	-	-	-	-	AT3G27340.1	-	-	-	-	104582	-
		-	+	-	-	-	-	GB12093-PA	-	14167	-	-	-	104547	-
		-	+	-	-	-	-	-	AT5G54160.1	-	-	-	173689	104544	-
		-	-	-	-	-	-	-	-	-	-	-	-	104519	-
		-	+	-	-	-	-	-	AT2G33840.1	26371	-	CE33722	-	104512	-
PYK3	pyruvate kinase	-	-	-	-	-	-	-	-	-	-	-	-	104490	-
		-	-	-	-	-	-	-	-	-	-	-	-	104450	-
		-	+	-	-	-	-	GB19069-PA	AT5G58020.1	-	BDEG_08482	CE07787	157491	104448	274506
		-	-	-	-	-	-	-	-	69511	-	-	-	104441	-
		-	+	-	-	-	-	-	AT2G43180.2	13413	-	-	-	104431	-
		-	-	-	-	-	-	-	-	-	-	-	-	104389	-
		-	+	-	-	-	-	GB19211-PA	AT3G16060.1	2307	BDEG_00712	CE29333	167419	104388	250400
		-	+	-	-	-	-	-	-	-	-	-	-	104382	-
RPS6	Ribosomal protein S6, component of cytosolic 80S ribosome and 40S small subunit	-	+	+	-	-	-	GB18619-PA	AT4G31700.1	55398	BDEG_02299	CE24592	160712	104348	275754
		-	-	-	-	-	-	-	-	-	-	-	-	104339	-
		-	-	-	-	-	-	-	-	-	-	-	-	104322	-
NIFU1,NFU1	Fe-S cluster assembly protein NifU	-	+	-	-	-	-	-	AT5G49940.1	6483	-	-	-	104310	-
		-	-	-	-	-	-	-	-	-	-	-	-	104305	-
HCP3401	Sin3 complex component	-	+	-	-	-	-	-	AT2G45640.1	7873	BDEG_01793	CE01495	170532	104302	256725
		-	+	-	-	-	-	-	-	-	-	-	-	104300	-
NDE1	NAD-dependent epimerase/dehydratase	-	+	-	-	-	-	-	AT1G30620.3	-	BDEG_07987	-	-	104299	-
		-	-	-	-	-	-	-	-	-	-	-	-	104296	-
	speract/scavenger receptor cystein-rich (SRCR) family protein, transmembrane glycoprotein	-	-	-	-	-	-	-	-	31907	-	-	-	104292	-
DSP6	Dual-Specificity Protein Phosphatase Homolog 6	-	+	-	-	-	-	-	AT3G10940.1	-	-	-	-	104286	-
		-	+	-	-	-	-	-	AT5G61670.2	60471	-	-	-	104223	262969
		-	-	-	-	-	-	-	-	-	-	-	-	104193	-
		-	+	-	-	-	-	-	-	-	-	CE14136	222927	104136	-
COQ2	para-hydroxybenzoate-polyprenyltransferase, mitochondrial precursor	-	+	-	-	-	-	GB16212-PA	AT4G23660.2	12429	BDEG_07800	CE28469	154146	104114	253869
		-	-	-	-	-	-	-	-	-	-	-	-	104111	-
		-	-	-	-	-	-	-	-	-	-	-	-	104097	-
		-	+	-	-	-	-	GB11736-PA	-	-	-	-	51873	104082	-
		-	-	-	-	-	-	-	-	-	-	-	-	104037	-
		-	-	-	-	-	-	-	AT1G80160.1	-	-	-	-	104003	-
		-	+	-	-	-	-	-	AT3G16190.1	-	-	-	-	103992	-
		-	-	-	-	-	-	-	-	-	-	-	-	103983	-
	Serine/threonine protein kinase	-	-	-	-	-	-	-	-	-	-	-	-	103962	-
		-	+	-	-	-	-	GB12024-PA	AT4G20760.1	39421	-	CE09002	110121	103881	-
		-	+	-	-	-	-	GB18449-PA	AT5G10330.3	-	BDEG_00710	-	-	103841	-
ALKA	ALKA; Aurora Like Kinase A	-	-	-	-	-	-	-	-	-	-	-	-	103830	-
		-	+	-	-	-	-	GB12498-PA	AT3G55730.1	7774	-	-	87885	103798	264269
PF16	Central pair associated protein	+	+	-	+	-	-	GB19953-PA	-	55534	BDEG_01859	-	154846	103782	298236
GCHI	Putative GTP cyclohydrolase I	-	+	-	-	-	-	GB15785-PA	AT3G07270.2	24239	BDEG_01266	CE05795	182286	103770	206129
		-	-	-	-	-	-	-	-	-	-	-	-	103754	-
		-	+	-	-	-	-	-	AT3G19490.1	-	-	-	-	103745	-
		-	-	-	-	-	-	-	-	-	-	-	-	103689	-
		-	-	-	-	-	-	-	-	-	-	-	-	103666	-
		-	+	-	-	-	-	-	AT5G22790.1	-	-	-	-	103640	-
		-	+	-	-	-	-	GB18431-PA	AT4G34580.1	-	-	-	168514	103623	-
		-	-	-	-	-	-	-	-	-	-	-	-	103617	-
		-	-	-	-	-	-	-	-	-	-	-	-	103596	-
PDE6	3',5'-cyclic-nucleotide phosphodiesterase	-	-	-	-	-	-	-	-	-	-	-	-	103590	-
		-	+	+	-	-	-	GB16992-PA	AT1G67070.1	25430	BDEG_02638	CE07925	229052	103567	224783
		-	+	-	-	-	-	-	AT5G64400.1	-	-	-	-	103549	-

Gene	Description	Data columns
	Serine/threonine protein kinase	[illegible]
HDA3406	Histone deacetylase	[illegible]
	Hypothetical translation initiation factor 2 -like.	[illegible]
POLD2	DNA polymerase delta small subunit	[illegible]
CDKC1		[illegible]
AK5	Adenylate Kinase	[illegible]
FAP121	Flagellar Associated Protein	[illegible]
ADCY25	adenylate cyclase	[illegible]
RABC1	RABC1, RabGTPase	[illegible]
MKP4	MAP Kinase Phosphatase Homolog 4	[illegible]
ADCY23	adenylate cyclase	[illegible]
RANBP1	RAN binding protein	[illegible]
ADCY26	adenylate cyclase	[illegible]
ARP3402	Actin-related protein	[illegible]
ADCY27	adenylate cyclase	[illegible]
BCKDC_E1a	mitochondrial branched-chain alpha-ketoacid deshydrogenase complex, E1 component, alpha subunit.	[illegible]
NFR1	NADPH-dependent flavin reductase	[illegible]
GAPN1	Glyceraldehyde 3-phosphate dehydrogenase, nonphosphorylating	[illegible]
MME4	NADP malic enzyme	[illegible]
MAPKKK3	MAP Kinase Kinase Kinase Homolog 3, putative	[illegible]
CYCA1		[illegible]
GDE1	Glycerophosphodiester phosphodiesterease	[illegible]
DSP7	Dual-Specificity Protein Phosphatase Homolog 7	[illegible]
IF2Be		[illegible]
FAP247	Flagellar Associated Protein	[illegible]
	putative flavodoxin-like protein	[illegible]
HTB38	Histone H2B	[illegible]
GCHII/DBPS	Putative Bifunctional GTP cyclohydrolase II/3,4-dihydroxy-2butanone-4-phosphate synthase	[illegible]
HCF101	ATP-binding protein, MRP/NBP35 family with N-terminal DUF59 domain	[illegible]
	PfkB-type carbohydrate kinase family protein, ribokinase	[illegible]
PDE1	3',5'-cyclic-nucleotide phosphodiesterase	[illegible]
MRPL20	Putative mitochondrial ribosomal protein L20, imported to mitochondria	[illegible]
TEF15	Unknown function, chloroplast location proposed	[illegible]
	GCIP domain protein	[illegible]
ARG7	Argininosuccinate lyase	[illegible]
ANK2,FAP87	flagellar protein with ankyrin repeats	[illegible]
TRM2C	tRNA (uracil-5-)-methyltransferase	[illegible]
PKL1	Protein Phosphatase 1 beta with Kelch Like Repeat	[illegible]
PGLS		[illegible]
PDE4	3',5'-cyclic-nucleotide phosphodiesterase	[illegible]
CGE1	Chloroplast GrpE homolog 1	[illegible]
PDE14	3',5'-cyclic-nucleotide phosphodiesterase	[illegible]
UAP1	UDP-N-acetylglucosamine-pyrophosphorylase like protein	[illegible]
LCIE		[illegible]
BBS9		[illegible]
UCP3	uncoupling protein, probably mitochondrial	[illegible]
GTR8	glycosyl transferase	[illegible]
TBY2	Tubby protein	[illegible]
CBLE	putative methionine synthase reductase	[illegible]
FAP267	Flagellar Associated Protein with similarity to tubulin tyrosine ligase	[illegible]
HTB19	Histone H2B	[illegible]
GDPD3	glycerophosphoryl diester phosphodiesterase family protein	[illegible]
HTB3	Histone H2B	[illegible]
HFO20	Histone H4	[illegible]

		-	-	-	-	-	-	-	-	[illegible]
HFO36	Histone H4	-	-	-	-	-	-	-	-	[illegible]
GSH2		-	+	-	-	-	-	GB11231-PA	AT5G27380.1	[illegible]
		-	-	-	-	-	-	-	-	[illegible]
		-	+	+	-	-	-	GB16093-PA	AT5G10070.1	[illegible]
		-	+	-	-	-	-	-	AT5G58240.1	[illegible]
		-	+	+	-	-	-	GB11332-PB	AT1G03190.2	[illegible]
		-	+	-	-	-	-	-	-	[illegible]
		-	+	-	-	-	-	GB13213-PA	AT3G60240.4	[illegible]
		-	+	-	-	-	-	-	AT3G53110.1	[illegible]
		-	+	-	-	-	-	-	-	[illegible]
		-	-	-	-	-	-	-	-	[illegible]
CYN37	Peptidyl-prolyl cis-trans isomerase, cyclophilin-type	-	+	-	-	-	-	-	AT3G15520.1	[illegible]
		-	-	-	-	-	-	-	-	[illegible]
ARL13	ARF-like 13 GTPase, probably associated with the flagellum	-	-	-	-	-	-	-	-	[illegible]
		-	+	-	-	-	-	-	AT5G20140.1	[illegible]
		-	-	-	-	-	-	-	-	[illegible]
HTB17	Histone H2B	-	-	-	-	-	-	-	-	[illegible]
		-	+	+	-	-	-	GB11320-PA	AT5G08420.1	[illegible]
HTA17	Histone H2A	-	-	-	-	-	-	-	-	[illegible]
		-	+	-	-	-	-	-	AT5G07910.1	[illegible]
PPT1	putative translocator	-	+	-	-	-	-	-	-	[illegible]
		-	+	-	-	-	-	GB15829-PA	AT1G48175.1	[illegible]
HTB18	Histone H2B	-	-	-	-	-	-	-	-	[illegible]
		-	-	-	-	-	-	-	-	[illegible]
HTA18	Histone H2A	-	-	-	-	-	-	-	-	[illegible]
STPK3	Serine/Threonine Protein Kinase Homolog 3, putative	-	-	-	-	-	-	-	AT5G60550.1	[illegible]
QCR2	Ubiquinol:cytochrome c oxidoreductase (Complex III) 50 kDa subunit, Core 2 subunit, mitochondrial	-	+	-	-	-	-	GB19004-PA	-	[illegible]
TPR2		-	+	-	-	-	-	-	AT3G05625.1	[illegible]
		-	-	-	-	-	-	-	-	[illegible]
HTR35	Histone H3	-	-	-	-	-	-	-	-	[illegible]
HFO35	Histone H4	-	-	-	-	-	-	-	-	[illegible]
HTB16	Histone H2B	-	-	-	-	-	-	-	-	[illegible]
HTB15	Histone H2B	-	-	-	-	-	-	-	-	[illegible]
HFO17	Histone H4	-	-	-	-	-	-	-	-	[illegible]
HFO15	Histone H4	-	-	-	-	-	-	-	-	[illegible]
PSAL		-	+	-	-	-	-	-	AT4G12800.1	[illegible]
		-	+	-	-	-	-	GB18295-PA	AT3G09560.1	[illegible]
HTA15	Histone H2A	-	-	-	-	-	-	-	-	[illegible]
		-	-	-	-	-	-	-	AT3G28970.1	[illegible]
		-	+	-	-	-	-	-	AT4G35890.1	[illegible]
		-	-	-	-	-	-	-	-	[illegible]
		-	+	-	-	-	-	GB16302-PA	AT1G54350.1	[illegible]
		-	+	-	-	-	-	GB11456-PA	AT2G04690.2	[illegible]
		-	-	-	-	-	-	-	-	[illegible]
		-	+	-	-	-	-	GB10626-PA	AT5G23535.1	[illegible]
		-	-	-	-	-	-	-	-	[illegible]
		-	+	-	-	-	-	GB16110-PA	AT5G21160.1	[illegible]
CNK8	Chlamydomonas NIMA-related Kinase Homolog 8	-	-	-	-	-	-	-	-	[illegible]
		-	+	-	-	-	-	GB14789-PA	AT5G47620.2	[illegible]
PSB28	Photosystem II subunit 28, chloroplast precursor	-	+	-	-	-	-	-	AT4G28660.1	[illegible]
ATM3	half-size ABC transporter, membrane protein	-	-	-	-	-	-	-	-	[illegible]
C_450057		-	+	+	-	-	-	GB12263-PA	AT4G21520.1	[illegible]
		-	+	+	-	-	-	GB19273-PA	AT2G03120.1	[illegible]
		-	-	-	-	-	-	-	-	[illegible]
		-	+	-	-	-	-	-	AT1G53650.2	[illegible]
		-	-	-	-	-	-	-	-	[illegible]
		-	-	-	-	-	-	-	-	[illegible]
		-	-	-	-	-	-	-	-	[illegible]
		-	+	-	-	-	-	-	AT5G63290.1	[illegible]
		-	+	-	-	-	-	-	-	[illegible]
		-	-	-	-	-	-	GB13450-PA	-	[illegible]
PANE	Ketopantoate reductase	-	-	-	-	-	-	-	-	[illegible]
		-	+	-	-	-	-	-	AT4G34640.1	[illegible]
CBF5	pseudouridine synthase; centromere/microtubule binding protein	-	+	+	+	-	-	GB11348-PA	AT3G57150.1	[illegible]
		-	+	-	-	-	-	-	AT5G60540.1	[illegible]
CRB3	C3, RNA-binding protein of the CELF	-	-	-	-	-	-	-	-	[illegible]
PGM1	phosphoglycerate/bisphosphoglycerate mutase family protein	-	+	-	-	-	-	GB15052-PA	AT1G22170.1	[illegible]
		-	-	-	-	-	-	-	-	[illegible]
		-	-	-	-	-	-	-	-	[illegible]
		-	-	-	-	-	-	-	-	[illegible]
		-	+	-	-	-	-	GB16126-PA	AT4G18440.1	[illegible]
PRPL7/L12	Plastid ribosomal protein L12, imported to chloroplast, large ribosomal subunit	-	+	-	-	-	-	-	AT3G27830.1	[illegible]
		-	+	-	-	-	-	GB19068-PA	-	[illegible]
		-	+	-	-	-	-	-	AT1G79790.1	[illegible]
		-	-	-	-	-	-	-	-	[illegible]
		-	+	-	-	-	-	GB18843-PA	AT5G10910.1	[illegible]
		-	+	-	-	-	-	GB13595-PA	AT5G65620.1	[illegible]
		-	-	-	-	-	-	-	-	[illegible]
PDE15	3' 5' cyclic phosphodiesterase	-	-	-	-	-	-	-	-	[illegible]
		-	+	-	-	-	-	GB17822-PA	AT3G10180.1	[illegible]
GTR3	glycosyl transferase	-	-	-	-	-	-	-	-	[illegible]
		-	+	-	-	-	-	GB15281-PC	-	[illegible]
		-	+	-	-	-	-	-	AT1G31860.1	[illegible]
		-	-	-	-	-	-	-	-	[illegible]
EFG4,EGF4		-	+	-	-	-	-	-	AT5G10630.1	[illegible]
HAM3402	Histone acetyltransferase	-	-	-	-	-	-	-	-	[illegible]
		-	+	-	-	-	-	-	-	[illegible]
		-	+	-	-	-	-	-	AT5G07590.1	[illegible]
CYB5_4		-	+	-	-	-	-	-	AT1G60660.1	[illegible]
RRA1	Regulator of ribonuclease activity	-	+	-	-	-	-	-	AT5G56260.1	[illegible]
		-	+	-	-	-	-	GB18891-PA	AT5G48960.1	[illegible]
		-	-	-	-	-	-	-	-	[illegible]
ANK26	protein of unknown function with ankyrin repeats	-	-	-	-	-	-	-	-	[illegible]
		-	+	-	-	-	-	-	-	[illegible]
CYB5_3		-	+	-	-	-	-	GB18586-PA	AT5G09680.2	[illegible]
		-	-	-	-	-	-	-	-	[illegible]
		+	+	-	-	-	-	GB10742-PA	-	[illegible]
		-	-	-	-	-	-	-	-	[illegible]
C_1630018		-	+	-	-	-	-	-	AT5G57040.1	[illegible]
		-	+	-	-	-	-	-	AT2G45620.1	[illegible]
		-	+	-	-	-	-	GB18553-PA	AT5G63060.1	[illegible]
		-	-	-	-	-	-	-	AT1G18370.1	[illegible]
		-	+	-	-	-	-	-	-	[illegible]
ADCY22	adenylate cyclase	-	-	-	-	-	-	-	-	[illegible]
		-	-	-	-	-	-	-	-	[illegible]
FAP232	Conserved Uncharacterized Flagellar Associated Protein	+	+	-	+	-	-	-	-	[illegible]
		-	+	-	-	-	-	-	-	[illegible]
	Serine/threonine protein kinase	-	-	-	-	-	-	-	-	[illegible]
		-	+	-	-	-	-	-	AT1G09000.1	[illegible]
		-	-	-	-	-	-	-	-	[illegible]
		-	-	-	-	-	-	-	-	[illegible]
KAT2		-	+	-	-	+	-	-	AT2G34560.2	[illegible]
IFT57	Intraflagellar transport protein 57	+	+	-	+	-	-	GB10748-PA	-	[illegible]
		-	-	-	-	-	-	-	-	[illegible]
		-	+	-	-	-	-	-	AT5G05930.2	[illegible]
		-	-	-	-	-	-	-	AT5G23230.1	[illegible]
		-	-	-	-	-	-	-	-	[illegible]
		-	-	-	-	-	-	-	-	[illegible]
	putative serine-type peptidase;	-	+	-	-	-	-	-	AT3G57680.1	[illegible]
		-	-	-	-	-	-	-	-	[illegible]
		-	-	-	-	-	-	-	-	[illegible]
		-	+	-	-	-	-	GB18997-PA	AT5G49650.1	[illegible]
		-	-	-	-	-	-	-	-	[illegible]
ADCY4	adenylate cyclase	-	-	-	-	-	-	GB20021-PA	-	[illegible]
TRM8	tRNA (guanine-7)- methyltransferase	-	+	+	-	-	-	GB15383-PA	AT5G24840.1	[illegible]
		-	+	+	-	-	-	GB16646-PA	AT5G14600.1	[illegible]
		-	-	-	-	-	-	GB11725-PA	-	[illegible]
PIK	Phosphatidylinositol 4-kinase (PI4K)	-	+	-	-	-	-	-	-	[illegible]
		-	+	-	-	-	-	GB10538-PA	AT2G43760.2	[illegible]
ANK25	protein of unknown function with ankyrin repeats	-	+	-	-	-	-	-	AT3G04710.1	[illegible]
		-	-	-	-	-	-	-	-	[illegible]
		-	-	-	-	-	-	-	-	[illegible]
		-	+	-	-	-	-	-	AT1G48420.1	[illegible]
		-	-	-	-	-	-	-	AT2G47390.1	[illegible]
		-	+	-	-	-	-	-	AT1G14345.1	[illegible]
		-	-	-	-	-	-	-	-	[illegible]
		-	+	-	-	-	-	-	AT5G44070.1	[illegible]
		-	+	+	-	-	-	GB14253-PA	AT4G28450.1	[illegible]
		-	+	+	-	-	-	GB18416-PA	AT5G40530.1	[illegible]
		-	+	-	-	-	-	-	AT3G18290.1	[illegible]
		-	-	-	-	-	-	-	AT1G03900.1	[illegible]
		-	-	-	-	-	-	-	-	[illegible]
ADCY6	adenylate cyclase	-	-	-	-	-	-	-	-	[illegible]
ADCY7	adenylate cyclase	-	-	-	-	-	-	-	-	[illegible]
		-	+	+	-	-	-	-	-	[illegible]
EEF1	mitochondrial translation factor Tu	-	+	-	+	-	-	GB18497-PA	AT4G02930.1	[illegible]
HFO13	Histone H4	-	-	-	-	-	-	-	-	[illegible]
		-	-	-	-	-	-	-	-	[illegible]
		-	+	-	-	-	-	-	-	[illegible]
HTA8	Histone H2A	-	-	-	-	-	-	-	-	[illegible]
		-	+	-	-	-	-	-	AT3G23610.1	[illegible]
		-	+	+	-	-	-	GB12059-PA	AT4G14147.1	[illegible]
		-	+	+	-	-	-	GB19560-PA	AT5G14050.1	[illegible]
HTB6	Histone H2B	-	-	-	-	-	-	-	-	[illegible]
		-	+	-	-	-	-	-	AT1G14700.2	[illegible]

HTB12	Histone H2B	[illegible]
HFO34	Histone H4	[illegible]
PSBR		[illegible]
HTA14	Histone H2A	[illegible]
HTA7	Histone H2A	[illegible]
HTA6	Histone H2A	[illegible]
ADCY17	adenylate cyclase	[illegible]
HTB7	Histone H2B	[illegible]
NMT1		[illegible]
	protein of unknown function	[illegible]
HFO8	Histone H4	[illegible]
HTA4	Histone H2A	[illegible]
ADCY18	adenylate cyclase	[illegible]
HTR12	Histone H3	[illegible]
HFO14	Histone H4	[illegible]
HTR9	Histone H3	[illegible]
HTB9	Histone H2B	[illegible]
HFO4	Histone H4	[illegible]
HFO7	Histone H4	[illegible]
HFO32	Histone H4	[illegible]
EYE3	ABC1 ser/thr kinase	[illegible]
ADCY14	adenylate cyclase	[illegible]
HTV1	Histone H3 variant	[illegible]
FBA4	fructose-1,6-bisphosphate aldolase	[illegible]
ARFC1,ARL5	ARL5, ARF-like 5 GTPase	[illegible]
PACRG1	ciliary protein: Parkin Co-Regulated Gene	[illegible]
EFG	putative chloroplast elongation factor G	[illegible]
PANC	Pantothenate synthetase	[illegible]
JMJ3401	Jumonji protein	[illegible]
HSP90B	Heat shock protein 90B	[illegible]
PUS3	RNA pseudourine synthase, mitochondrial precursor, putative	[illegible]
MITC6	putative mitochondrial carrier protein	[illegible]
TPR3		[illegible]
PUS4	tRNA pseudouridine synthase, mitochondrial precursor, putative	[illegible]
C_130164		[illegible]
TRM1	N2,N2-dimethylguanosine tRNA methyltransferase	[illegible]
PYK1	pyruvate kinase	[illegible]
	Putative 6-hydroxymethyl-7,8-dihydropterin pyrophosphokinase/7,8-dihydropteroate synthase	[illegible]
DNJ35	DnaJ-like protein 34, probably cytosolic	[illegible]
IF1	organellar translation initiation factor 1	[illegible]
TPK	Thiamine pyrophosphokinase	[illegible]
FTSH4	membrane AAA-metalloprotease, mitochondrial	[illegible]
PRFA2		[illegible]
CLPY2	ATP-dependent subunit of the HslUV protease	[illegible]
CYN59	Peptidyl-prolyl cis-trans isomerase, cyclophilin-type	[illegible]
RPS26	Ribosomal protein S26, component of cytosolic 80S ribosome and 40S small subunit	[illegible]
MKP1	MAP Kinase Phosphatase Homolog 1	[illegible]
MRP7	ABC transporter, Multidrug resistance associated protein, membrane protein	[illegible]
SDC1		[illegible]
C_370034		[illegible]
C_150155		[illegible]
S6K1	Ribosomal protein S6 kinase	[illegible]
LCI8	Putative amino-acid N-acetyltransferase	[illegible]

APC8	Subunit of anaphase promoting complex	-	+	-	-	-	-	GB19210-PA	AT3G48150.1	71780	BDEG_02904	CE26888	222334	38821	242875	cgd7_1440	CML190C	OTTDARP00000021850	DDB0168974	CG2508-PA	19074393	-	ENSGALP00000012291	-	OTTHUMP00000159484	LmjF12.0610	114719	26870	806	204335	NCU01174	Os02g43920.1	33882	GSPATP00028711001	38886	130182	158535	PF14_0031	834319	RO3G_17010.1	YHR166C	SPAC6F12.14	GLEAN3_12696	SINFRUP00000159406	120.m00130	35095	-	80.m00069	21637	93446.m00263	Tb927.1.3910	UM00651.1
		-	+	-	-	-	-	-	AT2G38840.1	29376	-	CE32967	-	38740	223870	-	-	OTTDARP00000022596	DDB0204578	-	-	262.m00062	ENSGALP00000001014	-	OTTHUMP00000012394	-	-	-	65393	246436	-	Os03g58240.1	-	GSPATP00016803001	54726	133051	143525	-	-	-	-	-	-	-	-	-	TA13590	-	30624	-	-	-
		-	+	-	-	-	-	GB11830-PA	AT1G50200.1	53556	BDEG_03571	CE09768	155717	38643	372386	cgd6_560	CMM314C	OTTDARP00000009833	DDB0218104	CG13391-PB	19068710	-	ENSGALP00000003043	-	OTTHUMP00000080084	LmjF22.1540	197664	14194	58374	189992	NCU02566	Os10g10244.1	376	-	47999	140193	109348	PF13_0354	558710	RO3G_07558.1	YOR335C	SPAC23C11.09	GLEAN3_04893	SINFRUP00000160311	-	40774	TA16780	38.m01067	23916	-	Tb927.6.700	UM05233.1
		-	+	-	-	-	-	-	AT4G00630.1	3042	-	-	-	38583	-	-	CMS232C	-	-	-	-	-	-	-	-	-	-	-	-	-	-	Os04g58620.1	29166	-	11843	147518	-	-	823952	-	-	-	-	-	-	-	-	-	-	-	-	-
GIDA1	tRNA uridine 5-carboxymethylaminomethyl modification enzyme (Glucose-inhibited division protein A)	-	+	-	-	-	-	GB13702-PA	AT2G13440.1	58702	-	CE03398	-	38547	-	-	CMF066C	-	DDB0188295	CG4610-PA	-	-	ENSGALP00000025621	-	OTTHUMP00000016738	-	217378	15339	617	-	NCU11170	Os01g72980.1	1012	-	19579	112486	131207	PFL2115c	207152	RO3G_11664.1	YGL236C	SPBC30B4.06c	GLEAN3_27226	SINFRUP00000167699	27.m00310	268238	TA09230	49.m05694	30217	-	-	UM04402.1
		-	+	-	-	-	-	GB17112-PA	AT5G26240.1	72054	-	CE29336	152561	38456	220634	-	CMQ133C	-	DDB0217756	CG8594-PA	-	18.m00292	ENSGALP00000012212	-	OTTHUMP00000041762	LmjF32.3370	155388	14112	82075	172190	NCU06624	Os03g48940.1	-	-	28245	151168	132758	-	231287	RO3G_08188.1	YJR040W	SPBC19C7.11	GLEAN3_06772	SINFRUP00000169437	-	263848	-	80.m02270	20821	-	Tb09.211.0430	-
FBB1		-	-	-	-	-	-	-	-	-	-	-	-	38409	-	-	-	-	-	-	-	-	-	-	-	-	-	-	-	-	-	-	-	-	-	-	-	-	-	-	-	-	-	-	-	-	-	-	-	-	-	-
		-	+	-	-	-	-	GB16904-PA	AT3G01610.1	55474	BDEG_08666	CE28132	169679	38371	-	cgd5_2010	CMS124C	-	-	CG8571-PB	-	211.m00073	ENSGALP00000015157	-	NP_996671.1	LmjF27.1050	166366	-	81676	171694	-	Os06g01980.1	838	-	769	117191	131401	-	571847	RO3G_03713.1	YLL034C	SPBC16E9.10c	GLEAN3_18934	SINFRUP00000130725	503.m00005	37748	-	-	11565	-	Tb11.55.0014	UM01296.1
HYP8		-	+	+	-	-	-	GB18149-PA	AT1G80930.1	987	BDEG_06152	CE17753	167187	38363	260885	cgd8_2960	-	OTTDARP00000011599	DDB0189551	CG12750-PA	-	3.m00585	ENSGALP00000014627	-	NP_065994.1	LmjF36.5160	138828	32571	1038	139133	NCU00066	Os12g15420.1	591	GSPATP00003845001	11785	164611	129650	PFL1855w	577016	RO3G_08160.1	YGR278W	SPBC13E7.01	-	SINFRUP00000180336	16.m00503	32217	TA09690	641.m01576	23262	97324.m00246	Tb11.01.2520	UM02033.1
		-	+	-	-	-	-	-	-	29805	-	-	-	38137	-	-	-	-	-	-	-	-	-	-	-	-	-	-	-	-	-	-	288	-	12452	-	-	-	-	-	-	-	-	-	-	7268	-	52.m01553	-	-	Tb10.389.1170	-
TOP2		-	+	+	-	-	-	GB11911-PA	AT3G23890.1	22331	BDEG_00424	CE06184	159718	38019	220809	cgd4_780	CMB013C	OTTDARP00000016537	DDB0191045	CG10223-PA	19068754	138.m00082	ENSGALP00000036921	GL50803_16975	NP_001059.2	LmjF28.2280	223462	11792	29557	162970	NCU06338	Os02g47150.1	36039	GSPATP00006180001	52174	134247	137320	PF14_0316	557797	RO3G_08724.1	YNL088W	SPBC1A4.03c	GLEAN3_04587	SINFRUP00000139068	53.m00207	36420	TA13325	583.m05471	23201	81617.m00189	Tb11.01.3390	UM03501.1
		-	-	-	-	-	-	-	-	-	-	-	-	-	-	-	-	-	-	-	-	-	-	-	-	-	-	-	-	-	-	-	-	-	-	-	-	-	-	-	-	-	-	-	-	-	-	-	-	-	-	-
		-	-	-	-	-	-	-	-	-	-	-	-	181839	-	-	-	-	-	-	-	-	-	-	-	-	-	-	-	-	-	-	23973	-	-	-	-	-	-	-	-	-	-	-	-	-	-	-	-	-	-	-
		-	-	-	-	-	-	-	-	-	-	-	-	181812	-	-	-	-	-	-	-	-	-	-	-	-	-	-	-	-	-	-	-	-	-	-	-	-	-	-	-	-	-	-	-	-	-	-	-	-	-	-
		-	-	-	-	-	-	-	-	-	-	-	-	181808	-	-	-	-	-	-	-	-	-	-	-	-	-	-	-	-	-	-	-	-	-	-	-	-	-	-	-	-	-	-	-	-	-	-	-	-	-	-
		-	-	-	-	-	-	-	-	-	-	-	-	181801	-	-	-	-	-	-	-	-	-	-	-	-	-	-	-	-	-	-	-	-	-	-	-	-	-	-	-	-	-	-	-	-	-	-	-	-	-	-
		-	-	-	-	-	-	-	-	-	-	-	-	181780	-	-	-	-	-	-	-	-	-	-	-	-	-	-	-	-	-	-	-	-	-	-	-	-	-	-	-	-	-	-	-	-	-	-	-	-	-	-
		-	-	-	-	-	-	-	-	-	-	-	-	-	-	-	-	-	-	-	-	-	-	-	-	-	-	-	-	-	-	-	-	-	-	-	-	-	-	-	-	-	-	-	-	-	-	-	-	-	-	-
		-	+	-	-	-	-	-	-	-	-	-	-	181777	-	-	-	-	-	-	-	-	-	-	-	-	-	-	-	-	-	-	-	-	11126	-	143577	-	-	RO3G_02178.1	-	-	GLEAN3_12818	-	-	-	-	-	-	-	-	UM04788.1
		-	-	-	-	-	-	-	-	-	-	-	-	181776	-	-	-	-	-	-	-	-	-	-	-	-	-	-	-	-	-	-	-	-	-	-	-	-	-	-	-	-	-	-	-	-	-	-	-	-	-	-
		-	-	-	-	-	-	-	-	-	-	-	-	181768	-	-	-	-	-	-	-	-	-	-	-	-	-	-	-	-	-	-	-	-	-	-	-	-	-	-	-	-	-	-	-	-	-	-	-	-	-	-
		-	-	-	-	-	-	-	-	-	-	-	-	181767	-	-	-	-	-	-	-	-	-	-	-	-	-	-	-	-	-	-	-	-	-	-	-	-	-	-	-	-	-	-	-	-	-	-	-	-	-	-
		-	-	-	-	-	-	-	-	-	-	-	-	181765	-	-	-	-	-	-	-	-	-	-	-	-	-	-	-	-	-	-	-	-	-	-	-	-	-	-	-	-	-	-	-	-	-	-	-	-	-	-
		-	-	-	-	-	-	-	-	-	-	-	-	181751	-	-	-	-	-	-	-	-	-	-	-	-	-	-	-	-	-	-	-	-	-	-	-	-	-	-	-	-	-	-	-	-	-	-	-	-	-	-
		-	-	-	-	-	-	-	-	-	-	-	-	181747	-	-	-	-	-	-	-	-	-	-	-	-	-	-	-	-	-	-	-	-	-	-	-	-	-	-	-	-	-	-	-	-	-	-	-	-	-	-
		-	-	-	-	-	-	-	-	-	-	-	-	181746	-	-	-	-	-	-	-	-	-	-	-	-	-	-	-	-	-	-	-	-	-	-	-	-	-	-	-	-	-	-	-	-	-	-	-	-	-	-
		-	-	-	-	-	-	-	-	-	-	-	-	181742	-	-	-	-	-	-	-	-	-	-	-	-	-	-	-	-	-	-	-	-	-	-	-	-	-	-	-	-	-	-	-	-	-	-	-	-	-	-
		-	-	-	-	-	-	-	-	-	-	-	-	181740	-	-	-	-	-	-	-	-	-	-	-	-	-	-	-	-	-	-	-	-	-	-	-	-	-	-	-	-	-	-	-	-	-	-	-	-	-	-
		-	-	-	-	-	-	-	-	-	-	-	-	181739	-	-	-	-	-	-	-	-	-	-	-	-	-	-	-	-	-	-	-	-	-	-	-	-	-	-	-	-	-	-	-	-	-	-	-	-	-	-
		-	-	-	-	-	-	-	-	-	-	-	-	-	-	-	-	-	-	-	-	-	-	-	-	-	-	-	-	-	-	-	-	-	-	-	-	-	-	-	-	-	-	-	-	-	-	-	-	-	-	-
		-	-	-	-	-	-	-	-	-	-	-	-	181737	-	-	-	-	-	-	-	-	-	-	-	-	-	-	-	-	-	-	-	-	-	-	-	-	-	-	-	-	-	-	-	-	-	-	-	-	-	-
		-	-	-	-	-	-	-	-	-	-	-	-	181734	-	-	-	-	-	-	-	-	-	-	-	-	-	-	-	-	-	-	-	-	-	-	-	-	-	-	-	-	-	-	-	-	-	-	-	-	-	-
		-	-	-	-	-	-	-	-	-	-	-	-	181733	-	-	-	-	-	-	-	-	-	-	-	-	-	-	-	-	-	-	-	-	-	-	-	-	-	-	-	-	-	-	-	-	-	-	-	-	-	-
		-	-	-	-	-	-	-	-	-	-	-	-	181732	-	-	-	-	-	-	-	-	-	-	-	-	-	-	-	-	-	-	-	-	-	-	-	-	-	-	-	-	-	-	-	-	-	-	-	-	-	-
		-	-	-	-	-	-	-	-	-	-	-	-	181731	-	-	-	-	-	-	-	-	-	-	-	-	-	-	-	-	-	-	-	-	-	-	-	-	-	-	-	-	-	-	-	-	-	-	-	-	-	-
		-	-	-	-	-	-	-	-	-	-	-	-	-	-	-	-	-	-	-	-	-	-	-	-	-	-	-	-	-	-	-	-	-	-	-	-	-	-	-	-	-	-	-	-	-	-	-	-	-	-	-
		-	-	-	-	-	-	-	-	-	-	-	-	181729	-	-	-	-	-	-	-	-	-	-	-	-	-	-	-	-	-	-	-	-	-	-	-	-	-	-	-	-	-	-	-	-	-	-	-	-	-	-
		-	-	-	-	-	-	-	-	-	-	-	-	181727	-	-	-	-	-	-	-	-	-	-	-	-	-	-	-	-	-	-	-	-	-	-	-	-	-	-	-	-	-	-	-	-	-	-	-	-	-	-
		-	-	-	-	-	-	-	-	-	-	-	-	181726	-	-	-	-	-	-	-	-	-	-	-	-	-	-	-	-	-	-	-	-	-	-	-	-	-	-	-	-	-	-	-	-	-	-	-	-	-	-
		-	-	-	-	-	-	-	-	-	-	-	-	181725	-	-	-	-	-	-	-	-	-	-	-	-	-	-	-	-	-	-	-	-	-	-	-	-	-	-	-	-	-	-	-	-	-	-	-	-	-	-
		-	-	-	-	-	-	-	-	-	-	-	-	181720	-	-	-	-	-	-	-	-	-	-	-	-	-	-	-	-	-	-	-	-	-	-	-	-	-	-	-	-	-	-	-	-	-	-	-	-	-	-
		-	-	-	-	-	-	-	-	-	-	-	-	181714	-	-	-	-	-	-	-	-	-	-	-	-	-	-	-	-	-	-	-	-	-	-	-	-	-	-	-	-	-	-	-	-	-	-	-	-	-	-
		-	-	-	-	-	-	-	-	-	-	-	-	181713	-	-	-	-	-	-	-	-	-	-	-	-	-	-	-	-	-	-	-	-	-	-	-	-	-	-	-	-	-	-	-	-	-	-	-	-	-	-
		-	-	-	-	-	-	-	-	-	-	-	-	181707	-	-	-	-	-	-	-	-	-	-	-	-	-	-	-	-	-	-	-	-	-	-	-	-	-	-	-	-	-	-	-	-	-	-	-	-	-	-
		-	-	-	-	-	-	-	-	-	-	-	-	181705	-	-	-	-	-	-	-	-	-	-	-	-	-	-	-	-	-	-	-	-	-	-	-	-	-	-	-	-	-	-	-	-	-	-	-	-	-	-
		-	-	-	-	-	-	-	-	-	-	-	-	181704	-	-	-	-	-	-	-	-	-	-	-	-	-	-	-	-	-	-	-	-	-	-	-	-	-	-	-	-	-	-	-	-	-	-	-	-	-	-
		-	-	-	-	-	-	-	-	-	-	-	-	181703	-	-	-	-	-	-	-	-	-	-	-	-	-	-	-	-	-	-	-	-	46093	-	-	-	-	-	-	-	-	-	-	-	-	76.m01588	-	-	-	-
		-	-	-	-	-	-	-	-	-	-	-	-	181701	-	-	-	-	-	-	-	-	-	-	-	-	-	-	-	-	-	-	-	-	-	-	-	-	-	-	-	-	-	-	-	-	-	-	-	-	-	-
		-	-	-	-	-	-	-	-	-	-	-	-	181700	-	-	-	-	-	-	-	-	-	-	-	-	-	-	-	-	-	-	-	-	-	-	-	-	-	-	-	-	-	-	-	-	-	-	-	-	-	-
		-	-	-	-	-	-	-	-	-	-	-	-	181699	-	-	-	-	-	-	-	-	-	-	-	-	-	-	-	-	-	-	-	-	-	-	-	-	-	-	-	-	-	-	-	-	-	-	-	-	-	-
		-	-	-	-	-	-	-	-	-	-	-	-	181697	-	-	-	-	-	-	-	-	-	-	-	-	-	-	-	-	-	-	-	-	-	-	-	-	-	-	-	-	-	-	-	-	-	-	-	-	-	-
		-	-	-	-	-	-	-	-	-	-	-	-	181696	-	-	-	-	-	-	-	-	-	-	-	-	-	-	-	-	-	-	-	-	-	-	-	-	-	-	-	-	-	-	-	-	-	-	-	-	-	-
		-	-	-	-	-	-	-	-	-	-	-	-	181695	-	-	-	-	-	-	-	-	-	-	-	-	-	-	-	-	-	-	-	-	-	-	-	-	-	-	-	-	-	-	-	-	-	-	-	-	-	-
		-	-	-	-	-	-	-	-	-	-	-	-	181694	-	-	-	-	-	-	-	-	-	-	-	-	-	-	-	-	-	-	-	-	-	-	-	-	-	-	-	-	-	-	-	-	-	-	-	-	-	-
		-	-	-	-	-	-	-	-	-	-	-	-	181691	-	-	-	-	-	-	-	-	-	-	-	-	-	-	-	-	-	-	-	-	-	-	-	-	-	-	-	-	-	-	-	-	-	-	-	-	-	-
		-	-	-	-	-	-	-	-	-	-	-	-	181690	-	-	-	-	-	-	-	-	-	-	-	-	-	-	-	-	-	-	-	-	-	-	-	-	-	-	-	-	-	-	-	-	-	-	-	-	-	-
		-	-	-	-	-	-	-	-	-	-	-	-	181684	-	-	-	-	-	-	-	-	-	-	-	-	-	-	29554	-	-	-	-	-	-	-	-	-	-	-	-	-	-	-	-	-	-	-	-	-	-	-
		-	-	-	-	-	-	-	-	-	-	-	-	181681	-	-	-	-	-	-	-	-	-	-	-	-	-	-	-	-	-	-	-	-	-	-	-	-	-	-	-	-	-	-	-	-	-	-	-	-	-	-
		-	-	-	-	-	-	-	-	-	-	-	-	181679	-	-	-	-	-	-	-	-	-	-	-	-	-	-	-	-	-	-	-	-	-	-	-	-	-	-	-	-	-	-	-	-	-	-	-	-	-	-
		-	-	-	-	-	-	-	-	-	-	-	-	181662	-	-	-	-	-	-	-	-	-	-	-	-	-	-	-	-	-	-	-	-	-	-	-	-	-	-	-	-	-	-	-	-	-	-	-	-	-	-
		-	-	-	-	-	-	-	-	-	-	-	-	181659	-	-	-	-	-	-	-	-	-	-	-	-	-	-	-	-	-	-	-	-	-	-	-	-	-	-	-	-	-	-	-	-	-	-	-	-	-	-
		-	-	-	-	-	-	-	-	-	-	-	-	181651	-	-	-	-	-	-	-	-	-	-	-	-	-	-	-	-	-	-	-	-	-	-	-	-	-	-	-	-	-	-	-	-	-	-	-	-	-	-
		-	-	-	-	-	-	-	-	-	-	-	-	181650	-	-	-	-	-	-	-	-	-	-	-	-	-	-	-	-	-	-	-	-	-	-	-	-	-	-	-	-	-	-	-	-	-	-	-	-	-	-
		-	-	-	-	-	-	-	-	-	-	-	-	181634	-	-	-	-	-	-	-	-	-	-	-	-	-	-	-	-	-	-	-	-	-	-	-	-	-	-	-	-	-	-	-	-	-	-	-	-	-	-
		-	-	-	-	-	-	-	-	-	-	-	-	181625	-	-	-	-	-	-	-	-	-	-	-	-	-	-	-	-	-	-	-	-	-	-	-	-	-	-	-	-	-	-	-	-	-	-	-	-	-	-
		-	-	-	-	-	-	-	-	-	-	-	-	181624	-	-	-	-	-	-	-	-	-	-	-	-	-	-	-	-	-	-	-	-	-	-	-	-	-	-	-	-	-	-	-	-	-	-	-	-	-	-
EZY2	protein of unknown function	-	-	-	-	-	-	-	-	-	-	-	-	181623	-	-	-	-	-	-	-	-	-	-	-	-	-	-	-	-	-	-	-	-	-	-	-	-	-	-	-	-	-	-	-	-	-	-	-	-	-	-
		-	-	-	-	-	-	-	-	-	-	-	-	-	-	-	-	-	-	-	-	-	-	-	-	-	-	-	-	-	-	-	-	-	-	-	-	-	-	-	-	-	-	-	-	-	-	-	-	-	-	-
		-	-	-	-	-	-	-	-	-	-	-	-	181609	-	-	-	-	-	-	-	-	-	-	-	-	-	-	-	-	-	-	-	-	-	-	-	-	-	-	-	-	-	-	-	-	-	-	-	-	-	-
		-	-	-	-	-	-	-	-	-	-	-	-	181604	-	-	-	-	-	-	-	-	-	-	-	-	-	-	-	-	-	-	-	-	-	-	-	-	-	-	-	-	-	-	-	-	-	-	-	-	-	-
		-	-	-	-	-	-	-	-	-	-	-	-	181599	-	-	-	-	-	-	-	-	-	-	-	-	-	-	-	-	-	-	-	-	-	-	-	-	-	-	-	-	-	-	-	-	-	-	-	-	-	-
		-	-	-	-	-	-	-	-	-	-	-	-	181595	-	-	-	-	-	-	-	-	-	-	-	-	-	-	-	-	-	-	-	-	-	-	-	-	-	-	-	-	-	-	-	-	-	-	-	-	-	-
		-	-	-	-	-	-	-	-	-	-	-	-	181592	-	-	-	-	-	-	-	-	-	-	-	-	-	-	-	-	-	-	-	-	-	-	-	-	-	-	-	-	-	-	-	-	-	-	-	-	-	-
		-	-	-	-	-	-	-	-	-	-	-	-	-	-	-	-	-	-	-	-	-	-	-	-	-	-	-	-	-	-	-	-	-	-	-	-	-	-	-	-	-	-	-	-	-	-	-	-	-	-	-
		-	-	-	-	-	-	-	-	-	-	-	-	-	-	-	-	-	-	-	-	-	-	-	-	-	-	-	-	-	-	-	-	-	-	-	-	-	-	-	-	-	-	-	-	-	-	-	-	-	-	-
		-	-	-	-	-	-	-	-	-	-	-	-	181582	-	-	-	-	-	-	-	-	-	-	-	-	-	-	-	-	-	-	-	-	-	-	-	-	-	-	-	-	-	-	-	-	-	-	-	-	-	-
		-	-	-	-	-	-	-	-	-	-	-	-	-	-	-	-	-	-	-	-	-	-	-	-	-	-	-	-	-	-	-	-	-	-	-	-	-	-	-	-	-	-	-	-	-	-	-	-	-	-	-
		-	-	-	-	-	-	-	-	-	-	-	-	-	-	-	-	-	-	-	-	-	-	-	-	-	-	-	-	-	-	-	-	-	-	-	-	-	-	-	-	-	-	-	-	-	-	-	-	-	-	-
		-	-	-	-	-	-	-	-	-	-	-	-	181567	-	-	-	-	-	-	-	-	-	-	-	-	-	-	-	-	-	-	-	-	-	-	-	-	-	-	-	-	-	-	-	-	-	-	-	-	-	-
		-	-	-	-	-	-	-	-	-	-	-	-	181563	-	-	-	-	-	-	-	-	-	-	-	-	-	-	-	-	-	-	-	-	-	-	-	-	-	-	-	-	-	-	-	-	-	-	-	-	-	-
		-	+	-	-	-	-	GB13778-PA	-	-	-	CE31173	202300	181561	251386	-	-	-	DDB0205657	-	-	-	-	GL50803_92495	-	-	238278	28035	-	41069	-	-	-	GSPATP00008010001	50153	-	-	PFC1045c	-	-	-	-	GLEAN3_27006	-	-	23615	-	83.m01260	57306	-	-	-
PYK7	pyruvate kinase	-	-	-	-	-	-	-	-	-	-	-	-	-	-	-	-	-	-	-	-	-	-	-	-	-	-	-	-	-	-	-	-	-	-	-	-	-	-	-	-	-	-	-	-	-	-	-	-	-	-	-
		-	-	-	-	-	-	-	-	-	-	-	-	181539	-	-	-	-	-	-	-	-	-	-	-	-	-	-	-	-	-	-	-	-	-	-	-	-	-	-	-	-	-	-	-	-	-	-	-	-	-	-
		-	-	-	-	-	-	-	-	-	-	-	-	181536	-	-	-	-	-	-	-	-	-	-	-	-	-	-	-	-	-	-	-	-	-	-	-	-	-	-	-	-	-	-	-	-	-	-	-	-	-	-
		-	-	-	-	-	-	-	-	-	-	-	-	181533	-	-	-	-	-	-	-	-	-	-	-	-	-	-	-	-	-	-	-	-	-	-	-	-	-	-	-	-	-	-	-	-	-	-	-	-	-	-
		-	-	-	-	-	-	-	-	-	-	-	-	181529	-	-	-	-	-	-	-	-	-	-	-	-	-	-	-	-	-	-	-	-	-	-	-	-	-	-	-	-	-	-	-	-	-	-	-	-	-	-
		-	+	+	-	-	-	GB16621-PA	AT5G41370.1	521	BDEG_01851	CE28998	153297	181520	290040	cgd8_1940	CMH245C	OTTDARP00000020153	DDB0206281	CG8019-PA	19068509	281.m00049	ENSGALP00000037325	GL50803_16512	OTTHUMP00000162265	LmjF32.3920	207144	32554	50960	96883	NCU06438	Os01g49680.1	8301	GSPATP00016096001	20574	168477	108640	PF10_0369	751297	RO3G_09652.1	YIL143C	SPAC17A5.06	GLEAN3_25786	SINFRUP00000133176	67.m00146	33970	TA11585	268.m00004	22911	85889.m00522	Tb11.01.7950	UM04069.1
		-	-	-	-	-	-	-	-	-	-	-	-	181517	-	-	-	-	-	-	-	-	-	-	-	-	-	-	-	-	-	-	-	-	-	-	-	-	-	-	-	-	-	-	-	-	-	-	-	-	-	-
		-	-	-	-	-	-	-	-	-	-	-	-	-	-	-	-	-	-	-	-	-	-	-	-	-	-	-	-	-	-	-	-	-	-	-	-	-	-	-	-	-	-	-	-	-	-	-	-	-	-	-
		-	-	-	-	-	-	-	-	-	-	-	-	181513	-	-	-	-	-	-	-	-	-	-	-	-	-	-	-	-	-	-	-	-	-	-	-	-	-	-	-	-	-	-	-	-	-	-	-	-	-	-
		-	-	-	-	-	-	-	-	-	-	-	-	181511	-	-	-	-	-	-	-	-	-	-	-	-	-	-	-	-	-	-	-	-	-	-	-	-	-	-	-	-	-	-	-	-	-	-	-	-	-	-
		-	-	-	-	-	-	-	-	-	-	-	-	181505	-	-	-	-	-	-	-	-	-	-	-	-	-	-	-	-	-	-	-	-	-	-	-	-	-	-	-	-	-	-	-	-	-	-	-	-	-	-
GTR15	glycosyl transferase	-	-	-	-	-	-	-	-	-	-	-	-	181503	-	-	-	-	-	-	-	-	-	-	-	-	-	-	-	-	-	-	-	-	-	-	-	-	-	-	-	-	-	-	-	-	-	-	-	-	-	-
		-	-	-	-	-	-	-	-	-	-	-	-	181502	-	-	-	-	-	-	-	-	-	-	-	-	-	-	-	-	-	-	-	-	-	-	-	-	-	-	-	-	-	-	-	-	-	-	-	-	-	-
		-	-	-	-	-	-	-	-	-	-	-	-	-	-	-	-	-	-	-	-	-	-	-	-	-	-	-	-	-	-	-	-	-	-	-	-	-	-	-	-	-	-	-	-	-	-	-	-	-	-	-
		-	-	-	-	-	-	-	-	-	-	-	-	181491	-	-	-	-	-	-	-	-	-	-	-	-	-	-	-	-	-	-	12972	-	-	-	-	-	-	-	-	-	-	-	94.m00116	-	-	-	-	-	-	-
		-	-	-	-	-	-	-	-	-	-	-	-	181490	-	-	-	-	-	-	-	-	-	-	-	-	-	-	-	-	-	-	-	-	-	-	-	-	-	-	-	-	-	-	-	-	-	-	-	-	-	-
		-	-	-	-	-	-	-	-	-	-	-	-	-	-	-	-	-	-	-	-	-	-	-	-	-	-	-	-	-	-	-	-	-	-	-	-	-	-	-	-	-	-	-	-	-	-	-	-	-	-	-
		-	+	-	-	-	-	-	-	-	-	-	-	181488	-	-	-	-	DDB0190137	-	-	-	-	-	-	-	-	-	-	-	-	Os04g29469.1	-	-	-	-	126223	-	-	RO3G_13909.1	-	-	GLEAN3_02879	-	-	-	-	-	-	-	-	UM06265.1
		-	-	-	-	-	-	-	-	-	-	-	-	181485	-	-	-	-	-	-	-	-	-	-	-	-	-	-	-	-	-	-	-	-	-	-	-	-	-	-	-	-	-	-	-	-	-	-	-	-	-	-
		-	-	-	-	-	-	-	-	-	-	-	-	181483	-	-	-	-	-	-	-	-	-	-	-	-	-	-	-	-	-	-	-	-	-	-	-	-	-	-	-	-	-	-	-	-	-	-	-	-	-	-
		-	-	-	-	-	-	-	-	-	-	-	-	181482	-	-	-	-	-	-	-	-	-	-	-	-	-	-	-	-	-	-	-	-	-	-	-	-	-	-	-	-	-	-	-	-	-	-	-	-	-	-
		-	-	-	-	-	-	-	-	-	-	-	-	181481	-	-	-	-	-	-	-	-	-	-	-	-	-	-	-	-	-	-	-	-	-	-	-	-	-	-	-	-	-	-	-	-	-	-	-	-	-	-
		-	-	-	-	-	-	-	-	-	-	-	-	181479	-	-	-	-	-	-	-	-	-	-	-	-	-	-	-	-	-	-	-	-	-	-	-	-	-	-	-	-	-	-	-	-	-	-	-	-	-	-
		-	-	-	-	-	-	-	-	-	-	-	-	181478	-	-	-	-	-	-	-	-	-	-	-	-	-	-	-	-	-	-	-	-	-	-	-	-	-	-	-	-	-	-	-	-	-	-	-	-	-	-
		-	-	-	-	-	-	-	-	-	-	-	-	181476	-	-	-	-	-	-	-	-	-	-	-	-	-	-	-	-	-	-	-	-	-	-	-	-	-	-	-	-	-	-	-	-	-	-	-	-	-	-
		-	-	-	-	-	-	-	-	-	-	-	-	181474	-	-	-	-	-	-	-	-	-	-	-	-	-	-	-	-	-	-	-	-	-	-	-	-	-	-	-	-	-	-	-	-	-	-	-	-	-	-
		-	-	-	-	-	-	-	-	-	-	-	-	181463	-	-	-	-	-	-	-	-	-	-	-	-	-	-	-	-	-	-	-	-	-	-	-	-	-	-	-	-	-	-	-	-	-	-	-	-	-	-
		-	-	-	-	-	-	-	-	-	-	-	-	181462	-	-	-	-	-	-	-	-	-	-	-	-	-	-	-	-	-	-	-	-	-	-	-	-	-	-	-	-	-	-	-	-	-	-	-	-	-	-
		-	-	-	-	-	-	-	-	-	-	-	-	181456	-	-	-	-	-	-	-	-	-	-	-	-	-	-	-	-	-	-	-	-	-	-	-	-	-	-	-	-	-	-	-	-	-	-	-	-	-	-
		-	-	-	-	-	-	-	-	-	-	-	-	181453	-	-	-	-	-	-	-	-	-	-	-	-	-	-	-	-	-	-	-	-	-	-	-	-	-	-	-	-	-	-	-	-	-	-	-	-	-	-
		-	-	-	-	-	-	-	-	-	-	-	-	181452	-	-	-	-	-	-	-	-	-	-	-	-	-	-	-	-	-	-	-	-	-	-	-	-	-	-	-	-	-	-	-	-	-	-	-	-	-	-
		-	-	-	-	-	-	-	-	-	-	-	-	181451	-	-	-	-	-	-	-	-	-	-	-	-	-	-	-	-	-	-	-	-	-	-	-	-	-	-	-	-	-	-	-	-	-	-	-	-	-	-
		-	-	-	-	-	-	-	-	-	-	-	-	181447	-	-	-	-	-	-	-	-	-	-	-	-	-	-	-	-	-	-	-	-	-	-	-	-	-	-	-	-	-	-	-	-	-	-	-	-	-	-
		-	-	-	-	-	-	-	-	-	-	-	-	-	-	-	-	-	-	-	-	-	-	-	-	-	-	-	-	-	-	-	-	-	-	-	-	-	-	-	-	-	-	-	-	-	-	-	-	-	-	-
		-	-	-	-	-	-	-	-	-	-	-	-	-	-	-	-	-	-	-	-	-	-	-	-	-	-	-	-	-	-	-	-	-	-	-	-	-	-	-	-	-	-	-	-	-	-	-	-	-	-	-
		-	-	-	-	-	-	-	-	-	-	-	-	-	-	-	-	-	-	-	-	-	-	-	-	-	-	-	-	-	-	-	-	-	-	-	-	-	-	-	-	-	-	-	-	-	-	-	-	-	-	-
		-	-	-	-	-	-	-	-	-	-	-	-	181438	-	-	-	-	-	-	-	-	-	-	-	-	-	-	-	-	-	-	Os07g16360.1	-	-	-	-	-	-	-	-	-	-	-	-	-	-	-	-	-	-	-	-
		-	-	-	-	-	-	-	-	-	-	-	-	181437	-	-	-	-	-	-	-	-	-	-	-	-	-	-	-	-	-	-	-	-	-	-	-	-	-	-	-	-	-	-	-	-	-	-	-	-	-	-
		-	-	-	-	-	-	-	-	-	-	-	-	181433	-	-	-	-	-	-	-	-	-	-	-	-	-	-	-	-	-	-	-	-	-	-	-	-	-	-	-	-	-	-	-	-	-	-	-	-	-	-
		-	-	-	-	-	-	-	-	-	-	-	-	181431	-	-	-	-	-	-	-	-	-	-	-	-	-	-	-	-	-	-	-	-	-	-	-	-	-	-	-	-	-	-	-	-	-	-	-	-	-	-
		-	-	-	-	-	-	-	-	-	-	-	-	181428	-	-	-	-	-	-	-	-	-	-	-	-	-	-	-	-	-	-	-	-	-	-	-	-	-	-	-	-	-	-	-	-	-	-	-	-	-	-
		-	-	-	-	-	-	-	-	-	-	-	-	181424	-	-	-	-	-	-	-	-	-	-	-	-	-	-	-	-	-	-	-	-	-	-	-	-	-	-	-	-	-	-	-	-	-	-	-	-	-	-
		-	-	-	-	-	-	-	-	-	-	-	-	181419	-	-	-	-	-	-	-	-	-	-	-	-	-	-	-	-	-	-	-	-	-	-	-	-	-	-	-	-	-	-	-	-	-	-	-	-	-	-
		-	-	-	-	-	-	-	-	-	-	-	-	-	-	-	-	-	-	-	-	-	-	-	-	-	-	-	-	-	-	-	-	-	-	-	-	-	-	-	-	-	-	-	-	-	-	-	-	-	-	-
		-	-	-	-	-	-	-	-	-	-	-	-	181414	-	-	-	-	-	-	-	-	-	-	-	-	-	-	-	-	-	-	-	-	-	-	-	-	-	-	-	-	-	-	-	-	-	-	-	-	-	-
		-	-	-	-	-	-	-	-	-	-	-	-	181412	-	-	-	-	-	-	-	-	-	-	-	-	-	-	-	-	-	-	-	-	-	-	-	-	-	-	-	-	-	-	-	-	-	-	-	-	-	-
		-	-	-	-	-	-	-	-	-	-	-	-	-	-	-	-	-	-	-	-	-	-	-	-	-	-	-	-	-	-	-	-	-	-	-	-	-	-	-	-	-	-	-	-	-	-	-	-	-	-	-
		-	-	-	-	-	-	-	-	-	-	-	-	181399	-	-	-	-	-	-	-	-	-	-	-	-	-	-	-	-	-	-	-	-	-	-	-	-	-	-	-	-	-	-	-	-	-	-	-	-	-	-
		-	-	-	-	-	-	-	-	-	-	-	-	181393	-	-	-	-	-	-	-	-	-	-	-	-	-	-	-	-	-	-	-	-	-	-	-	-	-	-	-	-	-	-	-	-	-	-	-	-	-	-
		-	-	-	-	-	-	-	-	-	-	-	-	181390	-	-	-	-	-	-	-	-	-	-	-	-	-	-	-	-	-	-	-	-	-	-	-	-	-	-	-	-	-	-	-	-	-	-	-	-	-	-
		-	-	-	-	-	-	-	-	-	-	-	-	181389	-	-	-	-	-	-	-	-	-	-	-	-	-	-	-	-	-	-	-	-	-	-	-	-	-	-	-	-	-	-	-	-	-	-	-	-	-	-
		-	-	-	-	-	-	-	-	-	-	-	-	181388	-	-	-	-	-	-	-	-	-	-	-	-	-	-	-	-	-	-	-	-	-	-	-	-	-	-	-	-	-	-	-	-	-	-	-	-	-	-
		-	-	-	-	-	-	-	-	-	-	-	-	181387	-	-	-	-	-	-	-	-	-	-	-	-	-	-	-	-	-	-	-	-	-	-	-	-	-	-	-	-	-	-	-	-	-	-	-	-	-	-
		-	-	-	-	-	-	-	-	-	-	-	-	181385	-	-	-	-	-	-	-	-	-	-	-	-	175158	-	-	-	-	-	-	-	-	-	-	-	-	-	-	-	-	-	-	-	-	-	-	-	-	-	-
		-	-	-	-	-	-	-	-	-	-	-	-	181384	-	-	-	-	-	-	-	-	-	-	-	-	-	-	-	-	-	-	-	-	-	-	-	-	-	-	-	-	-	-	-	-	-	-	-	-	-	-
		-	-	-	-	-	-	-	-	-	-	-	-	-	-	-	-	-	-	-	-	-	-	-	-	-	-	-	-	-	-	-	-	-	-	-	-	-	-	-	-	-	-	-	-	-	-	-	-	-	-	-
		-	-	-	-	-	-	-	-	-	-	-	-	181381	-	-	-	-	-	-	-	-	-	-	-	-	-	-	-	-	-	-	-	-	-	-	-	-	-	-	-	-	-	-	-	-	-	-	-	-	-	-
		-	-	-	-	-	-	-	-	-	-	-	-	181379	-	-	-	-	-	-	-	-	-	-	-	-	-	-	-	-	-	-	-	-	-	-	-	-	-	-	-	-	-	-	-	-	-	-	-	-	-	-
		-	-	-	-	-	-	-	-	-	-	-	-	181378	-	-	-	-	-	-	-	-	-	-	-	-	-	-	-	-	-	-	-	-	-	-	-	-	-	-	-	-	-	-	-	-	-	-	-	-	-	-
		-	-	-	-	-	-	-	-	-	-	-	-	181377	-	-	-	-	-	-	-	-	-	-	-	-	-	-	-	-	-	-	-	-	-	-	-	-	-	-	-	-	-	-	-	-	-	-	-	-	-	-
		-	-	-	-	-	-	-	-	-	-	-	-	181376	-	-	-	-	-	-	-	-	-	-	-	-	-	-	-	-	-	-	-	-	-	-	-	-	-	-	-	-	-	-	-	-	-	-	-	-	-	-
		-	-	-	-	-	-	-	-	-	-	-	-	181372	-	-	-	-	-	-	-	-	-	-	-	-	-	-	-	-	-	-	-	-	-	-	-	-	-	-	-	-	-	-	-	-	-	-	-	-	-	-
		-	-	-	-	-	-	-	-	-	-	-	-	181370	-	-	-	-	-	-	-	-	-	-	-	-	-	-	-	-	-	-	-	-	-	-	-	-	-	-	-	-	-	-	-	-	-	-	-	-	-	-
		-	-	-	-	-	-	-	-	-	-	-	-	181368	-	-	-	-	-	-	-	-	-	-	-	-	-	-	-	-	-	-	-	-	-	-	-	-	-	-	-	-	-	-	-	-	-	-	-	-	-	-
		-	-	-	-	-	-	-	-	-	-	-	-	181364	-	-	-	-	-	-	-	-	-	-	-	-	-	-	-	-	-	-	-	-	-	-	-	-	-	-	-	-	-	-	-	-	-	-	-	-	-	-
		-	-	-	-	-	-	-	-	-	-	-	-	181363	-	-	-	-	-	-	-	-	-	-	-	-	-	-	-	-	-	-	-	-	-	-	-	-	-	-	-	-	-	-	-	-	-	-	-	-	-	-
		-	-	-	-	-	-	-	-	-	-	-	-	181362	-	-	-	-	-	-	-	-	-	-	-	-	-	-	-	-	-	-	-	-	-	-	-	-	-	-	-	-	-	-	-	-	-	-	-	-	-	-
		-	-	-	-	-	-	-	-	-	-	-	-	181360	-	-	-	-	-	-	-	-	-	-	-	-	-	-	-	-	-	-	-	-	-	-	-	-	-	-	-	-	-	-	-	-	-	-	-	-	-	-
CLR8	Coiled-Like Repeat protein	-	-	-	-	-	-	-	-	-	-	-	-	181359	-	-	-	-	-	-	-	-	-	-	-	-	-	-	-	-	-	-	-	-	-	-	-	-	-	-	-	-	-	-	-	-	-	-	-	-	-	-
		-	-	-	-	-	-	-	-	-	-	-	-	181358	-	-	-	-	-	-	-	-	-	-	-	-	-	-	-	-	-	-	-	-	-	-	-	-	-	-	-	-	-	-	-	-	-	-	-	-	-	-
		-	-	-	-	-	-	-	-	-	-	-	-	181357	-	-	-	-	-	-	-	-	-	-	-	-	-	-	-	-	-	-	-	-	-	-	-	-	-	-	-	-	-	-	-	-	-	-	-	-	-	-
		-	-	-	-	-	-	-	-	-	-	-	-	181353	-	-	-	-	-	-	-	-	-	-	-	-	-	-	-	-	-	-	-	-	-	-	-	-	-	-	-	-	-	-	-	-	-	-	-	-	-	-
		-	-	-	-	-	-	-	-	-	-	-	-	181349	-	-	-	-	-	-	-	-	-	-	-	-	-	-	-	-	-	-	-	-	-	-	130739	-	-	RO3G_17087.1	-	-	-	-	-	-	-	-	-	-	-	-
HFO45	Histone H4	-	-	-	-	-	-	-	-	-	-	-	-	-	-	-	-	-	-	-	-	-	-	-	-	-	-	-	-	-	-	-	-	-	-	-	-	-	-	-	-	-	-	-	-	-	-	-	-	-	-	-
		-	-	-	-	-	-	-	-	-	-	-	-	181340	-	-	-	-	-	-	-	-	-	-	-	-	-	-	-	-	-	-	-	-	-	-	-	-	-	-	-	-	-	-	-	-	-	-	-	-	-	-

		-	-	-	-	-	-	-	-	-	-	-	-	181339	-	-	-	-	-	-	-	-	-	-	-	-	-	-	-	-	-	-	-	-	-	-	-	-	-	-	-	-	-	-	-	-	-	-	-	-	-	-
		-	-	-	-	-	-	-	-	-	-	-	-	181335	-	-	-	-	-	-	-	-	-	-	-	-	-	-	-	-	-	-	-	-	-	-	-	-	-	-	-	-	-	-	-	-	-	-	-	-	-	-
HRP2-1		-	-	-	-	-	-	-	-	-	-	-	-	181333	-	-	-	-	-	-	-	-	-	-	-	-	-	-	-	-	-	-	-	-	-	-	-	-	-	-	-	-	-	-	-	-	-	-	-	-	-	-
		-	-	-	-	-	-	-	-	-	-	-	-	181331	-	-	-	-	-	-	-	-	-	-	-	-	-	-	-	-	-	-	-	-	-	-	-	-	-	-	-	-	-	-	-	-	-	-	-	-	-	-
		-	-	-	-	-	-	-	-	-	-	-	-	181330	-	-	-	-	-	-	-	-	-	-	-	-	-	-	-	-	-	-	-	-	-	-	-	-	-	-	-	-	-	-	-	-	-	-	-	-	-	-
		-	-	-	-	-	-	-	-	-	-	-	-	181329	-	-	-	-	-	-	-	-	-	-	-	-	-	-	-	-	-	-	-	-	-	-	-	-	-	-	-	-	-	-	-	-	-	-	-	-	-	-
		-	-	-	-	-	-	-	-	-	-	-	-	181326	-	-	-	-	-	-	-	-	-	-	-	-	-	-	-	-	-	-	-	-	-	-	-	-	-	-	-	-	-	-	-	-	-	-	-	-	-	-
		-	-	-	-	-	-	-	-	-	-	-	-	181325	-	-	-	-	-	-	-	-	-	-	-	-	-	-	-	-	-	-	-	-	-	-	-	-	-	-	-	-	-	-	-	-	-	-	-	-	-	-
		-	-	-	-	-	-	-	-	-	-	-	-	181317	-	-	-	-	-	-	-	-	-	-	-	-	-	-	-	-	-	-	-	-	-	-	-	-	-	-	-	-	-	-	-	-	-	-	-	-	-	-
		-	-	-	-	-	-	-	-	-	-	-	-	181315	-	-	-	-	-	-	-	-	-	-	-	-	-	-	-	-	-	-	-	-	-	-	-	-	-	-	-	-	-	-	-	-	-	-	-	-	-	-
		-	-	-	-	-	-	-	-	-	-	-	-	181312	-	-	-	-	-	-	-	-	-	-	-	-	-	-	-	-	-	-	-	-	-	-	-	-	-	-	-	-	-	-	-	-	-	-	-	-	-	-
		-	-	-	-	-	-	-	-	-	-	-	-	181311	-	-	-	-	-	-	-	-	-	-	-	-	-	-	-	-	-	-	-	-	-	-	-	-	-	-	-	-	-	-	-	-	-	-	-	-	-	-
		-	-	-	-	-	-	-	-	-	-	-	-	181308	-	-	-	-	-	-	-	-	-	-	-	-	-	-	-	-	-	-	-	-	-	-	-	-	-	-	-	-	-	-	-	-	-	-	-	-	-	-
		-	-	-	-	-	-	-	-	-	-	-	-	-	-	-	-	-	-	-	-	-	-	-	-	-	-	-	-	-	-	-	-	-	-	-	-	-	-	-	-	-	-	-	-	-	-	-	-	-	-	-
		-	-	-	-	-	-	-	-	-	-	-	-	181305	-	-	-	-	-	-	-	-	-	-	-	-	-	-	-	-	-	-	-	-	-	-	-	-	-	-	-	-	-	-	-	-	-	-	-	-	-	-
		-	-	-	-	-	-	-	-	-	-	-	-	181297	-	-	-	-	-	-	-	-	-	-	-	-	-	-	-	-	-	-	-	-	-	-	-	-	-	-	-	-	-	-	-	-	-	-	-	-	-	-
		-	-	-	-	-	-	-	-	-	-	-	-	181290	-	-	-	-	-	-	-	-	-	-	-	-	-	-	-	-	-	-	-	-	-	-	-	-	-	-	-	-	-	-	-	-	-	-	-	-	-	-
		-	-	-	-	-	-	-	-	-	-	-	-	-	-	-	-	-	-	-	-	-	-	-	-	-	-	-	-	-	-	-	-	-	-	-	-	-	-	-	-	-	-	-	-	-	-	-	-	-	-	-
		-	-	-	-	-	-	-	-	-	-	-	-	-	-	-	-	-	-	-	-	-	-	-	-	-	-	-	-	-	-	-	-	-	-	-	-	-	-	-	-	-	-	-	-	-	-	-	-	-	-	-
		-	-	-	-	-	-	-	-	-	-	-	-	181280	-	-	-	-	-	-	-	-	-	-	-	-	-	-	-	-	-	-	-	-	-	-	-	-	-	-	-	-	-	-	-	-	-	-	-	-	-	-
		-	-	-	-	-	-	-	-	-	-	-	-	181278	-	-	-	-	-	-	-	-	-	-	-	-	-	-	-	-	-	-	-	-	-	-	-	-	-	-	-	-	-	-	-	-	-	-	-	-	-	-
		-	-	-	-	-	-	-	-	-	-	-	-	181273	-	-	-	-	-	-	-	-	-	-	-	-	-	-	-	-	-	-	-	-	-	-	-	-	-	-	-	-	-	-	-	-	-	-	-	-	-	-
HTR2B	Histone H3	-	-	-	-	-	-	-	-	-	-	-	-	-	-	-	-	-	-	-	-	-	-	-	-	-	-	-	-	-	-	-	-	-	-	-	-	-	-	-	-	-	-	-	-	-	-	-	-	-	-	-
		-	-	-	-	-	-	-	-	-	-	-	-	181264	-	-	-	-	-	-	-	-	-	-	-	-	-	-	-	-	-	-	-	-	-	-	-	-	-	-	-	-	-	-	-	-	-	-	-	-	-	-
		-	-	-	-	-	-	-	-	-	-	-	-	181263	-	-	-	-	-	-	-	-	-	-	-	-	-	-	-	-	-	-	-	-	-	-	-	-	-	-	-	-	-	-	-	-	-	-	-	-	-	-
		-	-	-	-	-	-	-	-	-	-	-	-	181262	-	-	-	-	-	-	-	-	-	-	-	-	-	-	-	-	-	-	-	-	-	-	-	-	-	-	-	-	-	-	-	-	-	-	-	-	-	-
		-	-	-	-	-	-	-	-	-	-	-	-	181259	-	-	-	-	-	-	-	-	-	-	-	-	-	-	-	-	-	-	-	-	-	-	-	-	-	-	-	-	-	-	-	-	-	-	-	-	-	-
		-	-	-	-	-	-	-	-	-	-	-	-	181254	-	-	-	-	-	-	-	-	-	-	-	-	-	-	-	-	-	-	-	-	-	-	-	-	-	-	-	-	-	-	-	-	-	-	-	-	-	-
		-	-	-	-	-	-	-	-	-	-	-	-	181253	-	-	-	-	-	-	-	-	-	-	-	-	-	-	-	-	-	-	-	-	-	-	-	-	-	-	-	-	-	-	-	-	-	-	-	-	-	-
		-	-	-	-	-	-	-	-	-	-	-	-	181249	-	-	-	-	-	-	-	-	-	-	-	-	-	-	-	-	-	-	-	-	-	-	-	-	-	-	-	-	-	-	-	-	-	-	-	-	-	-
		-	-	-	-	-	-	-	-	-	-	-	-	181247	-	-	-	-	-	-	-	-	-	-	-	-	-	-	-	-	-	-	-	-	-	-	-	-	-	-	-	-	-	-	-	-	-	-	-	-	-	-
		-	-	-	-	-	-	-	-	-	-	-	-	181246	-	-	-	-	-	-	-	-	-	-	-	-	-	-	-	-	-	-	-	-	-	-	-	-	-	-	-	-	-	-	-	-	-	-	-	-	-	-
		-	-	-	-	-	-	-	-	-	-	-	-	181245	-	-	-	-	-	-	-	-	-	-	-	-	-	-	-	-	-	-	-	-	-	-	-	-	-	-	-	-	-	-	-	-	-	-	-	-	-	-
		-	-	-	-	-	-	-	-	-	-	-	-	181244	-	-	-	-	-	-	-	-	-	-	-	-	-	-	-	-	-	-	-	-	-	-	-	-	-	-	-	-	-	-	-	-	-	-	-	-	-	-
		-	-	-	-	-	-	-	-	-	-	-	-	181243	-	-	-	-	-	-	-	-	-	-	-	-	-	-	-	-	-	-	-	-	-	-	-	-	-	-	-	-	-	-	-	-	-	-	-	-	-	-
		-	+	-	-	-	-	-	AT4G17420.1	-	BDEG_03128	-	-	181241	-	-	-	-	DDB0189298	-	-	-	-	-	-	-	-	-	-	-	NCU04645	-	-	-	-	-	-	-	-	RO3G_11146.1	-	-	-	-	-	4012	-	-	-	-	-	-
		-	-	-	-	-	-	-	-	-	-	-	-	181240	-	-	-	-	-	-	-	-	-	-	-	-	-	-	-	-	-	-	-	-	-	-	-	-	-	-	-	-	-	-	-	-	-	-	-	-	-	-
		-	-	-	-	-	-	-	-	-	-	-	-	181239	-	-	-	-	-	-	-	-	-	-	-	-	-	-	-	-	-	-	-	-	-	-	-	-	-	-	-	-	-	-	-	-	-	-	-	-	-	-
		-	-	-	-	-	-	-	-	-	-	-	-	181237	-	-	-	-	DDB0188334	-	-	-	-	-	-	-	-	-	-	-	-	-	-	-	-	-	-	-	-	-	-	-	-	-	-	-	-	-	-	-	-	-
		-	-	-	-	-	-	-	-	-	-	-	-	-	-	-	-	-	-	-	-	-	-	-	-	-	-	-	-	-	-	-	-	-	-	-	-	-	-	-	-	-	-	-	-	-	-	-	-	-	-	-
HTB27	Histone H2B	-	-	-	-	-	-	-	-	-	-	-	-	181233	401207	-	-	-	-	-	-	-	-	-	-	LmjF19.0030	233239	-	-	-	-	-	-	-	-	-	-	-	-	-	-	-	-	-	-	-	-	-	-	-	-	-
		-	-	-	-	-	-	-	-	-	-	-	-	181232	-	-	-	-	-	-	-	-	-	-	-	-	-	-	-	-	-	-	-	-	-	-	-	-	-	-	-	-	-	-	-	-	-	-	-	-	-	-
		-	-	-	-	-	-	-	-	-	-	-	-	181231	-	-	-	-	-	-	-	-	-	-	-	-	-	-	-	-	-	-	-	-	-	-	-	-	-	-	-	-	-	-	-	-	-	-	-	-	-	-
		-	-	-	-	-	-	-	-	-	-	-	-	181230	-	-	-	-	-	-	-	-	-	-	-	-	-	-	-	-	-	-	-	-	-	-	-	-	-	-	-	-	-	-	-	-	-	-	-	-	-	-
		-	-	-	-	-	-	-	-	-	-	-	-	-	-	-	-	-	-	-	-	-	-	-	-	-	-	-	-	-	-	-	-	-	-	-	-	-	-	-	-	-	-	-	-	-	-	-	-	-	-	-
		-	-	-	-	-	-	-	-	-	-	-	-	181222	-	-	-	-	-	-	-	-	-	-	-	-	-	-	-	-	-	-	-	-	-	-	-	-	-	-	-	-	-	-	-	-	-	-	-	-	-	-
		-	-	-	-	-	-	-	-	-	-	-	-	181218	-	-	-	-	-	-	-	-	-	-	-	-	-	-	-	-	-	-	-	-	-	-	-	-	-	-	-	-	-	-	-	-	-	-	-	-	-	-
		-	-	-	-	-	-	-	-	-	-	-	-	181215	-	-	-	-	-	-	-	-	-	-	-	-	-	-	-	-	-	-	-	-	-	-	-	-	-	-	-	-	-	-	-	-	-	-	-	-	-	-
		-	-	-	-	-	-	-	-	-	-	-	-	181213	-	-	-	-	-	-	-	-	-	-	-	-	-	-	-	-	-	-	-	-	-	-	-	-	-	-	-	-	-	-	-	-	-	-	-	-	-	-
		-	-	-	-	-	-	-	-	-	-	-	-	181212	-	-	-	-	-	-	-	-	-	-	-	-	-	-	-	-	-	-	-	-	-	-	-	-	-	-	-	-	-	-	-	-	-	-	-	-	-	-
		-	-	-	-	-	-	-	-	-	-	-	-	181211	-	-	-	-	-	-	-	-	-	-	-	-	-	-	-	-	-	-	-	-	-	-	-	-	-	-	-	-	-	-	-	-	-	-	-	-	-	-
		-	-	-	-	-	-	-	-	-	-	-	-	181207	-	-	-	-	-	-	-	-	-	-	-	-	-	-	-	-	-	-	-	-	-	-	-	-	-	-	-	-	-	-	-	-	-	-	-	-	-	-
		-	-	-	-	-	-	-	-	-	-	-	-	181206	-	-	-	-	-	-	-	-	-	-	-	-	-	-	-	-	-	-	-	-	-	-	-	-	-	-	-	-	-	-	-	-	-	-	-	-	-	-
		-	-	-	-	-	-	-	-	-	-	-	-	181203	-	-	-	-	-	-	-	-	-	-	-	-	-	-	-	-	-	-	-	-	-	-	-	-	-	-	-	-	-	-	-	-	-	-	-	-	-	-
		-	-	-	-	-	-	-	-	-	-	-	-	181202	-	-	-	-	-	-	-	-	-	-	-	-	-	-	-	-	-	-	-	-	-	-	-	-	-	-	-	-	-	-	-	-	-	-	-	-	-	-
		-	-	-	-	-	-	-	-	-	-	-	-	-	-	-	-	-	-	-	-	-	-	-	-	-	-	-	-	-	-	-	-	-	-	-	-	-	-	-	-	-	-	-	-	-	-	-	-	-	-	-
		-	-	-	-	-	-	-	-	-	-	-	-	-	-	-	-	-	-	-	-	-	-	-	-	-	-	-	-	-	-	-	-	-	-	-	-	-	-	-	-	-	-	-	-	-	-	-	-	-	-	-
		-	-	-	-	-	-	-	-	-	-	-	-	181198	-	-	-	-	-	-	-	-	-	-	-	-	-	-	-	-	-	-	-	-	-	-	-	-	-	-	-	-	-	-	-	-	-	-	-	-	-	-
		-	-	-	-	-	-	-	-	-	-	-	119250	181195	235376	-	-	-	-	-	-	-	-	-	-	-	-	-	-	-	-	Os02g38020.2	-	-	-	-	-	-	-	-	-	-	-	SINFRUP00000136265	-	-	-	-	-	-	-	-
		-	-	-	-	-	-	-	-	-	-	-	-	181192	-	-	-	-	-	-	-	-	-	-	-	-	-	-	-	-	-	-	-	-	-	-	-	-	-	-	-	-	-	-	-	-	-	-	-	-	-	-
		-	-	-	-	-	-	-	-	-	-	-	-	181191	-	-	-	-	-	-	-	-	-	-	-	-	-	-	-	-	-	-	-	-	-	-	-	-	-	-	-	-	-	-	-	-	-	-	-	-	-	-
		-	-	-	-	-	-	-	-	-	-	-	-	181190	-	-	-	-	-	-	-	-	-	-	-	-	-	-	-	-	-	-	-	-	-	-	-	-	-	-	-	-	-	-	-	-	-	-	-	-	-	-
CLR14	Coxiella-Like Repeat protein	-	-	-	-	-	-	-	-	-	-	-	-	181188	-	-	-	-	-	-	-	-	-	-	-	-	-	-	-	-	-	-	-	-	-	-	-	-	-	-	-	-	-	-	-	-	-	-	-	-	-	-
		-	-	-	-	-	-	-	-	-	-	-	-	181186	-	-	-	-	-	-	-	-	-	-	-	-	-	-	-	-	-	-	-	-	-	-	-	-	-	-	-	-	-	-	-	-	-	-	-	-	-	-
		-	-	-	-	-	-	-	-	-	-	-	-	181182	-	-	-	-	-	-	-	-	-	-	-	-	-	-	-	-	-	-	-	-	GSPATP00028813001	-	-	-	-	-	-	-	-	-	-	-	-	-	-	-	-	-	-
		-	-	-	-	-	-	-	-	-	-	-	-	181181	-	-	-	-	-	-	-	-	-	-	-	-	-	-	-	-	-	-	-	-	-	-	-	-	-	-	-	-	-	-	-	-	-	-	-	-	-	-
		-	-	-	-	-	-	-	-	-	-	-	-	181176	-	-	-	-	-	-	-	-	-	-	-	-	-	-	-	-	-	-	-	-	-	-	-	-	-	-	-	-	-	-	-	-	-	-	-	-	-	-
		-	-	-	-	-	-	-	-	-	-	-	-	181173	-	-	-	-	-	-	-	-	-	-	-	-	-	-	-	-	-	-	-	19719	-	-	-	119646	-	-	RO3G_16319.1	-	-	-	-	-	-	-	-	-	86088.m00376	-	-
		-	-	-	-	-	-	-	-	-	-	-	-	181170	-	-	-	-	-	-	-	-	-	-	-	-	-	-	-	-	-	-	-	-	-	-	-	-	-	-	-	-	-	-	-	-	-	-	-	-	-	-
		-	-	-	-	-	-	-	-	-	-	-	-	181168	-	-	-	-	-	-	-	-	-	-	-	-	-	-	-	-	-	-	-	-	-	-	-	-	-	-	-	-	-	-	-	-	-	-	-	-	-	-
		-	-	-	-	-	-	-	-	-	-	-	-	181162	-	-	-	-	-	-	-	-	-	-	-	-	-	-	-	-	-	-	-	-	-	-	-	-	-	-	-	-	-	-	-	-	-	-	-	-	-	-
		-	-	-	-	-	-	-	-	-	-	-	-	181161	-	-	-	-	-	-	-	-	-	-	-	-	-	-	-	-	-	-	-	-	-	-	-	-	-	-	-	-	-	-	-	-	-	-	-	-	-	-
		-	-	-	-	-	-	-	-	-	-	-	-	181156	-	-	-	-	-	-	-	-	-	-	-	-	-	-	-	-	-	-	-	-	-	-	-	-	-	-	-	-	-	-	-	-	-	-	-	-	-	-
		-	-	-	-	-	-	-	-	-	-	-	-	181152	-	-	-	-	-	-	-	-	-	-	-	-	-	-	-	-	-	-	-	-	-	-	-	-	-	-	-	-	-	-	-	-	-	-	-	-	-	-
		-	-	-	-	-	-	-	-	-	-	-	-	181151	-	-	-	-	-	-	-	-	-	-	-	-	-	-	-	-	-	-	-	-	-	-	-	-	-	-	-	-	-	-	-	-	-	-	-	-	-	-
		-	-	-	-	-	-	-	-	-	-	-	-	181150	-	-	-	-	-	-	-	-	-	-	-	-	-	-	-	-	-	-	-	-	-	-	-	-	-	-	-	-	-	-	-	-	-	-	-	-	-	-
		-	-	-	-	-	-	-	-	-	-	-	-	181148	-	-	-	-	-	-	-	-	-	-	-	-	-	-	-	-	-	-	-	-	-	-	-	-	-	-	-	-	-	-	-	-	-	-	-	-	-	-
		-	-	-	-	-	-	-	-	-	-	-	-	-	-	-	-	-	-	-	-	-	-	-	-	-	-	-	-	-	-	-	-	-	-	-	-	-	-	-	-	-	-	-	-	-	-	-	-	-	-	-
		-	-	-	-	-	-	-	-	-	-	-	-	181146	-	-	-	-	-	-	-	-	-	-	-	-	-	-	-	-	-	-	-	-	-	-	-	-	-	-	-	-	-	-	-	-	-	-	-	-	-	-
IF3D	Eukaryotic translation initiation factor 3 subunit 7	-	+	-	-	-	-	GB13888-PA	AT4G20980.3	31918	BDEG_03230	CE00291	156498	181144	288739	cgd2_270	CMO260C	OTTDARP00000019642	DDB0217568	-	-	-	ENSGALP00000020439	-	OTTHUMP00000028733	-	177433	19728	80297	235689	NCU07380	Os05g49150.1	18758	-	13088	12801	141581	PF10_0077	758889	RO3G_11326.1	-	SPAC637.07	GLEAN3_06773	SINFRUP00000167613	25.m00405	-	TA18225	611.m00038	20515	82360.m00307	Tb927.6.4370	UM01321.1
	plant calcium-transporting ATPase 2	-	-	-	-	-	-	-	-	-	-	-	-	-	-	-	-	-	-	-	-	-	-	-	-	-	-	-	-	-	-	-	-	-	-	-	-	-	-	-	-	-	-	-	-	-	-	-	-	-	-	-
		-	-	-	-	-	-	-	-	-	-	-	-	181141	-	-	-	-	-	-	-	-	-	-	-	-	-	-	-	-	-	-	-	-	-	-	-	-	-	-	-	-	-	-	-	-	-	-	-	-	-	-
		-	-	-	-	-	-	-	-	-	-	-	-	181140	-	-	-	-	-	-	-	-	-	-	-	-	-	-	-	-	-	-	-	-	-	-	-	-	-	-	-	-	-	-	-	-	-	-	-	-	-	-
		-	-	-	-	-	-	-	-	-	-	-	-	181139	-	-	-	-	-	-	-	-	-	-	-	-	-	-	-	-	-	-	-	-	-	-	-	-	-	-	-	-	-	-	-	-	-	-	-	-	-	-
		-	-	-	-	-	-	-	-	-	-	-	-	181138	-	-	-	-	-	-	-	-	-	-	-	-	-	-	-	-	-	-	-	-	-	-	-	-	-	-	-	-	-	-	-	-	-	-	-	-	-	-
		-	-	-	-	-	-	-	-	-	-	-	-	181137	-	-	-	-	-	-	-	-	-	-	-	-	-	-	-	-	-	-	-	-	-	-	-	-	-	-	-	-	-	-	-	-	-	-	-	-	-	-
		-	-	-	-	-	-	-	-	-	-	-	-	-	-	-	-	-	-	-	-	-	-	-	-	-	-	-	-	-	-	-	-	-	-	-	-	-	-	-	-	-	-	-	-	-	-	-	-	-	-	-
		-	-	-	-	-	-	-	-	-	-	-	-	181134	-	-	-	-	-	-	-	-	-	-	-	-	-	-	-	-	-	-	-	-	-	-	-	-	-	-	-	-	-	-	-	-	-	-	-	-	-	-
		-	-	-	-	-	-	-	-	-	-	-	-	181133	-	-	-	-	-	-	-	-	-	-	-	-	-	-	-	-	-	-	-	-	-	-	-	-	-	-	-	-	-	-	-	-	-	-	-	-	-	-
		-	-	-	-	-	-	-	-	-	-	-	-	181126	-	-	-	-	-	-	-	-	-	-	-	-	-	-	-	-	-	-	-	-	-	-	-	-	-	-	-	-	-	-	-	-	-	-	-	-	-	-
		-	-	-	-	-	-	-	-	-	-	-	-	181121	-	-	-	-	-	-	-	-	-	-	-	-	-	-	-	-	-	-	-	-	-	-	-	-	-	-	-	-	-	-	-	-	-	-	-	-	-	-
		-	-	-	-	-	-	-	-	-	-	-	-	181120	-	-	-	-	-	-	-	-	-	-	-	-	-	-	-	-	-	-	-	-	-	-	-	-	-	-	-	-	-	-	-	-	-	-	-	-	-	-
		-	-	-	-	-	-	-	-	-	-	-	-	181119	-	-	-	-	-	-	-	-	-	-	-	-	-	-	-	-	-	-	-	-	-	-	-	-	-	-	-	-	-	-	-	-	-	-	-	-	-	-
		-	-	-	-	-	-	-	-	-	-	-	-	181114	-	-	-	-	-	-	-	-	-	-	-	-	-	-	-	-	-	-	-	-	-	-	-	-	-	-	-	-	-	-	-	-	-	-	-	-	-	-
		-	-	-	-	-	-	-	-	-	-	-	-	181112	-	-	-	-	-	-	-	-	-	-	-	-	-	-	-	-	-	-	-	-	-	-	-	-	-	-	-	-	-	-	-	-	-	-	-	-	-	-
STPK6	Serine/Threonine Protein Kinase Homolog 6, putative	-	-	-	-	-	-	-	-	-	-	-	-	181109	-	-	-	-	-	-	-	-	-	-	-	-	-	-	-	-	-	-	-	-	-	-	-	-	-	-	-	-	-	-	-	-	-	-	-	-	-	-
		-	-	-	-	-	-	-	AT3G18524.1	-	-	-	-	181107	-	-	-	-	-	-	-	-	-	-	-	-	-	-	-	-	-	Os05g19270.1	35193	-	-	-	-	-	569858	-	-	-	-	-	-	-	-	-	-	-	-	-
		-	-	-	-	-	-	-	-	-	-	-	-	181105	-	-	-	-	-	-	-	-	-	-	-	-	-	-	-	-	-	-	-	-	-	-	-	-	-	-	-	-	-	-	-	-	-	-	-	-	-	-
		-	-	-	-	-	-	-	-	-	-	-	-	181104	-	-	-	-	-	-	-	-	-	-	-	-	-	-	-	-	-	-	-	-	-	-	-	-	-	-	-	-	-	-	-	-	-	-	-	-	-	-
		-	-	-	-	-	-	-	-	-	-	-	-	181099	-	-	-	-	-	-	-	-	-	-	-	-	-	-	-	-	-	-	-	-	-	-	-	-	-	-	-	-	-	-	-	-	-	-	-	-	-	-
		-	-	-	-	-	-	-	-	-	-	-	-	181098	-	-	-	-	-	-	-	-	-	-	-	-	-	-	-	-	-	-	-	-	-	-	-	-	-	-	-	-	-	-	-	-	-	-	-	-	-	-
		-	-	-	-	-	-	-	-	-	-	-	-	181097	-	-	-	-	-	-	-	-	-	-	-	-	-	-	-	-	-	-	-	-	-	-	-	-	-	-	-	-	-	-	-	-	-	-	-	-	-	-
		-	-	-	-	-	-	-	-	-	-	-	-	181094	-	-	-	-	-	-	-	-	-	-	-	-	-	-	-	-	-	-	-	-	-	-	-	-	-	-	-	-	-	-	-	-	-	-	-	-	-	-
		-	-	-	-	-	-	-	-	-	-	-	-	-	-	-	-	-	-	-	-	-	-	-	-	-	-	-	-	-	-	-	-	-	-	-	-	-	-	-	-	-	-	-	-	-	-	-	-	-	-	-
		-	-	-	-	-	-	-	-	-	-	-	-	181091	-	-	-	-	-	-	-	-	-	-	-	-	-	-	-	-	-	-	-	-	-	-	-	-	-	-	-	-	-	-	-	-	-	-	-	-	-	-
		-	-	-	-	-	-	-	-	-	-	-	-	181089	-	-	-	-	-	-	-	-	-	-	-	-	-	-	-	-	-	-	-	-	-	-	-	-	-	-	-	-	-	-	-	-	-	-	-	-	-	-
		-	-	-	-	-	-	-	-	-	-	-	-	181088	-	-	-	-	-	-	-	-	-	-	-	-	-	-	-	-	-	-	-	-	-	-	-	-	-	-	-	-	-	-	-	-	-	-	-	-	-	-
		-	-	-	-	-	-	-	-	-	-	-	-	181085	-	-	-	-	-	-	-	-	-	-	-	-	-	-	-	-	-	-	-	-	-	-	-	-	-	-	-	-	-	-	-	-	-	-	-	-	-	-
		-	-	-	-	-	-	-	-	-	-	-	-	181084	-	-	-	-	-	-	-	-	-	-	-	-	-	-	-	-	-	-	-	-	-	-	-	-	-	-	-	-	-	-	-	-	-	-	-	-	-	-
		-	-	-	-	-	-	-	-	-	-	-	-	-	-	-	-	-	-	-	-	-	-	-	-	-	-	-	-	-	-	-	-	-	-	-	-	-	-	-	-	-	-	-	-	-	-	-	-	-	-	-
		-	-	-	-	-	-	-	-	-	-	-	-	181080	-	-	-	-	-	-	-	-	-	-	-	-	-	-	-	-	-	-	-	-	-	-	-	-	-	-	-	-	-	-	-	-	-	-	-	-	-	-
		-	-	-	-	-	-	-	-	-	-	-	-	-	-	-	-	-	-	-	-	-	-	-	-	-	-	-	-	-	-	-	-	-	-	-	-	-	-	-	-	-	-	-	-	-	-	-	-	-	-	-
		-	-	-	-	-	-	-	-	-	-	-	-	181075	-	-	-	-	-	-	-	-	-	-	-	-	-	-	-	-	-	-	-	-	-	-	-	-	-	-	-	-	-	-	-	-	-	-	-	-	-	-
		-	-	-	-	-	-	-	-	-	-	-	-	181074	-	-	-	-	-	-	-	-	-	-	-	-	-	-	-	-	-	-	-	32597	-	-	-	-	-	-	-	-	-	-	-	-	-	-	-	-	-	-
		-	-	-	-	-	-	-	-	-	-	-	-	181071	-	-	-	-	-	-	-	-	-	-	-	-	-	-	-	-	-	-	-	-	-	-	-	-	-	-	-	-	-	-	-	-	-	-	-	-	-	-
		-	-	-	-	-	-	-	-	-	-	-	-	181068	-	-	-	-	-	-	-	-	-	-	-	-	-	-	-	-	-	-	-	-	-	-	-	-	-	-	-	-	-	-	-	-	-	-	-	-	-	-
		-	-	-	-	-	-	-	-	-	-	-	-	181062	-	-	-	-	-	-	-	-	-	-	-	-	-	-	-	126308	-	-	-	-	-	-	-	-	-	-	-	-	-	-	-	-	-	-	-	-	-	-
		-	-	-	-	-	-	-	-	-	-	-	-	181060	-	-	-	-	-	-	-	-	-	-	-	-	-	-	-	-	-	-	-	-	-	-	-	-	-	-	-	-	-	-	-	-	-	-	-	-	-	-
		-	-	-	-	-	-	-	-	-	-	-	-	181059	-	-	-	-	-	-	-	-	-	-	-	-	-	-	-	-	-	-	-	-	-	-	-	-	-	-	-	-	-	-	-	-	-	-	-	-	-	-
		-	+	+	-	-	-	GB17050-PA	AT5G49930.1	-	BDEG_01255	CE33002	151175	181058	250004	cgd5_1780	CMK242C	-	DDB0188474	-	19074389	231.m00078	ENSGALP00000020002	GL50803_4043	NP_004704.2	LmjF09.0940	127397	628	81846	10072	NCU09191	Os12g37720.1	-	GSPATP00006421001	42643	135179	131857	PFL0130c	765880	RO3G_05884.1	YPL009C	SPCC132.01c	-	SINFRUP00000176370	14.m00378	270000	TA04725	33.m00313	-	86827.m00511	Tb11.01.4630	-
		-	-	-	-	-	-	-	-	-	-	-	-	181057	-	-	-	-	-	-	-	-	-	-	-	-	-	-	-	-	-	-	-	-	-	-	-	-	-	-	-	-	-	-	-	-	-	-	-	-	-	-
		-	-	-	-	-	-	-	-	-	-	-	-	-	-	-	-	-	-	-	-	-	-	-	-	-	-	-	-	-	-	-	-	-	-	-	-	-	-	-	-	-	-	-	-	-	-	-	-	-	-	-
		-	-	-	-	-	-	-	-	-	-	-	-	181054	-	-	-	-	-	-	-	-	-	-	-	-	-	-	-	-	-	-	-	-	-	-	-	-	-	-	-	-	-	-	-	-	-	-	-	-	-	-
		-	-	-	-	-	-	-	-	-	-	-	-	181051	-	-	-	-	-	-	-	-	-	-	-	-	-	-	-	-	-	-	-	-	-	-	-	-	-	-	-	-	-	-	-	-	-	-	-	-	-	-
		-	-	-	-	-	-	-	-	-	-	-	-	181050	-	-	-	-	-	-	-	-	-	-	-	-	-	-	-	-	-	-	-	-	-	-	-	-	-	-	-	-	-	-	-	-	-	-	-	-	-	-
		-	-	-	-	-	-	-	-	-	-	-	-	181049	-	-	-	-	-	-	-	-	-	-	-	-	-	-	-	-	-	-	-	-	-	-	-	-	-	-	-	-	-	-	-	-	-	-	-	-	-	-
		-	-	-	-	-	-	-	-	-	-	-	-	181044	-	-	-	-	-	-	-	-	-	-	-	-	-	-	-	-	-	-	-	-	-	-	-	-	-	-	-	-	-	-	-	-	-	-	-	-	-	-
		-	-	-	-	-	-	-	-	-	-	-	-	181043	-	-	-	-	-	-	-	-	-	-	-	-	-	-	-	-	-	-	-	-	-	-	-	-	-	-	-	-	-	-	-	-	-	-	-	-	-	-
		-	-	-	-	-	-	-	-	-	-	-	-	181040	-	-	-	-	-	-	-	-	-	-	-	-	-	-	-	-	-	-	-	-	-	-	-	-	-	-	-	-	-	-	-	-	-	-	-	-	-	-
		-	-	-	-	-	-	-	-	-	-	-	-	181039	-	-	-	-	-	-	-	-	-	-	-	-	-	-	-	-	-	-	-	-	-	-	-	-	-	-	-	-	-	-	-	-	-	-	-	-	-	-
		-	-	-	-	-	-	-	-	-	-	-	-	181038	-	-	-	-	-	-	-	-	-	-	-	-	-	-	-	-	-	-	-	-	-	-	-	-	-	-	-	-	-	-	-	-	-	-	-	-	-	-
		-	-	-	-	-	-	-	-	-	-	-	-	-	-	-	-	-	-	-	-	-	-	-	-	-	-	-	-	-	-	-	-	-	-	-	-	-	-	-	-	-	-	-	-	-	-	-	-	-	-	-
		-	-	-	-	-	-	-	-	-	-	-	-	181036	-	-	-	-	-	-	-	-	-	-	-	-	-	-	-	-	-	-	-	-	-	-	-	-	-	-	-	-	-	-	-	-	-	-	-	-	-	-
		-	-	-	-	-	-	-	-	-	-	-	-	181035	-	-	-	-	-	-	-	-	-	-	-	-	-	-	-	-	-	-	-	-	-	-	-	-	-	-	-	-	-	-	-	-	-	-	-	-	-	-
		-	-	-	-	-	-	-	-	-	-	-	-	181028	-	-	-	-	-	-	-	-	-	-	-	-	-	-	-	-	-	-	-	-	-	-	-	-	-	-	-	-	-	-	-	-	-	-	-	-	-	-
		-	-	-	-	-	-	-	-	-	-	-	-	181027	-	-	-	-	-	-	-	-	-	-	-	-	-	-	-	-	-	-	-	-	-	-	-	-	-	-	-	-	-	-	-	-	-	-	-	-	-	-
		-	-	-	-	-	-	-	-	-	-	-	-	181025	-	-	-	-	-	-	-	-	-	-	-	-	-	-	-	-	-	-	-	-	-	-	-	-	-	-	-	-	-	-	-	-	-	-	-	-	-	-
		-	-	-	-	-	-	-	-	-	-	-	-	181023	-	-	-	-	-	-	-	-	-	-	-	-	-	-	-	-	-	-	-	-	-	-	-	-	-	-	-	-	-	-	-	-	-	-	-	-	-	-
		-	-	-	-	-	-	-	-	-	-	-	-	181022	-	-	-	-	-	-	-	-	-	-	-	-	-	-	-	-	-	-	-	-	-	-	-	-	-	-	-	-	-	-	-	-	-	-	-	-	-	-
		-	-	-	-	-	-	-	-	-	-	-	-	181019	-	-	-	-	-	-	-	-	-	-	-	-	-	-	-	-	-	-	-	-	-	-	-	-	-	-	-	-	-	-	-	-	-	-	-	-	-	-
		-	-	-	-	-	-	-	-	-	-	-	-	181017	-	-	-	-	-	-	-	-	-	-	-	-	-	-	-	-	-	-	-	-	-	-	-	-	-	-	-	-	-	-	-	-	-	-	-	-	-	-
		-	-	-	-	-	-	-	-	-	-	-	-	-	-	-	-	-	-	-	-	-	-	-	-	-	-	-	-	-	-	-	-	-	-	-	-	-	-	-	-	-	-	-	-	-	-	-	-	-	-	-
		-	-	-	-	-	-	-	-	-	-	-	-	181014	-	-	-	-	-	-	-	-	-	-	-	-	-	-	-	-	-	-	-	-	-	-	-	-	-	-	-	-	-	-	-	-	-	-	-	-	-	-
		-	-	-	-	-	-	-	-	-	-	-	-	181012	-	-	-	-	-	-	-	-	-	-	-	-	-	-	-	-	-	-	-	-	-	-	-	-	-	-	-	-	-	-	-	-	-	-	-	-	-	-
		-	-	-	-	-	-	-	-	-	-	-	-	181011	-	-	-	-	-	-	-	-	-	-	-	-	-	-	-	-	-	-	-	-	-	-	-	-	-	-	-	-	-	-	-	-	-	-	-	-	-	-
		-	-	-	-	-	-	-	-	-	-	-	-	181010	-	-	-	-	-	-	-	-	-	-	-	-	-	-	-	-	-	-	-	-	-	-	-	-	-	-	-	-	-	-	-	-	-	-	-	-	-	-
CLR5	Coxiella-Like Repeat protein	-	-	-	-	-	-	-	-	-	-	-	-	181009	-	-	-	-	-	-	-	-	-	-	-	-	-	-	-	-	-	-	-	-	-	-	-	-	-	-	-	-	-	-	-	-	-	-	-	-	-	-
		-	-	-	-	-	-	-	-	-	-	-	-	181008	-	-	-	-	-	-	-	-	-	-	-	-	-	-	-	-	-	-	-	-	-	-	-	-	-	-	-	-	-	-	-	-	-	-	-	-	-	-
		-	-	-	-	-	-	-	-	-	-	-	-	181007	-	-	-	-	-	-	-	-	-	-	-	-	-	-	-	-	-	-	-	-	-	-	-	-	-	-	-	-	-	-	-	-	-	-	-	-	-	-
		-	-	-	-	-	-	-	-	-	-	-	-	181006	-	-	-	-	-	-	-	-	-	-	-	-	-	-	-	-	-	-	-	-	-	-	-	-	-	-	-	-	-	-	-	-	-	-	-	-	-	-
		-	-	-	-	-	-	-	-	-	-	-	-	181005	-	-	-	-	-	-	-	-	-	-	-	-	-	-	-	-	-	-	-	-	-	-	-	-	-	-	-	-	-	-	-	-	-	-	-	-	-	-
		-	-	-	-	-	-	-	-	-	-	-	-	181004	-	-	-	-	-	-	-	-	-	-	-	-	-	-	-	-	-	-	-	-	-	-	-	-	-	-	-	-	-	-	-	-	-	-	-	-	-	-
		-	-	-	-	-	-	-	-	-	-	-	-	181003	-	-	-	-	-	-	-	-	-	-	-	-	-	-	-	-	-	-	-	-	-	-	-	-	-	-	-	-	-	-	-	-	-	-	-	-	-	-
		-	-	-	-	-	-	-	-	-	-	-	-	180995	-	-	-	-	-	-	-	-	-	-	-	-	-	-	-	-	-	-	-	-	-	-	-	-	-	-	-	-	-	-	-	-	-	-	-	-	-	-
		-	-	-	-	-	-	-	-	-	-	-	-	180994	-	-	-	-	-	-	-	-	-	-	-	-	-	-	-	-	-	-	-	-	-	-	-	-	-	-	-	-	-	-	-	-	-	-	-	-	-	-
		-	-	-	-	-	-	-	-	-	-	-	-	180990	-	-	-	-	-	-	-	-	-	-	-	-	-	-	-	-	-	-	-	-	-	-	-	-	-	-	-	-	-	-	-	-	-	-	-	-	-	-

		-	-	-	-	-	-	-	-	-	-	-	-	180986	-	-	-	-	-	-	-	-	-	-	-	-	-	-	-	-	-	-	-	-	-	-	-	-	-	-	-	-	-	-	-	-	-	-	-	-	-	-
		-	-	-	-	-	-	-	-	-	-	-	-	180984	-	-	-	-	-	-	-	-	-	-	-	-	-	-	-	-	-	-	-	-	-	-	-	-	-	-	-	-	-	-	-	-	-	-	-	-	-	-
		-	-	-	-	-	-	-	-	-	-	-	-	180982	-	-	-	-	-	-	-	-	-	-	-	-	-	-	-	-	-	-	-	-	-	-	-	-	-	-	-	-	-	-	-	-	-	-	-	-	-	-
		-	-	-	-	-	-	-	-	-	-	-	-	180981	-	-	-	-	-	-	-	-	-	-	-	-	-	-	-	-	-	-	-	-	-	-	-	-	-	-	-	-	-	-	-	-	-	-	-	-	-	-
		-	-	-	-	-	-	-	-	-	-	-	-	180980	-	-	-	-	-	-	-	-	-	-	-	-	-	-	-	-	-	-	-	-	-	-	-	-	-	-	-	-	-	-	-	-	-	-	-	-	-	-
		-	-	-	-	-	-	-	-	-	-	-	-	180976	-	-	-	-	-	-	-	-	-	-	-	-	-	-	-	-	-	-	-	-	-	-	-	-	-	-	-	-	-	-	-	-	-	-	-	-	-	-
CLR20	Coxiella-Like Repeat protein	-	-	-	-	-	-	-	-	-	-	-	-	180972	-	-	-	-	-	-	-	-	-	-	-	-	-	-	-	-	-	-	-	-	-	-	-	-	-	-	-	-	-	-	-	-	-	-	-	-	-	-
		-	-	-	-	-	-	-	-	-	-	-	-	180967	-	-	-	-	-	-	-	-	-	-	-	-	-	-	-	-	-	-	-	-	-	-	-	-	-	-	-	-	-	-	-	-	-	-	-	-	-	-
		-	-	-	-	-	-	-	-	-	-	-	-	180962	-	-	-	-	-	-	-	-	-	-	-	-	-	-	-	-	-	-	-	-	-	-	-	-	-	-	-	-	-	-	-	-	-	-	-	-	-	-
		-	-	-	-	-	-	-	-	-	-	-	-	180960	-	-	-	-	-	-	-	-	-	-	-	-	-	-	-	-	-	-	-	-	-	-	-	-	-	-	-	-	-	-	-	-	-	-	-	-	-	-
		-	-	-	-	-	-	-	-	-	-	-	-	180953	-	-	-	-	-	-	-	-	-	-	-	-	-	-	-	-	-	-	-	-	-	-	-	-	-	-	-	-	-	-	-	-	-	-	-	-	-	-
		-	-	-	-	-	-	-	-	-	-	-	-	180952	-	-	-	-	-	-	-	-	-	-	-	-	-	-	-	-	-	-	-	-	-	-	-	-	-	-	-	-	-	-	-	-	-	-	-	-	-	-
		-	-	-	-	-	-	-	-	-	-	-	-	180950	-	-	-	-	-	-	-	-	-	-	-	-	-	-	-	-	-	-	-	-	-	-	-	-	-	-	-	-	-	-	-	-	-	-	-	-	-	-
		-	-	-	-	-	-	-	-	-	-	-	-	180946	-	-	-	-	-	-	-	-	-	-	-	-	-	-	-	-	-	-	-	-	-	-	-	-	-	-	-	-	-	-	-	-	-	-	-	-	-	-
		-	-	-	-	-	-	-	-	-	-	-	-	180941	-	-	-	-	-	-	-	-	-	-	-	-	-	-	-	-	-	-	-	-	-	-	-	-	-	-	-	-	-	-	-	-	-	-	-	-	-	-
		-	-	-	-	-	-	-	-	-	-	-	-	180938	-	-	-	-	-	-	-	-	-	-	-	-	-	-	-	-	-	-	-	-	-	-	-	-	-	-	-	-	-	-	-	-	-	-	-	-	-	-
		-	-	-	-	-	-	-	-	-	-	-	-	180936	-	-	-	-	-	-	-	-	-	-	-	-	-	-	-	-	-	-	-	-	-	-	-	-	-	-	-	-	-	-	-	-	-	-	-	-	-	-
		-	-	-	-	-	-	-	-	-	-	-	-	180935	-	-	-	-	-	-	-	-	-	-	-	-	-	-	-	-	-	-	-	-	-	-	-	-	-	-	-	-	-	-	-	-	-	-	-	-	-	-
		-	-	-	-	-	-	-	-	-	-	-	-	180930	-	-	-	-	-	-	-	-	-	-	-	-	-	-	-	-	-	-	-	-	-	-	-	-	-	-	-	-	-	-	-	-	-	-	-	-	-	-
		-	-	-	-	-	-	-	-	-	-	-	-	180929	-	-	-	-	-	-	-	-	-	-	-	-	-	-	-	-	-	-	-	-	-	-	-	-	-	-	-	-	-	-	-	-	-	-	-	-	-	-
		-	-	-	-	-	-	-	-	-	-	-	-	180928	-	-	-	-	-	-	-	-	-	-	-	-	-	-	-	-	-	-	-	-	-	-	-	-	-	-	-	-	-	-	-	-	-	-	-	-	-	-
		-	-	-	-	-	-	-	-	-	-	-	-	-	-	-	-	-	-	-	-	-	-	-	-	-	-	-	-	-	-	-	-	-	-	-	-	-	-	-	-	-	-	-	-	-	-	-	-	-	-	-
		-	-	-	-	-	-	-	-	-	-	-	-	180922	-	-	-	-	-	-	-	-	-	-	-	-	-	-	-	-	-	-	-	-	-	-	-	-	-	-	-	-	-	-	-	-	-	-	-	-	-	-
		-	-	-	-	-	-	-	-	-	-	-	-	180916	-	-	-	-	-	-	-	-	-	-	-	-	-	-	-	-	-	-	-	-	-	-	-	-	-	-	-	-	-	-	-	-	-	-	-	-	-	-
		-	-	-	-	-	-	-	-	-	-	-	-	180915	-	-	-	-	-	-	-	-	-	-	-	-	-	-	-	-	-	-	-	-	-	-	-	-	-	-	-	-	-	-	-	-	-	-	-	-	-	-
		-	-	-	-	-	-	-	-	-	-	-	-	180913	-	-	-	-	-	-	-	-	-	-	-	-	-	-	-	-	-	-	-	-	-	-	-	-	-	-	-	-	-	-	-	-	-	-	-	-	-	-
		-	-	-	-	-	-	-	-	-	-	-	-	180911	-	-	-	-	-	-	-	-	-	-	-	-	-	-	-	-	-	-	-	-	-	-	-	-	-	-	-	-	-	-	-	-	-	-	-	-	-	-
		-	-	-	-	-	-	-	-	-	-	-	-	180910	-	-	-	-	-	-	-	-	-	-	-	-	-	-	-	-	-	-	-	-	-	-	-	-	-	-	-	-	-	-	-	-	-	-	-	-	-	-
		-	-	-	-	-	-	-	-	-	-	-	-	180909	-	-	-	-	-	-	-	-	-	-	-	-	-	-	-	-	-	-	-	-	-	-	-	-	-	-	-	-	-	-	-	-	-	-	-	-	-	-
		-	-	-	-	-	-	-	-	-	-	-	-	180908	-	-	-	-	-	-	-	-	-	-	-	-	-	-	-	-	-	-	-	-	-	-	-	-	-	-	-	-	-	-	-	-	-	-	-	-	-	-
		-	-	-	-	-	-	-	-	-	-	-	-	180907	-	-	-	-	-	-	-	-	-	-	-	-	-	-	-	-	-	-	-	-	-	-	-	-	-	-	-	-	-	-	-	-	-	-	-	-	-	-
		-	-	-	-	-	-	-	-	-	-	-	-	180906	-	-	-	-	-	-	-	-	-	-	-	-	-	-	-	-	-	-	-	-	-	-	-	-	-	-	-	-	-	-	-	-	-	-	-	-	-	-
		-	+	-	-	-	-	-	AT1G62870.1	-	-	-	121688	180901	-	-	-	-	-	-	-	-	-	-	-	-	-	-	-	-	-	Os02g25230.1	-	-	-	55862	137168	-	784351	-	-	-	-	-	-	-	-	-	-	-	-	-
		-	-	-	-	-	-	-	-	-	-	-	-	180896	-	-	-	-	-	-	-	-	-	-	-	-	-	-	-	-	-	-	-	-	-	-	-	-	-	-	-	-	-	-	-	-	-	-	-	-	-	-
		-	-	-	-	-	-	-	-	-	-	-	-	180893	-	-	-	-	-	-	-	-	-	-	-	-	-	-	-	-	-	-	-	-	-	-	-	-	-	-	-	-	-	-	-	-	-	-	-	-	-	-
		-	-	-	-	-	-	-	-	-	-	-	-	180890	-	-	-	-	-	-	-	-	-	-	-	-	-	-	-	-	-	-	-	-	-	-	-	-	-	-	-	-	-	-	-	-	-	-	-	-	-	-
		-	-	-	-	-	-	-	-	-	-	-	-	180886	-	-	-	-	-	-	-	-	-	-	-	-	-	-	-	-	-	-	-	-	-	-	-	-	-	-	-	-	-	-	-	-	-	-	-	-	-	-
		-	-	-	-	-	-	-	-	-	-	-	-	180878	-	-	-	-	-	-	-	-	-	-	-	-	-	-	-	-	-	-	-	-	-	-	-	-	-	-	-	-	-	-	-	-	-	-	-	-	-	-
		-	-	-	-	-	-	-	-	-	-	-	-	180876	-	-	-	-	-	-	-	-	-	-	-	-	-	-	-	-	-	-	-	-	-	-	-	-	-	-	-	-	-	-	-	-	-	-	-	-	-	-
		-	-	-	-	-	-	-	-	-	-	-	-	180875	-	-	-	-	-	-	-	-	-	-	-	-	-	-	-	-	-	-	-	-	-	-	-	-	-	-	-	-	-	-	-	-	-	-	-	-	-	-
		-	-	-	-	-	-	-	-	-	-	-	-	180874	-	-	-	-	-	-	-	-	-	-	-	-	-	-	-	-	-	-	-	-	-	-	-	-	-	-	-	-	-	-	-	-	-	-	-	-	-	-
		-	-	-	-	-	-	-	-	-	BDEG_02022	-	-	180873	-	-	-	-	-	-	-	-	-	-	-	-	-	-	-	-	-	-	-	-	-	-	-	-	-	-	-	-	-	-	-	-	-	-	-	-	-	-
		-	-	-	-	-	-	-	-	-	-	-	-	-	-	-	-	-	-	-	-	-	-	-	-	-	-	-	-	-	-	-	-	-	-	-	-	-	-	-	-	-	-	-	-	-	-	-	-	-	-	-
		-	-	-	-	-	-	-	-	-	-	-	-	180869	-	-	-	-	-	-	-	-	-	-	-	-	-	-	-	-	-	-	-	-	-	-	-	-	-	-	-	-	-	-	-	-	-	-	-	-	-	-
		-	-	-	-	-	-	-	-	-	-	-	-	180867	-	-	-	-	-	-	-	-	-	-	-	-	-	-	-	-	-	-	-	-	-	-	-	-	-	-	-	-	-	-	-	-	-	-	-	-	-	-
		-	-	-	-	-	-	-	-	-	-	-	-	180865	-	-	-	-	-	-	-	-	-	-	-	-	-	-	-	-	-	-	-	-	-	-	-	-	-	-	-	-	-	-	-	-	-	-	-	-	-	-
		-	-	-	-	-	-	-	-	-	-	-	-	180860	-	-	-	-	-	-	-	-	-	-	-	-	-	-	-	-	-	-	-	-	-	-	-	-	-	-	-	-	-	-	-	-	-	-	-	-	-	-
		-	-	-	-	-	-	-	-	-	-	-	-	180858	-	-	-	-	-	-	-	-	-	-	-	-	-	-	-	-	-	-	-	-	-	-	-	-	-	-	-	-	-	-	-	-	-	-	-	-	-	-
		-	-	-	-	-	-	-	-	-	-	-	-	180852	-	-	-	-	-	-	-	-	-	-	-	-	-	-	-	-	-	-	-	-	-	-	-	-	-	-	-	-	-	-	-	-	-	-	-	-	-	-
		-	-	-	-	-	-	-	-	-	-	-	-	-	-	-	-	-	-	-	-	-	-	-	-	-	-	-	-	-	-	-	-	-	-	-	-	-	-	-	-	-	-	-	-	-	-	-	-	-	-	-
		-	-	-	-	-	-	-	-	-	-	-	-	180850	-	-	-	-	-	-	-	-	-	-	-	-	-	-	-	-	-	-	-	-	-	-	-	-	-	-	-	-	-	-	-	-	-	-	-	-	-	-
		-	-	-	-	-	-	-	-	-	-	-	-	-	-	-	-	-	-	-	-	-	-	-	-	-	-	-	-	-	-	-	-	-	-	-	-	-	-	-	-	-	-	-	-	-	-	-	-	-	-	-
CLR2	Coxiella-Like Repeat protein	-	-	-	-	-	-	-	-	-	-	-	-	180839	-	-	-	-	-	-	-	-	-	-	-	-	-	-	-	-	-	-	-	-	-	-	-	-	-	-	-	-	-	-	-	-	-	-	-	-	-	-
		-	-	-	-	-	-	-	-	-	-	-	-	180834	-	-	-	-	-	-	-	-	-	-	-	-	-	-	-	-	-	-	-	-	-	-	-	-	-	-	-	-	-	-	-	-	-	-	-	-	-	-
		-	-	-	-	-	-	-	-	-	-	CE36117	-	180826	-	-	-	-	-	-	-	-	-	-	-	-	-	-	-	-	-	-	23618	-	-	-	-	-	-	-	-	-	-	SINFRUP00000156380	-	-	-	-	-	-	-	-
		-	-	-	-	-	-	-	-	-	-	-	-	180821	-	-	-	-	-	-	-	-	-	-	-	-	-	-	-	-	-	-	-	-	-	-	-	-	-	-	-	-	-	-	-	-	-	-	-	-	-	-
HAV2	Histone H2A variant	-	-	-	-	-	-	-	-	-	-	-	-	180819	-	-	-	-	-	-	-	-	-	-	-	-	-	-	-	-	-	-	-	-	-	-	-	-	-	-	-	-	-	-	-	-	-	-	-	-	-	-
		-	+	-	-	-	-	-	-	-	-	-	-	180814	-	-	-	-	-	-	-	-	-	-	-	LmjF34.1240	-	-	-	215911	-	Os01g19430.1	-	-	-	-	-	-	576089	-	-	-	-	-	-	-	-	-	-	92043.m00118	-	-
		-	-	-	-	-	-	-	-	-	-	-	-	180812	-	-	-	-	-	-	-	-	-	-	-	-	-	-	-	-	-	-	-	-	-	-	-	-	-	-	-	-	-	-	-	-	-	-	-	-	-	-
		-	-	-	-	-	-	-	-	-	-	-	-	180810	-	-	-	-	-	-	-	-	-	-	-	-	-	-	-	-	-	-	-	-	-	-	-	-	-	-	-	-	-	-	-	-	-	-	-	-	-	-
		-	-	-	-	-	-	-	-	-	-	-	-	180809	-	-	-	-	-	-	-	-	-	-	-	-	-	-	-	-	-	Os07g06644.3	-	-	-	113286	-	-	412055	-	-	-	-	-	-	-	-	-	-	-	-	-
		-	-	-	-	-	-	-	-	-	-	-	-	180807	-	-	-	-	-	-	-	-	-	-	-	-	-	-	-	-	-	-	-	-	-	-	-	-	-	-	-	-	-	-	-	-	-	-	-	-	-	-
		-	-	-	-	-	-	-	-	-	-	-	-	180806	-	-	-	-	-	-	-	-	-	-	-	-	-	-	-	-	-	-	-	-	-	-	-	-	-	-	-	-	-	-	-	-	-	-	-	-	-	-
		-	-	-	-	-	-	-	-	-	-	-	-	180802	-	-	-	-	-	-	-	-	-	-	-	-	-	-	-	-	-	-	-	-	-	-	-	-	-	-	-	-	-	-	-	-	-	-	-	-	-	-
		-	+	-	-	-	-	-	AT3G25620.1	70485	-	-	-	180799	-	-	-	-	DDB0190309	-	-	-	-	-	-	-	-	-	-	-	-	Os11g07600.1	-	-	-	127566	-	PF14_0244	415196	-	-	-	-	-	-	-	-	583.m05692	-	-	-	UM04811.1
		-	-	-	-	-	-	-	-	-	-	-	-	180798	-	-	-	-	-	-	-	-	-	-	-	-	-	-	-	-	-	-	-	-	-	-	-	-	-	-	-	-	-	-	-	-	-	-	-	-	-	-
		-	-	-	-	-	-	-	-	-	-	-	-	180792	-	-	-	-	-	-	-	-	-	-	-	-	-	-	-	-	-	-	-	-	-	-	-	-	-	-	-	-	-	-	-	-	-	-	-	-	-	-
		-	-	-	-	-	-	-	-	-	-	-	-	180786	-	-	-	-	-	-	-	-	-	-	-	-	-	-	-	-	-	-	-	-	-	-	-	-	-	-	-	-	-	-	-	-	-	-	-	-	-	-
		-	-	-	-	-	-	-	-	-	-	-	-	180784	-	-	-	-	-	-	-	-	-	-	-	-	-	-	-	-	-	-	-	-	-	-	-	-	-	-	-	-	-	-	-	-	-	-	-	-	-	-
		-	-	-	-	-	-	-	-	-	-	-	-	180782	-	-	-	-	-	-	-	-	-	-	-	-	-	-	-	-	-	Os02g10350.1	-	-	-	-	-	-	-	-	-	-	-	-	-	-	-	-	-	-	-	-
		-	-	-	-	-	-	-	-	-	-	-	-	180779	-	-	-	-	-	-	-	-	-	-	-	LmjF32.3910	-	-	-	-	-	Os03g57100.1	-	-	-	-	-	-	-	-	-	-	-	-	-	-	-	-	-	-	-	UM02487.1
		-	-	-	-	-	-	-	-	-	-	-	-	180777	-	-	-	-	-	-	-	-	-	-	-	-	-	-	-	-	-	-	-	-	-	-	-	-	-	-	-	-	-	-	-	-	-	-	-	-	-	-
		-	-	-	-	-	-	-	-	-	-	-	-	180775	-	-	-	-	-	-	-	-	-	-	-	-	-	-	-	-	-	-	-	-	-	-	-	-	-	-	-	-	-	-	-	-	-	-	-	-	-	-
		-	-	-	-	-	-	-	-	-	-	-	-	180771	-	-	-	-	-	-	-	-	-	-	-	-	-	-	-	-	-	-	-	-	-	-	-	-	-	-	-	-	-	-	-	-	-	-	-	-	-	-
		-	-	-	-	-	-	-	-	-	-	-	-	180770	-	-	-	-	-	-	-	-	-	-	-	-	-	-	-	-	-	-	-	-	-	-	-	-	-	-	-	-	-	-	-	-	-	-	-	-	-	-
		-	-	-	-	-	-	-	-	-	-	-	-	180768	-	-	-	-	-	-	-	-	-	-	-	-	-	-	-	-	-	-	-	-	-	-	-	-	-	RO3G_01017.1	-	-	-	-	-	-	-	-	-	-	-	-
		-	-	-	-	-	-	-	-	-	-	-	-	180767	-	-	-	-	-	-	-	-	-	-	-	-	-	-	-	-	-	-	-	-	-	-	-	-	-	-	-	-	-	-	-	-	-	-	-	-	-	-
		-	-	-	-	-	-	-	-	-	-	-	-	180766	-	-	-	-	-	-	-	-	-	-	-	-	-	-	-	-	-	-	-	-	-	-	-	-	-	-	-	-	-	-	-	-	-	-	-	-	-	-
		-	-	-	-	-	-	-	-	-	-	-	-	180761	-	-	-	-	-	-	-	-	-	-	-	-	-	-	-	-	-	-	-	-	-	-	-	-	-	-	-	-	-	-	-	-	-	-	-	-	-	-
		-	-	-	-	-	-	-	-	-	-	-	-	180745	-	-	-	-	-	-	-	-	-	-	-	-	-	-	-	-	-	-	-	-	-	-	-	-	-	-	-	-	-	-	-	-	-	-	-	-	-	-
		-	-	-	-	-	-	-	-	-	-	-	-	180740	-	-	-	-	-	-	-	-	-	-	-	-	-	-	-	-	-	-	-	-	-	-	-	-	-	-	-	-	-	-	-	-	-	-	-	-	-	-
		-	-	-	-	-	-	-	-	-	-	-	-	180739	-	-	-	-	-	-	-	-	-	-	-	-	-	-	-	-	-	-	-	-	-	-	-	-	-	-	-	-	-	-	-	-	-	-	-	-	-	-
		-	-	-	-	-	-	-	-	-	-	-	-	180737	-	-	-	-	-	-	-	-	-	-	-	-	-	-	-	-	-	-	-	-	-	-	-	-	-	-	-	-	-	-	-	-	-	-	-	-	-	-
		-	-	-	-	-	-	-	-	-	-	-	-	180728	-	-	-	-	-	-	-	-	-	-	-	-	-	-	-	-	-	-	-	-	-	-	-	-	-	-	-	-	-	-	-	-	-	-	-	-	-	-
		-	-	-	-	-	-	-	-	-	-	-	-	180727	-	-	-	-	-	-	-	-	-	-	-	-	-	-	-	-	-	-	30068	-	-	-	-	-	-	-	-	-	-	-	-	-	-	-	-	-	-	-
		-	+	-	-	-	-	-	-	-	BDEG_00182	-	-	180724	-	-	-	-	DDB0186492	-	-	-	-	-	-	LmjF36.4050	-	12050	-	-	-	-	34864	-	11690	-	-	-	-	RO3G_00472.1	-	SPAC688.13	-	-	-	-	-	-	-	-	Tb11.01.2090	UM04485.1
		-	+	-	-	-	-	-	AT5G20040.2	-	-	-	-	180720	-	-	CMS475C	-	-	-	-	-	-	-	-	-	-	-	-	-	-	Os06g51350.1	33635	-	43183	165772	-	-	642383	-	-	-	-	-	-	-	-	-	-	-	-	-
		-	-	-	-	-	-	-	AT3G15550.1	-	-	-	-	180718	-	-	-	-	-	-	-	-	-	-	-	-	-	-	-	-	-	-	-	-	-	134642	-	-	414216	-	-	-	-	-	-	-	-	-	-	-	-	-
		-	-	-	-	-	-	-	-	-	-	-	-	180717	-	-	-	-	-	-	-	-	-	-	-	-	-	-	-	-	-	Os12g13070.1	-	-	-	-	-	-	-	RO3G_17263.1	-	-	-	-	-	-	-	-	-	-	-	-
		-	-	-	-	-	-	-	-	-	-	-	-	180716	-	-	-	-	-	-	-	-	-	-	-	-	-	-	-	-	-	-	-	-	-	-	-	-	-	-	-	-	-	-	-	-	-	-	-	-	-	-
		-	+	-	-	-	-	-	AT2G41620.1	-	BDEG_02975	-	177509	180714	213542	-	-	-	-	CG11092-PA	-	-	ENSGALP00000004746	-	NP_055484.2	-	179365	-	-	242660	-	Os03g22690.1	-	-	-	159641	130105	-	823177	-	-	-	-	-	-	-	-	-	49781	-	-	UM03813.1
FAP157	Flagellar Associated Protein	-	+	-	+	-	-	-	-	-	BDEG_07688	-	-	180708	291671	-	-	OTTDARP00000019314	-	-	-	-	-	-	OTTHUMP00000022194	-	190312	-	-	-	-	-	-	GSPATP00020513001	-	-	144504	-	-	-	-	-	-	-	45.m00258	-	-	-	20472	-	-	-
		-	-	-	-	-	-	-	AT4G19645.2	-	-	-	-	180707	-	-	-	-	-	-	-	-	-	-	-	-	-	-	-	-	-	Os01g60120.2	-	-	-	126739	-	-	250135	-	-	-	-	-	-	-	-	-	-	-	-	-
		-	-	-	-	-	-	-	-	-	-	-	-	180702	-	-	-	-	-	-	-	-	-	-	-	-	-	-	-	-	-	-	-	-	-	-	-	-	-	-	-	-	-	-	-	-	-	-	-	-	-	-
		-	-	-	-	-	-	-	-	-	-	-	-	180699	-	-	-	-	-	-	-	-	-	-	-	-	-	-	-	-	-	-	33873	-	-	-	-	-	-	-	-	-	-	-	-	-	-	-	-	-	-	-
		-	-	-	-	-	-	-	-	-	-	-	-	180696	-	-	-	-	-	-	-	-	-	-	-	-	-	-	-	-	-	-	-	-	-	-	-	-	-	-	-	-	-	-	-	-	-	-	-	-	-	-
		-	-	-	-	-	-	-	-	-	-	-	-	180694	-	-	-	-	-	-	-	-	-	-	-	-	-	-	-	-	-	-	-	-	-	-	-	-	-	-	-	-	-	-	-	-	-	-	-	-	-	-
		-	-	-	-	-	-	-	-	-	-	-	-	180693	-	-	-	-	-	-	-	-	-	-	-	-	-	-	-	-	-	-	-	-	-	-	-	-	-	-	-	-	-	-	-	-	-	-	-	-	-	-
		-	-	-	-	-	-	-	-	-	-	-	-	180692	-	-	-	-	-	-	-	-	-	-	-	-	-	-	-	-	-	-	-	-	-	-	-	-	-	-	-	-	-	-	-	-	-	-	-	-	-	-
		-	-	-	-	-	-	-	-	-	-	-	-	180688	-	-	-	-	-	-	-	-	-	-	-	-	-	-	-	-	-	-	-	-	-	-	-	-	-	-	-	-	-	-	-	-	-	-	-	-	-	-
		-	-	-	-	-	-	-	-	-	-	-	-	180684	-	-	-	-	-	-	-	-	-	-	-	-	-	-	-	-	-	-	-	-	-	-	-	-	-	-	-	-	-	-	-	-	-	-	-	-	-	-
		-	-	-	-	-	-	-	-	-	-	-	-	180678	-	-	-	-	-	-	-	-	-	-	-	-	-	-	-	-	-	-	-	-	-	-	-	-	-	-	-	-	-	-	-	-	-	-	-	-	-	-
		-	-	-	-	-	-	-	-	-	-	-	-	180676	-	-	-	-	-	-	-	-	-	-	-	-	-	-	-	-	-	-	-	-	-	-	-	-	-	-	-	-	-	-	-	-	-	-	-	-	-	-
		-	-	-	+	-	-	-	-	-	-	-	-	180664	-	-	-	-	-	-	-	-	-	-	-	-	-	-	-	10113	-	-	-	-	-	-	-	-	-	-	-	-	-	-	-	-	-	-	-	-	-	-
		-	+	-	-	-	-	-	-	-	-	-	-	180630	-	-	-	-	-	-	-	-	-	-	OTTHUMP00000021966	-	-	21609	-	94578	-	-	34760	GSPATP00029605001	-	132697	121181	-	-	-	-	-	-	-	46.m00250	-	-	-	-	-	-	-
		-	-	-	-	-	-	-	-	-	-	-	-	180628	-	-	-	-	-	-	-	-	-	-	-	-	-	-	-	-	-	-	-	-	-	-	-	-	-	-	-	-	-	-	-	-	-	-	-	-	-	-
		-	-	-	-	-	-	-	AT4G13670.1	-	-	-	-	180627	-	-	-	-	-	-	-	-	-	-	-	-	-	-	-	-	-	Os04g43420.1	-	-	-	170164	-	-	-	-	-	-	-	-	-	-	-	-	-	-	-	-
		-	-	-	-	-	-	-	-	-	-	-	-	180626	-	-	-	-	-	-	-	-	-	-	-	-	-	-	-	-	-	-	-	-	-	-	-	-	-	-	-	-	-	-	-	-	-	-	-	-	-	-
CLR15	Coxiella-Like Repeat protein	-	-	-	-	-	-	-	-	-	-	-	-	180625	-	-	-	-	-	-	-	-	-	-	-	-	-	-	-	-	-	-	-	-	-	-	-	-	-	-	-	-	-	-	-	-	-	-	-	-	-	-
		-	-	-	-	-	-	-	-	-	-	-	-	180612	-	-	-	-	-	-	-	-	-	-	-	-	-	-	-	-	-	-	-	-	-	-	-	-	-	-	-	-	-	-	-	-	-	-	-	-	-	-
		+	+	-	-	-	-	-	-	-	BDEG_01038	-	-	180607	-	-	-	-	-	-	-	-	ENSGALP00000025445	-	OTTHUMP00000016831	LmjF17.0150	218665	-	-	-	-	-	-	-	-	-	-	-	-	-	-	-	-	SINFRUP00000167763	-	-	-	44.m02586	50604	89416.m00125	Tb927.7.6280	-
		-	-	-	-	-	-	-	-	-	-	-	-	180606	-	-	-	-	-	-	-	-	-	-	-	-	-	-	-	-	-	-	-	-	-	-	-	-	-	-	-	-	-	-	-	-	-	-	-	-	-	-
		-	-	-	-	-	-	-	-	-	-	-	-	180605	-	-	-	-	-	-	-	-	-	-	-	-	-	-	-	-	-	-	-	-	-	-	-	-	-	-	-	-	-	-	-	-	-	-	-	-	-	-
		-	-	-	-	-	-	-	-	-	-	-	-	180604	-	-	-	-	-	-	-	-	-	-	-	-	-	-	-	-	-	-	-	-	-	-	-	-	-	-	-	-	-	-	-	-	-	-	-	-	-	-
		-	-	-	-	-	-	-	-	-	-	-	-	180593	-	-	-	-	-	-	-	-	-	-	-	-	-	-	-	-	-	-	-	-	-	-	-	-	-	-	-	-	-	-	-	-	-	-	-	-	-	-
		-	-	-	-	-	-	-	-	-	-	-	-	180584	-	-	-	-	-	-	-	-	-	-	-	-	-	-	-	-	-	-	-	-	-	-	-	-	-	-	-	-	-	-	-	-	-	-	-	-	-	-
		-	-	-	-	-	-	-	-	-	-	-	-	180576	-	-	-	-	-	-	-	-	-	-	-	-	-	-	-	-	-	-	-	-	-	-	-	-	-	-	-	-	-	-	-	-	-	-	-	-	-	-
		-	-	-	-	-	-	-	-	-	-	-	-	180573	-	-	-	-	-	-	-	-	-	-	-	-	-	-	-	-	-	-	-	-	-	-	-	-	-	-	-	-	-	-	-	-	-	-	-	-	-	-
		-	-	-	-	-	-	-	-	-	-	-	-	180565	-	-	-	-	-	-	-	-	-	-	-	-	-	-	-	-	-	-	-	-	-	-	-	-	-	-	-	-	-	-	-	-	-	-	-	-	-	-
		-	-	-	-	-	-	-	-	-	-	-	-	180560	-	-	-	-	-	-	-	-	-	-	-	-	-	-	-	-	-	-	-	-	-	-	-	-	-	-	-	-	-	-	-	-	-	-	-	-	-	-
		-	-	-	-	-	-	-	-	-	-	-	-	180559	-	-	-	-	-	-	-	-	-	-	-	-	-	-	-	-	-	-	-	-	-	-	-	-	-	-	-	-	-	-	-	-	-	-	-	-	-	-
		-	-	-	-	-	-	-	-	-	-	-	-	180554	-	-	-	-	-	-	-	-	-	-	-	-	-	-	-	-	-	-	-	-	-	-	-	-	-	-	-	-	-	-	-	-	-	-	-	-	-	-
		-	+	-	-	-	-	-	AT2G25605.1	-	-	-	-	180553	-	-	CMN035C	-	-	-	-	-	-	-	-	-	-	-	-	-	-	Os05g49910.1	-	-	-	219361	-	-	819577	-	-	-	-	-	-	-	-	-	-	-	-	-
		-	-	-	-	-	-	-	-	-	-	-	-	180552	-	-	-	-	-	-	-	-	-	-	-	-	-	-	-	-	-	-	-	-	-	-	-	-	-	-	-	-	-	-	-	-	-	-	-	-	-	-
		-	-	-	-	-	-	-	-	-	-	-	-	180551	-	-	-	-	-	-	-	-	-	-	-	-	-	-	-	-	-	-	-	-	-	-	-	-	-	-	-	-	-	-	-	-	-	-	-	-	-	-
		-	-	-	-	-	-	-	-	-	-	-	-	180546	-	-	-	-	-	-	-	-	-	-	-	-	-	-	-	-	-	-	-	-	-	-	-	-	-	-	-	-	-	-	-	-	-	-	-	-	-	-
		-	-	-	-	-	-	-	-	-	-	-	-	180542	-	-	-	-	-	-	-	-	-	-	-	-	-	-	-	-	-	-	-	-	-	-	-	-	-	-	-	-	-	-	-	-	-	-	-	-	-	-
		-	-	-	-	-	-	-	-	-	-	-	-	180538	-	-	-	-	-	-	-	-	-	-	-	-	-	-	-	-	-	-	-	-	-	-	-	-	-	-	-	-	-	-	-	-	-	-	-	-	-	-
		-	-	-	-	-	-	-	-	-	-	-	-	180526	-	-	-	-	-	-	-	-	-	-	-	-	-	-	-	-	-	-	-	-	-	-	-	-	-	-	-	-	-	-	-	-	-	-	-	-	-	-
		-	-	-	-	-	-	-	-	-	-	-	-	180525	-	-	-	-	-	-	-	-	-	-	-	-	-	-	75185	-	-	-	32861	-	-	-	-	-	-	-	-	-	-	-	-	-	-	-	-	-	-	-
		-	-	-	-	-	-	-	-	-	-	-	-	180514	-	-	-	-	-	-	-	-	-	-	-	-	-	-	-	-	-	-	-	-	-	-	-	-	-	-	-	-	-	-	-	-	-	-	-	-	-	-
		-	-	-	-	-	-	-	-	-	-	-	-	180513	-	-	-	-	-	-	-	-	-	-	-	-	-	-	-	-	-	-	-	-	-	-	-	-	-	-	-	-	-	-	-	-	-	-	-	-	-	-
		-	-	-	-	-	-	-	-	-	-	-	-	180512	-	-	-	-	-	-	-	-	-	-	-	-	-	-	-	-	-	-	-	-	-	-	-	-	-	-	-	-	-	-	-	-	-	-	-	-	-	-
		-	-	-	-	-	-	-	-	-	-	-	-	180511	-	-	-	-	-	-	-	-	-	-	-	-	-	-	-	-	-	-	-	-	-	-	-	-	-	-	-	-	-	-	-	-	-	-	-	-	-	-
		-	-	-	-	-	-	-	-	-	-	-	-	180508	-	-	-	-	-	-	-	-	-	-	-	-	-	-	-	-	-	-	-	-	-	-	-	-	-	-	-	-	-	-	-	-	-	-	-	-	-	-
		-	-	-	-	-	-	-	-	-	-	-	-	180503	-	-	-	-	-	-	-	-	-	-	-	-	-	-	-	-	-	-	-	-	-	-	-	-	-	-	-	-	-	-	-	-	-	-	-	-	-	-
		-	-	-	-	-	-	-	-	-	-	-	-	-	-	-	-	-	-	-	-	-	-	-	-	-	-	-	-	-	-	-	-	-	-	-	-	-	-	-	-	-	-	-	-	-	-	-	-	-	-	-
		-	-	-	-	-	-	-	-	-	-	-	-	180494	-	-	-	-	-	-	-	-	-	-	-	-	-	-	-	-	-	-	-	-	-	-	-	-	-	-	-	-	-	-	-	-	-	-	-	-	-	-
		-	-	-	-	-	-	-	-	-	-	-	-	180493	-	-	-	-	-	-	-	-	-	-	-	-	-	-	-	-	-	-	-	-	-	-	-	-	-	-	-	-	-	-	-	-	-	-	-	-	-	-
		-	+	-	-	-	-	GB13773-PA	-	-	-	-	-	180492	291312	-	-	-	DDB0205053	-	-	-	ENSGALP00000011367	-	OTTHUMP00000171669	LmjF07.0440	-	36649	4983	-	-	Os05g09370.3	-	GSPATP00007727001	44982	163280	-	-	589371	-	-	-	-	SINFRUP00000157798	1.m01105	36013	-	-	52813	-	Tb927.8.1440	-
		-	-	-	-	-	-	-	-	-	-	-	-	180482	-	-	-	-	-	-	-	-	-	-	-	-	-	-	-	-	-	-	-	-	-	-	-	-	-	-	-	-	-	-	-	-	-	-	-	-	-	-
		-	-	-	-	-	-	-	-	-	-	-	-	180481	-	-	-	-	-	-	-	-	-	-	-	-	-	-	-	-	-	-	-	-	-	-	-	-	-	-	-	-	-	-	-	-	-	-	-	-	-	-
ELG19	exostosin-like glycosyltransferase	-	-	-	-	-	-	-	-	-	-	-	-	180472	-	-	-	-	-	-	-	-	-	-	-	-	-	-	-	-	-	-	-	-	-	-	-	-	-	-	-	-	-	-	-	-	-	-	-	-	-	-
ELG18	exostosin-like glycosyltransferase	-	-	-	-	-	-	-	-	-	-	-	-	180471	-	-	-	-	-	-	-	-	-	-	-	-	-	-	-	217093	-	-	-	GSPATP00014251001	-	-	-	-	-	-	-	-	-	-	-	-	-	-	-	-	-	-
		-	-	-	-	-	-	-	-	-	-	-	-	180470	-	-	-	-	-	-	-	-	-	-	-	-	-	-	-	-	-	-	-	-	-	-	-	-	-	-	-	-	-	-	-	-	-	-	-	-	-	-
		-	-	-	-	-	-	-	-	-	-	-	-	180466	-	-	-	-	-	-	-	-	-	-	-	-	-	-	-	-	-	-	-	-	-	-	-	-	-	-	-	-	-	-	-	-	-	-	-	-	-	-
		-	-	-	-	-	-	-	-	-	-	-	-	180460	-	-	-	-	-	-	-	-	-	-	-	-	-	-	-	-	-	-	-	-	-	-	-	-	-	-	-	-	-	-	-	-	-	-	-	-	-	-
		-	-	-	-	-	-	-	-	-	-	-	-	180456	-	-	-	-	-	-	-	-	-	-	-	-	-	-	-	-	-	-	-	-	-	-	-	-	-	-	-	-	-	-	-	-	-	-	-	-	-	-
		-	-	-	-	-	-	-	-	-	-	-	-	180455	-	-	-	-	-	-	-	-	-	-	-	-	-	-	-	-	-	-	-	-	-	-	-	-	-	-	-	-	-	-	-	-	-	-	-	-	-	-
		-	-	-	-	-	-	-	-	-	-	-	-	180454	-	-	-	-	-	-	-	-	-	-	-	-	-	-	-	-	-	-	-	-	-	-	-	-	-	-	-	-	-	-	-	-	-	-	-	-	-	-
		-	-	-	-	-	-	-	-	-	-	-	-	180449	-	-	-	-	-	-	-	-	-	-	-	-	-	-	-	-	-	-	-	-	-	-	-	-	-	-	-	-	-	-	-	-	-	-	-	-	-	-
		-	+	-	-	-	-	GB18851-PA	AT5G58040.1	-	-	-	218798	180447	-	-	-	OTTDARP00000008970	-	CG1078-PA	-	-	ENSGALP00000012440	-	OTTHUMP00000158885	-	-	-	-	215008	-	-	23337	-	-	-	-	-	-	RO3G_03336.1	-	SPAC22G7.10	-	SINFRUP00000162592	-	-	-	-	60103	-	-	-
		-	-	-	-	-	-	-	-	-	-	-	-	180440	-	-	-	-	-	-	-	-	-	-	-	-	-	-	-	-	-	-	-	-	-	-	-	-	-	-	-	-	-	-	-	-	-	-	-	-	-	-
		-	-	-	-	-	-	-	-	-	-	-	-	180434	230361	-	-	-	-	-	-	-	-	-	-	-	-	-	-	-	-	-	-	-	-	-	-	-	-	-	-	-	-	-	-	-	-	-	-	-	-	-
		-	-	-	-	-	-	-	-	-	-	-	-	180433	-	-	-	-	-	-	-	-	-	-	-	-	-	-	-	-	-	-	-	-	-	-	-	-	-	-	-	-	-	-	-	-	-	-	-	-	-	-
		-	-	-	-	-	-	-	-	-	-	-	-	180430	-	-	-	-	-	-	-	-	-	-	-	-	-	-	-	-	-	-	-	-	-	-	-	-	-	-	-	-	-	-	-	-	-	-	-	-	-	-

		- - - - - -	180419
		- - - - - -	180414
		- - - - - -	180413
		- - - - - -	180412
		- - - - - -	180411
		- - - - - -	180410
		- - - - - -	AT1G68280.1 · 135463 · 180405 · Os04g46730.1 · 833617
		- - - - - -	180403 · 36586
CLR9	Coxiella-Like Repeat protein	- - - - - -	180400
		- - - - - -	180399
		- - - - - -	180396
		- - - - - -	180395
		- - - - - -	180391
		- - - - - -	180388 · 31900
		- - - - - -	180387
		- - - - - -	180385
		- + - - - -	AT3G55160.1 · 180384 · 69592 · Os08g07290.2 · 23875 · 135116 · 800663
		- - - - - -	180381
		- - - - - -	180364
		- - - - - -	AT5G43860.1 · 180363 · 37159
		- + - - - -	GB10189-PA · AT1G30470.1 · BDEG_06976 · CE38162 · 184298 · 180347 · 270350 · CMN046C · OTTDARP00000008351 · DDB0189736 · CG10289-PA · 10.m00317 · ENSGALP00000011376 · OTTHUMP00000196589 · 106669 · 57747 · 175233 · NCU10018 · Os05g51490.2 · GSPATP00036171001 · 134420 · PFD0875c · 204274 · RO3G_00919.1 · YKR028W · SPCC663.01c · SINFRUP00000146753 · 46.m00165 · 641.m01543 · 23113 · UM03301.1
		- - - - - -	180346
		- + - - - -	GB16384-PA · AT1G48090.2 · BDEG_05468 · CE30353 · 226182 · 180327 · 289994 · CML142C · DDB0187116 · CG2093-PA · 19068788 · ENSGALP00000024437 · GL50803_87358 · NP_060550.2 · LmjF34.1390 · 230253 · 32976 · 77909 · 246510 · NCU05837 · Os02g27130.1 · 31938 · GSPATP00034047001 · 47968 · 96917 · 136943 · 564344 · RO3G_15840.1 · YLL040C · SPBC16C6.02c · SINFRUP00000134332 · 8.m00485 · 25297 · TA17650 · 57473 · 93793.m00241 · Tb927.8.5580
		- + - - - -	AT1G53800.2 · 180316 · DDB0188211 · 53111 · Os10g12354.1 · 46452 · 168110 · 813228
		- - - - - -	180315 · 38031
		- - - - - -	180300
		- - - - - -	180295
		- - - - - -	-
		- - - - - -	180291
		+ + - - - -	GB16560-PA · 72004 · CE03301 · 180289 · 261740 · CG10806-PB · ENSGALP00000020101 · OTTHUMP00000161768 · LmjF14.0980 · 186688 · 141605 · 31536 · SINFRUP00000134501 · 4725 · 57482
		- - - - - -	180286
ETFB	putative electron transfer flavoprotein beta-subunit , mitochondrial	- + - + - -	GB16132-PA · AT5G43430.1 · 28553 · BDEG_01271 · CE29774 · 170982 · 180281 · 297451 · CMS315C · DDB0204353 · OTTHUMP00000083400 · LmjF25.0120 · 203788 · 36967 · 75058 · 192806 · NCU07733 · Os04g10400.1 · GSPATP00009160001 · 17860 · 203561 · 109126 · 791393 · RO3G_10300.1 · YGR207C · SPAC1805.02c · GLEAN3_19957 · SINFRUP00000155202 · 24.m00347 · 41314 · 57.m01881 · 37485 · Tb11.03.0950 · UM00519.1
		- - - - - -	180280
		- + - - - -	AT1G78915.1 · 180279 · Os07g18720.1 · 30564 · 132176 · 563429
		- - - - - -	180278 · LmjF33.0030 · 31.m00909
		- - - - - -	180277
		- - - - - -	180275
		- - - - - -	180274
MRS2C	Mg2+ transporter protein, CorA-like	- - - - - -	180272
		- - - - - -	180271 · 82391 · NCU02923
		- - - - - -	180265
		- - - - - -	180264
		- - - - - -	180260 · 211488 · NCU01964 · 128260 · RO3G_01331.1
		- - - - - -	180258 · 72137 · 8420
		- - - - - -	180257
		- + - - - -	AT3G61080.1 · 27452 · CE23333 · 92436 · 180256 · 295510 · CMR459C · OTTDARP00000022409 · ENSGALP00000002324 · GL50803_2969 · OTTHUMP00000180772 · 121864 · 166358 · NCU09611 · Os03g02640.1 · 33360 · 26375 · 165144 · 409473 · RO3G_05073.1 · GLEAN3_06264 · SINFRUP00000172970 · 263819 · 28558 · UM03441.1
MRE11	DNA damage signaling and repair protein.	- - - - - -	180252
		- - - - - -	180245 · 293646 · NCU05069 · 159773
		- + - - - -	GB13930-PA · 59184 · BDEG_06439 · CE11882 · 226963 · 180240 · 287084 · CMJ162C · OTTDARP00000023307 · DDB0188804 · CG1942-PA · ENSGALP00000006956 · NP_001002254.1 · LmjF25.1630 · 206209 · 25681 · 244419 · NCU02665 · 8948 · GSPATP00029120001 · 43469 · 131364 · RO3G_01707.1 · YOR245C · SPCC1235.15 · GLEAN3_24478 · SINFRUP00000141951 · 38490 · 42.m00127 · 63184 · Tb927.3.1700 · UM03937.1
		- - - - - -	180238
		- - - - - -	180231
SPS1	spermine synthase, putative	- + - - - -	GB12895-PA · AT1G70310.1 · 59112 · BDEG_08227 · CE31114 · 156944 · 180226 · 232387 · CMP329C · DDB0190077 · CG8327-PA · ENSGALP00000004799 · OTTHUMP00000002170 · LmjF04.0580 · 218994 · 23668 · 55563 · 165376 · NCU06727 · Os07g22600.1 · 4750 · 55177 · 201920 · 109524 · PF11_0301 · 833545 · RO3G_16619.1 · YPR069C · SPBC12C2.07c · GLEAN3_18922 · SINFRUP00000152096 · 46.m00154 · 30691 · 57643 · Tb09.v1.0380 · UM05818.1
FAP67	Flagellar Associated Protein	+ + - + - -	GB15059-PA · 70441 · BDEG_03287 · 160391 · 180221 · 369349 · OTTDARP00000020202 · CG8362-PA · ENSGALP00000024531 · GL50803_14135 · OTTHUMP00000032536 · LmjF35.3870 · 187020 · 33146 · 125694 · 35618 · GSPATP00004758001 · 91902 · 130886 · GLEAN3_22006 · SINFRUP00000178540 · 3779 · 20.m03702 · 51403 · 97465.m00158 · Tb09.211.2560
		- - - - - -	180210
		- + - - - -	GB14115-PA · AT2G06040.1 · 191279 · 180209 · 218611 · CMI121C · CG9952-PA · ENSGALP00000009102 · NP_659469.2 · 184154 · 1762 · 214518 · NCU01216 · Os08g35700.1 · 54119 · 152586 · 819452 · SPBC25B2.11 · 1588
ClpT2	Clp protease adaptor protein	- - - - - -	180207 · CMJ276C · Os01g61700.1 · 48630 · 822021
ARM1	protein of unknown function, contains armadillo (Arm) repeat	- - - - - -	BDEG_03436 · 180203 · 222704
		- - - - - -	180200
		- - - - - -	180197
		- - - - - -	180196
	putative lipopolysaccharide-induced tumor necrosis factor alpha factor	- - - - - -	182700 · 180194 · SINFRUP00000177523
		- + - - - -	GB19061-PA · AT4G10920.2 · BDEG_05616 · CE13621 · 102830 · 180184 · 208368 · CMI225C · OTTDARP00000018887 · DDB0190769 · CG8396-PA · 69.m00181 · NP_006704.2 · 112486 · NCU04584 · Os01g50960.1 · 31219 · 37144 · 148329 · 158780 · 409488 · SPAC16A10.02 · SINFRUP00000130285 · 3.m01995 · 5643 · 88737.m00062
		- - - - - -	180177
		- + - - - -	AT5G63310.1 · 180172 · Os12g36194.1 · 8860 · 112996 · 773138
		- + - - - -	GB14050-PA · AT5G20165.1 · 23438 · CE29595 · 129515 · 180169 · 247460 · cgd3_1420 · CMR096C · OTTDARP00000024124 · DDB0188425 · CG14199-PA · NP_777569.1 · LmjF35.0210 · 121854 · 14967 · 187198 · NCU03465 · 21867 · GSPATP00003101001 · 12535 · 227620 · 565050 · YNL024C-A · TA12850 · 583.m09205 · 25901 · Tb10.70.3125
		- + - - - -	GB17310-PA · 180160 · 287381 · OTTDARP00000015006 · NP_699197.3 · 8830 · 218152 · GLEAN3_22974 · SINFRUP00000138761
		- - - - - -	180159
		- - - - - -	180154
STPK14	Serine/Threonine Protein Kinase Homolog 14	- - - - - -	180153
		- - - - - -	180151
		- - - - - -	AT3G23980.1 · 180132
RPS11	Ribosomal protein S11, component of cytosolic 80S ribosome and 40S small subunit	- + + - - -	GB15755-PA · AT3G48930.1 · 34507 · BDEG_03276 · CE05860 · 165333 · 180130 · 221236 · CMO287C · DDB0204861 · CG8857-PC · 19068783 · 302.m00048 · ENSGALP00000035595 · GL50803_14827 · OTTHUMP00000077985 · LmjF20.1650 · 202957 · 9264 · 56310 · 239074 · NCU03102 · Os04g42380.1 · 17884 · GSPATP00016795001 · 28562 · 163092 · 142976 · PFC0775w · 677637 · RO3G_06840.1 · YDR025W · SPAC31G5.03 · GLEAN3_22682 · SINFRUP00000138156 · 239.m00049 · 22535 · TA12390 · 42.m00125 · 38115 · 94179.m00299 · Tb10.70.7695 · UM01317.1
		- - - - - -	180122
		- - - - - -	180118
		- - - - - -	180101 · 88217.m00082
		- + - - - -	BDEG_00814 · 180097 · 72538 · NCU01055 · 37358 · GSPATP00012675001 · 48511 · 160600 · 66.m00229 · 262849 · UM02213.1
		- - - - - -	180090
		- + - - - -	AT1G11240.1 · 180089 · CG32418-PA · ENSGALP00000020252 · 236121 · 244581 · GSPATP00028386001 · 822798 · SPAC29A4.09 · 3919 · UM05438.1
CHR3408	SWI/SNF chromatin remodeling complex component	- + - - - -	AT5G63950.1 · 180087 · 271235 · DDB0219387 · 11528 · 73828 · Os04g59624.1 · 71851 · 562497 · SINFRUP00000174358
		- - - - - -	180086
		- + - - - -	CE08574 · 227363 · 180081 · 281436 · 172704 · 86719 · GLEAN3_14828
		- - - - - -	180072
		- - - - - -	AT2G44850.1 · 180044 · 144798 · 551953
		- - - - - -	180043
		+ + - - - -	72455 · BDEG_07133 · 228697 · 180040 · 291742 · OTTDARP00000017662 · ENSGALP00000007243 · OTTHUMP00000196113 · 231625 · 65759 · GSPATP00021758001 · GLEAN3_06742 · SINFRUP00000162630 · 5970 · 53074 · Tb11.01.1590
		- + - - - -	GB17438-PA · BDEG_06269 · CE12146 · 118429 · 180018 · 374792 · DDB0188836 · OTTHUMP00000013912 · 154157 · 38125 · 157243 · NCU02108 · GSPATP00006326001 · 11230 · 158648 · 422611 · RO3G_11187.1 · YAL044W-A · SPBC16E9.06c · GLEAN3_17745 · SINFRUP00000180585 · 49.m05649 · 63109
		- - - - - -	180011
		- - - - - -	AT3G48980.1 · 180010 · NCU11268 · Os08g01150.1 · UM04451.1
		- - - - - -	180000
		- + - - - -	AT3G10630.1 · 21759 · BDEG_04037 · 179989 · GL50803_11487 · LmjF31.1870 · 25815 · 52212 · 218200 · Os09g12530.1 · GSPATP00036577001 · 115810 · 144448 · 563555 · 91.m00134
		- - - - - -	179981
		- - - - - -	179979
		- - - - - -	179978
PSD1		- + - - - -	AT4G25970.1 · BDEG_02163 · 179976 · DDB0204189 · NCU11273 · Os01g72940.1 · 142294 · 640632 · RO3G_11316.1 · YGR170W · SPAC31G5.15 · GLEAN3_28562
		- - - - - -	179974
		- - - - - -	179969
		- - - - - -	179962
		- - - - - -	179961
		- - - - - -	179951
		- - - - - -	179949
		- - - - - -	179940
		- - - - - -	179939
		- - - - - -	179935
		- - - - - -	179934
		- - - - - -	179933
		- - - - - -	179932
		- - - - - -	179930
		- - - - - -	179927
		- - - - - -	179926
		- - - - - -	179920
		- - - - - -	179907
		- - - - - -	179906
		- - - - - -	179904
		- - - - - -	179888
		- - - - - -	179887
	hypothetical protein, conserved	- + - - - -	BDEG_05006 · 209775 · 179884 · 384787 · 161769 · 6280 · 242959 · 46105 · 25748 · 56707 · Tb11.02.5240
		- - - - - -	179872 · DDB0219347
		- - - - - -	179870
		- - - - - -	179869
		- - - - - -	179859
		- - - - - -	179855
		- - - - - -	179848
		- - - - - -	179847
		- - - - - -	179846
		- - - - - -	179840
		- - - - - -	179839
		- - - - - -	179827 · 262721
		- - - - - -	179812
		- - - - - -	179808
		- - - - - -	179807
		- - - - - -	179806
		- - - - - -	179805
		- - - - - -	179790
		- - - - - -	179771
		- - - - - -	179769
		- - - - - -	179764
		- - - - - -	179761
		- - - - - -	228536 · 179747 · OTTHUMP00000181801 · 109931 · 198419
	related to haem peroxidases	- + - - - -	220641 · 179737 · LmjF34.3190 · 75681 · Os01g03670.1 · 93.m00114 · 8349 · Tb927.4.1540
		- - - - - -	179730
		- - - - - -	179728
		- + - - - -	GB16393-PA · BDEG_08248 · CE03024 · 159495 · 179719 · 206279 · OTTDARP00000022933 · CG10480-PC · 19068871 · NP_001041659.1 · 178675 · 33844 · 237483 · NCU05513 · 16486 · 135771 · RO3G_16655.1 · YGL097W · SPBC557.03c · SINFRUP00000153741 · 67.m00216 · 58118 · UM00556.1
RPS7	Ribosomal protein S7, component of cytosolic 80S ribosome and 40S small subunit	- + + - - -	GB15225-PB · AT1G48830.2 · 70049 · BDEG_05536 · CE06577 · 181102 · 179706 · 391223 · cgd5_3040 · CMA122C · OTTDARP00000005528 · DDB0188217 · 4.m00645 · ENSGALP00000026392 · GL50803_14329 · OTTHUMP00000115354 · LmjF01.0420 · 190901 · 35710 · 83145 · 54581 · NCU00258 · Os03g18580.1 · 28876 · GSPATP00014990001 · 31073 · 208894 · 109563 · PF13_0014 · 749404 · RO3G_15787.1 · YNL096C · SPAC18G6.14c · GLEAN3_21322 · SINFRUP00000131272 · 56.m00227 · 39550 · TA11270 · 49.m00013 · 63335 · 86827.m00507 · Tb09.160.2550 · UM05040.1
		- + - - - -	4745 · BDEG_06497 · 179694 · cgd6_4770 · OTTDARP00000019051 · DDB0218618 · 465.m00045 · ENSGALP00000002626 · 33553 · 65237 · 71062 · NCU01184 · 15073 · GSPATP00019151001 · 141842 · PFE0750c · RO3G_05386.1 · YDL209C · SPAC3A12.11c · SINFRUP00000134423 · 176.m00085 · TA08595 · 57.m03133 · 55671 · UM02977.1
		- - - - - -	-
		- - - - - -	179671 · OTTHUMP00000165993 · 206692 · 577195 · RO3G_07684.1
		- - - - - -	179663 · GL50803_113565 · 100746 · 156429 · UM05154.1
		- - - - - -	179658
		- - - - - -	179656
		- - - - - -	179647
		- - - - - -	179646
CYP741A1	cytochrome P450, unkown function	- - - - - -	179637
		- - - - - -	179634
		- - - - - -	179633
		- + - - - -	AT5G53940.1 · 179628 · Os03g49150.1 · 208394 · YBL049W · SPAPJ691.02 · TA12420
		- - - - - -	179614

		-	-	-	-	-	-	-	-	-	-	-	-	179605	-	-	-	-	-	-	-	-	-	-	-	-	-	-	-	-	-	-	-	-	-	-	-	-	-	-	-	-	-	-	-	-	-	-	-	-	-	-
		-	+	-	-	-	-	-	AT3G07080.1	-	-	-	-	179601	-	-	CMB075C	-	DDB0202524	-	-	-	-	-	-	LmjF07.0400	-	-	-	-	-	Os06g46820.2	24366	-	26807	192642	127772	-	573321	-	-	-	-	-	-	-	-	-	-	-	-	-
		-	+	-	-	-	-	-	AT1G31830.2	32482	-	-	-	179587	-	-	CMR055C	-	DDB0189332	-	-	151.m00099	-	GL50803_16433	-	LmjF14.0320	-	-	-	-	-	Os02g47210.1	-	-	11160	146161	143871	-	646369	-	-	-	-	-	-	681	-	-	-	-	Tb09.211.1760	-
		-	+	-	-	-	-	-	AT2G46100.1	-	-	-	-	179586	-	-	-	-	-	-	-	-	-	-	-	-	-	-	-	-	-	-	32859	-	31684	26445	-	-	816607	-	-	-	-	-	-	4131	-	-	-	-	-	-
		-	-	-	-	-	-	-	-	-	-	-	-	179575	-	-	-	-	DDB0203300	-	-	-	-	-	-	-	-	-	-	159012	-	-	-	-	-	-	-	-	-	RO3G_05696.1	-	-	-	-	-	-	-	-	-	-	-	-
		-	-	-	-	-	-	-	-	-	-	-	-	179569	-	-	-	-	-	-	-	-	-	-	-	-	-	-	-	-	-	-	-	-	-	-	-	-	-	-	-	-	-	-	-	-	-	-	-	-	-	-
		-	-	-	-	-	-	-	-	-	-	-	-	179568	-	-	-	-	-	-	-	-	-	-	-	-	-	-	-	-	-	-	-	-	-	-	-	-	-	-	-	-	-	-	-	-	-	-	-	-	-	-
		-	-	-	-	-	-	-	-	-	-	-	-	179567	-	-	-	-	-	-	-	-	-	-	-	-	-	-	-	-	-	-	-	-	-	-	-	-	-	-	-	-	-	-	-	-	-	-	-	-	-	-
		-	-	-	-	-	-	-	-	-	-	-	-	179566	-	-	-	-	-	-	-	-	-	-	-	-	-	-	-	-	-	-	-	-	-	-	-	-	-	-	-	-	-	-	-	-	-	-	-	-	-	-
		-	-	-	-	-	-	-	-	-	-	-	-	179565	-	-	-	-	-	-	-	-	-	-	-	-	-	-	-	-	-	-	-	-	-	-	-	-	-	-	-	-	-	-	-	-	-	-	-	-	-	-
		-	-	-	-	-	-	-	-	-	-	-	-	179556	-	-	-	-	-	-	-	-	-	-	-	-	-	-	-	-	-	-	-	-	-	-	-	-	-	-	-	-	-	-	-	-	-	-	-	-	-	-
		-	-	-	-	-	-	-	-	-	-	-	-	179545	-	-	-	-	-	-	-	-	-	-	-	LmjF35.3000	-	-	-	-	-	-	-	-	-	-	-	-	-	-	-	-	-	-	-	-	-	-	-	Tb09.211.3660	-
		-	-	-	-	-	-	-	-	-	-	-	-	179532	-	-	-	-	-	-	-	-	-	-	-	-	-	-	-	-	-	-	-	-	-	-	-	-	-	-	-	-	-	-	-	-	-	-	-	-	-	-
		-	-	-	-	-	-	-	-	-	-	-	-	179531	-	-	-	-	-	-	-	-	-	-	-	-	-	-	-	-	-	-	-	-	-	-	-	-	-	-	-	-	-	-	-	-	-	-	-	-	-	-
		-	-	-	-	-	-	-	-	-	-	-	-	179525	-	-	-	-	-	-	-	-	-	-	-	-	-	-	-	-	-	-	-	-	-	-	-	-	-	-	-	-	-	-	-	-	-	-	-	-	-	-
		-	-	-	-	-	-	-	-	-	-	-	-	179524	-	-	-	-	-	-	-	-	-	-	-	-	-	-	-	-	-	-	-	-	-	-	-	-	-	-	-	-	-	-	-	-	-	-	-	-	-	-
		-	-	-	-	-	-	-	-	-	-	-	-	179515	-	-	-	-	-	-	-	-	-	-	-	-	-	-	-	-	-	-	-	-	-	-	-	-	-	-	-	-	-	-	-	-	-	-	-	-	-	-
		-	-	-	-	-	-	-	-	-	-	-	-	179511	-	-	-	-	-	-	-	-	-	-	-	-	-	-	-	-	-	-	-	-	-	-	-	-	-	-	-	-	-	-	-	-	-	-	-	-	-	-
		-	-	-	-	-	-	-	-	-	-	-	-	179509	-	-	-	-	-	-	-	-	-	-	-	-	-	-	-	-	-	-	-	-	-	-	-	-	-	-	-	-	-	-	-	-	-	-	-	-	-	-
		-	-	-	-	-	-	-	-	-	-	-	-	179491	-	-	-	-	-	-	-	-	-	-	-	-	-	-	-	-	-	-	-	-	-	-	-	-	-	-	-	-	-	-	-	-	-	-	-	-	-	-
		-	-	-	-	-	-	-	-	-	-	-	-	179487	-	-	-	-	-	-	-	-	-	-	-	-	-	-	-	-	-	-	-	-	-	-	-	-	-	-	-	-	-	-	-	-	-	-	-	-	-	-
		-	-	-	-	-	-	-	-	-	-	-	-	179483	-	-	-	-	-	-	-	-	-	-	-	-	-	-	-	-	-	-	-	-	-	-	-	-	-	-	-	-	-	-	-	-	-	-	-	-	-	-
		-	-	-	-	-	-	-	-	-	-	-	-	179472	-	-	-	-	-	-	-	-	-	-	-	-	-	-	-	-	-	-	-	-	-	-	-	-	-	-	-	-	-	-	-	-	-	-	-	-	-	-
		-	-	-	-	-	-	-	-	-	-	-	-	179469	-	-	-	-	-	-	-	-	-	-	-	-	-	-	-	-	-	-	-	-	-	-	-	-	-	-	-	-	-	-	-	-	-	-	-	-	-	-
		-	-	-	-	-	-	-	-	-	-	-	-	179468	-	-	-	-	-	-	-	-	-	-	-	-	-	-	-	-	-	-	-	-	-	-	-	-	-	-	-	-	-	-	-	-	-	-	-	-	-	-
GOX5	putative glyoxal or galactose oxidase	-	-	-	-	-	-	-	-	-	-	-	-	179467	-	-	-	-	-	-	-	-	-	-	-	-	-	-	-	-	-	-	-	-	-	-	-	-	-	-	-	-	-	-	-	-	-	-	-	-	-	-
		-	+	-	-	-	-	GB19409-PA	AT5G18530.1	72120	-	CE38961	196137	179466	296176	-	-	-	DDB0189768	CG6734-PA	-	-	-	-	-	-	233981	24458	80587	206862	-	Os01g22390.3	31539	-	-	-	143104	-	226070	RO3G_11139.1	-	-	-	SINFRUP00000162958	-	25722	-	-	57150	-	-	-
		-	-	-	-	-	-	-	-	-	-	-	-	179464	-	-	-	-	-	-	-	-	-	-	-	-	-	-	-	-	-	-	-	-	-	-	-	-	-	-	-	-	-	-	-	-	-	-	-	-	-	-
		-	-	-	-	-	-	-	-	-	-	-	-	179460	280398	-	-	-	-	-	-	-	-	-	-	-	-	-	-	-	-	-	11582	-	-	-	-	-	-	-	-	-	-	-	-	-	-	-	-	-	-	-
		-	-	-	-	-	-	-	-	-	-	-	-	179459	-	-	-	-	-	-	-	-	-	-	-	LmjF02.0240	-	-	-	-	-	-	-	-	-	-	-	-	-	-	-	-	-	-	-	-	-	-	-	-	-	-
		-	-	-	-	-	-	-	-	-	-	-	-	179458	-	-	-	-	-	-	-	-	-	-	-	-	-	-	-	-	-	-	-	-	-	-	-	-	-	-	-	-	-	-	-	-	-	-	-	-	-	-
		-	-	-	-	-	-	-	-	-	-	-	-	179453	-	-	-	-	-	-	-	-	-	-	-	-	-	-	-	-	-	-	-	-	-	-	-	-	-	-	-	-	-	-	-	-	-	-	-	-	-	-
		-	-	-	-	-	-	-	-	-	-	-	-	179448	-	-	-	-	-	-	-	-	-	-	-	-	-	-	-	-	-	-	-	-	-	-	-	-	-	-	-	-	-	-	-	-	-	-	-	-	-	-
		-	-	-	-	-	-	-	-	-	-	-	-	179447	-	-	-	-	-	-	-	-	-	-	-	-	-	-	-	-	-	-	-	-	-	-	-	-	-	-	-	-	-	-	-	-	-	-	-	-	-	-
		-	-	-	-	-	-	-	-	-	-	-	-	179445	-	-	-	-	-	-	-	-	-	-	-	-	-	-	-	-	-	-	12734	-	-	-	-	-	-	-	-	-	-	-	-	-	-	-	-	-	-	-
		-	+	-	-	-	-	-	AT4G14110.1	-	-	-	154607	179443	-	-	-	-	-	-	-	-	ENSGALP00000006264	-	OTTHUMP00000164415	-	-	-	81492	-	-	Os04g34930.1	-	-	-	130004	-	-	705676	-	-	-	-	-	-	-	-	-	-	-	-	-
		-	-	-	-	-	-	-	-	-	-	-	-	179439	-	-	-	-	-	-	-	-	-	-	-	-	-	-	-	-	-	-	-	-	-	-	-	-	-	-	-	-	-	-	-	-	-	-	-	-	-	-
		-	-	-	-	-	-	-	-	-	-	-	-	179436	-	-	-	-	-	-	-	-	-	-	-	-	-	-	-	-	-	-	-	-	-	-	-	-	-	-	-	-	-	-	-	-	-	-	-	-	-	-
		-	-	-	-	-	-	-	AT5G35735.1	-	-	-	-	179431	-	-	-	-	-	-	-	-	-	-	-	-	-	-	-	-	-	-	-	-	-	168863	-	-	664838	-	-	-	-	-	-	-	-	-	-	-	-	-
		-	-	-	-	-	-	-	-	-	-	-	-	179430	-	-	-	-	-	-	-	-	-	-	-	-	-	-	-	-	-	-	-	-	-	-	-	-	-	-	-	-	-	-	-	-	-	-	-	-	-	-
		-	-	-	-	-	-	-	-	-	-	-	-	179428	-	-	-	-	-	-	-	-	-	-	-	-	-	-	-	-	-	-	-	-	-	-	-	-	-	-	-	-	-	-	-	-	-	-	-	-	-	-
		-	-	-	-	-	-	-	-	-	-	-	-	179427	-	-	-	-	-	-	-	-	-	-	-	-	-	-	-	-	-	-	-	-	-	-	-	-	-	-	-	-	-	-	-	-	-	-	-	-	-	-
		-	-	-	-	-	-	-	-	-	-	-	-	179412	-	-	-	-	-	-	-	-	-	-	-	-	-	-	-	-	-	-	-	-	-	-	-	-	-	-	-	-	-	-	-	-	-	-	-	-	-	-
POLQ1	DNA polymerase theta	-	-	-	-	-	-	-	-	-	-	-	-	179410	-	-	-	-	-	-	-	150.m00115	-	-	-	-	-	-	-	-	NCU07411	-	-	-	-	-	-	-	-	-	-	-	GLEAN3_03269	-	-	-	-	-	-	-	-	-
		-	+	+	-	-	-	GB15239-PA	AT5G11710.1	30389	BDEG_02908	CE26821	134812	179407	265265	cgd8_1910	CMJ133C	OTTDARP00000022045	DDB0183945	CG8532-PD	-	192.m00070	ENSGALP00000005857	GL50803_3256	NP_055481.1	LmjF25.0670	211098	33107	58510	82777	NCU04783	Os02g56270.1	34944	GSPATP00014884001	8741	161419	137190	-	819519	RO3G_01660.1	YJR125C	SPCC794.11c	GLEAN3_07942	SINFRUP00000167875	97.m00156	1311	-	33.m01266	28370	92603.m00056	Tb11.50.0006	UM05992.1
		-	-	-	-	-	-	-	-	-	-	-	-	179396	-	-	-	-	-	-	-	-	-	-	-	-	-	-	-	-	-	-	-	-	-	-	-	-	-	-	-	-	-	-	-	-	-	-	-	-	-	-
		-	-	-	-	-	-	-	-	-	-	-	-	179359	-	-	-	-	-	-	-	-	-	-	-	-	-	-	-	-	-	-	-	-	-	-	-	-	-	-	-	-	-	-	-	-	-	-	-	-	-	-
		-	-	-	-	-	-	-	-	-	-	-	-	179352	-	-	-	-	-	-	-	-	-	-	-	-	-	-	-	-	-	-	-	-	-	-	-	-	-	-	-	-	-	-	-	-	-	-	-	-	-	-
		-	-	-	-	-	-	-	-	-	-	-	-	179350	-	-	-	-	-	-	-	-	-	-	-	-	-	-	-	-	-	-	-	-	-	-	-	-	-	-	-	-	-	-	-	-	-	-	-	-	-	-
		-	-	-	-	-	-	-	-	-	-	-	-	179347	-	-	-	-	-	-	-	-	-	-	-	-	-	-	-	-	-	Os07g14300.1	-	-	-	-	126031	-	-	-	-	-	-	-	-	-	-	-	-	-	-	-
		-	-	-	-	-	-	-	-	71669	-	-	-	179344	-	-	CMT254C	-	-	-	-	-	-	-	-	-	-	-	-	-	-	-	-	-	34088	-	-	-	-	-	-	-	-	-	-	-	-	-	-	-	-	-
		-	-	-	-	-	-	-	-	-	-	-	-	179341	-	-	-	-	-	-	-	-	-	-	-	-	-	-	-	-	-	-	-	-	-	-	-	-	-	-	-	-	-	-	-	-	-	-	-	-	-	-
		-	-	-	-	-	-	-	-	-	-	-	-	179338	-	-	-	-	-	-	-	-	-	-	-	-	-	-	-	-	-	-	-	-	-	-	-	-	-	-	-	-	-	-	-	-	-	-	-	-	-	-
		-	-	-	-	-	-	-	-	-	-	-	-	179331	-	-	-	-	-	-	-	-	-	-	-	-	-	-	-	-	-	-	-	-	-	-	-	-	-	-	-	-	-	-	-	-	-	-	-	-	-	-
		-	-	-	-	-	-	-	-	-	-	-	-	179329	-	-	-	-	-	-	-	-	-	-	-	-	-	-	-	-	-	-	-	-	-	-	-	-	-	-	-	-	-	-	-	-	-	-	-	-	-	-
		-	-	-	-	-	-	-	-	-	-	-	-	179324	-	-	-	-	-	-	-	-	-	-	-	-	-	-	-	-	-	-	-	-	-	-	-	-	-	-	-	-	-	-	-	-	-	-	-	-	-	-
		-	-	-	-	-	-	-	-	-	-	-	-	179320	-	-	-	-	-	-	-	-	-	-	-	-	-	-	-	-	-	-	-	-	-	-	-	-	-	-	-	-	-	-	-	-	-	-	-	-	-	-
		-	-	-	-	-	-	-	-	-	-	-	-	179319	-	-	-	-	-	-	-	-	-	-	-	-	-	-	-	-	-	-	-	-	-	-	-	-	-	-	-	-	-	-	-	-	-	-	-	-	-	-
		-	-	-	-	-	-	-	-	-	-	-	-	179315	-	-	-	-	-	-	-	-	-	-	-	-	-	-	-	-	-	-	-	-	-	-	-	-	-	-	-	-	-	-	-	-	-	-	-	-	-	-
PFK2	phosphofructokinase	-	-	-	-	-	-	-	-	-	-	-	-	179311	-	-	-	-	-	-	-	-	-	-	-	-	-	-	-	-	-	-	-	-	-	-	-	-	-	-	-	-	-	-	-	-	-	-	-	-	-	-
		-	-	-	-	-	-	-	-	-	-	-	-	179305	-	-	-	-	-	-	-	-	-	-	-	-	-	-	-	-	-	-	-	-	-	-	-	-	-	-	-	-	-	-	-	-	-	-	-	-	-	-
		-	-	-	-	-	-	-	-	-	-	-	-	179302	-	-	-	-	-	-	-	-	-	-	-	-	-	-	-	-	-	-	-	-	-	-	-	-	-	-	-	-	-	-	-	-	-	-	-	-	-	-
		-	-	-	-	-	-	-	-	63556	-	-	-	179292	-	-	-	-	-	-	-	-	-	-	-	-	-	-	-	-	-	-	-	-	36590	-	-	-	-	-	-	-	-	-	-	-	-	-	-	-	-	-
		-	+	-	-	-	-	GB14174-PA	AT3G61120.1	-	-	CE25156	78380	179291	394626	-	CMA095C	OTTDARP00000021632	DDB0202276	CG1429-PE	-	6.m00448	ENSGALP00000030858	-	OTTHUMP00000031853	-	77597	-	30837	170111	-	Os08g33488.1	15144	-	-	109598	143579	-	174544	RO3G_06615.1	YPL089C	SPBC317.01	GLEAN3_16168	SINFRUP00000179375	-	-	-	-	22691	-	-	-
		-	+	-	-	-	-	GB11761-PA	-	-	-	CE03799	121659	179289	-	-	-	-	-	-	-	-	-	-	-	-	-	-	-	196609	-	-	11601	-	-	-	-	-	-	RO3G_05554.1	-	-	-	-	-	-	-	-	-	-	-	-
		-	-	-	-	-	-	-	-	-	-	-	-	179287	-	-	-	-	-	-	-	-	-	-	-	-	-	-	-	-	-	-	-	-	-	-	-	-	-	-	-	-	-	-	-	-	-	-	-	-	-	-
		-	-	-	-	-	-	-	-	-	-	-	-	179285	-	-	-	-	-	-	-	-	-	-	-	-	-	-	-	-	-	-	-	-	-	-	-	-	-	-	-	-	-	-	-	-	-	-	-	-	-	-
		-	-	-	-	-	-	-	-	-	-	-	-	179281	-	-	-	-	-	-	-	-	-	-	-	-	-	-	-	-	-	-	-	-	-	-	-	-	-	-	-	-	-	-	-	-	-	-	-	-	-	-
		-	+	-	-	-	-	-	-	-	-	CE21254	178707	179276	368720	-	CMT309C	OTTDARP00000008274	DDB0190263	-	-	117.m00169	ENSGALP00000011487	GL50803_8842	OTTHUMP00000043269	-	123204	-	58785	186301	-	Os08g36774.1	37985	-	-	-	-	-	759183	-	-	-	-	-	116.m00139	-	-	551.m00234	63291	-	Tb927.5.2720	-
		-	-	-	-	-	-	-	-	-	-	-	-	179273	-	-	-	-	-	-	-	-	-	-	-	-	-	-	-	-	-	-	-	-	-	-	-	-	-	-	-	-	-	-	-	-	-	-	-	-	-	-
		-	-	-	-	-	-	-	-	-	-	-	-	179271	-	-	-	-	-	-	-	-	-	-	-	-	-	-	-	-	-	-	-	-	-	-	-	-	-	-	-	-	-	-	-	-	-	-	-	-	-	-
		-	-	-	-	-	-	-	-	-	-	-	-	179255	-	-	-	-	-	-	-	-	-	-	-	-	-	-	-	-	-	-	-	-	-	-	-	-	-	-	-	-	-	-	-	-	-	-	-	-	-	-
		-	+	-	-	-	-	-	AT2G21385.1	-	-	-	-	179251	-	-	-	-	-	-	-	-	-	-	-	-	-	-	-	-	-	Os09g36130.1	35599	-	47477	148298	-	-	195633	-	-	-	-	-	-	2291	-	-	-	-	-	-
		-	-	-	-	-	-	-	-	-	-	-	-	179249	-	-	-	-	-	-	-	-	-	-	-	-	-	-	-	-	-	-	-	-	-	-	-	-	-	-	-	-	-	-	-	-	-	-	-	-	-	-
		-	-	-	-	-	-	-	-	-	-	-	-	179248	-	-	-	-	-	-	-	-	-	-	-	-	-	-	-	-	-	-	-	-	-	-	-	-	-	-	-	-	-	-	-	-	-	-	-	-	-	-
		-	-	-	-	-	-	-	-	-	-	-	-	179245	-	-	-	-	-	-	-	-	-	-	-	-	-	-	-	-	-	-	-	-	-	-	-	-	-	-	-	-	-	-	-	-	-	-	-	-	-	-
		-	-	-	-	-	-	-	-	-	-	-	-	179244	-	-	-	-	-	-	-	-	-	-	-	-	-	-	-	-	-	-	-	-	-	-	-	-	-	-	-	-	-	-	-	-	-	-	-	-	-	-
		-	-	-	-	-	-	-	-	-	-	-	-	179239	-	-	-	-	-	-	-	-	-	-	-	-	-	-	-	-	-	-	-	-	-	-	-	-	-	-	-	-	-	-	-	-	-	-	-	-	-	-
		-	-	-	-	-	-	-	-	-	-	-	-	179236	-	-	-	-	-	-	-	-	-	-	-	-	-	-	-	-	-	-	-	-	-	-	-	-	-	-	-	-	-	-	-	-	-	-	-	-	-	-
		-	-	-	-	-	-	-	-	-	-	-	-	179229	-	-	-	-	-	-	-	-	-	-	-	-	-	-	-	-	-	-	-	-	-	-	-	-	-	-	-	-	-	-	-	-	-	-	-	-	-	-
		-	-	-	-	-	-	-	-	-	-	-	-	179223	-	-	-	-	-	-	-	-	-	-	-	-	-	-	-	-	-	-	-	-	-	-	-	-	-	-	-	-	-	-	-	-	-	-	-	-	-	-
		-	-	-	-	-	-	-	-	-	-	-	-	179218	-	-	-	-	-	-	-	-	-	-	-	-	-	-	-	-	-	-	-	-	-	-	-	-	-	-	-	-	-	-	-	-	-	-	-	-	-	-
		-	-	-	-	-	-	-	-	-	-	-	-	179214	-	-	-	-	-	-	-	-	-	-	-	-	-	-	-	-	-	-	-	-	-	-	-	-	-	-	-	-	-	-	-	-	-	-	-	-	-	-
		-	-	-	-	-	-	-	-	-	-	-	-	179213	-	-	-	-	-	-	-	-	-	-	-	-	-	-	-	-	-	-	-	-	-	-	-	-	-	-	-	-	-	-	-	-	-	-	-	-	-	-
		-	-	-	-	-	-	-	-	-	-	-	-	179212	-	-	-	-	-	-	-	-	-	-	-	-	-	-	-	-	-	-	-	-	-	-	-	-	-	-	-	-	-	-	-	-	-	-	-	-	-	-
		-	+	-	-	-	-	GB10946-PA	AT3G59820.2	2663	BDEG_05156	CE20894	90824	179211	294853	-	CMN072C	OTTDARP00000008769	DDB0205475	CG4589-PC	-	-	ENSGALP00000025284	-	OTTHUMP00000149960	LmjF29.0920	140711	33385	32474	20815	NCU03217	Os02g39550.3	3648	-	3241	188728	128250	PFD0835c	226748	RO3G_01891.1	YOL027C	SPAC23C11.17	-	SINFRUP00000154948	-	32503	TA08985	80.m00032	60795	-	-	UM04169.1
		-	-	-	-	-	-	-	-	-	-	-	-	179210	-	-	-	-	-	-	-	-	-	-	-	-	-	-	-	-	-	-	-	-	-	-	-	-	-	-	-	-	-	-	-	-	-	-	-	-	-	-
		-	-	-	-	-	-	-	-	-	-	-	-	179208	-	-	-	-	-	-	-	-	-	-	-	-	-	-	-	-	-	-	-	-	-	-	-	-	-	-	-	-	-	-	-	-	-	-	-	-	-	-
		-	-	-	-	-	-	-	-	-	-	-	-	179204	-	-	-	-	-	-	-	-	-	-	-	-	-	-	-	-	-	-	-	-	-	-	-	-	-	-	-	-	-	-	-	-	-	-	-	-	-	-
		-	-	-	-	-	-	-	-	-	-	-	-	179202	-	-	-	-	-	-	-	-	-	-	-	-	-	-	-	-	-	-	-	-	-	-	-	-	-	-	-	-	-	-	-	-	-	-	-	-	-	-
		-	-	-	-	-	-	-	-	-	-	-	-	179196	-	-	-	-	-	-	-	-	-	-	-	-	-	-	-	-	-	-	-	-	-	-	-	-	-	-	-	-	-	-	-	-	-	-	-	-	-	-
		-	-	-	-	-	-	-	-	-	-	-	-	179195	-	-	-	-	-	-	-	-	-	-	-	-	-	-	-	-	-	-	-	-	-	-	-	-	-	-	-	-	-	-	-	-	-	-	-	-	-	-
		-	-	-	-	-	-	-	-	-	-	-	-	179194	-	-	-	-	-	-	-	-	-	-	-	-	-	-	-	-	-	-	-	-	-	-	-	-	-	-	-	-	-	-	-	-	-	-	-	-	-	-
CLR4	Coxiella-Like Repeat protein	-	-	-	-	-	-	-	-	-	-	-	-	179193	-	-	-	-	-	-	-	-	-	-	-	-	-	-	-	-	-	-	-	-	-	-	-	-	-	-	-	-	-	-	-	-	-	-	-	-	-	-
		-	-	-	-	-	-	-	-	-	-	-	-	179192	-	-	-	-	-	-	-	-	-	-	-	-	-	-	-	-	-	-	-	-	-	-	-	-	-	-	-	-	-	-	-	-	-	-	-	-	-	-
		-	-	-	-	-	-	-	-	-	-	-	-	179191	-	-	-	-	-	-	-	-	-	-	-	-	-	-	-	246933	-	-	-	-	-	125471	-	-	-	-	-	-	-	-	-	-	-	-	54985	-	-	-
		-	-	-	-	-	-	-	-	-	-	-	-	179189	-	-	-	-	-	-	-	-	-	-	-	-	-	-	-	-	-	-	-	-	-	-	-	-	-	-	-	-	-	-	-	-	-	-	-	-	-	-
		-	-	-	-	-	-	-	-	-	-	-	-	179188	-	-	-	-	-	-	-	-	-	-	-	-	-	-	-	-	-	-	-	-	-	-	-	-	-	-	-	-	-	-	-	-	-	-	-	-	-	-
		-	+	-	-	-	-	GB18001-PA	AT2G17190.1	-	BDEG_06607	CE31480	19493	179183	261637	-	CMD018C	OTTDARP00000021927	DDB0189668	CG14224-PA	-	81.m00168	ENSGALP00000022674	-	OTTHUMP00000018848	-	128630	-	37601	132153	-	Os03g03920.5	24661	-	44323	138051	137944	-	741844	RO3G_09990.1	YMR276W	-	GLEAN3_25405	SINFRUP00000172788	-	21756	TA16530	42.m00022	63757	-	-	-
		-	-	-	-	-	-	-	-	-	-	-	-	179180	-	-	-	-	-	-	-	-	-	-	-	-	-	-	-	-	-	-	-	-	-	-	-	-	-	-	-	-	-	-	-	-	-	-	-	-	-	-
		-	-	-	-	-	-	-	-	-	-	-	-	179179	-	-	-	-	-	-	-	-	-	-	-	-	-	-	-	-	-	-	-	-	-	-	-	-	-	-	-	-	-	-	-	-	-	-	-	-	-	-
		-	-	-	-	-	-	-	-	-	-	-	-	179177	-	-	-	-	-	-	-	-	-	-	-	-	-	-	-	-	-	-	-	-	-	-	-	-	-	-	-	-	-	-	-	-	-	-	-	-	-	-
		-	-	-	-	-	-	-	-	-	-	-	-	179175	-	-	-	-	-	-	-	-	-	-	-	-	-	-	-	-	-	-	-	-	-	-	-	-	-	-	-	-	-	-	-	-	-	-	-	-	-	-
		-	-	-	-	-	-	-	-	-	-	-	-	179162	-	-	-	-	-	-	-	-	-	-	-	-	-	-	-	-	-	-	-	-	-	-	-	-	-	-	-	-	-	-	-	-	-	-	-	-	-	-
		-	-	-	-	-	-	-	-	-	-	-	-	179159	-	-	-	-	-	-	-	-	-	-	-	-	-	-	-	-	-	-	-	-	-	-	-	-	-	-	-	-	-	-	-	-	-	-	-	-	-	-
FAD5b		-	-	-	-	-	-	-	-	-	-	-	-	179158	-	-	-	-	-	-	-	-	-	-	-	-	-	-	-	-	-	-	-	-	-	-	-	-	-	-	-	-	-	-	-	-	-	-	-	-	-	-
		-	-	-	-	-	-	-	AT4G35985.1	-	-	-	-	179155	-	-	-	-	-	-	-	-	-	-	-	-	-	-	-	-	-	-	-	-	-	-	-	-	-	-	-	-	-	-	-	-	-	-	-	-	-	-
		-	-	-	+	-	-	-	-	-	-	-	-	179152	-	-	-	-	-	-	-	-	-	-	-	-	-	-	-	-	-	-	-	-	-	-	-	-	-	-	-	-	-	-	-	-	-	-	-	-	-	-
		-	-	-	-	-	-	-	-	-	-	-	-	179147	-	-	-	-	-	-	-	-	-	-	-	-	-	-	-	-	-	-	-	-	-	-	-	-	-	-	-	-	-	-	-	-	-	-	-	-	-	-
		-	-	-	-	-	-	-	-	-	-	-	-	179146	-	-	-	-	-	-	-	-	-	-	-	-	-	-	-	-	-	-	-	-	-	-	-	-	-	-	-	-	-	-	-	-	-	-	-	-	-	-
		-	-	-	-	-	-	-	-	-	-	-	-	179145	-	-	-	-	-	-	-	-	-	-	-	-	-	-	-	-	-	-	-	-	-	-	-	-	-	-	-	-	-	-	-	-	-	-	-	-	-	-
		-	-	-	-	-	-	-	-	-	-	-	-	179141	-	-	-	-	-	-	-	-	-	-	-	-	-	-	-	-	-	-	-	-	-	-	-	-	-	-	-	-	-	-	-	-	-	-	-	-	-	-
		-	-	-	-	-	-	-	-	66031	-	-	213446	179139	-	-	-	-	-	-	-	-	-	-	-	-	-	-	-	-	-	-	-	-	50417	-	-	-	-	-	-	-	-	-	-	5898	-	-	-	-	-	-
	Ca^{2+}-ATPase	-	-	-	-	-	-	-	-	-	-	-	-	179136	-	-	-	-	-	-	-	-	-	-	-	-	-	-	-	-	-	-	-	-	-	-	-	-	-	-	-	-	-	-	-	-	-	-	-	-	-	-
		-	-	-	-	-	-	-	-	-	-	-	-	179132	-	-	-	-	-	-	-	-	-	-	-	-	-	-	-	-	-	-	-	-	-	-	-	-	-	-	-	-	-	-	-	-	-	-	-	-	-	-
		-	-	-	-	-	-	-	-	-	-	-	-	179131	-	-	-	-	-	-	-	-	-	-	-	-	-	-	-	-	-	-	-	-	-	-	-	-	-	-	-	-	-	-	-	-	-	-	-	-	-	-
		-	-	-	-	-	-	-	-	-	-	-	-	179128	-	-	-	-	-	-	-	-	-	-	-	-	-	-	-	-	-	-	-	-	-	-	-	-	-	-	-	-	-	-	-	-	-	-	-	-	-	-
		-	-	-	-	-	-	-	-	-	-	-	-	179125	-	-	-	-	-	-	-	-	-	-	-	-	-	-	-	-	-	-	-	-	-	-	-	-	-	-	-	-	-	-	-	-	-	-	-	-	-	-
		-	-	-	-	-	-	-	-	-	-	-	-	179124	-	-	-	-	-	-	-	-	-	-	-	-	-	-	-	-	-	-	-	-	-	-	-	-	-	-	-	-	-	-	-	-	-	-	-	-	-	-
		-	-	-	-	-	-	-	-	-	-	-	-	179123	-	-	-	-	-	-	-	-	-	-	-	-	-	-	-	-	-	-	-	-	-	-	-	-	-	-	-	-	-	-	-	-	-	-	-	-	-	-
		-	-	-	-	-	-	-	-	-	-	-	-	179122	-	-	-	-	-	-	-	-	-	-	-	-	-	-	-	-	-	-	-	-	-	-	-	-	-	-	-	-	-	-	-	-	-	-	-	-	-	-
		-	-	-	-	-	-	-	-	-	-	-	-	179121	-	-	-	-	-	-	-	-	-	-	-	-	-	-	-	-	-	-	-	-	-	-	-	-	-	-	-	-	-	-	-	-	-	-	-	-	-	-
		-	-	-	-	-	-	-	-	-	-	-	-	179120	-	-	-	-	-	-	-	-	-	-	-	-	-	-	-	-	-	-	-	-	-	-	-	-	-	-	-	-	-	-	-	-	-	-	-	-	-	-
		-	-	-	-	-	-	-	-	-	-	-	-	179119	-	-	-	-	-	-	-	-	-	-	-	-	-	-	-	-	-	-	-	-	-	-	-	-	-	-	-	-	-	-	-	-	-	-	-	-	-	-
		-	-	-	-	-	-	-	-	-	-	-	-	179118	-	-	-	-	-	-	-	-	-	-	-	-	-	-	-	-	-	-	-	-	-	-	-	-	-	-	-	-	-	-	-	-	-	-	-	-	-	-
		-	-	-	-	-	-	-	-	-	-	-	-	179113	-	-	-	-	-	-	-	-	-	-	-	-	-	-	-	-	-	-	-	-	-	-	-	-	-	-	-	-	-	-	-	-	-	-	-	-	-	-
		-	-	-	-	-	-	-	-	-	-	-	-	179112	-	-	-	-	-	-	-	-	-	-	-	-	-	-	-	-	-	-	-	-	-	-	-	-	-	-	-	-	-	-	-	-	-	-	-	-	-	-
		-	-	-	-	-	-	-	-	-	-	-	-	179107	-	-	-	-	-	-	-	-	-	-	-	-	-	-	-	-	-	-	-	-	-	-	-	-	-	-	-	-	-	-	-	-	-	-	-	-	-	-
		-	-	-	-	-	-	-	-	-	-	-	-	179102	-	-	-	-	-	-	-	-	-	-	-	-	-	-	-	-	-	-	-	-	-	-	-	-	-	-	-	-	-	-	-	-	-	-	-	-	-	-
		-	-	-	-	-	-	-	-	61197	-	-	-	179100	-	-	-	-	-	-	-	-	-	-	-	-	-	-	-	-	-	-	34941	-	-	-	-	-	-	-	-	-	-	-	-	23274	-	-	-	-	-	-
		-	-	-	-	-	-	-	-	-	-	-	-	179099	-	-	-	-	-	-	-	-	-	-	-	-	-	-	-	-	-	-	-	-	-	-	-	-	-	-	-	-	-	-	-	-	-	-	-	-	-	-
		-	-	-	-	-	-	-	-	-	-	-	-	179098	-	-	-	-	-	-	-	-	-	-	-	-	-	-	-	-	-	-	-	-	-	-	-	-	-	-	-	-	-	-	-	-	-	-	-	-	-	-
	hypothetical protein	-	-	-	-	-	-	-	-	-	-	-	-	179094	-	-	-	-	-	-	-	-	-	-	-	-	-	-	-	-	-	-	-	-	-	-	-	-	-	-	-	-	-	-	-	-	-	-	-	-	-	-
		-	-	-	-	-	-	-	-	-	-	-	-	179092	-	-	-	-	-	-	-	-	-	-	-	-	-	-	-	-	-	-	-	-	-	-	-	-	-	-	-	-	-	-	-	-	-	-	-	-	-	-
		-	-	-	-	-	-	-	-	-	-	-	-	179083	-	-	-	-	-	-	-	-	-	-	-	-	-	-	-	-	-	-	-	-	-	-	-	-	-	-	-	-	-	-	-	-	-	-	-	-	-	-
		-	-	-	-	-	-	-	-	-	-	-	-	179080	-	-	-	-	-	-	-	-	-	-	-	-	-	-	-	-	-	-	-	-	-	-	-	-	-	-	-	-	-	-	-	-	-	-	-	-	-	-
		-	-	-	-	-	-	-	-	-	-	-	-	-	-	-	-	-	-	-	-	-	-	-	-	-	-	-	-	-	-	-	-	-	-	-	-	-	-	-	-	-	-	-	-	-	-	-	-	-	-	-
		-	-	-	-	-	-	-	-	-	-	-	-	179074	-	-	-	-	-	-	-	-	-	-	-	-	-	-	-	245738	-	-	-	-	-	-	-	-	-	-	-	-	-	-	-	-	-	-	-	-	-	-
		-	-	-	-	-	-	-	-	-	-	-	-	179060	-	-	-	-	-	-	-	-	-	-	-	-	-	-	-	-	-	-	-	-	-	-	-	-	-	-	-	-	-	-	-	-	-	-	-	-	-	-
		-	-	-	-	-	-	-	-	-	-	-	-	179059	-	-	-	-	-	-	-	-	-	-	-	-	-	-	-	-	-	-	-	-	-	-	-	-	-	-	-	-	-	-	-	-	-	-	-	-	-	-
		-	-	-	-	-	-	-	-	-	-	-	-	179052	-	-	-	-	-	-	-	-	-	-	-	-	-	-	-	-	-	-	-	-	-	-	-	-	-	-	-	-	-	-	-	-	-	-	-	-	-	-
BCS1	putative Ubiquinol-cytochrome c oxidoreductase (Complex III) biogenesis factor	-	+	-	-	-	-	GB16474-PA	AT5G17760.1	67965	BDEG_03024	CE38194	169645	179044	-	-	-	OTTDARP00000021648	DDB0184131	CG4908-PB	-	-	ENSGALP00000037425	-	OTTHUMP00000164147	LmjF22.0940	185574	-	72457	190500	NCU03921	Os12g24320.1	-	-	-	17855	131264	PFF0155w	201239	-	-	SPAC644.07	-	SINFRUP00000128643	-	-	-	49.m00045	25447	-	Tb927.6.2930	-
		-	+	-	-	-	-	-	-	-	BDEG_06414	-	-	179032	-	-	-	-	DDB0202122	-	-	-	-	-	-	-	-	-	-	-	NCU04511	-	-	GSPATP00030668001	-	-	-	-	-	RO3G_10355.1	-	SPBC31F10.16	-	-	180.m00045	-	-	44.m02582	-	-	-	UM04959.1
		-	+	+	-	-	-	GB17688-PA	AT4G13720.1	18344	BDEG_07710	CE25662	225505	179025	243709	cgd4_4150	CMM146C	-	DDB0215619	CG8891-PA	19068838	226.m00068	ENSGALP00000005130	GL50803_7511	OTTHUMP00000030094	LmjF36.4630	-	13033	34772	163483	NCU01441	Os10g31940.1	14811	GSPATP00005189001	44176	185304	108811	MAL7P1.110	814879	-	YJR069C	SPCC830.10	GLEAN3_14507	SINFRUP00000178010	2.m02312	1165	TA17785	20.m03709	21568	87339.m00086	Tb10.6k15.0290	UM02007.1
		-	-	-	-	-	-	-	-	-	-	-	-	179004	-	-	-	-	-	-	-	-	-	-	-	-	-	-	-	-	-	-	-	-	-	-	-	-	-	-	-	-	-	-	-	-	-	-	-	-	-	-
		-	-	-	-	-	-	-	AT2G39140.1	-	-	-	117146	179001	-	-	-	-	DDB0167086	-	-	-	-	-	-	-	-	-	-	-	-	-	-	-	-	-	-	-	820240	-	-	-	-	-	-	-	-	-	-	-	-	-
		-	-	-	-	-	-	-	-	-	-	-	-	178998	-	-	-	-	-	-	-	-	-	-	-	-	-	-	-	-	-	-	-	-	-	-	-	-	-	-	-	-	-	-	-	-	-	-	-	-	-	-
		-	-	-	-	-	-	-	-	-	-	-	-	178994	-	-	-	-	-	-	-	-	-	-	-	-	-	-	-	-	-	-	-	-	-	-	-	-	-	-	-	-	-	-	-	-	-	-	-	-	-	-
		-	-	-	-	-	-	-	AT1G72640.2	-	-	-	-	178990	-	-	-	-	-	-	-	-	-	-	-	-	-	-	-	-	-	Os03g29730.2	-	-	-	122302	-	-	415586	-	-	-	-	-	-	-	-	-	-	-	-	-
		-	-	-	-	-	-	-	-	-	-	-	-	178979	-	-	-	-	-	-	-	-	-	-	-	-	-	-	-	-	-	-	-	-	-	-	-	-	-	-	-	-	-	-	-	-	-	-	-	-	-	-
		-	-	-	-	-	-	-	-	-	-	-	-	178976	-	-	-	-	-	-	-	-	-	-	-	-	-	-	-	-	-	-	34844	-	-	161935	-	-	-	-	-	-	-	-	-	-	-	-	-	-	-	-
		-	+	-	-	-	-	-	AT5G07630.1	-	-	-	-	178966	250496	-	-	-	-	CG3149-PA	-	2.m00515	-	-	-	-	-	-	73590	-	-	Os07g30250.1	32206	-	-	117127	-	-	423550	RO3G_09999.1	-	-	-	-	-	-	-	-	-	-	-	-
		-	+	-	-	-	-	-	AT4G15180.1	-	-	-	-	178960	-	-	-	-	-	-	-	-	-	-	-	-	-	-	-	-	-	Os08g08210.1	17811	GSPATP00035094001	-	216087	-	-	763921	-	-	-	-	-	20.m00342	-	-	-	-	-	-	-
		-	-	-	-	-	-	-	-	-	-	-	-	178953	-	-	-	-	-	-	-	-	-	-	-	-	-	-	-	-	-	-	-	-	-	-	-	-	-	-	-	-	-	-	-	-	-	-	-	-	-	-
		-	-	-	-	-	-	-	-	-	-	-	-	178948	-	-	-	-	-	-	-	-	-	-	-	-	-	-	-	-	-	-	-	-	-	-	-	-	-	-	-	-	-	-	-	-	-	-	-	-	-	-
		-	-	-	-	-	-	-	-	-	-	-	-	178937	-	-	-	-	-	-	-	-	-	-	-	-	-	-	-	-	-	-	-	-	-	-	-	-	-	-	-	-	-	-	-	-	-	-	-	-	-	-

		-	-	-	-	-	-	-	-	-	-	-	-	178936	-	-	-	-	-	-	-	-	-	-	-	-	-	-	-	-	-	-	-	-	-	-	-	-	-	-	-	-	-	-	-	-	-	-	-	-	-	-
		-	-	-	-	-	-	-	-	-	-	-	-	178935	-	-	-	-	-	-	-	-	-	-	-	-	-	-	-	-	-	-	-	-	-	-	-	-	-	-	-	-	-	-	-	-	-	-	-	-	-	-
		-	+	-	-	-	-	GB15289-PA	AT1G56290.1	-	BDEG_05848	CE33549	116793	178934	237253	-	-	-	DDB0167930	CG9213-PA	-	51.m00157	ENSGALP00000027682	-	NP_689647.1	-	191734	1359	51750	180108	NCU04505	Os03g52980.1	34984	GSPATP00037397001	44735	135148	130200	-	823770	RO3G_05212.1	YGR093W	SPAC30D11.09	-	SINFRUP00000159642	19.m00402	10946	TA13015	46.m01640	25069	-	-	UM02692.1
		-	-	-	-	-	-	-	-	-	-	-	-	178915	-	-	-	-	-	-	-	-	-	-	-	-	-	-	-	-	-	-	-	-	-	-	-	-	-	-	-	-	-	-	-	-	-	-	-	-	-	-
APC3	Subunit of anaphase promoting complex	-	+	-	-	-	-	GB16427-PA	AT2G20000.1	-	-	-	112715	178914	-	-	-	-	-	-	-	-	-	-	-	-	141697	-	-	-	-	Os06g41750.1	11248	GSPATP00033101001	-	-	156896	-	835890	-	-	-	-	-	106.m00166	-	-	-	-	-	-	-
		-	-	-	-	-	-	-	-	-	-	-	-	178913	-	-	-	OTTDARP00000022140	-	-	-	-	ENSGALP00000038536	-	-	-	-	-	-	-	NCU06846	-	-	-	-	-	-	-	-	-	-	-	-	-	-	-	-	-	-	-	-	-
		-	-	-	-	-	-	-	-	-	-	-	-	178912	-	-	-	-	-	-	-	-	-	-	-	-	-	-	-	-	-	-	-	-	-	-	-	-	-	-	-	-	-	-	-	-	-	-	-	-	-	-
		-	-	-	-	-	-	-	-	-	-	-	-	178904	-	-	-	-	-	-	-	-	-	-	-	-	-	-	-	-	-	-	-	-	-	-	-	-	-	-	-	-	-	-	-	-	-	-	-	-	-	-
		-	+	-	-	-	-	-	AT5G47970.2	36241	-	-	-	178903	-	cgd2_3570	CMK105C	-	-	-	-	-	-	-	-	-	-	-	-	-	-	Os04g20990.1	3685	-	17115	102618	127943	PFI0920c	557785	-	-	-	-	-	310.m00034	2552	TA19730	-	-	-	-	-
		-	-	-	-	-	-	-	-	-	-	-	-	178899	-	-	-	-	-	-	-	-	-	-	OTTHUMP00000021069	-	-	-	61253	91986	-	-	-	-	-	-	-	-	-	-	-	-	-	SINFRUP00000132821	-	-	-	-	-	-	-	-
		-	-	-	-	-	-	-	-	-	-	-	-	178887	-	-	-	-	-	-	-	-	-	-	-	-	-	-	-	-	-	-	-	-	-	-	-	-	-	-	-	-	-	-	-	-	-	-	-	-	-	-
		-	-	-	-	-	-	-	-	-	-	-	-	178885	-	-	-	-	-	-	-	-	-	-	-	-	-	-	-	-	-	-	-	-	-	-	-	-	-	-	-	-	-	-	-	-	-	-	-	-	-	-
CPC3404,SMC1	Structural Maintenance of Chromosomes protein 1 homolog.	-	+	-	-	-	-	GB17522-PA	AT3G54670.1	70503	-	CE29497	228943	178882	-	-	CMI192C	-	DDB0219689	-	-	10.m00342	-	-	-	LmjF35.3510	188369	-	56724	237840	-	Os12g44390.1	31819	GSPATP00030065001	-	131965	132505	PF11_0317	559701	-	-	SPBC29A10.04	GLEAN3_21629	-	-	35499	-	-	25837	85805.m00062	Tb09.211.2970	-
		-	-	-	-	-	-	-	-	-	-	-	-	178881	-	-	-	-	-	-	-	-	-	-	-	-	-	-	-	-	-	-	-	-	-	-	-	-	-	-	-	-	-	-	-	-	-	-	-	-	-	-
		-	+	-	-	-	-	-	-	-	-	-	-	178874	-	-	-	-	-	-	19168729	-	-	-	-	-	-	-	-	-	NCU07543	-	23226	-	-	-	-	-	-	-	-	SPCC1919.14c	-	-	54.m00181	-	-	-	-	-	-	-
		-	-	-	-	-	-	-	-	-	-	-	-	178864	-	-	-	-	-	-	-	-	-	-	-	-	-	-	-	-	-	-	-	-	-	-	-	-	-	-	-	-	-	-	-	-	-	-	-	-	-	-
		-	-	-	-	-	-	-	-	-	-	-	-	178863	-	-	-	-	-	-	-	-	-	-	-	-	-	-	-	-	-	-	-	-	-	-	-	-	-	-	-	-	-	-	-	-	-	-	-	-	-	-
		-	+	-	-	-	-	-	AT3G57090.1	-	BDEG_03804	-	-	178862	-	-	-	-	-	-	-	-	-	-	-	-	-	-	-	-	-	Os03g24060.1	-	-	-	27579	-	-	825060	-	-	-	-	-	-	-	-	-	-	-	-	-
		-	-	-	-	-	-	-	-	-	-	-	-	178858	-	-	-	-	-	-	-	-	-	-	-	-	-	-	-	-	-	-	-	-	-	-	-	-	-	-	-	-	-	-	-	-	-	-	-	-	-	-
CLR19	Coxiella-Like Repeat protein	-	-	-	-	-	-	-	-	-	-	-	-	178853	-	-	-	-	-	-	-	-	-	-	-	-	-	-	-	-	-	-	-	-	-	-	-	-	-	-	-	-	-	-	-	-	-	-	-	-	-	-
		-	-	-	-	-	-	-	-	-	-	-	-	178852	-	-	-	-	-	-	-	-	-	-	-	-	-	-	-	-	-	-	-	-	-	-	-	-	-	-	-	-	-	-	-	-	-	-	-	-	-	-
		-	-	-	-	-	-	-	-	-	-	-	-	178848	-	-	-	-	-	-	-	-	-	-	-	-	-	-	-	-	-	-	-	-	-	-	-	-	-	-	-	-	-	-	-	-	-	-	-	-	-	-
		-	-	-	-	-	-	-	-	-	-	-	-	178844	-	-	-	-	-	-	-	-	-	-	-	-	-	-	-	-	-	-	-	-	-	-	-	-	-	-	-	-	-	-	-	-	-	-	-	-	-	-
		-	+	-	-	-	-	-	AT4G29940.1	-	-	-	-	178843	-	-	-	-	-	-	-	154.m00129	-	-	-	-	-	-	-	-	-	Os06g12400.1	36515	-	33896	170895	130254	-	826053	-	-	-	-	-	-	24078	-	-	-	-	-	-
		-	-	-	-	-	-	-	-	-	-	-	-	178838	-	-	-	-	-	-	-	-	-	-	-	-	-	-	-	-	-	-	-	-	-	-	-	-	-	-	-	-	-	-	-	-	-	-	-	-	-	-
		-	-	-	-	-	-	-	-	-	-	-	-	178835	-	-	-	-	-	-	-	-	-	-	-	-	-	-	-	-	-	-	-	-	-	-	-	-	-	-	-	-	-	-	-	-	-	-	-	-	-	-
		-	+	-	-	-	-	-	AT5G11800.1	-	-	-	-	178824	-	-	-	-	-	-	-	-	-	-	-	-	-	34373	-	-	-	Os06g36590.2	27555	-	-	130244	-	-	253600	-	-	-	-	-	-	-	-	-	-	-	-	-
		-	-	-	-	-	-	-	-	-	-	-	-	178823	-	-	-	-	-	-	-	-	-	-	-	-	-	-	-	-	-	-	-	-	-	-	-	-	-	-	-	-	-	-	-	-	-	-	-	-	-	-
		-	-	-	-	-	-	-	-	-	-	-	-	178819	-	-	-	-	-	-	-	-	-	-	-	-	-	-	-	-	-	-	-	-	-	-	-	-	-	-	-	-	-	-	-	-	-	-	-	-	-	-
		-	-	-	-	-	-	-	-	-	-	-	-	178817	-	-	-	-	-	-	-	-	-	-	-	-	-	-	-	-	-	-	-	-	-	-	-	-	-	-	-	-	-	-	-	-	-	-	-	-	-	-
		-	+	-	-	-	-	GB17027-PA	AT1G76630.1	-	-	-	188519	178812	-	-	-	-	-	-	-	-	ENSGALP00000023595	-	OTTHUMP00000149977	-	230642	-	-	116212	-	-	14036	-	-	-	-	-	-	-	YPR189W	SPCC1919.05	-	SINFRUP00000140442	-	-	-	-	54351	-	-	-
		-	-	-	-	-	-	-	-	-	-	-	-	178809	-	-	-	-	-	-	-	-	-	-	-	-	-	-	-	-	-	-	-	-	-	-	-	-	-	-	-	-	-	-	-	-	-	-	-	-	-	-
SF3b1	Nuclear pre-mRNA splicing factor, component of splicing factor 3b	-	+	+	-	-	-	GB16777-PA	AT5G64270.1	37713	BDEG_07340	CE03641	154841	178803	279707	cgd6_4750	CMB002C	OTTDARP00000021390	DDB0169544	CG2807-PA	19069615	902.m00008	ENSGALP00000013029	-	NP_036565.2	LmjF28.2570	199416	37521	31740	241911	NCU03042	Os02g05310.4	23425	GSPATP00025933001	16704	175218	109252	PFC0375c	577374	RO3G_07617.1	YMR288W	SPAC27F1.09c	GLEAN3_24101	SINFRUP00000130715	67.m00150	27556	TA03380	20.m03882	54034	91714.m00094	Tb11.01.3690	UM05241.1
		-	-	-	-	-	-	-	-	-	-	-	-	178802	-	-	-	-	-	-	-	-	-	-	-	-	-	-	-	-	-	-	-	-	-	-	-	-	-	-	-	-	-	-	-	-	59.m03701	-	-	-	-
		-	-	-	-	-	-	-	-	-	-	-	-	178800	-	-	-	-	-	-	-	-	-	-	-	-	-	-	-	-	-	-	-	-	-	-	-	-	-	-	-	-	-	-	-	-	-	-	-	-	-	-
		-	-	-	-	-	-	-	-	-	-	-	-	178795	-	-	-	-	-	-	-	-	-	-	-	-	-	-	-	-	-	-	-	-	-	-	-	-	-	-	-	-	-	-	-	-	-	-	-	-	-	-
		-	-	-	-	-	-	-	-	-	-	-	-	178789	-	-	-	-	-	-	-	-	-	-	-	-	-	-	-	-	-	-	-	-	-	-	-	-	-	-	-	-	-	-	-	-	-	-	-	-	-	-
		-	-	-	-	-	-	-	-	-	-	-	-	178787	-	-	-	-	-	-	-	-	-	-	-	-	-	-	-	-	-	-	-	-	-	-	-	-	-	-	-	-	-	-	-	-	-	-	-	-	-	-
		-	-	-	-	-	-	-	-	-	-	-	-	178778	-	-	-	-	-	-	-	-	-	-	-	-	-	-	-	-	-	-	-	-	-	-	-	-	-	-	-	-	-	-	-	-	-	-	-	-	-	-
		-	-	-	-	-	-	-	-	-	-	-	-	178766	-	-	-	-	-	-	-	-	-	-	-	-	-	-	-	-	-	-	-	-	-	-	-	-	-	-	-	-	-	-	-	-	-	-	-	-	-	-
		-	-	-	-	-	-	-	-	-	-	-	-	178763	-	-	-	-	-	-	-	-	-	-	-	-	-	-	-	-	-	-	-	-	-	-	-	-	-	-	-	-	-	-	-	-	-	-	-	-	-	-
		-	-	-	-	-	-	-	-	-	-	-	-	178758	-	-	-	-	-	-	-	-	-	-	-	-	-	-	-	-	-	-	-	-	-	-	-	-	-	-	-	-	-	-	-	-	-	-	-	-	-	-
		-	-	-	-	-	-	-	-	-	-	-	-	178739	-	-	-	-	-	-	-	-	-	-	-	-	-	-	-	-	-	-	-	-	-	-	-	-	-	-	-	-	-	-	-	-	-	-	-	-	-	-
		-	-	-	-	-	-	-	-	-	-	-	-	178737	-	-	-	-	-	-	-	-	-	-	-	-	-	-	-	-	-	-	-	-	-	-	-	-	-	-	-	-	-	-	-	-	-	-	-	-	-	-
		-	-	-	-	-	-	-	-	-	-	-	-	178734	-	-	-	-	-	-	-	-	-	-	-	-	-	-	-	-	-	-	-	-	-	-	-	-	-	-	-	-	-	-	-	-	-	-	-	-	-	-
		-	-	-	-	-	-	-	-	-	-	-	-	178733	-	-	-	-	-	-	-	-	-	-	-	-	-	-	-	-	-	-	-	-	-	-	-	-	-	-	-	-	-	-	-	-	-	-	-	-	-	-
		-	-	-	-	-	-	-	-	-	-	-	-	178725	-	-	-	-	-	-	-	-	-	-	-	-	-	-	-	-	-	-	-	-	-	-	-	-	-	-	-	-	-	-	-	-	-	-	-	-	-	-
		-	-	-	-	-	-	-	AT4G16570.1	-	-	-	-	178720	-	-	-	-	-	-	-	-	-	-	-	-	-	-	-	-	-	Os06g01640.1	-	-	-	-	-	-	-	-	-	-	-	-	-	-	-	-	-	-	-	-
		-	-	-	-	-	-	-	-	-	-	-	-	178718	-	-	-	-	-	-	-	-	-	-	-	-	-	-	-	-	-	-	-	-	-	-	-	-	-	-	-	-	-	-	-	-	-	-	-	-	-	-
		-	-	-	-	-	-	-	-	-	-	-	-	178715	-	-	-	-	-	-	-	-	-	-	-	-	-	-	-	-	-	-	-	-	-	-	-	-	-	-	-	-	-	-	-	-	-	-	-	-	-	-
		-	-	-	-	-	-	-	-	-	-	-	-	178713	-	-	-	-	-	-	-	-	-	-	-	-	-	-	-	-	-	-	-	-	-	-	-	-	-	-	-	-	-	-	-	-	-	-	-	-	-	-
		-	-	-	-	-	-	-	-	-	-	-	-	178708	-	-	-	-	-	-	-	-	-	-	-	-	-	-	-	-	-	-	-	-	-	-	-	-	-	-	-	-	-	-	-	-	-	-	-	-	-	-
		-	-	-	-	-	-	-	-	-	-	CE19001	-	178706	-	-	-	-	-	-	-	-	-	-	-	-	-	-	-	-	-	-	-	-	-	-	-	-	-	-	-	-	-	-	-	-	-	-	-	-	-	-
		-	+	-	-	-	-	-	-	-	-	-	-	178701	-	-	-	OTTDARP00000016601	-	-	-	-	-	-	-	-	-	-	-	-	-	-	-	-	-	-	-	-	565852	RO3G_15692.1	-	-	-	SINFRUP00000144906	-	-	-	-	51955	-	-	-
		-	+	-	-	-	-	GB13550-PA	AT2G32410.2	71360	BDEG_03267	-	19394	178700	372001	-	-	-	DDB0219295	CG7828-PA	-	228.m00062	ENSGALP00000008356	-	NP_001018169.1	-	231724	-	83134	182319	NCU08040	Os03g60550.3	-	-	15355	212439	109228	-	807616	-	-	SPAC323.06c	-	SINFRUP00000168673	-	-	-	-	27487	92719.m00103	-	UM05834.1
		-	+	-	-	-	-	GB16987-PA	AT5G12220.1	68377	-	-	-	178693	-	-	-	-	DDB0168741	-	-	-	-	-	-	-	-	-	-	-	-	-	-	-	-	-	-	-	804410	-	-	-	-	-	-	-	-	-	-	-	-	-
TIG1		-	-	-	-	-	-	-	AT5G55220.1	-	-	-	-	178686	-	-	-	-	-	-	-	-	-	-	-	-	-	-	-	-	-	Os06g20320.1	-	-	-	177197	-	-	251763	-	-	-	-	-	-	-	-	-	-	-	-	-
		-	-	-	-	-	-	-	-	-	-	-	-	178681	-	-	-	-	-	-	-	-	-	-	-	-	-	-	-	-	-	-	-	-	-	-	-	-	-	-	-	-	-	-	-	-	-	-	-	-	-	-
		-	-	-	-	-	-	-	-	-	-	-	-	178680	-	-	-	-	-	-	-	-	-	-	-	-	-	-	-	-	-	-	-	-	-	-	-	-	-	-	-	-	-	-	-	-	-	-	-	-	-	-
		-	-	-	-	-	-	-	-	-	-	-	-	178677	-	-	-	-	-	-	-	-	-	-	-	-	-	-	-	-	-	-	-	-	-	-	-	-	-	-	-	-	-	-	-	-	-	-	-	-	-	-
		-	-	-	-	-	-	-	-	-	-	-	-	178675	-	-	-	-	-	-	-	-	-	-	-	-	-	-	-	-	-	-	-	-	-	-	-	-	-	-	-	-	-	-	-	-	-	-	-	-	-	-
		-	-	-	-	-	-	-	-	-	-	-	-	178673	-	-	-	-	-	-	-	-	-	-	-	-	-	-	-	-	-	-	-	-	-	-	-	-	-	-	-	-	-	-	-	-	-	-	-	-	-	-
		-	-	-	-	-	-	-	-	-	-	-	-	178671	-	-	-	-	-	-	-	-	-	-	-	-	-	-	-	-	-	-	-	-	-	-	-	-	-	-	-	-	-	-	-	-	-	-	-	-	-	-
		-	-	-	-	-	-	-	-	-	-	-	-	178667	-	-	-	-	-	-	-	-	-	-	-	-	-	-	-	-	-	-	-	-	-	-	-	-	-	-	-	-	-	-	-	-	-	-	-	-	-	-
AMX2	copper amine oxidase	-	-	-	-	-	-	-	-	-	-	-	-	178662	-	-	-	-	-	-	-	-	-	-	-	-	-	-	-	-	-	-	-	-	-	-	-	-	-	-	-	-	-	-	-	-	-	-	-	-	-	-
		-	+	-	-	-	-	GB10500-PA	AT5G52820.1	36560	BDEG_08189	CE17294	179885	178661	236886	cgd2_330	CMM029C	-	-	CG2863-PA	19168670	50.m00165	ENSGALP00000007463	GL50803_13667	OTTHUMP00000163848	LmjF24.2260	209088	34170	38666	136836	NCU00794	Os10g01540.1	13229	GSPATP00014623001	22868	160972	122583	PF11_0471	650010	RO3G_09394.1	YCR072C	SPCC18.05c	-	SINFRUP00000141396	171.m00067	42152	TA07639	33.m01394	28728	92649.m00054	Tb927.8.5990	UM03609.1
		-	-	-	-	-	-	-	-	-	-	-	-	178660	-	-	-	-	-	-	-	-	-	-	-	-	-	-	-	-	-	-	-	-	-	-	-	-	-	-	-	-	-	-	-	-	-	-	-	-	-	-
		-	-	-	-	-	-	-	-	-	-	-	-	178654	-	-	-	-	-	-	-	-	-	-	-	-	-	-	-	-	-	-	-	-	-	-	-	-	-	-	-	-	-	-	-	-	-	-	-	-	-	-
		-	-	-	-	-	-	-	-	-	-	-	-	178652	-	-	-	-	-	-	-	-	-	-	-	-	-	-	-	-	-	-	-	-	-	-	-	-	-	-	-	-	-	-	-	-	-	-	-	-	-	-
		-	-	-	-	-	-	-	-	-	-	-	-	178645	-	-	-	-	-	-	-	-	-	-	-	-	-	-	-	-	-	-	-	-	-	-	-	-	-	-	-	-	-	-	-	-	-	-	-	-	-	-
LhcbM1		-	-	-	-	-	-	-	AT2G05070.1	-	-	-	-	178631	-	-	-	-	-	-	-	-	-	-	-	-	-	-	-	-	-	Os03g39610.1	-	-	-	173457	-	-	830495	-	-	-	-	-	-	-	-	-	-	-	-	-
		-	-	-	-	-	-	-	-	-	-	-	-	178596	-	-	-	-	-	-	-	-	-	-	-	-	-	-	-	-	-	-	-	-	-	-	-	-	-	-	-	-	-	-	-	-	-	-	-	-	-	-
		-	-	-	-	-	-	-	-	65268	-	-	-	178589	-	-	-	-	-	-	-	-	-	-	-	-	-	-	-	-	-	-	-	-	-	-	-	-	-	-	-	-	-	-	-	-	-	-	-	-	-	-
MSCL4	protein of unknown function with mechanosensitive-ion-channel-like domain	-	+	-	-	-	-	-	AT1G58200.2	19147	-	-	-	178587	-	-	CMP261C	-	-	-	-	-	-	-	-	-	-	-	-	-	-	Os03g31839.1	-	-	44327	22204	-	-	219693	-	-	-	-	-	-	10403	-	-	63009	-	-	-
		-	+	-	-	-	-	GB18321-PA	AT1G61280.1	-	BDEG_03088	-	-	178585	-	-	-	-	DDB0168617	-	-	-	ENSGALP00000025807	-	NP_710148.1	-	193093	-	-	-	-	-	-	GSPATP00030989001	-	-	-	-	-	RO3G_01980.1	-	-	-	-	-	-	-	-	-	-	-	-
		-	+	-	-	-	-	GB17653-PA	AT5G57120.1	-	BDEG_05166	-	227369	178578	-	-	-	OTTDARP00000021288	-	CG7421-PB	-	-	ENSGALP00000008926	-	OTTHUMP00000020356	-	236060	-	-	-	-	Os12g41930.1	24672	-	50527	233976	130718	-	652849	RO3G_12554.1	YKR092C	SPBC1711.05	-	SINFRUP00000127549	-	23336	TA17815	25.m00202	59313	-	-	-
		-	+	-	-	-	-	-	AT3G62010.1	-	BDEG_07096	-	103765	178567	-	-	-	-	-	-	-	-	ENSGALP00000007093	-	NP_055246.1	LmjF23.1680	210442	-	-	-	-	Os03g38970.1	-	-	-	107526	131937	-	644705	RO3G_00474.1	-	-	-	SINFRUP00000142494	-	-	-	-	59685	-	Tb927.8.3410	UM06016.1
		-	-	-	-	-	-	-	-	-	-	-	-	178562	-	-	-	-	-	-	-	-	-	-	-	-	-	-	-	-	-	-	-	-	-	-	-	-	-	-	-	-	-	-	-	-	-	-	-	-	-	-
		-	-	-	-	-	-	-	-	-	-	-	-	178555	-	-	-	-	-	-	-	-	-	-	-	-	-	-	-	-	-	-	-	-	-	-	-	-	-	-	-	-	-	-	-	-	-	-	-	-	-	-
		-	-	-	-	-	-	-	-	-	-	-	-	178548	-	-	-	-	-	-	-	-	-	-	-	-	-	-	-	128303	-	-	-	-	-	-	-	-	-	-	-	-	-	-	-	-	-	-	-	-	-	-
HTR5	Histone H3	-	-	-	-	-	-	-	-	-	-	-	-	-	-	-	-	-	-	-	-	-	-	-	-	-	-	-	-	-	-	-	-	-	-	-	-	-	-	-	-	-	-	-	-	-	-	-	-	-	-	-
		-	-	-	-	-	-	-	-	-	-	-	-	178536	-	-	-	-	-	-	-	-	-	-	-	-	-	32137	-	-	-	-	-	-	-	-	-	-	-	-	-	-	-	-	-	-	-	-	-	-	-	-
		-	+	-	-	-	-	GB13394-PA	AT1G26660.1	-	BDEG_04740	-	1365	178535	209915	-	-	OTTDARP00000023158	DDB0218702	-	-	-	-	-	OTTHUMP00000023226	-	-	-	-	140887	-	Os07g40460.1	13515	-	47237	130918	138376	-	820499	-	-	-	-	SINFRUP00000176542	-	-	-	-	-	-	-	-
		-	-	-	-	-	-	-	-	-	-	-	-	178520	-	-	-	-	-	-	-	-	-	-	-	-	-	-	-	-	-	-	-	-	-	-	-	-	-	-	-	-	-	-	-	-	-	-	-	-	-	-
		-	-	-	-	-	-	-	-	-	-	-	-	178519	-	-	-	-	-	-	-	-	-	-	-	-	-	-	-	-	-	-	-	-	-	-	-	-	-	-	-	-	-	-	-	-	-	-	-	-	-	-
		-	-	-	-	-	-	-	-	-	-	-	-	178518	-	-	-	-	-	-	-	-	-	-	-	-	-	-	-	-	-	-	-	-	-	-	-	-	-	-	-	-	-	-	-	-	-	-	-	-	-	-
		-	+	-	-	-	-	GB15245-PA	-	16464	-	CE20819	-	178513	-	-	-	-	-	-	-	-	ENSGALP00000014368	-	-	-	-	-	-	100694	-	-	7644	-	11781	-	-	PF13_0272	-	-	-	-	-	SINFRUP00000143840	-	18503	-	50.m00058	64389	-	-	-
		-	-	-	-	-	-	-	-	-	-	-	-	178508	-	-	-	-	-	-	-	-	-	-	-	-	-	-	-	-	-	-	36527	-	-	-	-	-	-	-	-	-	-	-	-	-	-	-	-	-	-	-
	Likely di or tri-carboxylate translocator (oxoglutarate/malate translocator)	-	-	-	-	-	-	-	-	-	-	-	-	-	-	-	-	-	-	-	-	-	-	-	-	-	-	-	-	-	-	-	-	-	-	-	-	-	-	-	-	-	-	-	-	-	-	-	-	-	-	-
HTR22	Histone H3	-	-	-	-	-	-	-	-	-	-	-	-	-	-	-	-	-	-	-	-	-	-	-	-	-	-	-	-	-	-	-	-	-	-	-	-	-	-	-	-	-	-	-	-	-	-	-	-	-	-	-
		-	-	-	-	-	-	-	-	-	-	-	-	178493	-	-	-	-	-	-	-	-	-	-	-	-	-	-	-	-	-	-	-	-	-	-	-	-	-	-	-	-	-	-	-	-	-	-	-	-	-	-
		-	-	-	-	-	-	-	-	-	-	-	-	178474	-	-	-	-	-	-	-	-	-	-	-	-	-	-	-	-	-	-	-	-	-	-	-	-	-	-	-	-	-	-	-	-	-	-	-	-	-	-
		-	-	-	-	-	-	-	-	-	-	-	-	178466	-	-	-	-	-	-	-	-	-	-	-	-	-	-	-	-	-	-	-	-	-	-	-	-	-	-	-	-	-	-	-	-	-	-	-	-	-	-
		-	+	-	-	-	-	GB16154-PA	AT4G31420.2	9680	BDEG_08502	CE04004	174182	178439	290062	-	-	-	DDB0188691	CG6769-PA	-	219.m00081	-	GL50803_13007	OTTHUMP00000115678	-	238085	17002	-	184519	NCU04022	Os06g47840.1	24006	GSPATP00008101001	47919	113536	128477	-	835863	RO3G_02131.1	-	SPCC550.15c	-	-	10.m00467	22203	-	-	18203	-	-	UM02439.1
		-	-	-	-	-	-	-	-	-	-	-	-	178411	-	-	-	-	-	-	-	-	-	-	-	-	-	-	-	-	-	-	-	-	-	-	-	-	-	-	-	-	-	-	-	-	-	-	-	-	-	-
		-	-	-	-	-	-	-	-	-	-	-	-	178410	-	-	-	-	-	-	-	-	-	-	-	-	-	-	-	-	-	-	-	-	-	-	-	-	-	-	-	-	-	-	-	-	-	-	-	-	-	-
		-	+	-	-	-	-	GB15099-PA	AT3G04820.1	-	BDEG_02386	CE05155	160486	178406	-	cgd2_1510	-	-	DDB0216877	-	-	-	ENSGALP00000013183	GL50803_6702	NP_061915.2	-	113644	-	79094	139787	NCU06428	Os01g56620.1	19446	-	-	117182	127182	-	294776	-	YOR243C	SPBC1A4.09	-	SINFRUP00000134025	-	22356	-	-	23629	-	-	-
		-	-	-	-	-	-	-	-	-	-	-	-	178399	-	-	-	-	-	-	-	-	-	-	-	-	-	-	-	-	-	-	-	-	-	-	-	-	-	-	-	-	-	-	-	-	-	-	-	-	-	-
		-	-	-	-	-	-	-	-	-	-	-	-	178392	-	-	-	-	-	-	-	-	-	-	-	-	-	-	-	-	-	-	-	-	-	-	-	-	-	-	-	-	-	-	-	-	-	-	-	-	-	-
		-	-	-	-	-	-	-	-	-	-	-	-	178390	-	-	-	-	-	-	-	-	-	-	-	-	-	-	-	-	-	-	-	-	-	-	-	-	-	-	-	-	-	-	-	-	-	-	-	-	-	-
		-	-	-	-	-	-	-	-	-	-	-	-	-	-	-	-	-	-	-	-	-	-	-	-	-	-	-	-	-	-	-	-	-	-	-	-	-	-	-	-	-	-	-	-	-	-	-	-	-	-	-
		-	-	-	-	-	-	-	-	-	-	-	-	178385	-	-	-	-	-	-	-	-	-	-	-	-	-	-	-	-	-	-	-	-	-	-	-	-	-	-	-	-	-	-	-	-	-	-	-	-	-	-
		-	-	-	-	-	-	-	-	-	-	-	-	178384	-	-	-	-	-	-	-	-	-	-	-	-	-	-	-	-	-	-	-	-	-	-	-	-	-	-	-	-	-	-	-	-	-	-	-	-	-	-
		-	-	-	-	-	-	-	-	-	-	-	-	178374	-	-	-	-	-	-	-	-	-	-	-	-	-	-	-	-	-	-	-	-	-	-	-	-	-	-	-	-	-	-	-	-	-	-	-	-	-	-
NEP1		-	+	+	-	-	-	GB15063-PA	AT3G57000.1	7809	BDEG_00055	CE19133	161336	178371	207004	cgd4_2840	CMC063C	-	DDB0168247	CG3527-PA	19068449	3.m00667	ENSGALP00000023447	GL50803_5786	NP_006322.3	LmjF16.1390	208870	15167	60179	166546	NCU02428	Os02g18830.1	37777	GSPATP00008918001	11653	46413	158605	PF08_0041	831805	RO3G_14073.1	YLR186W	SPAC18G6.07c	-	SINFRUP00000156102	32.m00254	36986	TA18685	583.m09177	30169	-	Tb927.8.5040	UM06195.1
		-	-	-	-	-	-	-	-	-	-	-	-	178367	-	-	-	-	-	-	-	-	-	-	-	-	-	-	-	-	-	-	-	-	-	-	-	-	-	-	-	-	-	-	-	-	-	-	-	-	-	-
		-	-	-	-	-	-	-	-	-	-	-	-	178366	-	-	-	-	-	-	-	-	-	-	-	-	-	-	-	-	-	-	-	-	-	-	-	-	-	-	-	-	-	-	-	-	-	-	-	-	-	-
		-	-	-	-	-	-	-	-	-	-	-	-	178363	-	-	-	-	-	-	-	-	-	-	-	-	-	-	-	-	-	-	-	-	-	-	-	-	-	-	-	-	-	-	-	-	-	-	-	-	-	-
		-	+	-	-	-	-	-	-	-	-	-	-	178361	-	-	-	-	-	-	-	-	ENSGALP00000023963	-	XP_001128291.1	-	123828	-	81181	-	-	-	-	-	-	172314	-	-	-	-	-	-	-	SINFRUP00000128250	-	-	-	-	-	94489.m00097	-	-
		-	-	-	-	-	-	-	-	-	-	-	-	178357	-	-	-	-	-	-	-	-	-	-	-	-	-	-	-	-	-	-	-	-	-	-	-	-	-	-	-	-	-	-	-	-	-	-	-	-	-	-
		-	-	-	-	-	-	-	-	-	-	-	-	178353	-	-	-	-	-	-	-	-	-	-	-	-	-	-	-	-	-	Os03g21630.1	4783	-	-	-	-	-	819241	-	-	-	-	-	-	-	-	-	-	-	-	-
		-	-	-	-	-	-	-	-	-	-	-	-	178345	-	-	-	-	-	-	-	-	-	-	-	-	-	-	-	-	-	-	-	-	-	-	-	-	-	-	-	-	-	-	-	-	-	-	-	-	-	-
		-	-	-	-	-	-	-	-	-	-	-	-	178343	-	-	-	-	-	-	-	-	-	-	-	-	-	-	-	-	-	-	-	-	-	-	-	-	-	-	-	-	-	-	-	-	-	-	-	-	-	-
		-	-	-	-	-	-	-	-	-	-	-	-	178337	-	-	-	-	-	-	-	-	-	-	-	-	-	-	-	-	-	-	-	-	-	-	-	-	-	-	-	-	-	-	-	-	-	-	-	-	-	-
		-	-	-	+	-	-	-	-	-	-	-	-	178333	-	-	-	-	-	-	-	-	-	-	-	-	-	-	-	-	-	-	-	-	-	-	-	-	-	-	-	-	-	-	-	-	-	-	-	-	-	-
		-	-	-	-	-	-	-	-	-	-	-	-	178322	-	-	-	-	-	-	-	-	-	-	-	-	-	-	-	-	-	-	-	-	-	-	-	-	-	-	-	-	-	-	-	-	-	-	-	-	-	-
SCC3	Cohesin complex subunit.	-	+	-	-	-	-	GB20029-PA	AT2G47980.1	-	BDEG_03735	CE05664	182123	178320	259991	-	CMO331C	-	DDB0185626	CG3423-PA	-	-	ENSGALP00000013808	-	OTTHUMP00000081755	-	113757	-	51437	20215	NCU01247	Os05g09620.1	23222	-	-	143170	157789	-	246523	RO3G_04056.1	YIL026C	SPAC17H9.20	-	SINFRUP00000160407	-	-	-	-	55216	-	Tb10.70.0870	UM02053.1
		-	-	-	-	-	-	-	-	-	-	-	-	178318	-	-	-	-	-	-	-	-	-	-	-	-	-	-	-	-	-	-	-	-	-	-	-	PF14_0112	-	-	-	-	-	-	-	-	TA20960	55.m04962	-	-	-	-
		-	-	-	-	-	-	-	-	-	-	-	-	178313	-	-	-	-	-	-	-	-	-	-	-	-	-	-	-	-	-	-	-	-	-	-	-	-	-	-	-	-	-	-	-	-	-	-	-	-	-	-
		-	-	-	-	-	-	-	-	-	-	-	-	178312	-	-	-	-	-	-	-	-	-	-	-	-	-	-	-	-	-	-	-	-	-	-	-	-	-	-	-	-	-	-	-	-	-	-	-	-	-	-
		-	+	-	-	-	-	-	-	10968	-	-	127783	178301	-	-	-	-	-	-	-	-	-	-	-	LmjF13.0090	-	-	80579	-	-	-	13571	-	45064	103467	-	-	-	-	-	-	-	-	-	37838	-	-	-	-	Tb11.02.0100	-
		-	-	-	-	-	-	-	-	-	-	-	-	178294	-	-	-	-	-	-	-	-	-	-	-	-	-	-	-	-	-	-	-	-	-	-	-	-	-	-	-	-	-	-	-	-	-	-	-	-	-	-
		-	-	-	-	-	-	-	-	-	-	-	-	178293	-	-	-	-	-	-	-	-	-	-	-	-	-	-	-	-	-	-	-	-	-	-	-	-	-	-	-	-	-	-	-	-	-	-	-	-	-	-
		-	+	-	-	-	-	-	AT1G55915.1	-	BDEG_08142	-	-	178289	-	-	-	-	-	-	-	-	-	-	-	LmjF32.1310	-	-	-	-	NCU02060	Os06g09170.2	-	-	-	128879	130447	PF10_0092	563478	-	YHR134W	-	-	-	-	7220	-	-	-	-	Tb11.01.6130	-
		-	-	-	-	-	-	-	-	-	-	-	-	178285	-	-	-	-	-	-	-	-	-	-	-	-	-	-	-	-	-	-	-	-	-	-	-	-	-	-	-	-	-	-	-	-	-	-	-	-	-	-
		-	+	-	-	-	-	GB16400-PA	AT5G51280.1	21789	BDEG_01748	CE23832	148841	178281	292627	cgd7_4600	-	-	DDB0187443	CG14637-PA	-	-	ENSGALP00000004785	-	OTTHUMP00000161451	-	163615	10818	1087	158506	-	Os02g05660.1	-	GSPATP00034714001	12990	221558	108692	PFE1390w	810609	RO3G_01360.1	-	-	GLEAN3_17117	SINFRUP00000128198	164.m00084	269450	TA12495	26.m00241	51106	-	-	UM04587.1
	SET domain containing protein, putative histone methyltransferase	-	-	-	-	-	-	-	-	-	-	-	-	178280	-	-	-	-	-	-	-	-	-	-	-	-	-	-	-	-	-	-	-	-	-	-	-	-	-	-	-	-	-	-	-	-	-	-	-	-	-	-
		-	-	-	-	-	-	-	-	-	-	-	-	178277	-	-	-	-	-	-	-	-	-	-	-	-	-	-	-	-	-	-	-	-	-	-	-	-	-	-	-	-	-	-	-	-	-	-	-	-	-	-
		-	-	-	-	-	-	-	-	-	-	-	-	178273	-	-	-	-	-	-	-	-	-	-	-	-	-	-	-	-	-	-	-	-	-	-	-	-	-	-	-	-	-	-	-	-	-	-	-	-	-	-
		-	+	-	-	-	-	-	AT4G21070.1	-	-	CE31438	99768	178271	-	cgd7_100	-	-	-	-	-	-	ENSGALP00000004383	-	OTTHUMP00000069824	-	174527	-	65872	-	-	Os05g43610.1	33962	GSPATP00019312001	49232	-	138292	PFB0440c	550126	-	-	-	GLEAN3_04827	SINFRUP00000128538	113.m00091	11354	-	-	-	-	Tb11.01.0860	UM03267.1
		-	-	-	-	-	-	-	-	-	-	-	-	178269	-	-	-	-	-	-	-	-	-	-	-	-	-	-	-	-	-	-	-	-	-	-	-	-	-	-	-	-	-	-	-	-	-	-	-	-	-	-
		-	-	-	-	-	-	-	-	-	-	-	-	178264	-	-	-	-	-	-	-	-	-	-	-	-	-	-	-	-	-	-	-	-	-	-	-	-	-	-	-	-	-	-	-	-	-	-	-	-	-	-
		-	-	-	-	-	-	-	-	-	-	-	-	178262	-	-	-	-	-	-	-	-	-	-	-	-	-	-	-	-	-	-	-	-	-	-	-	-	-	-	-	-	-	-	-	-	-	-	-	-	-	-
		-	-	-	-	-	-	-	-	-	-	-	-	178261	-	-	-	-	-	-	-	-	-	-	-	-	-	-	-	-	-	-	-	-	-	-	-	-	-	-	-	-	-	-	-	-	-	-	-	-	-	-
		-	-	-	-	-	-	-	-	-	-	-	-	178253	-	-	-	-	-	-	-	-	-	-	-	-	-	-	-	-	-	-	-	-	-	-	-	-	-	-	-	-	-	-	-	-	-	-	-	-	-	-
TWI1	Similar to D. rerio cystic kidney disease gene twister	+	+	-	-	-	-	-	-	26390	BDEG_01480	-	158550	178244	276785	-	-	OTTDARP00000020500	-	CG5048-PA	-	-	ENSGALP00000007762	GL50803_14328	OTTHUMP00000023804	-	182066	-	64485	182461	-	-	-	GSPATP00015465001	-	82265	-	-	-	-	-	-	-	GLEAN3_25629	SINFRUP00000179903	122.m00090	-	-	50.m03162	63939	84285.m00201	-	-
		-	-	-	-	-	-	-	-	-	-	-	-	178235	-	-	-	-	-	-	-	-	-	-	-	-	-	-	-	-	-	-	-	-	-	-	-	-	-	-	-	-	-	-	-	-	-	-	-	-	-	-
AMD,AMD1	alpha-1,2-mannosidase	-	+	-	-	-	-	GB18388-PA	AT1G30000.1	71340	-	CE09078	191990	178232	250705	-	CMF045C	OTTDARP00000006069	DDB0190008	CG32684-PG	-	-	ENSGALP00000014758	-	NP_057303.1	-	120396	13888	33949	13029	NCU02778	Os05g11850.1	1744	-	52346	210011	118482	-	831216	RO3G_11920.1	YJR131W	SPAC2E1P5.01c	GLEAN3_00383	SINFRUP00000131191	-	34968	-	-	25192	90563.m00069	Tb927.8.2940	UM02227.1
		-	+	-	-	-	-	GB13038-PA	AT2G26350.1	-	-	-	174511	178228	278748	-	CMT012C	OTTDARP00000022226	-	CG7864-PA	-	436.m00052	ENSGALP00000001853	GL50803_8731	NP_002608.1	LmjF25.2290	237439	-	65721	229451	NCU03277	Os07g41800.1	36442	GSPATP00000200001	47516	194754	-	-	717083	-	YDR265W	SPBC17A3.10	-	SINFRUP00000148663	53.m00183	260886	-	-	57553	-	Tb927.3.2410	UM02641.1
		-	-	-	-	-	-	-	-	-	-	-	-	178225	-	-	-	-	-	-	-	-	-	-	-	-	-	-	-	-	-	-	-	-	-	-	-	-	-	-	-	-	-	-	-	-	-	-	-	-	-	-
		-	-	-	-	-	-	-	-	-	-	-	-	178219	-	-	-	-	-	-	-	-	-	-	-	-	-	-	-	-	-	-	-	-	-	-	-	-	-	-	-	-	-	-	-	-	-	-	-	-	-	-
		-	-	-	-	-	-	-	-	-	-	-	-	178218	-	-	-	-	-	-	-	-	-	-	-	-	-	-	-	-	-	-	-	-	-	-	-	-	-	-	-	-	-	-	-	-	-	-	-	-	-	-
		-	-	-	-	-	-	-	AT5G64150.1	-	-	-	-	178217	-	-	-	-	-	-	-	-	-	-	-	-	-	-	-	-	-	-	15420	-	-	15168	-	-	647438	-	-	-	-	-	-	-	-	-	-	-	-	-
		-	-	-	-	-	-	-	-	-	-	-	-	178216	-	-	-	-	-	-	-	-	-	-	-	-	-	-	-	-	-	-	-	-	-	-	-	-	-	-	-	-	-	-	-	-	-	-	-	-	-	-
		-	-	-	-	-	-	GB17151-PA	-	-	-	-	-	178213	-	-	-	-	-	-	-	-	-	-	-	-	-	-	-	-	-	-	-	-	-	-	-	-	-	-	-	-	-	SINFRUP00000161669	-	-	-	-	-	-	-	-
		-	-	-	-	-	-	-	-	-	-	-	-	178211	-	-	-	-	-	-	-	-	-	-	-	-	-	-	-	-	-	-	-	-	-	-	-	-	-	-	-	-	-	-	-	-	-	-	-	-	-	-
		-	+	-	-	-	-	-	AT5G03900.2	-	-	-	-	178204	-	-	CMQ153C	-	-	-	-	-	-	-	-	-	-	-	-	-	-	Os02g33010.1	16790	-	11883	2268	-	-	198404	-	-	-	-	-	-	263883	-	-	-	-	-	-
		-	+	-	-	-	-	-	AT4G17620.1	37889	BDEG_01602	CE11044	186717	178203	-	-	-	-	-	CG9125-PA	-	-	-	-	OTTHUMP00000014849	-	54612	-	-	3946	NCU04191	Os05g32580.1	31182	-	-	181621	129199	-	836316	RO3G_11438.1	YGL246C	SPAC19D5.06c	-	SINFRUP00000171070	-	-	-	-	-	-	-	UM05956.1
		-	-	-	-	-	-	-	-	-	-	-	-	178198	-	-	-	-	-	-	-	-	-	-	-	-	-	-	-	-	-	-	-	-	-	-	-	-	-	-	-	-	-	-	-	-	-	-	-	-	-	-
POLI1	DNA polymerase eta/iota	-	+	-	-	-	-	-	AT5G44740.2	26642	BDEG_07557	CE40206	172544	178197	211312	cgd4_3920	CMM284C	OTTDARP00000023970	-	CG7143-PA	-	-	ENSGALP00000037252	-	-	LmjF21.0630	-	-	-	101604	NCU01936	Os01g55300.4	12867	GSPATP00007370001	44910	164441	-	-	413203	RO3G_00117.1	YDR419W	SPBC16A3.11	-	-	-	24019	-	46.m01619	-	-	Tb10.70.6190	UM00101.1
		-	+	-	-	-	-	GB10744-PA	AT5G56900.2	-	BDEG_01685	CE19798	168674	178196	230559	-	-	OTTDARP00000008144	DDB0218683	CG7741-PA	-	-	-	-	NP_060764.3	-	205527	16291	-	85587	-	-	10328	-	49146	176001	135404	-	589197	RO3G_06173.1	-	SPAC1F3.09	GLEAN3_12076	-	-	-	-	-	50726	-	-	-
		-	-	-	-	-	-	-	-	-	-	-	-	178194	-	-	-	-	-	-	-	-	-	-	-	-	-	-	-	-	-	-	-	-	-	-	-	-	-	-	-	-	-	-	-	-	-	-	-	-	-	-
		-	-	-	-	-	-	-	-	-	-	-	-	178193	-	-	-	-	-	-	-	-	-	-	-	-	-	-	-	-	-	-	-	-	-	-	-	-	-	-	-	-	-	-	-	-	-	-	-	-	-	-
		-	-	-	-	-	-	-	-	-	-	-	-	178192	-	-	-	-	-	-	-	-	-	-	-	-	-	-	-	213762	-	Os01g24480.1	-	-	-	88601	-	-	-	-	-	-	-	-	-	-	-	-	-	-	-	-
		-	+	-	-	-	-	-	AT2G26590.3	-	-	CE30640	-	178186	249150	-	-	OTTDARP00000021989	-	-	-	-	ENSGALP00000008336	-	OTTHUMP00000031471	-	-	-	-	-	-	Os05g51640.1	35609	-	-	148926	-	-	560698	RO3G_15490.1	-	SPBC342.04	-	SINFRUP00000130299	192.m00068	21911	-	-	-	-	-	-
		-	-	-	-	-	-	-	-	-	-	-	-	178184	-	-	-	-	-	-	-	-	-	-	-	-	-	-	-	-	-	-	-	-	-	-	-	-	-	-	-	-	-	-	-	-	-	-	-	-	-	-

FAP107	Flagellar Associated Protein	-	-	-	+	-	-	-	-	-	-	-	-	178177	-	-	-	-	-	-	-	-	-	-	-	-	-	-	-	-	-	-	-	-	-	-	-	-	-	-	-	-	-	-	-	-	-	-	-	-	-	-
		-	-	-	-	-	-	-	-	-	-	-	-	178170	-	-	-	-	-	-	-	-	-	-	-	-	-	-	-	-	-	-	-	-	-	-	-	-	-	-	-	-	-	-	-	-	-	-	-	-	-	-
		-	-	-	-	-	-	-	-	-	-	-	-	178161	-	-	-	-	-	-	-	-	-	-	-	-	-	-	-	-	-	-	-	-	-	-	-	-	-	-	-	-	-	-	-	-	-	-	-	-	-	-
		-	-	-	-	-	-	-	-	-	-	-	-	178159	-	-	-	-	-	-	-	-	-	-	-	-	-	-	-	-	-	-	-	-	-	-	-	-	-	-	-	-	-	-	-	-	-	-	-	-	-	-
		-	-	-	-	-	-	-	-	-	-	-	-	178158	-	-	-	-	-	-	-	-	-	-	-	-	-	-	-	-	-	-	-	-	-	-	-	-	-	-	-	-	-	-	-	-	-	-	-	-	-	-
		-	-	-	-	-	-	-	-	-	-	-	-	178155	-	-	-	-	-	-	-	-	-	-	-	-	-	-	-	-	-	-	-	-	-	-	-	-	-	-	-	-	-	-	-	-	-	-	-	-	-	-
		-	-	-	-	-	-	-	-	-	-	-	-	178147	-	-	-	-	-	-	-	-	-	-	-	-	-	-	-	-	-	-	-	-	-	-	-	-	-	-	-	-	-	-	-	-	-	-	-	-	-	-
		-	-	-	-	-	-	-	-	-	-	-	-	178146	-	-	-	-	-	-	-	-	-	-	-	-	-	-	-	-	-	-	-	-	-	-	-	-	-	-	-	-	-	-	-	-	-	-	-	-	-	-
		-	-	-	-	-	-	-	-	-	-	-	-	178142	-	-	-	-	-	-	-	-	-	-	-	-	-	-	-	-	-	-	-	-	-	-	-	-	-	-	-	-	-	-	-	-	-	-	-	-	-	-
		-	-	-	-	-	-	-	-	-	-	-	-	178141	-	-	-	-	-	-	-	-	-	-	-	-	-	-	-	-	-	-	-	-	-	-	-	-	-	-	-	-	-	-	-	-	-	-	-	-	-	-
		-	+	-	-	-	-	-	AT4G22930.1	22206	BDEG_05204	-	-	178140	-	-	CMT584C	-	-	-	-	-	-	-	-	-	-	27072	-	-	NCU04323	Os01g54370.1	20691	-	19692	124889	108496	PF14_0697	763726	RO3G_17039.1	YLR420W	SPAC16.03c	-	-	-	27776	TA11110	83.m00001	-	-	-	UM01521.1
		-	+	-	-	-	-	-	AT5G06970.1	-	-	-	-	178139	-	-	-	-	-	-	-	-	-	-	-	-	-	-	-	-	-	Os03g47930.1	30266	-	-	200163	-	-	708460	-	-	-	-	-	-	-	-	-	-	-	-	-
		-	-	-	-	-	-	-	-	-	-	-	-	178138	-	-	-	-	-	-	-	-	-	-	-	-	-	-	-	-	-	-	-	-	-	-	-	-	-	-	-	-	-	-	-	-	-	-	-	-	-	-
		-	-	-	-	-	-	-	-	-	-	-	-	178132	-	-	-	-	-	-	-	-	-	-	-	-	-	-	-	-	-	-	-	-	-	-	-	-	-	-	-	-	-	-	-	-	-	-	-	-	-	-
		-	-	-	-	-	-	-	-	-	-	-	-	178131	-	-	-	-	-	-	-	-	-	-	-	-	-	-	-	-	-	-	-	-	-	-	-	-	-	-	-	-	-	-	-	-	-	-	-	-	-	-
		-	-	-	-	-	-	-	-	-	-	-	-	178127	-	-	-	-	-	-	-	-	-	-	-	-	-	-	-	-	-	-	36010	-	-	-	-	-	-	-	-	-	-	-	-	-	-	-	-	-	-	-
		-	+	-	-	-	-	-	AT2G15290.1	-	-	-	-	178114	-	-	CMN128C	-	-	-	-	-	-	-	-	-	-	-	-	-	-	Os06g43130.1	-	-	-	46757	-	-	642036	-	-	-	-	-	-	-	-	-	-	-	-	-
		-	-	-	-	-	-	-	-	-	-	-	-	178112	-	-	-	-	-	-	-	-	-	-	-	-	-	-	-	-	-	-	-	-	-	-	-	-	-	-	-	-	-	-	-	-	-	-	-	-	-	-
		-	-	-	-	-	-	-	-	-	-	-	-	178101	-	-	-	-	-	-	-	-	-	-	-	-	-	-	-	-	-	-	-	-	-	-	-	-	-	-	-	-	-	-	-	-	-	-	-	-	-	-
		-	-	-	-	-	-	-	-	-	-	-	-	178096	-	-	-	-	-	-	-	-	-	-	-	-	-	-	-	-	-	-	-	-	-	-	-	-	-	-	-	-	-	-	-	-	-	-	-	-	-	-
		-	-	-	-	-	-	-	-	-	-	-	-	178095	-	-	-	-	-	-	-	-	-	-	-	-	-	-	-	-	-	-	-	-	-	-	-	-	-	-	-	-	-	-	-	-	-	-	-	-	-	-
		-	-	-	-	-	-	-	-	-	-	-	-	178094	-	-	-	-	-	-	-	-	-	-	-	-	-	-	-	-	-	-	-	-	-	-	-	-	-	-	-	-	-	-	-	-	-	-	-	-	-	-
DNJ10	DnaJ-like protein 10, probably nuclear/cytosolic	-	+	-	-	-	-	-	AT5G45420.1	-	-	-	-	178083	-	-	-	-	-	-	-	-	-	-	-	LmjF36.4470	-	-	-	-	-	-	34581	-	-	211784	-	-	647622	-	-	-	-	-	-	-	-	20.m03793	-	-	Tb11.01.8750	-
		-	-	-	-	-	-	-	-	-	-	-	-	178081	-	-	-	-	-	-	-	-	-	-	-	-	-	-	-	-	-	-	-	-	-	-	-	-	-	-	-	-	-	-	-	-	-	-	-	-	-	-
		-	+	-	-	-	-	GB14575-PA	AT5G09860.1	-	-	-	184685	178080	298651	-	-	-	-	-	-	-	ENSGALP00000024009	-	OTTHUMP00000071816	-	138363	-	-	219410	-	Os03g01970.1	-	-	-	174480	142943	-	414862	RO3G_12734.1	-	-	GLEAN3_00283	SINFRUP00000130262	-	-	-	-	1612	-	-	-
		-	-	-	-	-	-	-	-	-	-	-	-	178071	-	-	-	-	-	-	-	-	-	-	-	-	-	-	-	-	-	-	-	-	-	-	-	-	-	-	-	-	-	-	-	-	-	-	-	-	-	-
		-	+	-	-	-	-	-	AT1G06200.1	-	-	-	-	178070	-	-	-	-	-	-	-	-	-	-	-	-	-	-	-	-	-	Os04g01240.1	35507	-	-	208572	-	-	816082	-	-	-	-	-	-	-	-	-	-	-	-	-
		-	-	-	-	-	-	-	-	-	-	-	-	178069	-	-	-	-	-	-	-	-	-	-	-	-	-	-	-	-	-	-	-	-	-	-	-	-	-	-	-	-	-	-	-	-	-	-	-	-	-	-
		-	+	-	-	-	-	-	AT3G20320.1	-	-	-	-	178067	-	-	CMV057C	-	-	-	-	-	-	-	-	-	-	-	-	-	-	Os01g74280.1	4205	-	-	146632	-	-	782272	-	-	-	-	-	-	-	-	-	-	-	-	-
		-	-	-	-	-	-	-	-	-	-	-	-	178066	-	-	-	-	-	-	-	-	-	-	-	-	-	-	-	-	-	-	-	-	-	-	-	-	-	-	-	-	-	-	-	-	-	-	-	-	-	-
		-	-	-	-	-	-	-	-	-	-	-	-	178064	-	-	-	-	-	-	-	-	-	-	-	-	-	-	-	-	-	-	-	-	-	-	-	-	-	-	-	-	-	-	-	-	-	-	-	-	-	-
		-	-	-	-	-	-	-	-	-	-	-	-	178063	-	-	-	-	-	-	-	-	-	-	-	-	-	-	-	-	-	-	-	-	-	-	-	-	-	-	-	-	-	-	-	-	-	-	-	-	-	-
		-	-	-	-	-	-	-	-	-	-	-	-	178062	-	-	-	-	-	-	-	-	-	-	-	-	-	-	-	-	-	-	-	-	-	-	-	-	-	-	-	-	-	-	-	-	-	-	-	-	-	-
		-	-	-	-	-	-	-	-	-	-	-	-	178059	-	-	-	-	-	-	-	-	-	-	-	-	-	-	-	-	-	-	-	-	-	-	-	-	-	-	-	-	-	-	-	-	-	-	-	-	-	-
		-	-	-	-	-	-	-	-	-	-	-	-	178053	-	-	-	-	-	-	-	-	-	-	-	-	-	-	-	-	-	-	-	-	-	-	-	-	-	-	-	-	-	-	-	-	-	-	-	-	-	-
ISG-C1	hydroxyproline-rich cell wall protein	-	-	-	+	-	-	-	-	-	-	-	-	178049	-	-	-	-	-	-	-	-	-	-	-	-	-	-	-	-	-	-	-	-	-	-	-	-	-	-	-	-	-	-	-	-	-	-	-	-	-	-
		-	-	-	-	-	-	-	-	-	-	-	-	178046	-	-	-	-	-	-	-	-	-	-	-	-	-	-	-	-	-	-	-	-	-	-	-	-	-	-	-	-	-	-	-	-	-	-	-	-	-	-
		-	-	-	-	-	-	-	-	-	-	-	-	178044	-	-	-	-	-	-	-	-	-	-	-	-	-	-	-	-	-	-	-	-	-	-	-	-	-	-	-	-	-	-	-	-	-	-	-	-	-	-
		-	+	-	-	-	-	-	-	-	-	-	-	178042	-	-	CML263C	-	-	-	-	-	-	-	-	-	-	-	-	-	-	-	35664	GSPATP00021101001	-	-	-	-	-	RO3G_08195.1	-	-	-	-	49.m00262	-	-	-	-	-	-	-
		-	+	-	-	-	-	-	AT3G58800.1	-	-	-	-	178040	-	-	-	-	-	-	-	-	-	-	-	-	-	-	-	-	-	Os02g13650.2	32713	-	-	72778	-	-	409604	-	-	-	-	-	-	-	-	-	-	-	-	-
		-	+	-	-	-	-	-	-	-	BDEG_03040	-	-	178039	-	-	-	-	-	-	-	-	-	-	-	-	-	-	50485	-	-	Os03g22400.1	31766	-	-	60415	-	-	587320	-	-	-	-	-	-	-	-	-	23232	-	-	-
		-	+	-	-	-	-	GB17999-PA	AT1G02740.1	-	BDEG_05763	-	-	178035	-	-	CMS194C	OTTDARP00000013921	DDB0218436	-	-	-	-	-	OTTHUMP00000185040	-	177197	-	-	177252	NCU06787	Os11g34300.2	-	-	-	122205	144750	-	287204	-	YPR023C	SPAC23H4.12	-	SINFRUP00000136064	-	-	-	-	60384	-	-	UM03736.1
MMP6	metalloproteinase, cell wall protein, homolog of Volvox VMPs	-	-	-	-	-	-	-	-	-	-	-	-	178030	-	-	-	-	-	-	-	-	-	-	-	-	-	-	-	-	-	-	-	-	-	-	-	-	-	-	-	-	-	-	-	-	-	-	-	-	-	-
		-	-	-	-	-	-	-	-	-	-	-	-	178028	-	-	-	-	-	-	-	-	-	-	-	-	-	-	-	-	-	-	-	-	-	-	-	-	-	-	-	-	-	-	-	-	-	-	-	-	-	-
		-	-	-	-	-	-	-	-	-	-	-	-	178021	-	-	-	-	-	-	-	-	-	-	-	-	-	-	-	-	-	-	-	-	-	-	-	-	-	-	-	-	-	-	-	-	-	-	-	-	-	-
		-	-	-	-	-	-	-	-	-	-	-	-	178020	-	-	-	-	-	-	-	-	-	-	-	-	-	-	-	-	-	-	-	-	-	-	-	-	-	-	-	-	-	-	-	-	-	-	-	-	-	-
		-	-	-	-	-	-	-	-	-	-	-	-	178019	-	-	-	-	-	-	-	-	-	-	-	-	-	-	-	-	-	-	-	-	-	-	-	-	-	-	-	-	-	-	-	-	-	-	-	-	-	-
		-	-	-	-	-	-	-	-	-	-	-	-	178017	-	-	-	-	-	-	-	-	-	-	-	-	-	-	-	-	-	-	-	-	-	-	-	-	-	-	-	-	-	-	-	-	-	-	-	-	-	-
		-	-	-	-	-	-	-	-	-	-	-	-	178008	-	-	-	-	-	-	-	-	-	-	-	-	-	-	-	-	-	-	-	-	-	-	-	-	-	-	-	-	-	-	-	-	-	-	-	-	-	-
		-	+	-	-	-	-	-	AT1G25290.1	9027	-	CE39642	-	178003	-	-	CML338C	-	DDB0218357	CG17212-PB	-	-	-	-	-	-	-	33060	-	-	-	Os09g28100.1	11016	-	47107	171507	-	-	817004	RO3G_11078.1	-	-	-	-	-	21098	-	55.m05050	-	-	-	-
		-	-	-	-	-	-	-	-	-	-	-	-	178000	-	-	-	-	-	-	-	-	-	-	-	-	-	-	-	-	-	-	-	-	-	-	-	-	-	-	-	-	-	-	-	-	-	-	-	-	-	-
		-	+	-	-	-	-	-	AT5G56730.1	28190	-	-	-	177997	-	-	-	-	-	-	-	-	-	-	-	-	-	-	-	-	-	Os01g37825.1	43	-	-	141088	-	-	-	-	-	-	-	-	-	-	-	-	-	-	-	-
		-	-	-	-	-	-	-	-	-	-	-	-	177992	-	-	-	-	-	-	-	-	-	-	-	-	-	-	-	-	-	-	-	-	-	-	-	-	-	-	-	-	-	-	-	-	-	-	-	-	-	-
DNJ4	DnaJ-like protein 4	-	-	-	-	-	-	-	-	-	-	-	-	177989	-	-	-	-	-	-	-	-	-	-	-	-	-	-	-	-	-	-	36097	-	-	-	-	-	-	-	-	-	GLEAN3_13575	-	-	-	-	-	-	-	-	-
		-	-	-	-	-	-	-	-	-	-	-	-	177988	-	-	-	-	-	-	-	-	-	-	-	-	-	-	-	-	-	-	-	-	-	-	-	-	-	-	-	-	-	-	-	-	-	-	-	-	-	-
		-	-	-	-	-	-	-	-	-	-	-	-	177968	-	-	-	-	-	-	-	-	-	-	-	-	-	-	-	-	-	-	-	-	-	-	138348	-	-	-	-	-	-	-	-	-	-	-	-	-	-	-
		-	-	-	-	-	-	-	-	-	-	-	-	177953	-	-	-	-	-	-	-	-	-	-	-	-	-	-	-	-	-	-	-	-	-	-	-	-	-	-	-	-	-	-	-	-	-	-	-	-	-	-
		-	-	-	-	-	-	-	-	-	-	-	-	177949	-	-	-	-	-	-	-	-	-	-	-	-	-	-	-	-	-	-	-	-	-	-	-	-	-	-	-	-	-	-	-	-	-	-	-	-	-	-
		-	+	-	-	-	-	GB12702-PA	AT2G26460.1	-	-	CE25338	225605	177943	381246	-	-	-	-	CG18005-PA	-	-	ENSGALP00000001219	-	NP_006074.2	-	214293	34240	-	211930	-	Os11g04950.1	32189	-	-	197602	135360	MAL13P1.34	778660	RO3G_04550.1	-	-	-	SINFRUP00000177552	-	38180	TA02605	50.m03295	64358	-	-	-
		-	-	-	-	-	-	-	-	-	-	-	-	177938	-	-	-	-	-	-	-	-	-	-	-	-	-	-	-	-	-	-	-	-	-	-	-	-	-	-	-	-	-	-	-	-	-	-	-	-	-	-
		-	-	-	-	-	-	-	-	-	-	-	-	177936	-	-	-	-	-	-	-	-	-	-	-	-	-	-	-	-	-	-	-	-	-	-	-	-	-	-	-	-	-	-	-	-	-	-	-	-	-	-
		-	-	-	-	-	-	-	-	-	-	-	-	177933	-	-	-	-	-	-	-	9.m00386	-	-	-	LmjF06.0510	-	-	60032	-	-	-	-	-	-	-	-	-	-	-	-	-	-	-	-	-	-	-	-	-	-	-
		-	-	-	-	-	-	-	-	-	-	-	-	177931	-	-	-	-	-	-	-	-	-	-	-	-	-	-	-	-	-	-	-	-	-	-	-	-	-	-	-	-	-	-	-	-	-	-	-	-	-	-
		-	-	-	-	-	-	-	-	-	-	-	-	177930	-	-	-	-	-	-	-	-	-	-	-	-	-	-	-	-	-	-	-	-	-	-	-	-	-	-	-	-	-	-	-	-	-	-	-	-	-	-
		-	-	-	-	-	-	-	-	-	-	-	-	177924	-	-	-	-	-	-	-	-	-	-	-	-	-	-	-	-	-	-	-	-	-	-	-	-	-	-	-	-	-	-	-	-	-	-	-	-	-	-
		-	-	-	-	-	-	-	-	-	-	-	-	177923	-	-	-	-	-	-	-	-	-	-	-	-	-	-	-	-	-	-	-	-	-	-	-	-	-	-	-	-	-	-	-	-	-	-	-	-	-	-
		-	-	-	-	-	-	-	AT1G22610.1	-	-	-	-	177919	-	-	-	-	-	-	-	-	-	-	-	-	-	-	-	-	-	Os01g40480.1	-	-	-	135464	-	-	773811	-	-	-	-	-	-	-	-	-	-	-	-	-
DNJ5	DnaJ-like protein 5	-	-	-	-	-	-	-	AT3G14200.1	-	-	-	-	177910	-	-	-	-	-	-	-	-	-	-	-	-	-	-	-	-	-	-	-	-	-	169319	-	-	-	RO3G_10168.1	-	-	-	-	-	-	-	-	-	-	Tb11.02.3430	-
		-	-	-	-	-	-	-	-	-	-	-	-	177906	-	-	-	-	-	-	-	-	-	-	-	-	-	-	-	-	-	-	-	-	-	-	-	-	-	-	-	-	-	-	-	-	-	-	-	-	-	-
		-	-	-	-	-	-	-	-	-	-	-	-	177899	-	-	-	-	-	-	-	-	-	-	-	-	-	-	-	-	-	-	-	-	-	-	-	-	-	-	-	-	-	-	-	-	-	-	-	-	-	-
		-	-	-	-	-	-	-	-	-	-	-	-	177897	-	-	-	-	-	-	-	-	-	GL50803_14833	-	-	-	-	-	-	-	-	-	GSPATP00000408001	-	-	-	-	-	-	-	-	-	-	30.m00305	-	-	-	-	91860.m00078	-	-
		-	+	+	-	-	-	GB11813-PA	AT4G18230.1	60055	BDEG_03105	CE12326	161579	177895	202788	cgd7_4930	CMS225C	OTTDARP00000023764	DDB0188847	CG6308-PA	-	21.m00271	ENSGALP00000008947	-	OTTHUMP00000012469	-	165683	28936	34380	216472	NCU03344	Os03g30934.2	6223	GSPATP00013198001	14444	142865	158733	PFB0515w	815480	RO3G_11288.1	YBR070C	SPAC5D6.06c	-	SINFRUP00000146116	88.m00090	17966	-	23.m00239	22933	81617.m00181	-	-
		-	-	-	-	-	-	-	-	-	-	-	-	177892	-	-	-	-	-	-	-	-	-	-	-	-	-	-	-	-	-	-	-	-	-	-	-	-	-	-	-	-	-	-	-	-	-	-	-	-	-	-
		-	-	-	-	-	-	-	-	-	-	-	-	177887	-	-	-	-	-	-	-	-	-	-	-	-	-	-	-	-	-	-	-	-	-	-	-	-	-	-	-	-	-	-	-	-	-	-	-	-	-	-
		-	+	-	-	-	-	GB14350-PA	AT5G01960.1	64494	BDEG_02257	CE35812	184542	177885	287155	-	CMG126C	OTTDARP00000023300	DDB0189490	-	-	9.m00424	ENSGALP00000013360	-	NP_001103373.1	-	171133	29282	80806	110696	-	Os05g25180.1	35792	-	-	117817	158806	-	836277	RO3G_03024.1	-	-	GLEAN3_19875	SINFRUP00000136307	-	-	-	-	28618	94483.m00060	-	-
		-	-	-	-	-	-	-	-	-	-	-	-	177883	-	-	-	-	-	-	-	-	-	-	-	-	-	-	-	-	-	-	-	-	-	-	-	-	-	-	-	-	-	-	-	-	-	-	-	-	-	-
		-	-	-	-	-	-	-	-	-	-	-	-	177882	-	-	-	-	-	-	-	-	-	-	-	-	-	-	68422	-	-	-	-	-	-	104028	-	-	-	-	-	-	-	SINFRUP00000180597	-	-	-	-	-	-	-	-
		-	-	-	-	-	-	-	-	-	-	-	-	177865	-	-	-	-	-	-	-	-	-	-	-	-	-	-	-	-	-	-	-	-	-	-	-	-	-	-	-	-	-	-	-	-	-	-	-	-	-	-
		-	-	-	-	-	-	-	AT2G20310.1	29916	-	-	-	177855	226923	-	-	-	-	-	-	-	-	-	-	-	-	-	-	-	-	-	-	-	-	-	-	-	-	-	-	-	-	-	-	-	-	-	-	-	-	-
		-	-	-	-	-	-	-	-	-	-	-	-	-	-	-	-	-	-	-	-	-	-	-	-	-	-	-	-	-	-	-	-	-	-	-	-	-	-	-	-	-	-	-	-	-	-	-	-	-	-	-
		-	-	-	-	-	-	-	-	-	-	-	-	177851	-	-	-	-	-	-	-	-	-	-	-	-	-	-	-	-	-	-	-	-	-	-	-	-	-	-	-	-	-	-	-	-	-	-	-	-	-	-
		-	+	-	-	-	-	-	AT3G17470.1	-	-	-	-	177832	-	-	-	-	-	-	-	-	-	-	-	-	-	-	-	-	-	Os05g06890.1	31569	-	-	140875	-	-	228942	-	-	-	-	-	-	-	-	-	-	-	-	-
		-	-	-	-	-	-	-	-	-	-	-	-	177830	-	-	-	-	-	-	-	-	-	-	-	-	-	-	-	-	-	-	-	-	-	-	-	-	-	-	-	-	-	-	-	-	-	-	-	-	-	-
		-	-	-	-	-	-	-	-	63415	-	-	-	177821	-	-	-	-	-	-	-	-	-	-	-	-	-	-	-	-	-	-	-	-	-	-	-	-	585632	-	-	-	-	-	-	-	-	-	-	-	-	-
		-	-	-	-	-	-	-	-	-	-	-	-	177815	-	-	-	-	-	-	-	-	-	-	-	-	-	-	-	-	-	-	-	-	-	-	-	-	-	-	-	-	-	-	-	-	-	-	-	-	-	-
		-	-	-	-	-	-	-	-	-	-	-	-	177813	-	-	-	-	-	-	-	-	-	-	-	-	-	-	-	-	-	-	-	-	-	-	-	-	-	-	-	-	-	-	-	-	-	-	-	-	-	-
		-	-	-	-	-	-	-	-	-	-	-	-	177811	-	-	-	-	-	-	-	-	-	-	-	-	-	-	-	-	NCU07036	-	-	-	-	-	-	-	-	-	-	-	-	-	-	-	-	-	-	-	-	-
		-	-	-	-	-	-	-	-	-	-	-	-	177809	-	-	-	-	-	-	-	-	-	GL50803_5249	-	-	-	-	-	-	-	-	-	-	-	-	-	-	-	-	-	-	-	-	-	-	-	-	-	-	-	-
		-	-	-	-	-	-	-	-	-	-	-	-	177795	-	-	-	-	-	-	-	-	-	-	-	-	-	-	-	-	-	-	-	-	-	-	-	-	-	-	-	-	-	-	-	-	-	-	-	-	-	-
		-	-	-	-	-	-	-	-	-	-	-	-	177794	-	-	-	-	-	-	-	-	-	-	-	-	-	-	-	-	-	-	-	-	-	-	-	-	-	-	-	-	-	-	-	-	-	-	-	-	-	-
		-	-	-	-	-	-	-	-	-	-	-	-	177788	-	-	-	-	-	-	-	-	-	-	-	-	-	-	-	-	-	-	-	-	-	-	-	-	-	-	-	-	-	-	-	-	-	-	-	-	-	-
		-	-	-	-	-	-	-	-	62782	-	-	-	177786	-	-	-	-	-	-	-	-	-	-	-	-	-	-	-	-	-	-	-	-	-	74531	-	-	-	-	-	-	-	-	-	-	-	-	-	-	-	-
		-	-	-	-	-	-	-	-	-	-	-	-	177785	-	-	-	-	-	-	-	-	-	-	-	-	-	-	-	-	-	-	-	-	-	-	-	-	-	-	-	-	-	-	-	-	-	-	-	-	-	-
		+	+	-	-	-	-	-	-	-	-	-	170026	177784	256530	-	-	OTTDARP00000014582	-	-	-	-	ENSGALP00000017862	-	OTTHUMP00000021361	-	102605	-	-	118996	-	-	-	GSPATP00028110001	-	-	141128	-	-	-	-	-	-	SINFRUP00000153450	35.m00289	-	-	57.m01723	55076	-	-	-
		-	-	-	-	-	-	-	-	-	-	-	-	177782	-	-	-	-	-	-	-	-	-	-	-	-	-	-	-	-	-	-	-	-	-	-	-	-	-	-	-	-	-	-	-	-	-	-	-	-	-	-
		-	-	-	-	-	-	-	-	-	-	-	-	177781	-	-	-	-	-	-	-	-	-	-	-	-	-	-	-	-	-	-	-	-	-	-	-	-	-	-	-	-	-	-	-	-	-	-	-	-	-	-
MMP8	metalloproteinase, cell wall protein, homolog of Volvox VMPs	-	-	-	-	-	-	-	-	-	-	-	-	177780	-	-	-	-	-	-	-	-	-	-	-	-	-	-	-	-	-	-	-	-	-	-	-	-	-	-	-	-	-	-	-	-	-	-	-	-	-	-
		-	-	-	-	-	-	-	-	18387	-	-	-	177779	-	-	-	-	-	-	-	-	-	-	-	-	-	-	-	-	-	-	-	-	-	-	-	-	-	-	-	-	-	-	-	-	-	-	-	-	-	-
		-	-	-	-	-	-	-	-	-	-	-	-	177776	-	-	-	-	-	-	-	-	-	-	-	-	-	-	-	-	-	-	-	-	-	-	-	-	-	-	-	-	-	-	-	-	-	-	-	-	-	-
		-	-	-	-	-	-	-	-	64764	-	-	-	177775	-	-	-	-	-	-	-	-	-	-	-	-	154011	-	-	-	-	Os11g18690.1	-	-	-	-	-	-	278212	-	-	-	-	-	-	-	-	-	-	-	-	-
		-	-	-	-	-	-	-	-	-	-	-	-	177753	-	-	-	-	-	-	-	-	-	-	-	-	-	-	-	-	-	-	-	-	-	-	-	-	-	-	-	-	-	-	-	-	-	-	-	-	-	-
		-	-	-	-	-	-	-	-	-	-	-	-	177751	-	-	-	-	-	-	-	-	-	-	-	-	-	-	-	-	-	-	-	-	-	-	-	-	-	-	-	-	-	-	-	-	-	-	-	-	-	-
		-	-	-	-	-	-	-	-	-	-	-	-	177740	-	-	-	-	-	-	-	-	-	-	-	-	-	-	-	-	-	-	-	-	-	-	-	-	-	-	-	-	-	-	-	-	-	-	-	-	-	-
		-	-	-	-	-	-	-	-	-	-	-	-	177730	-	-	-	-	-	-	-	-	-	-	-	-	-	-	-	-	-	-	-	-	-	-	-	-	-	-	-	-	-	-	-	-	-	-	-	-	-	-
		-	-	-	-	-	-	-	-	-	-	-	-	177725	-	-	-	-	-	-	-	-	-	-	-	-	-	-	-	-	-	-	-	-	-	-	-	-	-	-	-	-	-	-	-	-	-	-	-	-	-	-
		-	-	-	-	-	-	-	-	-	-	-	-	177721	-	-	-	-	-	-	-	-	-	-	-	-	-	-	-	-	-	-	-	-	-	-	-	-	-	-	-	-	-	-	-	-	-	-	-	-	-	-
		-	-	-	-	-	-	-	-	-	-	-	-	177720	-	-	-	-	-	-	-	-	-	-	-	-	-	-	-	-	-	-	-	-	-	-	-	-	-	-	-	-	-	-	-	-	-	-	-	-	-	-
		-	+	-	-	-	-	-	AT1G06560.1	-	-	-	182050	177717	248406	-	-	-	DDB0188951	CG11109-PA	-	-	ENSGALP00000013934	-	OTTHUMP00000019261	-	174713	24394	50302	211081	-	Os08g27824.1	-	-	-	11660	144879	-	205300	-	-	-	-	-	-	33094	-	-	28123	-	-	-
		-	-	-	-	-	-	-	-	-	-	-	-	177706	-	-	-	-	-	-	-	-	-	-	-	-	-	-	-	-	-	-	-	-	-	-	-	-	-	-	-	-	-	-	-	-	-	-	-	-	-	-
		-	-	-	-	-	-	-	-	-	-	-	-	177705	-	-	-	-	-	-	-	-	-	-	-	-	-	-	-	-	-	-	-	-	-	-	-	-	-	-	-	-	-	-	-	-	-	-	-	-	-	-
		-	-	-	-	-	-	-	-	-	-	-	-	177702	-	-	-	-	-	-	-	-	-	-	-	-	-	-	-	-	-	-	-	-	-	-	-	-	-	-	-	-	-	-	-	-	-	-	-	-	-	-
		-	-	-	-	-	-	-	-	-	-	-	-	177694	-	-	-	-	-	-	-	-	-	-	-	-	-	-	-	-	-	-	-	-	-	-	-	-	-	-	-	-	-	-	-	-	-	-	-	-	-	-
PGP1c		-	+	-	-	-	-	GB14601-PA	AT4G04870.1	-	BDEG_02834	CE37763	224166	177689	-	-	CMN196C	-	-	-	-	-	ENSGALP00000014982	-	OTTHUMP00000030219	-	107885	28834	-	108180	NCU00135	Os01g57930.1	36513	-	-	164548	140356	-	196659	-	YDL142C	-	-	SINFRUP00000177846	-	260941	-	-	33397	-	-	-
		-	-	-	-	-	-	-	-	-	-	-	-	177682	-	-	-	-	-	-	-	-	-	-	-	-	-	-	-	-	-	-	-	-	-	-	-	-	-	-	-	-	-	-	-	-	-	-	-	-	-	-
		-	-	-	-	-	-	-	-	-	-	-	-	177681	-	-	-	-	-	-	-	-	-	-	-	-	-	-	-	-	-	-	-	-	-	-	-	-	-	-	-	-	-	-	-	-	-	-	-	-	-	-
		-	-	-	-	-	-	-	-	-	-	-	-	177678	-	-	-	-	-	-	-	-	-	-	-	-	-	-	70704	-	-	-	-	-	-	-	-	-	-	-	-	-	-	-	-	-	-	-	-	-	Tb11.22.0005	-
		-	-	-	-	-	-	-	-	-	-	-	-	177670	-	-	-	-	-	-	-	-	-	-	-	-	-	-	-	-	-	-	-	-	-	-	-	-	-	-	-	-	-	-	-	-	-	-	-	-	-	-
		-	-	-	-	-	-	-	-	-	-	-	-	177668	-	-	-	-	-	-	-	-	-	-	-	-	-	-	-	-	-	-	-	-	-	-	-	-	-	-	-	-	-	-	-	-	-	-	-	-	-	-
CHR3424	SNF2 domain-containing protein	-	+	-	-	-	-	-	-	-	-	-	-	177665	-	-	-	-	-	-	-	-	ENSGALP00000036810	-	-	-	-	-	-	-	-	-	-	-	-	112144	-	-	581093	-	-	-	-	SINFRUP00000143713	-	-	-	-	53804	-	-	-
		-	-	-	-	-	-	-	-	-	-	-	-	177662	-	-	-	-	-	-	-	-	-	-	-	-	-	-	-	-	-	-	-	-	-	-	-	-	-	-	-	-	-	-	-	-	-	-	-	-	-	-
		-	+	-	-	-	-	-	-	-	-	-	-	177660	-	-	CMP337C	-	-	-	-	-	-	-	-	-	-	-	-	-	-	-	12759	-	45350	159570	-	-	-	-	-	-	-	-	-	264064	-	-	-	-	-	-
		-	-	-	-	-	-	-	-	-	-	-	-	177657	-	-	-	-	-	-	-	-	-	-	-	-	-	-	-	-	-	-	-	-	-	-	-	-	-	-	-	-	-	-	683.m00005	-	-	-	-	-	-	-
		-	-	-	-	-	-	-	-	-	-	-	-	177656	-	-	-	-	-	-	-	-	-	-	-	-	-	-	-	-	-	-	-	-	-	-	-	-	-	-	-	-	-	-	-	-	-	-	-	-	-	-
		-	+	-	-	-	-	-	-	-	-	-	-	177646	-	cgd6_2030	-	-	-	-	-	-	-	-	-	LmjF04.0020	-	-	-	-	-	-	-	GSPATP00011223001	-	-	-	MAL8P1.13	-	-	-	-	-	-	-	-	TA04055	-	-	-	-	-
		-	-	-	-	-	-	-	-	-	-	-	-	177645	-	-	-	-	-	-	-	-	-	-	-	-	-	-	-	-	-	-	-	-	-	-	-	-	-	-	-	-	-	-	-	-	-	-	-	-	-	-
		-	-	-	-	-	-	-	-	-	-	-	-	177644	-	-	-	-	-	-	-	-	-	-	-	-	-	-	-	-	-	-	33329	-	-	-	-	-	-	-	-	-	-	-	-	-	-	-	-	-	-	-
		-	-	-	-	-	-	-	-	-	-	-	-	177642	-	-	-	-	-	-	-	-	-	-	-	-	-	-	-	-	-	-	-	-	-	-	-	-	-	-	-	-	-	-	-	-	-	-	-	-	-	-
		-	-	-	-	-	-	-	-	-	-	-	-	177641	-	-	-	-	-	-	-	-	-	-	-	-	-	-	-	-	-	-	-	-	-	-	-	-	-	-	-	-	-	-	-	-	-	-	-	-	-	-
CLR7	Coxiella-Like Repeat protein	-	-	-	-	-	-	-	-	-	-	-	-	177619	-	-	-	-	-	-	-	-	-	-	-	-	-	-	-	-	-	-	-	-	-	-	-	-	-	-	-	-	-	-	-	-	-	-	-	-	-	-
CHR3418		-	-	-	-	-	-	-	-	-	-	-	-	177618	-	-	-	-	-	-	-	-	-	-	-	-	-	-	-	-	-	-	-	-	-	-	-	-	255259	-	-	-	-	-	-	-	-	-	-	-	-	-
		-	+	-	+	-	-	-	-	-	-	-	-	177605	-	-	-	-	-	-	-	-	ENSGALP00000003985	-	NP_660337.3	-	-	-	62107	-	-	-	-	GSPATP00017756001	-	-	-	-	-	-	-	-	-	-	18.m00315	-	-	-	20616	-	-	-
		-	+	-	-	-	-	-	-	-	-	-	-	177603	-	-	-	-	-	-	-	-	-	-	-	-	-	9768	-	239965	-	-	-	-	-	165172	-	-	-	-	-	-	-	-	-	-	-	65.m01155	61091	-	-	-
		-	-	-	-	-	-	-	-	-	-	-	-	177600	-	-	-	-	-	-	-	-	-	-	-	-	-	-	-	-	-	-	-	-	-	-	-	-	-	-	-	-	-	-	-	-	-	-	-	-	-	-
FAP50	Conserved Uncharacterized Flagellar Associated Protein	-	+	-	+	-	-	-	AT2G36360.2	456	BDEG_05718	-	151783	177591	-	-	-	-	DDB0201976	-	-	-	ENSGALP00000001591	GL50803_37452	NP_115645.3	LmjF05.1020	115813	32767	68117	243403	-	Os01g19480.1	-	GSPATP00025242001	-	80639	134109	-	-	RO3G_00122.1	YGR238C	-	-	SINFRUP00000159832	57.m00171	-	-	641.m01512	57170	80470.m00056	Tb927.5.500	-
		-	-	-	-	-	-	-	-	-	-	-	-	177582	-	-	-	-	-	-	-	-	-	-	-	-	-	-	-	-	-	-	-	-	-	-	-	-	-	-	-	-	-	-	-	-	-	-	-	-	-	-
		-	-	-	-	-	-	-	-	-	-	-	-	177577	-	-	-	-	-	-	-	-	-	-	-	-	-	-	-	-	-	-	-	-	-	-	-	-	-	-	-	-	-	-	-	-	-	-	-	-	-	-
		-	+	-	-	-	-	-	-	-	BDEG_06006	-	208596	177575	285923	-	-	-	-	CG15737-PA	-	-	ENSGALP00000011874	-	-	LmjF20.0600	-	-	-	-	NCU04364	Os01g62780.1	34821	-	49177	-	129943	PF10_0152	-	-	-	-	-	SINFRUP00000181226	-	-	-	74.m00422	54458	-	-	-
RWP3	hypothetical protein	-	-	-	-	-	-	-	-	-	-	-	-	177573	-	-	-	-	-	-	-	-	-	-	-	-	-	-	-	-	-	-	-	-	-	-	-	-	-	-	-	-	-	-	-	-	-	-	-	-	-	-
		-	-	-	-	-	-	-	-	-	-	-	-	177566	-	-	-	-	-	-	-	-	-	-	-	-	-	-	-	-	-	-	-	-	-	-	-	-	-	-	-	-	-	-	-	-	-	-	-	-	-	-
		-	-	-	-	-	-	-	-	-	-	-	-	177563	-	-	-	-	-	-	-	-	-	-	-	-	-	-	-	-	-	-	-	-	20885	-	-	-	-	-	-	-	-	-	-	-	-	-	-	-	-	-
		-	-	-	+	-	-	-	-	-	-	CE19200	-	177558	-	-	-	-	-	-	-	-	-	-	-	-	-	-	-	-	-	-	-	-	-	-	-	-	-	-	-	-	-	-	-	-	-	-	-	-	-	-
		-	-	-	-	-	-	-	-	-	-	-	-	177556	-	-	-	-	-	-	-	-	-	-	-	-	-	-	-	-	-	-	-	-	-	-	-	-	-	-	-	-	-	-	-	-	-	-	-	-	-	-
		-	-	-	-	-	-	-	-	-	-	-	-	177553	-	-	-	-	-	-	-	-	-	-	OTTHUMP00000018501	-	-	-	73664	239527	-	-	-	-	-	-	-	-	-	-	-	-	-	-	-	-	-	-	-	-	-	-
		-	-	-	-	-	-	-	-	-	-	-	-	177549	-	-	-	-	-	-	-	-	-	-	-	-	-	-	-	-	-	-	-	-	-	-	-	-	-	-	-	-	-	-	-	-	-	-	-	-	-	-
		-	-	-	-	-	-	-	-	-	-	-	-	177546	-	-	-	-	-	-	-	-	-	-	-	-	-	-	-	-	-	-	-	-	-	-	-	-	-	-	-	-	-	-	-	-	-	-	-	-	-	-
		-	-	-	-	-	-	-	-	-	-	-	-	177542	-	-	-	-	-	-	-	-	-	-	-	-	-	-	-	-	-	-	-	-	-	-	-	-	-	-	-	-	-	-	-	-	-	-	-	-	-	-
		-	-	-	-	-	-	-	-	-	-	-	-	177541	-	-	-	-	-	-	-	-	-	-	-	-	-	-	-	-	-	-	-	-	-	-	-	-	-	-	-	-	-	-	-	-	-	-	-	-	-	-
		-	-	-	-	-	-	-	-	-	-	-	-	177540	-	-	-	-	-	-	-	-	-	-	-	-	-	-	-	-	-	-	-	-	-	-	-	-	-	-	-	-	-	-	-	-	-	-	-	-	-	-
		-	-	-	-	-	-	-	-	-	-	-	-	177538	-	-	-	-	-	-	-	-	-	-	-	-	-	-	-	-	-	-	-	-	-	-	-	-	-	-	-	-	-	-	-	-	-	80.m02124	-	-	-	-
		-	-	-	-	-	-	-	-	-	-	-	-	177536	-	-	-	-	-	-	-	-	-	-	-	-	-	-	-	-	-	-	-	-	-	-	-	-	-	-	-	-	-	-	-	-	-	-	-	-	-	-
FAS6	fasciclin-like protein	-	-	-	-	-	-	-	-	-	-	-	-	177529	-	-	-	-	-	-	-	-	-	-	-	-	-	-	-	-	-	-	-	-	-	-	-	-	-	-	-	-	-	-	-	-	-	-	-	-	-	-
FAS5	fasciclin-like protein	-	-	-	-	-	-	-	-	-	-	-	-	177528	-	-	-	-	-	-	-	-	-	-	-	-	-	-	-	-	-	-	-	-	-	-	-	-	-	-	-	-	-	-	-	-	-	-	-	-	-	-
		-	-	-	-	-	-	-	-	-	-	-	-	177526	-	-	-	-	-	-	-	-	-	-	-	-	-	-	-	-	-	-	-	-	-	-	-	-	-	-	-	-	-	-	-	-	-	-	-	-	-	-
		-	+	-	-	-	-	GB17205-PA	AT5G66160.1	-	BDEG_04114	CE33946	209816	177524	286077	-	CMT443C	-	-	CG10277-PC	19069097	-	ENSGALP00000016932	-	OTTHUMP00000125245	-	-	-	-	212981	-	Os08g01360.2	-	GSPATP00010282001	-	213315	-	-	249669	RO3G_14159.1	-	-	-	SINFRUP00000147097	-	-	-	-	63462	-	-	-
		-	-	-	-	-	-	-	-	-	-	-	-	177514	-	-	-	-	-	-	-	-	-	-	-	-	-	-	-	-	-	-	-	-	-	-	-	-	-	-	-	-	-	-	-	-	-	-	-	-	-	-
		-	-	-	-	-	-	-	-	-	-	-	-	177503	-	-	-	-	-	-	-	-	-	-	-	-	-	-	-	-	-	-	-	-	-	-	-	-	-	-	-	-	-	-	-	-	-	-	-	-	-	-

		-	-	-	-	-	-	-	-	-	-	-	-	177502	-	-	-	-	-	-	-	-	-	-	-	-	-	-	-	-	-	-	-	-	-	-	-	-	-	-	-	-	-	-	-	-	-	-	-	-	-	-
		-	-	-	-	-	-	-	-	-	-	-	-	177482	-	-	-	-	-	-	-	-	-	-	-	-	-	-	-	-	-	-	-	-	-	-	-	-	-	-	-	-	-	-	-	-	-	-	-	-	-	-
		-	-	-	-	-	-	-	-	-	-	-	-	177476	-	-	-	-	-	-	-	-	-	-	-	-	-	-	-	-	-	-	-	-	-	-	-	-	-	-	-	-	-	-	-	-	-	-	-	-	-	-
		-	-	-	-	-	-	-	-	-	-	-	-	177470	-	-	-	-	-	-	-	-	-	-	-	-	-	-	-	-	-	-	-	-	-	-	-	-	-	-	-	-	-	-	-	-	-	-	-	-	-	-
		-	-	-	-	-	-	-	-	-	-	-	-	177469	-	-	-	-	-	-	-	-	-	-	-	-	-	-	-	-	-	-	-	-	-	-	-	-	-	-	-	-	-	-	-	-	-	-	-	-	-	-
		-	-	-	-	-	-	-	-	-	-	-	-	177465	-	-	-	-	-	-	-	-	-	-	-	-	-	-	-	-	-	-	-	-	-	-	-	-	-	-	-	-	-	-	-	-	-	-	-	-	-	-
		-	-	-	-	-	-	-	-	-	-	-	-	177458	-	-	-	-	-	-	-	-	-	-	-	-	-	-	-	-	-	-	-	-	-	-	-	-	-	-	-	-	-	-	-	-	-	-	-	-	-	-
		-	-	-	-	-	-	-	-	-	-	-	-	177453	-	-	-	-	-	-	-	-	-	-	-	-	-	-	-	-	-	-	-	-	-	-	-	-	-	-	-	-	-	-	-	-	-	-	-	-	-	-
		-	-	-	-	-	-	-	-	-	-	-	-	177450	-	-	-	-	-	-	-	-	-	-	-	-	-	-	-	-	-	-	-	-	-	-	-	-	-	-	-	-	-	-	-	-	-	-	-	-	-	-
		-	-	-	-	-	-	-	-	71160	-	-	-	177448	-	-	-	-	-	-	-	-	-	GL50803_9036	-	-	-	-	-	-	-	-	-	-	-	-	-	-	-	-	-	-	-	-	-	-	-	-	-	80444.m00130	-	-
		-	-	-	-	-	-	-	-	-	-	-	-	177447	-	-	-	-	-	-	-	-	-	-	-	-	-	-	-	-	-	-	-	-	-	-	-	-	-	-	-	-	-	-	-	-	-	-	-	-	-	-
AGO3402		-	-	-	-	-	-	-	-	-	-	-	-	177446	-	-	-	-	-	-	-	-	-	-	-	-	-	-	-	-	-	-	-	-	-	-	-	-	-	-	-	-	-	-	-	-	-	-	-	-	-	-
		-	-	-	-	-	-	-	-	-	-	-	-	177442	-	-	-	-	-	-	-	-	-	-	-	-	-	-	-	-	-	-	-	-	-	-	-	-	-	-	-	-	-	-	-	-	-	-	-	-	-	-
		-	+	-	-	-	-	GB10279-PA	AT1G70330.1	-	-	-	221180	177440	-	-	-	OTTDARP00000023928	-	CG31911-PA	-	-	ENSGALP00000016532	-	OTTHUMP00000016512	-	237985	34097	83250	233134	NCU00356	Os08g10450.1	-	-	-	178772	-	-	259649	-	-	-	GLEAN3_28310	SINFRUP00000127559	-	-	-	-	61365	-	-	-
EF1G	putative elongation factor 1, gamma chain	-	+	-	-	-	-	-	-	-	-	-	-	177436	-	-	-	-	-	-	-	-	-	-	-	-	-	-	-	-	NCU03826	Os02g12794.3	-	-	-	-	-	PF13_0214	-	RO3G_13129.1	YPL048W	-	-	-	-	-	-	-	-	-	-	-
		-	-	-	-	-	-	-	-	-	-	-	-	177432	-	-	-	-	-	-	-	-	-	-	-	-	-	-	-	-	-	-	-	-	-	-	-	-	-	-	-	-	-	-	-	-	-	-	-	-	-	-
		-	+	-	-	-	-	GB12819-PA	-	-	-	-	23970	177421	262022	-	-	-	-	CG14439-PA	-	-	-	-	-	-	-	27436	-	-	-	-	3107	-	32014	-	-	-	-	-	-	-	-	-	-	261312	-	-	20550	-	-	-
		-	-	-	-	-	-	-	-	-	-	-	-	177420	-	-	-	-	-	-	-	-	-	-	-	-	-	-	-	-	-	-	-	-	-	-	-	-	-	-	-	-	-	-	-	-	-	-	-	-	-	-
		-	-	-	-	-	-	-	-	-	-	-	-	177412	-	-	-	-	-	-	-	-	-	-	-	-	-	-	-	-	-	-	-	-	-	-	-	-	-	-	-	-	-	-	-	-	-	-	-	-	-	-
		-	-	-	-	-	-	-	-	-	-	-	-	177406	-	-	-	-	-	-	-	-	-	-	-	-	-	-	-	-	-	-	-	-	-	-	-	-	-	-	-	-	-	-	-	-	-	-	-	-	-	-
		-	-	-	-	-	-	-	-	-	-	-	-	177397	-	-	-	-	-	-	-	-	-	-	-	-	-	-	-	-	-	-	-	-	-	-	-	-	-	-	-	-	-	-	-	-	-	-	-	-	-	-
	gametolysin-like protein	-	-	-	-	-	-	-	-	-	-	-	-	177395	-	-	-	-	-	-	-	-	-	-	-	-	-	-	-	-	-	-	-	-	33107	-	-	-	-	-	-	-	-	-	-	-	-	-	-	-	-	-
		-	-	-	-	-	-	-	AT5G46070.1	-	-	-	-	177392	-	-	-	-	-	-	-	-	-	-	-	-	-	-	-	-	-	Os02g20410.1	-	-	-	-	-	-	555746	-	-	-	-	-	-	-	-	-	-	-	-	-
		-	-	-	-	-	-	-	-	-	-	-	-	177387	-	-	-	-	-	-	-	-	-	-	-	-	-	-	-	-	-	-	-	-	-	-	-	-	-	-	-	-	-	-	-	-	-	-	-	-	-	-
		-	-	-	-	-	-	-	-	-	-	-	-	177386	-	-	-	-	-	-	-	-	-	-	-	-	-	-	-	-	-	-	-	-	-	-	-	-	-	-	-	-	-	-	-	-	-	-	-	-	-	-
		-	+	-	-	+	-	GB11727-PA	AT5G42320.1	-	BDEG_05427	CE31658	227948	177379	226733	-	-	OTTDARP00000022757	-	CG18417-PA	-	-	ENSGALP00000013044	-	OTTHUMP00000024880	-	159841	23753	-	87987	-	Os02g02710.1	11914	GSPATP00032829001	-	44208	-	-	818859	-	-	-	GLEAN3_08959	SINFRUP00000155189	44.m00202	-	-	-	59794	-	-	-
		-	-	-	-	-	-	-	-	-	-	-	-	177375	-	-	-	-	-	-	-	-	-	-	-	-	-	-	-	-	-	-	-	-	-	-	-	-	-	-	-	-	-	-	-	-	-	-	-	-	-	-
		-	-	-	-	-	-	-	-	-	-	-	-	177368	-	-	-	-	-	-	-	-	-	-	-	-	-	-	-	-	-	-	-	-	-	-	-	-	-	-	-	-	-	-	-	-	-	-	-	-	-	-
		-	-	-	-	-	-	-	-	-	-	-	-	177363	-	-	-	-	-	-	-	-	-	-	-	-	-	-	-	-	-	-	-	-	-	-	-	-	-	-	-	-	-	-	-	-	-	-	-	-	-	-
		-	-	-	-	-	-	-	-	-	-	-	-	177357	-	-	-	-	-	-	-	-	-	-	-	-	-	-	-	-	-	-	-	-	-	-	-	-	-	-	-	-	-	-	-	-	-	-	-	-	-	-
		-	-	-	-	-	-	-	-	-	-	-	-	177356	-	-	-	-	-	-	-	-	-	-	-	-	-	-	-	-	-	-	-	-	-	-	-	-	-	-	-	-	-	-	-	-	-	-	-	-	-	-
		-	+	-	-	-	-	-	AT3G21350.1	-	-	-	168225	177355	-	-	-	-	DDB0186006	-	-	-	ENSGALP00000000046	-	-	-	-	-	-	-	-	Os06g11370.1	-	-	-	47270	133130	-	276485	-	-	-	-	-	-	-	-	-	-	-	-	UM00507.1
		-	-	-	-	-	-	-	-	-	-	-	-	177343	-	-	-	-	-	-	-	-	-	-	-	-	-	-	-	-	-	-	-	-	-	-	-	-	-	-	-	-	-	-	-	-	-	-	-	-	-	-
		-	-	-	-	-	-	-	-	-	-	-	-	177336	-	-	-	-	-	-	-	-	-	-	-	-	-	-	-	-	-	-	-	-	-	-	-	-	-	-	-	-	-	-	-	-	-	-	-	-	-	-
		-	-	-	-	-	-	-	-	-	-	-	-	177332	-	-	-	-	-	-	-	-	-	-	-	-	-	-	-	-	-	-	10618	-	-	-	-	-	-	-	-	-	-	-	75.m00165	-	-	-	-	-	-	-
		-	-	-	-	-	-	-	-	-	-	-	-	177330	-	-	-	-	-	-	-	-	-	-	-	-	-	-	-	-	-	Os04g37710.1	-	-	-	120495	-	-	816374	-	-	-	-	-	-	-	-	-	-	-	-	-
NDSE3	nucleoside-diphosphate-sugar epimerase	-	-	-	-	-	-	-	-	-	-	-	-	177328	-	-	-	-	-	-	-	-	-	-	-	-	-	-	-	-	-	-	-	-	-	166484	-	-	-	-	-	-	-	-	-	-	-	-	-	-	-	-
		-	-	-	-	-	-	-	-	7474	-	-	-	177324	-	-	-	-	-	-	-	-	-	-	-	-	-	21180	-	-	-	Os11g14040.1	-	-	-	-	-	-	-	-	-	-	-	-	-	-	-	-	-	-	-	-
		-	-	-	-	-	-	-	-	-	-	-	-	177318	-	-	-	-	-	-	-	-	-	-	-	-	-	-	-	-	-	-	-	-	-	-	-	-	-	-	-	-	-	-	-	-	-	-	-	-	-	-
HDA3412	Histone deacetylase	-	-	-	-	-	-	-	-	-	-	-	-	177316	-	-	-	-	-	-	-	-	-	-	-	-	-	-	-	-	-	-	-	-	-	-	-	-	-	-	-	-	-	-	-	-	-	-	-	-	-	-
		-	-	-	-	-	-	-	-	-	-	-	-	177306	-	-	-	-	-	-	-	-	-	-	-	-	-	-	-	-	-	-	-	-	-	-	-	-	-	-	-	-	-	-	-	-	-	-	-	-	-	-
		-	-	-	-	-	-	-	-	-	-	-	-	-	-	-	-	-	-	-	-	-	-	-	-	-	-	-	-	-	-	-	-	-	-	-	-	-	-	-	-	-	-	-	-	-	-	-	-	-	-	-
		-	-	-	-	-	-	-	-	-	-	-	-	177295	-	-	-	-	-	-	-	-	-	-	-	-	-	-	-	-	-	-	-	-	-	-	-	-	-	-	-	-	-	-	-	-	-	-	-	-	-	-
		-	-	-	-	-	-	-	-	-	-	-	-	177293	-	-	-	-	-	-	-	-	-	-	-	-	-	-	-	-	-	-	-	-	-	-	-	-	-	-	-	-	-	-	-	-	-	-	-	-	-	-
		-	+	-	-	-	-	-	AT5G17660.1	63647	-	-	-	177276	-	-	CMQ129C	-	-	-	-	-	-	-	-	-	-	-	-	-	-	Os01g35170.1	8393	-	14138	55779	156701	-	242124	-	-	-	-	-	-	33718	-	-	-	-	-	-
		-	-	-	-	-	-	-	-	-	-	-	-	177269	-	-	-	-	-	-	-	-	-	-	-	-	-	-	-	-	-	-	-	-	-	-	-	-	-	-	-	-	-	-	-	-	-	-	-	-	-	-
AGO2	Argonaute-like protein	-	-	-	-	-	-	-	-	-	-	-	-	177263	-	-	-	-	-	-	-	-	-	-	-	-	-	-	-	-	-	-	-	-	-	-	-	-	-	-	-	-	-	-	-	-	-	-	-	-	-	-
POLA1	DNA polymerase alpha subunit one.	-	+	+	-	-	-	GB18821-PA	AT5G67100.1	30667	BDEG_01496	CE32262	228337	177262	285501	cgd3_4290	CMI176C	OTTDARP00000022224	DDB0205256	-	-	2.m00554	ENSGALP00000026266	-	OTTHUMP00000023089	LmjF16.1540	109334	107	61128	168297	NCU07870	Os01g64820.1	18655	GSPATP00014027001	-	60547	132002	PFD0590c	218639	RO3G_04261.1	-	SPAC3H5.06c	GLEAN3_00210	SINFRUP00000160398	47.m00199	-	TA03330	37.m00002	49541	-	Tb927.8.4880	UM04529.1
IFM1		-	-	-	-	-	-	-	-	-	-	-	-	177252	-	-	-	-	-	-	-	-	-	-	-	-	-	-	-	-	-	-	-	-	-	-	-	-	-	-	-	-	-	-	-	-	-	-	-	-	-	-
		-	-	-	-	-	-	-	-	-	-	-	-	177241	-	-	-	-	-	-	-	-	-	-	-	-	-	-	-	-	-	-	-	-	-	-	-	-	-	-	-	-	-	-	-	-	-	-	-	-	-	-
		-	+	-	-	-	-	-	-	-	-	-	-	177237	-	-	-	-	-	-	-	10.m00356	ENSGALP00000024430	GL50803_11309	NP_001400.3	-	111806	-	-	-	-	-	-	GSPATP00031274001	-	-	-	-	-	-	-	-	-	-	-	9790	TA16985	-	-	89296.m00054	-	-
		-	+	-	-	-	-	-	-	-	-	CE14412	226664	177235	221918	-	CMK078C	-	DDB0218730	CG9138-PA	-	-	-	-	-	LmjF04.0180	-	25768	-	40018	-	-	15076	-	-	-	131304	MAL7P1.92	-	-	-	-	GLEAN3_26217	SINFRUP00000143670	145.m00121	-	-	25.m02918	-	-	-	-
		-	-	-	-	-	-	-	AT3G56770.1	-	-	-	-	177225	-	-	CML239C	-	-	-	-	-	-	-	-	-	-	-	-	-	-	-	-	-	-	-	-	-	-	-	-	-	-	-	-	-	-	-	-	-	-	-
NRX4	nucleoredoxin	-	+	-	-	-	-	-	-	-	-	CE13792	-	177214	-	-	-	-	-	-	-	-	-	-	-	-	-	34499	-	-	-	Os04g51920.2	-	GSPATP00007863001	-	-	-	PFI0945w	-	-	-	-	-	-	13.m00376	-	-	-	-	-	Tb927.3.3760	-
		-	-	-	-	-	-	-	-	-	-	-	-	177210	-	-	-	-	-	-	-	-	-	-	-	-	-	-	-	-	-	-	-	-	-	-	-	-	-	-	-	-	-	-	-	-	-	-	-	-	-	-
		-	-	-	-	-	-	-	-	-	-	-	-	177207	-	-	-	-	-	-	-	-	-	-	-	-	-	-	-	-	-	-	-	-	-	-	-	-	-	-	-	-	-	-	-	-	-	-	-	-	-	-
		-	-	-	-	-	-	-	-	-	-	-	-	177206	-	-	-	-	-	-	-	-	-	-	-	-	-	-	-	-	-	-	-	-	-	-	-	-	-	-	-	-	-	-	-	-	-	-	-	-	-	-
		-	-	-	-	-	-	-	-	-	-	-	-	177204	-	-	-	-	-	-	-	-	-	-	-	-	-	-	-	-	-	-	-	-	-	-	-	-	-	-	-	-	-	-	-	-	-	-	-	-	-	-
		-	+	+	-	-	-	GB14874-PA	AT2G30910.2	59181	BDEG_00332	CE24672	115556	177203	294403	-	-	OTTDARP00000019645	DDB0204434	CG8978-PA	-	349.m00042	ENSGALP00000007451	-	OTTHUMP00000025215	LmjF18.0920	167800	35994	82184	236117	NCU02781	Os02g57220.1	-	GSPATP00002218001	-	160291	133648	-	824237	RO3G_17081.1	YBR234C	SPBC14C8.06	-	SINFRUP00000155695	37.m00186	-	-	-	60814	81529.m00505	Tb10.389.0270	UM05906.1
		-	-	-	-	-	-	-	-	-	-	-	-	177202	-	-	-	-	-	-	-	-	-	-	-	-	-	-	-	-	-	-	-	-	-	-	-	-	-	-	-	-	-	-	-	-	-	-	-	-	-	-
CYP744C1	cytochrome P450, unkown function, in CYP711 clan	-	-	-	-	-	-	-	-	-	-	-	-	177201	-	-	-	-	-	-	-	-	-	-	-	-	-	-	-	-	-	-	-	-	-	-	-	-	-	-	-	-	-	-	-	-	-	-	-	-	-	-
		-	-	-	-	-	-	-	-	-	-	-	-	177196	-	-	-	-	-	-	-	-	-	-	-	-	-	-	-	-	-	-	-	-	-	-	-	-	-	-	-	-	-	-	-	-	-	-	-	-	-	-
SMG7		-	+	-	-	-	-	GB19528-PA	AT1G28260.2	-	BDEG_07094	-	170037	177188	287108	-	-	-	-	-	-	-	ENSGALP00000007425	-	NP_963862.1	-	225561	33077	68780	86432	NCU00611	Os08g21350.3	-	-	-	96795	129723	-	560381	RO3G_15814.1	-	-	-	SINFRUP00000130266	-	21870	-	-	59186	-	-	UM04177.1
		-	+	-	-	-	-	-	AT3G07220.1	-	-	-	-	177187	-	-	CMP115C	-	DDB0186809	-	-	-	-	GL50803_7800	-	-	-	-	-	-	NCU11368	Os09g32750.1	-	GSPATP00031344001	-	104829	142483	-	711559	RO3G_09853.1	YDR530C	-	-	-	97.m00155	-	-	-	-	86603.m00086	-	UM03318.1
		-	-	-	-	-	-	-	-	-	-	-	-	177173	-	-	-	-	-	-	-	-	-	-	-	-	-	-	-	-	-	-	-	-	-	-	-	-	-	-	-	-	-	-	-	-	-	-	-	-	-	-
PETN	Subunit of the chloroplast cytochrome b6f complex.	-	-	-	-	-	-	-	ATCG00210.1	-	-	-	-	177155	-	-	-	-	-	-	-	-	-	-	-	-	-	-	-	-	-	-	-	-	-	-	-	-	-	-	-	-	-	-	-	-	-	-	-	-	-	-
		-	-	-	-	-	-	-	-	-	-	-	-	177153	-	-	-	-	-	-	-	-	-	-	-	-	-	-	-	-	-	-	-	-	-	-	-	-	-	-	-	-	-	-	-	-	-	-	-	-	-	-
HTR39	Histone H3	-	-	-	-	-	-	-	-	-	-	-	-	-	-	-	-	-	-	-	-	-	-	-	-	-	-	-	-	-	-	-	-	-	-	-	-	-	-	-	-	-	-	-	-	-	-	-	-	-	-	-
		-	-	-	-	-	-	-	-	-	-	-	-	177142	-	-	-	-	-	-	-	-	-	-	-	-	-	-	-	-	-	-	-	-	-	-	-	-	-	-	-	-	-	-	-	-	-	-	-	-	-	-
		-	-	-	-	-	-	-	-	-	-	-	-	177140	-	-	-	-	-	-	-	-	-	-	-	-	-	25261	-	-	-	-	-	-	-	-	-	-	-	-	-	-	-	-	-	-	-	-	-	-	-	-
TIM13	TIM13, import inner membrane translocase subunit, mitochondrial.	-	+	-	-	-	-	GB17630-PA	-	-	-	CE15745	184287	177137	-	-	CMB148C	OTTDARP00000009358	-	CG11611-PA	-	-	-	-	OTTHUMP00000078157	-	100075	-	-	230807	NCU06244	Os02g45820.1	-	-	-	110219	-	-	829931	RO3G_06006.1	-	SPAC17C9.09c	-	SINFRUP00000127332	-	-	-	-	25370	-	-	UM00681.1
		-	+	-	-	-	-	-	AT4G30790.1	-	-	-	-	177124	-	-	-	-	-	-	-	-	-	-	-	-	-	-	79634	-	-	Os02g08350.1	-	-	-	149629	130177	-	832027	-	-	-	-	-	-	-	-	-	-	-	-	-
		-	-	-	-	-	-	-	-	-	-	-	-	177120	-	-	-	-	-	-	-	-	-	-	-	-	-	-	-	-	-	-	-	-	-	-	-	-	-	-	-	-	-	-	-	-	-	-	-	-	-	-
		-	-	-	-	-	-	-	-	-	-	-	-	177118	-	-	-	-	-	-	-	-	-	-	-	-	-	-	-	-	-	-	-	-	-	-	-	-	-	-	-	-	-	-	-	-	-	-	-	-	-	-
		-	+	-	-	-	-	-	-	63054	-	-	-	177116	-	-	-	-	-	-	-	-	-	-	-	LmjF31.1210	-	-	-	-	-	-	-	-	-	-	-	PF14_0084	-	-	-	-	-	-	1449.m00002	23645	-	583.m05712	-	-	-	-
		-	-	-	-	-	-	-	-	-	-	-	-	177104	-	-	-	-	-	-	-	-	-	-	-	-	-	-	-	-	-	-	-	-	-	-	-	-	-	-	-	-	-	-	-	-	-	-	-	-	-	-
		-	-	-	-	-	-	-	-	-	-	-	-	177098	-	-	-	-	-	-	-	-	-	-	-	-	-	-	-	-	-	-	-	-	-	-	-	-	-	-	-	-	-	-	-	-	-	-	-	-	-	-
		-	-	-	-	-	-	-	-	-	-	-	-	177094	-	-	-	-	-	-	-	-	-	-	-	-	-	-	-	-	-	-	-	-	-	-	-	-	-	-	-	-	-	-	-	-	-	-	-	-	-	-
		-	+	-	-	-	-	-	AT1G24793.1	-	-	-	-	177090	-	-	-	-	-	-	-	-	-	-	-	-	-	-	-	-	-	Os03g12320.2	17648	-	-	110980	-	-	231721	-	-	-	-	-	-	-	-	-	-	-	-	-
		-	-	-	-	-	-	-	-	-	-	-	-	177081	-	-	-	-	-	-	-	-	-	-	-	-	-	-	-	-	-	-	-	-	-	-	-	-	-	-	-	-	-	-	-	-	-	-	-	-	-	-
		-	-	-	-	-	-	-	-	-	-	-	-	177061	-	-	-	-	-	-	-	-	-	-	-	-	-	-	-	-	-	-	-	-	-	-	-	-	-	-	-	-	-	-	-	-	-	-	-	-	-	-
		-	-	-	-	-	-	-	-	-	-	-	-	177059	-	-	-	-	-	-	-	-	-	-	-	-	-	-	-	-	-	-	-	-	-	-	-	-	-	-	-	-	-	-	-	-	-	-	-	-	-	-
		-	-	-	-	-	-	-	-	-	-	-	-	177057	-	-	-	-	-	-	-	-	-	-	-	-	-	-	-	-	-	-	-	-	-	-	-	-	-	-	-	-	-	-	-	-	-	-	-	-	-	-
		-	+	-	-	-	-	GB11274-PA	AT2G20410.1	-	-	-	221890	177055	388952	-	-	OTTDARP00000001987	-	CG11710-PB	-	-	ENSGALP00000003639	-	OTTHUMP00000164011	LmjF08.0070	162501	35853	80899	180626	-	Os03g64250.4	-	GSPATP00024340001	-	-	-	-	248095	-	-	-	-	SINFRUP00000146972	-	-	-	59.m03487	58126	-	Tb927.5.3590	-
		-	-	-	-	-	-	-	-	-	-	-	-	177041	-	-	-	-	-	-	-	-	-	-	-	-	-	-	-	-	-	-	-	-	-	-	-	-	-	-	-	-	-	-	-	-	-	-	-	-	-	-
IF2Bb	eukaryotic translation initiation factor 2B, subunit 2	-	+	+	-	-	-	GB10265-PA	AT3G07300.2	-	BDEG_05713	CE20267	162605	177038	210984	cgd5_1600	CMT272C	OTTDARP00000006522	DDB0183786	CG2677-PA	-	93.m00160	ENSGALP00000016737	-	OTTHUMP00000028156	LmjF10.0950	204385	38213	51423	240614	NCU08952	Os10g25320.1	-	GSPATP00031243001	21290	232764	-	PFL2430c	804823	RO3G_12831.1	YLR291C	SPAC343.14c	GLEAN3_04173	SINFRUP00000133481	16.m00336	6056	-	583.m05524	58132	81190.m00232	Tb927.8.4390	UM00997.1
		-	-	-	-	-	-	-	-	-	-	-	-	177032	-	-	-	-	-	-	-	-	-	-	-	-	-	-	-	-	-	-	-	-	-	-	-	-	-	-	-	-	-	-	-	-	-	-	-	-	-	-
		-	-	-	-	-	-	-	-	-	-	-	-	177031	-	-	-	-	-	-	-	-	-	-	-	-	-	-	-	-	-	-	-	-	-	-	-	-	-	-	-	-	-	-	-	-	-	-	-	-	-	-
		-	-	-	-	-	-	-	-	-	-	-	-	177030	-	-	-	-	-	-	-	-	-	-	-	-	-	-	-	-	-	-	-	-	-	-	-	-	-	-	-	-	-	-	-	-	-	-	-	-	-	-
		-	+	-	-	-	-	-	-	-	BDEG_06867	-	-	177027	-	cgd7_110	-	OTTDARP00000011765	DDB0188715	-	-	-	-	-	OTTHUMP00000013784	-	160891	-	79647	-	-	-	33926	GSPATP00007628001	-	-	-	PF14_0493	-	RO3G_02487.1	-	-	-	SINFRUP00000128585	-	-	TA19555	-	64119	-	-	UM05324.1
		-	-	-	-	-	-	-	-	-	-	-	-	177008	-	-	-	-	-	-	-	-	-	-	-	-	-	-	-	-	-	-	-	-	-	-	-	-	-	-	-	-	-	-	-	-	-	-	-	-	-	-
		-	-	-	-	-	-	-	-	-	-	-	-	177005	-	-	-	-	-	-	-	-	-	-	-	-	-	-	-	-	-	-	-	-	-	-	-	-	-	-	-	-	-	-	-	-	-	-	-	-	-	-
		-	-	-	-	-	-	-	-	-	-	-	-	176998	-	-	-	-	-	-	-	-	-	-	OTTHUMP00000080616	-	-	-	-	-	-	-	-	-	-	-	-	-	-	-	-	-	-	-	-	-	-	-	-	-	-	-
		-	-	-	-	-	-	-	AT3G59800.1	-	-	-	-	176996	-	-	-	-	-	-	-	-	-	-	-	-	-	-	-	-	-	-	-	-	-	138240	-	-	216205	-	-	-	-	-	-	-	-	-	-	-	-	-
TIM44	mitochondrial import inner membrane translocase subunit Tim44	-	+	-	-	-	-	GB17122-PA	AT2G36070.1	-	BDEG_06897	CE13473	183511	176995	262379	cgd3_2930	CMJ323C	OTTDARP00000014749	-	-	-	-	ENSGALP00000000471	-	OTTHUMP00000067198	-	127623	37779	50923	-	-	Os07g22700.2	35466	GSPATP00036865001	44351	143404	-	PF11_0265	831627	RO3G_05991.1	YIL022W	SPBC14C8.02	GLEAN3_13396	SINFRUP00000127287	-	262136	TA06570	42.m03586	54556	-	-	UM04594.1
		+	+	-	-	-	-	-	-	72175	BDEG_06092	-	155172	176993	269793	-	-	-	-	-	-	-	ENSGALP00000023381	-	XP_945439.2	LmjF01.0730	219375	-	77673	30054	-	-	-	GSPATP00007873001	-	-	-	-	-	-	-	-	-	SINFRUP00000150241	36.m00254	6295	-	-	28071	96723.m00091	Tb09.160.3240	-
		-	-	-	-	-	-	-	-	-	-	-	-	176992	-	-	-	-	-	-	-	-	-	-	-	-	-	-	-	-	-	-	-	-	-	-	-	-	-	-	-	-	-	-	-	-	-	-	-	-	-	-
		-	+	-	-	-	-	-	-	-	-	-	-	176982	-	-	-	-	-	-	-	-	ENSGALP00000027191	-	OTTHUMP00000018668	-	115858	-	-	159282	-	-	-	-	-	-	-	-	584863	-	-	-	-	-	-	-	-	-	57133	-	-	-
		-	-	-	-	-	-	-	AT2G35500.1	-	-	-	-	176977	-	-	-	-	-	-	-	-	-	-	-	-	-	-	-	-	-	-	-	-	-	-	-	-	817230	-	-	-	-	-	-	-	-	-	-	-	-	-
		-	+	-	-	-	-	-	AT1G79150.1	-	-	-	-	176971	-	-	-	-	-	-	-	-	-	-	-	-	-	-	-	-	-	-	9889	-	-	129150	-	-	-	RO3G_09664.1	-	-	-	-	-	-	-	-	-	-	-	UM03421.1
CYB5_5	putative cytochrome b5 protein	-	-	-	-	-	-	-	-	-	-	-	-	176967	-	-	-	-	-	-	-	-	-	-	-	-	-	-	-	-	-	-	-	-	-	-	-	-	-	-	-	-	-	-	-	-	-	-	-	89348.m00039	-	-
		-	-	-	-	-	-	-	-	-	-	-	-	176958	-	-	-	-	-	-	-	-	-	-	-	-	-	-	-	-	-	-	-	-	-	-	-	-	-	-	-	-	-	-	-	-	-	-	-	-	-	-
		-	-	-	-	-	-	-	-	69496	-	-	-	176946	-	cgd5_1360	-	-	-	-	-	-	-	-	-	-	-	-	-	-	-	-	-	-	-	-	-	-	570318	-	-	-	-	-	-	-	-	-	-	-	-	-
		+	+	-	-	-	-	GB15549-PA	-	62948	-	CE31845	178424	176942	233172	-	-	OTTDARP00000008197	-	CG3829-PA	-	-	ENSGALP00000038958	-	OTTHUMP00000025108	-	165058	-	-	115992	-	-	-	GSPATP00017244001	-	-	142425	-	-	-	-	-	GLEAN3_20799	SINFRUP00000143804	-	-	-	-	56450	-	-	-
		-	-	-	-	-	-	-	-	-	-	-	-	176937	-	-	-	-	-	-	-	-	-	-	-	-	-	-	-	-	-	-	-	-	-	-	-	-	-	-	-	-	-	-	-	-	-	-	-	-	-	-
	hypothetical thioredoxin-like protein	-	-	-	-	-	-	-	-	-	-	-	-	176918	-	-	-	-	-	-	-	-	-	-	-	-	-	-	-	-	-	-	-	-	-	-	-	-	-	-	-	-	-	-	-	-	-	-	-	-	-	-
		-	-	-	-	-	-	-	-	-	-	-	-	176916	-	-	-	-	-	-	-	-	-	-	-	-	-	-	-	-	-	-	-	-	-	-	-	-	-	-	-	-	-	-	-	-	-	-	-	-	-	-
		-	-	-	-	-	-	-	-	-	-	-	-	176913	-	-	-	-	-	-	-	-	-	-	-	-	-	-	-	-	-	-	-	-	-	-	-	-	-	-	-	-	-	-	-	-	-	-	-	-	-	-
		-	-	-	-	-	-	-	-	-	-	-	-	176903	-	-	-	-	-	-	-	-	-	-	-	-	-	-	-	-	-	-	-	-	-	-	-	-	-	-	-	-	-	-	-	-	-	-	-	-	-	-
		-	-	-	-	-	-	-	-	-	-	-	-	176900	-	-	-	-	-	-	-	-	-	-	-	-	-	-	-	-	-	-	-	-	-	-	-	-	-	-	-	-	-	-	-	-	-	-	-	-	-	-
		-	-	-	-	-	-	-	-	-	-	-	-	176895	-	-	-	-	-	-	-	-	-	-	-	-	-	-	-	-	-	-	-	-	-	-	-	-	-	-	-	-	-	-	-	-	-	-	-	-	-	-
		-	-	-	-	-	-	-	-	27566	-	-	-	176894	-	-	-	-	-	-	-	-	-	-	-	-	-	-	64405	-	-	-	35095	-	-	-	-	-	-	-	-	-	-	-	-	262257	-	-	-	-	-	-
		-	-	-	-	-	-	-	-	-	-	-	212491	176892	-	-	-	-	-	-	-	-	ENSGALP00000026697	-	NP_001010906.1	-	-	-	-	-	NCU06980	-	-	-	-	-	-	-	-	-	-	-	-	-	-	-	-	-	-	-	-	-
		-	-	-	-	-	-	-	-	-	-	-	-	176890	-	-	-	-	-	-	-	-	-	-	-	-	-	-	-	-	-	-	-	-	-	-	-	-	-	-	-	-	-	-	-	-	-	-	-	-	-	-
		-	-	-	-	-	-	-	-	-	-	-	-	176889	-	-	-	-	-	-	-	-	-	-	-	-	-	-	-	-	-	-	-	-	-	-	-	-	-	-	-	-	-	-	-	-	-	-	-	-	-	-
		-	-	-	-	-	-	-	-	-	-	-	-	176884	-	-	-	-	-	-	-	-	-	-	-	-	238932	-	-	-	-	-	-	-	-	-	-	-	-	-	-	-	-	-	-	-	-	-	-	-	-	-
		-	-	-	-	-	-	-	-	62188	-	-	-	176879	-	-	-	-	-	-	-	-	-	-	-	-	-	-	-	-	-	-	34755	-	49300	-	-	-	-	-	-	-	-	-	-	21227	-	-	-	-	-	-
		-	-	-	-	-	-	-	-	-	-	-	-	176878	-	-	-	-	-	-	-	-	-	-	-	-	-	-	-	-	-	-	-	-	-	-	-	-	-	-	-	-	-	-	-	-	-	-	-	-	-	-
		-	+	-	-	-	-	-	AT2G01220.1	61408	-	-	-	176872	-	-	CMQ088C	-	-	-	-	3.m00622	-	-	-	LmjF20.0500	-	-	67443	-	-	Os01g49670.3	36797	GSPATP00020438001	-	177711	142653	-	769602	-	-	-	-	-	1.m00812	5414	-	-	-	-	-	-
		-	-	-	-	-	-	-	-	63200	-	-	-	176869	-	-	-	-	-	-	-	-	-	-	-	-	-	-	-	-	-	-	11857	-	49810	-	-	-	-	-	-	-	-	-	-	-	-	-	-	-	-	UM02820.1
		-	-	-	-	-	-	-	-	-	-	-	-	176866	-	-	-	-	-	-	-	-	-	-	-	-	-	-	-	-	-	-	-	-	-	-	-	-	-	-	-	-	-	-	-	-	-	-	-	-	-	-
		-	-	-	-	-	-	-	-	-	-	-	-	176861	-	-	-	-	-	-	-	-	-	-	-	-	-	-	-	-	-	-	-	-	-	-	-	-	-	-	-	-	-	-	-	-	-	-	-	-	-	-
		-	-	-	-	-	-	-	-	-	-	-	-	176850	-	-	-	-	-	-	-	-	-	-	-	-	-	-	-	-	-	-	-	-	-	-	-	-	-	-	-	-	-	-	-	-	-	-	-	-	-	-
PRL4		-	+	-	-	-	-	-	AT3G09590.1	-	-	CE05907	-	176836	293459	-	-	-	-	-	-	-	ENSGALP00000000778	-	OTTHUMP00000016309	-	161350	-	69796	-	-	Os05g51680.1	-	-	-	-	-	-	649730	-	-	-	-	-	-	-	-	46.m01751	-	-	-	-
		+	+	-	-	-	-	-	-	-	-	-	222827	176830	269878	-	-	-	-	-	-	-	ENSGALP00000017565	GL50803_17541	NP_733828.2	LmjF08.0410	-	-	48518	91326	-	-	-	-	-	-	-	-	-	-	-	-	-	SINFRUP00000156634	-	-	-	-	25870	-	Tb927.5.3260	-
		-	-	-	-	-	-	-	-	53245	-	-	-	176829	-	-	-	-	-	-	-	-	-	-	-	-	-	8473	-	-	-	-	-	-	-	-	-	-	555902	-	-	-	-	-	-	-	-	-	-	-	-	-
		-	-	-	-	-	-	-	-	-	-	-	-	176826	-	-	-	-	-	-	-	-	-	-	-	-	-	-	-	-	-	-	-	-	-	-	-	-	-	-	-	-	-	-	-	-	-	-	-	-	-	-
		+	+	-	-	-	-	GB14862-PA	-	-	BDEG_00723	-	43242	176821	262200	-	-	OTTDARP00000019001	-	CG11984-PB	-	-	-	GL50803_15868	NP_872327.2	-	237091	-	-	36807	-	-	-	GSPATP00014223001	-	-	137473	-	-	-	-	-	-	SINFRUP00000182058	65.m00212	21720	-	-	55075	92153.m00175	Tb927.5.2920	-
		-	+	-	-	-	-	GB12229-PA	-	-	BDEG_05205	CE14888	44414	176815	275428	-	-	-	DDB0191830	-	-	2.m00573	ENSGALP00000009081	-	OTTHUMP00000162307	LmjF35.1800	129117	-	39279	128306	-	-	-	-	-	-	129384	-	-	RO3G_06209.1	-	-	GLEAN3_15152	SINFRUP00000133029	46.m00188	4825	-	35.m00917	-	-	Tb09.244.2810	-
		-	-	-	-	-	-	-	-	-	-	-	-	176809	-	-	-	-	-	-	-	-	-	-	-	-	-	-	-	-	-	-	-	-	-	-	-	-	-	-	-	-	-	-	-	-	-	-	-	-	-	-
		-	+	-	-	-	-	-	AT1G79090.2	-	-	-	-	176806	-	-	-	-	-	-	-	-	-	-	-	-	-	-	-	-	-	Os02g19220.2	35255	-	-	116582	-	-	550538	-	-	-	-	-	-	-	-	-	-	-	-	-
		-	-	-	-	-	-	-	-	-	-	-	-	176801	-	-	-	-	-	-	-	-	-	-	-	-	-	-	-	-	-	-	-	-	-	-	-	-	-	-	-	-	-	-	-	-	-	-	-	-	-	-
		-	+	-	-	-	-	GB30370-PA	AT2G32080.2	-	-	CE09497	179772	176799	387101	-	CMJ237C	OTTDARP00000016052	-	CG1507-PD	-	-	ENSGALP00000035043	-	OTTHUMP00000025196	-	121721	-	-	52380	-	Os01g15600.1	-	-	-	179503	-	-	647415	-	-	-	GLEAN3_01859	SINFRUP00000183104	-	-	-	-	51711	-	-	-
		-	+	-	-	-	-	-	AT4G24610.1	-	-	-	-	176794	-	-	-	-	-	-	-	-	-	-	-	-	-	-	-	-	-	Os02g52280.1	24349	-	-	113406	-	-	551641	-	-	-	-	-	-	-	-	-	-	-	-	-
		-	+	-	-	-	-	-	-	-	BDEG_07401	CE25738	91714	176788	-	-	-	-	-	-	-	-	-	-	OTTHUMP00000178915	-	185560	-	-	83210	-	-	-	GSPATP00029503001	-	-	-	-	-	-	-	-	-	SINFRUP00000145061	16.m00287	-	-	-	57071	-	-	-
		-	-	-	-	-	-	-	-	-	-	-	-	176777	-	-	-	-	-	-	-	-	-	-	-	-	-	-	-	-	-	-	-	-	-	-	-	-	-	-	-	-	-	-	-	-	-	-	-	-	-	-
		-	-	-	-	-	-	-	-	-	-	-	-	176774	-	-	-	-	-	-	-	-	-	-	-	-	-	-	-	-	-	-	-	-	-	-	-	-	-	-	-	-	-	-	-	-	-	-	-	-	-	-
		-	-	-	-	-	-	-	-	-	-	-	-	176773	-	-	-	-	-	-	-	-	-	-	-	-	-	-	-	-	-	-	-	-	-	-	-	-	-	-	-	-	-	-	-	-	-	-	-	-	-	-
		-	-	-	-	-	-	-	-	-	-	-	-	176768	-	-	-	-	-	-	-	-	-	-	-	-	-	-	-	-	-	-	-	-	-	-	-	-	-	-	-	-	-	-	-	-	-	-	-	-	-	-
		-	-	-	-	-	-	-	-	-	-	-	-	176760	-	-	-	-	-	-	-	-	-	-	-	-	-	-	-	-	-	-	-	-	-	-	-	-	-	-	-	-	-	-	-	-	-	-	-	-	-	-
		-	-	-	-	-	-	-	-	-	-	-	-	176755	-	-	-	-	-	-	-	-	-	-	-	-	-	-	-	-	-	-	-	-	-	-	-	-	-	-	-	-	-	-	-	-	-	-	-	-	-	-
		-	-	-	-	-	-	-	-	-	-	-	-	176750	-	-	-	-	-	-	-	-	ENSGALP00000030597	-	-	-	-	-	-	-	-	-	-	-	-	-	-	-	580093	-	-	-	-	-	-	-	-	-	-	-	-	-
		-	-	-	-	-	-	-	AT2G19270.1	-	-	-	-	176749	-	-	-	-	-	-	-	-	-	-	-	-	-	-	-	-	-	Os02g07920.1	-	-	-	95922	-	-	652859	-	-	-	-	-	-	-	-	-	-	-	-	-
		-	-	-	-	-	-	-	-	-	-	-	-	176748	-	-	-	-	-	-	-	-	-	-	-	-	-	-	-	-	-	-	-	-	-	-	-	-	-	-	-	-	-	-	-	-	-	-	-	-	-	-
		-	-	-	-	-	-	-	-	-	-	-	-	176734	-	-	-	-	-	-	-	-	-	-	-	-	-	-	-	-	-	-	-	-	-	-	-	-	-	-	-	-	-	-	-	-	-	-	-	-	-	-
ELG10	exostosin-like glycosyltransferase	-	-	-	-	-	-	-	-	-	-	-	-	176731	-	-	-	-	-	-	-	-	-	-	-	-	-	-	-	-	-	-	-	-	-	-	-	-	-	-	-	-	-	-	-	-	-	-	-	-	-	-
		-	-	-	-	-	-	-	-	-	-	-	-	176730	-	-	-	-	-	-	-	-	-	-	-	-	-	-	-	-	-	-	-	-	-	-	-	-	-	-	-	-	-	-	-	-	-	-	-	-	-	-
		-	-	-	-	-	-	-	-	-	-	-	-	176728	-	-	-	-	-	-	-	-	-	-	-	-	-	-	-	-	-	-	-	-	-	-	-	-	-	-	-	-	-	-	-	-	-	-	-	-	-	-
		-	+	-	-	-	-	-	AT1G03780.2	-	BDEG_07664	-	156379	176720	-	-	-	-	-	-	-	-	ENSGALP00000010115	-	-	-	-	9948	45723	-	-	Os07g32390.1	-	-	-	159866	-	-	594654	-	-	-	-	SINFRUP00000162486	4.m00614	-	-	-	-	-	-	-
		-	+	-	-	-	-	-	AT1G55140.1	-	-	-	-	176719	-	-	-	-	-	-	-	-	-	-	-	-	-	-	-	-	-	Os11g13420.2	7359	-	13944	121410	-	-	-	-	-	-	-	-	-	-	-	-	-	-	-	-
STPK5		-	-	-	-	-	-	-	-	-	-	-	-	176718	-	-	-	-	-	-	-	-	-	-	-	-	-	-	-	-	-	-	-	-	-	-	-	-	-	-	-	-	-	-	-	-	-	-	-	-	-	-
		-	-	-	-	-	-	-	-	-	-	-	-	176711	-	-	-	-	-	-	-	-	-	-	-	-	-	-	-	-	-	-	-	-	-	-	-	-	-	-	-	-	-	-	-	-	-	-	-	-	-	-
		-	-	-	-	-	-	-	-	-	-	-	-	176708	-	-	-	-	-	-	-	-	-	-	-	-	-	-	-	-	-	-	-	-	-	-	-	-	-	-	-	-	-	-	-	-	-	-	-	-	-	-
		-	-	-	-	-	-	-	-	-	-	-	-	176707	-	-	-	-	-	-	-	-	-	-	-	-	-	-	-	-	-	-	-	-	-	-	-	-	-	-	-	-	-	-	-	-	-	-	-	-	-	-
HIS5	imidazole glycerol phosphate synthase	-	+	-	-	-	-	-	AT4G26900.1	34011	BDEG_00903	-	-	176694	-	-	-	-	-	-	-	-	-	-	-	-	-	16620	-	235787	NCU07156	Os03g15120.1	12742	-	19324	208049	109060	-	173228	RO3G_10293.1	YBR248C	SPBC418.01c	-	-	-	30478	-	-	-	-	-	UM03858.1
		-	-	-	-	-	-	-	-	-	-	-	-	176684	-	-	-	-	-	-	-	-	-	-	-	-	-	-	-	-	-	-	-	-	-	-	-	-	-	-	-	-	-	-	-	-	-	-	-	-	-	-

		-	-	-	-	-	-	-	AT5G53100.1	-	-	-	-	176680	-	-	-	-	-	-	-	-	-	-	-	-	-	-	-	-	-	-	-	-	-	-	-	-	-	-	-	-	-	-	-	-	-	-	-	-	-	-
		-	-	-	-	-	-	-	-	-	-	-	-	176675	-	-	-	-	-	-	-	-	-	-	-	-	-	-	-	-	-	-	-	-	-	-	-	-	-	-	-	-	-	-	-	-	-	-	-	-	-	-
		-	-	-	-	-	-	-	-	-	-	-	-	176668	-	-	-	-	-	-	-	-	-	-	-	-	-	-	-	-	-	-	-	-	-	-	-	-	-	-	-	-	-	-	-	-	-	-	-	-	-	-
		-	-	-	-	-	-	-	-	-	-	-	-	176666	-	-	-	-	-	-	-	-	-	-	-	-	-	-	-	-	-	-	-	-	-	-	-	-	-	-	-	-	-	-	-	-	-	-	-	-	-	-
		-	-	-	-	-	-	-	-	-	-	-	-	176665	-	-	-	-	-	-	-	-	-	-	-	-	-	-	-	-	-	-	-	-	-	-	-	-	-	-	-	-	-	-	-	-	-	-	-	-	-	-
		-	-	-	-	-	-	-	-	60951	-	-	-	176656	-	-	-	-	-	-	-	-	-	-	-	-	-	-	-	-	-	-	-	GSPATP00029961001	-	-	143184	-	-	-	-	-	-	-	-	-	-	641.m02546	-	-	-	-
		-	-	-	-	-	-	-	-	-	-	-	-	176649	-	-	-	-	-	-	-	-	-	-	-	-	-	-	-	-	-	-	-	-	-	-	-	-	-	-	-	-	-	-	-	-	-	-	-	-	-	-
		-	+	-	-	-	-	-	AT4G02400.1	-	-	-	-	176639	-	-	-	-	-	CG12301-PA	-	-	-	-	-	-	183349	-	-	-	-	Os03g22320.1	32143	-	-	233408	-	-	664422	-	-	-	-	-	-	-	-	-	-	-	-	-
		-	-	-	-	-	-	-	-	-	-	-	-	176634	-	-	-	-	-	-	-	-	-	-	-	-	-	-	-	-	-	-	-	-	-	-	-	-	-	-	-	-	-	-	-	-	-	-	-	-	-	-
		-	-	-	-	-	-	-	-	-	-	-	-	176631	-	-	-	-	-	-	-	-	-	-	-	-	-	-	-	-	-	-	-	-	-	-	-	-	-	-	-	-	-	-	-	-	-	-	-	-	-	-
		-	+	-	-	-	-	GB16618-PA	AT5G42970.1	-	-	-	-	176627	-	-	-	-	DDB0229823	-	-	-	ENSGALP00000018122	-	OTTHUMP00000160811	-	-	-	-	11167	NCU07361	Os03g10120.2	-	-	-	195046	-	-	665031	-	-	-	-	SINFRUP00000136528	-	-	-	-	-	-	-	-
		-	+	-	-	-	-	GB20027-PA	-	-	-	-	225581	176626	260620	-	-	-	DDB0217722	CG12582-PA	-	-	ENSGALP00000020088	-	OTTHUMP00000161766	-	183459	13400	71143	30416	NCU00890	-	-	GSPATP00005634001	-	-	-	-	-	-	-	-	GLEAN3_08458	SINFRUP00000158225	10.m00473	-	-	-	21862	97007.m00147	-	UM05229.1
		-	-	-	-	-	-	-	-	-	-	-	-	176625	-	-	-	-	-	-	-	-	-	-	-	-	-	-	-	-	-	-	-	-	-	-	-	-	-	-	-	-	-	-	-	-	-	-	-	-	-	-
		-	-	-	-	-	-	-	-	-	-	-	-	176622	-	-	-	-	-	-	-	-	-	-	-	-	-	-	-	-	-	-	-	-	-	-	-	-	-	-	-	-	-	-	-	-	-	-	-	-	-	-
		-	-	-	-	-	-	-	-	-	-	-	-	176621	-	-	-	-	-	-	-	-	-	-	-	-	-	-	-	-	-	-	-	-	-	-	-	-	-	-	-	-	-	-	-	-	-	-	-	-	-	-
		-	+	-	-	-	-	-	AT3G62580.1	-	-	-	-	176615	-	-	-	-	-	-	-	-	-	-	-	-	-	-	-	-	-	Os12g37060.1	-	-	40063	-	-	-	644819	-	-	-	-	-	-	31894	-	-	-	-	-	-
		-	+	-	-	-	-	-	AT5G04360.1	67201	-	-	-	176593	-	-	-	-	-	-	-	-	-	-	-	-	-	-	-	-	NCU07619	Os04g08270.1	30315	-	-	167283	-	-	833878	-	-	-	-	-	-	-	-	-	-	-	-	-
		-	-	-	-	-	-	-	-	-	-	-	-	176590	-	-	-	-	-	-	-	-	-	-	-	-	-	-	-	-	-	-	-	-	-	-	-	-	-	-	-	-	-	SINFRUP00000134120	-	-	TA05685	-	-	-	-	-
FAP221	Flagellar Associated Protein	+	+	-	+	-	-	-	-	-	BDEG_04666	-	225888	176588	294940	-	-	-	-	-	-	-	-	GL50803_10034	-	-	144748	29913	-	60162	-	-	-	GSPATP00016605001	-	-	157989	-	-	-	-	-	-	SINFRUP00000156419	15.m00414	-	-	-	54019	-	-	-
		-	-	-	-	-	-	-	-	-	-	-	-	176587	-	-	-	-	-	-	-	-	-	-	-	-	-	-	-	-	-	-	-	-	-	-	-	-	-	-	-	-	-	-	-	-	-	-	-	-	-	-
		-	-	-	-	-	-	GB14994-PA	-	30294	-	-	-	176584	-	-	-	-	-	-	-	-	-	-	-	-	-	-	-	-	-	-	-	-	-	-	-	-	-	-	-	-	-	-	-	-	-	-	-	-	-	-
		-	-	-	-	-	-	-	-	-	-	-	-	176583	-	-	-	-	-	-	-	-	-	-	-	-	-	-	-	-	-	-	-	-	-	-	-	-	-	-	-	-	-	-	-	-	-	-	-	-	-	-
		-	-	-	-	-	-	-	-	-	-	-	-	176572	-	-	-	-	-	-	-	-	-	-	-	-	-	-	-	183264	-	-	-	-	-	-	-	-	-	-	-	-	-	-	-	-	-	-	-	-	-	-
		-	-	-	-	-	-	-	-	-	-	-	-	176570	-	-	-	-	-	-	-	-	-	-	-	-	-	-	-	-	-	-	-	-	-	-	-	-	-	-	-	-	-	-	-	-	-	-	-	-	-	-
		-	-	-	-	-	-	-	-	-	-	-	156921	176569	264923	-	-	-	-	-	-	-	-	-	-	-	-	-	-	-	-	-	-	-	-	-	-	-	-	-	-	-	-	-	-	-	-	-	-	-	-	-
		-	-	-	-	-	-	-	-	-	-	-	-	176568	-	-	-	-	-	-	-	-	-	-	-	-	-	-	-	-	-	-	-	-	-	-	-	-	-	-	-	-	-	-	-	-	-	-	-	-	-	-
		-	-	-	-	-	-	-	-	-	-	-	-	176566	-	-	-	-	-	-	-	-	-	-	-	-	-	-	-	-	-	-	-	-	-	-	-	-	-	-	-	-	-	-	-	-	-	-	-	-	-	-
		-	-	-	-	-	-	-	-	-	-	-	-	176562	-	-	-	-	-	-	-	-	-	-	-	-	-	-	-	-	-	-	-	-	-	-	-	-	-	-	-	-	-	-	-	-	-	-	-	-	-	-
		-	-	-	-	-	-	GB16414-PA	-	-	-	-	-	176559	-	-	-	-	-	-	-	-	-	-	-	-	-	-	-	-	-	-	-	-	-	-	-	-	-	-	-	-	-	-	-	-	-	-	-	-	-	-
		-	-	-	-	-	-	-	-	-	-	-	-	176557	-	-	-	-	-	-	-	-	-	-	-	-	-	-	-	-	-	-	-	-	-	-	-	-	-	-	-	-	-	-	-	-	-	-	-	-	-	-
		-	-	-	-	-	-	-	-	-	-	-	-	176555	-	-	-	-	-	-	-	-	-	-	-	-	-	-	-	-	-	-	-	-	-	-	-	-	-	-	-	-	-	-	-	-	-	-	-	-	-	-
PYRG		-	+	-	-	-	-	GB17378-PA	AT3G12670.1	59818	BDEG_03541	CE29830	148482	176554	228188	cgd5_1710	CMP216C	OTTDARP00000015320	DDB0206047	CG6854-PC	19069573	-	ENSGALP00000000955	GL50803_17587	OTTHUMP00000022986	LmjF20.0560	205900	14740	59376	239752	NCU00261	Os01g46570.2	27383	GSPATP00001512001	50034	218659	109608	PF14_0100	711917	RO3G_03216.1	YJR103W	SPAC10F6.03c	GLEAN3_21934	SINFRUP00000165668	53.m00272	269080	TA15425	129.m00261	50347	-	Tb927.1.1240	UM00770.1
		-	-	-	-	-	-	-	-	-	-	-	-	176537	-	-	-	-	-	-	-	-	-	-	-	-	-	-	-	-	-	-	-	-	-	-	-	-	-	-	-	-	-	-	-	-	-	-	-	-	-	-
		-	-	-	-	-	-	-	-	-	-	-	-	176532	-	-	-	-	-	-	-	-	-	-	-	-	-	-	-	-	-	-	-	-	-	-	-	-	-	-	-	-	-	-	-	-	-	-	-	-	-	-
GTB3401	glycine rich protein	-	+	-	-	-	-	GB11564-PA	AT1G65440.2	-	BDEG_02768	CE13120	160659	176530	245628	-	-	OTTDARP00000021847	DDB0216963	CG12225-PA	-	-	ENSGALP00000000199	-	OTTHUMP00000180867	-	103494	34162	50018	192816	NCU04611	Os05g41510.1	36068	-	47879	118200	129800	-	560322	RO3G_16465.1	YGR116W	SPAC1F7.01c	-	SINFRUP00000130246	-	269534	-	-	59208	-	-	UM00174.1
		-	-	-	-	-	-	-	-	-	-	-	-	176529	-	-	-	-	-	-	-	-	-	-	-	-	-	-	-	-	-	-	-	-	-	-	-	-	-	-	-	-	-	-	-	-	-	-	-	-	-	-
		-	-	-	-	-	-	-	-	-	-	-	-	176526	-	-	-	-	-	-	-	-	-	-	-	-	-	-	-	-	-	-	-	-	-	-	-	-	-	-	-	-	-	-	-	-	-	-	-	-	-	-
		-	-	-	-	-	-	-	-	-	-	-	-	176525	-	-	-	-	-	-	-	-	-	-	-	-	-	-	-	-	-	-	-	-	-	-	-	-	-	-	-	-	-	-	-	-	-	-	-	-	-	-
		-	-	-	-	-	-	-	-	-	-	-	-	176524	-	-	-	-	-	-	-	-	-	-	-	-	-	-	-	-	-	-	-	-	-	-	-	-	-	-	-	-	-	-	-	-	-	-	-	-	-	-
		-	+	-	-	-	-	-	-	-	BDEG_01457	-	-	176522	-	cgd5_310	-	-	DDB0190512	-	19074392	-	-	GL50803_16516	OTTHUMP00000033085	LmjF09.0220	96895	-	-	-	NCU08110	-	34124	-	50019	208238	144090	PFL1455w	-	-	YJL012C	SPCC1322.14c	-	-	-	43150	-	129.m00254	-	-	Tb11.01.4040	UM06230.1
AMX1	copper amine oxidase	-	+	-	-	-	-	-	AT2G42490.1	-	-	-	-	176519	-	-	CMS002C	-	-	-	-	-	-	-	-	-	-	-	-	-	NCU05518	Os04g40040.1	-	-	12808	174842	109693	-	726235	RO3G_05166.1	-	SPAC2E1P3.04	-	-	-	-	-	-	-	-	-	UM03524.1
		-	-	-	-	-	-	-	-	-	-	-	-	176518	-	-	-	-	-	-	-	-	-	-	-	-	-	-	-	-	-	-	-	-	-	-	-	-	-	-	-	-	-	-	-	-	-	-	-	-	-	-
		-	-	-	-	-	-	-	-	-	-	-	-	176517	-	-	-	-	-	-	-	-	-	-	-	-	-	-	-	-	-	-	-	-	-	-	-	-	-	-	-	-	-	-	-	-	-	-	-	-	-	-
		-	-	-	-	-	-	-	-	-	-	-	-	176516	-	-	-	-	-	-	-	-	-	-	-	-	-	-	-	-	-	-	-	-	-	-	-	-	-	-	-	-	-	-	-	-	-	-	-	-	-	-
		-	-	-	-	-	-	-	-	-	-	-	-	176508	-	-	-	-	-	-	-	-	-	-	-	-	-	-	-	-	-	-	-	-	-	-	-	-	-	-	-	-	-	-	-	-	-	-	-	-	-	-
		-	-	-	-	-	-	-	-	-	-	-	-	176490	-	-	-	-	-	-	-	-	-	-	-	-	-	-	-	-	-	-	-	-	-	-	-	-	-	-	-	-	-	-	-	-	-	-	-	-	-	-
		-	-	-	-	-	-	-	-	-	-	-	-	176488	-	-	-	-	-	-	-	-	-	-	-	-	-	-	-	-	-	-	-	-	-	-	-	-	-	-	-	-	-	-	-	-	-	-	-	-	-	-
		-	-	-	-	-	-	-	-	-	-	-	-	176481	-	-	-	-	-	-	-	-	-	-	-	-	-	-	-	-	-	-	-	-	-	-	-	-	-	-	-	-	-	-	-	-	-	-	-	-	-	-
TOR1	target of rapamycin; growth-regulatory PI3K-like protein kinase	-	+	+	-	-	-	GB11213-PA	AT1G50030.1	52494	BDEG_05727	CE32560	155489	176480	269720	-	CMR018C	-	DDB0204659	CG5092-PA	-	177.m00128	ENSGALP00000005274	GL50803_35180	OTTHUMP00000001983	LmjF36.6320	214800	5087	80599	162805	NCU05608	Os05g14550.1	74	GSPATP00018651001	21660	173356	139795	-	175598	RO3G_11879.1	YJR066W	SPBC216.07c	GLEAN3_06054	SINFRUP00000165820	12.m00292	29771	-	583.m05752	57462	88200.m00261	Tb10.6k15.2060	UM03216.1
		-	-	-	-	-	-	-	-	-	-	-	-	176472	-	-	-	-	-	-	-	-	-	-	-	-	-	-	-	-	-	-	-	-	-	-	-	-	-	-	-	-	-	-	-	-	-	-	-	-	-	-
		-	-	-	-	-	-	-	-	-	-	-	-	176471	-	-	-	-	-	-	-	-	-	-	-	-	-	-	-	-	-	-	-	-	-	-	-	-	-	-	-	-	-	-	-	-	-	-	-	-	-	-
		-	-	-	-	-	-	-	-	-	-	-	-	176462	-	-	-	-	-	-	-	-	-	-	-	-	-	-	-	-	-	-	-	-	-	-	-	-	-	-	-	-	-	-	-	-	-	-	-	-	-	-
		-	-	-	-	-	-	-	-	-	-	-	-	176449	-	-	-	-	-	-	-	-	-	-	-	-	-	-	-	-	-	-	-	-	-	-	-	-	-	-	-	-	-	-	-	-	-	-	-	-	-	-
		-	-	-	-	-	-	-	-	-	-	-	-	176446	-	-	-	-	-	-	-	-	-	-	-	-	-	-	-	-	-	-	-	-	-	-	-	-	-	-	-	-	-	-	-	-	-	-	-	-	-	-
		-	-	-	-	-	-	-	-	-	-	-	-	176424	-	-	-	-	-	-	-	-	-	-	-	-	-	-	-	-	-	-	-	-	-	-	-	-	-	-	-	-	-	-	-	-	-	-	-	-	-	-
		-	-	-	-	-	-	-	-	-	-	-	-	176422	-	-	-	-	-	-	-	-	-	-	-	-	-	-	-	-	-	-	-	-	-	-	-	-	-	-	-	-	-	-	-	-	-	-	-	-	-	-
NOP10	Nucleolar protein, Structural Component of H/ACA snoRNPs	-	+	+	-	-	-	GB13763-PA	AT2G20490.1	60207	-	CE27803	198	176419	259005	cgd1_530	CMH198C	-	DDB0187899	CG7637-PA	-	41.m00233	-	GL50803_8242	OTTHUMP00000159812	LmjF36.0340	134010	14965	5785	103779	NCU08903	Os02g40514.2	32703	GSPATP00004125001	9197	197604	-	PF14_0784	830071	RO3G_13539.1	YHR072W-A	SPAP27G11.13c	-	SINFRUP00000156828	99.m00118	42169	TA02850	65.m02513	35903	89678.m00165	Tb10.70.2465	UM03354.1
		-	-	-	-	-	-	-	-	-	-	-	-	176416	-	-	-	-	-	-	-	-	-	-	-	-	-	-	-	-	-	-	-	-	-	-	-	-	-	-	-	-	-	-	-	-	-	-	-	-	-	-
		-	-	-	-	-	-	-	-	-	-	-	-	176415	-	-	-	-	-	-	-	-	-	-	-	-	-	-	-	-	-	-	-	-	-	-	-	-	-	-	-	-	-	-	-	-	-	-	-	-	-	-
		-	-	-	-	-	-	-	-	-	-	-	-	176413	-	-	-	-	-	-	-	-	-	-	-	-	-	-	-	-	-	-	-	-	-	-	-	-	-	-	-	-	-	-	-	-	-	-	-	-	-	-
		-	-	-	-	-	-	-	-	-	-	-	-	176410	-	-	-	-	-	-	-	-	-	-	-	-	-	-	-	-	-	-	-	-	-	-	-	-	-	-	-	-	-	-	-	-	-	-	-	-	-	-
		-	-	-	-	-	-	-	-	-	-	-	-	176409	-	-	-	-	-	-	-	-	-	-	-	-	-	10956	-	-	-	-	-	34059	-	-	-	-	-	-	-	-	-	-	-	-	-	-	-	-	-	-	-	-
FAP205	Flagellar Associated Protein	-	-	-	+	-	-	-	-	-	-	-	-	176405	-	-	-	-	-	-	-	-	-	-	-	-	-	-	-	-	-	-	-	-	-	-	-	-	-	-	-	-	-	-	-	-	-	-	-	-	-	-
TEF27	Unknown function, mitochondrial localization assumed	-	-	-	-	-	-	-	-	-	-	-	-	176396	-	-	-	-	-	-	-	-	-	-	-	-	-	-	-	-	-	-	-	-	-	-	-	-	-	-	-	-	-	-	-	-	-	-	-	-	-	-
		-	-	-	-	-	-	-	-	-	-	-	-	176395	-	-	-	-	-	-	-	-	-	-	-	-	-	-	-	-	-	-	-	-	-	-	-	-	-	-	-	-	-	-	-	-	-	-	-	-	-	-
		-	+	-	-	-	-	GB16950-PA	AT3G09720.1	-	BDEG_00585	-	187719	176386	-	-	-	-	DDB0167865	CG5589-PA	-	-	ENSGALP00000038280	-	NP_689513.2	LmjF32.0570	172563	33989	1095	116885	-	Os07g45360.1	16435	-	32758	41894	111765	-	581044	RO3G_04268.1	YGL171W	SPAC22F3.08c	GLEAN3_05998	SINFRUP00000128018	-	33252	-	52.m01563	33010	-	Tb11.01.5370	UM04754.1
		-	-	-	-	-	-	-	-	-	-	-	-	176384	-	-	-	-	-	-	-	-	-	-	-	-	-	-	-	-	-	-	-	-	-	-	-	-	-	-	-	-	-	-	-	-	-	-	-	-	-	-
		-	+	-	-	-	-	-	-	-	-	-	40643	176383	-	-	-	-	DDB0167391	-	-	-	-	-	-	-	-	-	-	-	-	-	-	-	-	145128	-	-	-	-	-	-	-	SINFRUP00000162117	-	-	-	20.m03712	-	-	-	UM04193.1
		-	-	-	-	-	-	-	-	-	-	-	-	176381	-	-	-	-	-	-	-	-	-	-	-	-	-	-	-	-	-	-	-	-	-	-	-	-	-	-	-	-	-	-	-	-	-	-	-	-	-	-
		-	-	-	-	-	-	-	-	-	-	-	-	176370	-	-	-	-	-	-	-	-	-	-	-	-	-	-	-	-	-	-	-	-	-	-	-	-	-	-	-	-	-	-	-	-	-	-	-	-	-	-
		-	-	-	-	-	-	-	-	-	-	-	-	176365	-	-	-	-	-	-	-	-	-	-	-	-	-	-	-	-	-	-	-	-	-	-	-	-	-	-	-	-	-	-	-	-	-	-	-	-	-	-
		-	-	-	-	-	-	-	-	-	-	-	-	176364	-	-	-	-	-	-	-	-	-	-	-	-	-	-	-	-	-	-	-	-	-	-	-	-	-	-	-	-	-	-	-	-	-	-	-	-	-	-
		-	-	-	-	-	-	-	-	-	-	-	-	176362	-	-	-	-	-	-	-	-	-	-	-	-	-	-	-	-	-	-	-	-	-	-	-	-	-	-	-	-	-	-	-	-	-	-	-	-	-	-
		-	-	-	-	-	-	-	-	-	-	-	-	176361	-	-	-	-	-	-	-	-	-	-	-	-	-	-	-	-	NCU07818	Os08g06100.1	-	-	-	-	-	-	-	-	-	-	-	-	-	-	-	-	-	-	-	-
		-	-	-	-	-	-	-	-	-	-	-	-	176359	-	-	-	-	-	-	-	-	-	-	-	-	-	-	-	-	-	-	31711	-	-	212537	-	-	-	-	-	-	-	-	-	-	-	-	-	-	-	-
		-	-	-	-	-	-	-	-	-	-	-	-	176358	-	-	-	-	-	-	-	-	-	-	-	-	-	-	-	-	-	-	-	-	-	-	-	-	-	-	-	-	-	-	-	-	-	-	-	-	-	-
		-	-	-	-	-	-	-	-	-	-	-	-	176340	-	-	-	-	-	-	-	-	-	-	-	-	-	-	-	-	-	-	-	-	-	-	-	-	-	-	-	-	-	-	-	-	-	-	-	-	-	-
SAS-6		-	+	-	+	-	-	GB15158-PA	-	-	BDEG_07637	-	198072	176336	271175	-	CMX010C	OTTDARP00000009325	-	-	-	-	ENSGALP00000008489	-	OTTHUMP00000012455	LmjF35.4280	-	29189	68996	-	-	-	30207	GSPATP00008149001	-	66367	143809	-	-	-	-	-	-	SINFRUP00000151492	41.m00222	23096	-	541.m01235	-	-	Tb09.211.1930	-
MAPKKK7	Mitogen Activated Protein Kinase Kinase Kinase Homolog 7	-	-	-	-	-	-	-	-	-	-	-	-	176335	-	-	-	-	-	-	-	-	-	-	-	-	-	-	-	-	-	-	-	-	-	-	-	-	-	-	-	-	-	-	-	-	-	-	-	-	-	-
		-	+	-	-	-	-	GB16968-PA	AT1G71360.1	-	BDEG_06639	CE28764	97284	176330	290339	-	-	OTTDARP00000004412	DDB0186751	CG31678-PA	-	-	ENSGALP00000004863	-	NP_057311.1	LmjF30.0320	125008	-	73406	-	NCU00119	Os01g41600.1	-	GSPATP00033744001	45250	71659	138483	-	554505	RO3G_08483.1	YOR154W	SPBC3E7.09	-	SINFRUP00000145917	51.m00209	24613	-	-	53396	-	Tb927.6.1740	UM04407.1
		-	-	-	-	-	-	-	-	-	-	-	-	176321	-	-	-	-	-	-	-	-	-	-	-	-	-	-	-	-	-	-	-	-	-	-	-	-	-	-	-	-	-	-	-	-	-	-	-	-	-	-
		-	-	-	-	-	-	-	-	-	-	-	-	176317	-	-	-	-	-	-	-	-	-	-	-	-	-	-	-	-	-	-	-	-	-	-	-	-	-	-	-	-	-	-	-	-	-	-	-	-	-	-
		-	+	-	-	-	-	GB12388-PA	AT3G05070.1	-	BDEG_05650	CE27517	179668	176312	282989	-	-	OTTDARP00000021537	DDB0184501	-	-	-	ENSGALP00000008798	-	OTTHUMP00000170950	-	233303	26027	-	238072	-	Os06g51420.1	-	GSPATP00019651001	44784	8736	130081	-	817783	RO3G_10424.1	-	-	-	-	120.m00109	7902	-	69.m00202	53602	-	-	-
ADCY11	adenylate cyclase	-	-	-	-	-	-	-	-	-	-	-	-	176311	-	-	-	-	-	-	-	-	-	-	-	-	-	-	-	-	-	-	-	-	-	-	-	-	-	-	-	-	-	-	-	-	-	-	-	-	-	-
		-	-	-	-	-	-	-	-	28375	-	-	-	176301	-	-	-	-	-	-	-	-	-	-	-	-	-	-	-	-	-	-	-	-	-	15562	-	-	-	-	-	-	-	-	-	-	-	-	-	-	-	-
		-	+	-	-	-	-	GB10296-PA	AT2G22010.1	70837	BDEG_07962	CE17679	219187	176296	216164	-	-	OTTDARP00000013056	-	-	-	321.m00062	ENSGALP00000003908	-	OTTHUMP00000171171	-	234381	31870	-	93222	-	-	-	-	45020	10281	157491	-	217827	RO3G_02342.1	-	-	-	SINFRUP00000145629	-	7627	-	-	64223	-	-	-
		-	-	-	-	-	-	-	-	-	-	-	-	176288	-	-	-	-	-	-	-	-	-	-	-	-	-	-	-	-	-	-	-	-	-	-	-	-	-	-	-	-	-	-	-	-	-	-	-	-	-	-
		-	-	-	-	-	-	-	-	-	-	-	-	176285	-	-	-	-	-	-	-	-	-	-	-	-	-	-	-	-	-	-	-	-	-	-	-	-	-	-	-	-	-	-	-	-	-	-	-	-	-	-
		-	-	-	-	-	-	-	AT3G01720.1	-	-	-	-	176269	-	-	-	-	-	-	-	-	-	-	-	-	-	-	-	-	-	Os01g67750.1	-	-	44709	-	-	-	174246	-	-	-	-	-	-	-	-	-	-	-	-	-
		-	-	-	+	-	-	-	-	-	-	-	-	176257	-	-	-	-	-	-	-	-	-	-	-	-	-	-	-	-	-	-	-	-	-	-	-	-	-	-	-	-	-	-	-	-	-	-	-	-	-	-
		-	+	-	-	-	-	-	AT1G54570.1	-	-	-	-	176249	-	-	-	-	-	-	-	-	-	-	-	-	-	-	-	-	-	Os01g26039.1	18770	-	43541	135745	-	-	174651	-	-	-	-	-	-	-	-	-	-	-	-	-
		-	-	-	-	-	-	-	-	-	-	-	-	176247	-	-	-	-	-	-	-	-	-	-	-	-	-	-	-	-	-	-	-	-	-	-	-	-	-	-	-	-	-	-	-	-	-	-	-	-	-	-
CYC	cytochrome c	-	+	-	-	-	-	GB19293-PA	AT4G10040.1	59926	BDEG_06931	CE16968	21934	176233	239074	-	CMJ212C	OTTDARP00000023027	DDB0217692	CG17903-PA	-	-	ENSGALP00000017934	-	OTTHUMP00000158609	LmjF16.1320	219589	28483	77897	164353	NCU01808	Os05g34770.1	7786	GSPATP00014720001	20948	159360	109170	PF14_0038	738754	RO3G_09833.1	YJR048W	SPCC191.07	GLEAN3_16096	SINFRUP00000181158	128.m00127	41530	TA12950	38.m01073	37186	-	Tb927.8.5120	UM02708.1
		-	-	-	-	-	-	-	-	-	-	-	-	176230	-	-	-	-	-	-	-	-	-	-	-	-	-	-	-	-	-	-	-	-	-	-	-	-	-	-	-	-	-	-	-	-	-	-	-	-	-	-
		-	-	-	-	-	-	-	-	-	-	-	-	176229	-	-	-	-	-	-	-	-	-	-	-	-	-	-	82293	-	-	-	-	-	-	-	-	-	-	-	-	-	-	-	-	-	-	-	-	-	-	-
		-	-	-	-	-	-	-	-	-	-	-	-	176214	-	-	-	-	-	-	-	-	-	-	-	-	-	-	-	-	-	-	-	-	-	-	-	-	-	-	-	-	-	-	-	-	-	-	-	-	-	-
		-	-	-	-	-	-	-	-	-	-	-	-	176211	-	-	-	-	-	-	-	-	-	-	-	-	-	-	-	-	-	-	-	-	-	-	-	-	-	-	-	-	-	-	-	20964	-	-	-	-	-	-
		-	-	-	-	-	-	-	-	-	-	-	-	176208	-	-	-	-	-	-	-	-	-	-	-	-	-	-	-	-	-	-	-	-	-	-	-	-	-	-	-	-	-	-	-	-	-	-	-	-	-	-
		-	-	-	-	-	-	-	-	-	-	-	-	176207	-	-	-	-	-	-	-	-	-	-	-	-	-	-	-	-	-	-	-	-	-	104757	-	-	-	-	-	-	-	-	-	-	-	-	-	-	-	-
POLI2	DNA polymerase eta/iota	-	-	-	-	-	-	-	-	-	-	-	-	176206	-	-	-	-	-	-	-	-	-	-	-	-	-	-	-	-	-	-	-	-	-	-	-	-	-	-	-	-	-	-	-	-	-	-	-	-	-	-
		-	-	-	-	-	-	-	-	-	-	-	-	176193	-	-	CMM061C	-	-	-	-	-	-	-	-	-	-	-	-	-	-	-	-	-	-	-	-	-	570757	-	-	-	-	-	-	-	-	-	-	-	-	-
		-	-	-	-	-	-	-	-	-	-	-	-	176182	-	-	-	-	-	-	-	-	-	-	-	-	-	-	-	-	-	-	-	-	-	-	-	-	-	-	-	-	-	-	-	-	-	-	-	-	-	-
		-	-	-	-	-	-	-	AT4G33210.1	-	-	-	-	176179	-	-	-	-	-	-	-	-	-	-	-	-	-	-	-	-	-	Os10g25680.1	-	-	-	197139	-	-	260487	-	-	-	-	-	-	-	-	-	-	-	-	-
		-	-	-	-	-	-	-	-	-	-	-	-	176176	-	-	-	-	-	-	-	-	-	-	-	-	-	-	-	-	-	-	-	-	-	-	-	-	-	-	-	-	-	-	-	-	-	-	-	-	-	-
		-	-	-	-	-	-	-	-	-	-	-	-	176173	-	-	-	-	-	-	-	-	-	-	-	-	-	-	-	-	-	-	-	-	-	-	-	-	-	-	-	-	-	-	-	-	-	-	-	-	-	-
		-	-	-	-	-	-	-	-	-	-	-	-	176172	-	-	-	-	-	-	-	-	-	-	-	-	-	-	-	-	-	-	-	-	-	-	-	-	-	-	-	-	-	-	-	-	-	-	-	-	-	-
		-	-	-	-	-	-	-	-	-	-	-	-	176171	-	-	-	-	-	-	-	-	-	-	-	-	-	-	-	-	-	-	-	-	-	-	-	-	-	-	-	-	-	-	-	-	-	-	-	-	-	-
		-	-	-	-	-	-	-	-	-	-	-	-	176170	-	-	-	-	-	-	-	-	-	-	-	-	-	-	-	-	-	-	-	-	-	-	-	-	-	-	-	-	-	-	-	-	-	-	-	-	-	-
		-	-	-	-	-	-	-	-	-	-	-	-	176169	-	-	-	-	-	-	-	-	-	-	-	-	-	-	-	-	-	-	-	-	-	-	-	-	-	-	-	-	-	-	-	-	-	-	-	-	-	-
		-	-	-	-	-	-	-	-	-	-	-	-	176167	-	-	-	-	-	-	-	-	-	-	-	-	-	-	-	-	-	-	-	-	-	-	-	-	-	-	-	-	-	-	-	-	-	-	-	-	-	-
		-	-	-	-	-	-	-	-	-	-	-	-	176166	-	-	-	-	-	-	-	-	-	-	-	-	-	-	-	-	-	-	-	-	-	-	-	-	-	-	-	-	-	-	-	-	-	-	-	-	-	-
		-	-	-	-	-	-	-	-	-	-	-	-	176165	-	-	-	-	-	-	-	-	-	-	-	-	-	-	-	-	-	-	-	-	-	-	-	-	-	-	-	-	-	-	-	-	-	-	-	-	-	-
		-	-	-	-	-	-	-	-	-	-	-	-	176163	-	-	-	-	-	-	-	-	-	-	-	-	-	-	-	-	-	-	-	-	-	-	-	-	-	-	-	-	-	-	-	-	-	-	-	-	-	-
HCS3	cytochrome c heme lyase (CCHL) (holocytochrome c synthase)	-	-	-	-	-	-	-	-	-	-	-	-	176161	-	-	-	-	-	-	-	-	-	-	-	-	-	-	-	-	-	-	-	-	-	-	-	-	-	-	-	-	-	-	-	-	-	-	-	-	-	-
STPK17	Serine/Threonine Protein Kinase Homolog 17	-	-	-	-	-	-	-	-	-	-	-	-	176160	-	-	-	-	-	-	-	-	-	-	-	-	-	-	-	-	-	-	-	-	-	-	-	-	-	-	-	-	-	-	-	-	-	-	-	-	-	-
		-	+	-	-	-	-	GB13733-PA	AT2G41020.1	-	-	CE31622	152591	176140	206174	-	-	OTTDARP00000001267	-	-	-	-	-	-	OTTHUMP00000025808	-	116530	-	-	218753	-	Os01g68760.1	34643	-	-	146595	-	-	652518	-	-	-	-	SINFRUP00000177615	-	-	-	-	55399	-	-	-
		-	-	-	-	-	-	-	-	-	-	-	-	176133	-	-	-	-	-	-	-	-	-	-	-	-	-	-	-	-	-	-	-	-	-	-	-	-	-	-	-	-	-	-	-	-	-	-	-	-	-	-
		-	+	-	-	-	-	GB18817-PA	-	16401	-	-	47512	176119	-	-	CMM105C	OTTDARP00000020777	-	-	-	-	ENSGALP00000006740	-	OTTHUMP00000115539	-	-	14719	69068	244663	-	-	36402	GSPATP00017854001	-	-	138429	-	-	RO3G_16156.1	-	-	GLEAN3_21946	SINFRUP00000138140	1.m00773	-	-	-	21035	-	-	-
		-	-	-	-	-	-	-	-	-	-	-	-	176117	-	-	-	-	-	-	-	-	-	-	-	-	-	-	-	-	-	-	-	-	-	-	-	-	-	-	-	-	-	-	-	-	-	-	-	-	-	-
		-	+	-	-	-	-	-	-	71955	BDEG_07608	-	-	176101	-	-	-	-	-	-	-	-	-	-	-	LmjF16.0960	58001	-	-	224984	NCU04764	-	32423	GSPATP00029532001	34078	-	132021	-	-	RO3G_02300.1	YDR316W	SPBC389.04	-	-	8.m00521	2944	TA04405	-	-	-	Tb927.8.5420	-
RPA1		-	+	-	-	-	-	GB12611-PA	AT3G57660.1	65384	BDEG_03907	CE22157	154177	176094	269737	-	CMK037C	OTTDARP00000021613	DDB0202491	CG10122-PA	19068861	-	ENSGALP00000035290	-	NP_056240.2	-	152026	16642	53	110368	NCU01638	-	9009	GSPATP00017474001	-	-	141082	-	560551	RO3G_08187.1	YOR341W	SPBC4C3.05c	GLEAN3_10926	SINFRUP00000157438	3.m01913	40884	TA05785	-	27970	138136.m00002	-	UM02628.1
		-	-	-	-	-	-	-	-	-	-	-	-	176089	-	-	-	-	-	-	-	-	-	-	-	-	-	-	-	248535	-	-	-	-	-	-	-	-	-	-	-	-	-	-	-	-	-	-	-	-	-	-
		-	-	-	-	-	-	-	AT5G65950.1	-	-	-	-	176087	-	-	-	-	-	-	-	-	-	-	-	-	-	-	-	-	-	Os02g48380.2	-	-	-	-	-	-	772085	-	-	-	-	-	-	-	-	-	-	-	-	-
		-	-	-	-	-	-	-	-	-	-	-	-	176085	-	-	-	-	-	-	-	-	-	-	-	-	-	-	-	-	-	-	-	-	-	-	-	-	-	-	-	-	-	-	-	-	-	-	-	-	-	-
	putative regulator of chromosome condensation	-	-	-	-	-	-	-	-	-	-	-	-	176084	-	-	-	-	-	-	-	-	-	-	-	-	-	-	-	-	-	-	-	-	-	-	-	-	-	-	-	-	-	-	-	-	-	-	-	-	-	-
		-	-	-	-	-	-	-	-	-	-	-	-	176079	-	-	-	-	-	-	-	-	-	-	-	-	-	-	-	-	-	-	-	-	-	-	-	-	-	-	-	-	-	-	-	-	-	-	-	-	-	-
		-	-	-	-	-	-	-	-	-	-	-	-	176078	-	-	-	-	-	-	-	-	-	-	-	-	-	-	-	-	-	-	-	-	-	-	-	-	-	-	-	-	-	-	-	-	-	-	-	-	-	-
FSA1	Putative fructose-6-P aldolase	-	+	-	-	-	-	-	-	15080	-	-	-	176076	-	-	-	-	-	-	-	-	-	-	-	-	-	-	-	-	-	-	6272	-	20779	97381	-	-	-	-	-	-	-	-	-	4331	-	-	-	-	-	-
		-	-	-	-	-	-	-	-	-	-	-	-	176075	-	-	-	-	-	-	-	-	-	-	-	-	-	-	-	-	-	-	-	-	-	-	-	-	-	-	-	-	-	-	-	-	-	-	-	-	-	-
		-	-	-	-	-	-	-	-	-	-	-	-	176067	-	-	-	-	-	-	-	-	-	-	-	-	-	-	-	-	-	-	-	-	-	-	-	-	-	-	-	-	-	-	-	-	-	-	-	-	-	-
		-	-	-	-	-	-	-	-	-	-	-	-	176066	-	-	-	-	-	-	-	-	-	-	-	-	-	-	-	-	-	-	-	-	-	-	-	-	-	-	-	-	-	-	-	-	-	-	-	-	-	-
		-	-	-	-	-	-	-	-	-	-	-	-	176053	-	-	-	-	-	-	-	-	-	-	-	-	-	-	-	-	-	-	-	-	-	-	-	-	-	-	-	-	-	-	-	-	-	-	-	-	-	-
		-	+	-	-	-	-	GB14076-PA	AT5G24740.1	-	-	-	-	176049	-	-	-	-	-	-	-	-	-	-	-	-	-	-	-	248208	-	Os08g02350.1	-	-	44551	94056	-	-	550521	-	-	-	-	-	-	-	-	-	-	-	-	-
		-	-	-	-	-	-	-	-	-	-	-	-	176048	-	-	-	-	-	-	-	-	-	-	-	-	-	-	-	-	-	-	-	-	-	-	-	-	-	-	-	-	-	-	-	-	-	-	-	-	-	-
		-	-	-	-	-	-	-	-	-	-	-	-	176040	-	-	-	-	-	-	-	-	-	-	-	-	-	-	-	-	-	-	-	-	-	-	-	-	-	-	-	-	-	-	-	-	-	-	-	-	-	-
		-	+	-	-	-	-	GB12850-PA	AT5G19930.1	-	-	-	219280	176026	-	-	-	-	-	CG10171-PB	-	-	ENSGALP00000016531	-	OTTHUMP00000168500	-	134535	-	30273	177046	-	Os01g32280.1	5344	-	-	-	136940	-	737545	-	-	-	-	SINFRUP00000153828	-	-	-	-	-	-	-	UM06135.1
		-	+	-	-	-	-	-	AT4G04180.1	-	-	-	-	176020	-	cgd1_2180	CML131C	-	-	-	-	-	-	-	-	-	-	-	-	-	-	Os02g50680.1	-	GSPATP00035954001	-	-	136490	-	274621	-	-	-	-	-	20.m00360	-	-	-	-	-	-	-
		-	-	-	-	-	-	-	-	-	-	-	-	176016	-	-	-	-	-	-	-	-	-	-	-	-	-	29872	-	-	-	-	-	-	-	-	-	-	-	-	-	-	-	-	-	268435	-	-	-	-	-	-
		-	-	-	-	-	-	-	-	-	-	-	-	176014	-	-	-	-	-	-	-	-	-	-	-	-	-	-	-	-	-	-	-	-	-	-	-	-	-	-	-	-	-	-	-	-	-	-	-	-	-	-
		-	-	-	-	-	-	-	-	-	-	-	-	176011	-	-	-	-	-	-	-	-	-	-	-	-	-	-	-	-	-	-	-	-	-	-	-	-	-	-	-	-	-	-	-	-	-	-	-	-	-	-
		-	+	-	-	-	-	GB15076-PA	AT4G31160.1	-	-	CE33014	224355	176008	243147	-	-	-	-	CG10080-PA	-	419.m00051	ENSGALP00000003768	-	NP_055518.1	LmjF33.1780	184499	-	59453	93064	-	Os04g59460.1	11577	-	-	233514	-	-	271685	-	-	-	-	SINFRUP00000133793	-	-	-	-	-	-	Tb11.02.0690	-
		-	-	-	-	-	-	-	-	-	-	-	-	175993	-	-	-	-	-	-	-	-	-	-	-	-	-	-	67363	-	NCU09412	-	-	-	-	169264	127244	-	-	-	-	-	-	-	-	-	-	-	-	-	-	-
		-	-	-	-	-	-	-	-	-	-	-	-	175984	-	-	-	-	-	-	-	-	-	-	-	-	-	-	-	-	-	-	-	-	-	-	-	-	-	-	-	-	-	-	-	-	-	-	-	-	-	-
		-	-	-	-	-	-	-	-	-	-	-	-	175974	-	-	-	-	-	-	-	-	-	-	-	-	-	-	-	-	-	-	-	-	-	-	-	-	-	-	-	-	-	-	-	-	-	-	-	-	-	-
		-	-	-	-	-	-	-	-	-	-	-	-	175973	-	-	-	-	-	-	-	-	-	-	-	-	-	-	-	-	-	-	-	-	-	-	-	-	-	-	-	-	-	-	-	-	-	-	-	-	-	-
		-	-	-	-	-	-	-	-	65103	-	-	-	175972	-	-	-	-	-	-	-	-	-	-	-	-	-	-	-	-	-	-	-	-	-	-	-	-	-	-	-	-	-	-	-	-	-	-	-	-	-	-
		-	+	-	-	-	-	-	-	63013	-	-	-	175950	-	-	-	-	-	-	-	-	-	-	-	LmjF36.4260	-	-	-	-	-	-	-	GSPATP00018707001	-	76327	138073	-	-	-	-	-	-	-	41.m00239	23345	-	-	-	86756.m00081	Tb10.6k15.0710	-
		-	+	-	-	-	-	-	-	12176	-	-	-	175931	-	-	-	-	-	-	-	-	-	-	-	-	-	-	-	-	-	-	37778	-	44096	162500	-	-	579226	-	-	-	-	-	-	21929	-	-	-	-	-	-
		-	+	-	-	-	-	-	AT4G34370.1	-	-	CE08416	96432	175924	296877	-	-	-	-	-	-	-	-	-	-	-	178640	32739	34212	-	-	Os03g13100.1	-	-	-	133395	-	-	821209	-	-	-	-	-	-	-	-	-	-	-	-	-
		-	-	-	-	-	-	-	-	-	-	-	-	175917	-	-	-	-	-	-	-	-	-	-	-	-	-	-	-	-	-	-	-	-	-	-	-	-	-	-	-	-	-	-	-	-	-	-	-	-	-	-
		-	+	-	-	-	-	-	-	61365	BDEG_01944	-	-	175912	-	-	-	-	-	-	-	-	-	-	-	-	-	-	-	31622	NCU11204	-	10632	-	50012	-	129494	-	-	-	-	-	-	-	-	20656	-	541.m01156	-	-	-	UM02953.1
		-	-	-	-	-	-	-	-	-	-	-	-	175903	-	-	-	-	-	-	-	-	-	-	-	-	-	-	-	-	-	-	-	-	-	-	-	-	-	-	-	-	-	-	-	-	-	-	-	-	-	-
		-	-	-	-	-	-	-	-	-	-	-	-	175901	-	-	-	-	-	-	-	-	-	-	-	-	-	-	-	-	-	-	-	-	-	-	-	-	-	-	-	-	-	-	-	-	-	-	-	-	-	-
		-	-	-	-	-	-	-	-	-	-	-	-	175900	-	-	-	-	-	-	-	-	-	-	-	-	-	-	-	-	-	-	-	-	-	-	-	-	-	-	-	-	-	-	-	-	-	-	-	-	-	-
		-	-	-	-	-	-	-	-	-	-	-	-	175899	-	-	-	-	-	-	-	-	-	-	-	-	-	-	-	-	-	-	-	-	-	-	-	-	-	-	-	-	-	-	-	-	-	-	-	-	-	-
		-	-	-	-	-	-	-	-	-	-	-	-	175886	-	-	-	-	-	-	-	-	-	-	-	-	-	-	-	-	-	-	-	-	-	-	-	-	-	-	-	-	-	-	-	-	-	-	-	-	-	-

		-	+	-	-	-	-	GB15318-PA	AT1G58350.1	-	BDEG_07604	CE34256	90541	175885	274109	cgd3_50	-	-	DDB0206251	CG32333-PA	-	-	ENSGALP00000026064	GL50803_90418	NP_001099001.1	-	102128	10487	53507	86648	-	Os12g06800.1	-	GSPATP00005993001	-	-	-	-	249517	-	-	-	-	SINFRUP00000129326	275.m00019	-	-	52.m01672	58113	-	-	-
		-	-	-	-	-	-	-	-	-	-	-	-	175880	-	-	-	-	-	-	-	-	-	-	-	-	-	-	-	-	-	-	-	-	-	-	-	-	-	-	-	-	-	-	-	-	-	-	-	-	-	-
		-	-	-	-	-	-	-	-	-	-	-	-	175870	-	-	-	-	-	-	-	-	-	-	-	-	-	-	-	-	-	-	-	-	-	-	-	-	-	-	-	-	-	-	-	-	-	-	-	-	-	-
		-	-	-	-	-	-	-	-	-	-	-	-	175867	-	-	-	-	-	-	-	-	-	-	-	-	-	-	-	-	-	-	-	-	-	-	-	-	-	-	-	-	-	-	-	-	-	-	-	-	-	-
		-	-	-	-	-	-	-	-	-	-	-	-	175866	-	-	-	-	-	-	-	-	-	-	-	-	-	-	-	-	-	-	-	-	-	-	-	-	-	-	-	-	-	-	-	-	-	-	-	-	-	-
		-	-	-	-	-	-	-	-	-	-	-	-	175858	-	-	-	-	-	-	-	-	-	-	-	-	-	-	-	-	-	-	-	-	-	-	-	-	-	-	-	-	-	-	-	-	-	-	-	-	-	-
		-	+	-	-	-	-	GB13048-PA	AT2G30320.1	12283	BDEG_04399	CE20158	83969	175853	242243	-	CMH251C	-	DDB0204564	-	-	15.m00311	ENSGALP00000003706	-	OTTHUMP00000158623	LmjF34.0310	117320	32742	80005	32495	NCU07528	Os03g21980.1	14069	-	-	140242	-	-	239920	-	YPL212C	-	-	SINFRUP00000153426	-	33985	-	-	57795	83237.m00068	Tb927.7.1510	UM05416.1
		-	-	-	-	-	-	-	-	-	-	-	-	175847	-	-	-	-	-	-	-	-	-	-	-	-	-	-	-	-	-	-	-	-	-	-	-	-	-	-	-	-	-	-	-	-	-	-	-	-	-	-
		-	-	-	-	-	-	-	-	-	-	-	-	175846	253008	-	-	-	-	-	-	-	-	-	-	-	-	-	-	-	-	-	-	-	-	-	-	-	-	-	-	-	-	-	-	-	-	-	-	-	-	-
		-	-	-	-	-	-	-	-	-	-	-	-	175841	-	-	-	-	-	-	-	-	-	-	-	LmjF33.0390	-	-	-	-	-	-	-	-	-	-	141861	-	-	-	-	-	-	-	81.m00224	-	-	-	-	-	Tb10.26.1040	-
		-	-	-	-	-	-	-	-	-	-	-	-	175837	-	-	-	-	-	-	-	-	-	-	-	-	-	-	-	-	-	-	-	-	-	-	-	-	-	-	-	-	-	-	-	-	-	-	-	-	-	UM06333.1
		-	-	-	-	-	-	-	-	-	-	-	-	175836	-	-	-	-	-	-	-	-	-	-	-	-	-	-	-	-	-	-	-	-	-	-	-	-	-	-	-	-	-	-	-	-	-	-	-	-	-	-
		-	-	-	-	-	-	-	-	-	-	-	-	175835	-	-	-	-	-	-	-	-	-	-	-	-	-	-	-	-	-	-	-	-	-	-	-	-	-	-	-	-	-	-	-	-	-	-	-	-	-	-
MAPKKK6	Mitogen Activated Protein Kinase Kinase Kinase Homolog 6	-	-	-	-	-	-	-	-	-	-	-	-	175829	-	-	-	-	-	-	-	-	-	-	-	-	-	-	-	-	-	-	-	-	-	-	-	-	-	-	-	-	-	-	-	-	-	-	-	-	-	-
		-	-	-	-	-	-	-	-	-	-	-	222889	175823	-	-	-	-	-	-	-	-	-	-	OTTHUMP00000017021	-	-	-	67068	-	-	-	-	-	-	-	-	-	-	-	-	-	-	-	-	-	-	-	-	-	-	-	-
		-	-	-	-	-	-	-	-	-	-	-	-	175817	-	-	CMS436C	-	-	-	-	-	-	-	-	-	-	-	-	-	-	-	-	-	-	-	-	-	-	-	-	-	-	-	-	-	-	-	-	-	-	-
		-	-	-	-	-	-	-	-	-	-	-	-	175816	-	-	-	-	-	-	-	-	-	-	-	-	-	-	-	-	-	-	-	-	-	-	-	-	-	-	-	-	-	-	-	-	-	-	-	-	-	-
		-	+	+	-	-	-	GB15255-PA	AT1G27980.1	27290	BDEG_00886	CE20348	149914	175809	390668	-	CMT631C	-	DDB0205010	CG8946-PB	-	73.m00140	ENSGALP00000007235	-	OTTHUMP00000019769	LmjF30.2350	129959	14889	1152	159899	NCU06761	Os01g01080.3	8156	GSPATP00013947001	15730	131389	130206	-	413691	RO3G_12509.1	YDR294C	-	GLEAN3_20001	SINFRUP00000131149	23.m00203	29322	-	-	28705	-	Tb927.6.3630	UM05120.1
		-	-	-	-	-	-	-	-	-	-	-	-	175803	-	-	-	-	-	-	-	-	-	-	-	-	-	-	-	-	-	-	-	-	-	-	-	-	-	-	-	-	-	-	-	-	-	-	-	-	-	-
		-	-	-	-	-	-	-	-	-	-	-	-	175797	-	-	-	-	-	-	-	-	-	-	-	-	-	-	-	-	-	-	-	-	-	-	-	-	-	-	-	-	-	-	-	-	-	-	-	-	-	-
		-	-	-	-	-	-	-	-	-	-	-	-	175796	-	-	-	-	-	-	-	-	-	-	-	-	-	-	-	-	-	-	-	-	-	-	-	-	-	-	-	-	-	-	-	-	-	-	-	-	-	-
		-	-	-	-	-	-	-	-	-	-	-	-	175793	-	-	-	-	-	-	-	-	-	-	-	-	-	-	-	-	-	-	-	-	-	-	-	-	-	-	-	-	-	-	-	-	-	-	-	-	-	-
FAP185	Conserved Uncharacterized Flagellar Associated Protein	-	-	-	+	-	-	-	-	-	-	-	-	175790	-	-	-	-	-	-	-	-	-	-	-	-	-	-	-	-	-	-	-	-	-	-	-	-	-	-	-	-	-	-	-	-	-	-	-	-	-	-
		-	-	-	-	-	-	-	-	-	-	-	-	175787	-	-	-	-	-	-	-	-	-	-	-	-	-	-	-	-	-	-	-	-	-	-	-	-	-	-	-	-	-	-	-	-	-	-	-	-	-	-
		-	-	-	-	-	-	-	-	-	-	-	-	175772	-	-	-	-	-	-	-	-	-	-	-	-	-	-	-	-	-	-	-	-	-	-	-	-	-	-	-	-	-	-	-	-	-	-	-	-	-	-
		-	-	-	-	-	-	-	-	-	-	-	-	175768	-	-	-	-	-	-	-	-	-	-	-	-	-	-	-	-	-	Os03g50980.1	13166	-	-	-	-	-	-	-	-	SPAC1687.05	-	-	-	-	-	-	-	-	-	-
		-	-	-	-	-	-	-	-	-	-	-	-	175764	-	-	-	-	-	-	-	-	-	-	-	-	-	-	-	-	-	-	-	-	-	-	-	-	-	-	-	-	-	-	-	-	-	-	-	-	-	-
		-	-	-	-	-	-	-	-	-	-	-	-	175762	-	-	-	-	-	-	-	-	-	-	-	-	-	-	-	-	-	-	-	-	-	-	-	-	-	-	-	-	-	-	-	-	-	-	-	-	-	-
		-	-	-	-	-	-	-	-	65000	-	-	-	175761	-	-	-	-	-	-	-	-	-	-	-	-	-	-	-	-	-	-	-	-	-	-	-	-	-	-	-	-	-	-	-	-	-	-	-	-	-	-
		-	-	-	-	-	-	-	-	-	BDEG_02284	-	-	175752	-	-	-	-	-	-	-	-	-	-	-	-	-	-	-	-	-	-	-	-	-	-	-	-	-	-	-	-	-	SINFRUP00000142601	-	-	-	-	-	-	-	-
	Putative Esterase	-	+	-	-	-	-	GB13082-PA	AT2G41530.1	22416	BDEG_07548	CE25312	21698	175746	238167	-	CMQ397C	OTTDARP00000022436	DDB0185342	-	-	-	ENSGALP00000027399	-	OTTHUMP00000018374	-	120210	32406	62013	243067	NCU00173	Os01g71300.1	21650	-	-	126818	109641	-	652670	-	YJL068C	-	-	SINFRUP00000182774	-	-	-	-	20067	-	-	UM04430.1
		-	-	-	-	-	-	-	-	-	-	-	-	175730	-	-	-	-	-	-	-	-	-	-	-	-	-	-	-	-	-	-	-	-	-	-	-	-	-	-	-	-	-	-	-	-	-	-	-	-	-	-
		-	+	+	-	-	-	GB13463-PA	AT4G04880.1	-	BDEG_06143	CE39336	219075	175699	-	-	-	OTTDARP00000022085	DDB0187451	CG11994-PA	-	103.m00164	ENSGALP00000006420	-	OTTHUMP00000108990	LmjF10.0840	178823	26707	62042	215457	NCU00438	Os07g46630.1	33476	GSPATP00004773001	22117	195414	134850	PF10_0289	837657	RO3G_10095.1	-	-	GLEAN3_07696	SINFRUP00000132403	59.m00257	37724	-	20.m03932	61081	94652.m00118	Tb927.8.4270	UM00564.1
		-	-	-	-	-	-	-	-	-	-	-	-	175696	-	-	-	-	-	-	-	-	-	-	-	-	-	-	-	-	-	-	-	-	-	-	-	-	-	-	-	-	-	-	-	-	-	-	-	-	-	-
		-	-	-	-	-	-	-	-	-	-	-	-	175692	-	-	-	-	-	-	19171339	-	-	-	-	LmjF20.0250	-	-	-	-	-	-	-	-	-	-	-	-	-	-	-	-	-	-	-	-	-	-	-	-	Tb927.1.870	-
		-	-	-	-	-	-	-	-	-	-	-	-	175687	-	-	-	-	-	-	-	-	-	-	-	-	-	-	-	-	-	Os03g43040.1	-	-	-	-	-	-	-	-	-	-	-	-	-	-	-	-	-	-	-	-
		-	-	-	-	-	-	-	-	-	-	-	-	175682	-	-	-	-	-	-	-	-	-	-	-	-	-	-	-	-	-	-	-	-	-	-	-	-	-	-	-	-	-	-	-	-	-	-	-	-	-	-
		-	-	-	-	-	-	-	-	-	-	-	-	175680	-	-	-	-	-	-	-	-	-	-	-	-	-	-	-	-	-	-	-	-	-	-	-	-	-	-	-	-	-	-	-	-	-	-	-	-	-	-
C_400040		-	+	-	-	-	-	-	-	-	-	-	50237	175679	-	-	-	-	-	-	-	-	ENSGALP00000012442	-	-	-	-	-	-	62604	-	Os09g36800.1	-	-	20809	-	-	-	796681	RO3G_01377.1	-	-	-	-	81.m00222	-	-	542.m00002	-	-	-	-
		-	-	-	-	-	-	-	-	-	-	-	-	175676	-	-	-	-	-	-	-	-	-	-	-	-	-	-	-	-	-	-	-	-	-	-	-	-	-	-	-	-	-	-	-	-	-	-	-	-	-	-
		-	-	-	-	-	-	-	-	-	-	-	-	175672	-	-	-	-	-	-	-	-	-	-	-	-	-	-	-	-	-	-	-	-	-	-	-	-	-	-	-	-	-	-	-	-	-	-	-	-	-	-
		-	+	-	-	-	-	GB20152-PA	AT4G21150.1	-	-	CE12232	224049	175668	205164	-	-	-	DDB0219439	CG6370-PA	-	-	ENSGALP00000033827	-	OTTHUMP00000030900	-	220939	-	30174	245416	NCU11248	Os01g68324.2	-	-	-	6306	142830	-	830072	-	-	-	-	SINFRUP00000151515	-	-	-	-	64329	-	-	-
		-	-	-	-	-	-	-	-	-	-	-	-	175660	-	-	-	-	-	-	-	-	-	-	-	-	-	-	-	-	-	-	-	-	-	-	-	-	-	-	-	-	-	-	-	-	-	-	-	-	-	-
		-	-	-	-	-	-	-	-	-	-	-	-	175659	-	-	-	-	-	-	-	-	-	-	-	-	-	-	-	-	-	-	-	-	-	-	-	-	-	-	-	-	-	-	-	-	-	-	-	-	-	-
FAP176	Flagellar Associated Protein	-	-	-	-	-	-	-	-	-	-	-	-	175641	-	-	-	-	-	-	-	-	-	-	-	-	-	-	-	-	-	-	-	-	-	-	-	-	-	-	-	-	-	-	-	-	-	-	-	-	-	-
		-	-	-	-	-	-	-	-	-	-	-	-	175631	-	-	-	-	-	-	-	-	-	-	-	-	-	-	-	-	-	-	-	-	-	-	-	-	-	-	-	-	-	-	-	-	-	-	-	-	-	-
		-	+	-	-	-	-	GB17059-PA	AT1G31870.2	-	BDEG_01923	CE00289	-	175630	-	-	-	-	DDB0188062	-	-	-	ENSGALP00000011403	-	OTTHUMP00000069345	-	-	27011	47716	-	NCU04362	Os08g08080.1	12457	GSPATP00003748001	-	57686	-	-	264661	RO3G_04479.1	YGL174W	SPCC1620.10	-	SINFRUP00000128039	-	261566	TA15640	-	28663	-	-	UM00191.1
		-	-	-	-	-	-	-	-	-	-	-	-	175629	-	-	-	-	-	-	-	-	-	-	-	-	-	-	-	-	-	-	-	-	-	-	-	-	-	-	-	-	-	-	-	-	-	-	-	-	-	-
		-	-	-	-	-	-	-	-	-	-	-	-	175628	-	-	-	-	-	-	-	113.m00165	-	-	-	-	-	-	-	-	-	-	-	-	-	-	-	-	-	-	-	-	-	SINFRUP00000142858	-	-	-	-	-	-	-	-
		-	-	-	-	-	-	-	-	-	-	-	-	175627	-	-	-	-	-	-	-	-	-	-	-	-	-	-	-	-	-	-	-	-	-	-	-	-	-	-	-	-	-	-	-	-	-	-	-	-	-	-
		-	-	-	-	-	-	-	-	-	-	-	-	175626	-	-	-	-	-	-	-	-	-	-	-	-	-	-	-	-	-	-	-	-	-	-	-	-	-	-	-	-	-	-	-	-	-	-	-	-	-	-
		-	-	-	-	-	-	-	-	-	-	-	-	175624	-	-	-	-	-	-	-	-	-	-	-	-	-	-	-	-	-	-	-	-	-	-	-	-	-	-	-	-	-	-	-	-	-	-	-	-	-	-
		-	-	-	-	-	-	-	-	-	-	-	-	175623	-	-	-	-	-	-	-	-	-	-	-	-	-	-	-	-	-	-	-	-	-	-	-	-	-	-	-	-	-	-	-	-	-	-	-	-	-	-
		-	+	-	-	-	-	-	-	64372	BDEG_07323	-	-	175619	-	-	-	-	-	-	-	-	-	-	-	-	-	-	-	-	NCU06546	-	-	-	-	-	-	-	-	RO3G_03385.1	YDR393W	SPAC823.13c	-	-	-	-	-	-	-	-	-	UM04166.1
		-	-	-	-	-	-	-	-	-	-	-	-	175618	-	-	-	-	-	-	-	-	-	-	-	-	-	-	-	-	-	-	-	-	-	71167	-	-	218739	-	-	-	-	-	-	-	-	-	-	-	-	-
		-	-	-	-	-	-	-	-	65314	-	-	-	175615	-	-	CMB069C	-	-	-	-	-	-	-	-	-	-	-	-	-	-	-	-	-	-	-	-	-	-	-	-	-	-	-	-	20950	-	-	-	-	-	-
		-	-	-	-	-	-	-	-	-	-	-	-	175612	-	-	-	-	-	-	-	-	-	-	-	-	-	-	-	-	-	-	-	-	-	-	-	-	-	-	-	-	-	-	-	-	-	-	-	-	-	-
		-	-	-	-	-	-	-	-	-	-	-	-	175598	218399	-	-	-	-	-	-	-	-	-	-	-	-	-	-	-	-	-	-	-	-	-	-	-	-	-	-	-	-	-	-	-	-	-	-	-	-	-
		-	-	-	-	-	-	-	-	-	-	-	-	175595	-	-	-	-	-	-	-	-	-	-	-	-	-	-	-	-	-	-	-	-	-	-	-	-	-	-	-	-	-	-	-	-	-	-	-	-	-	-
		-	-	-	-	-	-	-	-	-	-	-	-	175589	-	-	-	-	-	-	-	-	-	-	-	-	-	-	-	-	-	-	-	-	-	-	-	-	-	-	-	-	-	-	-	-	-	-	-	-	-	-
		-	+	-	-	-	-	-	AT3G20540.1	27651	-	-	-	175586	-	-	CMO270C	-	DDB0189782	-	-	-	-	-	-	-	-	-	-	-	-	Os08g07840.3	24110	GSPATP00010188001	51407	227947	142429	-	232507	-	-	-	-	-	3.m01785	270013	-	-	-	-	-	-
POL1a	Poll-like DNA polymerase	-	-	-	-	-	-	-	-	-	-	-	-	175585	-	-	-	-	-	-	-	-	-	-	-	-	-	-	-	-	-	-	-	-	-	234378	-	-	-	-	-	-	-	-	-	-	-	-	-	-	-	-
		-	-	-	-	-	-	-	-	-	-	-	-	175584	-	-	-	-	-	-	-	-	-	-	-	-	-	-	-	-	-	-	-	-	-	-	-	-	-	-	-	-	-	-	-	-	-	-	-	-	-	-
		-	-	-	-	-	-	-	-	-	-	-	-	175576	-	-	-	-	-	-	-	-	-	-	-	-	-	-	-	-	-	-	-	-	-	-	-	-	-	-	-	-	-	-	-	-	-	-	-	-	-	-
		-	-	-	-	-	-	-	-	-	-	-	-	175575	-	-	-	-	-	-	-	-	-	-	-	-	-	-	-	-	-	-	-	-	-	-	-	-	-	-	-	-	-	-	-	-	-	-	-	-	-	-
		-	-	-	-	-	-	-	-	-	-	-	223573	175573	-	-	-	-	-	-	-	-	-	-	-	-	-	-	-	180912	-	-	-	-	-	-	-	-	-	-	-	-	-	-	-	-	-	-	-	-	-	-
		-	+	-	-	-	-	GB16539-PB	AT5G22120.1	-	-	-	47776	175567	-	-	-	-	-	CG2162-PA	-	-	ENSGALP00000038755	-	NP_055287.4	-	231658	-	-	-	-	-	-	-	-	-	-	-	-	-	-	-	-	SINFRUP00000143373	-	-	-	-	-	-	-	-
		-	-	-	-	-	-	-	-	-	-	-	-	175566	-	-	-	-	-	-	-	-	-	-	-	-	-	-	-	-	-	-	-	-	-	-	-	-	-	-	-	-	-	-	-	-	-	-	-	-	-	-
		-	-	-	-	-	-	-	-	-	-	-	-	175565	-	-	-	-	-	-	-	-	-	-	-	-	-	-	-	-	-	-	-	-	-	-	-	-	-	-	-	-	-	-	-	-	-	-	-	-	-	-
	putative DNA/RNA non-specific endonuclease	-	+	-	-	-	-	GB19949-PA	-	-	BDEG_07884	CE29488	120680	175564	254139	cgd5_2190	-	OTTDARP00000023169	DDB0187526	CG32463-PA	-	-	ENSGALP00000007374	-	OTTHUMP00000022301	LmjF10.0610	174581	2310	2950	228708	NCU00030	-	4798	-	29606	-	139372	-	-	RO3G_15601.1	YJL208C	SPAC17C9.08	GLEAN3_09130	SINFRUP00000146826	-	24666	-	25.m02938	23653	-	Tb927.8.4090	UM05674.1
		-	-	-	-	-	-	-	-	-	-	-	-	175557	-	-	-	-	-	-	-	-	-	-	-	-	-	-	-	-	-	-	-	-	-	-	-	-	-	-	-	-	-	-	-	-	-	-	-	-	-	-
		-	-	-	-	-	-	-	-	-	-	-	-	175549	-	-	-	-	-	-	-	-	-	-	-	-	-	-	-	-	-	-	-	-	-	-	-	-	-	-	-	-	-	-	-	-	-	-	-	-	-	-
		-	-	-	-	-	-	-	-	71577	-	-	-	175546	-	-	-	-	-	-	-	-	-	-	-	-	-	-	-	-	-	-	-	-	-	-	-	-	-	-	-	-	-	-	-	-	-	-	-	-	-	-
		-	-	-	-	-	-	-	-	-	-	-	-	175541	-	-	-	-	-	-	-	-	-	-	-	-	-	-	-	-	-	-	-	-	-	-	-	-	-	-	-	-	-	-	-	-	-	-	-	-	-	-
		-	-	-	-	-	-	-	-	-	-	-	-	175540	-	-	-	-	-	-	-	-	-	-	-	-	-	-	-	-	-	-	-	-	-	-	-	-	-	-	-	-	-	-	-	-	-	-	-	-	-	-
		-	-	-	-	-	-	-	-	-	-	-	-	175538	-	-	-	-	-	-	-	-	-	-	-	-	-	-	-	-	-	-	-	-	-	-	-	-	-	-	-	-	-	-	-	-	-	-	-	-	-	-
		-	-	-	-	-	-	-	-	-	-	-	-	175537	-	-	-	-	-	-	-	-	-	-	-	-	-	-	-	-	-	-	34704	-	-	-	-	-	-	-	-	-	-	-	-	-	-	-	-	-	-	-
CNX3	molybdenum cofactor synthesis-step 1 protein	-	+	-	-	-	-	GB16374-PA	AT1G01290.1	29248	-	-	163794	175536	-	-	CMN213C	-	-	-	-	-	ENSGALP00000016314	-	-	-	119234	-	-	-	-	Os04g38700.1	8221	-	10532	191704	109536	-	572402	-	-	-	-	SINFRUP00000181808	-	9118	-	49.m03245	-	-	-	-
		-	-	-	-	-	-	-	AT5G05310.3	-	-	-	-	175535	-	-	-	-	-	-	-	-	-	-	-	-	-	-	-	-	-	Os11g02159.1	-	-	-	59354	-	-	-	-	-	-	-	-	-	-	-	-	-	-	-	-
		-	-	-	-	-	-	-	-	-	-	-	-	175534	-	-	CMF025C	-	-	-	-	-	-	-	-	-	-	-	-	-	-	-	-	-	-	-	-	-	-	-	-	-	-	-	-	-	-	-	-	-	-	-
		-	-	-	-	-	-	-	AT3G10970.2	-	-	-	-	175530	-	-	-	-	-	-	-	-	-	-	-	-	-	-	-	-	-	Os05g07632.1	-	-	-	119315	-	-	248451	-	-	-	-	-	-	-	-	-	-	-	-	-
		-	-	-	-	-	-	-	-	-	-	-	-	175528	-	-	-	-	-	-	-	-	-	-	-	-	-	-	-	-	-	-	-	-	-	-	-	-	-	-	-	-	-	-	-	-	-	-	-	-	-	-
		-	-	-	-	-	-	-	-	-	-	-	-	175527	-	-	-	-	-	-	-	-	-	-	-	-	-	-	-	-	-	-	-	-	-	-	-	-	-	-	-	-	-	-	-	-	-	-	-	-	-	-
		-	-	-	-	-	-	-	-	-	-	-	-	175513	-	-	-	-	-	-	-	-	-	-	-	-	-	-	-	-	-	-	-	-	-	-	-	-	-	-	-	-	-	-	-	-	-	-	-	-	-	-
		-	-	-	-	-	-	-	-	-	-	-	-	175511	-	-	-	-	-	-	-	-	-	-	-	-	-	-	-	-	-	-	-	-	-	-	-	-	-	-	-	-	-	-	-	-	-	-	-	-	-	-
		-	-	-	-	-	-	-	-	-	-	-	-	175502	-	-	-	-	-	-	-	-	-	-	-	-	-	-	-	-	-	-	-	-	-	-	-	-	-	-	-	-	-	-	-	-	-	-	-	-	-	-
		-	-	-	-	-	-	-	-	-	-	-	-	175495	-	-	-	-	-	-	-	-	-	-	-	-	-	-	-	-	-	-	-	-	-	-	-	-	-	-	-	-	-	-	-	-	-	-	-	-	-	-
DNJ11	DnaJ-like protein 11, probably cytosolic	-	+	-	-	-	-	GB19680-PA	AT3G57340.2	54950	BDEG_07881	CE05169	228546	175485	-	cgd5_3430	CMT623C	OTTDARP00000021458	DDB0204663	-	-	-	ENSGALP00000022679	-	OTTHUMP00000161679	-	185246	33717	-	117533	NCU03335	Os01g37560.3	7945	GSPATP00027260001	-	173974	142015	PF11_0380	717666	RO3G_01859.1	-	SPBC17A3.05c	-	SINFRUP00000159470	156.m00086	32713	TA16620	55.m04854	55761	-	-	UM01207.1
		-	+	-	-	-	-	GB10936-PA	AT1G28060.1	20031	BDEG_01199	CE03498	19567	175482	270970	cgd5_1740	-	OTTDARP00000021566	DDB0167575	CG7757-PB	-	-	ENSGALP00000000633	-	OTTHUMP00000014935	-	186812	20505	59222	235197	NCU01637	Os09g07500.2	33549	GSPATP00010170001	34712	170971	139348	MAL13P1.45	227722	RO3G_03328.1	YDR473C	SPAC29E6.02	GLEAN3_19769	SINFRUP00000139891	83.m00131	9108	TA07965	38.m01074	37477	81444.m00050	-	UM03964.1
		-	-	-	-	-	-	-	-	-	-	-	-	175470	-	-	-	-	-	-	-	-	-	-	-	-	-	-	-	-	-	-	-	-	-	-	-	-	-	-	-	-	-	-	-	-	-	-	-	-	-	-
		-	+	-	-	-	-	-	-	2300	-	-	-	175469	-	-	-	-	-	-	-	-	-	GL50803_7874	-	LmjF25.1970	-	-	71374	-	-	-	20262	GSPATP00017497001	-	-	128334	-	-	-	-	-	-	-	12.m00549	-	-	-	-	-	Tb927.3.2040	-
		-	+	-	-	-	-	GB17371-PA	AT5G24350.1	-	-	-	162693	175468	227490	-	-	OTTDARP00000005840	DDB0204358	-	-	-	ENSGALP00000026506	-	OTTHUMP00000149964	-	212786	-	-	91657	-	Os12g13150.1	14285	-	-	172369	-	-	775658	-	-	-	-	SINFRUP00000130200	-	-	-	-	54605	-	-	-
		-	-	-	-	-	-	-	-	-	-	-	-	175467	-	-	-	-	-	-	-	-	-	-	-	-	-	-	-	-	-	-	-	-	-	-	-	-	-	-	-	-	-	-	-	-	-	-	-	-	-	-
		-	-	-	-	-	-	-	-	-	-	-	-	175451	-	-	-	-	-	-	-	-	-	-	-	-	-	-	-	-	-	-	-	-	-	-	-	-	-	-	-	-	-	-	-	-	-	-	-	-	-	-
		-	-	-	-	-	-	-	-	-	-	-	-	175440	-	-	-	-	-	-	-	-	-	-	-	-	-	-	-	-	-	-	-	-	-	-	-	-	-	-	-	-	-	-	-	-	-	-	-	-	-	-
		-	-	-	-	-	-	-	-	-	-	-	-	175435	-	-	-	-	-	-	-	-	-	-	-	-	-	-	-	-	-	-	-	-	-	-	-	-	-	-	-	-	-	-	-	-	-	-	-	-	-	-
		-	-	-	-	-	-	-	-	-	-	-	-	175429	-	-	CMI048C	-	-	-	-	-	-	-	-	-	-	-	-	-	-	-	-	-	-	-	-	-	-	-	-	-	-	-	-	-	-	-	-	-	-	-
		-	-	-	-	-	-	-	-	-	-	-	-	175426	-	-	-	-	-	-	-	-	-	-	-	-	-	-	-	-	-	-	-	-	-	-	-	-	-	-	-	-	-	-	-	-	-	-	-	-	-	-
		-	-	-	-	-	-	-	-	-	-	-	-	175423	-	-	-	-	-	-	-	-	-	-	-	-	-	-	-	-	-	-	-	-	-	-	-	-	-	-	-	-	-	-	-	-	-	-	-	-	-	-
SCC1b	Cohesin subunit, Rad21/Rec8 homolog	-	+	-	-	-	-	GB13290-PA	AT5G16270.1	64106	BDEG_02471	CE02628	161206	175413	266834	-	CML311C	OTTDARP00000015859	DDB0202625	CG17436-PA	-	3.m00568	ENSGALP00000025921	-	OTTHUMP00000178212	LmjF05.1090	133906	35988	47930	169110	NCU03291	Os01g67250.2	33474	-	44595	129589	131594	-	820452	RO3G_01121.1	YDL003W	SPCC338.17c	GLEAN3_02553	SINFRUP00000136090	-	263720	-	-	58420	90210.m00218	Tb927.7.6900	UM02591.1
		-	+	-	-	-	-	-	-	-	-	-	-	175411	-	-	-	-	DDB0190646	-	-	-	-	-	-	-	-	-	-	-	-	Os09g24660.1	30433	-	-	-	-	-	569973	-	-	-	-	-	-	11078	-	-	-	-	-	-
		-	-	-	-	-	-	-	-	-	-	-	-	175408	-	-	-	-	-	-	-	-	-	-	-	-	-	-	-	-	-	-	-	-	-	-	-	-	-	-	-	-	-	-	-	-	-	-	-	-	-	-
		-	-	-	-	-	-	-	-	-	-	-	-	175407	-	-	-	-	-	-	-	-	-	-	-	-	-	-	-	-	-	-	-	-	-	-	-	-	-	-	-	-	-	-	-	-	-	-	-	-	-	-
		-	-	-	-	-	-	-	-	-	-	-	-	175402	-	-	-	-	-	-	-	-	-	-	-	-	-	-	-	-	-	-	-	-	-	-	-	-	-	-	-	-	-	-	-	-	-	-	-	-	-	-
		-	-	-	-	-	-	-	-	-	-	-	-	175400	-	-	-	-	-	-	-	-	-	-	-	-	-	-	-	-	-	-	-	-	-	-	-	-	-	-	-	-	-	-	-	-	-	-	-	-	-	-
		-	-	-	-	-	-	-	-	-	-	-	-	175399	-	-	-	-	-	-	-	-	-	-	-	-	-	-	-	-	-	-	-	-	-	-	-	-	-	-	-	-	-	-	-	-	-	-	-	-	-	-
		-	-	-	-	-	-	-	-	-	-	-	-	175397	-	-	-	-	-	-	-	-	-	-	-	LmjF29.2800	-	-	-	-	-	-	-	-	-	-	-	-	-	-	-	-	-	-	-	-	-	-	-	-	Tb927.3.2960	-
		+	+	-	-	-	-	GB10915-PA	-	71976	-	-	-	175396	213306	-	-	-	-	-	-	-	-	-	-	-	-	-	80717	-	-	-	-	GSPATP00018386001	-	232007	133048	PFI1540w	-	-	-	-	-	SINFRUP00000180875	11.m00430	1057	-	-	-	-	Tb10.389.0670	-
		-	-	-	-	-	-	-	-	-	-	-	-	175390	-	-	-	-	-	-	-	-	-	-	-	-	-	-	-	-	-	-	-	-	-	-	-	-	-	-	-	-	-	-	-	-	-	-	-	-	-	-
LDE1		-	+	-	-	-	-	-	AT1G58080.1	-	-	-	-	175375	-	-	CMK245C	-	-	-	-	-	-	-	-	-	-	-	-	-	-	Os03g04169.1	9365	-	-	64865	-	-	249204	-	-	-	-	-	-	-	-	-	-	-	-	-
		-	-	-	-	-	-	-	-	-	-	-	-	175372	-	-	-	-	-	-	-	-	-	-	-	-	-	-	-	-	-	-	-	-	-	-	-	-	-	-	-	-	-	-	-	-	-	-	-	-	-	-
		-	-	-	-	-	-	-	-	-	-	-	-	175368	-	-	-	-	-	-	-	-	-	-	-	-	-	-	-	-	-	-	-	-	-	-	-	-	-	-	-	-	-	-	-	-	-	-	-	-	-	-
		-	-	-	-	-	-	-	-	-	-	-	-	175367	-	-	-	-	-	-	-	-	-	-	-	-	-	-	-	-	-	-	-	-	-	-	-	-	-	-	-	-	-	-	-	-	-	-	-	-	-	-
		-	+	-	-	-	-	GB14074-PA	AT2G26470.1	-	BDEG_06251	-	219289	175364	253362	-	-	-	-	CG11986-PA	-	-	ENSGALP00000035471	-	OTTHUMP00000172699	-	211838	9856	67685	98333	NCU03985	Os06g27650.1	35180	GSPATP00000863001	44809	166537	-	-	553242	-	YMR114C	-	-	SINFRUP00000138707	-	11193	-	50.m00087	24378	83996.m00273	-	UM01074.1
		-	-	-	+	-	-	-	-	-	-	-	-	175363	-	-	-	-	-	-	-	-	-	-	-	-	-	-	-	-	-	-	-	-	-	-	-	-	-	-	-	-	-	-	-	-	-	-	-	-	-	-
		-	+	-	-	-	-	-	-	-	-	-	-	175359	270910	-	-	-	DDB0217029	-	19168638	-	-	-	-	-	-	-	-	246030	-	-	-	-	-	-	133986	-	-	-	-	-	GLEAN3_02618	SINFRUP00000154831	-	-	-	-	18236	-	-	-
		-	-	-	-	-	-	-	-	-	-	-	-	175350	-	-	-	-	-	-	-	-	-	-	-	-	-	-	-	-	-	-	-	-	-	-	-	-	-	-	-	-	-	-	-	-	-	-	-	-	-	-
		-	-	-	-	-	-	-	-	-	-	-	-	175340	-	-	-	-	-	-	-	-	-	-	-	-	-	-	-	-	-	-	-	-	-	-	-	-	-	-	-	-	-	-	-	-	-	-	-	-	-	-
		-	+	-	+	-	-	-	AT3G16730.1	61860	-	CE30620	219248	175339	295893	cgd3_950	CMI207C	OTTDARP00000007986	DDB0184121	-	-	-	ENSGALP00000039615	-	OTTHUMP00000196604	-	233379	26851	-	232486	-	Os07g33360.1	-	-	50280	-	131122	-	596112	-	-	-	-	SINFRUP00000181500	-	-	-	-	52130	87150.m00179	-	-
		-	-	-	-	-	-	-	AT2G40935.1	-	-	-	-	175336	-	-	-	-	-	-	-	-	-	-	-	-	-	-	-	-	-	Os01g16210.1	-	-	-	-	-	-	716965	-	-	-	-	-	-	-	-	-	-	82174.m00120	-	-
		-	-	-	-	-	-	-	-	-	-	-	-	175330	-	-	-	-	-	-	-	-	-	-	-	-	-	-	-	-	-	-	-	-	-	-	-	-	-	-	-	-	-	-	-	-	-	-	-	-	-	-
		-	-	-	-	-	-	-	-	-	-	-	-	175323	-	-	-	-	-	-	-	-	-	-	-	-	-	-	-	-	-	-	-	-	-	-	-	-	-	-	-	-	-	-	-	-	-	-	-	-	-	-
		-	-	-	-	-	-	-	-	-	-	-	-	175321	-	-	-	-	-	-	-	-	-	-	-	-	-	-	-	-	-	-	-	-	-	-	-	-	-	-	-	-	-	-	-	-	-	-	-	-	-	-
		-	-	-	-	-	-	-	-	-	-	-	-	175320	-	-	-	-	-	-	-	-	-	-	-	-	-	-	-	-	-	-	-	-	-	-	-	-	-	-	-	-	-	-	-	-	-	-	-	-	-	-
		-	-	-	-	-	-	-	-	-	-	-	-	175308	-	-	-	-	-	-	-	-	-	-	-	-	-	-	-	-	-	-	36871	-	39961	169092	-	-	-	-	-	-	-	-	-	3113	-	-	-	-	-	-
		-	+	-	-	-	-	-	AT5G21010.1	-	-	-	-	175298	-	-	-	-	-	-	-	-	-	-	-	-	-	-	-	-	-	Os07g46160.1	3719	-	-	217835	-	-	227343	-	-	-	-	-	-	-	-	-	-	-	-	-
		-	-	-	-	-	-	-	-	-	-	-	-	175296	-	-	-	-	DDB0218342	-	-	-	-	-	-	-	-	-	-	-	-	-	-	-	-	-	-	-	-	-	-	-	-	-	-	-	-	-	-	-	-	-
		-	-	-	-	-	-	-	AT3G14075.1	-	-	-	-	175290	-	-	-	-	-	-	-	-	-	-	-	-	-	-	-	-	-	Os02g54010.1	-	-	-	191863	-	-	817045	-	-	-	-	-	-	-	-	-	-	-	-	-
CLPS4	ClpS-like protein	-	-	-	-	-	-	-	-	-	-	-	-	175283	-	-	-	-	-	-	-	-	-	-	-	-	-	-	-	-	-	-	-	-	-	-	-	-	-	-	-	-	-	-	-	-	-	-	-	-	-	-
		-	-	-	-	-	-	-	-	-	-	-	-	175275	-	-	-	-	-	-	-	-	-	-	-	-	-	-	-	-	-	-	-	-	-	-	-	-	-	-	-	-	-	-	-	-	-	-	-	-	-	-
		-	-	-	-	-	-	-	-	-	-	-	-	175267	-	-	-	-	-	-	-	-	-	-	-	-	-	-	-	-	-	-	-	-	-	-	-	-	-	-	-	-	-	-	-	-	-	-	-	-	-	-
		-	-	-	-	-	-	-	-	-	-	-	-	175264	-	-	-	-	-	-	-	-	-	-	-	-	-	-	-	-	-	-	-	-	-	-	-	-	-	-	-	-	-	-	-	-	-	-	-	-	-	-
		-	+	-	-	-	-	GB13309-PA	AT4G26750.1	-	BDEG_03039	CE32500	178049	175262	-	-	CMI268C	OTTDARP00000005897	DDB0215061	CG7967-PA	-	-	ENSGALP00000022375	-	OTTHUMP00000017318	LmjF05.0740	137913	28340	78566	94572	-	Os06g20390.1	36894	-	47496	58799	157070	-	234542	RO3G_00346.1	-	-	-	SINFRUP00000150116	-	24658	-	-	31759	-	Tb927.7.7140	UM01645.1
		-	-	-	-	-	-	-	-	-	-	-	-	175258	-	-	-	-	-	-	-	-	-	-	-	-	-	-	-	-	-	-	-	-	-	-	-	-	-	-	-	-	-	-	-	-	-	-	-	-	-	-
		-	-	-	-	-	-	-	-	-	-	-	-	175256	-	-	-	-	-	-	-	-	-	-	-	-	-	-	-	-	-	-	-	-	-	-	-	-	-	-	-	-	-	-	-	-	-	-	-	-	-	-
		-	-	-	-	-	-	-	-	-	-	-	-	175242	-	-	-	-	-	-	-	-	-	-	-	-	-	-	-	-	-	-	-	-	-	-	-	-	-	-	-	-	-	-	-	-	-	-	-	-	-	-
		-	+	-	-	-	-	-	AT5G65490.1	-	BDEG_00765	-	20272	175239	-	-	-	-	-	-	-	-	ENSGALP00000004223	-	OTTHUMP00000019795	-	145160	-	-	-	-	Os08g34740.1	-	-	-	-	-	-	836197	RO3G_16837.1	-	-	-	-	-	-	-	-	21872	-	-	-
		-	+	-	-	-	-	GB30583-PA	-	-	BDEG_07400	CE02145	99256	175235	251088	cgd5_100	CMF177C	-	DDB0203581	-	-	-	ENSGALP00000004870	-	OTTHUMP00000002177	LmjF34.3080	-	33488	80761	129645	NCU02256	-	20576	GSPATP00031563001	22703	-	142047	-	409246	RO3G_16964.1	-	SPAC1F3.01	GLEAN3_27408	SINFRUP00000133183	243.m00042	17935	TA04265	-	27020	-	Tb927.4.1630	UM06132.1
		-	-	-	-	-	-	-	-	-	-	-	-	175232	-	-	-	-	-	-	-	-	-	-	-	-	-	-	-	225649	-	-	-	-	-	-	-	-	-	-	-	-	-	-	-	-	-	-	-	-	-	-
		-	-	-	-	-	-	-	-	-	-	-	-	175230	-	-	-	-	-	-	-	-	-	-	-	-	-	-	-	-	-	-	-	-	-	-	-	-	-	-	-	-	-	-	-	-	-	-	-	-	-	-
		-	-	-	-	-	-	-	-	-	-	-	-	175227	-	-	-	-	-	-	19068872	-	-	-	-	-	-	-	-	-	-	-	-	-	-	-	-	-	-	-	-	-	-	-	-	-	-	-	-	-	-	-
		-	-	-	-	-	-	-	-	-	-	-	-	175225	-	-	-	-	-	-	-	-	-	-	-	-	-	-	-	-	-	-	-	-	-	-	-	-	-	-	-	-	-	-	-	-	-	-	-	-	-	-
		-	-	-	-	-	-	-	-	-	-	-	-	175224	-	-	-	-	-	-	-	-	-	-	-	-	-	-	-	33052	-	-	-	-	-	-	-	-	-	-	-	-	-	-	-	-	-	-	-	-	-	-
		-	+	-	-	-	-	-	-	-	-	-	112116	175223	274049	-	-	-	-	-	-	-	-	-	-	-	133518	-	-	-	-	-	35054	-	44026	-	-	-	-	-	-	-	-	SINFRUP00000146623	-	20825	-	-	-	-	-	-
		-	-	-	-	-	-	-	-	-	-	-	-	175221	-	-	-	-	-	-	-	-	-	-	-	-	-	-	-	-	-	-	-	-	-	-	-	-	-	-	-	-	-	-	-	-	-	-	-	-	-	-
		-	-	-	-	-	-	-	-	-	-	-	-	175209	-	-	-	-	-	-	-	-	-	-	-	-	-	-	-	-	-	-	-	-	-	-	-	-	-	-	-	-	-	-	-	-	-	-	-	-	-	-
		-	-	-	-	-	-	-	-	-	-	-	-	175207	-	-	-	-	-	-	-	-	-	-	-	-	-	-	-	-	-	-	-	-	-	-	-	-	-	-	-	-	-	-	-	-	-	-	-	-	-	-
		-	-	-	-	-	-	-	-	-	-	-	-	175185	-	-	-	-	-	-	-	-	-	-	-	-	-	-	-	-	-	-	-	-	-	-	-	-	-	-	-	-	-	-	-	-	-	-	-	-	-	-
		-	-	-	-	-	-	-	-	-	-	-	-	175174	-	-	-	-	-	-	-	-	-	-	-	-	-	-	-	-	-	-	-	-	-	120760	-	-	817441	-	-	-	-	-	-	-	-	-	-	-	-	-
		-	-	-	-	-	-	-	-	-	-	-	-	175170	-	-	-	-	-	-	-	-	-	-	-	-	-	-	-	-	-	-	-	-	-	-	-	-	-	-	-	-	-	-	-	-	-	-	-	-	-	-
		-	-	-	-	-	-	-	-	-	-	-	-	175169	-	-	-	-	-	-	-	-	-	-	-	-	-	-	-	-	-	-	-	-	-	-	-	-	-	-	-	-	-	-	-	-	-	-	-	-	-	-
		-	-	-	-	-	-	-	-	-	-	-	-	175168	-	-	-	-	-	-	-	-	-	-	-	-	-	-	-	-	-	-	-	-	-	-	-	-	-	-	-	-	-	-	-	-	-	-	-	-	-	-
		-	-	-	-	-	-	-	-	-	-	-	-	175167	-	-	-	-	-	-	-	-	-	-	-	-	-	-	-	-	-	-	-	-	-	-	-	-	-	-	-	-	-	-	-	-	-	-	-	-	-	-
		-	-	-	-	-	-	-	-	-	-	-	-	175159	-	-	-	-	-	-	-	-	-	-	-	-	-	-	-	-	-	-	-	-	-	-	-	-	-	-	-	-	-	-	-	-	-	-	-	-	-	-
		-	-	-	-	-	-	-	-	-	-	-	-	175158	218408	-	-	-	-	-	-	-	-	-	-	-	232041	-	-	243101	-	-	-	-	-	-	-	-	-	-	-	-	-	-	-	-	-	-	-	-	-	-
		-	-	-	-	-	-	-	-	-	-	-	-	175147	-	-	-	-	-	-	-	-	-	-	-	-	-	-	-	-	-	-	-	-	-	-	-	-	-	-	-	-	-	-	-	-	-	-	-	-	-	-

TOG1		-	+	-	-	-	-	GB10660-PA	AT2G35630.1	-	BDEG_04589	CE20707	154948	175143	-	-	CMO196C	-	DDB0168274	-	-	-	ENSGALP00000013536	-	OTTHUMP00000081794	LmjF30.1760	228176	32952	49612	183698	NCU04535	Os01g60040.2	20299	GSPATP00034778001	47936	106216	156611	-	727336	RO3G_06225.1	YLR045C	SPCC895.07	GLEAN3_15952	SINFRUP00000155410	60.m00119	7217	-	-	51833	88808.m00117	Tb927.6.3090	UM06328.1
		-	-	-	-	-	-	-	-	-	-	-	-	175128	-	-	-	-	-	-	-	-	-	-	-	-	-	-	-	-	-	-	-	-	-	-	-	-	-	-	-	-	-	-	-	-	-	-	-	-	-	-
		-	-	-	-	-	-	-	-	-	-	-	-	175127	-	-	-	-	-	-	-	-	-	-	-	-	-	-	-	-	-	-	-	-	-	-	-	-	-	-	-	-	-	-	-	-	-	-	-	-	-	-
		-	+	-	-	-	-	-	-	64579	-	-	225493	175110	210046	-	-	-	-	-	-	-	-	-	-	-	219658	33883	56751	216488	-	-	-	-	-	-	138607	-	-	RO3G_09629.1	-	SPAC1039.06	-	SINFRUP00000182375	-	-	-	-	56315	-	-	-
		-	-	-	-	-	-	-	-	-	-	-	-	175109	-	-	-	-	-	-	-	-	-	-	-	-	-	-	-	-	-	-	-	-	-	-	-	-	-	-	-	-	-	-	-	-	-	-	-	-	-	-
		-	-	-	-	-	-	-	-	-	-	-	-	175108	-	-	-	-	-	-	-	-	-	-	-	-	-	-	-	-	-	-	-	-	-	-	-	-	-	-	-	-	-	-	-	-	-	-	-	-	-	-
		-	+	-	-	-	-	-	AT4G29520.1	-	-	-	-	175106	-	-	-	-	-	-	-	-	-	-	-	LmjF36.5650	-	-	-	108064	-	Os06g05740.1	5099	-	-	115335	-	-	582950	-	-	-	-	-	-	-	-	-	-	-	Tb10.6k15.1270	-
		-	-	-	-	-	-	-	-	-	-	-	-	175100	-	-	-	-	-	-	-	-	-	-	-	-	-	-	-	-	NCU03011	-	-	-	-	-	-	-	-	-	-	-	-	-	-	261324	-	-	-	-	-	-
		-	-	-	-	-	-	-	-	-	-	-	-	-	-	-	-	-	-	-	-	-	-	-	-	-	-	-	-	-	-	-	-	-	-	-	-	-	-	-	-	-	-	-	-	-	-	-	-	-	-	-
		-	-	-	-	-	-	-	-	-	-	-	-	175097	-	-	-	-	-	-	-	-	-	-	-	-	-	-	-	-	-	-	-	-	-	-	-	-	-	-	-	-	-	-	-	-	-	-	-	-	-	-
		-	-	-	-	-	-	-	-	-	-	-	-	175095	-	-	-	-	-	-	-	-	-	-	-	-	-	6960	-	-	-	-	-	-	-	-	-	-	-	-	-	-	-	-	-	7102	-	-	-	-	-	-
		-	-	-	-	-	-	-	-	-	-	-	-	175090	-	-	-	-	-	-	-	-	-	-	-	-	-	-	-	-	-	-	-	-	-	-	-	-	-	-	-	-	-	-	-	-	-	-	-	-	-	-
		-	-	-	-	-	-	-	-	-	-	-	-	175082	-	-	-	-	-	-	-	-	-	-	-	-	-	30755	-	-	-	-	-	-	-	-	-	-	-	-	-	-	-	-	-	-	-	-	-	-	-	-
		-	-	-	-	-	-	-	-	-	-	-	-	175081	-	-	-	-	-	-	-	-	-	-	-	-	-	-	-	-	-	-	-	-	-	-	-	-	-	-	-	-	-	-	-	-	-	-	-	-	-	-
		-	-	-	-	-	-	-	-	-	-	-	-	175079	-	-	-	-	-	-	-	-	-	-	-	-	-	-	-	-	-	-	-	-	-	-	-	-	-	-	-	-	-	-	-	-	-	-	-	-	-	-
		-	-	-	-	-	-	-	-	-	-	-	-	175066	-	-	-	-	-	-	-	-	-	-	-	-	-	-	-	-	-	-	-	-	-	-	-	-	-	-	-	-	-	-	-	-	-	-	-	-	-	-
		-	-	-	-	-	-	-	-	-	-	-	-	175060	-	-	-	-	-	-	-	-	-	-	-	-	-	-	-	-	-	-	-	-	-	-	-	-	-	-	-	-	-	-	-	-	-	-	-	-	-	-
		-	-	-	-	-	-	-	-	-	-	-	-	175053	-	-	-	-	-	-	-	-	-	-	-	-	-	-	-	-	-	-	-	-	-	-	-	-	-	-	-	-	-	-	-	-	-	-	-	-	-	-
		-	-	-	-	-	-	-	-	-	-	-	-	175050	-	-	-	-	-	-	-	-	-	-	-	-	-	-	-	-	-	-	-	-	-	-	-	-	-	-	-	-	-	-	-	-	-	-	-	-	-	-
		-	+	-	-	-	-	-	AT1G09830.1	64345	-	-	90257	175039	270291	-	CMJ084C	-	DDB0188738	CG31628-PB	-	-	ENSGALP00000025711	-	OTTHUMP00000068337	-	207583	-	-	114087	NCU00177	Os08g09210.2	35838	-	47067	141208	129332	-	297189	RO3G_16313.1	YGL234W	SPBC405.01	GLEAN3_23531	SINFRUP00000132027	-	-	-	-	61620	-	-	UM00812.1
		-	+	-	-	-	-	-	AT3G52220.1	-	-	-	-	175037	-	-	-	-	-	-	-	-	-	-	OTTHUMP00000035918	-	-	4886	-	196760	NCU03810	Os08g44440.1	33068	-	-	126526	128257	-	725409	-	-	-	-	SINFRUP00000156900	-	-	-	-	54046	-	-	UM00100.1
		-	+	-	-	-	-	-	-	-	-	-	227957	175036	294753	-	CMP269C	-	-	-	-	-	-	-	-	-	232463	23736	-	222046	-	-	-	-	50441	159943	140323	-	-	-	-	-	GLEAN3_00758	-	25.m00326	21546	-	-	51538	-	-	-
		-	-	-	-	-	-	-	-	-	-	-	-	175035	-	-	-	-	-	-	-	-	-	-	-	-	-	-	-	-	-	-	-	-	-	-	-	-	-	-	-	-	-	-	-	-	-	-	-	-	-	-
		-	-	-	-	-	-	-	-	-	-	-	-	175034	-	-	-	-	-	-	-	-	-	-	-	-	-	-	-	-	-	-	-	-	-	-	-	-	-	-	-	-	-	-	-	-	-	-	-	-	-	-
		-	-	-	-	-	-	-	-	-	-	-	-	175028	-	-	-	-	-	-	-	-	-	-	-	-	-	-	-	-	-	-	-	-	-	-	-	-	-	-	-	-	-	-	-	-	-	-	-	-	-	-
		-	-	-	-	-	-	-	-	-	-	-	-	175027	-	-	-	-	-	-	-	-	-	-	-	-	-	-	-	-	-	-	-	-	-	-	-	-	-	-	-	-	-	-	-	-	-	-	-	-	-	-
		-	-	-	-	-	-	-	-	-	-	-	-	175024	-	-	-	-	-	-	-	-	-	-	-	-	-	-	-	-	-	-	-	-	-	-	-	-	-	-	-	-	-	-	-	-	-	-	-	-	-	-
		-	-	-	-	-	-	-	-	-	-	-	-	175020	-	-	-	-	-	-	-	-	-	-	-	-	-	-	-	-	-	-	-	-	-	-	-	-	-	-	-	-	-	-	-	-	-	-	-	-	-	-
		-	-	-	-	-	-	-	-	-	-	-	-	175016	-	-	-	-	-	-	-	-	-	-	-	-	-	-	-	-	-	-	-	-	-	-	-	-	-	-	-	-	-	-	-	-	-	-	-	-	-	-
		-	-	-	-	-	-	-	-	-	-	-	-	175014	-	-	-	-	-	-	-	-	-	-	-	-	-	-	-	-	-	-	-	-	-	-	-	-	-	-	-	-	-	-	-	-	-	-	-	-	-	-
		-	-	-	-	-	-	-	-	-	-	-	-	175013	-	-	-	-	-	-	-	-	-	-	-	-	-	-	-	-	-	-	-	-	-	-	-	-	-	-	-	-	-	-	-	-	-	-	-	-	-	-
		-	-	-	-	-	-	-	-	-	-	-	-	175011	-	-	-	-	-	-	-	-	-	-	-	-	-	-	-	-	-	-	-	-	-	-	-	-	-	-	-	-	-	-	-	-	-	-	-	-	-	-
		-	-	-	-	-	-	-	-	-	-	-	-	175006	-	-	-	-	-	-	-	-	-	-	-	-	-	-	-	-	-	-	-	-	-	-	-	-	-	-	-	-	-	-	-	-	-	-	-	-	-	-
		-	+	-	-	-	-	-	AT3G04490.1	-	-	-	151878	175002	245824	-	-	OTTDARP00000021040	-	-	-	-	ENSGALP00000027623	-	OTTHUMP00000018105	-	106479	-	-	40746	-	Os10g33710.1	36949	-	-	148515	-	-	248156	RO3G_12299.1	-	-	-	SINFRUP00000137142	-	-	-	-	-	-	-	-
		-	-	-	-	-	-	-	-	-	-	-	-	175001	-	-	-	-	-	-	-	-	-	-	-	-	-	-	-	-	-	-	-	-	-	-	-	-	-	-	-	-	-	-	-	-	-	-	-	-	-	-
		-	-	-	-	-	-	-	-	-	-	-	-	174986	-	-	-	-	-	-	-	-	-	-	-	-	-	-	-	-	-	-	-	-	-	-	-	-	-	-	-	-	-	-	-	-	-	-	-	-	-	-
		-	-	-	-	-	-	-	-	-	-	-	-	174977	-	-	-	-	-	-	-	-	-	-	-	-	-	-	-	-	-	-	-	-	-	-	-	-	-	-	-	-	-	-	-	-	-	-	-	-	-	-
		-	-	-	-	-	-	-	-	-	-	-	-	-	-	-	-	-	-	-	-	-	-	-	-	-	-	-	-	-	-	-	-	-	-	-	-	-	-	-	-	-	-	-	-	-	-	-	-	-	-	-
		-	+	-	-	-	-	-	AT5G08540.1	-	-	-	-	174967	-	-	-	-	-	-	-	-	-	-	-	-	-	-	-	-	-	Os04g44560.1	36688	-	-	45363	-	-	567751	-	-	-	-	-	-	-	-	-	-	-	-	-
		-	-	-	-	-	-	-	-	-	-	-	-	174965	-	-	-	-	-	-	-	-	-	-	-	-	-	-	-	-	-	-	-	-	-	-	-	-	-	-	-	-	-	-	-	-	-	-	-	-	-	-
		-	-	-	-	-	-	-	-	-	-	-	-	174963	-	-	-	-	-	-	-	-	-	-	-	-	-	-	-	-	-	Os07g47100.3	-	-	-	193885	-	-	570935	-	-	-	-	-	-	-	-	-	-	-	-	-
		-	-	-	-	-	-	-	-	-	-	-	-	174960	-	-	-	-	-	-	-	-	-	-	-	-	-	-	-	-	-	-	-	-	-	-	-	-	-	-	-	-	-	-	-	-	-	-	-	-	-	-
		-	-	-	-	-	-	-	-	-	-	-	-	174942	-	-	-	-	-	-	-	-	-	-	-	-	-	-	-	-	-	-	-	-	-	-	-	-	-	-	-	-	-	-	-	-	-	-	-	-	-	-
		-	-	-	-	-	-	-	-	-	-	-	-	174935	-	-	-	-	-	-	-	-	-	-	-	-	-	-	-	-	-	-	-	-	-	-	-	-	-	-	-	-	-	-	-	-	-	-	-	-	-	-
		-	+	-	-	-	-	-	AT4G26965.1	-	-	-	-	174933	-	-	-	OTTDARP00000023230	-	-	-	-	ENSGALP00000023711	-	OTTHUMP00000161882	-	228046	-	78931	-	-	-	-	GSPATP00018866001	-																	
		-	-	-	-	-	-	-	-	-	-	-	-	174932	-	-	-	-	-	-	-	-	-	-	-	-	-	-	-	-	-	-	-	-	-	-	-	-	-	-	-	-	-	-	-	-	-	-	-	-	-	-
		-	-	-	-	-	-	-	-	-	-	-	-	174929	-	-	-	-	-	-	-	-	-	-	-	-	-	-	-	-	-	-	-	-	-	-	-	-	-	-	-	-	-	-	-	-	-	-	-	-	-	-
		-	-	-	-	-	-	-	-	-	-	-	-	174928	-	-	-	-	-	-	-	-	-	-	-	-	-	-	-	-	-	-	-	-	-	-	-	-	-	-	-	-	-	-	-	-	-	-	-	-	-	-
		-	-	-	-	-	-	-	-	-	-	-	-	174923	-	-	-	-	-	-	-	-	-	-	-	-	-	-	-	-	-	-	-	-	-	-	-	-	-	-	-	-	-	-	-	-	-	-	-	-	-	-
NOS1		-	+	-	-	-	-	GB19759-PA	AT3G47450.2	-	-	-	-	174922	-	-	-	-	DDB0204555	CG10914-PA	-	-	ENSGALP00000031537	-	-	-	-	-	-	-	-	Os02g01440.1	27995	-	-	21462	156294	-	423804	-	-	-	-	SINFRUP00000148781	-	-	-	55.m04852	-	-	-	-
		-	-	-	-	-	-	-	-	-	-	-	-	174920	-	-	-	-	-	-	-	-	-	-	-	-	-	-	-	-	-	-	-	-	-	-	-	-	-	-	-	-	-	-	-	-	-	-	-	-	-	-
		-	-	-	-	-	-	-	-	-	-	-	-	174918	-	-	-	-	-	-	-	-	-	-	-	-	-	-	-	-	-	-	-	-	-	-	-	-	-	-	-	-	-	-	-	-	-	-	-	-	-	-
		-	+	-	-	-	-	-	AT2G45700.1	-	-	-	-	174912	-	-	-	-	-	-	-	-	-	-	-	-	-	-	-	-	-	Os09g26760.1	20225	-	-	135525	-	-	802288	-	-	-	-	-	-	-	-	-	-	-	-	-
		-	-	-	-	-	-	-	-	-	-	-	-	174910	-	-	-	-	-	-	-	-	-	-	-	-	-	-	-	-	-	-	-	-	1473	-	-	-	-	-	-	-	-	-	-	-	-	-	-	-	-	-
		-	-	-	-	-	-	-	-	-	-	-	-	174906	-	-	-	-	-	-	-	-	-	-	-	-	-	-	-	-	-	-	-	-	-	-	-	-	-	-	-	-	-	-	-	-	-	-	-	-	-	-
		-	-	-	-	-	-	-	-	-	-	-	-	174903	-	-	-	-	-	-	-	-	-	-	-	-	-	-	-	-	-	-	-	-	-	-	-	-	-	-	-	-	-	-	-	-	-	-	-	-	-	-
		-	+	-	-	-	-	-	AT1G54870.1	-	-	-	-	174902	-	-	-	-	DDB0168766	-	-	-	-	-	-	-	-	-	52733	-	NCU09821	Os05g04870.1	-	-	-	-	-	-	729630	RO3G_07820.1	-	-	-	-	-	-	-	-	-	-	-	UM05507.1
		-	-	-	-	-	-	-	-	-	-	-	-	174898	-	-	-	-	-	-	-	-	-	-	-	-	-	-	-	-	-	-	-	-	-	-	-	-	-	-	-	-	-	-	-	-	-	-	-	-	-	-
		-	-	-	-	-	-	-	-	-	-	-	-	174893	-	-	-	-	-	-	-	-	-	-	-	-	-	-	-	-	-	-	-	-	-	-	-	-	-	-	-	-	-	-	-	-	-	-	-	-	-	-
		-	-	-	-	-	-	-	-	-	-	-	-	174892	-	-	-	-	-	-	-	-	-	-	-	-	-	-	-	-	-	-	-	-	-	-	-	-	-	-	-	-	-	-	-	-	-	-	-	-	-	-
		-	-	-	-	-	-	-	-	-	-	-	-	174891	-	-	-	-	-	-	-	-	-	-	-	-	-	-	-	-	-	-	-	-	-	-	-	-	-	-	-	-	-	-	-	-	-	-	-	-	-	-
		-	+	-	-	-	-	-	AT4G32760.1	38976	BDEG_02389	CE27055	174498	174889	392799	-	-	-	-	CG3529-PB	-	-	ENSGALP00000007825	-	OTTHUMP00000065655	-	93474	13859	-	100273	NCU04015	Os06g22650.1	-	GSPATP00017453001	-	196153	128941	-	260731	RO3G_15686.1	YDR358W	-	GLEAN3_07405	SINFRUP00000154348	-	-	-	-	20773	-	-	UM04619.1
		-	-	-	-	-	-	-	-	-	-	-	-	174888	-	-	-	-	-	-	-	-	-	-	-	-	-	-	-	-	-	-	-	-	-	-	-	-	-	-	-	-	-	-	-	-	-	-	-	-	-	-
		-	-	-	-	-	-	-	-	-	-	-	-	174886	-	-	-	-	-	-	-	-	-	-	-	-	-	-	-	-	-	-	-	-	-	-	-	-	-	-	-	-	-	-	-	-	-	-	-	-	-	-
		-	-	-	-	-	-	-	-	-	-	-	-	174884	-	-	-	-	-	-	-	-	-	-	-	-	-	-	-	-	-	-	-	-	-	-	-	-	-	-	-	-	-	-	-	-	-	-	-	-	-	-
	possible 2Fe-2S ferredoxin	-	+	-	-	-	-	-	AT4G32590.1	68800	-	-	-	174881	-	-	CME070C	-	-	-	-	-	-	-	-	-	-	-	-	-	-	Os05g48160.3	7730	-	47958	161774	-	-	654077	-	-	-	-	-	-	-	-	-	-	-	-	-
		-	+	-	-	-	-	-	AT1G26300.2	-	-	-	-	174875	-	-	-	-	DDB0190284	-	-	-	-	-	-	-	-	-	-	-	-	Os02g49450.1	-	-	-	184063	-	-	564448	-	-	-	-	-	-	-	-	-	-	-	-	-
		-	-	-	-	-	-	-	-	-	-	-	-	174861	-	-	-	-	-	-	-	-	-	-	-	-	-	-	-	-	-	-	-	-	-	-	-	-	-	-	-	-	-	-	-	-	-	-	-	-	-	-
		-	+	+	-	-	-	GB14060-PA	AT4G29380.1	-	BDEG_01275	CE36103	159025	174860	385050	cgd4_3410	-	OTTDARP00000010252	DDB0204876	CG9746-PA	-	120.m00107	ENSGALP00000036848	GL50803_113456	OTTHUMP00000172774	LmjF29.1370	132962	16599	77948	209974	NCU06626	Os02g55340.1	31853	-	42828	144257	144363	PF13_0315	653324	-	YBR097W	SPBC119.07	GLEAN3_15850	SINFRUP00000132685	-	261541	TA20580	59.m03367	57612	90455.m00097	Tb11.01.0930	UM01033.1
		-	-	-	-	-	-	-	-	-	-	-	-	174857	-	-	-	-	-	-	-	-	-	-	-	-	-	-	-	-	-	-	-	-	-	140595	-	-	-	-	-	-	-	-	-	-	-	-	-	-	-	-
		-	+	-	-	-	-	-	AT1G69210.1	-	-	-	-	174855	-	-	-	-	-	-	-	-	-	-	-	-	-	-	-	-	-	Os04g35760.1	13086	-	-	166194	-	-	769921	-	-	-	-	-	-	-	-	-	-	-	-	-
		-	+	-	-	-	-	-	AT5G35560.1	-	-	-	-	174839	-	-	-	-	DDB0202195	-	-	43.m00183	-	-	-	-	-	-	-	-	NCU01659	Os03g05200.2	22114	-	-	127755	-	-	409307	-	-	-	-	-	231.m00042	-	-	-	-	89294.m00055	-	-
		-	-	-	-	-	-	-	-	-	-	-	-	174837	-	-	-	-	-	-	-	-	-	-	-	-	-	-	-	-	-	-	-	-	-	-	-	-	-	-	-	-	-	-	-	-	-	-	-	-	-	-
		-	-	-	-	-	-	-	-	-	-	-	-	174835	-	-	-	-	-	-	-	-	-	-	-	-	-	-	-	-	-	-	-	-	-	-	-	-	-	-	-	-	-	-	-	-	-	-	-	-	-	-
DES6a		-	-	-	-	-	-	-	-	-	-	-	-	174831	-	-	-	-	-	-	-	-	-	-	-	-	-	-	-	-	-	-	-	-	-	-	-	-	-	-	-	-	-	-	-	-	-	-	-	-	-	-
		-	+	-	-	-	-	-	-	-	BDEG_06038	-	-	174824	-	-	CME116C	-	-	-	-	-	-	-	-	-	-	-	72415	-	NCU08328	-	12828	GSPATP00027862001	-	-	-	-	-	RO3G_01664.1	-	-	-	-	110.m00152	-	-	-	-	84843.m00308	-	-
		-	-	-	-	-	-	-	-	-	-	-	-	174821	-	-	-	-	-	-	-	-	-	-	-	-	-	-	-	-	-	-	-	-	-	-	-	-	-	-	-	-	-	-	-	-	-	-	-	-	-	-
		-	-	-	-	-	-	-	-	-	-	-	-	174808	-	-	-	-	-	-	-	-	-	-	-	-	-	-	-	-	-	-	-	-	-	-	-	-	-	-	-	-	-	-	-	-	-	-	-	-	-	-
STPK23	Serine/Threonine Protein Kinase Homolog 23 (hypothetical)	-	-	-	-	-	-	-	-	-	-	-	-	174807	-	-	-	-	-	-	-	-	-	-	-	-	-	-	-	-	-	-	-	-	-	-	-	-	-	-	-	-	-	-	-	-	-	-	-	-	-	-
		-	-	-	-	-	-	-	-	-	-	-	-	174803	-	-	-	OTTDARP00000022663	DDB0188129	-	-	-	-	-	-	-	-	-	-	-	-	-	-	-	-	166162	-	-	-	-	YPL199C	-	-	-	-	-	-	-	-	-	-	-
		-	-	-	-	-	-	-	AT2G33380.2	-	-	-	-	174800	-	-	-	-	-	-	-	-	-	-	-	-	-	-	-	-	-	-	-	-	-	125936	-	-	-	-	-	-	-	-	-	-	-	-	-	-	-	-
		-	-	-	-	-	-	-	-	-	-	-	-	174782	-	-	-	-	-	-	-	-	-	-	-	-	-	-	-	-	-	-	-	-	-	-	-	-	-	-	-	-	-	-	-	-	-	-	-	-	-	-
		-	+	-	-	-	-	GB18717-PA	-	-	BDEG_08404	-	165918	174780	-	-	CMH075C	-	-	-	-	-	ENSGALP00000019470	-	OTTHUMP00000161963	-	235746	12003	3276	162204	NCU02973	-	-	-	-	231107	129423	-	-	-	-	SPAC823.10c	-	SINFRUP00000152762	-	-	-	83.m01207	63689	-	-	-
RWP5	hypothetical protein	-	-	-	-	-	-	-	-	-	-	-	-	174777	-	-	-	-	-	-	-	-	-	-	-	-	-	-	-	-	-	Os12g12970.1	-	-	-	-	-	-	-	-	-	-	-	-	-	-	-	-	-	-	-	-
		-	-	-	-	-	-	-	-	-	-	-	-	174774	-	-	-	-	-	-	-	-	-	-	-	-	-	-	-	-	-	-	-	-	-	-	-	-	-	-	-	-	-	-	-	-	-	-	-	-	-	-
		-	-	-	-	-	-	-	-	-	-	-	-	174772	-	-	-	-	-	-	-	-	-	-	-	-	-	-	-	-	-	-	-	-	-	-	-	-	-	-	-	-	-	-	-	-	-	-	-	-	-	-
		-	-	-	-	-	-	-	-	-	-	-	-	174759	-	-	-	-	-	-	-	-	-	-	-	-	-	-	-	-	-	-	-	-	-	-	-	-	-	-	-	-	-	-	-	-	-	-	-	-	-	-
RBD2	putative rubredoxin	-	-	-	-	-	-	-	-	-	-	-	-	174753	-	-	-	-	-	-	-	-	-	-	-	-	-	-	-	-	-	-	33130	-	34177	114321	-	-	-	-	-	-	-	-	-	-	-	-	-	-	-	-
		-	+	-	-	-	-	GB14884-PA	AT1G13330.1	-	BDEG_06681	-	203418	174750	-	cgd2_510	CMP311C	OTTDARP00000022877	DDB0203941	-	19068499	91.m00200	ENSGALP00000030714	GL50803_17044	OTTHUMP00000181379	-	103502	33648	75325	197451	-	Os03g50220.1	32332	GSPATP00005445001	-	151373	130587	-	564514	RO3G_09536.1	-	-	-	SINFRUP00000138203	268.m00039	-	-	55.m00160	54965	85380.m00117	-	-
		-	-	-	-	-	-	-	-	-	-	-	-	174735	-	-	-	-	-	-	-	-	-	-	-	-	-	-	-	-	-	-	34009	-	-	-	-	-	-	-	-	-	-	-	-	-	-	-	-	-	-	-
Unca1		-	+	-	-	-	-	-	AT3G54890.1	-	-	-	-	174723	-	-	-	-	-	-	-	-	-	-	-	-	-	-	-	-	-	Os06g21590.1	15720	-	-	139567	-	-	820261	-	-	-	-	-	-	-	-	-	-	-	-	-
		-	-	-	-	-	-	-	-	-	-	-	-	174713	-	-	-	-	-	-	-	-	-	-	-	-	-	-	-	-	-	-	-	-	-	-	-	-	-	-	-	-	-	-	-	-	-	-	-	-	-	-
RPL23a	Ribosomal protein L23a, component of cytosolic 80S ribosome and 60S large subunit	-	+	+	-	-	-	GB12508-PA	AT2G39460.1	39329	BDEG_05372	CE02777	158372	174711	227127	cgd8_650	CMK273C	-	DDB0191977	CG7977-PA	19069267	28.m00285	ENSGALP00000006308	GL50803_7870	OTTHUMP00000077401	LmjF06.0580	127930	38968	35035	160446	NCU06226	Os04g42270.3	26182	GSPATP00017162001	-	190449	157934	PF13_0132	650312	RO3G_15366.1	YOL127W	SPBC4F6.04	GLEAN3_00379	SINFRUP00000128020	10.m00367	6363	TA17715	49.m00008	36440	87835.m00286	Tb927.7.5180	UM05998.1
		-	-	-	-	-	-	-	-	-	-	-	-	174707	-	-	-	-	-	-	-	-	-	-	-	-	-	-	-	-	-	-	-	-	-	-	-	-	-	-	-	-	-	-	-	-	-	-	-	-	-	-
		-	-	-	-	-	-	-	-	-	-	-	-	174693	-	-	-	-	-	-	-	-	-	-	-	-	-	-	-	-	-	-	13306	-	-	163437	-	-	-	-	-	-	-	-	-	23127	-	-	-	-	-	-
NAH	cation antiporter	-	+	-	-	-	-	-	-	-	-	-	-	174692	-	-	-	-	-	-	19068868	-	-	-	-	-	-	-	-	-	NCU00430	-	-	-	-	-	-	-	-	RO3G_12380.1	YLR138W	SPAC3A11.09	-	-	-	-	-	-	-	-	-	UM02285.1
		-	-	-	-	-	-	-	-	-	-	-	-	174688	-	-	-	-	-	-	-	-	-	-	-	-	-	-	-	-	-	-	-	-	-	-	-	-	-	-	-	-	-	-	-	-	-	-	-	-	-	-
		-	+	-	-	-	-	-	-	-	-	-	220529	174686	259831	-	-	OTTDARP00000012017	-	-	-	-	ENSGALP00000003681	-	OTTHUMP00000034411	LmjF32.3150	232956	13032	48956	50004	-	Os07g33370.1	32544	-	35233	160117	-	-	-	-	-	-	-	SINFRUP00000127507	-	2653	-	-	53434	-	-	-
		-	-	-	-	-	-	-	-	-	-	-	-	174681	-	-	CMF050C	-	-	-	-	-	-	-	-	-	-	-	-	-	-	-	-	-	-	-	-	-	-	-	-	-	-	-	-	-	-	-	-	-	-	-
		-	+	-	-	-	-	GB13995-PA	AT4G37210.1	-	BDEG_03533	-	227744	174677	240637	-	CMA034C	OTTDARP00000020420	DDB0184122	-	-	-	ENSGALP00000037604	-	OTTHUMP00000009509	-	55307	-	49321	-	-	-	-	-	-	4158	-	-	208393	RO3G_01341.1	-	-	-	SINFRUP00000175793	-	-	-	-	64051	-	-	UM02530.1
		-	+	-	-	-	-	-	AT4G19190.1	-	-	CE11194	-	174676	-	-	-	-	-	-	-	-	-	-	-	-	223519	-	-	117089	-	-	-	-	-	57148	-	-	827503	-	-	-	-	-	-	-	-	-	54065	-	-	-
		-	+	-	-	-	-	-	-	-	-	-	225333	174674	-	-	-	-	DDB0187664	-	-	-	-	-	-	-	170634	-	-	237975	-	-	-	-	-	-	-	-	-	-	-	-	-	-	-	-	-	-	53976	-	-	-
		-	-	-	-	-	-	-	-	-	-	-	-	174654	-	-	-	-	-	-	-	-	-	-	-	-	-	-	-	-	-	-	-	-	-	-	-	-	-	-	-	-	-	-	-	-	-	-	-	-	-	-
		-	-	-	-	-	-	-	-	-	-	-	-	174653	-	-	-	-	-	-	-	-	-	-	-	-	-	-	-	-	-	-	-	-	-	-	-	-	-	-	-	-	-	-	-	-	-	-	-	-	-	-
		-	-	-	-	-	-	-	-	-	-	-	-	174644	-	-	-	-	-	-	-	-	-	-	-	-	-	-	-	-	-	-	-	-	-	-	-	-	-	-	-	-	-	-	-	-	-	-	-	-	-	-
		-	-	-	-	-	-	-	-	-	-	-	-	174629	-	-	-	-	-	-	-	-	-	-	-	-	-	-	-	-	-	-	-	-	-	-	-	-	-	-	-	-	-	-	-	-	-	-	-	-	-	-
		-	-	-	-	-	-	-	-	-	-	-	-	174617	-	-	-	-	-	-	-	-	-	-	-	-	-	-	-	-	-	-	-	-	-	-	-	-	-	-	-	-	-	-	-	-	-	-	-	-	-	-
		-	+	-	-	-	-	-	AT5G58100.1	-	-	-	-	174615	-	-	-	-	DDB0218668	-	-	-	-	-	-	-	-	-	-	-	-	Os02g26984.1	-	-	-	206630	-	-	560714	-	-	-	-	-	-	-	-	-	-	-	-	-
		-	-	-	-	-	-	-	-	-	-	-	-	174614	-	-	-	-	-	-	-	-	-	-	-	-	-	-	-	-	-	-	-	-	-	-	-	-	-	-	-	-	-	-	-	-	-	-	-	-	-	-
		-	-	-	-	-	-	-	-	-	-	-	-	174609	-	-	-	-	-	-	-	-	-	-	-	-	-	-	-	-	-	-	-	-	-	-	-	-	-	-	-	-	-	-	-	-	-	-	-	-	-	-
		-	+	-	-	-	-	-	-	-	-	-	90932	174594	280087	-	-	-	-	-	-	-	ENSGALP00000004539	-	NP_065894.1	LmjF18.0490	215534	-	-	208963	-	Os06g12882.1	-	-	-	76835	-	-	569942	-	-	-	-	SINFRUP00000148561	-	-	-	-	60496	-	-	-
		-	-	-	-	-	-	-	-	-	-	-	-	174588	-	-	-	-	-	-	-	-	-	-	-	-	-	-	-	-	-	-	-	-	-	-	-	-	-	-	-	-	-	-	-	-	-	-	-	-	-	-
		-	-	-	-	-	-	-	-	-	-	-	-	174584	-	-	-	-	-	-	-	-	-	-	-	-	-	-	-	-	-	-	-	-	-	-	-	-	-	-	-	-	-	-	-	-	-	-	-	-	-	-
		-	+	-	-	-	-	GB11513-PA	AT2G29210.1	30707	BDEG_04959	CE30346	163872	174571	247920	-	-	-	DDB0186969	CG11274-PA	-	14.m00325	ENSGALP00000040312	-	OTTHUMP00000003373	-	137917	32808	53975	132837	NCU03285	Os03g16369.2	-	GSPATP00009687001	43565	182200	120321	PFC0465c	172546	-	-	SPCC825.05c	GLEAN3_19555	SINFRUP00000136964	2.m02458	-	TA12780	583.m00632	7832	-	-	UM01915.1
ARP	NADPH:quinone oxidoreductase, mitochondrial	-	+	-	-	-	-	GB17125-PA	AT1G49670.1	-	BDEG_07988	CE40393	173764	174569	262349	-	-	-	DDB0186919	-	-	-	ENSGALP00000036151	-	OTTHUMP00000073365	LmjF36.4170	201415	-	31511	82630	-	Os09g28570.1	-	GSPATP00020431001	41413	179921	108301	-	201674	RO3G_11246.1	-	SPAPB24D3.08c	GLEAN3_18882	SINFRUP00000133668	1.m00983	-	-	-	19171	-	Tb10.6k15.0820	-
		-	-	-	-	-	-	-	-	-	-	-	-	174551	-	-	-	-	-	-	-	-	-	-	-	-	-	-	-	-	-	-	-	-	-	-	-	-	-	-	-	-	-	-	-	-	-	-	-	-	-	-
		-	-	-	-	-	-	-	-	-	-	-	-	174550	-	-	-	-	-	-	-	-	-	-	-	-	-	-	-	-	-	-	-	-	-	-	-	-	-	-	-	-	-	-	-	-	-	-	-	-	-	-
		-	-	-	-	-	-	-	-	-	-	-	-	174547	-	-	-	-	-	-	-	-	-	-	-	-	192633	-	-	-	-	-	-	-	-	169073	-	-	-	-	-	-	-	-	-	-	-	-	-	-	-	-
		-	-	-	-	-	-	-	-	-	-	-	-	174537	-	-	-	-	-	-	-	-	-	-	-	-	-	-	-	-	-	Os06g06770.1	-	-	-	-	-	-	-	-	-	-	-	-	-	-	-	-	-	-	-	-
		-	-	-	-	-	-	-	-	-	-	-	-	174533	-	-	-	-	-	-	-	-	-	-	-	-	-	-	-	-	-	-	-	-	-	-	-	-	-	-	-	-	-	-	-	-	-	-	-	-	-	-
		-	-	-	-	-	-	-	-	-	-	-	-	174530	-	-	-	-	-	-	-	-	-	-	-	-	-	-	-	-	-	-	-	-	-	-	-	-	-	-	-	-	-	-	-	-	-	-	-	-	-	-
		-	+	-	-	-	-	GB13954-PA	AT4G20850.1	18489	BDEG_08541	CE01917	228048	174517	-	-	-	-	-	CG3991-PA	-	-	ENSGALP00000027194	-	OTTHUMP00000018664	-	129137	34612	78168	176183	-	Os02g44520.1	-	-	26948	111640	137699	-	823475	RO3G_04302.1	-	SPAP8A3.12c	-	SINFRUP00000179411	-	-	-	-	61672	-	-	UM00517.1
		-	-	-	-	-	-	-	-	-	-	-	-	174508	-	-	-	-	-	-	-	-	ENSGALP00000026295	-	-	-	-	11169	-	94752	-	-	-	-	-	-	-	-	-	-	-	-	-	-	-	-	-	-	-	-	-	-
		-	-	-	-	-	-	-	-	-	-	-	-	174504	-	-	-	-	-	-	-	-	-	-	-	-	-	-	-	-	-	-	-	-	-	-	-	-	-	-	-	-	-	-	-	-	-	-	-	-	-	-
		-	-	-	-	-	-	-	-	-	-	-	-	174500	-	-	-	-	-	-	-	-	-	-	-	-	-	-	-	-	-	-	-	-	-	-	-	-	-	-	-	-	-	-	-	-	-	-	-	-	-	-
		-	-	-	-	-	-	-	-	-	-	-	-	174499	-	-	-	-	-	-	-	-	-	-	-	-	-	-	-	-	-	-	-	-	-	-	-	-	-	-	-	-	-	-	-	-	-	-	-	-	-	-
		-	-	-	-	-	-	-	-	-	-	-	-	174498	-	-	-	-	-	-	-	-	-	-	-	-	-	-	-	-	-	-	-	-	-	-	-	-	-	-	-	-	-	-	-	-	-	-	-	-	-	-
		-	-	-	-	-	-	-	-	-	-	-	-	174493	-	-	-	-	-	-	-	-	-	-	-	-	-	-	-	-	-	-	-	-	43308	169861	-	-	-	-	-	-	-	-	-	4292	-	-	-	-	-	-
		-	-	-	-	-	-	-	-	-	-	-	-	174492	-	-	-	-	-	-	-	-	-	-	-	-	-	-	-	-	-	-	-	-	-	-	-	-	-	-	-	-	-	-	-	-	-	-	-	-	-	-
		-	+	-	-	-	-	GB18629-PA	AT3G49000.1	60884	BDEG_07692	CE08817	158707	174488	297911	-	-	OTTDARP00000021597	DDB0189763	-	-	24.m00281	-	-	OTTHUMP00000015591	LmjF02.0680	157828	35969	66106	117247	NCU03560	Os01g48720.1	30753	-	47116	142596	144553	-	253945	RO3G_12146.1	YPR190C	SPAPB1E7.03	-	SINFRUP00000131470	-	269018	-	-	54190	85661.m00067	Tb927.2.2990	UM03058.1
		-	+	+	-	-	-	GB15859-PA	AT1G42440.1	18509	BDEG_02071	CE02624	173748	174486	-	cgd5_1240	CMA015C	OTTDARP00000021438	DDB0205380	CG7338-PA	19068705	114.m00133	ENSGALP00000005037	GL50803_5294	NP_060598.3	LmjF07.0470	105531	15285	57582	170367	NCU01503	Os11g19250.1	17263	GSPATP00036913001	6258	119852	133691	PF14_0494	818858	RO3G_12091.1	YDL060W	SPAC23H4.15	GLEAN3_15087	SINFRUP00000173476	-	24590	-	57.m03994	527	94962.m00269	Tb927.8.1410	UM04986.1
		-	-	-	-	-	-	-	-	-	-	-	-	174481	-	-	-	-	-	-	-	-	-	-	-	-	-	-	-	-	-	-	-	-	-	-	-	-	-	-	-	-	-	-	-	-	-	-	-	-	-	-
		-	-	-	-	-	-	-	-	-	-	-	-	174480	-	-	-	-	-	-	-	-	-	-	-	-	-	-	-	-	-	-	-	-	-	-	-	-	-	-	-	-	-	-	-	-	-	-	-	-	-	-
		-	-	-	-	-	-	-	-	-	-	-	-	174476	-	-	-	-	-	-	-	-	-	-	-	-	-	-	-	-	-	-	-	-	-	-	-	-	-	-	-	-	-	-	-	-	-	-	-	-	-	-
		-	+	-	-	-	-	-	-	-	-	-	223950	174475	-	cgd3_1860	-	-	DDB0218061	CG7526-PB	-	-	-	-	NP_066548.2	-	133234	-	-	212	-	Os09g29600.1	-	-	-	-	-	-	-	-	-	-	-	-	-	-	-	-	-	-	-	-
		-	+	-	-	-	-	-	AT2G37330.1	13990	BDEG_05066	-	-	174471	-	-	CMS400C	-	-	-	-	-	-	-	-	-	-	-	-	-	-	Os05g02750.1	-	-	15102	114214	-	-	561531	RO3G_06745.1	-	-	-	-	-	-	-	-	-	-	-	-
		-	-	-	-	-	-	-	-	-	-	-	-	174455	-	-	-	-	-	-	-	-	-	-	-	-	-	-	-	-	-	-	-	-	-	-	-	-	-	-	-	-	-	-	-	-	-	-	-	-	-	-
		-	-	-	-	-	-	-	-	-	-	-	-	174446	-	-	-	-	-	-	-	-	-	-	-	-	-	-	-	-	-	-	-	-	-	-	-	-	-	-	-	-	-	-	-	-	-	-	-	-	-	-
		-	+	-	-	-	-	-	-	-	-	-	-	174445	-	cgd3_590	-	-	DDB0202532	-	-	78.m00172	-	-	-	-	-	-	-	-	-	-	-	-	-	-	140737	MAL13P1.298	-	-	-	-	-	-	-	-	TA12220	80.m03996	-	-	-	-
		-	-	-	-	-	-	-	-	-	-	-	-	174438	-	-	-	-	-	-	-	-	-	-	-	-	-	-	-	-	-	-	-	-	-	-	-	-	-	-	-	-	-	-	-	-	-	-	-	-	-	-
		-	-	-	-	-	-	-	-	-	-	-	-	174437	-	-	-	-	-	-	-	-	-	-	-	-	-	-	-	-	-	-	-	-	-	-	-	-	-	-	-	-	-	-	-	-	-	-	-	-	-	-
PRFA	peptide chain release factor RF-1	-	+	-	-	-	-	-	AT3G62910.1	-	-	-	-	174432	-	-	CMH024C	-	-	-	-	-	-	-	-	LmjF25.1450	117048	-	80818	-	-	Os11g43600.1	8896	GSPATP00031367001	36081	136596	-	PF14_0265	572734	-	-	-	-	-	1.m00827	33161	-	25.m01748	31311	-	Tb927.3.1070	-
		-	+	-	-	-	-	GB16812-PB	AT5G55160.1	-	-	-	165362	174416	278795	-	-	OTTDARP00000020160	-	CG4494-PA	-	-	ENSGALP00000033053	GL50803_7760	OTTHUMP00000115275	-	-	-	-	91283	-	Os01g68940.1	-	-	-	-	-	PFE0285c	552739	-	YDR510W	-	GLEAN3_18833	SINFRUP00000142614	-	-	-	-	-	86622.m00280	-	-
	Serine/threonine protein kinase	-	-	-	-	-	-	-	-	-	-	-	-	174410	-	-	-	-	-	-	-	-	-	-	-	-	-	-	-	-	-	-	-	-	-	-	-	-	-	-	-	-	-	-	-	-	-	-	-	-	-	-
		-	-	-	-	-	-	-	AT5G61380.1	-	-	-	-	174408	-	-	-	-	-	-	-	-	-	-	-	-	-	-	-	-	-	Os02g40510.3	-	-	-	-	-	-	784463	-	-	-	-	-	-	-	-	-	-	-	-	-
FAP200	Flagellar Associated Protein	-	-	-	+	-	-	-	-	-	-	-	-	174407	-	-	-	-	-	-	-	-	-	-	-	-	-	-	-	-	-	-	-	-	-	-	-	-	-	-	-	-	-	-	-	-	-	-	-	-	-	-
		-	-	-	-	-	-	-	-	-	-	-	-	174403	-	-	-	-	-	-	-	-	-	-	-	-	-	-	-	-	-	-	-	-	-	-	-	-	-	-	-	-	-	SINFRUP00000145587	-	-	-	-	-	-	-	-
		-	-	-	-	-	-	-	-	-	-	-	-	174401	-	-	-	-	-	-	-	-	-	-	-	-	-	-	-	-	-	-	-	-	-	-	-	-	-	-	-	-	-	-	-	-	-	-	-	-	-	-
		-	-	-	-	-	-	-	-	-	-	-	-	174398	-	-	-	-	-	-	-	-	-	-	-	-	-	-	-	-	-	-	-	-	-	-	-	-	592552	-	-	-	-	-	-	-	-	-	-	-	-	-
		-	-	-	-	-	-	-	-	-	-	-	-	174377	-	-	-	-	-	-	-	-	-	-	-	-	-	-	-	-	-	-	-	-	-	-	-	-	-	-	-	-	-	-	-	-	-	-	-	-	-	-
		-	-	-	-	-	-	-	-	-	-	-	-	174368	-	-	-	-	-	-	-	-	-	-	-	-	-	-	-	-	-	-	-	-	-	-	-	-	-	-	-	-	-	-	-	-	-	-	-	-	-	-
		-	-	-	-	-	-	-	-	-	-	-	-	174367	-	-	-	-	-	-	-	-	-	-	-	-	-	-	-	-	-	-	-	-	-	-	-	-	-	-	-	-	-	-	-	-	-	-	-	-	-	-
		-	+	-	-	-	-	-	AT5G66030.1	-	-	-	155764	174364	-	-	-	-	DDB0188296	-	-	-	-	-	-	-	212469	-	-	-	-	Os07g28940.1	-	-	-	114643	-	-	818360	-	-	-	-	-	-	-	-	-	-	-	-	-
		-	-	-	-	-	-	-	-	-	-	-	-	174363	-	-	-	-	-	-	-	-	-	-	-	-	-	-	-	-	-	-	-	-	-	-	-	-	-	-	-	-	-	-	-	-	-	-	-	-	-	-
		-	-	-	-	-	-	-	-	-	-	-	-	174362	-	-	-	-	-	-	-	-	-	-	-	-	-	-	-	-	-	-	-	-	-	-	-	-	-	-	-	-	-	-	-	-	-	-	-	-	-	-
		-	-	-	-	-	-	-	-	-	-	-	-	174361	-	-	-	-	-	-	-	-	-	-	-	-	-	-	-	-	-	-	-	-	-	-	-	-	-	-	-	-	-	-	-	-	-	-	-	-	-	-

		-	-	-	-	-	-	-	-	-	-	-	-	174360	-	-	-	-	-	-	-	-	-	-	-	-	-	-	-	-	-	-	-	-	-	-	-	-	-	-	-	-	-	-	-	-	-	-	-	-	-	-
		-	-	-	-	-	-	-	-	-	-	-	-	174359	-	-	-	-	-	-	-	-	-	-	-	-	-	-	-	-	-	-	-	-	-	-	-	-	-	-	-	-	-	-	-	-	-	-	-	-	-	-
		-	+	-	-	-	-	-	AT4G30580.1	34654	-	-	-	174358	-	-	CMF185C	-	-	-	-	-	-	-	-	-	-	-	-	-	-	-	Os10g35390.1	33965	-	-	140302	-	-	762928	-	-	-	-	-	-	-	-	-	-	-	-	-
		-	-	-	-	-	-	-	AT1G27750.1	-	-	-	-	174352	-	-	-	-	-	-	-	-	-	-	-	-	-	-	-	-	-	-	Os01g56000.1	-	-	-	176266	-	-	243881	-	-	-	-	-	-	-	-	-	-	-	-	-
		-	-	-	-	-	-	-	-	-	-	-	-	174351	-	-	-	-	-	-	-	-	-	-	-	-	-	-	-	-	-	-	-	-	-	-	-	-	-	-	-	-	-	-	-	-	-	-	-	-	-	-
		-	-	-	-	-	-	-	-	-	-	-	-	174349	-	-	-	-	-	-	-	-	-	-	-	-	-	-	-	-	-	-	-	-	-	-	-	-	-	-	-	-	-	-	-	-	-	-	-	-	-	-
		-	-	-	-	-	-	-	-	-	-	-	-	174348	-	-	-	-	-	-	-	-	-	-	-	-	-	-	-	-	-	-	-	-	-	-	-	-	-	-	-	-	-	-	-	-	-	-	-	-	-	-
		-	-	-	-	-	-	-	-	-	-	-	-	174341	-	-	-	-	-	-	-	-	-	-	-	-	-	-	-	-	-	-	-	-	-	-	-	-	-	-	-	-	-	-	-	-	-	-	-	-	-	-
		-	-	-	-	-	-	-	-	-	-	-	213087	174339	-	-	-	-	-	-	-	-	-	-	-	-	-	-	-	-	-	-	-	-	-	-	-	-	-	-	-	-	-	-	-	-	-	-	-	-	-	-
		-	-	-	-	-	-	-	-	-	-	-	-	174338	-	-	-	-	-	-	-	-	-	-	-	-	-	-	-	-	-	-	-	-	-	-	-	-	-	-	-	-	-	-	-	-	-	-	-	-	-	-
		-	-	-	-	-	-	-	-	-	-	-	-	174337	-	-	-	-	-	-	-	-	-	-	-	-	-	-	-	-	-	-	-	-	-	-	-	-	-	-	-	-	-	-	-	-	-	57.m01765	-	-	-	-
		-	-	-	-	-	-	-	-	-	-	-	-	174330	-	-	-	-	-	-	-	-	-	-	-	-	-	-	-	-	-	-	-	-	-	-	-	-	-	-	-	-	-	-	-	-	-	-	-	-	-	-
		-	-	-	-	-	-	-	-	-	-	-	-	174319	-	-	-	-	-	-	-	-	-	-	-	-	-	-	-	-	-	-	-	-	-	-	-	-	-	-	-	-	-	-	-	-	-	-	-	-	-	-
FAP177	Flagellar Associated Protein	-	+	-	+	-	-	-	AT3G54780.1	-	-	-	-	174303	-	-	-	OTTDARP00000023610	-	-	-	-	ENSGALP00000011148	-	-	-	73285	33043	78811	-	-	Os03g04890.1	-	GSPATP00006023001	54892	-	-	-	179717	-	-	-	-	-	111.m00142	23377	-	-	-	-	-	-
DNJ12	DnaJ-like protein 12, rather cytosolic	-	-	-	-	-	-	-	-	-	-	-	-	174302	-	-	-	-	-	-	-	-	-	-	-	-	-	-	-	-	-	-	-	-	-	-	-	-	-	-	-	-	-	-	-	-	-	-	-	-	-	-
		-	-	-	-	-	-	-	-	-	-	-	-	174301	-	-	-	-	-	-	-	-	-	-	-	-	-	-	-	-	-	-	-	-	-	-	-	-	-	-	-	-	-	-	-	-	-	-	-	-	-	-
		-	-	-	-	-	-	-	-	-	-	-	-	174299	-	-	-	-	-	-	-	-	-	-	-	-	-	-	-	-	-	-	-	-	-	-	-	-	-	-	-	-	-	-	-	-	-	-	-	-	-	-
		-	-	-	-	-	-	-	-	-	-	-	-	174294	-	-	-	OTTDARP00000019831	-	-	-	-	-	-	-	-	-	-	-	112507	-	-	-	-	-	-	-	-	-	-	-	-	-	-	-	-	-	-	-	-	-	-
		-	-	-	-	-	-	-	-	-	-	-	-	174292	-	-	-	-	-	-	-	-	-	-	-	-	-	-	-	-	-	-	-	-	-	-	-	-	-	-	-	-	-	-	-	-	-	-	-	-	-	-
		-	+	-	-	-	-	-	AT5G11760.1	-	-	-	-	174291	-	-	CMT406C	-	DDB0218223	-	19168672	19.m00308	-	GL50803_6365	-	-	-	-	47253	-	-	-	-	GSPATP00033609001	-	-	135265	-	577927	-	-	SPAC6G9.01c	-	-	32.m00255	-	-	-	-	90925.m00110	-	-
		-	-	-	-	-	-	-	-	-	-	-	-	174289	-	-	-	-	-	-	-	-	-	-	-	-	-	-	-	-	-	-	-	-	-	-	-	-	-	-	-	-	-	-	-	-	-	-	-	-	-	-
C_80019		-	+	-	-	-	-	GB30069-PA	-	-	-	-	205981	174287	-	-	-	-	DDB0188642	CG12787-PA	-	-	ENSGALP00000026970	-	OTTHUMP00000175497	-	-	29712	69397	122951	-	Os10g31040.6	8380	-	44788	-	-	-	587601	RO3G_16454.1	-	-	-	SINFRUP00000162273	-	262258	-	-	-	93857.m00324	-	UM01138.1
		-	-	-	-	-	-	-	-	-	-	-	-	174284	-	-	-	-	-	-	-	-	-	-	-	-	-	-	-	-	-	-	-	-	-	-	-	-	-	-	-	-	-	-	-	-	-	-	-	-	-	-
		-	+	-	-	-	-	GB17101-PA	AT3G57550.2	-	BDEG_02350	CE29460	152418	174270	298222	cgd7_2190	CMD076C	OTTDARP00000016990	-	CG11811-PA	19068527	-	ENSGALP00000008546	GL50803_7203	OTTHUMP00000037742	-	151231	-	55119	205628	NCU06300	Os12g33100.1	-	GSPATP00009496001	11281	148828	-	PFI1420w	652750	RO3G_03408.1	YDR454C	SPBC1198.05	GLEAN3_03457	SINFRUP00000150429	87.m00251	3448	TA13945	49.m00068	50000	95540.m00037	Tb10.26.0200	UM02849.1
		-	-	-	-	-	-	-	-	-	-	-	-	174264	-	-	-	-	-	-	-	-	-	-	-	-	-	-	-	-	-	-	10541	-	-	84414	-	-	-	-	-	-	-	-	-	-	-	-	-	-	-	-
		-	-	-	-	-	-	-	-	-	-	-	-	174261	-	-	-	-	-	-	-	-	-	-	-	-	-	-	-	-	-	-	-	-	43946	-	-	-	-	-	-	-	-	-	-	-	-	-	-	-	-	-
		-	-	-	-	-	-	-	-	-	-	-	-	174259	-	-	-	-	-	-	-	-	-	-	-	-	-	-	-	-	-	-	-	-	-	-	-	-	-	-	-	-	-	-	-	-	-	-	-	-	-	-
		-	-	-	-	-	-	-	-	-	-	-	-	174249	-	-	-	-	-	-	-	-	-	-	-	-	-	-	-	-	-	-	-	-	-	-	-	-	-	-	-	-	-	-	-	-	-	-	-	-	-	-
		-	-	-	-	-	-	-	-	-	-	-	-	174242	-	-	-	-	-	-	-	-	-	-	-	-	-	-	-	-	-	-	-	-	-	-	-	-	-	-	-	-	-	-	-	-	-	-	-	-	-	-
		-	-	-	-	-	-	-	-	-	-	-	-	174238	-	-	-	-	-	-	-	-	-	-	-	-	-	-	-	-	-	-	-	-	-	-	-	-	-	-	-	-	-	-	-	-	-	-	-	-	-	-
IRT2	iron-nutrition responsive ZIP family transporter	-	-	-	-	-	-	-	-	-	-	-	-	174212	-	-	-	-	-	-	-	-	-	-	-	-	-	-	-	-	-	-	34352	-	-	-	-	-	-	-	-	-	-	-	-	-	-	-	-	-	-	-
		-	-	-	-	-	-	-	-	-	-	-	-	174205	-	-	-	-	-	-	-	-	-	-	-	-	-	-	-	-	-	-	-	-	-	-	-	-	-	-	-	-	-	-	-	-	-	-	-	-	-	-
		-	-	-	-	-	-	-	-	-	-	-	-	174201	-	-	-	-	-	-	-	-	-	-	-	-	-	-	-	-	-	-	-	-	-	-	-	-	-	-	-	-	-	-	-	-	-	-	-	-	-	-
		-	+	-	-	-	-	GB12267-PA	AT2G18220.1	-	BDEG_03265	CE15606	141038	174200	230313	-	-	-	DDB0185982	CG9246-PA	-	9.m00434	ENSGALP00000003474	-	NP_056473.2	LmjF18.1610	228789	37390	77748	247088	NCU02066	Os10g35280.1	1664	GSPATP00005057001	32036	117081	127868	-	178110	RO3G_04874.1	YOR206W	SPAC1142.04	-	SINFRUP00000134605	-	-	-	-	57673	-	Tb10.389.1290	UM02343.1
		-	-	-	-	-	-	-	-	-	-	-	-	174199	-	-	-	-	-	-	-	-	-	-	-	-	-	-	-	-	-	-	-	-	-	-	-	-	-	-	-	-	-	-	-	-	-	-	-	-	-	-
		-	-	-	-	-	-	-	-	-	-	-	-	174194	-	-	-	-	-	-	-	-	-	-	-	-	-	-	-	-	-	-	-	-	-	-	-	-	-	-	-	-	-	-	-	-	-	-	-	-	-	-
		-	-	-	-	-	-	-	-	-	-	-	-	174192	-	-	-	-	-	-	-	-	-	-	-	-	-	-	-	-	-	-	-	-	-	-	-	-	-	-	-	-	-	-	-	-	-	-	-	-	-	-
		-	-	-	+	-	-	-	-	-	-	-	-	174181	-	-	-	-	-	-	-	-	-	-	-	-	-	-	-	-	-	-	-	-	-	-	-	-	-	-	-	-	-	-	-	-	-	-	-	-	-	-
		-	-	-	-	-	-	-	-	-	-	-	-	174180	-	-	-	-	-	-	-	-	-	-	-	-	-	-	-	-	-	-	-	-	-	-	-	-	-	-	-	-	-	-	-	-	-	-	-	-	-	-
		-	+	-	-	-	-	GB14184-PA	AT3G20550.1	6160	BDEG_02111	CE08529	219735	174174	-	cgd1_3470	-	-	-	CG17168-PA	-	110.m00135	ENSGALP00000003126	-	OTTHUMP00000004404	-	117888	9812	68818	23431	NCU06883	Os05g46780.1	32965	GSPATP00033008001	15338	117331	127413	PF13_0042	242678	RO3G_05804.1	YLR016C	-	-	-	1.m01106	262193	TA15135	20.m03676	24288	95068.m00184	Tb927.3.5360	-
		-	-	-	-	-	-	-	-	-	-	-	-	174168	-	-	-	-	-	-	-	-	-	-	-	-	-	-	-	-	-	-	-	-	-	-	-	-	-	-	-	-	-	-	-	-	-	-	-	-	-	-
		-	-	-	-	-	-	-	AT2G28910.1	-	-	-	-	174154	-	-	-	-	-	-	-	-	-	-	-	-	-	-	-	-	-	Os09g01640.1	-	-	-	183075	-	-	589953	-	-	-	-	-	-	-	-	-	-	-	-	-
		-	-	-	-	-	-	-	-	-	-	-	-	174138	-	-	-	-	-	-	-	-	-	-	-	-	-	-	-	-	-	-	-	-	-	-	-	-	-	-	-	-	-	-	-	-	-	-	-	-	-	-
		-	+	-	-	-	-	GB18842-PA	-	-	-	-	-	174137	-	-	-	-	-	-	-	-	ENSGALP00000015663	-	OTTHUMP00000000554	-	-	27045	-	236347	-	Os08g19170.1	-	-	-	-	-	-	578806	-	-	-	-	SINFRUP00000130210	-	-	-	-	-	-	-	-
		-	-	-	-	-	-	-	-	-	-	CE32497	-	174136	-	-	-	-	-	-	-	-	-	-	-	-	-	-	-	-	-	-	-	-	-	-	-	-	-	-	-	-	-	-	-	-	-	-	-	-	-	-
		-	+	-	-	-	-	-	-	-	-	-	-	174129	-	-	CMS249C	-	-	-	-	-	-	-	-	-	-	-	-	-	-	-	31365	-	45660	-	-	-	-	-	-	-	-	-	-	2537	-	59.m03696	-	-	-	-
		-	-	-	-	-	-	-	-	-	-	-	-	174124	-	-	-	-	-	-	-	-	-	-	-	-	-	-	-	-	-	-	-	-	-	-	-	-	-	-	-	-	-	-	-	-	-	-	-	-	-	-
		-	-	-	-	-	-	-	-	-	-	-	-	174122	-	-	-	-	-	-	-	-	-	-	-	-	-	-	-	-	-	-	-	-	-	-	-	-	-	-	-	-	-	-	-	-	-	-	-	-	-	-
		-	-	-	-	-	-	-	-	-	-	-	-	174121	-	-	-	-	-	-	-	-	-	-	-	-	-	-	-	-	-	-	-	-	-	-	-	-	-	-	-	-	-	-	-	-	-	-	-	-	-	-
		-	+	-	-	-	-	GB10636-PA	AT3G11620.1	-	BDEG_04520	-	-	174114	-	-	CMB101C	-	DDB0187018	CG9186-PA	-	-	ENSGALP00000030947	-	OTTHUMP00000151185	-	152065	-	-	127691	-	Os01g72870.2	-	-	-	181824	135492	-	835473	-	YPR147C	-	-	SINFRUP00000127847	-	-	-	-	28821	-	-	-
MRPS18	Putative mitochondrial ribosomal protein S18, imported to mitochondria	-	+	-	-	-	-	-	AT1G07210.1	-	-	-	-	174113	-	-	-	-	-	-	-	-	-	-	-	-	-	-	77960	-	-	Os10g35690.1	21094	GSPATP00012801001	-	-	-	-	585168	-	-	-	-	-	-	-	-	-	-	-	-	-
IPY3		-	+	-	-	-	-	-	AT3G53620.1	-	-	-	-	174103	-	-	-	-	-	-	-	-	-	-	-	-	-	-	34640	78577	-	Os05g02310.1	-	GSPATP00001997001	-	137938	-	-	652945	-	-	-	-	-	188.m00068	-	-	-	34969	88043.m00098	-	-
AST1	aspartate aminotransferase	-	+	-	+	-	-	-	AT5G11520.1	27395	-	-	21487	174097	238339	-	CMC148C	OTTDARP00000023278	DDB0204794	-	-	-	ENSGALP00000003646	GL50803_91056	-	LmjF35.0820	-	-	-	86861	NCU07941	Os01g55540.1	-	GSPATP00023219001	23871	136815	108281	-	835881	-	-	SPBC725.01	GLEAN3_15274	-	351.m00013	269248	TA12970	50.m03247	63196	-	Tb11.02.2740	-
		-	-	-	-	-	-	-	-	-	-	-	-	174095	-	-	-	-	-	-	-	-	-	-	-	-	-	-	-	-	-	-	-	-	-	-	-	-	-	-	-	-	-	-	-	-	-	-	-	-	-	-
		-	-	-	-	-	-	-	-	-	-	-	-	174078	-	-	-	-	-	-	-	-	-	-	-	-	-	-	-	-	-	-	-	-	-	-	-	-	-	-	-	-	-	-	-	-	-	-	-	-	-	-
		-	-	-	-	-	-	-	-	-	-	-	-	174076	-	-	-	-	-	-	-	-	-	-	-	-	-	-	-	-	-	-	-	-	-	-	-	-	-	-	-	-	-	-	-	-	-	-	-	-	-	-
		-	-	-	-	-	-	-	-	-	-	-	-	174074	-	-	-	-	-	-	-	-	-	-	-	-	-	-	-	-	-	-	-	-	-	-	-	-	-	-	-	-	-	-	-	-	-	-	-	-	-	-
		-	-	-	-	-	-	-	-	-	-	-	-	174073	-	-	-	-	-	-	-	-	-	-	-	-	-	-	-	-	-	-	-	-	-	-	-	-	-	-	-	-	-	-	-	-	-	-	-	-	-	-
		-	-	-	-	-	-	-	-	-	-	-	-	174072	-	-	-	-	-	-	-	-	-	-	-	-	-	-	-	-	-	-	-	-	-	-	-	-	-	-	-	-	-	-	-	-	-	-	-	-	-	-
DBR1	Debranching enzyme that hydrolyzes the 2'-5' phosphodiester bond of lariat introns produced during mRNA splicing	-	+	-	-	-	-	GB12483-PA	-	-	-	CE30437	170958	174055	-	-	-	-	DDB0206474	-	-	-	ENSGALP00000001803	-	OTTHUMP00000172997	LmjF17.0830	63389	28011	69895	-	-	Os03g51540.1	-	-	27234	-	156669	-	825797	RO3G_10360.1	YKL149C	SPAC17A5.02c	GLEAN3_01207	SINFRUP00000155308	-	-	-	49.m03094	-	-	Tb927.5.2130	UM01011.1
		-	-	-	-	-	-	-	-	-	-	-	-	174034	-	-	-	-	-	-	-	-	-	-	-	-	-	-	-	-	-	-	-	-	-	-	-	-	-	-	-	-	-	-	-	-	-	-	-	-	-	-
FAP112	Flagellar Associated Protein	-	-	-	+	-	-	-	-	-	-	-	-	174030	-	-	-	-	-	-	-	-	-	-	-	-	-	-	-	-	-	-	-	-	-	-	-	-	-	-	-	-	-	-	-	-	-	-	-	-	-	-
		-	-	-	-	-	-	-	-	-	-	-	-	174026	-	-	-	-	-	-	-	-	-	-	-	-	-	-	-	-	-	-	-	-	-	-	-	-	-	-	-	-	-	-	-	-	-	-	-	-	-	-
		-	-	-	-	-	-	-	-	-	-	-	-	174024	-	-	-	-	-	-	-	-	-	-	-	-	-	-	-	-	-	-	-	-	-	-	-	-	-	-	-	-	-	-	-	-	-	-	-	-	-	-
		-	+	-	-	-	-	-	AT4G34730.1	-	-	-	-	174010	-	-	CMT486C	-	-	-	-	-	-	-	-	-	-	-	-	-	-	Os06g04610.1	33952	-	-	36552	-	-	800817	-	-	-	-	-	-	-	-	-	-	-	-	-
		-	-	-	-	-	-	-	-	-	-	-	-	174005	-	-	-	-	-	-	-	-	-	-	-	-	-	-	-	-	-	-	-	-	-	-	-	-	-	-	-	-	-	-	-	-	-	-	-	-	-	-
CYP769A1	cytochrome P450, unkown function	-	-	-	-	-	-	-	-	-	-	-	-	173996	-	-	-	-	-	-	-	-	-	-	-	-	-	-	-	-	-	-	-	-	-	-	-	-	-	-	-	-	-	-	-	-	-	-	-	-	-	-
		-	-	-	-	-	-	-	-	-	-	-	-	173990	-	-	-	-	-	-	-	-	-	-	-	-	-	-	-	-	-	-	-	-	-	-	-	-	-	-	-	-	-	-	-	-	-	-	-	-	-	-
		-	-	-	-	-	-	-	-	-	-	-	-	173988	-	-	-	-	-	-	-	-	-	-	-	-	-	-	-	-	-	-	-	-	-	-	-	-	-	-	-	-	-	-	-	-	-	-	-	-	-	-
		-	-	-	-	-	-	-	-	-	-	-	-	173987	-	-	-	-	-	-	-	-	-	-	-	-	-	-	-	-	-	-	-	-	-	92861	-	-	-	-	-	-	-	-	-	-	-	-	-	-	-	-
		-	-	-	-	-	-	-	-	-	-	-	-	173986	-	-	-	-	-	-	-	-	-	-	-	-	-	-	-	-	-	-	-	-	-	-	-	-	-	-	-	-	-	-	-	-	-	-	-	-	-	-
		-	-	-	-	-	-	-	-	-	-	-	-	173984	-	-	-	-	-	-	-	-	-	-	-	-	-	-	-	-	-	-	-	-	-	-	-	-	-	-	-	-	-	-	-	-	-	-	-	-	-	-
		-	+	-	-	-	-	-	AT3G06290.1	-	BDEG_05219	-	224663	173981	-	-	-	OTTDARP00000021650	-	-	-	-	-	-	-	-	139852	-	-	41021	-	Os07g45160.1	30354	-	-	163375	158285	-	178300	RO3G_13942.1	-	SPCC70.06	GLEAN3_11695	-	-	-	-	-	-	-	-	UM03505.1
		-	+	-	-	-	-	-	AT2G36885.1	63268	-	-	-	173978	-	-	CMT137C	-	-	-	-	-	-	-	-	-	-	-	-	-	-	Os03g04470.1	-	-	37602	156721	-	-	735575	-	-	-	-	-	-	10598	-	-	-	-	-	-
		-	-	-	-	-	-	-	-	-	-	-	-	173976	-	-	-	-	-	-	-	-	-	-	-	-	-	-	-	-	-	-	-	-	-	-	-	-	-	-	-	-	-	-	-	-	-	-	-	-	-	-
		-	-	-	-	-	-	-	-	-	-	-	-	173965	-	-	-	-	-	-	-	-	-	-	-	-	-	-	-	-	-	-	-	-	-	-	-	-	-	-	-	-	-	-	-	-	-	-	-	-	-	-
		-	-	-	-	-	-	-	-	-	-	-	-	173960	-	-	-	-	-	-	-	-	-	-	-	-	-	-	-	-	-	-	-	-	-	-	-	-	-	-	-	-	-	-	-	-	-	-	-	-	-	-
		-	-	-	-	-	-	-	-	-	-	-	-	173947	-	-	-	-	-	-	-	-	-	-	-	-	-	-	-	-	-	-	-	-	-	-	-	-	-	-	-	-	-	-	-	-	-	-	-	-	-	-
		-	-	-	-	-	-	-	-	-	-	-	-	173944	-	-	-	-	-	-	-	-	-	-	-	-	-	-	-	-	-	-	-	-	-	-	-	-	-	-	-	-	-	-	-	-	-	-	-	-	-	-
		-	-	-	-	-	-	-	-	-	-	-	-	173941	-	-	-	-	-	-	-	-	-	-	-	-	-	-	-	-	-	-	-	-	-	-	-	-	-	-	-	-	-	-	-	-	-	-	-	-	-	-
		-	-	-	-	-	-	-	-	-	-	-	-	173935	-	-	-	-	-	-	-	-	-	-	-	-	-	-	-	-	-	-	-	-	-	-	-	-	-	-	-	-	-	-	-	-	-	-	-	-	-	-
		-	-	-	-	-	-	-	-	-	-	-	-	173931	-	-	-	-	-	-	-	-	-	-	-	-	-	-	-	-	-	-	-	-	-	-	-	-	-	-	-	-	-	-	-	-	-	-	-	-	-	-
DCL1	Dicer-like protein	-	+	-	-	-	-	GB30289-PA_2	AT1G01040.1	-	BDEG_03386	CE25057	209996	173928	209576	-	-	OTTDARP00000020903	DDB0168963	CG6493-PA	-	-	ENSGALP00000036777	-	OTTHUMP00000028446	-	164234	-	57506	-	NCU08270	Os03g02970.1	-	GSPATP00033115001	-	86688	158395	-	552372	RO3G_15435.1	YPR204W	-	GLEAN3_25885	SINFRUP00000154659	31.m00232	-	-	-	51673	-	-	-
		-	-	-	-	-	-	-	-	-	-	-	-	173926	-	-	-	-	-	-	-	-	-	-	-	-	-	-	-	-	-	-	-	-	-	-	-	-	-	-	-	-	-	-	-	-	-	-	-	-	-	-
		-	-	-	-	-	-	-	-	-	-	-	-	173925	-	-	-	-	-	-	-	-	-	-	-	-	-	-	-	-	-	-	-	-	-	-	-	-	-	-	-	-	-	-	-	-	-	-	-	-	-	-
		-	-	-	-	-	-	-	-	-	-	-	-	173920	-	-	-	-	-	-	-	-	-	-	-	-	-	-	-	-	-	-	-	-	-	-	-	-	-	-	-	-	-	-	-	-	-	-	-	-	-	-
		-	-	-	-	-	-	-	-	-	-	-	-	173919	-	-	-	-	-	-	-	-	-	-	-	-	-	-	63671	-	-	-	-	-	-	-	-	-	-	-	-	-	-	-	-	-	-	-	-	-	-	-
		-	-	-	-	-	-	-	-	-	-	-	-	173918	-	-	-	-	-	-	-	-	-	-	-	-	-	-	-	-	-	-	-	-	-	-	-	-	-	-	-	-	-	-	-	-	-	-	-	-	-	-
		-	-	-	-	-	-	-	-	-	-	-	-	173915	-	-	-	-	-	-	-	-	-	-	-	-	-	-	-	-	-	-	-	-	-	-	-	-	-	-	-	-	-	-	-	-	-	-	-	-	-	-
		-	-	-	-	-	-	-	-	-	-	-	-	173908	-	-	-	-	-	-	-	-	-	-	-	-	-	-	-	-	-	-	-	-	-	-	-	-	-	-	-	-	-	-	-	-	-	-	-	-	-	-
		+	+	-	-	-	-	-	-	-	BDEG_04827	-	228860	173906	252059	-	-	-	-	-	-	-	-	-	-	-	-	6574	73968	61117	-	-	-	-	-	102141	142269	-	-	-	-	-	-	SINFRUP00000145425	3.m01671	-	-	-	-	85357.m00061	-	-
		-	-	-	-	-	-	-	-	-	-	-	-	173902	-	-	-	-	-	-	-	-	-	-	-	-	-	-	-	-	-	-	-	-	-	-	-	-	-	-	-	-	-	-	-	-	-	-	-	-	-	-
		-	-	-	-	-	-	-	-	-	-	-	-	173901	-	-	-	-	-	-	-	-	-	-	-	-	-	-	-	-	-	-	-	-	-	-	-	-	-	-	-	-	-	-	-	-	-	-	-	-	-	-
		-	-	-	-	-	-	-	-	-	-	-	-	173900	-	-	-	-	-	-	-	-	-	-	-	-	-	-	-	-	-	-	-	-	-	-	-	-	-	-	-	-	-	-	-	-	-	-	-	-	-	-
		-	-	-	-	-	-	-	-	-	-	-	-	173881	-	-	-	-	-	-	-	-	-	-	-	-	-	-	-	-	-	-	-	-	-	-	-	-	-	-	-	-	-	-	-	-	-	-	-	-	-	-
		-	-	-	-	-	-	-	-	-	-	-	-	173878	-	-	-	-	-	-	-	-	-	-	-	-	-	-	-	-	-	-	-	-	-	-	-	-	-	-	-	-	-	-	-	-	-	-	-	-	-	-
		-	-	-	-	-	-	-	-	-	-	-	-	173877	-	-	-	-	-	-	-	-	-	-	-	-	-	-	-	-	-	-	-	-	-	-	-	-	-	-	-	-	-	-	-	-	-	-	-	-	-	-
		-	+	-	-	-	-	-	-	-	-	CE38605	55853	173868	-	-	-	-	DDB0189417	CG7890-PA	-	-	ENSGALP00000008174	-	OTTHUMP00000163902	-	-	-	-	-	-	-	34248	-	-	-	-	-	-	-	-	-	GLEAN3_17899	SINFRUP00000182599	-	-	-	-	-	-	-	-
		-	-	-	-	-	-	-	-	-	-	-	-	173864	-	-	-	-	-	-	-	-	-	-	-	-	-	-	-	-	-	-	-	-	-	-	-	-	-	-	-	-	-	-	-	-	-	-	-	-	-	-
		+	+	-	-	-	-	-	-	-	-	CE00938	102709	173863	375158	-	-	OTTDARP00000007984	-	CG11755-PA	-	-	ENSGALP00000022985	-	OTTHUMP00000028822	-	-	-	-	197701	-	-	-	-	-	-	-	-	-	-	-	-	-	SINFRUP00000173326	-	-	-	-	55296	-	-	-
		-	+	-	-	-	-	GB10132-PA	-	-	-	CE28305	217638	173860	391014	-	-	OTTDARP00000023388	DDB0219887	CG10747-PA	-	-	ENSGALP00000026934	-	OTTHUMP00000195158	-	-	-	48365	183374	-	-	36123	-	48445	-	-	-	-	-	-	-	-	SINFRUP00000128066	-	7138	-	-	-	-	-	-
		-	+	-	-	-	-	GB14150-PA	-	-	BDEG_00606	CE40019	152090	173858	297186	-	-	OTTDARP00000023954	DDB0190640	CG9319-PA	-	-	ENSGALP00000020115	-	OTTHUMP00000115916	-	180274	27495	33626	82280	NCU07298	-	-	GSPATP00017825001	-	-	130125	-	580427	RO3G_06109.1	-	-	GLEAN3_07210	SINFRUP00000143781	141.m00098	262312	-	-	23229	-	-	UM01923.1
CHR3405	SWI/SNF chromatin remodeling complex component	-	-	-	-	-	-	-	-	-	-	-	-	173857	-	-	-	-	-	-	-	-	-	-	-	-	-	-	-	-	-	-	-	-	-	-	-	-	-	-	-	-	-	-	-	-	-	-	-	-	-	-
		-	-	-	-	-	-	-	-	-	-	-	-	173855	-	-	-	-	-	-	-	-	-	-	-	-	-	-	-	-	-	-	-	-	-	-	-	-	-	-	-	-	-	-	-	-	-	-	-	-	-	-
		-	-	-	-	-	-	-	-	-	-	-	-	173850	-	-	-	-	-	-	-	-	-	-	-	-	-	-	-	-	-	-	-	-	-	-	-	-	-	-	-	-	-	-	-	-	-	-	-	-	-	-
		-	-	-	-	-	-	-	-	-	-	-	-	173849	-	-	-	-	-	-	-	-	-	-	-	-	-	-	-	-	-	-	-	-	-	-	-	-	-	-	-	-	-	-	-	-	-	-	-	-	-	-
GLD2-Amino	glucose-6-phosphate dehydrogenase, plastidic, amino portion	-	-	-	-	-	-	-	-	-	-	-	-	173841	-	-	-	-	-	-	-	-	-	-	OTTHUMP00000026040	-	-	-	-	-	-	-	-	-	-	-	-	-	-	-	-	-	GLEAN3_21377	-	-	-	-	-	-	-	-	-
		-	+	+	-	-	-	GB18102-PA	AT4G14000.1	-	BDEG_04074	CE03450	-	173838	281149	-	CMS075C	-	DDB0201783	CG17219-PA	-	6.m00492	-	-	OTTHUMP00000033956	-	139771	32107	3144	126398	-	Os09g19570.1	21084	-	44287	208117	136043	-	586604	-	-	SPAC1071.05	-	SINFRUP00000151557	1.m01023	-	TA05520	583.m05485	-	97193.m00061	Tb09.160.4920	UM02979.1
		-	-	-	-	-	-	-	-	-	-	-	-	173830	-	-	-	-	-	-	-	-	-	-	-	-	-	-	-	-	-	-	-	-	-	-	-	-	-	-	-	-	-	-	-	-	-	-	-	-	-	-
		-	-	-	-	-	-	-	-	-	-	-	-	173828	-	-	-	-	-	-	-	-	-	-	-	-	-	-	-	-	-	-	-	-	-	-	-	-	-	-	-	-	-	-	-	-	-	-	-	-	-	-
		-	-	-	-	-	-	-	-	-	-	-	-	173822	-	-	-	-	-	-	-	-	-	-	-	-	-	-	-	-	-	-	-	-	-	-	-	-	-	-	-	-	-	-	-	-	-	-	-	-	-	-
		-	+	-	-	-	-	-	-	-	-	-	192100	173820	288738	-	-	-	DDB0206296	-	-	-	ENSGALP00000008599	-	NP_055020.1	-	138548	28183	-	-	-	Os10g37640.1	-	GSPATP00005191001	47227	130271	-	PFI0590c	757677	-	-	SPAC4F10.19c	-	SINFRUP00000144932	1.m00834	22739	-	-	-	-	-	-
		-	+	-	-	-	-	-	AT1G34630.1	-	-	-	-	173818	-	-	-	-	-	-	-	-	-	-	-	LmjF18.0540	-	-	-	-	-	Os03g09090.1	-	-	-	199394	-	-	816345	-	-	-	-	-	-	-	-	-	-	-	-	-
		-	-	-	-	-	-	-	-	-	-	-	-	173809	-	-	-	-	-	-	-	-	-	-	-	-	-	-	-	-	-	-	-	-	-	-	-	-	-	-	-	-	-	-	-	-	-	-	-	-	-	-
		-	-	-	-	-	-	-	-	-	-	-	-	173804	-	-	-	-	DDB0219267	-	-	-	-	-	-	-	-	-	79443	-	-	-	-	-	-	-	-	-	-	-	-	-	-	-	-	-	-	35.m00846	-	-	-	-
		-	-	-	-	-	-	-	-	-	-	-	-	173799	-	-	-	-	-	-	-	-	-	-	-	-	-	-	-	-	-	-	-	-	-	-	-	-	-	-	-	-	-	-	-	-	-	-	-	-	-	-
		-	-	-	-	-	-	-	-	-	-	-	-	173788	-	-	-	-	-	-	-	-	-	-	-	-	-	-	-	-	-	-	-	-	-	-	-	-	-	-	-	-	-	-	-	-	-	-	-	-	-	-
		-	-	-	-	-	-	-	-	-	-	-	-	173786	-	-	-	-	-	-	-	-	-	-	-	-	-	-	-	-	-	-	-	-	-	-	-	-	-	-	-	-	-	-	-	-	-	-	-	-	-	-
		-	-	-	+	-	-	-	-	-	-	-	-	173768	-	-	-	-	-	-	-	-	-	-	-	-	-	-	-	-	-	-	-	-	-	-	-	-	-	-	-	-	-	-	-	-	-	-	-	-	-	-
		-	-	-	-	-	-	-	-	-	-	-	-	173758	-	-	-	-	-	-	-	-	-	-	-	-	-	-	-	-	-	-	-	-	-	-	-	-	-	-	-	-	-	-	-	-	-	-	-	-	-	-
		-	-	-	-	-	-	-	-	-	-	-	-	173756	-	-	-	-	DDB0187939	-	-	-	-	-	-	-	-	-	-	-	-	-	-	-	-	-	-	-	-	-	-	-	-	-	-	-	-	-	-	-	-	-
		-	-	-	-	-	-	-	-	-	-	-	-	173751	-	-	-	-	-	-	-	-	-	-	-	-	-	-	-	-	-	-	-	-	-	-	-	-	-	-	-	-	-	-	-	-	-	-	-	-	-	-
		-	+	-	-	-	-	-	-	-	-	CE25481	-	173750	-	-	-	OTTDARP00000009322	-	-	-	-	-	-	OTTHUMP00000171423	-	-	29980	-	198839	-	-	-	-	-	-	-	-	-	-	-	-	-	SINFRUP00000147298	-	-	-	-	-	-	-	-
NAT1	similar to N-acetyl transferase	-	+	+	-	-	-	GB13955-PA	AT1G03150.1	23282	BDEG_00498	CE25639	178459	173749	226858	-	-	-	DDB0166998	CG14222-PA	-	64.m00175	ENSGALP00000020525	-	OTTHUMP00000030374	LmjF25.0020	199122	16627	82883	238091	-	Os03g49230.1	8349	GSPATP00012803001	28723	108609	109420	-	818659	-	-	SPCC16C4.12	-	SINFRUP00000161447	69.m00209	269576	-	-	32129	-	Tb927.3.610	UM04277.1
		-	-	-	-	-	-	-	-	-	-	-	-	173748	-	-	-	-	-	-	-	-	-	-	-	-	-	-	-	-	-	-	-	-	-	-	-	-	-	-	-	-	-	-	-	-	-	-	-	-	-	-
		-	-	-	-	-	-	-	-	-	-	-	-	173744	-	-	-	-	-	-	-	-	-	-	-	-	-	-	-	-	-	-	-	-	-	-	-	-	-	-	-	-	-	-	-	-	-	-	-	-	-	-
		-	-	-	-	-	-	-	-	-	-	-	-	173743	-	-	-	-	-	-	-	-	-	-	-	-	-	-	-	-	-	-	-	-	-	-	-	-	-	-	-	-	-	-	-	-	-	-	-	-	-	-
		-	-	-	-	-	-	-	-	-	-	-	-	173742	-	-	-	-	-	-	-	-	-	-	-	-	-	-	-	-	-	-	-	-	-	-	-	-	-	-	-	-	-	-	-	-	-	-	-	-	-	-
		-	-	-	-	-	-	-	-	-	-	-	-	173740	-	-	-	-	-	-	-	-	-	-	-	-	-	-	-	-	-	-	-	-	-	-	-	-	-	-	-	-	-	-	-	-	-	-	-	-	-	-
		-	-	-	-	-	-	-	-	-	-	-	-	173735	-	-	-	-	-	-	-	-	-	-	-	-	-	-	-	-	-	-	-	-	-	-	-	-	-	-	-	-	-	-	-	-	-	-	-	-	-	-
NGB2	Putative nucleolar GTP-binding protein 2.	-	-	-	-	-	-	-	AT1G80770.1	-	-	-	-	173731	-	-	-	-	-	-	-	-	-	-	-	-	-	-	-	-	-	Os08g06230.1	30254	-	-	-	-	-	411796	-	-	-	-	-	-	-	-	-	-	-	-	-
		-	-	-	-	-	-	-	-	-	-	-	-	173727	-	-	-	-	-	-	-	-	-	-	-	-	-	-	-	-	-	-	-	-	-	-	-	-	-	-	-	-	-	-	-	-	-	-	-	-	-	-
	alpha-amylase like protein	-	-	-	-	-	-	-	-	-	-	-	-	173725	-	-	-	-	-	-	-	-	-	-	-	-	-	-	-	-	-	-	-	-	-	-	-	-	-	-	-	-	-	SINFRUP00000156856	-	-	-	-	-	-	-	-
		-	-	-	-	-	-	-	-	-	-	-	-	173723	-	-	-	-	-	-	-	-	-	-	-	-	-	-	-	-	-	-	-	-	-	-	-	-	-	-	-	-	-	-	-	-	-	-	-	-	-	-
		-	-	-	-	-	-	-	-	-	-	-	-	173712	-	-	-	-	-	-	-	-	-	-	-	-	-	-	-	-	-	-	-	-	-	-	-	-	-	-	-	-	-	-	-	-	-	-	-	-	-	-
SNAPG1	gamma SNAP	-	+	-	-	-	-	GB16415-PA	AT4G20410.1	-	BDEG_01410	-	177891	173710	371638	-	-	OTTDARP00000023584	DDB0191054	CG3988-PA	-	-	ENSGALP00000022539	-	OTTHUMP00000071876	-	184553	36581	77767	245363	-	Os04g52760.1	-	-	-	228189	128551	-	586863	RO3G_08862.1	-	-	-	SINFRUP00000156037	-	-	-	-	55641	-	-	-
		-	-	-	-	-	-	-	-	-	-	-	-	173705	-	-	-	-	-	-	-	-	-	-	-	-	-	-	-	-	-	-	-	-	-	-	-	-	-	-	-	-	-	-	-	-	-	-	-	-	-	-
		-	-	-	-	-	-	-	-	-	-	-	-	173704	-	-	-	-	-	-	-	-	-	-	-	-	-	-	-	-	-	-	-	-	-	-	-	-	-	-	-	-	-	-	-	-	-	-	-	-	-	-
		-	-	-	-	-	-	-	-	-	-	-	-	173696	-	-	CML118C	-	-	-	-	-	-	-	-	-	-	-	-	-	-	-	37825	-	-	-	-	-	-	-	-	-	-	-	-	-	-	-	-	-	-	-
		-	+	-	-	-	-	GB14565-PA	AT5G50310.1	15388	BDEG_00685	CE26914	226649	173694	227375	cgd7_3580	CME001C	-	DDB0204907	CG4069-PA	-	-	ENSGALP00000012743	-	OTTHUMP00000175012	LmjF16.0080	169748	34776	-	202453	NCU09227	Os04g40740.1	1600	-	23798	176143	132086	PF10_0219	252514	-	YPL263C	-	-	SINFRUP00000177965	-	37705	TA10910	44.m02532	-	-	Tb927.5.3900	UM01598.1
		-	-	-	-	-	-	-	-	-	-	-	-	173692	-	-	-	-	-	-	-	-	-	-	-	-	-	-	-	-	-	-	-	-	-	-	-	-	-	-	-	-	-	-	-	-	-	-	-	-	-	-
		-	-	-	-	-	-	-	-	-	-	-	-	173688	-	-	-	-	-	-	-	-	-	-	-	-	-	-	-	-	-	-	-	-	-	-	-	-	-	-	-	-	-	-	-	-	-	-	-	-	-	-
		-	-	-	-	-	-	-	-	-	-	-	-	173685	-	-	-	-	-	-	-	-	-	-	-	-	-	-	-	-	-	-	-	-	-	-	-	-	-	-	-	-	-	-	-	-	-	-	-	-	-	-
		-	-	-	-	-	-	-	-	-	-	-	-	173683	-	-	-	-	-	-	-	-	-	-	-	-	-	-	-	-	-	-	-	-	-	-	-	-	-	-	-	-	-	-	-	-	-	-	-	-	-	-
		-	-	-	-	-	-	-	-	-	-	-	-	173682	-	-	-	-	-	-	-	-	-	-	-	-	-	-	-	-	-	-	-	-	-	-	-	-	-	-	-	-	-	-	-	-	-	-	-	-	-	-
HEL1	ATP-dependent DNA helicase. Probably mitochondrial.	-	+	-	-	-	-	GB13205-PA	AT1G20720.1	62227	BDEG_05203	CE17764	219962	173679	388748	cgd6_2660	CMQ261C	OTTDARP00000023653	DDB0187049	CG4078-PA	19068670	167.m00119	ENSGALP00000008452	GL50803_5631	NP_114432.1	LmjF29.2660	144987	27129	34204	30571	-	Os09g37920.1	34138	GSPATP00016196001	24915	81444	142420	PF14_0081	550758	RO3G_00824.1	-	-	GLEAN3_03472	SINFRUP00000139789	104.m00094	32522	TA09995	25.m01889	30066	95674.m00220	Tb927.3.3090	-
		-	-	-	-	-	-	-	-	-	-	-	-	173669	-	-	-	-	-	-	-	-	-	-	-	-	-	-	-	-	-	-	-	-	-	-	-	-	-	-	-	-	-	-	-	-	-	-	-	-	-	-
		-	-	-	-	-	-	-	-	-	-	-	-	173667	-	-	-	-	-	-	-	-	-	-	-	-	-	-	-	-	-	-	-	-	-	-	-	-	-	-	-	-	-	-	-	23334	-	-	-	-	-	-
		-	+	-	-	-	-	-	AT5G04490.1	-	-	-	-	173650	-	-	CMR252C	-	DDB0216976	-	-	189.m00100	-	-	-	-	-	-	-	-	-	Os01g61560.1	33713	-	34441	16953	-	-	228270	-	-	-	-	-	-	22177	-	-	-	-	-	-
		-	-	-	-	-	-	-	-	-	-	-	-	173648	-	-	-	-	-	-	-	-	-	-	-	-	-	-	-	-	-	-	-	-	-	-	-	-	-	-	-	-	-	-	-	-	-	-	-	-	-	-
		-	-	-	-	-	-	-	-	-	-	-	-	173643	-	-	-	-	-	-	-	-	-	-	-	-	-	-	-	-	-	-	-	-	-	-	-	-	-	-	-	-	-	-	-	-	-	-	-	-	-	-
		-	+	-	-	-	-	GB17791-PA	AT4G32930.1	-	BDEG_01722	CE26972	183158	173642	297180	cgd3_1630	-	OTTDARP00000019708	DDB0190870	CG4646-PA	-	-	ENSGALP00000017361	-	OTTHUMP00000010492	LmjF12.0410	192905	-	73257	245604	NCU08505	Os01g38480.1	18371	GSPATP00020932001	45958	210689	130822	MAL13P1.257	819516	RO3G_10270.1	YCR090C	SPBC2D10.03c	-	-	188.m00080	-	TA14715	80.m00075	57890	-	Tb927.1.3390	UM05492.1
		-	-	-	-	-	-	-	-	-	-	-	-	173638	-	-	-	-	-	-	-	-	-	-	-	-	-	-	-	-	-	Os03g58540.2	4848	-	-	234170	-	-	-	-	-	-	-	-	-	-	-	583.m05726	-	-	-	-
		-	-	-	-	-	-	-	-	-	BDEG_02061	-	-	173633	-	-	-	-	-	-	-	-	-	-	OTTHUMP00000159283	-	-	-	-	-	-	-	-	-	-	-	-	-	-	-	-	-	-	-	-	-	-	-	-	-	-	-
FAP36	Flagellar Associated Protein	+	+	-	-	-	-	-	-	66223	-	CE29094	227946	173632	222495	-	-	-	-	-	-	-	ENSGALP00000013078	-	NP_542398.3	LmjF36.1400	155072	-	78620	212454	-	-	-	GSPATP00002898001	-	98562	140404	-	-	-	-	-	-	SINFRUP00000129310	5.m00322	-	-	-	-	-	Tb10.70.1130	-
MITC16	mitochondrial substrate carrier	-	-	-	-	-	-	-	-	-	-	-	-	173626	-	-	-	-	-	-	-	-	-	-	-	-	-	-	-	-	-	-	-	-	-	-	-	-	-	-	-	-	-	-	-	-	-	-	-	-	-	-

		-	-	-	-	-	-	-	-	-	-	-	-	173620	-	-	-	-	-	-	-	-	-	-	-	-	-	-	-	-	-	-	-	-	-	-	-	-	-	-	-	-	-	-	-	-	-	-	-	-	-	-
		-	-	-	-	-	-	-	-	-	-	-	-	173617	-	-	-	-	-	-	-	-	-	-	-	-	-	-	-	-	-	-	-	-	-	-	-	-	-	-	-	-	-	-	-	-	-	-	-	-	-	-
MAPKKK2	MAP Kinase Kinase Kinase Homolog 2, putative	-	-	-	-	-	-	-	-	-	-	-	-	173608	-	-	-	-	-	-	-	-	-	-	-	-	-	-	-	-	-	-	-	-	-	-	-	-	-	-	-	-	-	-	-	-	-	-	-	-	-	-
		-	-	-	-	-	-	-	-	-	-	-	-	173607	-	-	-	-	-	-	-	-	-	-	-	-	-	-	-	-	-	-	11681	-	-	-	-	-	-	-	-	-	-	-	-	-	-	-	-	-	-	-
		-	+	-	-	-	-	-	AT3G19840.1	-	-	-	-	173600	-	-	-	-	-	-	-	-	-	-	-	-	-	-	-	-	-	Os10g34380.1	-	-	-	64282	-	-	-	-	-	-	-	-	-	22716	-	-	10795	-	-	-
		-	-	-	-	-	-	-	-	-	-	-	-	173594	-	-	-	-	-	-	-	-	-	-	-	-	-	-	-	-	-	-	-	-	-	-	-	-	-	-	-	-	-	-	-	-	-	-	-	-	-	-
		-	-	-	-	-	-	-	-	-	-	-	-	173593	-	-	-	-	-	-	-	-	-	-	-	-	-	-	-	240129	-	Os02g32930.1	-	-	-	-	-	-	-	-	-	-	-	-	-	-	-	-	-	-	-	-
		-	-	-	-	-	-	-	-	-	-	-	-	173588	-	-	-	-	-	-	-	-	-	-	-	-	-	-	-	-	-	-	-	-	-	-	-	-	-	-	-	-	-	-	-	-	-	-	-	-	-	-
		-	-	-	-	-	-	-	-	-	-	-	-	173585	-	-	-	-	-	-	-	-	-	-	-	-	-	-	-	-	-	-	-	-	-	-	-	-	-	-	-	-	-	-	-	-	-	-	-	-	-	-
		-	-	-	-	-	-	-	-	-	-	-	-	173584	-	-	-	-	-	-	-	-	-	-	-	-	-	-	-	-	-	-	-	-	-	-	-	-	-	-	-	-	-	-	-	-	-	-	-	-	-	-
		-	+	-	-	-	-	-	-	-	BDEG_07123	-	222016	173581	210610	-	-	-	-	-	-	337.m00050	ENSGALP00000010301	-	OTTHUMP00000025276	LmjF35.2520	161737	-	72718	217045	-	-	-	GSPATP00025480001	-	65158	-	-	-	-	YPR093C	-	-	SINFRUP00000137531	3.m01777	-	-	-	20747	85335.m00175	Tb09.211.4150	-
		-	-	-	-	-	-	-	-	-	-	-	-	173579	-	-	-	-	-	-	-	-	-	-	-	-	-	-	-	-	-	-	-	-	-	-	-	-	-	-	-	-	-	-	-	-	-	-	-	-	-	-
		-	+	-	-	-	-	-	-	26419	-	-	227475	173570	-	-	-	-	-	CG3251-PA	-	-	-	-	-	LmjF35.1390	53774	-	-	109460	-	-	-	-	-	-	-	PFI1135c	-	-	-	-	-	-	-	-	-	55.m00099	-	-	Tb927.8.5050	-
		-	-	-	-	-	-	-	-	-	-	-	-	173568	-	-	-	-	-	-	-	-	-	-	-	-	-	-	-	-	-	-	-	-	-	-	-	-	-	-	-	-	-	-	-	-	-	-	-	-	-	-
		-	-	-	-	-	-	-	-	-	-	-	-	173556	-	-	-	-	-	-	-	-	-	-	-	-	-	-	-	-	-	-	-	-	-	-	-	-	-	-	-	-	-	-	-	-	-	-	-	-	-	-
		-	-	-	-	-	-	-	-	-	-	-	-	173555	-	-	-	-	-	-	-	-	-	-	-	-	-	-	-	-	-	-	-	-	-	-	-	-	-	-	-	-	-	-	-	-	-	-	-	-	-	-
		-	-	-	-	-	-	-	-	-	-	-	-	173552	-	-	-	-	-	-	-	-	-	-	-	-	-	-	3976	-	-	-	-	-	-	-	144530	-	-	-	-	-	-	-	-	-	-	-	-	-	-	-
		-	-	-	-	-	-	-	-	-	-	-	-	173551	-	-	-	-	-	-	-	-	-	-	-	-	-	-	-	-	-	-	-	-	-	-	-	-	-	-	-	-	-	-	-	-	-	-	-	-	-	-
		-	-	-	-	-	-	-	-	-	-	-	-	173550	-	-	-	-	-	-	-	-	-	-	-	-	-	-	-	-	-	-	-	-	-	-	-	-	-	-	-	-	-	-	-	-	-	-	-	-	-	-
		-	+	-	-	-	-	-	AT1G18030.2	-	-	-	169445	173547	204554	-	-	-	-	-	-	-	ENSGALP00000010092	-	OTTHUMP00000164472	-	-	36991	-	110119	-	Os09g14540.1	-	-	-	-	-	-	733020	-	-	-	-	SINFRUP00000151530	-	-	-	583.m05384	26627	-	-	-
		-	-	-	-	-	-	-	-	-	-	-	-	173545	-	-	-	-	-	-	-	-	-	-	-	-	-	-	-	-	-	-	-	-	-	-	-	-	-	-	-	-	-	-	-	-	-	-	-	-	-	-
		-	-	-	-	-	-	-	-	-	-	-	-	173543	-	-	-	-	-	-	-	-	-	-	-	-	-	-	-	-	-	-	-	-	-	-	-	-	-	-	-	-	-	-	-	-	-	-	-	-	-	-
CHR3409		-	-	-	-	-	-	-	-	36628	-	-	-	173539	-	-	-	-	-	-	-	-	-	-	-	-	-	-	-	-	-	-	-	-	-	-	-	-	-	-	-	-	-	-	-	-	-	-	-	-	-	-
		-	-	-	-	-	-	-	-	-	-	-	-	173533	-	-	-	-	-	-	-	-	-	-	-	-	-	-	-	-	-	-	-	-	-	-	-	-	-	-	-	-	-	-	-	-	-	-	-	-	-	-
		-	+	-	-	-	-	-	AT5G39940.1	11765	-	-	-	173528	-	-	CMQ128C	-	-	-	-	-	-	-	-	-	-	-	63696	-	-	-	33046	-	14937	130421	-	-	199306	-	-	-	-	-	-	35087	-	-	-	-	-	-
		-	-	-	-	-	-	-	-	-	-	-	-	173527	-	-	-	-	-	-	-	-	-	-	-	-	-	-	-	-	-	-	-	-	-	-	-	-	-	-	-	-	-	-	-	-	-	-	-	-	-	-
AOF1	related to amine oxidase, flavin-containing	-	+	-	-	-	-	-	-	63162	-	-	-	173525	-	-	-	-	DDB0188339	-	-	-	-	-	-	-	-	-	55992	213942	-	-	32082	-	46399	65133	130325	-	-	-	-	-	GLEAN3_10627	-	27.m00386	-	-	-	-	-	-	UM05423.1
		-	-	-	-	-	-	-	-	-	-	-	-	173524	-	-	-	-	-	-	-	-	-	-	-	-	-	-	-	-	-	-	6841	GSPATP00034309001	-	-	-	-	-	-	-	-	-	-	-	-	-	-	-	-	-	-
		-	-	-	-	-	-	-	-	-	-	-	-	173519	-	cgd7_2320	-	-	-	-	-	-	-	-	-	-	-	-	-	-	-	-	5986	-	-	-	-	-	-	-	-	-	-	-	-	-	-	59.m03597	-	-	-	-
		-	-	-	-	-	-	-	-	-	-	-	-	173507	-	-	-	-	-	-	-	-	-	-	-	-	-	-	-	-	-	-	-	-	-	-	-	-	-	-	-	-	-	-	-	-	-	-	-	-	-	-
		-	-	-	-	-	-	-	-	-	-	-	-	173496	-	-	-	-	-	-	-	-	-	-	-	-	-	-	-	-	-	-	-	-	-	-	-	-	-	-	-	-	-	-	-	-	-	-	-	-	-	-
		-	-	-	-	-	-	GB18982-PA	-	-	-	-	-	173475	-	-	-	-	-	-	-	-	-	-	-	-	-	-	-	-	-	-	-	-	-	-	-	-	-	-	-	-	-	-	-	-	-	-	-	-	-	-
		-	+	-	-	-	-	GB15248-PA	-	-	-	CE03169	-	173472	-	-	CMD063C	-	-	-	-	-	ENSGALP00000013663	-	-	-	236999	-	-	-	-	-	-	-	-	-	-	-	-	-	-	-	-	SINFRUP00000127936	-	-	-	-	-	-	-	-
		-	+	-	-	-	-	-	AT3G57790.1	26417	-	-	-	173470	-	-	-	-	-	-	-	-	-	-	-	-	-	8397	-	-	-	Os09g31270.1	-	-	16052	199574	-	-	255709	-	-	-	-	-	-	-	-	-	-	-	-	-
		-	-	-	-	-	-	-	-	-	-	-	-	173443	-	-	-	-	-	-	-	-	-	-	-	-	-	-	-	-	-	-	-	-	-	-	-	-	-	-	-	-	-	-	-	-	-	-	-	-	-	-
		-	+	-	-	-	-	GB15332-PA	AT5G39770.1	-	BDEG_03087	-	101818	173442	271909	-	CMI043C	-	-	CG3026-PA	19068700	-	-	-	NP_079404.3	-	115731	22361	48155	20603	NCU07457	Os01g71960.1	-	GSPATP00005346001	-	176090	-	-	758112	-	YDR386W	SPCC4G3.05c	-	SINFRUP00000158181	87.m00255	-	-	55.m04944	-	-	-	UM04630.1
		-	+	-	-	-	-	-	AT5G57860.2	-	BDEG_00263	-	-	173432	-	-	-	OTTDARP00000020405	DDB0169101	-	-	-	-	-	-	-	-	-	-	-	-	Os03g24920.1	-	-	-	192019	143812	-	561357	-	-	-	-	SINFRUP00000180183	-	-	-	-	59247	-	-	-
		-	-	-	-	-	-	-	-	-	-	-	-	173428	-	-	-	-	-	-	-	-	-	-	-	-	-	-	-	-	-	-	-	-	-	-	-	-	-	-	-	-	-	-	-	-	-	-	-	-	-	-
		-	-	-	-	-	-	-	-	-	-	-	-	173421	-	-	-	-	-	-	-	-	-	-	-	-	-	-	-	-	-	-	-	-	-	-	-	-	-	-	-	-	-	-	-	-	-	-	-	-	-	-
		-	-	-	-	-	-	-	-	-	-	-	-	173420	-	-	-	-	-	-	-	-	-	-	-	-	-	-	-	-	-	-	-	-	-	-	-	-	-	-	-	-	-	-	-	-	-	-	-	-	-	-
		+	+	-	-	-	-	GB19445-PA	-	28825	-	CE27805	146497	173414	211575	-	-	OTTDARP00000020429	-	CG3536-PA	-	-	ENSGALP00000018034	-	OTTHUMP00000041920	-	111466	30002	68977	21809	-	-	20710	-	-	-	142935	-	-	-	-	-	-	SINFRUP00000150512	88.m00109	-	-	-	-	85241.m00230	-	-
		-	-	-	-	-	-	-	-	-	-	-	-	173413	-	-	-	-	-	-	-	-	-	-	-	-	-	-	-	-	-	-	-	-	-	-	-	-	-	-	-	-	-	-	-	-	-	-	-	-	-	-
		-	-	-	-	-	-	-	-	-	-	-	-	173408	-	-	-	-	-	-	-	-	-	-	-	-	-	-	-	-	-	-	-	-	-	-	-	-	-	-	-	-	-	-	-	-	-	-	-	-	-	-
		-	+	-	-	-	-	-	-	66310	-	-	-	173402	-	-	-	-	-	-	-	-	-	-	-	-	-	29429	-	91046	NCU08642	-	-	-	-	-	-	-	-	-	-	-	-	-	-	-	-	-	55030	-	-	-
		-	-	-	-	-	-	-	-	-	-	-	-	173386	-	-	-	-	-	-	-	-	-	-	-	-	-	-	-	-	-	-	-	-	-	-	-	-	-	-	-	-	-	-	-	-	-	-	-	-	-	-
		-	-	-	-	-	-	-	-	-	-	-	-	173378	-	-	-	-	-	-	-	-	-	-	-	-	-	-	-	-	-	-	-	-	-	-	-	-	-	-	-	-	-	-	-	-	-	-	-	-	-	-
		-	-	-	-	-	-	-	-	-	-	-	-	173367	-	-	-	-	-	-	-	-	-	-	-	-	-	-	-	-	-	-	-	-	-	-	-	-	-	-	-	-	-	-	-	-	-	-	-	-	-	-
REV1	Deoxycytidyl transferase	-	+	-	-	-	-	GB12986-PA	AT5G44750.1	68491	-	CE01721	166412	173359	292082	-	CMS282C	-	-	CG12189-PA	-	59.m00189	ENSGALP00000027018	-	OTTHUMP00000161215	LmjF36.0100	170636	27193	67675	30213	NCU02053	Os06g47580.3	19527	-	49385	-	145400	-	767640	-	YOR346W	SPBC1347.01c	GLEAN3_15889	SINFRUP00000163834	-	268460	-	46.m01755	53871	-	Tb10.70.2740	UM02220.1
		-	-	-	-	-	-	-	-	-	-	-	-	173338	-	-	-	-	-	-	-	-	-	-	-	-	-	-	-	240124	-	-	-	-	-	140074	-	-	-	-	-	-	-	-	-	-	-	-	-	-	-	-
		-	+	-	-	-	-	GB11372-PA	AT5G42850.2	-	-	CE27323	-	173335	395089	-	-	-	-	CG11024-PA	-	-	ENSGALP00000038086	-	OTTHUMP00000182762	-	214285	-	-	165587	-	-	-	-	-	151861	157878	-	548701	RO3G_05788.1	-	-	-	-	-	-	-	-	61314	-	-	UM02768.1
		-	-	-	-	-	-	-	-	-	-	-	-	173331	-	-	-	-	-	-	-	-	-	-	-	-	-	-	-	-	-	-	-	-	-	-	-	-	-	-	-	-	-	-	-	-	-	-	-	-	-	-
		-	-	-	-	-	-	-	-	-	-	-	-	173323	-	-	-	-	-	-	-	-	-	-	-	-	-	-	-	-	-	-	-	-	-	-	-	-	-	-	-	-	-	-	-	-	-	-	-	-	-	-
		-	-	-	-	-	-	-	-	-	-	-	-	173322	-	-	-	-	DDB0218703	-	-	-	-	-	NP_067049.2	-	-	-	-	-	-	-	-	-	-	-	-	-	-	-	-	-	-	SINFRUP00000149004	-	-	-	-	-	-	-	-
		-	-	-	-	-	-	-	-	-	-	-	-	173319	-	-	-	-	-	-	-	-	-	-	-	-	-	-	-	-	-	-	-	-	-	-	-	-	-	-	-	-	-	-	-	-	-	-	-	-	-	-
		-	+	-	-	-	-	-	AT5G47790.1	-	-	CE34246	180917	173312	-	-	-	OTTDARP00000023063	-	CG8980-PA	-	-	ENSGALP00000001030	-	OTTHUMP00000003849	-	102248	-	-	239278	-	Os08g23680.3	-	-	43291	128445	-	-	269560	-	-	-	GLEAN3_26145	SINFRUP00000133292	13.m00354	-	-	42.m03600	58109	-	-	-
		-	-	-	-	-	-	-	-	-	-	-	-	173311	-	-	-	-	-	-	-	-	-	-	-	-	-	-	-	-	-	-	-	-	-	-	-	-	-	-	-	-	-	-	-	-	-	-	-	-	-	-
		-	-	-	-	-	-	-	-	-	-	-	-	173298	-	-	-	-	-	-	-	-	-	-	-	-	-	-	-	-	-	-	-	-	-	-	-	-	-	-	-	-	-	-	-	-	-	-	-	-	-	-
		-	-	-	+	-	-	-	-	-	-	-	-	173297	-	-	-	-	-	-	-	-	-	-	-	-	-	-	-	-	-	-	-	-	-	-	-	-	-	-	-	-	-	-	-	-	-	-	-	-	-	-
VPS9	involved in vacuolar protein transport	-	+	+	-	-	-	GB12354-PA	AT3G19770.1	17718	BDEG_06246	CE38712	170742	173295	384303	cgd8_1880	-	OTTDARP00000023337	DDB0218662	CG9139-PA	-	70.m00164	ENSGALP00000001464	-	NP_056450.2	LmjF36.4660	130207	-	51184	183590	NCU07301	Os03g15650.1	36941	GSPATP00026989001	43641	181478	138309	MAL8P1.82	209688	-	YML097C	SPBC4F6.10	-	SINFRUP00000136262	1.m00854	-	TA14470	44.m02581	20735	84160.m00148	Tb10.6k15.0260	UM05369.1
		-	+	-	-	-	-	GB14636-PA	-	-	-	-	165096	173291	-	-	-	-	-	CG1764-PA	-	-	-	-	OTTHUMP00000012304	LmjF08.1225	119867	15669	-	-	-	-	-	-	-	170447	-	-	-	RO3G_05016.1	-	-	-	SINFRUP00000139677	-	264274	-	-	21132	-	-	-
		-	+	-	-	-	-	GB12324-PA	AT4G24270.2	-	BDEG_03531	CE05167	222846	173284	250187	-	-	OTTDARP00000016317	DDB0191602	-	-	-	ENSGALP00000038101	-	OTTHUMP00000169100	-	188648	-	-	189386	-	Os08g02130.2	10689	-	-	135986	-	-	596792	RO3G_17145.1	-	SPBC1861.04c	GLEAN3_23990	SINFRUP00000155115	-	-	-	-	62320	-	-	-
FEA2	Fe-assimilating protein 2	-	-	-	-	-	-	-	-	-	-	-	-	173281	-	-	-	-	-	-	-	-	-	-	-	-	-	-	-	-	-	-	-	-	-	-	-	-	-	-	-	-	-	-	-	-	-	-	-	-	-	-
TSF22	unknown function, mitochondrial localization assumed	-	-	-	-	-	-	-	AT3G07570.1	-	-	-	-	173277	-	-	-	-	-	-	-	-	-	-	-	-	-	-	-	-	-	-	12308	-	-	-	-	-	732561	-	-	-	-	-	-	-	-	-	-	-	-	-
	Hypothetical Protein	-	+	-	-	-	-	-	AT3G14910.1	-	-	-	-	173276	-	-	-	-	-	-	-	-	-	-	-	-	-	-	-	-	-	-	-	-	-	182914	136314	-	746463	-	-	-	-	-	-	-	-	-	61281	-	-	-
MAPKKK9	MAPKKK9; Mitogen Activated Protein Kinase Kinase Kinase Homolog 9	-	-	-	-	-	-	-	-	-	-	-	-	173267	-	-	-	-	-	-	-	-	-	-	-	-	-	-	-	-	-	-	-	-	-	-	-	-	-	-	-	-	-	-	-	-	-	-	-	-	-	-
		-	-	-	-	-	-	-	-	-	-	-	-	173263	-	-	-	-	-	-	-	-	-	-	-	-	-	-	-	-	-	-	-	-	-	-	-	-	-	-	-	-	-	-	-	-	-	-	-	-	-	-
		-	-	-	-	-	-	-	-	-	-	-	-	173262	-	-	-	-	-	-	-	-	-	-	-	-	-	-	-	-	-	-	-	-	-	-	-	-	-	-	-	-	-	-	-	-	-	-	-	-	-	-
		-	-	-	-	-	-	-	-	-	-	-	-	173261	-	-	-	-	-	-	-	-	-	-	-	-	-	-	-	-	-	-	-	-	-	-	-	-	-	-	-	-	-	-	-	-	-	-	-	-	-	-
		-	-	-	-	-	-	-	-	-	-	-	-	173255	-	-	-	-	-	-	-	-	-	-	-	-	-	-	-	-	-	-	-	-	-	-	-	-	-	-	-	-	-	-	-	-	-	-	-	-	-	-
		+	+	-	-	-	-	GB17016-PA	-	-	BDEG_06046	-	182879	173252	274858	-	-	-	-	-	-	-	ENSGALP00000039489	-	OTTHUMP00000159076	-	235090	32939	-	93761	-	-	-	-	-	-	-	-	-	-	-	-	-	SINFRUP00000158758	-	-	-	-	20400	-	-	-
		-	-	-	-	-	-	-	-	-	-	-	-	173251	-	-	-	-	-	-	-	-	-	-	-	-	-	-	-	-	-	-	-	-	-	-	-	-	-	-	-	-	-	-	-	-	-	-	-	-	-	-
		-	-	-	-	-	-	-	-	-	-	-	-	173248	-	-	-	-	-	-	-	-	-	-	-	-	-	-	-	-	-	-	-	-	-	-	-	-	-	-	-	-	-	-	-	-	-	-	-	-	-	-
		-	-	-	-	-	-	-	-	-	-	-	-	173246	-	-	-	-	-	-	-	-	-	-	-	-	-	-	-	-	-	-	-	-	-	-	-	-	-	-	-	-	-	-	-	-	-	-	-	-	-	-
		-	-	-	-	-	-	-	-	-	-	-	-	173242	-	-	-	-	-	-	-	-	-	-	-	-	-	-	-	-	-	-	-	-	-	-	-	-	-	-	-	-	-	-	-	-	-	-	-	-	-	-
		-	-	-	-	-	-	-	-	-	-	-	-	173231	-	-	-	-	-	-	-	-	-	-	-	-	-	-	-	-	-	-	-	-	-	-	-	-	-	-	-	-	-	-	-	-	-	-	-	-	-	-
		-	-	-	-	-	-	-	-	-	-	-	-	173222	-	-	-	-	-	-	-	-	-	-	-	-	-	-	-	-	-	-	-	-	-	-	-	-	-	-	-	-	-	-	-	-	-	-	-	-	-	-
		-	+	-	-	-	-	-	AT1G24310.1	-	-	-	154507	173216	272199	-	-	-	-	-	-	-	-	-	OTTHUMP00000160589	-	-	-	74425	165839	-	Os07g19494.1	30510	-	-	138242	133144	-	726047	RO3G_02729.1	-	-	-	-	-	22359	-	541.m01245	-	-	-	-
		-	-	-	-	-	-	-	-	-	-	-	-	173213	-	-	-	-	-	-	-	-	-	-	-	-	-	-	-	-	-	-	-	-	-	-	-	-	-	-	-	-	-	-	-	-	-	-	-	-	-	-
		-	+	-	-	-	-	-	AT1G23890.1	62887	-	CE07924	90967	173207	290077	-	-	-	-	-	-	-	-	GL50803_17193	-	-	114344	33840	64360	200190	-	Os06g45720.1	9458	-	-	-	-	-	552955	-	-	-	GLEAN3_01721	-	-	1080	-	-	-	-	-	-
		-	-	-	-	-	-	-	-	-	-	-	-	173174	-	-	-	-	-	-	-	-	-	-	-	-	-	-	-	-	-	-	-	-	-	-	-	-	-	-	-	-	-	-	-	-	-	-	-	-	-	-
		-	-	-	-	-	-	-	-	-	-	-	-	173173	-	-	-	-	-	-	-	-	-	-	-	-	-	-	-	-	-	-	-	-	-	-	-	-	-	-	-	-	-	-	-	-	-	-	-	-	-	-
		-	-	-	-	-	-	-	-	-	-	-	-	173172	-	-	-	-	-	-	-	-	-	-	-	-	-	-	-	-	-	-	-	-	-	-	-	-	-	-	-	-	-	-	-	-	-	-	-	-	-	-
	hypothetical protein	-	-	-	-	-	-	-	-	-	-	-	-	173170	-	-	-	-	-	-	-	-	-	-	-	-	-	-	-	-	-	-	-	-	-	-	-	-	-	-	-	-	-	-	-	-	-	-	-	-	-	-
		-	-	-	-	-	-	-	-	-	-	-	-	173168	-	-	-	-	-	-	-	-	-	-	-	-	-	-	-	-	-	-	-	-	-	-	-	-	-	-	-	-	-	-	-	-	-	-	-	-	-	-
		-	-	-	-	-	-	-	-	-	-	-	-	173167	-	-	-	-	-	-	-	-	-	-	-	-	-	-	-	214543	-	-	-	-	-	-	-	-	-	-	-	-	-	-	-	-	-	-	-	-	-	-
		-	-	-	-	-	-	-	-	-	-	-	-	173157	-	-	-	-	-	-	-	-	-	-	-	-	-	-	-	-	-	-	-	-	-	-	-	-	-	-	-	-	-	-	-	-	-	-	-	-	-	-
		-	-	-	-	-	-	-	-	-	-	-	-	173156	-	-	-	-	-	-	-	-	-	-	-	-	-	-	-	-	-	-	-	-	-	-	-	-	-	-	-	-	-	-	-	-	-	-	-	-	-	-
		-	-	-	-	-	-	-	-	-	-	-	-	173152	-	-	-	-	-	-	-	-	-	-	-	-	-	-	-	-	-	-	-	-	-	-	-	-	-	-	-	-	-	-	-	-	-	-	-	-	-	-
	HesB/MoeB/ThiF family protein	-	-	-	-	-	-	-	-	-	-	-	-	173148	-	-	-	-	-	-	-	-	-	-	-	-	-	-	-	-	-	-	-	-	-	-	-	-	-	-	-	-	-	-	-	-	-	-	-	-	-	-
		-	-	-	-	-	-	-	-	-	-	-	-	173146	-	-	-	-	-	-	-	-	-	-	-	-	-	-	-	-	-	-	-	-	-	-	-	-	-	-	-	-	-	-	-	-	-	-	-	-	-	-
CPR1	related to NADPH--cytochrome P450 reductase	-	+	-	-	-	-	-	AT1G75200.1	21732	-	-	180119	173136	249935	-	-	-	-	-	-	-	ENSGALP00000001664	-	OTTHUMP00000024549	LmjF05.0750	135774	14102	-	163128	NCU06633	Os03g56390.1	16137	-	21682	141934	108818	PFE1240w	412724	RO3G_16246.1	YPL207W	SPCC1020.08	GLEAN3_06162	SINFRUP00000177458	-	13378	TA18985	583.m05720	28630	-	Tb927.7.7130	-
		-	-	-	-	-	-	-	-	-	-	-	-	173135	-	-	-	-	-	-	-	-	-	-	-	-	-	-	-	-	-	-	-	-	-	-	-	-	-	-	-	-	-	-	-	-	-	-	-	-	-	-
		-	-	-	-	-	-	-	-	-	-	-	-	173133	-	-	-	-	-	-	-	-	-	-	-	-	-	-	-	-	-	-	-	-	-	-	-	-	-	-	-	-	-	-	-	-	-	-	-	-	-	-
		-	-	-	-	-	-	-	-	-	-	-	-	173122	-	-	-	-	-	-	-	-	-	-	-	-	-	-	-	-	-	-	-	-	-	-	-	-	-	-	-	-	-	-	-	-	-	-	-	-	-	-
		-	-	-	-	-	-	-	-	-	-	-	-	173118	-	-	-	-	-	-	-	-	-	-	-	-	-	-	-	-	-	-	-	-	-	-	-	-	-	-	-	-	-	-	-	-	-	-	-	-	-	-
		-	-	-	-	-	-	-	-	-	-	-	-	173117	-	-	-	-	-	-	-	-	-	-	-	-	-	-	-	-	-	-	-	-	-	-	-	-	-	-	-	-	-	-	-	-	-	-	-	-	-	-
RRP46		-	+	-	-	-	-	GB12618-PA	AT3G46210.5	-	BDEG_02463	CE02146	152902	173111	385951	cgd4_1950	-	OTTDARP00000015153	DDB0185805	CG4043-PA	19069278	-	-	-	OTTHUMP00000068577	-	110121	22822	32409	99151	NCU03268	Os03g63720.1	31660	-	-	192949	-	-	260656	RO3G_13498.1	YGR095C	SPBC115.01c	-	SINFRUP00000156655	-	-	-	-	-	-	-	UM03145.1
		-	-	-	-	-	-	-	AT3G53440.2	-	-	-	-	173109	-	-	-	-	-	-	-	-	-	-	-	-	-	-	-	-	-	-	-	-	-	-	135765	-	785511	-	-	-	-	-	-	-	-	-	-	-	-	-
		-	-	-	-	-	-	-	-	-	-	-	-	173094	-	-	-	-	-	-	-	-	-	-	-	-	-	-	-	-	-	-	-	-	-	-	-	-	-	-	-	-	-	-	-	-	-	-	-	-	-	-
		-	-	-	-	-	-	-	-	-	-	-	-	173092	-	-	-	-	-	-	-	-	-	-	-	-	109275	-	-	197133	-	-	-	-	-	-	-	-	-	-	-	-	-	-	-	-	-	-	-	-	-	-
		-	+	-	-	-	-	GB17534-PA	AT2G44950.1	-	-	CE37396	-	173081	-	cgd2_880	-	OTTDARP00000002018	DDB0167764	-	19171256	-	-	-	NP_055586.1	-	223318	38442	-	247601	-	Os04g46450.1	-	-	-	152947	157439	PFF0165c	830340	-	YDL074C	SPCC970.10c	-	SINFRUP00000146146	-	-	TA11615	35.m01589	-	85889.m00433	-	UM05186.1
		-	-	-	-	-	-	-	-	-	-	-	-	173078	-	-	-	-	-	-	-	-	-	-	-	-	-	-	-	-	-	-	-	-	-	-	-	-	-	-	-	-	-	-	-	-	-	-	-	-	-	-
		-	-	-	-	-	-	-	-	-	-	-	-	173067	-	-	-	-	-	-	-	-	-	-	-	-	-	-	-	-	-	-	-	-	-	-	-	-	-	-	-	-	-	-	-	-	-	-	-	-	-	-
		-	-	-	-	-	-	-	-	-	-	-	-	173066	-	-	-	-	-	-	-	-	-	-	-	-	-	-	-	-	-	-	-	-	-	-	-	-	-	-	-	-	-	-	-	-	-	-	-	-	-	-
		-	-	-	-	-	-	-	-	-	-	-	-	173056	-	-	-	-	-	-	-	-	-	-	-	-	-	-	-	-	-	-	-	-	-	-	-	-	-	-	-	-	-	-	-	-	-	-	-	-	-	-
		-	-	-	-	-	-	-	-	-	-	-	-	173054	-	-	-	-	-	-	-	-	-	-	-	-	-	-	-	-	-	-	-	-	-	-	-	-	-	-	-	-	-	-	-	-	-	-	-	-	-	-
		-	+	-	-	-	-	-	-	-	-	-	-	173053	-	-	CMI179C	-	-	-	-	-	-	-	-	-	-	-	-	-	-	-	20780	-	-	82183	-	PFL1045w	-	-	-	-	-	-	-	2777	TA16770	-	-	-	-	-
		-	+	-	-	-	-	GB16019-PA	-	20462	BDEG_04569	CE02516	-	173050	205714	-	CMQ371C	OTTDARP00000005301	-	CG10007-PA	-	142.m00150	ENSGALP00000026725	-	OTTHUMP00000158508	-	159919	14488	38497	-	NCU00025	Os03g51650.1	-	GSPATP00023689001	47075	125582	132483	-	-	RO3G_17004.1	-	SPAC12G12.12	-	SINFRUP00000182066	62.m00229	55	-	-	26497	97417.m00097	-	UM00662.1
		-	-	-	-	-	-	-	-	-	-	-	-	173044	-	-	-	-	-	-	-	-	-	-	-	-	-	-	-	-	-	-	-	-	-	-	-	-	-	-	-	-	-	-	-	-	-	-	-	-	-	-
		-	-	-	-	-	-	-	-	-	-	-	-	173043	-	-	-	-	-	-	-	-	-	-	-	-	-	-	-	-	-	-	-	-	-	-	-	-	-	-	-	-	-	-	-	-	-	-	-	-	-	-
		-	+	-	-	-	-	-	AT1G19690.1	-	-	-	-	173029	-	-	CMF166C	-	-	-	-	-	-	-	-	-	-	-	-	-	-	Os01g15660.1	36045	-	41088	141442	-	-	410525	-	-	-	-	-	-	-	-	-	-	-	-	-
		-	-	-	-	-	-	-	-	-	-	-	-	173027	-	-	-	-	-	-	-	-	-	-	-	-	-	-	-	-	-	Os03g21290.1	-	-	-	-	-	-	-	-	-	-	-	-	-	-	-	-	-	-	-	-
		-	-	-	-	-	-	-	-	-	-	-	-	173020	-	-	-	-	-	-	-	-	-	-	-	-	-	-	-	-	-	-	-	-	-	-	-	-	-	-	-	-	-	-	-	-	-	-	-	-	-	-
		-	+	-	-	-	-	-	AT5G12410.1	-	-	-	-	173010	-	-	-	-	-	-	-	-	-	-	-	-	-	-	-	-	-	Os11g36060.1	35495	-	-	63868	138047	-	549036	-	-	-	-	-	-	-	-	-	-	-	-	-
		-	-	-	-	-	-	-	-	-	-	-	-	173009	-	-	-	-	-	-	-	-	-	-	-	-	-	-	-	-	-	-	-	-	-	-	-	-	796227	-	-	-	-	-	-	-	-	-	-	-	-	-
NIK1	related to plant putative high affinity nickel transporter family	-	+	-	-	-	-	-	AT2G16800.1	-	-	-	-	173008	-	-	-	-	-	-	-	-	-	-	-	-	-	-	-	-	-	Os06g02580.1	21483	-	48510	133373	-	-	767267	-	-	-	-	-	-	23951	-	-	-	-	-	-
		-	-	-	-	-	-	-	-	-	-	-	-	173001	-	-	-	-	-	-	-	-	-	-	-	-	-	-	-	-	-	-	-	-	-	-	-	-	-	-	-	-	-	-	-	-	-	-	-	-	-	-
		-	-	-	-	-	-	-	AT3G23070.1	-	-	-	-	173000	-	-	-	-	-	-	-	-	-	-	-	-	-	-	-	-	-	Os11g37990.1	-	-	-	2185	-	-	230630	-	-	-	-	-	-	-	-	-	-	-	-	-
		-	-	-	-	-	-	-	-	-	-	-	-	172999	-	-	-	-	-	-	-	-	-	-	-	-	-	-	-	-	-	-	-	-	-	-	-	-	-	-	-	-	-	-	-	-	-	-	-	-	-	-
		-	+	-	-	-	-	GB19814-PA	-	-	BDEG_03598	-	218766	172997	212914	-	CMH233C	OTTDARP00000013594	DDB0217302	-	-	-	-	-	OTTHUMP00000077181	-	236781	33205	70083	134862	NCU08032	-	-	-	-	-	-	-	-	-	-	-	-	SINFRUP00000181671	-	-	-	55.m10284	-	-	-	-
		-	-	-	-	-	-	-	-	-	-	-	-	172996	-	-	-	-	-	-	-	-	-	-	-	-	-	-	-	-	-	-	-	-	-	-	-	-	-	-	-	-	-	-	-	-	-	-	-	-	-	-
MAPKKK10	Mitogen Activated Protein Kinase Kinase Kinase Homolog 10	-	-	-	-	-	-	-	-	-	-	-	-	172994	-	-	-	-	-	-	-	-	-	-	-	-	-	-	-	-	-	-	-	-	-	-	-	-	-	-	-	-	-	-	-	-	-	-	-	-	-	-
		-	-	-	-	-	-	-	-	-	-	-	-	172993	-	-	-	-	-	-	-	-	-	-	-	-	-	-	-	-	-	-	-	-	-	-	-	-	-	-	-	-	-	-	-	-	-	-	-	-	-	-
		-	-	-	-	-	-	-	-	-	-	-	-	172988	-	-	-	-	-	-	-	-	-	-	-	-	-	-	-	-	-	-	-	-	-	-	-	-	-	-	-	-	-	-	-	-	-	-	-	-	-	-
		-	-	-	-	-	-	-	-	-	-	-	-	172974	-	-	-	-	-	-	-	-	-	-	-	-	-	-	-	-	-	-	-	-	-	-	-	-	-	-	-	-	-	-	-	-	-	-	-	-	-	-
		-	-	-	-	-	-	-	-	-	-	-	-	172969	-	-	-	-	-	-	-	-	-	-	-	-	-	-	-	-	-	-	-	-	-	-	-	-	-	-	-	-	-	-	-	-	-	-	-	-	-	-
		-	-	-	-	-	-	-	-	-	-	-	-	172968	-	-	-	-	-	-	-	-	-	-	-	-	-	-	-	-	-	-	-	-	-	-	-	-	-	-	-	-	-	-	-	-	-	-	-	-	-	-
		-	-	-	-	-	-	-	-	-	-	-	34929	172967	-	-	-	-	-	-	-	-	ENSGALP00000014618	-	OTTHUMP00000162093	-	-	-	-	-	-	-	-	-	-	-	-	-	-	-	-	-	-	SINFRUP00000154433	-	-	-	-	-	-	-	-
		-	-	-	-	-	-	-	-	-	-	-	-	172958	-	-	-	-	-	-	-	-	-	-	-	-	-	-	-	-	-	-	-	-	-	-	-	-	-	-	-	-	-	-	-	-	-	-	-	-	-	-
		-	+	+	-	-	-	GB11260-PA	AT5G09900.2	69882	BDEG_03174	CE02632	161938	172949	235697	-	CMG093C	OTTDARP00000020399	DDB0203976	CG1100-PA	-	33.m00215	ENSGALP00000005854	-	OTTHUMP00000179135	LmjF21.0760	212047	34487	32805	113443	NCU02650	Os03g63430.1	27017	GSPATP00011424001	24865	182594	108269	PF10_0174	819875	RO3G_11498.1	YDL147W	SPAPB8E5.02c	-	SINFRUP00000127955	78.m00159	38129	TA14975	55.m00252	35460	95400.m00107	Tb10.70.6360	UM00585.1
		-	-	-	-	-	-	-	AT5G27620.1	-	-	CE36037	-	172948	-	-	-	-	-	-	-	-	-	-	-	-	-	-	-	-	-	-	30059	-	-	-	-	-	-	-	-	-	-	-	-	-	-	-	-	-	-	-
		-	-	-	-	-	-	-	-	-	-	-	-	172944	-	-	-	-	-	-	-	-	-	-	-	-	-	-	-	-	-	-	-	-	-	-	-	-	-	-	-	-	-	-	-	-	-	-	-	-	-	-
		-	-	-	-	-	-	-	-	-	-	-	-	172943	-	-	-	-	-	-	-	-	-	-	-	-	-	-	-	-	-	-	-	-	-	-	-	-	-	-	-	-	-	-	-	-	-	-	-	-	-	-
		-	-	-	-	-	-	-	-	-	-	-	-	172940	-	-	-	-	-	-	-	-	-	-	-	-	-	-	-	-	-	-	-	-	-	-	-	-	-	-	-	-	-	-	-	-	-	-	-	-	-	-
		-	-	-	-	-	-	-	-	-	-	-	-	172939	-	-	-	-	-	-	-	-	-	-	-	-	-	-	-	-	-	-	-	-	-	-	-	-	-	-	-	-	-	-	-	-	-	-	-	-	-	-
		-	-	-	-	-	-	-	-	-	-	-	-	172938	-	-	-	-	-	-	-	-	-	-	-	-	-	-	-	-	-	-	-	-	-	-	-	-	-	-	-	-	-	-	-	-	-	-	-	-	-	-
		-	-	-	-	-	-	-	-	-	-	-	-	172935	-	-	-	-	-	-	-	-	-	-	-	-	-	-	-	-	-	-	-	-	-	-	-	-	-	-	-	-	-	-	-	-	-	-	-	-	-	-
		-	-	-	-	-	-	-	-	-	-	-	-	172932	-	-	-	-	-	-	-	-	-	-	-	-	-	-	-	-	-	-	-	-	-	-	-	-	-	-	-	-	-	-	-	-	-	-	-	-	-	-
		-	-	-	-	-	-	-	-	-	-	-	-	172928	-	-	-	-	-	-	-	-	-	-	-	-	-	-	-	-	-	-	-	-	-	-	-	-	-	-	-	-	-	-	-	-	-	-	-	-	-	-
		-	-	-	-	-	-	-	-	-	-	-	-	172920	-	-	-	-	-	-	-	-	-	-	-	-	-	-	-	-	-	-	-	-	-	-	-	-	-	-	-	-	-	-	-	-	-	-	-	-	-	-
		-	-	-	-	-	-	-	-	-	-	-	-	172919	-	-	-	-	DDB0167668	-	19068478	-	-	-	-	-	-	-	48527	-	-	-	-	-	-	-	-	-	-	-	-	-	-	-	-	-	-	-	-	-	-	-
		-	-	-	-	-	-	-	-	-	-	-	-	172918	-	-	-	-	-	-	-	-	-	-	-	-	-	-	-	-	-	-	-	-	-	-	-	-	-	-	-	-	-	-	-	-	-	-	-	-	-	-
		-	-	-	-	-	-	-	-	-	-	-	-	172911	-	-	-	-	-	-	-	-	-	-	-	-	-	-	-	-	-	-	-	-	-	-	-	-	-	-	-	-	-	-	-	-	-	-	-	-	-	-
CHR3423		-	+	-	-	-	-	-	-	-	-	-	-	172906	-	cgd5_150	-	-	-	-	-	31.m00193	-	-	-	-	-	-	-	-	-	Os07g44210.1	-	-	26634	-	-	-	-	-	-	-	-	-	222.m00048	-	-	-	-	-	-	-
ORC2	Origin recognition complex subunit 2, DNA replication control protein.	-	+	-	-	-	-	GB19049-PA	AT2G37560.2	63471	BDEG_00546	CE11492	126602	172905	294784	cgd4_1930	CMS058C	-	DDB0190652	-	-	-	ENSGALP00000013386	-	OTTHUMP00000163672	-	161593	26897	-	100805	NCU01370	Os03g08640.1	4045	-	42935	112122	144268	-	414285	RO3G_11176.1	YBR060C	SPBC685.09	GLEAN3_10533	SINFRUP00000137842	-	260866	-	50.m03076	61999	-	-	-
MINE2		-	-	-	-	-	-	-	-	-	-	-	-	172904	-	-	-	-	-	-	-	-	-	-	-	-	-	-	-	-	-	-	-	-	-	-	-	-	-	-	-	-	-	-	-	-	-	-	-	-	-	-
		-	+	-	-	-	-	-	-	-	-	-	-	172899	285046	-	-	-	-	CG4456-PA	-	-	-	-	-	-	79406	-	-	246755	-	-	36187	-	-	163854	-	-	-	-	-	SPAC4H3.07c	-	SINFRUP00000131592	-	-	-	-	-	-	-	-
		-	-	-	-	-	-	-	-	-	-	-	-	172894	-	-	-	-	-	-	-	-	-	-	-	-	-	-	-	-	-	-	-	-	-	-	-	-	-	-	-	-	-	-	-	-	-	-	-	-	-	-
FAP283	Hypothetical Flagellar Associated Protein	-	-	-	-	-	-	-	-	-	-	-	-	172868	-	-	-	-	-	-	-	-	-	-	-	-	-	-	-	-	-	-	-	-	-	-	-	-	-	-	-	-	-	-	-	-	-	-	-	-	-	-
	similar to dopamine beta-monooxygenase	-	+	-	-	-	-	GB16337-PA	-	-	-	CE31553	141249	172866	261666	-	-	OTTDARP00000006891	-	CG1543-PB	-	-	ENSGALP00000004609	-	OTTHUMP00000022501	-	120180	-	-	79900	-	-	24493	-	36922	-	-	-	-	-	-	-	GLEAN3_22703	SINFRUP00000151722	-	-	-	-	55461	-	-	-
		-	-	-	-	-	-	-	AT1G09160.1	-	-	-	-	172865	-	-	-	-	-	-	-	-	-	-	-	-	-	-	-	-	-	-	23844	-	-	-	-	-	836440	-	-	-	-	-	-	-	-	-	-	-	-	-
		-	-	-	-	-	-	-	-	-	-	-	-	172864	-	-	-	-	-	-	-	-	-	-	-	-	-	-	-	-	-	-	-	-	-	-	-	-	-	-	-	-	-	-	-	-	-	-	-	-	-	-
		-	-	-	-	-	-	-	-	-	-	-	-	172858	-	-	-	-	-	-	-	-	-	-	-	-	-	-	-	244894	-	-	-	-	-	162790	-	-	-	-	-	-	-	-	-	-	-	-	-	-	-	-
		-	-	-	-	-	-	-	-	-	-	-	-	172852	-	-	-	-	-	-	-	-	-	-	-	-	-	-	-	-	-	-	-	-	-	-	-	-	-	-	-	-	-	-	-	-	-	-	-	-	-	-
		-	-	-	-	-	-	-	-	-	-	-	-	-	-	-	-	-	-	-	-	-	-	-	-	-	-	-	-	-	-	-	-	-	-	-	-	-	-	-	-	-	-	-	-	-	-	-	-	-	-	-
		-	-	-	-	-	-	-	-	-	-	-	-	172841	-	-	-	-	-	-	-	-	-	-	-	-	-	-	-	-	-	-	-	-	-	-	-	-	-	-	-	-	-	-	-	-	-	-	-	-	-	-

		-	-	-	-	-	-	-	-	-	-	-	-	172840	-	-	-	-	-	-	-	-	-	-	-	-	-	-	-	-	-	-	-	-	-	-	-	-	-	-	-	-	-	-	-	-	-	-	-	-	-	-
		-	-	-	-	-	-	-	-	-	-	-	-	172839	-	-	-	-	-	-	-	-	-	-	-	-	-	-	-	-	-	-	-	-	-	-	-	-	-	-	-	-	-	-	-	-	-	-	-	-	-	-
		-	-	-	-	-	-	-	-	-	-	-	-	172838	-	-	-	-	-	-	-	-	-	-	-	-	-	-	-	-	-	-	-	-	-	-	-	-	-	-	-	-	-	-	-	-	-	-	-	-	-	-
		-	-	-	-	-	-	-	-	-	-	-	-	172836	-	-	-	-	-	-	-	-	-	-	-	-	-	-	-	-	-	-	-	-	-	-	-	-	-	-	-	-	-	-	-	-	-	-	-	-	-	-
		-	-	-	-	-	-	-	-	-	-	-	-	172835	-	-	-	-	-	-	-	-	-	-	-	-	-	-	-	-	-	-	-	-	-	-	-	-	-	-	-	-	-	-	-	-	-	-	-	-	-	-
		-	-	-	-	-	-	-	-	-	-	-	-	172820	-	-	-	-	-	-	-	-	-	-	-	-	-	-	-	-	-	-	-	-	-	-	-	-	139246	-	-	-	-	-	-	-	-	-	-	-	-	-	-	-	-	-
ELG6	exostosin-like glycosyltransferase	-	-	-	-	-	-	-	-	-	-	-	-	172818	-	-	-	-	-	-	-	-	-	-	-	-	-	-	-	-	-	-	30479	-	-	-	-	-	-	-	-	-	-	-	-	-	-	-	-	88599.m00176	-	-
		-	+	-	-	-	-	-	AT5G16810.1	-	-	-	-	172808	-	-	-	-	-	-	-	-	-	-	-	-	-	-	-	-	-	Os02g46750.5	32880	-	-	137387	-	-	738378	-	-	-	-	-	-	-	-	-	-	-	-	-
		-	-	-	-	-	-	-	-	-	-	-	-	172805	-	-	-	-	-	-	-	-	-	-	-	-	-	-	-	-	-	-	-	-	-	-	-	-	-	-	-	-	-	-	-	-	-	-	-	-	-	-
MRG7MT	Catalyzes the addition of a methyl group to the GpppN terminus of capped mRNA	-	+	-	-	-	-	GB13432-PA	AT3G20650.2	-	BDEG_05239	-	-	172799	262503	-	-	-	-	CG3688-PA	-	82.m00166	-	-	-	-	-	29289	-	-	-	Os08g08200.2	-	GSPATP00017945001	-	124439	141377	-	726996	RO3G_02523.1	YBR236C	SPCC330.10	-	-	-	-	TA12560	-	30586	84433.m00172	-	-
		-	-	-	-	-	-	-	-	-	-	-	-	172798	-	-	-	-	-	-	-	-	-	-	-	-	-	-	-	-	-	-	-	-	-	-	-	-	-	-	-	-	-	-	-	-	-	-	-	-	-	-
		-	-	-	-	-	-	-	-	-	-	-	-	172787	-	-	-	-	-	-	-	-	-	-	-	-	-	-	-	-	-	-	-	-	-	-	-	-	-	-	-	-	-	-	-	-	-	-	-	-	-	-
		-	+	-	-	-	-	-	AT2G12550.1	-	-	-	227081	172781	244984	-	-	-	-	CG15445-PC	-	-	-	-	-	-	224600	-	74939	178169	-	Os04g55150.2	-	-	-	150235	-	-	745627	-	-	-	GLEAN3_12136	-	-	-	-	-	54183	-	-	-
		-	+	+	-	-	-	GB12634-PA	AT1G65030.1	64932	BDEG_07970	CE32468	202465	172780	274396	cgd4_2450	-	OTTDARP00000020309	DDB0167713	CG12608-PA	-	106.m00128	ENSGALP00000026942	GL50803_7655	OTTHUMP00000023189	LmjF18.0530	103457	24034	64818	171129	-	Os12g42150.1	34006	GSPATP00031544001	47198	136779	137026	-	423071	RO3G_16260.1	YKL021C	SPCC16C4.08c	GLEAN3_26946	SINFRUP00000159246	-	268880	TA12020	49.m05682	18240	86319.m00304	Tb10.61.2870	UM04067.1
		-	-	-	-	-	-	-	-	-	-	-	-	172777	-	-	-	-	-	-	-	-	-	-	-	-	-	-	-	-	-	-	-	-	-	-	-	-	-	-	-	-	-	-	-	-	-	-	-	-	-	-
		-	+	+	-	-	-	-	-	64322	-	CE20291	222178	172776	226636	cgd2_2430	CMK140C	-	DDB0187616	-	-	14.m00330	ENSGALP00000024299	GL50803_15984	NP_060909.1	LmjF36.4940	129058	23477	-	92085	NCU09542	-	-	-	48866	233499	158288	-	-	RO3G_02781.1	YCR059C	SPAC27E2.02	-	SINFRUP00000146124	-	-	-	583.m05481	55540	90019.m00248	Tb11.01.2750	UM02130.1
		-	-	-	-	-	-	-	-	-	-	-	-	172775	-	-	-	-	-	-	-	-	-	-	-	-	-	-	-	-	-	-	-	-	-	-	-	-	-	-	-	-	-	-	-	-	-	-	-	-	-	-
		-	-	-	-	-	-	-	-	-	-	-	-	172761	-	-	-	-	-	-	-	-	-	-	-	-	-	-	-	-	-	-	-	-	-	-	-	-	-	-	-	-	-	-	-	-	-	-	-	-	-	-
		-	+	-	-	-	-	GB16685-PA	-	27507	-	-	-	172747	-	-	-	-	DDB0192205	-	-	-	-	-	OTTHUMP00000020194	-	115412	-	-	-	-	-	10510	-	-	63910	-	-	-	RO3G_04298.1	-	-	-	-	-	-	-	-	38435	-	-	-
		-	-	-	-	-	-	-	-	-	-	-	-	172734	-	-	-	-	-	-	-	-	-	-	-	-	-	-	-	-	-	-	-	-	-	-	-	-	-	-	-	-	-	-	-	-	-	-	-	-	-	-
		-	-	-	-	-	-	-	-	-	-	-	-	172728	-	-	-	-	-	-	-	-	-	-	-	-	-	-	-	-	-	-	-	-	-	-	-	-	-	-	-	-	-	-	-	-	-	-	-	-	-	-
		-	-	-	-	-	-	-	-	-	-	-	-	172727	-	-	-	-	-	-	-	-	-	-	-	-	-	-	-	-	-	-	-	-	-	-	-	-	-	-	-	-	-	-	-	-	-	-	-	-	-	-
		-	-	-	-	-	-	-	-	-	-	-	-	172726	-	-	-	-	-	-	-	-	-	-	-	-	-	-	-	-	-	-	-	-	-	-	-	-	-	-	-	-	-	-	-	-	-	-	-	-	-	-
		-	-	-	-	-	-	-	-	-	-	-	-	172715	-	-	-	-	-	-	-	-	-	-	-	-	-	-	-	-	-	-	-	-	-	-	-	-	-	-	-	-	-	-	-	-	-	-	-	-	-	-
	SET domain containing protein, putative histone methyltransferase	-	-	-	-	-	-	-	-	-	-	-	-	172711	-	-	-	-	-	-	-	-	-	-	-	-	174941	-	-	-	-	-	-	-	-	-	-	PFF1440w	-	-	-	-	-	-	-	-	TA09890	-	-	-	-	-
		-	-	-	-	-	-	-	-	-	-	-	-	172701	-	-	-	-	-	-	-	-	-	-	-	-	-	-	-	-	-	-	-	-	-	-	-	-	-	-	-	-	-	-	-	-	-	-	-	-	-	-
		-	-	-	-	-	-	-	-	-	-	-	-	172688	-	-	-	-	-	-	-	-	-	-	-	-	-	-	-	-	-	-	-	-	-	-	-	-	-	-	-	-	-	-	-	-	-	-	-	-	-	-
		-	-	-	-	-	-	-	-	-	-	-	-	172672	-	-	-	-	-	-	-	-	-	-	-	-	-	22803	-	-	-	-	-	-	-	-	-	-	-	-	-	-	-	-	-	-	-	-	-	-	-	-
		-	-	-	-	-	-	-	-	-	-	-	-	172671	-	-	-	-	-	-	-	-	-	-	-	-	-	-	-	-	-	-	-	-	-	-	-	-	-	-	-	-	-	-	-	-	-	-	-	-	-	-
		-	+	-	-	-	-	-	AT1G28100.2	-	-	-	-	172656	-	-	-	-	-	-	-	-	-	-	-	-	-	-	-	-	-	Os10g35644.1	22328	-	-	179574	-	-	554701	-	-	-	-	-	-	-	-	-	-	-	-	-
		-	+	+	-	-	-	GB12242-PA	AT1G65660.1	26976	BDEG_03332	CE06116	33844	172647	261481	cgd7_1750	-	OTTDARP00000023990	DDB0204278	-	-	85.m00132	ENSGALP00000040271	-	OTTHUMP00000160841	LmjF08.1180	124539	37998	34959	197614	NCU01950	Os08g03390.1	36451	GSPATP00003554001	28285	176942	137629	PFF0500c	574877	RO3G_03854.1	YDR088C	SPBC365.05c	GLEAN3_22770	SINFRUP00000152455	31.m00305	-	TA07860	583.m00017	32543	84943.m00184	Tb927.5.3020	UM04951.1
		-	-	-	-	-	-	-	-	-	-	-	-	172638	-	-	-	-	-	-	-	-	-	-	-	-	-	-	-	-	-	-	-	-	-	-	-	-	-	-	-	-	-	-	-	-	-	-	-	-	-	-
		-	-	-	-	-	-	-	-	-	-	-	-	172632	-	-	-	-	-	-	-	-	-	-	-	-	-	-	-	-	-	-	-	-	-	-	-	-	-	-	-	-	-	-	-	-	-	-	-	-	-	-
		-	-	-	-	-	-	-	-	-	-	-	-	172620	-	-	-	-	-	-	-	-	-	-	-	-	-	-	-	-	-	-	-	-	-	-	-	-	-	-	-	-	-	-	-	-	-	-	-	-	-	-
		-	-	-	-	-	-	-	-	-	-	-	-	172618	-	-	-	-	-	-	-	-	-	-	-	-	-	-	-	-	-	-	-	-	-	-	-	-	-	-	-	-	-	-	-	-	-	-	-	-	-	-
		-	-	-	-	-	-	-	-	-	-	-	-	172615	-	-	-	-	-	-	-	-	-	-	-	-	-	-	-	-	-	-	-	-	-	-	-	-	-	-	-	-	-	-	128.m00148	-	-	-	-	-	-	-
		-	-	-	-	-	-	-	-	-	-	-	-	172614	-	-	-	-	-	-	-	-	-	-	-	-	-	-	-	-	-	-	-	-	-	-	-	-	-	-	-	-	-	-	-	-	-	-	-	-	-	-
		-	-	-	-	-	-	-	-	-	-	-	-	172610	-	-	-	-	-	-	-	-	-	-	-	-	-	-	-	-	-	-	-	-	-	-	-	-	-	-	-	-	-	-	-	-	-	-	-	-	-	-
		-	-	-	-	-	-	-	-	-	-	-	-	172608	-	-	-	-	-	-	-	-	-	-	-	-	-	-	-	-	-	-	-	-	-	-	-	-	-	-	-	-	-	-	-	-	-	-	-	-	-	-
SEC13L	COP-II coat subunit, nucleoporin	-	-	-	-	-	-	-	-	-	-	-	-	172606	-	-	-	-	-	-	-	-	-	-	-	-	-	-	-	-	-	-	-	-	-	-	-	-	-	-	-	-	-	-	-	-	-	-	-	-	-	-
		-	+	-	-	-	-	-	-	62763	-	-	117326	172603	205606	-	-	-	-	-	-	-	ENSGALP00000007839	-	-	-	233089	-	-	247106	-	-	-	GSPATP00018373001	-	-	-	-	-	-	-	-	-	-	11.m00543	-	-	-	54634	-	-	-
		-	-	-	-	-	-	-	-	-	-	-	-	172602	-	-	-	-	-	-	-	-	-	-	-	-	-	-	-	-	-	-	-	-	-	-	-	-	-	-	-	-	-	-	-	-	-	-	-	-	-	-
		-	-	-	-	-	-	-	-	-	-	-	-	172601	-	-	-	-	-	-	-	-	-	-	-	-	-	-	-	-	-	-	-	-	-	-	-	-	-	-	-	-	-	-	-	-	-	-	-	-	-	-
	Putative FAD synthetase	-	+	-	-	-	-	-	AT5G03430.1	-	-	-	-	172594	-	-	-	-	-	-	-	-	-	-	-	-	-	-	-	-	-	Os01g15490.3	-	-	12894	-	-	-	282187	-	-	-	-	-	-	31599	-	-	-	-	-	-
		-	-	-	-	-	-	-	-	-	-	-	-	172576	-	-	-	-	-	-	-	-	-	-	-	-	-	-	-	-	-	-	-	-	-	-	-	-	-	-	-	-	-	-	-	-	-	-	-	-	-	-
		-	-	-	-	-	-	-	-	-	-	-	-	172572	-	-	-	-	-	-	-	-	-	-	-	-	-	-	-	-	-	-	-	-	-	-	-	-	-	-	-	-	-	-	-	-	-	-	-	-	-	-
		-	-	-	-	-	-	-	-	-	-	-	-	172564	-	-	-	-	-	-	-	-	-	-	-	-	-	-	-	-	-	-	-	-	-	-	-	-	-	-	-	-	-	-	-	-	-	-	-	-	-	-
HTB37	Histone H2B	-	+	-	-	-	-	-	AT5G59910.1	-	-	-	-	172560	-	-	-	-	DDB0218827	-	-	-	-	GL50803_121045	-	-	-	-	-	-	-	-	20501	-	-	-	-	-	725114	-	-	-	-	-	-	-	-	-	-	-	-	-
		-	-	-	-	-	-	-	-	-	-	-	-	172529	-	-	-	-	-	-	-	-	-	-	-	-	-	-	-	-	-	-	-	-	-	-	-	-	-	-	-	-	-	-	-	-	-	-	-	-	-	-
		-	+	-	-	-	-	GB18688-PA	AT4G34270.1	-	BDEG_00047	CE40006	149664	172525	289916	-	CMM193C	-	DDB0186748	CG9578-PA	-	-	ENSGALP00000024553	-	OTTHUMP00000032581	-	131242	6025	36423	232080	NCU00109	Os03g55270.1	33739	GSPATP00000895001	15286	210994	158140	-	833109	RO3G_09243.1	YPR040W	SPCC4B3.16	GLEAN3_12988	SINFRUP00000157863	69.m00196	-	-	-	56416	-	-	-
		-	-	-	-	-	-	-	-	-	-	-	-	172519	-	-	-	-	-	-	-	-	-	-	-	-	-	-	-	-	-	-	-	-	-	-	-	-	-	-	-	-	-	-	-	-	-	-	-	-	-	-
		-	+	-	-	-	-	-	AT3G61770.1	-	-	-	-	172512	-	-	-	-	-	-	-	-	-	-	-	-	-	-	-	-	-	Os05g45770.1	7332	-	-	128229	-	-	755397	-	-	-	-	-	-	-	-	-	-	-	-	-
		-	-	-	-	-	-	-	-	-	-	-	-	172511	-	-	-	-	-	-	-	-	-	-	-	-	-	-	-	-	-	-	-	-	-	-	-	-	-	-	-	-	-	-	-	-	-	-	-	-	-	-
		-	-	-	-	-	-	-	-	-	BDEG_04961	-	-	172506	-	-	-	-	DDB0218120	-	-	-	-	-	-	-	-	-	-	-	-	-	-	-	-	-	-	-	-	-	-	-	-	-	-	-	-	-	-	-	-	-
		-	-	-	-	-	-	-	-	-	-	-	-	172505	-	-	-	-	-	-	-	-	-	-	-	-	-	-	-	-	-	-	-	-	-	173097	-	-	-	-	-	-	-	-	-	-	-	-	-	-	-	-
		-	+	-	-	-	-	GB18576-PA	AT3G10070.1	-	BDEG_02575	-	162600	172500	266827	-	CMS144C	-	DDB0203246	CG17358-PA	-	-	ENSGALP00000040339	-	OTTHUMP00000003785	-	58640	-	-	139976	-	Os01g63940.1	9439	-	-	27365	-	-	560743	RO3G_00934.1	YDR145W	SPAC15A10.02	-	SINFRUP00000134942	-	-	-	-	31979	-	-	-
		-	-	-	-	-	-	-	-	-	-	-	160077	172498	-	-	-	-	-	-	-	-	-	-	-	-	-	-	-	167154	-	-	35161	-	-	-	-	-	-	-	-	-	-	SINFRUP00000181516	-	-	-	-	-	-	-	-
AMT8	ammonia channel	-	-	-	-	-	-	-	-	-	-	-	-	172494	-	-	-	-	-	-	-	-	-	-	-	-	-	-	-	-	-	-	-	-	-	-	-	-	-	-	-	-	-	-	-	-	-	-	-	-	-	-
		-	+	-	-	-	-	-	AT1G67780.1	-	-	-	-	172486	369499	-	-	-	-	-	-	-	-	-	-	-	-	-	65903	-	-	-	32111	-	-	72234	-	-	-	-	-	-	-	-	-	-	-	-	-	-	-	-
FAP134	Conserved Uncharacterized Flagellar Associated Protein	+	+	-	+	-	-	GB14746-PA	-	32964	BDEG_06423	-	114721	172483	287704	-	-	-	-	CG13125-PA	-	-	ENSGALP00000007847	GL50803_17567	OTTHUMP00000182927	LmjF30.3640	212018	37452	74561	244599	-	-	-	GSPATP00022674001	-	95190	133309	-	-	-	-	-	-	SINFRUP00000133398	31.m00250	-	-	27.m00860	20807	89416.m00096	Tb927.6.5030	-
		-	-	-	-	-	-	-	-	-	BDEG_07341	-	-	172481	-	-	-	-	-	-	-	-	-	-	-	-	-	-	-	-	NCU05916	-	-	-	-	-	-	-	-	-	-	-	-	-	-	-	-	-	-	-	-	UM00845.1
		-	-	-	-	-	-	-	-	67624	-	-	-	172474	-	-	-	-	-	-	-	-	-	-	-	-	-	-	-	-	-	-	-	-	-	-	-	-	-	-	-	-	-	-	-	-	-	-	-	-	-	-
		-	-	-	-	-	-	-	-	-	-	-	-	172473	-	-	-	-	-	-	-	-	-	-	-	-	-	-	-	-	-	-	-	-	-	-	-	-	-	-	-	-	-	-	-	-	-	-	-	-	-	-
	RabGAP/TBC Domain Protein	-	-	-	-	-	-	-	-	-	-	-	-	172469	-	-	-	-	-	-	-	-	-	-	-	-	-	-	-	-	-	-	-	-	-	31780	-	-	-	-	-	-	-	-	-	-	-	-	-	-	-	-
		-	-	-	-	-	-	-	-	-	-	-	-	172467	-	-	-	-	-	-	-	-	-	-	-	-	-	-	-	-	-	-	-	-	-	-	-	-	-	-	-	-	-	-	-	-	-	-	-	-	-	-
		-	+	-	-	-	-	-	AT4G36050.2	-	-	-	176829	172466	246976	-	-	OTTDARP00000023293	-	-	19074386	-	-	-	OTTHUMP00000023390	LmjF36.5270	200437	-	-	161616	NCU01961	Os09g36530.2	5834	-	-	-	155906	-	799764	-	-	SPBC3D6.10	GLEAN3_02644	SINFRUP00000131313	-	-	-	-	19413	-	-	UM00618.1
		-	-	-	-	-	-	-	-	-	-	-	-	172465	-	-	-	-	-	-	-	-	-	-	-	-	-	-	-	-	-	-	-	-	-	-	-	-	-	-	-	-	-	-	-	-	-	-	-	-	-	-
		-	-	-	-	-	-	-	-	-	-	-	-	172460	-	-	-	-	-	-	-	-	-	-	-	-	-	-	-	223118	-	-	-	-	-	-	-	-	-	-	-	-	-	-	-	-	-	-	-	-	-	-
		-	-	-	-	-	-	-	-	-	-	-	-	172456	-	-	-	-	-	-	-	-	-	-	-	-	-	-	-	-	-	-	-	-	-	-	-	-	-	-	-	-	-	-	-	-	-	-	-	-	-	-
		-	-	-	-	-	-	-	-	-	-	-	-	172454	-	-	-	-	-	-	-	-	-	-	-	-	-	-	-	-	-	-	-	-	-	-	-	-	-	-	-	-	-	-	-	-	-	-	-	-	-	-
		-	-	-	-	-	-	-	-	-	-	-	-	172452	-	-	-	-	-	-	-	-	-	-	-	-	-	-	-	-	-	-	-	-	-	-	-	-	-	-	-	-	-	-	-	-	-	-	-	-	-	-
		-	+	-	-	-	-	-	AT4G10360.2	-	-	-	161581	172450	289549	-	-	-	DDB0217869	-	-	-	ENSGALP00000008941	-	OTTHUMP00000012540	-	219318	37345	38487	182635	NCU02977	-	-	-	-	-	-	-	205333	RO3G_05475.1	YJR116W	SPAC17A2.02c	-	SINFRUP00000179487	-	-	-	52.m03468	-	-	-	UM00087.1
		-	-	-	-	-	-	-	-	-	-	-	-	172442	270470	-	-	-	DDB0191653	-	-	-	-	-	-	-	-	-	-	-	-	-	-	-	-	-	-	-	-	-	-	-	-	-	-	-	-	-	-	-	-	-
		-	-	-	-	-	-	-	-	-	-	-	-	172438	-	-	-	-	-	-	-	-	-	-	-	-	-	-	-	-	-	-	-	-	-	-	-	-	-	-	-	-	-	-	-	-	-	-	-	-	-	-
		-	-	-	-	-	-	-	-	-	-	-	-	172436	-	-	-	-	-	-	-	-	-	-	-	-	-	-	-	-	-	-	-	-	-	-	-	-	-	-	-	-	-	-	-	-	-	-	-	-	-	-
		-	-	-	-	-	-	-	AT5G51510.1	-	-	-	-	172434	-	-	-	-	-	-	-	-	-	-	-	-	-	-	-	-	-	Os10g36480.1	-	-	-	167783	-	-	662465	-	-	-	-	-	-	-	-	-	-	-	-	-
TOM20	Translocase of outer membrane TOM20, mitochondrial	-	+	-	-	-	-	-	AT3G27080.1	-	-	-	-	172432	-	-	-	-	-	-	-	-	-	-	-	-	-	-	-	-	-	Os01g69250.1	35937	-	-	224035	-	-	829357	-	-	-	-	-	-	-	-	-	-	-	-	-
		-	-	-	-	-	-	-	-	-	-	-	-	172429	-	-	-	-	-	-	-	-	-	-	-	-	-	-	-	-	-	-	-	-	-	-	-	-	-	-	-	-	-	-	-	-	-	-	-	-	-	-
		-	-	-	-	-	-	-	-	-	-	-	-	172427	-	-	-	-	-	-	-	-	-	-	-	-	-	-	-	-	-	-	-	-	-	-	-	-	-	-	-	-	-	-	-	-	-	-	-	-	-	-
		-	-	-	-	-	-	-	-	-	-	-	-	172418	-	-	-	-	-	-	-	-	-	-	-	-	-	-	-	-	-	-	-	-	-	-	-	-	-	-	-	-	-	-	-	-	-	-	-	-	-	-
		-	-	-	-	-	-	-	AT5G24710.1	-	-	-	-	172416	-	-	-	-	-	-	-	-	-	-	-	-	-	-	-	-	-	Os01g46510.2	-	-	-	205506	-	-	819144	-	-	-	-	-	-	-	-	-	-	-	-	-
ALA1	putative phospholipid-transporting ATPase (Aminophospholipid flippase)	-	-	-	-	-	-	-	-	-	-	-	-	172401	-	-	-	-	-	-	-	-	-	-	-	-	-	-	-	-	NCU07443	-	-	-	-	-	-	-	-	-	-	-	-	-	-	-	TA04770	-	-	-	-	-
		-	-	-	-	-	-	-	-	-	-	-	-	172398	-	-	-	-	-	-	-	-	-	-	-	-	-	-	-	-	-	-	-	-	-	-	-	-	-	-	-	-	-	-	-	-	-	-	-	-	-	-
FAP78	Flagellar Associated Protein	-	-	-	+	-	-	-	-	70906	-	-	-	172392	-	-	-	-	-	-	-	-	-	-	-	-	-	-	-	-	-	-	-	GSPATP00036877001	-	-	132661	-	-	-	-	-	-	-	216.m00041	-	-	-	-	-	-	-
		-	-	-	-	-	-	-	-	-	-	-	-	172390	-	-	-	-	-	-	-	-	-	-	-	-	-	-	-	-	-	-	-	-	-	-	-	-	-	-	-	-	-	-	-	-	-	-	-	-	-	-
		-	-	-	-	-	-	-	-	-	-	-	-	172376	-	-	-	-	-	-	-	-	-	-	-	-	-	-	-	-	-	-	-	-	-	-	-	-	-	-	-	-	-	-	-	-	-	-	-	-	-	-
CHR3422	SWI/SNF chromatin remodeling complex component	-	-	-	-	-	-	-	-	-	-	-	-	172374	-	-	-	-	-	-	-	-	-	-	-	-	-	-	-	-	-	-	-	-	-	-	-	-	-	-	-	-	-	-	-	-	-	-	-	-	-	-
		-	-	-	-	-	-	-	-	-	-	-	-	172364	-	-	-	-	-	-	-	-	-	-	-	-	-	-	-	-	-	-	-	-	-	-	-	-	-	-	-	-	-	-	-	-	-	-	-	-	-	-
		-	+	-	-	-	-	-	-	72094	BDEG_06185	-	219007	172362	-	cgd6_1670	-	-	DDB0185667	-	-	-	-	-	-	LmjF32.3110	-	32543	1986	70832	NCU06659	-	-	-	3131	-	109516	-	-	-	YGR210C	SPBC428.15	-	-	-	21326	-	42.m03364	-	-	Tb11.01.8680	-
		-	-	-	-	-	-	-	-	-	-	-	-	172356	-	-	-	-	-	-	-	-	-	-	-	-	-	-	-	-	-	-	-	-	-	-	-	-	-	-	-	-	-	-	-	-	-	-	-	-	-	-
		-	-	-	-	-	-	-	-	-	-	-	-	172355	-	-	-	-	-	-	-	-	-	-	-	-	-	-	-	-	-	-	-	-	-	-	-	-	-	-	-	-	-	-	-	-	-	-	-	-	-	-
		-	-	-	-	-	-	-	-	-	-	-	-	172348	-	-	-	-	-	-	-	-	-	-	-	-	-	-	-	-	-	-	-	-	-	-	-	-	-	-	-	-	-	-	-	-	-	-	-	-	-	-
		-	-	-	-	-	-	-	-	-	-	-	-	172344	-	-	-	-	-	-	-	-	-	-	-	-	-	-	-	-	-	-	-	-	-	-	-	-	-	-	-	-	-	-	-	-	-	-	-	-	-	-
		-	-	-	-	-	-	-	-	-	-	-	-	172338	-	-	-	-	-	-	-	-	-	-	-	-	-	-	-	-	-	-	-	-	-	-	-	-	-	-	-	-	-	-	-	-	-	-	-	-	-	-
		-	-	-	-	-	-	-	AT4G19160.1	-	-	-	-	172333	-	-	-	-	-	-	-	-	-	-	-	-	-	-	-	-	-	-	-	-	-	139801	-	-	815263	-	-	-	-	-	-	-	-	-	-	-	-	-
		-	-	-	-	-	-	-	-	-	-	-	-	172331	-	-	-	-	-	-	-	-	-	-	-	-	-	-	-	-	-	-	-	-	-	-	-	-	-	-	-	-	-	-	-	-	-	-	-	-	-	-
		-	-	-	-	-	-	-	-	-	-	-	-	172329	-	-	-	-	-	-	-	-	-	-	-	-	-	-	-	-	-	-	-	-	-	-	-	-	-	-	-	-	-	-	-	-	-	-	-	-	-	-
		-	-	-	-	-	-	-	-	-	-	-	-	172327	-	-	-	-	-	-	-	-	-	-	-	-	-	-	-	-	-	-	-	-	-	-	-	-	-	-	-	-	-	-	-	-	-	-	-	-	-	-
		-	-	-	-	-	-	-	-	-	-	-	-	172317	-	-	-	-	-	-	-	-	-	-	-	-	-	-	-	-	-	-	35934	-	-	-	-	-	-	-	-	-	-	-	-	-	-	-	-	-	-	-
		-	-	-	-	-	-	-	-	-	-	-	-	172314	-	-	-	-	-	-	-	-	-	-	-	-	-	-	-	-	-	-	-	-	-	-	-	-	-	-	-	-	-	-	-	-	-	-	-	-	-	-
		-	-	-	-	-	-	-	-	-	-	-	-	172308	-	-	-	-	-	-	-	-	-	-	-	-	-	-	-	-	-	-	-	-	-	-	-	-	-	-	-	-	-	-	-	-	-	-	-	-	-	-
		-	-	-	-	-	-	-	-	-	-	-	-	172301	-	-	-	-	-	-	-	-	-	-	-	-	-	-	-	-	-	-	-	-	-	-	-	-	-	-	-	-	-	-	-	-	-	-	-	-	-	-
		-	-	-	-	-	-	-	-	-	-	-	-	172297	-	-	-	-	-	-	-	-	-	-	-	-	-	-	-	-	-	-	-	-	-	-	-	-	-	-	-	-	-	-	-	-	-	-	-	-	-	-
		-	+	-	-	-	-	-	AT5G51880.1	-	-	-	-	172294	-	-	-	-	-	-	-	-	-	-	-	-	-	-	-	-	NCU02176	Os03g02730.1	-	-	-	148314	139930	-	423168	RO3G_04420.1	-	-	-	-	-	-	-	-	-	-	Tb927.5.3950	-
SPO11b	Topoisomerase, involved in meiosis.	-	-	-	-	-	-	-	-	-	-	-	-	172293	-	-	-	-	-	-	-	-	-	-	-	LmjF16.0630	-	-	-	-	-	-	-	-	-	-	-	-	-	-	-	-	-	-	-	-	-	-	-	-	-	-
		-	-	-	-	-	-	-	-	-	-	-	-	172290	-	-	-	-	-	-	-	-	-	-	-	-	-	-	-	-	-	-	-	-	-	-	-	-	-	-	-	-	-	-	-	-	-	-	-	-	-	-
		-	-	-	-	-	-	-	-	-	-	-	-	172288	-	-	-	-	-	-	-	-	-	-	-	-	-	-	-	198549	-	-	-	-	-	-	-	-	-	-	-	-	-	-	24.m00192	-	-	-	-	-	-	-
		-	-	-	-	-	-	-	-	-	-	-	-	172274	-	-	-	-	-	-	-	-	-	-	-	-	-	-	-	-	-	-	-	-	-	-	-	-	-	-	-	-	-	-	-	-	-	-	-	-	-	-
		-	-	-	-	-	-	-	-	-	-	-	-	172268	-	-	-	-	-	-	-	-	-	-	-	-	-	-	-	-	-	-	-	-	-	-	-	-	-	-	-	-	-	-	-	-	-	-	-	-	-	-
		-	-	-	-	-	-	-	-	-	-	-	-	172264	-	-	-	-	-	-	-	-	-	-	-	-	-	-	-	-	-	-	-	-	-	-	-	-	-	-	-	-	-	-	-	-	-	-	-	-	-	-
RSP14	Radial spoke protein 14	-	+	-	+	-	-	-	-	-	-	-	198826	172252	224607	-	-	-	-	-	-	-	ENSGALP00000010843	-	OTTHUMP00000028948	-	-	-	-	161307	-	-	-	-	-	-	-	-	-	-	-	-	-	-	-	-	-	-	63484	-	-	-
		-	-	-	-	-	-	-	-	-	-	-	-	172235	-	-	-	-	-	-	-	-	-	-	-	-	-	-	-	-	-	-	-	-	-	-	-	-	-	-	-	-	-	-	-	-	-	-	-	-	-	-
		-	-	-	-	-	-	-	AT4G26980.1	-	-	-	-	172229	-	-	-	-	-	-	-	-	-	-	-	-	-	-	-	-	-	Os06g46590.1	-	-	-	131553	-	-	234858	-	-	-	-	-	-	-	-	-	-	-	-	-
		-	-	-	-	-	-	-	-	-	-	-	-	172225	-	-	-	-	-	-	-	-	-	-	-	-	-	-	-	-	-	-	-	-	-	-	-	-	-	-	-	-	-	-	-	-	-	-	-	-	-	-
		-	-	-	-	-	-	-	-	-	-	-	-	172222	-	-	-	-	-	-	-	-	-	-	-	-	-	-	-	-	-	-	-	-	-	-	-	-	-	-	-	-	-	-	-	-	-	-	-	-	-	-
		-	+	-	-	-	-	GB16542-PA	AT3G15410.1	53551	-	-	182426	172216	-	-	CMD073C	OTTDARP00000020167	-	-	-	124.m00125	ENSGALP00000018412	GL50803_9618	OTTHUMP00000010944	-	190595	15699	-	159727	-	Os02g58030.1	-	GSPATP00015320001	-	171710	129270	-	568927	-	-	-	-	SINFRUP00000160917	113.m00129	-	-	-	51723	-	-	-
		-	-	-	-	-	-	-	-	-	-	-	-	172215	-	-	-	-	-	-	-	-	-	-	-	-	-	-	-	-	-	-	-	-	-	-	-	-	-	-	-	-	-	-	-	-	-	-	-	-	-	-
		-	+	-	-	-	-	-	AT1G79350.1	34285	-	-	195718	172202	239891	-	-	-	-	CG1903-PB	-	-	ENSGALP00000005353	-	NP_060653.2	-	117101	-	-	30047	-	Os08g12680.1	-	-	-	-	-	-	765689	-	-	-	-	SINFRUP00000160104	-	21138	-	-	29150	-	-	-
		-	-	-	-	-	-	-	AT5G15170.1	-	-	-	-	172201	-	-	-	-	-	-	-	-	-	-	-	-	-	-	-	-	-	Os07g34598.2	-	-	-	137862	-	-	196766	-	-	-	-	-	-	-	-	-	-	-	-	-
		-	-	-	-	-	-	-	-	-	-	-	-	172198	-	-	-	-	-	-	-	-	-	-	-	-	-	-	-	-	-	-	-	-	-	-	-	-	-	-	-	-	-	-	-	-	-	-	-	-	-	-
		-	-	-	-	-	-	-	-	-	-	-	-	172194	-	-	-	-	-	-	-	-	-	-	-	-	-	-	-	-	-	-	-	-	-	-	-	-	-	-	-	-	-	-	-	-	-	-	-	-	-	-
		-	-	-	-	-	-	-	-	-	-	-	-	172169	-	-	-	-	-	-	-	-	-	-	-	-	-	-	-	-	-	-	-	-	-	-	-	-	-	-	-	-	-	-	-	-	-	-	-	-	-	-
		+	+	-	-	-	-	GB20136-PA	-	-	-	CE33853	-	172167	210269	-	-	OTTDARP00000015220	-	CG32533-PA	-	-	-	-	OTTHUMP00000195509	-	-	-	-	241791	-	-	12886	-	-	-	-	-	-	-	-	-	GLEAN3_26336	SINFRUP00000156676	-	-	-	-	22905	-	-	-
		-	-	-	-	-	-	-	-	-	-	-	-	172142	-	-	-	-	-	-	-	-	-	-	-	-	-	-	-	-	-	-	-	-	-	-	-	-	-	-	-	-	-	-	-	-	-	-	-	-	-	-
		-	-	-	-	-	-	-	-	-	-	-	-	172140	-	-	-	-	-	-	-	-	-	-	-	-	-	-	-	-	-	-	-	-	-	-	-	-	-	-	-	-	-	-	-	-	-	-	-	-	-	-
		-	+	-	-	-	-	-	AT2G32380.1	15943	BDEG_02208	-	77981	172136	-	-	-	-	-	-	-	-	ENSGALP00000009132	-	OTTHUMP00000163319	-	180041	25127	70654	-	-	Os07g27450.1	34935	-	36620	8495	-	-	201293	RO3G_10324.1	YLR050C	-	-	SINFRUP00000139807	-	-	-	-	26143	-	-	UM01876.1
		-	-	-	-	-	-	-	AT5G59500.1	-	-	-	-	172133	-	-	-	-	-	-	-	-	-	-	-	-	-	-	-	-	-	-	-	-	-	187807	-	-	650244	-	-	-	-	-	-	-	-	-	-	-	-	-
		-	-	-	-	-	-	-	-	-	-	-	-	172117	-	-	-	-	-	-	-	-	-	-	-	-	-	-	-	-	-	-	34614	-	-	-	-	-	-	RO3G_08196.1	-	-	-	-	-	-	-	-	-	-	-	-
		+	+	-	-	-	-	-	-	65979	BDEG_02391	-	214413	172110	278024	-	-	OTTDARP00000020114	-	-	-	-	-	-	NP_775814.1	-	236380	7253	66420	247454	-	-	-	-	-	172994	142078	-	-	-	-	-	GLEAN3_05514	SINFRUP00000165791	-	-	-	-	-	94339.m00007	-	-
		-	-	-	-	-	-	-	-	-	-	-	-	172108	-	-	-	-	-	-	-	-	-	-	-	-	-	-	-	-	-	-	-	-	-	-	-	-	-	-	-	-	-	-	-	-	-	-	-	-	-	-
		-	+	-	-	-	-	-	AT4G15475.1	23360	-	CE30418	133834	172092	208847	-	-	OTTDARP00000009471	-	-	-	-	ENSGALP00000019508	-	NP_036289.2	-	164359	-	-	-	-	Os11g42160.1	-	-	-	-	155414	-	-	RO3G_06247.1	-	-	GLEAN3_27743	SINFRUP00000161829	-	-	-	-	-	-	Tb927.3.580	UM01349.1
		-	-	-	-	-	-	-	-	-	-	-	-	172089	-	-	-	-	-	-	-	-	-	-	-	-	-	-	-	-	-	-	-	-	-	-	-	-	-	-	-	-	-	-	-	-	-	-	-	-	-	-
		-	-	-	-	-	-	-	-	-	-	-	-	172085	-	-	-	-	-	-	-	-	-	-	-	-	-	-	-	-	-	-	-	-	-	-	-	-	-	-	-	-	-	-	-	-	-	-	-	-	-	-
		-	-	-	-	-	-	-	-	-	-	-	-	172080	-	-	-	-	-	-	-	-	-	-	-	-	-	-	-	-	-	-	-	-	-	-	-	-	-	-	-	-	-	-	-	-	-	-	-	-	-	-
		-	-	-	-	-	-	-	-	-	-	-	-	172076	-	-	-	-	-	-	-	-	-	-	-	-	-	-	-	-	-	-	-	-	-	-	-	-	-	-	-	-	-	-	-	-	-	-	-	-	-	-
		-	-	-	-	-	-	-	-	-	-	-	-	172075	-	-	-	-	-	-	-	-	-	-	-	-	-	-	-	-	-	-	-	-	-	-	-	-	-	-	-	-	-	-	-	-	-	-	-	-	-	-
		-	-	-	-	-	-	-	-	-	-	-	-	172074	-	-	-	-	-	-	-	-	-	-	-	-	-	-	-	-	-	-	-	-	-	-	-	-	-	-	-	-	-	-	-	-	-	-	-	-	-	-
		-	-	-	-	-	-	-	-	-	-	-	-	172072	-	-	-	-	-	-	-	-	-	-	-	-	-	-	-	-	-	-	-	-	-	231106	-	-	-	-	-	-	-	-	-	-	-	-	-	-	-	-
		-	-	-	-	-	-	-	-	-	-	-	-	172065	-	-	-	-	-	-	-	-	-	-	-	-	-	-	-	-	-	-	2831	-	-	165977	-	-	-	-	-	-	-	-	-	-	-	-	-	-	-	-
		-	-	-	-	-	-	-	-	-	-	-	-	172060	-	-	-	-	-	-	-	-	-	-	-	-	-	-	-	-	-	-	-	-	-	-	-	-	-	-	-	-	-	-	-	-	-	-	-	-	-	-
		-	-	-	-	-	-	-	-	-	-	-	-	172054	-	-	-	-	-	-	-	-	-	-	-	-	-	-	-	-	-	-	-	-	-	-	-	-	-	RO3G_11158.1	-	-	-	-	-	-	-	-	-	-	-	UM04813.1
		-	-	-	-	-	-	-	-	-	-	-	-	172051	-	-	-	-	-	-	-	-	-	-	-	-	-	-	-	-	-	-	-	-	-	-	-	-	-	-	-	-	-	-	-	-	-	-	-	-	-	-
		-	-	-	-	-	-	-	-	-	-	-	-	172048	-	-	-	-	-	-	-	-	-	-	-	-	-	-	-	-	-	-	-	-	-	-	-	-	-	-	-	-	-	-	-	-	-	-	-	-	-	-
		-	-	-	-	-	-	-	-	-	-	-	-	172047	-	-	-	-	-	-	-	-	-	-	-	-	-	-	-	-	-	-	-	-	-	-	-	-	-	-	-	-	-	-	-	-	-	-	-	-	-	-
		-	+	-	-	-	-	-	AT5G23110.1	67157	BDEG_05556	-	195081	172046	-	-	-	-	DDB0190082	-	-	-	ENSGALP00000027606	-	OTTHUMP00000018111	-	164873	-	-	199235	-	-	-	-	-	129734	140064	-	558698	RO3G_07931.1	-	-	GLEAN3_28108	SINFRUP00000174895	-	-	-	-	-	-	-	-
		-	-	-	-	-	-	-	-	-	-	-	-	172039	-	-	-	-	-	-	-	-	-	-	-	-	-	-	-	-	-	-	-	-	-	-	-	-	-	-	-	-	-	-	-	-	-	-	-	-	-	-
		-	-	-	-	-	-	-	-	-	-	-	-	172033	-	-	-	-	-	-	-	-	-	-	-	-	-	-	-	-	-	-	-	-	-	-	-	-	-	-	-	-	-	-	-	-	-	-	-	-	-	-
		-	-	-	-	-	-	-	-	-	-	-	-	172026	-	-	-	-	-	-	-	-	-	-	-	-	-	-	-	-	-	-	-	-	-	-	-	-	-	-	-	-	-	-	-	-	-	-	-	-	-	-
		-	-	-	-	-	-	-	-	-	-	-	-	172025	-	-	-	-	-	-	-	-	-	-	-	-	-	-	-	-	-	-	-	-	-	-	-	-	-	-	-	-	-	-	-	-	-	-	-	-	-	-
		-	-	-	-	-	-	-	-	-	-	-	-	172018	-	-	-	-	-	-	-	-	-	-	-	-	-	-	-	-	-	-	-	-	-	-	-	-	-	-	-	-	-	-	-	-	-	-	-	-	-	-
		-	-	-	-	-	-	-	-	-	-	-	214819	171996	-	-	-	-	-	-	-	-	-	-	-	-	-	-	-	-	-	-	-	GSPATP00032271001	-	-	-	-	-	-	-	-	-	-	-	-	-	-	-	85859.m00379	-	-
		-	-	-	-	-	-	-	-	-	-	-	-	171994	-	-	-	-	-	-	-	-	-	-	-	-	-	-	-	-	-	-	-	-	-	-	-	-	-	-	-	-	-	-	-	-	-	-	-	-	-	-
		-	-	-	-	-	-	-	-	-	-	-	-	171993	-	-	-	-	-	-	-	-	-	-	-	-	-	-	-	-	-	-	-	-	-	-	-	-	-	-	-	-	-	-	-	-	-	-	-	-	-	-
		-	-	-	-	-	-	-	-	-	-	-	-	171992	-	-	-	-	-	-	-	-	-	-	-	-	-	-	-	-	-	-	-	-	-	-	-	-	-	-	-	-	-	-	-	-	-	-	-	-	-	-
		-	-	-	-	-	-	-	-	-	-	-	-	171990	-	-	-	-	-	-	-	-	-	-	-	-	-	-	-	-	-	-	-	-	-	-	-	-	-	-	-	-	-	-	-	-	-	-	-	-	-	-
		-	-	-	-	-	-	-	-	-	-	-	-	171981	-	-	-	-	-	-	-	-	-	-	-	-	-	-	-	-	-	-	-	-	-	-	-	-	-	-	-	-	-	-	-	-	-	-	-	-	-	-

		-	-	-	-	-	-	-	-	-	-	-	-	171976	-	-	-	-	-	-	-	-	-	-	-	-	-	-	-	-	-	-	-	-	-	-	-	-	-	-	-	-	-	-	-	-	-	-	-	-	-	-
		-	-	-	-	-	-	-	-	-	-	-	-	171973	-	-	-	-	-	-	-	-	-	-	-	-	-	-	-	-	-	-	-	-	-	-	-	-	-	-	-	-	-	-	-	-	-	-	-	-	-	-
		-	-	-	-	-	-	-	-	-	-	-	-	-	-	-	-	-	-	-	-	-	-	-	-	-	-	-	-	-	-	-	-	-	-	-	-	-	-	-	-	-	-	-	-	-	-	-	-	-	-	-
		-	-	-	-	-	-	-	-	-	-	-	-	171963	-	-	-	-	-	-	-	-	-	-	-	-	-	-	-	-	-	-	-	-	-	-	-	-	-	-	-	-	-	-	-	-	-	-	-	-	-	-
		-	-	-	-	-	-	-	-	-	-	-	-	171961	-	-	-	-	-	-	-	-	-	-	-	-	-	-	-	-	-	-	-	-	-	-	-	-	-	-	-	-	-	-	-	-	-	-	-	-	-	-
		-	-	-	-	-	-	-	-	-	-	-	-	171955	-	-	-	-	-	-	-	-	-	-	-	-	-	-	-	-	-	-	-	-	-	-	-	-	-	-	-	-	-	-	-	-	-	-	-	-	-	-
		-	+	-	-	-	-	-	AT5G50520.1	61312	BDEG_03687	-	-	171950	-	-	-	-	DDB0218873	-	-	5.m00449	-	-	-	LmjF11.0670	230330	38621	72846	-	-	Os08g42010.1	-	-	47069	42040	136318	-	298105	-	-	-	-	-	-	-	-	-	59935	-	Tb11.02.4420	-
		-	+	-	-	-	-	-	-	-	-	CE36588	228344	171949	-	cgd7_1580	-	-	-	-	-	33.m00224	-	-	-	-	-	-	-	108544	-	Os01g13150.1	-	GSPATP00000491001	-	216224	-	PF14_0620	829561	-	YKR079C	SPAC1D4.10	-	-	-	-	TA08320	46.m01672	-	96301.m00083	-	-
		-	-	-	-	-	-	-	-	-	-	-	-	171943	-	-	-	-	DDB0187806	-	-	-	-	-	-	LmjF04.0820	-	-	-	-	-	-	-	GSPATP00039585001	-	-	-	-	-	-	-	-	-	-	-	-	-	-	-	-	-	-
		-	-	-	-	-	-	-	-	-	-	-	-	171941	-	-	-	-	-	-	-	-	-	-	-	-	-	-	-	-	-	-	-	-	-	-	-	-	-	-	-	-	-	-	-	-	-	-	-	-	-	-
		-	-	-	-	-	-	-	-	-	-	-	-	171929	-	-	-	-	-	-	-	-	-	-	-	-	-	-	-	-	-	-	-	-	-	-	-	-	-	-	-	-	-	-	-	-	-	-	-	-	-	-
BRAT3401	Bromodomain protein with a AAA ATPase domain	-	+	-	-	-	-	GB16942-PA	AT1G05910.1	-	-	CE40608	211001	171910	-	-	CMB071C	-	DDB0218307	-	-	-	ENSGALP00000026359	-	OTTHUMP00000178229	-	213026	26358	68098	10407	NCU06484	Os09g33980.1	20741	-	-	185326	157176	-	594506	-	YGR270W	SPAC31G5.19	GLEAN3_01508	SINFRUP00000152287	-	-	-	-	58090	-	Tb11.02.4210	UM04657.1
		-	-	-	-	-	-	-	AT4G37020.1	-	-	-	-	171908	-	-	-	-	-	-	-	-	-	-	-	-	-	-	-	-	-	Os10g25000.1	-	-	-	172679	-	-	218699	-	-	-	-	-	-	-	-	-	-	-	-	-
		-	-	-	-	-	-	-	-	-	-	-	-	171899	-	-	-	-	-	-	-	-	-	-	-	-	-	27688	-	-	-	-	-	-	-	-	-	-	-	-	-	-	-	-	-	-	-	-	-	-	-	-
		-	+	-	-	-	-	-	AT5G48370.1	-	-	-	-	171897	-	-	-	-	-	-	-	-	-	-	-	-	-	-	-	-	-	Os09g34190.1	17478	-	-	199811	-	-	258022	-	-	-	-	-	-	14923	-	-	-	-	-	-
		-	-	-	-	-	-	-	-	-	-	-	-	171888	-	-	-	-	-	-	-	-	-	-	-	-	-	-	-	-	-	-	-	-	-	-	-	-	-	-	-	-	-	-	-	-	-	-	-	-	-	-
		-	-	-	-	-	-	-	-	-	-	-	-	171886	-	-	-	-	-	-	-	-	-	-	-	-	-	-	-	-	-	-	-	-	-	-	-	-	-	-	-	-	-	-	-	-	-	-	-	-	-	-
		-	-	-	-	-	-	-	-	-	-	-	-	171878	-	-	-	-	-	-	-	-	-	-	-	-	-	-	-	-	-	-	-	-	-	-	-	-	-	-	-	-	-	-	-	-	-	-	-	-	-	-
		-	-	-	-	-	-	-	-	-	-	-	-	171875	-	-	-	-	-	-	-	-	-	-	-	-	-	-	-	-	-	-	32153	-	-	-	-	-	-	-	-	-	-	-	-	-	-	-	-	-	-	-
		-	-	-	-	-	-	-	-	-	-	-	-	171865	-	-	-	-	-	-	-	-	-	-	-	-	-	-	-	-	-	-	-	-	-	-	-	-	-	-	-	-	-	-	-	-	-	-	-	-	-	-
		-	+	-	-	-	-	-	-	-	-	-	-	171861	-	-	-	-	-	-	-	-	-	-	OTTHUMP00000080196	-	134983	-	-	108619	-	-	-	-	-	-	-	-	-	-	-	-	-	SINFRUP00000156853	-	-	-	20.m03803	-	96955.m00134	-	-
		-	-	-	-	-	-	-	-	-	-	-	-	171854	-	-	-	-	-	-	-	-	-	-	-	-	-	-	-	-	-	-	-	-	-	-	-	-	-	-	-	-	-	-	-	-	-	-	-	-	-	-
		-	-	-	-	-	-	-	-	-	-	-	-	171847	-	-	-	-	-	-	-	-	-	-	-	-	-	-	-	-	-	-	-	-	-	-	-	-	-	-	-	-	-	-	-	-	-	-	-	-	-	-
		-	-	-	-	-	-	-	-	-	-	-	-	171846	-	-	-	-	-	-	-	-	-	-	-	-	-	-	-	-	-	-	-	-	-	-	-	-	-	-	-	-	-	-	-	-	-	-	-	-	-	-
		-	-	-	-	-	-	-	-	-	-	-	-	171845	-	-	-	-	-	-	-	-	-	-	-	-	-	-	-	-	-	-	-	-	-	-	-	-	-	-	-	-	-	-	-	-	-	-	-	-	-	-
		-	+	-	-	-	-	-	AT3G09080.1	-	BDEG_01899	-	22608	171844	260941	-	-	OTTDARP00000015746	-	-	-	-	-	-	NP_001077430.1	-	-	-	-	-	-	-	-	-	-	69976	158298	-	762468	RO3G_04817.1	-	-	-	-	-	-	-	-	-	-	-	-
		-	-	-	-	-	-	-	-	-	-	-	-	171842	-	-	-	-	-	-	-	-	-	-	-	-	-	-	-	-	-	-	-	-	-	-	-	-	-	-	-	-	-	-	-	-	-	-	-	-	-	-
		-	-	-	-	-	-	-	-	-	-	-	-	171833	-	-	-	-	-	-	-	-	-	-	-	-	-	-	-	-	-	-	-	-	-	-	-	-	-	-	-	-	-	-	-	-	-	-	-	-	-	-
		-	-	-	-	-	-	-	-	-	-	-	-	171830	-	-	-	-	-	-	-	-	-	-	-	-	-	9087	-	-	-	-	-	-	-	-	-	-	-	-	-	-	-	-	-	-	-	-	-	-	-	-
		-	+	-	-	-	-	-	AT3G54970.2	-	BDEG_00929	-	-	171827	-	-	-	-	-	-	-	-	-	-	-	-	-	-	-	-	-	Os02g15360.1	-	-	-	5780	136751	-	805566	-	-	-	-	-	-	-	-	74.m00431	-	-	-	-
		-	-	-	-	-	-	-	-	-	-	-	-	171826	-	-	-	-	-	-	-	-	-	-	-	-	-	-	-	-	-	-	-	-	-	-	-	-	-	-	-	-	-	-	-	-	-	-	-	-	-	-
		-	-	-	-	-	-	-	-	-	-	-	-	171825	-	-	-	-	-	-	-	-	-	-	-	-	-	-	-	-	-	-	-	-	-	-	-	-	-	-	-	-	-	-	-	-	-	-	-	-	-	-
		-	-	-	-	-	-	-	-	-	-	-	-	171815	-	-	-	-	-	-	-	-	-	-	-	-	-	-	-	-	-	-	-	-	-	-	-	-	-	-	-	-	-	-	-	-	-	-	-	-	-	-
		-	-	-	-	-	-	-	-	-	-	-	-	171802	-	-	-	-	-	-	-	-	-	-	-	-	-	-	-	-	-	-	-	-	-	-	-	-	-	-	-	-	-	-	-	-	-	-	-	-	-	-
		-	+	-	-	-	-	-	-	8230	-	-	-	171788	-	-	-	OTTDARP00000014522	DDB0219819	CG31690-PB	-	-	-	-	-	LmjF20.0150	116110	23301	-	-	-	-	-	-	32557	-	127123	-	-	-	-	-	-	-	-	-	-	-	-	-	-	-
		-	-	-	-	-	-	-	-	-	-	-	-	171768	-	-	-	-	-	-	-	-	-	-	-	-	-	-	-	-	-	-	-	-	-	-	-	-	-	-	-	-	-	-	-	-	-	-	-	-	-	-
		-	-	-	-	-	-	-	-	-	-	-	-	171767	-	-	-	-	-	-	-	-	-	-	-	-	-	-	-	-	-	-	-	-	-	-	-	-	-	-	-	-	-	-	-	-	-	-	-	-	-	-
CPH-like-2		-	-	-	-	-	-	-	-	-	-	-	-	171766	-	-	-	-	-	-	-	-	-	-	-	-	-	-	-	-	-	-	-	-	55091	-	-	-	-	-	-	-	-	-	-	-	-	-	-	-	-	-
		-	+	-	-	-	-	-	AT3G49600.1	30997	-	-	-	171763	-	-	-	-	-	-	-	78.m00162	ENSGALP00000022836	-	NP_001027902.1	-	-	-	-	107575	-	Os03g09260.1	-	GSPATP00013081001	-	116138	137424	-	818535	-	-	-	GLEAN3_08736	SINFRUP00000162633	-	8820	-	-	-	-	Tb927.8.5620	-
		-	-	-	-	-	-	-	-	-	-	-	-	171762	-	-	-	-	-	-	-	-	-	-	-	-	-	-	-	-	-	-	-	-	-	-	-	-	-	-	-	-	-	-	-	-	-	-	-	-	-	-
		-	+	-	-	-	-	-	AT4G24460.1	-	-	-	-	171760	-	-	CMM254C	-	-	-	-	-	-	-	-	-	-	-	-	-	-	Os01g72570.2	-	-	-	203314	-	-	202642	-	-	-	-	-	-	-	-	-	-	-	-	-
		-	-	-	-	-	-	-	-	-	-	-	-	171756	-	-	-	-	-	-	-	-	-	-	-	-	-	-	-	-	-	-	-	-	-	-	-	-	-	-	-	-	-	-	-	-	-	-	-	-	-	-
		-	-	-	-	-	-	-	-	-	-	-	-	171754	225207	-	-	-	-	-	-	-	-	-	-	-	-	-	-	-	-	-	-	-	-	-	-	-	-	-	-	-	-	-	-	-	-	-	-	-	-	-
		-	-	-	-	-	-	-	-	-	-	-	-	171751	-	-	-	-	-	-	-	-	-	-	-	-	-	-	-	-	-	-	-	-	-	-	-	-	-	-	-	-	-	-	-	-	-	-	-	-	-	-
		-	-	-	-	-	-	-	-	-	-	-	-	171741	-	-	-	-	-	-	-	-	-	-	-	-	-	-	-	-	-	-	-	-	-	-	-	-	-	-	-	-	-	-	-	-	-	-	-	-	-	-
		-	+	-	-	-	-	-	AT4G22970.1	-	-	CE22098	-	171732	-	-	-	-	-	-	-	-	-	-	NP_036423.4	-	-	-	-	241520	-	Os02g53120.1	-	-	-	-	-	-	756563	-	-	-	GLEAN3_12475	-	-	-	-	-	-	-	-	-
		-	-	-	-	-	-	-	-	-	-	-	-	171726	-	-	-	-	-	-	-	-	-	-	-	-	-	-	-	210306	-	-	-	-	-	-	-	-	-	-	-	-	-	-	-	-	-	-	-	-	-	-
		-	-	-	-	-	-	-	-	31866	-	-	-	171720	-	-	CMM122C	-	-	-	-	-	-	-	-	-	-	-	-	-	-	-	-	-	-	-	-	-	590633	-	-	-	-	-	-	-	-	541.m01237	-	-	-	-
		-	-	-	-	-	-	-	-	-	-	-	-	171716	-	-	-	-	-	-	-	-	-	-	-	-	-	-	-	-	-	-	-	-	-	-	-	-	-	-	-	-	-	-	-	-	-	-	-	-	-	-
		-	-	-	-	-	-	-	-	-	-	-	-	171709	-	-	-	-	-	-	-	-	-	-	-	-	-	-	-	-	-	-	-	-	-	-	-	-	-	-	-	-	-	-	-	-	-	-	-	-	-	-
		-	-	-	-	-	-	-	-	-	-	-	-	171691	-	-	-	-	-	-	-	-	-	-	-	-	-	-	-	-	-	-	-	-	-	-	137028	-	-	-	-	-	-	-	-	-	-	-	-	-	Tb09.211.1680	-
		-	+	-	-	-	-	GB13903-PA	-	63229	BDEG_07809	CE06267	180884	171688	289743	-	CMT174C	OTTDARP00000021642	-	CG1555-PA	-	-	ENSGALP00000012115	-	NP_003670.1	-	220321	33218	-	92823	NCU06924	-	32403	-	34964	163844	-	-	-	RO3G_06617.1	YBL098W	-	GLEAN3_12242	SINFRUP00000143708	-	-	-	-	24922	-	-	-
		-	-	-	-	-	-	-	-	-	-	-	-	171687	-	-	-	-	-	-	-	-	-	-	-	-	-	-	-	-	-	-	-	-	-	-	-	-	-	-	-	-	-	-	-	-	-	-	-	-	-	-
		-	+	-	-	-	-	-	-	27859	-	-	-	171682	-	cgd1_3690	-	-	-	-	-	-	-	-	-	-	-	-	-	-	-	-	-	GSPATP00010330001	46389	-	-	PF13_0133	-	-	-	-	-	-	15.m00421	7584	-	-	-	-	-	-
		-	-	-	-	-	-	-	-	-	BDEG_06994	-	-	171678	-	-	-	-	-	-	-	-	-	-	-	-	-	-	-	-	-	-	34318	-	-	-	139538	-	-	-	-	-	-	-	-	-	-	-	-	-	-	-
		-	-	-	-	-	-	-	-	-	-	-	-	171676	-	-	-	-	-	-	-	-	-	-	-	-	-	-	-	-	-	-	-	-	-	-	-	-	-	-	-	-	-	-	-	-	-	-	-	-	-	-
		-	-	-	-	-	-	-	-	-	-	-	-	171675	-	-	-	-	-	-	-	-	-	-	-	-	-	-	-	-	-	-	-	-	-	-	-	-	-	-	-	-	-	-	-	-	-	-	-	-	-	-
		-	+	-	-	-	-	GB10747-PA	AT1G04635.1	-	BDEG_00439	-	150632	171674	-	-	-	OTTDARP00000020995	DDB0219669	CG14057-PA	19171311	-	ENSGALP00000011525	-	OTTHUMP00000169480	-	231547	38368	-	240928	-	Os04g22870.1	7284	GSPATP00020510001	-	109940	-	-	740697	-	YAL033W	SPCC830.09c	-	-	-	-	-	-	27469	-	-	-
		-	-	-	-	-	-	-	-	-	-	-	-	171673	-	-	-	-	-	-	-	-	-	-	-	-	-	-	-	-	-	-	-	-	-	-	-	-	-	-	-	-	-	-	-	-	-	-	-	-	-	-
		-	-	-	-	-	-	-	-	-	-	-	-	171659	-	-	-	-	-	-	-	-	-	-	-	-	-	-	-	-	NCU00902	-	-	-	-	135573	-	-	-	-	-	-	-	-	-	-	-	-	-	-	-	-
RDP2	Rhodanese domain phosphatase	-	+	-	-	-	-	GB19251-PC	-	-	BDEG_02025	CE00429	140715	171654	277993	-	-	-	DDB0218521	-	19068790	-	ENSGALP00000007879	GL50803_4369	OTTHUMP00000171145	LmjF32.2740	147241	-	52839	109363	NCU06966	-	15399	-	-	-	134840	-	282198	RO3G_11220.1	YGR203W	SPBC839.07	-	-	-	-	-	-	-	91931.m00028	-	UM00277.1
		-	-	-	-	-	-	-	-	-	-	-	107949	171650	-	-	-	-	-	-	-	-	-	-	-	-	-	-	-	-	-	-	-	-	-	-	-	-	-	-	-	-	-	-	-	-	-	-	-	-	-	-
		-	+	-	-	-	-	-	-	-	-	-	220120	171647	-	-	-	-	-	-	-	-	-	-	NP_775901.3	-	231452	26842	-	212660	-	-	-	-	-	-	-	-	-	-	-	-	-	SINFRUP00000163023	-	-	-	-	52559	-	-	-
		-	+	-	-	-	-	-	-	-	-	CE31322	148520	171638	281271	-	-	-	DDB0187110	-	-	-	ENSGALP00000035850	-	XP_941447.2	LmjF19.1400	236543	34478	-	247741	-	-	32896	-	-	-	136613	-	-	-	-	-	-	-	-	-	-	-	57227	-	-	-
		-	-	-	-	-	-	-	-	-	-	-	-	171636	-	-	-	-	-	-	-	-	-	-	-	-	-	-	-	-	-	-	-	-	-	-	-	-	-	-	-	-	-	-	-	-	-	-	-	-	-	-
		-	-	-	-	-	-	-	-	-	-	-	-	171633	-	-	-	-	-	-	-	-	-	-	-	-	-	-	-	-	-	-	-	-	-	-	-	-	-	-	-	-	-	-	-	-	-	-	-	-	-	-
		-	-	-	-	-	-	-	-	-	-	-	-	171631	-	-	-	-	-	-	-	-	-	-	-	-	-	-	-	-	-	-	-	-	-	-	-	-	-	-	-	-	-	-	-	-	-	-	-	-	-	-
		-	-	-	-	-	-	-	-	-	-	-	-	171630	-	-	-	-	-	-	-	-	-	-	-	-	-	-	-	-	-	-	-	-	-	-	-	-	-	-	-	-	-	-	-	-	-	-	-	-	-	-
FAP234	Conserved Uncharacterized Flagellar Associated Protein	-	+	-	+	-	-	-	-	39219	BDEG_04372	-	137449	171628	269923	-	-	-	-	-	-	-	ENSGALP00000019566	-	-	LmjF24.0490	155146	-	-	50014	-	-	30150	GSPATP00027761001	-	76613	-	-	-	-	-	SPCC1739.11c	-	SINFRUP00000132121	63.m00201	-	-	55.m10248	10167	-	Tb11.02.2850	-
MCM5	minichromosome maintenance protein 5	-	+	+	-	-	-	GB12920-PA	AT2G07690.1	39241	BDEG_07765	CE03558	155362	171623	291004	cgd7_2920	CMF173C	-	DDB0219794	CG4082-PA	19074284	393.m00040	ENSGALP00000020463	GL50803_89112	NP_006730.2	LmjF24.0910	191134	27771	29938	238608	NCU01171	Os02g55410.1	16835	GSPATP00031570001	11490	152166	135552	PFL0580w	832008	RO3G_10698.1	YLR274W	SPAC1B2.05	GLEAN3_12431	SINFRUP00000154343	4.m00631	31609	TA06695	49.m00049	50365	97026.m00047	Tb11.02.3270	UM05064.1
		-	-	-	-	-	-	-	-	-	-	-	-	171618	-	-	-	-	-	-	-	-	-	-	-	-	-	-	-	-	-	-	-	-	-	-	-	-	-	-	-	-	-	-	-	-	-	-	-	-	-	-
		-	-	-	-	-	-	-	-	-	-	-	-	171615	-	-	-	-	-	-	-	-	-	-	-	-	-	-	-	-	-	-	-	-	-	-	-	-	-	-	-	-	-	-	-	-	-	-	-	-	-	-
		-	-	-	-	-	-	-	-	-	-	-	-	171614	-	-	-	-	-	-	-	-	-	-	-	-	-	-	-	-	-	-	-	-	-	-	-	-	-	-	-	-	-	-	-	-	-	-	-	-	-	-
		-	-	-	-	-	-	-	-	-	-	-	-	171609	-	-	-	-	-	-	-	-	-	-	-	-	-	-	-	-	-	-	-	-	-	-	-	-	-	-	-	-	-	-	-	-	-	-	-	-	-	-
		-	+	-	-	-	-	-	-	-	-	-	-	171608	-	-	-	-	DDB0204240	-	-	-	-	-	-	LmjF36.4400	-	34727	-	-	-	-	-	GSPATP00036436001	45727	-	129425	-	766517	-	-	-	GLEAN3_16488	-	10.m00294	42599	-	-	-	-	Tb10.6k15.0530	UM00242.1
MRPL3	Putative mitochondrial ribosomal protein L3, imported to mitochondria	-	+	-	-	-	-	GB13776-PA	AT3G17465.1	19295	BDEG_07799	CE39477	177551	171607	-	-	CMF065C	OTTDARP00000015192	DDB0188198	CG8288-PA	-	-	ENSGALP00000018917	-	OTTHUMP00000172798	LmjF29.0030	108795	23696	2751	128033	NCU06749	Os01g14830.1	29865	GSPATP00012557001	10617	124350	113670	PFL2180w	833354	RO3G_12202.1	YGR220C	SPAC644.17c	GLEAN3_14060	SINFRUP00000161065	91.m00158	35780	TA10270	44.m00018	3973	-	Tb927.3.5610	UM03529.1
		-	-	-	-	-	-	-	-	-	-	-	-	171600	-	-	-	-	-	-	-	-	-	-	-	-	-	-	-	-	-	-	-	-	-	-	-	-	-	-	-	-	-	-	-	-	-	-	-	-	-	-
		-	+	-	-	-	-	GB18386-PA	AT1G29220.1	-	-	-	-	171599	265077	-	-	-	-	-	-	-	-	-	-	-	159498	-	-	244938	-	Os01g55490.4	-	-	-	171312	156036	-	-	RO3G_08486.1	-	-	-	-	-	-	-	-	-	-	-	-
		-	-	-	-	-	-	-	-	-	-	-	-	171596	-	-	-	-	-	-	-	-	-	-	-	-	-	-	-	-	-	-	-	-	-	-	-	-	-	-	-	-	-	-	-	-	-	-	-	-	-	-
		-	+	-	-	-	-	-	AT5G45360.1	-	-	-	-	171590	218622	-	-	-	-	-	-	-	-	-	-	-	-	-	-	-	-	Os06g49530.1	-	-	-	131539	-	-	827502	-	-	-	-	-	-	-	-	-	53018	-	-	-
		-	-	-	-	-	-	-	-	-	-	-	-	171585	-	-	-	-	-	-	-	-	-	-	-	-	-	-	-	-	-	-	-	-	-	-	-	-	-	-	-	-	-	-	-	-	-	-	-	-	-	-
		-	+	-	-	-	-	-	AT2G37990.1	-	-	CE08186	-	171584	205289	-	-	OTTDARP00000022223	DDB0167740	-	-	-	-	-	-	-	-	-	-	108595	-	Os04g58830.1	-	-	-	169101	-	-	206051	-	-	-	-	-	-	-	-	-	-	-	-	-
		-	-	-	-	-	-	-	-	-	-	-	-	171580	-	-	-	-	-	-	-	-	-	-	-	-	-	-	-	-	-	-	-	-	-	-	-	-	-	-	-	-	-	-	-	-	-	-	-	-	-	-
		-	-	-	-	-	-	-	-	-	-	-	-	171578	-	-	-	-	-	-	-	-	-	-	-	-	-	-	-	-	-	-	-	-	-	-	-	-	-	-	-	-	-	-	-	-	-	-	-	-	-	-
		-	-	-	-	-	-	-	-	-	-	-	-	171570	231181	-	-	-	-	-	-	-	-	-	-	-	233095	-	-	-	-	-	-	-	-	96383	-	-	-	-	-	-	-	-	-	-	-	-	-	-	-	-
		-	-	-	-	-	-	-	-	-	-	-	-	171562	-	-	-	-	-	-	-	76.m00137	-	-	-	-	-	-	-	-	-	-	-	-	-	-	-	-	-	-	-	-	-	-	-	-	-	-	-	-	-	-
		-	-	-	-	-	-	-	-	-	-	-	-	171560	-	-	-	-	-	-	-	-	-	-	-	-	-	-	-	-	-	-	-	-	-	-	-	-	-	-	-	-	-	-	-	-	-	-	-	-	-	-
		-	-	-	-	-	-	-	-	-	-	-	-	171555	-	-	-	-	-	-	-	-	-	-	-	-	-	-	-	-	-	-	-	-	-	-	-	-	-	-	-	-	-	-	-	-	-	-	-	-	-	-
		-	+	-	-	-	-	-	AT5G13390.1	-	-	-	-	171536	-	-	-	-	DDB0192194	-	-	-	-	-	-	-	-	-	80771	-	-	Os11g32470.1	36193	-	-	121066	-	-	554672	-	-	-	-	-	-	-	-	-	-	-	Tb11.01.7670	-
		-	-	-	-	-	-	-	-	-	-	-	-	171534	-	-	-	-	-	-	-	-	-	-	-	-	-	-	-	-	-	-	-	-	-	-	-	-	-	-	-	-	-	-	-	-	-	-	-	-	-	-
		-	-	-	-	-	-	-	-	-	-	-	-	171532	-	-	-	-	-	-	-	-	-	-	-	-	-	-	-	-	-	-	-	-	-	-	-	-	-	-	-	-	-	-	-	-	-	-	-	-	-	-
		-	-	-	-	-	-	-	-	-	-	-	-	171527	-	-	-	-	-	-	-	-	-	-	-	-	-	26415	-	-	-	-	-	-	-	-	-	-	-	-	-	-	-	-	-	-	-	-	-	-	-	-
FKB99	peptidyl-prolyl cis-trans isomerase, FKBP-type	-	+	-	-	-	-	-	AT3G54010.1	-	-	-	-	171523	-	cgd4_400	-	-	-	-	-	-	-	-	-	-	-	-	-	-	-	Os03g25140.1	-	-	-	233642	-	-	735662	-	-	-	-	-	-	-	-	-	-	-	-	-
		-	-	-	-	-	-	-	-	-	-	-	-	171517	-	-	-	-	-	-	-	-	-	-	-	-	-	-	-	-	-	-	-	-	-	-	-	-	-	-	-	-	-	-	-	-	-	-	-	-	-	-
		-	-	-	-	-	-	-	-	-	-	-	-	171513	-	-	-	-	-	-	-	-	-	-	-	-	-	-	-	-	-	-	-	-	-	-	-	-	-	-	-	-	-	-	-	-	-	-	-	-	-	-
		-	+	-	-	-	-	-	AT1G11000.1	-	-	-	-	171506	-	-	-	-	-	-	-	-	-	-	-	-	-	-	-	-	-	Os11g07912.1	20776	-	-	126723	-	-	584470	-	-	-	-	-	-	-	-	-	-	-	-	-
		-	+	-	-	-	-	-	-	-	-	-	182182	171505	-	-	-	-	-	-	-	-	-	-	-	-	159996	-	-	-	-	-	-	-	39635	-	-	-	-	-	-	-	-	SINFRUP00000182584	-	1020	-	-	-	-	-	-
		-	-	-	-	-	-	-	-	-	-	-	-	171504	-	-	-	-	-	-	-	-	-	-	-	-	-	-	-	-	-	-	-	-	-	-	-	-	-	-	-	-	-	-	-	-	-	-	-	-	-	-
		-	-	-	-	-	-	-	-	-	-	-	-	171485	-	-	-	-	-	-	-	-	-	-	-	-	-	-	-	-	-	-	34582	-	-	-	-	-	-	-	-	-	-	-	-	-	-	-	-	-	-	-
		-	-	-	-	-	-	-	-	-	-	-	-	171484	-	-	-	-	-	-	-	-	-	-	-	-	-	-	-	-	-	-	-	-	-	-	-	-	-	-	-	-	-	-	-	-	-	-	-	-	-	-
		-	-	-	-	-	-	-	-	-	-	-	-	-	-	-	-	-	-	-	-	-	-	-	-	-	-	-	-	-	-	-	-	-	-	-	-	-	-	-	-	-	-	-	-	-	-	-	-	-	-	-
		-	+	-	-	-	-	-	AT1G65070.1	61371	-	-	-	171477	-	-	CML164C	-	-	-	-	-	-	-	-	-	-	-	-	-	-	Os04g58410.1	-	-	-	186791	-	-	198071	-	-	-	-	-	-	-	-	-	-	90371.m00073	-	-
		-	-	-	-	-	-	-	-	-	-	-	-	171444	-	-	-	-	-	-	-	-	-	-	-	-	-	-	-	-	-	-	-	-	43416	-	-	-	-	-	-	-	-	-	-	-	-	-	-	-	-	-
		-	-	-	-	-	-	-	-	-	-	-	-	171443	-	-	-	-	-	-	-	-	-	-	-	-	-	-	-	-	-	-	-	-	-	-	-	-	-	-	-	-	-	-	-	-	-	-	-	-	-	-
		-	-	-	-	-	-	-	-	-	-	-	-	171442	-	-	-	-	-	-	-	-	-	-	-	-	-	-	-	-	-	-	-	-	-	-	-	-	-	-	-	-	-	-	-	-	-	-	-	-	-	-
		-	-	-	-	-	-	-	AT5G46240.1	-	-	-	-	171437	-	-	-	-	-	-	-	-	-	-	-	-	-	-	-	-	-	Os06g14030.1	36077	-	-	-	-	-	-	-	-	-	-	-	-	-	-	-	-	-	-	-
		-	-	-	-	-	-	-	-	-	-	-	-	171435	-	-	-	-	-	-	-	-	-	-	-	-	-	-	-	-	-	-	-	-	-	-	-	-	-	-	-	-	-	-	-	-	-	-	-	-	-	-
PRA1	prenylated rab acceptor (PRA1) family protein	-	+	-	-	-	-	-	AT5G01640.1	-	-	-	-	171434	-	-	-	-	DDB0206263	-	-	-	-	-	NP_006414.1	LmjF33.1530	153327	33098	-	-	-	Os05g39670.1	12748	-	-	196148	-	-	832605	-	-	-	-	SINFRUP00000130719	-	-	-	-	-	-	Tb10.389.1550	-
		-	+	-	-	-	-	GB18736-PA	-	-	-	-	-	171432	269989	-	-	-	-	-	-	-	-	-	-	-	125574	6887	-	-	-	-	13439	-	36043	-	-	-	-	-	-	-	-	-	-	-	-	86.m00840	-	-	-	UM05564.1
		-	+	-	-	-	-	-	AT5G64420.1	-	-	-	-	171431	-	-	-	-	-	-	-	-	-	-	-	-	-	9230	-	240401	NCU02497	Os02g04270.1	32476	-	-	78751	-	-	-	RO3G_04646.1	-	SPBC14C8.14c	-	-	-	-	-	-	-	-	-	UM00275.1
ARS4		-	-	-	-	-	-	-	-	-	-	-	-	171420	-	-	-	-	-	-	-	-	-	-	-	-	-	-	-	-	-	-	-	-	-	-	-	-	-	-	-	-	-	SINFRUP00000146394	-	-	-	-	-	-	-	-
		-	-	-	-	-	-	-	-	-	-	-	-	171414	-	-	-	-	-	-	-	-	-	-	-	-	-	-	-	-	-	-	-	-	-	-	-	-	-	-	-	-	-	-	-	-	-	-	-	-	-	-
		-	+	-	-	-	-	GB12305-PA	AT4G20280.1	-	BDEG_07615	CE07258	182628	171402	255825	-	CMH222C	-	DDB0206231	CG4079-PA	19068686	-	ENSGALP00000004305	-	XP_373058.2	-	133658	-	45427	138459	-	Os01g48810.1	15516	-	48008	147903	137221	-	179507	-	-	SPAC1002.04c	GLEAN3_08669	SINFRUP00000150490	-	-	-	-	52029	-	-	-
		-	-	-	-	-	-	-	-	-	-	-	-	171400	-	-	-	-	-	-	-	-	-	-	-	-	-	-	-	-	-	-	-	-	-	-	-	-	-	-	-	-	-	-	-	-	-	-	-	-	-	-
		-	+	-	-	-	-	-	AT1G55300.2	-	-	-	-	171399	294496	-	-	-	-	-	-	113.m00149	-	-	-	-	-	-	-	-	-	Os05g28010.1	-	GSPATP00003765001	-	18130	139969	-	639652	RO3G_11871.1	-	-	-	-	-	-	-	-	-	-	-	-
		-	-	-	-	-	-	-	-	-	-	-	-	171398	-	-	-	-	-	-	-	-	-	-	-	-	-	-	-	-	-	-	-	-	-	-	-	-	-	-	-	-	-	-	-	-	-	-	-	-	-	-
		-	-	-	-	-	-	-	-	-	-	-	-	171392	-	-	-	-	-	-	-	-	-	-	-	-	-	-	-	-	-	-	-	-	-	-	-	-	-	-	-	-	-	-	-	-	-	-	-	-	-	-
		-	-	-	-	-	-	-	-	-	-	-	-	171390	-	-	-	-	-	-	-	-	-	-	-	-	-	-	-	-	-	-	-	-	-	-	-	-	-	-	-	-	-	-	-	-	-	-	-	-	-	-
		-	-	-	-	-	-	-	-	-	-	-	-	171386	-	-	-	-	-	-	-	-	-	-	-	-	-	-	-	-	-	-	-	-	-	-	-	-	-	-	-	-	-	-	-	-	-	-	-	-	-	-
		-	-	-	-	-	-	-	-	-	-	-	-	171378	-	-	-	-	-	-	-	-	-	-	-	-	-	-	-	-	-	-	-	-	-	-	-	-	-	-	-	-	-	-	-	-	-	-	-	-	-	-
		-	-	-	-	-	-	-	AT1G07910.1	-	-	-	-	171374	-	-	-	-	-	-	-	-	-	-	-	-	-	-	-	-	-	Os07g09400.1	-	-	46676	125536	-	-	-	-	-	-	-	-	-	-	-	-	-	-	-	-
		-	-	-	-	-	-	-	-	-	-	-	-	171371	-	-	-	-	-	-	-	-	-	-	-	-	-	-	-	-	-	-	-	-	-	-	-	-	-	-	-	-	-	-	-	-	-	-	-	-	-	-
		-	-	-	-	-	-	-	-	-	-	-	-	171369	-	-	-	-	-	-	-	-	-	-	-	-	-	-	-	-	-	-	-	-	-	-	-	-	-	-	-	-	-	-	-	-	-	-	-	-	-	-
		-	-	-	-	-	-	-	-	-	-	-	-	171361	-	-	-	-	-	-	-	-	-	-	-	-	-	-	-	-	-	-	-	-	-	-	-	-	-	-	-	-	-	-	-	-	-	-	-	-	-	-
		-	-	-	-	-	-	-	-	-	-	-	-	171360	-	-	-	-	-	-	-	-	-	-	-	-	-	-	-	-	-	-	-	-	-	-	-	-	-	-	-	-	-	-	-	-	-	-	-	-	-	-
		-	-	-	-	-	-	-	-	-	-	-	-	171355	-	-	-	-	-	-	-	-	-	-	-	-	-	-	-	-	-	-	-	-	-	-	-	-	-	-	-	-	-	-	-	-	-	-	-	-	-	-
		-	-	-	-	-	-	-	-	-	-	-	-	171354	-	-	-	-	-	-	-	-	-	-	-	-	-	-	-	-	-	-	-	-	-	-	-	-	-	-	-	-	-	-	-	-	-	-	-	-	-	-
		-	-	-	-	-	-	-	-	-	-	-	-	171353	-	-	-	-	-	-	-	-	-	-	-	-	-	-	-	-	-	-	-	-	-	-	-	-	-	-	-	-	-	-	-	-	-	-	-	-	-	-
		-	-	-	-	-	-	-	-	-	-	-	-	171348	-	-	-	-	-	-	-	-	-	-	-	-	-	-	-	-	-	-	-	-	-	22197	-	-	-	-	-	-	-	-	-	-	-	-	-	-	-	-
		-	+	-	-	-	-	GB18886-PA	AT2G30350.2	-	BDEG_02310	CE11216	226573	171346	239033	cgd2_4280	CMC047C	-	-	CG18271-PA	-	100.m00123	-	GL50803_16475	OTTHUMP00000045748	LmjF25.1330	163304	8836	-	174298	NCU01236	Os07g12710.1	37060	-	-	93627	135180	-	250296	-	YBR228W	SPAP27G11.15	-	SINFRUP00000140913	-	2122	-	28.m00297	-	83584.m00134	Tb927.3.1220	UM01857.1
		-	+	-	-	-	-	GB13899-PA	AT2G33560.1	68540	BDEG_04106	-	118720	171345	254994	-	CMK144C	-	DDB0184518	CG7838-PA	-	-	ENSGALP00000039721	-	NP_001202.4	-	185215	32082	66972	83438	NCU10043	Os02g10020.1	20479	-	10954	148229	155828	-	550712	-	YJL013C	SPCC1795.01c	GLEAN3_04518	SINFRUP00000165671	42.m00283	261219	-	-	55391	-	Tb09.160.0630	UM06142.1
		-	-	-	-	-	-	-	-	-	-	-	-	171344	-	-	-	-	-	-	-	-	-	-	-	-	-	-	-	-	-	-	-	-	-	-	-	-	-	-	-	-	-	-	-	-	-	-	-	-	-	-
		-	-	-	-	-	-	-	-	-	-	-	-	171338	-	-	-	-	-	-	-	-	-	-	-	-	-	-	-	-	-	-	-	-	-	-	-	-	-	-	-	-	-	-	-	-	-	-	-	-	-	-
		-	-	-	-	-	-	-	-	-	-	-	-	171324	-	-	-	-	-	-	-	-	-	-	-	-	-	-	-	-	-	-	-	-	-	-	-	-	-	-	-	-	-	-	-	-	-	-	-	-	-	-
		-	-	-	-	-	-	-	-	-	-	-	-	171323	-	-	-	-	-	-	-	-	-	-	-	-	-	-	-	-	-	-	-	-	-	-	-	-	-	-	-	-	-	-	-	-	-	-	-	-	-	-
		-	+	-	-	-	-	-	-	-	-	CE20701	-	171322	-	-	-	-	-	-	-	-	-	-	-	-	158685	28079	-	-	-	-	37013	-	-	-	-	-	-	-	-	-	-	-	-	-	-	-	54670	-	-	-
		-	-	-	-	-	-	-	AT3G51970.1	-	-	-	-	171320	-	-	-	-	-	-	-	-	-	-	-	-	-	-	-	-	-	Os04g20880.1	-	-	-	161387	-	-	-	-	-	-	-	-	-	-	-	-	-	-	-	-
		-	-	-	-	-	-	-	-	-	-	-	-	171316	-	-	-	-	-	-	-	-	-	-	-	-	-	-	-	-	-	-	-	-	-	-	-	-	-	-	-	-	-	-	-	-	-	-	-	-	-	-
		-	-	-	-	-	-	-	-	-	-	-	-	171313	-	-	-	-	-	-	-	-	-	-	-	-	-	-	-	-	-	-	-	-	-	-	-	-	-	-	-	-	-	-	-	-	-	-	-	-	-	-
		-	-	-	-	-	-	-	-	-	-	-	-	171307	-	-	-	-	-	-	-	-	-	-	-	-	-	-	-	-	-	-	-	-	-	-	-	-	-	-	-	-	-	-	-	-	-	-	-	-	-	-
		-	-	-	-	-	-	-	-	-	-	-	-	171304	-	-	-	-	-	-	-	-	-	-	-	-	-	-	-	-	-	-	-	-	-	-	-	-	-	-	-	-	-	-	-	-	-	-	-	-	-	-
		-	-	-	-	-	-	-	AT2G21195.1	-	-	-	-	171285	-	-	-	-	-	-	-	-	-	-	-	-	-	-	-	-	-	Os07g16150.1	-	-	-	-	-	-	249374	-	-	-	-	-	-	-	-	-	-	-	-	-
		-	-	-	-	-	-	-	-	-	-	-	-	171284	-	-	-	-	-	-	-	-	-	-	-	-	-	-	-	-	-	-	-	-	-	-	-	-	-	-	-	-	-	-	-	-	-	-	-	-	-	-
		-	-	-	-	-	-	-	-	-	-	-	-	171283	-	-	-	-	-	-	-	-	-	-	-	-	-	-	-	-	-	-	-	-	-	-	-	-	-	-	-	-	-	-	-	-	-	-	-	-	-	-
		-	-	-	-	-	-	-	-	-	-	-	-	-	-	-	-	-	-	-	-	-	-	-	-	-	-	-	-	-	-	-	-	-	-	-	-	-	-	-	-	-	-	-	-	-	-	-	-	-	-	-
		-	-	-	-	-	-	-	-	-	-	-	-	171281	-	-	-	-	-	-	-	-	-	-	-	-	-	-	-	-	-	-	-	-	-	-	-	-	-	-	-	-	-	-	-	-	-	-	-	-	-	-
		-	-	-	-	-	-	-	-	-	-	-	-	171276	-	-	-	-	-	-	-	-	-	-	-	-	-	-	-	-	-	-	-	-	-	-	-	-	-	-	-	-	-	-	-	-	-	-	-	-	-	-
		-	-	-	-	-	-	-	-	-	-	-	-	171274	-	-	-	-	-	-	-	-	-	-	-	-	-	-	-	-	-	-	-	-	-	-	-	-	-	-	-	-	-	-	-	-	-	-	-	-	-	-
		-	-	-	-	-	-	-	-	-	-	-	-	171258	-	-	-	-	-	-	-	-	-	-	-	-	-	-	-	-	-	-	30252	-	-	-	-	-	-	-	-	-	-	-	-	-	-	-	-	-	-	-
		-	-	-	-	-	-	-	-	-	-	-	-	171257	-	-	-	-	-	-	-	-	-	-	-	-	-	-	-	-	-	-	-	-	-	-	-	-	-	-	-	-	-	-	-	-	-	-	-	-	-	-
		-	-	-	-	-	-	-	-	-	-	-	-	171253	-	-	-	-	-	-	-	-	ENSGALP00000011584	-	-	-	-	-	-	-	-	-	-	-	-	-	-	-	-	-	-	-	-	-	-	-	-	-	-	-	-	-
		-	+	-	-	-	-	-	AT3G56570.1	-	BDEG_02003	-	-	171251	-	-	-	-	DDB0167139	-	-	-	-	-	-	-	-	37907	-	-	NCU02158	Os01g65730.1	37391	-	48815	121200	-	-	199501	-	-	SPBC1709.13c	-	-	-	-	-	-	-	-	-	-
		-	-	-	-	-	-	-	-	-	-	-	-	171239	-	-	-	-	-	-	-	-	-	-	-	-	-	-	-	-	-	-	-	-	-	-	-	-	-	-	-	-	-	-	-	-	-	-	-	-	-	-
		-	-	-	-	-	-	-	-	-	-	-	-	171226	-	-	-	-	-	-	-	-	-	-	-	-	-	-	-	-	-	-	-	GSPATP00033579001	-	-	-	-	-	-	-	-	-	-	-	-	-	-	-	-	-	-
		-	-	-	-	-	-	-	-	-	-	-	-	171225	-	-	-	-	-	-	-	-	-	-	-	-	-	-	-	-	-	-	-	-	-	-	-	-	-	-	-	-	-	-	-	-	-	-	-	-	-	-
		-	-	-	-	-	-	-	-	-	-	-	-	171221	-	-	-	-	-	-	-	-	-	-	-	-	-	-	-	-	-	-	-	-	-	-	-	-	-	-	-	-	-	-	-	-	-	-	-	-	-	-
																								-	-	-	-	-	-	-	Os03g64030.1	-	-	-	177306	-	-	649618	-	-	-	-	-	-	-	-	-	-	-	-	-

		-	-	-	-	-	-	-	-	-	-	-	-	171220	-	-	-	-	-	-	-	-	-	-	-	-	-	-	-	-	-	-	-	-	-	-	-	-	-	-	-	-	-	-	-	-	-	-	-	-	-	-
		-	-	-	-	-	-	-	-	-	-	-	-	171213	-	-	-	-	-	-	-	-	-	-	-	-	-	-	-	-	-	-	-	-	-	-	-	-	-	-	-	-	-	-	-	-	-	-	-	-	-	-
		-	+	-	-	-	-	-	-	63493	-	-	218148	171212	-	-	CMM102C	-	-	-	-	-	-	-	-	LmjF09.0590	-	8248	-	226051	NCU04733	Os07g30980.1	756	-	-	158274	-	-	581274	RO3G_12875.1	YJL092W	SPAC4H3.05	GLEAN3_11270	-	-	262303	-	-	62806	90175.m00092	-	UM01691.1
		-	-	-	-	-	-	-	-	-	-	-	-	171211	-	-	-	-	-	-	-	-	-	-	-	-	-	-	-	-	-	-	-	-	-	-	-	-	-	-	-	-	-	-	-	-	-	-	-	-	-	-
		-	-	-	-	-	-	-	-	-	-	-	-	171205	-	-	-	-	-	-	-	-	-	-	-	-	-	-	-	-	-	-	-	-	-	-	-	-	-	-	-	-	-	-	-	-	-	-	-	-	-	-
		-	-	-	-	-	-	-	-	-	-	-	-	171199	-	-	-	-	-	-	-	-	-	-	-	-	-	-	-	-	-	-	-	-	-	-	-	-	-	-	-	-	-	-	-	-	-	-	-	-	-	-
		-	-	-	-	-	-	-	-	-	-	-	-	171195	-	-	-	-	-	-	-	-	-	-	-	-	-	-	-	-	-	-	-	-	-	-	-	-	-	-	-	-	-	-	-	-	-	-	-	-	-	-
		-	-	-	-	-	-	-	-	-	-	-	-	171193	-	-	-	-	-	-	-	-	-	-	-	-	-	-	-	188066	-	-	-	-	-	-	-	-	-	-	-	-	-	-	-	-	-	-	-	-	-	-
		-	-	-	-	-	-	-	-	-	-	-	-	171186	-	-	-	-	-	-	-	-	-	-	-	-	-	-	-	-	-	-	-	-	-	-	-	-	-	-	-	-	-	-	-	-	-	-	-	-	-	-
		-	-	-	-	-	-	-	-	-	-	-	-	171176	-	-	-	-	-	-	-	-	-	-	-	-	-	-	-	-	-	Os09g32190.1	-	-	-	-	-	-	-	-	-	-	-	-	-	-	-	-	-	-	-	-
		-	-	-	-	-	-	-	-	-	-	-	-	171171	-	-	-	-	-	-	-	-	-	-	-	-	-	-	-	-	-	-	-	-	-	-	-	-	-	-	-	-	-	-	-	-	-	-	-	-	-	-
PKHD1-1	Polycystic kidney and hepatic disease gene product fibrocystin	+	+	-	+	-	-	-	-	-	-	-	226160	171165	269711	-	-	OTTDARP00000014459	-	-	-	-	-	-	OTTHUMP00000177980	-	131466	-	-	50043	-	-	-	-	-	-	-	-	-	-	-	-	GLEAN3_15359	SINFRUP00000162185	-	24694	-	-	58466	-	-	-
		-	-	-	-	-	-	-	-	-	-	-	-	171152	-	-	-	-	-	-	-	-	-	-	-	-	-	-	-	-	-	-	-	-	-	-	-	-	-	-	-	-	-	-	-	-	-	-	-	-	-	-
		-	-	-	-	-	-	-	-	-	-	-	-	171151	-	-	-	-	-	-	-	-	-	-	-	-	-	-	-	-	-	-	-	-	-	-	-	-	-	-	-	-	-	-	-	-	-	-	-	-	-	-
		-	-	-	-	-	-	-	-	-	-	-	-	171146	-	-	-	-	-	-	-	-	-	-	-	-	-	-	-	-	-	-	-	-	-	-	-	-	-	-	-	-	-	-	-	-	-	-	-	-	-	-
		-	-	-	-	-	-	-	-	-	-	-	-	171142	-	-	-	-	-	-	-	-	-	-	-	-	-	-	-	-	-	-	-	-	-	-	-	-	-	-	-	-	-	-	-	-	-	-	-	-	-	-
		-	+	-	-	-	-	-	-	-	-	-	-	171141	-	-	-	-	-	-	-	-	-	-	OTTHUMP00000014153	-	-	31000	-	-	-	Os06g51360.1	-	-	-	64160	-	-	422780	-	-	-	-	-	-	-	-	-	-	-	-	-
FAP206	Flagellar Associated Protein	-	+	-	+	-	-	-	-	-	-	-	163417	171124	250712	-	-	-	-	-	-	-	-	-	NP_849145.1	-	-	-	-	20990	-	-	-	GSPATP00026758001	-	-	137007	-	-	-	-	-	-	-	139.m00128	-	-	-	-	-	-	-
		-	-	-	-	-	-	-	-	-	-	-	-	171120	-	-	-	-	-	-	-	-	-	-	-	-	-	-	-	-	-	-	-	-	-	-	-	-	-	-	-	-	-	-	-	-	-	-	-	-	-	-
		-	-	-	-	-	-	-	-	-	-	-	-	171119	-	-	-	-	-	-	-	-	-	-	-	-	-	-	-	-	-	-	-	-	50141	-	-	-	-	-	-	-	-	-	-	-	-	-	-	-	-	-
		-	-	-	-	-	-	-	-	60024	-	-	-	171117	-	-	-	-	-	-	-	-	-	-	-	-	-	-	-	-	-	-	-	-	-	-	-	-	-	-	-	-	-	-	-	-	-	-	-	-	-	-
		-	-	-	-	-	-	-	-	-	-	-	-	-	-	-	-	-	-	-	-	-	-	-	-	-	-	-	-	-	-	-	-	-	-	-	-	-	-	-	-	-	-	-	-	-	-	-	-	-	-	-
		-	-	-	-	-	-	-	-	-	-	-	-	171114	-	-	-	-	-	-	-	-	-	-	-	-	-	-	-	-	-	-	-	-	-	-	-	-	-	-	-	-	-	-	-	-	-	-	-	-	-	-
		-	-	-	-	-	-	-	-	-	-	-	-	171113	-	-	-	-	-	-	-	-	-	-	-	-	-	-	-	-	-	-	-	-	-	-	-	-	-	-	-	-	-	-	-	-	-	-	-	-	-	-
		-	-	-	-	-	-	-	-	-	-	-	-	171106	-	-	-	-	-	-	-	-	-	-	-	-	-	-	-	-	-	-	-	-	-	-	-	-	-	-	-	-	-	-	-	-	-	-	-	-	-	-
		-	-	-	-	-	-	-	-	-	-	-	-	171105	-	-	-	-	-	-	-	-	-	-	-	-	-	-	-	-	-	-	-	-	-	-	-	-	-	-	-	-	-	-	-	-	-	-	-	-	-	-
		-	-	-	-	-	-	-	-	-	BDEG_04618	-	-	171104	-	-	-	-	DDB0187902	-	-	-	-	-	-	-	-	-	-	-	-	-	-	-	-	101657	-	-	410535	-	-	-	-	-	-	-	-	-	-	-	-	-
		-	-	-	-	-	-	-	AT1G22100.1	-	-	-	-	171103	-	-	-	-	-	-	-	-	-	-	-	-	-	-	-	-	-	-	-	-	-	119257	-	-	-	-	-	-	-	-	-	-	-	-	-	-	-	-
		-	-	-	-	-	-	-	-	-	-	-	-	171087	-	-	-	-	-	-	-	-	-	-	-	-	-	-	-	-	-	-	-	-	-	-	-	-	-	-	-	-	-	-	-	-	-	-	-	-	-	-
		-	-	-	-	-	-	-	AT1G56230.1	-	-	-	-	171083	-	-	-	-	-	-	-	-	-	-	-	-	-	-	-	-	-	-	-	-	-	-	-	-	-	-	-	-	-	-	-	-	-	-	-	-	-	-
		-	-	-	-	-	-	-	-	-	-	-	-	171080	-	-	-	-	-	-	-	-	-	-	-	-	-	-	-	-	-	-	-	-	-	-	-	-	-	-	-	-	-	-	-	-	-	-	-	-	-	-
	matrix metalloproteinase-like protein	-	-	-	-	-	-	-	-	-	-	-	-	171078	-	-	-	-	-	-	-	-	-	-	-	-	-	-	-	-	-	-	-	-	-	-	-	-	-	-	-	-	-	-	-	-	-	-	-	-	-	-
		-	-	-	-	-	-	-	-	-	-	-	-	171058	-	-	-	-	-	-	-	-	-	-	-	-	-	-	-	-	-	-	-	-	-	-	-	-	-	-	-	-	-	-	-	-	-	-	-	-	-	-
		-	-	-	-	-	-	-	-	-	-	-	-	171055	-	-	-	-	-	-	-	-	-	-	-	-	-	-	-	-	-	-	-	-	-	-	-	-	-	-	-	-	-	-	-	-	-	-	-	-	-	-
		-	-	-	-	-	-	-	-	-	BDEG_01746	-	-	171053	-	-	-	-	-	-	-	-	-	-	-	-	-	-	-	-	-	-	-	-	-	-	-	-	-	-	-	-	-	-	-	-	-	-	-	-	-	-
BRD3401	Bromodomain containing protein	-	+	-	-	-	-	GB11114-PA	AT5G55040.1	71405	BDEG_04406	CE05190	220552	171050	-	-	-	OTTDARP00000023208	-	CG7154-PA	-	-	ENSGALP00000020415	-	NP_001009877.1	LmjF36.2980	238111	-	-	200933	-	-	23520	-	-	-	143076	-	-	-	-	-	-	SINFRUP00000139859	-	-	-	-	52538	81679.m00130	Tb10.6k15.3240	-
		-	+	-	-	-	-	-	-	18663	BDEG_07976	-	-	171044	-	-	-	-	-	-	-	-	-	-	-	-	-	-	-	-	NCU10480	-	18068	-	13606	135503	137107	PFL0305c	-	-	YOR155C	SPBC30D10.03c	-	-	-	34077	-	41.m00024	-	-	-	-
		-	-	-	-	-	-	-	-	-	-	-	-	171032	-	-	-	-	-	-	-	-	-	-	-	-	-	-	-	-	-	-	-	-	-	-	-	-	-	-	-	-	-	-	-	-	-	-	-	-	-	-
		-	-	-	-	-	-	-	-	-	-	-	-	171031	-	-	-	-	-	-	-	-	-	-	-	-	-	-	-	-	-	-	-	-	-	-	-	-	-	-	-	-	-	-	-	-	-	-	-	-	-	-
		-	-	-	-	-	-	-	-	-	-	-	-	171030	-	-	-	-	-	-	-	-	-	-	-	-	-	-	-	-	-	-	-	-	-	-	-	-	-	-	-	-	-	-	-	-	-	-	-	-	-	-
		-	-	-	-	-	-	-	-	-	-	-	-	171026	-	-	-	-	-	-	-	-	-	-	-	-	-	-	-	-	-	-	-	-	-	-	-	-	-	-	-	-	-	-	-	-	-	-	-	-	-	-
		-	-	-	-	-	-	-	-	-	-	-	-	171021	-	-	-	-	-	-	-	-	-	-	-	-	-	-	-	-	-	-	-	-	-	-	-	-	-	-	-	-	-	-	-	-	-	-	-	-	-	-
		-	-	-	-	-	-	-	-	-	-	-	-	171013	-	-	CMS084C	-	DDB0168244	-	-	-	-	-	-	-	-	-	-	-	-	-	30211	-	-	-	-	-	-	-	-	-	-	-	-	-	-	-	-	-	-	-
		-	+	-	-	-	-	GB18368-PA	AT1G31600.3	-	-	-	156565	171012	241199	-	-	-	-	-	-	-	-	-	-	-	-	-	-	235894	-	Os04g51360.1	-	-	-	15669	-	-	414000	-	-	-	-	SINFRUP00000151453	-	-	-	-	26003	-	-	-
		-	-	-	-	-	-	-	-	-	-	-	-	171001	-	-	-	-	-	-	-	-	-	-	-	-	-	-	-	-	-	-	-	-	-	-	-	-	-	-	-	-	-	-	-	-	-	-	-	-	-	-
		-	-	-	-	-	-	-	-	-	-	-	-	170997	-	-	-	-	-	-	-	-	-	-	-	-	-	-	-	-	-	-	-	-	-	-	-	-	-	-	-	-	-	-	-	-	-	-	-	-	-	-
CLR18	Coxiella-Like Repeat protein	-	-	-	-	-	-	-	-	-	-	-	-	170988	-	-	-	-	-	-	-	-	-	-	-	-	-	-	-	-	-	-	-	-	-	-	-	-	-	-	-	-	-	-	-	-	-	-	-	-	-	-
		-	+	-	-	-	-	-	-	-	-	-	-	170982	228974	-	-	-	-	-	-	-	ENSGALP00000034129	-	NP_059973.2	-	-	-	-	-	-	-	-	GSPATP00021400001	-	-	-	-	-	-	-	-	-	SINFRUP00000137720	-	-	-	-	-	-	-	-
		-	-	-	-	-	-	-	-	-	-	-	-	170976	-	-	-	-	-	-	-	-	-	-	-	-	-	-	-	-	-	-	-	-	-	-	-	-	-	-	-	-	-	-	-	-	-	-	-	-	-	-
		-	-	-	-	-	-	-	-	-	-	-	-	170973	-	-	-	-	-	-	-	-	-	-	-	-	-	-	-	-	-	-	-	-	-	-	-	-	-	-	-	-	-	-	-	-	-	-	-	-	-	-
		-	-	-	-	-	-	-	-	-	-	-	-	170972	-	-	-	-	-	-	-	-	-	-	-	-	-	-	-	-	-	-	-	-	-	-	-	-	-	-	-	-	-	-	-	-	-	-	-	-	-	-
		-	-	-	-	-	-	-	-	-	-	-	-	170971	-	-	-	-	-	-	-	-	-	-	-	-	-	-	-	-	-	-	-	-	-	-	-	-	-	-	-	-	-	-	-	-	-	-	-	-	-	-
		-	+	-	-	-	-	GB19196-PA	AT4G31200.1	-	-	-	228166	170970	-	-	-	-	-	CG33522-PB	-	-	-	-	-	-	95622	-	-	-	-	Os06g46880.1	-	-	-	41668	-	-	586364	-	-	-	GLEAN3_05681	-	-	-	-	645.m00322	-	-	-	-
		-	-	-	-	-	-	-	-	-	-	-	-	170967	-	-	-	-	-	-	-	-	-	-	-	-	-	-	-	-	-	-	-	-	-	-	-	-	-	-	-	-	-	-	-	-	-	-	-	-	-	-
		-	-	-	-	-	-	-	-	-	-	-	-	170956	-	-	-	-	-	-	-	-	-	-	-	-	-	-	-	-	-	-	-	-	-	-	-	-	-	-	-	-	-	-	-	-	-	-	-	-	-	-
		-	+	-	-	-	-	-	AT2G32640.1	32203	-	-	-	170954	-	-	CMC032C	-	-	-	-	-	-	-	-	-	-	-	-	-	-	Os08g32630.4	15078	-	3296	-	-	-	664770	-	-	-	-	-	-	-	-	-	-	-	-	-
		-	+	-	-	-	-	-	AT5G09390.2	-	-	-	-	170950	-	-	-	-	-	-	-	-	-	-	-	-	-	-	-	-	-	Os09g20590.1	-	-	-	171099	-	-	712237	-	-	-	-	-	-	-	-	35.m00932	28719	-	-	-
		-	-	-	-	-	-	-	-	-	-	-	-	170949	-	-	-	-	-	-	-	-	-	-	-	-	-	-	-	-	-	-	-	-	-	-	-	-	-	-	-	-	-	-	-	-	-	-	-	-	-	-
		-	-	-	-	-	-	-	-	-	-	-	-	170946	-	-	-	-	-	-	-	-	-	-	-	-	-	-	-	-	-	-	-	-	-	-	-	-	-	-	-	-	-	-	-	-	-	-	-	-	-	-
		-	-	-	-	-	-	-	-	-	-	-	-	170932	-	-	-	-	-	-	-	-	-	-	-	-	-	-	-	-	-	-	-	-	-	-	-	-	-	-	-	-	-	-	-	-	-	-	-	-	-	-
CYP770A1	cytochrome P450, unkown function	-	-	-	-	-	-	-	-	-	-	-	-	170931	-	-	-	-	-	-	-	-	-	-	-	-	-	-	-	-	-	-	-	-	-	-	-	-	-	-	-	-	-	-	-	-	-	-	-	-	-	-
		-	-	-	-	-	-	-	-	-	-	-	-	170929	-	-	-	-	-	-	-	-	-	-	-	-	-	-	-	-	-	-	37398	-	-	-	-	-	-	-	-	-	-	-	-	-	-	-	-	-	-	-
		-	-	-	-	-	-	-	-	-	-	-	-	170922	-	-	-	-	-	-	-	-	-	-	-	-	-	-	-	-	-	-	-	-	-	-	-	-	-	-	-	-	-	-	-	-	-	-	-	-	-	-
		-	-	-	-	-	-	-	-	-	-	-	-	170920	-	-	-	-	-	-	-	-	-	-	-	-	-	-	-	-	-	-	-	-	-	-	-	-	-	-	-	-	-	-	-	-	-	-	-	-	-	-
		-	-	-	-	-	-	-	-	-	-	-	-	170913	-	-	-	-	-	-	-	-	-	-	-	-	-	-	-	-	-	-	-	GSPATP00024765001	-	-	-	-	-	-	-	-	-	-	-	-	-	-	-	-	-	-
		-	-	-	-	-	-	-	-	-	-	-	-	170911	-	-	-	-	-	-	-	-	-	-	-	-	-	-	-	-	-	-	-	-	-	-	-	-	-	-	-	-	-	-	-	-	-	-	-	-	-	-
		-	-	-	-	-	-	-	-	-	-	-	-	170899	-	-	-	-	-	-	-	-	-	-	-	-	-	-	-	-	-	-	-	GSPATP00015701001	-	74319	-	-	-	-	-	-	-	-	67.m00145	-	-	-	-	-	-	-
		-	+	-	-	-	-	-	AT2G41450.1	-	-	-	-	170898	-	-	-	-	-	-	-	-	-	-	-	-	-	-	-	-	-	Os01g13200.1	30015	-	-	138101	-	-	734840	-	-	-	-	-	-	-	-	-	-	-	-	-
		-	-	-	-	-	-	-	-	-	-	CE10456	-	170897	-	-	-	-	-	-	-	-	-	-	-	-	-	-	-	-	-	-	-	-	-	-	-	-	-	-	-	-	-	-	-	-	-	-	-	-	-	-
		-	-	-	-	-	-	-	-	-	-	-	-	170889	-	-	-	-	-	-	-	-	-	-	-	-	-	-	-	-	-	-	-	-	-	-	-	-	-	-	-	-	-	-	-	-	-	-	-	-	-	-
		-	-	-	-	-	-	-	-	-	-	-	-	170886	-	-	-	-	-	-	-	-	-	-	-	-	-	-	-	-	-	-	-	-	-	-	-	-	-	-	-	-	-	-	-	-	-	-	-	-	-	-
		-	+	-	-	-	-	GB17280-PA	AT1G54650.1	13646	BDEG_05665	CE39654	137298	170884	202707	cgd8_4270	CMJ023C	OTTDARP00000022603	DDB0167566	CG13929-PB	-	194.m00110	ENSGALP00000015559	-	OTTHUMP00000025173	-	223580	23351	-	234398	NCU04647	Os07g23169.1	19947	GSPATP00019083001	33853	144584	125078	PFL2305w	571076	RO3G_08157.1	YOR239W	SPBC3H7.11	-	SINFRUP00000161796	19.m00326	21163	-	-	22540	-	Tb09.211.2860	-
		-	-	-	-	-	-	-	-	-	-	-	-	170883	-	-	-	-	-	-	-	-	-	-	-	-	-	-	-	-	-	-	24041	-	54765	64219	-	-	-	-	-	-	-	-	-	11734	-	-	-	-	-	-
		-	-	-	-	-	-	-	-	-	-	-	-	170879	-	-	-	-	-	-	-	-	-	-	-	-	-	-	-	-	-	-	-	-	-	-	-	-	-	-	-	-	-	-	-	-	-	-	-	-	-	-
		-	-	-	-	-	-	-	-	-	-	-	-	170876	-	-	-	-	-	-	-	-	-	-	-	-	-	-	-	-	-	-	-	-	-	-	-	-	-	-	-	-	-	-	-	-	-	-	-	-	-	-
		-	-	-	-	-	-	-	-	-	-	-	-	170874	-	-	-	-	-	-	-	-	-	-	-	-	-	-	-	-	-	-	-	-	-	-	-	-	-	-	-	-	-	-	-	-	-	-	-	-	-	-
		-	+	-	-	-	-	-	AT3G19990.1	-	-	-	-	170872	-	-	-	-	-	-	-	-	-	-	-	-	-	-	-	-	-	Os02g58170.1	-	-	35461	164339	-	-	563393	-	-	-	-	-	-	-	-	-	-	-	-	-
		-	+	-	-	-	-	GB16112-PA	AT1G79610.1	19894	BDEG_01943	CE31015	184481	170866	-	cgd6_130	CMN286C	OTTDARP00000023077	-	CG11328-PB	-	-	ENSGALP00000026953	-	OTTHUMP00000024091	-	-	16608	46137	106621	NCU00453	Os09g11450.2	14730	GSPATP00033903001	14403	-	130175	-	269653	RO3G_00551.1	YDR456W	SPAC15A10.06	GLEAN3_06845	SINFRUP00000159805	51.m00240	262960	-	541.m01159	11627	-	-	UM03812.1
		-	-	-	-	-	-	-	-	-	-	-	-	170864	-	-	-	-	-	-	-	-	-	-	-	-	-	26990	-	-	-	-	-	-	-	-	-	-	-	-	-	-	-	-	-	771	-	-	-	-	-	-
		-	-	-	-	-	-	-	-	-	-	-	-	170858	-	-	-	-	-	-	-	-	-	-	-	-	-	-	-	-	-	-	-	-	-	-	-	-	-	-	-	-	-	-	-	-	-	-	-	-	-	-
		-	-	-	-	-	-	-	-	-	-	-	-	170857	-	-	-	-	-	-	-	-	-	-	-	-	-	-	-	-	-	-	-	-	-	-	-	-	-	-	-	-	-	-	-	-	-	-	-	-	-	-
		-	-	-	-	-	-	-	-	-	-	-	-	170855	-	-	-	-	-	-	-	-	-	-	-	-	-	-	-	-	-	-	-	-	-	-	-	-	-	-	-	-	-	-	-	-	-	-	-	-	-	-
		-	-	-	-	-	-	-	-	-	-	-	-	170854	239620	-	-	-	-	-	-	-	-	-	-	-	-	-	-	-	NCU02762	-	-	-	-	-	-	-	-	RO3G_16787.1	-	-	-	-	-	-	-	-	-	-	-	-
LMR3	protein of unknown function, contains LysM-domain	-	+	-	-	-	-	-	AT1G55000.3	-	-	CE10356	-	170846	-	-	-	OTTDARP00000013540	DDB0192068	-	-	-	ENSGALP00000023566	-	OTTHUMP00000176803	-	-	-	-	-	-	Os03g49250.1	-	-	-	116069	-	-	240724	-	-	-	-	SINFRUP00000165314	-	-	-	-	-	-	-	-
		-	-	-	-	-	-	-	-	-	-	-	-	170844	-	-	-	-	-	-	-	-	-	-	-	-	-	-	-	-	-	-	-	-	-	-	-	-	-	-	-	-	-	-	-	-	-	-	-	-	-	-
		-	-	-	-	-	-	-	-	-	-	-	-	170843	-	-	-	-	-	-	-	-	-	-	-	-	-	-	-	-	-	-	-	-	-	-	-	-	-	-	-	-	-	-	-	-	-	-	-	-	-	-
		-	-	-	-	-	-	-	-	-	-	-	-	170836	-	-	-	-	-	-	-	-	-	-	-	-	-	-	-	-	-	-	-	-	-	-	-	-	-	-	-	-	-	-	-	-	-	-	-	-	-	-
		-	-	-	-	-	-	-	-	-	-	-	-	170832	-	-	-	-	-	-	-	-	-	-	-	-	-	-	-	-	-	-	-	-	-	-	-	-	-	-	-	-	-	-	-	-	-	-	-	-	-	-
		-	-	-	-	-	-	-	-	-	-	-	-	170830	-	-	-	-	-	-	-	-	-	-	-	-	-	-	-	-	-	-	-	-	-	-	-	-	-	-	-	-	-	-	-	-	-	-	-	-	-	-
		-	+	-	-	-	-	-	AT1G06290.1	-	-	-	182867	170822	-	-	-	-	DDB0188084	-	-	-	-	-	-	-	-	-	-	86003	-	Os06g24704.1	-	-	-	207164	-	-	582787	-	-	-	-	-	-	-	-	-	-	-	-	-
CHR3425	SNF2 Superfamily protein	-	-	-	-	-	-	-	-	-	-	-	-	170820	-	-	-	-	-	-	-	-	-	-	-	-	-	-	-	-	-	-	-	-	-	-	-	-	-	-	-	-	GLEAN3_14974	-	-	35248	-	-	-	-	-	-
		-	+	-	-	-	-	-	AT2G38460.1	-	-	CE12624	161975	170818	383572	-	-	OTTDARP00000003646	-	-	-	-	ENSGALP00000003828	-	OTTHUMP00000163497	-	171641	33674	72259	97531	NCU08804	Os06g36450.1	-	-	54495	114248	141344	-	577095	-	-	-	GLEAN3_03229	SINFRUP00000129888	-	-	-	-	1773	-	-	-
		-	+	-	-	-	-	-	-	-	-	-	-	170809	-	-	-	-	-	-	-	-	-	-	-	-	-	-	-	113833	-	-	-	-	47700	-	137328	-	-	-	-	-	-	SINFRUP00000140696	-	2227	-	-	-	-	-	-
		-	-	-	-	-	-	-	-	-	-	-	-	170806	-	-	-	-	-	-	-	-	-	-	-	-	-	-	-	-	-	Os05g09660.1	-	-	-	-	-	-	-	-	-	-	-	-	-	-	-	-	-	-	-	-
		-	+	-	-	-	-	GB11175-PA	AT4G34310.1	-	-	CE03226	73042	170805	243180	-	CMP162C	OTTDARP00000005303	-	CG10383-PA	-	-	ENSGALP00000022278	-	OTTHUMP00000017498	-	108969	-	-	147735	NCU05896	Os03g08700.1	-	-	-	160793	140331	-	591901	-	-	-	-	SINFRUP00000146965	-	-	-	583.m05349	-	-	-	-
		-	-	-	-	-	-	-	-	-	-	-	-	170801	-	-	-	-	-	-	-	-	-	-	-	-	-	-	-	-	-	-	-	-	-	-	-	-	-	-	-	-	-	-	-	-	-	-	-	-	-	-
		-	-	-	-	-	-	-	-	-	-	-	-	170799	-	-	-	-	-	-	-	-	-	-	-	-	-	-	-	-	-	-	30415	-	-	-	-	-	-	-	-	-	-	-	-	-	-	-	-	-	-	-
		-	-	-	-	-	-	-	-	-	-	-	-	170794	-	-	-	-	-	-	-	-	-	-	-	-	-	-	-	-	-	-	-	-	-	-	-	-	-	-	-	-	-	-	-	-	-	-	-	-	-	-
		-	-	-	-	-	-	-	-	-	-	-	-	170789	-	-	-	-	-	-	-	-	-	-	-	-	-	-	-	-	-	-	-	-	-	-	-	-	-	-	-	-	-	-	-	-	-	-	-	-	-	-
		-	-	-	-	-	-	-	-	-	-	-	-	170787	-	-	-	-	-	-	-	-	-	-	-	-	-	-	-	-	-	-	-	-	-	-	-	-	-	-	-	-	-	-	-	-	-	-	-	-	-	-
		-	-	-	-	-	-	-	AT2G44760.1	-	-	-	-	170771	-	-	-	-	-	-	-	-	-	-	-	-	-	-	-	-	-	Os04g46100.1	-	-	-	162323	-	-	410121	-	-	-	-	-	-	-	-	-	-	-	-	-
		-	-	-	-	-	-	-	-	-	-	-	-	170753	-	-	-	-	-	-	-	-	-	-	-	-	-	-	-	-	-	-	-	-	-	-	-	-	-	-	-	-	-	-	-	-	-	-	-	-	-	-
		-	-	-	-	-	-	-	-	-	-	-	-	170751	-	-	-	-	-	-	-	-	-	-	-	-	-	-	-	-	-	-	-	-	-	-	-	-	-	-	-	-	-	-	-	-	-	-	-	-	-	-
RAD9	DNA damage checkpoint protein.	-	-	-	-	-	-	-	AT3G05480.1	-	-	-	-	170734	-	-	-	-	-	-	-	-	-	-	-	-	-	-	-	-	-	Os03g22450.1	-	-	-	136626	-	-	806774	-	-	-	-	-	-	-	-	-	-	-	-	-
		-	+	-	-	-	-	-	AT5G40870.1	5257	BDEG_05206	CE20700	-	170716	271859	cgd8_2810	CME192C	OTTDARP00000002042	-	-	-	-	-	-	-	-	-	17791	59011	206949	NCU05224	Os08g41790.1	22125	GSPATP00005347001	10398	160149	144051	-	815577	RO3G_13324.1	YHR128W	SPCC162.11c	GLEAN3_12804	SINFRUP00000128310	113.m00113	-	-	-	50478	-	-	-
		-	+	-	-	-	-	-	-	21491	-	-	223996	170707	-	-	-	OTTDARP00000011479	-	-	-	-	-	-	OTTHUMP00000030360	-	204460	-	67664	234790	-	-	-	-	37595	-	-	-	-	-	-	-	-	-	-	6505	-	-	-	-	-	-
		-	-	-	-	-	-	-	-	-	-	-	-	170703	-	-	-	-	-	-	-	-	-	-	-	-	-	-	-	-	-	-	-	-	-	-	-	-	-	-	-	-	-	-	-	-	-	-	-	-	-	-
		-	-	-	-	-	-	-	-	-	-	-	-	170702	-	-	-	-	-	-	-	-	-	-	-	-	-	-	-	-	-	-	-	-	-	-	-	-	-	-	-	-	-	-	-	-	-	-	-	-	-	-
		-	-	-	-	-	-	-	-	-	-	-	-	170700	-	-	-	-	-	-	-	-	-	-	-	-	-	-	-	-	-	-	-	-	-	-	-	-	-	-	-	-	-	-	-	-	-	-	-	-	-	-
		-	-	-	-	-	-	-	-	-	-	-	-	170697	-	-	-	-	-	-	-	-	-	-	-	-	-	-	-	-	-	-	-	-	-	-	-	-	-	-	-	-	-	-	-	-	-	-	-	-	-	-
		-	-	-	-	-	-	-	-	-	-	-	-	170694	-	-	-	-	-	-	-	-	-	-	-	-	-	-	-	-	-	-	-	-	-	-	-	-	-	-	-	-	-	-	-	-	-	-	-	-	-	-
		-	-	-	-	-	-	-	-	-	-	-	-	170688	-	-	-	-	-	-	-	-	-	-	-	-	-	-	-	-	-	-	-	-	-	-	-	-	-	-	-	-	-	-	-	-	-	-	-	-	-	-
		-	-	-	-	-	-	-	-	-	-	-	-	170687	-	-	-	-	-	-	-	-	-	-	-	-	-	-	-	-	-	-	-	-	-	-	-	-	-	-	-	-	-	-	-	-	-	-	-	-	-	-
		-	-	-	-	-	-	-	-	-	-	-	-	170684	-	-	-	-	-	-	-	-	-	-	-	-	-	-	-	-	-	-	-	-	-	-	-	-	-	-	-	-	-	-	-	-	-	-	-	-	-	-
		-	-	-	-	-	-	-	-	-	-	-	-	170683	-	-	-	-	-	-	-	-	-	-	-	-	-	-	-	-	-	-	-	-	-	-	-	-	-	-	-	-	-	-	-	-	-	-	-	-	-	-
		-	-	-	-	-	-	-	-	-	-	-	-	170678	-	-	-	-	-	-	-	-	-	-	-	-	172963	-	-	124472	-	-	-	-	-	-	144313	-	-	-	-	-	-	-	-	-	-	-	-	-	-	-
		-	-	-	-	-	-	-	-	-	-	-	-	170676	-	-	-	-	-	-	-	-	-	-	-	-	-	-	-	-	-	-	-	-	-	-	-	-	-	-	-	-	-	-	-	-	-	-	-	-	-	-
		-	-	-	-	-	-	-	-	-	-	-	-	170673	-	-	-	-	-	-	-	-	-	-	-	-	-	-	-	-	NCU03220	-	-	-	-	-	-	-	-	-	YGR266W	-	-	-	-	-	-	-	-	-	-	-
		-	-	-	-	-	-	-	-	-	-	-	-	170664	-	-	-	-	-	-	-	-	-	-	-	-	-	-	-	-	-	-	-	-	-	-	-	-	-	-	-	-	-	-	-	-	-	-	-	-	-	-
PAFC3401	Paf1 complex component	-	+	-	-	-	-	GB19815-PA	AT2G06210.1	72385	BDEG_08206	CE20456	179822	170652	260476	-	-	-	DDB0205397	CG2469-PA	19074372	-	ENSGALP00000009002	-	NP_055448.1	LmjF29.2550	177207	34178	47250	173010	NCU00095	Os07g29360.1	-	GSPATP00000544001	42758	177738	145066	-	281458	RO3G_13172.1	YOL145C	SPAC27D7.14c	GLEAN3_08914	SINFRUP00000145185	333.m00017	268346	-	55.m04611	19990	-	-	-
		-	-	-	-	-	-	-	-	-	-	-	-	170642	-	-	-	-	-	-	-	-	-	-	-	-	-	-	-	-	-	-	-	-	-	-	-	-	-	-	-	-	-	-	-	-	-	-	-	-	-	-
		-	-	-	-	-	-	-	-	-	-	-	-	170635	-	-	-	-	-	-	-	-	-	-	-	-	-	-	-	-	-	-	-	-	-	-	-	-	-	-	-	-	-	-	-	-	-	-	-	-	-	-
		-	-	-	-	-	-	-	-	-	-	-	201139	170621	-	-	-	-	-	CG32085-PA	-	-	-	-	OTTHUMP00000081171	-	-	-	-	-	-	-	-	-	-	-	-	-	-	-	-	-	-	-	-	-	-	-	-	-	-	-
		-	-	-	-	-	-	-	-	-	-	-	-	170619	-	-	-	-	-	-	-	-	-	-	-	-	-	-	-	207487	-	-	-	-	-	-	-	PF10_0099	-	-	-	-	-	-	-	-	-	-	-	-	-	-
		-	-	-	-	-	-	-	-	-	-	-	-	170607	-	-	-	-	-	-	-	-	-	-	-	-	-	-	-	-	-	-	-	-	-	-	-	-	-	-	-	-	-	-	-	-	-	-	-	-	-	-
		-	-	-	-	-	-	-	-	-	-	-	-	170601	-	-	-	-	-	CG33981-PA	-	-	-	-	OTTHUMP00000172910	-	-	-	-	-	-	-	-	-	-	81312	-	-	-	-	-	-	-	SINFRUP00000178689	-	-	-	-	-	-	-	-
		-	-	-	-	-	-	-	-	-	-	-	-	170600	-	-	-	-	-	-	-	-	-	-	-	-	-	-	-	-	-	-	-	-	-	-	-	-	-	-	-	-	-	-	-	-	-	-	-	-	-	-
		-	-	-	-	-	-	-	AT3G48470.1	-	-	-	-	170597	-	-	-	-	-	-	-	-	-	-	-	-	-	-	-	-	-	Os02g38680.1	-	-	-	147457	-	-	666601	-	-	-	-	-	-	-	-	-	-	-	-	-
HAG3403	Histone acetyltransferase	-	+	-	-	-	-	GB15432-PA	AT5G56740.1	-	BDEG_04389	-	169509	170595	285986	cgd7_60	CMS083C	-	DDB0167844	CG2051-PA	-	67.m00100	ENSGALP00000015492	-	NP_001028257.1	-	232538	37635	65261	-	-	Os09g17850.1	-	-	-	169354	-	-	663183	-	YPL001W	SPAC139.06	-	SINFRUP00000149397	3.m01941	-	-	-	53567	-	-	UM02567.1
		-	-	-	-	-	-	-	-	-	-	-	-	170570	-	-	-	-	-	-	-	-	-	-	-	-	-	-	-	-	-	-	-	-	3369	-	-	-	-	-	-	-	-	-	-	-	-	-	-	-	-	-
		-	+	-	-	-	-	-	AT2G02970.1	-	BDEG_01581	CE28748	169207	170567	-	-	-	-	-	-	-	-	-	-	-	-	-	28117	-	-	NCU08027	Os08g33850.2	-	-	44968	31384	-	-	833712	RO3G_00180.1	-	-	-	-	104.m00093	-	-	551.m00224	-	82360.m00240	-	UM00987.1
		-	-	-	-	-	-	-	-	-	-	-	-	170566	-	-	-	-	-	-	-	-	-	-	-	-	-	-	-	-	-	Os05g41670.1	-	-	-	-	-	-	-	-	-	-	-	-	-	-	-	-	-	-	-	-
		-	+	-	-	-	-	-	-	-	BDEG_01617	-	222156	170565	-	-	-	-	-	-	-	-	ENSGALP00000003458	-	OTTHUMP00000163853	-	-	-	-	-	-	Os09g01680.1	13084	-	-	137435	-	-	-	-	-	-	-	-	-	-	-	-	-	-	-	-
		-	-	-	-	-	-	-	-	-	-	-	-	170553	-	-	-	-	-	-	-	-	-	-	-	-	-	-	-	-	-	-	-	-	-	-	-	-	-	-	-	-	-	-	-	-	-	-	-	-	-	-
		-	+	-	-	-	-	-	-	-	-	CE18505	-	170552	-	-	CMG096C	OTTDARP00000023235	DDB0187984	-	-	-	ENSGALP00000038703	-	OTTHUMP00000077839	-	-	21969	-	118883	-	-	-	-	-	150929	-	-	-	RO3G_13356.1	-	-	-	-	-	-	-	-	56646	81529.m00494	-	-
		-	-	-	-	-	-	-	-	-	-	-	-	170550	-	-	-	-	-	-	-	-	-	-	-	-	-	-	-	-	-	-	-	-	-	18548	-	-	-	-	-	-	-	-	-	-	-	-	-	-	-	-
ClpT1	Clp protease adaptor protein	-	+	-	-	-	-	-	AT1G68660.1	-	-	-	-	170547	-	-	CML200C	-	-	-	-	-	-	-	-	-	-	-	-	-	-	Os08g33540.2	-	-	-	27818	-	MAL13P1.111	822150	-	-	-	-	-	-	2352	-	-	-	-	-	-
		-	-	-	-	-	-	-	-	-	-	-	-	170546	-	-	-	-	-	-	-	-	-	-	-	-	-	-	-	-	-	-	-	-	-	-	-	-	-	-	-	-	-	-	-	-	-	-	-	-	-	-
		-	-	-	-	-	-	-	-	-	-	-	-	170540	-	-	-	-	-	-	-	-	-	-	-	-	-	-	-	-	-	-	-	-	-	-	-	-	-	-	-	-	-	-	-	-	-	-	-	-	-	-
	hypothetical protein of unknown function	-	-	-	-	-	-	-	-	-	-	-	-	170535	-	-	-	-	-	-	-	-	-	-	-	-	-	-	-	-	-	-	-	-	-	-	-	-	-	-	-	-	-	-	-	-	-	-	-	-	-	-
	hypothetical protein	-	+	-	-	-	-	-	-	64375	-	-	76413	170531	-	-	-	-	-	-	-	45.m00156	-	-	-	-	-	-	-	-	-	-	34213	-	-	-	-	-	707191	-	-	-	-	-	-	-	-	540.m00202	51886	-	-	-
		-	-	-	-	-	-	-	-	-	-	-	-	170526	-	-	-	-	-	-	-	-	-	-	-	-	-	37688	-	-	-	-	-	-	-	-	-	-	-	RO3G_06263.1	-	-	-	-	-	-	-	-	-	-	-	-
		-	-	-	-	-	-	-	-	-	-	-	-	170520	-	-	-	-	-	-	-	-	-	-	-	-	-	-	-	-	-	-	-	-	-	-	-	-	-	-	-	-	-	-	-	-	-	-	-	-	-	-
		-	-	-	-	-	-	-	-	-	-	-	-	170514	-	-	-	-	-	-	-	-	-	-	-	-	-	-	-	-	-	-	-	-	-	-	-	-	-	-	-	-	-	-	-	-	-	-	-	-	-	-
FAP172	Flagellar Associated Protein	+	+	-	+	-	-	-	-	23276	BDEG_08492	-	177430	170513	-	-	-	-	-	-	-	-	ENSGALP00000011394	-	OTTHUMP00000181398	LmjF33.0590	-	-	-	-	-	-	-	GSPATP00019408001	-	92247	140957	-	-	-	-	-	-	-	42.m00201	-	-	83.m02701	-	-	Tb10.26.0760	-
THFD	tetrahydrofolate-dehydrogenase/cyclohydrolase-like protein	-	-	-	-	-	-	-	-	-	-	-	-	170511	-	-	-	-	-	-	-	-	-	-	-	-	-	-	-	-	-	-	-	-	-	-	-	-	-	-	-	-	-	-	-	-	-	-	-	-	-	-
		-	+	-	-	-	-	GB20121-PA	AT4G39640.2	-	BDEG_03505	CE23612	2729	170508	213764	-	CMQ101C	OTTDARP00000023498	-	CG17636-PA	19170870	-	ENSGALP00000010584	-	OTTHUMP00000159078	-	188739	-	963	129931	-	Os04g38450.1	-	-	19513	87161	-	-	760175	RO3G_04486.1	YLR299W	SPAC664.09	GLEAN3_12790	SINFRUP00000165566	-	262573	-	-	59244	-	-	UM02120.1
		-	-	-	-	-	-	-	-	-	-	-	-	170507	-	-	-	-	-	-	-	-	-	-	-	-	-	-	-	-	-	-	-	-	-	-	-	-	-	-	-	-	-	-	-	-	-	-	-	-	-	-
		-	+	-	-	-	-	-	AT1G79810.2	-	BDEG_02769	-	-	170506	-	-	-	-	DDB0168812	-	-	-	-	-	-	-	-	-	-	130528	NCU02070	Os05g19480.1	20947	-	-	198063	-	-	645972	RO3G_03275.1	-	-	-	SINFRUP00000149883	-	-	-	-	-	-	-	UM00325.1
		-	-	-	-	-	-	-	-	-	-	-	-	170504	-	-	-	-	-	-	-	-	-	-	-	-	-	-	-	-	-	-	-	-	-	-	-	-	-	-	-	-	-	-	-	-	-	-	-	-	-	-
		-	-	-	-	-	-	-	-	-	-	-	-	170495	-	-	-	-	-	-	-	-	-	-	-	-	-	-	-	-	-	-	-	-	-	-	-	-	-	-	-	-	-	-	-	-	-	-	-	-	-	-
		-	+	-	-	-	-	-	AT5G01650.1	30541	-	CE22574	221346	170481	298518	-	CMT251C	-	-	-	-	26.m00273	ENSGALP00000010214	GL50803_12091	OTTHUMP00000043703	LmjF33.1750	231585	-	-	189079	-	Os12g01680.1	13159	-	49660	166244	157309	PFL1420w	825385	RO3G_07231.1	-	-	GLEAN3_11299	SINFRUP00000156582	-	-	-	80.m00086	53604	87640.m00209	-	-
		-	-	-	-	-	-	-	-	-	-	-	-	170472	-	-	-	-	-	-	-	-	-	-	-	-	-	-	-	-	-	-	-	-	-	-	-	-	-	-	-	-	-	-	-	-	-	-	-	-	-	-
		-	-	-	-	-	-	-	-	-	-	-	-	170468	-	-	-	-	-	-	-	-	-	-	-	-	-	-	-	-	-	-	-	-	-	-	-	-	-	-	-	-	-	-	-	-	-	-	-	-	-	-
		-	-	-	-	-	-	-	AT1G49350.1	-	-	-	-	170463	-	-	-	-	-	-	-	-	-	-	-	-	-	-	-	-	-	Os05g09370.2	-	-	-	214732	-	-	195342	-	-	-	-	-	-	-	-	-	-	-	-	-
		-	+	+	-	-	-	GB14118-PA	AT3G06670.1	64954	BDEG_03508	CE37911	182297	170459	389693	cgd3_2400	-	OTTDARP00000005815	DDB0188242	-	-	424.m00035	ENSGALP00000017444	-	OTTHUMP00000028305	LmjF31.2980	169795	8951	81298	245349	NCU11315	Os01g23590.1	21492	GSPATP00011993001	40325	138057	143606	-	803495	RO3G_03223.1	YNL201C	SPBC216.01c	-	SINFRUP00000152830	16.m00442	269314	TA18585	59.m03638	50359	-	-	UM01152.1

		-	+	+	-	-	-	GB14057-PA	AT2G25100.1	12637	BDEG_00736	CE28960	183401	170458	294456	cgd4_2160	CMK297C	OTTDARP00000019865	DDB0184459	CG13690-PA	19168694	36.m00210	-	GL50803_6483	OTTHUMP00000083190	LmjF36.0640	200807	25972	3404	210941	NCU09608	Os11g05570.1	-	GSPATP00029234001	32830	163152	130400	PFF1150w	765150	RO3G_00624.1	YNL072W	SPAC4G9.02	-	SINFRUP00000142676	11.m00332	17008	TA19790	74.m00432	51212	89002.m00290	Tb10.70.2140	UM06131.1
DNJ1	DnaJ-like protein 1, probably cytosolic	-	+	-	+	-	-	GB12215-PA	-	59942	-	-	164620	170453	-	-	CML030C	-	-	CG9828-PA	19069140	2.m00517	-	-	-	-	-	20146	-	118966	-	-	-	-	42151	-	-	PF14_0359	-	RO3G_03192.1	-	SPBC1734.11	GLEAN3_28919	-	-	37083	-	583.m05418	-	-	Tb10.70.5440	-
		-	-	-	-	-	-	-	-	-	-	-	-	170437	-	-	-	-	-	-	-	-	-	-	-	-	-	-	-	-	-	-	-	-	-	-	-	-	-	-	-	-	-	-	-	-	-	-	-	-	-	-
		-	-	-	-	-	-	-	-	-	-	-	-	170429	-	-	-	-	-	-	-	-	-	-	-	-	-	-	-	-	-	-	-	32463	-	-	-	-	-	-	-	-	-	-	-	-	-	-	-	-	-	-
		-	-	-	-	-	-	-	-	-	-	-	-	170428	-	-	-	-	-	-	-	-	-	-	-	-	-	-	-	-	-	-	-	-	-	-	-	-	-	-	-	-	-	-	-	-	-	-	-	-	-	-
DUR3C	urea active transporter	-	-	-	-	-	-	GB12014-PA	-	-	-	-	-	170417	-	-	-	-	-	-	-	-	-	-	-	-	-	-	-	-	-	-	-	-	-	-	-	-	-	-	-	-	GLEAN3_11497	-	-	-	-	-	-	-	-	-
		-	-	-	-	-	-	-	-	-	-	-	-	170413	-	-	-	-	-	-	-	-	-	-	-	-	-	-	-	-	-	-	-	-	-	-	160806	-	-	-	-	-	-	-	-	-	-	-	-	-	-	-
		-	-	-	-	-	-	-	-	-	-	-	-	170412	-	-	-	-	-	-	-	-	-	-	-	-	-	-	-	-	-	-	-	-	-	-	-	-	-	-	-	-	-	-	-	-	-	-	-	-	-	-
		-	-	-	-	-	-	-	-	-	-	-	-	170409	-	-	-	-	-	-	-	-	-	-	-	-	-	-	-	-	-	Os02g29050.1	-	-	-	-	-	-	-	-	-	-	-	-	-	-	-	-	-	-	-	-
		-	-	-	-	-	-	-	-	-	-	-	-	170405	-	-	-	-	-	-	-	-	-	-	-	-	-	-	-	-	-	-	-	-	-	-	-	-	-	-	-	-	-	-	-	-	-	-	-	-	-	-
		-	-	-	-	-	-	-	-	-	-	-	-	170392	227945	-	-	-	-	-	-	-	-	-	-	-	-	-	-	-	-	-	-	-	-	-	97805	141350	-	-	-	-	-	-	-	-	-	-	-	-	-	-	-
		-	+	-	-	-	-	GB13062-PA	AT5G42920.2	-	-	CE24199	18172	170375	293555	-	-	-	-	-	-	-	ENSGALP00000037250	-	OTTHUMP00000028862	-	236170	32095	-	167837	NCU03239	Os02g52820.1	35030	-	-	186713	132382	-	836447	RO3G_06183.1	-	-	-	SINFRUP00000154981	-	-	-	-	59730	-	-	-
		-	+	-	-	-	-	-	-	-	BDEG_05252	-	-	170374	-	-	CMP318C	-	-	-	-	-	-	-	-	-	-	39277	-	-	NCU09858	-	35892	-	-	-	-	-	-	-	-	-	-	-	9.m00502	-	-	-	-	-	-	-
PROB2	glutamate 5-kinase	-	+	-	-	-	-	-	-	-	BDEG_08119	-	-	170370	-	cgd2_2300	CMQ021C	-	-	-	-	-	-	-	-	-	-	-	-	-	NCU00106	-	29522	-	-	-	-	-	791562	RO3G_05395.1	YDR300C	SPAC17H9.13c	-	-	-	-	-	57.m00007	-	-	-	-
		-	-	-	-	-	-	-	AT1G03650.1	-	-	-	-	170368	-	-	-	-	-	-	-	-	-	-	-	-	-	-	-	-	-	-	-	-	-	117862	-	-	-	-	-	-	-	-	-	-	-	-	-	-	-	-
		-	-	-	-	-	-	-	-	-	-	-	-	170367	-	-	-	-	-	-	-	-	-	-	-	-	-	-	-	-	-	-	-	-	-	-	-	-	-	-	-	-	-	-	-	-	-	-	-	-	-	-
		-	-	-	-	-	-	-	-	-	-	-	-	170366	-	-	-	-	-	-	-	-	-	-	-	-	-	39369	-	-	-	-	-	-	-	-	-	-	-	-	-	-	-	-	-	-	-	-	-	-	-	-
	Putative ARF/Sar1-like GTPase	-	-	-	-	-	-	-	-	-	-	-	-	170363	-	-	-	-	-	-	-	-	-	-	-	-	-	-	-	-	-	-	-	-	-	-	-	-	-	-	-	-	-	-	-	-	-	-	-	-	-	-
		-	-	-	-	-	-	-	AT3G12590.1	-	-	-	-	170354	-	-	-	-	-	-	-	-	-	-	-	-	-	-	-	-	-	Os01g74310.5	-	-	-	153153	-	-	227584	-	-	-	-	-	-	-	-	-	-	-	-	-
		-	+	-	-	-	-	-	-	29101	-	-	-	170351	-	-	-	-	-	-	-	-	-	-	-	LmjF15.0870	-	-	-	-	-	-	-	-	47374	-	135537	PF11_0059	-	-	-	-	-	-	-	-	-	583.m05296	-	-	Tb09.v2.0020	-
		-	-	-	-	-	-	-	-	-	-	-	-	-	-	-	-	-	-	-	-	-	-	-	-	-	-	-	-	-	-	-	-	-	-	-	-	-	-	-	-	-	-	-	-	-	-	-	-	-	-	-
		-	-	-	-	-	-	-	-	-	-	-	-	170344	-	-	-	-	-	-	-	-	-	-	OTTHUMP00000014716	-	-	-	-	-	-	-	-	-	-	-	-	-	-	-	-	-	-	-	-	-	-	-	-	-	-	-
		-	-	-	-	-	-	-	-	-	-	-	-	170342	-	-	-	-	-	-	-	-	-	-	-	-	-	-	-	-	-	-	-	-	-	-	-	-	-	-	-	-	-	-	-	-	-	-	-	-	-	-
HEP2	Hsp70 escorting protein 2	-	+	-	-	-	-	-	AT5G27280.1	-	-	-	-	170340	-	-	-	-	-	-	-	-	-	-	-	-	-	-	-	-	-	Os06g50870.1	37177	-	-	162324	-	-	652153	-	-	-	-	-	-	-	-	-	-	-	-	-
		-	-	-	-	-	-	-	-	-	-	-	-	170338	-	-	-	-	-	-	-	-	-	-	-	-	-	-	-	-	-	-	-	-	-	-	-	-	-	-	-	-	-	-	-	-	-	-	-	-	-	-
		-	-	-	-	-	-	-	AT3G24160.1	-	-	-	-	170329	-	-	-	-	-	-	-	-	-	-	-	-	-	-	-	-	-	Os08g45220.2	-	-	-	164917	-	-	817566	-	-	-	-	-	-	-	-	-	-	-	-	-
HTR20	Histone H3	-	-	-	-	-	-	-	-	-	-	-	-	-	-	-	-	-	-	-	-	-	-	-	-	-	-	-	-	-	-	-	-	-	-	-	-	-	-	-	-	-	-	-	-	-	-	-	-	-	-	-
		-	-	-	-	-	-	-	-	-	-	-	-	170317	-	-	-	-	-	-	-	-	-	-	-	-	-	-	-	-	-	-	-	-	-	-	-	-	-	-	-	-	-	-	22.m00273	-	-	-	-	-	-	-
		-	-	-	-	-	-	-	-	62732	-	-	-	170313	-	-	-	-	-	-	-	-	-	-	-	-	-	-	-	-	-	-	-	-	-	-	-	-	-	-	-	-	-	-	-	21676	-	-	56771	-	-	-
JMJ3402		-	+	-	-	-	-	-	AT1G08620.1	-	BDEG_08273	-	-	170309	-	-	-	-	DDB0190030	-	-	-	-	-	-	-	-	-	-	-	-	Os05g10770.1	10306	GSPATP00035495001	-	136290	111627	-	248138	-	YER169W	SPAC1002.05c	-	-	-	-	TA19925	542.m00225	-	-	-	-
		-	-	-	-	-	-	-	-	-	-	-	-	170307	-	-	-	-	-	-	-	-	-	-	-	-	-	-	-	-	-	-	-	-	-	-	-	-	-	-	-	-	-	-	-	-	-	-	-	-	-	-
		-	-	-	-	-	-	-	-	-	-	-	-	170305	-	-	-	-	-	-	-	-	-	-	-	-	-	-	-	-	-	-	-	-	-	-	-	-	-	-	-	-	-	-	-	-	-	-	-	-	-	-
		-	-	-	-	-	-	-	-	-	-	-	-	170303	-	-	-	-	-	-	-	-	-	-	-	-	-	-	-	-	-	-	-	-	-	-	-	-	-	-	-	-	-	-	-	-	-	-	-	-	-	-
		-	+	-	-	-	-	-	-	65560	BDEG_03790	-	200823	170294	-	-	-	-	-	-	-	-	ENSGALP00000039861	-	XP_001127080.1	-	236096	31854	-	244579	NCU05752	-	-	-	-	-	-	-	-	RO3G_01228.1	YJR078W	-	GLEAN3_28601	SINFRUP00000143857	2.m02058	-	-	-	-	-	-	UM03832.1
		-	+	-	-	-	-	-	-	-	BDEG_01613	-	114690	170281	216146	-	CMS488C	OTTDARP00000008089	-	-	-	-	ENSGALP00000016141	-	NP_061126.2	LmjF19.1510	234034	10334	38765	163756	-	-	-	-	-	-	-	-	-	-	-	-	GLEAN3_16090	SINFRUP00000175834	-	-	-	-	-	-	Tb927.6.3960	-
		-	-	-	-	-	-	-	-	-	-	-	-	170260	-	-	-	-	-	-	-	-	-	-	-	-	-	-	-	-	-	-	-	-	-	70697	-	-	-	-	-	-	-	-	-	-	-	-	-	-	-	-
		-	-	-	-	-	-	-	-	-	-	-	-	170252	-	-	-	-	-	-	-	-	-	-	-	-	-	-	-	-	-	-	-	-	-	-	-	-	-	-	-	-	-	-	-	-	-	-	-	-	-	-
		-	-	-	-	-	-	-	AT5G62720.1	-	-	-	-	170242	-	-	-	-	-	-	-	-	-	-	-	-	-	-	-	-	-	Os03g48030.1	34468	-	-	-	-	-	251738	-	-	-	-	-	-	-	-	-	-	-	-	-
		-	-	-	-	-	-	-	-	-	-	-	-	170239	-	-	-	-	-	-	-	-	-	-	-	-	-	-	-	-	-	-	-	-	-	-	-	-	-	-	-	-	-	-	-	-	-	-	-	-	-	-
		-	+	-	-	-	-	-	AT1G19250.1	-	-	CE30676	-	170238	-	-	-	-	-	-	-	-	-	-	-	-	172967	913	72677	231029	NCU05885	Os04g14710.1	34721	-	-	-	-	-	779640	-	-	-	GLEAN3_24261	-	-	-	-	-	29160	-	-	-
		-	-	-	-	-	-	-	-	-	-	-	-	170237	-	-	-	-	-	-	-	-	-	-	-	-	-	-	-	-	-	-	-	-	-	-	-	-	-	-	-	-	-	-	-	-	-	-	-	-	-	-
		-	-	-	-	-	-	-	-	-	-	-	-	170221	-	-	-	-	-	-	-	-	-	-	-	-	-	-	-	-	-	-	-	-	-	-	-	-	-	-	-	-	-	-	-	-	-	-	-	-	-	-
		-	-	-	-	-	-	-	-	-	-	-	-	170220	221999	-	-	-	-	-	-	-	-	-	-	-	-	-	-	-	-	-	-	-	-	-	-	-	-	-	-	-	-	-	-	-	-	-	-	-	-	-
		-	-	-	-	-	-	-	-	-	-	-	-	170215	-	-	-	-	-	-	-	-	-	-	-	-	-	-	-	-	-	-	-	-	-	-	-	-	-	-	-	-	-	-	-	-	-	-	-	-	-	-
		-	+	-	-	-	-	GB14552-PA	AT4G34340.1	-	-	CE35154	183324	170213	274448	-	-	OTTDARP00000008160	DDB0186530	CG7128-PA	-	14.m00336	ENSGALP00000005523	-	NP_612639.2	-	206383	-	-	240727	-	Os12g38620.3	-	-	-	16291	132574	-	577762	-	-	-	-	SINFRUP00000134298	-	-	-	-	52057	-	-	-
		-	-	-	-	-	-	-	-	-	-	-	-	170212	-	-	-	-	-	-	-	-	-	-	-	-	-	-	-	-	-	-	-	-	47881	-	-	-	-	-	-	-	-	-	-	21421	-	-	-	-	-	-
		-	-	-	-	-	-	-	-	-	-	-	-	170204	-	-	-	-	-	-	-	-	-	-	-	-	-	-	-	-	-	-	-	-	-	-	-	-	-	-	-	-	-	-	-	-	-	-	-	-	-	-
		-	-	-	-	-	-	-	-	-	-	-	-	170203	-	-	-	-	-	-	-	-	-	-	-	-	-	-	-	-	-	-	-	-	-	-	-	-	-	-	-	-	-	-	-	-	-	-	-	-	-	-
		-	-	-	-	-	-	-	-	-	-	-	-	170200	-	-	-	-	-	-	-	-	-	-	-	-	-	-	-	-	-	-	-	-	-	-	-	-	-	-	-	-	-	-	-	-	-	-	-	-	-	-
		-	-	-	-	-	-	-	-	-	-	-	-	170199	-	-	-	-	-	-	-	-	-	-	-	-	-	-	-	-	-	-	-	-	-	-	-	-	-	-	-	-	-	-	-	-	-	-	-	-	-	-
		-	-	-	-	-	-	-	-	-	-	-	-	170196	-	-	-	-	-	-	-	-	-	-	-	-	-	-	-	-	-	-	-	-	-	-	-	-	-	-	-	-	-	-	-	-	-	-	-	-	-	-
		-	-	-	-	-	-	-	-	-	-	-	-	170184	-	-	-	-	-	-	-	-	-	-	-	-	-	-	-	-	-	-	-	-	-	-	-	-	-	-	-	-	-	-	-	-	-	-	-	-	-	-
		-	-	-	-	-	-	-	-	-	-	-	-	170157	-	-	-	-	-	-	-	-	-	-	-	-	-	-	-	-	-	-	-	-	-	-	-	-	-	-	-	-	-	-	-	-	-	-	-	-	-	-
		-	-	-	-	-	-	-	-	-	-	-	-	170155	-	-	-	-	-	-	-	-	-	-	-	-	-	-	-	-	-	-	-	-	-	-	-	-	-	-	-	-	-	-	-	-	-	-	-	-	-	-
		-	-	-	-	-	-	-	-	-	-	-	-	170153	-	-	-	-	-	-	-	-	-	-	-	-	-	-	-	-	-	-	-	-	-	-	-	-	-	-	-	-	-	-	-	-	-	-	-	-	-	-
		-	-	-	-	-	-	-	-	-	-	-	-	170150	-	-	-	-	-	-	-	-	-	-	-	-	-	-	-	-	-	-	-	-	-	-	-	-	-	-	-	-	-	-	-	-	-	-	-	-	-	-
		-	-	-	-	-	-	-	-	-	-	-	-	170149	-	-	-	-	-	-	-	-	-	-	-	-	-	-	-	-	-	-	-	-	-	-	-	-	-	-	-	-	-	-	-	-	-	-	-	-	-	-
		-	-	-	-	-	-	-	-	-	-	-	-	170146	-	-	-	-	-	-	-	-	-	-	-	-	-	-	-	-	-	Os02g55950.2	-	-	-	60094	-	-	-	-	-	-	-	-	-	-	-	-	-	-	-	-
		-	-	-	-	-	-	-	-	-	-	-	-	170126	-	-	-	-	-	-	-	-	-	-	-	-	-	-	-	-	-	-	-	-	-	-	-	-	-	-	-	-	-	-	-	-	-	-	-	-	-	-
		-	+	-	-	-	-	-	-	-	-	-	119101	170120	-	-	-	-	-	-	-	-	-	-	NP_001071174.1	-	155489	30945	-	-	-	-	-	-	-	-	-	-	-	-	-	-	-	-	-	-	-	-	56986	-	-	-
		-	-	-	-	-	-	-	-	-	-	-	-	170116	-	-	-	-	-	-	-	-	-	-	-	-	-	-	-	-	-	-	-	-	-	-	-	-	-	-	-	-	-	-	-	-	-	-	-	-	-	-
		-	+	-	-	-	-	-	AT1G10510.1	-	-	-	151888	170113	-	-	-	OTTDARP00000012225	DDB0219591	-	-	-	ENSGALP00000012708	GL50803_4165	NP_849172.2	LmjF26.0500	125332	29356	912	98567	NCU03207	Os06g13050.1	16156	-	42557	128227	-	-	721160	RO3G_03167.1	-	-	-	SINFRUP00000146443	263.m00037	263734	-	31.m00924	-	81869.m00158	Tb10.6k15.0810	-
		-	-	-	-	-	-	-	-	66248	-	-	-	170099	-	-	-	-	-	-	-	-	-	-	-	-	-	-	-	-	-	-	-	-	-	-	-	-	-	-	-	-	-	-	-	-	-	-	-	-	-	-
		-	-	-	-	-	-	-	-	-	-	-	-	170096	-	-	-	-	-	-	-	-	-	-	-	-	-	-	-	-	-	-	-	-	-	-	-	-	-	-	-	-	-	-	-	-	-	-	-	-	-	-
		-	-	-	-	-	-	-	-	-	-	-	-	170093	-	-	-	-	-	-	-	-	-	-	-	-	-	-	-	-	-	-	-	-	-	-	-	-	-	-	-	-	-	-	-	-	-	-	-	-	-	-
CHR3419	SNF2 Superfamily Protein	-	+	-	-	-	-	-	AT2G40770.1	64133	-	-	-	170090	-	-	CML287C	-	-	-	-	-	-	-	NP_001036148.1	-	-	-	-	162318	-	-	33205	-	45116	-	-	-	-	-	-	-	GLEAN3_20764	-	-	-	-	-	-	-	-	-
PAN2	putative PAN2-like protein (PAB-dependent poly(A)-specific ribonuclease subunit PAN2)	-	+	-	-	-	-	GB12069-PA	-	-	BDEG_04557	-	225482	170081	210807	-	CMJ136C	-	DDB0190477	CG8232-PA	-	-	ENSGALP00000004379	-	NP_055686.2	LmjF30.0250	143977	26638	33522	85947	NCU10733	-	3390	-	46460	76382	-	-	-	RO3G_12252.1	YGL094C	SPAC22G7.04	GLEAN3_03537	SINFRUP00000129698	-	-	-	44.m02720	30968	-	-	UM04591.1
		-	+	-	-	-	-	-	AT4G11860.1	11009	BDEG_06037	CE25491	179036	170077	267078	-	CML052C	OTTDARP00000014497	DDB0205586	-	-	-	ENSGALP00000033819	-	NP_060849.2	-	214327	34177	80571	130466	NCU03336	Os06g49800.1	36761	-	46063	234243	142984	PF13_0106	180613	-	YPL191C	SPAC12G12.11c	-	SINFRUP00000142937	-	885	TA13475	86.m00369	18339	-	-	UM06258.1
		-	-	-	-	-	-	-	-	-	-	-	-	170072	-	-	-	-	-	-	-	-	-	-	-	-	-	-	-	-	-	-	-	-	-	-	-	-	-	-	-	-	-	-	-	-	-	-	-	-	-	-
		-	-	-	-	-	-	-	AT5G23220.1	-	-	-	191742	170071	-	-	-	-	-	-	-	-	-	-	-	-	-	-	-	-	-	-	-	-	-	-	144924	-	-	-	-	-	-	-	-	-	-	-	-	-	-	-
GOX3	putative glyoxal or galactose oxidase	-	-	-	-	-	-	-	-	-	-	-	-	170066	-	-	-	-	-	-	-	-	-	-	-	-	-	-	-	-	-	-	-	-	-	-	-	-	-	-	-	-	-	-	-	-	-	-	-	-	-	-
		-	-	-	-	-	-	-	-	-	-	-	-	170063	-	-	-	-	-	-	-	-	-	-	-	-	-	-	-	-	-	-	-	-	-	-	-	-	-	-	-	-	-	-	-	-	-	-	-	-	-	-
		-	+	-	-	-	-	-	AT3G49700.1	-	-	CE39626	208240	170056	-	-	CMS398C	OTTDARP00000023790	DDB0203257	-	-	-	ENSGALP00000015308	-	NP_115981.1	-	190920	24686	30256	132278	-	Os03g51740.1	18718	-	51422	152777	-	-	582947	-	-	-	GLEAN3_23118	SINFRUP00000155239	-	31551	-	-	64185	96086.m00065	-	UM05676.1
ANT2	putative ADP/ATP carrier protein, probably mitochondrial	-	-	-	-	-	-	-	-	-	-	-	-	170037	-	-	-	-	-	-	-	-	-	-	-	-	-	-	-	-	-	-	-	-	-	-	-	-	-	-	-	-	-	-	-	-	-	-	-	-	-	-
SPL3		-	+	+	-	-	-	GB11796-PA	AT1G14650.1	-	BDEG_07282	CE17296	220618	170034	256353	cgd7_2160	-	OTTDARP00000020971	DDB0168938	CG3019-PA	-	39.m00227	ENSGALP00000012879	-	NP_001005409.1	-	190371	33566	46938	233857	NCU01913	Os02g14780.2	9417	GSPATP00005955001	-	174116	134534	PF14_0713	801052	RO3G_07324.1	-	SPAC22A12.09c	GLEAN3_27895	SINFRUP00000181006	1.m00929	21026	TA12905	50.m03114	19623	89741.m00034	-	UM00305.1
NFA3405	Nucleosome assembly protein	-	+	-	-	-	-	GB11007-PA	AT1G74560.1	-	-	CE27804	157590	170031	230084	cgd8_3970	-	OTTDARP00000020627	DDB0205333	-	-	-	ENSGALP00000007476	-	NP_003002.1	-	143784	-	-	163440	-	Os02g36710.1	-	-	-	174052	128839	PFI0930c	259013	-	-	SPBC3687.08c	GLEAN3_01780	SINFRUP00000155617	-	-	TA16235	80.m00014	53112	-	-	-
FAP141	Flagellar Associated Protein	-	-	-	+	-	-	-	-	-	-	-	-	170025	-	-	-	-	-	-	-	-	-	-	-	-	-	-	-	-	-	-	-	-	-	-	-	-	-	-	-	-	-	-	-	-	-	-	-	-	-	-
		-	-	-	-	-	-	-	-	-	-	-	-	170024	-	-	-	-	-	-	-	-	-	-	-	-	-	-	-	-	-	-	-	-	-	-	-	-	-	-	-	-	-	-	-	-	-	-	-	-	-	-
		-	-	-	-	-	-	-	-	-	-	-	-	170023	-	-	-	-	-	-	-	-	-	-	-	-	-	-	-	-	-	-	-	-	-	-	-	-	-	-	-	-	-	-	-	-	-	-	-	-	-	-
		-	+	-	-	-	-	-	AT1G76150.1	-	-	CE01215	-	170021	289735	-	-	-	DDB0205505	CG3415-PA	-	-	-	-	-	-	-	-	-	-	-	Os09g37280.2	-	GSPATP00003144001	-	126709	137019	-	652170	RO3G_08530.1	-	-	-	SINFRUP00000169678	44.m00211	974	-	44.m04695	-	-	-	-
		-	-	-	-	-	-	-	-	-	-	-	-	170015	-	-	-	-	-	-	-	-	-	-	-	-	-	-	-	-	-	-	-	-	-	-	-	-	-	-	-	-	-	-	-	-	-	-	-	-	-	-
		-	-	-	-	-	-	-	-	-	-	-	-	170008	-	-	-	-	-	-	-	-	-	-	-	-	-	-	-	-	-	-	-	-	-	-	-	-	-	-	-	-	-	-	-	-	-	-	-	-	-	-
		-	-	-	-	-	-	-	-	-	-	-	-	170005	-	-	-	-	-	-	-	-	-	-	-	-	-	-	-	-	-	-	-	-	-	-	-	-	-	-	-	-	-	-	-	-	-	-	-	-	-	-
IF3X		-	+	-	-	-	-	-	AT5G37475.1	-	-	-	-	170004	-	-	-	OTTDARP00000023135	DDB0187426	CG12131-PA	-	-	ENSGALP00000013321	-	OTTHUMP00000175736	-	-	-	-	240395	-	Os02g02990.1	35206	-	-	57006	-	-	714222	RO3G_14947.1	-	-	GLEAN3_13398	-	-	-	-	-	54369	-	-	-
		-	+	-	-	-	-	GB18504-PA	AT3G11070.1	-	BDEG_03266	-	156414	170003	-	-	-	OTTDARP00000023707	-	-	-	-	ENSGALP00000022917	-	NP_056195.2	-	-	-	-	-	-	Os05g39390.1	33172	-	-	224626	134227	-	564065	-	-	-	-	SINFRUP00000146321	-	-	-	-	53259	-	-	-
DMT1		-	-	-	-	-	-	-	-	-	-	-	-	169993	-	-	-	-	-	-	-	-	-	-	-	-	-	-	-	-	-	Os02g03050.2	-	-	-	-	-	-	-	-	-	-	-	-	-	-	-	-	-	-	-	-
		-	-	-	-	-	-	-	-	-	-	-	-	169989	-	-	-	-	-	-	-	-	-	-	-	-	-	-	-	-	-	-	-	-	-	-	-	-	-	-	-	-	-	-	-	-	-	-	-	-	-	-
		-	-	-	-	-	-	-	-	-	-	-	-	169983	-	-	-	-	-	-	-	-	-	-	-	-	-	-	-	-	-	-	-	-	-	-	-	-	-	-	-	-	-	-	-	-	-	-	-	-	-	-
MST2		-	-	-	-	-	-	-	-	-	-	-	-	169981	-	-	-	-	-	-	-	-	-	-	-	-	-	-	-	-	-	-	-	-	-	-	-	-	-	-	-	-	-	-	-	-	-	-	-	-	-	-
		-	+	-	-	-	-	-	-	-	-	-	-	169967	-	-	-	-	-	-	-	-	-	-	-	-	-	-	79379	-	-	Os01g07880.1	30391	GSPATP00027759001	-	213225	-	-	809109	RO3G_01852.1	-	-	-	-	-	-	-	-	-	-	-	-
		-	-	-	-	-	-	-	-	-	-	-	-	169965	-	-	-	-	-	-	-	-	-	-	-	-	-	-	-	-	-	-	-	-	-	-	-	-	-	-	-	-	-	-	-	-	-	-	-	-	-	-
		-	-	-	-	-	-	-	-	-	-	-	-	169959	-	cgd3_3100	-	-	-	-	-	-	-	-	-	-	-	-	-	-	-	-	-	-	-	-	-	-	-	-	-	-	-	-	-	-	-	-	-	-	-	-
		-	+	+	-	-	-	GB11091-PA	AT5G41150.2	16709	BDEG_06462	CE24855	178005	169954	260300	cgd4_2960	CMH034C	-	DDB0186004	CG3697-PA	19173402	1.m00721	ENSGALP00000004707	-	NP_005227.1	LmjF08.0140	104289	13638	62721	168557	NCU07440	Os03g01100.1	8629	-	30908	201653	129382	MAL13P1.346	589928	RO3G_01472.1	YPL022W	SPCC970.01	GLEAN3_01009	SINFRUP00000137921	13.m00408	22869	TA04325	541.m02057	50503	95725.m00313	Tb927.5.3670	UM03190.1
		-	-	-	-	-	-	-	-	-	-	-	-	169953	-	-	-	-	-	-	-	-	-	-	-	-	-	-	-	-	-	-	-	-	-	-	-	-	-	-	-	-	-	-	-	-	-	-	-	-	-	-
		-	-	-	-	-	-	-	-	-	-	-	-	169951	-	-	-	-	-	-	-	-	-	-	-	-	-	-	-	-	-	-	-	-	-	-	-	-	-	-	-	-	-	-	-	-	-	-	-	-	-	-
IRK1		-	+	-	-	-	-	GB17183-PA	-	12951	-	CE04787	3998	169948	252230	-	-	OTTDARP00000021947	-	CG10369-PA	-	-	ENSGALP00000007506	-	OTTHUMP00000162917	-	131645	32445	-	170615	-	-	-	-	49496	-	134701	-	-	-	-	-	GLEAN3_12834	SINFRUP00000179073	-	3092	-	76.m01571	3835	-	-	-
		-	-	-	-	-	-	-	-	-	-	-	-	169946	-	-	-	-	-	-	19171265	-	-	-	-	-	-	-	-	-	-	-	14545	-	-	169483	-	-	-	-	-	-	-	-	-	-	-	-	-	-	-	-
		-	+	-	-	-	-	-	-	-	-	-	-	169945	-	-	CMO100C	-	-	-	-	-	-	-	-	LmjF27.0920	-	14787	-	-	NCU00080	-	-	-	47763	-	141845	-	-	RO3G_08164.1	-	SPCC594.04c	-	-	-	36373	-	-	-	-	-	UM04724.1
ELG3	exostosin-like glycosyltransferase	-	-	-	-	-	-	-	-	-	-	-	-	169942	-	-	-	-	-	-	-	-	-	-	-	-	-	-	-	-	-	-	-	-	-	-	-	-	-	-	-	-	-	-	-	-	-	-	-	-	-	-
		-	-	-	-	-	-	-	-	-	-	-	-	169939	-	-	-	-	-	-	-	-	-	-	-	-	-	-	-	-	-	-	-	-	-	-	-	-	-	-	-	-	-	-	-	-	-	-	-	-	-	-
		-	-	-	-	-	-	-	-	-	-	-	-	169938	-	-	-	-	-	-	-	-	-	-	-	-	-	-	-	-	-	-	-	-	-	-	-	-	-	-	-	-	-	-	-	-	-	-	-	-	-	-
		-	-	-	-	-	-	-	-	-	-	-	-	169932	-	-	-	-	-	-	-	-	-	-	-	-	-	-	-	-	-	-	-	-	-	-	-	-	-	-	-	-	-	-	-	-	-	-	-	-	-	-
VPS55	involved in vacuolar trafficking	-	+	-	-	-	-	-	AT1G32410.5	-	BDEG_04349	-	226625	169931	-	-	-	-	-	-	-	-	-	-	-	-	120896	-	-	-	NCU06713	Os01g73570.2	-	-	-	217595	-	-	717662	-	-	-	-	-	-	-	-	-	55514	-	-	UM02695.1
		-	-	-	-	-	-	-	-	-	-	-	-	169924	-	-	-	-	-	-	-	-	-	-	-	-	-	-	-	-	-	-	-	-	-	-	-	-	-	-	-	-	-	-	-	-	-	-	-	-	-	-
		-	-	-	-	-	-	-	-	-	-	-	-	169916	-	-	-	-	-	-	-	-	-	-	-	-	-	-	-	-	-	-	-	-	-	-	-	-	-	-	-	-	-	-	-	-	-	-	-	-	-	-
		-	-	-	-	-	-	-	-	-	-	-	-	169885	-	-	-	-	-	-	-	-	-	-	-	-	-	-	-	-	-	-	-	-	-	-	-	-	-	-	-	-	-	-	-	-	-	-	-	-	-	-
		-	-	-	-	-	-	-	-	-	-	-	-	169884	-	-	-	-	-	-	-	-	-	-	-	-	-	-	-	-	-	-	-	-	-	-	-	-	-	-	-	-	-	-	-	-	-	-	-	-	-	-
		-	-	-	-	-	-	-	-	-	-	-	216172	169882	-	-	-	-	-	-	-	-	-	-	-	-	-	-	-	-	-	-	-	-	-	-	-	-	-	-	-	-	-	-	-	-	-	-	-	-	-	-
		-	-	-	-	-	-	-	-	-	-	-	-	169877	-	-	-	-	-	-	-	-	-	-	-	-	-	-	-	-	-	-	-	-	-	-	-	-	-	-	-	-	-	-	-	-	-	-	-	-	-	-
		-	+	-	-	-	-	-	AT5G42480.1	-	-	-	-	169875	-	-	-	-	-	-	-	-	-	-	-	-	-	-	-	-	-	Os02g03000.1	10503	-	-	195672	-	-	206210	-	-	-	-	-	-	-	-	-	-	-	-	-
UPF3	putative UPF3	-	+	-	-	-	-	GB10949-PA	AT1G33980.1	-	BDEG_01074	-	137621	169865	244571	-	-	OTTDARP00000021999	-	CG11184-PC	-	-	ENSGALP00000014025	-	OTTHUMP00000023924	-	235411	38659	-	115210	NCU03435	Os04g35920.2	-	-	-	170493	-	-	672843	RO3G_02019.1	-	SPAC13G7.03	GLEAN3_00502	SINFRUP00000129034	-	-	-	583.m00698	-	-	-	UM00233.1
		-	-	-	+	-	-	-	-	-	-	-	-	169843	-	-	-	-	-	-	-	-	-	-	-	-	-	-	-	-	-	-	-	-	-	-	-	-	-	-	-	-	-	-	-	-	-	-	-	-	-	-
		-	+	-	-	-	-	-	AT5G17520.1	-	-	-	-	169838	-	-	-	-	-	-	-	-	-	-	-	-	-	-	-	-	-	Os04g51330.1	34850	-	-	147872	-	-	730212	-	-	-	-	-	-	-	-	-	-	-	-	-
		-	+	-	-	-	-	GB11546-PA	AT1G68080.1	-	-	-	182740	169836	208883	-	-	OTTDARP00000009861	-	-	-	-	ENSGALP00000038430	-	OTTHUMP00000008735	-	106996	-	-	-	-	Os03g13550.1	-	-	-	-	128715	-	720769	-	-	-	-	SINFRUP00000140099	-	-	-	-	-	-	-	-
	Receptor Mediated Endocytosis family member-related	-	+	-	-	-	-	GB17256-PA	AT2G26890.1	-	-	CE30345	227625	169826	269703	-	-	-	DDB0186910	CG8014-PA	-	9.m00400	ENSGALP00000019064	-	NP_056083.3	LmjF30.2210	238096	17362	-	102958	-	Os10g42439.1	-	-	-	133871	-	-	261749	-	-	-	-	SINFRUP00000142607	-	-	-	-	21978	-	Tb927.6.3500	-
		-	-	-	-	-	-	-	-	-	-	-	-	169813	-	-	-	-	-	-	-	-	-	-	-	-	-	-	-	-	-	-	-	-	-	-	-	-	-	-	-	-	-	-	-	-	-	-	-	-	-	-
		-	+	-	-	-	-	-	AT1G21660.1	-	-	CE27221	-	169796	256148	cgd8_2860	CML319C	-	DDB0167087	-	-	23.m00308	ENSGALP00000017962	-	OTTHUMP00000010633	-	-	-	-	163770	NCU03622	Os12g36180.1	32010	GSPATP00031558001	43133	19885	138195	PF14_0111	816257	RO3G_00978.1	YDR320C	SPAC17A5.12	-	SINFRUP00000127761	129.m00076	11286	-	55.m00251	-	-	-	UM05447.1
		-	-	-	-	-	-	-	-	-	-	-	-	169793	-	-	-	-	-	-	-	-	-	-	-	-	-	-	-	-	-	-	-	-	-	-	-	-	-	-	-	-	-	-	-	-	-	-	-	-	-	-
PPRC4		-	+	-	-	-	-	GB13994-PA	-	61206	-	-	-	169792	-	-	CMQ136C	-	DDB0187627	CG4611-PA	-	-	ENSGALP00000007493	-	NP_056360.2	LmjF36.4810	-	33149	45424	-	-	Os07g11280.2	36704	GSPATP00015707001	42688	233327	-	PFL1605w	763598	-	-	SPAC1093.01	-	SINFRUP00000134930	146.m00111	23415	-	52.m00015	53824	95287.m00255	Tb10.6k15.0120	-
		-	-	-	-	-	-	-	-	-	-	-	-	169777	-	-	-	-	-	-	-	-	-	-	-	-	-	-	-	-	-	-	-	-	-	-	-	-	-	-	-	-	-	-	-	-	-	-	-	-	-	-
		-	-	-	-	-	-	-	-	-	-	-	-	169768	-	-	-	-	-	-	-	-	-	-	-	-	-	-	-	-	-	-	-	-	-	-	-	-	-	-	-	-	-	-	-	-	-	-	-	-	-	-
		-	-	-	-	-	-	-	AT4G25440.1	-	-	-	-	169764	-	-	-	-	-	-	-	-	-	-	-	-	-	-	-	-	-	-	-	-	-	3694	-	-	422910	-	-	-	-	-	-	-	-	-	-	-	-	-
APG4	Autophagy protein	-	+	+	-	-	-	GB16585-PA	AT2G44140.2	65498	BDEG_02879	CE23129	225251	169763	263372	-	-	OTTDARP00000020188	DDB0217191	CG4428-PA	-	72.m00167	ENSGALP00000010165	-	OTTHUMP00000067119	LmjF32.3890	187088	37862	51562	234424	NCU02433	Os03g27350.3	3675	GSPATP00027262001	50253	22659	132301	-	562066	RO3G_08593.1	YNL223W	SPAC19B12.08	-	SINFRUP00000139264	66.m00164	-	-	20.m03953	20340	81079.m00026	Tb927.6.1690	UM05142.1
		-	+	-	-	-	-	-	AT1G77230.1	-	-	-	154287	169761	-	-	-	OTTDARP00000018268	-	-	-	-	ENSGALP00000023888	-	OTTHUMP00000161794	-	56543	-	-	-	-	Os02g34830.1	-	-	-	-	134945	-	411898	-	-	-	-	SINFRUP00000155268	-	-	-	-	-	-	-	-
		-	-	-	-	-	-	-	-	-	-	-	-	169748	-	-	-	-	-	-	-	-	-	-	-	-	-	-	-	-	-	-	-	GSPATP00015697001	-	-	-	-	-	-	-	-	-	-	-	-	-	-	-	-	-	-
FAP238	Flagellar Associated Protein	-	-	-	+	-	-	-	-	66670	-	-	-	169745	-	-	-	-	-	-	-	-	-	-	-	-	-	-	-	-	-	-	-	GSPATP00011299001	-	-	135449	-	-	-	-	-	-	-	62.m00265	-	-	-	-	-	-	-
		-	-	-	-	-	-	-	-	-	-	-	-	169732	-	-	-	-	-	-	-	-	-	-	-	-	-	-	-	-	-	-	-	-	-	-	-	-	-	-	-	-	-	-	-	-	-	-	-	-	-	-
		-	+	-	-	-	-	-	-	-	BDEG_01642	-	224678	169728	-	-	-	-	-	-	-	20.m00273	-	-	-	-	227820	-	69033	-	NCU05516	-	-	-	-	-	-	-	-	RO3G_10271.1	YDL141W	SPBC30D10.07c	-	-	-	-	-	-	55000	-	-	UM03839.1
		-	-	-	-	-	-	-	-	-	-	-	-	169707	-	-	-	-	-	-	-	-	-	-	-	-	-	-	-	-	-	-	-	-	-	-	-	-	-	-	-	-	-	-	-	-	-	-	-	-	-	-
		-	-	-	-	-	-	-	-	-	-	-	-	169700	-	-	-	-	-	-	-	-	-	-	-	-	-	-	-	-	-	-	-	-	-	-	-	-	-	-	-	-	-	-	-	-	-	-	-	-	-	-
		-	-	-	-	-	-	-	-	-	-	-	-	169699	-	-	-	-	-	-	-	-	-	-	-	-	-	-	-	-	-	-	-	-	-	-	-	-	-	-	-	-	-	-	-	-	-	-	-	-	-	-
		-	-	-	-	-	-	-	-	-	-	-	-	169696	-	-	-	-	-	-	-	-	-	-	-	-	-	-	-	-	-	-	-	-	-	-	-	-	-	-	-	-	-	-	-	-	-	-	-	-	-	-
		-	-	-	-	-	-	-	-	-	-	-	-	169691	-	-	-	-	-	-	-	-	-	-	-	-	-	-	-	-	-	-	-	-	-	-	-	-	-	-	-	-	-	-	-	-	-	-	-	-	-	-
		-	+	-	-	-	-	GB13323-PA	AT1G20410.1	-	BDEG_06877	CE24383	94284	169686	230659	-	-	-	DDB0189550	CG3709-PA	-	20.m00319	ENSGALP00000012703	-	NP_653310.1	LmjF11.0530	111864	-	75786	131503	-	Os04g47380.1	36476	GSPATP00033426001	-	200134	156393	-	559906	RO3G_02431.1	-	-	-	SINFRUP00000139555	81.m00121	-	-	-	52642	80746.m00349	Tb11.02.4510	-
		-	-	-	-	-	-	-	-	-	-	-	-	169685	-	-	-	-	-	-	-	-	-	-	-	-	-	-	-	-	-	-	-	-	-	-	-	-	-	-	-	-	-	-	-	-	-	-	-	-	-	-
		-	-	-	-	-	-	-	-	-	-	-	-	169676	-	-	-	-	-	-	-	-	-	-	-	-	-	-	-	-	-	-	-	-	-	-	-	-	-	-	-	-	-	-	-	-	-	-	-	-	-	-
		-	-	-	-	-	-	-	-	-	-	-	-	-	-	-	-	-	-	-	-	-	-	-	-	-	-	-	-	-	-	-	-	-	-	-	-	-	-	-	-	-	-	-	-	-	-	-	-	-	-	-
TIM50	TIM50, translocase of inner mitochondrial membrane, mitochondrial.	-	+	-	-	-	-	GB19639-PA	AT1G55900.1	24304	BDEG_06177	CE06481	220287	169672	-	cgd8_3400	CMN179C	OTTDARP00000021346	-	CG2713-PA	-	-	-	-	OTTHUMP00000068424	-	127482	25683	29368	122269	NCU02943	Os01g55700.1	16518	-	3171	137071	128399	PF07_0110	568582	RO3G_10350.1	YPL063W	SPBC17A3.01c	-	-	-	-	TA16800	-	26300	-	-	UM01344.1
		-	-	-	-	-	-	-	-	-	-	-	-	169660	-	-	-	-	-	-	-	-	-	-	-	-	-	-	-	-	-	-	-	-	-	-	-	-	-	-	-	-	-	-	-	-	-	-	-	-	-	-
HTA16	Histone H2A	-	-	-	-	-	-	-	-	-	-	-	-	-	-	-	-	-	-	-	-	-	-	-	-	-	-	-	-	-	-	-	-	-	-	-	-	-	-	-	-	-	-	-	-	-	-	-	-	-	-	-
HTR15	Histone H3	-	-	-	-	-	-	-	-	-	-	-	-	-	-	-	-	-	-	-	-	-	-	-	-	-	-	-	-	-	-	-	-	-	-	-	-	-	-	-	-	-	-	-	-	-	-	-	-	-	-	-
		-	-	-	-	-	-	-	-	-	-	-	-	169648	-	-	-	-	-	-	-	-	-	-	-	-	-	-	-	-	-	-	-	-	-	-	-	-	-	-	-	-	-	-	-	-	-	-	-	-	-	-
		-	+	-	-	-	-	-	-	16893	-	-	-	169645	-	-	-	-	-	-	-	-	-	-	-	LmjF31.0270	-	29809	52382	-	-	-	30462	-	-	-	131183	-	-	-	-	-	-	-	-	9436	-	-	-	-	-	-
		-	-	-	-	-	-	-	-	-	-	-	-	169642	-	-	-	-	-	-	-	-	-	-	-	-	-	-	-	-	-	-	-	-	-	-	-	-	-	-	-	-	-	-	-	-	-	-	-	-	-	-
ARPL2,ARLP2	ARF-like GTPase	-	-	-	-	-	-	-	-	-	-	-	-	169640	-	-	-	-	-	-	-	-	-	-	-	-	-	-	-	-	-	-	-	-	-	-	-	-	-	-	-	-	-	-	-	-	-	-	-	-	-	-
		-	-	-	-	-	-	-	-	-	-	-	-	169638	-	-	-	-	-	-	-	-	-	-	-	-	-	-	-	-	-	-	-	-	-	-	-	-	-	-	-	-	-	-	-	-	-	-	-	-	-	-
		-	+	-	-	-	-	-	-	-	-	-	169490	169636	-	-	-	-	-	-	-	-	-	-	-	-	236265	31008	-	178601	-	-	-	-	-	-	157656	-	-	-	-	-	-	-	-	-	-	-	57054	-	-	-
		-	-	-	-	-	-	-	-	-	-	-	-	169633	-	-	-	-	-	-	-	-	-	-	-	-	-	-	-	-	-	-	-	-	-	-	-	-	-	-	-	-	-	-	-	-	-	-	-	-	-	-
		-	-	-	-	-	-	-	-	-	-	-	-	169629	-	-	-	-	-	-	-	-	-	-	-	-	-	-	-	-	-	-	-	-	-	172374	-	-	-	-	-	-	-	-	-	-	-	-	-	-	-	-
		-	-	-	-	-	-	-	-	-	-	-	-	169627	-	-	-	-	-	-	-	-	-	-	-	-	-	-	-	-	-	-	-	-	-	-	-	-	-	-	-	-	-	-	-	-	-	-	-	-	-	-
		-	-	-	-	-	-	-	-	-	-	-	-	169626	-	-	-	-	-	-	-	-	-	-	-	-	-	-	-	-	-	-	-	-	-	-	-	-	-	-	-	-	-	-	-	-	-	-	-	-	-	-
		-	-	-	-	-	-	-	-	-	-	-	-	169625	-	-	-	-	-	-	-	-	-	-	-	-	-	-	-	-	-	-	-	-	-	-	-	-	-	-	-	-	-	-	-	-	-	-	-	-	-	-
		-	+	+	-	-	-	GB17596-PA	AT2G34970.1	-	BDEG_00582	CE03107	153443	169610	250710	cgd6_3420	CMR397C	-	DDB0185388	CG3806-PA	-	27.m00234	ENSGALP00000013630	-	OTTHUMP00000173769	LmjF28.2290	225095	25519	30583	247758	NCU02414	Os02g56740.1	8630	GSPATP00007943001	46479	40855	131867	PFL0675c	294934	RO3G_02317.1	YDR211W	SPAC8C9.15c	GLEAN3_15443	SINFRUP00000137932	67.m00234	25729	-	59.m03643	64196	97465.m00134	-	UM04869.1
		-	-	-	-	-	-	-	-	-	-	-	-	169605	-	-	-	-	-	-	-	-	-	-	-	-	-	-	-	-	-	-	-	-	-	-	-	-	-	-	-	-	-	-	-	-	-	-	-	-	-	-
		-	-	-	-	-	-	-	-	-	-	-	-	169599	-	-	-	-	-	-	-	-	-	-	-	-	-	-	-	-	-	-	-	-	-	-	-	-	-	-	-	-	-	-	-	-	-	-	-	-	-	-
		-	-	-	-	-	-	-	-	-	-	-	-	169589	-	-	-	-	-	-	-	-	-	-	-	-	-	-	-	-	-	-	-	-	-	-	-	-	-	-	-	-	-	-	-	-	-	-	-	-	-	-

		-	-	-	-	-	-	-	-	-	-	-	-	169581	-	-	-	-	-	-	-	-	-	-	-	-	-	-	-	-	-	-	-	-	-	-	-	-	-	-	-	-	-	-	-	-	-	-	-	-	-	-
C_370061		-	-	-	-	-	-	-	-	-	-	-	-	169578	-	-	-	-	-	-	-	-	-	-	-	-	-	-	72789	-	-	-	-	-	29551	-	-	-	-	-	-	-	-	-	-	268671	-	-	-	-	-	-
		-	+	-	-	-	-	-	AT5G51180.2	-	-	-	-	169573	-	-	-	-	-	-	-	-	-	-	-	-	-	-	-	-	-	Os08g34750.1	36503	-	-	137321	-	-	837611	-	-	-	-	-	-	-	TA04050	-	-	-	-	-
FAP82	Conserved Uncharacterized Flagellar Associated Protein	+	+	-	+	-	-	GB13345-PA	-	33779	BDEG_03497	-	153643	169559	260148	-	-	OTTDARP00000011748	-	CG16789-PA	-	-	ENSGALP00000006331	GL50803_94322	NP_079002.3	LmjF13.1520	204242	24068	79290	162416	-	-	-	GSPATP00022010001	-	81048	129598	-	-	-	-	-	-	SINFRUP00000133133	12.m00515	10172	-	-	24164	-	Tb11.02.0860	-
		-	-	-	-	-	-	-	-	-	-	-	-	169552	-	-	-	-	-	-	-	-	-	-	-	-	-	-	-	-	-	-	-	-	-	-	-	-	-	-	-	-	-	-	-	-	-	-	-	-	-	-
		-	-	-	-	-	-	-	-	-	-	-	-	169550	-	-	-	-	-	-	-	-	-	-	-	-	-	-	-	-	-	-	-	-	-	-	-	-	-	-	-	-	-	-	-	-	-	-	-	-	-	-
VPS5B	subunit of Retromer complex	-	+	-	-	-	-	-	-	-	-	-	-	169548	207774	-	-	-	-	-	-	-	ENSGALP00000019663	-	OTTHUMP00000172551	-	-	-	-	-	-	-	36908	-	-	-	127738	-	-	-	-	-	-	-	-	-	-	-	-	-	-	UM02548.1
		-	-	-	-	-	-	-	-	-	-	-	-	169536	-	-	-	-	-	-	-	-	-	-	-	-	-	-	-	-	-	-	-	-	-	-	-	-	-	-	-	-	-	-	-	-	-	-	-	-	-	-
		-	-	-	-	-	-	-	-	-	-	-	-	169530	-	-	-	-	-	-	-	-	-	-	-	-	-	-	-	-	-	-	-	-	-	-	-	-	-	-	-	-	-	-	-	-	-	-	-	-	-	-
		-	-	-	-	-	-	-	-	-	-	-	-	169528	-	-	-	-	-	-	-	-	-	-	-	-	-	-	-	-	-	-	-	-	-	-	-	-	-	-	-	-	-	-	-	-	-	-	-	-	-	-
TRP-3		-	-	-	-	-	-	-	-	-	-	-	-	169526	-	-	-	-	-	-	-	-	-	-	-	-	-	-	-	-	-	-	-	-	-	-	-	-	-	-	-	-	-	-	-	-	-	-	-	-	-	-
		-	-	-	-	-	-	-	-	-	-	-	-	169525	-	-	-	-	-	-	-	-	-	GL50803_42048	-	-	-	-	-	-	-	-	-	-	-	-	-	-	-	-	-	-	-	-	-	-	-	-	-	-	-	-
		-	-	-	-	-	-	-	-	-	-	-	-	169521	-	-	-	-	-	-	-	-	-	-	-	-	-	-	-	-	-	-	-	-	-	-	-	-	-	-	-	-	-	-	-	-	-	-	-	-	-	-
		-	+	-	-	-	-	-	AT4G19100.1	63000	-	-	-	169505	-	-	CML309C	-	-	-	-	-	-	-	-	-	-	-	-	-	-	Os06g21530.1	7124	-	44899	8497	-	-	826656	-	-	-	-	-	-	7957	-	-	-	-	-	-
		-	-	-	-	-	-	-	-	-	-	-	-	169496	-	-	-	-	-	-	-	-	-	-	-	-	-	-	-	-	-	-	-	-	-	-	-	-	-	-	-	-	-	-	-	-	-	-	-	-	-	UM05784.1
		-	-	-	-	-	-	-	-	-	-	-	-	169493	-	-	-	-	-	-	-	-	-	-	-	-	-	-	-	-	-	-	-	-	-	-	-	-	-	-	-	-	-	-	-	-	-	-	-	-	-	-
		-	+	-	-	-	-	GB16802-PA	AT4G15802.1	25423	-	-	21369	169488	291521	-	-	OTTDARP00000012796	DDB0217419	CG5446-PA	-	-	ENSGALP00000033119	-	OTTHUMP00000107495	LmjF35.2680	-	-	-	237320	-	Os09g20830.2	-	GSPATP00032287001	36485	39101	-	-	814125	-	-	-	-	SINFRUP00000176566	91.m00171	8110	-	-	-	-	Tb09.211.3955	-
		-	-	-	-	-	-	-	-	-	-	-	-	169484	-	-	-	-	-	-	-	-	-	-	-	-	-	-	-	-	-	-	-	-	-	-	-	-	-	-	-	-	-	-	-	-	-	-	-	-	-	-
		-	+	-	-	-	-	-	-	65167	-	-	-	169483	-	-	-	-	-	-	-	-	-	-	-	-	-	-	-	-	-	-	35925	-	44727	-	128277	-	-	RO3G_14065.1	-	-	-	-	-	10506	-	-	-	-	-	-
		-	-	-	-	-	-	-	-	72414	-	-	-	169481	-	-	-	-	-	-	-	-	-	-	-	-	-	-	-	-	-	-	-	-	-	-	-	-	-	-	-	-	-	-	-	-	-	-	-	-	-	-
		-	-	-	-	-	-	-	-	-	-	-	-	169476	-	-	-	-	-	-	-	-	-	-	-	-	-	-	-	-	-	-	-	-	-	-	-	-	-	-	-	-	-	-	-	-	-	-	-	-	-	-
		-	-	-	-	-	-	-	-	-	-	-	-	169464	-	-	-	-	-	-	-	-	-	-	-	-	-	-	-	-	-	-	-	-	-	-	-	-	-	-	-	-	-	-	-	-	-	-	-	-	-	-
		-	-	-	-	-	-	-	AT1G71040.1	-	-	-	-	169463	-	-	-	-	-	-	-	-	-	-	-	-	-	-	-	-	NCU05042	Os01g03530.2	-	-	-	-	-	-	769549	-	-	-	-	-	-	-	-	-	-	-	-	-
		-	-	-	-	-	-	-	-	-	-	-	-	169462	-	-	-	-	-	-	-	-	-	-	-	-	-	-	-	-	-	-	-	-	-	-	-	-	-	-	-	-	-	-	-	-	-	-	-	-	-	-
	protein of unknown function	-	+	-	-	-	-	GB15023-PA	AT4G03200.1	-	-	CE29948	-	169461	-	-	-	-	DDB0202012	CG8613-PA	-	-	ENSGALP00000011087	-	OTTHUMP00000181658	-	156342	-	70896	171824	NCU09980	Os01g70950.1	-	-	-	205191	-	-	824472	-	-	-	-	SINFRUP00000135049	-	-	-	-	-	-	-	-
		-	-	-	-	-	-	-	-	-	-	-	-	169460	-	-	-	-	-	-	-	-	-	-	-	-	-	-	-	-	-	-	-	-	-	-	-	-	-	-	-	-	-	-	-	-	-	-	-	-	-	-
		-	-	-	-	-	-	-	-	-	-	-	-	169459	-	-	-	-	-	-	-	-	-	-	-	-	-	-	-	-	-	-	-	-	-	-	-	-	-	-	-	-	-	-	-	-	-	-	-	-	-	-
		-	+	-	-	-	-	-	AT1G19600.1	-	-	-	-	169453	-	-	-	-	-	-	-	95.m00147	-	-	-	-	-	-	2897	-	-	Os01g01620.1	37221	-	-	119080	-	-	205994	-	-	-	-	-	-	-	-	-	-	-	-	-
		-	-	-	-	-	-	-	-	-	-	-	-	169452	-	-	-	-	-	-	-	-	-	-	-	-	-	-	-	-	-	-	-	-	-	-	-	-	-	-	-	-	-	-	-	-	-	-	-	-	-	-
RPS13	Ribosomal protein S13, component of cytosolic 80S ribosome and 40S small subunit	-	+	+	-	-	-	GB15692-PA	AT4G00100.1	54773	BDEG_01051	CE04009	149191	169450	-	cgd5_2210	CME054C	OTTDARP00000022815	DDB0186065	CG13389-PB	19173433	17.m00294	ENSGALP00000009821	GL50803_16652	NP_001008.1	LmjF19.0390	181799	35536	58574	235818	NCU03038	Os08g02400.1	27442	GSPATP00015512001	9199	214095	108227	PF13_0316	816561	RO3G_12597.1	YDR064W	SPAC6F6.07c	GLEAN3_22161	SINFRUP00000157487	128.m00102	26221	TA20545	59.m03516	36727	84943.m00196	Tb10.61.1390	UM00658.1
	R1 Protein, alpha-glucan water dikinase	-	-	-	-	-	-	-	-	-	-	-	-	169444	-	-	-	-	-	-	-	-	-	-	-	-	-	-	-	-	-	-	-	-	-	-	-	-	-	-	-	-	-	-	-	-	-	-	-	-	-	-
		-	-	-	-	-	-	-	-	-	-	-	-	169442	-	-	-	-	-	-	-	-	-	-	-	-	-	-	-	-	-	-	-	-	-	-	-	-	-	-	-	-	-	-	-	-	-	-	-	-	-	-
	cathepsin L-like proteinase	-	+	-	-	-	-	GB18912-PA	-	-	-	-	-	169428	-	-	-	-	DDB0204556	CG8947-PA	-	2.m00545	ENSGALP00000021835	GL50803_14983	-	-	-	33873	-	96020	-	-	-	-	-	-	-	-	-	-	-	-	-	-	106.m00141	-	-	-	36902	96201.m00006	-	-
		-	-	-	-	-	-	-	-	-	-	-	-	169418	-	-	-	-	-	-	-	-	-	-	-	-	-	-	-	-	-	-	-	-	-	-	-	-	-	-	-	-	-	-	-	-	-	-	-	-	-	-
EFT2	elongation factor Ts-like protein;	-	+	-	-	-	-	GB13585-PA	AT4G11120.1	-	-	-	-	169416	-	-	-	-	-	-	-	-	-	-	-	-	-	-	-	-	-	Os08g30884.1	33606	-	-	126100	108971	-	807701	-	-	-	-	-	-	-	-	-	-	-	-	-
HPT1	putative hexose-phosphate transporter	-	-	-	-	-	-	-	-	-	-	-	-	169412	-	-	-	-	-	-	-	-	-	-	-	-	-	-	-	-	-	-	-	-	-	-	-	-	-	-	-	-	-	-	-	-	-	-	-	-	-	-
STPK21	Serine/Threonine Protein Kinase Homolog 21 (hypothetical)	-	-	-	-	-	-	-	-	-	-	-	-	169411	-	-	-	-	-	-	-	-	-	-	-	-	-	-	-	-	-	-	-	-	-	-	-	-	-	-	-	-	-	-	-	-	-	-	-	-	-	-
		-	-	-	-	-	-	-	-	-	-	-	-	169407	-	-	-	-	-	-	-	-	-	-	-	-	-	-	-	-	-	-	-	-	-	-	-	-	-	-	-	-	-	-	-	-	-	-	-	-	-	-
		-	-	-	-	-	-	-	-	-	-	-	-	169403	-	-	-	-	-	-	-	-	-	-	-	-	-	-	-	-	-	-	-	-	-	-	-	-	-	-	-	-	-	-	-	-	-	-	-	-	-	-
		-	-	-	-	-	-	-	-	-	-	-	-	169400	-	-	-	-	-	-	-	-	-	-	-	-	-	-	-	-	-	-	-	-	-	-	-	-	-	-	-	-	-	-	-	-	-	-	-	-	-	-
		-	-	-	-	-	-	-	-	-	-	-	-	169395	-	-	-	-	-	-	-	-	-	-	-	-	-	-	-	-	-	-	-	-	42918	-	-	-	-	-	-	-	-	-	-	20870	-	-	-	-	-	-
		-	+	-	-	-	-	-	-	66529	-	-	-	169394	-	-	-	OTTDARP00000023737	-	-	-	-	-	-	-	-	-	36465	-	-	-	-	32934	-	44774	-	-	-	-	-	-	-	-	-	-	-	-	-	-	-	-	UM02626.1
		-	+	-	-	-	-	-	AT3G06980.1	-	-	-	-	169389	-	-	-	-	-	-	-	-	-	-	-	-	-	-	-	-	-	Os03g01830.1	-	-	47365	-	-	-	725593	-	-	-	-	-	-	8997	-	-	-	-	-	-
		-	+	-	-	-	-	-	AT5G06260.1	-	-	-	224517	169386	-	-	-	-	-	CG5149-PA	-	-	-	-	-	-	-	-	-	-	-	Os12g06100.2	-	GSPATP00019729001	48755	171445	-	-	717790	RO3G_10250.1	-	-	-	-	-	1465	-	-	-	-	-	-
ATP11	Putative assembly factor 1 for F1 component of mitochondrial ATP synthase	-	+	-	-	-	-	GB11124-PA	AT2G34050.1	16801	BDEG_02000	-	19411	169384	-	-	CME118C	OTTDARP00000010000	DDB0206464	-	-	-	ENSGALP00000016986	-	OTTHUMP00000009681	LmjF35.1950	-	33853	69735	95270	NCU03296	Os02g20860.1	-	GSPATP00024420001	43752	47204	134305	PFL0490c	197805	RO3G_04994.1	YNL315C	SPAC3A12.12	-	SINFRUP00000179881	146.m00081	261387	TA15600	31.m00931	57874	-	Tb09.244.2700	UM03867.1
		-	-	-	-	-	-	-	-	-	-	-	-	169380	-	-	-	-	-	-	-	-	-	-	-	-	-	-	-	-	-	-	14131	-	-	-	-	-	-	-	-	-	-	-	-	-	-	-	-	-	-	-
		-	-	-	-	-	-	-	-	-	-	-	-	169376	-	-	-	-	-	-	-	-	-	-	-	-	-	-	-	-	-	-	-	-	-	-	-	-	-	-	-	-	-	-	-	-	-	-	-	-	-	-
		-	-	-	-	-	-	-	-	-	-	-	-	169374	-	-	-	-	-	-	-	-	-	-	-	-	-	-	-	-	-	-	-	-	-	-	-	-	-	-	-	-	-	-	-	-	-	-	-	-	-	-
		-	+	+	-	-	-	GB19661-PA	AT5G44200.2	-	BDEG_03090	CE09667	149941	169371	234886	cgd6_1700	CMQ282C	OTTDARP00000011456	DDB0218899	-	19168701	100.m00125	ENSGALP00000011061	-	OTTHUMP00000174211	LmjF30.0540	186993	-	54297	167764	NCU00210	Os02g39890.2	21085	GSPATP00018795001	13520	-	-	PFD0750w	718232	-	YPL178W	SPBC13A2.01c	GLEAN3_09838	SINFRUP00000128783	56.m00285	19374	TA06645	80.m02223	19119	92210.m00098	Tb927.6.1970	UM04861.1
		-	-	-	-	-	-	-	-	-	-	-	-	169364	-	-	-	-	-	-	-	-	-	-	-	-	-	-	-	-	-	-	-	-	-	-	-	-	-	-	-	-	-	-	-	-	-	-	-	-	-	-
		-	-	-	-	-	-	-	-	-	-	-	-	169360	-	-	-	-	-	-	-	-	-	-	-	-	-	-	-	-	-	-	-	-	-	-	-	-	-	-	-	-	-	-	-	-	-	-	-	-	-	-
		-	-	-	-	-	-	-	-	-	-	-	-	169345	-	-	-	-	-	-	-	-	-	-	-	-	-	-	-	-	-	-	-	-	-	-	-	-	-	-	-	-	-	-	-	-	-	-	-	-	-	-
MITC7	mitochondrial substrate carrier	-	+	-	-	-	-	GB10745-PA	AT5G51050.1	-	BDEG_03613	CE37355	181015	169342	298275	-	-	OTTDARP00000004451	-	CG32103-PC	-	-	ENSGALP00000002996	-	NP_998816.1	LmjF34.3060	170757	16512	80319	174876	NCU01564	Os02g10740.1	-	-	-	144666	-	-	-	RO3G_00736.1	YNL083W	SPBC12D12.05c	GLEAN3_09107	SINFRUP00000131855	-	-	-	-	21490	82039.m00036	Tb927.4.1660	UM01222.1
		-	-	-	-	-	-	-	-	-	-	-	-	169324	-	-	-	-	-	-	-	-	-	-	-	-	-	-	-	-	-	-	-	-	-	-	-	-	-	-	-	-	-	-	-	-	-	-	-	-	-	-
		-	-	-	-	-	-	-	-	-	-	-	-	169310	-	-	-	-	-	-	-	-	-	-	-	-	-	-	-	-	-	-	-	-	-	-	-	-	-	-	-	-	-	-	-	-	-	-	-	-	-	-
SMC5,SMC5a	Structural Maintenance of Chromosomes protein 5 homolog.	-	-	-	-	-	-	-	AT5G15920.1	-	-	-	-	169308	293083	-	CMH246C	-	-	-	-	-	-	-	-	-	-	28020	-	-	-	-	-	-	-	-	-	-	-	-	-	-	-	-	-	-	-	-	-	-	-	-
		-	-	-	-	-	-	-	-	-	-	-	-	169303	-	-	-	-	-	-	-	-	-	-	-	-	-	-	-	-	-	-	-	-	-	-	-	-	-	-	-	-	-	-	-	-	-	-	-	-	-	-
		-	-	-	-	-	-	-	-	-	-	-	-	169302	-	-	-	-	-	-	-	-	-	-	-	-	-	-	-	-	-	-	-	-	-	-	-	-	-	-	-	-	-	-	-	-	-	-	-	-	-	-
FAP192	Conserved Uncharacterized Flagellar Associated Protein	+	+	-	+	-	-	-	-	-	BDEG_06211	-	178180	169295	288690	-	-	OTTDARP00000013322	-	-	-	-	ENSGALP00000012822	-	NP_001026885.1	LmjF29.0130	194358	-	69956	116184	-	-	-	GSPATP00015882001	-	-	155269	-	-	-	-	-	-	-	33.m00218	-	-	-	63760	95068.m00197	Tb927.3.5510	-
		-	+	+	-	-	-	GB11773-PA	AT3G24080.1	-	BDEG_01189	CE20865	193918	169281	290746	cgd8_4280	CMT188C	OTTDARP00000020092	DDB0184416	CG5645-PA	-	71.m00152	-	GL50803_10569	OTTHUMP00000067113	-	216912	35624	-	228736	NCU06409	Os05g41100.1	34422	GSPATP00003272001	44555	165236	137618	PF08_0026	826683	RO3G_03014.1	YNL308C	SPAC22G7.05	-	SINFRUP00000167705	15.m00426	-	TA08735	38.m01101	28670	82511.m00170	Tb927.5.840	UM04361.1
		-	-	-	-	-	-	-	-	-	-	-	-	169277	-	-	-	-	-	-	-	-	-	-	-	-	-	-	-	-	-	-	-	-	-	-	-	-	-	-	-	-	-	-	-	-	-	-	-	-	-	-
		-	-	-	-	-	-	-	-	-	-	-	-	169274	-	-	-	-	-	-	-	-	-	-	-	-	-	-	-	-	-	-	-	-	-	-	-	-	-	-	-	-	-	-	-	-	-	-	-	-	-	-
		-	-	-	-	-	-	-	-	-	-	-	-	169270	-	-	-	-	-	-	-	-	-	-	-	-	-	-	-	-	-	-	-	-	-	-	-	-	-	-	-	-	-	-	-	-	-	-	-	-	-	-
		-	-	-	-	-	-	-	-	-	-	-	-	169266	-	-	-	-	-	-	-	-	-	-	-	-	-	-	-	-	-	-	-	-	-	-	157105	-	-	-	-	-	-	-	-	-	-	-	-	-	-	-
		-	-	-	-	-	-	-	-	-	-	-	-	169265	-	-	-	-	-	-	-	-	-	-	-	-	-	-	-	-	-	-	-	-	-	-	-	-	-	-	-	-	-	-	-	-	-	-	-	-	-	-
UAPA2	uric acid-xanthine permease	-	-	-	-	-	-	-	-	-	-	-	-	169264	-	-	-	-	-	-	-	-	-	-	-	-	-	-	-	-	-	-	-	-	52619	-	-	-	-	-	-	SPAC1399.01c	-	-	-	-	-	-	-	-	-	UM00076.1
UAPA1	uric acid-xanthine permease	-	-	-	-	-	-	-	-	55502	-	-	-	169259	-	-	-	-	-	-	-	-	-	-	-	-	-	-	-	-	NCU06918	-	-	-	-	31105	-	-	-	-	-	-	-	-	-	-	-	-	-	-	-	-
		-	+	-	-	-	-	-	-	19895	-	CE35084	138807	169251	388839	-	-	-	DDB0185957	CG6115-PA	-	-	ENSGALP00000022684	-	OTTHUMP00000166945	-	105652	-	-	177835	-	-	-	-	37964	-	142947	-	-	-	-	-	-	SINFRUP00000178318	-	-	-	-	29914	-	-	-
		-	-	-	-	-	-	-	-	-	-	-	-	169250	-	-	-	-	-	-	-	-	-	-	-	-	-	-	-	-	-	-	-	-	-	-	-	-	-	-	-	-	-	-	-	-	-	-	-	-	-	-
		-	-	-	-	-	-	-	-	-	-	-	-	169241	-	-	-	-	-	-	-	-	-	-	-	-	-	-	-	-	-	-	-	-	-	-	-	-	-	-	-	-	-	-	-	-	-	-	-	-	-	-
		-	-	-	-	-	-	-	-	-	-	-	-	169238	-	-	-	-	-	-	-	-	-	-	-	-	-	-	-	-	-	-	-	-	-	-	-	-	-	-	-	-	-	-	-	-	-	-	-	-	-	-
		-	-	-	-	-	-	-	-	-	-	-	-	169232	-	-	-	-	-	-	-	-	-	-	-	-	-	-	-	-	-	-	-	-	-	-	-	-	-	-	-	-	-	-	-	-	-	-	-	-	-	-
		-	-	-	-	-	-	-	-	-	-	-	-	169230	-	-	-	-	-	-	-	-	-	-	-	-	-	-	-	-	-	-	-	-	-	-	-	-	-	-	-	-	-	-	-	-	-	-	-	-	-	-
		-	-	-	-	-	-	-	-	-	-	-	-	169228	-	-	-	-	-	-	-	-	-	-	-	-	-	-	-	-	-	-	-	-	-	-	-	-	-	-	RO3G_14116.1	-	-	-	-	-	-	-	-	-	-	-	-
		-	-	-	-	-	-	-	-	-	-	-	-	169227	-	-	-	-	-	-	-	-	-	-	-	-	-	-	-	-	-	-	-	-	-	-	-	-	-	-	-	-	-	-	-	-	-	-	-	-	-	-
		-	-	-	-	-	-	-	-	-	-	-	-	169226	-	-	-	-	-	-	-	-	-	-	-	-	-	-	-	-	-	-	-	-	-	-	-	-	-	-	-	-	-	-	-	-	-	-	-	-	-	-
		+	+	-	+	-	-	GB16672-PA	-	-	-	-	100218	169222	239079	-	-	-	-	CG9170-PB	-	-	-	GL50803_24423	NP_055771.4	LmjF28.2390	105990	13726	70383	128876	-	-	-	GSPATP00021670001	-	232018	143662	-	-	-	-	-	-	SINFRUP00000163765	57.m00243	-	-	-	-	84963.m00116	Tb927.1.3560	-
		-	-	-	-	-	-	-	-	-	-	-	-	169208	-	-	-	-	-	-	-	-	-	-	-	-	-	-	-	-	-	-	-	-	-	-	-	-	-	-	-	-	-	-	-	-	-	-	-	-	-	-
		-	+	-	-	-	-	GB10957-PA	-	-	-	-	-	169205	382324	-	-	-	-	CG9247-PA	-	-	ENSGALP00000014554	-	-	-	-	-	-	-	-	-	-	-	-	189851	-	-	250852	-	-	-	-	-	-	-	-	-	-	-	-	-
		-	-	-	-	-	-	-	-	-	-	-	-	169198	-	-	-	-	-	-	-	-	-	-	-	-	-	-	-	-	-	-	-	-	-	-	-	-	-	-	-	-	-	-	-	-	-	-	-	-	-	-
		-	+	-	-	-	-	-	AT1G53490.1	-	-	-	-	169191	-	-	-	-	-	-	-	-	-	-	-	-	-	-	68456	-	-	Os02g13810.1	-	-	-	134834	-	-	589793	-	-	-	-	-	-	-	-	-	-	-	-	-
		-	+	-	-	-	-	GB30237-PA	AT1G67120.1	69477	BDEG_04073	CE30377	156074	169185	269724	cgd5_3110	CMJ083C	OTTDARP00000005242	-	CG13185-PA	19173514	45.m00162	ENSGALP00000025327	GL50803_39312	OTTHUMP00000016865	LmjF20.0260	109875	32359	78945	20034	NCU06468	Os10g31864.1	14929	GSPATP00019339001	23838	33	130101	PF14_0326	782171	RO3G_01807.1	YLR106C	SPCC737.08	GLEAN3_23525	SINFRUP00000149074	4.m00662	12982	TA18495	49.m03297	27999	88420.m00063	Tb927.1.880	UM04878.1
		-	-	-	-	-	-	-	-	-	-	-	-	169179	-	-	-	-	-	-	-	-	-	-	-	-	-	-	-	-	-	-	-	-	-	-	-	-	-	-	-	-	-	-	-	-	-	-	-	-	-	-
		-	-	-	-	-	-	-	-	-	-	-	-	169177	-	-	-	-	-	-	-	-	-	-	-	-	-	-	-	-	-	-	-	-	-	-	-	-	-	-	-	-	-	-	-	-	-	-	-	-	-	-
CHR3407	A SWI2/SNF2-like protein	-	-	-	-	-	-	-	-	-	-	-	-	169174	-	-	-	-	-	-	-	-	-	-	-	-	-	-	-	-	-	-	-	-	-	-	-	-	-	-	-	-	-	-	-	-	-	-	-	-	-	-
		-	+	-	-	-	-	-	AT1G27510.1	-	-	-	224504	169161	-	-	-	-	-	-	-	-	-	-	-	-	-	-	-	240172	-	Os01g01790.1	37748	-	47439	183711	135817	-	661990	-	-	-	-	-	-	3770	-	-	57437	-	-	-
		-	-	-	-	-	-	-	-	-	-	-	-	169159	-	-	-	-	-	-	-	-	-	-	-	-	-	-	-	-	-	-	-	-	-	-	-	-	-	-	-	-	-	-	-	-	-	-	-	-	-	-
		-	-	-	-	-	-	-	-	-	-	-	-	169157	-	-	-	-	-	-	-	-	-	-	-	-	-	-	-	-	-	-	-	-	-	-	-	-	-	-	-	-	-	-	-	-	-	-	-	-	-	-
SETi?	possible SET domain protein	-	+	-	-	-	-	GB16860-PA	AT1G57820.2	-	-	-	221977	169156	288107	-	-	OTTDARP00000014750	-	-	-	-	ENSGALP00000006565	GL50803_9850	NP_037414.3	-	206317	24134	46701	226911	-	Os05g01230.1	33391	-	-	152968	-	-	817505	-	-	-	GLEAN3_20441	SINFRUP00000162015	-	-	-	-	-	-	-	-
CDO2	putative cysteine dioxygenase	-	-	-	-	-	-	-	-	-	-	-	-	169155	-	-	-	-	-	-	-	-	-	-	-	-	-	-	-	-	-	-	-	-	-	-	-	-	581995	-	-	-	-	-	-	-	-	-	-	-	-	-
		-	-	-	-	-	-	-	-	-	-	-	-	169146	-	-	-	-	-	-	-	-	-	-	-	-	-	-	-	-	-	-	-	-	-	-	-	-	-	-	-	-	-	-	-	-	-	-	-	-	-	-
		-	-	-	-	-	-	-	-	-	-	-	-	169145	-	-	-	-	-	-	-	-	-	-	-	-	-	-	-	-	-	-	-	-	-	-	-	-	-	-	-	-	-	-	-	-	-	-	-	-	-	-
		-	+	-	-	-	-	GB14294-PA	-	72732	-	-	151537	169142	262301	-	-	-	-	CG32732-PA	-	-	-	-	OTTHUMP00000080580	-	217400	-	-	115756	NCU09827	-	32956	-	-	-	142568	-	-	RO3G_01488.1	YDR257C	-	-	SINFRUP00000151941	-	-	-	-	-	-	-	UM03621.1
		-	-	-	-	-	-	-	-	-	-	-	-	169140	-	-	-	-	-	-	-	-	-	-	-	-	-	-	-	-	-	-	-	-	-	-	-	-	-	-	-	-	-	-	-	-	-	-	-	-	-	-
		-	-	-	-	-	-	-	-	-	BDEG_03432	-	-	169138	-	-	-	-	-	-	-	-	-	-	-	-	-	-	-	-	-	-	-	-	-	-	145621	-	-	-	-	-	-	-	-	-	-	-	-	-	-	-
		-	-	-	-	-	-	-	-	-	-	-	-	169127	-	-	-	-	-	-	-	-	-	-	-	-	-	-	-	-	-	-	-	-	-	-	-	-	-	-	-	-	-	-	-	-	-	-	-	-	-	-
		-	-	-	-	-	-	-	-	-	-	-	-	169121	-	-	-	-	-	-	-	-	-	-	-	-	-	-	-	-	-	-	-	-	-	-	-	-	-	-	-	-	-	-	-	-	-	-	-	-	-	-
		-	+	-	-	-	-	-	AT3G60450.1	-	-	CE34458	193119	169120	-	-	-	-	DDB0189383	-	-	-	-	-	-	-	-	-	-	-	-	-	-	-	-	233005	-	-	551987	-	-	-	-	-	-	-	-	-	-	-	Tb11.02.1940	-
		-	-	-	-	-	-	-	-	-	-	-	-	169116	-	-	-	-	-	-	-	-	-	-	-	-	-	-	-	-	-	-	-	-	-	-	-	-	-	-	-	-	-	-	-	-	-	-	-	-	-	-
SBDP		-	+	-	-	-	-	GB11771-PA	AT4G14040.1	-	-	CE19075	21645	169114	271462	-	-	OTTDARP00000021895	-	-	-	-	ENSGALP00000001274	-	OTTHUMP00000082403	-	203681	-	-	105540	-	Os01g68770.1	-	-	-	159322	-	-	707134	-	-	-	GLEAN3_15423	SINFRUP00000181743	50.m00162	-	-	-	19817	-	-	-
		-	-	-	-	-	-	-	-	-	-	-	-	169112	-	-	-	-	-	-	-	-	-	-	-	-	-	-	-	79346	-	-	-	-	-	-	-	-	-	-	-	-	-	-	-	-	-	-	-	-	-	-
CYP767A1	cytochrome P450, unkown function	-	-	-	-	-	-	-	-	-	-	-	-	169101	-	-	-	-	-	-	-	-	-	-	-	-	-	-	-	-	-	-	-	-	-	-	-	-	-	-	-	-	-	-	-	-	-	-	-	-	-	-
		-	-	-	-	-	-	-	-	-	-	-	-	169098	-	-	-	-	-	-	-	-	-	-	-	-	-	-	-	-	-	-	-	-	-	-	-	-	-	-	-	-	-	-	-	-	-	-	-	-	-	-
		-	-	-	-	-	-	-	-	-	-	-	-	169096	-	-	-	-	-	-	-	-	-	-	-	-	-	38827	-	-	-	-	-	-	-	-	-	-	-	-	-	-	-	-	-	-	-	-	-	-	-	-
		-	-	-	-	-	-	-	-	-	BDEG_01167	-	217708	169091	247667	-	-	-	-	-	-	-	-	-	-	-	-	-	-	20626	-	-	-	-	-	-	-	-	-	-	-	-	-	-	-	-	-	-	-	-	-	-
		-	-	-	-	-	-	-	-	-	-	-	-	169088	-	-	-	-	-	-	-	-	-	-	-	-	-	-	-	-	-	-	-	-	-	-	-	-	-	-	-	-	-	-	-	-	-	-	-	-	-	-
		-	+	-	-	-	-	-	-	67082	-	-	212148	169086	-	-	-	-	-	-	-	-	ENSGALP00000009448	-	-	-	169789	-	-	196090	NCU00039	-	-	-	-	-	-	-	-	-	-	-	-	-	-	-	-	-	-	-	-	-
		-	-	-	-	-	-	-	-	-	-	-	-	169084	-	-	-	-	-	-	-	-	-	-	-	-	-	-	-	-	-	-	-	-	-	-	-	-	-	-	-	-	-	-	-	-	-	-	-	-	-	-
		-	-	-	-	-	-	-	-	-	-	-	-	169066	-	-	-	-	-	-	-	-	-	-	-	-	-	-	-	-	-	-	-	-	-	-	-	-	-	-	-	-	-	-	-	-	-	-	-	-	-	-
		-	-	-	-	-	-	-	-	-	-	-	-	169062	-	-	-	-	-	-	-	-	-	-	-	-	-	-	-	-	-	-	-	-	-	-	-	-	-	-	-	-	-	-	-	-	-	-	-	-	-	-
		-	-	-	-	-	-	-	-	-	-	-	-	169048	-	-	-	-	-	-	-	-	-	-	-	-	-	-	-	-	-	-	-	-	-	-	-	-	-	-	-	-	-	-	-	-	-	-	-	-	-	-
		-	-	-	-	-	-	-	-	-	-	-	-	169044	-	-	-	-	-	-	-	-	-	-	-	-	-	-	-	-	-	-	-	-	-	-	-	-	-	-	-	-	-	-	-	-	-	-	-	-	-	-
		-	+	-	-	-	-	GB17730-PA	AT5G47090.1	-	BDEG_05132	-	157459	169042	-	-	-	-	-	CG7810-PA	-	-	-	-	OTTHUMP00000068574	-	179232	31188	-	83464	-	Os06g04780.1	-	-	-	146343	-	-	796367	RO3G_05685.1	-	-	-	-	-	-	-	-	33385	-	-	-
		-	-	-	-	-	-	-	-	-	-	-	-	169031	-	-	-	-	-	-	-	-	-	-	-	-	-	-	-	-	-	-	-	-	-	-	-	-	-	-	-	-	-	-	-	-	-	-	-	-	-	-
		-	-	-	-	-	-	-	-	-	-	-	-	169030	-	-	-	-	-	-	-	-	-	-	-	-	-	-	-	-	-	-	-	-	-	-	-	-	-	-	-	-	-	-	-	-	-	-	-	-	-	-
		-	+	-	-	-	-	-	AT3G06510.1	1771	-	-	166781	169029	-	-	-	-	-	-	-	-	-	-	-	-	-	-	-	201226	-	Os11g45710.7	24257	-	-	121831	136981	-	225986	-	-	-	-	-	-	-	-	-	-	92777.m00069	-	-
TFB4		-	+	-	-	-	-	GB12027-PA	AT1G18340.1	-	BDEG_05791	CE01686	169977	169016	-	cgd6_5040	-	-	DDB0167192	CG5041-PA	-	222.m00074	ENSGALP00000038750	-	OTTHUMP00000169724	-	159336	-	68603	-	NCU01580	Os02g03340.4	30223	-	44108	201953	130067	-	209865	RO3G_09858.1	YPR056W	SPBC30B4.07c	GLEAN3_28430	SINFRUP00000127843	-	24004	-	-	51576	-	-	UM00922.1
PDF1A		-	+	-	-	-	-	GB10535-PA	AT1G15390.1	22058	BDEG_04839	-	-	169015	-	-	-	-	-	CG31373-PA	-	-	-	-	OTTHUMP00000081353	-	-	-	-	121345	-	Os01g37510.1	31407	-	6914	117886	130591	-	180822	-	-	-	-	SINFRUP00000145596	-	-	-	-	55505	-	-	-
	SEC7/BIG-like ARF-GEF	-	-	-	-	-	-	-	-	-	-	-	-	169014	-	-	-	-	-	-	-	-	-	-	-	-	-	-	-	-	-	-	-	-	-	-	-	-	-	-	-	-	-	-	-	-	-	-	-	-	-	-
		-	-	-	-	-	-	-	-	-	-	-	-	169010	-	-	-	-	-	-	-	-	-	-	-	-	-	-	-	-	-	-	-	-	-	-	-	-	-	-	-	-	-	-	-	-	-	-	-	-	-	-
		-	-	-	-	-	-	-	-	-	-	-	-	169007	-	-	-	-	-	-	-	-	-	-	-	-	-	-	-	-	-	-	-	-	-	-	-	-	-	-	-	-	-	-	-	-	-	-	-	-	-	-
		-	-	-	-	-	-	-	-	-	-	-	-	168996	-	-	-	-	-	-	-	-	-	-	-	-	-	-	-	-	-	-	-	-	-	-	-	-	-	-	-	-	-	-	-	-	-	-	-	-	-	-
		-	-	-	-	-	-	-	-	-	-	-	-	168990	-	-	-	-	-	-	-	-	-	-	-	-	-	-	-	-	-	-	-	-	-	-	-	-	-	-	-	-	-	-	-	-	-	-	-	-	-	-
		-	-	-	-	-	-	-	-	-	-	-	-	168984	-	-	-	-	-	-	-	-	-	-	-	-	-	-	-	-	-	-	30377	-	-	-	-	-	-	-	-	-	-	-	-	-	-	-	-	-	-	-
		-	-	-	-	-	-	-	-	-	-	-	-	168980	-	-	-	-	-	-	-	-	-	-	-	-	-	-	-	-	-	-	-	-	-	-	-	-	-	-	-	-	-	-	-	-	-	-	-	-	-	-
		-	+	-	-	-	-	-	-	-	-	-	130729	168973	-	-	CMK157C	-	-	-	-	-	-	-	-	-	104642	-	-	194978	-	-	-	-	-	-	-	-	-	RO3G_02774.1	-	-	-	-	-	-	-	-	-	-	-	-
		-	-	-	-	-	-	-	-	-	-	-	-	168969	-	-	-	-	-	-	-	-	-	-	-	-	-	-	-	-	-	-	-	-	-	-	-	-	-	-	-	-	-	-	-	-	-	-	-	-	-	-
		-	-	-	-	-	-	-	-	-	-	-	-	168966	-	-	-	-	-	-	-	-	-	-	-	-	-	-	-	-	-	-	-	-	-	-	-	-	-	-	-	-	-	-	-	-	-	-	-	-	-	-
		-	-	-	+	-	-	-	-	-	-	-	-	168963	-	-	-	-	-	-	-	-	-	-	-	-	-	-	-	-	-	-	-	-	-	-	-	-	-	-	-	-	-	-	-	-	-	-	-	-	-	-
		-	-	-	-	-	-	-	-	-	-	-	-	168957	-	-	-	-	-	-	-	-	-	-	-	-	-	-	-	-	-	-	33958	-	-	-	-	-	-	-	-	-	-	-	-	-	-	-	-	-	-	-
		-	-	-	-	-	-	-	-	-	-	-	-	168953	-	-	-	-	-	-	-	-	-	-	-	-	-	-	-	-	-	-	-	-	-	-	-	-	-	-	-	-	-	-	-	-	-	-	-	-	-	-
		-	-	-	-	-	-	-	-	-	-	-	-	168943	-	-	-	-	-	-	-	-	-	-	-	-	-	-	-	-	-	-	-	GSPATP00036969001	-	-	128048	-	-	-	-	-	-	-	61.m00284	-	-	65.m01093	-	-	-	-
		-	+	-	-	-	-	GB14412-PA	-	66499	BDEG_00048	-	227777	168936	-	-	CMO347C	OTTDARP00000008548	DDB0204301	-	-	-	ENSGALP00000017647	-	XP_946889.1	-	-	26083	-	31202	-	-	-	-	-	-	134555	-	-	RO3G_00983.1	-	-	-	-	91.m00155	269666	-	-	-	-	-	-
NAP1	Novel Actin-Like Protein	-	-	-	-	-	-	-	-	-	-	-	-	168932	-	-	-	-	-	-	-	-	-	-	-	-	-	-	-	-	-	-	-	-	-	-	-	-	-	-	-	-	-	-	-	-	-	-	-	-	-	-
		-	-	-	-	-	-	-	-	-	-	-	-	168931	-	-	-	-	-	-	-	-	-	-	-	-	-	-	-	-	-	-	-	-	-	-	-	-	-	-	-	-	-	-	-	-	-	-	-	-	-	-
		-	-	-	-	-	-	-	-	-	-	-	-	168930	-	-	-	-	-	-	-	-	-	-	-	-	-	-	-	-	-	-	-	-	-	-	-	-	-	-	-	-	-	-	-	-	-	-	-	-	-	-
		-	+	-	-	-	-	-	AT5G63080.1	-	-	-	158070	168928	273217	-	-	-	-	-	-	-	-	-	-	LmjF27.1150	128855	29847	-	117188	-	Os01g36630.1	-	-	-	-	-	-	776125	RO3G_09714.1	-	-	-	SINFRUP00000144303	-	6982	-	-	-	-	-	-
		-	+	-	-	-	-	-	AT5G12470.1	-	-	-	-	168924	-	-	CMX001C	-	-	-	-	-	-	-	-	-	-	-	-	-	-	Os01g72710.1	-	-	-	213098	-	-	815393	-	-	-	-	-	-	-	-	-	-	-	-	-
		-	-	-	-	-	-	-	-	-	-	-	-	168923	-	-	-	-	-	-	-	-	-	-	-	-	-	-	-	-	-	-	-	-	-	-	-	-	-	-	-	-	-	-	-	-	-	-	-	-	-	-
		-	-	-	-	-	-	-	AT1G69530.2	-	-	-	-	168912	-	-	-	-	-	-	-	-	-	-	-	-	-	-	-	-	-	-	-	-	-	-	-	-	656451	-	-	-	-	-	-	-	-	-	-	-	-	-
		-	+	-	-	-	-	GB15480-PA	AT2G19080.1	-	BDEG_04183	CE16016	223609	168911	274348	-	-	OTTDARP00000014829	-	CG8004-PA	-	-	ENSGALP00000023859	-	OTTHUMP00000033824	-	234567	-	-	235919	-	Os06g07160.1	-	-	-	223504	143699	-	-	RO3G_10434.1	-	-	GLEAN3_16404	SINFRUP00000138306	-	-	-	-	56159	-	-	-
		-	+	-	-	-	-	GB19633-PA	-	33409	-	CE15999	-	168908	227406	-	-	-	-	CG13466-PA	-	-	-	-	OTTHUMP00000163482	-	-	-	66832	10482	-	-	22493	GSPATP00030564001	44022	166571	128270	-	824186	-	-	-	GLEAN3_26643	SINFRUP00000162789	65.m00225	2178	TA09935	-	52050	-	-	-
		-	-	-	-	-	-	-	-	-	-	-	-	168905	-	-	-	-	-	-	-	-	-	-	-	-	-	-	-	-	-	-	-	-	-	-	-	-	-	-	-	-	-	-	-	-	-	-	-	-	-	-
		-	+	-	-	-	-	GB16483-PA	-	63024	-	CE01381	180902	168887	299268	-	-	OTTDARP00000009401	DDB0188883	CG31663-PA	-	-	ENSGALP00000003574	-	OTTHUMP00000163510	LmjF33.1290	107720	29375	-	116714	-	-	33558	-	46361	-	155511	-	-	-	-	-	-	SINFRUP00000141117	-	3756	-	-	1695	-	Tb10.389.1870	-
		-	-	-	-	-	-	-	-	-	-	-	-	168883	-	-	CMR215C	-	-	-	-	-	-	-	-	-	-	-	-	-	-	-	-	-	33726	164231	-	-	-	-	-	-	-	-	-	-	-	-	-	-	-	-
		-	-	-	-	-	-	-	-	-	-	-	-	168881	-	-	-	-	-	-	-	-	-	-	-	-	-	-	-	-	-	-	23480	-	-	-	-	-	-	-	-	-	-	-	-	-	-	-	-	-	-	-
		-	-	-	-	-	-	-	-	-	-	-	-	168878	-	-	-	-	-	-	-	-	-	-	-	-	-	-	-	-	-	-	-	-	-	-	-	-	-	-	-	-	-	-	-	-	-	-	-	-	-	-
CPH3401	Condensin complex subunit H protein	-	+	-	-	-	-	-	AT2G32590.1	-	-	-	-	168860	-	-	CMF069C	OTTDARP00000016719	-	-	-	-	-	-	-	-	-	28164	-	-	-	-	30276	GSPATP00016468001	-	178582	-	-	246678	-	-	-	-	-	-	-	-	-	60794	-	-	-
		-	-	-	-	-	-	-	-	-	-	-	-	168857	-	-	-	-	-	-	-	-	-	-	-	-	-	-	-	-	-	-	-	-	-	-	-	-	-	-	-	-	-	-	-	-	-	-	-	-	-	-
		-	-	-	-	-	-	-	-	-	-	-	-	168855	-	-	-	-	-	-	-	-	-	-	-	-	-	-	-	-	-	-	-	-	-	-	-	-	-	-	-	-	-	-	-	-	-	-	-	-	-	-
		-	-	-	-	-	-	-	-	-	-	-	-	168853	-	-	-	-	-	-	-	-	-	-	-	-	-	-	-	-	-	-	-	-	-	-	-	-	-	-	-	-	-	-	-	-	-	-	-	-	-	-
		-	+	-	-	-	-	-	AT4G13870.2	62671	-	-	-	168851	-	-	CMD071C	-	-	-	-	-	-	-	-	-	106399	-	-	97672	NCU03765	Os04g03990.1	35493	-	-	-	-	-	297936	RO3G_05744.1	-	-	-	-	-	-	-	-	-	-	-	UM00869.1
		-	-	-	-	-	-	-	-	-	-	-	-	168849	-	-	-	-	-	-	-	-	-	-	-	-	-	-	-	-	-	-	-	-	-	-	-	-	-	RO3G_04233.1	-	-	-	-	-	-	-	-	-	-	-	-
		-	-	-	-	-	-	-	-	-	-	-	-	168848	-	-	-	-	-	-	-	-	-	-	-	-	-	-	-	-	-	-	-	-	-	-	-	-	-	-	-	-	-	-	-	-	-	-	-	-	-	-
		-	-	-	-	-	-	-	-	-	-	-	-	168840	-	-	-	-	-	-	-	-	-	-	-	-	-	-	-	-	-	-	-	-	-	-	-	-	-	-	-	-	-	-	-	-	-	-	-	-	-	-
		-	-	-	-	-	-	-	-	-	-	-	-	168829	-	-	-	-	-	-	-	-	-	-	-	-	-	-	-	-	-	-	-	-	-	-	-	-	-	-	-	-	-	-	-	-	-	-	-	-	-	-

		-	-	-	-	-	-	-	-	-	-	-	-	168823	-	-	-	-	-	-	-	-	-	-	-	-	-	-	-	-	-	-	-	-	-	-	-	-	-	-	-	-	-	-	-	-	-	-	-	-	-	-
		-	-	-	-	-	-	-	-	-	-	-	-	168822	-	-	-	-	-	-	-	-	-	-	-	-	-	-	-	-	-	-	-	-	-	-	-	-	-	-	-	-	-	-	-	-	-	-	-	-	-	-
		-	+	-	-	-	-	GB16306-PA	AT4G17910.1	-	BDEG_03481	CE33941	138269	168820	297949	-	-	-	DDB0218787	CG9870-PA	-	63.m00158	ENSGALP00000038373	-	OTTHUMP00000180706	-	181424	-	28895	209576	NCU00913	Os03g26090.1	11561	-	54588	233732	127588	-	415103	RO3G_14113.1	YJL091C	SPAC144.10c	GLEAN3_26238	SINFRUP00000181706	-	-	-	-	28955	-	-	UM06316.1
		-	-	-	-	-	-	-	-	-	-	-	-	168810	-	-	-	-	-	-	-	-	-	-	-	-	-	-	-	-	-	-	-	-	-	-	-	-	-	-	-	-	-	-	-	-	-	-	-	-	-	-
		-	-	-	-	-	-	-	-	-	-	-	-	168803	-	-	-	-	-	-	-	-	-	-	-	-	-	-	-	-	-	-	-	-	-	-	-	-	-	-	-	-	-	-	-	-	-	-	-	-	-	-
		-	-	-	-	-	-	-	-	-	-	-	-	168789	-	-	-	-	-	-	-	-	-	-	-	-	-	-	-	-	-	-	-	-	-	-	-	-	-	-	-	-	-	-	-	-	-	-	-	-	-	-
		-	-	-	-	-	-	-	-	-	-	-	-	168785	-	-	-	-	-	-	-	-	-	-	-	-	-	-	-	-	-	-	-	-	-	-	-	-	-	-	-	-	-	-	-	-	-	-	-	-	-	-
		-	-	-	-	-	-	-	-	-	-	-	-	168784	-	-	-	-	-	-	-	-	-	-	-	-	-	-	-	-	-	-	-	-	-	-	-	-	-	-	-	-	-	-	-	-	-	-	-	-	-	-
		-	+	-	-	-	-	-	-	-	-	CE33547	225637	168782	252734	-	-	-	-	CG8442-PA	-	-	ENSGALP00000016913	-	OTTHUMP00000161600	-	112937	-	58252	239847	-	-	-	-	-	-	-	-	779200	-	-	-	GLEAN3_24978	SINFRUP00000161099	-	-	-	-	18262	-	-	-
		+	+	-	-	-	-	-	-	-	-	-	97867	168773	285395	-	-	-	-	-	-	-	ENSGALP00000025642	-	NP_714915.2	-	192673	-	62841	-	-	-	-	GSPATP00015939001	-	-	-	-	-	-	-	-	-	SINFRUP00000161036	273.m00023	1783	-	-	55538	-	Tb927.5.3540	-
		-	-	-	-	-	-	-	-	-	-	-	-	168761	-	-	-	-	-	-	-	-	-	-	-	-	-	-	-	-	-	-	-	-	-	-	-	-	-	-	-	-	-	-	-	-	-	-	-	-	-	-
		-	-	-	-	-	-	-	-	-	-	-	-	168759	-	-	-	-	-	-	-	-	-	-	-	-	-	-	-	-	-	-	-	-	-	-	-	-	-	-	-	-	-	-	-	-	-	-	-	-	-	-
		-	-	-	-	-	-	-	-	-	-	-	-	168757	-	-	-	-	-	-	-	-	-	-	-	-	-	-	-	-	-	-	-	-	-	-	-	-	-	-	-	-	-	-	-	-	-	-	-	-	-	-
		-	-	-	-	-	-	-	-	-	-	-	-	168755	-	-	-	-	-	-	-	-	-	-	-	-	-	-	-	-	-	-	-	-	-	-	-	-	-	-	-	-	-	-	-	-	-	-	-	-	-	-
		-	+	-	-	-	-	GB16704-PA	AT1G73960.2	-	BDEG_03576	CE19614	221667	168749	288511	-	-	-	DDB0219766	CG6711-PA	-	-	ENSGALP00000026441	-	NP_003175.1	-	228096	27288	65369	10142	NCU02052	Os09g24440.1	36303	-	-	142028	140691	-	835078	-	YCR042C	SPAC3A12.05c	-	SINFRUP00000153216	-	-	-	-	27954	-	-	-
		-	-	-	-	-	-	-	-	-	-	-	-	168745	-	-	-	-	-	-	-	-	-	-	-	-	-	-	-	-	-	-	-	-	-	-	-	-	-	-	-	-	-	-	-	-	-	-	-	-	-	-
		-	-	-	-	-	-	-	-	-	-	-	-	168740	-	-	-	-	-	-	-	-	-	-	-	-	-	-	-	-	-	-	-	-	-	-	-	-	-	-	-	-	-	-	-	-	-	-	-	-	-	-
		-	-	-	-	-	-	-	-	-	-	-	184970	168736	-	-	-	-	-	-	-	-	-	-	-	-	-	-	-	-	-	-	-	-	-	-	-	-	-	-	-	-	-	-	-	-	-	-	-	-	-	-
		-	-	-	-	-	-	-	-	-	-	-	-	168730	-	-	-	-	-	-	-	-	-	-	-	-	-	-	-	-	-	-	-	-	-	-	-	-	-	-	-	-	-	-	-	-	-	-	-	-	-	-
		-	-	-	-	-	-	-	-	-	-	-	-	168727	-	-	-	-	-	-	-	-	-	-	-	-	-	-	-	-	-	-	-	-	-	-	-	-	-	-	-	-	-	-	-	-	-	-	-	-	-	-
		-	-	-	-	-	-	-	-	-	-	-	-	168725	-	-	-	-	-	-	-	-	-	-	-	-	-	-	-	-	-	-	-	-	-	-	-	-	-	-	-	-	-	-	-	-	-	-	-	-	-	-
		-	-	-	-	-	-	-	-	-	-	-	-	168722	-	-	-	-	-	-	-	-	-	-	-	-	-	-	-	-	-	-	-	-	-	-	-	-	-	-	-	-	-	-	-	-	-	-	-	-	-	-
		-	-	-	-	-	-	-	-	-	-	-	-	168713	-	-	-	-	-	-	-	-	-	-	-	-	-	-	-	-	-	-	-	-	-	-	-	-	-	-	-	-	-	-	-	-	-	-	-	-	-	-
		-	-	-	-	-	-	-	-	-	-	-	-	168709	-	-	-	-	-	-	-	-	-	-	-	-	-	-	-	246296	-	-	-	-	-	-	-	-	-	-	-	-	-	-	-	-	-	-	-	-	-	-
		-	-	-	-	-	-	-	-	-	-	-	-	168698	-	-	-	-	-	-	-	-	-	-	-	-	-	-	-	-	-	-	-	-	-	-	-	-	-	-	-	-	-	-	-	-	-	641.m02556	-	90052.m00097	-	-
		-	-	-	-	-	-	-	-	-	-	-	-	168694	-	-	-	-	-	-	-	-	-	-	-	-	-	-	-	-	-	-	-	-	-	-	-	-	-	-	-	-	-	-	-	-	-	-	-	-	-	-
		-	-	-	-	-	-	-	-	-	-	-	-	168690	-	-	-	-	-	-	-	-	-	-	-	-	-	-	-	-	-	-	-	-	-	-	-	-	-	-	-	-	-	-	-	-	-	-	-	-	-	-
		-	+	-	-	-	-	-	AT1G62780.1	-	-	-	-	168688	-	-	-	-	-	-	-	-	-	-	-	-	-	-	-	-	-	Os04g01540.1	23151	-	-	62970	-	-	581217	-	-	-	-	-	-	-	-	-	-	-	-	-
		-	-	-	-	-	-	-	-	-	-	-	-	168686	-	-	-	-	-	-	-	-	-	-	-	-	-	-	-	-	-	-	-	-	-	-	-	-	-	-	-	-	-	-	-	-	-	-	-	-	-	-
APC1	Anaphase promoting complex subunit 1.	-	+	-	-	-	-	GB19084-PA	AT5G05560.1	38761	BDEG_05220	CE21288	175155	168679	263458	-	CMR081C	-	DDB0186456	CG9198-PA	-	-	ENSGALP00000013420	-	OTTHUMP00000161951	LmjF30.1990	232653	22150	45282	208175	NCU05901	-	11409	GSPATP00038568001	-	166727	-	-	765590	RO3G_07752.1	YNL172W	SPBC106.09	-	SINFRUP00000182657	141.m00129	269532	-	63.m00150	52656	97114.m00222	Tb927.6.3280	UM02427.1
		-	-	-	-	-	-	-	-	-	-	-	-	168676	-	-	-	-	-	-	-	-	-	-	-	-	-	-	-	-	-	-	-	-	-	-	-	-	-	-	-	-	-	-	-	-	-	-	-	-	-	-
		-	-	-	-	-	-	-	-	-	-	-	-	168675	-	-	-	-	-	-	-	-	-	-	-	-	-	-	-	-	-	-	-	-	-	-	-	-	-	-	-	-	-	-	-	-	-	-	-	-	-	-
		-	-	-	-	-	-	-	-	-	-	-	-	168671	-	-	-	-	-	-	-	-	-	-	-	-	-	-	-	-	-	-	-	-	-	-	-	-	-	-	-	-	-	-	-	-	-	-	-	-	-	-
		-	-	-	-	-	-	-	-	-	-	-	-	168669	-	-	-	-	-	-	-	-	-	-	-	-	-	-	-	-	-	Os06g02480.1	-	-	-	122475	-	-	247467	-	-	-	-	-	-	-	-	-	-	-	-	-
		-	-	-	-	-	-	-	-	-	-	-	-	168667	-	-	-	-	-	-	-	-	-	-	-	-	-	-	-	-	-	-	-	-	-	-	-	-	659373	-	-	-	-	-	-	-	-	-	-	-	-	-
		-	-	-	-	-	-	-	-	-	-	-	-	168666	-	-	-	-	-	-	-	-	-	-	-	-	-	-	-	-	-	-	-	-	-	-	-	-	-	-	-	-	-	-	-	-	-	-	-	-	-	-
		-	-	-	-	-	-	-	-	-	-	-	-	168661	-	-	-	-	-	-	-	-	-	-	-	-	-	-	-	-	-	-	-	-	-	-	-	-	-	-	-	-	-	-	-	-	-	-	-	-	-	-
		-	-	-	-	-	-	-	-	-	-	-	-	168658	-	-	-	-	-	-	-	-	-	-	-	-	-	-	-	-	-	-	-	-	-	-	-	-	-	-	-	-	-	-	-	-	-	-	-	-	-	-
		-	-	-	-	-	-	-	-	-	-	-	-	168657	-	-	-	-	-	-	-	-	-	-	-	-	-	-	-	-	-	-	36072	-	-	-	-	-	-	-	-	-	-	-	-	-	-	-	-	-	-	-
DDB2	UV-damaged DNA binding complex subunit 2 protein	-	-	-	-	-	-	-	AT5G58760.1	-	-	-	-	168655	-	-	-	-	-	-	-	-	-	-	-	-	-	-	-	-	-	Os01g04870.1	-	-	-	188047	-	-	829607	-	-	-	-	-	-	-	-	-	-	-	-	-
ELG16	exostosin-like glycosyltransferase	-	-	-	-	-	-	-	-	-	-	-	-	168652	-	-	-	-	DDB0206567	-	-	-	-	-	-	-	-	-	-	-	-	-	-	-	-	-	-	-	-	-	-	-	-	-	-	-	-	-	-	-	-	-
		-	-	-	-	-	-	-	-	-	-	-	-	168651	-	-	-	-	-	-	-	-	-	-	-	-	-	-	-	-	-	-	-	-	-	-	-	-	-	-	-	-	-	-	-	-	-	-	-	-	-	-
		-	-	-	-	-	-	-	-	-	-	-	-	168639	-	-	-	-	-	-	-	-	-	-	-	-	-	-	-	-	-	-	-	-	-	-	-	-	-	-	-	-	-	-	-	-	-	-	-	-	-	-
		-	+	-	-	-	-	GB18380-PA	-	70853	-	CE40254	229252	168638	273898	-	-	OTTDARP00000009256	DDB0169393	CG31543-PB	-	-	ENSGALP00000016230	-	OTTHUMP00000028235	-	107905	27914	-	88939	-	-	35443	-	45723	-	-	-	-	RO3G_10927.1	-	-	GLEAN3_26471	SINFRUP00000146426	-	11752	-	44.m00048	50295	-	-	-
		-	-	-	-	-	-	-	-	-	-	-	-	168636	-	-	-	-	-	-	-	-	-	-	-	-	-	-	-	-	-	-	-	-	-	-	-	-	-	-	-	-	-	-	-	-	-	-	-	-	-	-
		-	-	-	-	-	-	-	-	-	-	-	-	168635	-	-	-	-	-	-	-	-	-	-	-	-	-	-	-	-	-	-	-	-	-	-	-	-	-	-	-	-	-	-	-	-	-	-	-	-	-	-
		-	-	-	-	-	-	-	-	-	-	-	-	168632	-	-	-	-	-	-	-	-	-	-	-	-	-	-	-	-	-	-	-	-	-	-	-	-	-	-	-	-	-	-	-	-	-	-	-	-	-	-
		-	-	-	-	-	-	-	AT1G48380.1	-	-	-	-	168630	-	-	-	-	-	-	-	-	-	-	-	-	-	-	-	-	-	-	-	-	-	74926	-	-	197320	-	-	-	-	-	-	-	-	-	-	-	-	-
		-	+	-	-	-	-	-	AT1G03760.1	-	-	-	210509	168627	-	cgd8_740	-	-	DDB0188440	-	-	-	ENSGALP00000007166	-	OTTHUMP00000076168	-	232137	23733	52353	215973	-	Os01g70330.1	-	-	-	86114	-	PFI0350c	292982	RO3G_04771.1	-	-	-	SINFRUP00000158204	-	-	-	-	55810	-	-	-
		-	+	-	-	-	-	GB17002-PA	AT5G24450.1	-	-	-	-	168625	262286	-	-	-	-	-	-	-	-	-	-	-	-	-	-	-	-	-	-	-	-	136248	-	-	778154	RO3G_10422.1	-	-	-	-	-	-	-	-	-	-	-	-
		-	-	-	-	-	-	-	-	-	-	-	-	168621	-	-	-	-	-	-	-	-	-	-	-	-	-	-	-	-	-	-	-	-	-	-	-	-	-	-	-	-	-	-	-	-	-	-	-	-	-	-
		-	-	-	-	-	-	-	-	-	-	-	-	168618	-	-	-	-	-	-	-	-	-	-	-	-	-	-	-	-	-	-	-	-	-	-	-	-	-	-	-	-	-	-	-	-	-	-	-	-	-	-
		-	-	-	-	-	-	-	-	-	-	-	-	168613	-	-	-	-	-	-	-	-	-	-	-	-	-	-	-	-	-	-	-	-	-	168879	-	-	-	-	-	-	-	-	-	-	-	-	-	-	-	-
		-	-	-	-	-	-	-	-	-	-	-	-	168604	-	-	-	-	-	-	-	-	-	-	-	-	-	-	-	-	-	-	-	-	-	-	-	-	-	-	-	-	-	-	-	-	-	-	-	-	-	-
		-	-	-	-	-	-	-	-	-	-	-	-	168599	-	-	-	-	-	-	-	-	-	-	-	-	-	-	-	-	-	-	-	-	-	-	-	-	-	-	-	-	-	-	-	-	-	-	-	-	-	-
		-	-	-	-	-	-	-	-	-	-	-	-	168598	-	-	-	-	-	-	-	-	-	-	-	-	-	-	-	-	-	-	-	-	-	-	-	-	-	-	-	-	-	-	-	-	-	-	-	-	-	-
		-	-	-	-	-	-	-	-	-	-	-	-	168590	-	-	-	-	-	-	-	-	-	-	-	-	-	-	-	-	-	-	-	-	-	-	-	-	-	-	-	-	-	-	-	-	-	-	-	-	-	-
		-	-	-	-	-	-	-	-	-	-	-	-	168587	-	-	-	-	-	-	-	-	-	-	-	-	-	-	-	-	-	-	-	-	-	-	-	-	-	-	-	-	-	-	-	-	-	-	-	-	-	-
ZRT3	zinc-nutrition responsive transporter	-	+	-	-	-	-	-	AT1G60960.1	-	BDEG_05758	CE37575	-	168584	-	-	CMS155C	-	-	-	-	-	-	-	-	LmjF31.3070	-	-	-	145007	NCU11414	Os07g12890.1	-	-	-	110147	-	-	-	-	YLR130C	-	-	-	-	-	-	-	-	-	-	UM03110.1
		-	+	-	-	-	-	-	AT5G51660.1	-	-	-	-	168575	260006	-	-	-	-	-	-	-	-	-	OTTHUMP00000178468	-	-	-	-	-	-	Os04g18010.1	33689	-	-	57317	-	-	-	-	-	-	-	-	-	-	-	-	-	-	-	-
		-	+	-	-	-	-	-	-	-	-	CE29932	161045	168573	-	-	CMS297C	-	DDB0204541	CG10110-PA	-	-	-	-	-	-	128853	33188	80983	-	-	Os04g18010.3	-	-	-	-	-	-	-	-	-	-	-	SINFRUP00000174522	-	-	-	-	59883	82761.m00581	-	-
		-	-	-	-	-	-	-	-	-	-	-	-	168568	-	-	-	-	-	-	-	-	-	-	-	-	-	-	-	-	-	-	-	-	-	-	-	-	-	-	-	-	-	-	-	-	-	-	-	-	-	-
E2FR1	related to E2F and DP transcription factors; Chlamydomonas specific	-	+	-	-	-	-	-	AT2G18850.1	-	-	-	-	168563	-	-	-	OTTDARP00000022866	DDB0218318	-	-	-	-	-	-	LmjF30.0170	-	-	-	-	NCU08733	Os08g14660.1	34097	GSPATP00003961001	-	-	-	-	580469	-	-	-	-	-	-	-	-	-	-	-	-	-
		-	-	-	-	-	-	-	-	-	-	-	-	168555	-	-	-	-	-	-	-	-	-	-	-	-	-	-	-	-	-	-	-	-	-	-	-	-	-	-	-	-	-	-	-	-	-	-	-	-	-	-
		-	+	-	-	-	-	-	AT2G24940.1	16376	BDEG_05313	-	201522	168536	-	-	CMN188C	OTTDARP00000024043	DDB0204917	CG9066-PA	-	-	ENSGALP00000015952	-	OTTHUMP00000034886	LmjF36.6670	77624	4813	29788	28213	NCU07286	Os02g55060.2	31341	GSPATP00000614001	49495	214053	158525	-	733950	RO3G_14022.1	YPL170W	SPAC25B8.01	GLEAN3_18716	SINFRUP00000182544	1.m00782	260846	-	65.m01095	-	-	Tb927.4.1670	UM03008.1
		-	-	-	-	-	-	-	-	-	-	-	-	168526	-	-	-	-	-	-	-	-	-	-	-	-	-	-	-	-	-	-	-	-	-	-	-	-	-	-	-	-	-	-	-	-	-	-	-	-	-	-
HTB2	Histone H2B	-	-	-	-	-	-	-	-	-	-	-	-	168516	-	-	CMN173C	-	-	-	-	-	-	-	-	-	-	-	-	-	NCU02435	-	-	-	-	-	-	-	-	-	YDR224C	-	-	-	-	-	-	-	-	-	-	UM01505.1
HFO2	Histone H4	-	-	-	-	-	-	-	-	-	-	-	-	168515	-	-	-	-	-	-	-	-	-	-	-	-	-	-	-	-	-	-	-	GSPATP00024550001	-	-	-	-	-	-	-	-	-	-	-	-	-	-	-	-	-	-
		-	-	-	-	-	-	-	-	-	-	-	-	168511	-	-	-	-	-	-	-	-	-	-	-	-	-	-	-	-	-	-	-	-	-	-	-	-	-	-	-	-	-	-	-	-	-	-	-	-	-	-
		-	-	-	-	-	-	-	-	-	-	-	-	168508	-	-	-	-	-	-	-	-	-	-	-	-	-	-	-	-	-	-	-	-	-	-	-	-	-	-	-	-	-	-	-	-	-	-	-	-	-	-
		-	-	-	-	-	-	-	-	-	-	-	-	168504	-	-	-	-	-	-	-	-	-	-	-	-	-	-	-	-	-	-	-	-	-	-	-	-	-	-	-	-	-	-	-	21925	-	-	-	-	-	-
		-	-	-	-	-	-	-	-	-	-	-	-	168503	-	-	-	-	-	-	-	-	-	-	-	-	-	-	-	-	-	-	-	-	-	-	-	-	-	-	-	-	-	-	-	-	-	-	-	-	-	-
		-	+	-	-	-	-	GB13987-PA	AT5G23080.2	-	BDEG_01406	CE00658	39455	168486	297327	-	-	-	-	CG8833-PA	-	-	ENSGALP00000007714	-	-	-	218024	4895	45689	208440	NCU10519	Os06g29400.1	-	-	-	171742	144086	-	569839	RO3G_17129.1	-	SPAC20H4.06c	-	SINFRUP00000153529	-	-	-	-	52573	-	-	UM01045.1
RPS3a	Ribosomal protein S3a, component of cytosolic 80S ribosome and 40S small subunit	-	+	+	-	-	-	GB16500-PA	AT4G34670.1	38843	BDEG_08222	CE00664	170468	168484	286777	cgd4_3160	CMS189C	OTTDARP00000019810	DDB0217947	CG2168-PA	19170767	425.m00056	ENSGALP00000016361	GL50803_16265	NP_000997.1	LmjF35.0420	202499	34920	57012	242621	NCU01452	Os02g18550.1	23124	GSPATP00037365001	17545	212515	131269	PFC1020c	231096	RO3G_12122.1	YML063W	SPAC13G6.02c	GLEAN3_11352	SINFRUP00000129833	3.m01917	26046	TA17795	44.m02744	36460	85052.m00045	Tb10.70.3360	UM05041.1
		-	+	-	-	-	-	-	AT1G09870.1	-	-	-	-	168483	-	-	-	-	DDB0186447	-	-	-	-	-	-	-	-	-	-	197800	-	Os03g60370.1	-	-	-	199305	135057	-	833751	-	-	-	-	-	-	-	-	-	-	-	-	-
		-	-	-	-	-	-	-	-	-	-	-	-	168480	-	-	-	-	-	-	-	-	-	-	-	-	-	-	-	-	-	-	-	-	-	-	-	-	-	-	-	-	-	-	-	-	-	-	-	-	-	-
		-	-	-	-	-	-	-	-	-	-	-	-	168479	-	-	-	-	-	-	-	-	-	-	-	-	-	-	-	-	-	-	-	-	-	-	-	-	-	-	-	-	-	-	-	-	-	-	-	-	-	-
		-	-	-	-	-	-	-	-	-	-	-	-	168477	-	-	-	-	-	-	-	-	-	-	-	-	-	-	-	-	-	-	-	-	-	-	-	-	-	-	-	-	-	-	-	-	-	-	-	-	-	-
HON1	Histone H1	-	+	-	-	-	-	-	-	-	-	CE11442	203039	168461	287378	-	-	-	DDB0186458	CG33864-PA	-	-	ENSGALP00000020094	-	-	-	207166	-	-	8103	-	Os04g18090.1	-	-	54381	232290	129764	-	552493	RO3G_10149.1	-	-	GLEAN3_16087	-	-	9993	-	-	-	-	-	-
		-	-	-	-	-	-	-	-	-	-	-	-	168457	-	-	-	-	-	-	-	-	-	-	-	-	-	-	-	-	-	-	-	-	-	-	-	-	-	-	-	-	-	-	-	-	-	-	-	-	-	-
		-	+	-	-	-	-	-	AT3G47930.1	70295	-	-	-	168454	-	-	CMD093C	-	-	-	-	-	-	-	-	-	-	38173	-	-	-	Os12g04520.1	16741	-	23292	120832	112240	-	255436	-	-	-	-	-	-	268544	-	-	-	-	-	-
		-	-	-	-	-	-	-	-	-	-	-	-	168450	-	-	-	-	-	-	-	-	-	-	-	-	-	-	-	-	-	-	-	-	-	-	-	-	-	-	-	-	-	-	-	-	-	-	-	-	-	-
		-	-	-	-	-	-	-	-	-	-	-	-	168448	-	-	-	-	-	-	-	-	-	-	-	-	-	-	-	-	-	-	-	-	-	-	-	-	-	-	-	-	-	-	-	-	-	-	-	-	-	-
		-	-	-	-	-	-	-	-	-	-	-	-	168438	-	-	-	-	-	-	-	-	-	-	-	-	-	-	-	-	-	-	-	-	-	136618	-	-	-	-	-	-	-	-	-	-	-	-	-	-	-	-
		-	-	-	-	-	-	-	-	-	-	-	-	168437	-	-	-	-	-	-	-	-	-	-	-	-	-	-	-	-	-	-	-	-	-	-	-	-	-	-	-	-	-	-	-	-	-	-	-	-	-	-
		-	-	-	-	-	-	-	-	-	-	-	-	168428	-	-	-	-	-	-	-	-	-	-	-	-	-	-	-	-	-	-	-	-	-	-	-	-	-	-	-	-	-	-	-	-	-	-	-	-	-	-
		-	-	-	-	-	-	-	-	-	-	-	-	168420	-	-	-	-	-	-	-	-	-	-	-	-	-	-	-	-	-	-	-	-	-	-	-	-	-	-	-	-	-	-	-	-	-	-	-	-	-	-
		-	-	-	-	-	-	-	-	-	-	-	-	168417	-	-	-	-	-	-	-	-	-	-	-	-	-	-	-	-	-	-	-	-	-	-	-	-	-	-	-	-	-	-	-	-	-	-	-	-	-	-
		-	-	-	-	-	-	-	-	-	-	-	-	168416	-	-	-	-	-	-	-	-	-	-	-	-	-	-	-	-	-	-	-	-	-	-	-	-	-	-	-	-	-	-	-	-	-	-	-	-	-	-
		-	+	-	-	-	-	-	AT2G22660.2	-	-	-	-	168414	-	-	-	-	-	-	-	-	-	-	-	-	153725	-	-	200527	-	Os11g40590.3	-	-	-	-	-	-	816409	-	-	-	-	-	-	-	-	-	-	-	-	-
		-	-	-	-	-	-	-	-	-	-	-	-	168407	-	-	-	-	-	-	-	-	-	-	-	-	-	-	-	-	-	-	-	-	-	-	-	-	-	-	-	-	-	-	-	-	-	-	-	-	-	-
		-	-	-	-	-	-	-	-	-	-	-	-	168404	-	-	-	-	-	-	-	-	-	-	-	-	-	-	-	-	-	-	-	-	-	-	-	-	-	-	-	-	-	-	-	-	-	-	-	-	-	-
		-	-	-	-	-	-	-	-	-	-	-	-	168402	-	-	-	-	-	-	-	-	-	-	-	-	-	-	-	-	-	-	-	-	-	-	-	-	-	-	-	-	-	-	-	-	-	-	-	-	-	-
		-	-	-	-	-	-	-	-	-	-	-	-	168397	-	-	-	-	-	-	-	-	-	-	-	-	-	-	-	-	-	-	-	-	-	-	-	-	-	-	-	-	-	-	-	-	-	-	-	-	-	-
		-	-	-	-	-	-	-	-	-	-	-	-	168392	-	-	-	-	-	-	-	-	-	-	-	-	-	-	-	-	-	-	-	-	-	-	-	-	-	-	-	-	-	-	-	-	-	-	-	-	-	-
		-	+	-	-	-	-	GB11376-PA	-	61635	-	-	98393	168390	244232	-	-	OTTDARP00000001908	DDB0205872	CG3573-PA	-	155.m00103	ENSGALP00000006906	-	OTTHUMP00000004726	-	106380	27082	58601	174916	-	-	-	GSPATP00003284001	-	33820	-	-	-	-	-	-	GLEAN3_26772	SINFRUP00000153548	81.m00130	-	-	-	32656	93234.m00166	-	-
		-	-	-	-	-	-	-	-	-	-	-	-	168389	-	-	-	-	-	-	-	-	-	-	-	-	-	-	-	-	-	-	-	-	-	-	-	-	-	-	-	-	-	SINFRUP00000146114	-	-	-	-	-	-	-	-
		-	-	-	-	-	-	-	-	-	-	-	-	168386	-	-	-	-	-	-	-	-	-	-	-	-	-	-	-	-	NCU05389	-	30671	-	-	-	-	-	-	-	-	-	-	-	-	-	-	-	-	-	-	-
APC6	Anaphase promoting complex subunit 6	-	-	-	-	-	-	-	AT1G78770.1	-	-	-	-	168379	-	-	-	-	-	-	-	-	-	-	-	-	-	-	-	-	-	-	-	-	-	-	-	-	-	-	-	-	-	-	-	-	-	-	-	-	-	-
		-	-	-	-	-	-	-	-	-	-	-	-	168373	-	-	-	-	-	-	-	-	-	-	-	-	-	-	-	-	-	-	-	-	-	-	-	-	-	-	-	-	-	-	-	-	-	-	-	-	-	-
		-	-	-	-	-	-	-	AT2G30470.1	-	-	-	-	168369	-	-	-	-	-	-	-	-	-	-	-	-	-	-	-	-	-	Os07g37610.1	-	-	-	120152	-	-	234125	-	-	-	-	-	-	-	-	-	-	-	-	-
		-	+	-	-	-	-	-	AT1G56050.1	54165	-	-	-	168365	-	-	CMT184C	-	DDB0184484	-	-	-	-	-	-	LmjF27.1830	-	20905	44703	-	-	Os03g56840.1	27695	-	37067	57046	126407	-	550540	-	-	-	-	-	-	511	-	-	-	-	-	-
		-	-	-	-	-	-	-	-	-	-	-	-	168363	-	-	-	-	-	-	-	-	-	-	-	-	-	-	-	-	-	-	-	-	-	-	-	-	-	-	-	-	-	-	-	-	-	-	-	-	-	-
		-	-	-	-	-	-	-	-	-	-	-	-	168356	-	-	-	-	-	-	-	-	-	-	-	-	-	-	-	-	-	-	-	-	-	-	-	-	-	-	-	-	-	-	-	-	-	-	-	-	-	-
		-	-	-	-	-	-	-	-	-	-	-	-	168351	-	-	-	-	-	-	-	-	-	-	-	-	-	-	-	-	-	-	-	-	-	-	-	-	-	-	-	-	-	-	-	-	-	-	-	-	-	-
		-	-	-	-	-	-	-	-	-	-	-	-	168339	-	-	-	-	-	-	-	-	-	-	-	-	-	-	-	-	-	-	-	-	-	-	-	-	-	-	-	-	-	-	-	-	-	-	-	-	-	-
		-	-	-	-	-	-	-	-	-	-	-	-	-	-	-	-	-	-	-	-	-	-	-	-	-	-	-	-	-	-	-	-	-	-	-	-	-	-	-	-	-	-	-	-	-	-	-	-	-	-	-
		-	-	-	-	-	-	-	-	-	-	-	-	168317	-	-	-	-	-	-	-	-	-	-	-	-	-	-	-	-	-	-	-	-	-	-	-	-	-	-	-	-	-	-	-	-	-	-	-	-	-	-
		-	-	-	-	-	-	-	-	-	-	-	-	168313	-	-	-	-	-	-	-	-	-	GL50803_8960	-	-	-	-	-	-	-	-	30452	-	-	118305	-	-	-	-	-	-	-	-	-	-	-	-	56446	-	-	-
		-	-	-	-	-	-	-	-	-	-	-	-	168312	299319	-	-	OTTDARP00000021806	-	-	-	-	-	-	-	-	-	-	-	-	-	-	-	-	42741	-	-	-	-	-	-	-	-	-	-	268904	-	-	-	-	-	-
		-	-	-	-	-	-	-	-	-	-	-	164816	168311	-	-	-	-	-	-	-	-	-	-	-	-	206245	-	-	140529	-	-	-	-	-	-	-	-	-	-	-	-	-	-	-	-	-	-	-	-	-	-
		-	-	-	-	-	-	-	-	-	-	-	-	168310	-	-	-	-	-	-	-	-	-	-	-	-	-	-	-	-	-	-	-	-	-	-	-	-	-	-	-	-	-	-	-	-	-	-	-	-	-	-
		-	-	-	-	-	-	-	-	-	-	-	-	168307	-	-	-	-	-	-	-	-	-	-	-	-	-	-	-	-	-	-	-	-	-	-	-	-	-	-	-	-	-	-	-	-	-	-	-	-	-	-
		-	+	-	-	-	-	-	-	-	-	-	31306	168306	230648	-	-	-	-	-	-	-	-	-	NP_003763.2	-	115278	11035	-	245290	-	-	-	-	-	-	133113	-	-	-	-	-	-	SINFRUP00000141973	928.m00002	-	-	-	-	90434.m00249	-	UM06436.1
		-	-	-	-	-	-	-	-	-	-	-	-	168302	-	-	-	-	-	-	-	-	-	-	-	-	-	-	-	-	-	-	-	-	-	-	-	-	-	-	-	-	-	-	-	-	-	-	-	-	-	-
		-	-	-	-	-	-	-	AT1G17277.1	-	-	-	-	168290	-	-	-	-	-	-	-	-	-	-	-	-	-	-	-	-	-	Os02g30480.1	-	-	-	-	-	-	767643	-	-	-	-	-	-	-	-	-	-	-	-	-
		-	-	-	-	-	-	-	-	-	-	-	-	168285	-	-	-	-	-	-	-	-	-	-	-	-	-	-	-	-	-	-	-	-	-	-	-	-	-	-	-	-	-	-	-	-	-	-	-	-	-	-
		-	-	-	-	-	-	-	-	63473	-	-	-	168283	-	-	-	-	-	-	-	-	-	-	-	-	-	-	-	-	NCU08076	-	-	-	-	-	-	-	-	-	-	-	-	-	-	-	-	-	-	-	-	-
		-	-	-	-	-	-	-	-	-	-	-	-	168281	-	-	-	-	-	-	-	-	-	-	-	-	-	-	-	-	-	-	-	-	-	-	-	-	-	-	-	-	-	-	-	-	-	-	-	-	-	-
		-	-	-	-	-	-	-	AT4G16170.1	-	-	-	-	168275	-	-	-	-	-	-	-	-	-	-	-	-	-	-	-	-	-	-	-	-	-	191923	-	-	833666	-	-	-	-	-	-	-	-	-	-	-	-	-
		-	-	-	-	-	-	-	-	-	-	-	-	168272	-	-	-	-	-	-	-	-	-	-	-	-	-	-	-	-	-	-	-	-	-	-	-	-	-	-	-	-	-	-	-	-	-	-	-	-	-	-
		-	-	-	-	-	-	-	-	-	-	-	-	168271	-	-	-	-	-	-	-	-	-	-	-	-	-	-	-	-	-	-	-	-	-	-	-	-	-	-	-	-	-	-	-	-	-	-	-	-	-	-
		-	-	-	-	-	-	-	-	-	-	-	-	168268	-	-	-	-	-	-	-	-	-	-	-	-	-	-	-	-	-	-	-	-	-	-	-	-	-	-	-	-	-	-	-	-	-	-	-	-	-	-
		-	-	-	-	-	-	-	-	-	-	-	-	168264	-	-	-	-	-	-	-	-	-	-	-	-	-	-	-	-	-	-	-	-	-	-	-	-	-	-	-	-	-	-	-	-	-	-	-	-	-	-
		-	-	-	-	-	-	-	-	-	-	-	-	168263	-	-	-	-	-	-	-	-	-	-	-	-	-	-	-	-	-	-	-	-	-	-	-	-	-	-	-	-	-	-	-	-	-	-	-	-	-	-
		-	-	-	-	-	-	-	-	-	-	-	-	168262	-	-	-	-	-	-	-	-	-	-	-	-	-	-	-	-	-	-	-	-	-	-	-	-	-	-	-	-	-	-	-	-	-	-	-	-	-	-
REF1	RNA Export Factor required for mRNA export out of the nucleus	-	+	-	-	-	-	GB18402-PA	AT5G59950.1	-	BDEG_05851	-	161051	168261	288644	-	-	-	DDB0186471	CG1101-PA	-	-	ENSGALP00000011696	-	NP_005773.2	-	-	30246	-	126384	NCU07421	Os03g17010.1	36431	-	-	57202	157747	PFF0760w	815363	RO3G_01210.1	-	SPBC106.12c	GLEAN3_10685	SINFRUP00000132916	-	-	TA14065	-	53742	-	-	UM01000.1
		-	-	-	-	-	-	-	-	-	-	-	-	168243	-	-	-	-	-	-	-	-	-	-	-	-	-	-	-	-	-	-	-	-	-	-	-	-	-	-	-	-	-	-	-	-	-	-	-	-	-	-
		-	-	-	-	-	-	-	-	-	-	-	-	168240	-	-	-	-	-	-	-	-	-	-	-	-	-	-	-	-	-	-	-	-	-	-	-	-	-	-	-	-	-	-	-	-	-	-	-	-	-	-
		-	-	-	-	-	-	-	-	-	-	-	-	168238	-	-	-	-	-	-	-	-	-	-	-	-	-	-	-	-	-	-	-	-	-	-	-	-	-	-	-	-	-	-	-	-	-	-	-	-	-	-
		-	+	-	-	-	-	GB18448-PA	AT4G30990.1	71075	BDEG_07614	CE02652	223374	168235	270582	-	-	-	DDB0205024	CG4554-PA	-	-	ENSGALP00000025653	-	XP_941315.1	LmjF35.0670	233343	11008	65627	130178	NCU02494	Os01g40250.1	-	-	43464	150212	135013	-	174788	RO3G_14433.1	YBL004W	SPBC56F2.04	-	SINFRUP00000139133	73.m00135	268455	-	-	54820	-	Tb10.70.3570	UM04356.1
		-	-	-	-	-	-	-	-	-	-	-	-	168234	-	-	-	-	-	-	-	-	-	-	-	-	-	-	-	-	-	-	-	-	-	-	-	-	-	-	-	-	-	-	-	-	-	-	-	-	-	-
		-	-	-	-	-	-	-	-	-	-	-	-	168224	-	-	-	-	-	-	-	-	-	-	-	-	-	-	-	-	-	-	-	-	-	201778	-	-	-	-	-	-	-	-	-	-	-	-	-	-	-	-
		-	-	-	-	-	-	-	-	-	-	-	-	168223	-	-	-	-	-	-	-	-	-	-	-	-	-	-	-	-	-	-	-	-	-	-	-	-	-	-	-	-	-	-	-	-	-	-	-	-	-	-
PGM6	phosphoglycerate/bisphosphoglycerate mutase family protein	-	-	-	-	-	-	-	-	-	-	-	-	-	-	-	-	-	-	-	-	-	-	-	-	-	-	-	-	-	-	-	-	-	-	-	-	-	-	-	-	-	-	-	-	-	-	-	-	-	-	-
		-	-	-	-	-	-	-	-	-	-	-	-	168220	-	-	-	-	-	-	-	-	-	-	-	-	-	-	-	-	-	-	-	-	-	-	-	-	-	-	-	-	-	-	-	-	-	-	-	-	-	-
		-	-	-	-	-	-	-	-	-	-	-	-	168216	-	-	-	-	-	-	-	-	-	-	-	-	-	-	-	-	-	-	-	-	-	-	-	-	-	-	-	-	-	-	-	-	-	-	-	-	-	-
		-	-	-	-	-	-	-	-	-	-	-	-	168204	-	-	-	-	-	-	-	-	-	-	-	-	-	-	-	-	-	-	-	-	-	-	-	-	-	-	-	-	-	-	-	-	-	-	-	-	-	-
		-	-	-	-	-	-	-	-	-	-	-	-	168198	-	-	-	-	-	-	-	-	-	-	-	-	-	-	-	-	-	-	-	-	-	-	-	-	-	-	-	-	-	-	-	-	-	-	-	-	-	-
		-	-	-	-	-	-	-	-	-	-	-	-	168196	-	-	-	-	-	-	-	-	-	-	-	-	-	-	-	-	-	-	-	-	-	-	-	-	-	-	-	-	-	-	-	-	-	-	-	-	-	-
		-	-	-	-	-	-	-	-	-	-	-	-	168182	-	-	-	-	-	-	-	-	-	-	-	-	-	-	-	-	-	-	-	-	-	-	-	-	-	-	-	-	-	-	-	-	-	-	-	-	-	-
		-	-	-	-	-	-	-	-	-	-	-	-	168180	-	-	-	-	-	-	-	-	-	-	-	-	-	-	-	-	-	-	-	-	-	-	-	-	-	-	-	-	-	-	-	-	-	-	-	-	-	-
		-	-	-	-	-	-	-	-	-	-	-	-	168177	-	-	-	-	-	-	-	-	-	-	-	-	-	-	-	-	-	-	-	-	-	-	-	-	-	-	-	-	-	-	-	-	-	-	-	-	-	-
		-	-	-	-	-	-	-	-	-	-	-	-	168173	-	-	-	-	-	-	-	-	-	-	-	-	-	-	-	-	-	-	-	-	-	-	-	-	-	-	-	-	-	-	-	-	-	-	-	-	-	-
		-	-	-	-	-	-	-	-	-	-	-	-	168133	-	-	-	-	-	-	-	-	-	-	-	-	-	-	-	-	-	-	34970	-	-	-	-	-	-	-	-	-	-	-	-	-	-	-	-	-	-	-
CHR3415		-	-	-	-	-	-	-	-	15768	-	-	-	168128	-	-	-	-	-	CG7376-PA	-	-	-	-	-	-	-	-	-	-	-	-	-	-	-	-	-	-	-	-	-	SPAC144.05	-	-	-	-	-	-	-	-	-	UM00434.1
		-	-	-	-	-	-	-	-	-	-	-	-	168124	-	-	-	-	-	-	-	-	-	-	-	-	-	-	-	-	-	-	-	-	-	-	-	-	-	-	-	-	-	-	-	-	-	-	-	-	-	-
		-	-	-	-	-	-	-	-	-	-	-	-	168123	-	-	-	-	-	-	-	-	-	-	-	-	-	-	-	-	-	-	-	-	-	-	-	-	-	-	-	-	-	-	-	-	-	-	-	-	-	-
		-	-	-	-	-	-	-	AT5G13610.1	-	-	-	-	168121	-	-	-	-	-	-	-	-	-	-	-	-	-	-	-	-	-	-	-	-	-	-	-	-	-	-	-	-	-	-	-	-	-	-	-	-	-	-
		-	-	-	-	-	-	-	-	-	-	-	-	168119	-	-	-	-	-	-	-	-	-	-	-	-	-	-	-	-	-	-	-	-	-	-	-	-	-	-	-	-	-	-	-	-	-	-	-	-	-	-
		-	-	-	-	-	-	-	-	-	-	-	-	168107	-	-	-	-	-	-	-	-	-	-	-	-	-	-	-	-	-	-	-	-	-	-	-	-	-	-	-	-	-	-	-	-	-	-	-	-	-	-
		-	-	-	-	-	-	-	-	-	-	-	-	168106	-	-	-	-	-	-	-	-	-	-	-	-	-	-	-	-	-	-	-	-	-	-	-	-	-	-	-	-	-	-	-	-	-	-	-	-	-	-
		-	-	-	-	-	-	-	-	-	-	-	-	168098	-	-	-	-	-	-	-	-	-	-	-	-	-	-	-	113873	-	-	-	-	-	-	-	-	-	-	-	-	-	-	-	-	-	-	-	-	-	-
		-	+	-	-	-	-	GB18267-PA	-	-	BDEG_01584	CE34214	173602	168092	-	-	-	-	-	CG12034-PA	-	15.m00313	ENSGALP00000001406	-	OTTHUMP00000016965	LmjF08.0200	135867	-	-	239044	-	-	-	GSPATP00018489001	-	-	-	-	-	-	-	SPBC32F12.01c	-	SINFRUP00000150555	71.m00179	-	-	-	30983	-	Tb927.5.3710	UM02847.1
		-	-	-	-	-	-	-	-	-	-	-	-	168091	-	-	-	-	-	-	-	-	-	-	-	-	-	-	-	-	-	-	-	-	-	-	-	-	-	-	-	-	-	-	-	-	-	-	-	-	-	-
		-	-	-	-	-	-	-	-	-	-	-	-	168087	-	-	-	-	-	-	-	-	-	-	-	-	-	-	-	-	-	-	-	-	-	-	-	-	-	-	-	-	-	-	-	-	-	-	-	-	-	-

		-	+	-	-	-	-	-	-	-	-	-	-	168079	-	-	CMD131C	-	-	-	-	-	-	-	-	-	-	-	46356	-	-	-	33303	GSPATP00024232001	-	-	-	-	-	RO3G_17115.1	-	-	-	-	-	-	-	-	-	-	-	-
		-	-	-	-	-	-	-	-	-	-	-	-	168076	-	-	-	-	-	-	-	-	-	-	-	-	-	-	-	-	-	-	-	-	-	-	-	-	-	-	-	-	-	-	-	-	-	-	-	-	-	-
PSBY	Ycf32-related subunit of photosystem II	-	-	-	-	-	-	-	-	-	-	-	-	168074	-	-	-	-	-	-	-	-	-	-	-	-	-	-	-	-	-	-	24292	-	-	-	-	-	-	-	-	-	-	-	-	-	-	-	-	-	-	-
		-	-	-	-	-	-	-	-	-	-	-	-	168066	-	-	-	-	-	-	-	-	-	-	-	-	-	-	-	-	-	-	-	-	-	-	-	-	-	-	-	-	-	-	-	-	-	-	-	-	-	-
		-	-	-	-	-	-	-	-	-	-	-	-	168064	-	-	-	-	-	-	-	-	-	-	-	-	-	-	-	-	-	-	-	-	-	-	-	-	-	-	-	-	-	-	-	-	-	-	-	-	-	-
		-	-	-	-	-	-	-	-	-	-	-	-	168060	-	-	-	-	-	-	-	-	-	-	-	-	-	-	-	-	-	-	-	-	-	-	-	-	-	-	-	-	-	-	-	-	-	-	-	-	-	-
		-	-	-	-	-	-	-	-	-	-	-	-	168057	-	-	-	-	-	-	-	-	-	-	-	-	-	-	-	-	-	-	-	-	-	-	-	-	-	-	-	-	-	-	-	-	-	-	-	-	-	-
		-	-	-	-	-	-	-	-	-	-	-	-	168055	-	-	-	-	-	-	-	-	-	-	-	-	-	-	-	-	-	-	-	-	-	-	-	-	-	-	-	-	-	-	-	-	-	-	-	-	-	-
		-	+	-	-	-	-	GB18118-PA	AT5G14870.1	-	-	-	63273	168044	250137	-	-	OTTDARP00000010274	-	CG8585-PD	-	-	ENSGALP00000038153	-	OTTHUMP00000176458	-	118006	-	-	82457	-	Os03g44440.1	-	-	-	-	137202	-	413558	-	-	-	GLEAN3_26288	SINFRUP00000160459	14.m00269	5488	-	-	-	-	-	-
		-	-	-	-	-	-	-	-	-	-	-	-	168043	-	-	-	-	-	-	-	-	-	-	-	-	-	-	-	-	-	-	-	-	-	-	-	-	-	-	-	-	-	-	-	-	-	-	-	-	-	-
		-	+	-	-	-	-	-	AT5G10700.1	32278	BDEG_06142	CE36903	219097	168037	-	cgd8_3410	CMP293C	OTTDARP00000005490	-	-	-	-	ENSGALP00000026758	GL50803_28232	NP_001013685.1	-	138047	14689	71370	229113	-	Os04g51570.1	18862	-	-	116860	-	PFF0515c	809187	RO3G_14125.1	-	-	-	SINFRUP00000142001	-	-	TA07835	38.m01105	58974	-	-	UM01357.1
		-	-	-	-	-	-	-	-	-	-	-	-	168036	-	-	-	-	-	-	-	-	-	-	-	-	-	-	-	-	-	-	-	-	-	-	-	-	-	-	-	-	-	-	-	-	-	-	-	-	-	-
FAP135	Flagellar Associated Protein	-	-	-	+	-	-	-	-	-	-	-	-	168028	-	-	-	-	-	-	-	-	-	-	-	-	-	-	-	-	-	-	-	GSPATP00000882001	-	-	132832	-	647445	-	-	-	-	-	59.m00281	-	-	-	-	-	-	-
		-	-	-	-	-	-	-	-	-	-	-	-	168024	-	-	-	-	-	-	-	-	-	-	-	-	-	-	-	-	-	-	-	-	-	-	-	-	-	-	-	-	-	-	-	-	-	-	-	-	-	-
KDG5	diacylglycerol kinase	-	-	-	-	-	-	-	-	-	-	-	-	168023	-	-	-	-	-	-	-	-	-	-	-	-	-	-	-	-	-	-	-	-	-	-	-	-	-	-	-	-	-	-	-	-	-	-	-	-	-	-
		-	+	-	-	-	-	GB11393-PA	-	-	-	-	229238	168022	271170	-	-	-	DDB0205275	-	-	-	ENSGALP00000006597	-	OTTHUMP00000019712	LmjF25.2110	123781	24946	70052	220648	-	-	-	GSPATP00003159001	-	-	138971	-	-	-	-	-	-	SINFRUP00000130234	-	-	-	59.m03359	60261	-	Tb927.3.2210	-
		-	-	-	-	-	-	-	-	65211	-	-	-	168021	-	-	-	-	-	-	-	-	-	-	-	-	-	-	-	-	-	-	-	-	-	-	-	-	-	-	-	-	-	-	-	-	-	-	-	-	-	-
XUV2	putative xanthine/uracil/vitamin C permease	-	-	-	-	-	-	-	-	-	-	-	-	168019	-	-	-	-	-	-	-	-	-	-	-	-	-	-	-	-	-	-	-	-	-	-	-	-	-	-	-	-	-	-	-	-	-	-	88428.m00087	-	-
		-	-	-	-	-	-	-	-	-	-	-	-	168017	-	-	-	-	-	-	-	-	-	-	-	-	-	-	-	-	-	-	-	-	-	-	-	-	-	-	-	-	-	-	-	-	-	-	-	-	-	-
		-	-	-	-	-	-	-	-	-	-	-	-	168013	-	-	-	-	-	-	-	-	-	-	-	-	-	-	-	-	-	-	-	-	-	-	-	-	-	-	-	-	-	-	-	-	-	-	-	-	-	-
		-	-	-	-	-	-	-	-	-	-	-	-	168003	-	-	-	-	-	-	-	-	-	-	-	-	-	-	-	-	-	-	-	-	-	-	-	-	-	-	-	-	-	-	-	-	-	-	-	-	-	-
		-	-	-	-	-	-	-	-	-	-	-	-	168002	-	-	-	-	-	-	-	-	-	-	-	-	-	-	-	-	-	-	-	-	-	-	-	-	-	-	-	-	-	-	-	-	-	-	-	-	-	-
		-	-	-	-	-	-	-	-	-	-	-	-	168001	-	-	-	-	-	-	-	-	-	-	-	-	-	-	-	-	-	-	-	-	-	-	-	-	-	-	-	-	-	-	-	-	-	-	-	-	-	-
		-	+	-	-	-	-	GB19676-PA	AT1G11950.1	-	-	-	156796	167987	261020	-	-	-	-	CG8165-PA	-	-	ENSGALP00000036346	-	NP_057688.2	-	214375	-	-	167409	-	Os03g31594.2	-	-	-	154856	-	-	274566	RO3G_02003.1	-	-	-	SINFRUP00000164664	-	-	-	-	32600	-	-	-
		-	+	-	-	-	-	-	AT1G76450.1	-	-	-	-	167973	-	-	-	-	-	-	-	-	-	-	-	-	-	-	-	-	-	Os08g40160.1	30596	-	-	175716	-	-	206841	-	-	-	-	-	-	-	-	-	-	-	-	-
		-	-	-	-	-	-	-	-	-	-	-	-	167963	-	-	-	-	-	-	-	-	-	-	-	-	-	-	-	-	-	-	-	-	-	-	-	-	-	-	-	-	-	-	-	-	-	-	-	-	-	-
		-	-	-	-	-	-	-	-	-	-	-	-	167955	-	-	-	-	-	-	-	-	-	-	-	-	-	-	-	-	-	-	-	-	-	-	-	-	-	-	-	-	-	-	-	-	-	-	-	-	-	-
		-	-	-	-	-	-	-	-	-	-	-	-	167940	-	-	-	-	-	-	-	-	-	-	-	-	-	-	-	-	-	-	-	-	-	-	-	-	-	-	-	-	-	-	-	-	-	-	-	-	-	-
		-	-	-	-	-	-	-	-	-	-	-	-	167939	-	-	-	-	-	-	-	-	-	-	-	-	-	-	-	-	-	-	-	-	-	-	-	-	-	-	-	-	-	-	-	-	-	-	-	-	-	-
		-	-	-	-	-	-	-	-	-	-	-	-	167938	-	-	-	-	-	-	-	-	-	-	-	-	-	-	-	-	-	-	-	-	-	-	-	-	-	-	-	-	-	-	-	-	-	-	-	-	-	-
CYP738A1	cytochrome P450, unkown function, in CYP85 clan	-	-	-	-	-	-	-	-	-	-	-	-	167934	-	-	-	-	-	-	-	-	ENSGALP00000010878	-	-	-	189041	-	-	-	-	-	-	-	-	-	-	-	-	-	-	-	-	-	-	-	-	-	-	-	-	-
	related to haem peroxidases	-	-	-	-	-	-	-	-	-	-	-	-	167932	-	-	-	-	-	-	-	-	-	-	-	-	-	-	-	-	-	-	-	-	-	-	-	-	-	-	-	-	-	-	-	-	-	-	-	-	-	-
		-	-	-	-	-	-	-	-	-	-	-	-	167923	-	-	-	-	-	-	-	-	-	-	-	-	-	-	-	-	-	-	-	-	-	-	-	-	-	-	-	-	-	-	-	-	-	-	-	-	-	-
		-	-	-	-	-	-	-	-	-	-	-	-	167922	-	-	-	-	-	-	-	-	-	-	-	-	-	-	-	-	-	-	-	-	-	-	-	-	-	-	-	-	-	-	-	-	-	-	-	-	-	-
		-	-	-	-	-	-	-	-	-	-	-	-	167916	-	-	-	-	-	-	-	-	-	-	-	-	-	-	-	-	-	-	-	-	-	-	-	-	-	-	-	-	-	-	-	-	-	-	-	-	-	-
		-	-	-	-	-	-	-	-	-	-	-	-	167909	-	-	-	-	-	-	-	-	-	-	-	-	-	-	-	-	-	-	-	-	-	-	-	-	-	-	-	-	-	-	-	-	-	-	-	-	-	-
		-	-	-	-	-	-	-	-	-	-	-	-	167904	-	-	-	-	-	-	-	-	-	-	-	-	-	-	-	-	-	-	-	-	-	-	-	-	-	-	-	-	-	-	-	-	-	-	-	-	-	-
STPK7	Serine/Threonine Protein Kinase Homolog 7, putative	-	+	+	-	-	-	GB13956-PA	AT2G32850.2	-	BDEG_06450	CE04586	20343	167901	270150	-	-	OTTDARP00000018326	DDB0206384	CG10637-PC	-	118.m00167	ENSGALP00000000025	GL50803_12223	XP_001127411.1	LmjF34.0030	174596	22473	34142	96644	NCU06202	Os09g10720.1	-	GSPATP00015448001	46154	166336	138119	PFL2280w	-	RO3G_13069.1	YBR059C	SPBC557.04	GLEAN3_24035	SINFRUP00000165589	122.m00100	4105	-	46.m01693	24131	85165.m00032	Tb09.160.4770	UM03081.1
	SET domain containing protein, putative histone methyltransferase	-	-	-	-	-	-	-	-	-	-	-	-	167893	-	-	-	-	-	-	-	-	-	-	-	-	-	-	-	-	-	-	4775	-	-	-	-	-	-	-	-	-	-	-	-	-	-	-	-	-	-	-
		-	-	-	-	-	-	-	-	-	-	-	-	167887	-	-	-	-	-	-	-	-	-	-	-	-	-	-	-	-	-	-	-	-	-	-	-	-	-	-	-	-	-	-	-	-	-	-	-	-	-	-
NIFS2	cysteine desulfurase	-	-	-	-	-	-	-	-	11983	-	-	-	167884	-	-	-	-	-	-	-	-	-	-	-	-	-	-	-	-	-	-	28825	-	-	-	-	-	-	-	-	-	-	-	-	-	-	-	-	-	-	-
		-	+	-	-	-	-	-	AT5G58110.1	-	-	-	-	167882	-	-	-	-	-	-	-	-	-	-	-	-	-	-	-	-	-	Os06g49030.1	34437	GSPATP00031134001	42981	122720	132700	-	274192	-	-	-	-	-	-	-	-	-	-	-	-	-
		-	-	-	-	-	-	-	-	-	-	-	-	167880	-	-	-	-	-	-	-	-	-	-	-	-	-	-	-	-	-	Os04g24460.1	-	-	-	-	-	-	-	-	-	-	-	-	-	-	-	-	-	-	-	-
		-	+	+	-	-	-	GB14690-PA	AT2G03667.1	8081	BDEG_01484	CE06195	43016	167865	271398	cgd3_110	CMB009C	OTTDARP00000001540	DDB0204494	CG17486-PA	19068803	11.m00365	ENSGALP00000003773	-	NP_061921.1	LmjF29.1490	181114	16593	64759	240001	NCU07300	Os12g38630.1	31108	GSPATP00008685001	49902	167036	-	-	769925	RO3G_11171.1	YML096W	SPBC4F6.11c	-	SINFRUP00000173234	65.m00171	1882	TA20880	-	50459	96713.m00123	Tb927.3.4060	UM03499.1
		-	+	-	-	-	-	GB18896-PA	AT5G54570.1	-	-	CE04248	-	167861	288776	-	-	-	-	CG9701-PA	-	-	ENSGALP00000020127	-	OTTHUMP00000162531	-	131754	-	68368	-	NCU00130	Os10g17650.1	-	-	16856	117888	108505	-	574973	-	-	-	GLEAN3_08633	SINFRUP00000160250	-	28413	-	-	-	82184.m00121	-	-
		-	-	-	-	-	-	-	-	-	-	-	-	167851	-	-	-	-	-	-	-	-	-	-	-	-	-	-	-	-	-	-	-	-	-	-	-	-	-	-	-	-	-	-	-	-	-	-	-	-	-	-
		-	-	-	-	-	-	-	-	-	-	-	-	-	-	-	-	-	-	-	-	-	-	-	-	-	-	-	-	-	-	-	-	-	-	-	-	-	-	-	-	-	-	-	-	-	-	-	-	-	-	-
		-	-	-	-	-	-	-	-	-	-	-	-	167849	-	-	-	-	-	-	-	-	-	-	-	-	-	-	-	-	-	-	36256	-	-	118582	-	-	-	-	-	-	-	-	-	-	-	-	-	-	-	-
		-	-	-	-	-	-	-	-	-	-	-	-	167842	-	-	-	-	-	-	-	-	-	-	-	-	-	-	-	-	-	-	-	-	-	-	-	-	-	-	-	-	-	-	-	-	-	-	-	-	-	-
		-	+	-	-	-	-	-	-	-	-	-	-	167836	-	cgd2_110	-	-	-	-	-	-	-	-	-	-	238800	-	-	-	-	Os02g55260.2	-	-	-	141399	-	-	195550	-	-	-	-	-	-	-	-	27.m00856	-	-	-	-
		-	-	-	-	-	-	-	-	-	-	-	-	167834	-	-	-	-	-	-	-	-	-	-	-	-	-	-	-	-	-	-	-	-	-	-	-	-	-	-	-	-	-	-	-	-	-	-	-	-	-	-
DNJ2	DnaJ-like protein 2, rather cytosolic	-	-	-	-	-	-	-	-	-	-	CE01057	-	167824	-	-	-	-	-	-	-	-	-	-	-	-	-	-	-	-	-	-	-	-	-	-	-	-	-	-	-	-	-	-	-	-	-	-	-	-	-	-
HDMA3401	amine oxidase family protein	-	-	-	-	-	-	-	-	-	-	-	-	167818	-	-	-	-	-	-	-	-	-	-	-	-	-	-	-	-	-	-	-	-	-	-	-	-	-	-	-	-	-	-	-	-	-	-	-	-	-	-
		-	-	-	-	-	-	-	-	-	-	-	-	167816	-	-	-	-	-	-	-	-	-	-	-	-	-	-	-	-	-	-	-	-	-	-	-	-	-	-	-	-	-	-	-	-	-	-	-	-	-	-
		-	-	-	-	-	-	-	-	-	-	-	-	167804	-	-	-	-	-	-	-	-	-	-	-	-	-	-	-	-	-	-	-	-	-	-	-	-	-	-	-	-	-	-	-	-	-	-	-	-	-	-
		-	-	-	-	-	-	-	-	-	-	-	-	167803	-	-	-	-	-	-	-	-	-	-	-	-	-	-	-	-	-	-	-	-	-	-	-	-	-	-	-	-	-	-	-	-	-	-	-	-	-	-
		-	-	-	-	-	-	-	-	-	-	-	-	167801	-	-	-	-	-	-	-	-	-	-	-	-	-	-	-	-	-	-	-	-	-	-	-	-	-	-	-	-	-	-	-	-	-	-	-	-	-	-
		-	-	-	-	-	-	-	-	-	-	-	-	167797	-	-	-	-	-	-	-	-	-	-	-	-	-	-	-	-	-	-	-	-	-	-	-	-	-	-	-	-	-	-	-	-	-	-	-	-	-	-
		-	-	-	-	-	-	-	-	-	-	-	-	167787	-	-	-	-	-	-	-	-	-	-	-	-	-	-	-	-	-	-	-	-	-	-	-	-	-	-	-	-	-	-	-	-	-	-	-	-	-	-
		-	-	-	-	-	-	-	-	-	-	-	-	167786	-	-	-	-	-	-	-	-	-	-	-	-	-	-	-	-	-	-	-	-	-	-	-	-	-	-	-	-	-	-	-	-	-	-	-	-	-	-
		-	-	-	-	-	-	-	-	-	-	-	-	167780	-	-	-	-	-	-	-	-	-	-	-	-	-	-	-	-	-	-	-	-	-	-	-	-	-	-	-	-	-	-	-	-	-	-	-	-	-	-
		-	-	-	-	-	-	-	-	-	-	-	-	167776	-	-	-	-	-	-	-	-	-	-	-	-	-	-	-	-	-	-	-	-	-	-	-	-	-	-	-	-	-	-	-	-	-	-	-	-	-	-
		-	-	-	-	-	-	-	-	-	-	-	-	167758	-	-	-	-	-	-	-	-	-	-	-	-	-	-	-	-	-	-	-	-	-	-	-	-	-	-	-	-	-	-	-	-	-	-	-	-	-	-
		-	-	-	-	-	-	-	-	-	-	-	-	167757	-	-	-	-	-	-	-	-	-	-	-	-	-	-	-	-	-	-	-	-	-	-	133752	-	-	-	-	-	-	-	-	-	-	-	-	-	-	-
		-	-	-	-	-	-	-	-	-	-	-	-	167755	-	-	-	-	-	-	-	-	-	-	-	-	-	-	-	-	-	-	-	-	-	-	-	-	-	-	-	-	-	-	-	-	-	-	-	-	-	-
		-	+	-	-	-	-	-	AT3G57180.1	-	BDEG_01333	CE28989	19738	167754	-	-	CME141C	-	-	-	-	-	-	-	OTTHUMP00000158975	-	124576	-	77993	21779	-	Os09g19980.1	17927	-	40200	21847	-	PF14_0564	416546	RO3G_02308.1	-	-	-	-	27.m00311	3583	TA19855	55.m04867	60700	-	Tb927.6.2350	-
		-	-	-	-	-	-	-	-	-	-	-	-	167745	-	-	-	-	-	-	-	-	-	-	-	-	-	-	-	-	-	-	-	-	-	-	-	-	-	-	-	-	-	-	-	-	-	-	-	-	-	-
		-	+	-	-	-	-	-	AT5G44520.1	-	-	-	-	167738	-	-	-	-	-	-	-	-	-	-	-	-	-	-	-	-	-	Os03g56869.1	37581	-	-	203525	-	-	772185	-	-	-	-	-	-	-	-	-	-	-	-	-
		-	-	-	-	-	-	-	-	-	-	-	-	167735	-	-	-	-	-	-	-	-	-	-	-	-	-	-	-	-	-	-	-	-	-	-	-	-	-	-	-	-	-	-	-	-	-	-	-	-	-	-
		-	-	-	-	-	-	-	-	-	-	-	-	167734	-	-	-	-	-	-	-	-	-	-	-	-	-	-	-	-	-	-	-	-	-	-	-	-	-	-	-	-	-	-	-	-	-	-	-	-	-	-
		-	-	-	-	-	-	-	-	-	-	-	-	167729	-	-	-	-	-	-	-	-	-	-	-	-	-	-	3999	-	-	-	-	-	-	-	-	-	-	-	-	-	-	-	-	-	-	-	-	-	-	-
	Hypothetical protein	+	+	-	-	-	-	GB18656-PA	-	-	BDEG_03119	-	225055	167727	270127	-	-	-	-	-	-	-	-	-	-	-	108617	-	57344	209659	-	-	-	GSPATP00037575001	-	-	141155	-	-	-	-	-	-	SINFRUP00000146984	57.m00244	-	-	-	55170	-	-	-
		-	+	-	-	-	-	-	-	-	-	-	-	167716	-	-	-	-	DDB0183941	-	-	-	-	-	OTTHUMP00000181362	-	-	-	-	87071	-	-	-	-	-	-	-	-	-	-	-	SPCC188.13c	GLEAN3_14310	SINFRUP00000153317	277.m00034	-	-	-	-	-	Tb927.7.2970	-
		-	-	-	-	-	-	-	-	-	-	-	-	167712	-	-	-	-	-	-	-	-	-	-	-	-	-	-	-	-	-	-	-	-	-	-	-	-	-	-	-	-	-	-	-	-	-	-	-	-	-	-
		-	-	-	-	-	-	-	-	-	-	-	-	167707	-	-	-	-	-	-	-	-	-	-	-	-	-	-	-	-	-	-	-	-	-	-	-	-	-	-	-	-	-	-	-	-	-	-	-	-	-	-
		-	+	-	-	-	-	GB12989-PA	AT4G05440.1	16812	BDEG_04405	-	169051	167698	263564	cgd6_1830	CMB007C	-	DDB0169525	-	-	-	ENSGALP00000022692	-	OTTHUMP00000019106	-	228703	27954	30015	233898	NCU06497	-	32313	GSPATP00017041001	11084	146294	138241	-	258279	RO3G_10812.1	YLR215C	SPAP27G11.03	GLEAN3_17645	-	4.m00507	2157	TA10770	-	55290	93605.m00049	-	UM04862.1
		-	-	-	-	-	-	-	-	-	-	-	-	167697	-	-	-	-	-	-	-	-	-	-	-	-	-	-	-	-	-	-	-	-	-	-	-	-	-	-	-	-	-	-	-	-	-	-	-	-	-	-
		-	-	-	-	-	-	-	-	-	-	-	-	167694	-	-	-	-	-	-	-	-	ENSGALP00000036721	-	OTTHUMP00000017321	-	-	-	-	-	-	-	-	-	-	-	-	MAL8P1.205	-	-	-	-	-	-	-	-	-	641.m01476	-	-	-	-
		-	+	-	-	-	-	-	AT3G28760.2	24264	-	-	-	167688	-	-	CMF179C	-	-	-	-	-	-	-	-	-	-	-	-	-	-	Os03g07420.1	3964	-	-	213398	-	-	259008	-	-	-	-	-	-	-	-	-	-	-	-	-
		-	+	-	-	-	-	-	AT3G55760.2	-	-	-	-	167685	-	-	-	-	-	-	-	-	-	-	-	-	-	-	-	-	-	Os11g37560.2	21090	-	-	40110	-	-	419087	-	-	-	-	-	-	-	-	20.m03972	-	-	-	-
		-	-	-	-	-	-	-	-	-	-	-	-	167682	-	-	-	-	-	-	-	-	-	-	-	-	-	-	-	-	-	-	-	-	-	-	-	-	-	-	-	-	-	-	-	-	-	-	-	-	-	-
		-	-	-	-	-	-	-	-	-	-	-	-	167680	-	-	-	-	-	-	-	-	-	-	-	-	-	-	-	-	-	-	-	-	-	-	-	-	-	-	-	-	-	-	-	-	-	-	-	-	-	-
		-	+	-	-	-	-	GB12047-PA	AT5G41210.1	-	-	-	173236	167675	283120	-	-	-	DDB0187641	CG30000-PA	-	-	ENSGALP00000010240	-	OTTHUMP00000063803	-	207042	-	-	-	-	Os11g37730.1	-	GSPATP00014815001	-	222347	156296	-	180407	-	-	-	-	SINFRUP00000140331	13.m00496	-	-	-	59204	-	-	-
		-	-	-	-	-	-	-	AT3G59780.1	-	-	-	-	167673	-	-	-	-	-	-	-	-	-	-	-	-	-	-	-	-	-	Os01g67120.1	-	-	-	164062	-	-	828175	-	-	-	-	-	-	-	-	-	-	-	-	-
		-	-	-	-	-	-	-	-	-	-	-	-	167663	-	-	-	-	-	-	-	-	-	-	-	-	-	-	-	-	-	-	-	-	-	-	-	-	-	-	-	-	-	-	-	-	-	-	-	-	-	-
		-	-	-	-	-	-	-	-	-	-	-	-	167662	-	-	-	-	-	-	-	-	-	-	-	-	-	-	-	-	-	-	-	-	-	-	-	-	-	-	-	-	-	-	-	-	-	-	-	-	-	-
		-	+	-	-	-	-	-	-	-	-	-	-	167658	-	-	CMH009C	-	-	-	-	-	-	-	-	LmjF35.2160	-	-	-	-	-	-	-	-	-	-	-	-	792460	-	YNL141W	SPBC1683.02	-	-	-	-	-	-	-	-	-	-
		-	-	-	-	-	-	-	-	-	-	-	-	167657	-	-	-	-	-	-	-	-	-	-	-	-	-	-	-	-	-	-	-	-	-	-	-	-	-	-	-	-	-	-	-	-	-	-	-	-	-	-
		-	-	-	-	-	-	-	-	-	-	-	-	167654	-	-	-	-	-	-	-	-	-	-	-	-	-	-	-	-	-	Os02g07200.3	-	-	-	-	-	-	-	-	-	-	-	-	-	-	-	-	-	-	-	-
		-	+	-	-	-	-	-	AT5G67170.2	-	BDEG_04792	CE40597	108175	167653	380668	cgd6_2750	CMT549C	OTTDARP00000023003	DDB0189630	CG6091-PB	-	-	ENSGALP00000022882	-	OTTHUMP00000002782	-	163820	10797	81362	126147	-	Os04g57480.1	34900	GSPATP00034792001	41252	165219	138470	PF10_0308	760490	RO3G_12574.1	-	-	-	SINFRUP00000130514	72.m00158	264466	-	44.m02558	51431	-	-	UM02372.1
		-	-	-	-	-	-	-	-	-	-	-	-	167640	-	-	-	-	-	-	-	-	-	-	-	-	-	-	-	-	-	Os11g14460.1	-	-	-	-	-	-	-	-	-	-	-	-	-	-	-	-	-	-	-	-
		-	-	-	-	-	-	-	-	-	-	-	-	167639	-	-	-	-	-	-	-	-	-	-	-	-	-	-	-	-	-	-	-	-	-	-	-	-	-	-	-	-	-	-	-	-	-	-	-	-	-	-
		-	-	-	-	-	-	-	-	-	-	-	-	167638	-	-	-	-	-	-	-	-	-	-	-	-	-	-	-	-	-	-	-	-	-	-	-	-	-	-	-	-	-	-	-	-	-	-	-	-	-	-
		-	-	-	-	-	-	-	-	-	-	-	-	167625	-	-	-	-	-	-	-	-	-	-	-	-	-	-	-	-	-	-	-	-	-	-	-	-	-	-	-	-	-	-	-	-	-	-	-	-	-	-
		-	-	-	-	-	-	-	-	-	-	-	-	167623	-	-	-	-	-	-	-	-	-	-	-	-	-	-	-	-	-	-	-	-	-	-	-	-	-	-	-	-	-	-	-	-	-	-	-	-	-	-
		-	-	-	-	-	-	-	-	-	-	-	-	167618	-	-	-	-	-	-	-	-	-	-	-	-	-	-	-	-	-	-	-	-	-	-	-	-	-	-	-	-	-	-	-	-	-	-	-	-	-	-
		-	-	-	-	-	-	-	-	-	-	-	-	167615	-	-	-	-	-	-	-	-	-	-	-	-	-	-	-	-	-	-	-	-	-	-	-	-	-	-	-	-	-	-	-	-	-	-	-	-	-	-
		-	-	-	-	-	-	-	-	-	-	CE30526	208874	167614	-	-	-	-	-	-	-	-	-	-	-	-	-	-	-	-	-	-	-	-	-	160875	-	-	-	-	-	-	-	-	-	-	-	-	-	-	-	-
		-	-	-	-	-	-	-	-	-	-	-	-	167612	-	-	-	-	-	-	-	-	-	-	-	-	-	-	-	-	-	-	-	-	-	-	-	-	-	-	-	-	-	-	-	-	-	-	-	-	-	-
		-	-	-	-	-	-	-	-	-	-	-	-	167609	-	-	-	-	-	-	-	-	-	-	-	-	-	-	-	-	-	-	-	-	-	-	-	-	-	-	-	-	-	-	-	-	-	-	-	-	-	-
		-	-	-	-	-	-	-	-	-	-	-	-	167608	-	-	-	-	-	-	-	-	-	-	-	-	-	-	-	-	-	-	-	-	-	-	-	-	-	-	-	-	-	-	-	-	-	-	-	-	-	-
		-	-	-	-	-	-	-	-	-	-	-	-	167604	-	-	-	-	-	-	-	-	-	-	-	-	-	-	-	-	-	-	-	-	-	-	-	-	-	-	-	-	-	-	-	-	-	-	-	-	-	-
		-	-	-	-	-	-	-	-	-	-	-	-	167601	-	-	-	-	-	-	-	-	-	-	-	-	-	-	-	-	-	-	-	-	-	-	-	-	-	-	-	-	-	-	-	-	-	-	-	-	-	-
		-	-	-	-	-	-	-	-	-	-	-	-	167598	-	-	-	-	-	-	-	-	-	-	-	-	-	-	-	-	-	-	-	-	-	-	-	-	-	-	-	-	-	-	-	-	-	-	-	-	-	-
		-	+	-	-	-	-	-	-	-	-	-	-	167597	-	-	-	-	-	-	-	-	-	-	-	-	-	-	-	-	-	-	-	-	3676	151296	-	-	820162	-	YJL093C	-	-	-	-	-	-	-	-	-	-	UM05925.1
		-	-	-	-	-	-	-	-	-	-	-	-	167596	-	-	-	-	-	-	-	-	-	-	-	-	-	-	-	-	-	-	-	-	-	-	-	-	-	-	-	-	-	-	-	-	-	-	-	-	-	-
		-	-	-	-	-	-	-	-	-	-	-	-	167593	-	-	-	-	-	-	-	-	-	-	-	-	-	-	-	-	-	-	-	-	-	-	-	-	-	-	-	-	-	-	-	-	-	-	-	-	-	-
		-	-	-	-	-	-	-	AT2G25660.1	-	-	-	-	167592	-	-	-	-	-	-	-	-	-	-	-	-	-	-	-	-	-	Os01g08420.1	17814	-	-	196483	-	-	-	-	-	-	-	-	-	-	-	-	-	-	-	-
		-	-	-	-	-	-	-	-	-	-	-	-	167587	-	-	-	-	-	-	-	-	ENSGALP00000014242	-	OTTHUMP00000159964	-	-	-	-	-	-	-	-	-	-	161134	-	-	669168	-	-	-	-	-	-	-	-	-	-	-	-	-
		-	-	-	-	-	-	-	-	-	-	-	-	167579	-	-	-	-	-	-	-	-	-	-	-	-	-	-	-	-	-	-	-	-	-	-	-	-	-	-	-	-	-	-	-	-	-	-	-	-	-	-
		-	-	-	-	-	-	-	-	-	-	-	-	167573	-	-	-	-	-	-	-	-	-	-	-	-	-	-	-	-	-	-	-	-	-	-	-	-	-	-	-	-	-	-	-	-	-	-	-	-	-	-
		-	+	-	-	-	-	GB15395-PA	-	-	BDEG_02311	-	-	167571	-	-	-	-	-	-	-	-	-	-	OTTHUMP00000081180	-	103735	-	80097	248432	-	-	-	GSPATP00017088001	-	-	-	-	-	-	-	-	-	SINFRUP00000159507	-	-	-	-	-	-	Tb11.01.0580	-
		-	-	-	-	-	-	-	-	-	-	-	-	167570	-	-	-	-	-	-	-	-	-	-	-	-	-	-	-	-	-	-	-	-	-	-	-	-	-	-	-	-	-	-	-	-	-	-	-	-	-	-
		-	+	-	-	-	-	GB19295-PA	AT4G14570.1	5744	BDEG_06521	CE29985	154305	167562	296060	cgd5_4370	CMT228C	OTTDARP00000023345	DDB0184409	-	-	33.m00237	ENSGALP00000004388	-	OTTHUMP00000171167	-	201138	10456	74773	167135	-	Os10g28020.3	-	GSPATP00018893001	13777	30590	141725	PFC0950c	657231	-	-	-	-	SINFRUP00000174050	52.m00197	262750	-	50.m03138	24080	82012.m00136	-	-
		-	-	-	-	-	-	-	-	-	-	-	-	167556	-	-	-	-	-	-	-	-	-	-	-	-	-	-	-	-	-	-	-	-	-	-	-	-	-	-	-	-	-	-	-	-	-	-	-	-	-	-
		-	-	-	-	-	-	-	-	-	-	-	-	167555	-	-	-	-	-	-	-	-	-	-	-	-	-	-	-	-	-	-	-	-	-	-	-	-	-	-	-	-	-	-	-	-	-	-	-	-	-	-
		-	-	-	-	-	-	-	-	-	-	-	-	167554	-	-	-	-	-	-	-	-	-	-	-	-	-	-	-	-	-	-	-	-	-	-	-	-	-	-	-	-	-	-	-	-	-	-	-	-	-	-
		-	-	-	-	-	-	-	-	-	-	-	-	167528	-	-	-	-	-	-	-	-	-	-	-	-	-	-	-	-	-	-	-	-	-	-	-	-	-	-	-	-	-	-	-	-	-	-	-	-	-	-
		-	-	-	-	-	-	-	-	-	-	-	-	167510	-	-	-	-	-	-	-	-	-	-	-	-	-	-	-	-	-	-	-	-	-	-	-	-	-	-	-	-	-	-	-	-	-	-	-	-	-	-
		-	-	-	-	-	-	-	-	-	-	-	-	167508	-	cgd4_2570	-	-	-	-	-	-	-	-	OTTHUMP00000066387	-	-	-	-	-	-	Os07g22534.4	-	-	-	117083	-	-	-	-	-	-	-	-	-	-	-	-	-	-	-	-
		-	+	-	-	-	-	GB15134-PA	AT5G13480.1	19281	BDEG_03617	CE21113	149680	167492	271563	cgd7_4140	CMQ221C	-	-	CG1109-PB	19068701	77.m00151	ENSGALP00000003172	-	OTTHUMP00000162269	LmjF30.0410	125354	34049	80419	105436	NCU00037	Os01g72220.1	14942	-	29608	51117	131550	PFL1975c	415574	RO3G_07379.1	YNL317W	SPAC12G12.14c	-	SINFRUP00000161556	-	38469	TA16450	59.m03372	26233	84270.m00597	Tb927.6.1830	UM00218.1
		-	-	-	-	-	-	-	-	-	-	-	-	167487	-	-	-	-	-	-	-	-	-	-	-	-	-	-	-	-	-	-	-	-	-	-	-	-	-	-	-	-	-	-	-	-	-	-	-	-	-	-
	GTP-binding protein, putative	-	+	+	-	-	-	GB12295-PA	AT4G39520.1	29702	BDEG_04811	CE24022	152844	167482	205664	cgd3_3580	CMG124C	OTTDARP00000020033	DDB0188361	CG8340-PA	19170785	168.m00120	ENSGALP00000037584	GL50803_16200	OTTHUMP00000028883	LmjF36.2250	103560	31858	56319	208563	NCU00389	Os07g43470.1	15277	PTETP15100001001	51277	160797	108292	PFE1215c	718912	RO3G_15244.1	YAL036C	SPAC9.07c	GLEAN3_09555	SINFRUP00000173400	235.m00035	12088	TA21445	49.m00057	38057	91481.m00047	Tb10.6k15.3970	UM03007.1
		-	-	-	-	-	-	-	-	-	-	-	-	167473	-	-	-	-	-	-	-	-	-	-	-	-	-	-	-	-	-	-	-	-	-	-	-	-	-	-	-	-	-	-	-	-	-	-	-	-	-	-
		-	-	-	-	-	-	-	-	-	-	-	-	167472	-	-	-	-	-	-	-	-	-	-	-	-	-	-	-	-	-	-	-	-	-	-	-	-	-	-	-	-	-	-	-	-	-	-	-	-	-	-
		-	-	-	-	-	-	-	-	-	-	-	-	167439	-	-	-	-	-	-	-	-	-	-	-	-	-	-	-	-	-	-	-	-	-	-	-	-	-	-	-	-	-	-	-	-	-	-	-	-	-	-
		-	-	-	-	-	-	-	-	-	-	-	-	167410	-	-	-	-	-	-	-	-	-	-	-	-	-	-	-	-	-	-	-	-	-	-	-	-	-	-	-	-	-	-	-	-	-	-	-	-	-	-
		-	-	-	-	-	-	-	-	-	-	-	-	167408	-	-	-	-	-	-	-	-	-	-	-	-	-	-	-	-	-	-	-	-	-	-	-	-	-	-	-	-	-	-	-	-	-	-	-	-	-	-
		-	-	-	-	-	-	-	-	-	-	-	-	167400	-	-	-	-	-	-	-	-	-	-	-	-	-	-	-	-	-	-	-	-	-	-	-	-	-	-	-	-	-	-	-	-	-	-	-	-	-	-
		-	-	-	-	-	-	-	-	-	-	-	-	167391	-	-	-	-	-	-	-	-	-	-	-	-	-	-	-	-	-	-	-	-	-	-	-	-	-	-	-	-	-	-	-	-	-	-	-	-	-	-
		-	-	-	-	-	-	-	-	-	-	-	-	167388	-	-	-	-	-	-	-	-	-	-	-	-	-	-	-	-	-	-	-	-	-	-	-	-	-	-	-	-	-	-	-	-	-	-	-	-	-	-
		-	+	-	+	-	-	-	-	-	-	-	-	167376	-	-	-	OTTDARP00000012868	-	-	-	-	-	-	-	LmjF34.0690	-	-	-	-	-	-	-	-	-	-	-	PF10_0356	-	RO3G_04331.1	-	-	-	-	-	-	-	-	-	-	-	UM02603.1
		-	-	-	-	-	-	-	-	-	-	-	-	167370	-	cgd6_2980	-	-	-	-	-	-	-	-	-	-	-	-	-	-	-	Os02g37940.2	-	GSPATP00031417001	-	-	155722	-	-	-	-	-	-	-	-	-	-	-	-	-	-	-
		-	-	-	-	-	-	-	-	-	-	-	-	167365	-	-	-	-	-	-	-	-	-	-	-	-	-	-	-	-	-	-	-	-	-	-	-	-	-	-	-	-	-	-	-	-	-	-	-	-	-	-
		-	-	-	-	-	-	-	-	-	-	-	-	167359	-	-	-	-	-	-	-	-	-	-	-	-	-	-	-	-	-	-	-	-	-	-	-	-	-	-	-	-	-	-	-	-	-	-	-	-	-	-
		-	-	-	-	-	-	-	-	-	-	-	-	167355	-	-	-	-	-	-	-	-	-	-	-	-	-	-	-	-	-	-	-	-	-	-	-	-	-	-	-	-	-	-	-	-	-	-	-	-	-	-
		-	-	-	-	-	-	-	-	-	-	-	-	167352	-	-	-	-	-	-	-	-	-	-	-	-	-	-	-	-	-	-	-	-	-	-	-	-	-	-	-	-	-	-	-	-	-	-	-	-	-	-
		-	-	-	-	-	-	-	-	-	-	-	-	167348	-	-	-	-	-	-	-	-	-	-	-	-	-	-	-	-	-	-	-	-	-	-	-	-	-	-	-	-	-	-	-	-	-	-	-	-	-	-
CYG2	putative guanylate cyclase;	-	-	-	-	-	-	-	-	-	-	-	-	167344	-	-	-	-	-	-	-	-	-	-	-	-	-	-	-	-	-	-	-	-	-	-	-	-	-	-	-	-	-	-	-	-	-	-	-	-	-	-
		-	-	-	-	-	-	-	-	-	-	-	-	167339	-	-	-	-	-	-	-	-	-	-	-	-	-	-	-	-	-	-	-	-	-	-	-	-	-	-	-	-	-	-	-	-	-	-	-	-	-	-
CYG1	putative guanylate cyclase;	-	-	-	-	-	-	-	-	-	-	-	-	167324	-	-	-	-	-	-	-	-	-	-	-	-	-	-	-	-	-	-	-	-	-	-	-	-	-	-	-	-	-	-	-	-	-	-	-	-	-	-
		-	-	-	-	-	-	-	-	-	-	-	-	167317	-	-	-	-	-	-	-	-	-	-	-	-	-	-	-	-	-	-	-	-	-	-	-	-	-	-	-	-	-	-	-	-	-	-	-	-	-	-
		-	-	-	-	-	-	-	-	63019	-	-	-	167306	-	-	-	-	-	-	-	-	-	-	-	-	-	-	-	-	-	-	-	-	-	-	-	-	-	-	-	-	-	-	-	4049	-	-	-	-	-	-
		-	-	-	-	-	-	-	-	-	-	-	-	167295	-	-	-	-	DDB0202566	-	-	-	-	-	-	-	-	-	-	-	-	-	-	-	-	-	-	-	-	-	-	-	-	-	7.m00446	-	-	-	-	-	-	UM06420.1
		-	+	-	-	-	-	GB10208-PA	-	-	BDEG_03776	CE34455	138805	167286	259846	-	-	-	-	CG2212-PA	19170750	-	ENSGALP00000039467	-	OTTHUMP00000066877	-	61439	16206	-	115565	NCU08276	-	31748	-	-	-	130240	-	-	RO3G_10193.1	YML059C	SPCC4B3.04c	-	SINFRUP00000135019	-	263945	-	-	10294	-	-	UM01230.1
		-	+	-	-	-	-	GB12984-PA	-	-	-	CE06460	83368	167272	-	cgd3_2160	-	-	-	CG9203-PA	-	415.m00039	ENSGALP00000017996	GL50803_115694	OTTHUMP00000036140	-	73580	12453	49599	183924	-	Os09g24210.2	-	GSPATP00033419001	-	-	118156	MAL13P1.191	-	-	-	-	-	SINFRUP00000130662	146.m00118	-	TA08700	50.m03093	30208	-	-	-
		-	-	-	-	-	-	-	-	-	-	-	-	167270	-	-	-	-	-	-	-	-	-	-	-	-	-	-	-	-	-	-	-	-	-	-	-	-	-	-	-	-	-	-	-	-	-	-	-	-	-	-
		-	-	-	-	-	-	-	-	-	-	-	-	-	-	-	-	-	-	-	-	-	-	-	-	-	-	-	-	-	-	-	-	-	-	-	-	-	-	-	-	-	-	-	-	-	-	-	-	-	-	-
PEX11		-	+	-	-	-	-	-	AT2G45740.3	-	-	-	175631	167266	-	-	-	-	-	-	-	-	-	-	-	-	-	-	45694	-	-	Os03g02590.4	37965	-	-	200510	-	-	552115	-	YOL147C	-	-	SINFRUP00000160910	-	-	-	-	63752	-	-	-
		-	+	-	-	-	-	-	AT3G04460.1	-	BDEG_00331	CE03163	149232	167259	-	-	CMO084C	OTTDARP00000019037	DDB0186545	-	-	-	ENSGALP00000003367	-	OTTHUMP00000163882	-	136681	-	31793	234615	NCU05245	Os10g32960.1	23866	-	-	105104	127732	-	571109	RO3G_16110.1	-	-	GLEAN3_22225	SINFRUP00000145935	-	-	-	-	60559	-	-	UM00297.1
		-	-	-	-	-	-	-	-	-	-	-	-	167258	-	-	-	-	-	-	-	-	-	-	-	-	-	-	-	-	-	-	-	-	-	-	-	-	-	-	-	-	-	-	-	-	-	-	-	-	-	-
		-	+	-	-	-	-	-	AT4G15570.1	-	BDEG_05286	-	-	167253	-	cgd1_830	CMP054C	-	DDB0167601	-	-	1.m00609	-	-	-	-	-	-	-	-	NCU02036	Os03g32526.1	28654	GSPATP00003038001	43802	168544	127237	-	778317	RO3G_01272.1	-	SPAC6G9.10c	-	-	15.m00401	264317	TA10355	641.m00190	-	-	-	UM02781.1
		-	-	-	-	-	-	-	-	-	-	-	-	167236	-	-	-	-	-	-	-	-	-	-	-	-	-	-	-	-	-	-	-	-	-	-	-	-	-	-	-	-	-	-	-	-	-	-	-	-	-	-
HTR13	Histone H3	-	-	-	-	-	-	-	-	-	-	-	-	-	-	-	-	-	-	-	-	-	-	-	-	-	-	-	-	-	-	-	-	-	-	-	-	-	-	-	-	-	-	-	-	-	-	-	-	-	-	-
HTA12	Histone H2A	-	-	-	-	-	-	-	-	-	-	-	-	-	-	-	-	-	-	-	-	-	-	-	-	-	-	-	-	-	-	-	-	-	-	-	-	-	-	-	-	-	-	-	-	-	-	-	-	-	-	-
		-	-	-	-	-	-	-	-	-	-	-	-	167211	-	-	-	-	-	-	-	-	-	-	-	-	-	-	-	-	-	-	-	-	-	-	-	-	-	-	-	-	-	-	-	-	-	-	-	-	-	-

		-	-	-	-	-	-	-	-	-	-	-	-	167210	-	-	-	-	-	-	-	-	-	-	-	-	-	-	-	-	-	-	-	-	-	-	-	-	-	-	-	-	-	-	-	-	-	-	-	-	-	-
		-	-	-	-	-	-	-	-	-	-	-	-	167200	-	-	-	-	-	-	-	-	-	-	-	-	-	-	-	-	-	-	-	-	-	-	-	-	-	-	-	-	-	-	-	-	-	-	-	-	-	-
		-	-	-	-	-	-	-	-	-	-	-	-	167199	-	-	-	-	-	-	-	-	-	-	-	-	-	-	-	-	-	-	-	-	-	-	-	-	-	-	-	-	-	-	-	-	-	-	-	-	-	-
HFO11	Histone H4	-	-	-	-	-	-	-	-	-	-	-	-	-	-	-	-	-	-	-	-	-	-	-	-	-	-	-	-	-	-	-	-	-	-	-	-	-	-	-	-	-	-	-	-	-	-	-	-	-	-	-
HTR11	Histone H3	-	-	-	-	-	-	-	-	-	-	-	-	-	-	-	-	-	-	-	-	-	-	-	-	-	-	-	-	-	-	-	-	-	-	-	-	-	-	-	-	-	-	-	-	-	-	-	-	-	-	-
HTA11	Histone H2A	-	-	-	-	-	-	-	-	-	-	-	-	-	-	-	-	-	-	-	-	-	-	-	-	-	-	-	-	-	-	-	-	-	-	-	-	-	-	-	-	-	-	-	-	-	-	-	-	-	-	-
		-	-	-	-	-	-	-	-	-	-	-	-	167188	-	-	-	-	-	-	-	-	-	-	-	-	-	-	-	-	-	-	-	-	-	-	-	-	-	-	-	-	-	-	-	-	-	-	-	-	-	-
		-	-	-	-	-	-	-	-	-	-	-	-	167172	-	-	-	-	-	-	-	-	-	-	-	-	-	-	-	-	-	-	-	-	-	-	-	-	-	-	-	-	-	-	-	-	-	-	-	-	-	-
HFO10	Histone H4	-	-	-	-	-	-	-	-	-	-	-	-	-	-	-	-	-	-	-	-	-	-	-	-	-	-	-	-	-	-	-	-	-	-	-	-	-	-	-	-	-	-	-	-	-	-	-	-	-	-	-
		-	-	-	-	-	-	-	-	-	-	-	-	167154	-	-	-	-	-	-	-	-	-	-	-	-	-	-	-	-	-	-	-	-	-	-	-	-	653315	-	-	-	-	-	-	-	-	-	-	-	-	-
		-	-	-	-	-	-	-	-	-	-	-	-	167153	-	-	-	-	-	-	-	-	-	-	-	-	-	-	-	-	-	-	-	-	-	-	-	-	-	-	-	-	-	-	-	-	-	-	-	-	-	-
		-	+	-	-	-	-	-	AT5G03760.1	-	-	-	-	167152	-	-	-	-	-	-	-	-	-	-	-	-	-	-	-	146403	-	Os09g39920.1	9161	-	-	183385	-	-	589559	-	-	-	-	-	-	-	-	-	-	-	-	-
		-	-	-	-	-	-	-	-	-	-	-	-	167148	-	-	-	-	-	-	-	-	-	-	-	-	-	-	-	-	-	-	-	-	-	-	-	-	-	-	-	-	-	-	-	-	-	-	-	-	-	-
		-	-	-	-	-	-	-	-	-	-	-	-	167136	-	-	-	-	-	-	-	-	-	-	-	-	-	-	-	-	-	-	-	-	-	-	-	-	-	-	-	-	-	-	-	-	-	-	-	-	-	-
		-	-	-	-	-	-	-	-	-	-	-	-	167134	-	-	-	-	-	-	-	-	-	-	-	-	-	-	-	-	-	-	-	-	-	-	-	-	-	-	-	-	-	-	-	-	-	-	-	-	-	-
		-	-	-	-	-	-	-	-	-	-	-	-	167132	-	-	-	-	-	-	-	-	-	-	-	-	-	-	-	-	-	-	-	-	-	-	-	-	-	-	-	-	-	-	-	-	-	-	-	-	-	-
		-	-	-	-	-	-	-	-	-	-	-	-	167128	-	-	-	-	-	-	-	-	-	-	-	-	-	-	-	-	-	-	-	-	-	-	-	-	-	-	-	-	-	-	-	-	-	-	-	-	-	-
		-	-	-	-	-	-	-	-	-	-	-	-	167112	-	-	-	-	-	-	-	-	-	-	-	-	-	-	-	-	-	-	-	-	-	-	-	-	-	-	-	-	-	-	-	-	-	-	-	-	-	-
FAP74	Conserved Uncharacterized Flagellar Associated Protein	+	+	-	+	-	-	-	-	71251	BDEG_02186	-	225037	167096	-	-	-	-	-	-	-	-	ENSGALP00000001965	GL50803_9283	XP_001128171.1	LmjF18.0570	123269	14595	-	30846	-	-	-	GSPATP00016562001	-	-	138665	-	-	-	-	-	-	SINFRUP00000152101	67.m00211	-	-	39.m00351	57490	94663.m00097	Tb10.05.0270	-
HTR8	Histone H3	-	-	-	-	-	-	-	-	-	-	-	-	-	-	-	-	-	-	-	-	-	-	-	-	-	-	-	-	-	-	-	-	-	-	-	-	-	-	-	-	-	-	-	-	-	-	-	-	-	-	-
		-	-	-	-	-	-	-	-	-	-	-	-	167090	-	-	-	-	-	-	-	-	-	-	-	-	-	-	-	-	-	-	-	-	-	-	-	-	-	-	-	-	-	-	-	-	-	-	-	-	-	-
		-	-	-	-	-	-	-	-	-	-	-	-	167089	-	-	-	-	-	-	-	-	-	-	-	-	-	-	-	-	-	-	-	-	-	-	-	-	-	-	-	-	-	-	-	-	-	-	-	-	-	-
		-	-	-	-	-	-	-	-	-	-	-	-	167085	-	-	-	-	-	-	-	-	-	-	-	-	-	-	-	-	-	-	-	-	-	-	-	-	-	-	-	-	-	-	-	-	-	-	-	-	-	-
		-	+	-	-	-	-	-	AT3G27530.1	-	BDEG_07883	-	-	167079	-	-	-	-	-	-	-	-	-	-	-	-	-	-	-	-	-	Os12g35360.1	-	-	-	199606	-	-	797875	-	-	-	-	-	-	-	-	-	-	-	-	-
		-	+	-	-	-	-	-	-	-	-	-	162540	167073	218852	-	-	-	-	-	-	-	ENSGALP00000017179	-	OTTHUMP00000010300	-	234262	-	-	163663	-	-	-	-	-	-	-	-	-	-	-	-	-	SINFRUP00000181535	-	-	-	-	-	-	-	-
		-	-	-	-	-	-	-	-	-	-	-	-	167072	-	-	-	-	-	-	-	-	-	-	-	-	-	-	-	-	-	-	-	-	-	-	-	-	-	-	-	-	-	-	-	-	-	-	-	-	-	-
		-	+	-	-	-	-	-	AT5G62840.1	60602	-	-	-	167070	-	-	CMR304C	-	-	-	-	-	-	-	-	-	-	-	-	-	-	Os01g47190.1	-	-	14334	142571	-	-	733291	-	-	-	-	-	-	20600	-	-	-	-	-	-
CLR24	Coiled-Like Repeat protein	-	-	-	-	-	-	-	-	-	-	-	-	167067	-	-	-	-	-	-	-	-	-	-	-	-	-	-	-	-	-	-	-	-	-	-	-	-	-	-	-	-	-	-	-	-	-	59.m06070	-	-	-	-
		-	-	-	-	-	-	-	-	-	-	-	-	167060	-	-	-	-	-	-	-	-	-	-	-	-	-	-	-	-	-	-	-	-	-	-	-	-	-	-	-	-	-	-	-	-	-	-	-	-	-	-
		-	+	+	-	-	-	GB18404-PA	AT3G56490.1	16970	-	CE05678	149440	167053	212076	cgd1_1600	-	OTTDARP00000023531	DDB0219437	CG2862-PB	-	25.m00272	ENSGALP00000000583	GL50803_4156	OTTHUMP00000021363	-	232738	23586	33528	231092	-	Os01g59750.1	34965	GSPATP00028811001	16130	58766	108169	PF08_0059	255039	-	YDL125C	-	-	SINFRUP00000156464	104.m00164	30979	TA19280	-	35755	-	Tb927.7.4480	UM06004.1
		-	+	+	+	-	-	GB16918-PA	AT5G26830.1	29703	BDEG_00391	CE33765	158346	167047	208953	cgd7_1710	CMS208C	OTTDARP00000019379	DDB0187861	CG5353-PC	19069220	85.m00160	ENSGALP00000006337	GL50803_13998	OTTHUMP00000194834	LmjF35.1410	103202	29593	39159	219591	NCU02380	Os08g19850.1	33761	GSPATP00017423001	18524	227167	129616	PF11_0270	420678	RO3G_06614.1	YIL078W	SPBC25H2.02	GLEAN3_20801	SINFRUP00000175918	16.m00405	22770	TA06605	145.m00604	50865	87280.m00076	Tb927.5.1090	UM04848.1
		-	+	-	-	-	-	-	AT5G18280.1	52656	BDEG_05186	CE18877	173954	167044	252381	-	-	OTTDARP00000009730	-	CG3059-PA	19069191	-	ENSGALP00000016566	-	OTTHUMP00000028127	LmjF15.0030	123541	2652	-	80717	NCU03713	Os11g25260.1	16918	GSPATP00021167001	-	175090	-	-	729947	RO3G_15652.1	YEL042W	SPAC824.08	GLEAN3_11727	SINFRUP00000158159	9.m00503	263386	-	-	61888	-	Tb927.8.3800	UM00548.1
EFG5	elongation factor-like protein	-	-	-	-	-	-	-	-	-	-	-	-	167037	-	-	-	-	-	-	-	-	-	-	-	-	-	26245	-	-	-	-	-	GSPATP00015933001	-	-	-	-	-	-	-	-	-	-	-	-	-	-	-	-	Tb927.4.2190	-
		-	-	-	-	-	-	-	-	-	-	-	-	167032	-	-	-	-	-	-	-	-	-	-	-	-	-	-	-	-	-	-	33937	-	-	-	-	-	-	-	-	-	-	-	-	-	-	-	-	-	-	-
HTR32	Histone H3	-	-	-	-	-	-	-	-	-	-	-	-	-	-	-	-	-	-	-	-	-	-	-	-	-	-	-	-	-	-	-	-	-	-	-	-	-	-	-	-	-	-	-	-	-	-	-	-	-	-	-
		-	+	-	-	-	-	-	AT5G44450.1	-	-	-	-	167028	-	-	-	-	DDB0190440	-	-	-	ENSGALP00000028252	-	OTTHUMP00000022340	LmjF30.0810	-	21077	-	-	-	Os03g56830.2	18137	GSPATP00030451001	-	175325	-	-	569328	-	-	-	-	-	29.m00257	-	-	20.m03670	-	-	Tb927.6.2330	-
		-	+	+	-	-	-	GB16012-PA	AT1G58230.1	22311	BDEG_03314	CE32621	180999	167021	220077	cgd4_3390	-	OTTDARP00000023358	DDB0185992	CG6775-PB	-	156.m00112	ENSGALP00000024862	GL50803_113143	OTTHUMP00000177700	LmjF36.1720	228503	38432	80471	161851	NCU00671	Os02g06720.1	17977	GSPATP00023765001	9886	173228	136443	PF11_0252	244133	RO3G_16111.1	YCR032W	SPBC28E12.06c	GLEAN3_10706	SINFRUP00000173090	217.m00054	269169	TA04385	55.m05025	52484	96396.m00067	Tb10.70.0690	UM03883.1
		-	+	-	-	-	-	-	AT3G07400.1	-	-	-	-	167017	-	-	-	-	-	-	-	-	-	-	-	LmjF13.0210	-	-	-	-	-	Os02g28040.1	30642	-	-	114654	-	-	645482	-	-	-	-	-	-	-	-	-	-	-	Tb10.61.0300	-
		-	+	+	-	-	-	GB16825-PA	AT5G11500.1	28204	BDEG_04089	CE22473	179529	167014	287775	-	CMJ167C	OTTDARP00000006123	DDB0190552	CG4593-PA	-	10.m00358	ENSGALP00000026722	GL50803_16794	OTTHUMP00000163035	LmjF30.2070	216779	26344	33927	170210	NCU06750	Os05g13440.1	18579	-	50753	107835	155937	-	814477	RO3G_14847.1	YMR132C	-	-	SINFRUP00000175519	118.m00087	8487	-	42.m00099	50705	95645.m00124	Tb927.6.3340	UM00470.1
		-	-	-	-	-	-	-	-	-	-	-	-	167010	-	-	-	-	-	-	-	-	-	-	-	-	-	-	-	-	-	-	-	-	-	-	-	-	-	-	-	-	-	-	-	-	-	-	-	-	-	-
HTR4	Histone H3	-	-	-	-	-	-	-	-	-	-	-	-	-	-	-	-	-	-	-	-	-	-	-	-	-	-	-	-	-	-	-	-	-	-	-	-	-	-	-	-	-	-	-	-	-	-	-	-	-	-	-
		-	-	-	-	-	-	-	-	-	-	-	-	166999	-	-	-	-	-	-	-	-	-	-	-	-	-	-	-	-	-	-	-	-	-	-	-	-	-	-	-	-	-	-	-	-	-	-	-	-	-	-
		-	-	-	-	-	-	-	-	-	-	-	-	166998	-	-	-	-	-	-	-	-	-	-	-	-	-	-	-	-	-	-	-	-	-	-	-	-	-	-	-	-	-	-	-	-	-	-	-	-	-	-
		-	-	-	-	-	-	-	-	-	-	-	-	166979	-	-	-	-	-	-	-	-	-	-	-	-	-	-	-	-	-	-	-	-	-	-	-	-	-	-	-	-	-	-	-	-	-	-	-	-	-	-
		-	-	-	-	-	-	-	-	-	-	-	-	166977	-	-	-	-	-	-	-	-	-	-	-	-	-	-	-	-	-	-	-	-	-	-	-	-	-	-	-	-	-	-	-	-	-	-	-	-	-	-
		-	-	-	-	-	-	-	-	-	-	-	-	166975	-	-	-	-	-	-	-	-	-	-	-	-	-	-	-	-	-	-	-	-	-	-	-	-	-	-	-	-	-	-	-	-	-	-	-	-	-	-
		-	-	-	-	-	-	-	-	-	-	-	-	166972	-	-	-	-	-	-	-	-	-	-	-	-	-	-	-	-	-	-	-	-	-	-	-	-	-	-	-	-	-	-	-	-	-	-	-	-	-	-
		-	-	-	-	-	-	-	-	-	-	-	-	166969	-	-	-	-	-	-	-	-	-	-	-	-	-	-	-	-	-	-	-	-	-	-	-	-	825711	-	-	-	-	-	-	-	-	-	-	-	-	-
		-	-	-	-	-	-	-	-	-	-	-	-	166963	-	-	-	-	-	-	-	-	-	-	-	-	-	-	-	-	-	-	-	-	-	-	-	-	-	-	-	-	-	-	-	-	-	-	-	-	-	-
		-	+	-	-	-	-	GB16332-PA	AT3G45590.2	-	-	-	222272	166960	265390	-	-	-	DDB0204993	-	-	-	ENSGALP00000007969	-	OTTHUMP00000160187	-	200224	-	-	108482	-	Os11g38930.1	-	-	-	125452	-	-	-	-	-	-	-	-	-	-	-	-	-	-	-	-
		-	-	-	-	-	-	-	-	-	-	-	-	166958	-	-	-	-	-	-	-	-	-	-	-	-	-	-	-	-	-	-	-	-	-	-	-	-	-	-	-	-	-	-	-	-	-	-	-	-	-	-
		-	-	-	-	-	-	-	-	-	-	-	-	166952	-	-	-	-	-	-	-	-	-	-	-	-	-	-	-	-	-	-	-	-	-	-	-	-	-	-	-	-	-	-	-	-	-	-	-	-	-	-
		-	-	-	-	-	-	-	-	-	-	-	-	166947	-	-	-	-	-	-	-	-	-	-	-	-	-	-	-	-	-	-	-	-	-	-	-	-	-	-	-	-	-	-	-	-	-	-	-	-	-	-
		-	+	-	-	-	-	-	AT2G21370.1	29217	-	-	-	166945	-	-	-	-	-	-	-	-	-	-	-	-	-	-	-	-	-	Os07g22650.1	3971	-	33450	-	-	-	201134	-	-	-	-	-	-	36494	-	-	-	-	-	-
		-	+	-	-	-	-	-	AT3G24800.1	-	-	-	214662	166944	-	-	-	-	-	-	-	-	ENSGALP00000014603	-	OTTHUMP00000011235	-	187172	-	-	-	-	Os02g40810.1	-	-	-	189221	-	-	409024	RO3G_11876.1	-	-	-	-	-	-	-	59.m00078	-	-	-	-
		-	+	-	-	-	-	GB11321-PA	-	27976	-	-	193609	166940	213947	-	-	-	-	CG3759-PA	-	-	-	-	-	-	231844	-	-	143408	NCU00526	-	-	-	-	-	137993	-	-	-	-	-	GLEAN3_24508	-	-	-	-	-	-	-	-	-
		-	-	-	-	-	-	-	-	-	-	-	-	166937	-	-	-	-	-	-	-	-	-	-	-	-	-	-	-	-	-	-	-	-	-	-	-	-	-	-	-	-	-	-	-	-	-	-	-	-	-	-
		-	-	-	-	-	-	-	AT1G68610.1	-	-	-	-	166936	-	-	-	-	-	-	-	-	-	-	-	-	-	-	-	-	-	-	-	-	-	-	-	-	822064	-	-	-	-	-	-	-	-	-	-	-	-	-
		-	-	-	-	-	-	-	-	-	-	-	-	166935	-	-	-	-	-	-	-	-	-	-	-	-	-	-	-	-	-	-	-	-	-	-	-	-	-	-	-	-	-	-	-	-	-	-	-	-	-	-
		-	-	-	-	-	-	-	-	-	-	-	-	166924	-	-	-	-	-	-	-	-	-	-	-	-	-	-	-	-	-	-	-	-	-	-	-	-	-	-	-	-	-	-	-	-	-	-	-	88481.m00079	-	-
		-	-	-	-	-	-	-	-	-	-	-	-	166922	-	-	-	-	-	-	-	-	-	-	-	-	-	-	-	-	-	-	-	-	-	-	-	-	-	-	-	-	-	-	-	-	-	-	-	-	-	-
		-	-	-	-	-	-	-	-	-	-	-	-	166918	-	-	-	-	-	-	-	-	-	-	-	-	-	-	-	-	-	-	-	-	-	-	-	-	-	-	-	-	-	-	-	-	-	-	-	-	-	-
		-	-	-	-	-	-	-	-	-	-	-	-	166903	-	-	-	-	-	-	-	-	-	-	-	-	-	-	-	-	-	-	-	-	-	-	-	-	-	-	-	-	-	-	-	-	-	-	-	-	-	-
		-	-	-	-	-	-	-	-	-	-	-	-	166902	-	-	-	-	-	-	-	-	-	-	-	-	-	-	-	-	-	-	-	-	-	-	-	-	-	-	-	-	-	-	-	-	-	-	-	-	-	-
		-	-	-	-	-	-	-	-	-	-	-	-	166895	-	-	-	-	-	-	-	-	-	-	-	-	-	-	-	-	-	-	-	-	-	-	-	-	-	-	-	-	-	-	-	-	-	-	-	-	-	-
		-	-	-	-	-	-	-	-	-	-	-	-	166894	-	-	-	-	-	-	-	-	-	-	-	-	-	-	-	-	-	-	-	-	-	-	-	-	-	-	-	-	-	-	-	-	-	-	-	-	-	-
		-	-	-	-	-	-	-	-	-	-	-	-	166892	-	-	-	-	-	-	-	-	-	-	-	-	-	-	-	-	-	-	-	-	-	-	-	-	-	-	-	-	-	-	-	-	-	-	-	-	-	-
		-	-	-	-	-	-	-	-	-	-	-	-	166891	-	-	-	-	-	-	-	-	-	-	-	-	-	-	-	-	-	-	-	-	-	-	-	-	-	-	-	-	-	-	-	-	-	-	-	-	-	-
		-	-	-	-	-	-	-	-	-	-	-	-	166890	-	-	-	-	-	-	-	-	-	-	-	-	-	-	-	-	-	-	-	-	-	-	-	-	-	-	-	-	-	-	-	-	-	-	-	-	-	-
		-	-	-	-	-	-	-	-	-	-	-	-	166885	-	-	-	-	-	-	-	-	-	-	-	-	234942	-	-	-	-	-	23384	-	46813	165131	-	-	-	-	-	-	-	-	-	-	-	-	-	-	-	-
		-	-	-	-	-	-	-	-	-	-	-	-	166883	-	-	-	-	-	-	-	-	-	-	-	-	-	-	-	-	-	-	-	-	-	-	-	-	-	-	-	-	-	-	-	-	-	-	-	-	-	-
		-	-	-	-	-	-	-	-	-	-	-	-	166874	-	-	-	-	-	-	-	-	-	-	-	-	-	-	-	-	-	-	-	-	-	-	-	-	-	-	-	-	-	-	-	-	-	-	-	-	-	-
		-	-	-	-	-	-	-	-	-	-	-	-	166860	-	-	-	-	-	-	-	-	-	-	-	-	-	-	-	-	-	-	-	-	-	-	-	-	-	-	-	-	-	-	-	-	-	-	-	-	-	-
		-	-	-	-	-	-	-	-	-	-	-	-	166850	-	-	-	-	-	-	-	-	-	-	-	-	-	-	-	-	-	-	-	-	-	-	-	-	-	-	-	-	-	-	-	-	-	-	-	-	-	-
		-	-	-	-	-	-	-	-	-	-	-	-	166843	-	-	-	-	-	-	-	-	-	-	-	-	-	-	-	-	-	-	-	-	-	-	-	-	-	-	-	-	-	-	-	-	-	-	-	-	-	-
		-	-	-	-	-	-	-	-	-	-	-	-	166839	-	-	-	-	-	-	-	-	-	-	-	-	-	-	-	-	-	-	-	-	-	-	-	-	-	-	-	-	-	-	-	-	-	-	-	-	-	-
		-	-	-	-	-	-	-	-	-	-	-	-	166834	-	-	-	-	-	-	-	-	-	-	-	-	-	-	-	-	-	-	-	-	-	-	-	-	-	-	-	-	-	-	-	-	-	-	-	-	-	-
		-	-	-	-	-	-	-	-	64516	-	-	65900	166823	-	-	-	-	-	-	-	-	-	-	-	-	-	-	-	-	-	-	-	-	-	-	-	-	-	-	-	-	-	-	-	-	-	-	-	-	-	-
		-	-	-	-	-	-	-	AT2G04790.2	-	-	-	-	166819	-	-	-	-	-	-	-	-	-	-	-	-	-	-	-	-	-	-	-	-	-	-	-	-	-	-	-	-	-	-	-	-	-	-	-	-	-	-
		-	-	-	-	-	-	-	-	-	-	-	-	166818	-	-	-	-	-	-	-	-	-	-	-	-	-	-	-	-	-	-	-	-	-	-	-	-	-	-	-	-	-	-	-	-	-	-	-	-	-	-
		-	-	-	-	-	-	-	-	-	-	-	-	166811	-	-	-	-	-	-	-	-	-	-	-	-	-	-	-	-	-	-	-	-	-	-	-	-	-	-	-	-	-	-	-	-	-	-	-	-	-	-
		-	-	-	-	-	-	-	-	-	-	-	-	166806	-	-	-	-	-	-	-	-	-	-	-	-	-	-	-	-	-	-	-	-	-	-	-	-	-	-	-	-	-	-	-	-	-	-	-	-	-	-
		-	-	-	-	-	-	-	-	-	-	-	-	166799	-	-	-	-	-	-	-	-	-	-	-	-	-	-	-	-	-	-	-	-	-	-	-	-	-	-	-	-	-	-	-	-	-	-	-	-	-	-
		-	-	-	-	-	-	-	AT5G24630.1	-	-	-	-	166797	-	-	-	-	-	-	-	-	-	-	-	-	-	-	-	-	-	Os02g05440.1	-	-	-	161308	-	-	-	-	-	-	-	-	-	-	-	-	-	-	-	-
		-	-	-	-	-	-	-	-	-	-	-	-	166780	-	-	-	-	-	-	-	-	-	-	-	-	-	-	-	-	-	-	-	-	-	-	-	-	-	-	-	-	-	-	-	-	-	-	-	-	-	-
		-	-	-	-	-	-	-	-	-	-	-	-	166776	-	-	-	-	-	-	-	-	-	-	-	-	-	-	-	-	-	-	-	-	-	-	-	-	-	-	-	-	-	-	-	-	-	-	-	-	-	UM02063.1
		-	-	-	-	-	-	-	-	-	-	-	-	166757	-	-	-	-	-	-	-	-	-	-	-	-	-	-	-	-	-	-	-	-	-	-	-	-	-	-	-	-	-	-	-	-	-	-	-	-	-	-
		-	-	-	-	-	-	-	-	-	-	-	-	166756	-	-	-	-	-	-	-	-	-	-	-	-	-	-	-	-	-	-	-	-	-	-	-	-	-	-	-	-	-	-	-	-	-	-	-	-	-	-
		-	-	-	-	-	-	-	-	-	-	-	-	166746	-	-	-	-	-	-	-	-	-	-	-	-	-	-	-	-	-	-	-	-	-	-	-	-	-	-	-	-	-	-	-	-	-	-	-	-	-	-
		-	-	-	-	-	-	-	AT5G09995.1	-	-	-	-	166730	-	-	-	-	-	-	-	-	-	-	-	-	-	-	-	-	-	-	32168	-	-	168860	-	-	217835	-	-	-	-	-	-	-	-	-	-	-	-	-
		-	-	-	-	-	-	-	-	-	-	-	-	166724	-	-	-	-	-	-	-	-	-	-	-	-	-	-	-	-	-	-	-	-	-	-	-	-	-	-	-	-	-	-	-	-	-	-	-	-	-	-
		-	-	-	-	-	-	-	-	-	-	-	-	166722	-	-	-	-	-	-	-	-	-	-	-	-	-	-	-	-	-	-	-	-	-	-	-	-	-	-	-	-	-	-	-	-	-	-	-	-	-	-
		-	-	-	-	-	-	-	-	-	-	-	-	166720	-	-	-	-	-	-	-	-	-	-	-	-	-	-	-	-	-	-	-	-	-	-	-	-	-	-	-	-	-	-	-	-	-	-	-	-	-	-
		-	-	-	-	-	-	-	-	-	-	-	-	166709	-	-	-	-	-	-	-	-	-	-	-	-	-	-	-	-	-	-	-	-	-	-	-	-	-	-	-	-	-	-	-	-	-	-	-	-	-	-
		-	+	-	-	-	-	GB10154-PA	AT1G20540.1	20923	BDEG_02632	CE24682	182296	166708	297037	-	-	-	DDB0167836	CG10646-PA	-	-	ENSGALP00000026380	-	OTTHUMP00000115381	LmjF15.0225	206565	32424	44525	185644	-	Os08g44010.5	-	GSPATP00014146001	8918	-	136592	PFL1040w	206647	RO3G_06051.1	-	-	-	SINFRUP00000180547	9.m00543	-	TA16775	-	27591	97294.m00127	Tb927.5.1670	-
		-	-	-	-	-	-	-	-	-	-	-	-	166702	-	-	-	-	-	-	-	-	-	-	-	-	-	-	-	-	-	-	-	-	-	-	-	-	-	-	-	-	-	-	-	-	-	-	-	-	-	-
		-	+	-	-	-	-	-	AT2G22650.1	-	-	-	-	166701	-	-	CMS211C	-	-	-	-	-	-	-	-	-	-	-	-	-	-	Os06g08300.2	36518	-	-	119132	-	-	578795	-	-	-	-	-	-	3863	-	-	-	-	-	-
		-	+	-	-	-	-	-	-	-	-	-	157649	166690	287519	-	-	OTTDARP00000010312	DDB0191604	CG2999-PB	-	-	-	-	-	LmjF31.0680	-	-	-	-	-	-	-	-	-	-	-	-	-	RO3G_12458.1	-	-	GLEAN3_07413	SINFRUP00000161204	-	-	-	-	-	-	-	UM06398.1
		-	+	-	-	-	-	-	AT2G25950.1	35373	BDEG_00558	CE25685	20036	166687	266051	cgd1_3660	CMS257C	-	DDB0204321	CG6153-PA	-	-	ENSGALP00000006338	-	OTTHUMP00000002997	LmjF12.0020	204391	13362	-	172171	NCU06751	Os01g37832.1	33362	GSPATP00014742001	14294	213747	129293	MAL13P1.159	835755	RO3G_15603.1	-	SPBP35G2.02	-	SINFRUP00000129918	147.m00083	7474	-	80.m03992	-	-	-	UM06176.1
		-	-	-	-	-	-	-	-	-	-	-	-	166686	-	-	-	-	-	-	-	-	-	-	-	-	-	-	-	-	-	-	-	-	-	-	-	-	-	-	-	-	-	-	-	-	-	-	-	-	-	-
		-	-	-	-	-	-	-	-	-	-	-	-	166682	-	-	-	-	-	-	-	-	-	-	-	-	-	-	-	-	-	-	-	-	-	-	-	-	-	-	-	-	-	-	-	-	-	-	-	-	-	-
		-	-	-	-	-	-	-	-	-	-	-	-	166670	-	-	-	-	-	-	-	-	-	-	-	-	-	-	-	-	-	-	-	-	-	-	-	-	-	-	-	-	-	-	-	-	-	-	-	-	-	-
		-	+	-	-	-	-	-	AT5G20150.1	-	-	-	-	166663	-	-	-	-	-	-	-	-	-	-	-	-	-	-	-	-	-	Os06g40120.1	6339	-	-	117077	-	-	416925	-	-	-	-	-	-	-	-	-	-	-	-	-
		-	-	-	-	-	-	-	-	-	-	-	-	166657	-	-	-	-	-	-	-	-	-	-	-	-	-	-	-	-	-	-	-	-	-	-	-	-	-	-	-	-	-	-	-	-	-	-	-	-	-	-
		-	-	-	-	-	-	-	-	-	-	-	-	166642	-	-	-	-	-	-	-	-	-	-	-	-	-	-	-	-	-	-	-	-	-	-	-	-	-	-	-	-	-	-	-	-	-	-	-	-	-	-
		-	-	-	-	-	-	-	-	-	-	-	-	166640	-	-	-	-	DDB0168739	-	-	-	-	-	-	-	-	-	80915	-	-	-	34532	-	-	-	-	-	-	-	-	-	-	-	-	-	-	-	-	-	-	-
		-	-	-	-	-	-	-	-	-	-	-	-	166632	-	-	-	-	-	-	-	-	-	-	-	-	-	-	-	-	-	-	-	-	-	-	-	-	-	-	-	-	-	-	-	-	-	-	-	-	-	-
		-	-	-	-	-	-	-	-	-	-	-	-	166621	-	-	-	-	-	-	-	-	-	-	-	-	-	-	-	-	-	-	-	-	-	-	-	-	-	-	-	-	-	-	-	-	-	-	-	-	-	-
		-	-	-	-	-	-	-	-	-	-	-	-	166618	-	-	-	-	-	-	-	-	-	-	-	-	-	-	-	-	-	-	-	-	-	-	-	-	-	-	-	-	-	-	-	-	-	-	-	-	-	-
		-	-	-	-	-	-	-	-	-	-	-	-	166616	-	-	-	-	-	-	-	-	-	-	-	-	-	-	-	-	-	-	13154	-	-	-	-	-	-	RO3G_02654.1	-	-	-	-	-	-	-	-	-	-	-	-
		-	-	-	-	-	-	-	-	61182	-	-	-	166609	-	-	-	-	-	-	-	-	-	-	-	-	-	-	-	-	-	-	-	-	-	-	-	-	-	-	-	-	-	-	-	-	-	-	-	-	-	-
PBGD2	Porphobilinogen deaminase, chloroplast precursor	-	-	-	-	-	-	-	-	-	-	-	-	166608	-	-	-	-	-	-	-	-	-	-	-	-	-	-	-	-	-	-	-	-	-	-	-	-	-	-	-	-	-	-	-	-	-	-	-	-	-	-
		-	-	-	-	-	-	-	-	-	-	-	-	166605	-	-	-	-	-	-	-	-	-	-	-	-	-	-	-	-	-	-	-	-	-	-	-	-	-	-	-	-	-	-	-	-	-	-	-	-	-	-
		-	-	-	-	-	-	-	-	-	-	-	-	166604	-	-	-	-	-	-	-	-	-	-	-	-	-	-	-	-	-	-	-	-	-	-	-	-	-	-	-	-	-	-	-	-	-	-	-	-	-	-
		-	+	-	-	-	-	GB18583-PA	-	1964	BDEG_07827	CE32269	229032	166574	295925	cgd8_3740	-	OTTDARP00000020728	-	CG30394-PB	-	254.m00077	ENSGALP00000015771	GL50803_17214	NP_001033073.1	LmjF35.5350	109866	24806	71189	83628	NCU01481	-	23171	GSPATP00038536001	49278	-	135776	-	-	RO3G_05710.1	YEL064C	SPBC1685.07c	GLEAN3_22627	SINFRUP00000164484	-	260998	-	42.m03476	53014	96713.m00135	Tb11.01.7500	UM02253.1
		-	-	-	-	-	-	-	-	-	-	-	-	166572	-	-	-	-	-	-	-	-	-	-	-	-	-	-	-	-	-	-	-	-	-	-	-	-	-	-	-	-	-	-	-	-	-	-	-	-	-	-
		-	-	-	-	-	-	-	-	-	-	-	-	166570	-	-	-	-	-	-	-	-	-	-	-	-	-	-	-	-	-	-	-	-	-	-	-	-	-	-	-	-	-	-	-	-	-	-	-	-	-	-
		-	+	-	-	-	-	-	-	60578	BDEG_01730	-	54872	166557	-	-	CMT636C	-	-	-	-	-	-	-	-	-	165932	31736	-	-	NCU00609	-	-	-	-	-	-	-	-	-	YGL038C	SPAC1006.05c	-	-	-	264481	-	-	-	-	-	-
		-	-	-	-	-	-	-	-	-	-	-	-	166556	-	-	-	-	-	-	-	-	-	-	-	-	-	-	-	-	-	-	-	-	-	-	-	-	-	-	-	-	-	-	-	-	-	-	-	-	-	-
GTG3401	Transcription initiation Spt4	-	+	+	-	-	-	GB16091-PA	AT5G08565.1	25503	BDEG_08134	CE19891	157886	166554	290196	cgd2_2820	CMJ060C	-	DDB0186435	CG12372-PA	19173491	1.m00717	ENSGALP00000001392	-	OTTHUMP00000182062	LmjF35.2390	237930	23363	70690	185016	NCU03838	Os07g43060.2	-	-	27082	232370	129991	PF10_0293	820153	RO3G_00728.1	YGR063C	SPBC21C3.16c	GLEAN3_27461	SINFRUP00000138917	-	32339	TA20345	55.m04923	28457	83938.m00047	-	-
		-	-	-	-	-	-	-	-	-	-	-	-	166553	-	-	-	-	-	-	-	-	-	-	-	-	-	-	-	-	-	-	-	-	-	-	-	-	-	-	-	-	-	-	-	-	-	-	-	-	-	-
		-	-	-	-	-	-	-	-	-	-	-	-	166543	-	-	-	-	-	-	-	-	-	-	-	-	-	-	-	-	-	-	-	-	-	-	-	-	-	-	-	-	-	-	-	-	-	-	-	-	-	-
		-	-	-	-	-	-	-	-	-	-	-	-	166539	-	-	-	-	-	-	-	-	-	-	-	-	-	-	-	-	-	Os04g54350.1	-	-	-	-	-	-	758599	-	-	-	GLEAN3_22265	-	-	-	-	-	-	-	-	-
		-	-	-	-	-	-	-	-	-	-	-	-	166537	-	-	-	-	-	-	-	-	-	-	-	-	-	-	-	-	-	-	-	-	-	-	-	-	-	-	-	-	-	-	-	-	-	-	-	-	-	-
		-	-	-	-	-	-	-	-	-	-	-	-	166530	-	-	-	-	-	-	-	-	-	-	-	-	-	-	-	-	-	-	-	-	-	-	-	-	-	-	-	-	-	-	-	-	-	-	-	-	-	-
		-	-	-	-	-	-	-	-	-	-	-	-	166524	-	-	-	-	-	-	-	-	-	-	-	-	-	-	-	-	-	-	-	-	-	-	-	-	-	-	-	-	-	-	-	-	-	-	-	-	-	-
		-	+	-	-	-	-	-	AT3G13050.1	-	-	CE32291	220893	166517	282860	-	-	OTTDARP00000016294	-	CG4324-PA	-	-	ENSGALP00000007956	-	NP_061181.1	-	131851	33459	65611	21316	-	Os07g38400.1	19850	-	45672	136367	-	-	215882	-	-	-	-	SINFRUP00000173795	392.m00008	-	-	-	28017	-	-	-
		-	-	-	-	-	-	-	AT2G46790.1	-	-	-	-	166515	-	-	-	-	-	-	-	-	-	-	-	-	-	-	-	-	-	Os11g05930.1	-	-	-	-	-	-	574629	-	-	-	-	-	-	-	-	-	-	-	-	-
		-	-	-	-	-	-	-	-	-	-	-	-	166509	-	-	-	-	-	-	-	-	-	-	-	-	-	-	-	-	-	-	-	-	-	-	-	-	-	-	-	-	-	-	-	-	-	-	-	-	-	-
		-	+	-	-	-	-	-	-	-	BDEG_03901	-	-	166502	-	-	-	-	-	-	-	-	-	-	-	-	-	-	-	-	NCU09895	-	-	-	-	-	-	-	-	RO3G_01997.1	-	SPBC26H8.11c	-	-	-	-	-	76.m01613	-	-	-	-
		-	-	-	-	-	-	-	-	-	-	-	-	166497	-	-	-	-	-	-	-	-	-	-	-	-	-	-	-	-	-	-	-	-	-	-	-	-	-	-	-	-	-	-	-	-	-	-	-	-	-	-
FAP136	Flagellar Associated Protein	-	-	-	+	-	-	-	-	-	-	-	-	166488	-	-	-	-	-	-	-	-	-	-	-	-	-	-	-	-	-	-	-	-	-	-	-	-	-	-	-	-	-	-	-	-	-	-	-	-	-	-
		-	-	-	-	-	-	-	-	-	-	-	-	166484	-	-	-	-	-	-	-	-	-	-	-	-	-	-	-	-	-	-	-	-	-	-	-	-	-	-	-	-	-	-	-	-	-	-	-	-	-	-
TPR1		-	+	-	-	-	-	-	AT5G21990.1	-	-	-	169118	166482	-	-	-	-	-	-	-	-	-	-	-	-	-	-	-	-	NCU06340	Os07g06710.1	35446	GSPATP00032698001	-	164982	-	-	293499	-	-	-	-	-	9.m00462	23596	TA13000	-	-	-	-	-
		-	+	-	-	-	-	-	AT5G60980.2	-	-	-	179128	166479	210029	-	-	OTTDARP00000023727	-	-	-	-	ENSGALP00000006604	-	OTTHUMP00000160588	-	110043	-	79095	-	NCU07574	Os02g29480.1	-	-	-	158945	140019	-	596417	RO3G_04007.1	-	SPBP8B7.11	GLEAN3_17827	SINFRUP00000174312	-	-	-	49.m05723	64182	-	-	-
		-	+	-	-	-	-	-	AT1G67960.1	53582	BDEG_06095	CE19819	5824	166451	261790	cgd4_1760	-	OTTDARP00000019768	DDB0217931	-	-	36.m00184	ENSGALP00000023336	-	NP_699196.2	-	82778	32336	78322	98970	NCU07125	Os03g50730.1	13825	-	34116	151908	141926	-	805090	RO3G_05448.1	YER140W	SPBC13G1.05	-	SINFRUP00000160373	-	22944	-	-	28365	-	-	UM03087.1
MAPKKKK	Mitogen Activated Protein Kinase Kinase Kinase Homolog B putative	-	-	-	-	-	-	-	-	-	-	-	-	166448	-	-	-	-	-	-	-	-	-	-	-	-	-	-	-	-	-	-	-	-	-	-	-	-	-	-	-	-	-	-	-	-	-	-	-	-	-	-
		-	-	-	-	-	-	-	-	-	-	-	-	166444	-	-	-	-	-	-	-	-	-	-	-	-	-	-	-	-	-	-	-	-	-	-	-	-	-	-	-	-	-	-	-	-	-	-	-	-	-	-
		+	+	-	-	-	-	-	-	-	BDEG_00371	-	125185	166442	-	-	-	-	-	-	-	-	ENSGALP00000031566	-	NP_001035247.1	-	110175	23873	-	108477	-	-	-	-	-	95518	157844	-	-	-	-	-	-	-	-	-	-	-	26095	-	-	-
		-	+	-	-	-	-	GB14260-PA	AT1G55870.1	-	BDEG_01803	-	222843	166441	271440	-	-	-	-	-	-	-	-	-	-	-	-	-	-	205373	-	-	-	-	-	163366	158783	-	-	RO3G_06685.1	-	-	-	-	-	-	-	-	-	-	-	-
		-	+	-	-	-	-	-	-	15243	-	-	-	166440	-	-	-	-	-	-	-	-	-	-	-	LmjF18.0340	-	-	70628	-	-	-	-	GSPATP00000170001	-	-	-	-	-	-	-	-	-	-	31.m00358	-	-	-	-	-	Tb10.61.3080	-
		-	+	-	-	-	-	-	AT3G04970.1	72462	-	-	184154	166438	-	-	-	-	DDB0185344	-	-	27.m00253	-	-	-	-	155477	-	3070	195499	NCU04080	Os03g24900.1	36089	GSPATP00036640001	4634	129688	140337	-	782822	-	-	SPBC13G1.07	-	-	129.m00116	2550	-	55.m04626	21672	-	-	-
FAP170	Flagellar Associated Protein	-	-	-	+	-	-	-	-	-	-	-	-	166436	-	-	-	-	-	-	-	-	-	-	-	-	-	-	-	-	-	-	-	-	-	-	-	-	-	-	-	-	-	-	-	-	-	-	-	-	-	-
SCAMP	secretory carrier membrane protein	-	+	-	-	-	-	-	AT2G20840.1	-	BDEG_03818	-	-	166432	-	-	-	OTTDARP00000020845	-	CG9195-PA	-	-	ENSGALP00000002580	-	NP_004857.4	-	-	34705	66538	218524	-	Os02g47010.1	37541	-	-	147248	-	-	234069	RO3G_15695.1	-	-	-	SINFRUP00000137748	-	-	-	-	-	90052.m00084	-	UM03218.1
		-	-	-	-	-	-	-	-	-	-	-	-	166424	-	-	-	-	-	-	-	-	-	-	-	-	-	-	-	-	-	-	-	-	-	-	-	-	-	-	-	-	-	-	-	-	-	-	-	-	-	-
		-	-	-	-	-	-	-	-	-	-	-	-	166418	-	-	-	-	-	-	-	-	-	-	-	-	-	-	-	-	-	-	-	-	-	-	-	-	-	-	-	-	-	-	-	-	-	-	-	-	-	-
		-	-	-	-	-	-	-	-	-	-	-	-	166412	-	-	-	-	-	-	-	-	-	-	-	-	-	-	-	-	-	-	-	-	-	-	-	-	-	-	-	-	-	-	-	-	-	-	-	-	-	-
		-	+	-	-	-	-	-	AT5G59980.1	-	BDEG_03422	-	220335	166399	265123	cgd3_1620	CMM152C	-	DDB0190712	-	-	94.m00168	ENSGALP00000010470	-	NP_001098016.1	-	-	29592	-	205577	-	-	35402	-	-	162971	143859	-	203934	-	-	-	-	-	-	-	-	-	-	92349.m00224	-	UM03124.1
		-	-	-	-	-	-	-	-	-	-	-	-	166395	-	-	-	-	-	-	-	-	-	-	-	-	-	-	-	-	-	-	-	-	-	-	-	-	-	-	-	-	-	-	-	-	-	-	-	-	-	-
		-	-	-	-	-	-	-	-	-	-	-	-	166391	-	-	-	-	-	-	-	-	-	-	-	-	-	-	-	-	-	-	-	-	-	-	-	-	-	-	-	-	-	-	-	-	-	-	-	-	-	-
		+	+	-	-	-	-	GB16153-PA	-	-	-	CE40239	158768	166388	376201	-	-	OTTDARP00000015785	-	CG3604-PA	-	-	ENSGALP00000033380	-	NP_001027452.1	-	124792	-	-	-	-	-	-	-	-	-	-	-	-	-	-	-	GLEAN3_23903	SINFRUP00000144132	-	-	-	-	-	-	-	-
ZIL1	ZIP family transporter-like	-	-	-	-	-	-	-	-	-	-	-	-	166383	-	-	-	-	-	-	-	-	-	-	-	-	-	-	-	-	-	-	-	-	-	-	-	-	-	-	-	-	-	-	-	-	-	-	-	-	-	-
		-	-	-	-	-	-	-	AT2G41960.1	-	-	-	-	166376	-	-	-	-	-	-	-	-	-	-	-	-	-	-	-	-	-	Os03g12550.1	-	-	-	142915	-	-	762813	-	-	-	-	-	-	-	-	-	-	-	-	-
		-	+	-	-	-	-	GB13574-PA	AT1G75670.2	-	BDEG_06601	-	197917	166368	-	cgd1_1620	-	-	DDB0189850	CG13773-PA	-	-	ENSGALP00000017640	GL50803_17422	NP_001002926.1	-	228177	7890	-	98605	-	Os12g34854.1	36534	-	-	127692	133135	-	715393	RO3G_05052.1	-	SPBC3B9.07c	-	SINFRUP00000144186	-	-	-	-	51936	94613.m00052	-	-
	predicted Fe-S-cluster redox enzyme	-	-	-	-	-	-	-	AT1G60230.1	-	-	-	-	166362	-	-	-	-	-	-	-	-	-	-	-	-	-	-	-	-	-	Os01g45370.1	-	-	-	162301	-	-	230695	-	-	-	-	-	-	-	-	-	-	-	-	-
		-	-	-	-	-	-	-	-	-	-	-	-	166357	-	-	-	-	-	-	-	-	-	-	-	-	-	-	-	-	-	-	-	-	-	-	-	-	-	-	-	-	-	-	-	-	-	-	-	-	-	-

		-	-	-	-	-	-	-	-	-	-	-	-	166349	-	-	-	-	-	-	-	-	-	-	-	-	-	-	-	-	-	-	-	-	-	-	-	-	-	-	-	-	-	-	-	-	-	-	-	-	-	UM04484.1
		-	-	-	-	-	-	-	-	-	-	-	-	166346	-	-	-	-	-	-	-	-	-	-	-	-	-	-	-	-	-	-	-	-	-	-	-	-	-	-	-	-	-	-	-	-	-	-	-	-	-	-
		-	-	-	-	-	-	-	-	-	-	-	-	166334	-	-	-	-	-	-	-	-	-	-	-	-	-	-	-	-	-	-	-	-	-	-	-	-	-	-	-	-	-	-	-	-	-	-	-	-	-	-
		-	-	-	-	-	-	-	-	-	-	-	-	166327	-	-	-	-	-	-	-	-	-	-	-	-	-	-	-	-	-	-	-	-	-	-	-	-	-	-	-	-	-	-	-	-	-	-	-	-	-	-
		-	+	-	-	-	-	-	AT4G28030.1	-	-	-	-	166320	-	-	-	-	-	-	-	-	-	-	-	-	-	-	-	-	-	Os08g16010.1	34947	-	-	123523	-	-	416378	-	-	-	-	-	-	-	-	-	-	-	-	-
		-	-	-	-	-	-	-	-	-	-	-	-	166317	-	-	-	-	-	-	-	-	-	-	-	-	-	-	-	-	-	-	-	-	-	-	-	-	-	-	-	-	-	-	-	-	-	-	-	-	-	-
		-	-	-	-	-	-	-	-	-	-	-	-	166312	-	-	-	-	-	-	-	-	-	-	-	-	-	-	-	-	-	-	-	-	-	-	-	-	-	-	-	-	-	-	-	-	-	-	-	-	-	-
		-	-	-	-	-	-	-	-	-	-	-	-	166308	-	-	-	-	-	-	-	-	-	-	-	-	-	-	-	-	-	-	-	-	-	-	-	-	-	-	-	-	-	-	-	-	-	-	-	-	-	-
		-	-	-	-	-	-	-	-	-	-	-	-	166306	-	-	-	-	-	-	-	-	-	-	-	-	-	29632	-	-	-	Os05g28860.1	-	GSPATP00025763001	-	-	127414	-	-	-	-	-	-	-	-	-	-	-	-	-	-	-
		-	-	-	-	-	-	-	-	-	-	-	-	166302	-	-	-	-	-	-	-	-	-	-	-	-	-	-	-	-	-	-	-	-	-	-	-	-	-	-	-	-	-	-	-	-	-	-	-	-	-	-
		-	-	-	-	-	-	-	-	-	-	-	-	166299	-	-	-	-	-	-	-	-	-	-	-	-	-	-	-	-	-	-	-	-	-	-	-	-	-	-	-	-	-	-	-	-	-	-	-	-	-	-
		-	-	-	-	-	-	-	-	-	-	-	-	166298	-	-	-	-	-	-	-	-	-	-	-	-	-	-	-	-	-	-	-	-	-	-	-	-	-	-	-	-	-	-	-	-	-	-	-	-	-	-
		-	+	-	-	-	-	-	-	30994	-	CE28355	6286	166297	264409	-	-	-	-	-	-	-	ENSGALP00000005918	-	OTTHUMP00000169758	-	179605	-	-	31495	-	-	-	-	20026	-	-	-	-	-	-	-	GLEAN3_02238	-	-	-	-	-	29304	-	-	-
		-	-	-	-	-	-	-	-	-	-	-	-	166294	-	-	-	-	-	-	-	-	-	-	-	-	-	-	-	-	-	-	-	-	-	-	-	-	-	-	-	-	-	-	-	-	-	-	-	-	-	-
		-	-	-	-	-	-	-	-	-	-	-	-	166292	-	-	-	-	-	-	-	-	-	-	-	-	-	-	-	-	-	-	-	-	-	-	-	-	-	-	-	-	-	-	-	-	-	-	-	-	-	-
		-	-	-	-	-	-	-	-	-	-	-	-	166290	-	-	-	-	-	-	-	-	-	-	-	-	-	-	-	-	-	-	-	-	-	170846	-	-	-	-	-	-	-	-	-	-	-	-	-	-	-	-
		-	+	-	-	-	-	-	AT5G05210.2	-	-	-	-	166278	289762	-	CMT023C	-	-	-	-	-	-	-	-	-	-	33351	-	247520	NCU06090	Os05g30880.1	-	-	36927	72142	-	-	766510	RO3G_06085.1	YKL082C	SPBC947.07	-	-	-	-	-	-	7408	-	-	-
		-	-	-	-	-	-	-	-	-	-	-	-	166267	-	-	-	-	-	-	-	-	-	-	-	-	-	-	-	-	-	-	-	-	-	-	-	-	-	-	-	-	-	-	-	-	-	-	-	-	-	-
		-	+	-	-	-	-	-	AT4G23010.1	63869	-	CE01312	169370	166262	219600	-	-	-	-	CG7853-PA	-	-	ENSGALP00000020819	-	OTTHUMP00000016009	-	138397	-	-	41031	-	Os03g02670.2	25726	-	48904	128915	-	-	730998	-	-	-	-	SINFRUP00000131401	-	11069	-	-	24343	80222.m00124	Tb927.2.2920	-
		-	+	-	-	-	-	-	-	-	-	-	-	166259	237751	-	CMR399C	-	DDB0192048	-	-	-	-	GL50803_6757	-	-	126851	33390	60047	94959	NCU00713	-	-	GSPATP00004658001	-	-	-	-	-	RO3G_09883.1	YGL037C	-	-	-	56.m00180	-	-	-	-	-	-	-
	hypothetical protein	-	-	-	-	-	-	-	-	-	-	-	-	166254	-	-	-	-	-	-	-	-	-	-	-	-	-	-	-	-	-	-	-	-	50343	-	-	-	-	-	-	-	-	-	-	-	-	-	-	-	-	-
		-	-	-	-	-	-	-	-	-	-	-	-	166251	-	-	-	-	-	-	-	-	-	-	-	-	-	-	-	-	-	-	-	-	-	-	-	-	-	-	-	-	-	-	-	-	-	-	-	-	-	-
		-	-	-	-	-	-	-	-	-	-	-	-	166245	-	-	-	-	-	-	-	-	-	-	-	-	-	-	-	-	-	-	-	-	-	-	-	-	-	-	-	-	-	-	-	-	-	-	-	-	-	-
		-	+	-	-	-	-	GB14627-PA	AT3G58140.1	-	BDEG_05463	CE26224	219626	166227	213429	-	CMQ354C	-	DDB0167933	CG13348-PA	-	-	ENSGALP00000020860	-	OTTHUMP00000015986	-	138971	38184	29964	91541	NCU01512	Os12g34860.1	31060	GSPATP00028705001	54597	140667	109324	PFF0180w	836505	RO3G_08760.1	YPR047W	SPCC736.03c	-	SINFRUP00000153358	102.m00109	24163	TA11685	27.m00821	50723	-	-	UM01177.1
		-	-	-	-	-	-	-	-	-	-	-	-	166217	-	-	-	-	-	-	-	-	-	-	-	-	-	-	-	-	-	-	37243	-	-	96490	-	-	-	-	-	-	-	-	-	-	-	-	-	-	-	-
		-	-	-	-	-	-	-	-	-	-	-	-	166215	-	-	CMO027C	-	-	-	-	-	-	-	-	-	-	-	72319	-	-	-	-	-	34920	-	-	-	-	-	-	-	-	-	-	-	-	-	-	-	-	UM05891.1
		-	+	-	-	-	-	GB16094-PA	AT3G08960.1	61792	BDEG_02770	-	149791	166208	291684	-	-	-	DDB0205990	CG33139-PA	-	-	ENSGALP00000037950	-	NP_057422.3	-	219538	26661	70706	248196	NCU02069	Os07g49260.1	-	-	-	136021	-	-	777866	RO3G_13260.1	YPL125W	SPCC1322.06	GLEAN3_22398	SINFRUP00000154639	-	-	-	-	22262	-	-	-
	hypothetical protein	-	-	-	-	-	-	-	-	-	-	-	-	166206	-	-	-	-	-	-	-	-	-	-	-	-	-	-	-	-	-	-	-	-	-	-	-	-	-	-	-	-	-	-	-	-	-	-	-	-	-	-
FAP210	Flagellar Associated Protein	-	+	-	+	-	-	-	-	-	-	-	176680	166203	291706	-	-	-	-	-	-	-	-	-	-	-	225029	-	59931	81851	-	-	-	GSPATP00019721001	-	-	129031	-	-	-	-	-	-	SINFRUP00000149269	92.m00165	-	-	-	55009	-	-	-
		-	-	-	-	-	-	-	-	-	-	-	-	166201	-	-	-	-	-	-	-	-	-	-	-	-	-	-	-	-	-	-	-	-	-	-	-	-	-	-	-	-	-	-	-	-	-	-	-	-	-	-
		-	-	-	-	-	-	-	-	-	-	-	-	166199	-	-	-	-	-	-	-	-	-	-	-	-	-	-	-	244238	-	-	-	-	-	-	-	-	-	-	-	-	-	-	-	-	-	-	-	-	-	-
		-	+	-	-	-	-	GB13646-PA	AT5G49430.1	-	BDEG_04348	-	224246	166194	386100	-	-	-	DDB0218748	-	-	-	ENSGALP00000025835	-	OTTHUMP00000068796	-	140025	-	-	30007	-	Os03g19340.1	30412	-	-	176689	158518	-	664353	RO3G_13482.1	-	-	-	SINFRUP00000171900	-	262607	-	-	25819	-	-	-
		-	+	-	-	-	-	-	-	-	-	-	147020	166190	289368	-	-	-	-	-	-	-	ENSGALP00000038300	-	NP_056300.2	-	-	-	-	239604	-	-	10232	-	-	-	138411	-	-	-	-	-	-	SINFRUP00000178604	-	-	-	-	-	-	-	-
		-	-	-	-	-	-	-	-	-	-	-	-	166187	-	-	-	-	-	-	-	-	-	-	-	-	-	-	-	-	-	-	-	-	-	-	-	-	-	-	-	-	-	-	-	-	-	-	-	-	-	-
	RabGAP/TBC Domain Protein	-	+	-	-	-	-	-	AT3G55020.1	-	-	-	-	166182	-	-	CML175C	-	DDB0169520	-	-	-	-	-	-	-	-	-	-	-	-	Os05g38950.2	31553	-	-	120221	-	-	227764	-	YOL112W	-	-	-	-	-	-	-	-	-	-	-
FAP51	Flagellar Associated Coiled-Coil Protein	-	-	-	+	-	-	-	-	-	-	-	-	166180	-	-	-	-	-	-	-	-	-	-	-	-	-	-	-	-	-	-	-	-	-	-	144452	-	-	-	-	-	-	-	81.m00163	-	-	-	-	-	-	-
		-	+	-	-	-	-	-	-	-	-	-	-	166168	-	-	-	-	DDB0215059	-	-	-	-	-	-	-	-	-	-	-	-	-	31904	-	46762	-	127568	-	-	-	-	-	-	-	-	1197	-	-	-	-	-	-
		-	-	-	-	-	-	-	-	-	-	-	-	166158	-	-	-	-	-	-	-	-	-	-	-	-	-	-	-	-	-	-	-	-	-	-	-	-	-	-	-	-	-	-	-	-	-	-	-	-	-	-
		-	-	-	-	-	-	-	-	-	-	-	-	166151	-	-	-	-	-	-	-	-	-	-	-	-	-	-	-	-	-	-	-	-	-	-	-	-	-	-	-	-	-	-	-	-	-	-	-	-	-	-
		-	+	-	-	-	-	GB13772-PA	AT4G33090.1	27603	BDEG_01963	CE37649	150577	166144	298343	-	CMS341C	OTTDARP00000020905	DDB0217184	CG1009-PF	19068418	114.m00131	ENSGALP00000022242	-	OTTHUMP00000194690	LmjF26.0300	204438	34460	44691	240852	NCU09228	Os08g30810.1	-	-	29758	224579	136251	-	417380	RO3G_00534.1	YKL157W	SPBC1921.05	GLEAN3_13660	SINFRUP00000159528	-	269937	-	-	22803	90729.m00086	Tb11.02.1070	UM00791.1
		-	+	-	-	-	-	-	-	-	BDEG_00569	-	227301	166135	-	-	-	-	DDB0168740	CG13175-PA	-	-	ENSGALP00000012204	-	NP_703152.1	-	232746	-	-	-	NCU08929	Os07g34610.2	29960	-	48910	146163	138759	-	243218	-	-	-	-	SINFRUP00000152104	-	-	-	-	55229	-	-	UM04834.1
ADCY2	adenylate cyclase	-	-	-	-	-	-	-	-	-	-	-	-	166116	-	-	-	-	-	-	-	-	-	-	-	-	-	-	-	-	-	-	-	GSPATP00028233001	-	-	-	-	-	-	-	-	-	-	-	-	-	-	-	-	-	-
ADCY1	adenylate cyclase	-	-	-	-	-	-	-	-	-	-	-	-	166115	-	-	-	-	-	-	-	-	-	-	-	-	-	-	-	-	-	-	-	-	-	-	-	-	-	-	-	-	-	-	-	-	-	-	-	-	-	-
		-	-	-	-	-	-	-	-	-	-	-	-	166106	-	-	-	-	-	-	-	-	-	-	-	-	-	-	-	-	-	-	-	-	-	-	-	-	-	-	-	-	-	-	-	-	-	-	-	-	-	-
		-	-	-	-	-	-	-	AT4G27010.1	-	-	-	-	166103	-	-	-	-	-	-	-	-	-	-	-	-	-	-	-	-	-	-	30094	-	-	69173	-	-	-	-	-	-	-	-	-	-	-	-	-	-	-	-
		-	+	-	-	-	-	-	AT5G42070.1	-	-	-	-	166099	-	-	CMK090C	-	-	-	-	-	-	-	-	-	-	-	-	-	-	Os01g13390.1	31153	-	36434	-	-	-	646769	-	-	-	-	-	-	-	-	-	-	-	-	-
		-	-	-	-	-	-	-	-	-	-	-	-	166097	-	-	-	-	-	CG16896-PA	-	-	-	-	-	-	-	-	-	-	-	-	-	-	-	-	-	-	-	-	-	-	-	-	-	-	-	-	-	-	-	-
		-	-	-	-	-	-	-	-	-	-	-	-	166082	-	-	-	-	-	-	-	-	-	-	-	-	-	-	-	-	-	-	-	-	-	-	-	-	-	-	-	-	-	-	-	-	-	-	-	-	-	-
		-	-	-	-	-	-	-	-	-	-	-	-	166081	-	-	-	-	-	-	-	-	-	-	-	-	-	-	-	-	-	-	-	-	-	-	-	-	-	-	-	-	-	-	-	-	-	-	-	-	-	-
		-	-	-	-	-	-	-	-	-	-	-	-	166080	-	-	-	-	-	-	-	-	-	-	-	-	-	-	-	-	-	-	-	-	-	-	-	-	-	-	-	-	-	-	-	-	-	-	-	-	-	-
		-	-	-	-	-	-	-	-	-	-	-	-	166077	-	-	-	-	-	-	-	-	-	-	-	-	-	-	-	-	-	-	-	-	-	-	-	-	-	-	-	-	-	-	-	-	-	-	-	-	-	-
		-	-	-	-	-	-	-	-	-	-	-	-	166073	-	-	-	-	-	-	-	-	-	-	-	-	-	-	-	-	-	-	-	-	-	-	-	-	-	-	-	-	-	-	-	-	-	-	-	-	-	-
		-	-	-	-	-	-	-	-	-	-	-	-	166067	-	-	-	-	-	-	-	-	-	-	-	-	-	-	-	-	-	-	-	-	-	-	-	-	-	-	-	-	-	-	-	-	-	-	-	-	-	-
		+	+	-	+	-	-	-	-	-	BDEG_01407	-	173417	166062	269885	-	-	OTTDARP00000011178	-	-	-	-	ENSGALP00000022332	-	NP_079285.2	-	213275	-	-	-	-	-	30149	GSPATP00009438001	-	69693	133301	-	-	-	-	-	-	SINFRUP00000171523	260.m00025	-	-	-	-	80874.m00020	-	-
		-	-	-	-	-	-	-	-	-	-	-	218110	166061	-	-	-	-	-	-	-	-	-	-	-	-	-	-	-	-	-	-	-	-	-	-	-	-	-	-	-	-	-	-	-	-	-	-	-	-	-	-
		-	-	-	-	-	-	-	-	-	-	-	-	166055	-	-	-	-	-	-	-	-	-	-	-	-	-	-	-	-	-	-	10448	-	-	-	-	-	-	-	-	-	-	-	-	-	-	-	-	-	-	-
		-	-	-	-	-	-	-	-	-	-	-	-	166054	-	-	-	-	-	-	-	-	-	-	-	-	-	-	-	-	-	-	-	-	-	-	-	-	-	-	-	-	-	-	-	-	-	-	-	-	-	-
		-	+	-	-	-	-	-	-	-	-	-	183814	166046	-	-	-	OTTDARP00000019619	-	CG8295-PD	-	-	ENSGALP00000023316	-	OTTHUMP00000166450	-	-	-	-	-	-	-	-	-	-	-	-	-	-	-	-	-	-	SINFRUP00000156168	-	-	-	-	64011	-	-	-
		-	-	-	-	-	-	-	-	-	-	-	-	166043	-	-	-	-	-	-	-	-	-	-	-	-	-	-	-	-	-	-	-	-	-	-	-	-	-	-	-	-	-	-	-	-	-	-	-	-	-	-
		-	-	-	-	-	-	-	-	-	-	-	-	166042	-	-	-	-	-	-	-	-	-	-	-	-	-	-	-	-	-	-	-	-	-	-	-	-	-	-	-	-	-	-	-	-	-	-	-	-	-	-
		-	-	-	-	-	-	-	-	-	-	-	-	166041	-	-	-	-	-	-	-	-	-	-	-	-	-	-	-	-	-	-	-	-	-	-	-	-	-	-	-	-	-	-	-	-	-	-	-	-	-	-
		-	-	-	-	-	-	-	-	-	-	-	-	166038	-	-	-	-	-	-	-	-	-	-	-	-	-	-	-	-	-	-	-	-	-	-	-	-	-	-	-	-	-	-	-	-	-	-	-	-	-	-
		-	-	-	-	-	-	-	-	-	-	-	-	166037	-	-	-	-	-	-	-	-	-	-	-	-	-	-	-	-	-	-	-	-	-	-	-	-	-	-	-	-	-	-	-	-	-	-	-	-	-	-
		-	-	-	-	-	-	-	AT4G18940.1	-	-	-	-	166035	-	-	-	-	-	-	-	-	-	-	-	-	-	-	-	-	-	Os05g44170.1	-	-	-	148335	135035	-	-	-	-	-	-	-	-	-	-	-	-	-	-	-
		-	+	-	-	-	-	-	AT5G11960.1	-	-	-	-	166032	-	-	-	-	-	-	-	-	-	-	-	-	-	-	-	-	-	Os01g51190.1	36405	-	-	136507	-	-	811964	-	-	-	-	-	-	-	-	-	-	-	-	-
		-	-	-	-	-	-	-	-	-	-	-	-	166025	-	-	-	-	-	-	-	-	-	-	-	-	-	-	-	-	-	-	-	-	-	-	-	-	-	-	-	-	-	-	-	-	-	-	-	-	-	-
		-	-	-	-	-	-	-	-	-	-	-	-	166022	-	-	-	-	-	-	-	-	-	-	-	-	-	-	-	-	-	-	-	-	-	-	-	-	-	-	-	-	-	-	-	-	-	-	-	-	-	-
		-	-	-	-	-	-	-	-	-	-	CE19113	-	166018	-	-	-	-	-	-	-	-	ENSGALP00000029440	-	-	-	-	-	-	-	-	-	-	-	-	-	-	-	-	-	-	-	-	SINFRUP00000133809	-	-	-	-	-	-	-	-
RPL30	Ribosomal protein L30, component of cytosolic 80S ribosome and 60S large subunit	-	+	+	-	-	-	GB12284-PA	AT1G77940.1	31107	BDEG_03572	CE20413	220577	166012	394728	cgd3_3890	CMM040C	OTTDARP00000016256	DDB0191012	CG10652-PA	19170895	76.m00150	ENSGALP00000035832	GL50803_14321	OTTHUMP00000178121	LmjF35.0240	236815	7877	81081	180772	NCU08963	Os01g16890.1	22120	GSPATP00033407001	43840	181527	108147	PF10_0187	827216	RO3G_04160.1	YGL030W	SPAC9G1.03c	GLEAN3_10626	SINFRUP00000140275	51.m00292	29217	TA12435	44.m00044	64110	97324.m00298	Tb10.70.3160	UM04828.1
		-	-	-	-	-	-	-	-	-	-	-	-	166009	-	-	-	-	-	-	-	-	-	-	-	-	-	-	78138	-	-	-	-	-	-	-	-	-	-	-	-	-	-	-	-	-	-	-	-	-	-	-
		-	-	-	-	-	-	-	-	-	-	-	-	166008	-	-	-	-	-	-	-	-	-	-	-	-	-	-	-	-	-	-	-	-	-	-	-	-	-	-	-	-	-	-	-	-	-	-	-	-	-	-
		-	-	-	-	-	-	-	-	-	-	-	-	166001	-	cgd5_3980	-	-	-	-	-	-	-	-	-	-	-	-	-	-	-	-	-	-	42786	-	-	-	-	-	-	-	-	-	-	20892	-	50.m00023	-	-	-	-
		-	-	-	-	-	-	-	-	-	-	-	-	165998	-	-	-	-	-	-	-	-	-	-	-	-	-	-	-	-	-	-	-	-	-	-	-	-	-	-	-	-	-	-	-	-	-	-	-	-	-	-
		-	-	-	-	-	-	-	-	-	-	-	-	165985	-	-	-	-	-	-	-	-	-	-	-	-	-	-	-	-	-	-	-	-	-	-	-	-	-	-	-	-	-	-	-	-	-	-	-	-	-	-
		-	-	-	-	-	-	-	-	-	-	-	-	165982	-	-	-	-	-	-	-	-	-	-	-	-	-	-	-	-	-	-	-	-	-	-	-	-	-	-	-	-	-	-	-	-	-	-	-	-	-	-
		-	-	-	-	-	-	-	-	-	-	-	-	165974	-	-	-	-	-	-	-	-	-	-	-	-	-	-	-	-	-	-	-	-	-	-	-	-	-	-	-	-	-	-	-	-	-	-	-	-	-	-
		-	+	-	-	-	-	GB10286-PA	AT5G36210.1	28304	-	CE01231	219862	165967	-	-	-	-	DDB0188030	CG11319-PA	-	-	-	GL50803_6148	-	-	238013	-	80545	163628	-	Os06g11180.1	-	-	-	189977	-	-	798903	RO3G_15177.1	-	SPBC1711.12	-	-	-	27377	-	-	-	-	-	UM01559.1
		-	-	-	-	-	-	-	-	-	-	-	-	165954	-	-	-	-	-	-	-	-	-	-	-	-	-	-	-	-	-	-	34127	-	-	-	-	-	-	-	-	-	-	-	-	-	-	-	-	-	-	-
		-	-	-	-	-	-	-	-	-	-	-	-	165939	-	-	-	-	-	-	-	-	-	-	-	-	-	-	-	-	-	-	-	-	-	-	-	-	-	-	-	-	-	-	-	-	-	-	-	-	-	-
		-	-	-	-	-	-	-	-	-	-	-	-	165923	-	-	-	-	-	-	-	-	-	-	-	-	-	-	-	-	-	-	-	-	-	-	-	-	-	-	-	-	-	-	-	-	-	-	-	-	-	-
HAF3401	TafII-250 protein	-	+	-	-	-	-	GB30349-PA	AT1G32750.1	-	-	CE26022	225982	165922	269811	-	-	OTTDARP00000018755	DDB0184289	CG17603-PC	-	-	ENSGALP00000008763	-	NP_004597.2	-	212720	-	-	178700	NCU02556	Os06g43790.1	34964	-	42944	94126	-	-	736035	RO3G_02398.1	-	-	-	SINFRUP00000178776	-	-	-	-	56340	-	-	-
		-	-	-	-	-	-	-	-	-	-	-	-	165921	-	-	-	-	-	-	-	-	-	-	-	-	-	-	-	-	-	-	-	-	-	-	-	-	-	-	-	-	-	-	-	-	-	-	-	-	-	-
		-	-	-	-	-	-	-	-	-	-	-	-	165911	-	-	-	-	-	-	-	-	-	-	-	-	-	-	-	-	-	-	-	-	-	-	-	-	-	-	-	-	-	-	-	-	-	-	-	-	-	-
		-	+	-	-	-	-	-	AT5G27990.1	-	-	-	-	165909	-	-	-	-	DDB0190875	-	-	-	-	-	-	-	-	-	-	-	-	Os08g39820.1	-	-	-	18647	142827	PFA0315w	570967	RO3G_09801.1	YLR435W	-	-	-	-	-	-	-	-	-	-	-
		-	-	-	-	-	-	-	-	-	-	-	-	165904	-	-	-	-	-	-	-	-	-	-	-	-	-	-	-	-	-	-	-	-	-	-	-	-	-	-	-	-	-	-	-	-	-	-	-	-	-	-
		-	-	-	-	-	-	-	-	-	-	-	-	165903	-	-	-	-	-	-	-	-	-	-	-	-	-	-	-	-	-	-	-	-	-	-	-	-	-	-	-	-	-	-	-	-	-	-	-	-	-	-
CYP739A4	cytochrome P450, unkown function, in CYP85 clan	-	-	-	-	-	-	-	-	-	-	-	-	165902	-	-	-	-	-	-	-	-	-	-	-	-	-	-	-	-	-	-	-	-	-	-	-	-	-	-	-	-	-	-	-	-	-	-	-	-	-	-
CYP739A5	cytochrome P450, unkown function, in CYP85 clan	-	-	-	-	-	-	-	-	-	-	-	-	165900	-	-	-	-	-	-	-	-	-	-	-	-	-	-	-	175215	-	-	-	-	-	-	-	-	-	-	-	-	-	-	-	-	-	-	-	-	-	-
		-	-	-	-	-	-	-	-	-	-	-	-	165891	-	-	-	-	-	-	-	-	-	-	-	-	-	-	-	-	-	-	-	-	-	-	-	-	-	-	-	-	-	-	-	-	-	-	-	-	-	-
		-	-	-	-	-	-	-	-	-	-	-	-	165881	-	-	-	-	-	-	-	-	-	-	-	-	-	-	-	-	-	-	-	-	-	-	-	-	-	-	-	-	-	-	-	-	-	-	-	-	-	-
		-	-	-	-	-	-	-	-	-	-	-	-	165872	-	-	-	-	-	-	-	-	-	-	-	-	-	-	-	-	-	-	-	-	-	-	-	-	-	-	-	-	-	-	-	-	-	-	-	-	-	-
		-	-	-	-	-	-	-	AT3G56330.1	-	-	-	-	165870	-	-	-	-	-	-	-	-	-	-	-	-	-	-	-	-	-	Os05g25870.1	-	-	-	114954	-	-	571638	-	-	-	-	-	-	-	-	-	-	-	-	-
		-	-	-	-	-	-	-	-	-	-	-	-	165867	-	-	-	-	-	-	-	-	-	-	-	-	-	-	-	-	-	-	-	-	-	-	-	-	-	-	-	-	-	-	-	-	-	-	-	-	-	-
PUS7	Pseudouridine synthase, mitochondrial precursor, putative	-	-	-	-	-	-	-	-	-	BDEG_07819	-	-	165864	-	-	-	-	-	-	-	-	-	-	-	-	-	-	-	-	-	-	-	-	-	-	-	-	-	-	-	-	-	-	-	-	-	-	-	-	-	-
		-	+	-	-	-	-	-	AT1G52500.2	-	-	-	118139	165863	-	-	CMP170C	-	-	-	-	-	-	-	OTTHUMP00000175991	-	-	-	-	-	NCU05984	Os06g43610.1	32844	-	44981	198646	-	-	740875	RO3G_12331.1	-	-	-	-	-	24744	-	-	-	-	-	-
		-	-	-	-	-	-	-	-	-	-	-	-	165855	-	-	-	-	-	-	-	-	-	-	-	-	-	-	-	-	-	-	-	-	-	-	-	-	-	-	-	-	-	-	-	-	-	-	-	-	-	-
	putative RNA binding/rRNA processing protein	-	+	+	-	-	-	GB15193-PA	AT3G11964.1	5211	BDEG_07375	CE27690	150563	165854	275411	cgd8_2050	CMO023C	OTTDARP00000019958	DDB0188761	-	-	420.m00020	ENSGALP00000013431	GL50803_14702	OTTHUMP00000020409	LmjF20.0650	144090	22367	56431	244138	NCU06272	Os07g10350.1	19861	GSPATP00000173001	15354	133341	128675	PF14_0042	256037	RO3G_09720.1	YMR229C	SPCC1183.07	-	SINFRUP00000134892	178.m00060	37965	TA19565	42.m03406	32523	90019.m00235	Tb927.1.1370	UM00678.1
		-	-	-	-	-	-	-	-	-	-	-	-	165849	-	-	-	-	-	-	-	-	-	-	-	-	-	-	-	-	-	-	-	-	-	-	-	-	-	-	-	-	-	-	-	-	-	-	-	-	-	-
		-	-	-	-	-	-	-	-	70762	-	-	-	165841	-	-	-	-	-	-	-	-	-	-	-	-	-	-	-	-	-	-	36083	-	-	-	-	-	-	-	-	-	-	-	-	-	-	-	-	-	-	-
		-	-	-	-	-	-	-	-	-	-	-	-	165836	-	-	-	-	DDB0187467	-	-	-	-	-	-	LmjF03.0750	-	-	-	-	-	-	-	-	-	-	-	-	-	-	-	-	-	-	-	5799	-	-	-	-	-	-
		-	-	-	-	-	-	-	-	-	-	-	-	165814	-	-	-	-	-	-	-	-	-	-	-	-	-	-	-	-	-	-	-	-	-	-	-	-	-	-	-	-	-	-	-	-	-	-	-	-	-	-
		-	-	-	-	-	-	-	-	-	-	-	-	165812	-	-	-	-	-	-	-	-	-	-	-	-	-	-	-	-	-	-	-	-	-	-	-	-	-	-	-	-	-	-	-	-	-	-	-	-	-	-
	speract/scavenger receptor cystein-rich (SRCR) family protein, transmembrane glycoprotein	-	-	-	-	-	-	-	-	-	-	CE38630	-	165809	-	-	-	-	-	-	-	-	-	-	-	-	-	-	-	-	-	-	-	-	-	-	-	PF14_0067	-	-	-	-	-	-	-	-	-	-	-	-	-	-
		-	-	-	-	-	-	-	-	-	-	-	-	165808	-	-	-	-	-	-	-	-	-	-	-	-	-	-	-	-	-	-	-	-	-	-	-	-	-	-	-	-	-	-	-	-	-	-	-	-	-	-
		-	-	-	-	-	-	-	-	-	-	-	-	165806	-	-	-	-	-	-	-	-	-	-	-	-	-	-	-	-	-	-	-	-	-	-	-	-	-	-	-	-	-	-	-	-	-	-	-	-	-	-
		-	+	-	-	-	-	GB19402-PA	AT1G18335.1	-	BDEG_04082	CE28976	94915	165799	265491	cgd6_1800	-	OTTDARP00000022713	-	-	-	-	ENSGALP00000021929	-	NP_079047.1	-	56489	29960	-	233638	-	Os05g32180.1	32241	-	11358	25965	134294	-	729076	RO3G_11858.1	-	SPCC825.04c	-	SINFRUP00000176346	-	21598	TA17725	-	26664	91121.m00064	-	UM00279.1
		-	-	-	-	-	-	-	-	-	-	-	-	165795	-	-	-	-	-	-	-	-	-	-	-	-	-	-	-	-	-	-	-	-	-	-	-	-	-	-	-	-	-	-	-	-	-	-	-	-	-	-
		-	+	-	-	-	-	-	-	-	-	CE23076	-	165793	-	cgd8_1970	-	-	-	-	19069170	2.m00568	-	-	-	-	-	-	-	-	-	-	-	GSPATP00006279001	-	-	-	-	-	RO3G_15040.1	-	-	-	-	3.m01931	-	TA04160	-	-	-	-	-
		-	-	-	-	-	-	-	-	-	-	-	-	165781	-	-	-	-	-	-	-	-	-	-	-	-	-	-	-	-	-	-	-	-	-	-	-	-	-	-	-	-	-	-	-	-	-	-	-	-	-	-
		-	-	-	-	-	-	-	-	-	-	-	-	165778	-	-	-	-	-	-	-	-	-	-	-	-	-	-	-	-	-	-	-	-	-	-	-	-	-	-	-	-	-	-	-	-	-	-	-	-	-	-
		-	+	-	-	-	-	GB19350-PA	-	-	-	CE37401	-	165774	285172	-	CMP253C	-	-	CG1066-PB	-	-	-	-	OTTHUMP00000024387	-	-	-	81831	109242	-	-	-	-	-	-	129034	-	-	-	-	-	-	-	-	-	-	-	2482	-	-	-
		-	-	-	-	-	-	-	-	-	-	-	-	165764	-	-	-	-	-	-	-	-	-	-	-	-	-	-	-	-	-	-	-	-	-	-	-	-	-	-	-	-	-	-	-	-	-	-	-	-	-	-
		-	-	-	-	-	-	-	-	-	-	-	-	165763	-	-	-	-	-	-	-	-	-	-	-	-	-	-	-	-	-	-	-	-	-	-	-	-	-	-	-	-	-	-	-	-	-	-	-	-	-	-
UAPA3	uric acid-xanthine permease	-	-	-	-	-	-	-	-	-	-	-	143404	165762	-	-	-	-	DDB0215824	-	-	-	-	-	-	-	-	-	-	-	-	-	-	-	-	-	-	-	-	RO3G_00119.1	-	-	-	-	-	13485	-	-	-	-	-	-
		-	-	-	-	-	-	-	-	-	-	-	-	165760	-	-	-	-	-	-	-	-	-	-	-	-	-	-	-	-	-	-	-	-	-	-	-	-	-	-	-	-	-	-	-	-	-	-	-	-	-	-
		-	-	-	-	-	-	-	-	-	-	-	-	165757	-	-	-	-	-	-	-	-	-	-	-	-	-	-	-	-	-	-	-	-	-	-	-	-	-	-	-	-	-	-	-	-	-	-	-	-	-	-
		-	-	-	-	-	-	-	-	-	-	-	-	165748	-	-	-	-	-	-	-	-	-	-	-	-	-	-	-	-	-	-	-	-	-	-	-	-	-	-	-	-	-	-	-	-	-	-	-	-	-	-
		-	+	-	-	-	-	GB16740-PA	AT5G63010.1	66715	BDEG_01148	CE40666	91418	165744	248257	cgd6_5020	-	-	DDB0190862	CG3184-PA	-	-	ENSGALP00000014223	-	OTTHUMP00000022705	LmjF02.0620	-	26922	-	83583	NCU02388	Os04g42880.1	12950	-	31975	129722	144654	PFF0395c	671916	-	YBR246W	SPCC18.15	-	SINFRUP00000130292	42.m00251	24785	-	49.m00065	20606	-	-	UM01730.1
		-	-	-	-	-	-	-	-	-	-	-	-	165731	-	-	-	-	-	-	-	-	-	-	-	-	-	-	-	-	-	-	-	-	-	-	-	-	-	-	-	-	-	-	-	-	-	-	-	-	-	-
		-	-	-	-	-	-	-	-	-	-	-	-	165727	-	-	-	-	-	-	-	-	-	-	-	-	-	-	-	-	-	-	-	-	-	-	-	-	-	-	-	-	-	-	-	-	-	-	-	-	-	-
		-	-	-	-	-	-	-	-	-	-	-	-	165708	-	-	-	-	-	-	-	-	-	-	-	-	-	-	-	-	-	-	-	-	-	-	-	-	-	-	-	-	-	-	-	-	-	-	-	-	-	-
		-	-	-	-	-	-	-	-	-	-	-	-	165695	-	-	-	-	-	-	-	-	-	-	-	-	-	-	-	-	-	-	-	-	-	-	-	-	-	-	-	-	-	-	-	-	-	-	-	-	-	-
		-	-	-	-	-	-	-	-	-	-	-	-	165682	-	-	-	-	-	-	-	-	-	-	-	-	-	-	-	-	-	-	-	-	-	-	-	-	-	-	-	-	-	-	-	-	-	-	-	-	-	-
HRP2-2	hydroxyproline-rich cell wall protein	-	-	-	-	-	-	-	-	-	-	-	-	165669	-	-	-	-	-	-	-	-	-	-	-	-	-	-	-	-	-	-	-	-	-	-	-	-	-	-	-	-	-	-	-	-	-	-	-	-	-	-
		-	-	-	-	-	-	-	-	-	-	-	-	165649	-	-	-	-	-	-	-	-	-	-	-	-	-	-	-	-	-	-	-	-	-	-	-	-	-	-	-	-	-	-	-	-	-	-	-	-	-	-
		-	-	-	-	-	-	-	-	-	-	-	-	-	-	-	-	-	-	-	-	-	-	-	-	-	-	-	-	-	-	-	-	-	-	-	-	-	-	-	-	-	-	-	-	-	-	-	-	-	-	-
		-	-	-	-	-	-	-	-	-	-	-	-	-	-	-	-	-	-	-	-	-	-	-	-	-	-	-	-	-	-	-	-	-	-	-	-	-	-	-	-	-	-	-	-	-	-	-	-	-	-	-
		-	-	-	-	-	-	-	-	-	-	-	-	-	-	-	-	-	-	-	-	-	-	-	-	-	-	-	-	-	-	-	-	-	-	-	-	-	-	-	-	-	-	-	-	-	-	-	-	-	-	-
		-	-	-	-	-	-	-	-	-	-	-	-	165627	-	-	-	-	-	-	-	-	-	-	-	-	-	-	-	-	-	-	-	-	-	-	-	-	-	-	-	-	-	-	-	-	-	-	-	-	-	-
		-	-	-	-	-	-	-	-	-	-	-	-	165617	-	-	-	-	-	-	-	-	-	-	-	-	-	-	-	-	-	-	-	-	-	-	-	-	-	-	-	-	-	-	-	-	-	-	-	-	-	-
		-	-	-	-	-	-	-	-	-	-	-	-	-	-	-	-	-	-	-	-	-	-	-	-	-	-	-	-	-	-	-	-	-	-	-	-	-	-	-	-	-	-	-	-	-	-	-	-	-	-	-
		-	-	-	-	-	-	-	-	-	-	-	-	165588	-	-	-	-	-	-	-	-	-	-	-	-	-	-	-	-	-	-	-	-	-	-	-	-	-	-	-	-	-	-	-	-	-	-	-	-	-	-
		-	-	-	-	-	-	-	-	-	-	-	-	165566	-	-	-	-	-	-	-	-	-	-	-	-	-	-	-	-	-	-	-	-	-	-	-	-	-	-	-	-	-	-	-	-	-	-	-	-	-	-
		-	-	-	-	-	-	-	-	-	-	-	-	165549	-	-	-	-	-	-	-	-	-	-	-	-	-	-	-	-	-	-	-	-	-	-	-	-	-	-	-	-	-	-	-	-	-	-	-	-	-	-
		-	-	-	-	-	-	-	-	-	-	-	-	165493	-	-	-	-	-	-	-	-	-	-	-	-	-	-	-	-	-	-	-	-	-	-	-	-	-	-	-	-	-	-	-	-	-	-	-	-	-	-
		-	-	-	-	-	-	-	-	-	-	-	-	-	-	-	-	-	-	-	-	-	-	-	-	-	-	-	-	-	-	-	-	-	-	-	-	-	-	-	-	-	-	-	-	-	-	-	-	-	-	-
MITC9	putative mitochondrial substrate carrier protein	-	+	-	-	-	-	-	AT2G33820.1	-	-	CE02041	176506	165448	292636	-	-	OTTDARP00000016446	-	CG1628-PB	-	-	-	-	OTTHUMP00000178580	-	209317	35981	32196	160795	NCU07263	Os11g23170.3	-	-	-	-	-	-	811009	-	YOR130C	-	GLEAN3_06143	SINFRUP00000134315	-	5924	-	-	-	-	Tb927.8.5810	-
NAT1	N-acetyltransferase-like protein	-	-	-	-	-	-	-	-	-	-	-	-	-	-	-	-	-	-	-	-	-	-	-	-	-	-	-	-	-	-	-	-	-	-	-	-	-	-	-	-	-	-	-	-	-	-	-	-	-	-	-
PSAG	photosystem I reaction center subunit V, chloroplast precursor	-	+	-	-	-	-	-	AT1G55670.1	-	-	-	-	165416	-	-	-	-	-	-	-	-	-	-	-	-	-	-	-	-	-	Os09g30340.1	32716	-	-	130477	-	-	550669	-	-	-	-	-	-	-	-	-	-	-	-	-
		-	-	-	-	-	-	-	-	-	-	-	-	165363	-	-	-	-	-	-	-	-	-	-	-	-	-	-	-	-	-	-	-	GSPATP00036884001	-	-	-	-	-	-	-	-	-	-	-	-	-	-	-	-	-	-
		-	-	-	-	-	-	-	-	-	-	-	-	165344	-	-	-	-	-	-	-	-	-	-	-	-	-	-	-	-	-	-	-	-	-	-	-	-	-	-	-	-	-	-	-	-	-	-	-	-	-	-
		-	-	-	-	-	-	-	-	-	-	-	-	165329	-	-	-	-	-	-	-	-	-	-	-	-	-	-	-	-	-	-	-	-	-	-	-	-	-	-	-	-	-	-	-	-	-	-	-	-	-	-
		-	-	-	-	-	-	-	-	-	-	-	-	165266	-	-	-	-	-	-	-	-	-	-	-	-	-	-	-	-	-	-	-	-	-	-	-	-	-	-	-	-	-	-	-	-	-	-	-	-	-	-
		-	+	-	-	-	-	-	-	23512	BDEG_00162	-	-	165256	-	-	-	-	DDB0219223	-	-	250.m00108	-	-	-	-	-	-	-	-	NCU11040	-	-	-	38754	-	-	-	-	-	-	-	-	-	-	22625	-	-	-	-	-	-
		-	-	-	-	-	-	-	-	-	-	-	-	165230	-	-	-	-	-	-	-	-	-	-	-	-	-	-	-	-	-	-	-	-	-	-	-	-	-	-	-	-	-	-	-	-	-	-	-	-	-	-
		-	-	-	-	-	-	-	-	-	-	-	-	165208	-	-	-	-	-	-	-	-	-	-	-	-	-	-	-	-	-	-	-	-	-	-	-	-	-	-	-	-	-	-	-	-	-	-	-	-	-	-
		-	-	-	-	-	-	-	AT4G09010.1	-	-	-	-	165193	-	-	-	-	-	-	-	-	-	-	-	-	-	-	-	-	-	Os04g51300.1	-	-	-	123146	-	-	209372	-	-	-	-	-	-	-	-	-	-	-	-	-
		-	-	-	-	-	-	-	-	-	-	-	-	165185	-	-	-	-	-	-	-	-	-	-	-	-	-	-	-	-	-	-	-	-	-	-	-	-	-	-	-	-	-	-	-	-	-	-	-	-	-	-
		-	-	-	-	-	-	-	-	-	-	-	-	165144	-	-	-	-	-	-	-	-	-	-	-	-	-	-	-	-	-	-	-	-	-	-	-	-	-	-	-	-	-	-	-	-	-	-	-	-	-	-
		-	-	-	-	-	-	-	-	-	-	-	-	-	-	-	-	-	-	-	-	-	-	-	-	-	-	-	-	-	-	-	-	-	-	-	-	-	-	-	-	-	-	-	-	-	-	-	-	-	-	-
		-	-	-	-	-	-	-	-	-	-	-	-	165086	-	-	-	-	-	-	-	-	-	-	-	-	-	-	-	-	-	-	-	-	-	-	-	-	-	-	-	-	-	-	-	-	-	-	-	-	-	-
KIR2	putative ketoacid isomerase-related protein	-	-	-	-	-	-	-	-	-	-	-	-	165065	-	-	-	-	-	-	-	-	-	-	-	-	-	-	-	-	-	-	-	-	-	-	-	-	-	-	-	-	-	-	-	-	-	-	-	-	-	-
		-	+	-	-	-	-	-	AT5G38650.1	-	-	-	-	165050	-	-	-	-	-	-	-	-	ENSGALP00000029464	-	-	-	-	-	-	236587	-	Os02g55640.1	-	-	-	218370	136578	-	827805	RO3G_08265.1	-	-	-	-	-	-	-	-	-	-	-	-
COP,COP1	chlamyopsin, opsin-related, retinal binding protein	-	-	-	-	-	-	-	-	-	-	-	-	164843	-	-	-	-	-	-	-	-	-	-	-	-	-	-	-	-	-	-	-	-	-	-	-	-	-	-	-	-	-	-	-	-	-	-	-	-	-	-
		-	-	-	-	-	-	-	-	-	-	-	-	164835	-	-	-	-	-	-	-	-	-	-	-	-	-	-	-	-	-	-	-	-	-	-	-	-	-	-	-	-	-	-	-	-	-	-	-	-	-	-
		-	-	-	-	-	-	-	-	-	-	-	-	164826	-	-	-	-	-	-	-	-	-	-	-	-	-	-	-	-	-	-	-	-	-	169823	-	-	827585	-	-	-	-	-	-	-	-	-	-	-	-	-

PET191	similar to yeast PET191 protein required for assembly of cytochrome c oxidase	-	+	-	-	-	-	GB11696-PA	AT1G10865.1	-	-	CE21412	218889	164824	-	-	-	-	-	CG13018-PA	-	-	ENSGALP00000029610	-	NP_001008216.1	-	229366	-	-	191169	NCU02149	Os09g34310.2	31961	-	-	140178	-	-	660079	-	-	-	-	-	-	-	-	-	31742	-	-	UM01896.1
		-	-	-	-	-	-	-	-	-	-	-	-	164800	-	-	-	-	-	-	-	-	-	-	-	-	-	-	-	-	-	-	-	-	-	-	-	-	-	-	-	-	-	-	-	-	-	-	-	-	-	-
VPS20	subunit of ESCRT-III complex	-	+	-	-	-	-	GB18556-PA	AT5G09260.1	-	BDEG_07115	CE22731	145235	164783	384910	-	CMQ184C	-	DDB0218879	CG4071-PA	-	-	ENSGALP00000030825	-	OTTHUMP00000181419	LmjF16.0930	229048	37339	4284	179119	-	Os02g38300.2	-	-	49220	61690	-	-	558455	-	-	SPBC215.14c	GLEAN3_19157	SINFRUP00000163585	-	-	-	-	52939	96800.m00260	Tb927.8.5430	UM04736.1
		-	-	-	-	-	-	-	-	-	-	-	-	-	-	-	-	-	-	-	-	-	-	-	-	-	-	-	-	-	-	-	-	-	-	-	-	-	-	-	-	-	-	-	-	-	-	-	-	-	-	-
		-	-	-	-	-	-	-	-	-	-	-	-	164741	-	-	-	-	-	-	-	-	-	-	-	-	-	-	-	-	-	-	-	-	-	-	-	-	-	-	-	-	-	-	-	-	-	-	-	-	-	-
		-	-	-	-	-	-	-	-	-	-	-	-	164737	-	-	-	-	-	-	-	-	-	-	-	-	-	-	-	-	-	-	-	-	-	-	-	-	-	-	-	-	-	-	-	-	-	-	-	-	-	-
		-	-	-	-	-	-	-	-	-	-	-	-	164726	-	-	-	-	-	-	-	-	-	-	-	-	-	-	-	-	-	-	-	-	-	-	-	-	-	-	-	-	-	-	-	-	-	-	-	-	-	-
		-	-	-	-	-	-	-	-	-	-	-	-	164652	-	-	-	-	-	-	-	-	-	-	-	-	-	-	-	-	-	-	-	-	-	-	-	-	-	-	-	-	-	-	-	-	-	-	-	-	-	-
PP2C;2c	Protein Phosphatase 2C catalytic subunit Homolog 2	-	+	-	-	-	-	-	-	-	-	-	-	164613	-	-	CMK117C	-	DDB0169508	-	-	-	-	-	-	-	180731	-	-	-	-	-	-	-	-	-	-	-	-	-	-	-	-	SINFRUP00000130872	-	-	TA15545	-	-	-	Tb11.01.6540	-
	glutathione S-transferase	+	+	-	-	-	-	GB14372-PA	-	35132	-	CE22420	165166	164603	-	-	-	OTTDARP00000021255	-	CG8938-PC	-	-	-	-	OTTHUMP00000016611	-	238691	-	82475	113255	-	-	-	-	-	-	145197	-	-	-	-	-	GLEAN3_23664	SINFRUP00000164401	-	-	-	-	63816	-	-	-
		-	-	-	-	-	-	-	-	-	-	-	-	164600	-	-	-	-	-	-	-	-	-	-	-	-	-	-	-	-	-	-	-	-	-	-	-	-	-	-	-	-	-	-	-	-	-	-	-	-	-	-
		-	-	-	-	-	-	-	-	-	-	-	-	164589	-	-	-	-	-	-	-	-	-	-	-	-	-	-	-	-	-	-	-	-	-	-	-	-	-	-	-	-	-	-	-	-	-	-	-	-	-	-
		-	-	-	-	-	-	-	-	-	-	-	-	164558	-	-	-	-	-	-	-	-	-	-	-	-	-	-	-	-	-	-	-	-	-	-	-	-	-	-	-	-	-	-	-	-	-	-	-	-	-	-
		-	-	-	-	-	-	-	-	-	-	-	-	-	-	-	-	-	-	-	-	-	-	-	-	-	-	-	-	-	-	-	-	-	-	-	-	-	-	-	-	-	-	-	-	-	-	-	-	-	-	-
		-	-	-	-	-	-	-	-	-	-	-	-	164531	-	-	-	-	-	-	-	-	-	-	-	-	-	-	-	-	-	-	-	-	-	-	-	-	-	-	-	-	-	-	-	-	-	-	-	-	-	-
		-	-	-	-	-	-	-	-	-	-	-	-	164497	-	-	-	-	-	-	-	-	-	-	-	-	-	-	-	-	-	-	-	-	-	-	-	-	-	-	-	-	-	-	-	-	-	-	-	-	-	-
		-	-	-	-	-	-	-	-	-	-	-	-	164448	-	-	-	-	-	-	-	-	-	-	-	-	-	-	-	-	-	-	-	-	-	-	-	-	-	-	-	-	-	-	-	-	-	-	-	-	-	-
NUOB22	NADH:ubiquinone oxidoreductase (Complex I) 14 kD subunit, homolog to bovine B22 subunit, mitochondrial	-	+	-	-	-	-	GB10916-PA	AT4G34700.1	9735	-	-	160682	164424	298143	-	CMM030C	OTTDARP00000023071	-	CG9306-PA	-	-	ENSGALP00000026301	-	OTTHUMP00000178018	-	-	27131	-	119247	-	Os06g50000.1	-	-	-	159623	144183	-	195701	RO3G_00955.1	-	-	GLEAN3_12368	SINFRUP00000131100	-	-	-	-	8831	-	-	UM05625.1
CHYB	beta-carotene hydroxylase, putative chloroplast precursor; PMID:15849308	-	+	-	-	-	-	-	AT4G25700.1	-	-	-	-	164400	-	-	-	-	-	-	-	-	-	-	-	-	-	-	-	-	-	Os03g03370.1	5089	-	-	34090	-	-	826998	-	-	-	-	-	-	263437	-	-	-	-	-	-
FAP276	Flagellar Associated Protein	-	-	-	-	-	-	-	-	-	-	-	-	164394	-	-	-	-	-	-	-	-	-	-	-	-	-	-	-	-	-	-	-	-	-	-	-	-	-	-	-	-	-	-	-	-	-	-	-	-	-	-
		-	+	-	-	-	-	-	AT1G54310.2	2373	-	-	-	164377	-	-	CMM194C	-	-	-	-	-	-	-	-	-	-	-	-	-	-	Os02g07740.1	14362	-	44311	225566	-	-	724233	-	-	-	-	-	-	35911	-	-	-	-	-	-
		-	-	-	-	-	-	-	AT4G27700.1	-	-	-	-	164369	-	-	-	-	-	-	-	-	-	-	-	-	-	-	-	-	-	-	-	-	-	-	-	-	-	-	-	-	-	-	-	-	-	-	-	-	-	-
		-	-	-	-	-	-	-	-	-	-	-	-	164347	-	-	-	-	-	-	-	-	-	-	-	-	-	-	-	-	-	-	-	-	-	-	-	-	-	-	-	-	-	-	-	-	-	-	-	-	-	-
		-	-	-	-	-	-	-	-	-	-	-	-	164345	-	-	-	-	-	-	-	-	-	-	-	-	-	-	-	-	-	-	-	-	44321	103465	-	-	-	-	-	-	-	-	-	9410	-	-	-	-	-	-
SPC12	Signal Peptidase, 12 kDa subunit	-	+	-	-	-	-	GB30557-PA	AT4G40042.1	17929	-	CE16898	182747	164331	399158	-	-	OTTDARP00000023615	-	-	-	-	ENSGALP00000002523	GL50803_3154	NP_054760.2	-	-	13509	-	169043	NCU11313	Os12g25210.1	36345	-	13921	9503	-	-	658139	-	-	-	-	-	-	34186	-	-	57735	-	-	-
		-	+	-	-	-	-	-	AT2G11890.1	-	BDEG_02608	-	-	164328	-	-	-	-	-	-	-	-	-	GL50803_17583	-	-	-	-	-	-	-	Os08g20400.1	-	-	-	170344	-	-	825994	-	-	-	-	-	-	-	-	-	-	88599.m00158	-	-
		-	-	-	-	-	-	-	-	-	-	-	-	164303	-	-	-	-	-	-	-	-	-	-	-	-	-	-	-	-	-	-	-	-	-	-	-	-	-	-	-	-	-	-	-	-	-	-	-	-	-	-
NUOB18	NADH:ubiquinone oxidoreductase 10 kD protein (Complex I B18 subunit), mitochondrial	-	+	-	-	-	-	GB15252-PA	AT2G02050.1	9255	BDEG_07367	CE09081	69485	164272	234157	-	-	-	DDB0206064	CG5548-PA	-	-	-	-	OTTHUMP00000067434	-	234648	33481	-	232250	NCU11348	Os03g03770.1	7737	-	-	211358	111793	-	822033	RO3G_07804.1	-	-	GLEAN3_03071	SINFRUP00000149424	-	34739	-	-	8916	-	-	-
		-	-	-	-	-	-	-	-	-	-	-	-	164267	-	-	-	-	-	-	-	-	-	-	-	-	-	-	-	-	-	-	-	-	-	-	-	-	-	-	-	-	-	-	-	-	-	-	-	-	-	-
		-	+	-	-	-	-	-	-	8446	-	-	-	164251	290785	-	CMM041C	OTTDARP00000014879	DDB0167226	-	-	-	-	-	OTTHUMP00000024525	-	215760	-	65741	100698	NCU09255	-	31623	GSPATP00015276001	-	-	-	-	-	RO3G_17078.1	-	SPBP23A10.03c	-	SINFRUP00000183015	112.m00113	-	-	-	53866	-	-	-
		-	-	-	-	-	-	-	-	-	-	-	-	164218	-	-	-	-	-	-	-	-	-	-	-	-	-	-	-	-	-	-	-	-	-	-	-	-	-	-	-	-	-	-	-	-	-	-	-	-	-	-
		-	-	-	-	-	-	-	-	-	-	-	-	164198	-	-	-	-	-	-	-	-	-	-	-	-	-	-	-	-	-	-	-	GSPATP00033461001	-	-	-	-	-	-	-	-	-	-	-	-	-	-	-	-	-	-
		-	+	-	-	-	-	-	AT4G12960.1	-	-	-	-	164197	-	-	-	-	-	-	-	-	-	GL50803_5795	-	-	-	-	-	-	NCU06997	-	-	-	-	-	-	-	-	-	-	-	-	-	39.m00269	-	-	-	-	-	-	UM06365.1
		-	-	-	-	-	-	-	AT4G15790.1	-	-	-	-	164196	-	-	-	-	-	-	-	-	-	-	-	-	-	-	-	-	-	Os12g35620.1	-	-	-	68068	-	-	768814	-	-	-	-	-	-	-	-	-	-	-	-	-
		-	-	-	-	-	-	-	-	-	-	-	-	164180	-	-	-	-	-	-	-	-	-	-	-	-	-	-	-	-	-	-	-	-	-	-	-	-	-	-	-	-	-	-	-	-	-	-	-	-	-	-
PHC17	cell wall protein pherophorin-C17	-	-	-	-	-	-	-	-	-	-	-	-	164137	-	-	-	-	-	-	-	-	-	-	-	-	-	-	-	-	-	-	-	-	-	-	-	-	-	-	-	-	-	-	-	-	-	-	-	-	-	-
RPP0	Acidic ribosomal protein P0, component of cytosolic 80S ribosome and 60S large subunit	-	+	+	-	-	-	GB12338-PA	AT3G09200.1	39351	BDEG_03513	CE09655	167366	164097	255020	cgd4_2260	CMQ445C	-	DDB0218835	CG7490-PA	19069146	20.m00312	ENSGALP00000011717	GL50803_17054	OTTHUMP00000082939	LmjF27.1380	237709	18498	83230	237425	NCU07408	Os12g03880.1	23034	GSPATP00031832001	30660	179848	109515	PF11_0313	822404	RO3G_16997.1	YLR340W	SPCC18.14c	GLEAN3_28366	SINFRUP00000139433	91.m00115	25812	TA21355	38.m00002	63360	92204.m00046	Tb11.46.0002	UM06055.1
		-	+	-	-	-	-	-	AT3G50560.1	29475	-	-	-	163989	-	-	-	-	DDB0185839	-	-	-	-	-	-	-	-	-	-	-	-	Os08g37130.1	-	-	20677	136475	-	-	218742	-	-	-	-	-	-	-	-	-	-	-	-	UM06020.1
		-	-	-	-	-	-	-	-	-	-	-	-	163935	-	-	-	-	-	-	-	-	-	-	-	-	-	-	-	-	-	-	-	-	-	-	-	-	-	-	-	-	-	-	-	-	-	-	-	-	-	-
		-	-	-	-	-	-	-	-	-	-	-	-	163916	-	-	-	-	-	-	-	-	-	-	-	-	-	-	-	-	-	-	-	-	-	161165	157495	-	-	-	-	-	-	-	-	-	-	-	-	-	-	-
		-	-	-	+	-	-	-	-	-	-	-	-	163874	-	-	-	-	-	-	-	-	-	-	-	-	-	-	-	-	-	-	-	-	-	-	-	-	-	-	-	-	-	-	-	-	-	-	-	-	-	-
CAH3	carbonic anhydrase 3	-	+	-	-	-	-	GB12834-PA	AT1G08080.1	-	BDEG_03440	CE04755	224291	163824	272723	-	CMI270C	OTTDARP00000022230	-	CG7820-PA	-	-	ENSGALP00000025460	-	OTTHUMP00000177821	LmjF28.0480	202179	-	-	200803	NCU05653	Os08g32780.1	-	GSPATP00030154001	-	168709	132952	-	294146	-	-	-	GLEAN3_13459	SINFRUP00000170263	12.m00449	262006	-	-	63940	-	-	-
		-	-	-	-	-	-	-	-	-	-	-	-	163818	-	-	-	-	-	-	-	-	-	-	-	-	-	-	-	-	-	-	-	-	-	-	-	-	-	-	-	-	-	-	-	-	-	-	-	-	-	-
		-	-	-	-	-	-	-	-	-	-	-	-	163761	-	-	-	-	-	-	-	-	-	-	-	-	-	-	-	-	-	-	-	-	-	-	-	-	-	-	-	-	-	-	-	-	-	-	-	-	-	-
CBR1	NADH-cytochrome b5 reductase	-	+	-	-	-	-	-	AT5G17770.1	-	BDEG_01027	-	-	163751	-	-	CMS286C	-	DDB0218707	-	-	-	-	-	-	LmjF15.0050	-	-	3483	-	NCU00216	Os05g40990.1	22984	-	-	218930	108379	PF13_0353	741708	RO3G_03220.1	YIL043C	SPCC970.03	-	-	-	-	-	-	-	-	-	UM00646.1
		-	-	-	-	-	-	-	-	-	-	-	-	163673	-	-	-	-	-	-	-	-	-	-	-	-	-	-	-	-	-	-	-	-	-	-	-	-	-	-	-	-	-	-	-	-	-	-	-	-	-	-
RPT3	26S proteasome regulatory subunit	-	+	+	-	-	-	GB19878-PA	AT5G58290.1	27118	BDEG_05794	CE01253	159938	163663	222847	cgd4_2540	CMF078C	OTTDARP00000009638	DDB0188209	CG16916-PA	19069264	402.m00033	-	GL50803_7950	XP_947602.1	LmjF12.0210	212332	33222	60529	236753	NCU02260	Os02g21970.1	8704	GSPATP00025927001	11735	164900	122821	PFD0665c	734681	RO3G_11703.1	YDR394W	SPCC576.10c	GLEAN3_16198	SINFRUP00000179380	4.m00599	24475	TA17225	44.m00017	50249	84816.m00021	Tb927.6.1090	UM00546.1
		-	-	-	-	-	-	-	-	-	-	-	-	163661	-	-	-	-	-	-	-	-	-	-	-	-	-	-	-	-	-	-	-	-	-	-	-	-	-	-	-	-	-	-	-	-	-	-	-	-	-	-
		-	-	-	-	-	-	-	-	-	-	-	-	-	-	-	-	-	-	-	-	-	-	-	-	-	-	-	-	-	-	-	-	-	-	-	-	-	-	-	-	-	-	-	-	-	-	-	-	-	-	-
		-	+	-	-	-	-	GB14340-PA	AT5G63220.1	-	BDEG_06219	-	220393	163582	-	-	-	-	-	CG9853-PA	-	-	ENSGALP00000006013	-	OTTHUMP00000025223	-	154705	-	-	231906	-	Os01g07100.1	-	-	-	161327	-	-	724894	RO3G_04846.1	-	-	-	SINFRUP00000149082	-	-	-	-	-	-	-	-
		-	-	-	-	-	-	-	-	-	-	-	-	163517	-	-	-	-	-	-	-	-	-	-	-	-	-	-	-	-	-	-	-	-	-	-	-	-	-	-	-	-	-	-	-	-	-	-	-	-	-	-
ATX1	putative copper chaperone	-	+	-	-	-	-	GB14210-PA	AT3G56240.1	-	-	CE29163	179263	163414	-	-	-	-	DDB0185901	CG32446-PA	-	-	ENSGALP00000006626	-	-	-	226396	-	-	-	-	Os08g10480.1	32350	-	-	187462	158523	-	420077	-	YNL259C	-	GLEAN3_18329	SINFRUP00000138642	-	-	-	-	30441	-	-	-
GLD2-Carboxy	glucose-6-phosphate dehydrogenase, plastidic; carboxy portion	-	-	-	-	-	-	-	-	-	-	-	-	163301	-	-	-	-	-	-	-	-	-	-	-	-	-	-	-	-	-	-	-	-	-	-	-	-	-	-	-	-	-	-	-	-	-	-	-	-	-	-
		-	-	-	-	-	-	-	-	-	-	-	-	163283	-	-	-	-	-	-	-	-	-	-	-	-	-	-	-	-	-	-	-	-	-	-	-	-	-	-	-	-	-	-	-	-	-	-	-	-	-	-
		-	-	-	-	-	-	-	-	-	-	-	-	163282	-	-	-	-	-	-	-	-	-	-	-	-	-	-	-	-	-	-	-	-	-	-	-	-	-	-	-	-	-	-	-	-	-	-	-	-	-	-
		-	-	-	-	-	-	-	-	-	-	-	-	163263	-	-	-	-	-	-	-	-	-	-	-	-	-	-	-	-	-	-	-	-	-	-	-	-	-	-	-	-	-	-	-	-	-	-	-	-	-	-
RIBA	Putative Bifunctional GTP cyclohydrolase II/3,4-dihydroxy-2butanone-4-phosphate synthase	-	+	-	-	-	-	-	-	-	BDEG_04540	-	-	163238	-	-	-	-	-	-	-	-	-	-	-	-	-	-	-	-	NCU07188	-	-	-	10882	-	135349	-	-	RO3G_07444.1	YBL033C	SPAP27G11.09c	-	-	-	33619	-	-	-	-	-	UM04826.1
		-	+	-	-	-	-	-	AT5G39790.1	-	-	-	-	163196	-	-	-	-	-	-	-	-	-	-	-	-	-	-	-	-	-	Os02g04330.2	38040	-	-	161880	-	-	553002	-	-	-	-	-	-	-	-	-	-	-	-	-
		-	+	-	-	-	-	GB20144-PA	AT3G07090.1	29814	BDEG_04289	-	163285	163188	223508	cgd1_1270	CML296C	OTTDARP00000022790	DDB0191673	-	19171317	-	ENSGALP00000019438	GL50803_24425	OTTHUMP00000042148	LmjF33.2260	87444	-	79040	83774	-	Os02g56900.1	6714	GSPATP00026519001	-	129993	144479	PFL0865w	816898	RO3G_11253.1	-	-	-	SINFRUP00000145193	98.m00193	38948	-	583.m05445	17307	-	Tb09.160.2050	UM04578.1
		-	-	-	-	-	-	-	-	-	-	-	-	163170	-	-	-	-	-	-	-	-	-	-	-	-	-	-	-	-	-	-	-	-	-	-	-	-	-	-	-	-	-	-	-	-	-	-	-	-	-	-
		-	+	-	-	-	-	-	AT4G03150.1	-	-	-	-	163128	-	-	-	-	-	-	-	-	-	-	-	-	-	-	-	-	-	-	32928	-	47833	-	-	-	245989	-	-	-	-	-	-	2516	-	-	-	-	-	-
		-	-	-	-	-	-	-	-	-	-	-	-	163105	-	-	-	-	-	-	-	-	-	-	-	-	-	-	-	-	-	-	-	-	-	-	-	-	-	-	-	-	-	-	-	-	-	-	-	-	-	-
		-	-	-	-	-	-	-	-	-	-	-	-	163086	-	-	-	-	-	-	-	-	-	-	-	-	-	-	-	-	-	-	-	-	-	-	-	-	-	-	-	-	-	-	-	-	-	-	-	-	-	-
		-	-	-	-	-	-	-	-	-	-	-	-	163028	-	-	-	-	-	-	-	-	-	-	-	-	-	-	-	-	-	-	-	-	-	-	-	-	-	-	-	-	-	-	-	-	-	-	-	-	-	-
		-	+	-	-	-	-	-	AT5G42890.1	-	BDEG_03268	-	179150	163013	267458	-	-	OTTDARP00000023005	-	CG11151-PA	-	-	ENSGALP00000017306	-	NP_001007100.1	-	199394	-	-	207642	NCU06643	Os02g52720.1	-	GSPATP00024903001	-	204058	139933	-	836444	RO3G_01383.1	-	-	GLEAN3_06383	SINFRUP00000135532	-	-	-	-	59384	-	-	UM03005.1
		-	-	-	-	-	-	-	-	-	-	-	-		-	-	-	-	-	-	-	-	-	-	-	-	-	-	-	-	-	-	-	-	-	-	-	-	-	-	-	-	-	-	-	-	-	-	-	-	-	-
		-	-	-	-	-	-	-	-	-	-	-	-	162860	-	-	-	-	-	-	-	-	-	-	-	-	-	-	-	-	-	-	-	-	-	-	-	-	-	-	-	-	-	-	-	-	-	-	-	-	-	-
RPL13a	Ribosomal protein L13a, component of cytosolic 80S ribosome and 60S large subunit	-	+	+	-	-	-	GB16111-PA	AT4G13170.1	58864	BDEG_02033	CE01030	220822	162845	216912	cgd5_970	CMR122C	OTTDARP00000009674	DDB0217704	CG1475-PB	19068859	199.m00089	-	GL50803_11247	OTTHUMP00000077978	LmjF34.0860	183721	34035	83175	188032	NCU01221	Os07g01870.1	26663	GSPATP00007693001	25375	183175	109046	PF10_0043	835660	RO3G_15704.1	YIL133C	SPAC23A1.11	GLEAN3_06213	SINFRUP00000157137	119.m00110	46	TA08170	80.m00062	36021	95725.m00251	Tb927.5.1610	UM00926.1
		-	-	-	-	-	-	-	-	-	-	-	-	162827	-	-	-	-	-	-	-	-	-	-	-	-	-	-	-	-	-	-	-	-	-	-	-	-	-	-	-	-	-	-	-	-	-	-	-	-	-	-
		-	+	-	-	-	-	-	AT5G05360.1	-	-	-	-	162817	-	-	-	-	-	-	-	-	-	-	-	-	-	-	-	-	-	Os03g18700.1	24457	-	-	92981	-	-	833745	-	-	-	-	-	-	-	-	-	-	-	-	-
		-	+	-	-	-	-	GB14546-PA	AT5G62290.1	-	-	CE30069	184496	162806	-	-	-	-	-	-	-	-	-	-	-	-	-	-	-	240068	-	Os10g08879.3	-	-	-	162258	128386	-	570580	-	-	-	-	-	-	-	TA12600	39.m00622	59514	-	-	-
NDUFA11	NADH:ubiquinone oxidoreductase 23 kDa subunit (Complex I B14.7 subunit), mitochondrial	-	-	-	-	-	-	-	-	-	-	-	-	162765	-	-	-	-	-	-	-	-	-	-	-	-	-	-	-	-	-	-	-	-	-	-	-	-	-	-	-	-	-	-	-	-	-	-	-	-	-	UM02893.1
		-	-	-	-	-	-	-	-	-	-	-	-	162698	-	-	-	-	-	-	-	-	-	-	-	-	-	-	-	-	-	-	-	-	-	-	-	-	-	-	-	-	-	-	-	-	-	-	-	-	-	-
WNK2	With no Lysine Homolog 2	-	+	-	-	+	-	GB16910-PB	AT5G41990.1	26387	BDEG_03110	CE37332	-	162667	253799	-	-	OTTDARP00000011134	-	CG7177-PA	-	-	ENSGALP00000004717	-	OTTHUMP00000181227	-	52941	32672	31865	-	-	Os05g01780.1	18914	-	-	1987	136369	-	252319	-	-	-	GLEAN3_22200	-	75.m00159	35929	-	-	4059	-	-	-
FAP109	Flagellar Associated Protein	-	-	-	+	-	-	-	-	-	-	-	-	162635	-	-	-	-	-	-	-	-	-	-	-	-	-	-	-	-	-	-	-	-	-	-	-	-	-	-	-	-	-	-	-	-	-	-	-	-	-	-
		-	-	-	-	-	-	-	-	-	-	-	-	162620	-	-	-	-	-	-	-	-	-	-	-	-	-	-	-	-	-	-	-	-	-	-	-	-	-	-	-	-	-	-	-	-	-	-	-	-	-	-
		-	+	-	-	-	-	-	AT3G29170.1	-	-	-	-	162615	212482	-	-	-	-	-	-	-	-	-	-	-	177673	-	-	170431	-	-	-	-	-	-	135286	-	-	-	-	-	-	-	-	-	-	-	-	-	-	-
		-	+	-	-	-	-	-	AT4G04330.1	-	-	-	-	162607	-	-	-	-	-	-	-	-	-	-	-	-	-	-	-	-	-	Os08g32930.1	37731	-	-	131396	-	-	233489	-	-	-	-	-	-	-	-	-	-	-	-	-
		-	+	-	-	-	-	-	AT2G24390.1	-	BDEG_08197	-	-	162552	-	-	CMC017C	-	-	-	-	-	-	-	-	-	-	-	-	-	-	Os06g48960.1	-	-	39079	116387	-	-	785490	-	-	-	-	-	-	6348	-	-	-	-	-	-
		-	-	-	-	-	-	-	-	-	-	CE06739	-	162535	-	-	-	-	-	CG2614-PA	-	-	-	-	OTTHUMP00000173800	-	-	-	-	-	-	-	-	-	-	-	-	-	-	-	-	-	-	SINFRUP00000160056	-	-	-	-	-	-	-	-
		-	-	-	-	-	-	-	-	-	-	-	-	162526	-	-	-	-	-	-	-	-	-	-	-	-	-	-	-	-	-	-	-	-	-	-	-	-	-	-	-	-	-	-	-	-	-	-	-	-	-	-
		-	-	-	-	-	-	-	-	-	-	-	-	162515	-	-	-	-	-	-	-	-	-	-	-	-	-	-	-	-	-	-	-	-	-	-	-	-	-	-	-	-	-	-	-	-	-	-	-	-	-	-
		-	-	-	-	-	-	-	-	-	-	-	-	162504	-	-	-	-	-	-	-	-	-	-	-	-	-	-	-	-	-	-	-	-	-	-	-	-	-	-	-	-	-	-	-	-	-	-	-	-	-	-
		-	+	-	-	-	-	-	AT3G07910.1	-	-	-	-	162495	-	-	-	-	-	CG6878-PA	-	-	-	-	-	-	215964	-	-	-	-	-	-	-	-	224512	-	-	641469	-	-	-	-	-	-	-	-	-	-	-	-	-
		-	+	+	-	-	-	GB10254-PA	AT3G22660.1	26344	BDEG_07927	CE28890	17805	162486	206304	cgd6_3450	CMD030C	-	DDB0186212	-	-	134.m00118	ENSGALP00000014708	-	OTTHUMP00000008354	LmjF35.1820	126319	16531	50673	239593	NCU01257	Os07g29320.1	24321	-	8777	181901	137795	-	575699	RO3G_05540.1	YKL172W	SPAC17H9.05	GLEAN3_10328	SINFRUP00000143162	62.m00228	24016	-	50.m05615	57829	-	Tb09.244.2790	UM04809.1
		-	-	-	+	-	-	-	-	-	-	-	-	162449	-	-	-	-	-	-	-	-	-	-	-	-	-	-	-	-	-	-	-	-	-	-	-	-	-	-	-	-	-	-	-	-	-	-	-	-	-	-
		-	+	-	-	-	-	GB16932-PA	AT3G05760.1	31817	-	CE00458	20763	162439	248700	cgd3_1060	-	-	DDB0206391	CG11586-PA	-	-	ENSGALP00000040487	-	OTTHUMP00000159885	-	228476	33398	47922	165407	-	Os03g61640.1	32329	GSPATP00000971001	50130	193667	158437	PFL2075c	729449	RO3G_16163.1	-	-	GLEAN3_10009	SINFRUP00000158832	4.m00527	11166	TA08155	63.m00147	59979	92066.m00115	-	UM05159.1
		-	+	-	-	-	-	GB12175-PA	-	-	-	-	184521	162432	-	-	-	-	-	-	-	-	-	-	-	-	-	-	-	100325	-	-	35694	-	-	-	138791	PF13_0208	-	-	-	-	-	-	-	2991	-	-	-	-	-	-
		-	-	-	-	-	-	-	-	-	-	-	-	162377	-	-	-	-	-	-	-	-	-	-	-	-	-	-	-	-	-	-	-	-	-	-	-	-	-	-	-	-	-	-	-	-	-	-	-	-	-	-
		-	-	-	-	-	-	-	-	-	-	-	-	162327	-	-	-	-	-	-	-	-	-	-	-	-	-	-	-	-	-	-	-	-	-	-	-	-	-	-	-	-	-	-	-	-	-	-	-	-	-	-
		-	-	-	-	-	-	-	-	-	-	-	-	162312	-	-	-	-	-	-	-	-	-	-	-	-	-	-	-	-	-	-	-	-	-	-	-	-	-	-	-	-	-	-	-	-	-	-	-	-	-	-
LCI15	PRLI-interacting factor L	-	-	-	-	-	-	-	-	-	-	-	-	162303	-	-	-	-	-	-	-	-	-	-	-	-	-	-	-	-	-	-	-	-	-	-	-	-	-	-	-	-	-	-	-	-	-	-	-	-	-	-
		-	-	-	-	-	-	-	-	-	-	-	-	-	-	-	-	-	-	-	-	-	-	-	-	-	-	-	-	-	-	-	-	-	-	-	-	-	-	-	-	-	-	-	-	-	-	-	-	-	-	-
		-	-	-	-	-	-	-	-	-	-	-	-	162235	-	-	-	-	-	-	-	-	-	-	-	-	-	-	-	-	-	-	-	-	-	-	-	-	-	-	-	-	-	-	-	-	-	-	-	-	-	-
	soluble starch synthase II, ADP-glucose alpha-1,4 glucane alpha-4-glucanotransferase	-	+	-	-	-	-	-	AT3G01180.1	-	-	-	-	162226	-	-	CMM317C	-	-	-	-	94.m00134	-	-	-	-	-	-	-	-	-	Os06g12450.1	27007	-	-	202950	-	-	259135	-	-	SPCC63.04	-	-	-	-	-	-	-	-	-	-
		-	-	-	-	-	-	-	-	-	-	-	-	162196	-	-	-	-	-	-	-	-	-	-	-	-	-	-	-	-	-	-	-	-	-	-	-	-	-	-	-	-	-	-	-	-	-	-	-	-	-	-
		-	-	-	-	-	-	-	-	-	-	-	-	162192	-	-	-	-	-	-	-	-	-	-	-	-	-	-	-	-	-	-	-	-	-	-	-	-	-	-	-	-	-	-	-	-	-	-	-	-	-	-
		-	-	-	-	-	-	-	-	-	-	-	-	162117	-	-	-	-	-	-	-	-	-	-	-	-	-	-	-	-	-	-	-	-	-	-	-	-	-	-	-	-	-	-	-	-	-	-	-	-	-	-
		-	-	-	-	-	-	-	-	-	-	-	-	162086	-	-	-	-	-	-	-	-	-	-	-	-	-	-	-	-	-	-	-	-	-	-	-	-	-	-	-	-	-	-	-	-	-	-	-	-	-	-
		-	-	-	-	-	-	-	-	-	-	-	-	162043	-	-	-	-	-	-	-	-	-	-	-	-	-	-	-	-	-	-	-	-	-	-	-	-	-	-	-	-	-	-	-	-	-	-	-	-	-	-
		-	-	-	-	-	-	-	-	-	-	-	-	162029	-	-	-	-	-	-	-	-	-	-	-	-	-	-	-	-	-	-	-	-	-	-	-	-	-	-	-	-	-	-	-	-	-	-	-	-	-	-
		-	-	-	-	-	-	-	-	-	-	-	-	161992	-	-	-	-	-	-	-	-	-	-	-	-	-	-	-	-	-	-	-	-	-	-	-	-	-	-	-	-	-	-	-	-	-	-	-	-	-	-
		-	-	-	-	-	-	-	-	-	-	-	-	161971	-	-	-	-	-	-	-	-	-	-	-	-	-	-	-	-	-	-	-	-	-	-	-	-	-	-	-	-	-	-	-	-	-	-	-	-	-	-
SMT1		-	+	-	-	-	-	-	AT1G20330.1	59110	BDEG_01565	CE29973	193200	161904	-	-	CMP275C	-	DDB0188166	-	-	-	-	-	-	LmjF36.2380	-	33702	81379	-	NCU03006	Os03g04340.1	18637	-	10824	86618	-	-	559888	RO3G_16049.1	YML008C	SPBC16E9.05	-	-	-	36203	-	-	-	-	Tb10.6k15.3820	UM03182.1
		-	+	-	-	-	-	-	-	18609	-	-	-	161874	-	-	CMO088C	-	-	-	-	-	-	-	-	-	-	35888	76076	237353	-	-	33498	-	41348	160611	-	-	597327	-	-	-	-	-	11.m00448	33480	-	-	-	-	-	UM05053.1
		-	-	-	-	-	-	-	-	-	-	-	-	161866	-	-	-	-	-	-	-	-	-	-	-	-	-	-	-	-	-	-	-	-	-	-	-	-	-	-	-	-	-	-	-	-	-	-	-	-	-	-
		-	-	-	-	-	-	-	-	-	-	-	-	161860	-	-	-	-	-	-	-	-	-	-	-	-	-	-	-	-	-	-	-	-	-	-	-	-	-	-	-	-	-	-	-	-	-	-	-	-	-	-
		-	-	-	-	-	-	-	-	-	-	-	-	161832	-	-	-	-	-	-	-	-	-	-	-	-	-	-	-	-	-	-	-	-	-	-	-	-	-	-	-	-	-	-	-	-	-	-	-	-	-	-
		-	-	-	-	-	-	-	-	-	-	-	-	161829	-	-	-	-	-	-	-	-	-	-	-	-	-	-	-	-	-	-	-	-	-	-	-	-	-	-	-	-	-	-	-	-	-	-	-	-	-	-
		-	+	-	-	-	-	-	AT3G19910.1	52667	BDEG_03759	-	-	161785	-	cgd4_4310	-	-	-	-	-	-	-	GL50803_9155	NP_689683.2	LmjF23.1730	88425	3794	79281	-	NCU06815	Os09g36460.1	35225	GSPATP00024197001	-	135034	-	PFC0610c	551425	-	-	SPBP4H10.07	-	-	67.m00222	32557	-	55.m04967	-	85686.m00140	Tb927.8.3460	-
		-	-	-	-	-	-	-	-	-	-	-	-	161782	-	-	-	-	-	-	-	-	-	-	-	-	-	-	-	-	-	-	-	-	-	-	-	-	-	-	-	-	-	-	-	-	-	-	-	-	-	-
		-	+	-	-	-	-	-	AT4G09620.1	-	-	-	-	161769	-	-	-	-	-	-	-	-	-	-	-	-	-	-	-	-	-	Os03g62240.1	35716	-	-	116483	-	-	592231	-	-	-	-	-	-	-	-	-	-	-	-	-
		-	-	-	-	-	-	-	-	-	-	-	-	-	-	-	-	-	-	-	-	-	-	-	-	-	-	-	-	-	-	-	-	-	-	-	-	-	-	-	-	-	-	-	-	-	-	-	-	-	-	-
		-	-	-	-	-	-	-	-	-	-	-	-	161656	-	-	-	-	-	-	-	-	-	-	-	-	-	-	-	-	-	-	-	-	-	-	-	-	-	-	-	-	-	-	-	-	-	-	-	-	-	-
		-	-	-	-	-	-	-	-	-	-	-	-	161603	-	-	-	-	-	-	-	-	-	-	-	-	-	-	-	-	-	-	-	-	-	-	-	-	-	-	-	-	-	-	-	-	-	-	-	-	-	-
		-	-	-	-	-	-	-	-	-	-	-	-	-	-	-	-	-	-	-	-	-	-	-	-	-	-	-	-	-	-	-	-	-	-	-	-	-	-	-	-	-	-	-	-	-	-	-	-	-	-	-
		-	-	-	-	-	-	-	-	-	-	-	-	161546	-	-	-	-	-	-	-	-	-	-	-	-	-	-	-	-	-	-	-	-	-	-	-	-	-	-	-	-	-	-	-	-	-	-	-	-	-	-
		-	-	-	-	-	-	-	-	-	-	-	-	161528	-	-	-	-	-	-	-	-	-	-	-	-	-	-	-	-	-	-	-	-	-	-	-	-	-	-	-	-	-	-	-	-	-	-	-	-	-	-
		-	+	-	-	-	-	-	AT2G39725.2	-	-	-	92308	161525	-	-	-	-	DDB0188439	-	-	-	-	-	-	LmjF18.1200	-	-	-	83142	NCU07507	Os08g17650.2	-	GSPATP00033245001	-	138082	-	-	178680	-	YDR379C-A	SPAC664.12c	-	SINFRUP00000180583	-	-	-	-	-	-	Tb10.389.0620	UM04300.1
		-	-	-	-	-	-	-	-	-	-	-	-	161497	-	-	-	-	-	-	-	-	-	-	-	-	-	-	-	-	-	-	-	-	-	-	-	-	-	-	-	-	-	-	-	-	-	-	-	-	-	-
		-	-	-	-	-	-	-	-	-	-	-	-	161469	-	-	-	-	-	-	-	-	-	-	-	-	-	-	-	-	-	-	-	-	-	-	-	-	-	-	-	-	-	-	-	-	-	-	-	-	-	-
ASK1	aspartate kinase, monofunctional, putative	-	+	-	-	-	-	-	AT5G13280.1	52842	BDEG_06413	-	-	161455	-	-	CMJ016C	-	-	-	-	-	-	-	-	-	-	22547	-	224979	NCU04118	Os07g20544.2	15092	-	52316	140062	-	-	246339	RO3G_04565.1	YER052C	SPBC19F5.04	-	-	-	263424	-	42.m00124	62879	-	-	UM00872.1
		-	-	-	-	-	-	-	-	-	-	-	-	161441	-	-	-	-	-	-	-	-	-	-	-	-	-	-	-	-	-	-	-	-	-	-	-	-	-	-	-	-	-	-	-	-	-	-	-	-	-	-
LCIB	low-CO2-inducible protein	-	-	-	-	-	-	-	-	-	-	-	-	161422	-	-	-	-	-	-	-	-	-	-	-	-	-	-	-	-	-	-	34478	-	-	-	-	-	-	-	-	-	-	-	-	-	-	-	-	-	-	-
		-	-	-	-	-	-	-	-	-	-	-	-	161384	-	-	-	-	-	-	-	-	-	-	-	-	-	-	-	-	-	-	-	-	-	-	-	-	-	-	-	-	-	-	-	-	-	-	-	-	-	-
		-	-	-	-	-	-	-	-	-	-	-	-	161248	-	-	-	-	-	-	-	-	-	-	-	-	-	-	-	-	-	-	-	-	-	-	-	-	567813	-	-	-	-	-	-	-	-	-	-	-	-	-
		-	-	-	-	-	-	-	-	-	-	-	-	161193	-	-	-	-	-	-	-	-	-	-	-	-	-	-	-	-	-	-	-	-	-	-	-	-	-	-	-	-	-	-	-	-	-	-	-	-	-	-
		-	-	-	-	-	-	-	-	-	-	-	-	161171	-	-	-	-	-	-	-	-	-	-	-	-	-	-	-	-	-	-	-	-	-	-	-	-	-	-	-	-	-	-	-	-	-	-	-	-	-	-
		-	-	-	-	-	-	-	-	-	-	-	-	161127	-	-	-	-	-	-	-	-	-	-	-	-	-	-	-	-	-	-	-	-	-	-	-	-	-	-	-	-	-	-	-	-	-	-	-	-	-	-
		-	-	-	-	-	-	-	-	-	-	-	-	161105	-	-	-	-	-	-	-	-	-	-	-	-	-	-	-	-	-	-	-	-	-	-	-	-	-	-	-	-	-	-	-	-	-	-	-	-	-	-
		-	-	-	-	-	-	-	-	-	-	-	-	161093	-	-	-	-	-	-	-	-	-	-	-	-	-	-	-	-	-	-	-	-	-	-	-	-	-	-	-	-	-	-	-	-	-	-	-	-	-	-
PGM1b	Phosphoglycerate mutase, possible cytosolic form	-	+	-	-	-	-	-	AT1G09780.1	-	BDEG_04539	CE33113	45833	161085	-	-	CMK188C	-	-	-	19069357	60.m00168	-	GL50803_8822	-	LmjF36.6650	-	-	-	183697	NCU02252	Os03g21260.1	34354	-	-	169645	-	-	825441	RO3G_06292.1	-	-	GLEAN3_10377	-	-	-	-	-	32533	86587.m00076	Tb10.6k15.2620	-
		-	+	-	-	-	-	GB10283-PA	-	-	-	-	208996	161043	-	-	-	-	-	CG7225-PA	-	-	ENSGALP00000038209	-	OTTHUMP00000169271	-	214161	30030	-	-	-	-	-	-	-	-	-	-	-	-	-	-	-	-	-	-	-	-	-	-	-	-
		-	-	-	-	-	-	-	-	-	-	-	-	161040	-	-	-	-	-	-	-	-	-	-	-	-	-	-	-	-	-	-	-	-	-	-	-	-	-	-	-	-	-	-	-	-	-	-	-	-	-	-
		-	-	-	-	-	-	-	-	-	-	-	-	161039	-	-	-	-	-	-	-	-	-	-	-	-	-	-	-	-	-	-	-	-	-	-	-	-	-	-	-	-	-	-	-	-	-	-	-	-	-	-
		-	-	-	-	-	-	-	-	-	-	-	-	160987	-	-	-	-	-	-	-	-	-	-	-	-	-	-	-	-	-	-	-	-	-	-	-	-	-	-	-	-	-	-	-	-	-	-	-	-	-	-
		-	-	-	-	-	-	-	-	-	-	-	-	160970	-	-	-	-	-	-	-	-	-	-	-	-	-	-	-	-	-	-	-	-	-	-	-	-	-	-	-	-	-	-	-	-	-	-	-	-	-	-
		-	-	-	-	-	-	-	-	-	-	-	-	160921	-	-	-	-	-	-	-	-	-	-	-	-	-	-	-	-	-	-	-	-	-	-	-	-	-	-	-	-	-	-	-	-	-	-	-	-	-	-
		-	-	-	-	-	-	-	-	-	-	-	-	160895	-	-	-	-	-	-	-	-	-	-	-	-	-	-	-	-	-	-	-	-	-	-	-	-	658248	-	-	-	-	-	-	-	-	-	-	-	-	-
		-	-	-	-	-	-	-	-	-	-	-	-	160872	-	-	-	-	-	-	-	-	-	-	-	-	-	-	-	-	-	-	-	-	-	-	-	-	-	-	-	-	-	SINFRUP00000143006	-	-	-	-	-	-	-	-
		-	-	-	-	-	-	-	-	-	-	-	-	160820	-	-	-	-	-	-	-	-	-	-	-	-	-	-	-	-	-	-	-	-	-	-	-	-	-	-	-	-	-	-	-	-	-	-	-	-	-	-
		-	-	-	-	-	-	-	-	-	-	-	-	160790	-	-	-	-	-	-	-	-	-	-	-	-	-	-	-	-	-	-	-	-	-	-	-	-	-	-	-	-	-	-	-	-	-	-	-	-	-	-
PRX4	Peroxiredoxin, type II, probably targeted to mitochondria or chloroplast	-	-	-	-	-	-	-	-	-	-	-	-	160787	-	-	-	-	-	-	-	-	-	-	-	-	-	-	-	-	-	-	-	-	-	-	-	-	-	-	-	-	-	-	-	-	-	-	-	-	-	-
		-	-	-	-	-	-	-	-	-	-	-	-	160784	-	-	-	-	-	-	-	-	-	-	-	-	-	-	-	-	-	-	-	-	-	-	-	-	-	-	-	-	-	-	-	-	-	-	-	-	-	-
		-	-	-	-	-	-	-	-	-	-	-	-	160761	-	-	-	-	-	-	-	-	-	-	-	-	-	-	-	-	-	-	-	-	34403	-	-	-	-	-	-	-	-	-	-	-	-	-	-	-	-	-
		-	-	-	-	-	-	-	-	-	-	-	-	160760	-	-	-	-	-	-	-	-	-	-	-	-	-	-	-	-	-	-	-	-	-	-	-	-	-	-	-	-	-	-	-	-	-	-	-	-	-	-
		-	-	-	-	-	-	-	-	-	-	-	-	160735	-	-	-	-	-	-	-	-	-	-	-	-	-	-	-	-	-	-	-	-	-	-	-	-	827043	-	-	-	-	-	-	-	-	-	-	-	-	-
		-	-	-	-	-	-	-	-	-	-	-	-	160684	-	-	-	-	-	-	-	-	-	-	-	-	-	-	-	-	-	-	-	-	-	-	-	-	-	-	-	-	-	-	-	-	-	-	-	-	-	-
OAT1		-	-	-	-	-	-	-	-	-	-	-	-	943	-	-	-	-	-	-	-	-	ENSGALP00000037866	-	OTTHUMP00000020690	-	-	-	-	159041	-	-	-	-	-	-	-	-	-	-	-	-	-	-	-	-	-	-	-	-	-	-
HTR47	Histone H3	-	-	-	-	-	-	-	-	-	-	-	-	-	-	-	-	-	-	-	-	-	-	-	-	-	-	-	-	-	-	-	-	-	-	-	-	-	-	-	-	-	-	-	-	-	-	-	-	-	-	-
HTB31	Histone H2B	-	-	-	-	-	-	-	-	-	-	-	-	-	-	-	-	-	-	-	-	-	-	-	-	-	-	-	-	-	-	-	-	-	-	-	-	-	-	-	-	-	-	-	-	-	-	-	-	-	-	-
HTA30	Histone H2A	-	-	-	-	-	-	-	-	-	-	-	-	-	-	-	-	-	-	-	-	-	-	-	-	-	-	-	-	-	-	-	-	-	-	-	-	-	-	-	-	-	-	-	-	-	-	-	-	-	-	-
HTR3	Histone H3	-	-	-	-	-	-	-	-	-	-	-	-	-	-	-	-	-	-	-	-	-	-	-	-	-	-	-	-	-	-	-	-	-	-	-	-	-	-	-	-	-	-	-	-	-	-	-	-	-	-	-
HTR29	Histone H3	-	-	-	-	-	-	-	-	-	-	-	-	-	-	-	-	-	-	-	-	-	-	-	-	-	-	-	-	-	-	-	-	-	-	-	-	-	-	-	-	-	-	-	-	-	-	-	-	-	-	-
HTR45	Histone H3	-	-	-	-	-	-	-	-	-	-	-	-	-	-	-	-	-	-	-	-	-	-	-	-	-	-	-	-	-	-	-	-	-	-	-	-	-	-	-	-	-	-	-	-	-	-	-	-	-	-	-
HTA28	Histone H2A	-	-	-	-	-	-	-	-	-	-	-	-	-	-	-	-	-	-	-	-	-	-	-	-	-	-	-	-	-	-	-	-	-	-	-	-	-	-	-	-	-	-	-	-	-	-	-	-	-	-	-

HTR27	Histone H3	-	-	-	-	-	-	-	-	-	-	-	-	-	-	-	-	-	-	-	-	-	-	-	-	-	-	-	-	-	-	-	-	-	-	-	-	-	-	-	-	-	-	-	-	-	-	-	-	-	-	-
HTR26	Histone H3	-	-	-	-	-	-	-	-	-	-	-	-	-	-	-	-	-	-	-	-	-	-	-	-	-	-	-	-	-	-	-	-	-	-	-	-	-	-	-	-	-	-	-	-	-	-	-	-	-	-	-
PP2A-2c	Protein Phosphatase 2A catalytic subunit Homolog 2	-	-	-	-	-	-	-	-	-	-	-	-	-	-	-	-	-	-	-	-	-	-	-	-	-	-	-	-	-	-	-	-	-	-	-	-	-	-	-	-	-	-	-	-	-	-	-	-	-	-	-
HTA24	Histone H2A	-	-	-	-	-	-	-	-	-	-	-	-	-	-	-	-	-	-	-	-	-	-	-	-	-	-	-	-	-	-	-	-	-	-	-	-	-	-	-	-	-	-	-	-	-	-	-	-	-	-	-
HTR24	Histone H3	-	-	-	-	-	-	-	-	-	-	-	-	-	-	-	-	-	-	-	-	-	-	-	-	-	-	-	-	-	-	-	-	-	-	-	-	-	-	-	-	-	-	-	-	-	-	-	-	-	-	-
HFO43	Histone H4	-	-	-	-	-	-	-	-	-	-	-	-	796	-	-	-	-	-	-	-	-	-	-	-	-	-	-	-	-	-	-	-	-	-	-	-	-	-	-	-	-	-	-	-	-	-	-	-	-	-	-
HTR43	Histone H3	-	-	-	-	-	-	-	-	-	-	-	-	795	-	-	-	-	-	-	-	-	-	-	-	-	176498	-	-	220974	-	-	-	-	-	-	-	-	-	-	-	-	-	-	-	-	-	-	-	-	-	-
HTA23	Histone H2A	-	-	-	-	-	-	-	-	-	-	-	-	-	-	-	-	-	-	-	-	-	-	-	-	-	-	-	-	-	-	-	-	-	-	-	-	-	-	-	-	-	-	-	-	-	-	-	-	-	-	-
HTA42	Histone H2A	-	+	-	-	-	-	-	-	58825	-	-	-	702	-	-	CMN174C	-	-	-	-	117.m00168	-	-	-	-	-	-	-	-	-	Os12g34510.1	-	-	-	-	-	-	-	-	-	-	-	-	-	-	TA07845	-	-	-	-	-
		-	+	+	-	-	-	GB13168-PA	AT2G18600.1	53859	BDEG_03491	-	149080	692	204809	-	CME162C	OTTDARP00000022355	DDB0204633	CG7375-PA	-	98.m00134	ENSGALP00000006035	-	OTTHUMP00000068137	-	228262	35275	80939	171977	NCU03610	Os08g28680.1	8373	GSPATP00025354001	48810	214589	144101	PFL2175w	827109	RO3G_15307.1	YLR306W	SPCC777.10c	-	SINFRUP00000159413	68.m00229	21129	-	72.m00407	32811	81617.m00226	-	UM02848.1
HTA22	Histone H2A	-	-	-	-	-	-	-	-	-	-	-	-	-	-	-	-	-	-	-	-	-	-	-	-	-	-	-	-	-	-	-	-	-	-	-	-	-	-	-	-	-	-	-	-	-	-	-	-	-	-	-
HTB22	Histone H2B	-	-	-	-	-	-	-	-	-	-	-	-	-	-	-	-	-	-	-	-	-	-	-	-	-	-	-	-	-	-	-	-	-	-	-	-	-	-	-	-	-	-	-	-	-	-	-	-	-	-	-
HFO22	Histone H4	-	-	-	-	-	-	-	-	-	-	-	-	-	-	-	-	-	-	-	-	-	-	-	-	-	-	-	-	-	-	-	-	-	-	-	-	-	-	-	-	-	-	-	-	-	-	-	-	-	-	-
HTB21	Histone H2B	-	-	-	-	-	-	GB13012-PA	-	-	-	-	-	648	-	-	-	-	-	-	-	-	-	-	-	-	-	-	-	-	-	-	-	-	-	-	-	-	-	-	-	-	-	-	-	-	-	-	-	-	-	-
HTA21	Histone H2A	-	-	-	-	-	-	-	-	-	-	-	-	-	-	-	-	-	-	-	-	-	-	-	-	-	-	-	-	-	-	-	-	-	-	-	-	-	-	-	-	-	-	-	-	-	-	-	-	-	-	-
HTA1	Histone H2A	-	-	-	-	-	-	-	-	-	-	-	-	-	-	-	-	-	-	-	-	-	-	-	-	-	-	-	-	-	-	-	-	-	-	-	-	-	-	-	-	-	-	-	-	-	-	-	-	-	-	-
HTR1	Histone H3	-	-	-	-	-	-	-	-	-	-	-	-	-	-	-	-	-	-	-	-	-	-	-	-	-	-	-	-	-	-	-	-	-	-	-	-	-	-	-	-	-	-	-	-	-	-	-	-	-	-	-
EIF42		-	+	+	-	-	-	GB13469-PA	AT3G19760.1	58937	BDEG_07770	CE09901	155818	608	298564	-	-	-	DDB0201848	CG7483-PA	19171365	5.m00474	ENSGALP00000038179	-	OTTHUMP00000181437	LmjF28.1530	225283	21732	44232	236387	NCU01234	Os03g36930.1	20289	GSPATP00029794001	41785	60709	109154	PFD1070w	832316	RO3G_05719.1	YDR021W	SPAC1F5.10	GLEAN3_14808	SINFRUP00000178910	18.m00429	354	TA06920	55.m04649	38398	-	Tb11.12.0011	UM06129.1
		-	+	+	-	-	-	GB10614-PA	AT3G55360.1	64002	BDEG_06952	CE20509	167372	602	237788	cgd2_1200	CMD146C	OTTDARP00000022390	DDB0190926	CG10849-PA	-	40.m00219	ENSGALP00000009263	GL50803_88581	OTTHUMP00000067432	LmjF25.1770	232335	-	33241	147144	NCU03362	Os01g05670.1	16084	GSPATP00012181001	-	203665	-	PF11_0370	720621	RO3G_01177.1	YDL015C	SPBC646.07c	-	SINFRUP00000149423	43.m00257	33911	TA18840	76.m00008	29423	88043.m00118	Tb927.3.1840	UM03622.1
IF2Ba		-	+	+	-	-	-	GB11975-PA	AT1G72340.1	35783	-	CE00367	222112	596	207008	-	CMK064C	-	DDB0219393	-	-	6.m00436	ENSGALP00000005151	GL50803_91911	OTTHUMP00000169733	LmjF12.0010	187625	19405	82768	246207	NCU04344	Os12g31380.1	-	GSPATP00015208001	10674	185227	133014	PF08_0009	584158	RO3G_06936.1	YKR026C	SPCC11E10.07c	-	SINFRUP00000136413	132.m00093	264824	-	27.m00082	20160	84270.m00730	Tb927.6.1280	UM03171.1
		-	+	-	-	-	-	-	AT5G53580.1	20069	-	-	-	582	-	-	-	-	-	-	-	-	-	-	-	-	-	-	-	-	-	Os10g37330.1	35050	-	47510	74042	-	-	250752	RO3G_11815.1	-	-	-	-	-	261284	-	-	-	-	-	-
HTR41	Histone H3	-	-	-	-	-	-	-	-	-	-	-	-	581	-	-	-	-	-	-	-	-	-	-	-	-	-	-	-	-	-	-	-	-	-	-	-	-	-	-	-	-	-	-	-	-	-	-	-	-	-	-
HTR40	Histone H3	-	-	-	-	-	-	-	-	-	-	-	-	-	-	-	-	-	-	-	-	-	-	-	-	-	-	-	-	-	-	-	-	-	-	-	-	-	-	-	-	-	-	-	-	-	-	-	-	-	-	-
HFO40	Histone H4	-	-	-	-	-	-	-	-	-	-	-	-	-	-	-	-	-	-	-	-	-	-	-	-	-	-	-	-	-	-	-	-	-	-	-	-	-	-	-	-	-	-	-	-	-	-	-	-	-	-	-
MLP	mastigoneme-like flagellar protein	-	-	-	+	-	-	-	-	-	-	-	-	574	-	-	-	-	-	-	-	-	ENSGALP00000025304	-	-	-	-	-	-	-	-	-	-	-	-	-	-	-	-	-	-	-	-	-	-	-	-	-	-	-	-	-
HFV1	Histone H4 variant	-	-	-	-	-	-	-	-	-	-	-	-	555	-	-	-	-	-	-	-	-	-	-	-	-	-	-	-	-	-	-	-	-	-	-	-	-	-	-	-	-	-	-	-	-	-	-	-	-	-	-
RFC5	DNA replication factor C complex subunit 5	-	+	+	-	-	-	GB19646-PA	AT1G77470.1	71065	BDEG_00681	CE37518	164264	519	-	cgd3_3170	CMO145C	-	DDB0205283	CG5313-PA	19068592	173.m00114	-	GL50803_24636	OTTHUMP00000169407	LmjF35.3250	152961	30540	29498	166542	NCU08427	Os02g53500.2	25075	GSPATP00036550001	11922	136893	109673	PF14_0601	816262	RO3G_03308.1	YNL290W	SPAC27E2.10c	-	SINFRUP00000164364	17.m00449	723	TA08130	31.m00866	-	95725.m00217	Tb09.211.3310	UM04623.1
STA2	Putative granule-bound starch synthase I	-	+	-	-	-	-	-	AT1G32900.1	-	-	-	-	503	-	-	-	-	-	-	-	-	-	-	-	-	-	-	-	-	NCU08132	Os06g04200.4	28387	-	-	120571	-	-	815839	-	-	SPCC1281.01	-	-	-	-	-	-	-	-	-	-
	isoamylase, starch debranching enzyme	-	+	-	-	-	-	-	AT2G39930.1	-	-	-	178960	496	-	-	CMS197C	-	-	-	-	-	-	-	-	-	-	-	-	-	-	Os08g40930.1	21174	-	-	179583	-	-	560622	-	-	-	-	-	-	-	-	-	-	82181.m00192	-	-
		-	+	-	-	-	-	-	AT2G32480.1	-	-	-	-	458	-	-	CMR030C	-	-	-	-	-	-	-	-	-	-	-	-	-	-	Os03g38230.1	33335	-	48723	144310	-	-	408975	-	-	-	-	-	-	261878	-	-	-	-	-	-
LZY1B	unknown protein; zygote-specific	-	-	-	-	-	-	-	-	-	-	-	-	449	-	-	-	-	-	-	-	-	-	-	-	-	-	-	-	-	-	-	-	-	-	-	-	-	-	RO3G_01065.1	-	-	-	-	-	-	-	-	-	-	-	-
PBA1		-	+	-	-	-	-	-	AT4G31300.1	-	BDEG_01049	-	156053	415	285384	-	CMH154C	-	DDB0189428	CG8392-PA	19069396	-	-	GL50803_9824	OTTHUMP00000182726	LmjF12.0030	153858	35094	60110	101124	NCU09290	Os06g04800.1	-	-	51691	112163	108552	-	673509	RO3G_05850.1	YJL001W	SPBC4C3.10c	-	-	2.m02218	33649	TA13865	-	37861	-	Tb927.6.1260	UM01275.1
PP2A-c3		-	-	-	-	-	-	-	-	-	-	-	-	399	-	-	-	-	-	-	-	-	-	-	-	-	-	-	-	-	-	-	-	-	-	-	-	-	-	-	-	-	-	-	-	-	-	-	-	-	-	-
RPS27B	Ribosomal protein S27 isoform B, component of cytosolic 80S ribosome and 40S small subunit	-	-	-	-	-	-	-	-	-	-	-	-	386	-	-	-	-	-	-	-	-	-	-	-	-	-	-	-	-	-	-	-	GSPATP00025430001	-	-	-	-	-	-	-	-	-	-	-	-	-	-	-	-	-	-
		-	+	+	-	-	-	GB15349-PA	AT2G45790.1	29117	BDEG_03263	CE36163	221704	350	297171	cgd4_960	CMT314C	OTTDARP00000019840	DDB0205701	CG10688-PA	19170768	26.m00333	ENSGALP00000011838	-	OTTHUMP00000081384	LmjF36.1960	171717	20131	31260	159761	NCU02829	Os04g58580.1	23158	GSPATP00014735001	28882	234483	109063	PF10_0169	823949	RO3G_07083.1	YFL045C	SPAC1556.07	GLEAN3_14312	SINFRUP00000178087	4.m00521	22301	TA07020	49.m03167	52968	84025.m00020	Tb10.70.0370	UM04703.1
HTA38	Histone H2A	-	+	-	-	-	-	-	-	-	-	-	137819	333	-	-	-	-	-	-	19068634	-	ENSGALP00000037264	-	OTTHUMP00000033967	-	-	-	-	-	-	-	-	GSPATP00034423001	-	-	-	-	729700	-	-	-	-	-	-	19793	-	55.m04926	-	-	-	-
DNJ7	DnaJ-like protein 7, probably cytosolic	-	-	-	-	-	-	-	-	-	BDEG_02052	-	-	298	-	-	CMO101C	-	-	-	-	-	-	-	-	-	-	-	-	-	-	-	-	-	-	-	-	-	-	-	YNL007C	SPCC830.07c	-	-	-	-	-	-	-	-	-	-
		-	+	+	-	-	-	GB18534-PA	AT5G27470.1	23294	BDEG_07581	CE05445	157579	265	283474	cgd8_4720	CMN178C	OTTDARP00000022130	DDB0168235	CG17259-PA	19068794	204.m00091	ENSGALP00000000739	GL50803_101501	OTTHUMP00000013432	LmjF11.0100	190601	37346	33055	239510	NCU01443	Os03g10190.1	24003	GSPATP00009630001	30572	115448	108754	PF07_0073	716082	RO3G_10204.1	YDR023W	SPAC29A4.15	-	SINFRUP00000128308	8.m00411	269399	TA03465	50.m00020	25588	90487.m00096	Tb11.02.5020	UM00865.1
RPL27	Ribosomal protein L27, component of cytosolic 80S ribosome and 60S large subunit	-	+	+	-	-	-	GB15660-PA	AT4G15000.1	8341	BDEG_07561	CE19381	151982	260	391069	cgd1_3000	CML305C	OTTDARP00000020383	DDB0202845	-	19068752	56.m00173	ENSGALP00000004469	GL50803_8462	OTTHUMP00000181240	LmjF32.2710	141455	-	58185	234712	NCU01827	Os10g41470.1	23676	GSPATP00009654001	17846	187092	156776	PF14_0579	560241	RO3G_04237.1	YDR471W	SPCC74.05	GLEAN3_19788	SINFRUP00000137482	130.m00118	30154	TA14565	55.m05006	63098	86603.m00081	Tb11.01.7545	UM05189.1
HTR36	Histone H3	-	-	-	-	-	-	-	-	-	-	-	-	-	-	-	-	-	-	-	-	-	-	-	-	-	-	-	-	-	-	-	-	-	-	-	-	-	-	-	-	-	-	-	-	-	-	-	-	-	-	-
HTA3	Histone H2A	-	-	-	-	-	-	-	-	-	-	-	-	-	-	-	-	-	-	-	-	-	-	-	-	-	-	-	-	-	-	-	-	-	-	-	-	-	-	-	-	-	-	-	-	-	-	-	-	-	-	-
HTA19	Histone H2A	-	-	-	-	-	-	-	-	-	-	-	-	-	-	-	-	-	-	-	-	-	-	-	-	-	-	-	-	-	-	-	-	-	-	-	-	-	-	-	-	-	-	-	-	-	-	-	-	-	-	-
HTR19	Histone H3	-	-	-	-	-	-	-	-	-	-	-	-	-	-	-	-	-	-	-	-	-	-	-	-	-	-	-	-	-	-	-	-	-	-	-	-	-	-	-	-	-	-	-	-	-	-	-	-	-	-	-
		-	+	+	-	-	-	GB17141-PA	AT5G60990.1	2416	BDEG_02297	CE00337	175937	223	207859	cgd4_3180	CMK083C	-	DDB0206406	CG9253-PA	19069295	54.m00208	ENSGALP00000019220	GL50803_9119	OTTHUMP00000166740	LmjF36.1850	229010	37739	1378	192845	NCU04504	Os03g46610.1	24293	GSPATP00021562001	28984	178010	108838	PFB0860c	218296	RO3G_03957.1	YHR065C	SPAC823.08c	GLEAN3_04912	SINFRUP00000166748	7.m00301	14597	TA14795	69.m00134	21567	96713.m00134	Tb10.70.0540	UM05214.1
HTR16	Histone H3	-	-	-	-	-	-	-	-	-	-	-	-	-	-	-	-	-	-	-	-	-	-	-	-	-	-	-	-	-	-	-	-	-	-	-	-	-	-	-	-	-	-	-	-	-	-	-	-	-	-	-
HTA2	Histone H2A	-	-	-	-	-	-	-	-	-	-	-	-	-	-	-	-	-	-	-	-	-	-	-	-	-	-	-	-	-	-	-	-	-	-	-	-	-	-	-	-	-	-	-	-	-	-	-	-	-	-	-
HTR2	Histone H3	-	-	-	-	-	-	-	-	-	-	-	-	-	-	-	-	-	-	-	-	-	-	-	-	-	-	-	-	-	-	-	-	-	-	-	-	-	-	-	-	-	-	-	-	-	-	-	-	-	-	-
ERD2B	KDEL Receptor B	-	+	-	-	-	-	-	AT1G19970.1	-	-	-	-	176	-	-	-	-	-	-	-	-	-	-	-	-	-	-	-	-	-	Os08g43470.1	17394	-	-	220428	-	MAL13P1.163	818156	-	-	-	-	-	90.m00152	-	TA04030	-	-	-	-	-
		-	+	-	-	-	-	GB16965-PA	-	28494	BDEG_03411	-	147986	158	-	-	-	-	-	-	-	-	-	-	NP_036226.1	-	169267	39400	35558	211830	-	-	-	-	22388	-	-	MAL7P1.159	-	RO3G_08417.1	YLR109W	-	-	SINFRUP00000138845	-	36078	-	76.m01670	18263	-	-	-
HTR10	Histone H3	-	-	-	-	-	-	-	-	-	-	-	-	-	-	-	-	-	-	-	-	-	-	-	-	-	-	-	-	-	-	-	-	-	-	-	-	-	-	-	-	-	-	-	-	-	-	-	-	-	-	-
TGD1		-	+	-	-	-	-	-	AT1G19800.1	-	-	-	123240	83	-	-	CMV212C	-	-	-	-	-	-	-	-	-	-	-	-	-	-	Os04g46700.2	20413	-	-	127149	-	-	411553	-	-	-	-	-	-	-	-	-	-	-	-	-
HTA9	Histone H2A	-	+	-	-	-	-	GB18954-PA	AT1G51060.1	16990	BDEG_00706	CE05477	198869	82	298480	cgd8_2170	-	OTTDARP00000008273	DDB0205974	CG33865-PA	-	-	-	GL50803_27521	-	-	181153	22990	5305	177240	NCU02437	-	9126	-	26802	135118	158257	PFF0860c	-	RO3G_16446.1	YDR225W	SPAC19G12.06c	GLEAN3_02818	SINFRUP00000177254	31.m00263	-	-	-	64252	83866.m00026	-	UM01504.1
HTR6	Histone H3	-	-	-	-	-	-	-	-	-	-	-	-	-	-	-	-	-	-	-	-	-	-	-	-	-	-	-	-	-	-	-	-	-	-	-	-	-	-	-	-	-	-	-	-	-	-	-	-	-	-	-
HTB4	Histone H2B	-	+	-	-	-	-	-	-	60369	BDEG_00910	CE05165	132737	60	-	-	-	OTTDARP00000023516	-	CG33868-PA	-	-	-	-	OTTHUMP00000016184	-	-	-	-	228163	-	Os01g05990.1	-	GSPATP00025378001	11823	-	108813	PF11_0062	-	RO3G_16447.1	-	-	GLEAN3_01312	SINFRUP00000129663	90.m00169	37309	-	-	-	-	Tb10.406.0440	-
HON2	Histone H1	-	-	-	-	-	-	-	AT2G18050.1	-	-	-	-	58	-	-	-	-	-	-	-	-	-	-	-	-	-	-	53171	-	-	-	-	-	-	-	128470	-	-	-	-	-	-	-	-	-	-	-	53828	-	-	-
PYR2		-	+	-	-	-	-	-	AT3G20330.1	13348	-	-	-	31	264473	-	-	-	-	-	-	-	-	-	-	LmjF16.0540	-	-	74661	-	-	Os08g15030.1	9051	-	25183	146128	109166	MAL13P1.221	550113	-	-	-	GLEAN3_00851	-	-	34595	TA20650	80.m00005	-	-	Tb927.5.3820	-
PYK8	pyruvate kinase	-	-	-	-	-	-	-	-	-	-	-	-	23556	-	-	-	-	-	-	-	-	-	-	-	-	-	-	-	-	-	-	-	-	-	-	-	-	-	-	-	-	-	-	-	-	-	-	-	-	-	-
FMG1-1	flagella membrane glycoprotein	-	-	-	-	-	-	-	-	-	-	-	-	-	-	-	-	-	-	-	-	-	-	-	-	-	-	-	-	-	-	-	-	-	-	-	-	-	-	-	-	-	-	-	-	-	-	-	-	-	-	-
EZY2	zygote-specific protein	-	-	-	-	-	-	-	-	-	-	-	-	23466	-	-	-	-	-	-	-	-	-	-	-	-	-	-	-	-	-	-	-	-	-	-	-	-	-	-	-	-	-	-	-	-	-	-	-	-	-	-
GTR23	glycosyl transferase	-	-	-	-	-	-	-	-	-	-	-	-	23032	-	-	-	-	-	-	-	-	-	-	-	-	-	-	-	-	-	-	-	-	-	-	-	-	-	-	-	-	-	-	-	-	-	-	-	-	-	-
CLR3	Coxiella-Like Repeat protein	-	-	-	-	-	-	-	-	-	-	-	-	22494	-	-	-	-	-	-	-	-	-	-	-	-	-	-	-	-	-	-	-	-	-	-	-	-	-	-	-	-	-	-	-	-	-	-	-	-	-	-
CAO,SRP43	Chloroplast SRP43 Subunit of Signal Recognition Particle	-	+	-	-	-	-	-	AT2G47450.1	-	-	-	-	22212	-	-	-	-	-	-	-	-	-	-	-	-	-	-	-	-	-	Os03g03990.1	31046	-	-	15522	-	-	557028	-	-	-	-	-	-	-	-	-	-	-	-	-
	Expressed potential RNA-binding protein.	-	+	-	-	-	-	-	AT5G06770.1	-	-	CE18657	-	22211	-	cgd6_1330	CMA121C	-	-	-	-	-	-	-	-	-	-	-	-	-	-	Os06g41384.1	37617	-	50624	162478	-	-	829302	-	-	-	-	-	12.m00381	-	-	50.m03220	-	-	Tb927.5.1580	UM01649.1
DNJ16	DnaJ-like protein 16	-	-	-	-	-	-	-	-	-	-	-	-	22090	-	-	-	-	-	-	-	-	-	-	-	-	-	-	-	-	-	-	-	-	-	-	-	-	-	-	-	-	-	-	-	-	-	-	-	-	-	-
CLR10	Coxiella-Like Repeat protein	-	-	-	-	-	-	-	AT5G66090.1	-	-	-	-	21996	-	-	-	-	-	-	-	-	-	-	-	-	-	-	-	-	-	-	-	-	-	144824	-	-	593803	-	-	-	-	-	-	-	-	-	-	-	-	-
	putative protein containing an engulfment and cell motility domain	-	+	-	-	-	-	GB12569-PA	AT1G03620.1	-	-	-	228466	21823	299311	-	-	-	DDB0204388	-	-	-	ENSGALP00000011711	-	OTTHUMP00000025319	-	-	-	-	171873	-	Os11g03530.1	16031	-	-	105068	134705	-	836403	-	-	-	-	SINFRUP00000164773	-	-	-	-	55703	-	-	-
ARM2	protein of unknown function, contains armadillo (Arm) repeat	+	+	-	-	-	-	-	-	65322	-	-	119092	21780	-	-	-	OTTDARP00000009017	-	CG32668-PA	-	-	ENSGALP00000024631	-	NP_115507.4	LmjF33.2030	140035	22668	49917	10901	-	-	-	GSPATP00025847001	-	-	129716	-	-	-	-	-	-	-	131.m00109	-	-	-	26025	-	Tb11.02.0470	-
SET4	SET domain containing protein, putative histone methyltransferase	-	+	-	-	-	-	GB12139-PA	-	-	-	-	106550	21717	-	-	-	OTTDARP00000020948	-	-	-	-	ENSGALP00000016588	-	NP_955352.1	-	53010	26877	-	-	NCU00045	-	-	-	-	-	-	-	-	RO3G_03266.1	-	-	-	SINFRUP00000147324	-	-	-	-	-	-	-	UM06029.1
GTR17	glycosyl transferase	-	+	-	-	-	-	-	AT3G06440.2	-	-	-	-	21670	264003	-	-	-	-	CG8673-PA	-	-	ENSGALP00000002874	-	-	-	138436	23896	-	175792	-	-	-	-	-	146959	134408	-	416975	-	-	-	GLEAN3_20773	SINFRUP00000148338	-	-	-	-	4567	-	-	-
HXK1	hexokinase	-	+	-	-	-	-	GB19387-PA	AT1G47840.1	-	BDEG_04378	CE05628	18168	21582	287325	cgd6_3800	-	OTTDARP00000024119	-	CG3001-PA	-	19.m00296	ENSGALP00000039963	-	NP_000179.1	LmjF21.0250	124707	-	-	229061	NCU02542	Os05g45590.1	-	-	-	115056	-	PFF1155w	797614	RO3G_01514.1	YCL040W	SPAC24H6.04	GLEAN3_20435	SINFRUP00000132593	-	-	TA19800	57.m00001	50939	-	Tb10.70.5820	UM02173.1
	Short-chain dehydrogenase/reductase	-	-	-	-	-	-	-	-	-	-	-	-	21459	-	-	-	-	-	-	-	-	-	-	-	-	-	-	-	-	-	-	-	-	-	-	-	-	-	-	-	-	-	-	-	-	-	-	-	-	-	-
VPS11	subunit of VPS-C complex	-	+	-	-	-	-	GB18493-PA	AT2G05170.1	-	BDEG_01258	CE01618	218778	21422	260122	cgd4_4010	-	-	DDB0204454	-	-	-	ENSGALP00000000392	GL50803_17109	NP_068375.3	LmjF31.2890	182193	10328	81916	208260	NCU05422	Os04g31390.1	-	GSPATP00006902001	43038	185907	130446	PFE0100w	247000	RO3G_06686.1	-	SPAC823.12	-	SINFRUP00000149663	273.m00021	24183	-	44.m05910	28801	98808.m00004	Tb927.8.7200	UM03820.1
PGM4	phosphoglycerate/bisphosphoglycerate mutase family protein	-	+	-	-	-	-	-	AT5G22620.1	-	-	-	-	21373	-	-	CMN311C	-	-	-	-	-	-	-	-	-	-	-	-	-	-	Os11g05260.1	18437	-	41063	124605	-	-	827189	-	YKR043C	-	-	-	-	263425	-	-	-	-	-	-
ELG30	exostosin-like glycosyltransferase	-	-	-	-	-	-	-	-	-	-	-	-	21356	-	-	-	-	-	-	-	-	-	-	-	-	-	-	-	-	-	-	-	-	-	-	-	-	-	-	-	-	-	-	-	-	-	-	-	-	-	-
ELG25	exostosin-like glycosyltransferase	-	-	-	-	-	-	-	-	-	-	CE35381	-	21310	-	-	-	-	-	-	-	-	ENSGALP00000013329	-	NP_005036.2	-	-	-	-	-	-	-	-	-	-	-	-	-	-	-	-	-	GLEAN3_23409	-	-	-	-	-	-	-	-	-
ORC5	Origin recognition complex subunit 5; DNA replication control protein.	-	-	-	-	-	-	-	-	-	-	-	-	21138	-	-	-	-	-	-	-	-	-	-	-	-	-	-	-	-	-	-	-	-	-	-	-	-	-	-	-	-	GLEAN3_18823	-	-	-	-	-	-	-	-	-
PFK3	phosphofructokinase	-	-	-	-	-	-	-	-	-	-	-	-	20801	-	-	-	-	-	-	-	-	-	-	-	-	-	-	-	-	-	-	-	-	-	-	-	-	-	-	-	-	-	-	-	-	-	-	-	-	-	-
TATB	TatB-like sec-independent protein translocon subunit	-	+	-	-	-	-	-	AT5G52440.1	-	-	-	-	20727	-	-	-	-	-	-	-	-	-	-	-	-	-	-	-	-	-	Os11g37130.1	7845	-	-	109121	-	-	734212	-	-	-	-	-	-	-	-	-	-	-	-	-
IRE1	key regulator in ER unfolded protein response	-	+	-	-	-	-	GB11520-PA	AT5G24360.1	71154	BDEG_06487	CE35836	1524	20714	259954	-	CMM326C	-	DDB0189432	-	-	61.m00176	ENSGALP00000005493	-	NP_150296.3	-	108604	-	-	93936	NCU02202	Os07g28820.1	-	-	11633	164091	-	-	206770	RO3G_17045.1	YHR079C	SPAC167.01	GLEAN3_09434	SINFRUP00000136357	92.m00119	-	-	-	53745	-	-	UM03841.1
CLR11	Coxiella-Like Repeat protein	-	-	-	-	-	-	-	-	-	-	-	-	20695	-	-	-	-	-	-	-	-	-	-	-	-	-	-	-	-	-	-	-	-	-	-	-	-	-	-	-	-	-	-	-	-	-	-	-	-	-	-
CLR12	Coxiella-Like Repeat protein	-	-	-	-	-	-	-	-	-	-	-	-	20689	-	-	-	-	-	-	-	-	-	-	-	-	-	-	-	-	-	-	-	-	-	-	-	-	-	-	-	-	-	-	-	-	-	-	-	-	-	-
CLR13	Coxiella-Like Repeat protein	-	-	-	-	-	-	-	-	-	-	-	-	20686	-	-	-	-	-	-	-	-	-	-	-	-	-	-	-	-	-	-	-	-	-	-	-	-	-	-	-	-	-	-	-	-	-	-	-	-	-	-
FAP79		-	-	-	+	-	-	-	-	-	-	-	-	20645	-	-	-	-	-	-	-	-	-	GL50803_16914	-	-	-	-	65375	-	-	-	-	-	42720	-	-	-	-	-	-	-	-	-	-	-	-	-	-	-	-	-
RLS6	hypothetical protein containing a SAND domain	-	-	-	-	-	-	-	-	-	-	-	-	20566	-	-	-	-	-	-	-	-	-	-	-	-	-	-	-	-	-	-	-	-	-	-	-	-	-	-	-	-	-	-	-	-	-	-	-	-	-	-
ANK18	protein of unknown function with ankyrin repeats	-	-	-	-	-	-	-	-	-	-	-	-	20444	-	-	-	-	-	-	-	-	-	-	-	-	-	-	-	-	-	-	-	-	-	-	-	-	-	-	-	-	-	-	-	-	-	-	-	-	-	-
FAS1	fasciclin-like protein	-	-	-	-	-	-	-	-	-	-	-	-	20257	-	-	-	-	-	-	-	-	-	-	-	-	-	-	-	-	-	-	-	-	-	-	-	-	-	-	-	-	GLEAN3_05198	-	-	-	-	-	-	-	-	-
POC12	centriole proteome protein	+	+	-	+	-	-	GB12794-PA	-	-	BDEG_00264	-	157534	20204	270563	-	-	OTTDARP00000018990	-	-	-	-	ENSGALP00000000998	-	OTTHUMP00000165111	-	228399	-	29577	209121	-	-	-	GSPATP00011904001	-	-	-	-	-	-	-	-	-	SINFRUP00000131495	89.m00156	-	-	-	61316	-	-	-
ZMP1	zinc-metallopeptidase-like protein	-	+	-	-	-	-	GB10205-PA	AT5G42620.1	-	-	CE16622	-	20076	215149	-	-	-	DDB0187000	CG3953-PA	-	111.m00118	ENSGALP00000019687	-	OTTHUMP00000174227	LmjF28.0570	212290	6773	74654	203012	-	Os03g52150.2	-	GSPATP00012897001	42588	132950	137810	-	755035	-	-	-	GLEAN3_10808	SINFRUP00000148324	172.m00054	263313	-	-	-	124862.m00003	Tb11.02.5310	-
GTR6	glycosyl transferase	-	+	+	-	-	-	GB13757-PA	AT2G40190.1	2187	BDEG_03160	CE26792	156664	19999	292389	cgd4_2990	CMI301C	-	DDB0184239	CG11306-PA	-	3.m00560	ENSGALP00000027453	-	NP_001004127.1	LmjF35.5250	198678	27085	35674	124985	NCU06779	Os12g39340.1	2131	GSPATP00031255001	54621	196599	108303	-	570235	RO3G_05010.1	YNL048W	SPCC330.08	-	SINFRUP00000145313	99.m00116	269819	-	50.m03145	50291	94829.m00054	Tb09.211.0860	UM05209.1
POLL1	Pol lambda-related Class X DNA repair polymerase	-	+	-	-	-	-	-	AT1G10520.1	-	-	-	169992	19822	219811	-	-	OTTDARP00000019851	DDB0215784	-	-	-	ENSGALP00000038555	-	OTTHUMP00000020322	LmjF08.0890	72491	33482	62711	190712	NCU07461	Os06g13020.2	34957	GSPATP00008489001	-	193840	-	-	805058	-	-	SPAC2F7.06c	GLEAN3_06768	SINFRUP00000134869	119.m00101	-	-	-	28268	-	Tb927.5.2780	-
FBA1	fructose-1,6-bisphosphate aldolase	-	-	-	-	-	-	-	-	-	-	-	-	-	-	-	-	-	-	-	-	-	-	-	-	-	-	-	-	-	-	-	-	-	-	-	-	-	-	-	-	-	-	-	-	-	-	-	-	-	-	-
BUG25	basal body protein of unknown function	-	+	-	-	-	-	-	AT4G36920.1	-	-	-	-	19751	-	-	-	-	-	-	-	-	-	-	-	-	-	-	-	-	-	Os04g55560.3	16482	-	-	147502	-	-	655117	-	-	-	-	-	-	-	-	-	-	-	-	-
CDJ3	Chloroplast DnaJ homolog 3	-	-	-	-	-	-	-	-	-	-	-	-	19746	-	-	-	-	-	-	-	-	-	-	-	-	-	-	-	-	-	Os05g45350.2	-	-	-	-	-	-	572857	-	-	-	-	-	-	-	-	-	-	-	-	-
FAP164	Flagella Associated Protein	-	+	-	+	-	-	-	-	-	-	-	27024	19665	-	-	-	-	-	-	-	-	ENSGALP00000025385	-	-	LmjF30.3260	-	25353	-	20239	-	-	-	GSPATP00001373001	-	-	-	PF11_0197	676092	-	-	-	-	-	-	-	TA10905	-	51290	-	Tb927.6.4610	UM04852.1
AKR	aldo-keto-reductase like protein	-	-	-	-	-	-	-	-	-	-	-	-	19081	-	-	-	-	-	-	-	-	-	-	-	-	-	-	-	-	-	-	-	-	-	-	-	-	-	-	-	-	-	-	-	-	-	-	-	-	-	-
CLR6	Coxiella-Like Repeat protein	-	-	-	-	-	-	-	-	-	-	-	-	18960	-	-	-	-	-	-	-	-	-	-	-	-	-	-	-	-	-	-	-	-	-	-	-	-	-	-	-	-	-	-	-	-	-	-	-	-	-	-
POC16	centriole proteome protein	-	+	-	+	-	-	-	-	71486	-	-	229205	18900	-	-	-	-	-	-	-	-	-	-	-	-	230310	-	-	40956	-	-	-	-	-	-	-	-	-	-	-	-	-	SINFRUP00000175722	-	-	-	20.m05891	-	-	-	-
DCL2	Dicer-like protein	-	+	-	-	-	-	-	-	6264	BDEG_03387	-	-	18765	-	-	-	-	-	-	-	-	-	-	-	-	-	-	48963	119416	-	-	-	-	-	234670	-	-	-	-	-	-	-	-	-	-	-	-	17843	-	-	-
AGO3	Argonaute-like protein	-	+	+	-	-	-	GB12654-PA	AT1G48410.2	-	BDEG_04085	CE13367	143595	18730	211168	-	-	OTTDARP00000014546	DDB0188857	CG6671-PB	-	11.m00378	ENSGALP00000040091	-	OTTHUMP00000178304	-	235802	-	63051	180719	NCU04730	Os06g39640.1	-	GSPATP00019939001	52260	141045	157676	-	282344	RO3G_10137.1	-	SPCC736.11	GLEAN3_19389	SINFRUP00000178378	259.m00042	-	-	583.m05335	62728	95940.m00269	-	-
ELG17	exostosin-like glycosyltransferase	-	-	-	-	-	-	-	-	-	-	-	-	18637	-	-	-	-	-	-	-	-	-	-	-	-	-	-	-	-	-	-	-	-	-	-	-	-	-	-	-	-	-	-	-	-	-	-	-	-	-	-
FAP268	Flagella Associated Protein	-	-	-	-	-	-	-	-	-	-	-	-	18398	-	-	-	-	-	-	-	-	-	-	-	-	-	-	-	-	-	-	-	-	-	-	-	-	753357	-	-	-	-	-	-	-	-	-	-	-	-	-
GTR4	glycosyl transferase	-	+	-	-	-	-	GB18712-PA	-	21068	BDEG_06325	CE30081	18905	18353	385208	cgd5_2590	CMK197C	-	DDB0219333	CG7870-PA	-	-	ENSGALP00000027494	-	OTTHUMP00000018263	-	187174	-	33611	234255	-	-	-	GSPATP00038317001	45980	115941	135762	-	-	RO3G_00404.1	YPL227C	SPBC56F2.10c	-	SINFRUP00000138920	3.m01715	269159	-	35.m00888	25674	-	-	UM01231.1
SEC63	ER-targeted preprotein translocase subunit of 63 kD	-	+	-	-	-	-	GB18907-PA	AT1G79940.1	70315	BDEG_02499	CE24546	160293	18187	393267	cgd7_3880	CMQ208C	-	DDB0186819	CG8583-PA	-	373.m00045	ENSGALP00000024664	-	OTTHUMP00000016937	LmjF35.4630	212166	30812	-	88731	NCU00169	Os04g24180.2	27852	GSPATP00031196001	27039	170320	128297	PF13_0102	759212	RO3G_02493.1	YOR254C	SPBC36B7.03	-	SINFRUP00000170006	50.m00270	26957	TA13480	55.m00017	56581	-	-	UM06175.1
RABC2	RABC2, small Rab-related GTPase	-	-	-	-	-	-	-	-	-	-	-	-	18094	-	-	-	-	-	-	-	-	-	-	-	-	-	-	-	-	-	-	-	-	-	-	-	-	-	-	-	-	-	-	-	-	-	-	-	-	-	-
GPM1b	Phosphoglucomutase, possible cytosolic form	-	-	-	-	-	-	-	-	-	-	-	-	-	-	-	-	-	-	-	-	-	-	-	-	-	-	-	-	-	-	-	-	-	-	-	-	-	-	-	-	-	-	-	-	-	-	-	-	-	-	-
FAP208	Found in the flagellar proteome (Pazour, Agrin, Leszyk, Witman; PMID: 15998802)	-	-	-	-	-	-	-	AT3G07790.1	-	-	-	-	17930	-	-	-	-	-	-	-	-	-	-	-	-	-	-	-	-	-	Os06g30710.3	-	-	-	170056	-	-	775129	-	-	-	-	-	-	-	-	-	-	-	-	-
DNJ32	DnaJ-like protein 32, perhaps nuclear/cytoplasmic	-	+	-	-	-	-	GB18830-PA	-	20729	-	CE16336	220238	17550	266086	cgd5_2950	CMO059C	-	DDB0187017	CG2790-PA	-	-	ENSGALP00000005343	-	OTTHUMP00000082746	LmjF25.2190	179278	34486	2538	201862	NCU02432	Os12g31840.1	-	-	36016	233176	136499	PF10_0058	-	RO3G_17058.1	YNL227C	-	-	-	60.m00169	-	-	162.m00513	5788	89339.m00177	Tb927.3.2290	-
		-	-	-	-	-	-	-	-	-	-	-	-	-	-	-	-	-	-	-	-	-	-	-	-	-	-	-	-	-	-	-	-	-	-	-	-	-	-	-	-	-	-	-	-	-	-	-	-	-	-	-
		-	-	-	-	-	-	-	-	-	-	-	-	-	-	-	-	-	-	-	-	-	-	-	-	-	-	-	-	-	-	-	-	-	-	-	-	-	-	-	-	-	-	-	-	-	-	-	-	-	-	-
ABH1	putative ortholog of CBP80; Arabidopsis ABH1	-	+	-	-	-	-	GB11434-PA	AT2G13540.1	-	BDEG_06812	CE29793	202900	17391	297352	-	CMJ189C	-	DDB0190586	CG7035-PA	-	-	ENSGALP00000003250	-	OTTHUMP00000021753	-	190753	-	-	193215	NCU04187	Os03g22570.2	33787	-	44773	-	-	-	722993	RO3G_03303.1	YMR125W	SPAC6G10.07	GLEAN3_11442	SINFRUP00000150573	-	-	-	-	54475	-	-	UM06211.1
ELG9	exostosin-like glycosyltransferase	-	-	-	-	-	-	-	-	70660	-	-	-	17088	293631	-	-	-	-	-	-	-	-	-	-	-	-	-	-	-	-	-	-	-	-	-	-	-	-	-	-	-	-	-	-	-	-	-	-	-	-	-
ANK14	protein of unknown function with ankyrin repeats	-	-	-	-	-	-	-	-	-	-	-	-	17065	-	-	-	-	-	-	-	-	-	-	-	-	-	-	-	-	-	-	-	-	-	-	-	-	-	-	-	-	-	-	-	-	-	-	-	-	-	-
MUT11	WD40-repeat containing protein, transcription repression	-	-	-	-	-	-	-	-	-	-	-	-	16794	-	-	-	-	-	-	-	-	-	-	-	-	-	-	2963	-	NCU03244	-	-	-	-	-	-	-	-	-	-	-	-	-	-	-	-	-	-	-	-	-
FAP154	Flagellar Associated Protein with PAS Sensory Domain	-	-	-	+	-	-	-	-	-	-	-	-	16685	-	-	-	-	-	-	-	-	-	-	-	-	-	-	-	-	-	-	-	-	-	-	-	-	-	-	-	-	-	-	-	-	-	-	-	-	-	-
FAP49	Found in the flagellar proteome (PMID: 15998802);	-	-	-	-	-	-	-	-	-	-	-	-	16684	-	-	-	-	-	-	-	-	-	-	-	-	-	-	-	-	-	-	-	-	-	-	-	-	-	-	-	-	-	-	-	-	-	-	-	-	-	-
ELG27	exostosin-like glycosyltransferase	-	-	-	-	-	-	-	-	-	-	-	-	16652	-	-	-	-	-	-	-	-	-	-	-	-	-	-	-	-	-	-	-	-	-	-	-	-	-	-	-	-	-	SINFRUP00000161280	-	-	-	-	-	-	-	-
HSP70F	Heat shock protein 70F	-	-	-	-	-	-	-	-	-	-	-	-	16541	-	-	-	-	-	-	-	-	-	-	-	-	-	-	-	-	-	-	-	-	-	-	-	-	-	-	-	-	-	-	-	-	-	-	-	-	-	-
	putative programmed cell death 2, C terminal	-	+	-	-	-	-	GB16114-PA	AT4G02220.1	-	-	CE18125	54196	16242	298031	cgd2_3420	CMC016C	OTTDARP00000018531	DDB0185817	CG3260-PA	-	-	-	-	NP_659005.1	-	220203	23423	-	159435	-	Os03g18830.2	24191	-	55067	202906	144440	MAL7P1.76	-	-	-	-	GLEAN3_04346	-	51.m00268	24632	TA16265	-	27184	-	-	-
FMG1-2	flagella membrane glycoprotein	-	-	-	+	-	-	-	-	-	-	-	-	16132	-	-	-	-	-	-	-	-	-	-	-	-	-	-	-	-	-	-	-	-	-	-	-	-	-	-	-	SPBPJ4664.02	-	-	-	-	-	-	-	-	-	-
SFI1	spindle pole body protein	-	+	-	-	-	-	-	-	62492	BDEG_07837	CE29321	227086	16085	-	-	-	-	DDB0215111	-	-	-	ENSGALP00000033167	-	-	-	233819	11095	-	242774	-	Os01g06700.1	-	-	-	-	114414	-	577115	RO3G_17279.1	YIL082W-A	SPAC9.04	-	SINFRUP00000165769	-	-	-	-	-	-	-	-
	Putative DNA (cytosine-C5) methyltransferase	-	-	-	-	-	-	-	-	-	-	-	-	15852	-	-	-	-	-	-	-	-	-	-	-	-	-	-	-	-	-	-	-	-	-	-	-	-	-	-	-	-	-	-	-	-	-	-	-	-	-	-
MUT9	Serine/threonine protein kinase, transcription repression	-	-	-	-	-	-	-	-	-	-	-	-	15736	-	-	-	-	-	-	-	-	-	-	-	-	-	-	-	-	-	-	-	-	-	-	-	-	-	-	-	-	-	-	-	-	-	-	-	-	-	-
HSP70E	Heat shock protein 70E	-	+	+	-	-	-	GB10836-PA	-	-	BDEG_03715	CE00103	221226	15589	-	cgd4_3270	-	OTTDARP00000021700	DDB0188788	CG6603-PC	-	197.m00082	ENSGALP00000016536	-	-	LmjF18.1370	111562	-	37170	233881	NCU05269	Os01g08560.2	35719	GSPATP00027191001	41417	180192	144810	-	-	RO3G_06488.1	YPL106C	SPAC110.04c	GLEAN3_02779	SINFRUP00000152426	105.m00092	39098	-	38.m01113	25019	-	Tb10.389.0880	UM05918.1
		-	-	-	-	-	-	-	-	-	-	-	-	15534	-	-	-	-	-	-	-	-	-	-	-	-	-	-	-	-	-	-	-	-	-	-	-	-	-	-	-	-	-	-	-	-	-	-	-	-	-	-
ELG13	exostosin-like glycosyltransferase	-	-	-	-	-	-	-	-	-	-	-	-	15136	-	-	-	-	DDB0203385	-	-	-	-	-	OTTHUMP00000030103	-	-	-	-	-	-	-	-	-	-	-	-	-	-	-	-	-	-	-	-	-	-	-	13000	-	-	-
AGO1	Argonaute-like protein	-	-	-	-	-	-	-	-	-	-	-	-	14936	-	-	-	-	-	-	-	-	-	-	-	-	-	-	-	-	-	-	-	-	-	-	-	-	-	-	-	-	-	-	-	-	-	-	-	-	-	-
GLL2	gametolysin-like protein	-	-	-	-	-	-	-	-	-	-	-	-	14859	-	-	-	-	-	-	-	-	-	-	-	-	-	-	-	-	-	-	-	-	33624	-	-	-	-	-	-	-	-	-	-	-	-	-	-	-	-	-
RPPK2	ribose-phosphate pyrophosphokinase (RPPK)	-	+	-	-	-	-	-	AT1G10700.1	21520	-	-	18492	14590	-	-	-	-	-	-	-	-	-	-	-	-	-	-	-	119080	-	Os01g52530.1	9132	-	9612	222025	-	-	230554	-	-	-	-	-	-	-	-	-	-	-	-	-
APC4	Subunit of anaphase promoting complex.	-	+	-	-	-	-	-	AT4G21530.1	72273	-	-	103123	14425	296586	-	CMF180C	OTTDARP00000006598	DDB0186409	CG32707-PA	-	-	ENSGALP00000036551	-	OTTHUMP00000123382	-	162086	24074	-	131737	-	Os02g54490.1	-	-	-	168296	-	-	817758	RO3G_04194.1	-	-	-	SINFRUP00000182345	-	-	-	-	55371	-	-	-
DNJ15	DnaJ-like protein 15, perhaps targeted to chloroplast	-	-	-	-	-	-	-	-	27288	-	-	-	14406	-	-	-	-	-	-	-	-	-	-	-	-	-	-	-	-	-	-	-	-	-	-	-	-	-	-	-	-	-	-	-	-	-	-	-	-	-	-
RCD2	Rcd1-like protein	-	-	-	-	-	-	-	-	-	-	-	-	14080	-	-	-	-	-	-	-	-	-	-	-	-	-	-	-	-	-	-	-	-	-	-	-	-	-	-	-	-	-	-	-	-	-	-	-	-	-	-
ELG4	exostosin-like glycosyltransferase	-	-	-	-	-	-	-	-	-	-	-	-	13675	-	-	-	-	-	-	-	-	-	-	-	-	-	-	-	-	-	-	-	-	-	-	-	-	-	-	-	-	-	-	-	-	-	-	-	91727.m00140	-	-
RAD17	DNA damage checkpoint protein.	-	-	-	-	-	-	-	-	-	-	-	-	13563	-	-	-	-	-	-	-	-	-	-	-	-	-	-	-	-	-	Os03g13850.1	10877	-	-	141878	-	-	-	-	-	-	-	-	-	-	-	-	-	-	-	-
POC11	centriole proteome protein	+	+	-	-	-	-	-	-	-	BDEG_02647	-	147477	13542	284799	-	-	OTTDARP00000023008	-	-	-	-	ENSGALP00000021170	-	OTTHUMP00000166123	-	198558	-	70454	214804	-	-	-	GSPATP00008891001	-	-	138192	-	-	-	-	-	GLEAN3_03045	SINFRUP00000178314	105.m00165	-	-	-	2488	-	Tb11.02.4410	-
RAD50	DNA repair protein	-	+	+	-	-	-	GB15340-PA	AT2G31970.1	5421	BDEG_05793	CE21149	122168	13480	259857	cgd1_2410	CMQ187C	-	DDB0184568	CG6339-PA	19069125	102.m00081	ENSGALP00000039135	-	OTTHUMP00000065928	LmjF28.0530	139054	14089	-	95716	NCU00901	Os02g29464.1	30141	GSPATP00010831001	51876	-	127181	PFF0285c	829809	RO3G_14378.1	YNL250W	SPAC1556.01c	GLEAN3_25732	SINFRUP00000153141	-	9195	TA11630	55.m04683	-	91714.m00088	Tb11.01.0340	UM01085.1
FADS1	Putative FAD synthetase	-	+	-	-	-	-	GB12247-PA	-	8492	BDEG_01874	CE38215	220999	13453	284858	-	CMS202C	-	DDB0185283	CG4407-PB	-	-	-	GL50803_16269	OTTHUMP00000035547	LmjF28.0270	106425	-	-	204022	NCU09233	-	-	GSPATP00015809001	-	-	155441	PF10_0147	-	-	YDL045C	SPCC1235.04c	GLEAN3_18748	SINFRUP00000145906	274.m00031	-	-	33.m01274	18732	85889.m00484	Tb11.02.5320	UM03965.1
TOC159	Similar to 159 kDa translocon at the outer membrane of chloroplasts	-	+	-	-	-	-	-	AT5G20300.3	-	-	-	-	13382	-	-	-	-	-	-	-	-	ENSGALP00000034853	-	-	-	-	-	-	-	-	Os12g09570.2	359	-	-	189669	-	-	200844	-	-	-	-	-	-	-	-	-	-	-	-	-
DNJ17	DnaJ-like protein 17, rather cytosolic	-	-	-	-	-	-	-	-	-	-	-	-	13259	-	-	-	-	-	-	-	-	-	-	-	-	-	-	-	-	-	-	-	-	-	-	-	-	-	-	-	-	-	-	-	-	-	-	-	-	-	-
HSP70D	Heat shock protein 70D	-	-	-	-	-	-	-	-	-	-	-	-	13257	-	-	-	-	-	-	-	-	-	-	-	-	-	-	-	-	-	-	-	-	-	-	-	-	-	-	-	-	-	-	-	-	-	-	-	-	-	-
CHR3422	SNF2 family Chromodomain-helicase protein	-	-	-	-	-	-	-	-	-	-	-	-	13217	-	-	-	-	-	-	-	-	-	-	-	-	-	-	-	-	-	-	-	-	-	-	-	-	-	-	-	-	-	-	-	-	-	-	-	-	-	-
GPP	reverse transcriptase, gag-pol polyprotein	-	+	-	-	-	-	-	AT4G23160.1	-	BDEG_04984	-	-	13035	-	-	-	-	-	-	-	-	-	-	-	-	-	13232	-	-	-	Os01g49300.1	18931	-	-	73915	145326	-	252325	-	YCL074W	-	-	-	-	-	-	-	-	-	-	UM04123.1
ESP1	Separase, cell cycle protease.	-	+	-	-	-	-	-	-	72664	BDEG_00309	-	168112	12539	294634	cgd7_2760	-	-	DDB0188839	-	19069099	182.m00140	-	-	-	-	230378	33070	-	-	NCU00205	-	-	-	-	161712	-	-	-	-	YGR098C	SPCC5E4.04	-	SINFRUP00000162593	27.m00387	260749	-	-	-	88601.m00431	-	UM03262.1
FAP159	Found in the flagellar proteome (Pazour, Agrin, Leszyk, Witman; PMID: 15998802)	-	-	-	+	-	-	-	-	-	-	-	-	12416	-	-	-	-	-	-	-	-	-	-	-	-	-	-	-	-	-	-	-	-	-	-	-	-	-	-	-	-	-	-	-	-	-	-	-	-	-	-
	Unknown hypothetical protein	-	-	-	-	-	-	-	-	-	-	-	-	12202	-	-	-	-	-	-	-	-	-	-	-	-	-	-	-	-	-	-	-	-	-	-	-	-	-	-	-	-	-	-	-	-	-	-	-	-	-	-
DNJ19	DnaJ-like protein 19, rather cytosolic	-	+	-	-	-	-	GB11895-PA	AT5G23590.2	-	-	-	-	12075	374172	-	-	-	-	-	-	-	ENSGALP00000039621	-	OTTHUMP00000176197	-	-	-	-	-	-	-	-	-	-	25801	-	-	557051	-	-	-	-	-	-	-	-	-	-	-	-	-
DCL3	Dicer-like protein	-	-	-	-	-	-	-	-	-	-	-	-	12013	-	-	-	-	-	-	-	-	-	-	-	-	-	-	-	-	-	-	-	-	-	-	-	-	-	-	-	-	-	-	-	-	-	-	-	-	-	-
DNJ20	DnaJ-like protein 20, rather cytosolic	-	-	-	-	-	-	-	-	-	-	-	-	11900	-	-	-	-	-	-	-	-	-	-	-	-	-	-	-	-	-	-	-	-	-	-	-	-	-	-	-	-	-	-	-	-	-	-	-	-	-	-
DNJ30	DnaJ-like protein 30	-	+	-	-	-	-	-	-	63122	-	-	159559	11662	-	-	-	OTTDARP00000010457	-	-	-	-	ENSGALP00000023598	-	OTTHUMP00000149981	LmjF25.2070	98133	-	64077	211264	-	-	-	-	50054	198650	138635	-	-	-	-	-	-	SINFRUP00000165649	29.m00326	22681	-	50.m00088	57450	-	Tb927.3.2160	UM04415.1
CTF18	DNA damage repair and chromosome cohesion protein.	-	+	-	-	-	-	GB16629-PA	AT1G04730.1	-	BDEG_01926	-	-	11559	250209	-	-	OTTDARP00000022358	DDB0168957	CG33122-PA	-	-	ENSGALP00000008496	-	OTTHUMP00000080307	LmjF17.0540	230720	-	-	-	NCU07313	Os03g15810.1	14300	-	45690	64140	136979	-	-	RO3G_14917.1	-	SPBC902.02c	-	SINFRUP00000129816	58.m00267	-	-	59.m03543	53969	-	-	UM02823.1
SEC5	Component of the Exocyst Complex	-	+	-	-	-	-	-	AT1G21170.1	-	BDEG_08121	CE03705	207105	11505	260526	-	-	OTTDARP00000005170	DDB0206363	CG8843-PA	-	107.m00106	ENSGALP00000020908	-	OTTHUMP00000164193	-	-	-	-	-	NCU07698	Os04g34450.1	-	-	-	121949	-	-	204897	-	-	-	GLEAN3_28263	SINFRUP00000131363	-	1471	-	-	55542	-	-	UM00710.1
ANK6	protein of unknown function with ankyrin repeats	-	-	-	-	-	-	-	-	-	-	-	55609	11440	-	-	-	-	-	-	-	-	-	-	-	-	-	-	-	-	-	-	-	-	-	-	-	-	-	-	-	-	-	-	-	-	-	-	-	-	-	-
GHMP1	galactokinase like protein	-	+	-	-	-	-	GB16893-PA	AT4G16130.1	-	-	-	-	11299	-	-	-	-	-	-	-	-	-	-	-	-	-	-	-	-	-	Os02g04840.1	-	-	-	176198	-	-	746579	-	-	-	GLEAN3_06153	-	-	-	-	-	-	-	-	-
PAT1	phosphate acetyltransferase	-	-	-	+	-	-	-	-	-	-	-	-	11226	-	-	-	-	-	-	-	-	-	-	-	-	-	-	-	-	-	-	-	-	-	-	-	-	-	-	-	-	-	-	-	-	-	-	-	-	-	-
HCF164	Thioredoxin-like protein similar to Arabidopsis HCF164	-	+	-	-	-	-	-	AT4G37200.1	8417	-	-	-	11164	-	-	-	-	-	-	-	-	-	-	-	-	-	-	-	-	-	Os03g55820.1	6671	-	12104	146960	-	-	832358	-	-	-	-	-	-	33417	-	-	-	-	-	-
ABC1	ABC1-transporter like protein	-	+	-	-	-	-	-	AT4G24810.2	-	-	-	-	10730	-	-	CMN159C	-	-	-	-	-	-	-	-	-	-	-	-	-	-	Os01g67720.1	-	-	-	112757	-	-	252534	-	-	-	-	-	-	-	-	-	-	-	-	-
DNJ23	DnaJ-like protein 23	-	+	-	-	-	-	GB13913-PA	AT2G35720.1	-	-	CE07060	177367	10705	239955	-	CMO337C	OTTDARP00000002059	DDB0206576	CG8531-PA	-	-	ENSGALP00000000889	-	NP_060668.1	-	217203	32622	-	193264	-	Os10g36370.1	23992	-	12221	105082	-	-	826532	RO3G_07747.1	-	SPCC63.03	-	SINFRUP00000140243	-	-	-	-	27061	-	-	UM02944.1

GHR	glycoside-hydrolase-like protein	-	-	-	-	-	-	-	AT2G36190.1	6143	-	-	-	10538
FBP	fructose-1,6-bisphosphatase	-	-	-	-	-	-	-	-	-	-	-	-	-
FCL	fibrocystin-L-like protein	-	+	-	-	-	-	-	-	-	-	-	-	10029
POC5	centriole proteome protein	-	-	-	-	-	-	-	-	-	-	-	-	9798
CDT1	DNA replication initiation factor	-	-	-	-	-	-	-	-	-	-	-	-	9793
POC3	centriole proteome protein	-	-	-	+	-	-	-	-	-	-	-	-	9715
POC4	centriole proteome protein	-	-	-	+	-	-	-	-	-	-	-	-	9658
LMR2	protein of unknown function, contains LysM-domains	-	-	-	-	-	-	-	-	-	-	-	-	9571
FAP272	Flagellar Associated Protein Similar to Calmodulin	-	-	-	-	-	-	-	-	-	-	-	-	9509
POLE1	DNA polymerase epsilon catalytic subunit A/1	-	+	-	-	-	-	GB13768-PA	AT2G27120.1	33973	BDEG_03258	CE17767	155313	9481
		-	+	+	-	-	-	GB16077-PA	AT5G40820.1	36497	BDEG_01424	-	164972	8906
	Putative DNA (cytosine-C5) methyltransferase	-	+	-	-	-	-	GB15130-PA	AT5G49160.1	-	-	-	160905	8793
BUG23	basal body protein of unknown function	-	-	-	-	-	-	-	-	-	-	-	-	8784
PGM2	phosphoglycerate/bisphosphoglycerate mutase family protein	-	+	-	-	-	-	-	AT5G04120.1	-	-	-	219666	8761
CHC3401	SWIB complex BAF60b domain-containing protein	-	+	-	-	-	-	GB19354-PA	AT5G14170.1	-	-	CE01687	158720	8547
ELG15	exostosin-like glycosyltransferase	-	+	-	-	-	-	-	AT3G57630.1	-	-	-	-	8449
HSP22B	heat shock protein 22B	-	+	-	-	-	-	-	-	-	-	CE34516	-	8329
HSP22H	heat shock protein 22H	-	-	-	-	-	-	-	-	-	-	-	-	8323
	unknown hypothetical protein	-	+	-	-	-	-	-	AT5G22760.1	-	BDEG_05640	-	-	8155
ANK24,FAP175	Flagellar Associated Protein	-	+	-	+	-	-	GB14009-PA	-	-	-	CE35821	-	8129
MTA4a	copy of the MTA4 gene; probable a pseudogene	-	-	-	-	-	-	-	-	-	-	-	-	7933
SRP68	Subunit of the Signal Recognition Particle	-	+	-	-	-	-	GB17079-PA	AT5G61970.1	-	BDEG_06480	CE20875	155942	7632
PLS	phospholipid-scramblase-like protein	-	+	-	-	-	-	-	AT2G04940.1	-	BDEG_08162	CE07359	227594	7611
CHR3411	SNF2/RAD54 family protein	-	+	+	-	-	-	GB14810-PA	AT2G18760.1	10276	BDEG_03483	CE33793	20149	7539
ELG31	exostosin-like glycosyltransferase	-	-	-	-	-	-	-	-	-	-	-	-	7341
DNJ26	DnaJ-like protein 26, perhaps chloroplastic	-	-	-	-	-	-	-	-	-	-	-	-	-
ELG26	exostosin-like glycosyltransferase	-	-	-	-	-	-	-	-	-	-	-	-	7088
RPE2	D-Ribulose-5-Phosphate 3-Epimerase	-	+	+	-	-	-	-	AT1G63290.1	59005	BDEG_01853	CE27934	149571	6964
SET3	SET domain containing protein, histone methyltransferase	-	+	-	-	-	-	-	AT2G22740.1	70556	-	CE25069	206547	6823
CHR3420	SNF2 superfamily protein; FUN30 subclass	-	+	-	-	-	-	-	AT2G02090.1	-	-	-	-	6783
POC18	centriole proteome protein	+	+	-	+	-	-	GB11026-PA	-	-	BDEG_07129	-	120736	6466
EXO70	Component of the Exocyst Complex	-	+	-	-	-	-	-	AT5G03540.1	-	BDEG_02804	-	153271	6366
CLR22	Coxiella-Like Repeat protein	-	-	-	-	-	-	-	-	-	-	-	-	6185
NAT2	N-acetyltransferase-like protein	-	+	+	-	-	-	GB10872-PA	AT5G15770.1	-	BDEG_06243	CE05156	141578	5762
CHS,CHLG	Chlorophyll synthetase, chloroplast precursor	-	+	-	-	-	-	-	AT3G51820.1	54854	-	-	-	5437
TRS33	Component of TRAPP complex	-	+	-	-	-	-	GB15690-PA	AT3G05000.1	59697	BDEG_00662	CE18881	126805	5404
ALSS1	acetolactate synthase, small subunit	-	+	-	+	-	-	-	AT5G16290.2	-	-	-	-	5137
	WD40 domain protein	-	+	-	-	-	-	GB15882-PA	AT3G19590.1	4153	BDEG_00071	CE19233	21337	4738
DPY30	subiunit of chromatin modifying protein	-	+	-	-	-	-	-	-	29507	-	CE15445	155367	3897
RBJL1	RABJL1, small Rab-related GTPase	+	+	-	-	-	-	-	-	35202	BDEG_01011	-	146879	3686
VPS34	VPS34-like, PI-3-kinase	-	+	+	-	-	-	GB11687-PA	AT1G60490.1	22047	BDEG_08109	CE37691	155274	2983
	non-conserved protein, function unknown	-	-	-	-	-	-	-	-	-	-	-	-	2180
	RabGAP/TBC Domain Protein	-	+	-	-	-	-	GB17979-PA	AT3G07890.2	-	BDEG_01865	CE40584	210789	1807
SEC61B	SEC61-beta subunit of ER-translocon	-	+	-	-	-	-	GB13924-PA	AT2G45070.4	17697	-	CE27617	175733	1670
		-	-	-	-	-	-	-	-	-	-	-	-	96513
		-	-	-	-	-	-	-	-	-	-	-	-	96506
		-	-	-	-	-	-	-	-	-	-	-	-	-
		-	-	-	-	-	-	-	-	-	-	-	-	96492
		-	+	-	-	-	-	-	AT3G16270.1	-	-	-	201129	96488
		-	-	-	-	-	-	-	-	-	-	-	-	96487
		-	-	-	-	-	-	-	-	-	-	-	-	96485
		-	-	-	-	-	-	-	-	8007	-	-	138433	96482
		-	-	-	-	-	-	-	-	-	-	-	-	96477
		-	-	-	-	-	-	-	-	-	-	-	-	96475
		-	-	-	-	-	-	-	-	-	-	-	-	96469
		-	-	-	-	-	-	-	-	-	-	-	-	96467
		-	-	-	-	-	-	-	-	-	-	-	-	96463
		-	-	-	-	-	-	-	-	-	-	-	-	96460
		-	-	-	-	-	-	-	-	-	-	-	-	-
		-	-	-	-	-	-	-	-	-	-	-	-	96409
		-	-	-	-	-	-	-	-	-	-	-	-	96408
		-	-	-	-	-	-	-	-	-	-	-	-	96400
		-	+	-	-	-	-	-	AT1G77320.1	-	-	-	-	96394
		-	-	-	-	-	-	-	-	-	-	-	-	96389
		-	+	-	-	-	-	-	-	-	-	-	-	96388
		-	-	-	-	-	-	-	-	-	-	-	-	96374
		-	-	-	+	-	-	-	-	-	-	-	-	96369
		-	-	-	-	-	-	-	-	-	-	-	-	96367
		-	-	-	-	-	-	-	-	-	-	-	-	96364
		-	-	-	-	-	-	-	-	-	-	-	-	96308
		-	-	-	-	-	-	-	-	-	-	-	-	96306
		-	-	-	-	-	-	-	-	-	-	-	-	96298
SRX1	sulfiredoxin	-	+	-	-	-	-	-	AT1G31170.2	-	-	-	-	96296
		-	-	-	-	-	-	-	-	-	-	-	-	96293
		-	-	-	-	-	-	-	-	-	-	-	-	96289
		-	-	-	-	-	-	-	-	-	-	-	-	96283
		-	-	-	-	-	-	-	-	-	-	-	-	96274
		-	-	-	-	-	-	-	-	-	-	-	-	96264
		-	-	-	-	-	-	-	-	62829	-	-	-	96215
		-	-	-	-	-	-	-	-	-	-	-	-	96210
		-	-	-	+	-	-	-	-	-	-	-	-	96206
		-	-	-	-	-	-	-	-	-	-	-	-	96182
MAPKKK1	Mitogen-Activated Protein Kinase Kinase Kinase Homolog 1	-	-	-	-	-	-	-	-	-	-	-	-	96175
TRM3	tRNA (guanosine)-2'-O-methyltransferase	-	+	-	-	-	-	GB12548-PA	AT4G17610.1	25148	BDEG_02931	CE32068	220697	96162
		-	-	-	-	-	-	-	-	-	-	-	-	96154
		-	-	-	-	-	-	-	-	-	-	-	-	96152
		-	-	-	-	-	-	-	-	-	-	-	-	96140
		-	-	-	-	-	-	-	-	-	-	-	-	96123
		-	-	-	-	-	-	-	-	-	-	-	-	96122
		-	-	-	-	-	-	-	-	-	-	-	-	96121
		-	-	-	-	-	-	-	-	-	-	-	-	96117
		-	-	-	-	-	-	-	-	-	-	-	-	96092
		-	-	-	-	-	-	-	-	-	-	-	-	96073
		-	-	-	-	-	-	-	-	-	-	-	-	-
		-	-	-	-	-	-	-	-	-	-	-	-	96058
		-	-	-	-	-	-	-	-	-	-	-	-	96044
		-	-	-	-	-	-	-	-	-	-	-	-	96038
		-	-	-	-	-	-	-	-	-	-	-	-	96027
		-	-	-	-	-	-	-	-	-	-	-	-	96026
		-	-	-	-	-	-	-	-	-	-	-	-	96020
		-	-	-	-	-	-	-	-	-	-	-	-	96017
		-	+	-	-	-	-	-	-	-	BDEG_08462	-	-	96016
		-	-	-	-	-	-	-	-	-	-	-	-	96010
		-	-	-	-	-	-	-	-	-	-	-	-	96009
		-	-	-	-	-	-	-	-	-	-	-	-	96006
		-	-	-	-	-	-	-	-	-	-	-	-	96004
		-	-	-	-	-	-	-	-	-	-	-	-	95996
		-	-	-	-	-	-	-	-	-	-	-	-	95982
		-	-	-	-	-	-	-	-	-	-	-	-	95979
		-	-	-	-	-	-	-	-	-	-	-	-	95973
		-	-	-	-	-	-	-	-	-	-	-	-	95969
		-	-	-	-	-	-	-	-	-	-	-	-	95958
		-	-	-	-	-	-	-	-	-	-	-	-	95946
		-	-	-	-	-	-	-	-	-	-	-	-	95938
COX18	putative cytochrome c oxidase assembly factor, mitochondrial	-	+	-	-	-	-	GB17861-PA	-	-	BDEG_01519	CE38582	171303	95928
		-	-	-	-	-	-	-	-	-	-	-	-	95911
		-	-	-	-	-	-	-	-	-	-	-	-	95895
		-	-	-	-	-	-	-	-	-	-	-	143947	95892
		-	-	-	-	-	-	-	-	-	-	-	-	95891
		-	+	-	-	-	-	GB18526-PA	AT5G19430.1	-	-	CE29688	-	95886
		-	-	-	-	-	-	-	-	-	-	-	-	95880
		-	-	-	-	-	-	-	-	-	-	-	-	95871
FAP203	Flagellar Associated Protein	-	+	-	+	-	-	-	-	-	BDEG_05202	-	-	95868
		-	-	-	-	-	-	-	-	-	-	-	-	95849
		-	-	-	-	-	-	-	-	-	-	-	-	95841
		-	-	-	-	-	-	-	-	-	-	-	-	95840
		-	-	-	-	-	-	-	-	-	-	-	-	95826
		-	-	-	-	-	-	-	-	-	-	-	-	95820
		-	-	-	-	-	-	-	-	-	-	-	-	95813
		-	-	-	-	-	-	-	-	-	-	-	-	95806
		-	+	-	-	-	-	GB13528-PA	AT2G18900.1	-	-	-	-	95788
		-	-	-	-	-	-	-	-	-	-	-	-	95782
		-	-	-	-	-	-	-	-	-	-	-	-	95760
		-	-	-	-	-	-	-	-	-	-	-	-	95738
		-	-	-	-	-	-	-	-	-	-	-	-	95704
		-	-	-	-	-	-	-	-	-	-	-	-	95693
		-	-	-	-	-	-	-	-	-	-	-	-	95682
		-	-	-	-	-	-	-	-	-	-	-	-	95661
		-	-	-	-	-	-	-	-	-	-	-	-	95650
		-	+	-	-	-	-	GB10601-PA	AT3G47550.6	-	BDEG_03117	CE00937	55512	95634
		-	-	-	-	-	-	-	-	-	-	-	-	95621
		-	-	-	-	-	-	-	-	-	-	-	-	95618
		-	-	-	-	-	-	-	-	-	-	-	-	95598
		-	-	-	-	-	-	-	-	-	-	-	-	95596
		-	-	-	-	-	-	-	-	-	-	-	-	95575
		-	-	-	-	-	-	-	-	-	-	-	-	95563
PP2C1c	Protein Phosphatase 2C catalytic subunit isoform 1	-	+	+	+	-	-	GB17110-PA	AT4G31750.1	23750	-	-	44132	95558
		-	-	-	-	-	-	-	-	-	-	-	-	95549
		-	+	-	-	-	-	-	-	32583	-	-	-	95535
		-	-	-	-	-	-	-	-	-	-	-	-	95505
		-	-	-	-	-	-	-	-	-	-	-	-	95498
		-	-	-	-	-	-	-	-	-	-	-	-	95493
		-	+	-	-	-	-	GB16396-PA	AT2G20190.1	-	-	CE29074	-	95490
NAT1		-	+	+	-	-	-	GB11203-PA	AT5G13780.1	21947	BDEG_07681	CE18023	180985	95444
		-	+	+	-	-	-	GB30013-PA_2	AT5G59770.1	-	BDEG_05315	-	151760	95439
		-	+	-	-	-	-	-	-	-	BDEG_03103	-	101892	95438

10538	-	-	-	-	-	-	-	-	-	-	-	-	-	-	-	-	-	-	-	-	-	-	-	-	819410	-	-	SPAC8E11.01c	-	-	-	-	-	-	-	-	-	-
-	-	-	-	-	-	-	-	-	-	-	-	-	-	-	-	-	-	-	-	-	-	-	-	-	-	-	-	-	-	-	-	-	-	-	-	-	-	-
10029	-	-	-	-	DDB0187448	-	-	-	ENSGALP00000026863	-	-	-	-	22411	73885	40040	-	-	-	GSPATP00013850001	48753	-	-	-	-	-	-	-	-	-	-	-	-	-	-	-	-	-
9798	-	-	-	-	-	-	-	-	-	-	-	-	-	-	-	-	-	-	-	-	-	-	-	-	-	-	-	-	-	-	-	-	-	-	-	-	-	-
9793	-	-	-	-	-	-	-	-	-	-	-	-	-	-	-	-	-	-	-	-	-	68809	-	-	-	-	-	-	-	-	-	-	-	-	-	-	-	-
9715	-	-	-	-	-	-	-	-	ENSGALP00000018199	-	NP_079390.3	-	-	-	-	-	-	-	-	-	-	-	-	-	-	-	-	-	-	SINFRUP00000172898	-	-	-	-	60280	-	-	-
9658	-	-	-	-	-	-	-	-	-	-	-	-	-	-	-	-	-	-	-	-	-	-	-	-	-	-	-	-	-	-	-	-	-	-	-	-	-	-
9571	-	-	-	-	-	-	-	-	-	-	-	-	-	-	-	-	-	-	-	-	-	-	-	-	-	-	-	-	-	-	-	-	-	-	-	-	-	-
9509	-	-	-	-	-	-	-	-	-	-	-	-	-	-	-	-	-	-	-	-	-	-	-	-	-	-	-	-	-	-	-	-	-	-	-	-	-	-
9481	269698	cgd8_1240	CMQ098C	-	DDB0185405	-	19069372	-	ENSGALP00000037402	GL50803_23064	OTTHUMP00000174875	LmjF35.4360	217825	15015	59151	119951	NCU04548	Os02g30800.1	101	GSPATP00033819001	52678	136896	134045	PFF1470c	594813	RO3G_00775.1	YNL262W	SPBC25H2.13c	GLEAN3_25749	SINFRUP00000155041	8.m00326	268553	TA09840	641.m01537	30078	-	Tb09.211.1820	UM01008.1
8906	249764	cgd4_2670	CMN075C	-	DDB0183856	CG4252-PA	19068674	66.m00162	ENSGALP00000004185	-	OTTHUMP00000173104	LmjF32.1460	190706	14645	31751	171012	NCU11188	Os06g50910.2	18131	-	44708	129584	155446	-	818220	RO3G_06833.1	YBR136W	SPBC216.05	GLEAN3_11017	SINFRUP00000137662	-	261045	TA15125	76.m01548	54713	97324.m00264	Tb11.01.6300	UM01110.1
8793	294397	-	-	-	-	-	-	-	-	-	NP_001370.1	-	171288	-	78038	125496	NCU02247	Os07g08500.1	-	-	-	40128	-	-	800689	-	-	-	GLEAN3_23599	SINFRUP00000181631	-	-	-	-	-	-	-	-
8784	-	-	-	-	-	-	-	-	-	-	-	-	-	-	-	-	-	-	-	-	-	-	-	-	-	-	-	-	-	-	-	-	-	-	-	-	-	-
8761	-	-	-	OTTDARP00000012649	-	-	-	146.m00114	ENSGALP00000027887	GL50803_135885	OTTHUMP00000166261	LmjF08.0060	-	-	52804	242088	-	Os08g37140.1	16424	GSPATP00002010001	-	127035	137597	-	655170	RO3G_16011.1	YOR283W	-	GLEAN3_28416	-	-	-	-	59.m03656	54326	-	Tb927.5.3580	UM02133.1
8547	254360	-	CMT195C	-	DDB0217276	CG4303-PA	-	-	ENSGALP00000010010	-	NP_001003801.1	-	140600	38471	-	200763	NCU03572	Os04g31320.1	-	GSPATP00010157001	-	113811	129388	-	806541	RO3G_01276.1	-	SPAC23G3.10c	-	SINFRUP00000134998	165.m00081	-	-	-	-	-	-	UM02958.1
8449	-	-	-	-	-	-	-	-	-	-	-	-	-	22722	-	-	-	Os03g07820.1	-	-	-	187754	-	-	762147	-	-	-	-	-	-	-	-	-	-	84270.m00617	-	-
8329	-	-	CMJ100C	-	-	-	-	-	-	-	-	LmjF29.2450	104895	-	-	-	-	Os03g16030.1	-	-	35158	70357	-	PF13_0021	563962	-	-	SPCC338.06c	-	SINFRUP00000179785	-	-	-	76.m01677	-	-	-	-
8323	-	-	-	-	-	-	-	-	-	-	-	-	-	-	-	-	-	-	-	-	-	-	-	-	-	-	-	-	-	-	-	-	-	-	-	-	-	-
8155	-	-	CMR472C	-	-	-	-	-	ENSGALP00000002018	-	OTTHUMP00000109529	-	-	-	-	-	-	Os07g46690.1	-	GSPATP00002737001	43759	28493	-	PF11_0429	172196	-	YMR075W	SPAC16C9.05	-	-	126.m00135	-	-	-	-	-	-	-
8129	259841	-	-	OTTDARP00000023416	-	CG7462-PC	-	-	ENSGALP00000019635	-	OTTHUMP00000163834	-	-	-	-	112951	-	-	22642	-	42811	-	131193	-	-	RO3G_00417.1	YGR233C	-	GLEAN3_04425	SINFRUP00000182968	211.m00038	-	-	55.m04924	-	97076.m00020	-	-
7933	-	-	-	-	-	-	-	-	-	-	-	-	-	-	-	-	-	-	-	-	-	-	-	-	-	-	-	-	-	-	-	-	-	-	-	-	-	-
7632	251170	cgd1_2500	-	-	DDB0186701	CG5064-PA	-	-	ENSGALP00000003205	-	OTTHUMP00000182488	LmjF34.2790	106123	39183	57981	131967	-	Os08g23360.2	34049	GSPATP00023673001	48430	196704	158774	-	835242	RO3G_11681.1	-	SPCC1682.05c	GLEAN3_24348	SINFRUP00000143458	147.m00090	23270	TA11190	55.m04737	52953	83413.m00046	Tb927.4.1850	UM01157.1
7611	-	-	CMN259C	OTTDARP00000016450	-	CG32056-PC	-	-	-	-	-	-	-	-	75212	-	-	Os04g51080.1	-	-	-	150195	-	-	252175	RO3G_13014.1	YJR100C	SPAC343.06c	GLEAN3_03914	-	-	-	-	-	-	-	-	UM06031.1
7539	296227	cgd5_4290	CMO092C	OTTDARP00000016498	DDB0205584	-	19171288	294.m00049	ENSGALP00000010093	GL50803_87205	OTTHUMP00000019581	LmjF14.0840	108551	38338	82093	83656	NCU07837	Os01g01312.1	15472	-	24775	77632	133358	-	205910	RO3G_12223.1	YJR035W	SPCP25A2.02c	GLEAN3_24896	SINFRUP00000151604	-	267997	-	55.m05039	53632	84178.m00075	Tb927.7.4080	-
7341	-	-	-	-	-	-	-	-	-	-	-	-	-	-	-	-	-	-	-	-	-	-	-	-	-	-	-	-	-	-	-	-	-	-	-	-	-	-
-	-	-	-	-	-	-	-	-	-	-	-	-	-	-	-	-	-	-	-	-	-	-	-	-	-	-	-	-	-	-	-	-	-	-	-	-	-	-
7088	-	-	-	-	DDB0188522	-	-	-	-	-	-	-	-	-	-	-	-	-	-	-	-	-	-	-	-	-	-	-	-	-	-	-	-	-	-	-	-	-
6964	284381	-	CMO229C	-	DDB0205339	CG30499-PA	-	53.m00211	ENSGALP00000033317	GL50803_10324	OTTHUMP00000163938	LmjF33.1570	230485	-	30757	158372	NCU00519	Os09g32810.1	17919	GSPATP00024341001	20015	158635	108981	PFL0960w	815572	RO3G_08745.1	YJL121C	SPAC31G5.05c	GLEAN3_18907	SINFRUP00000137316	61.m00196	-	-	50.m00038	21066	85715.m00030	Tb10.389.1530	UM04797.1
6823	261012	-	-	OTTDARP00000014335	-	CG30426-PA	-	-	-	GL50803_221691	-	-	-	-	-	-	-	Os09g19830.1	-	-	-	174853	-	-	264791	-	-	-	-	-	-	-	-	-	61660	-	-	-
6783	-	-	-	-	-	-	-	-	-	-	-	-	-	14642	-	-	-	Os04g47830.1	17347	-	17728	147434	130403	-	566361	-	-	-	-	-	-	-	-	-	-	-	-	-
6466	260092	-	-	-	-	-	-	-	ENSGALP00000026375	GL50803_32375	NP_663622.1	LmjF36.4730	227074	32998	-	214129	-	-	-	GSPATP00006872001	-	-	135715	-	-	-	-	-	-	SINFRUP00000164273	47.m00258	-	-	-	30536	87759.m00074	Tb10.6k15.0190	-
6366	297238	-	-	-	-	CG7127-PA	-	48.m00186	ENSGALP00000003183	-	OTTHUMP00000041941	-	210312	-	56566	83918	-	Os04g58880.1	-	-	-	161804	137766	-	765130	-	-	SPBC106.20	GLEAN3_12639	SINFRUP00000131960	-	-	-	-	53055	-	-	UM01845.1
6185	-	-	-	-	-	-	-	-	-	-	-	-	-	-	-	-	-	-	-	-	-	-	-	-	-	-	-	-	-	-	-	-	-	-	-	-	-	-
5762	288116	-	CMS020C	-	DDB0205790	-	19069239	405.m00046	ENSGALP00000020267	GL50803_14259	OTTHUMP00000028332	LmjF28.3005	180290	27826	29070	107874	NCU01902	Os09g31310.1	7806	GSPATP00011587001	-	206554	-	-	669373	RO3G_16355.1	YFL017C	SPAC16E8.03	-	SINFRUP00000159670	211.m00033	-	-	-	33438	83473.m00061	Tb11.01.2886	UM05957.1
5437	-	-	CMT220C	-	-	-	-	-	-	-	-	-	-	-	-	-	-	Os05g28200.1	-	-	12807	113036	-	-	819222	-	-	-	-	-	-	262279	-	-	-	-	-	-
5404	298863	cgd3_2700	CMN321C	OTTDARP00000021524	DDB0204612	-	-	-	ENSGALP00000016493	-	OTTHUMP00000027987	-	220856	-	32491	-	NCU06212	Os09g06970.1	37767	-	48567	142331	127057	PFF0905w	834509	RO3G_12899.1	YOR115C	SPAC13G6.05c	GLEAN3_27693	SINFRUP00000153375	97.m00106	3516	TA12490	25.m01845	30075	-	Tb927.8.6900	UM04017.1
5137	-	-	CMV018C	-	-	-	-	-	-	-	-	-	-	-	-	-	NCU01666	Os11g14950.1	2369	-	-	140003	134172	-	419949	RO3G_02885.1	YCL009C	SPBC14C8.04	-	-	-	-	-	-	-	-	-	UM05021.1
4738	205848	-	-	-	DDB0184247	-	-	-	ENSGALP00000032355	GL50803_102890	OTTHUMP00000020680	-	223691	24711	62982	226653	NCU09744	Os03g33580.1	-	-	32949	106330	132132	-	202067	-	-	-	-	SINFRUP00000160816	-	3058	TA14430	50.m03067	53336	80604.m00064	-	UM04052.1
3897	-	-	-	OTTDARP00000004054	-	CG6444-PA	-	-	ENSGALP00000017260	GL50803_4538	OTTHUMP00000158573	LmjF13.1350	118956	-	69688	239717	NCU03037	-	-	GSPATP00001134001	50120	66469	157197	-	-	-	-	SPCC18.11c	-	SINFRUP00000129453	6.m00346	262700	-	59.m07790	57103	86088.m00385	Tb11.02.0990	-
3686	299423	-	-	-	-	-	-	-	ENSGALP00000026751	GL50803_12157	OTTHUMP00000155184	-	224445	9396	4062	203554	-	-	-	GSPATP00014509001	-	-	108475	-	-	-	-	-	GLEAN3_02507	SINFRUP00000133528	40.m00258	36553	-	-	-	92876.m00121	Tb11.52.0014	-
2983	291013	cgd6_1760	-	OTTDARP00000020583	DDB0219468	CG5373-PA	-	6.m00453	ENSGALP00000038771	GL50803_17406	OTTHUMP00000073689	LmjF24.2010	181298	20335	67703	91901	NCU00656	Os05g08810.1	16281	GSPATP00022039001	30282	182554	129311	PFE0765w	806443	RO3G_07088.1	YLR240W	SPAC458.05	GLEAN3_22717	SINFRUP00000173534	94.m00170	36937	-	33.m01391	22684	91714.m00087	Tb927.8.6210	UM00453.1
2180	-	-	-	-	-	-	-	-	-	-	-	-	-	-	-	-	-	-	-	-	-	-	-	-	-	-	-	-	-	-	-	-	-	-	-	-	-	-
1807	262353	-	-	OTTDARP00000022515	-	CG7324-PA	-	-	ENSGALP00000027032	-	OTTHUMP00000018752	-	187086	33605	69318	-	NCU00681	Os08g25010.1	33758	-	-	107348	-	PF11_0151	824346	RO3G_07207.1	YGR100W	SPAC4G8.04	GLEAN3_21340	SINFRUP00000162937	-	-	-	-	51695	-	-	UM00935.1
1670	-	cgd2_620	-	-	DDB0204435	CG10130-PA	-	-	-	-	-	-	140618	-	-	-	-	Os12g01260.1	30425	-	-	29562	141671	MAL8P1.51	644302	RO3G_10062.1	-	SPBC2G2.03c	-	-	-	-	-	27.m01477	35611	-	-	-
96513	-	-	-	-	-	-	-	-	-	-	-	-	-	-	-	-	-	-	-	-	-	-	-	-	-	-	-	-	-	-	-	-	-	-	-	-	-	-
96506	-	-	-	-	-	-	-	-	-	-	-	-	-	-	-	-	-	-	-	-	-	-	-	-	-	-	-	-	-	-	-	-	-	-	-	-	-	-
-	-	-	-	-	-	-	-	-	-	-	-	-	-	-	-	-	-	-	-	-	-	-	-	-	-	-	-	-	-	-	-	-	-	-	-	-	-	-
96492	-	-	-	-	-	-	-	-	-	-	-	-	-	-	-	-	-	-	-	-	-	-	-	-	-	-	-	-	-	-	-	-	-	-	-	-	-	-
96488	-	cgd1_1120	-	-	-	-	-	-	ENSGALP00000011137	-	OTTHUMP00000181449	-	-	-	59583	219473	-	Os01g13160.1	-	-	-	228352	139866	PF14_0569	705784	-	-	-	-	-	-	-	-	162.m00325	62152	88601.m00410	-	-
96487	-	-	-	-	-	-	-	-	-	-	-	-	-	-	-	-	-	-	-	-	-	-	-	-	-	-	-	-	-	-	-	-	-	-	-	-	-	-
96485	-	-	-	-	-	-	-	-	-	-	-	-	-	-	-	-	-	-	-	-	-	-	-	-	-	-	-	-	-	-	-	-	-	-	-	-	-	-
96482	-	-	CMR312C	-	-	-	-	-	-	-	-	-	-	-	-	-	-	-	-	-	45498	-	-	-	-	-	-	-	-	-	-	-	-	-	-	-	-	-
96477	-	-	-	-	-	-	-	-	-	-	-	-	-	-	-	-	-	-	-	-	-	-	-	-	-	-	-	-	-	-	-	-	-	-	-	-	-	-
96475	-	-	-	-	-	-	-	-	-	-	-	-	-	-	-	-	-	-	-	-	-	-	-	-	-	-	-	-	-	-	-	-	-	-	-	-	-	-
96469	-	-	-	-	-	-	-	-	-	-	-	-	-	-	-	-	-	-	-	-	-	-	-	-	-	-	-	-	-	-	-	-	-	-	-	-	-	-
96467	-	-	-	-	-	-	-	-	-	-	-	-	-	-	-	-	-	-	-	-	-	-	-	-	-	-	-	-	-	-	-	-	-	-	-	-	-	-
96463	-	-	-	-	-	-	-	-	-	-	-	-	-	-	-	-	-	-	-	-	-	-	-	-	-	-	-	-	-	-	-	-	-	-	-	-	-	-
96460	-	-	-	-	-	-	-	-	-	-	-	-	-	-	-	-	-	-	-	-	-	-	-	-	-	-	-	-	-	-	-	-	-	-	-	-	-	-
-	-	-	-	-	-	-	-	-	-	-	-	-	-	-	-	-	-	-	-	-	-	-	-	-	-	-	-	-	-	-	-	-	-	-	-	-	-	-
96409	-	-	-	-	-	-	-	-	-	-	-	-	-	-	-	-	-	-	-	-	-	-	-	-	-	-	-	-	-	-	-	-	-	-	-	-	-	-
96408	-	-	-	-	-	-	-	-	-	-	-	-	-	-	-	-	-	-	-	-	-	-	-	-	-	-	-	-	-	-	-	-	-	-	-	-	-	-
96400	-	-	-	-	-	-	-	-	-	-	-	-	-	-	-	-	-	-	-	-	-	-	-	-	-	-	-	-	-	-	-	-	-	-	-	-	-	-
96394	-	-	-	-	-	-	-	-	-	-	-	-	-	-	61854	-	-	Os11g08660.1	-	-	-	111080	-	-	204634	-	-	-	-	-	-	4422	-	-	-	-	-	-
96389	-	-	-	-	-	-	-	-	-	-	-	-	-	-	-	-	-	-	-	-	-	-	-	-	-	-	-	-	-	-	-	-	-	-	-	-	-	-
96388	-	cgd4_1070	-	-	-	-	-	-	-	-	-	LmjF36.3540	-	-	-	-	-	-	30003	-	45182	-	127687	PFF0105w	-	-	-	-	-	-	-	-	TA21455	583.m05392	-	-	Tb11.01.1670	-
96374	-	-	-	-	-	-	-	-	-	-	-	-	-	-	-	-	-	-	-	-	-	-	-	-	-	-	-	-	-	-	-	-	-	-	-	-	-	-
96369	-	-	-	-	-	-	-	-	-	-	-	-	-	-	-	-	-	-	-	-	-	-	-	-	-	-	-	-	-	-	-	-	-	-	-	-	-	-
96367	-	-	-	-	-	-	-	-	-	-	-	-	-	-	-	-	-	-	-	-	-	-	-	-	-	-	-	-	-	-	-	-	-	-	-	-	-	-
96364	-	-	-	-	-	-	-	-	-	-	-	-	-	-	-	-	-	-	-	-	-	-	-	-	-	-	-	-	-	-	-	-	-	-	-	-	-	-
96308	-	-	-	-	-	-	-	-	-	-	-	-	-	-	-	-	-	-	-	-	-	-	-	-	-	-	-	-	-	-	-	-	-	-	-	-	-	-
96306	-	-	-	-	-	-	-	-	-	-	-	-	-	-	-	-	-	-	-	-	-	-	-	-	-	-	-	-	-	-	-	-	-	-	-	-	-	-
96298	-	-	-	-	-	-	-	-	-	-	-	-	-	-	-	-	-	-	-	-	-	-	-	-	-	-	-	-	-	-	-	-	-	-	-	-	-	-
96296	206663	-	-	-	DDB0185298	CG6762-PA	-	-	-	-	OTTHUMP00000029936	-	205973	-	67173	87155	-	Os06g07760.1	-	-	-	139066	-	-	814302	RO3G_07562.1	YKL086W	SPBC106.02c	-	SINFRUP00000130326	-	-	-	-	-	-	-	-
96293	-	-	-	-	-	-	-	-	-	-	-	-	-	-	-	-	-	-	-	-	-	-	-	-	-	-	-	-	-	-	-	-	-	-	-	-	-	-
96289	-	-	-	-	-	-	-	-	-	-	-	-	-	-	-	-	-	-	-	-	-	-	-	-	-	-	-	-	-	-	-	-	-	-	-	-	-	-
96283	-	-	-	-	-	-	-	-	-	-	-	-	-	-	-	-	-	-	-	-	-	-	-	-	-	-	-	-	-	-	-	-	-	-	-	-	-	-
96274	-	-	-	-	-	-	-	-	-	-	-	-	-	-	-	-	-	-	-	-	-	-	-	-	-	-	-	-	-	-	-	-	-	-	-	-	-	-
96264	-	-	-	-	-	-	-	-	-	-	-	-	-	-	-	-	-	-	-	-	-	-	-	-	-	-	-	-	-	-	-	-	-	-	-	-	-	-
96215	-	-	-	-	-	-	-	-	-	-	-	-	-	-	-	-	-	-	-	-	-	-	-	-	-	-	-	-	-	-	-	-	-	-	-	-	-	-
96210	-	-	-	-	-	-	-	-	-	-	-	-	-	-	-	-	-	-	-	-	-	-	-	-	-	-	-	-	-	-	-	-	-	-	-	-	-	-
96206	-	-	-	-	-	-	-	-	-	-	-	-	-	-	-	-	-	-	-	-	-	-	-	-	-	-	-	-	-	-	-	-	-	-	-	-	-	-
96182	-	-	-	-	-	-	-	-	-	-	-	-	-	-	-	-	-	-	-	-	-	-	-	-	-	RO3G_05567.1	-	-	-	-	-	-	-	-	-	-	-	-
96175	-	-	-	-	-	-	-	-	-	-	-	-	-	-	-	-	-	-	-	-	-	-	-	-	-	-	-	-	-	-	-	-	-	-	-	-	-	-
96162	292849	cgd1_1720	-	-	DDB0186533	-	-	-	ENSGALP00000017892	GL50803_32741	OTTHUMP00000036048	LmjF32.2650	-	22775	31998	92540	-	Os03g01110.1	-	GSPATP00008654001	8000	-	158551	PF14_0273	589922	RO3G_02261.1	YDL112W	-	-	SINFRUP00000148251	18.m00281	22808	-	38.m01087	5097	-	Tb11.01.7490	-
96154	-	-	-	-	-	-	-	-	-	-	-	-	-	-	-	-	-	-	-	-	-	-	-	-	-	-	-	-	-	-	-	-	-	-	-	-	-	-
96152	-	-	-	-	-	-	-	-	-	-	-	-	-	-	-	-	-	-	-	-	-	-	-	-	-	-	-	-	-	-	-	-	-	-	-	-	-	-
96140	-	-	-	-	-	-	-	-	-	-	-	-	-	39190	-	-	-	-	-	-	-	-	-	-	-	-	-	-	-	-	-	23215	-	-	-	-	-	-
96123	-	-	-	-	-	-	-	-	-	-	-	-	-	-	-	-	-	-	31342	-	47851	160481	-	-	-	-	-	-	-	-	-	-	-	-	-	-	-	-
96122	-	-	-	-	-	-	-	-	-	-	-	-	-	-	-	-	-	-	-	-	-	-	-	-	-	-	-	-	-	-	-	-	-	-	-	-	-	-
96121	-	-	-	-	-	-	-	-	-	-	-	-	-	-	-	-	-	-	-	-	-	-	-	-	-	-	-	-	-	-	-	-	-	-	-	-	-	-
96117	-	-	-	-	-	-	-	-	-	-	-	-	-	-	-	-	-	-	-	-	-	-	-	-	-	-	-	-	-	-	-	-	-	-	-	-	-	-
96092	-	-	-	-	-	-	-	-	-	-	-	-	-	-	-	-	-	-	-	-	-	-	-	-	-	-	-	-	-	-	-	-	-	-	-	-	-	-
96073	-	-	-	-	-	-	-	-	-	-	-	-	-	-	-	-	-	-	-	-	-	-	-	-	-	-	-	-	-	-	-	-	-	-	-	-	-	-
-	-	-	-	-	-	-	-	-	-	-	-	-	-	-	-	-	-	-	-	-	-	-	-	-	-	-	-	-	-	-	-	-	-	-	-	-	-	-
96058	-	-	-	-	-	-	-	-	-	-	-	-	-	-	-	-	-	-	-	-	-	-	-	-	-	-	-	-	-	-	-	-	-	-	-	-	-	-
96044	-	-	-	-	-	-	-	-	-	-	-	-	-	-	-	-	-	-	-	-	-	-	-	-	-	-	-	-	-	-	-	-	-	-	-	-	-	-
96038	-	-	-	-	-	-	-	-	-	-	-	-	-	-	-	-	-	-	-	-	-	-	-	-	-	-	-	-	-	-	-	-	-	-	-	-	-	-
96027	-	-	-	-	-	-	-	-	-	-	-	-	-	-	-	-	-	-	-	-	-	-	-	-	-	-	-	-	-	-	-	-	-	-	-	-	-	-
96026	-	-	-	-	-	-	-	-	-	-	-	-	-	-	-	-	-	-	-	-	-	-	-	-	-	-	-	-	-	-	-	-	-	-	-	-	-	-
96020	-	-	-	-	-	-	-	-	-	-	-	-	-	-	-	-	-	-	-	-	-	-	-	-	-	-	-	-	-	-	-	-	-	-	-	-	-	-
96017	-	-	-	-	-	-	-	-	-	-	-	-	-	-	-	-	-	-	-	-	-	-	-	-	-	-	-	-	-	-	-	-	-	-	-	-	-	-
96016	-	-	CMB012C	-	DDB0205843	-	19171416	-	-	-	OTTHUMP00000032179	-	-	22893	-	245107	NCU06568	-	10226	-	46838	-	133253	-	-	-	YOL069W	SPAC27F1.04c	-	-	-	11958	-	-	-	-	-	UM04224.1
96010	-	-	-	-	-	-	-	-	-	-	-	-	-	-	-	-	-	-	-	-	-	-	-	-	-	-	-	-	-	-	-	-	-	-	-	-	-	-
96009	-	-	-	-	-	-	-	-	-	-	-	-	-	-	-	-	-	-	-	-	-	-	-	-	-	-	-	-	-	-	-	-	-	-	-	-	-	-
96006	-	-	-	-	-	-	-	-	-	-	-	-	-	-	-	-	-	-	-	-	-	-	-	-	-	-	-	-	-	-	-	-	-	-	-	-	-	-
96004	-	-	-	-	-	-	-	-	-	-	-	-	-	-	-	-	-	-	-	-	-	-	-	-	-	-	-	-	-	-	-	-	-	-	-	-	-	-
95996	-	-	-	-	-	-	-	-	-	-	-	-	-	-	-	-	-	-	-	-	-	-	-	-	-	-	-	-	-	-	-	-	-	-	-	-	-	-
95982	-	-	-	-	-	-	-	-	-	-	-	-	-	-	-	-	-	-	-	-	-	-	-	-	-	-	-	-	-	-	-	-	-	-	-	-	-	-
95979	-	-	-	-	-	-	-	-	-	-	-	-	-	-	-	-	-	-	-	-	-	-	-	-	-	-	-	-	-	-	-	-	-	-	-	-	-	-
95973	-	-	-	-	-	-	-	-	-	-	-	-	-	-	-	-	-	-	-	-	-	-	-	-	-	-	-	-	-	-	-	-	-	-	-	-	-	-
95969	-	-	-	-	-	-	-	-	-	-	-	-	-	-	-	-	-	-	-	-	-	-	-	-	-	-	-	-	-	-	-	-	-	-	-	-	-	-
95958	-	-	-	-	-	-	-	-	-	-	-	-	-	-	-	-	-	-	-	-	-	-	-	-	-	-	-	-	-	-	-	-	-	-	-	-	-	-
95946	-	-	-	-	-	-	-	-	-	-	-	-	-	-	-	-	-	-	-	-	-	-	-	-	-	-	-	-	-	-	-	-	-	-	-	-	-	-
95938	-	-	-	-	-	-	-	-	-	-	-	-	-	-	-	-	-	-	-	-	-	-	-	-	-	-	-	-	-	-	-	3963	-	-	-	-	-	-
95928	254009	-	CMC118C	-	DDB0186234	CG6404-PB	-	-	ENSGALP00000037065	-	NP_005006.1	-	123644	1400	31420	33386	NCU01102	Os10g37690.1	33312	GSPATP00001160001	9161	172115	127695	-	560645	RO3G_04995.1	YER154W	SPAC9G1.04	-	SINFRUP00000178313	-	262457	-	-	23561	-	Tb11.02.4020	UM02457.1
95911	-	-	-	-	-	-	-	-	-	-	-	-	-	-	-	-	-	-	-	-	-	-	-	-	-	-	-	-	-	-	-	-	-	-	-	-	-	-
95895	-	-	-	-	-	-	-	-	-	-	-	-	-	-	-	-	-	-	-	-	-	-	-	-	-	-	-	-	-	-	-	-	-	-	-	-	-	-
95892	-	-	-	-	-	-	-	129.m00133	-	-	-	-	-	-	81522	-	-	-	-	-	-	-	-	-	-	-	-	-	-	-	-	-	-	-	-	-	-	-
95891	-	-	-	-	-	-	-	-	-	-	-	-	-	-	-	-	-	-	-	-	-	-	-	-	-	-	-	-	-	-	-	-	-	-	-	-	-	-
95886	-	-	CME030C	-	-	-	-	-	-	-	NP_848597.1	LmjF27.0570	-	-	-	-	-	Os01g72480.1	-	GSPATP00014151001	-	152204	156053	-	641460	-	-	-	-	-	7.m00534	-	-	-	-	-	-	-
95880	-	-	-	-	-	-	-	-	-	-	-	-	-	-	-	-	-	-	-	-	-	-	-	-	-	-	-	-	-	-	-	-	-	-	-	-	-	-
95871	-	-	-	-	-	-	-	-	-	-	-	-	-	-	-	-	-	-	-	-	-	-	-	-	-	-	-	-	-	-	-	-	-	-	-	-	-	-
95868	-	-	-	OTTDARP00000022744	-	-	-	-	-	-	NP_001008227.1	-	63801	-	-	-	-	-	-	-	-	89099	-	-	-	-	-	-	-	-	209.m00063	-	-	-	-	-	-	-
95849	-	-	-	-	-	-	-	-	-	-	-	-	-	-	-	-	-	-	-	-	-	-	-	-	-	-	-	-	-	-	-	-	-	-	-	-	-	-
95841	-	-	-	-	-	-	-	-	-	-	-	-	-	-	-	-	-	-	-	-	-	-	-	-	-	-	-	-	-	-	-	-	-	-	-	-	-	-
95840	-	-	-	-	-	-	-	-	-	-	-	-	-	-	-	-	-	-	-	-	-	-	-	-	-	-	-	-	-	-	-	-	-	-	-	-	-	-
95826	-	-	-	-	-	-	-	-	-	-	-	-	-	-	-	-	-	-	-	-	-	-	-	-	-	-	-	-	-	-	-	-	-	-	-	-	-	-
95820	-	-	-	-	-	-	-	-	-	-	-	-	-	-	-	-	-	-	-	-	-	-	-	-	-	-	-	-	-	-	-	-	-	-	-	-	-	-
95813	-	-	-	-	-	-	-	-	-	-	-	-	-	-	-	-	-	-	-	-	48834	-	-	-	-	-	-	-	-	-	-	-	-	-	-	-	-	-
95806	-	-	-	-	-	-	-	-	-	-	-	-	-	-	-	-	-	-	-	-	-	-	-	-	-	-	-	-	-	-	-	-	-	-	-	-	-	-
95788	219629	-	-	OTTDARP00000003695	-	-	-	-	-	-	OTTHUMP00000163494	-	236208	-	-	205321	-	Os12g41620.1	-	-	-	169380	-	-	578583	RO3G_07090.1	-	-	-	SINFRUP00000150371	-	-	-	-	55093	-	-	-
95782	-	-	-	-	-	-	-	-	-	-	-	-	-	-	-	-	-	-	-	-	-	-	-	-	-	-	-	-	-	-	-	-	-	-	-	-	-	-
95760	-	-	-	-	-	-	-	-	-	-	-	-	-	-	-	-	-	-	-	-	-	-	-	-	-	-	-	-	-	-	-	-	-	-	-	-	-	-
95738	-	-	-	-	-	-	-	-	-	-	-	-	-	-	-	-	-	-	-	-	-	-	-	-	-	-	-	-	-	-	-	-	-	-	-	-	-	-
95704	-	-	-	-	-	-	-	-	-	-	-	-	-	-	-	-	-	-	-	-	-	-	-	-	-	-	-	-	-	-	-	-	-	-	-	-	-	-
95693	-	-	-	-	-	-	-	-	-	-	-	-	-	-	-	-	-	-	-	-	-	-	-	-	-	-	-	-	-	-	-	5370	-	-	-	-	-	-
95682	-	-	-	-	-	-	-	-	-	-	-	-	-	-	-	-	-	-	-	-	-	-	-	-	-	-	-	-	-	-	-	-	-	-	-	-	-	-
95661	-	-	-	-	-	-	-	-	-	-	-	-	-	-	-	-	-	-	31646	-	-	169535	-	-	-	-	-	-	-	-	-	-	-	-	-	-	-	-
95650	-	-	-	-	-	-	-	-	-	-	-	-	-	-	-	-	-	-	-	-	-	-	-	-	-	-	-	-	-	-	-	-	-	-	-	-	-	-
95634	255772	cgd8_4800	CMT548C	OTTDARP00000011087	DDB0186668	CG4080-PA	19074333	-	ENSGALP00000028721	-	OTTHUMP00000168215	LmjF31.0030	66924	-	74786	189518	NCU07516	Os11g38800.2	12379	GSPATP00023811001	42799	317	142010	MAL13P1.405	720776	-	-	-	-	SINFRUP00000137967	30.m00210	-	TA08015	-	49644	-	Tb927.3.710	-
95621	-	-	-	-	-	-	-	-	-	-	-	-	-	-	-	-	-	-	-	-	-	-	-	-	-	-	-	-	-	-	-	-	-	-	-	-	-	-
95618	-	-	-	-	-	-	-	-	-	-	-	-	-	-	-	-	-	-	-	-	-	-	-	-	-	-	-	-	-	-	-	-	-	-	-	-	-	-
95598	-	-	-	-	-	-	-	-	-	-	-	-	-	-	-	-	-	-	-	-	-	-	-	-	-	-	-	-	-	-	-	-	-	-	-	-	-	-
95596	-	-	-	-	-	-	-	-	-	-	-	-	-	-	-	-	-	-	-	-	-	-	-	-	-	-	-	-	-	-	-	-	-	-	-	-	-	-
95575	-	-	-	-	-	-	-	-	-	-	-	-	-	-	-	-	-	-	-	-	-	-	-	-	-	-	-	-	-	-	-	-	-	-	-	-	-	-
95563	-	-	-	-	-	-	-	-	-	-	-	-	-	-	-	-	-	-	-	-	-	-	-	-	-	-	-	-	-	-	-	-	-	-	-	-	-	-
95558	263146	cgd7_3530	-	-	DDB0185742	CG7115-PB	-	10.m00346	ENSGALP00000008101	GL50803_14404	OTTHUMP00000181730	LmjF34.2500	174041	32120	75757	95978	-	Os02g05630.1	4137	-	-	176509	132776	PFD0505c	562025	RO3G_01402.1	-	SPCC4F11.02	GLEAN3_06132	SINFRUP00000154644	-	-	-	42.m03343	28894	86319.m00319	-	-
95549	-	-	-	-	-	-	-	-	-	-	-	-	-	-	-	-	-	-	-	-	-	-	-	-	-	-	-	-	-	-	-	-	-	-	-	-	-	-
95535	-	cgd1_3530	CMM078C	OTTDARP00000011906	-	-	-	-	-	-	-	-	-	-	-	-	-	-	-	-	51303	-	-	-	-	-	YNL004W	-	-	-	-	37126	-	-	-	-	-	-
95505	-	-	-	-	-	-	-	-	-	-	-	-	-	-	-	-	-	-	-	-	-	-	-	-	-	-	-	-	-	-	-	-	-	-	-	-	-	-
95498	-	-	-	-	-	-	-	-	-	-	-	-	-	-	-	-	-	-	-	-	-	-	-	-	-	-	-	-	-	-	-	-	-	-	-	-	-	-
95493	-	-	-	-	-	-	-	-	-	-	-	-	-	-	-	-	-	-	-	-	-	-	-	-	-	-	-	-	-	-	-	-	-	-	-	-	-	-
95490	210685	-	-	OTTDARP00000019498	-	CG32435-PC	-	-	ENSGALP00000019009	-	NP_055912.1	-	-	32317	62120	-	-	Os04g42840.1	-	-	-	75908	157648	-	816957	-	-	-	GLEAN3_00096	SINFRUP00000163620	-	-	-	-	-	96546.m00082	-	-
95444	296484	cgd5_3090	CMT358C	OTTDARP00000022088	DDB0167228	CG11989-PD	19069231	103.m00167	-	GL50803_8707	NP_116082.1	LmjF13.0260	236022	33780	32697	230324	NCU08944	Os04g54330.3	25906	GSPATP00025824001	51345	197972	109533	PF10_0036	650021	RO3G_01187.1	YHR013C	SPAC15E1.08	-	SINFRUP00000161869	100.m00125	385	TA09345	38.m01072	5398	92876.m00138	Tb11.02.2110	UM01384.1
95439	296801	cgd3_2150	CMR006C	OTTDARP00000021604	DDB0204430	CG9267-PA	-	58.m00145	ENSGALP00000037191	-	OTTHUMP00000172510	LmjF05.0280	222938	34801	35609	140243	NCU08985	Os05g38590.2	17157	GSPATP00009568001	44831	218307	-	-	706239	-	-	SPBC19C2.15c	GLEAN3_01280	SINFRUP00000142369	70.m00143	6781	-	583.m09194	64088	-	Tb10.406.0290	UM00007.1
95438	-	-	-	-	DDB0203939	-	-	41.m00237	-	GL50803_9779	-	LmjF10.1010	-	-	45426	-	-	-	-	-	-	-	-	-	-	-	-	-	-	-	-	-	-	583.m00630	-	82952.m00173	Tb927.8.4430	UM03504.1

ELG32	exostosin-like glycosyltransferase	-	-	-	-	-	-	-	-	-	-	-	-	95408	-	-	-	-	-	-	-	-	-	-	-	-	-	-	-	-	-	-	-	-	-	-	-	-	-	-	-	-	-	-	-	-	-	-	-	-	-	-
		-	-	-	-	-	-	-	-	-	-	-	-	95378	-	-	-	-	-	-	-	-	-	-	-	-	-	-	-	-	-	-	-	-	-	-	-	-	-	-	-	-	-	-	-	-	-	-	-	-	-	-
		-	-	-	-	-	-	-	-	-	-	-	-	95372	-	-	-	-	-	-	-	-	-	-	-	-	-	-	-	-	-	-	-	-	-	-	-	-	-	-	-	-	-	-	-	-	-	-	-	-	-	-
		-	-	-	-	-	-	-	-	-	-	-	-	95371	-	-	-	-	-	-	-	-	-	-	-	-	-	-	-	-	-	-	-	-	-	-	-	-	-	-	-	-	-	-	-	-	-	-	-	-	-	-
		-	-	-	-	-	-	-	-	65603	-	-	-	95368	-	-	-	-	-	-	-	-	-	-	-	-	-	30162	-	-	-	-	-	-	-	-	-	-	-	-	-	-	-	-	-	24792	-	-	-	-	-	-
		-	-	-	-	-	-	-	-	-	-	-	-	95364	-	-	-	-	-	-	-	-	-	-	-	-	-	-	-	-	-	-	-	-	-	-	-	-	-	-	-	-	-	-	-	-	-	-	-	-	-	-
		-	-	-	-	-	-	-	-	-	-	-	-	95353	-	-	-	-	-	-	-	-	-	-	-	-	-	-	-	-	-	-	-	-	-	-	-	-	-	-	-	-	-	-	-	-	-	-	-	-	-	-
		-	-	-	-	-	-	-	-	-	-	-	-	95328	-	-	-	-	-	-	-	-	-	-	-	-	-	-	-	-	-	-	-	-	-	-	-	-	-	-	-	-	-	-	-	-	-	-	-	-	-	-
		-	-	-	-	-	-	-	-	-	-	-	-	95324	-	-	-	-	-	-	-	-	-	-	-	-	-	-	-	-	NCU02608	-	-	-	-	-	-	-	-	-	YBR271W	SPCC338.11c	-	-	-	-	-	-	-	-	-	-
		-	-	-	-	-	-	-	-	-	-	-	-	95321	-	-	-	-	-	-	-	-	-	-	-	-	-	-	-	-	-	-	-	-	-	-	-	-	-	-	-	-	-	-	-	-	-	-	-	-	-	-
		-	-	-	-	-	-	-	-	64194	-	-	-	95316	-	-	-	-	-	-	-	-	-	-	-	-	-	-	-	-	-	-	-	-	-	-	-	-	-	-	-	-	-	-	-	-	-	-	-	-	-	-
		-	-	-	-	-	-	-	-	-	-	-	-	95308	-	-	-	-	-	-	-	-	-	-	-	-	-	-	-	-	-	-	-	-	-	-	-	-	-	-	-	-	-	-	-	-	-	-	-	-	-	-
		-	+	-	-	-	-	-	AT5G28960.1	-	-	CE32580	-	95307	241332	-	-	-	-	-	-	-	-	-	-	-	-	-	-	-	-	-	-	-	47349	165743	-	-	-	-	-	-	-	-	-	-	-	-	2213	-	-	-
		-	+	-	-	-	-	-	-	66792	BDEG_04459	-	150134	95290	-	-	-	-	-	CG17807-PA	-	-	ENSGALP00000002850	-	OTTHUMP00000024830	-	-	-	66602	34785	-	-	32263	-	-	-	-	-	-	-	-	-	-	-	-	-	-	-	-	-	-	-
		-	-	-	-	-	-	-	-	-	-	-	-	95285	-	-	-	-	-	-	-	-	-	-	-	-	-	-	-	-	-	-	-	-	-	-	-	-	-	-	-	-	-	-	-	-	-	-	-	-	-	-
		-	-	-	-	-	-	-	-	-	-	-	-	95280	-	-	-	-	-	-	-	-	-	-	-	-	-	-	-	-	-	-	-	-	-	-	-	-	-	-	-	-	-	-	-	-	-	-	-	-	-	-
		-	-	-	-	-	-	-	-	-	-	-	-	95279	-	-	-	-	-	-	-	-	-	-	-	-	-	-	-	-	-	-	-	-	-	-	-	-	-	-	-	-	-	-	-	-	-	-	-	-	-	-
		-	-	-	-	-	-	-	-	-	-	-	-	95269	-	-	-	-	-	-	-	-	-	-	-	-	-	-	-	-	-	Os03g57320.1	14411	-	-	182059	-	-	735220	-	-	-	-	-	-	-	-	-	-	-	-	-
		-	-	-	-	-	-	-	-	-	-	-	-	95267	-	-	-	-	-	-	-	-	-	-	-	-	-	-	-	-	-	Os02g54860.2	-	-	-	21854	-	-	262456	-	-	-	-	-	-	-	-	-	-	-	-	-
HBP1	Histone gene transcript 5' hairpin-binding protein	-	+	-	-	-	-	GB13630-PA	-	17941	BDEG_04581	CE01616	219525	95266	-	cgd7_2890	-	-	DDB0187841	-	-	15.m00358	ENSGALP00000031392	-	OTTHUMP00000113498	LmjF25.1830	124693	29745	-	81738	-	-	23366	-	43529	170461	156564	PFI1355w	-	-	-	-	GLEAN3_24780	SINFRUP00000180666	-	268247	TA12750	583.m05269	60746	-	Tb927.3.1910	-
		-	-	-	-	-	-	-	-	-	-	-	-	95265	-	-	-	-	-	-	-	-	-	-	-	-	-	-	-	-	-	-	-	-	-	-	-	-	-	-	-	-	-	-	-	-	-	-	-	-	-	-
		-	+	-	-	-	-	GB13643-PA	-	-	-	-	220846	95256	-	-	-	-	-	CG12120-PA	-	-	-	-	-	-	231453	-	-	-	-	Os01g12230.1	-	-	-	161044	157315	-	646704	-	-	-	-	-	-	-	-	-	52543	-	-	-
		-	-	-	-	-	-	-	-	-	-	-	-	95253	-	-	-	-	-	-	-	-	-	-	-	-	-	-	-	-	-	-	-	-	-	-	-	-	-	-	-	-	-	-	-	-	-	-	-	-	-	-
XRN1	Single-stranded RNA 5'-3' exonuclease	-	+	+	-	-	-	GB10706-PA	AT5G42540.1	22633	BDEG_07379	CE32750	-	95232	259698	cgd6_2080	CMQ316C	OTTDARP00000006419	DDB0169529	CG3291-PA	19171324	5.m00404	ENSGALP00000004257	GL50803_24133	NP_001036069.1	LmjF06.0260	-	13375	81445	-	NCU06678	Os01g65220.1	17818	GSPATP00000957001	15233	201067	130875	PFI0455w	715413	-	YGL173C	SPAC17A5.14	GLEAN3_13812	SINFRUP00000133891	11.m00432	31404	TA11675	49.m03316	26537	89154.m00229	Tb927.7.4900	UM01067.1
		-	-	-	-	-	-	-	-	-	-	-	-	95228	-	-	-	-	-	-	-	-	-	-	-	-	-	-	-	-	-	-	-	-	-	-	-	-	-	-	-	-	-	-	-	-	-	-	-	-	-	-
CYP744A4	cytochrome P450, unkown function, in CYP711 clan	-	-	-	-	-	-	-	-	-	-	-	-	95196	-	-	-	-	-	-	-	-	-	-	-	-	-	-	-	28519	-	-	-	-	-	-	-	-	-	-	-	-	-	-	-	-	-	-	-	-	-	-
		-	-	-	-	-	-	-	-	-	-	-	-	95157	-	-	-	-	-	-	-	-	-	-	-	-	-	-	-	-	-	-	-	-	-	-	-	-	-	-	-	-	-	-	-	-	-	-	-	-	-	-
		-	-	-	-	-	-	-	-	-	-	-	-	95148	-	-	-	-	-	-	-	-	-	-	-	-	-	-	-	-	-	-	-	-	-	-	-	-	-	-	-	-	-	-	-	-	-	-	-	-	-	-
		-	-	-	-	-	-	-	-	-	-	-	-	95143	-	-	-	-	-	-	-	-	-	-	-	-	-	-	-	-	-	-	-	-	-	-	-	-	-	-	-	-	-	-	-	-	-	-	-	-	-	-
		-	-	-	-	-	-	-	-	-	-	-	-	95135	-	-	-	-	-	-	-	-	-	-	-	-	-	-	-	-	-	-	-	-	-	-	-	-	-	-	-	-	-	-	-	-	-	-	-	-	-	-
		-	-	-	-	-	-	-	-	-	-	-	-	95118	-	-	-	-	-	-	-	-	-	-	-	-	-	-	-	-	-	-	-	-	-	-	-	-	-	-	-	-	-	-	-	-	-	-	-	-	-	-
		-	-	-	-	-	-	-	-	-	-	-	-	95117	-	-	-	-	-	-	-	-	-	-	-	-	-	-	-	-	-	-	-	-	-	-	-	-	-	-	-	-	-	-	-	-	-	-	-	-	-	-
		-	-	-	-	-	-	-	-	-	-	-	-	95108	-	-	-	-	-	-	-	-	-	-	-	-	-	-	-	-	-	-	-	-	-	-	-	-	-	-	-	-	-	-	-	-	-	-	-	-	-	-
		-	-	-	-	-	-	-	-	-	-	-	-	95092	-	-	-	-	-	-	-	-	-	-	-	-	-	-	-	-	-	-	-	-	-	-	-	-	-	-	-	-	-	-	-	-	-	-	-	-	-	-
		-	-	-	-	-	-	-	-	-	-	-	-	95072	-	-	-	-	-	-	-	-	-	-	-	-	-	-	-	-	-	-	-	-	-	-	-	-	-	-	-	-	-	-	-	-	-	-	-	-	-	-
		-	+	-	-	-	-	GB13291-PA	AT1G34350.1	24962	-	-	92248	95068	-	-	-	OTTDARP00000023001	-	CG30051-PB	-	-	ENSGALP00000026348	-	NP_690047.2	-	157958	22697	64002	218187	-	Os08g01350.1	11030	-	31918	125810	136861	-	826263	-	-	-	-	-	-	33950	-	-	33664	-	-	-
		-	-	-	-	-	-	-	-	-	-	-	-	95062	-	-	-	-	-	-	-	-	-	-	-	-	-	-	-	-	-	-	-	-	-	-	-	-	-	-	-	-	-	-	-	-	-	-	-	-	-	-
		-	+	-	-	-	-	-	AT1G19990.1	-	-	-	-	95055	-	-	CMX002C	-	-	-	-	-	-	-	-	-	-	-	-	-	-	Os05g51754.1	23370	GSPATP00001476001	37443	82929	128082	PFL0095c	652078	-	-	-	-	-	110.m00150	8827	TA08095	-	-	-	-	-
		-	+	-	-	-	-	GB13530-PA	AT2G39440.1	-	-	-	152358	95050	-	-	-	-	-	CG30105-PA	-	-	-	-	NP_115569.2	-	203942	28733	-	195792	-	Os02g07240.1	-	-	-	80138	-	-	586752	-	-	SPAC12B10.15c	-	-	-	-	-	-	57135	-	-	-
		+	+	-	-	-	-	GB11626-PA	-	-	-	-	180232	95044	286465	-	-	-	-	CG8116-PA	-	-	ENSGALP00000008149	-	NP_057583.2	LmjF34.0705	204590	-	-	175269	-	-	-	-	-	-	-	-	-	-	-	-	-	SINFRUP00000161502	147.m00088	-	-	-	-	82360.m00251	-	-
		-	-	-	-	-	-	-	-	-	-	-	-	95040	-	-	-	-	-	-	-	-	-	-	-	-	-	-	-	-	-	-	-	-	-	-	-	-	-	-	-	-	-	-	-	-	-	-	-	-	-	-
		-	-	-	-	-	-	-	-	-	-	-	-	95038	-	-	-	-	-	-	-	-	-	-	-	-	-	-	-	-	-	-	-	-	-	-	-	-	-	-	-	-	-	-	-	-	-	-	-	-	-	-
		-	-	-	-	-	-	-	-	-	-	-	-	95030	-	-	-	-	-	-	-	-	-	-	-	-	-	-	-	-	-	-	-	-	-	-	-	-	-	-	-	-	-	-	-	-	-	-	-	-	-	-
		-	-	-	-	-	-	-	-	-	-	-	-	95022	-	-	-	-	-	-	-	-	-	-	-	-	-	-	-	-	-	-	-	-	-	-	-	-	-	-	-	-	-	-	-	-	-	-	-	-	-	-
		-	-	-	-	-	-	-	-	-	-	-	-	95015	-	-	-	-	-	-	-	-	-	-	-	-	-	-	-	-	-	-	-	-	-	-	-	-	-	-	-	-	-	-	-	-	-	-	-	-	-	-
		-	+	-	-	-	-	-	-	-	-	-	-	95000	-	-	-	-	DDB0205583	-	-	-	-	-	-	-	-	-	-	-	NCU00365	Os03g47060.1	34490	-	-	-	-	-	651162	-	-	-	-	-	-	-	-	59.m06055	-	-	-	-
		-	-	-	-	-	-	-	-	-	-	-	-	94998	-	-	-	-	-	-	-	-	-	-	-	-	-	-	-	-	-	-	-	-	-	-	-	-	-	-	-	-	-	-	-	-	-	-	-	-	-	-
		-	-	-	-	-	-	-	-	-	-	-	-	94992	-	-	-	-	-	-	-	-	-	-	-	-	-	-	-	-	-	-	-	-	-	-	-	-	-	-	-	-	-	-	-	-	-	-	-	-	-	-
		-	-	-	-	-	-	-	-	-	-	-	-	94991	-	-	-	-	-	-	-	-	-	-	-	-	-	-	-	-	-	-	-	-	-	-	-	-	-	-	-	-	-	-	-	-	-	-	-	-	-	-
		-	+	-	-	-	-	-	AT4G38225.1	-	-	-	-	94979	-	-	CMM260C	-	-	-	-	-	-	-	-	-	-	-	-	-	-	Os03g03670.1	14340	-	-	127569	-	-	293964	-	-	-	-	-	-	-	-	-	-	-	-	-
		-	-	-	-	-	-	-	-	-	-	-	-	94934	-	-	-	-	-	-	-	-	-	-	-	-	-	-	-	-	-	-	-	-	-	-	-	-	-	-	-	-	-	-	-	-	-	-	-	-	-	-
		-	+	-	-	-	-	-	-	-	-	-	50626	94928	369369	-	-	OTTDARP00000004533	-	-	-	-	ENSGALP00000036861	-	OTTHUMP00000028505	-	-	-	-	-	-	-	-	-	-	168992	-	-	-	RO3G_06599.1	YOR194C	-	GLEAN3_09651	SINFRUP00000145656	-	-	-	-	61892	-	-	UM05183.1
		-	-	-	-	-	-	-	-	-	-	-	-	94915	-	-	-	-	-	-	-	-	-	-	-	-	-	-	-	-	-	-	-	-	-	-	-	-	-	-	-	-	-	-	-	-	-	-	-	-	-	-
		-	+	-	-	-	-	GB13577-PA	AT1G01760.1	-	BDEG_00702	-	224434	94911	293221	-	-	OTTDARP00000021825	DDB0218125	CG16889-PA	-	4.m00641	ENSGALP00000001334	-	OTTHUMP00000174951	-	231337	-	78282	90751	-	Os04g58690.3	35410	-	31980	144740	-	-	731067	-	-	-	GLEAN3_08095	SINFRUP00000158302	41.m00286	-	-	-	55696	82576.m00119	Tb10.406.0070	UM05895.1
		-	-	-	-	-	-	-	-	-	-	-	-	94907	-	-	-	-	-	-	-	-	-	-	-	-	-	-	-	-	-	-	-	-	-	-	-	-	-	-	-	-	-	-	-	-	-	-	-	95045.m00076	-	-
		-	-	-	-	-	-	-	-	-	-	-	-	94897	-	-	-	-	-	-	-	-	-	-	-	-	-	-	-	-	-	-	-	-	-	-	-	-	-	-	-	-	-	-	-	-	-	-	-	-	-	-
		-	-	-	-	-	-	-	-	-	-	-	-	94891	-	-	-	-	-	-	-	-	-	-	-	-	-	-	-	-	-	-	-	-	-	-	-	-	-	-	-	-	-	-	-	-	-	-	-	-	-	-
		-	-	-	-	-	-	-	-	-	-	-	-	94881	-	-	-	-	-	-	-	-	-	-	-	LmjF35.5160	-	-	46095	-	-	-	-	-	-	-	-	-	-	-	-	-	-	-	-	-	-	-	-	-	Tb09.211.3690	-
		-	-	-	-	-	-	-	-	-	-	-	-	94878	-	-	-	-	-	-	-	-	-	-	-	-	-	-	-	-	-	-	-	-	-	-	-	-	-	-	-	-	-	-	-	-	-	-	-	-	-	-
		-	-	-	-	-	-	-	-	-	-	-	-	94877	-	-	-	-	-	-	-	-	-	-	-	-	-	-	-	-	-	-	-	-	-	-	-	-	-	-	-	-	-	-	-	-	-	-	-	-	-	-
		-	-	-	-	-	-	-	-	-	-	-	-	94870	-	-	-	-	-	-	-	-	-	-	-	-	-	-	-	-	-	-	-	-	-	-	-	-	-	-	-	-	-	-	-	-	-	-	-	-	-	-
		-	+	-	-	-	-	-	-	-	-	-	-	94868	-	-	-	OTTDARP00000022445	-	-	-	-	-	-	-	-	225910	-	-	179785	NCU02336	-	-	-	-	-	134881	-	-	-	-	-	-	-	-	-	-	-	-	-	-	-
		-	-	-	-	-	-	-	-	-	-	-	-	94848	-	-	-	-	-	-	-	-	-	-	-	-	-	-	-	-	-	-	-	-	-	-	-	-	-	-	-	-	-	-	-	-	-	-	-	-	-	-
		-	-	-	-	-	-	-	-	-	-	-	-	94847	-	-	-	-	-	-	-	-	-	-	-	-	-	-	-	-	-	-	-	-	-	-	-	-	-	-	-	-	-	-	-	-	-	-	-	-	-	-
		-	-	-	-	-	-	-	-	-	-	-	-	94843	-	-	-	-	-	-	-	-	-	-	-	-	-	-	-	-	-	-	-	-	-	-	-	-	-	-	-	-	-	-	-	-	-	-	-	-	-	-
		-	-	-	-	-	-	-	-	-	-	-	-	94809	-	-	-	-	-	-	-	-	-	-	-	-	-	-	-	-	-	-	-	-	-	-	-	-	-	-	-	-	-	-	-	-	-	-	-	-	-	-
		-	-	-	-	-	-	-	-	-	-	-	-	94798	-	-	-	-	-	-	-	-	-	-	-	-	-	-	-	-	-	-	-	-	-	-	-	-	-	-	-	-	-	-	-	-	-	-	-	-	-	-
		-	-	-	-	-	-	-	-	-	-	-	-	94778	-	-	-	-	-	-	-	-	-	-	-	-	-	-	-	-	-	-	-	-	-	-	-	-	-	-	-	-	-	-	-	-	-	-	-	-	-	-
		-	-	-	-	-	-	-	-	-	-	-	-	94762	-	-	-	-	-	-	-	-	-	-	-	-	-	-	-	-	-	-	-	-	-	-	-	-	-	-	-	-	-	-	-	-	-	-	-	-	-	-
		-	-	-	-	-	-	-	-	-	-	-	-	94755	-	-	-	-	-	-	-	-	-	-	-	-	-	-	-	-	-	-	-	-	-	-	-	-	-	-	-	-	-	-	-	22830	-	-	-	-	-	-
		-	-	-	-	-	-	-	-	-	-	-	-	94750	-	-	-	-	-	-	-	-	-	-	-	-	-	-	-	-	-	-	-	-	-	-	-	-	-	-	-	-	-	-	-	-	-	-	-	-	-	-
		-	-	-	-	-	-	-	-	-	-	-	-	94742	-	-	-	-	-	-	-	-	-	-	-	-	-	-	-	-	-	-	-	-	-	-	-	-	-	-	-	-	-	-	-	-	-	-	-	-	-	-
		-	-	-	-	-	-	-	-	-	-	-	-	94741	-	-	-	-	-	-	-	-	-	-	-	-	-	-	-	-	-	-	-	-	-	-	-	-	-	-	-	-	-	-	-	-	-	-	-	-	-	-
		-	-	-	-	-	-	-	-	-	-	-	-	94705	-	-	-	-	-	-	-	-	-	-	-	-	-	-	-	-	-	-	-	-	-	-	-	-	-	-	-	-	-	-	-	-	-	-	-	-	-	-
		-	-	-	-	-	-	-	-	-	-	-	-	94700	-	-	-	-	-	-	-	-	-	-	-	-	-	-	-	-	-	-	-	-	-	-	-	-	-	-	-	-	-	-	-	-	-	-	-	-	-	-
		-	-	-	-	-	-	-	-	-	-	-	-	94697	-	-	-	-	-	-	-	-	-	-	-	-	-	-	-	-	-	-	-	-	-	-	-	-	-	-	-	-	-	-	-	-	-	-	-	-	-	-
		-	-	-	-	-	-	-	-	-	-	CE33410	-	94691	-	-	-	-	-	-	-	-	-	-	-	-	-	-	-	-	-	-	-	-	-	-	-	-	-	-	-	-	-	-	-	-	-	-	-	-	-	-
		-	-	-	-	-	-	-	-	-	-	-	-	94672	-	-	-	-	-	-	-	-	-	-	-	-	-	-	-	-	-	-	-	-	-	-	-	-	-	-	-	-	-	-	-	-	-	-	-	-	-	-
		-	-	-	-	-	-	-	-	-	-	-	-	94666	-	-	-	-	-	-	-	-	-	-	-	-	-	-	-	-	-	-	-	-	-	-	-	-	-	-	-	-	-	-	-	-	-	-	-	-	-	-
MAPKKK4	MAP Kinase Kinase Kinase Homolog 4, putative	-	-	-	-	-	-	-	-	-	-	-	-	94654	-	-	-	-	-	-	-	-	-	-	-	-	-	-	-	-	-	-	-	-	-	-	-	-	-	-	-	-	-	-	-	-	-	-	-	-	-	-
		-	-	-	-	-	-	-	-	-	-	-	-	94637	-	-	-	-	-	-	-	-	-	-	-	-	-	-	-	-	-	-	-	-	-	-	-	-	-	-	-	-	-	-	-	-	-	-	-	-	-	-
		-	-	-	-	-	-	-	-	-	BDEG_06902	-	-	94620	-	-	-	-	-	-	-	-	-	-	-	-	-	-	3578	-	-	-	-	-	-	-	-	-	-	-	-	-	-	-	-	-	-	-	-	-	-	-
		-	-	-	-	-	-	-	-	-	-	-	-	94616	-	-	-	-	-	-	-	-	-	-	-	-	-	-	-	-	-	-	-	-	-	-	-	-	-	-	-	-	-	-	-	-	-	-	-	-	-	-
		-	-	-	-	-	-	-	AT3G20790.1	-	-	-	-	94613	-	-	-	-	-	-	-	-	-	-	-	-	-	-	-	-	-	Os09g10820.1	-	-	-	125059	-	-	-	-	-	-	-	-	-	-	-	-	-	-	-	-
		-	-	-	-	-	-	-	-	-	-	-	-	94610	-	-	-	-	-	-	-	-	-	-	-	-	-	-	-	-	-	-	-	-	-	-	-	-	-	-	-	-	-	-	-	-	-	-	-	-	-	-
		-	-	-	-	-	-	-	-	-	-	-	-	94608	-	-	-	-	-	-	-	-	-	-	-	-	-	-	-	-	-	-	-	-	-	-	-	-	-	-	-	-	-	-	-	-	-	-	-	-	-	-
		-	-	-	-	-	-	-	-	-	-	-	-	94594	-	-	-	-	-	-	-	-	-	-	-	-	-	-	-	-	-	-	-	-	-	-	-	-	-	-	-	-	-	-	-	-	-	-	-	-	-	-
		-	-	-	-	-	-	-	-	-	-	-	-	94581	-	-	-	-	-	-	-	-	-	-	-	-	-	-	-	-	-	-	-	-	-	-	-	-	-	-	-	-	-	-	-	-	-	-	-	-	-	-
		-	-	-	-	-	-	-	-	-	-	-	-	94580	-	-	-	-	-	-	-	-	-	-	-	-	-	-	-	-	-	-	-	-	-	-	-	-	-	-	-	-	-	-	-	-	-	-	-	-	-	-
		-	-	-	-	-	-	-	-	-	-	-	-	94576	-	-	CMT386C	-	DDB0206194	-	-	-	-	-	-	-	-	-	-	-	-	-	-	GSPATP00020330001	-	-	-	-	-	-	-	-	-	-	225.m00034	-	-	-	-	-	-	-
		-	-	-	-	-	-	-	-	-	-	-	-	94569	-	-	-	-	-	-	-	-	-	-	-	-	-	-	-	-	-	-	-	-	-	-	-	-	-	-	-	-	-	-	-	-	-	-	-	-	-	-
		-	-	-	-	-	-	-	-	-	-	-	-	94549	-	-	-	-	-	-	-	-	-	-	-	-	-	-	-	-	-	-	-	-	-	-	-	-	-	-	-	-	-	-	-	-	-	-	-	-	-	-
		-	-	-	-	-	-	-	-	-	-	-	-	94548	-	-	-	-	-	-	-	-	-	-	-	-	-	-	-	-	-	-	-	-	-	-	-	-	-	-	-	-	-	-	-	-	-	-	-	-	-	-
		-	-	-	-	-	-	-	-	-	-	-	-	94545	-	-	-	-	-	-	-	-	-	-	-	-	-	-	-	-	-	-	-	-	-	-	-	-	-	-	-	-	-	-	-	-	-	-	-	-	-	-
		-	-	-	-	-	-	-	-	-	-	-	-	94536	-	-	-	-	-	-	-	-	-	-	-	-	-	-	-	-	-	-	-	-	-	-	-	-	-	-	-	-	-	-	-	-	-	-	-	-	-	-
		-	-	-	-	-	-	-	-	-	-	-	-	94533	-	-	-	-	-	-	-	-	-	-	-	-	-	-	-	-	-	-	-	-	-	-	-	-	-	-	-	-	-	-	-	-	-	-	-	-	-	-
		-	+	-	-	-	-	-	-	-	-	-	198746	94516	270128	cgd5_1620	-	-	-	-	-	-	ENSGALP00000025794	-	OTTHUMP00000109017	-	238097	-	-	21684	-	-	-	-	43179	-	136605	PFD0970c	-	-	-	-	-	-	-	24891	TA02495	41.m01279	-	-	-	-
		-	-	-	-	-	-	-	-	-	-	-	-	94506	-	-	-	-	-	-	-	-	-	-	-	-	-	-	-	-	-	-	-	-	-	-	-	-	-	-	-	-	-	-	-	-	-	-	-	-	-	-
		-	-	-	-	-	-	-	-	-	-	-	-	94488	-	-	-	-	-	-	-	-	-	-	-	-	-	-	-	-	-	-	-	-	-	-	-	-	-	-	-	-	-	-	-	-	-	-	-	-	-	-
		-	-	-	-	-	-	-	-	-	-	-	-	94476	-	-	-	-	-	-	-	-	-	-	-	-	-	-	-	-	-	-	-	-	-	-	-	-	-	-	-	-	-	-	-	-	-	-	-	-	-	-
		-	-	-	-	-	-	-	-	-	-	-	-	94475	-	-	-	-	-	-	-	-	-	-	-	-	-	-	-	-	-	-	-	-	-	-	-	-	-	-	-	-	-	-	-	-	-	-	-	-	-	-
		-	-	-	-	-	-	-	-	-	-	-	-	94460	-	-	-	-	-	-	-	-	-	-	-	-	-	-	-	-	-	-	-	-	-	-	-	-	-	-	-	-	-	-	-	-	-	-	-	-	-	-
		-	-	-	-	-	-	-	-	-	-	-	-	94454	-	-	-	-	-	-	-	-	-	-	-	-	-	-	-	-	-	-	-	GSPATP00012299001	-	-	131429	PF11_0268	-	RO3G_02582.1	-	-	-	-	-	-	-	-	-	-	-	-
		-	-	-	-	-	-	-	-	-	-	-	-	94452	-	-	-	-	-	-	-	-	-	-	-	-	-	-	-	-	-	-	-	-	-	-	-	-	-	-	-	-	-	-	-	-	-	-	-	-	-	-
		-	-	-	-	-	-	-	-	-	-	-	-	94447	-	-	-	-	-	-	-	-	-	-	-	-	-	-	-	-	-	-	-	-	-	-	-	-	-	-	-	-	-	-	-	-	-	-	-	-	-	-
		-	-	-	-	-	-	-	-	-	-	-	-	94429	-	-	-	-	-	-	-	-	-	-	-	-	-	-	-	-	-	-	-	-	-	-	-	-	-	RO3G_07053.1	-	-	-	-	-	-	-	-	-	-	-	-
		-	-	-	-	-	-	-	-	-	-	-	-	94422	-	-	-	-	-	-	-	-	-	-	-	-	-	-	-	-	-	-	-	-	-	-	-	-	-	-	-	-	-	-	-	-	-	-	-	-	-	-
		-	-	-	-	-	-	-	-	-	-	-	-	94396	-	-	-	-	-	-	-	-	-	-	-	-	-	-	-	-	-	-	-	-	-	-	-	-	-	-	-	-	-	-	-	-	-	-	-	-	-	-
PHC22	cell wall protein phosphorin-C22	-	-	-	-	-	-	-	-	-	-	-	-	94393	-	-	-	-	-	-	-	-	-	-	-	-	-	-	-	-	-	-	-	-	-	-	-	-	-	-	-	-	-	-	-	-	-	-	-	-	-	-
		-	+	-	-	-	-	-	AT3G03420.1	-	BDEG_00505	-	110443	94387	395227	-	CMA132C	-	-	CG5131-PA	-	-	ENSGALP00000015845	-	OTTHUMP00000168302	-	-	30281	79470	223597	NCU00107	Os04g40650.1	22147	-	12106	67986	137289	-	409058	RO3G_03849.1	YNR020C	SPCC320.12	-	SINFRUP00000164656	-	-	-	-	63972	-	-	UM04700.1
		-	-	-	-	-	-	-	-	-	-	-	-	94383	-	-	-	-	-	-	-	-	-	-	-	-	-	-	-	-	-	-	-	-	-	-	-	-	-	-	-	-	-	-	-	-	-	-	-	-	-	-
		-	-	-	-	-	-	-	AT4G31600.2	-	-	-	-	94370	248254	-	-	-	-	-	-	-	-	-	-	-	229077	-	-	-	-	-	-	-	-	16485	-	-	-	-	-	-	-	-	-	-	-	-	-	-	-	-
		-	-	-	-	-	-	-	-	-	-	-	-	94336	-	-	-	-	-	-	-	-	-	-	-	-	-	-	-	-	-	-	-	-	-	-	-	-	-	-	-	-	-	-	-	-	-	-	-	-	-	-
		-	-	-	-	-	-	-	-	-	-	-	-	94329	-	-	-	-	-	-	-	-	-	-	-	-	-	-	-	-	-	-	-	-	-	-	-	-	-	-	-	-	-	-	-	-	-	-	-	-	-	-
		-	-	-	-	-	-	-	-	-	-	-	-	94324	-	-	-	-	-	-	-	-	-	-	-	-	-	-	-	-	-	-	-	-	-	-	-	-	-	-	-	-	-	-	-	-	-	-	-	-	-	-
		-	+	-	-	-	-	-	-	-	-	CE34626	-	94296	-	-	-	OTTDARP00000002093	DDB0216964	CG33148-PB	-	-	ENSGALP00000023592	-	NP_078993.3	-	-	-	73557	-	-	-	-	-	-	-	-	-	-	RO3G_12361.1	-	-	-	-	-	-	-	-	-	-	Tb11.02.1630	-
		-	-	-	-	-	-	-	-	-	-	-	-	94286	-	-	-	-	-	-	-	-	-	-	-	-	-	-	-	-	-	-	-	-	-	-	-	-	-	-	-	-	-	-	-	-	-	-	-	-	-	-
		-	+	-	-	-	-	GB17419-PA	AT1G49590.1	-	BDEG_07288	CE18758	222399	94281	245211	-	-	-	-	CG4291-PA	-	-	-	-	-	-	239443	-	-	243326	NCU04032	Os06g11170.1	11133	-	-	147981	137138	PF14_0026	821220	-	-	SPBC18H10.07	-	-	-	-	TA10155	-	56241	-	-	UM00971.1
		-	-	-	-	-	-	-	-	-	-	-	-	94280	-	-	-	-	-	-	-	-	-	-	-	-	-	-	-	-	-	-	-	-	-	-	-	-	-	-	-	-	-	-	-	-	-	-	-	-	-	-
		-	-	-	-	-	-	-	-	-	-	-	-	94264	-	-	-	-	-	-	-	-	-	-	-	-	-	-	-	-	-	-	-	-	-	-	-	-	-	-	-	-	-	-	-	-	-	-	-	-	-	-
		-	-	-	-	-	-	-	-	-	-	-	-	94254	-	-	-	-	-	-	-	-	-	GL50803_8464	-	-	-	-	-	-	-	-	-	-	-	-	-	-	-	-	-	-	-	-	-	-	-	-	-	-	-	-
		-	+	-	-	-	-	GB14604-PA	AT1G62660.1	65284	-	-	-	94247	-	-	-	-	-	-	-	-	-	-	-	-	-	-	-	-	NCU04265	Os04g45290.2	-	-	-	106209	137802	-	-	-	YIL162W	SPCC191.11	-	-	-	-	-	-	-	88712.m00302	-	UM03605.1
		-	-	-	-	-	-	-	-	-	-	-	-	94245	-	-	-	-	-	-	-	-	-	-	-	-	-	-	-	-	-	-	-	-	-	-	-	-	-	-	-	-	-	-	-	-	-	-	-	-	-	-
		-	-	-	-	-	-	-	-	-	-	-	-	94234	-	-	-	-	-	-	-	-	-	-	-	-	-	-	-	-	-	-	-	-	-	-	-	-	-	-	-	-	-	-	-	-	-	-	-	-	-	-
GPD1		-	+	-	-	-	-	-	AT2G40690.1	-	-	-	-	94229	-	-	CMR476C	-	-	-	-	-	-	-	-	-	-	-	-	-	-	Os07g12640.1	32464	GSPATP00016407001	8975	137677	108162	-	242978	-	-	-	-	-	67.m00198	31003	-	-	-	-	-	-
		-	+	-	-	-	-	-	AT3G51140.1	-	-	-	-	94227	-	-	-	-	-	-	-	-	-	-	-	-	-	-	-	-	-	Os09g21250.1	22358	-	-	212552	-	-	421639	-	-	-	-	-	-	-	-	-	-	-	-	-
		-	-	-	-	-	-	-	-	-	-	-	-	94217	-	-	-	-	-	-	-	-	-	-	-	-	-	-	-	-	-	-	-	-	-	-	-	-	-	-	-	-	-	-	-	-	-	-	-	-	-	-
		-	-	-	-	-	-	-	-	-	-	-	-	94216	-	-	-	-	DDB0188983	-	-	-	-	-	-	-	-	-	-	-	-	-	-	-	-	-	144152	-	576399	-	-	-	-	-	-	-	-	-	-	-	-	-
		-	+	-	-	-	-	-	-	-	BDEG_08559	CE30265	177616	94202	-	-	-	OTTDARP00000005795	-	-	-	-	ENSGALP00000026919	-	OTTHUMP00000008596	-	-	-	-	-	-	-	-	-	-	-	-	-	-	-	-	SPBC106.11c	GLEAN3_04946	-	-	-	-	-	-	-	-	-
		-	-	-	-	-	-	-	-	-	-	-	-	94181	-	-	-	-	-	-	-	-	-	-	-	-	-	-	-	-	-	-	-	-	-	-	-	-	-	-	-	-	-	-	-	-	-	-	-	-	-	-
		-	-	-	-	-	-	-	-	-	-	-	170777	94169	-	-	-	-	-	-	-	-	ENSGALP00000010400	-	-	-	196428	-	-	-	-	-	-	-	44761	-	-	-	-	-	-	-	-	-	-	-	-	-	-	-	-	-
		-	-	-	-	-	-	-	-	-	-	-	-	94151	-	-	-	-	-	-	-	-	-	-	-	-	-	-	-	-	-	-	-	-	-	-	-	-	-	-	-	-	-	-	-	-	-	-	-	-	-	-
FBB8		+	+	-	+	-	-	GB15124-PA	-	-	BDEG_04593	-	90714	94144	288796	-	-	-	-	-	-	-	ENSGALP00000024121	-	NP_203528.2	-	142246	-	-	166745	-	-	-	-	-	-	-	-	-	-	-	-	-	SINFRUP00000142887	-	-	-	-	55191	-	Tb11.02.0354	-
		-	-	-	-	-	-	-	-	-	-	-	-	94128	293959	-	-	-	-	-	-	-	-	-	-	-	-	-	-	-	-	-	-	-	-	-	135681	-	-	-	-	-	-	-	-	-	-	-	-	-	-	-
		-	-	-	-	-	-	-	-	-	-	-	-	94123	-	-	-	-	-	-	-	-	-	-	-	-	-	-	-	-	-	-	-	-	-	-	-	-	-	-	-	-	-	-	-	-	-	-	-	-	-	-
		-	-	-	-	-	-	-	-	-	-	-	-	94122	-	-	-	-	-	-	-	-	-	-	-	-	-	-	-	-	-	-	-	-	-	77452	-	-	-	-	-	-	-	-	-	-	-	-	-	-	-	-
		-	-	-	-	-	-	-	-	-	-	-	-	94106	-	-	-	-	-	-	-	-	-	-	-	-	-	-	-	-	-	-	-	-	-	-	-	-	-	-	-	-	-	-	-	-	-	-	-	-	-	-
		-	-	-	-	-	-	-	-	-	-	-	-	94104	-	-	-	-	-	-	-	-	-	-	-	-	-	-	-	-	-	-	-	-	-	-	-	-	-	-	-	-	-	-	-	-	-	-	-	-	-	-
		-	-	-	-	-	-	-	-	-	-	-	-	94081	-	-	-	-	-	-	-	-	-	-	-	-	-	-	-	-	-	-	-	-	-	-	-	-	-	-	-	-	-	-	-	-	-	-	-	-	-	-
	Starch Branching Enzyme	-	+	-	-	-	-	GB30511-PA_2	AT2G36390.1	-	BDEG_06939	CE01076	228125	94079	-	cgd6_3280	CMH144C	-	DDB0203268	CG33138-PA	-	74.m00160	ENSGALP00000024956	GL50803_15823	OTTHUMP00000171891	-	195979	-	-	-	NCU05429	Os02g32660.1	31530	GSPATP00036828001	-	-	-	-	589574	RO3G_13160.1	YEL011W	-	GLEAN3_28105	SINFRUP00000151029	388.m00008	-	-	-	49690	-	-	-
		-	-	-	-	-	-	-	-	-	-	-	-	94063	-	-	-	-	-	-	-	-	-	-	-	-	-	-	-	-	-	-	-	-	-	-	-	-	-	-	-	-	-	-	-	-	-	-	-	-	-	-
		-	-	-	-	-	-	-	AT3G15110.1	-	-	-	-	94055	-	-	-	-	-	-	-	-	-	-	-	-	-	-	-	-	-	Os02g20490.1	-	-	-	188722	-	-	235083	-	-	-	-	-	-	-	-	-	-	-	-	-
		-	-	-	-	-	-	-	-	-	-	-	-	94027	-	-	-	-	-	-	-	-	-	-	-	-	-	-	-	-	-	-	-	-	-	-	-	-	-	-	-	-	-	-	-	-	-	-	-	-	-	-
		-	-	-	-	-	-	-	-	-	-	-	-	94026	-	-	-	-	-	-	-	-	-	-	-	-	-	-	-	-	-	-	-	-	-	-	-	-	-	-	-	-	-	-	-	-	-	-	-	-	-	-
		-	-	-	-	-	-	-	-	-	-	-	-	94004	-	-	-	-	-	-	-	-	-	-	-	-	-	-	-	-	-	-	-	-	-	-	-	-	-	-	-	-	-	-	-	-	-	-	-	-	-	-
		-	-	-	-	-	-	-	-	-	-	-	-	93996	-	-	-	-	-	-	-	-	-	-	-	-	-	-	-	-	-	-	-	-	-	-	-	-	-	-	-	-	-	-	-	-	-	-	-	-	-	-
		-	-	-	-	-	-	-	AT5G63390.1	-	-	-	-	93994	-	-	-	-	-	-	-	-	-	-	-	-	-	-	-	-	-	-	-	-	-	-	-	-	204006	-	-	-	-	-	-	-	-	-	-	-	-	-
		-	-	-	-	-	-	-	-	-	-	-	-	93992	-	-	-	-	-	-	-	-	-	-	-	-	-	-	-	-	-	-	-	-	-	-	-	-	-	-	-	-	-	-	-	-	-	-	-	-	-	-
		-	-	-	-	-	-	-	-	-	-	-	-	93991	-	-	-	-	-	-	-	-	-	-	-	-	-	-	-	-	-	-	-	-	-	-	-	-	-	-	-	-	-	-	-	-	-	-	-	-	-	-
		-	-	-	-	-	-	-	-	-	-	-	-	93985	-	-	-	-	-	-	-	-	-	-	-	-	-	-	-	-	-	-	-	-	-	-	-	-	-	-	-	-	-	-	-	-	-	-	-	-	-	-
		-	-	-	-	-	-	-	-	-	-	-	-	93982	-	-	-	-	-	-	-	-	-	-	-	-	-	-	-	-	-	-	-	-	-	-	-	-	-	-	-	-	-	-	-	-	-	-	-	-	-	-
		-	-	-	-	-	-	-	-	-	-	-	-	93974	-	-	-	-	-	-	-	-	-	-	-	-	-	-	-	-	-	-	-	-	-	-	-	-	-	-	-	-	-	-	-	-	-	-	-	-	-	-
		-	-	-	-	-	-	-	-	-	-	-	-	93970	-	-	-	-	-	-	-	-	-	-	-	-	-	-	-	-	-	-	-	-	-	-	-	-	-	-	-	-	-	-	-	-	-	-	-	-	-	-
		-	-	-	-	-	-	-	-	-	-	-	-	93960	-	-	-	-	-	-	-	-	-	-	-	-	-	-	-	-	-	-	-	-	-	-	-	-	-	-	-	-	-	-	-	-	-	-	-	-	-	-
		-	-	-	-	-	-	-	-	-	-	-	-	93957	-	-	-	-	-	-	-	-	-	-	-	-	-	-	-	-	-	-	-	-	-	-	-	-	-	-	-	-	-	-	-	-	-	-	-	-	-	-
		-	+	-	-	-	-	GB17471-PA	AT5G28220.1	18888	BDEG_01622	CE19639	108309	93946	294053	-	-	-	DDB0169127	-	-	-	ENSGALP00000025890	-	OTTHUMP00000177971	LmjF17.0120	205044	31493	3337	110221	NCU04651	Os02g03470.1	-	GSPATP00035658001	5873	15911	155492	PF14_0098	662899	-	-	SPBC15C4.01c	-	SINFRUP00000146667	288.m00019	32843	-	57.m03992	28044	-	-	-

		-	-	-	-	-	-	-	-	-	-	-	-	93944	-	-	-
		-	-	-	-	-	-	-	-	-	-	-	-	93923	-	-	-
		-	-	-	-	-	-	-	-	-	-	-	-	93919	-	-	-
		-	-	-	-	-	-	-	-	-	-	-	-	93918	-	-	-
		-	-	-	-	-	-	-	-	-	-	-	-	-	-	-	-
		-	+	-	-	-	-	GB19408-PA	AT3G02980.1	-	-	-	-	93906	-	-	-
		-	-	-	-	-	-	-	-	-	-	-	94711	93901	-	-	-
		-	-	-	-	-	-	-	-	-	-	-	-	93896	-	-	-
		-	-	-	-	-	-	-	-	-	-	-	-	93878	-	-	-
		-	-	-	-	-	-	-	-	-	-	-	-	93846	-	-	-
		-	-	-	-	-	-	-	-	-	-	-	-	93805	-	-	-
		-	-	-	-	-	-	-	-	-	-	-	-	93801	-	-	-
		-	-	-	-	-	-	-	-	-	-	-	-	93800	-	-	-
		-	-	-	-	-	-	-	-	-	-	-	-	93794	285613	-	-
		-	-	-	-	-	-	-	-	-	-	-	-	93783	-	-	-
		-	-	-	-	-	-	-	-	-	-	-	-	93780	-	-	-
		-	-	-	-	-	-	-	-	-	-	-	-	93766	-	-	-
FAP240	Flagellar Associated Protein	-	-	-	+	-	-	-	-	-	-	-	-	93765	-	-	-
		-	-	-	-	-	-	-	-	-	-	-	-	93762	-	-	-
		-	-	-	-	-	-	-	-	-	-	-	-	93758	-	-	-
		-	-	-	-	-	-	-	-	-	-	-	-	93750	-	-	-
		-	-	-	-	-	-	-	-	-	-	-	-	93748	-	-	-
		-	-	-	-	-	-	-	-	64381	-	-	-	93747	-	-	-
		-	-	-	-	-	-	-	-	-	-	-	-	93716	-	-	-
		-	-	-	-	-	-	-	-	-	-	-	59274	93667	-	-	-
		-	-	-	-	-	-	-	-	-	-	-	-	93664	-	-	-
		-	-	-	-	-	-	-	-	-	-	-	-	93657	-	-	-
		-	-	-	-	-	-	-	-	-	-	-	-	93643	-	-	-
		-	-	-	-	-	-	-	-	-	-	-	-	93641	-	-	-
		-	-	-	-	-	-	-	-	-	-	-	-	93626	-	-	-
		-	-	-	-	-	-	-	-	-	-	-	-	93612	-	-	-
		-	-	-	-	-	-	-	-	-	-	-	-	93587	-	-	-
		-	+	-	-	-	-	-	AT3G27330.1	-	-	CE19423	-	93561	-	-	-
		-	-	-	-	-	-	-	-	-	-	-	-	93550	-	-	-
		-	-	-	-	-	-	-	-	-	-	-	-	93548	-	-	-
		-	-	-	-	-	-	-	-	-	-	-	-	93538	-	-	-
	squamosa promoter binding-like protein	-	-	-	-	-	-	-	-	-	-	-	-	93505	-	-	-
		-	-	-	-	-	-	-	-	-	-	-	-	93502	-	-	-
		-	-	-	-	-	-	-	-	-	-	-	-	93467	-	-	-
PHC21	cell wall protein pherophorin-C21	-	-	-	-	-	-	-	-	-	-	-	-	93464	-	-	-
		-	-	-	-	-	-	-	-	-	-	-	-	93459	-	-	-
		-	-	-	-	-	-	-	-	-	-	-	-	93443	-	-	-
		-	-	-	-	-	-	-	-	-	-	-	-	93425	-	-	-
		-	-	-	-	-	-	-	-	-	-	-	-	93411	-	-	-
		-	+	-	-	-	-	GB10193-PA	AT1G25682.1	-	BDEG_01474	CE01697	94947	93400	381638	-	-
		-	-	-	-	-	-	-	-	-	-	-	-	93396	-	-	-
		-	-	-	-	-	-	-	-	-	-	-	-	93395	-	-	-
		-	+	-	-	-	-	-	AT4G25660.1	19518	-	-	-	93364	-	-	-
		-	+	-	-	-	-	GB15236-PA	AT5G67490.1	-	BDEG_08467	CE31079	157552	93361	257659	-	-
		-	-	-	-	-	-	-	-	-	-	-	-	93356	-	-	-
		-	-	-	-	-	-	-	-	-	-	-	-	93192	-	-	-
		-	-	-	-	-	-	-	AT5G18480.1	-	-	-	-	93189	-	-	-
		-	-	-	-	-	-	-	-	-	-	-	-	93187	-	-	-
		-	+	-	-	-	-	-	AT1G44835.2	-	-	-	-	93176	-	-	-
		-	-	-	-	-	-	-	-	-	-	-	-	93162	-	-	-
		-	-	-	-	-	-	-	-	-	-	-	-	93157	-	-	-
		-	-	-	-	-	-	-	-	-	-	-	-	93150	-	-	-
		-	-	-	-	-	-	-	-	-	-	-	-	93294	-	-	-
		-	-	-	-	-	-	-	-	-	-	-	-	93284	-	-	-
		-	-	-	-	-	-	-	-	-	-	-	-	140812	-	-	-
		-	+	-	-	-	-	GB17271-PA	AT5G19330.1	52893	-	CE00510	-	140706	290087	-	-
C_1220008		-	+	-	-	-	-	-	AT3G09580.1	-	-	-	-	140668	-	-	CMN162C
IF3C,EIF3C	Eukaryotic translation initiation factor 3 subunit 8	-	+	+	-	-	-	GB12747-PA	AT3G56150.1	25214	BDEG_07818	CE18958	221371	140639	206879	cgd4_3770	CMH126C
GAP1a	glyceraldehyde 3-phosphate dehydrogenase, plastid precursor	-	+	+	-	-	-	GB14798-PA	AT1G79530.1	70352	BDEG_06666	CE07371	159125	140618	-	cgd6_3790	CMM167C
		-	+	-	-	-	-	GB17942-PA	AT1G09020.1	53368	-	CE34111	93167	140612	261605	-	-
IF3K,EIF3K		-	+	-	-	-	-	GB18221-PA	AT4G33250.1	-	BDEG_00096	CE20081	154858	140593	284520	cgd3_4020	CMC106C
PCN1	PCNA poliferating cell nuclear antigen	-	+	+	-	-	-	GB12209-PA	AT1G07370.1	70163	BDEG_03027	CE37726	161800	140580	223015	cgd8_2150	CMS101C
		-	+	-	-	-	-	-	AT5G65205.1	-	-	-	-	140553	-	-	CMP110C
PRMT3402	Protein arginine N-methyltransferase	-	+	-	-	-	-	-	AT1G04870.2	32539	-	-	-	140543	-	-	-
		-	+	-	-	-	-	GB10142-PA	AT1G72970.1	59047	-	CE03052	227583	140509	294713	cgd8_2670	CMP087C
HSP90C	Heat shock protein 90C	-	+	-	+	-	-	-	AT2G04030.1	-	-	-	-	140500	-	-	-
TIM10	TIM10, import inner membrane translocase, mitochondrial.	-	+	-	-	-	-	GB14937-PA	AT2G29530.2	-	BDEG_08190	CE28800	129886	140499	282218	-	CMG033C
CNK6	Chlamydomonas NIMA-related protein kinase homolog 6	-	+	-	-	-	-	-	-	4743	-	-	-	140498	-	-	-
FGS1	Ferredoxin-dependent glutamate synthase, chloroplast precursor	-	+	-	-	-	-	-	AT5G04140.1	37118	-	-	-	140487	-	-	CMV060C
SNT3401	SIN3 component	-	+	-	-	-	-	GB15858-PA	AT1G70060.1	39114	BDEG_01886	CE33046	177539	140464	-	cgd5_2310	CMQ125C
STA11	4-alpha-glucanotransferase	-	+	-	-	-	-	-	AT5G64860.1	-	-	-	-	140452	-	-	-
ANK13	protein of unknown function with ankyrin repeats	-	+	-	-	-	-	-	AT2G03430.1	-	BDEG_05620	CE19848	-	140415	289297	cgd7_20	-
FtsZ1		-	+	-	-	-	-	-	AT5G55280.1	-	-	-	-	140409	-	-	-
		-	-	-	-	-	-	-	AT3G44730.1	-	-	-	-	140337	-	-	-
		-	+	+	-	-	-	GB17200-PA	AT3G13200.1	-	BDEG_02991	CE16380	224957	140320	-	cgd8_1950	-
PRFC	peptide-chain release factor RF-3	-	+	-	-	-	-	-	-	37063	-	-	40186	140260	-	-	-
ASNS		-	+	-	-	-	-	GB13219-PA	AT3G47340.1	71545	-	CE37263	218038	140252	212443	-	CMM145C
Amt1;6		-	-	-	-	-	-	-	-	-	-	-	-	140223	-	-	-
BBS8		+	+	-	-	-	-	GB10554-PA	-	54838	-	CE31071	163723	140113	250960	-	-
		-	+	-	-	-	-	-	-	-	-	-	-	140111	239988	-	-
UBC3,UBI1	bi-ubiquitin, major isoform UBI1a	-	+	-	-	-	-	GB16469-PA	AT5G03240.3	59045	BDEG_05300	CE31915	162204	140045	-	cgd5_2550	-
	related to phosphate/phosphoenolpyruvate translocator proteins	-	+	-	-	-	-	-	AT5G05820.1	-	BDEG_07967	-	-	139981	-	cgd4_3110	-
		-	+	-	-	-	-	-	AT1G17330.1	-	-	-	-	139957	-	-	-
FKB17-2	peptidyl-prolyl cis-trans isomerase, FKBP-type	-	+	-	-	-	-	-	AT1G18170.1	-	-	-	-	139925	-	-	CMT472C
		-	+	-	-	-	-	-	AT3G44100.1	-	BDEG_00414	-	-	139920	-	-	-
CDKG2	cyclin dependent kinase, Chlamydomonas specific	-	-	-	-	-	-	-	-	-	-	-	-	139908	-	-	-
CPN60B1		-	-	-	-	-	-	-	-	-	-	-	-	139902	-	-	-
LHL4	A high intensity light-inducible Lhc-like gene	-	-	-	-	-	-	-	-	-	-	-	-	139895	-	-	-
CPN2		-	+	-	-	-	-	GB16721-PA	AT3G10220.1	23381	BDEG_06572	CE10958	180954	139881	201724	-	CMA036C
		-	+	-	-	-	-	-	-	30097	-	-	182185	139865	232149	-	-
NUO10	Mitochondrial NADH:ubiquinone oxidoreductase (complex I) subunit 10; PSST-like protein.	-	+	-	-	-	-	GB10859-PA	AT5G11770.1	26347	BDEG_06109	CE14780	154203	139850	204478	-	CMI095C
RPL9	Ribosomal protein L9, component of cytosolic 80S ribosome and 60S large subunit	-	+	+	-	-	-	GB15648-PA	AT4G10450.1	60385	BDEG_04868	CE01380	151035	139831	207365	cgd8_400	CMC145C
		-	-	-	-	-	-	-	-	-	-	-	-	139792	-	-	-
		+	+	-	+	-	-	GB19147-PA	-	61399	BDEG_05701	CE40504	164190	139789	219295	-	-
TRXf1	Thioredoxin f1, chloroplast precursor	-	+	-	-	-	-	-	AT3G02730.1	-	-	-	-	139781	290783	-	-
BI-1	Putative Bax inhibitor-1 (BI-1)	-	+	-	-	-	-	GB18030-PA	AT5G47120.1	-	-	-	158085	139760	292109	-	CMT426C
NADTH	Probable NAD(P) transhydrogenase, mitochondrial localization assumed	-	+	-	-	-	-	-	-	51957	-	CE08176	163980	139758	254743	cgd8_2330	-
ACS2	Acetyl CoA synthetase	-	+	-	+	-	-	GB10851-PA	AT5G36880.2	-	-	-	133043	139750	-	cgd1_3710	-
		-	+	-	-	-	-	-	AT1G21350.3	-	-	-	-	139747	-	-	CMP081C
		-	+	-	-	-	-	GB13404-PA	AT4G34570.1	38942	BDEG_02023	CE24124	148645	139742	225972	cgd4_4460	CMS462C
MDAR	monodehydroascorbate reductase	-	+	-	-	-	-	GB16702-PA	AT3G52880.1	-	BDEG_05673	-	220923	139735	-	-	CMT414C
SEC13	COP-II coat subunit	-	+	+	-	-	-	GB18558-PA	AT3G01340.2	26185	BDEG_05648	CE25614	149284	139713	225177	cgd8_4110	CMJ112C
GTR18	glycosyl transferase	-	+	+	-	-	-	GB11377-PA	AT3G45100.2	-	BDEG_05275	CE03109	228243	139676	286971	cgd4_2100	-
		-	-	-	-	-	-	-	-	-	-	-	-	139672	-	-	-
		-	+	-	-	-	-	GB13321-PA	AT5G58220.3	27303	BDEG_04708	CE40007	223510	139658	-	-	-
		-	+	-	-	-	-	GB18015-PA	AT4G15030.1	16138	BDEG_00464	CE05732	105775	139643	267022	-	-
		-	+	-	-	-	-	-	-	23653	-	-	-	139628	-	-	CMJ216C
KAS2		-	+	-	-	-	-	-	AT1G74960.1	25463	-	-	-	139619	-	-	CMM286C
GBA1		-	+	-	-	-	-	-	AT5G13650.2	52055	-	-	-	139610	-	-	CMR109C
		-	-	-	-	-	-	-	-	-	-	-	-	139591	225853	-	-
		-	+	-	-	-	-	GB14373-PA	-	54616	-	CE23663	227476	139581	222833	-	CME059C
		-	+	-	-	-	-	-	-	64574	-	-	-	139577	-	-	-
PDH1a	pyruvate dehydrogenase, E1 beta subunit, mitochondrial precursor	-	+	-	-	-	-	GB17238-PA	AT5G50850.1	65618	BDEG_05932	CE27647	161753	139515	289542	-	-
		-	+	-	-	-	-	GB16362-PA	AT3G51800.1	55591	BDEG_06790	CE21275	152656	139416	227809	cgd3_2390	CMM200C
		-	+	-	-	-	-	GB15932-PA	AT3G02660.1	59618	-	CE29515	18730	139405	291269	-	CMQ275C
FAP173	Conserved Uncharacterized Flagellar Associated Protein	-	+	-	+	-	-	-	AT1G69840.6	37263	-	-	-	139332	-	-	-
LCI19	Putative gamma hydroxybutyrate dehydrogenase;	-	-	-	-	-	-	-	-	-	-	-	178346	139269	-	-	-
UGE	UDP-glucose 4-epimerase	-	-	-	-	-	-	-	-	-	-	-	-	-	-	-	-
IPA1	Importin alpha	-	+	+	-	-	-	GB15853-PA	AT3G06720.2	69702	BDEG_04832	CE20745	220826	139246	239957	cgd8_3260	CMT096C
	ABC1 family ser/thr kinase	-	+	-	-	-	-	-	AT3G24190.1	25238	-	-	-	139226	-	-	CMG161C
		-	+	+	-	-	-	GB12270-PA	AT4G10320.1	39368	BDEG_00226	CE06304	226043	139213	240599	cgd3_3840	CMT280C
RSP23	Radial spoke protein 23	-	+	-	+	-	-	GB19270-PA	AT1G17410.1	-	-	-	177417	139197	297839	-	-
MITC17	putative mitochondrial substrate carrier protein	-	+	-	-	-	-	GB16839-PA	AT5G66380.1	-	-	CE02268	174218	139183	298933	cgd1_2370	CMI146C
		-	+	+	-	-	-	GB10596-PA	AT3G57220.1	3004	-	CE24520	171012	139119	272814	cgd5_2240	CMQ042C
ARG9	acetylornithine aminotransferase	-	+	-	-	-	-	-	AT1G80600.1	-	-	-	-	139007	-	-	CMQ064C
RAE1	Similar to mRNA export protein	-	+	+	-	-	-	GB12111-PA	AT1G80670.1	58787	BDEG_02618	CE09338	20827	138990	236689	cgd6_4610	CMI077C
RCL1	RNA terminal 3' Phosphate cyclase-like	-	+	+	-	-	-	GB12601-PA	AT5G22100.1	30084	BDEG_00237	CE32083	218453	138936	209831	cgd8_3660	CMQ312C
		-	+	-	-	-	-	GB18847-PA	AT5G55940.1	-	BDEG_05767	CE09649	159144	138886	-	cgd7_3280	-
		-	+	-	-	-	-	GB11976-PA	AT4G29870.1	-	-	-	148642	138808	207800	-	CMR204C
QCR1	Ubiquinol:cytochrome c oxidoreductase (Complex III) 50 kDa subunit, Core 1 subunit, mitochondrial	-	+	-	-	-	-	GB11385-PA	AT3G02090.1	26725	BDEG_06151	CE35701	170570	138803	284342	cgd5_3400	CMM104C
	Putative metacaspase type I	-	+	-	-	-	-	-	AT4G25110.1	31552	BDEG_03693	-	-	138763	-	-	-
RPL12	Ribosomal protein L12, component of cytosolic 80S ribosome and 60S large subunit	-	+	+	-	-	-	GB19465-PA	AT2G37190.1	22183	BDEG_07721	CE17986	-	138697	220935	cgd3_1300	CMS284C
SECA1		-	+	-	-	-	-	-	AT4G01800.1	29609	-	-	-	138696	-	-	CMQ393C
	MDR-like ABC transporter	-	+	-	-	-	-	GB13224-PA	AT2G47000.1	20001	-	-	135774	138672	260086	-	-
		-	+	-	-	-	-	-	AT1G19440.1	72240	-	-	-	138664	-	-	CMD118C
IFT81	Intraflagellar Transport Protein 81	+	+	-	+	-	-	-	-	-	BDEG_00751	CE34008	145724	138649	288339	-	-
ATO1	acetyl-CoA acyltransferase ;	-	+	-	-	-	-	GB12956-PA	AT2G33150.1	69755	BDEG_06810	CE10878	229355	138637	213580	-	-
		-	+	+	-	-	-	GB10672-PA	AT2G32000.1	19616	BDEG_03093	CE32972	182010	138607	288537	cgd6_2390	CMG056C
	MDR-like ABC transporter	-	+	-	-	-	-	-	-	-	BDEG_01525	-	117978	138602	-	cgd6_4700	CMD148C
		-	-	-	-	-	-	GB18141-PA	-	-	-	-	-	138565	-	-	-
		-	+	-	-	-	-	-	AT1G54130.1	10270	-	-	49885	138526	-	-	CMT625C
GSA	Glutamate-1-semialdehyde aminotransferase, chloroplast precursor	-	+	-	-	-	-	-	AT5G63570.1	70752	-	-	58235	138524	-	-	CMP285C
		-	+	+	-	-	-	GB12891-PA	AT3G51270.1	33949	BDEG_04743	CE33542	225610	138512	290660	cgd7_440	CMN167C
		-	+	-	-	-	-	GB15677-PA	AT3G28480.1	-	-	CE20261	167710	138508	280092	-	-
LysRS	Lysyl-tRNA synthetase	-	+	+	-	-	-	GB12887-PA	AT3G11710.1	1254	BDEG_05628	CE28247	164486	138493	213508	cgd4_2370	CMM274C
	hypothetical protein	-	+	+	-	-	-	-	AT5G37530.2	3593	BDEG_05702	-	-	138485	-	-	-
PHX	prolyl 4-hydroxylase alpha-1 subunit precursor-like protein	-	+	-	-	-	-	-	-	26622	-	-	-	138462	-	cgd3_3910	-
MUT11		-	+	-	-	-	-	-	AT3G49660.1	22488	BDEG_06284	CE00901	150834	138445	298216	-	CMC158C
OASTL1	cysteine synthase, chloroplast precursor	-	+	-	-	-	-	-	-	-	BDEG_02702	-	-	138415	214443	-	-
		-	+	-	-	-	-	-	AT3G02790.1	-	-	CE37127	-	138304	-	-	-
		-	-	-	-	-	-	-	-	-	-	-	-	138215	-	-	-

GTR1	glycosyl transferase	[illegible]
XPO1	Exportin involved in mRNA and protein export out of the nucleus	[illegible]
ALSL1	acetolactate synthase, large subunit	[illegible]
HSP90A	Heat shock protein 90A	[illegible]
LhcbM9		[illegible]
	pyruvate kinase	[illegible]
LhcbM4		[illegible]
MND1		[illegible]
UCP1	uncoupling protein, probably mitochondrial	[illegible]
TF2F	Putative Transcription factor IIF	[illegible]
PF14	Radial spoke protein 3	[illegible]
PBG1		[illegible]
RPN1	26S proteasome regulatory subunit RPN1	[illegible]
VPS28	subunit of the ESCRT-I complex	[illegible]
RFC3	DNA replication factor C complex subunit 3	[illegible]
HDA3401	Histone deacetylase	[illegible]
MFT9	major facilitator superfamily transporter	[illegible]
IDA7	Flagellar inner dynein arm I1 intermediate chain IC140	[illegible]
PAM18	PAM18, presequence translocase-associated protein import motor subunit, mitochondrial.	[illegible]
FAP106	Conserved Uncharacterized Flagellar Associated Protein	[illegible]
ELI2*	Early light-inducible protein;	[illegible]
PPA	inorganic pyrophosphatase	[illegible]
ELI2	Early light-inducible protein;	[illegible]
HPD2		[illegible]
CMK	4-diphosphocytidyl-2-C-methyl-D-erythritol kinase, putative chloroplast precursor	[illegible]
DLC7a	Flagellar outer dynein arm light chain 7	[illegible]
MAPK2	Mitogen-Activated Protein Kinase Homolog 2	[illegible]
SMP3		[illegible]
RPPK1	ribose-phosphate pyrophosphokinase (RPPK)	[illegible]
CDKD1	CDK activating kinase	[illegible]
HSP70C	Heat shock protein 70C	[illegible]
RCD1	Rcd1-like protein	[illegible]
ECP88		[illegible]
NAR1;5		[illegible]
CK1	Ser-/Thr-kinase involved in cricadian clock	[illegible]
ARD1	Aci-reductone dioxygenase	[illegible]
IDA2	Flagellar inner arm dynein 1 heavy chain beta	[illegible]
ANK10,FAP254	Flagella Associated Protein	[illegible]
ZSP2-2		[illegible]
MDH1	malate dehydrogenase	[illegible]
FTR1	Iron permease, membrane protein	[illegible]
MITC10	related to mitochondrial carrier proteins	[illegible]
FPPS	farnesyl diphosphate synthase, putative	[illegible]
GPX3	Glutathione peroxidase, possibly mitochondrial	[illegible]
GCSP	Glycine cleavage system, P protein	[illegible]
PRP28		[illegible]
GLN3	glutamine synthetase, plastid precursor	[illegible]
NGS1	Glutamate synthase, NADH-dependent	[illegible]
RAA3,Amt1;8		[illegible]
ECT	CTP:ethanolamine cytidylyltransferase	[illegible]
LSM1	Sm protein-like	[illegible]
PYK1	Pyruvate Kinase, probably cytosolic	[illegible]
RPL26	Ribosomal protein L26, component of cytosolic 80S ribosome and 60S large subunit	[illegible]
GGR	geranylgeranyl reductase, putative chloroplast precursor	[illegible]
	EMP/nonaspanin domain family	[illegible]
	conserved protein of unknown function	[illegible]
POR	Light-dependent protochlorophyllide reductase, chloroplast precursor	[illegible]
ATP6	F1Fo ATP synthase subunit 6, mitochondrial	[illegible]
IFT74	Intraflagellar transport protein 74/72	[illegible]
TDL	transducin-like protein, contains WD-40 repeat	[illegible]
TRXm	Thioredoxin m, chloroplastic	[illegible]
ODA14	Outer dynein arm-docking complex subunit 3	[illegible]
CYN40	Peptidyl-prolyl cis-trans isomerase, cyclophilin-type	[illegible]
TRXx	Thioredoxin x, chloroplast precursor	[illegible]
DBP2	putative Dbp2	[illegible]
PIN3	Peptidyl-prolyl cis-trans isomerase, parvulin-type	[illegible]
CPN60B2	Chaperonin 60B2	[illegible]
CRD1	Copper response defect 1 protein, chloroplast precursor	[illegible]
VAMP74	R-SNARE, VAMP7-family	[illegible]
UNI3		[illegible]
GLH2	alpha-glucosidase	[illegible]
HOP1	HSP70-HSP90 organizing protein	[illegible]
STA6	ADP-glucose pyrophosphorylase small subunit	[illegible]
VPS46	subunit of the ESCRT-III complex	[illegible]
DHC8	Dynein heavy chain 8	[illegible]
PTA4	PTA4 Putative proton/phosphate symporter	[illegible]
MTDH/MTCH2	Putative 5,10-methylenetetrahydrofolate dehydrogenase/5,10-methenyltetrahydrofolate cyclohydrolase	[illegible]
	Fatty acid desaturase	[illegible]
DRT1	DR1-like protein	[illegible]
CHLI1	Magnesium chelatase subunit I, chloroplast precursor	[illegible]
LCIC	low-CO2-inducible protein	[illegible]
Nar1;2	Putative anion transporter	[illegible]
NUOS5	NADH:ubiquinone oxidoreductase 10 kDa subunit (Complex I PFFD subunit), mitochondrial	[illegible]
RPE1	Ribulose phosphate-3-epimerase, chloroplast precursor	[illegible]
C_370062		[illegible]
CHLI2	CHLI Magnesium chelatase subunit I, chloroplast precursor	[illegible]
CHR3404	SNF2 superfamily protein; ISW1/2 class	[illegible]
LCI30	low-CO2-inducible protein	[illegible]
PRP38	similar to pre-mRNA splicing factor PRP38 family protein	[illegible]
FAP253	Flagellar Associated Protein	[illegible]
NIFU2,NFU2	Putative Fe-S cluster assembly protein	[illegible]
RPB119aa		[illegible]
GPPS	geranyl diphosphate synthase, putative chloroplast precursor	[illegible]
CCT3	chaperone subunit	[illegible]
CSP41b	chloroplast stem-loop-binding protein	[illegible]
MFT4	putative MATE efflux family protein	[illegible]
PGI	phosphoglucose isomerase	[illegible]
MPC1	MPC1 Mitochondrial Phosphate Carrier 1	[illegible]
PDC2	pyruvate decarboxylase	[illegible]
ATPvD1	Vacuolar H+ ATPase V0 sector, subunit D	[illegible]
GAD2	UDP-D-glucuronic acid decarboxylase	[illegible]
UBC3	E2 ubiquitin-conjugating enzyme	[illegible]
GAD1	UDP-D-glucuronic acid decarboxylase	[illegible]

LF4	Long Flagella Protein 4	+	+	-	-	-	-	-
SMP2		-	+	+	-	-	-	GB10329-PA
APC10	Anaphase promoting complex subunit 10.	-	+	+	-	-	-	GB16034-PA
TBA1	psbA translation factor, chloroplast	-	+	-	-	-	-	GB19753-PA
ARH1	NADP adrenodoxin-like ferredoxin reductase	-	+	-	-	-	-	GB17540-PA
VIPP1	Vesicle inducing protein in plastids 1	-	+	-	-	-	-	-
NGB1	Putative nucleolar GTP-binding protein 1.	-	+	+	-	-	-	GB17562-PA
		-	+	-	-	-	-	-
		-	-	-	-	-	-	-
TRXy	Thioredoxin y, chloroplast precursor	-	+	-	-	-	-	-
		-	-	-	-	-	-	-
ANK28	protein of unknown function with ankyrin repeats	-	+	-	-	-	-	GB17410-PA
ERJ1	ER DnaJ-like protein 1	-	+	-	-	-	-	GB15525-PA
CDJ2	Chloroplast DnaJ-like protein 2	-	+	-	-	-	-	-
DHC6	Dynein heavy chain 6	+	+	-	+	-	-	GB11438-PA
CHLD	Magnesium chelatase subunit D, chloroplast precursor	-	+	-	-	-	-	-
CYCL1	L-type cyclin homologue	-	+	-	-	-	-	GB14538-PB
		-	-	-	-	-	-	-
LAObLAO2	L-amino-acid oxidase	-	-	-	-	-	-	-
HSP22A	heat shock protein 22A	-	+	-	-	-	-	-
		-	-	-	-	-	-	-
CNK3	Chlamydomonas NimA-Related Protein Kinase Homolog 3	-	+	-	-	-	-	-
CysA1	CysA-like protein, chloroplast location proposed	-	+	-	-	-	-	GB16489-PA
EF1A1	eukaryotic translation elongation factor 1 alpha 1	-	-	-	-	-	-	-
		-	+	-	-	-	-	-
		-	+	-	-	-	-	-
ATPC	ATP synthase gamma chain, chloroplast precursor	-	+	-	-	-	-	-
		-	-	-	-	-	-	-
Lhca8		-	-	-	-	-	-	-
ATPvL1	vacuolar H(+)-ATPase V0 sector, c/c' subunits	-	+	+	-	-	-	GB19405-PA
	protein of unknown function, contains zinc-finger domains	-	+	+	-	-	-	GB16780-PA
CDC48	Conserved AAA Domain Flagellar Associated Protein	-	+	+	+	-	-	GB20017-PA
GSHR1	glutathione reductase	-	+	-	-	-	-	-
		-	+	+	-	-	-	GB19410-PA
Hla3	putative ABC transporter	-	-	-	-	-	-	-
RPS3	Ribosomal protein S3, component of cytosolic 80S ribosome and 40S small subunit	-	+	+	+	-	-	GB15637-PA
		-	+	-	-	-	-	-
		-	-	-	-	-	-	-
MOB1	Cytokinesis-related protein.	-	+	+	-	-	-	GB15922-PA
GLN1	glutamine synthetase, cytosolic	-	+	-	-	-	-	GB10139-PA
HLIP		-	-	-	-	-	-	-
ATS2	ATP-sulfurylase	-	+	-	-	-	-	-
BIP1	Binding protein 1	-	+	-	+	-	-	-
COQ5C	ubiquinone/menaquinone biosynthesis methyltransferase	-	+	-	-	-	-	-
NRX1	Nucleoredoxin 1	-	-	-	-	-	-	-
PTA1	PTA1 Putative proton/phosphate symporter	-	+	-	-	-	-	-
CNK1	Chlamydomonas NimA-Related Protein Kinase Homolog 1	-	-	-	-	-	-	-
PSAN	photosystem I reaction center subunit N, chloroplast precursor	-	-	-	-	-	-	-
BIP2	Binding protein 2	-	+	-	-	-	-	GB15016-PA
IPY1		-	+	+	-	-	-	GB18261-PA
Lhca5		-	-	-	-	-	-	-
DDB1	UV-damaged DNA binding complex subunit 1 protein	-	+	-	-	-	-	GB18569-PA
PPD1	Pyruvate,phosphate dikinase (PPDK)	-	+	-	-	-	-	-
MNP1	ATP-binding protein, MRP/NBP35 family	-	+	-	-	-	-	GB10812-PA
ATP15	F1Fo ATP synthase, epsilon subunit, mitochondrial	-	+	-	-	-	-	-
POA3	20S proteasome alpha subunit C	-	+	+	-	-	-	GB11482-PA
		-	-	-	-	-	-	-
		-	-	-	-	-	-	-
		-	-	-	-	-	-	-
		-	-	-	-	-	-	-
		-	-	-	+	-	-	-
CYC1	ubiquinol:cytochrome c oxidoreductase cytochrome c1, mitochondrial	-	+	-	-	-	-	GB12510-PA
		-	+	-	-	-	-	-
		-	+	-	-	-	-	GB13100-PA
ZYS1b	putative transcription factor; zygote-specific	-	-	-	-	-	-	-
NDA2	putative type-II calcium-dependant NADH dehydrogenase, mitochondrial	-	+	-	-	-	-	-
ADH1		-	+	-	-	-	-	GB12901-PA
CCDA	c-type cytochrome biogenesis protein	-	+	-	-	-	-	-
UGD1	UDP-glucose dehydrogenase	-	-	-	-	-	-	-
CAH6	carbonic anhydrase 6	-	-	-	+	-	-	-
ACCasebetaCT		-	-	-	+	-	-	-
		-	-	-	-	-	-	-
CUL2		-	+	-	-	-	-	GB11554-PA
GTC3401	Spt16 transcriptional regulator	-	+	+	-	-	-	GB13130-PA
AK4	Adenylate Kinase Homolog 2	-	+	-	+	-	-	GB10282-PA
		-	+	+	-	-	-	GB18892-PA
LIG1	DNA ligase	-	+	+	-	-	-	GB30402-PA
SEC23A	COP-II coat subunit	-	+	+	-	-	-	GB19531-PA
SHKG1	5-enolpyruvylshikimate-3-phosphate (EPSP) synthase	-	+	-	-	-	-	-
AGT2	alanine-glyoxylate transaminase, putative	-	+	-	-	-	-	GB16475-PA
DEG7	DegP-type protease, cytosolic	-	+	-	-	-	-	-
ACBP		-	+	-	-	-	-	GB16162-PA
		-	+	+	-	-	-	GB14191-PA
DUR1	urea carboxylase	-	-	-	-	-	-	-
HPRT	hypoxanthine-guanine phosphoribosyltransferase	-	-	-	-	-	-	-
CNK4	Chlamydomonas NimA-Related Protein Kinase Homolog 4	-	+	-	-	-	-	-
MUT6	DEAH-box RNA helicase, post-transcriptional gene silencing	-	+	-	-	-	-	-
IF3E		-	+	-	-	-	-	GB19077-PA
NUO8	NADH:ubiquinone oxidoreductase (Complex I) subunit, homolog to bovine TYKY subunit, mitochondrial	-	+	-	-	-	-	GB17476-PA
EF1A2	eukaryotic translation elongation factor 1 alpha 2	-	+	+	+	-	-	GB10560-PA
CYN18-2	Peptidyl-prolyl cis-trans isomerase, cyclophilin-type	-	+	-	-	-	-	-
		-	+	-	-	-	-	-
		-	-	-	-	-	-	-
NTR3	NADPH dependent thioredoxin reductase 3	-	+	-	-	-	-	-
POC7	centriole proteome protein	-	-	-	-	-	-	-
UBC10	ubiquitin-conjugating enzyme	-	+	+	-	-	-	GB11856-PA
		-	-	-	-	-	-	-
CAG2	NADH:ubiquinone oxidoreductase (Complex I) subunit related to carbonic anhydrase (gamma-type) protein, mitochondrial	-	+	-	-	-	-	-
		-	+	-	-	-	-	GB15457-PA
		-	+	-	-	-	-	-
RAA2		-	-	-	-	-	-	-
ODA1	Flagellar outer dynein arm-docking complex protein 2	+	+	-	+	-	-	GB18568-PA
RPS30	Ribosomal protein S30, component of cytosolic 80S ribosome and 40S small subunit	-	+	+	-	-	-	GB17325-PA
		-	-	-	-	-	-	-
ATPD	ATP synthase delta chain, chloroplast precursor	-	+	-	-	-	-	-
		-	+	-	-	-	-	GB15814-PA
		-	+	+	-	-	-	GB20119-PA
MGE1	mitochondrial grpE-type co-chaperone of the HSP70 system	-	+	-	-	-	-	GB12067-PA
KAS1		-	+	-	-	-	-	-
BBS1	Bardet-Biedl syndrome 1	+	+	-	-	-	-	GB15554-PA
		-	-	-	-	-	-	-
CPN60B1	Chaperonin 60B1	-	-	-	-	-	-	-
PP2A-c4		-	+	+	-	-	-	-
RNP10	putative RNA-binding protein	-	-	-	-	-	-	-
IDA4	Flagellar inner arm dynein light chain p28	+	+	-	+	-	-	GB13866-PA
		-	+	-	-	-	-	-
		-	+	-	+	-	-	-
FtsZ2		-	+	-	-	-	-	-
		-	+	+	-	-	-	GB10251-PA
		-	+	-	-	-	-	-
GLPV		-	-	-	-	-	-	-
		-	-	-	-	-	-	-
		-	+	-	-	-	-	GB12621-PA
FAP151	Flagellar Associated Protein	-	+	+	+	-	-	GB18938-PA
PGK1	Phosphoglycerate Kinase	-	-	-	-	-	-	-
		-	+	-	-	-	-	-
UROD1	Uroporphyrinogen-III decarboxylase, chloroplast precursor	-	+	-	-	-	-	-
		-	+	-	-	-	-	-
AMI1		-	+	-	-	-	-	-
		-	+	-	-	-	-	GB16864-PA
ACP1	acyl-carrier protein, probably mitochondrial	-	+	-	-	-	-	GB15102-PA
FBB5	Similar to C21orf59	+	+	-	-	-	-	GB16023-PA
		-	-	-	-	-	-	-
		-	+	+	-	-	-	GB15362-PA
	isoamylase, starch debranching enzyme	-	+	-	-	-	-	-
	short-chain dehydrogenase/reductase SDR	-	-	-	-	-	-	-
		-	+	-	-	-	-	-
		-	-	-	-	-	-	-
CAH7		-	+	-	-	-	-	-
THTR	THTR, thiosulfate sulfurtransferase (Rhodanese), mitochondrial precursor.	-	+	-	-	-	-	-
MAPK5	Mitogen-Activated Protein Kinase Homolog 5	-	+	-	-	-	-	GB11031-PA
		-	+	-	-	-	-	GB17595-PA
		-	-	-	-	-	-	-
		-	-	-	-	-	-	-
		-	+	-	-	-	-	GB13861-PA
		-	+	-	-	-	-	-
		-	+	-	-	-	-	-
PRPS5	Plastid ribosomal protein S5, imported to chloroplast, small ribosomal subunit	-	+	-	-	-	-	-
	ABC transporter	-	+	-	-	-	-	-
		+	+	-	+	-	-	GB18394-PA
CYB5_1		-	+	-	-	-	-	GB12288-PA
		-	+	-	-	-	-	-
GPM	phosphoglucomutase	-	-	-	-	-	-	-
Crylike		-	+	-	-	-	-	GB10211-PA
		-	+	-	-	-	-	GB18226-PA
		-	+	-	-	-	-	GB13436-PA

		-	-	-	-	-	-	-	AT3G43540.1	-	[illegible]
		-	+	-	-	-	-	-	AT4G17770.1	70085	[illegible]
Lhca6		-	-	-	-	-	-	-	-	-	[illegible]
		-	-	-	-	-	-	-	-	-	[illegible]
		-	-	-	-	-	-	-	-	-	[illegible]
PP2A-2r	Protein Phosphatase 2A Homolog 2 regulatory subunit	-	+	+	+	-	-	GB15359-PA	AT3G25800.1	20037	[illegible]
ALK4	Aurora Like Protein Kinase Homolog 4	-	-	-	-	-	-	-	-	-	[illegible]
MAP1D	Methionyl aminopeptidase	-	+	-	-	-	-	-	AT4G37040.1	-	[illegible]
		-	+	-	-	-	-	-	AT5G25760.2	16391	[illegible]
NUO17	NADH:ubiquinone oxidoreductase (Complex I) 17 kD subunit, similar to bovine ESSS subunit, mitochondrial	-	-	-	-	-	-	-	-	-	[illegible]
SMPf		-	+	+	-	-	-	GB15877-PA	AT4G30220.1	22949	[illegible]
APR,MET16		-	+	-	-	-	-	-	AT4G04610.1	7244	[illegible]
SECA2		-	-	-	-	-	-	-	-	-	[illegible]
SLY1	SM/Sec1-family protein	-	+	+	-	-	-	GB12493-PA	AT2G17980.1	-	[illegible]
NFC3401	Nucleosome remodeling factor	-	+	+	-	-	-	GB10144-PA	AT5G58230.1	70326	[illegible]
NOP56	Nucleolar protein, Component of C/D snoRNPs	-	+	+	-	-	-	GB14895-PA	AT1G56110.1	59068	[illegible]
GTR22	glycosyl transferase	-	+	-	-	-	-	GB14947-PA	AT2G01720.1	-	[illegible]
		-	+	-	-	-	-	GB17313-PA	AT5G18520.1	18349	[illegible]
		-	-	-	-	-	-	-	-	-	[illegible]
DIP13	Deflagellation inducible protein, 13 kD.	+	+	-	+	-	-	GB13090-PA	-	55205	[illegible]
FAP131	Flagellar Associated Protein	-	-	-	+	-	-	-	-	-	[illegible]
LhcbM5		-	-	-	-	-	-	-	-	-	[illegible]
SAC1		-	+	-	-	-	-	-	-	60761	[illegible]
CCT5		-	+	+	+	-	-	GB10587-PA	AT1G24510.1	54967	[illegible]
		-	-	-	+	-	-	-	-	-	[illegible]
		-	-	-	-	-	-	-	-	-	[illegible]
	AIR synthase related protein	-	+	-	-	-	-	GB15524-PA	AT1G74260.1	22512	[illegible]
		-	+	-	-	-	-	-	AT5G58740.1	18373	[illegible]
		-	+	-	-	-	-	GB17837-PA	-	18967	[illegible]
	Uncharacterized protein conserved in bacteria	-	-	-	-	-	-	-	-	-	[illegible]
		-	+	-	-	-	-	GB10178-PA	AT5G13240.1	33964	[illegible]
		-	-	-	-	-	-	-	-	-	[illegible]
	calcium transporting ATPase	-	+	-	-	-	-	-	-	-	[illegible]
		-	+	-	-	-	-	-	AT4G15093.1	-	[illegible]
PSAF	photosystem I reaction center subunit III, chloroplast precursor	-	+	-	-	-	-	-	AT1G31330.1	30727	[illegible]
SMP6a		-	+	-	-	-	-	GB12358-PA	AT3G59810.1	21376	[illegible]
ODA11	Flagellar outer dynein arm heavy chain alpha	-	+	-	+	-	-	-	-	-	[illegible]
FEN1	nuclease, Rad2 (Class II) family.	-	+	+	-	-	-	GB15388-PA	AT5G26680.1	19112	[illegible]
		-	+	+	-	-	-	GB15406-PA	AT1G20575.1	14095	[illegible]
		-	+	-	-	-	-	-	AT4G39670.1	-	[illegible]
		-	+	-	-	-	-	GB15049-PA	AT3G55610.1	54959	[illegible]
RPL39	Ribosomal protein L39, component of cytosolic 80S ribosome and 60S large subunit	-	+	-	-	-	-	GB16604-PA	AT4G31985.1	28672	[illegible]
		-	+	+	-	-	-	GB15423-PA	AT4G12590.1	20443	[illegible]
CDC20	Activator and specificity subunit of anaphase promoting complex	-	+	+	-	-	-	GB10871-PA	AT4G33260.1	70027	[illegible]
ECP76		-	-	-	-	-	-	-	-	-	[illegible]
RNPH1	3'-5' Exoribonuclease PH component of the Exosome	-	+	+	-	-	-	GB17566-PA	AT3G61620.1	-	[illegible]
		-	+	-	-	-	-	GB17413-PA	AT3G17205.1	52421	[illegible]
CNX1C	molybdopterin biosynthesis protein	-	+	-	-	-	-	-	-	-	[illegible]
SYP6	Qc-SNARE, Tlg1/Syntaxin 6-family	-	+	-	-	-	-	GB13262-PA	AT1G28490.1	69625	[illegible]
		+	+	-	-	-	-	GB14717-PA	-	-	[illegible]
		-	+	+	-	-	-	GB13265-PA	AT1G12910.1	52888	[illegible]
		-	-	-	-	-	-	-	AT3G51510.1	-	[illegible]
MKS1		+	+	-	-	-	-	GB11632-PA	-	28991	[illegible]
		-	+	-	-	-	-	-	AT1G57770.1	21551	[illegible]
		-	+	-	-	-	-	GB15807-PA	AT2G14170.2	26083	[illegible]
GMP1	GDP-D-mannose pyrophosphorylase	-	-	-	-	-	-	-	-	-	[illegible]
Lhcb5		-	+	-	-	-	-	-	AT4G10340.1	-	[illegible]
D1bLIC	Cytoplasmic dynein 1b light intermediate chain	+	+	-	+	-	-	GB17212-PA	-	2677	[illegible]
		-	+	-	-	-	-	-	AT2G23390.1	-	[illegible]
VSP6	hydroxyproline-rich cell wall protein	-	-	-	-	-	-	-	-	-	[illegible]
Tctex2b	Similar to ODA-LC2	+	+	-	+	-	-	GB11051-PA	-	32194	[illegible]
CSN5		-	+	+	-	-	-	GB12145-PA	AT1G22920.2	4008	[illegible]
		-	+	-	-	-	-	GB12522-PA	-	-	[illegible]
DHC2	Dynein heavy chain 2	+	+	-	+	-	-	-	-	38572	[illegible]
PSBO	Oxygen-evolving enhancer protein 1 of photosystem II	-	+	-	-	-	-	-	AT5G66570.1	69678	[illegible]
GIDB1	methyltransferase, related to glucose inhibited division protein	-	+	-	-	-	-	-	AT5G50110.1	15293	[illegible]
		-	+	-	-	-	-	-	AT5G44370.1	-	[illegible]
		-	+	-	-	-	-	-	AT1G32200.2	3183	[illegible]
DYRK1	Dual-Specificity Tyrosine Regulated Protein Kinase Homolog 1	-	+	+	-	-	-	GB14600-PA	AT5G35980.2	37982	[illegible]
		-	-	-	-	-	-	-	-	-	[illegible]
MRPS12	Putative mitochondrial ribosomal protein S12, imported to mitochondria	-	+	-	-	-	-	GB10531-PA	ATCG00905.1	-	[illegible]
		-	+	-	-	-	-	GB18768-PA	AT1G73820.1	-	[illegible]
MAD2		-	+	-	-	-	-	GB12183-PA	AT3G25980.1	19390	[illegible]
		-	+	-	-	-	-	GB13855-PA	AT5G18070.1	10873	[illegible]
		-	+	-	-	-	-	GB19218-PA	AT4G24740.1	69555	[illegible]
		-	+	+	-	-	-	GB14977-PA	AT2G26060.1	3752	[illegible]
		-	-	-	-	-	-	-	-	-	[illegible]
MFT11	major facilitator superfamily protein	-	+	-	-	-	-	-	AT2G29650.3	-	[illegible]
MMP3		-	-	-	-	-	-	-	-	-	[illegible]
NRT2;3		-	+	-	-	-	-	-	-	24718	[illegible]
VTE4		-	+	-	-	-	-	-	AT1G64970.1	3921	[illegible]
ACK1	acetate kinase	-	+	-	-	-	-	-	-	-	[illegible]
CCT6	T-complex protein , zeta subunit	-	+	+	-	-	-	GB10382-PA	AT3G02530.1	54894	[illegible]
AOX1		-	+	-	+	-	-	-	-	-	[illegible]
BBS4		-	+	-	-	-	-	GB15760-PA	-	1883	[illegible]
FEA1	Fe-assimilating protein 1	-	-	-	-	-	-	-	-	-	[illegible]
		-	+	-	-	-	-	GB15471-PA	AT1G71900.1	-	[illegible]
TUB1		-	-	-	-	-	-	-	-	-	[illegible]
FDH1	formaldehyde dehydrogenase	-	+	-	+	-	-	GB16353-PA	AT5G43940.1	65636	[illegible]
TUB2		-	+	+	+	-	-	GB13049-PA	AT5G62700.1	29718	[illegible]
		-	+	+	-	-	-	GB17383-PA	AT5G07370.4	-	[illegible]
RPOIII2		-	+	+	-	-	-	GB14838-PA	AT5G45140.1	39265	[illegible]
PUF3	pumilio family (Puf) protein	-	+	-	-	-	-	-	AT1G78160.1	-	[illegible]
	hypothetical protein	-	+	-	-	+	-	GB10046-PA	AT3G51040.3	15834	[illegible]
RPL13	Ribosomal protein L13, component of cytosolic 80S ribosome and 60S large subunit	-	+	+	-	-	-	GB17286-PA	AT3G49010.3	7898	[illegible]
		-	+	-	-	-	-	-	-	-	[illegible]
PRP6		-	+	+	-	-	-	GB14450-PA	AT4G03430.1	38946	[illegible]
		-	-	-	-	-	-	-	-	59635	[illegible]
VTE3		-	+	-	-	-	-	-	AT3G63410.1	-	[illegible]
CHLRE_30181	RabGAP/TBC Domain Protein	-	+	+	-	-	-	GB13802-PA	AT4G13730.1	33549	[illegible]
RPS21	Ribosomal protein S21, component of cytosolic 80S ribosome and 40S small subunit	-	+	+	-	-	-	GB18769-PA	AT5G27700.1	28476	[illegible]
		-	+	-	-	-	-	-	AT2G33255.1	19633	[illegible]
SELL1	Sel-1 like protein	-	-	-	-	-	-	-	-	-	[illegible]
		-	+	+	-	-	-	GB13156-PA	AT1G09770.1	25571	[illegible]
AAD1	acetohydroxyacid dehydratase	-	+	-	-	-	-	-	AT3G23940.1	54952	[illegible]
SAH1	S-Adenosyl homocysteine hydrolase	-	+	+	+	-	-	GB14324-PA	AT4G13940.1	69644	[illegible]
		-	+	-	+	-	-	-	AT1G31970.1	26912	[illegible]
ATR1	aminotransferase, putative	-	+	-	-	-	-	-	AT4G33680.1	36703	[illegible]
FOX2	multicopper oxidase	-	-	-	-	-	-	-	-	-	[illegible]
		-	-	-	-	-	-	-	-	-	[illegible]
PBA2		-	+	-	-	-	-	GB18905-PA	-	52186	[illegible]
GLN2	glutamine synthetase, plastid precursor	-	-	-	-	-	-	-	-	-	[illegible]
ODA9	Flagellar outer dynein arm intemediate chain 1	+	+	-	+	-	-	GB19143-PA	-	30500	[illegible]
PRP8	Splicing factor, component of the U5 snRNP and of the spliceosome	-	+	+	-	-	-	GB18354-PA	AT1G80070.1	54877	[illegible]
		-	+	-	-	-	-	-	AT3G47780.1	72402	[illegible]
FAK1	ODA5-associated flagellar adenylate kinase	-	+	-	+	-	-	-	-	35017	[illegible]
		+	+	-	-	-	-	GB11772-PA	-	63797	[illegible]
		-	+	-	-	-	-	-	-	25630	[illegible]
	beta-amylase	-	-	-	-	-	-	-	-	-	[illegible]
		-	+	-	-	-	-	GB12051-PA	AT1G55860.1	55365	[illegible]
Nar1.6		-	-	-	-	-	-	-	-	-	[illegible]
FAP156	FAP156, small Rab-realted GTPase	+	+	-	+	-	-	-	-	6700	[illegible]
RIP1	Rieske iron-sulfur protein of mitochondrial ubiquinol-cytochrome c reductase.	-	+	-	-	-	-	-	AT5G13430.1	52117	[illegible]
		-	+	-	-	-	-	-	-	63926	[illegible]
HSP22F	heat shock protein 22F	-	-	-	-	-	-	-	-	-	[illegible]
		-	+	-	-	-	-	GB19498-PA	AT1G16890.2	7935	[illegible]
ATPvH	Probable vacuolar ATP synthase subunit H	-	+	+	-	-	-	GB17480-PA	AT3G42050.1	18324	[illegible]
ACH1	Aconitate hydratase, mitochondrial	-	+	-	-	-	-	GB12488-PA	AT4G26970.1	-	[illegible]
GAP3	Glyceraldehyde-3-Phosphate Dehydrogenase	-	+	-	+	-	-	-	AT1G42970.1	-	[illegible]
HLA8		-	+	-	-	-	-	-	AT2G40410.2	-	[illegible]
SRP14	Subunit of the Signal Recognition Particle	-	+	-	-	-	-	GB15372-PA	AT2G43640.2	-	[illegible]
IPB1	Importin beta	-	+	-	-	-	-	GB16423-PA	AT5G53480.1	55562	[illegible]
SULP,SULP1	membrane component of ATP-dependent sulfate-transport system of chloroplast enveloppe	-	-	-	-	-	-	-	-	-	[illegible]
LHCA9	light-harvesting protein of photosystem I;	-	-	-	-	-	-	-	-	-	[illegible]
		-	-	-	-	-	-	-	-	-	[illegible]
TPR5	Conserved Flagellar Associated Coiled-Coil Protein	+	+	-	-	-	-	GB14143-PA	-	72573	[illegible]
		-	+	+	-	-	-	GB14943-PA	AT1G52980.1	72347	[illegible]
VAMP71	R-SNARE, VAMP7-family	-	-	-	-	-	-	-	-	-	[illegible]
CCP2	low-CO2-inducible 36kD chloroplast envelope protein	-	+	-	-	-	-	GB11606-PA	-	-	[illegible]
		-	+	+	-	-	-	GB13023-PA	AT1G80460.1	26073	[illegible]
ARLC2	ARLC2, small Arf-like-realted GTPase	+	+	-	+	-	-	GB17719-PA	-	32239	[illegible]
RCA	rubisco activase	-	+	-	-	-	-	-	AT2G39730.3	-	[illegible]
CAH2	Carbonic anhydrase, alpha type, periplasmic	-	-	-	-	-	-	-	-	63566	[illegible]
		-	+	-	-	-	-	-	AT2G01170.1	-	[illegible]
ASP	aspartic proteinase	-	+	-	-	-	-	GB16903-PA	AT1G11910.1	23053	[illegible]
		-	+	-	-	-	-	-	AT3G09085.1	-	[illegible]
		-	+	-	-	-	-	GB12308-PA	AT5G11680.1	-	[illegible]
IF3B		-	+	+	-	-	-	GB10123-PA	AT5G27640.1	72539	[illegible]
GLH1	glycoside-hydrolase-like protein	-	+	+	-	-	-	GB11040-PA	AT5G63840.1	70514	[illegible]
		-	+	-	-	-	-	-	AT5G06060.1	19598	[illegible]
		-	+	-	-	-	-	-	AT1G16280.1	-	[illegible]
		-	-	-	-	-	-	-	-	-	[illegible]
		-	+	+	-	-	-	GB14797-PA	AT5G59440.3	35261	[illegible]
ODA13	Flagellar outer dynein arm light chain 6	-	-	-	+	-	-	-	-	-	[illegible]
STA6	ADP-glucose pyrophosphorylase small subunit	-	+	-	-	-	-	-	AT5G48300.1	-	[illegible]
CDS1a		-	+	-	-	-	-	GB15128-PA	AT1G62430.1	3513	[illegible]

TUA1		-	-	-	-	-	-	-	[illegible]
RAN1	RAN1, Ran-like small GTPase	-	+	+	-	-	-	GB13869-PA	[illegible]
ACK2	Acetate Kinase (Acetokinase)	-	-	-	-	-	-	-	[illegible]
		-	+	+	-	-	-	GB18035-PA	[illegible]
CDPK1	Calcium-dependent Protein Kinase Homolog 1	-	+	-	+	-	-	-	[illegible]
	Conserved protein of unknown function	-	+	-	-	-	-	GB11201-PA	[illegible]
		-	+	+	-	-	-	GB15998-PA	[illegible]
TAB2	RNA binding protein	-	+	-	-	-	-	-	[illegible]
NRAMP1	natural resistance-associated macrophage protein; manganese transport protein	-	+	-	-	-	-	-	[illegible]
POA1	20S proteasome alpha subunit A	-	+	+	+	-	-	-	[illegible]
		-	+	-	-	-	-	-	[illegible]
FMN/FHY	Putative riboflavin kinase/FMN hydrolase	-	+	-	-	-	-	GB12439-PA	[illegible]
		-	+	-	-	-	-	-	[illegible]
HPR1	Hydroxypyruvate reductase	-	+	-	-	-	-	-	[illegible]
RPL36a	Ribosomal protein L36a, component of cytosolic 80S ribosome and 60S large subunit	-	+	+	-	-	-	GB15143-PA	[illegible]
ClpR6	inactive subunit of chloroplast ClpP complex	-	-	-	-	-	-	-	[illegible]
		-	+	-	-	-	-	GB17304-PA	[illegible]
		-	+	-	-	-	-	GB10979-PA	[illegible]
		-	+	-	-	-	-	GB14673-PA	[illegible]
		-	+	-	-	-	-	-	[illegible]
HYDEF		-	-	-	-	-	-	-	[illegible]
		-	+	-	-	-	-	GB16540-PA	[illegible]
		-	+	-	-	-	-	-	[illegible]
		-	+	-	-	-	-	GB13850-PA	[illegible]
FAP52	Conserved Uncharacterized Flagellar Associated Protein	+	+	-	+	-	-	GB13234-PA	[illegible]
ACA2	plasma membrane calcium ATPase	-	+	+	+	-	-	GB13865-PA	[illegible]
		-	+	+	-	-	-	GB13589-PA	[illegible]
SUFS2	cysteine desulfurase (selenocysteine lyase)	-	-	-	-	-	-	-	[illegible]
		-	+	+	-	-	-	GB16722-PA	[illegible]
		-	+	-	-	-	-	-	[illegible]
	RabGAP/TBC Domain Protein	-	+	+	-	-	-	GB11670-PA	[illegible]
SKP1		-	+	+	-	-	-	GB18508-PA	[illegible]
CTH1	Copper target homolog 1, untranslated and nonfunctional alternative spliced	-	+	-	-	-	-	-	[illegible]
SPLH1	DEAH-box nuclear pre-mRNA splicing factor	-	+	+	-	-	-	GB16946-PA	[illegible]
SF-assemblin		-	+	-	+	-	-	-	[illegible]
		-	+	+	-	-	-	GB11429-PA	[illegible]
GTR25	oligsacharyl transferase STT3 subunit	-	+	+	-	-	-	GB15818-PA	[illegible]
CCT1	T-complex protein 1, alpha subunit	-	+	+	-	-	-	GB20080-PA	[illegible]
		-	+	-	-	-	-	-	[illegible]
		-	+	+	-	-	-	GB15856-PA	[illegible]
NOP58	Nucleolar protein, Component of C/D snoRNPs	-	+	+	-	-	-	GB17044-PA	[illegible]
		-	+	-	-	-	-	GB19930-PA	[illegible]
		-	+	-	-	-	-	-	[illegible]
CCT3	T-complex protein 1, gamma subunit	-	+	+	-	-	-	GB10800-PA	[illegible]
HSP70G	Heat shock protein 70G	-	+	-	-	-	-	GB10732-PA	[illegible]
		-	+	-	-	-	-	-	[illegible]
	EMP/nonaspanin domain family	-	+	-	-	-	-	GB19423-PA	[illegible]
		-	+	-	-	-	-	-	[illegible]
		-	+	-	-	-	-	-	[illegible]
PGP2	Phosphoglycolate phosphatase, chloroplast precursor	-	-	-	-	-	-	-	[illegible]
		-	+	-	-	-	-	-	[illegible]
		-	-	-	-	-	-	-	[illegible]
PDC1	pyruvate decarboxylase	-	+	-	-	-	-	-	[illegible]
GOX	Glycolate oxidase	-	+	-	-	-	-	GB13505-PA	[illegible]
		-	+	-	-	-	-	GB15798-PA	[illegible]
		-	+	+	-	-	-	GB16315-PA	[illegible]
		-	-	-	-	-	-	-	[illegible]
		+	+	-	-	-	-	GB14088-PA	[illegible]
		-	+	-	-	-	-	GB10311-PA	[illegible]
		-	+	+	-	-	-	GB11758-PA	[illegible]
MCM6	minichromosome maintenance protein 6	-	+	+	-	-	-	GB19871-PA	[illegible]
NUOB13	NADH:ubiquinone oxidoreductase (Complex I) 18 kD subunit, homolog to bovine B13 subunit, mitochondrial	-	-	-	-	-	-	-	[illegible]
PP2A2	PP2A-widerborst subunit	-	+	-	-	-	-	GB17069-PA	[illegible]
		-	+	+	-	-	-	GB16589-PA	[illegible]
		-	+	+	-	-	-	GB15729-PA	[illegible]
		-	+	+	-	-	-	GB14769-PA	[illegible]
		-	+	-	-	-	-	-	[illegible]
FTHFS	Putative 10-formyltetrahydrofolate synthetase	-	+	-	-	-	-	GB13160-PA	[illegible]
		-	+	-	-	-	-	GB18073-PA	[illegible]
FCL2	Predicted 5-Formyltetrahydrofolate cycloligase (5,10-methenytetrahydrofolate synthetase)	-	+	-	-	-	-	GB19969-PA	[illegible]
ITN7		-	+	-	-	-	-	-	[illegible]
SRP54	Subunit of the Signal Recognition Particle	-	+	+	-	-	-	GB30580-PA	[illegible]
C_930043		-	-	-	-	-	-	-	[illegible]
		-	+	+	-	-	-	GB16818-PA	[illegible]
CDD1	cytidine deaminase	-	+	+	-	-	-	GB18075-PA	[illegible]
MCM7	minichromosome maintenance protein 7	-	+	+	-	-	-	GB12635-PA	[illegible]
COX19	cytochrome c oxidase (Complex IV) assembly protein	-	+	-	-	-	-	-	[illegible]
THS1	threonine synthase, putative	-	+	-	-	-	-	-	[illegible]
FLU	FLU chloroplast precursor	-	+	-	-	-	-	-	[illegible]
NUO5	NADH:ubiquinone oxidoreductase (Complex I) 24 kDa subunit, homolog to bovine 24 kD subunit, mitochondrial	-	+	-	-	-	-	GB17038-PA	[illegible]
PBE1		-	+	+	-	-	-	GB19200-PA	[illegible]
CDKA1	cyclin dependent protein kinase	-	+	+	-	-	-	GB19434-PA	[illegible]
AAT1	alanine aminotranferase	-	+	+	-	-	-	GB17641-PA	[illegible]
RPL35a	Ribosomal protein L35a, component of cytosolic 80S ribosome and 60S large subunit	-	+	+	-	-	-	GB12441-PA	[illegible]
HRP3	hydroxyproline-rich cell wall protein	-	-	-	-	-	-	-	[illegible]
		-	+	-	-	-	-	GB19131-PA	[illegible]
	Uncharacterised ARF-like GTPase domain protein	-	-	-	-	-	-	-	[illegible]
VDAC	VDAC, Voltage-dependent anion-selective channel protein, mitochondrial precursor.	-	+	-	-	-	-	GB12113-PA	[illegible]
RRP40	3'-5' Exonuclease component of the Exosome	-	+	+	-	-	-	GB11366-PA	[illegible]
MAPKKKK1	MAP Kinase Kinase Kinase Kinase Homolog 1	-	+	+	-	-	-	GB18142-PA	[illegible]
CUL1		-	+	-	-	-	-	GB16348-PA	[illegible]
PGR5	thylakoid membrane protein	-	+	-	-	-	-	-	[illegible]
		-	-	-	-	-	-	GB12625-PA	[illegible]
		-	+	-	-	-	-	-	[illegible]
		-	+	-	-	-	-	-	[illegible]
RPN3	26S proteasome regulatory subunit RPN3	-	+	+	-	-	-	GB12827-PA	[illegible]
		-	+	-	-	+	-	GB14371-PA	[illegible]
		-	+	-	-	-	-	GB17494-PA	[illegible]
PRPL10	Plastid ribosomal protein L10, imported to chloroplast, large ribosomal subunit	-	+	-	+	-	-	-	[illegible]
AAT4	aspartate aminotransferase, putative	-	+	-	-	-	-	-	[illegible]
THI10	Hydroxyethylthiazole kinase	-	+	-	-	-	-	-	[illegible]
FAP60	Conserved Uncharacterized Flagellar Associated Protein	+	+	-	+	-	-	GB10762-PA	[illegible]
LEU1	isopropylmalate dehydratase	-	+	-	-	-	-	-	[illegible]
RPL37a	Ribosomal protein L37a, component of cytosolic 80S ribosome and 60S large subunit	-	+	+	-	-	-	GB19435-PA	[illegible]
RAB23	RAB23, small Rab-related GTPase	-	+	-	-	-	-	GB16875-PA	[illegible]
HSP70B	Heat shock protein 70B	-	+	-	-	-	-	-	[illegible]
MME6	NADP-dependent malic enzyme	-	+	-	-	-	-	GB16951-PA	[illegible]
NAB1	nucleic acid binding protein	-	+	-	-	-	-	-	[illegible]
CDKG1	cyclin dependent kinase, Chlamydomonas specific	-	-	-	-	-	-	-	[illegible]
		+	+	-	-	-	-	GB16076-PA	[illegible]
		-	-	-	-	-	-	-	[illegible]
		-	+	+	-	-	-	GB16073-PA	[illegible]
		-	+	-	-	-	-	-	[illegible]
		-	+	-	-	-	-	GB30530-PA_2	[illegible]
IF2G		-	+	+	-	-	-	GB11237-PA	[illegible]
CPY3		-	+	-	-	-	-	-	[illegible]
		-	+	-	-	-	-	GB10198-PA	[illegible]
		-	+	-	-	-	-	-	[illegible]
DHC4	Dynein heavy chain 4	-	-	-	+	-	-	-	[illegible]
		-	+	-	-	-	-	-	[illegible]
		+	+	-	-	-	-	GB11147-PA	[illegible]
IF5A	eukaryotic translation initiation factor 5A	-	+	+	-	-	-	GB18770-PA	[illegible]
FAP204	Flagellar Associated Protein	-	+	-	+	-	-	GB13161-PA	[illegible]
DPS1	dihydrodipicolinate synthase	-	+	-	-	-	-	-	[illegible]
	Serine/threonine protein kinase	-	-	-	-	-	-	-	[illegible]
RPB146aa		-	+	+	-	-	-	GB10191-PA	[illegible]
DHC5	Dynein heavy chain 5	-	-	-	+	-	-	-	[illegible]
		-	+	-	-	-	-	GB18680-PA	[illegible]
		-	+	+	-	-	-	GB10297-PA	[illegible]
ATPvB	Vacuolar ATP synthase subunit B	-	+	+	+	-	-	GB19171-PA	[illegible]
COP-5		-	+	-	-	-	-	-	[illegible]
CPX2	Coproporphyrinogen III oxidase, mitochondrial or chloroplast precursor	-	-	-	-	-	-	-	[illegible]
GUA1	GMP synthetase	-	+	-	-	-	-	GB10491-PA	[illegible]
SMC2	Condensin complex subunit.	-	+	+	-	-	-	GB15360-PA	[illegible]
Rib72	Protein associated with protofilament ribbons of flagellar microtubules	+	+	-	+	-	-	GB15971-PA	[illegible]
CYP51G1	cytochrome P450, sterol 14 alpha demethylase	-	+	-	-	-	-	-	[illegible]
BCA1	branched chain amino acid aminotransferase, putative	-	+	-	-	-	-	GB14457-PA	[illegible]
		-	+	-	-	-	-	-	[illegible]
PRPL1	Plastid ribosomal protein L1, imported to chloroplast, large ribosomal subunit	-	+	-	+	-	-	-	[illegible]
RPL3	Ribosomal protein L3, component of cytosolic 80S ribosome and 60S large subunit	-	+	+	-	-	-	GB15261-PA	[illegible]
		-	+	-	-	-	-	GB10570-PA	[illegible]
		-	-	-	-	-	-	-	[illegible]
RPL37	Ribosomal protein L37, component of cytosolic 80S ribosome and 60S large subunit	-	+	+	-	-	-	GB16664-PA	[illegible]
SPL5	pre-mRNA-splicing factor	-	+	+	-	-	-	GB18617-PA	[illegible]
PAP1	Poly(A) Polymerase	-	+	+	-	-	-	GB11344-PA	[illegible]
		-	-	-	-	-	-	-	[illegible]
		-	-	-	-	-	-	-	[illegible]
IAR1		-	-	-	-	-	-	-	[illegible]
		-	+	-	-	-	-	GB18100-PA	[illegible]
		-	+	-	-	-	-	GB16851-PA	[illegible]
ABC1	ABC family protein 70 kDa	-	+	-	-	-	-	GB17168-PA	[illegible]
RPSa	Ribosomal protein Sa, component of cytosolic 80S ribosome and 40S small subunit	-	+	+	+	-	-	GB19082-PA	[illegible]
PRT1		-	+	-	-	-	-	-	[illegible]
MDH2	malate dehydrogenase	-	+	-	-	-	-	-	[illegible]
UAP56	putative UAP56, subunit of EJC	-	+	+	-	-	-	GB13214-PA	[illegible]
		-	+	-	-	-	-	GB18490-PA	[illegible]

		-	+	-	-	-	-	GB19747-PA	[illegible]
		-	+	+	-	-	-	GB19165-PA	[illegible]
PGH,PGH1	Enolase (2-Phosphoglycerate Dehydratase)	-	+	+	+	-	-	GB11216-PA	[illegible]
RBCS1	ribulose-1,5-bisphosphate carboxylase/oxygenase small subunit 1, chloroplast precursor	-	+	-	+	-	-	-	[illegible]
		-	+	+	-	-	-	GB13467-PA	[illegible]
GDH2	glutamate dehydrogenase	-	+	-	-	-	-	GB15079-PA	[illegible]
	related to haem peroxidases	-	-	-	-	-	-	-	[illegible]
		-	-	-	-	-	-	-	[illegible]
		-	-	-	-	-	-	-	[illegible]
		-	+	-	-	-	-	GB16209-PA	[illegible]
		-	+	-	-	-	-	-	[illegible]
		-	+	-	-	-	-	-	[illegible]
		-	+	-	-	-	-	GB30492-PA	[illegible]
RPP1	Acidic ribosomal protein P1, component of cytosolic 80S ribosome and 60S large subunit	-	+	+	-	-	-	GB12230-PA	[illegible]
		-	-	-	-	-	-	-	[illegible]
SAR1	SAR1, Sar-type small GTPase	-	+	+	-	-	-	GB19208-PB	[illegible]
		-	+	-	-	-	-	-	[illegible]
		-	-	-	-	-	-	-	[illegible]
THB1	truncated hemoglobin, plastid-located	-	-	-	-	-	-	-	[illegible]
		-	+	-	-	-	-	GB19514-PA	[illegible]
DYF13	required for ciliogenesis	+	+	-	-	-	-	GB11148-PA	[illegible]
QCR6	Ubiquinol:cytochrome c oxidoreductase (Complex III) 8 kDa subunit, mitochondrial	-	+	-	-	-	-	GB12164-PA	[illegible]
RPN10	26S proteasome regulatory subunit RPN10	-	+	+	-	-	-	GB16189-PA	[illegible]
LCI11	low-CO2-inducible membrane protein	-	-	-	-	-	-	-	[illegible]
GPM1a	Phosphoglucomutase, plastid form	-	+	+	-	-	-	GB13237-PA	[illegible]
FFC	Chloroplast SRP54 Subunit of Signal Recognition Particle	-	+	-	-	-	-	-	[illegible]
		-	-	-	-	-	-	-	[illegible]
RABF1	RABF1, small Rab-related GTPase	-	+	+	-	-	-	GB15021-PA	[illegible]
TSA		-	+	-	-	-	-	-	[illegible]
		-	-	-	-	-	-	-	[illegible]
		-	+	-	-	-	-	-	[illegible]
MRPS14	Putative mitochondrial ribosomal protein S14, imported to mitochondria	-	+	-	-	-	-	GB11759-PA	[illegible]
		-	-	-	-	-	-	-	[illegible]
		-	-	-	-	-	-	-	[illegible]
		-	+	-	-	-	-	GB11071-PA	[illegible]
PF15	katanin p80 subunit	-	+	-	-	+	-	GB14488-PA	[illegible]
		-	+	+	-	-	-	GB11667-PA	[illegible]
MDH5	NADP-malate dehydrogenase	-	-	-	-	-	-	-	[illegible]
ANT1	mitochondrial ADP/ATP translocator protein	-	+	-	-	-	-	GB17499-PA	[illegible]
SNF7	component of the ESCRT-III complex	-	+	-	-	-	-	GB15628-PA	[illegible]
PDE11	3',5'-cyclic-nucleotide phosphodiesterase	-	-	-	-	-	-	-	[illegible]
NUOB8	NADH:ubiquinone oxidoreductase 11 kD protein (Complex I B8 subunit), mitochondrial	-	+	-	-	-	-	GB15438-PA	[illegible]
FKB16-2	FKBP-type peptidyl-prolyl cis-trans isomerase	-	-	-	-	-	-	-	[illegible]
		-	+	-	-	-	-	GB13251-PA	[illegible]
LCI2	Low CO2 inducible gene	-	-	-	-	-	-	-	[illegible]
		-	+	-	-	-	-	-	[illegible]
PEPC1	Phosphoenolpyruvate carboxylase, isoform 1	-	+	-	-	-	-	-	[illegible]
		-	-	-	-	-	-	-	[illegible]
		-	+	-	-	-	-	-	[illegible]
		-	-	-	-	-	-	-	[illegible]
PRPS1	Plastid ribosomal protein S1, imported to chloroplast, small ribosomal subunit	-	+	-	+	-	-	-	[illegible]
		-	-	-	-	-	-	-	[illegible]
		-	+	-	-	-	-	-	[illegible]
RBX1		-	+	+	-	-	-	GB11710-PA	[illegible]
		-	+	-	-	-	-	-	[illegible]
MOC1	Putative mitochondrial transcription termination factor.	-	+	-	-	-	-	-	[illegible]
		-	+	-	-	-	-	-	[illegible]
IF3I		-	+	+	-	-	-	GB13416-PA	[illegible]
SELL2	Sel-1 like protein	-	+	-	-	-	-	GB13395-PA	[illegible]
OGD1	2-oxoglutarate dehydrogenase, E1 subunit	-	+	-	+	-	-	GB12855-PA	[illegible]
FKB16-3	peptidyl-prolyl cis-trans isomerase, FKBP-type	-	+	-	-	-	-	-	[illegible]
		-	+	+	-	-	-	GB17735-PA	[illegible]
		-	-	-	-	-	-	-	[illegible]
		-	+	-	+	-	-	-	[illegible]
EPT	CDP-Ethanolamine:DAG Ethanolamine phosphotransferase	-	+	+	-	-	-	GB10071-PA	[illegible]
NUOS1	NADH:ubiquinone oxidoreductase 76 kD protein (Complex I 78 kD subunit), mitochondrial	-	+	-	-	-	-	GB19481-PA	[illegible]
		-	-	-	-	-	-	-	[illegible]
ASB1		-	+	-	-	-	-	-	[illegible]
	Acetylglutamate kinase-like protein, chloroplast precursor	-	+	-	-	-	-	-	[illegible]
GME1	GDP-D-mannose-3,5-epimerase	-	-	-	-	-	-	-	[illegible]
CRT2	Calreticulin 2, calcium-binding protein	-	+	-	-	-	-	GB17282-PA	[illegible]
NTRC	NADPH-dependent thioredoxin reductase C type (NTR/thioredoxin fusion), probably chloroplastic	-	+	-	-	-	-	-	[illegible]
		-	+	-	-	-	-	-	[illegible]
		-	+	-	-	-	-	-	[illegible]
		-	-	-	-	-	-	-	[illegible]
ASA1	F1Fo ATP synthase Associated 60,6 kDa protein 1, mitochondrial	-	-	-	-	-	-	-	[illegible]
PGD1	D-3-phosphoglycerate dehydrogenase	-	+	+	-	-	-	GB18599-PA	[illegible]
C_40034		-	+	-	-	-	-	GB11461-PA	[illegible]
		-	-	-	-	-	-	-	[illegible]
Lhca7		-	-	-	-	-	-	-	[illegible]
		-	-	-	-	-	-	-	[illegible]
ATP2	Beta subunit of mitochondrial ATP synthase.	-	+	-	+	-	-	GB13596-PA	[illegible]
COPG1	Gamma-COP	-	+	+	-	-	-	GB14298-PB	[illegible]
ARFB1	ARFB1, small Arf-related GTPase	-	+	-	-	-	-	-	[illegible]
PDS	phytoene desaturase, putative chloroplast precursor	-	+	-	-	-	-	-	[illegible]
RPL31	Ribosomal protein L31, component of cytosolic 80S ribosome and 60S large subunit	-	+	+	-	-	-	GB18564-PA	[illegible]
RPS23	Ribosomal protein S23, component of cytosolic 80S ribosome and 40S small subunit	-	+	+	-	-	-	GB18191-PA	[illegible]
MAPK3	Mitogen-Activated Protein Kinase Homolog 3	-	+	-	-	-	-	-	[illegible]
RSP16	Radial spoke protein 16	-	-	-	+	-	-	-	[illegible]
		-	+	+	-	-	-	GB18137-PA	[illegible]
FAP207	Flagellar Associated Protein	+	+	-	+	-	-	GB18437-PA	[illegible]
		-	-	-	-	-	-	-	[illegible]
RIB43a	Coiled-coil protein associated with protofilament ribbons of flagellar microtubules	+	+	-	+	-	-	GB11512-PA	[illegible]
		-	-	-	-	-	-	-	[illegible]
AOX2		-	+	-	-	-	-	-	[illegible]
		-	-	-	-	-	-	-	[illegible]
		-	-	-	-	-	-	-	[illegible]
		-	+	-	-	-	-	-	[illegible]
FKB12	peptidyl-prolyl cis-trans isomerase, FKBP-type	-	+	-	+	-	-	GB13529-PA	[illegible]
RPL17	Ribosomal protein L17, component of cytosolic 80S ribosome and 60S large subunit	-	+	+	-	-	-	GB17564-PA	[illegible]
		-	+	-	-	-	-	-	[illegible]
		-	+	-	-	-	-	GB15277-PA	[illegible]
		-	+	-	-	-	-	-	[illegible]
		-	-	-	-	-	-	-	[illegible]
		-	+	+	-	-	-	GB18630-PA	[illegible]
NUOA1	NADH:ubiquinone oxidoreductase 8 kDa subunit (Complex I MWFE subunit), mitochondrial	-	-	-	-	-	-	-	[illegible]
		-	-	-	-	-	-	-	[illegible]
BTA1		-	+	-	-	-	-	-	[illegible]
	calcium-transporting ATPase, endoplasmic reticulum-type	-	+	-	-	-	-	GB17876-PA	[illegible]
QCR7	Ubiquinol:cytochrome c oxidoredutase (Complex III) 14 kDa subunit subunit	-	+	-	-	-	-	GB11150-PA	[illegible]
		-	+	-	-	-	-	GB18325-PA	[illegible]
		-	-	-	-	-	-	-	[illegible]
		-	-	-	-	-	-	-	[illegible]
METH	Vitamin B12 (cobalamin) dependent methionine synthase	-	+	-	-	-	-	GB19746-PA	[illegible]
		-	-	-	-	-	-	-	[illegible]
		-	+	-	-	-	-	GB18704-PA	[illegible]
		-	+	-	-	-	-	-	[illegible]
ATP1	F1Fo ATP synthase, alpha subunit, mitochondrial	-	+	-	+	-	-	GB14791-PA	[illegible]
		-	+	+	-	-	-	GB14386-PA	[illegible]
FAP280	Flagellar Associated Protein Similar to Transcriptional Coactivator-Like Protein	-	+	+	-	-	-	GB16042-PA	[illegible]
		-	+	+	-	-	-	GB19368-PA	[illegible]
AMT6	ammonia channel	-	-	-	-	-	-	-	[illegible]
		-	+	-	-	-	-	GB11477-PA	[illegible]
RPL15	Ribosomal protein L15, component of cytosolic 80S ribosome and 60S large subunit	-	+	+	-	-	-	GB16952-PA	[illegible]
		-	-	-	-	-	-	-	[illegible]
SPO11a	Topoisomerase, involved in meiosis.	-	+	-	-	-	-	GB13628-PA	[illegible]
		-	-	-	-	-	-	-	[illegible]
PSAE		-	+	-	-	-	-	-	[illegible]
		-	+	+	-	-	-	GB13635-PA	[illegible]
		-	-	-	-	-	-	-	[illegible]
MMP1	Matrix Metalloprotease 1	-	-	-	-	-	-	-	[illegible]
RABD1	RABD1, small Rab-related GTPase	-	+	+	-	-	-	GB19166-PA	[illegible]
MDHM	Malate dehydrogenase	-	-	-	-	-	-	-	[illegible]
PF9	Flagellar inner arm dynein 1 heavy chain alpha	-	+	-	+	-	-	GB11197-PA	[illegible]
XAB2		-	+	+	-	-	-	GB15804-PA	[illegible]
TEF8	Protein of unknown function DUF477, chloroplast location proposed	-	+	-	-	-	-	-	[illegible]
RBCS2	ribulose-1,5-bisphosphate carboxylase/oxygenase small subunit 2,chloroplast precursor	-	-	-	-	-	-	-	[illegible]
TPPB	trehalose 6-phosphate phosphatase	-	+	-	-	-	-	-	[illegible]
		-	+	-	-	-	-	-	[illegible]
CPH1	deoxyribodipyrimidine photolyase, class 1	-	+	-	-	-	-	-	[illegible]
		-	+	-	-	-	-	-	[illegible]
SC5D	C-5 sterol desaturase	-	+	-	-	-	-	-	[illegible]
CDKB1	plant specific cyclin dependent kinase	-	+	-	-	-	-	-	[illegible]
IDS	4-hydroxy-3-methylbut-2-enyl diphosphate reductase, putative chloroplast precursor	-	+	-	-	-	-	-	[illegible]
SUOX	sulfite oxidase, mitochondrial precursor	-	+	-	-	-	-	GB19854-PA	[illegible]
FBA3	fructose-1,6-bisphosphate aldolase	-	-	-	-	-	-	-	[illegible]
RPS15a	Ribosomal protein S15a, component of cytosolic 80S ribosome and 40S small subunit	-	+	+	-	-	-	GB19442-PA	[illegible]
PSY,PSY1	phytoene synthase, putative chloroplast precursor	-	+	-	-	-	-	-	[illegible]
HNR1	Heterogeneous Nuclear Ribonucleoprotein	-	-	-	+	-	-	-	[illegible]
		-	+	+	-	-	-	GB19950-PA	[illegible]
		-	+	-	-	-	-	GB16471-PA	[illegible]
		-	-	-	-	-	-	-	[illegible]
		-	+	+	-	-	-	GB10968-PA	[illegible]
	Likely di or tri-carboxylate translocator (glutamate/malate translocator)	-	-	-	-	-	-	-	[illegible]
		-	+	-	-	-	-	-	[illegible]
		-	-	-	-	-	-	-	[illegible]

		-	-	-	-	-	-	-	-	-	-	-
NUOA8	NADH:ubiquinone oxidoreductase (Complex I) 12.5 kD subunit, homolog to bovine PGIV subunit, mitochondrial	-	+	-	-	-	-	-	AT3G06310.1	-	BDEG_06189	-
ATPvG	Transmembrane ATPase	-	+	-	-	-	-	-	AT3G01390.2	-	BDEG_00535	CE10604
SIR1	ferredoxin-sulfite reductase	-	+	-	+	-	-	-	AT5G04590.1	71245	-	-
APG8	Autophagy protein	-	+	+	-	-	-	GB14986-PA	AT2G05630.1	70347	BDEG_07689	CE01849
		-	-	-	-	-	-	-	-	-	-	-
		-	-	-	-	-	-	-	-	-	-	-
		-	-	-	-	-	-	-	-	-	-	-
PPR10	PPR-repeat protein	-	-	-	-	-	-	-	-	-	-	-
FAP103	Flagellar Associated Protein Similar to Nucleoside Diphosphate Kinase	-	+	-	+	-	-	GB17251-PA	AT4G09320.1	22626	BDEG_03309	CE09650
		-	-	-	-	-	-	-	-	-	-	-
MAK	MAK homolog, Male germ cell associated protein kinase homolog, Mitogen-activated protein kinase homolog 7	-	+	-	-	-	-	GB15002-PA	AT4G19110.2	71670	BDEG_02426	CE12408
		-	-	-	-	-	-	-	-	13431	-	-
NUO11	NADH:ubiquinone oxidoreductase (Complex I) ND4L subunit, mitochondrial	-	-	-	-	-	-	-	-	-	-	-
TrpRS	TrpRS, Tryptophanyl-tRNA synthetase (Tryptophan-tRNA ligase), mitochondrial precursor.	-	+	-	-	-	-	GB12719-PA	AT2G25840.1	39388	BDEG_01044	CE37710
ASO1	L-aspartate oxidase, putative	-	+	-	-	-	-	-	AT5G14760.1	-	-	-
PHB2	prohibitin	-	+	-	-	-	-	GB18647-PA	AT5G40770.1	59995	BDEG_02464	CE26775
HSD1	putative steroid dehydrogenase	-	+	-	-	-	-	-	AT1G47290.2	69617	BDEG_04228	-
		-	+	-	-	-	-	GB10127-PA	AT3G21280.1	27497	BDEG_05141	CE15615
MinE1		-	+	-	-	-	-	-	AT1G69390.1	-	-	-
FTSH2	membrane AAA-metalloprotease, chloroplast	-	+	-	-	-	-	-	AT1G06430.1	-	-	-
COQ4	ubiquinone biosynthesis protein COQ4 homolog	-	+	-	-	-	-	GB10170-PA	AT2G03690.1	26082	BDEG_04449	CE40117
EFG6		-	+	-	-	-	-	GB15374-PA	-	1185	BDEG_06040	CE21577
	alpha-amylase	-	+	-	-	-	-	GB18312-PA	AT1G76130.1	-	-	-
AGS1	arginosuccinate synthase	-	+	-	-	-	-	GB18646-PA	AT4G24830.1	26092	BDEG_03313	-
LSM5		-	+	-	-	-	-	GB18491-PA	AT5G48870.1	9330	-	CE09757
		-	+	-	-	-	-	GB18172-PA	-	70268	BDEG_01396	-
		-	-	-	-	-	-	-	-	-	-	-
DLDH1	dihydrolipoyl dehydrogenase, mitochondrial precursor	-	+	-	+	-	-	GB17626-PA	AT1G48030.2	26536	BDEG_04395	CE31971
AP1G1	Gamma-Adaptin	-	+	+	-	-	-	GB19569-PA	AT1G60070.1	38872	BDEG_04280	CE29847
UBC4		-	+	+	-	-	-	GB16281-PA	AT3G57870.1	31459	BDEG_08124	CE09784
		-	-	-	-	-	-	-	-	-	-	-
		-	+	-	-	-	-	-	-	5667	-	CE37582
RPS25	Ribosomal protein S25, component of cytosolic 80S ribosome and 40S small subunit	-	+	+	-	-	-	GB17668-PA	AT2G21580.2	59049	BDEG_00039	CE04691
		-	-	-	-	-	-	-	-	-	-	-
	conserved protein of unknown function	-	+	-	-	-	-	-	AT2G04900.1	-	BDEG_04245	CE20418
NUOB14	NADH:ubiquinone oxidoreductase (Complex I) subunit, homolog to bovine B14 subunit, mitochondrial	-	+	-	-	-	-	-	AT3G12260.1	-	BDEG_04382	-
SEC22	R-SNARE, Sec22-family	-	+	-	-	-	-	GB11322-PA	AT1G11890.1	24013	BDEG_04147	CE19895
		-	-	-	-	-	-	-	-	-	-	-
PHOB	Starch Phosphorylase, alpha-1,4-Glucan phosphorylase	-	+	+	-	-	-	GB11892-PA	AT3G46970.1	-	BDEG_06644	CE20769
CLPQ	peptidase subunit of ATP-dependent protease hslUV	-	+	-	-	-	-	-	-	-	-	-
		-	-	-	-	-	-	-	-	-	-	-
TEF25	Putative succinyl-CoA ligase, mitochondrial localization assumed	-	+	+	-	-	-	GB11655-PA	AT5G23250.1	28269	BDEG_00815	CE09611
		-	-	-	-	-	-	-	-	-	-	-
		-	+	-	-	-	-	GB15341-PA	AT3G49640.1	19007	BDEG_04978	CE20317
RPN9	26S proteasome regulatory subunit RPN9	-	+	+	-	-	-	GB16614-PA	AT5G45620.1	-	BDEG_01069	CE02329
DES6		-	+	-	-	-	-	-	AT4G30950.1	-	-	-
VALRS1	valyl-tRNA synthetase	-	+	-	-	-	-	-	AT1G14610.1	-	-	-
		-	+	-	-	-	-	GB17863-PA	-	59946	BDEG_01740	-
TIM9	Tim9, mitochondrial import inner membrane translocase subunit.	-	+	-	-	-	-	-	AT3G46560.1	23445	-	CE33518
ERV14	cornichon-like protein	-	+	-	-	-	-	-	AT1G62880.1	-	BDEG_00642	-
PIG8	p53-induced protein 8	-	+	-	-	-	-	GB12277-PA	AT4G06676.1	-	-	CE00741
		-	+	-	-	-	-	GB11680-PA	AT4G34412.1	-	-	CE17274
CYN19-1	Peptidyl-prolyl cis-trans isomerase, cyclophilin-type	-	-	-	-	-	-	-	-	-	-	-
		-	-	-	-	-	-	-	-	-	-	-
		-	-	-	-	-	-	-	-	-	-	-
FAD7		-	+	-	-	-	-	-	AT3G11170.1	-	-	-
		-	-	-	-	-	-	-	-	-	-	-
COX3	Cytochrome c oxidase subunit III	-	+	-	-	-	-	GB11138-PA	ATMG00730.1	-	-	CE34069
DEH1	RNA Helicase that enhances RNA decapping	-	+	-	-	-	-	GB16998-PA	AT4G00660.1	52587	BDEG_07386	CE00839
RPI	Ribose-5-phosphate isomerase, plastid precursor	-	+	-	-	-	-	GB13216-PA	AT3G04790.1	58899	BDEG_01538	CE00733
ARS1a	periplasmic arylsulfatase	-	-	-	-	-	-	-	-	-	-	-
		-	+	+	-	-	-	GB12189-PA	AT3G06060.1	4019	-	CE28011
CLPP5	active subuit of chloroplast ClpP complex	-	+	-	-	-	-	-	AT1G02560.1	38757	-	-
CDJ1	Chloroplast DnaJ-like protein 1	-	+	-	-	-	-	GB10850-PA	AT2G22360.1	29178	BDEG_04312	CE27991
FTSH1	membrane AAA-metalloprotease, chloroplast	-	+	-	-	-	-	-	AT5G42270.1	10538	-	-
HDS	1-hydroxy-2-methyl-2-(E)-butenyl 4-diphosphate synthase, putative chloroplast precursor	-	+	-	-	-	-	-	AT5G60600.1	71112	-	-
		-	-	-	-	-	-	-	-	-	-	-
		-	+	-	-	-	-	GB16420-PA	AT1G28120.1	26271	BDEG_04427	CE08394
RPT2	26S proteasome regulatory subunit	-	+	+	-	-	-	GB15218-PA	AT4G29040.1	-	BDEG_06506	CE09799
CPN10	Chaperonin 10	-	+	-	-	-	-	GB14205-PA	AT1G23100.1	27060	BDEG_08396	CE27242
LSM7	small nuclear riboprotein, U6 sm-like protein	-	+	-	-	-	-	GB15243-PA	AT2G03870.1	33181	BDEG_02370	CE06628
CCT8	T-complex protein , theta subunit	-	+	+	-	-	-	GB13033-PA	AT3G03960.1	-	BDEG_01620	CE35110
LCI1	low-CO2-inducible membrane protein	-	-	-	-	-	-	-	-	-	-	-
PMH1	plasma membrane hydrogen ATPase	-	-	-	-	-	-	-	-	-	-	-
		-	-	-	-	-	-	-	-	-	-	-
		-	+	+	-	-	-	GB15338-PA	AT1G06960.1	28680	BDEG_06739	CE07355
MRP3	ABC transporter, Multidrug resistance associated protein, membrane protein	-	+	+	-	-	-	GB18864-PA	AT2G34660.1	62908	BDEG_03605	-
ATPvA1	vacuolar ATP synthase, subunit A	-	+	+	+	-	-	GB10989-PA	AT1G78900.2	37110	BDEG_07758	CE22210
		-	+	-	-	-	-	-	AT2G24020.1	-	-	-
NUOB12	NADH:ubiquinone oxidoreductase 7 kDa subunit (Complex I B12 subunit), mitochondrial	-	-	-	-	-	-	-	AT1G14450.1	-	-	-
		-	+	-	-	-	-	GB15500-PA	AT3G55010.2	-	BDEG_08236	CE28304
PF26	Radial spoke protein 6	-	+	-	+	-	-	-	-	-	-	-
POLA4	Primase subunit of DNA polymerase alpha.	-	+	-	-	-	-	GB10597-PA	AT5G41880.1	58835	BDEG_06357	CE00220
		-	+	-	-	-	-	-	-	7400	-	-
	UNC-50 family protein	-	+	-	-	-	-	GB12521-PA	AT2G15240.1	60518	BDEG_03269	CE36501
MSD1	superoxide dismutase [Mn], mitochondrial precursor	-	+	-	-	-	-	-	-	6356	-	-
		-	-	-	-	-	-	-	AT1G31190.1	-	-	-
TRS23	Component of TRAPP complex	-	+	+	-	-	-	GB13313-PA	AT5G02280.1	-	BDEG_06665	CE07190
CPX1	Coproporphyrinogen III oxidase, chloroplast precursor	-	+	-	-	-	-	GB20030-PA	AT1G03475.1	52628	BDEG_07460	-
STPK9	STPK9; Serine/Threonine Protein Kinase Homolog 9	-	-	-	-	-	-	-	-	-	-	-
		-	+	-	-	-	-	-	-	23601	BDEG_02334	-
ZEP	zeaxanthin epoxidase, chloroplast precursor	-	+	-	-	-	-	-	AT5G67030.2	71741	-	-
CHLRE_260063		-	+	-	-	-	-	GB13942-PA	AT1G79975.1	28188	BDEG_08110	CE33164
		-	+	+	-	-	-	GB19920-PA	AT3G18030.1	54984	BDEG_08118	CE09663
FAP178	Flagellar Associated Protein	-	-	-	+	-	-	-	-	-	-	-
KAT1		-	+	-	-	-	-	GB19861-PA	AT1G80350.1	21746	BDEG_01037	CE32479
OTA1	Ornithine transaminase (ornithine aminotransferase)	-	+	-	-	-	-	GB12326-PA	AT5G46180.1	27885	BDEG_07882	CE04010
FAP118	Flagellar Associated Protein	+	+	-	+	-	-	GB16728-PA	-	51986	BDEG_05074	CE37621
		-	-	-	-	-	-	-	-	-	-	-
		-	-	-	-	-	-	-	-	-	-	-
		-	-	-	-	-	-	-	-	-	-	-
		-	-	-	-	-	-	-	-	-	-	-
		-	-	-	-	-	-	-	-	-	-	-
		-	-	-	-	-	-	-	-	-	-	-
		-	+	-	-	-	-	-	AT2G34860.2	-	-	-
		-	-	-	-	-	-	-	-	-	-	-
		-	-	-	-	-	-	-	-	-	-	-
		-	-	-	-	-	-	-	-	-	-	-
		-	-	-	-	-	-	-	-	-	-	-
		-	-	-	-	-	-	-	-	-	-	-
		-	-	-	-	-	-	-	-	-	-	-
		-	-	-	-	-	-	-	-	-	-	-
		-	-	-	-	-	-	-	-	-	-	-
		-	-	-	-	-	-	-	-	-	-	-
CCS1	c-type cytochrome synthesis 1	-	+	-	-	-	-	-	AT1G49380.1	-	-	-
		-	-	-	-	-	-	-	-	-	-	-
		-	-	-	-	-	-	-	-	-	-	-
		-	+	-	-	-	-	-	-	-	-	-
FAP242	Coiled-Coil Flagellar Associated Protein	-	-	-	+	-	-	-	-	-	-	-
		-	-	-	-	-	-	-	-	-	-	-
		-	-	-	-	-	-	-	-	-	-	-
		-	-	-	-	-	-	-	-	-	-	-
		-	-	-	-	-	-	-	-	-	-	-
		-	-	-	-	-	-	-	-	-	-	-
		-	-	-	-	-	-	-	-	-	-	-
		-	-	-	-	-	-	-	-	-	-	-
		-	-	-	+	-	-	-	-	-	-	-
		-	-	-	-	-	-	-	-	-	-	-
		-	-	-	-	-	-	-	-	-	-	-
		-	-	-	-	-	-	-	-	-	-	-
		-	-	-	-	-	-	-	-	-	-	-
		-	-	-	-	-	-	-	-	-	-	-
		-	-	-	-	-	-	-	-	-	-	-
STPK4	Serine/Threonine Protein Kinase Homolog 4, putative	-	-	-	-	-	-	-	-	-	-	-
		-	-	-	-	-	-	-	-	-	-	-
ACH1	aconitate hydratase	-	-	-	-	-	-	-	-	-	-	-
		-	-	-	-	-	-	-	-	-	-	-
		-	-	-	-	-	-	-	-	-	-	-
		-	-	-	-	-	-	-	-	-	-	-
		-	-	-	-	-	-	-	-	-	-	-
		-	-	-	-	-	-	-	-	-	-	-
		-	-	-	-	-	-	-	-	-	-	-
		-	-	-	-	-	-	-	-	-	-	-
		-	-	-	-	-	-	-	-	-	-	-
		-	-	-	-	-	-	-	-	-	-	-
		-	-	-	-	-	-	-	-	-	-	-
		-	-	-	-	-	-	-	-	-	-	-
		-	-	-	-	-	-	-	-	-	-	-
		-	-	-	-	-	-	-	-	-	-	-
		-	-	-	-	-	-	-	-	-	-	-
		-	-	-	-	-	-	-	-	-	-	-
		-	-	-	-	-	-	-	-	-	-	-
	putative Thioredoxin-like/ATP binding protein 1	-	+	-	-	-	-	-	AT3G50960.1	15778	BDEG_01035	-
		-	+	-	-	-	-	-	AT1G80300.1	27252	-	-
		-	-	-	-	-	-	-	-	-	-	-

		-	-	-	-	-	-	-	-	-	-	-	-	195227	-	-	-	-	-	-	-	-	-	-	-	-	-	-	-	-	-	-	-	-	-	-	-	-	-	-	-	-	-	-	-	-	-	-	-	-	-	-
		-	-	-	-	-	-	-	AT1G53760.1	-	-	-	-	195223	-	-	-	-	-	-	-	-	-	-	-	-	-	-	-	-	-	Os02g05870.1	-	-	-	-	-	-	826515	-	-	-	-	-	-	-	-	-	-	-	-	-
		-	-	-	-	-	-	-	-	-	-	-	-	195221	-	-	-	-	-	-	-	-	-	-	-	-	-	-	-	-	-	-	-	-	-	-	-	-	-	-	-	-	-	-	-	-	-	-	-	-	-	-
		-	-	-	-	-	-	-	-	-	-	-	-	195219	-	-	-	-	-	-	-	-	-	-	-	-	-	-	-	-	-	-	-	-	-	-	-	-	-	-	-	-	-	-	-	-	-	-	-	-	-	-
		-	-	-	-	-	-	-	-	-	-	-	-	195218	-	-	-	-	-	-	-	-	-	-	-	-	-	-	-	-	-	-	-	-	-	-	-	-	-	-	-	-	-	-	-	-	-	-	-	-	-	-
MMP2	Matrix Metalloprotease 2	-	-	-	-	-	-	-	-	-	-	-	-	195201	-	-	-	-	-	-	-	-	-	-	-	-	-	-	-	-	-	-	-	-	-	-	-	-	-	-	-	-	-	-	-	-	-	-	-	-	-	-
SRRB1	Beta Subunit of the SRP Receptor	-	+	-	-	-	-	GB15601-PA	AT2G18770.1	-	BDEG_07056	CE12874	224352	195198	296648	-	-	-	DDB0205523	CG33162-PA	-	-	ENSGALP00000040343	-	OTTHUMP00000172877	LmjF33.2620	228741	33681	63910	79420	-	Os08g41250.2	35433	GSPATP00028715001	34810	143333	128377	PFL2245w	642947	-	YKL154W	SPAC23H4.07c	-	SINFRUP00000136450	203.m00037	-	-	57.m01682	57475	82140.m00115	-	UM05688.1
		-	-	-	-	-	-	-	-	-	-	-	-	195193	-	-	-	-	-	-	-	-	-	-	-	-	-	-	-	-	-	-	-	-	-	-	-	-	-	-	-	-	-	-	-	-	-	-	-	-	-	-
		-	-	-	-	-	-	-	-	-	-	-	-	195187	-	-	-	-	-	-	-	-	-	-	-	-	-	-	-	-	-	-	-	-	-	-	-	-	-	-	-	-	-	-	-	-	-	-	-	-	-	-
		-	-	-	-	-	-	-	-	-	-	-	-	195186	-	-	-	-	-	-	-	-	-	-	-	-	-	-	-	-	-	-	-	-	-	-	-	-	-	-	-	-	-	-	-	-	-	-	-	-	-	-
		-	-	-	-	-	-	-	-	-	-	-	-	195183	-	-	-	-	-	-	-	-	-	-	-	-	-	-	-	-	-	-	-	-	-	-	-	-	-	-	-	-	-	-	-	-	-	-	-	-	-	-
		-	+	-	-	-	-	GB16758-PA	AT5G06120.1	-	-	-	-	195180	-	-	-	-	-	-	-	-	ENSGALP00000040115	-	-	-	160865	-	34624	-	-	-	-	-	18067	218180	141370	-	797064	-	-	-	-	SINFRUP00000153885	-	34505	-	-	-	-	Tb927.8.4660	-
		-	-	-	-	-	-	-	-	-	-	-	-	195172	-	-	-	-	-	-	-	-	-	-	-	-	-	-	-	-	-	-	-	-	-	-	-	-	-	-	-	-	-	-	-	-	-	-	-	-	-	-
		-	-	-	-	-	-	-	-	-	-	-	-	195169	-	-	-	-	-	-	-	-	-	-	-	-	-	-	-	-	-	-	-	-	-	-	-	-	-	-	-	-	-	-	-	-	-	-	-	-	-	-
		-	-	-	-	-	-	-	-	-	-	-	-	195161	-	-	-	-	-	-	-	-	-	-	-	-	-	-	-	-	-	-	-	-	-	-	-	-	-	-	-	-	-	-	-	-	-	-	-	-	-	-
		-	-	-	-	-	-	-	-	-	-	-	-	195160	-	-	-	-	-	-	-	-	-	-	-	-	-	-	-	-	-	-	-	-	-	-	-	-	-	-	-	-	-	-	-	-	-	-	-	-	-	-
		-	-	-	-	-	-	-	-	-	-	-	-	195158	-	-	-	-	-	-	-	-	-	-	-	-	-	32361	-	-	-	-	-	-	-	-	-	-	-	-	-	-	-	-	-	-	-	-	-	-	-	-
		-	-	-	-	-	-	-	-	-	-	-	-	195156	-	-	-	-	-	-	-	-	-	-	-	-	-	-	-	-	-	-	-	-	-	-	-	-	-	-	-	-	-	-	-	-	-	-	-	-	-	-
		-	+	-	-	-	-	-	-	-	-	-	100241	195154	-	-	-	-	-	-	-	-	-	-	-	LmjF26.2560	-	25176	78553	-	-	-	-	-	-	162913	-	-	-	-	-	-	-	-	-	10363	-	-	-	-	Tb927.3.1860	-
		-	-	-	-	-	-	-	-	-	-	-	-	195153	-	-	-	-	-	-	-	-	-	-	-	-	-	-	-	-	-	-	-	-	-	-	-	-	-	-	-	-	-	-	-	-	-	-	-	-	-	-
		-	-	-	-	-	-	-	-	-	-	-	-	195151	-	-	-	-	-	-	-	-	-	-	-	-	-	-	-	-	-	-	-	-	-	-	-	-	-	-	-	-	-	-	-	-	-	-	-	-	-	-
		-	-	-	-	-	-	-	-	-	-	-	-	195147	-	-	-	-	-	-	-	-	-	-	-	-	-	-	-	-	-	-	-	-	-	181926	-	-	-	-	-	-	-	-	-	-	-	-	-	-	-	-
		-	-	-	-	-	-	-	-	-	-	-	-	195145	-	-	-	-	-	-	-	-	-	-	-	-	-	-	-	-	-	-	-	-	-	-	-	-	-	-	-	-	-	-	-	-	-	-	-	-	-	-
		-	-	-	-	-	-	-	-	-	-	-	-	195138	-	-	-	-	-	-	-	-	-	-	-	-	-	-	-	-	-	-	-	-	-	-	-	-	-	-	-	-	-	-	-	-	-	-	-	-	-	-
		-	-	-	-	-	-	-	-	-	-	-	-	195137	-	-	-	-	-	-	-	-	-	-	-	-	-	-	-	-	-	-	-	-	-	-	-	-	-	-	-	-	-	-	-	-	-	-	-	-	-	-
		-	-	-	-	-	-	-	-	-	-	-	-	195136	-	-	-	-	-	-	-	-	-	-	-	-	-	-	-	-	-	-	-	-	-	-	-	-	-	-	-	-	-	-	-	-	-	-	-	-	-	-
RPL22	Ribosomal protein L22, component of cytosolic 80S ribosome and 60S large subunit	-	+	-	-	-	-	GB10003-PA	AT5G27770.1	8613	BDEG_04311	CE04102	149706	195131	285284	cgd7_2420	CMS080C	OTTDARP00000023741	DDB0187786	CG7434-PA	-	-	ENSGALP00000001013	-	NP_001093115.1	LmjF36.3270	178960	34459	5310	124889	NCU06661	Os07g47710.1	20387	GSPATP00021914001	39687	192476	127179	PF08_0039	240547	RO3G_03169.1	YFL034C-A	SPAC11E3.15	GLEAN3_02464	SINFRUP00000135372	19.m00329	6071	TA08205	49.m05661	18459	-	Tb10.6k15.0510	UM00686.1
		-	-	-	-	-	-	-	-	-	-	-	-	195123	-	-	-	-	-	-	-	-	-	-	-	-	-	-	-	-	-	-	-	-	-	-	-	-	-	-	-	-	-	-	-	-	-	-	-	-	-	-
		-	-	-	-	-	-	-	-	-	-	-	-	195121	-	-	-	-	-	-	-	-	-	-	-	-	-	-	-	-	-	-	-	-	-	-	-	-	-	-	-	-	-	-	-	-	-	-	-	-	-	-
		-	-	-	-	-	-	-	-	-	-	-	-	195119	-	-	-	-	-	-	-	-	-	-	-	-	-	-	-	-	-	-	-	-	-	-	-	-	-	-	-	-	-	-	-	-	-	-	-	-	-	-
		-	-	-	-	-	-	-	-	-	-	-	-	195117	-	-	-	-	-	-	-	-	-	-	-	-	-	-	-	-	-	-	-	-	-	-	-	-	-	-	-	-	-	-	-	-	-	-	-	-	-	-
		-	+	-	-	-	-	GB16441-PA	AT5G56130.1	4392	BDEG_07616	CE28927	180662	195115	297636	-	-	OTTDARP00000021701	DDB0169460	CG9615-PA	-	-	ENSGALP00000040051	-	OTTHUMP00000161240	-	229722	23390	-	172456	-	Os09g12710.1	16467	-	-	225435	129834	-	815913	RO3G_05440.1	-	SPCC18B5.10c	-	SINFRUP00000128280	54.m00193	-	TA10560	-	20906	-	-	UM02777.1
		-	-	-	-	-	-	-	-	-	-	-	-	195113	-	-	-	-	-	-	-	-	-	GL50803_16234	-	-	-	-	-	-	-	-	-	-	-	-	-	-	-	-	-	-	-	-	-	-	-	-	-	-	-	-
		-	+	-	-	-	-	-	AT5G49820.1	-	-	-	-	195109	-	-	CMI124C	-	-	-	-	-	-	-	-	-	-	-	-	-	-	Os05g34650.2	-	-	-	211695	-	-	297308	-	-	-	-	-	-	-	-	-	-	-	-	-
NMA2	Chlamydomonas NIMA-related protein kinase homolog 2	-	-	-	+	-	-	-	-	25668	-	-	-	195107	-	-	-	-	-	-	-	-	-	-	-	-	-	-	-	-	-	-	-	GSPATP00025833001	-	-	-	-	-	-	-	SPAC19E9.02	-	-	115.m00104	-	-	-	-	-	-	-
		-	-	-	-	-	-	-	-	64602	-	-	-	195095	-	-	-	-	-	-	-	-	-	GL50803_17543	-	-	-	-	-	-	-	-	-	-	47398	-	-	-	-	RO3G_15521.1	-	-	-	-	-	-	-	-	-	-	-	-
		-	-	-	-	-	-	-	-	-	-	-	-	195092	-	-	-	-	-	-	-	-	-	-	-	-	-	-	-	-	-	-	-	-	-	-	-	-	-	-	-	-	-	-	-	-	-	-	-	-	-	-
		-	-	-	-	-	-	-	-	-	-	-	-	195087	-	-	-	-	-	-	-	-	-	-	-	-	-	-	-	-	-	-	-	-	-	-	-	-	-	-	-	-	-	-	-	-	-	-	-	-	-	-
		-	-	-	-	-	-	-	-	-	-	-	-	195078	-	-	-	-	-	-	-	-	-	-	-	-	-	-	-	-	-	-	-	-	-	-	-	-	-	-	-	-	-	-	-	-	-	-	-	-	-	-
		+	+	-	-	-	-	GB12848-PA	-	32092	BDEG_07390	-	149938	195076	290296	-	-	OTTDARP00000013761	-	-	-	-	ENSGALP00000004727	-	OTTHUMP00000164246	LmjF10.0780	236399	-	60593	237381	-	-	-	GSPATP00033053001	-	-	118611	-	-	-	-	-	-	-	126.m00082	-	-	-	49686	-	Tb927.8.4210	-
		-	-	-	-	-	-	-	-	-	-	-	-	195070	-	-	-	-	-	-	-	-	-	-	-	-	-	-	-	-	-	-	-	-	-	-	-	-	-	-	-	-	-	-	-	-	-	-	-	-	-	-
		-	+	-	-	-	-	-	AT3G09090.1	-	-	-	206740	195069	-	-	-	-	DDB0206287	-	-	-	-	-	-	LmjF31.0110	159926	-	36616	161101	-	Os03g61050.1	12213	-	-	172150	-	-	735721	-	-	-	-	-	-	-	-	-	57158	-	Tb927.7.6460	-
		-	-	-	-	-	-	-	-	-	-	-	-	195067	-	-	-	-	-	-	-	-	-	-	-	LmjF09.1440	-	-	-	-	-	-	-	-	-	210777	-	-	-	-	-	-	-	-	-	-	-	-	-	-	-	-
		-	+	-	-	-	-	-	AT4G03115.1	22191	-	-	-	195065	-	-	CMH173C	-	-	-	-	-	-	-	-	LmjF30.1050	-	-	30741	-	-	-	-	GSPATP00020782001	-	-	108141	-	-	-	-	-	-	-	-	-	-	-	-	-	-	-
		-	+	-	-	-	-	GB13017-PA	AT3G18430.1	-	BDEG_03402	CE06665	227558	195064	-	-	CMJ269C	OTTDARP00000019507	DDB0218775	-	19173343	-	-	GL50803_16829	-	-	-	-	-	-	-	Os02g55880.1	7037	-	-	221299	-	-	835941	-	-	-	-	-	-	-	-	-	-	-	Tb10.70.7970	-
		-	-	-	-	-	-	-	-	-	-	-	-	195063	-	-	-	-	-	-	-	-	-	-	-	-	-	-	-	-	-	-	-	-	-	-	-	-	-	-	-	-	-	-	-	-	-	-	-	-	-	-
		-	+	-	+	-	-	GB18444-PA	AT2G26800.2	-	BDEG_01239	CE26261	172450	195057	219510	-	CMA068C	OTTDARP00000024050	DDB0185699	CG10399-PA	-	-	ENSGALP00000006445	-	NP_000182.2	-	222853	33622	80885	89324	NCU05419	Os11g04210.1	-	GSPATP00029666001	13643	58979	109053	-	833174	RO3G_01094.1	-	-	-	SINFRUP00000136985	104.m00156	-	-	20.m03867	61193	-	Tb927.4.2700	UM02001.1
		-	-	-	-	-	-	-	-	-	-	-	-	195055	-	-	-	-	-	-	-	-	-	-	-	-	-	-	-	-	-	-	-	-	-	-	-	-	-	-	-	-	-	-	-	-	-	-	-	-	-	-
FAP182	Flagellar Associated Protein	-	-	-	+	-	-	-	-	-	BDEG_01440	-	-	195050	282566	-	-	-	-	-	-	-	-	-	-	-	-	-	-	-	-	-	-	GSPATP00012354001	-	-	-	-	-	-	-	-	-	-	-	-	-	-	-	-	-	-
GGPS	geranylgeranyl diphosphate synthase, putative chloroplast precursor	-	+	-	-	-	-	-	AT4G36810.1	52464	-	-	-	195038	-	-	CMK058C	-	-	-	-	-	-	-	-	-	-	-	-	-	-	Os07g39270.2	26626	-	19000	88295	-	-	198398	-	-	-	-	-	-	268480	-	-	-	-	-	-
		-	-	-	-	-	-	-	-	-	-	-	-	195021	-	-	-	-	-	-	-	-	-	-	-	-	-	-	-	-	-	-	-	-	-	-	-	-	-	-	-	-	-	-	-	-	-	-	-	-	-	-
CAG1	NADH:ubiquinone oxidoreductase (Complex I) subunit related to carbonic anhydrase (gamma-type) protein, mitochondrial	-	+	-	-	-	-	-	-	-	-	-	-	195016	-	-	-	-	DDB0187805	CG10846-PA	-	-	-	-	-	LmjF08.0180	-	-	-	-	NCU07196	-	-	GSPATP00000747001	36906	-	-	PF07_0098	-	-	-	-	GLEAN3_28205	-	71.m00119	-	-	-	-	84270.m00524	Tb927.5.3700	-
		-	-	-	-	-	-	-	-	-	-	-	-	195012	-	-	-	-	-	-	-	-	-	-	-	-	-	-	-	-	-	-	-	-	-	-	-	-	-	-	-	-	-	-	-	-	-	-	-	-	-	-
		-	-	-	-	-	-	-	-	-	-	-	-	195007	-	-	-	-	-	-	-	-	-	-	-	-	-	-	-	-	-	-	37165	-	-	-	-	-	731296	-	-	-	-	-	-	-	-	-	-	-	-	-
		-	-	-	-	-	-	-	-	-	-	-	-	195003	-	-	-	-	-	-	-	-	-	-	-	-	-	-	-	-	-	-	-	-	-	-	-	-	-	-	-	-	-	-	-	36858	-	-	-	-	-	-
		-	-	-	-	-	-	-	-	-	-	-	-	194998	-	-	-	-	-	-	-	-	-	-	-	-	-	-	-	-	-	-	-	-	-	-	-	-	-	-	-	-	-	-	-	-	-	-	-	-	-	-
		-	-	-	-	-	-	-	-	-	-	-	-	194996	-	-	-	-	-	-	-	-	-	-	-	-	-	-	-	-	-	-	-	-	-	-	-	-	-	-	-	-	-	-	-	-	-	-	-	-	-	-
		-	-	-	-	-	-	-	-	-	-	-	-	194994	-	-	-	-	-	-	-	-	-	-	XP_001132039.1	-	-	-	-	-	-	-	-	-	-	-	-	-	-	-	-	-	-	-	-	-	-	-	-	-	-	-
		-	-	-	-	-	-	-	-	-	-	-	-	194989	-	-	-	-	-	-	-	-	-	-	-	-	-	-	-	-	-	-	-	-	-	-	-	-	-	-	-	-	-	-	-	-	-	-	-	-	-	-
		-	+	-	-	-	-	-	AT3G01800.1	-	-	-	226887	194988	-	-	CMT419C	OTTDARP00000020030	-	-	-	-	ENSGALP00000002036	-	-	-	-	-	4827	103700	-	-	-	-	-	-	140069	-	-	RO3G_04854.1	-	-	-	-	-	-	-	-	55709	-	-	-
APG12	Autophagy protein	-	+	-	-	-	-	-	AT3G13970.1	9220	BDEG_05795	CE00776	182898	194981	246915	-	-	-	DDB0185323	CG10861-PA	-	-	ENSGALP00000003688	-	OTTHUMP00000159129	-	106494	-	73824	179043	NCU10049	Os06g10340.1	-	-	-	187242	-	-	180753	-	YBR217W	SPAC1783.06c	-	-	-	34701	-	-	53755	-	-	UM03577.1
		-	-	-	-	-	-	-	-	-	-	-	-	194979	-	-	-	-	-	-	-	-	-	-	-	-	-	-	-	-	-	-	-	-	-	-	-	-	-	-	-	-	-	-	-	-	-	-	-	-	-	-
	sarcaleumin-like	-	+	-	-	-	-	-	-	-	-	-	164867	194978	252293	cgd8_3930	-	OTTDARP00000017655	-	-	-	-	-	-	-	-	231476	27581	-	171246	-	-	32446	-	-	-	-	MAL8P1.53	-	-	-	-	-	SINFRUP00000168508	-	-	-	-	18756	90976.m00061	-	-
MRPS6	Putative mitochondrial ribosomal protein S6, imported to mitochondria	-	+	-	-	-	-	-	AT3G18760.1	-	-	-	-	194972	-	-	-	-	-	-	-	-	-	-	OTTHUMP00000068540	-	-	-	45652	-	-	Os01g12090.1	-	-	-	123995	-	-	262842	RO3G_07224.1	-	SPAC343.08c	-	-	-	-	-	-	-	-	-	-
		-	-	-	-	-	-	-	-	-	-	-	-	194968	-	-	-	-	-	-	-	-	-	-	-	-	-	-	-	-	-	-	-	-	-	-	-	-	-	-	-	-	-	-	-	-	-	-	-	-	-	-
		-	+	+	-	-	-	GB16267-PA	-	21140	BDEG_06150	CE06654	19396	194967	298945	cgd7_830	CMN182C	OTTDARP00000022913	DDB0184073	CG4947-PA	19074281	17.m00348	ENSGALP00000024354	GL50803_95064	OTTHUMP00000078152	LmjF30.1800	205064	4956	60345	194779	NCU07015	Os09g29420.1	9442	GSPATP00020710001	17628	195351	108513	PF07_0071	411694	RO3G_13881.1	-	SPAC1687.19c	GLEAN3_05795	SINFRUP00000150601	61.m00234	31256	TA16175	583.m05724	20956	96800.m00201	Tb927.6.3130	-
		-	-	-	-	-	-	-	-	-	-	-	-	194965	-	-	-	-	-	-	-	-	-	-	-	-	-	-	-	-	-	-	-	-	-	-	-	-	-	-	-	-	-	-	-	-	-	-	-	-	-	-
	Hypothetical protein	-	-	-	-	-	-	-	-	-	-	CE38280	-	194959	-	-	-	-	-	-	-	-	-	-	-	-	-	-	-	240009	-	-	-	-	-	-	-	-	-	-	-	-	-	-	-	-	-	-	-	-	-	-
		-	-	-	-	-	-	-	-	-	-	-	-	194958	-	-	-	-	-	-	-	-	-	-	-	-	-	-	-	-	-	-	-	-	-	-	-	-	-	-	-	-	-	-	-	-	-	-	-	-	-	-
		-	-	-	-	-	-	-	-	-	-	-	-	194957	-	-	-	-	-	-	-	-	-	-	-	-	-	-	-	-	-	-	-	-	-	-	-	-	-	-	-	-	-	-	-	-	-	-	-	-	-	-
		-	-	-	-	-	-	-	-	-	-	-	-	194954	-	-	-	-	-	-	-	-	-	-	-	-	-	-	-	-	-	-	-	-	-	-	-	-	-	-	-	-	-	-	-	-	-	-	-	-	-	-
		-	-	-	-	-	-	-	-	-	-	-	-	194952	-	-	-	-	-	-	-	-	-	-	-	-	-	-	-	-	-	-	-	-	-	-	-	-	-	-	-	-	-	-	-	-	-	-	-	-	-	-
FAK1	Flagellar Adenylate Kinase Homolog1	-	-	-	-	-	-	-	-	-	-	-	-	194947	-	-	-	-	DDB0187669	-	-	-	-	-	-	-	-	32330	72729	-	-	-	-	-	-	-	-	-	-	RO3G_15348.1	-	-	-	-	-	-	-	-	-	-	-	-
PTP1	Protein Tyrosine Phosphatase Homolog 1	+	+	-	-	-	-	GB12206-PA	-	-	-	CE21176	229133	194946	272741	-	-	OTTDARP00000017772	-	-	-	-	ENSGALP00000008223	-	OTTHUMP00000021688	LmjF32.0640	174628	29751	67231	129991	-	-	-	GSPATP00028517001	-	-	136790	-	-	-	-	-	GLEAN3_12073	SINFRUP00000133409	190.m00076	-	-	-	56940	-	Tb11.01.5450	-
		-	-	-	-	-	-	-	-	-	-	-	-	194943	-	-	-	-	-	-	-	-	-	-	-	-	-	-	-	-	-	-	-	-	-	-	-	-	-	-	-	-	-	-	-	-	-	-	-	-	-	-
		-	-	-	-	-	-	-	-	-	-	-	-	194940	-	-	-	-	-	-	-	-	-	-	-	-	-	-	-	-	-	-	-	-	-	-	-	-	-	-	-	-	-	-	-	-	-	-	-	-	-	-
GRX3	Glutaredoxin 3, CGFS type, probably chloroplastic	-	+	-	-	-	-	GB18700-PA	AT3G54900.1	28514	BDEG_07534	CE22220	163758	194929	377514	-	CMM250C	OTTDARP00000004121	-	CG14407-PA	-	-	ENSGALP00000018029	-	NP_057501.2	LmjF01.0110	201172	22253	29135	131070	-	Os03g63420.1	6857	GSPATP00028587001	9035	153882	-	PFF0340c	246206	RO3G_14195.1	-	-	-	SINFRUP00000155050	-	37934	-	59.m06121	35126	-	-	UM02867.1
RPL38	Ribosomal protein L38, component of cytosolic 80S ribosome and 60S large subunit	-	+	+	-	-	-	GB14397-PA	AT3G59540.1	21514	BDEG_06511	CE20485	166318	194928	-	cgd7_4050	CMJ170C	OTTDARP00000022895	DDB0202100	CG18001-PA	-	484.m00041	ENSGALP00000002218	-	OTTHUMP00000182264	LmjF26.2220	211882	36190	66493	226298	NCU03635	Os11g24610.2	35617	GSPATP00039615001	19413	211044	117827	-	562207	RO3G_08326.1	YLR325C	SPBC577.02	-	SINFRUP00000181392	138.m00104	26889	TA21360	44.m04616	63143	-	Tb10.70.4155	UM03022.1
FAP258	Flagellar Associated Protein	-	-	-	-	-	-	-	-	-	-	-	-	194927	-	-	-	-	-	-	-	-	-	-	-	-	-	-	-	-	-	-	-	-	-	-	-	-	-	-	-	-	-	-	-	-	-	-	-	-	-	-
		-	-	-	-	-	-	-	-	-	-	-	-	194924	-	-	-	-	-	-	-	-	-	-	-	-	-	-	-	-	-	-	-	-	-	-	-	-	-	-	-	-	-	-	-	-	-	-	-	-	-	-
		-	-	-	-	-	-	-	-	-	-	-	-	194921	-	-	-	-	-	-	-	-	-	-	-	-	-	-	-	-	-	-	-	-	-	-	-	-	592304	-	-	-	-	-	-	-	-	-	-	-	-	-
CIS1	citrate synthase, putative mitochondrial isoform	-	+	-	+	-	-	GB12573-PA	AT2G44350.2	37826	BDEG_05745	CE00513	148793	194915	298739	-	CMQ191C	OTTDARP00000022132	DDB0217639	CG3861-PA	-	-	-	-	OTTHUMP00000082833	LmjF18.0680	170380	-	38914	82156	NCU01692	Os11g33240.2	28307	GSPATP00003586001	30145	179651	109571	PF10_0218	837325	RO3G_06689.1	YNR001C	SPAC6C3.04	GLEAN3_24102	SINFRUP00000127671	31.m00360	11411	TA14450	59.m03414	54073	-	Tb10.05.0150	UM01627.1
		-	-	-	-	-	-	-	-	-	-	-	-	194907	-	-	-	-	-	-	-	-	-	-	-	-	-	-	-	-	-	-	-	-	-	-	-	-	-	-	-	-	-	-	-	-	-	-	-	-	-	-
		-	-	-	-	-	-	-	-	-	-	-	-	194901	-	-	-	-	-	-	-	-	-	-	-	-	-	-	-	-	-	-	-	-	-	-	-	-	-	-	-	-	-	-	-	-	-	-	-	-	-	-
		-	+	-	-	-	-	-	AT2G32970.1	-	-	-	-	194900	-	-	-	-	-	-	-	-	-	-	-	LmjF15.0530	-	-	68620	-	-	Os05g51860.1	-	-	45187	165546	-	-	732312	-	-	-	-	-	-	-	-	-	-	-	Tb927.5.1890	-
		-	-	-	-	-	-	-	-	-	-	-	-	194894	-	-	-	-	-	-	-	-	-	-	-	-	-	-	-	-	-	-	-	-	-	-	-	-	-	-	-	-	-	-	-	-	-	-	-	-	-	-
		-	-	-	-	-	-	-	AT4G18372.1	-	-	-	-	194889	-	-	-	-	-	-	-	-	-	-	NP_115732.2	-	-	-	-	-	-	Os06g46390.1	-	-	-	-	-	-	575765	-	-	-	-	-	-	-	-	-	-	-	-	-
		-	-	-	-	-	-	-	-	-	-	-	-	194885	-	-	-	-	-	-	-	-	-	-	-	-	-	-	-	-	-	-	-	-	-	-	-	-	-	-	-	-	-	-	-	-	-	-	-	-	-	-
		-	-	-	-	-	-	-	-	-	-	-	-	194883	-	-	-	-	-	-	-	-	-	-	-	-	-	-	-	-	-	-	-	-	-	-	-	-	-	-	-	-	-	-	-	-	-	-	-	-	-	-
		-	-	-	-	-	-	-	AT3G55250.1	-	-	-	-	194882	-	-	-	-	-	-	-	-	-	-	-	-	-	-	-	-	-	Os05g45030.1	-	-	-	46771	-	-	835677	-	-	-	-	-	-	-	-	-	-	-	-	-
TIM22B	translocase of inner mitochondrial membrane 22 homolog	-	+	-	-	-	-	-	AT3G49560.1	-	-	-	-	194876	-	-	-	-	-	-	-	-	-	-	-	-	-	-	-	-	-	Os04g33220.1	23199	-	-	165454	-	-	835039	-	-	-	-	-	-	-	-	-	-	-	-	-
		-	+	-	-	-	-	GB20068-PA	-	-	-	CE36983	157263	194858	293499	-	CMP208C	-	DDB0188545	CG10721-PA	-	-	ENSGALP00000021481	-	OTTHUMP00000166881	-	178551	-	-	81327	-	-	37251	-	-	-	-	-	-	-	-	-	GLEAN3_07676	SINFRUP00000160318	-	-	-	-	22271	-	-	-
MTDH/MTCH1	Putative 5,10-methylenetetrahydrofolate dehydrogenase/5,10-methenyltetrahydrofolate cyclohydrolase	-	+	-	-	-	-	-	AT3G12290.1	29188	-	CE25047	-	194856	-	-	CMR167C	OTTDARP00000005095	DDB0185371	-	-	-	-	-	-	LmjF26.0320	-	-	55789	-	-	Os02g02850.1	11650	GSPATP00017156001	23623	129285	109451	-	409791	-	-	-	GLEAN3_10209	-	34.m00360	34399	-	55.m04709	50697	-	Tb927.7.1600	-
		-	-	-	-	-	-	-	-	-	-	-	-	194851	-	-	-	-	-	-	-	-	-	-	-	-	-	-	-	-	-	-	-	-	-	-	-	-	-	-	-	-	-	-	-	-	-	-	-	-	-	-
		-	-	-	-	-	-	-	-	-	-	-	-	194845	-	-	-	-	-	-	-	-	-	-	-	-	-	-	-	-	-	-	-	-	-	-	-	-	-	-	-	-	-	-	-	-	-	-	-	-	-	-
		-	-	-	-	-	-	-	-	-	-	-	-	194844	-	-	-	-	-	-	-	-	-	-	-	-	-	-	-	-	NCU06992	Os11g03590.1	33896	-	-	-	-	-	782488	-	-	-	-	-	-	-	-	-	-	-	-	-
FLA14	Outer dynein arm light chain 8, 8 kD	-	+	+	+	-	-	GB15711-PA	AT4G15930.1	34279	BDEG_02809	CE00788	371	194837	207904	cgd2_740	CMT356C	OTTDARP00000021888	DDB0217490	CG6998-PC	-	6.m00505	ENSGALP00000008845	GL50803_9848	OTTHUMP00000181715	LmjF32.0230	206820	35100	44199	236679	-	Os01g15260.1	30069	GSPATP00024306001	16008	151365	156491	PFL0660w	296043	RO3G_00997.1	YDR424C	SPAC926.07c	GLEAN3_18607	SINFRUP00000178763	94.m00175	35985	TA19540	41.m01383	19810	94782.m00201	Tb11.0845	UM04651.1
		-	-	-	-	-	-	-	-	-	-	-	-	194836	-	-	-	-	-	-	-	-	-	-	-	-	-	-	-	-	-	-	-	-	-	-	142555	-	-	-	-	-	-	-	-	-	-	-	-	-	-	-
		-	-	-	-	-	-	-	-	-	-	-	-	194835	-	-	-	-	-	-	-	-	-	-	-	-	-	-	-	-	-	-	-	-	-	-	-	-	-	-	-	-	-	-	-	-	-	-	-	-	-	-
		-	-	-	-	-	-	-	-	-	-	-	-	194832	-	-	-	-	-	-	-	-	-	-	-	-	-	-	-	-	-	-	-	-	-	-	-	-	-	-	-	-	-	-	-	-	-	-	-	-	-	-
C_66085		-	+	-	-	-	-	GB18578-PB	AT1G49520.1	64768	BDEG_05989	CE37993	227075	194816	-	-	CMM087C	-	-	CG1240-PA	-	-	-	-	-	-	235351	7547	-	241781	NCU02204	Os03g55570.1	-	-	32255	187352	142019	-	201590	RO3G_08576.1	YMR233W	SPCC285.17	-	-	-	3693	-	59.m00062	-	-	-	-
		-	-	-	-	-	-	-	-	-	-	-	-	194814	-	-	-	-	-	-	-	-	-	-	-	-	-	-	-	-	-	-	-	-	-	-	-	-	-	-	-	-	-	-	-	-	-	-	-	-	-	-
		-	-	-	-	-	-	-	-	-	-	-	-	194813	-	-	-	-	-	-	-	-	-	-	-	-	-	-	-	-	-	-	-	-	-	-	-	-	-	-	-	-	-	-	-	-	-	-	-	-	-	-
		-	-	-	-	-	-	-	-	-	-	-	-	194804	-	-	-	-	-	-	-	-	-	-	-	-	-	-	-	-	-	-	-	-	-	-	-	-	-	-	-	-	-	-	-	-	-	-	-	-	-	-
		-	-	-	-	-	-	-	AT3G05320.1	72029	-	-	-	194802	-	-	-	-	-	-	-	-	-	-	-	-	-	-	-	-	-	-	-	-	-	-	-	-	-	-	-	-	-	-	-	-	-	-	-	-	-	-
		-	-	-	-	-	-	-	-	-	-	-	-	194797	-	-	-	-	-	-	-	-	-	-	-	-	-	-	-	-	-	-	-	-	-	-	-	-	-	-	-	-	-	-	-	-	-	-	-	-	-	-
STT7	protein kinase involved in state transitions	-	+	-	-	-	-	-	AT1G68830.1	-	-	-	-	194793	-	-	CMS146C	-	-	-	-	-	-	-	-	-	-	-	-	-	-	Os05g47560.1	19625	-	-	191307	156248	-	832716	-	-	-	-	-	-	24596	-	-	-	-	-	-
		-	-	-	-	-	-	-	-	-	-	-	-	194780	-	-	-	-	-	-	-	-	-	-	-	-	-	-	-	-	-	-	-	-	-	-	-	-	-	-	-	-	-	-	-	-	-	-	-	-	-	-
		-	-	-	-	-	-	-	-	-	-	-	-	194779	-	-	-	-	-	-	-	-	-	-	-	-	-	-	-	-	-	-	-	-	-	-	-	-	-	-	-	-	-	-	-	-	-	-	-	-	-	-
		-	-	-	-	-	-	-	-	-	-	-	-	194776	-	-	-	-	-	-	-	-	-	-	-	-	-	-	-	-	-	-	-	-	-	-	-	-	-	-	-	-	-	-	-	-	-	-	-	-	-	-
		-	-	-	-	-	-	-	-	-	-	-	-	194775	-	-	-	-	-	-	-	-	-	-	-	-	-	-	-	-	-	-	-	-	-	-	-	-	-	-	-	-	-	-	-	-	-	-	-	-	-	-
		-	-	-	-	-	-	-	-	-	-	-	-	194774	-	-	-	-	-	-	-	-	-	-	-	-	-	-	-	242587	-	-	-	-	-	179421	139841	-	-	-	-	-	-	-	-	-	-	-	-	-	-	-
		-	+	-	-	-	-	-	-	-	-	-	-	194772	-	-	CMM139C	-	DDB0168738	-	-	-	-	GL50803_112103	-	-	-	-	47456	-	-	-	-	-	-	-	-	-	-	-	-	-	-	-	-	-	-	-	-	92185.m00054	-	-
LIPA1	lipoic acid synthetase	-	+	-	-	-	-	GB16729-PA	AT2G20860.1	29425	BDEG_06906	CE01031	-	194766	-	-	CML179C	-	-	-	-	-	-	-	-	LmjF19.0350	-	-	-	-	NCU00565	-	18101	-	-	184135	109562	-	558638	RO3G_12358.1	-	-	-	-	-	-	-	-	-	-	Tb10.61.1530	UM04653.1
		-	-	-	-	-	-	-	-	-	-	-	-	194765	-	-	-	-	-	-	-	-	-	-	-	-	-	-	-	-	-	-	-	-	-	-	-	-	-	-	-	-	-	-	-	-	-	-	-	-	-	-
		-	-	-	-	-	-	-	-	-	-	-	-	194764	-	-	-	-	-	-	-	-	-	-	-	-	-	24517	-	-	-	-	-	-	-	-	-	-	-	-	-	-	-	-	-	-	-	-	-	-	-	-
		-	-	-	-	-	-	-	-	-	-	-	-	194760	-	-	-	-	-	-	-	-	-	-	-	-	-	-	-	-	-	-	-	-	-	-	-	-	-	-	-	-	-	-	-	-	-	-	-	-	-	-
	hypothetical protein	-	-	-	-	-	-	-	-	-	-	-	-	194756	-	-	-	-	-	-	-	-	-	-	-	-	-	-	-	-	-	-	-	-	-	-	-	-	-	-	-	-	-	-	-	-	-	-	-	-	-	-
	Expressed protein of unknown function	-	-	-	-	-	-	-	-	-	-	-	-	194753	-	-	-	-	-	-	-	-	-	-	-	-	-	-	-	-	-	-	-	-	-	-	-	-	-	-	-	-	-	-	-	-	-	-	-	-	-	-
		-	-	-	-	-	-	-	-	-	-	-	-	194750	-	-	-	-	-	-	-	-	-	-	-	-	-	-	-	-	-	-	-	-	-	-	-	-	-	-	-	-	-	-	-	-	-	-	-	-	-	-
		-	-	-	-	-	-	-	-	-	-	-	-	194746	-	-	-	-	-	-	-	-	-	-	-	-	-	-	-	-	-	-	-	-	-	-	-	-	-	-	-	-	-	-	-	-	-	-	-	-	-	-
		-	-	-	-	-	-	-	-	-	-	-	-	194745	-	-	-	-	-	-	-	-	-	-	-	-	-	-	-	-	-	-	-	-	-	-	-	-	-	-	-	-	-	-	-	-	-	-	-	-	-	-
		-	-	-	-	-	-	-	-	-	-	-	-	194744	-	-	-	-	-	-	-	-	-	-	-	-	-	-	-	-	-	-	-	-	-	-	-	-	-	-	-	-	-	-	-	-	-	-	-	-	-	-
		-	-	-	-	-	-	-	-	-	-	-	-	194743	-	-	-	-	-	-	-	-	-	-	-	-	-	-	-	-	-	-	-	-	-	-	-	-	-	-	-	-	-	-	-	-	-	-	-	-	-	-
		-	-	-	-	-	-	-	-	-	-	-	-	194741	-	-	-	-	-	-	-	-	-	-	-	-	-	-	-	-	-	-	-	-	-	-	-	-	-	-	-	-	-	-	-	-	-	-	-	-	-	-
		-	-	-	-	-	-	-	-	-	-	-	-	194736	-	-	-	-	-	-	-	-	-	-	-	-	-	-	-	-	-	-	-	-	-	-	-	-	-	-	-	-	-	-	-	-	-	-	-	-	-	-
FAP162	Flagellar Associated Protein	-	-	-	+	-	-	-	-	-	-	-	-	194731	-	-	-	-	-	-	-	-	-	-	-	-	-	30745	-	-	-	-	33750	-	-	-	-	-	-	-	-	-	-	-	-	-	-	-	-	-	-	-
		-	-	-	-	-	-	-	-	-	-	-	-	194728	-	-	-	-	-	-	-	-	-	-	-	-	-	-	-	-	-	-	-	-	-	-	-	-	-	-	-	-	-	-	-	-	-	-	-	-	-	-
		-	-	-	-	-	-	-	-	-	-	-	-	194727	-	-	-	-	-	-	-	-	-	-	-	-	-	-	-	-	-	-	-	-	-	-	-	-	-	-	-	-	-	-	-	-	-	-	-	-	-	-
		-	-	-	-	-	-	-	-	-	-	-	-	194725	-	-	-	-	-	-	-	-	-	-	-	-	-	-	-	-	-	-	-	-	-	-	-	-	-	-	-	-	-	-	-	-	-	-	-	-	-	-
		-	-	-	-	-	-	-	-	-	-	-	-	194724	-	-	-	-	-	-	-	-	-	-	-	-	-	-	-	-	-	-	-	-	-	-	-	-	-	-	-	-	-	-	-	-	-	-	-	-	-	-
		-	-	-	-	-	-	-	-	-	-	-	-	194715	-	-	-	-	-	-	-	-	-	-	-	-	-	-	-	-	-	-	-	-	-	-	-	-	-	-	-	-	-	-	-	-	-	-	-	-	-	-
		-	+	-	-	-	-	GB12219-PA	AT3G61140.1	-	BDEG_08399	CE26200	174845	194712	205303	-	CMC138C	OTTDARP00000019650	DDB0215686	CG3889-PA	-	35.m00214	ENSGALP00000004453	-	NP_997657.1	-	184282	-	82742	189928	NCU00157	Os03g02540.1	16687	-	54772	178664	112609	-	710292	RO3G_03443.1	-	SPBC215.03c	-	SINFRUP00000152152	-	5185	-	-	53707	-	Tb10.70.5170	UM04548.1
		-	-	-	-	-	-	-	-	-	-	-	-	194704	-	-	-	-	-	-	-	-	-	-	-	-	-	-	-	-	-	-	-	-	-	-	-	-	-	-	-	-	-	-	-	-	-	-	-	-	-	-
		-	-	-	-	-	-	-	-	-	-	-	-	194701	-	-	-	-	-	-	-	-	-	-	-	-	-	-	-	-	-	-	-	-	-	-	-	-	-	-	-	-	-	-	-	-	-	-	-	-	-	-
LCI22	low-CO2-inducible protein	-	-	-	-	-	-	-	-	-	-	-	-	194693	-	-	-	-	-	-	-	-	-	-	-	-	-	-	-	-	-	-	-	-	-	-	-	-	-	-	-	-	-	-	-	-	-	-	-	-	-	-
FAP149	Flagellar Associated Protein	-	-	-	+	-	-	-	-	-	-	-	-	194692	-	-	-	-	-	-	-	-	-	-	-	-	-	-	-	-	-	-	-	-	-	-	-	-	-	-	-	-	-	-	-	-	-	-	-	-	-	-
		-	-	-	-	-	-	-	-	-	-	-	-	194689	-	-	-	-	-	-	-	-	-	-	-	-	-	-	-	-	-	-	-	-	-	-	-	-	-	-	-	-	-	-	-	-	-	-	-	-	-	-
	Thioredoxin-like protein, with unusual active site HCKPC	-	-	-	-	-	-	-	-	-	-	-	-	194687	-	-	CMN279C	-	-	-	-	-	-	-	-	-	-	-	-	-	-	-	-	-	-	150335	-	-	-	-	-	-	-	-	-	-	-	-	-	-	-	-
		-	-	-	-	-	-	-	-	-	-	-	-	194684	-	-	-	-	-	-	-	-	-	-	-	-	-	-	-	-	-	-	-	-	-	-	-	-	-	-	-	-	-	-	-	-	-	-	-	-	-	-
FAP14	Conserved Uncharacterized Flagellar Associated Protein	+	+	-	-	-	-	GB11078-PA	-	-	BDEG_04543	CE32892	195026	194683	270675	-	-	OTTDARP00000006719	-	-	-	-	ENSGALP00000017802	-	OTTHUMP00000028411	LmjF24.0190	-	-	50227	118006	-	-	31157	GSPATP00024888001	-	71070	-	-	-	-	-	-	-	SINFRUP00000146292	19.m00231	-	-	-	51742	-	Tb11.02.2560	-
FAP216	Flagellar Associated Protein	-	-	-	+	-	-	-	-	-	-	-	-	194679	-	-	-	-	-	-	-	-	-	-	-	-	-	-	-	-	-	-	-	-	-	-	-	-	-	-	-	-	-	-	-	-	-	-	-	-	-	-
TEF2	Rhodanese-like, Ca-sensing receptor, chloroplast location proposed	-	-	-	-	-	-	-	AT5G23060.1	-	-	-	-	194676	-	-	-	-	-	-	-	-	-	-	-	-	-	-	-	-	-	Os02g49680.1	-	-	-	217313	-	-	296649	-	-	-	-	-	-	-	-	-	-	-	-	-
		-	-	-	-	-	-	-	-	-	-	-	-	194675	-	-	-	-	-	-	-	-	-	-	-	LmjF35.1690	-	-	-	-	-	-	-	-	-	-	-	-	-	-	-	-	TlrP62	-	-	-	-	-	-	-	-	-
	related to haem peroxidases	-	+	-	-	-	-	-	AT1G09510.1	12599	-	CE37632	-	194674	-	cgd5_1140	CMN284C	-	DDB0219261	-	-	-	-	-	-	-	136119	34581	81010	92936	NCU03358	Os01g34480.2	-	-	31257	184659	130176	-	557814	-	YGL157W	SPAC513.07	GLEAN3_25176	-	93.m00111	-	-	-	52229	-	-	UM06374.1
		-	-	-	-	-	-	-	-	-	-	-	-	194669	-	-	-	-	-	-	-	-	-	-	-	-	-	-	-	-	-	-	-	-	-	-	-	-	-	-	-	-	-	-	-	-	-	-	-	-	-	-
		-	-	-	-	-	-	-	-	-	-	-	-	194668	-	-	-	-	-	-	-	-	-	-	-	-	-	-	-	-	-	-	-	-	-	-	-	-	-	-	-	-	-	-	-	-	-	-	-	-	-	-
FAP128	Flagellar Associated Protein	-	-	-	+	-	-	-	-	-	-	-	-	194662	-	-	-	-	-	-	-	-	-	-	-	-	-	-	-	-	-	-	-	-	-	-	-	-	-	-	-	-	-	-	-	-	-	-	-	-	-	-
		-	-	-	-	-	-	-	-	-	-	-	-	194660	-	-	-	-	-	-	-	-	-	-	-	-	-	-	-	-	-	-	-	-	-	-	-	-	-	-	-	-	-	-	-	-	-	-	-	-	-	-
RBM25	Putative mRNA splicing factor, similar to human RNA binding motif protein 25	-	-	-	-	-	-	-	-	-	-	-	-	194658	-	-	-	-	-	-	-	-	-	-	-	-	-	-	-	-	-	-	-	-	-	-	-	-	-	-	-	-	-	-	-	-	-	-	-	-	-	-
		-	-	-	-	-	-	-	-	-	-	-	-	194644	-	-	-	-	-	-	-	-	-	-	-	-	-	-	-	-	-	-	-	-	-	-	-	-	-	-	-	-	-	-	-	-	-	-	-	-	-	-
		-	-	-	-	-	-	-	-	-	-	-	-	194642	-	-	-	-	-	-	-	-	-	-	-	-	-	-	-	-	-	-	-	-	-	-	-	-	-	-	-	-	-	-	-	-	-	-	-	-	-	-
		-	-	-	-	-	-	-	-	-	-	-	-	194641	-	-	-	-	-	-	-	-	-	-	-	-	-	-	-	-	-	-	-	-	-	-	-	-	-	-	-	-	-	-	-	-	-	-	-	-	-	-
PDE10	3',5'-cyclic-nucleotide phosphodiesterase;	-	-	-	-	-	-	-	-	-	-	-	-	194640	-	-	-	-	-	-	-	-	-	-	-	-	-	-	-	-	-	-	-	-	-	-	-	-	-	-	-	-	-	-	-	-	-	-	-	-	-	-
		-	+	-	-	-	-	GB13051-PA	AT4G32060.1	-	-	CE28375	174710	194638	376591	-	-	OTTDARP00000018752	DDB0187661	CG4495-PA	-	-	ENSGALP00000006962	-	OTTHUMP00000019787	LmjF07.0110	110115	32612	66130	89117	-	Os02g39950.1	30333	-	-	153029	142865	-	261204	-	-	-	-	SINFRUP00000176886	-	-	-	-	-	-	Tb927.8.1850	-
		-	-	-	-	-	-	-	-	-	-	-	-	194634	-	-	-	-	-	-	-	-	-	-	-	-	-	-	-	-	-	-	-	-	-	-	-	-	-	-	-	-	-	-	-	-	-	-	-	-	-	-

		-	-	-	-	-	-	-	-	-	-	-	-	194631	-	-	-	-	-	-	-	-	-	-	-	-	-	-	-	-	-	-	-	-	-	-	-	-	-	-	-	-	-	-	-	-	-	-	-	-	-	-
		-	-	-	-	-	-	-	-	-	-	-	-	194630	-	-	-	-	-	-	-	-	-	-	-	-	-	-	-	-	-	-	-	-	-	-	-	-	-	-	-	-	-	-	-	-	-	-	-	-	-	-
		-	-	-	-	-	-	-	-	-	-	-	-	194628	-	-	-	-	-	-	-	-	-	-	-	-	-	-	-	-	-	-	-	-	-	-	-	-	-	-	-	-	-	-	-	-	-	-	-	-	-	-
		-	-	-	-	-	-	-	-	-	-	-	-	194627	-	-	-	-	-	-	-	-	-	-	-	-	-	-	-	-	-	-	-	-	-	-	-	-	-	-	-	-	-	-	-	-	-	-	-	-	-	-
	putative programmed cell death 6 interacting protein	-	+	-	-	-	-	GB11744-PA	AT1G15130.1	-	BDEG_00218	CE31853	127570	194623	213255	-	CMC051C	OTTDARP00000015191	DDB0167145	CG9311-PA	-	97.m00146	ENSGALP00000019634	-	OTTHUMP00000164799	LmjF27.1640	207048	32312	79832	181204	NCU08001	Os10g35250.1	35814	-	48529	61593	142675	-	820653	RO3G_15329.1	YOR275C	SPAC17G6.05c	GLEAN3_20140	SINFRUP00000148152	-	-	-	-	38299	-	Tb11.18.0002	UM03879.1
FAP190	Flagellar Associated Protein	-	-	-	+	-	-	-	-	-	-	-	-	194620	-	-	-	-	-	-	-	-	-	-	-	-	-	-	-	-	-	-	-	-	-	-	-	-	-	-	-	-	-	-	-	-	-	-	-	-	-	-
		-	-	-	-	-	-	-	-	-	-	-	94023	194618	-	-	-	-	-	-	-	-	-	-	-	-	-	-	-	-	-	-	-	-	-	-	-	-	-	-	-	-	-	-	-	-	-	-	-	-	-	-
		-	-	-	-	-	-	-	-	-	-	-	-	194617	-	-	-	-	-	-	-	-	-	-	-	-	-	-	-	-	-	-	-	-	-	-	-	-	-	-	-	-	-	-	-	-	-	-	-	-	-	-
		-	+	-	-	-	-	-	-	-	-	-	-	194613	-	-	-	-	DDB0218211	-	-	-	-	-	-	-	-	-	-	231045	NCU06603	-	14288	-	9266	-	-	-	-	RO3G_07202.1	YDR533C	SPBC947.09	-	-	-	6979	-	-	-	-	-	UM00094.1
		+	+	-	-	-	-	GB15769-PA	-	-	-	CE36926	177499	194611	269889	-	-	-	-	CG33214-PA	-	-	ENSGALP00000004317	-	OTTHUMP00000107687	-	114272	-	-	81294	-	-	-	-	-	-	-	-	-	-	-	-	GLEAN3_08505	SINFRUP00000179634	-	-	-	-	-	-	-	-
		-	-	-	-	-	-	-	-	-	-	-	-	194610	-	-	-	-	-	-	-	-	-	-	-	-	-	-	-	-	-	-	-	-	-	-	-	-	-	-	-	-	-	-	-	-	-	-	-	-	-	-
SGA1	Putative serine glyoxylate aminotransferase, variant SGA1a	-	+	-	-	-	-	-	AT2G13360.2	-	BDEG_01393	-	-	194609	-	-	CMS429C	-	-	-	-	-	-	GL50803_3313	-	LmjF03.0040	-	-	-	-	-	Os08g39300.1	-	GSPATP00034177001	-	199392	109249	-	815301	RO3G_11559.1	YFL030W	-	-	-	7.m00422	-	-	-	-	-	-	-
		-	-	-	-	-	-	-	-	-	-	-	-	194607	-	-	-	-	-	-	-	-	-	-	-	-	-	-	-	-	-	-	-	-	-	-	-	-	-	-	-	-	-	-	-	-	-	-	-	-	-	-
		-	-	-	-	-	-	-	-	-	-	-	-	194605	-	-	-	-	-	-	-	-	-	-	-	-	-	-	-	-	-	-	-	-	-	-	-	-	-	-	-	-	-	-	-	-	-	-	-	-	-	-
		-	-	-	-	-	-	-	-	-	-	-	-	194604	-	-	-	-	-	-	-	-	-	-	-	-	-	-	-	-	-	-	-	-	-	-	-	-	-	-	-	-	-	-	-	-	-	-	-	-	-	-
		-	+	-	-	-	-	-	AT1G06110.1	-	-	-	144235	194601	-	-	-	-	-	-	-	-	-	-	-	-	-	-	-	-	-	Os11g16390.2	-	-	-	224718	-	-	594546	-	-	-	-	SINFRUP00000142251	-	8509	-	-	-	-	-	-
		-	-	-	-	-	-	-	-	-	-	-	-	194600	-	-	-	-	-	-	-	-	-	-	-	-	-	-	-	-	-	-	-	-	-	159130	-	-	-	-	-	-	-	-	-	-	-	-	-	-	-	-
ADCY39	Adenylate cyclase	-	-	-	-	-	-	-	-	-	-	-	-	194597	-	-	-	-	-	-	-	-	-	-	-	-	-	-	-	-	-	-	-	-	-	-	-	-	-	-	-	-	-	-	-	-	-	-	-	-	-	-
WEE1	WEE1 kinase, CDK inhibitory kinase	-	+	-	-	-	-	GB19689-PA	AT1G02970.1	53628	-	CE14884	226192	194589	271460	-	CMT590C	OTTDARP00000012833	DDB0191605	CG32417-PA	-	-	ENSGALP00000020972	-	NP_003381.1	LmjF29.2720	93229	29323	48087	97030	-	Os02g04240.1	-	GSPATP00021978001	-	193308	-	-	245893	-	-	-	GLEAN3_27012	SINFRUP00000136340	11.m00512	33955	-	-	-	-	Tb927.4.3420	-
		-	-	-	-	-	-	-	-	-	-	-	-	194588	-	-	-	-	-	-	-	-	-	-	-	-	-	-	-	-	-	-	-	-	-	-	124777	-	-	-	-	-	-	-	-	-	-	-	-	-	-	-
		-	-	-	-	-	-	-	-	-	-	-	-	194587	-	-	-	-	-	-	-	-	-	-	-	-	-	-	-	-	-	-	-	-	-	-	-	-	-	-	-	-	-	-	-	-	-	-	-	-	-	-
		-	-	-	-	-	-	-	-	-	-	-	-	194579	-	-	-	-	-	-	-	-	-	-	-	-	-	-	-	-	-	-	-	-	-	-	-	-	-	-	-	-	-	-	-	-	-	-	-	-	-	-
MMP10	metalloproteinase, cell wall protein, homolog of Volvox VMPs	-	-	-	-	-	-	-	-	-	-	-	-	194578	-	-	-	-	-	-	-	-	-	-	-	-	-	-	-	-	-	-	-	-	-	-	-	-	-	-	-	-	-	-	-	-	-	-	-	-	-	-
		-	-	-	-	-	-	-	-	-	-	-	-	194577	-	-	-	-	-	-	-	-	-	-	-	-	-	-	-	-	-	-	-	-	-	-	-	-	-	-	-	-	-	-	-	-	-	-	-	-	-	-
MMP7	metalloproteinase, cell wall protein, homolog of Volvox VMPs	-	-	-	-	-	-	-	-	-	-	-	-	194576	-	-	-	-	-	-	-	-	-	-	-	-	-	-	-	-	-	-	-	-	-	-	-	-	-	-	-	-	-	-	-	-	-	-	-	-	-	-
		-	-	-	-	-	-	-	-	-	-	-	-	194571	-	-	-	-	-	-	-	-	-	-	-	-	-	-	-	-	-	-	-	-	-	-	-	-	-	-	-	-	-	-	-	-	-	-	-	-	-	-
		-	-	-	-	-	-	-	-	-	-	-	-	194565	-	-	-	-	-	-	-	-	-	-	-	-	-	-	-	-	-	-	-	-	-	-	-	-	-	-	-	-	-	-	-	-	-	-	-	-	-	-
		-	-	-	-	-	-	-	-	-	-	-	-	194558	-	-	-	-	-	-	-	-	-	-	-	-	-	-	-	-	-	-	-	-	-	-	-	-	-	-	-	-	-	-	-	-	-	-	-	-	-	-
MRPL7/L12	Putative mitochondrial ribosomal protein L7/L12, imported to mitochondria	-	+	-	-	-	-	GB14723-PA	AT4G36420.1	-	-	CE16564	152560	194556	-	-	CMR179C	-	DDB0167610	CG5012-PA	-	-	ENSGALP00000011641	-	OTTHUMP00000183333	LmjF14.0150	-	24571	-	123898	NCU06469	Os03g52400.1	7356	-	35513	18929	137290	PFB0545c	757002	RO3G_13823.1	YGL068W	SPCC4B3.09c	GLEAN3_24214	SINFRUP00000135573	163.m00048	268296	TA15030	50.m03428	20666	-	Tb927.7.4550	UM05060.1
		-	+	-	-	-	-	GB18356-PA	-	61826	-	CE25419	204990	194555	215001	cgd7_1120	CMF161C	OTTDARP00000002091	DDB0190840	CG3642-PA	19068444	134.m00122	ENSGALP00000028034	-	OTTHUMP00000173269	LmjF09.0720	200237	14495	-	242949	-	Os04g35800.1	23861	GSPATP00029861001	-	-	-	-	759960	-	-	-	GLEAN3_12597	SINFRUP00000142106	-	-	-	50.m05612	52522	-	Tb11.01.4600	-
		-	-	-	-	-	-	-	-	-	-	-	-	194549	-	-	-	-	-	-	-	-	-	-	-	-	-	-	-	-	-	-	-	-	-	-	-	-	-	-	-	-	-	-	-	-	-	-	-	-	-	-
		-	-	-	-	-	-	-	-	-	-	-	-	194548	-	-	-	-	-	-	-	-	-	-	-	-	-	-	-	-	-	-	-	-	-	-	-	-	-	-	-	-	-	-	-	-	-	-	-	-	-	-
		-	-	-	-	-	-	-	-	-	-	-	-	194547	-	-	-	-	-	-	-	-	-	-	-	-	-	-	-	-	-	-	-	-	-	-	-	-	-	-	-	-	-	-	-	-	-	-	-	-	-	-
AGT1	alanine-glyoxylate transaminase, putative	-	+	-	-	-	-	GB20058-PA	-	32627	-	CE23985	164164	194541	207348	-	-	OTTDARP00000020018	DDB0188646	CG3926-PA	-	-	ENSGALP00000032984	-	OTTHUMP00000164504	-	134376	-	-	112567	-	-	-	-	-	-	-	-	-	-	-	-	-	SINFRUP00000147844	-	22208	-	49.m03154	26208	-	-	UM02621.1
		-	-	-	-	-	-	-	-	-	-	-	-	194533	-	-	-	-	-	-	-	-	-	-	-	-	-	-	-	-	-	-	-	-	-	-	-	-	-	-	-	-	-	-	-	-	-	-	-	-	-	-
		-	+	-	-	-	-	-	AT5G66450.2	67050	-	-	-	194530	-	-	-	-	-	-	-	-	-	-	-	LmjF23.1665	-	-	-	-	-	Os02g47570.1	35063	-	32083	-	156389	-	563190	-	-	-	-	-	-	-	-	-	-	-	-	-
		-	-	-	-	-	-	-	-	-	-	-	-	194524	-	-	-	-	-	-	-	-	-	-	-	-	-	-	-	-	-	-	-	-	-	-	-	-	-	-	-	-	-	-	-	-	-	-	-	-	-	-
		-	+	-	-	-	-	GB13443-PA	AT1G48930.1	-	-	-	-	194523	243516	-	-	-	-	-	-	-	-	-	-	-	-	-	-	-	-	-	-	-	-	-	-	-	751215	-	-	-	-	-	-	-	-	-	-	-	-	UM00655.1
		-	-	-	-	-	-	-	-	-	-	-	-	194522	-	-	-	-	-	-	-	-	-	-	-	-	-	-	-	-	-	-	-	-	-	-	-	-	-	-	-	-	-	-	-	-	-	-	-	-	-	-
		-	-	-	-	-	-	-	-	-	-	-	-	194521	-	-	-	-	-	-	-	-	-	-	-	-	-	-	-	-	-	Os06g50140.1	-	-	-	-	-	-	424296	RO3G_11064.1	-	-	-	-	-	-	-	-	-	-	-	-
		+	+	-	-	-	-	GB19330-PA	-	-	-	CE40255	-	194519	-	-	-	-	-	CG32354-PA	-	-	ENSGALP00000003166	-	XP_001126326.1	-	210962	31785	59623	52039	-	-	-	-	-	-	-	-	-	-	-	-	-	SINFRUP00000180561	-	23814	-	55.m04849	-	-	-	-
	emp24/gp25L/p24 family protein	-	+	-	-	-	-	-	AT3G22845.1	-	-	CE14448	-	194512	299271	-	-	-	-	-	-	-	ENSGALP00000005231	-	-	LmjF32.2020	203874	34870	-	-	NCU03800	Os03g53310.1	-	-	41327	201908	143800	-	821950	-	YOR016C	-	-	-	-	-	-	-	-	-	-	-
MRPS15	Putative mitochondrial ribosomal protein S15, imported to mitochondria	-	+	-	-	-	-	GB15819-PA	-	-	BDEG_03925	-	159664	194511	-	-	CMH066C	-	-	CG4207-PA	-	-	-	-	OTTHUMP00000009727	-	-	-	31223	-	-	Os06g07996.1	-	GSPATP00003475001	-	170717	-	-	817568	RO3G_05038.1	YDR337W	SPBC11B10.04c	-	SINFRUP00000136765	13.m00330	-	TA10700	-	57003	-	-	UM04148.1
		-	+	-	-	-	-	GB15136-PA	AT1G25520.1	22875	BDEG_05760	CE27004	159789	194510	264431	cgd4_420	CMS279C	OTTDARP00000004827	-	-	-	-	ENSGALP00000022363	-	OTTHUMP00000158929	LmjF19.0310	212670	14366	-	129943	NCU01990	Os08g33630.1	16573	GSPATP00015091001	6052	136505	127222	-	810826	RO3G_11671.1	YBR187W	SPAC17G8.08c	-	SINFRUP00000133468	200.m00036	36568	-	641.m01520	30261	-	-	UM04425.1
		-	-	-	-	-	-	-	-	-	-	-	-	194509	-	-	-	-	-	-	-	-	-	-	-	-	-	-	-	-	-	-	-	-	-	-	-	-	-	-	-	-	-	-	-	-	-	-	-	-	-	-
		-	-	-	-	-	-	-	-	-	-	-	-	194508	-	-	-	-	-	-	-	-	-	-	-	-	-	-	-	-	-	-	-	-	-	-	-	-	-	-	-	-	-	-	-	-	-	-	-	-	-	-
FAP130	Flagellar Associated Protein	-	-	-	+	-	-	-	-	-	-	-	-	194498	-	-	-	-	-	-	-	-	-	-	-	-	-	-	-	-	-	-	-	-	-	-	-	-	-	-	-	-	-	-	-	-	-	-	-	-	-	-
		-	-	-	-	-	-	-	-	-	-	-	-	194497	-	-	-	-	-	-	-	-	-	-	-	-	-	-	-	-	-	-	-	-	-	-	-	-	-	-	-	-	-	-	-	-	-	-	-	-	-	-
		-	-	-	-	-	-	-	-	-	-	-	-	194495	-	-	-	-	-	-	-	-	-	-	-	-	-	-	-	-	-	-	-	-	-	-	-	-	-	-	-	-	-	-	-	-	-	-	-	-	-	-
CUTC	copper homeostasis protein cutC homolog	-	+	-	-	-	-	GB10405-PA	-	59883	-	CE00391	110971	194494	-	-	-	OTTDARP00000023902	DDB0187520	-	-	-	ENSGALP00000011996	-	OTTHUMP00000020260	-	117637	3296	62888	118443	NCU01374	-	-	GSPATP00031928001	-	139003	-	-	-	-	-	-	GLEAN3_01559	SINFRUP00000146486	37.m00255	-	-	-	-	89808.m00120	-	-
		-	-	-	-	-	-	-	-	-	-	-	-	194492	-	-	-	-	-	-	-	-	-	-	-	-	-	-	-	-	-	-	-	-	-	-	-	-	-	-	-	-	-	-	-	-	-	-	-	-	-	-
		-	-	-	-	-	-	-	-	-	-	-	-	194491	-	-	-	-	-	-	-	-	-	-	-	-	-	-	-	-	-	-	-	-	-	-	-	-	-	-	-	-	-	-	-	-	-	-	-	-	-	-
		-	-	-	-	-	-	-	-	68829	-	-	-	194490	-	-	-	-	-	-	-	-	-	-	-	-	-	-	-	-	-	-	-	-	-	-	-	-	752847	-	-	-	-	-	-	-	-	-	-	-	Tb927.7.5590	-
		-	-	-	-	-	-	-	-	-	-	-	-	194488	-	-	-	-	-	-	-	-	-	-	-	-	-	-	-	-	-	-	-	-	-	-	-	-	-	-	-	-	-	-	-	-	-	-	-	-	-	-
		-	-	-	-	-	-	-	-	-	-	-	-	194486	-	-	-	-	-	-	-	-	-	-	-	-	-	-	-	-	-	-	-	-	-	-	-	-	-	-	-	-	-	-	-	-	-	-	-	-	-	-
		-	-	-	-	-	-	-	AT5G04900.1	-	-	-	-	194485	-	-	-	-	-	-	-	-	-	-	-	-	-	-	-	-	-	Os03g45194.1	-	-	-	152699	-	-	563522	-	-	-	-	-	-	-	-	-	-	-	-	-
		-	-	-	-	-	-	-	-	65690	-	-	-	194484	-	-	-	-	-	-	-	-	-	-	-	-	-	-	-	-	-	-	10371	-	50384	-	-	-	-	-	-	-	-	-	-	23492	-	-	-	-	-	-
		-	-	-	-	-	-	-	-	-	-	-	-	194483	-	-	-	-	-	-	-	-	-	-	-	-	-	-	-	-	-	-	-	-	-	-	-	-	-	-	-	-	-	-	-	-	-	-	-	-	-	-
		-	-	-	-	-	-	-	-	-	-	-	-	194479	-	-	-	-	-	-	-	-	-	-	-	-	-	-	-	-	-	-	-	-	-	-	-	-	-	-	-	-	-	-	-	-	-	-	-	-	-	-
PBGD1	Porphobilinogen deaminase, putative chloroplast precursor	-	+	-	+	-	-	GB15699-PA	AT5G08280.1	27306	BDEG_07550	-	178034	194475	247224	-	CME132C	OTTDARP00000018175	DDB0186148	CG9165-PA	-	-	ENSGALP00000000379	-	NP_001019553.1	-	218866	-	-	11360	NCU10292	Os02g07230.1	28226	GSPATP00027866001	51811	135086	131527	PFL0480w	802785	RO3G_09329.1	YDL205C	SPAC24B11.13	GLEAN3_25015	SINFRUP00000128973	31.m00323	9021	-	59.m03576	56012	-	-	UM01134.1
SETc		-	-	-	-	-	-	-	-	-	-	-	-	194472	-	-	-	-	-	-	-	-	-	-	-	-	-	-	-	-	-	-	-	-	-	-	-	-	-	-	-	-	-	-	-	-	-	-	-	-	-	-
		-	+	-	-	-	-	GB15014-PA	AT1G02120.1	3287	-	CE34444	227616	194471	-	-	CMR062C	OTTDARP00000009569	DDB0189665	CG3304-PB	-	189.m00088	ENSGALP00000010480	-	NP_065946.2	-	104744	26412	80433	92968	NCU06999	Os09g34130.1	11306	GSPATP00005710001	46733	136794	155784	MAL8P1.143	677682	RO3G_02061.1	YDR326C	SPBC20F10.07	-	SINFRUP00000158542	37.m00278	-	-	583.m09111	54487	-	-	UM03373.1
		-	-	-	-	-	-	-	-	-	-	-	-	194469	-	-	-	-	-	-	-	-	-	-	-	-	-	-	-	-	-	-	-	-	-	-	-	-	-	-	-	-	-	-	-	-	-	-	-	-	-	-
PDE18	3',5'-cyclic-nucleotide phosphodiesterase	-	-	-	-	-	-	-	-	-	-	-	-	194468	-	-	-	-	DDB0216061	-	-	-	-	-	-	-	-	-	-	-	-	-	-	-	-	-	-	-	-	-	-	-	-	-	-	-	-	-	-	-	-	-
		-	+	-	-	-	-	-	-	-	-	-	-	194465	-	-	-	-	-	-	-	-	-	-	-	-	-	-	-	-	-	-	-	GSPATP00014992001	-	-	127573	-	-	-	-	-	-	-	230.m00032	-	-	583.m00708	-	86458.m00032	-	-
SHMT1	Serine hydroxymethyltransferase	-	+	-	-	-	-	-	AT4G37930.1	20758	BDEG_00944	CE29661	-	194461	282807	-	CMO142C	OTTDARP00000020249	-	CG3011-PA	-	-	ENSGALP00000008079	-	OTTHUMP00000168146	-	236570	-	34718	129467	NCU02274	Os03g52840.1	29475	GSPATP00031323001	54015	129878	108778	-	833897	RO3G_07399.1	YLR058C	SPAC18G6.04c	-	SINFRUP00000171194	54.m00253	269942	-	-	20682	-	-	-
NUOB16	NADH:ubiquinone oxidoreductase (Complex I) subunit, homolog to bovine B16.6 subunit, mitochondrial	-	+	-	-	-	-	GB11683-PA	AT1G04630.1	27550	BDEG_02630	CE16896	168607	194458	-	-	CMI118C	-	DDB0167875	CG3446-PA	-	-	-	-	OTTHUMP00000067882	-	-	11846	80507	108313	NCU09299	Os03g09210.1	34172	-	28620	162309	-	-	548007	RO3G_08458.1	-	-	GLEAN3_03408	SINFRUP00000163542	-	38261	-	-	54658	-	-	UM01311.1
		-	+	-	-	-	-	-	AT1G51350.1	-	BDEG_00475	-	150039	194456	270951	-	-	-	DDB0201835	-	-	-	ENSGALP00000001786	-	OTTHUMP00000172960	-	201652	-	-	162089	-	-	-	-	-	192889	-	-	722931	RO3G_08002.1	-	SPAC26H5.04	-	SINFRUP00000155301	-	-	-	-	-	-	-	-
		-	+	-	-	-	-	-	AT5G25752.1	-	-	-	-	194455	-	-	-	-	-	CG17212-PA	-	-	-	-	-	-	-	27089	-	-	-	Os05g13370.1	35635	GSPATP00007598001	-	135919	144957	-	260795	RO3G_04777.1	-	-	-	-	-	-	-	-	-	-	-	-
		-	-	-	-	-	-	-	-	-	-	-	-	194454	-	-	-	-	-	-	-	-	-	-	-	-	-	-	-	-	-	-	-	-	-	-	-	-	-	-	-	-	-	-	-	-	-	-	-	-	-	-
		-	+	-	-	-	-	-	-	10203	-	-	-	194451	-	-	-	-	-	-	-	-	ENSGALP00000008367	-	OTTHUMP00000080231	-	220094	22969	-	-	-	Os01g48680.2	-	-	43828	-	132791	-	-	RO3G_00403.1	-	-	GLEAN3_11692	SINFRUP00000165365	-	-	-	-	21513	-	-	-
		-	+	-	-	-	-	GB16323-PA	-	-	-	-	134859	194450	249763	-	-	OTTDARP00000021941	-	CG9071-PB	-	-	ENSGALP00000009909	-	NP_001092874.1	LmjF34.0480	118343	-	-	122010	-	-	-	GSPATP00005636001	-	-	-	-	837245	-	-	-	GLEAN3_25997	SINFRUP00000154265	40.m00187	-	-	-	-	-	Tb10.70.4750	-
		-	-	-	-	-	-	-	-	-	-	-	-	194449	-	-	-	-	-	-	-	-	-	-	-	-	-	-	-	-	-	-	33696	-	-	-	-	-	-	-	-	-	-	-	-	-	-	-	-	-	-	-
TEF6	unknown function, chloroplast location proposed	-	+	-	-	-	-	-	AT5G38660.1	-	-	-	-	194448	-	-	CMC040C	-	-	-	-	-	-	-	-	-	-	-	-	-	-	Os08g27010.1	5388	-	48524	148186	-	-	198975	-	-	-	-	-	-	4382	-	-	-	-	-	-
		-	-	-	-	-	-	-	-	-	-	-	-	194447	-	-	-	-	-	-	-	-	-	-	-	-	-	-	-	-	-	-	-	-	-	-	-	-	-	-	-	-	-	-	-	-	-	-	-	-	-	-
FAP48	Flagellar Associated Protein	+	+	-	+	-	-	GB11927-PA	-	-	-	CE31690	222687	194442	-	-	-	OTTDARP00000012867	-	CG1063-PB	-	-	ENSGALP00000013491	-	NP_002213.4	-	175913	-	52142	244707	-	-	-	-	-	-	-	-	-	-	-	-	GLEAN3_12078	SINFRUP00000168598	-	-	-	-	56365	-	-	-
		-	+	-	-	-	-	-	-	-	-	-	-	194438	-	-	-	-	-	-	-	-	ENSGALP00000007712	-	-	-	214465	-	-	161869	-	-	-	-	-	130478	-	-	726941	-	-	-	-	-	-	-	-	-	-	-	-	-
CK2A	casein kinase 2 alpha chain	-	+	+	-	-	-	GB11423-PA	AT5G67380.1	38704	BDEG_08078	CE17321	162678	194436	297237	cgd6_620	CMP128C	OTTDARP00000014271	DDB0217884	CG17520-PB	19170897	53.m00188	ENSGALP00000009985	GL50803_27520	OTTHUMP00000029926	LmjF35.1730	184685	35210	75313	166091	NCU03124	Os07g02350.1	8917	GSPATP00013852001	54091	218940	109488	PF11_0096	414425	RO3G_07160.1	YIL035C	SPAC23C11.11	GLEAN3_12605	SINFRUP00000180998	190.m00047	39539	TA10630	55.m00233	50201	82397.m00111	Tb09.211.4890	UM01180.1
		-	-	-	-	-	-	-	-	-	-	-	-	194434	-	-	-	-	-	-	-	-	-	-	-	-	-	-	-	-	-	-	-	-	-	-	-	-	-	-	-	-	-	-	-	-	-	-	-	-	-	-
		-	-	-	+	-	-	-	-	-	-	-	-	194431	-	-	-	-	-	-	-	-	-	-	-	-	-	-	-	-	-	-	-	-	-	-	-	-	-	-	-	-	-	-	-	-	-	-	-	-	-	-
		-	-	-	-	-	-	-	-	-	-	-	-	194421	-	-	-	-	-	-	-	-	-	-	-	-	-	-	-	-	-	-	-	-	-	-	-	-	-	-	-	-	-	-	-	-	-	-	-	-	-	-
		-	-	-	-	-	-	-	-	-	-	-	-	194408	-	-	-	-	-	-	-	-	-	-	-	-	-	-	-	-	-	-	-	-	-	-	-	-	-	-	-	-	-	-	-	-	-	-	-	-	-	-
		-	-	-	-	-	-	-	-	-	-	-	-	194403	-	-	-	-	-	-	-	-	-	-	OTTHUMP00000030101	-	-	-	-	-	-	-	-	-	-	-	139409	-	-	RO3G_09211.1	-	-	-	SINFRUP00000157404	-	-	-	-	-	-	-	-
		+	+	-	-	-	-	-	-	65844	BDEG_06556	-	222450	194402	220445	-	-	-	-	CG14921-PA	-	-	ENSGALP00000038345	-	NP_001028732.1	LmjF32.2850	112670	31996	-	501	-	-	-	-	-	-	128945	-	-	-	-	-	-	-	-	-	-	86.m00392	-	-	Tb11.01.7710	-
		-	-	-	-	-	-	-	-	-	-	-	-	194395	-	-	-	-	-	-	-	-	-	-	-	-	-	-	-	-	-	-	-	-	-	-	-	-	-	-	-	-	-	-	-	-	-	-	-	-	-	-
		-	-	-	-	-	-	-	-	-	-	-	-	194390	-	-	-	-	-	-	-	-	-	-	-	-	-	-	-	-	-	-	-	-	-	-	-	-	-	-	-	-	-	-	-	-	-	-	-	-	-	-
POA2	20S proteasome alpha subunitB	-	+	+	-	-	-	GB10390-PA	AT1G16470.2	28095	-	CE05521	154995	194387	210127	cgd7_3660	CML273C	OTTDARP00000015882	DDB0184241	-	-	31.m00225	ENSGALP00000030471	GL50803_11434	OTTHUMP00000025534	LmjF21.1700	189380	33583	55832	165493	NCU06764	Os03g26970.1	8478	GSPATP00036491001	49897	64434	109244	PFF0420c	823550	RO3G_00510.1	YML092C	SPCC1442.06	GLEAN3_26428	SINFRUP00000152285	-	30683	TA18320	76.m00005	38289	83787.m00062	Tb10.100.0170	UM05652.1
SF3b3	Nuclear pre-mRNA splicing factor, component of splicing factor 3b	-	+	+	-	-	-	GB11574-PA	AT3G55200.1	23619	BDEG_05068	CE17155	155561	194382	259962	cgd8_4240	CML103C	OTTDARP00000013028	DDB0204844	CG13900-PA	-	136.m00117	ENSGALP00000003978	-	OTTHUMP00000174907	LmjF05.0890	188836	36939	45594	238290	NCU00396	Os02g04480.1	33381	GSPATP00004043001	17408	227044	109120	PFL1680w	803217	RO3G_06174.1	YML049C	SPAPJ698.03c	GLEAN3_00101	SINFRUP00000148066	67.m00213	35239	TA07140	44.m00036	49802	80671.m00471	Tb927.7.6980	UM00737.1
		-	-	-	+	-	-	-	-	-	-	-	-	194369	-	-	-	-	-	-	-	-	-	-	-	-	-	-	-	-	-	-	-	-	-	-	-	-	-	-	-	-	-	-	-	-	-	-	-	-	-	-
		-	-	-	-	-	-	-	-	-	-	-	-	194368	-	-	-	-	-	-	-	-	-	-	-	-	-	-	-	-	-	-	-	-	-	-	-	-	-	-	-	-	-	-	-	-	-	-	-	-	-	-
		-	-	-	-	-	-	-	-	-	-	-	-	194361	-	-	-	-	-	-	-	-	-	-	-	-	-	-	-	-	-	-	-	-	-	-	-	-	-	-	-	-	-	-	-	-	-	-	-	-	-	-
		-	-	-	-	-	-	-	-	-	-	-	-	194360	-	-	-	-	-	-	-	-	-	-	-	-	-	-	-	-	-	-	-	-	-	-	-	-	-	-	-	-	-	-	-	-	-	-	-	-	-	-
		-	-	-	-	-	-	-	-	-	-	-	-	194359	-	-	-	-	-	-	-	-	-	-	-	-	232618	-	-	-	-	-	-	-	-	-	-	-	-	-	-	-	-	-	-	-	-	-	-	-	-	-
		-	-	-	-	-	-	-	-	-	-	-	-	194353	-	-	-	-	-	-	-	-	-	-	-	-	-	-	-	-	-	-	-	-	-	-	-	-	-	-	-	-	-	-	-	-	-	-	-	-	-	-
		-	-	-	-	-	-	-	-	-	-	-	-	194352	-	-	-	-	-	-	-	-	-	-	-	-	-	-	-	-	-	-	-	-	-	-	-	-	-	-	-	-	-	-	-	-	-	-	-	-	-	-
		-	-	-	-	-	-	-	-	-	-	-	-	194351	-	-	-	-	-	-	-	-	-	-	-	-	-	-	-	-	-	-	-	-	-	-	-	-	-	-	-	-	-	-	-	-	-	-	-	-	-	-
		-	-	-	-	-	-	-	-	-	-	-	-	194350	-	-	-	-	-	-	-	-	-	-	-	-	-	-	-	-	-	-	-	-	-	-	-	-	-	-	-	-	-	-	-	-	-	-	-	-	-	-
		-	+	+	-	-	-	GB16635-PA	AT2G03820.1	70174	BDEG_00359	CE30306	174567	194349	261681	cgd3_1340	CMT194C	-	DDB0202851	CG3460-PA	19171385	38.m00228	ENSGALP00000015500	GL50803_24860	OTTHUMP00000173426	LmjF26.0970	206277	34439	36359	144377	NCU08663	Os10g42320.1	37111	GSPATP00024934001	15862	132997	109580	PF07_0121	800704	RO3G_10376.1	YHR170W	SPAC16C9.03	-	SINFRUP00000152513	56.m00171	29018	TA05875	35.m00885	27425	92145.m00157	Tb927.7.970	UM04021.1
		-	-	-	-	-	-	-	-	-	-	-	-	194347	-	-	-	-	-	-	-	-	-	-	-	-	-	-	-	-	-	-	-	-	-	-	-	-	-	-	-	-	-	-	-	-	-	-	-	-	-	-
		-	-	-	-	-	-	-	-	-	-	-	-	194339	-	-	-	-	-	-	-	-	-	-	-	-	-	-	-	-	-	-	-	-	-	-	133141	-	-	-	-	-	-	-	-	-	-	-	-	-	-	-
FAP57	Conserved Uncharacterized Flagellar Associated Protein	+	+	-	+	-	-	GB18721-PA	-	205	BDEG_01561	-	108742	194338	225137	-	-	-	-	CG4329-PA	-	-	ENSGALP00000016085	-	NP_689711.1	LmjF16.1550	118162	13840	61313	213283	-	-	32847	GSPATP00024356001	-	66119	135376	PF14_0062	-	-	-	-	-	SINFRUP00000161845	7.m00479	-	-	57.m01729	27651	87414.m00361	Tb927.8.4870	-
		-	-	-	-	-	-	-	-	-	-	-	-	194337	-	-	-	-	-	-	-	87.m00154	-	-	-	-	-	-	-	-	-	-	-	-	-	-	-	-	-	-	YHL008C	-	-	-	-	-	-	25.m02951	-	94291.m00070	-	-
		-	-	-	-	-	-	-	-	-	-	-	-	194335	-	-	-	-	-	-	-	-	-	-	-	-	-	-	-	-	-	-	-	-	-	-	-	-	-	-	-	-	-	-	-	-	-	-	-	-	-	-
PRPL35	Plastid ribosomal protein L35, imported to chloroplast, large ribosomal subunit	-	+	-	-	-	-	-	AT2G24090.1	-	-	-	-	194331	-	-	-	-	-	-	-	-	-	-	-	-	-	-	78088	-	-	Os06g43900.1	-	-	-	151797	-	-	737939	-	-	-	-	-	-	-	-	-	-	-	-	-
		-	-	-	-	-	-	-	-	-	-	-	-	194328	-	-	-	-	-	-	-	-	-	-	-	-	-	-	-	-	-	-	-	-	-	-	-	-	-	-	-	-	-	-	-	-	-	-	-	-	-	-
LCI33	low-CO2-inducible protein	-	+	-	-	-	-	-	-	-	-	-	-	194325	-	-	-	-	-	-	-	-	-	-	-	LmjF05.0850	-	-	78083	-	-	-	-	GSPATP00021613001	-	161157	-	-	-	-	-	-	-	-	-	-	-	-	-	-	Tb927.7.7030	-
		-	-	-	-	-	-	-	-	-	-	-	-	194324	-	-	-	-	-	-	-	-	-	-	-	-	-	-	-	-	-	-	-	-	-	-	-	-	-	-	-	-	-	-	-	-	-	-	-	-	-	-
		-	-	-	-	-	-	-	-	-	-	-	-	194317	-	-	-	-	-	-	-	-	-	-	-	-	-	-	-	-	-	-	-	-	-	-	-	-	-	-	-	-	-	-	-	-	-	-	-	-	-	-
PDX1	pyridoxin biosynthesis protein	-	+	-	-	-	-	-	AT5G01410.1	-	BDEG_04257	-	222963	194316	298662	-	CMP070C	-	DDB0187880	-	-	-	-	-	-	-	232087	26702	80061	194582	NCU06550	Os07g01020.1	23422	-	29885	176118	156086	PFF1025c	576973	RO3G_11214.1	YFL059W	SPAC29B12.04	-	-	-	42612	-	46.m00006	-	-	-	UM03824.1
		-	-	-	-	-	-	-	-	-	-	-	-	194314	-	-	-	-	-	-	-	-	-	-	-	-	-	-	-	-	-	-	-	-	-	-	-	-	-	-	-	-	-	-	-	-	-	-	-	-	-	-
		-	-	-	-	-	-	-	-	-	-	-	-	194311	-	-	-	-	-	-	-	-	-	-	-	-	-	-	-	-	-	-	-	-	-	-	-	-	-	-	-	-	-	-	-	-	-	-	-	-	-	-
		-	-	-	-	-	-	-	-	-	-	-	-	-	-	-	-	-	-	-	-	-	-	-	-	-	-	-	-	-	-	-	-	-	-	-	-	-	-	-	-	-	-	-	-	-	-	-	-	-	-	-
		-	-	-	-	-	-	-	-	-	-	-	-	194308	-	-	-	-	-	-	-	-	-	-	-	-	-	-	-	-	-	-	-	-	-	-	-	-	-	-	-	-	-	-	-	-	-	-	-	-	-	-
		-	-	-	-	-	-	-	-	-	-	-	-	194304	-	-	-	-	-	-	-	-	-	-	-	-	-	-	-	-	-	-	-	-	-	-	-	-	-	-	-	-	-	-	-	-	-	-	-	-	-	-
		-	-	-	-	-	-	-	-	72034	-	-	-	194303	-	-	-	-	-	-	-	-	-	-	-	-	-	-	-	-	-	-	-	-	48329	-	-	-	-	-	-	-	-	-	-	5545	-	-	-	-	-	-
		-	-	-	-	-	-	-	-	-	-	-	-	194299	-	-	-	-	-	-	-	-	-	-	-	-	-	-	-	-	-	-	-	-	-	-	-	-	-	-	-	-	-	-	-	-	-	-	-	-	-	-
PFLA	Pyruvate formate lyase activating enzyme	-	-	-	-	-	-	-	-	-	-	-	-	194298	-	-	-	-	-	-	-	-	-	-	-	-	-	-	-	-	-	-	15818	-	-	-	-	-	-	-	-	-	-	-	-	-	-	-	-	-	-	-
		-	+	+	-	-	-	GB18336-PA	AT2G30000.1	-	BDEG_04604	CE33882	226225	194293	224423	cgd6_2400	CMS014C	OTTDARP00000021172	DDB0185995	CG9548-PA	-	135.m00108	ENSGALP00000019471	GL50803_32531	OTTHUMP00000028653	LmjF36.2920	210048	37730	60125	218593	NCU00348	Os05g30410.1	30411	GSPATP00026073001	51232	214625	109467	PF10_0179	706561	RO3G_02212.1	YPR094W	SPAC23H3.02c	GLEAN3_27966	SINFRUP00000181943	2.m02441	38802	TA11730	50.m05635	31103	95725.m00311	Tb10.6k15.3280	UM04493.1
		-	-	-	-	-	-	-	-	-	-	-	-	194290	-	-	-	-	-	-	-	-	-	-	-	-	-	-	-	-	-	-	-	-	-	-	-	-	-	-	-	-	-	-	-	-	-	-	-	-	-	-
		-	+	-	-	-	-	GB14794-PA	AT5G19680.1	-	BDEG_05382	CE01090	177567	194284	-	cgd6_750	CMM185C	-	DDB0185855	-	19171293	-	ENSGALP00000009351	-	OTTHUMP00000164571	LmjF05.1210	229573	14396	78842	181969	NCU08385	Os02g13640.1	-	GSPATP00031906001	14518	86576	136370	PF10_0320	828731	-	YKL193C	SPAC4A8.12c	-	SINFRUP00000135176	67.m00183	19351	TA07655	57.m01867	26327	-	Tb927.5.590	UM01477.1
		-	-	-	-	-	-	-	-	71695	-	-	-	194276	-	-	-	-	-	-	-	-	-	-	-	-	-	-	-	-	-	-	-	-	-	-	-	-	-	-	-	-	-	-	-	-	-	-	-	-	-	-
		-	-	-	-	-	-	-	-	-	-	-	-	194269	-	-	-	-	-	-	-	-	-	-	-	-	-	-	-	243959	-	-	-	-	-	-	-	-	-	-	-	-	-	-	-	-	-	-	-	-	-	-
		-	-	-	-	-	-	-	-	-	-	-	-	194265	-	-	-	-	-	-	-	-	-	-	-	-	-	-	-	-	-	-	-	-	-	-	-	-	-	-	-	-	-	-	-	-	-	-	-	-	-	-
PHC12	cell wall protein pherophorin-C12	-	-	-	+	-	-	-	-	-	-	-	-	194264	-	-	-	-	-	-	-	-	-	-	-	-	-	-	-	-	-	-	-	-	-	-	-	-	-	-	-	-	-	-	-	-	-	-	-	-	-	-
		-	-	-	-	-	-	-	-	-	-	-	-	194261	-	-	CMS187C	-	-	-	-	-	-	-	-	-	-	-	-	-	-	Os04g31620.1	-	-	46145	-	-	-	-	-	-	-	-	-	-	-	-	-	-	-	-	-
		-	-	-	-	-	-	-	-	-	-	-	-	194259	-	-	-	-	-	-	-	-	-	-	-	-	-	-	-	-	-	-	-	-	-	-	-	-	-	-	-	-	-	-	-	-	-	-	-	-	-	-
PGP1b		-	+	-	-	-	-	GB16521-PA	AT2G39290.1	15577	-	-	-	194257	-	-	CMJ134C	-	-	-	-	-	-	-	-	-	-	-	-	-	-	Os03g17520.1	-	-	8663	26407	-	-	832519	-	-	-	-	-	-	37097	-	-	-	83472.m00446	-	-
FAP255	Flagellar Associated Protein	-	-	-	-	-	-	-	-	-	-	-	-	194255	-	-	-	-	-	-	-	-	-	-	OTTHUMP00000014802	-	-	-	-	-	-	-	-	-	-	-	-	-	-	-	-	-	-	-	-	-	-	-	-	-	-	-
		-	-	-	-	-	-	-	-	-	-	-	-	194246	-	-	-	-	-	-	-	-	-	-	-	-	-	-	-	-	-	-	-	-	-	-	-	-	-	-	-	-	-	-	-	-	-	-	-	-	-	-
	hypothetical protein	-	-	-	-	-	-	-	-	-	-	-	-	194245	-	-	-	-	-	-	-	-	-	-	-	-	-	-	-	-	-	-	-	-	-	-	-	-	-	-	-	-	-	-	-	-	-	-	-	-	-	-
		-	-	-	-	-	-	-	-	-	-	-	-	194242	-	-	-	-	-	-	-	-	-	-	-	-	-	-	-	-	-	-	-	-	-	-	-	-	-	-	-	-	-	-	-	-	-	-	-	-	-	-
		-	-	-	-	-	-	-	-	-	-	-	-	194241	-	-	-	-	-	-	-	-	-	-	-	-	-	-	-	-	-	-	-	-	-	-	-	-	-	-	-	-	-	-	-	-	-	-	-	-	-	-
FAP143	Flagellar Associated Protein	-	-	-	-	-	-	-	-	-	-	-	-	-	-	-	-	-	-	-	-	-	-	-	-	-	-	-	-	-	-	-	-	-	-	-	-	-	-	-	-	-	-	-	-	-	-	-	-	-	-	-
		-	-	-	-	-	-	-	-	-	-	-	-	194236	-	-	-	-	-	-	-	-	-	-	-	-	-	-	-	-	-	-	-	-	-	-	-	-	-	-	-	-	-	-	-	-	-	-	-	-	-	-
		-	-	-	-	-	-	-	-	-	-	-	-	194235	-	-	-	-	-	-	-	-	-	-	-	-	-	-	-	-	-	-	-	-	-	-	-	-	-	-	-	-	-	-	-	-	-	-	-	-	-	-
		-	-	-	-	-	-	-	AT1G05205.1	-	-	-	-	194234	-	-	-	-	-	-	-	-	-	-	-	-	-	-	-	-	-	Os06g46700.1	37009	-	-	-	-	-	578604	-	-	-	-	-	-	-	-	-	-	-	-	-
		-	-	-	-	-	-	-	-	-	-	-	-	194231	-	-	-	-	-	-	-	-	-	-	-	-	-	-	-	-	-	-	3920	-	-	-	-	-	-	-	-	-	-	-	-	-	-	-	-	-	-	-
		-	-	-	-	-	-	-	-	-	-	-	-	194230	-	-	-	-	-	-	-	-	-	-	-	-	-	-	-	-	-	-	-	-	-	-	-	-	-	-	-	-	-	-	-	-	-	-	-	-	-	-
		-	-	-	-	-	-	-	-	-	-	-	-	194227	-	-	-	-	-	-	-	-	-	-	-	-	-	-	-	-	-	-	-	-	-	-	-	-	-	-	-	-	-	-	-	-	-	-	-	-	-	-
	glutathione S-transferase	-	-	-	-	-	-	-	-	-	-	-	21681	194226	-	-	-	-	-	-	-	-	-	-	OTTHUMP00000174659	-	237303	-	-	-	-	-	-	-	-	-	-	PF14_0187	-	-	-	-	-	-	-	-	-	-	-	-	-	-
		-	-	-	-	-	-	-	-	-	-	-	-	194225	-	-	-	-	-	-	-	-	-	-	-	-	-	-	-	-	-	-	-	-	-	-	-	-	-	-	-	-	-	-	-	-	-	-	-	-	-	-
FAP179	Flagellar Associated Protein	-	+	-	+	-	-	-	-	-	-	CE00302	-	194224	392965	-	-	OTTDARP00000020804	DDB0217453	-	-	-	-	-	-	-	-	-	72682	184426	-	-	-	GSPATP00032455001	-	-	-	-	-	-	-	-	-	SINFRUP00000148010	98.m00153	268449	-	-	-	-	-	-
		-	+	-	-	-	-	GB16824-PA	AT1G19880.1	71948	BDEG_07428	-	168471	194222	209202	-	CMI054C	-	-	CG9135-PB	-	-	ENSGALP00000039872	-	OTTHUMP00000002613	-	213485	-	-	179224	NCU02267	Os04g56720.1	14346	-	50520	114453	-	-	831668	-	-	-	-	SINFRUP00000128244	-	264699	-	-	-	-	-	UM04365.1
		-	-	-	-	-	-	-	-	-	-	-	-	194221	-	-	-	-	-	-	-	-	-	-	-	-	-	-	-	-	-	-	-	-	-	-	-	-	-	-	-	-	-	-	-	-	-	-	-	-	-	-
		-	-	-	-	-	-	-	-	-	-	-	-	194220	-	-	-	-	-	-	-	-	-	-	-	-	-	-	-	-	-	-	-	-	-	-	-	-	-	-	-	-	-	-	-	-	-	-	-	-	-	-
		-	-	-	-	-	-	-	-	-	-	-	-	194219	-	-	-	-	-	-	-	-	-	-	-	-	-	-	-	-	-	-	-	-	46332	-	-	-	-	-	-	-	-	-	-	-	-	-	-	-	-	-
		-	+	-	-	-	-	-	-	-	-	CE20024	-	194218	-	-	-	-	-	-	-	-	ENSGALP00000026998	-	NP_004018.1	-	-	18386	-	-	-	-	-	-	-	-	139152	-	-	-	-	-	-	-	-	-	-	-	-	-	-	-
		-	-	-	-	-	-	-	-	-	-	-	-	194214	-	-	-	-	-	-	-	-	-	-	-	-	-	-	-	-	-	-	-	-	-	-	-	-	-	RO3G_11499.1	-	-	-	-	-	-	-	-	-	-	-	-
EB1		-	+	-	-	-	-	GB19881-PA	AT5G67270.1	7035	BDEG_07433	CE26217	180168	194209	397555	cgd7_2370	CMJ156C	OTTDARP00000024111	DDB0218515	CG32371-PA	19068506	-	ENSGALP00000010754	GL50803_14048	OTTHUMP00000122582	-	177133	32858	44546	196354	NCU00243	Os10g35580.1	5403	GSPATP00027670001	54306	52398	108686	PFC0305w	208732	RO3G_14394.1	YER016W	SPAC18G6.15	GLEAN3_27631	SINFRUP00000143556	8.m00318	36137	TA18025	42.m03579	57922	89135.m00247	Tb09.160.1440	UM05761.1
		-	-	-	-	-	-	-	-	-	-	-	-	194202	-	-	-	-	-	-	-	-	-	-	-	-	-	-	-	-	-	-	-	-	-	-	-	-	-	-	-	-	-	-	-	-	-	-	-	-	-	-
		-	-	-	-	-	-	-	-	-	-	-	-	194201	-	-	-	-	-	-	-	-	-	-	-	-	-	-	-	-	-	-	-	-	-	-	-	-	-	-	-	-	-	-	-	-	-	-	-	-	-	-
		-	-	-	-	-	-	-	-	-	-	-	-	194199	-	-	-	-	-	-	-	-	-	-	-	-	-	-	-	-	-	-	-	-	-	-	-	-	-	-	-	-	-	-	-	-	TA06445	-	-	-	-	-
		-	-	-	-	-	-	-	-	-	-	-	-	194198	-	-	-	-	-	-	-	-	-	-	-	-	-	-	-	-	-	-	-	-	-	-	-	-	-	-	-	-	-	-	-	-	-	-	-	-	-	-
		-	-	-	-	-	-	-	-	-	-	-	-	194197	-	-	-	-	-	-	-	-	-	-	-	-	-	-	-	-	-	-	-	-	-	-	-	-	-	-	-	-	-	-	-	-	-	-	-	-	-	-
		-	-	-	-	-	-	-	-	-	-	-	-	194196	-	-	-	-	-	-	-	-	-	-	-	-	-	-	-	-	-	-	-	-	-	-	-	-	-	-	-	-	-	-	-	-	-	-	-	-	-	-
		-	+	-	-	-	-	GB19034-PA	AT5G51130.1	-	BDEG_00160	CE19053	150016	194190	273441	-	-	OTTDARP00000019231	DDB0189672	CG1239-PA	-	-	ENSGALP00000040462	-	NP_062552.2	-	109009	-	45643	120055	-	Os08g42760.1	11520	GSPATP00028980001	31665	165914	138148	PF11_0468	261963	RO3G_14293.1	-	SPBC2A9.10	GLEAN3_20929	SINFRUP00000129069	5.m00353	-	-	59.m06106	19869	-	-	UM00784.1

		-	-	-	-	-	-	-	-	-	-	-	-	194188	-	-	-	-	-	-	-	-	-	-	-	-	-	-	-	-	-	-	-	-	-	-	-	-	-	-	-	-	-	-	-	-	-	-	-	-	-	-
		-	-	-	-	-	-	-	-	-	-	-	-	194187	-	-	-	-	-	-	-	-	-	-	-	-	-	-	-	-	-	-	-	-	-	-	-	-	-	-	-	-	-	-	-	-	-	-	-	-	-	-
		-	-	-	-	-	-	-	-	-	-	-	-	194186	-	-	-	-	-	-	-	-	-	-	-	-	-	-	-	-	-	-	-	-	-	-	-	-	-	-	-	-	-	-	-	-	-	-	-	-	-	-
		-	-	-	-	-	-	-	-	-	-	-	-	194184	-	-	-	-	-	-	-	-	-	-	-	-	-	-	-	-	-	-	-	-	-	-	-	-	-	-	-	-	-	-	-	-	-	-	-	-	-	-
ATP3	F1F0 ATP synthase, gamma subunit, mitochondrial	-	+	-	+	-	-	GB15291-PA	AT2G33040.1	53834	BDEG_07357	CE27514	173744	194183	201047	-	CML029C	OTTDARP00000008159	DDB0184316	-	-	-	ENSGALP00000010899	-	OTTHUMP00000019068	LmjF21.1770	235900	28131	-	239595	NCU09119	Os10g17280.1	27351	GSPATP00026399001	18398	148150	109105	PF13_0061	836795	RO3G_06170.1	YBR039W	SPBC1734.13	GLEAN3_00077	SINFRUP00000140728	8.m00440	5026	TA19395	44.m02684	63188	-	Tb10.100.0070	UM05090.1
		-	-	-	-	-	-	-	-	-	-	-	-	194182	-	-	-	-	-	-	-	-	-	-	-	-	-	-	-	-	-	-	-	-	-	-	-	-	-	-	-	-	-	-	-	-	-	-	-	-	-	-
		-	-	-	-	-	-	-	-	-	-	-	-	194180	-	-	-	-	-	-	-	-	-	-	-	-	-	-	-	-	-	-	-	-	-	-	-	-	-	-	-	-	-	-	-	-	-	-	-	-	-	-
		-	-	-	-	-	-	-	-	-	-	-	-	194179	-	-	-	-	-	-	-	-	-	-	-	-	-	-	-	-	-	-	-	-	-	-	-	-	-	-	-	-	-	-	-	-	-	-	-	-	-	-
		-	-	-	-	-	-	-	-	-	-	-	-	194178	-	-	-	-	-	-	-	-	-	-	-	-	-	-	-	-	-	-	-	-	-	-	-	-	-	-	-	-	-	-	-	-	-	-	-	-	-	-
		-	-	-	-	-	-	-	-	-	-	-	-	194169	-	-	-	-	-	-	-	-	-	-	-	-	-	-	-	-	-	-	-	-	-	-	-	-	-	-	-	-	-	-	-	-	-	-	-	-	-	-
		-	+	-	-	-	-	-	AT1G25440.1	-	-	-	-	194168	-	-	CME043C	-	-	-	-	-	-	-	-	-	-	-	-	-	-	Os06g15330.1	-	-	43850	115089	-	-	829714	-	-	-	-	-	-	21967	-	-	-	-	-	-
		-	-	-	-	-	-	-	-	-	-	-	-	194166	-	-	-	-	-	-	-	-	-	-	-	-	-	-	-	-	-	-	-	-	-	-	-	-	-	-	-	-	-	-	-	-	-	-	-	-	-	-
		-	+	-	-	-	-	-	AT1G05270.1	-	-	-	-	194163	273817	-	-	-	DDB0192197	-	-	-	-	-	-	-	-	34519	68501	-	-	-	11085	-	-	172683	-	-	562305	-	-	-	-	-	-	-	-	-	56379	-	-	-
		-	-	-	-	-	-	-	-	-	-	-	-	194161	-	-	-	-	-	-	-	-	-	-	-	-	-	-	-	-	-	-	-	-	-	-	-	-	-	-	-	-	-	-	-	-	-	-	-	-	-	-
FAS2	fasciclin-like protein	-	+	-	-	-	-	-	-	29451	-	-	-	194159	-	-	-	-	-	-	-	-	ENSGALP00000035686	-	OTTHUMP00000018271	-	-	28185	-	-	-	-	34481	-	54395	60312	-	-	730906	RO3G_14315.1	-	-	GLEAN3_06345	SINFRUP00000144260	-	25610	-	-	-	-	-	UM04719.1
		-	-	-	-	-	-	-	-	-	-	-	-	194153	-	-	-	-	-	-	-	-	-	-	-	-	-	-	-	-	-	-	-	-	-	-	-	-	-	-	-	-	-	-	-	-	-	-	-	-	-	-
		-	-	-	-	-	-	-	-	-	-	-	-	194149	-	-	-	-	-	-	-	-	-	-	-	-	-	-	-	-	-	-	-	-	-	-	-	-	-	-	-	-	-	-	-	-	-	-	-	-	-	-
AK3	Adenylate Kinase Homolog 3	-	+	-	-	-	-	GB17392-PA	AT5G47840.1	21818	BDEG_07977	-	5941	194134	-	-	CMT571C	-	-	-	-	-	-	GL50803_28234	-	LmjF21.1250	195378	-	59535	-	-	Os08g01770.1	34121	-	12239	115656	132550	-	262851	-	-	-	-	-	-	9511	-	-	53152	-	Tb10.70.7330	-
FAP31	Conserved Uncharacterized Flagellar Associated Protein	-	+	-	+	-	-	-	-	11487	-	CE36917	219276	194130	247515	-	-	-	DDB0219761	-	-	-	ENSGALP00000019105	-	OTTHUMP00000172839	-	-	25293	-	244876	NCU07463	-	37184	-	-	127004	-	-	574726	-	-	-	GLEAN3_18898	SINFRUP00000157262	-	262535	-	-	53679	-	-	-
		-	-	-	-	-	-	-	-	-	-	-	-	194128	-	-	-	-	-	-	-	-	-	-	-	-	-	-	-	-	-	-	-	-	-	-	-	-	-	-	-	-	-	-	-	-	-	-	-	-	-	-
		-	-	-	-	-	-	-	-	-	-	-	-	194127	-	-	-	-	-	-	-	-	-	-	-	-	-	-	-	-	-	-	-	-	-	-	-	-	-	-	-	-	-	-	-	-	-	-	-	-	-	-
		-	-	-	-	-	-	-	-	-	-	-	-	194126	-	-	-	-	-	-	-	-	-	-	-	-	-	-	-	-	-	-	-	-	-	-	-	-	-	-	-	-	-	-	-	-	-	-	-	-	-	-
		-	-	-	-	-	-	-	-	-	-	-	-	194124	-	-	-	-	-	-	-	-	-	-	-	-	-	-	-	-	-	-	-	-	-	-	-	-	-	-	-	-	-	-	-	-	-	-	-	-	-	-
		-	-	-	-	-	-	-	-	-	-	-	-	194119	-	-	-	-	-	-	-	-	-	-	-	-	-	-	-	-	-	-	-	-	-	-	-	-	-	-	-	-	-	-	-	-	-	-	-	-	-	-
		-	-	-	-	-	-	-	-	-	-	-	-	194117	-	-	-	-	-	-	-	-	-	-	-	-	-	-	-	-	-	-	24508	-	-	-	-	-	-	-	-	-	-	-	-	-	-	-	-	-	-	-
		-	-	-	-	-	-	-	-	-	-	-	-	194114	-	-	-	-	-	-	-	-	-	-	-	-	-	-	-	-	-	-	-	-	-	-	-	-	-	-	-	-	-	-	-	-	-	-	-	-	-	-
FAP115	Flagellar Associated Protein	+	+	-	+	-	-	GB19267-PA	-	66672	BDEG_03650	-	182794	194105	241937	-	-	-	-	-	-	-	-	-	-	-	-	32704	59413	216927	-	-	-	GSPATP00028939001	-	79594	131593	-	-	-	-	-	-	-	16.m00315	-	-	-	24458	107665.m00013	Tb11.02.4320	-
		-	+	-	-	-	-	GB18420-PA	AT1G61620.1	20970	BDEG_01862	CE07417	223572	194103	226342	-	-	-	DDB0216662	CG6179-PA	-	-	-	-	OTTHUMP00000070006	-	196769	18592	-	243516	-	Os03g17170.1	33251	GSPATP00019788001	46175	196206	133485	PFE0900w	800358	-	-	-	-	SINFRUP00000182128	26.m00225	21910	TA07555	65.m01175	22121	-	-	-
		-	-	-	-	-	-	-	-	-	-	-	-	194098	-	-	-	-	-	-	-	-	-	-	-	-	-	-	-	-	-	-	-	-	-	-	-	-	-	-	-	-	-	-	-	-	-	-	-	-	-	-
		-	-	-	-	-	-	-	-	-	-	-	-	194097	-	-	-	-	-	-	-	-	-	-	-	-	-	-	-	-	-	-	-	-	-	-	-	-	-	-	-	-	-	-	-	-	-	-	-	-	-	-
		-	-	-	-	-	-	-	-	-	-	-	-	194096	-	-	-	-	-	-	-	-	-	-	-	-	-	-	-	-	-	-	-	-	-	-	-	-	-	-	-	-	-	-	-	-	-	-	-	-	-	-
		-	-	-	-	-	-	-	-	-	-	-	-	194094	-	-	-	-	-	-	-	-	-	-	-	-	-	-	-	-	-	-	-	-	-	-	-	-	-	-	-	-	-	-	-	-	-	-	-	-	-	-
DHC9	Dynein heavy chain 9	-	-	-	+	-	-	-	-	-	-	-	-	194093	-	-	-	-	-	-	-	-	-	-	-	-	-	-	-	-	-	-	-	-	-	-	-	-	-	-	-	-	-	-	-	-	-	-	-	-	-	-
		-	-	-	-	-	-	-	-	-	-	-	-	194086	-	-	-	-	-	-	-	-	-	-	-	-	-	-	-	-	-	-	-	-	-	-	-	-	-	-	-	-	-	-	-	-	-	-	-	-	-	-
		-	-	-	-	-	-	-	-	-	-	-	-	194085	-	-	-	-	-	-	-	-	-	-	-	-	-	-	-	-	-	-	-	-	-	-	-	-	-	-	-	-	-	-	-	-	-	-	-	-	-	-
		+	+	-	-	-	-	-	-	23783	-	-	106738	194082	274486	-	-	-	-	-	-	-	ENSGALP00000037901	-	NP_036356.1	LmjF21.1565	144675	29172	70275	79942	-	-	-	-	-	-	119629	-	-	-	-	-	GLEAN3_19021	SINFRUP00000130608	52.m00213	-	-	641.m02549	25231	93148.m00098	Tb10.70.7860	-
		-	-	-	-	-	-	-	-	-	-	-	-	194078	-	-	-	-	-	-	-	-	-	-	-	-	-	-	-	-	-	-	-	-	-	-	-	-	-	-	-	-	-	-	-	-	-	-	-	-	-	-
		-	-	-	-	-	-	-	-	-	-	-	106683	194076	-	-	-	-	-	-	-	-	-	-	-	-	-	-	-	-	-	-	-	-	-	-	-	-	-	-	-	-	-	-	-	-	-	-	-	-	-	-
		-	-	-	-	-	-	-	-	-	-	-	-	194075	-	-	-	-	-	-	-	-	-	-	-	-	-	-	-	-	-	-	-	-	-	-	-	-	-	-	-	-	-	-	-	-	-	-	-	-	-	-
ACS1	Acetyl CoA synthetase	-	+	-	-	-	-	-	-	53446	BDEG_00471	CE03076	-	194063	288911	-	CMK098C	OTTDARP00000024060	-	-	-	112.m00132	-	-	OTTHUMP00000030713	LmjF23.0710	128665	-	-	-	NCU06836	-	-	-	-	-	-	-	-	-	YLR153C	SPCC191.02c	GLEAN3_15048	-	72.m00172	33454	-	57.m03124	-	95287.m00273	-	UM03069.1
		-	-	-	-	-	-	-	-	-	-	-	-	194060	-	-	-	-	-	-	-	-	-	-	-	-	-	-	-	-	-	-	-	-	-	-	-	-	-	-	-	-	-	-	-	-	-	-	-	-	-	-
		-	-	-	-	-	-	-	-	-	-	-	-	194059	-	-	-	-	-	-	-	-	-	-	-	-	171689	-	-	-	-	-	-	-	-	-	-	-	-	-	-	-	-	-	-	-	-	-	-	-	-	-
		-	+	-	-	-	-	-	AT1G54170.1	64466	BDEG_07682	-	190227	194033	-	cgd7_3100	CMK070C	OTTDARP00000018859	DDB0190466	-	-	-	-	-	-	LmjF10.1110	228257	28426	78066	-	NCU05400	Os02g27950.2	10844	-	44892	172528	128866	PF14_0338	756757	RO3G_09416.1	YGR178C	SPBC21B10.03c	-	-	-	23386	-	44.m02662	-	-	-	UM02637.1
		-	-	-	-	-	-	-	-	-	-	-	-	194032	-	-	-	-	-	-	-	-	-	-	-	-	-	-	-	-	-	-	-	-	-	-	-	-	-	-	-	-	-	-	-	-	-	-	-	-	-	-
		-	+	-	-	-	-	-	AT5G04740.1	-	-	-	-	194028	-	-	-	-	-	-	-	-	-	-	-	-	-	-	-	-	-	Os08g14440.1	22291	-	-	118464	-	-	420126	-	-	-	-	-	-	-	-	-	-	-	-	-
		-	-	-	-	-	-	-	-	-	-	-	-	194027	-	-	-	-	-	-	-	-	-	-	-	-	-	-	-	-	-	-	-	-	-	-	-	-	-	-	-	-	-	-	-	-	-	-	-	-	-	-
DJ1	DJ-1 family protein	-	+	+	-	-	-	GB15202-PA	AT4G34020.1	29730	BDEG_07033	CE07740	155674	194026	370745	cgd5_1650	CMS007C	-	DDB0186988	CG6646-PA	-	1.m00756	ENSGALP00000000741	GL50803_9088	OTTHUMP00000001349	LmjF30.0750	223821	8547	67604	190290	-	Os06g34040.1	19940	GSPATP00005847001	32791	57347	142787	PFF1335c	201728	RO3G_06344.1	-	SPAC22E12.03c	GLEAN3_13608	SINFRUP00000147408	66.m00196	31785	TA04310	33.m02668	-	88289.m00176	Tb927.6.2200	-
		-	-	-	-	-	-	-	-	-	-	-	-	194023	-	-	-	-	-	-	-	-	-	-	-	-	-	-	-	-	-	-	35595	-	-	-	-	-	-	-	-	-	-	-	-	-	-	-	-	-	-	-
		-	-	-	-	-	-	-	-	-	-	-	-	194015	-	-	-	-	-	-	-	-	-	-	-	-	-	-	-	-	-	-	-	-	-	-	-	-	-	-	-	-	-	-	-	-	-	-	-	-	-	-
		-	+	-	-	-	-	GB10168-PA	AT4G00850.1	-	-	-	105352	194013	368867	-	-	-	-	-	-	-	ENSGALP00000024351	-	OTTHUMP00000031468	-	114542	-	-	244000	-	Os11g40100.1	33727	-	-	64084	-	-	644637	-	-	-	-	SINFRUP00000175726	-	-	-	-	-	90610.m00143	-	-
		-	+	-	-	-	-	GB18879-PA	AT5G60190.1	-	BDEG_06520	CE33875	108145	194011	275418	-	-	-	DDB0206197	CG8493-PB	-	-	-	-	NP_660205.2	-	182489	-	61576	238848	NCU00734	Os12g01180.2	-	-	44664	168150	140389	-	570658	-	-	SPBC17D11.01	-	SINFRUP00000129016	-	-	-	-	52470	88546.m00293	-	UM03372.1
		-	-	-	-	-	-	-	-	-	-	-	-	194008	-	-	-	-	-	-	-	-	-	-	-	-	-	-	-	-	-	-	-	-	-	-	-	-	-	-	-	-	-	-	-	-	-	-	-	-	-	-
		-	-	-	-	-	-	-	-	-	-	-	-	194007	-	-	-	-	-	-	-	-	-	-	-	-	-	-	-	-	-	-	-	-	-	-	-	-	-	-	-	-	-	-	-	-	-	-	-	-	-	-
		-	-	-	-	-	-	-	-	-	-	-	-	194006	-	-	-	-	-	-	-	-	-	-	-	-	-	-	-	109724	-	-	-	-	-	-	-	-	-	-	-	-	-	-	-	-	-	-	-	-	-	-
		-	+	-	-	-	-	-	-	-	-	-	82870	194003	-	-	-	-	DDB0184163	-	-	-	ENSGALP00000016627	-	-	-	-	-	-	234280	NCU00984	-	-	GSPATP00018072001	-	-	-	-	-	-	-	-	GLEAN3_09279	SINFRUP00000182703	137.m00120	-	-	-	-	82952.m00157	-	-
		-	-	-	-	-	-	-	-	-	-	-	-	194002	-	-	-	-	-	-	-	-	-	-	-	-	-	-	-	-	-	-	-	-	-	-	-	-	-	-	-	-	-	-	-	-	-	-	-	-	-	-
		-	-	-	-	-	-	-	-	-	-	-	-	193999	-	-	-	-	-	-	-	-	-	-	-	-	-	-	-	-	-	-	-	-	-	-	-	-	-	-	-	-	-	-	-	-	-	-	-	-	-	-
		-	-	-	-	-	-	-	-	-	-	-	-	193998	-	-	-	-	-	-	-	-	-	-	-	-	-	-	-	-	-	-	-	-	-	-	-	-	-	-	-	-	-	-	-	-	-	-	-	-	-	-
		-	-	-	-	-	-	-	-	-	BDEG_00204	-	-	193995	-	-	-	-	-	-	-	-	-	-	-	-	-	-	-	-	-	-	-	-	-	-	-	-	206728	-	-	-	-	-	-	-	-	-	-	-	-	-
		-	+	-	-	-	-	-	-	-	BDEG_03294	-	225652	193992	-	-	-	-	-	-	-	-	ENSGALP00000037410	-	OTTHUMP00000164146	-	-	24942	-	238058	-	-	35116	-	-	-	-	-	-	-	-	-	-	-	-	-	-	-	-	-	-	-
		-	-	-	-	-	-	-	-	-	-	-	-	193991	-	-	-	-	-	-	-	-	-	-	-	-	-	-	-	-	-	-	-	-	-	-	-	-	-	-	-	-	-	-	-	-	-	-	-	-	-	-
		-	-	-	-	-	-	-	-	-	-	-	-	193990	-	-	-	-	-	-	-	-	-	-	-	-	-	-	-	-	-	-	-	-	-	-	-	-	-	-	-	-	-	-	-	-	-	-	-	-	-	-
		-	-	-	-	-	-	-	-	-	-	-	-	193979	-	-	-	-	-	-	-	-	-	-	-	-	-	-	-	-	-	-	-	-	-	-	-	-	-	-	-	-	-	-	-	-	-	-	-	-	-	-
LST8	WD-40 containing protein	-	+	+	-	-	-	GB19722-PA	AT3G18140.1	34399	BDEG_06132	CE08097	165576	193970	-	-	CMH260C	-	DDB0184464	CG3004-PA	-	1004.m00013	ENSGALP00000009432	GL50803_17353	OTTHUMP00000159044	-	212084	34029	31385	141896	NCU04281	Os03g47780.1	11363	GSPATP00026515001	36142	167168	109544	-	667428	RO3G_06076.1	YNL006W	SPBC21B10.05c	-	SINFRUP00000141791	12.m00280	4260	-	-	21796	88271.m00399	-	UM03059.1
		-	-	-	-	-	-	-	-	-	-	-	-	193962	-	-	-	-	-	-	-	-	-	-	-	-	-	-	-	-	-	-	-	-	-	-	-	-	-	-	-	-	-	-	-	-	-	-	-	-	-	-
ISG-C3	hydroxyproline-rich cell wall protein	-	+	-	-	-	-	-	AT4G24930.1	-	-	-	-	193961	-	-	CMI167C	-	-	-	-	-	-	-	-	-	-	-	-	-	-	Os01g01280.1	33137	-	-	82453	-	-	575370	-	-	-	-	-	-	-	-	-	-	-	-	-
		-	-	-	-	-	-	-	-	-	-	-	-	193960	-	-	-	-	-	-	-	-	-	-	-	-	-	-	-	-	-	-	-	-	-	-	-	-	-	-	-	-	-	-	-	-	-	-	-	-	-	-
		-	-	-	-	-	-	-	AT5G17510.1	-	-	-	-	193956	-	-	-	-	-	-	-	-	-	-	-	-	-	-	-	-	-	Os12g27994.1	-	-	-	72978	-	-	-	-	-	-	-	-	-	-	-	-	-	-	-	-
		-	-	-	-	-	-	-	-	-	-	-	-	193951	-	-	-	-	-	-	-	-	-	-	-	-	-	-	62377	-	-	-	-	-	-	-	-	-	-	-	-	-	-	-	-	-	-	-	-	-	-	-
		-	-	-	-	-	-	-	-	-	-	-	-	193943	-	-	-	-	-	-	-	-	-	-	-	-	-	-	-	-	-	-	-	-	-	-	-	-	-	-	-	-	-	-	-	-	-	-	-	-	-	-
		-	+	-	-	-	-	-	AT1G18660.1	-	-	-	197542	193940	274289	-	CMO233C	-	-	-	-	-	ENSGALP00000006474	-	NP_940863.3	-	125925	-	-	106903	-	-	-	-	-	-	-	-	772889	RO3G_11464.1	-	-	-	SINFRUP00000152782	-	-	-	-	-	-	-	UM04947.1
		-	-	-	-	-	-	-	-	-	-	-	-	193937	-	-	-	-	-	-	-	-	-	-	-	-	-	-	-	-	-	-	-	-	-	-	-	-	-	-	-	-	-	-	-	-	-	-	-	-	-	-
		-	-	-	-	-	-	-	-	-	-	-	-	193924	-	-	-	-	-	-	-	-	-	-	-	-	-	-	-	-	-	-	-	-	-	-	-	-	-	-	-	-	-	-	-	-	-	-	-	-	-	-
		-	+	-	-	-	-	-	-	-	-	CE27109	217363	193923	-	-	-	-	-	-	-	-	ENSGALP00000027775	-	OTTHUMP00000161574	-	-	-	-	95565	-	-	-	-	50026	-	139077	-	597397	-	-	-	GLEAN3_09628	SINFRUP00000140639	-	-	-	-	-	-	-	-
		-	-	-	-	-	-	-	-	-	-	-	-	193922	-	-	-	-	-	-	-	-	-	-	-	-	-	-	-	-	-	-	-	-	-	-	-	-	-	-	-	-	-	-	-	-	-	-	-	-	-	-
		-	+	-	-	-	-	-	-	-	-	-	-	193918	-	-	-	-	-	-	-	-	-	-	-	-	-	-	1291	-	-	-	37346	GSPATP00021542001	-	-	-	-	-	-	-	SPAC11D3.06	-	-	-	1083	-	-	-	-	-	-
		-	-	-	-	-	-	-	-	-	-	-	-	193915	297591	-	-	-	-	-	-	-	-	-	-	LmjF33.0860	-	-	-	128899	-	-	-	-	-	-	-	-	-	-	-	-	-	-	-	-	-	-	-	-	-	-
		-	-	-	-	-	-	-	-	-	-	-	-	193914	-	-	-	-	-	-	-	-	-	-	-	-	-	-	-	-	-	-	-	-	-	-	-	-	-	-	-	-	-	-	-	-	-	-	-	-	-	-
		-	-	-	-	-	-	-	-	-	-	-	-	193913	-	-	-	-	-	-	-	-	-	-	-	-	-	-	-	-	-	-	-	-	-	-	-	-	-	-	-	-	-	-	-	-	-	-	-	-	-	-
		-	-	-	-	-	-	-	-	-	-	-	-	193906	-	-	-	-	-	-	-	-	-	-	-	-	-	-	-	-	-	-	-	-	-	-	-	-	-	-	-	-	-	-	-	-	-	-	-	-	-	-
		-	-	-	-	-	-	-	-	-	-	-	-	193905	-	-	-	-	-	-	-	-	-	-	-	-	-	-	-	-	-	-	-	-	-	-	-	-	-	-	-	-	-	-	-	-	-	-	-	-	-	-
		-	-	-	-	-	-	-	-	-	-	-	-	193904	-	-	-	-	-	-	-	-	-	-	-	-	-	-	-	-	-	-	-	-	-	-	-	-	-	-	-	-	-	-	-	-	-	-	-	-	-	-
MAPK8	Mitogen-Activated Protein Kinase Homolog 8	-	+	-	-	-	-	GB15499-PA	AT2G43790.1	20863	BDEG_00153	CE24971	98373	193901	284908	-	CMS295C	-	DDB0186926	-	-	-	ENSGALP00000002278	GL50803_17563	NP_620602.1	LmjF10.0490	231535	-	31331	-	NCU02393	Os06g06090.1	19271	GSPATP00001183001	21410	-	129405	-	718188	RO3G_01456.1	YHR030C	SPAC31G5.09c	GLEAN3_19715	SINFRUP00000166270	16.m00440	5018	-	-	814	88834.m00149	-	UM03305.1
		-	+	-	-	-	-	GB10472-PA	AT2G41670.1	27328	BDEG_05032	-	136843	193900	391713	-	-	-	DDB0202331	-	-	-	ENSGALP00000005605	-	OTTHUMP00000020814	LmjF35.5210	204918	-	68025	-	-	Os11g26030.1	-	GSPATP00002127001	-	183799	108367	-	560484	-	-	-	GLEAN3_00205	SINFRUP00000151626	25.m00424	-	TA11925	38.m01064	58045	-	Tb09.211.0810	UM00788.1
		-	-	-	-	-	-	-	-	-	-	-	-	193899	-	-	-	-	-	-	-	-	-	-	-	-	-	-	-	-	-	-	-	-	-	-	-	-	-	-	-	-	-	-	-	-	-	-	-	-	-	-
		-	-	-	-	-	-	-	AT5G40740.1	-	-	-	-	193898	-	-	-	-	-	-	-	-	-	-	-	-	-	-	-	-	-	Os02g22370.3	-	-	-	95807	-	-	581731	-	-	-	-	-	-	-	-	-	-	-	-	-
		-	-	-	-	-	-	-	-	-	-	-	-	193897	-	-	-	-	-	-	-	-	-	-	-	-	-	-	-	-	-	-	-	-	-	168769	-	-	-	-	-	-	-	-	-	-	-	-	-	-	-	-
		-	-	-	-	-	-	-	-	-	-	-	-	193895	-	-	-	-	-	-	-	-	-	-	-	-	-	-	-	-	-	-	36994	GSPATP00021215001	-	-	-	-	-	-	-	-	-	-	10.m00304	-	-	-	-	-	-	-
		-	-	-	-	-	-	-	-	-	-	-	-	193892	-	-	-	-	-	-	-	-	-	-	-	-	-	-	-	-	-	-	-	-	-	-	-	-	-	-	-	-	-	SINFRUP00000155477	-	-	-	-	-	-	-	-
		-	-	-	-	-	-	-	-	-	-	-	-	193884	-	-	-	-	-	-	-	-	-	-	-	-	-	-	-	-	-	-	-	-	-	-	-	-	-	-	-	-	-	-	-	-	-	-	-	-	-	-
		-	-	-	-	-	-	-	-	-	-	-	-	193882	-	-	-	-	-	-	-	-	-	-	-	-	-	-	-	-	-	-	-	-	-	-	-	-	-	-	-	-	-	-	-	-	-	-	-	-	-	-
		-	-	-	-	-	-	-	-	-	-	-	-	193881	-	-	-	-	-	-	-	-	-	-	-	-	-	-	-	-	-	-	-	-	-	-	-	-	-	-	-	-	-	-	-	-	-	-	-	-	-	-
		-	-	-	-	-	-	-	-	-	-	-	-	193878	-	-	-	-	-	-	-	-	-	-	-	-	-	-	-	-	-	-	-	-	-	-	-	-	-	-	-	-	-	-	-	-	-	-	-	-	-	-
	protein of unknown function	-	-	-	-	-	-	-	-	-	-	-	-	193873	-	-	-	-	-	-	-	-	-	-	-	-	-	-	-	-	-	-	-	-	-	-	-	-	-	-	-	-	-	-	-	-	-	-	-	-	-	-
		-	-	-	-	-	-	-	-	70915	-	-	-	193872	-	-	-	-	-	-	-	-	-	-	-	-	-	-	-	-	-	-	13907	-	-	-	-	-	-	-	-	-	-	-	-	-	-	-	-	-	-	-
		-	-	-	-	-	-	-	-	-	-	-	-	193870	-	-	-	-	-	-	-	-	-	-	-	-	-	-	-	-	-	-	-	-	-	-	-	-	-	-	-	-	-	-	-	-	-	-	-	-	-	-
		-	-	-	-	-	-	-	-	-	-	-	-	193868	-	-	-	-	-	-	-	-	-	-	-	-	-	-	-	-	-	-	-	-	-	-	-	-	-	-	-	-	-	-	-	-	-	-	-	-	-	-
		-	-	-	-	-	-	-	-	-	-	-	-	193863	-	-	-	-	-	-	-	-	-	-	-	-	-	-	-	-	-	-	-	-	-	-	-	-	-	-	-	-	-	-	-	-	-	-	-	-	-	-
FKB16-4	peptidyl-prolyl cis-trans isomerase, FKBP-type	-	+	-	-	-	-	-	AT3G10060.1	-	-	-	-	193859	-	-	-	-	-	-	-	-	-	-	-	-	-	-	-	-	-	Os07g09040.1	37519	-	-	26617	-	-	825269	-	-	-	-	-	-	-	-	-	-	-	-	-
		-	-	-	-	-	-	-	-	-	-	-	-	193851	-	-	-	-	-	-	-	-	-	-	-	-	-	-	-	-	-	-	-	-	-	-	-	-	-	-	-	-	-	-	-	-	-	-	-	-	-	-
		-	-	-	-	-	-	-	-	-	-	-	-	193848	-	-	-	-	-	-	-	-	-	-	-	-	-	-	-	-	-	-	-	-	-	-	-	-	-	-	-	-	-	-	-	-	-	-	-	-	-	-
PSAO	Photosystem I subunit O	-	+	-	-	-	-	-	AT1G08380.1	-	-	-	-	193847	-	-	CMP086C	-	-	-	-	-	-	-	-	-	-	-	-	-	-	Os04g33830.1	15550	-	-	108312	-	-	648969	-	-	-	-	-	-	-	-	-	-	-	-	-
		-	-	-	-	-	-	-	AT2G17240.1	-	-	-	-	193846	-	-	-	-	-	-	-	-	-	-	-	-	-	-	-	-	-	Os02g40040.1	-	-	-	56105	-	-	825841	-	-	-	-	-	-	-	-	-	-	-	-	-
IMP,IMP1	IMP1, inner membrane peptidase, mitochondrial	-	+	-	-	-	-	GB14463-PA	AT3G54826.1	17910	BDEG_01227	CE23764	70323	193843	258380	cgd5_3660	CMT595C	-	DDB0191660	CG12379-PA	-	-	ENSGALP00000002912	-	OTTHUMP00000022587	LmjF25.0800	140355	29574	-	95829	NCU02499	Os02g57430.1	30269	GSPATP00002088001	37590	127798	127526	PF14_0197	419633	RO3G_11646.1	YNL310C	SPAC24H6.02c	-	SINFRUP00000128557	63.m00156	-	TA20720	-	17997	-	Tb927.3.2300	UM00012.1
		-	-	-	-	-	-	-	-	-	-	-	-	193841	-	-	-	-	-	-	-	-	-	-	-	-	-	-	-	-	-	-	-	-	-	-	-	-	-	-	-	-	-	-	-	-	-	-	-	-	-	-
		-	-	-	-	-	-	-	-	-	-	-	-	193836	-	-	-	-	-	-	-	-	-	-	-	-	-	-	-	-	-	-	-	-	-	-	-	-	-	-	-	-	-	-	-	-	-	-	-	-	-	-
		-	-	-	-	-	-	-	-	-	-	-	-	193834	-	-	-	-	-	-	-	-	-	-	-	-	-	-	-	-	-	-	-	-	-	-	-	-	-	-	-	-	-	-	-	-	-	-	-	-	-	-
		-	+	-	-	-	-	GB17955-PA	AT1G71696.2	28590	-	CE09156	132113	193827	251407	-	CMH174C	OTTDARP00000021248	DDB0219394	CG4678-PB	-	-	ENSGALP00000006827	-	OTTHUMP00000020269	-	52083	27454	36346	836	-	Os06g05240.1	2867	-	-	22136	-	-	761088	-	-	-	GLEAN3_21609	SINFRUP00000167030	-	-	-	-	36680	-	-	-
		-	-	-	-	-	-	-	-	-	-	-	-	193823	-	-	-	-	-	-	-	-	-	-	-	-	-	-	-	-	-	-	-	-	-	-	-	-	-	-	-	-	-	-	-	-	-	-	-	-	-	-
		-	-	-	-	-	-	-	-	-	-	-	-	193822	-	-	-	-	-	-	-	-	-	-	-	-	-	-	-	-	-	-	31685	-	-	-	-	-	-	-	-	-	-	-	-	-	-	-	-	-	-	-
		-	-	-	-	-	-	-	-	-	-	-	-	193821	-	-	-	-	-	-	-	-	-	-	-	-	-	-	-	-	-	-	-	-	-	-	-	-	-	-	-	-	-	-	-	-	-	-	-	-	-	-
		-	-	-	-	-	-	-	-	-	-	-	-	193820	-	-	-	-	-	-	-	-	-	-	-	-	-	-	-	-	-	-	-	-	-	-	-	-	-	-	-	-	-	-	-	-	-	-	-	-	-	-
		-	-	-	-	-	-	-	-	-	-	-	-	193815	-	-	-	-	-	-	-	-	-	-	-	-	-	-	-	-	-	-	-	-	-	-	-	-	-	-	-	-	-	-	-	-	-	-	-	-	-	-
PDC1	mitochondrial pyruvate dehydrogenase complex, E1 component, alpha subunit	-	-	-	-	-	-	GB10779-PA	-	-	-	-	-	193810	-	-	-	-	-	-	-	-	-	-	-	-	-	-	-	-	NCU06482	-	-	-	-	-	-	-	657555	-	-	-	-	-	-	-	-	-	-	-	-	-
		-	-	-	-	-	-	-	-	-	-	-	-	193807	-	-	-	-	-	-	-	-	-	-	-	-	-	-	-	-	-	-	-	-	-	-	-	-	-	-	-	-	-	-	-	-	-	-	-	-	-	-
		-	-	-	-	-	-	-	-	-	-	-	-	193801	-	-	-	-	-	-	-	-	-	-	-	-	-	-	-	-	-	-	-	-	-	-	-	-	-	-	-	-	-	-	-	-	-	-	-	-	-	-
		-	-	-	-	-	-	-	-	-	-	-	-	193794	-	-	-	-	-	-	-	-	-	-	-	-	-	-	-	-	-	-	-	-	-	-	-	-	-	-	-	-	-	-	-	-	-	-	-	-	-	-
		-	-	-	-	-	-	-	-	-	-	-	-	193791	-	-	-	-	-	-	-	-	-	-	-	-	-	-	-	-	-	-	-	-	-	-	-	-	-	-	-	-	-	-	-	-	-	-	-	-	-	-
		-	+	-	-	-	-	-	AT3G60810.1	-	-	-	-	193790	-	-	CMS204C	-	-	-	-	-	-	-	-	-	-	-	-	-	-	Os03g64140.1	6492	-	47157	114376	-	-	816564	-	-	-	-	-	-	1761	-	-	-	-	-	-
		-	+	-	-	-	-	GB13071-PA	AT5G50400.1	62931	-	CE40042	180941	193786	-	-	-	-	DDB0204253	CG1637-PC	-	-	-	-	OTTHUMP00000068328	-	150028	28659	-	227634	NCU09649	Os03g11530.1	15401	GSPATP00008779001	-	111118	129839	-	554997	RO3G_07767.1	-	-	GLEAN3_01489	SINFRUP00000176334	-	-	-	-	54665	-	-	UM02188.1
GAS31	cell wall protein pherophorin GAS31	-	-	-	+	-	-	-	-	-	-	-	-	193780	-	-	-	-	-	-	-	-	-	-	-	-	-	-	-	-	-	-	-	-	-	-	-	-	-	-	-	-	-	-	-	-	-	-	-	-	-	-
		-	-	-	-	-	-	-	-	-	-	-	-	193779	-	-	-	-	-	-	-	-	-	-	-	-	-	-	-	-	-	-	-	-	-	-	-	-	-	-	-	-	-	-	-	-	-	-	-	-	-	-
		-	-	-	-	-	-	-	-	-	-	-	-	193776	-	-	-	-	-	-	-	-	-	-	-	-	-	-	-	-	-	-	-	-	-	-	-	-	-	-	-	-	-	-	-	-	-	-	-	-	-	-
		-	-	-	-	-	-	-	-	-	-	-	-	193772	-	-	-	-	-	-	-	-	-	-	-	-	-	-	-	-	-	-	-	-	-	-	-	-	-	-	-	-	-	-	-	-	-	-	-	-	-	-
		-	-	-	-	-	-	-	-	-	-	-	-	193770	291538	-	-	-	-	-	-	-	-	-	-	-	127454	-	-	-	-	-	-	-	-	-	-	-	-	-	-	-	-	-	128.m00085	-	-	-	-	-	-	-
CYP1_pseudo	cytochrome P450, pseudogene	-	-	-	-	-	-	-	-	-	-	-	-	193769	-	-	-	-	-	-	-	-	-	-	-	-	-	-	-	-	-	-	-	-	-	-	-	-	-	-	-	-	-	-	-	-	-	-	-	-	-	-
NUO13	NADH:ubiquinone oxidoreductase (Complex I) 18 kDa subunit, homolog to bovine B17.2 subunit, mitochondrial	-	+	-	-	-	-	-	AT3G03100.1	-	BDEG_02122	CE39995	164106	193762	368851	-	CMR083C	OTTDARP00000008261	DDB0218742	CG3214-PA	-	-	ENSGALP00000018456	-	OTTHUMP00000168775	-	209731	17089	-	181597	NCU01142	Os10g42840.1	37310	GSPATP00021563001	34392	116021	137365	-	828781	RO3G_04950.1	-	-	GLEAN3_02128	SINFRUP00000159951	-	21599	-	-	56942	-	-	UM04337.1
		-	-	-	-	-	-	-	-	-	-	-	-	193760	-	-	-	-	-	-	-	-	-	-	-	-	-	-	-	-	-	-	-	-	-	-	-	-	-	-	-	-	-	-	-	-	-	-	-	-	-	-
		-	-	-	-	-	-	-	-	-	-	-	-	193757	-	-	-	-	-	-	-	-	-	-	-	-	-	-	-	-	-	-	-	-	-	-	-	-	-	-	-	-	-	-	-	-	-	-	-	-	-	-
YCF39	Putative pyridine nucleotide binding protein	-	+	-	-	-	-	-	AT2G34460.1	19572	-	-	228103	193756	-	-	-	-	-	-	-	-	-	-	-	-	-	-	-	-	-	Os06g25439.1	-	-	10747	209343	-	-	727847	-	YMR090W	-	-	SINFRUP00000161100	-	38608	-	-	-	85972.m00023	-	UM03517.1
		-	+	-	-	-	-	-	AT1G76340.1	25386	-	-	-	193755	-	-	-	-	-	-	-	-	-	-	-	-	-	-	-	-	-	Os03g11590.5	29756	-	-	16335	-	-	230169	-	-	-	-	-	-	-	-	-	-	-	-	-
		-	-	-	-	-	-	-	-	-	-	-	-	193753	-	-	-	-	-	-	-	-	-	-	-	-	-	-	-	-	-	-	-	-	-	-	-	-	-	-	-	-	-	-	-	-	-	-	-	-	-	-
		-	+	-	-	-	-	-	-	-	-	-	-	193752	-	cgd7_2910	-	-	-	-	-	-	-	-	-	-	-	38875	-	-	-	-	30619	-	41071	97751	-	-	-	-	-	-	GLEAN3_00357	-	-	23550	-	-	59780	-	-	UM01201.1
		-	-	-	-	-	-	-	-	-	-	-	-	193748	-	-	-	-	-	-	-	-	-	-	-	-	-	-	-	-	-	-	-	-	-	-	-	-	-	-	-	-	-	-	-	-	-	-	-	-	-	-
		-	-	-	-	-	-	-	-	-	-	-	-	193742	-	-	-	-	-	-	-	-	-	-	-	-	-	-	-	-	-	-	-	-	-	-	-	-	-	-	-	-	-	-	-	-	-	-	-	84285.m00196	-	-
		-	-	-	-	-	-	-	-	-	-	-	-	193736	-	-	-	-	-	-	-	-	-	-	-	-	-	-	-	-	-	-	-	-	-	-	-	-	-	-	-	-	-	-	-	-	-	-	-	-	-	-
		-	-	-	-	-	-	-	AT3G18620.1	-	-	-	-	193733	-	-	-	-	-	-	-	-	-	-	-	-	-	-	-	-	-	-	-	-	-	15937	-	-	270564	-	-	-	-	-	-	-	-	-	-	-	-	-
PGE3401	Polycomb group protein	-	+	-	-	-	-	GB15930-PA	AT3G20740.1	-	-	-	1436	193732	298171	-	CMK173C	-	-	CG5202-PA	19171424	-	ENSGALP00000022724	-	NP_694536.1	-	229029	26014	63593	102199	NCU05300	Os08g04270.3	22117	-	9860	61985	144008	-	834623	-	-	-	GLEAN3_26082	-	-	270065	-	-	31681	-	-	-
ISG-C2	hydroxyproline-rich cell wall protein	-	-	-	-	-	-	-	-	-	-	-	-	193727	-	-	-	-	-	-	-	-	-	-	-	-	-	-	-	-	-	-	-	-	-	-	-	-	-	-	-	-	-	-	-	-	-	-	-	-	-	-
		-	-	-	-	-	-	-	-	-	-	-	-	193726	-	-	-	-	-	-	-	-	-	-	-	-	-	-	-	-	-	-	-	-	-	-	-	-	-	-	-	-	-	-	-	-	-	-	-	-	-	-
		-	-	-	-	-	-	-	-	-	-	-	-	193716	-	-	-	-	-	-	-	-	-	-	-	-	-	-	-	-	-	-	-	-	-	-	-	-	-	-	-	-	-	-	-	-	-	-	-	-	-	-
		-	-	-	-	-	-	-	-	-	-	-	-	193711	-	-	-	-	-	-	-	-	-	-	-	-	-	-	-	-	-	-	8191	-	-	160322	-	-	-	-	-	-	-	-	-	-	-	-	-	-	-	-
		-	-	-	-	-	-	-	-	-	-	-	-	193709	-	-	-	-	-	-	-	-	-	-	-	-	-	-	-	-	-	-	-	-	-	-	-	-	-	-	-	-	-	-	-	-	-	-	-	-	-	-
		-	-	-	-	-	-	-	-	-	-	-	-	193708	-	-	-	-	-	-	-	-	-	-	-	-	-	-	-	-	-	-	-	-	-	-	-	-	-	-	-	-	-	-	-	-	-	-	-	-	-	-
		-	-	-	-	-	-	-	-	-	-	-	-	193706	-	-	-	-	-	-	-	-	-	-	-	-	-	-	-	-	-	-	-	-	-	-	-	-	-	-	-	-	-	-	-	-	-	-	-	-	-	-
		-	-	-	-	-	-	-	AT5G65840.1	-	-	-	-	193704	-	-	-	-	-	-	-	-	-	-	-	-	-	-	-	-	-	-	-	-	-	-	-	-	-	-	-	-	-	-	-	-	-	-	-	-	-	-
		-	-	-	-	-	-	-	-	-	-	-	-	193702	-	-	-	-	-	-	-	-	-	-	-	-	-	-	-	-	-	-	-	-	-	-	-	-	-	-	-	-	-	-	-	-	-	-	-	-	-	-
		-	-	-	-	-	-	-	-	-	-	-	-	193699	-	-	-	-	-	-	-	-	-	-	-	-	-	-	-	-	-	-	-	-	-	-	-	-	-	-	-	-	-	-	-	-	-	-	-	-	-	-
		-	-	-	-	-	-	-	-	-	-	-	-	193694	-	-	-	-	-	-	-	-	-	-	-	-	-	-	-	-	-	-	-	-	-	-	-	-	-	-	-	-	-	-	-	-	-	-	-	-	-	-
		-	-	-	-	-	-	-	-	68492	-	-	-	193691	-	-	-	-	-	-	-	-	-	-	-	-	-	-	-	-	-	-	-	-	-	-	129902	-	-	-	-	-	-	-	-	-	-	-	-	-	-	-
		-	+	-	-	-	-	GB10852-PA	AT4G30820.3	65007	BDEG_07701	CE31957	89450	193689	296604	cgd1_3300	CMS215C	OTTDARP00000024231	DDB0188247	CG7614-PA	-	-	ENSGALP00000019408	-	OTTHUMP00000028071	-	230879	6728	-	241252	NCU02587	Os06g07480.1	35564	-	47392	64452	139992	-	561362	-	YDR460W	SPBC776.18c	-	SINFRUP00000172943	-	-	-	641.m02557	52675	-	-	UM00669.1
EBP3401	BAH domain protein	-	+	-	-	-	-	-	AT4G22140.2	-	-	-	-	193681	234102	-	-	-	DDB0184167	-	-	-	ENSGALP00000002412	-	OTTHUMP00000171526	-	-	12149	-	110445	NCU07505	Os07g08880.2	16045	GSPATP00035076001	-	153027	-	-	556513	RO3G_07458.1	-	SPBC4B4.03	GLEAN3_16588	SINFRUP00000161905	192.m00085	261258	-	-	19587	-	-	UM00443.1
	EMP/nonaspanin domain family	-	+	-	-	-	-	-	AT1G14670.1	55500	-	-	172435	193679	241925	-	-	-	DDB0217951	-	-	-	-	-	OTTHUMP00000028169	-	174735	-	-	89916	-	Os03g13380.1	1565	GSPATP00031489001	13662	193055	157453	-	289338	-	-	-	-	SINFRUP00000150927	16.m00432	263391	-	76.m01551	37943	-	-	-
IF3H		-	+	-	-	-	-	-	AT1G10840.1	53340	BDEG_02962	CE29220	161622	193677	264306	cgd7_5180	CMS354C	-	DDB0204044	CG9124-PB	-	-	ENSGALP00000025924	-	OTTHUMP00000178208	-	181127	9558	50689	168210	NCU07929	Os04g30780.1	36333	-	-	146180	136852	PF14_0191	732129	RO3G_02396.1	-	SPAC821.05	GLEAN3_23965	SINFRUP00000136092	95.m00127	41122	TA12445	50.m03396	37963	-	-	UM00199.1
		+	+	-	-	-	-	-	-	-	BDEG_01778	-	154890	193672	293759	-	-	-	-	-	-	-	ENSGALP00000031560	-	NP_653245.2	LmjF10.1040	76788	-	65518	141800	-	-	-	GSPATP00022979001	-	91055	145084	PF14_0595	-	-	-	-	-	-	110.m00145	-	-	-	60977	90867.m00190	Tb927.8.4460	-
		-	-	-	-	-	-	-	-	-	-	-	-	193665	-	-	-	-	-	-	-	-	-	-	-	-	-	-	-	-	-	-	-	-	-	-	-	-	-	-	-	-	-	-	-	-	-	-	-	-	-	-

FAP214	Flagellar Associated Protein	-	+	-	+	-	-	-	-	7645	-	-	-	193664	-	-	-	-	-	-	-	5.m00457	-	-	-	-	238227	-	81287	-	-	-	-	-	-	-	-	-	-	-	-	-	-	-	5.m00525	-	-	-	-	82119.m00083	-	-
		-	+	-	-	-	-	GB16959-PA	-	-	BDEG_05353	-	-	193661	385297	-	-	-	-	-	-	-	ENSGALP00000016919	-	-	-	-	32826	71505	162659	-	-	-	-	37658	-	133628	-	-	-	-	-	GLEAN3_25548	-	-	269866	-	-	-	-	-	-
HPT2	putative hexose-phosphate transporter	-	+	-	-	-	-	-	-	-	BDEG_00856	-	219246	193659	244136	-	CMQ264C	OTTDARP00000024318	-	-	-	-	ENSGALP00000012493	-	NP_001458.1	-	73040	33599	-	159917	-	-	9769	-	-	-	-	-	-	-	-	-	-	SINFRUP00000157547	-	-	-	65.m01191	51868	89692.m00565	-	-
		-	-	-	-	-	-	-	-	-	-	-	-	193656	-	-	-	-	-	-	-	-	-	-	-	-	-	-	-	-	-	-	-	-	-	-	-	-	-	-	-	-	-	-	-	-	-	-	-	-	-	-
		-	+	-	-	-	-	-	AT5G38460.1	32576	-	CE01478	169550	193646	251771	-	CMH028C	OTTDARP00000023038	DDB0185721	CG5091-PA	-	-	ENSGALP00000037189	-	OTTHUMP00000010712	-	102561	-	-	165140	-	Os07g07010.1	-	GSPATP00015853001	44117	149102	108422	-	555846	RO3G_05888.1	YOR002W	SPBC342.01c	GLEAN3_23829	SINFRUP00000134564	63.m00203	-	-	-	28993	-	-	-
		-	-	-	-	-	-	-	-	-	-	-	-	193642	-	-	-	-	-	-	-	-	-	-	-	-	-	-	-	-	-	-	-	-	-	-	-	-	-	-	-	-	-	-	-	-	-	-	-	-	-	-
NHP2	Nucleolar protein, Small subunit of H/ACA snoRNPs	-	+	+	-	-	-	GB14223-PA	AT5G08180.1	7967	BDEG_05087	CE19186	172931	193640	295037	cgd3_760	CMN132C	-	DDB0188528	CG5258-PA	19069619	250.m00081	ENSGALP00000005033	GL50803_13926	OTTHUMP00000161486	LmjF34.3980	211297	34007	75197	234038	NCU08951	Os02g49610.1	20902	GSPATP00020469001	36743	53765	108290	PF11_0250	564435	RO3G_16633.1	YDL208W	SPAC1782.10c	GLEAN3_00914	SINFRUP00000153223	213.m00083	37442	TA07115	583.m05562	7686	89678.m00190	Tb927.4.750	UM03340.1
		-	-	-	-	-	-	-	-	-	-	-	-	193639	-	-	-	-	-	-	-	-	-	-	-	-	-	-	-	-	-	-	-	-	-	-	-	-	-	-	-	-	-	-	-	-	-	-	-	-	-	-
		-	-	-	-	-	-	-	-	-	-	-	-	193638	-	-	-	-	-	-	-	-	-	-	NP_001013641.2	-	-	-	-	-	-	-	-	-	-	-	-	-	-	-	-	-	-	-	-	-	-	-	-	-	-	-
PRP39	nuclear pre-mRNA splicing factor and U1 snRNP Component	-	+	-	-	-	-	-	AT1G04080.1	-	-	CE28000	180947	193637	374517	-	-	OTTDARP00000001631	DDB0185449	-	-	-	ENSGALP00000020349	-	NP_060392.3	-	229414	-	76871	11203	NCU10810	Os03g11200.1	-	-	-	210141	155731	-	730568	RO3G_04887.1	YML046W	SPBC4B4.09	GLEAN3_27020	SINFRUP00000144596	-	-	-	50.m05616	59779	83757.m00018	-	-
		-	+	-	-	-	-	GB15983-PC	AT1G51450.1	-	BDEG_01306	CE20199	182172	193636	248255	cgd1_740	CML074C	-	DDB0203610	-	-	-	ENSGALP00000005109	-	OTTHUMP00000177396	-	218660	13024	56911	41253	NCU06993	Os11g04930.1	30442	-	46372	165579	-	PF08_0021	203136	RO3G_14001.1	YLR015W	SPBC13G1.08c	GLEAN3_18423	SINFRUP00000160738	128.m00083	21409	TA04475	-	58997	84458.m00219	-	UM01338.1
		-	+	-	-	-	-	GB14029-PA	AT1G61780.1	9132	-	CE39090	18809	193635	220095	-	-	-	-	CG4537-PA	-	-	ENSGALP00000016245	-	OTTHUMP00000159029	-	229474	-	-	247187	NCU00351	Os08g09180.1	32926	GSPATP00032937001	-	178368	-	-	713553	RO3G_15973.1	-	-	-	SINFRUP00000164081	18.m00330	-	-	50.m03357	-	-	-	-
		-	-	-	-	-	-	-	-	-	-	-	-	193634	-	-	-	-	-	-	-	-	-	-	-	-	-	-	-	-	-	-	-	-	-	-	-	-	-	-	-	-	-	-	-	-	-	-	-	-	-	-
		-	-	-	-	-	-	-	-	-	-	-	-	193630	-	-	-	-	-	-	-	-	-	-	-	-	-	-	-	-	-	-	-	-	-	-	-	-	-	-	-	-	-	-	-	-	-	-	-	-	-	-
		-	-	-	-	-	-	-	-	-	-	-	-	193626	-	-	-	-	-	-	-	-	-	-	-	-	-	-	-	-	-	-	-	-	-	-	-	-	-	-	-	-	-	-	-	-	-	-	-	-	-	-
		-	+	-	-	-	-	-	AT2G43470.1	-	-	-	-	193625	-	-	-	-	DDB0218604	-	-	-	-	-	-	-	235121	39237	-	203424	-	Os11g16420.1	-	-	-	141275	142784	-	562205	-	-	-	-	-	-	-	-	-	-	86827.m00364	-	-
		-	+	-	-	-	-	GB12454-PA	-	-	-	-	-	193624	212833	-	-	-	-	CG18146-PB	-	-	-	GL50803_17118	-	-	-	-	-	-	-	-	-	-	-	-	144450	-	-	-	-	-	GLEAN3_04895	-	42.m00229	-	-	-	-	-	-	-
	putative transcription factor	-	+	-	-	-	-	GB13433-PA	AT1G73230.1	38346	BDEG_04664	CE02573	163399	193614	381738	cgd6_2430	CML310C	OTTDARP00000018274	DDB0218319	CG3644-PB	-	-	ENSGALP00000017180	-	OTTHUMP00000010514	LmjF36.3770	181421	34637	58176	202084	NCU03148	Os03g63400.1	-	GSPATP00038137001	9697	48650	108876	PF14_0241	743498	RO3G_06065.1	YPL037C	SPAC4F10.14c	GLEAN3_12396	SINFRUP00000155287	111.m00081	-	TA15275	55.m11049	64133	-	Tb11.01.1465	UM03074.1
COX23	cytochrome c oxidase assembly protein 23, putative	-	-	-	-	-	-	-	AT4G12340.1	-	-	-	-	193612	-	-	-	-	-	-	-	-	-	-	-	-	-	-	-	-	-	-	-	-	-	163918	128447	-	799878	-	-	-	-	-	-	-	-	-	-	-	-	-
		-	-	-	-	-	-	-	-	-	-	-	-	193610	-	-	-	-	-	-	-	-	-	-	-	-	-	-	-	-	-	-	-	-	-	-	-	-	-	-	-	-	-	-	-	-	-	-	31777	-	-	-
		-	-	-	-	-	-	-	-	-	-	-	-	193607	-	-	-	-	-	-	-	-	-	-	-	-	-	-	-	-	-	Os10g32850.2	-	-	-	162488	-	-	775608	-	-	-	-	-	-	-	-	-	-	-	-	-
		-	-	-	-	-	-	-	-	-	-	-	-	193606	-	-	-	-	-	-	-	-	-	-	-	-	-	-	-	-	-	-	-	-	-	-	-	-	-	-	-	-	-	-	-	-	-	-	-	-	-	-
FAP165	Flagellar Associated Protein	-	-	-	+	-	-	-	-	33989	-	-	-	193605	-	-	-	-	-	-	-	-	-	-	-	-	-	-	47246	-	NCU01111	-	-	-	-	-	-	-	-	-	-	-	-	-	-	-	-	-	-	-	-	-
		-	-	-	-	-	-	-	-	-	-	-	-	193604	-	-	-	-	-	-	-	-	ENSGALP00000008468	-	-	-	-	-	-	-	-	-	-	-	-	-	-	-	756375	-	-	-	-	-	-	-	-	-	-	-	-	-
		-	+	-	-	-	-	-	AT4G08690.1	6155	BDEG_06131	-	-	193602	-	cgd6_950	CMT608C	-	DDB0192040	-	-	-	-	-	-	LmjF30.1680	-	-	78034	-	NCU03596	Os03g51430.2	32312	GSPATP00027393001	15063	34277	-	-	242050	RO3G_03225.1	YNL264C	SPCC23B6.04c	-	-	52.m00220	270121	TA05300	50.m03110	-	-	-	-
		-	-	-	-	-	-	-	-	-	-	-	-	193599	-	-	-	-	-	-	-	-	-	-	-	-	-	-	-	-	-	-	37950	-	-	232165	-	-	-	-	YNL024C	-	-	-	-	-	-	-	-	-	-	-
		-	-	-	-	-	-	-	-	-	-	-	-	193597	-	-	-	-	-	-	-	-	-	-	-	-	-	-	-	-	-	-	-	-	-	-	-	-	-	-	-	-	-	-	-	-	-	-	-	-	-	-
		-	-	-	-	-	-	-	-	33571	-	-	-	193596	-	-	-	-	-	-	-	-	-	-	-	-	-	-	-	-	-	-	-	-	-	-	-	-	-	-	-	-	-	-	-	-	-	-	-	-	-	-
		-	-	-	-	-	-	-	-	-	-	-	-	193592	-	-	-	-	-	-	-	-	-	-	-	-	-	-	-	-	-	-	-	-	-	-	-	-	-	-	-	-	-	-	-	-	-	-	-	-	-	-
		-	-	-	-	-	-	-	-	-	-	-	189817	193591	-	-	-	-	-	-	-	-	-	-	-	-	-	-	-	-	-	-	-	-	-	-	-	-	-	-	-	-	-	-	-	-	-	-	-	-	-	-
		-	-	-	-	-	-	-	-	-	-	-	-	193590	-	-	-	-	-	-	-	-	-	-	-	-	-	-	-	-	-	-	-	-	-	-	-	-	-	-	-	-	-	-	-	-	-	-	-	-	-	-
IF2Bc	Translation initiation factor eIF-2B gamma subunit	-	+	-	-	-	-	GB16418-PA	AT5G19485.1	-	BDEG_00616	CE23548	226439	193588	387969	cgd8_3940	CMM014C	OTTDARP00000020301	DDB0189012	CG8190-PA	-	-	ENSGALP00000037671	-	OTTHUMP00000010262	-	206598	34620	80872	106663	-	Os06g23160.1	10771	GSPATP00014128001	-	59092	-	MAL13P1.144	821380	RO3G_10990.1	-	SPAC4D7.09	-	SINFRUP00000164612	96.m00171	9867	-	35.m00873	58011	-	-	-
		-	-	-	-	-	-	-	-	-	-	-	-	193585	-	-	-	-	-	-	-	-	-	-	-	-	-	-	-	-	-	-	-	-	-	-	-	-	-	-	-	-	-	-	-	-	-	-	-	-	-	-
TEF23	Unknown function, mitochondrial localization assumed	-	+	-	-	-	-	-	AT5G35220.1	-	-	-	-	193583	-	-	CMQ013C	-	-	-	-	-	-	-	-	-	-	-	-	-	-	Os03g57840.2	20210	-	33462	156247	-	-	773851	-	-	-	-	-	-	8537	-	-	-	-	-	-
		-	-	-	-	-	-	-	-	-	-	-	-	193579	-	-	-	-	-	-	-	-	-	-	-	-	-	-	-	-	-	-	-	-	-	-	-	-	-	-	-	-	-	-	-	-	-	-	-	-	-	-
		+	+	-	-	-	-	GB14454-PA	-	-	BDEG_01041	-	215088	193577	264184	-	-	OTTDARP00000012188	-	CG8086-PD	-	-	-	GL50803_91354	OTTHUMP00000066364	LmjF05.0690	236494	38778	70051	-	-	-	-	GSPATP00036319001	-	163291	-	-	-	-	-	-	-	SINFRUP00000137449	5.m00468	-	-	-	33050	87331.m00050	Tb927.7.7200	-
ARLB1,ARFRP1	ARFRP1, ARF-related protein 1 GTPase	-	+	-	-	-	-	GB18659-PA	AT5G52210.2	54947	BDEG_03774	CE22471	226979	193574	241264	-	-	-	DDB0185588	CG7039-PA	-	-	ENSGALP00000009835	-	OTTHUMP00000031584	-	204069	2913	-	235187	NCU00333	Os03g27450.1	30663	-	-	71658	121947	-	728818	-	YPL051W	-	GLEAN3_06463	SINFRUP00000157402	-	-	-	-	58105	-	-	-
		-	-	-	-	-	-	-	-	-	-	-	-	193571	-	-	-	-	-	-	-	-	-	-	-	-	-	-	-	-	-	-	-	-	-	-	-	-	-	-	-	-	-	-	-	-	-	-	-	-	-	-
		-	-	-	-	-	-	-	-	-	-	-	-	193570	-	-	-	-	-	-	-	-	-	-	-	-	-	-	-	-	-	-	-	-	-	-	-	-	-	-	-	-	-	-	-	-	-	-	-	-	-	-
		-	-	-	-	-	-	-	-	-	-	-	-	193569	-	-	-	-	-	-	-	-	-	-	-	-	-	-	-	-	-	-	-	-	-	-	-	-	-	-	-	-	-	-	-	-	-	-	-	-	-	-
		-	-	-	-	-	-	-	-	-	-	-	-	193567	-	-	-	-	-	-	-	-	-	-	-	-	-	-	-	-	-	-	-	-	-	-	-	-	-	-	-	-	-	-	-	-	-	-	-	-	-	-
		-	+	-	-	-	-	GB14262-PA	AT3G21110.2	27954	BDEG_05666	-	-	193566	-	-	CME023C	-	-	CG17024-PA	-	-	-	-	-	-	-	-	-	-	NCU03166	Os09g29190.1	8999	-	18080	114429	109393	-	258353	RO3G_03348.1	YAR015W	SPBC409.10	-	-	-	268443	-	-	-	-	-	UM04726.1
		-	-	-	-	-	-	-	-	-	-	-	-	193565	-	-	-	-	-	-	-	-	-	-	-	-	-	-	-	-	-	-	-	-	-	-	-	-	-	-	-	-	-	-	-	-	-	-	-	-	-	-
		-	-	-	-	-	-	-	-	-	-	-	-	193564	-	-	-	-	-	-	-	-	-	-	-	-	-	-	-	-	-	-	-	-	-	-	-	-	-	-	-	-	-	-	-	-	-	-	-	-	-	-
PP2A-1c	Protein Phosphatase 2A catalytic subunit	-	+	+	-	-	-	GB14863-PA	AT1G59830.1	69972	BDEG_00890	CE10074	227677	193562	296817	cgd7_810	CML332C	OTTDARP00000023529	DDB0188828	CG7109-PA	19068789	16.m00291	ENSGALP00000010423	GL50803_5010	OTTHUMP00000159382	LmjF25.1320	184125	20632	55906	195622	NCU06630	Os03g59060.2	18276	GSPATP00002246001	22247	165449	109378	-	824629	RO3G_14499.1	YDL188C	SPBC16H5.07c	GLEAN3_25182	SINFRUP00000163289	104.m00157	1585	TA15515	42.m03342	63380	-	Tb927.3.1240	UM03957.1
		-	+	-	-	-	-	-	-	66773	-	-	-	193561	-	-	-	-	-	-	-	-	-	-	-	LmjF31.0090	-	-	59812	-	-	-	-	GSPATP00002045001	-	-	-	-	-	-	-	-	-	-	80.m00194	-	-	-	-	-	Tb927.6.1660	-
		-	+	-	-	-	-	-	AT4G17600.1	-	-	-	-	193552	-	-	-	-	-	-	-	-	-	-	-	-	-	-	-	-	-	Os02g03330.1	5873	-	-	6728	-	-	740033	-	-	-	-	-	-	-	-	-	-	-	-	-
		-	-	-	-	-	-	-	-	-	-	-	-	193545	-	-	-	-	-	-	-	-	-	-	-	-	-	-	-	-	-	-	-	-	-	-	-	-	-	-	-	-	-	-	-	-	-	-	-	-	-	-
		-	-	-	-	-	-	-	-	-	-	-	-	193544	-	-	-	-	-	-	-	-	-	-	-	-	-	-	-	-	-	-	-	-	-	-	-	-	-	-	-	-	-	-	-	-	-	-	-	-	-	-
		-	-	-	-	-	-	-	-	-	-	-	-	193541	-	-	-	-	-	-	-	-	-	-	-	-	-	-	-	-	-	-	-	-	-	-	-	-	-	-	-	-	-	-	-	-	-	-	-	-	-	-
CYG6	putative signal transduction protein	-	-	-	-	-	-	-	-	-	-	-	-	193537	-	-	-	-	-	-	-	-	-	-	-	-	-	-	-	-	-	-	-	-	-	-	-	-	-	-	-	-	-	-	-	-	-	-	-	-	-	-
		-	+	-	-	-	-	-	-	62835	-	-	-	193531	-	-	-	-	-	-	-	-	ENSGALP00000019669	-	OTTHUMP00000170625	-	-	-	-	79745	-	-	-	GSPATP00012619001	-	-	-	-	-	-	-	-	-	-	28.m00191	-	-	-	-	-	-	-
		-	-	-	-	-	-	-	-	-	-	-	-	193526	-	-	-	-	-	-	-	-	-	-	-	-	-	-	-	-	-	-	-	-	-	-	-	-	-	-	-	-	-	-	-	-	-	-	-	-	-	-
ADCY37	adenylate cyclase	-	-	-	-	-	-	-	-	-	-	-	-	193525	-	-	-	-	-	-	-	-	-	-	-	-	-	-	-	-	-	-	-	-	-	-	-	-	-	-	-	-	-	-	-	-	TA15475	-	-	-	-	-
		-	-	-	-	-	-	-	-	-	-	-	-	193521	-	-	-	-	-	-	-	-	-	-	-	-	-	-	-	-	-	-	-	-	-	-	-	-	-	-	-	-	-	-	-	-	-	-	-	-	-	-
		-	-	-	-	-	-	-	-	-	-	-	-	193520	-	-	-	-	-	-	-	-	-	-	-	-	-	-	-	-	-	-	37653	-	-	-	-	-	-	-	-	-	-	-	-	-	-	-	-	-	-	-
		-	-	-	-	-	-	-	-	-	-	-	-	193519	-	-	-	-	-	-	-	-	-	-	-	-	-	-	-	-	-	-	-	-	-	-	-	-	-	-	-	-	-	-	-	-	-	-	-	-	-	-
MSD2	superoxide dismutase [Mn]	-	+	-	-	-	-	GB14346-PA	AT3G10920.1	59085	BDEG_06625	CE09323	152709	193511	207182	-	CMR158C	OTTDARP00000005388	DDB0202901	CG8905-PA	-	-	ENSGALP00000019039	-	OTTHUMP00000017531	-	210661	34408	-	236349	NCU09560	Os05g25850.1	6206	GSPATP00028747001	42832	141133	109207	-	730452	RO3G_03033.1	YHR008C	SPAC1486.01	GLEAN3_24657	SINFRUP00000152842	37.m00311	40713	-	-	21084	-	-	UM02453.1
		-	-	-	-	-	-	-	-	-	-	-	-	193509	-	-	-	-	-	-	-	-	-	-	-	-	-	-	-	-	-	-	-	-	-	-	-	-	-	-	-	-	-	-	-	-	-	-	-	-	-	-
		-	-	-	-	-	-	-	AT5G22320.1	-	-	-	-	193505	-	-	-	-	-	-	-	-	-	-	-	-	-	-	-	-	-	-	-	-	-	-	-	-	-	-	-	-	-	-	-	-	-	-	-	-	-	-
		-	-	-	-	-	-	-	-	-	-	-	-	193503	-	-	-	-	-	-	-	-	-	-	-	-	-	-	-	237738	-	-	-	-	-	-	-	-	-	-	-	-	-	-	-	-	-	-	-	-	-	-
		-	-	-	-	-	-	-	-	-	-	-	-	193502	-	-	-	-	-	-	-	-	-	-	-	-	-	-	-	-	-	-	34233	-	45851	194212	-	-	-	-	-	-	-	-	-	-	-	-	-	-	-	-
		-	+	+	-	-	-	GB19053-PA	AT1G72550.2	58678	BDEG_02496	CE20708	158835	193500	298916	cgd8_3320	CMJ128C	OTTDARP00000024201	DDB0217926	-	19068809	196.m00076	ENSGALP00000008398	GL50803_16760	OTTHUMP00000164205	LmjF19.1040	217906	26444	82010	188942	NCU08888	Os05g48510.2	22353	GSPATP00032426001	27709	117470	108453	PF11_0051	828230	RO3G_13577.1	YLR060W	SPAC23A1.12c	-	SINFRUP00000169195	18.m00294	33046	TA11150	542.m00221	50402	97360.m00076	-	UM04176.1
		-	-	-	-	-	-	-	-	-	-	-	-	193499	-	-	-	-	-	-	-	-	-	-	-	-	-	-	-	-	-	-	30720	-	-	-	-	-	-	-	-	-	-	-	-	-	-	-	-	-	-	-
		-	-	-	-	-	-	-	-	-	-	-	-	193498	-	-	-	-	-	-	-	-	-	-	-	-	-	-	-	-	-	-	-	-	-	-	-	-	-	-	-	-	-	-	-	-	-	-	-	-	-	-
		-	-	-	-	-	-	-	-	-	-	-	-	193495	-	-	-	-	-	-	-	-	-	-	-	-	-	-	-	-	-	-	-	-	-	-	-	-	-	-	-	-	-	-	-	-	-	-	-	-	-	-
		-	-	-	-	-	-	-	-	-	-	-	-	193494	-	-	-	-	-	-	-	-	-	-	-	-	-	-	-	-	-	-	-	-	-	-	-	-	-	-	-	-	-	-	-	-	-	-	-	-	-	-
C_131007		-	-	-	-	-	-	-	-	-	-	-	-	193493	-	-	-	-	-	-	-	-	-	-	-	-	-	-	-	-	-	-	-	-	-	-	-	-	-	-	-	-	-	-	-	-	-	-	-	-	-	-
		-	-	-	-	-	-	-	-	-	-	-	-	193489	-	-	-	-	-	-	-	-	-	-	-	-	-	-	-	-	-	-	-	-	-	-	-	-	-	-	-	-	-	-	-	-	-	-	-	-	-	-
		-	+	-	-	-	-	GB15486-PA	AT4G21860.1	-	BDEG_01996	CE40061	-	193479	381306	-	-	-	-	CG6584-PC	-	-	ENSGALP00000028035	-	-	LmjF28.2660	156915	-	-	90236	-	-	-	GSPATP00029495001	7666	-	108335	-	707507	-	-	-	-	-	10.m00407	-	-	-	-	-	Tb11.01.3760	-
		-	-	-	-	-	-	-	-	-	-	-	-	193475	-	-	-	-	-	-	-	-	-	-	-	-	-	-	-	-	-	-	-	-	-	-	-	-	-	-	-	-	-	-	-	-	-	-	-	-	-	-
		-	-	-	-	-	-	-	-	-	-	-	-	193473	-	-	-	-	-	-	-	-	-	-	-	-	-	-	-	-	-	-	-	-	-	-	-	-	-	-	-	-	-	-	-	-	-	-	-	-	-	-
		-	-	-	-	-	-	-	-	-	-	-	-	193471	-	-	-	-	-	-	-	-	-	-	-	-	-	-	-	-	-	-	-	-	-	-	-	-	-	-	-	-	-	-	-	-	-	-	-	-	-	-
		-	+	-	-	-	-	GB13445-PA	AT5G05710.1	8195	-	-	-	193468	226325	cgd5_1970	-	OTTDARP00000020787	DDB0217466	CG12467-PA	-	32.m00252	-	-	-	-	104002	-	46998	237545	-	Os01g63800.1	20678	GSPATP00000209001	-	185888	-	PFB0257c	650148	RO3G_12847.1	-	-	-	-	105.m00160	-	-	55.m04879	-	-	-	-
		-	+	-	-	-	-	GB16346-PA	AT1G16350.1	53803	BDEG_06240	CE30188	173337	193467	210114	cgd6_20	CML243C	OTTDARP00000020807	DDB0185636	CG1799-PC	-	-	ENSGALP00000011185	-	OTTHUMP00000171078	LmjF19.1560	134377	38942	34674	177384	NCU03117	Os03g56800.1	23295	GSPATP00009689001	31718	223796	109162	PFI1020c	765701	RO3G_14462.1	YLR432W	SPBC2F12.14c	GLEAN3_05852	SINFRUP00000145930	59.m00250	27873	TA03405	44.m00049	50297	-	Tb10.61.0150	UM02566.1
		-	-	-	-	-	-	-	-	-	-	-	-	193464	-	-	-	-	-	-	-	-	-	-	-	-	-	-	-	-	-	Os03g13460.3	-	-	-	-	-	-	253557	-	-	-	-	-	-	-	-	-	-	-	-	-
CYCT1	T-type cyclin	-	-	-	-	-	-	-	-	28778	-	-	-	193461	-	-	CMN175C	-	-	-	-	-	-	-	-	-	-	-	-	-	-	-	-	-	-	-	-	-	-	-	-	-	-	-	-	-	-	-	-	-	-	-
		-	-	-	-	-	-	-	-	-	-	-	-	193460	-	-	-	-	-	-	-	-	-	-	-	-	-	-	-	-	-	-	-	-	-	-	-	-	-	-	-	-	-	-	-	-	-	-	-	-	-	-
		-	-	-	-	-	-	-	-	-	-	-	-	193456	-	-	-	-	-	-	-	-	-	-	-	-	-	-	-	-	-	-	-	-	-	-	-	-	-	-	-	-	-	-	-	-	-	-	-	-	-	-
		-	-	-	-	-	-	-	-	-	-	-	-	193454	-	-	-	-	-	-	-	-	-	-	-	-	-	-	-	-	-	-	-	-	-	-	-	-	-	-	-	-	-	-	-	-	-	-	-	-	-	-
		-	-	-	-	-	-	-	-	-	-	-	-	193453	-	-	-	-	-	-	-	-	-	-	-	-	-	-	-	-	-	-	-	-	-	-	-	-	-	-	-	-	-	-	-	-	-	-	-	-	-	-
FAP197	Flagellar Associated Protein	-	-	-	+	-	-	-	-	-	-	-	-	193450	-	-	-	-	-	-	-	-	-	-	-	-	-	-	-	-	-	-	-	-	-	-	-	-	-	-	-	-	-	-	-	-	-	-	-	-	-	-
		-	-	-	-	-	-	-	-	-	-	-	-	193449	-	-	-	-	-	-	-	-	-	-	-	-	-	-	-	-	-	-	-	-	-	-	-	-	-	-	-	-	-	-	-	-	-	-	-	-	-	-
		-	+	+	-	-	-	GB30029-PA	AT5G40280.1	24404	BDEG_08209	CE09598	168477	193447	288400	cgd1_1980	CMS122C	OTTDARP00000005680	DDB0216701	-	-	83.m00187	-	GL50803_9382	OTTHUMP00000028350	LmjF26.1470	222587	33884	35542	161979	NCU05999	Os01g53600.1	31264	GSPATP00029013001	16932	136150	128710	PF11_0483	258731	RO3G_15757.1	YDL090C	SPAC17G6.04c	-	SINFRUP00000132281	21.m00380	264607	TA15255	8.m00187	29686	-	Tb927.7.460	UM05348.1
		-	-	-	-	-	-	-	-	-	-	-	-	193444	-	-	-	-	-	-	-	-	-	-	-	-	-	-	-	-	-	-	-	-	-	-	-	-	-	-	-	-	-	-	-	-	-	-	-	-	-	-
		-	-	-	-	-	-	-	-	-	-	-	-	193441	-	-	-	-	-	-	-	-	-	-	-	-	-	-	-	-	-	-	-	-	-	-	-	-	-	-	-	-	-	-	-	-	-	-	-	-	-	-
		-	-	-	-	-	-	-	-	-	-	-	-	193440	-	-	-	-	-	-	-	-	-	-	-	-	-	-	-	-	-	-	-	-	-	-	-	-	-	-	-	-	-	-	-	-	-	-	-	-	-	-
		-	-	-	-	-	-	-	-	-	-	-	-	193438	-	-	-	-	-	-	-	-	-	-	-	-	-	-	-	-	-	-	-	-	-	-	-	-	-	-	-	-	-	-	-	-	-	-	-	-	-	-
		-	+	-	-	-	-	-	AT5G18700.1	26764	-	-	82100	193436	239638	-	-	-	-	-	-	-	-	GL50803_103838	XP_001133238.1	LmjF28.0620	220770	-	32341	173313	-	Os01g15480.1	21585	GSPATP00009892001	-	136143	133502	-	-	-	-	-	GLEAN3_08621	-	117.m00165	-	-	-	54294	82181.m00186	Tb11.01.0400	-
		-	-	-	-	-	-	-	AT1G64430.2	-	-	-	-	193435	-	-	-	-	-	-	-	-	-	-	-	-	-	-	-	-	-	Os08g24390.1	-	-	-	30899	-	-	179994	-	-	-	-	-	-	-	-	-	-	-	-	-
		-	-	-	-	-	-	-	-	-	-	-	-	193431	-	-	-	-	-	-	-	-	-	-	-	-	-	-	-	-	-	-	-	-	-	-	-	-	-	-	-	-	-	-	-	-	-	-	-	-	-	-
		-	-	-	-	-	-	-	-	-	-	-	-	193429	-	-	-	-	-	-	-	-	-	-	-	-	-	-	-	-	-	-	-	-	-	-	-	-	-	-	-	-	-	-	-	-	-	-	-	-	-	-
		-	-	-	-	-	-	-	-	-	-	-	-	193428	-	-	-	-	-	-	-	-	-	-	-	-	-	-	-	-	-	-	-	-	-	-	-	-	-	-	-	-	-	-	-	-	-	-	-	-	-	-
		-	-	-	-	-	-	-	-	-	-	-	-	193426	-	-	-	-	-	-	-	-	-	-	-	-	-	-	-	-	-	-	-	-	-	-	-	-	-	-	-	-	-	-	-	-	-	-	-	-	-	-
PBD1		-	+	+	-	-	-	GB10351-PA	AT4G14800.1	60167	BDEG_07122	CE13818	176004	193424	204801	cgd1_420	CMH119C	-	DDB0190287	CG17331-PA	19173354	166.m00093	ENSGALP00000003732	GL50803_3209	OTTHUMP00000004345	LmjF36.0320	181139	35785	35037	193516	NCU01368	Os03g48930.1	21715	GSPATP00001231001	19341	208929	119339	PF14_0676	814139	RO3G_02025.1	YER012W	SPAC31A2.04c	-	SINFRUP00000152343	12.m00560	7964	TA19315	52.m00007	50465	94208.m00087	Tb10.70.2490	UM04179.1
		-	-	-	-	-	-	-	-	-	-	-	-	193410	-	-	-	-	-	-	-	-	-	-	-	-	-	-	-	-	-	-	-	-	-	-	-	-	-	-	-	-	-	-	-	-	-	-	-	-	-	-
		-	-	-	-	-	-	-	-	-	-	-	-	193409	-	-	-	-	-	-	-	-	-	-	-	-	-	-	-	-	-	-	-	-	-	-	-	-	-	-	-	-	-	-	-	-	-	-	-	-	-	-
		-	-	-	-	-	-	-	-	-	-	-	-	193408	-	-	-	-	-	-	-	-	-	-	-	-	-	-	-	-	-	-	-	-	-	-	-	-	-	-	-	-	-	-	-	-	-	-	-	-	-	-
		-	-	-	-	-	-	-	-	-	-	-	-	193404	-	-	-	-	-	-	-	-	-	-	-	-	-	-	-	-	-	-	-	-	-	-	-	-	-	-	-	-	-	-	-	-	-	-	-	-	-	-
		-	-	-	-	-	-	-	-	-	-	-	-	193403	-	-	-	-	-	-	-	-	-	-	-	-	-	-	-	-	-	-	-	-	-	-	-	-	-	-	-	-	-	-	-	-	-	-	-	-	-	-
		-	-	-	-	-	-	-	-	64857	-	-	-	193400	-	-	-	-	-	-	-	-	-	-	-	-	-	-	-	-	-	-	-	-	-	-	-	-	-	-	-	-	-	-	-	-	-	-	-	-	-	-
		-	+	-	-	-	-	-	-	63683	-	-	-	193399	-	-	-	-	DDB0183792	-	-	-	-	-	-	-	-	-	2026	-	-	-	-	-	47032	-	135952	-	-	-	-	-	-	-	-	262792	-	-	-	-	Tb927.2.2400	-
		-	-	-	-	-	-	-	-	-	-	-	-	193398	-	-	-	-	-	-	-	-	-	-	-	-	-	-	-	-	-	-	-	-	-	-	-	-	-	-	-	-	-	-	-	-	-	-	-	-	-	-
		-	-	-	-	-	-	-	-	-	-	-	-	193391	-	-	-	-	-	-	-	-	-	-	-	-	-	-	-	-	-	-	-	-	-	-	-	-	-	-	-	-	-	-	-	-	-	-	-	-	-	-
		-	-	-	-	-	-	-	-	-	-	-	-	193388	-	-	-	-	-	-	-	-	-	-	-	-	-	-	-	-	-	-	-	-	-	-	-	-	-	-	-	-	-	-	-	-	-	-	-	-	-	-
		-	-	-	-	-	-	-	-	-	-	-	-	193387	-	-	-	-	-	-	-	-	-	-	-	-	-	-	-	-	-	-	-	-	-	-	-	-	-	-	-	-	-	-	-	-	-	-	-	-	-	-
		-	-	-	-	-	-	-	-	-	-	-	-	193385	-	-	-	-	-	-	-	-	-	-	-	-	-	-	-	-	-	-	-	-	-	-	-	-	-	-	-	-	-	-	-	-	-	-	-	-	-	-
		-	-	-	-	-	-	-	-	-	-	-	-	193384	-	-	-	-	-	-	-	-	-	-	-	-	-	-	-	-	-	-	-	-	-	-	-	-	-	-	-	-	-	-	-	-	-	-	-	-	-	-
		-	-	-	-	-	-	-	-	-	-	-	-	193381	-	-	-	-	-	-	-	-	-	-	-	-	-	-	-	-	-	-	-	-	-	-	-	-	-	-	-	-	-	-	-	-	-	-	-	-	-	-
		-	-	-	-	-	-	-	-	-	-	-	-	193380	-	-	-	-	-	-	-	-	-	-	-	-	-	-	-	-	-	-	-	-	-	-	-	-	-	-	-	-	-	-	-	-	-	-	-	-	-	-
		-	-	-	-	-	-	-	-	-	-	-	-	193379	-	-	-	-	-	-	-	-	-	-	-	-	-	-	-	-	-	-	-	-	-	-	-	-	-	-	-	-	-	-	-	-	-	-	-	-	-	-
		-	-	-	+	-	-	-	-	-	-	-	-	193378	-	-	-	-	-	-	-	-	-	-	-	-	-	-	-	-	-	-	-	-	-	-	-	-	-	-	-	-	-	-	-	-	-	-	-	-	-	-
		-	-	-	-	-	-	-	-	-	-	-	-	193373	-	-	-	-	-	-	-	-	-	-	-	-	-	-	-	-	-	-	-	-	-	-	-	-	-	-	-	-	-	-	-	-	-	-	-	-	-	-
FAP5	Conserved Uncharacterized Flagellar Associated Protein	-	-	-	+	-	-	-	-	-	-	-	-	193372	-	-	-	-	-	-	-	-	-	-	-	-	-	-	-	-	-	-	-	-	-	-	-	-	-	-	-	-	-	-	-	-	-	-	-	-	-	-
		-	+	-	-	-	-	-	-	-	-	-	-	193364	-	-	CMS109C	-	DDB0188287	-	-	-	-	-	-	-	-	-	56214	-	-	-	-	-	-	-	-	-	-	-	YGR175C	-	-	-	-	-	-	-	-	-	-	UM02398.1
FAP43	Conserved Uncharacterized Flagellar Associated Protein	+	+	-	+	-	-	GB12153-PA	-	-	-	-	160880	193355	279802	-	-	-	-	-	-	-	ENSGALP00000013656	-	NP_079421.5	LmjF31.3150	233795	27807	-	99994	-	-	11652	GSPATP00016772001	-	73462	135053	-	-	-	-	-	-	SINFRUP00000154147	16.m00460	-	-	65.m00013	60769	82761.m00582	Tb927.4.5380	-
		-	-	-	-	-	-	-	-	-	-	-	-	193354	-	-	-	-	-	-	-	-	-	-	-	-	-	-	-	-	-	-	-	-	-	-	-	-	-	-	-	-	-	-	-	-	-	-	-	-	-	-
		-	-	-	-	-	-	-	-	-	-	-	-	193349	-	-	-	-	-	-	-	-	-	-	-	-	-	-	-	-	-	-	-	-	-	-	-	-	-	-	-	-	-	-	-	-	-	-	-	-	-	-
		-	-	-	-	-	-	-	-	-	-	-	-	193346	-	-	-	-	-	-	-	-	-	-	-	-	-	-	-	-	-	-	-	-	-	-	-	-	-	-	-	-	-	-	-	-	-	-	-	-	-	-
		-	-	-	-	-	-	-	-	-	-	-	-	193345	-	-	-	-	-	-	-	-	-	-	-	-	-	-	-	-	-	-	-	-	-	-	-	-	-	-	-	-	-	-	-	-	-	-	-	-	-	-
		-	+	-	-	-	-	-	-	-	BDEG_04914	-	-	193344	-	-	-	-	-	-	-	-	-	-	-	-	-	-	-	-	NCU09847	-	-	GSPATP00029676001	-	161066	129468	-	-	RO3G_03657.1	-	SPBC83.18c	GLEAN3_21673	-	-	-	-	-	-	-	Tb927.8.8150	-
		-	-	-	-	-	-	-	-	-	-	-	-	-	-	-	-	-	-	-	-	-	-	-	-	-	-	-	-	-	-	-	-	-	-	-	-	-	-	-	-	-	-	-	-	-	-	-	-	-	-	-
		-	-	-	-	-	-	-	-	-	-	-	-	193342	-	-	-	-	-	-	-	-	-	-	-	-	-	-	-	-	-	-	34818	-	-	-	-	-	-	-	-	-	-	-	-	-	-	-	-	-	-	-
		-	-	-	-	-	-	-	-	-	-	-	-	193339	-	-	-	-	-	-	-	-	ENSGALP00000037724	-	-	-	-	-	-	-	-	-	-	-	-	-	-	-	-	-	-	-	-	-	8.m00463	-	-	20.m03748	-	-	-	-
		-	-	-	-	-	-	-	-	-	-	-	-	193336	-	-	-	-	-	-	-	-	-	-	-	-	-	-	-	-	-	-	-	-	-	-	-	-	-	-	-	-	-	-	-	-	-	-	-	-	-	-
		-	-	-	-	-	-	-	-	-	-	-	-	193335	-	-	-	-	-	-	-	-	-	-	-	-	-	-	-	-	-	-	-	-	-	-	-	-	-	-	-	-	-	-	-	-	-	-	-	-	-	-
		-	-	-	-	-	-	-	-	-	-	-	-	193334	-	-	-	-	-	-	-	-	-	-	-	-	-	-	-	-	-	-	-	-	-	-	-	-	-	-	-	-	-	-	-	-	-	-	-	-	-	-
		-	-	-	-	-	-	-	-	-	-	-	-	193327	-	-	-	-	-	-	-	-	-	-	-	-	-	-	-	-	-	-	-	-	-	-	-	-	-	-	-	-	-	-	-	-	-	-	-	-	-	-
		-	-	-	-	-	-	-	-	-	-	-	-	193326	-	-	-	-	-	-	-	-	-	-	-	-	-	-	-	-	-	-	-	-	-	-	-	-	-	-	-	-	-	-	-	-	-	-	-	-	-	-
		-	-	-	-	-	-	-	-	-	-	-	-	193324	-	-	-	-	-	-	-	-	-	-	-	-	-	-	-	-	-	-	-	-	-	-	-	-	-	-	-	-	-	-	-	-	-	-	-	-	-	-
		-	-	-	-	-	-	-	-	-	-	-	-	193313	-	-	-	-	-	-	-	-	-	-	-	-	-	-	-	-	-	-	-	-	-	-	-	-	-	-	-	-	-	-	-	-	-	-	-	-	-	-
IMP2	IMP2, inner membrane peptidase, mitochondrial	-	+	-	-	-	-	-	AT1G53530.1	16372	BDEG_00323	CE36686	-	193312	297430	cgd4_620	CMB077C	-	DDB0215647	CG9240-PA	-	-	ENSGALP00000031178	-	NP_659418.1	LmjF36.0200	103673	3212	61430	-	-	Os11g40500.3	19758	GSPATP00028596001	-	187631	133224	-	707831	-	YMR150C	SPBC2D10.07c	-	-	75.m00132	19301	TA16115	59.m03416	-	-	Tb10.70.2630	-
		-	-	-	-	-	-	-	-	-	-	-	-	193311	-	-	-	-	-	-	-	-	-	-	-	-	-	-	-	-	-	-	-	-	-	-	-	-	-	-	-	-	-	-	-	-	-	-	-	-	-	-
LCI13	low-CO2-inducible protein	-	-	-	-	-	-	-	-	-	-	-	-	193305	-	-	-	-	-	-	-	-	-	-	-	-	-	-	-	-	-	-	-	-	-	-	-	-	-	-	-	-	-	-	-	-	-	-	-	-	-	-
		-	-	-	-	-	-	GB10953-PA	-	-	-	-	-	193302	-	-	-	-	-	-	-	-	ENSGALP00000027658	-	NP_714916.3	-	-	-	-	-	-	-	-	-	-	-	-	-	-	-	-	-	-	SINFRUP00000132982	-	-	-	-	-	-	-	-
		-	-	-	-	-	-	-	-	-	-	-	-	193300	-	-	-	-	-	-	-	-	-	-	-	-	-	-	-	-	-	-	-	-	-	-	-	-	-	-	-	-	-	-	-	-	-	-	-	-	-	-
ADCY33	adenylate cyclase	-	-	-	-	-	-	-	-	-	-	-	-	193299	-	-	-	-	-	-	-	-	-	-	-	-	-	-	69917	-	-	-	-	-	-	-	-	-	-	-	-	-	-	-	57.m00225	-	-	-	-	-	-	-
		-	-	-	-	-	-	-	-	-	-	-	-	193298	-	-	-	-	-	-	-	-	-	-	-	-	-	-	-	-	-	-	-	-	-	-	-	-	-	-	-	-	-	-	-	-	-	-	-	-	-	-
		-	-	-	-	-	-	-	-	-	-	-	-	193297	-	-	-	-	-	-	-	-	-	-	-	-	-	-	-	-	-	-	-	-	-	-	-	-	-	-	-	-	-	-	-	-	-	-	-	-	-	-
PETC	Rieske iron-sulfur subunit of the Cytochrome B6-F complex , chloroplast precursor	-	+	-	-	-	-	-	AT4G03280.2	59217	-	-	-	193296	-	-	CMI281C	-	-	-	-	-	-	-	-	-	-	-	-	-	-	Os07g37030.1	27356	-	13358	121098	-	-	663556	-	-	-	-	-	-	26131	-	-	-	-	-	-
		-	-	-	-	-	-	-	-	-	-	-	-	193294	-	-	-	-	-	-	-	-	-	-	-	-	-	22124	-	-	-	-	-	-	-	-	-	-	-	-	-	-	-	-	-	-	-	-	-	-	-	-
		-	-	-	-	-	-	-	-	-	-	-	-	193292	-	-	-	-	-	-	-	-	-	-	-	-	-	-	-	-	-	-	-	-	-	-	-	-	-	-	-	-	-	-	-	-	-	-	-	-	-	-
		-	-	-	-	-	-	-	-	-	-	-	-	193290	-	-	-	-	-	-	-	-	-	-	-	-	-	-	-	-	-	-	-	-	-	-	-	-	-	-	-	-	-	-	-	-	-	-	-	-	-	-
		-	-	-	-	-	-	-	-	-	-	-	-	193287	-	-	-	-	-	-	-	-	-	-	-	-	-	-	-	-	-	-	-	-	-	-	-	-	-	-	-	-	-	-	-	-	-	-	-	-	-	-
		-	-	-	-	-	-	-	-	-	-	-	-	193283	-	-	-	-	-	-	-	-	-	-	-	-	-	-	-	-	-	-	-	-	-	-	-	-	-	-	-	-	-	-	-	-	-	-	-	-	-	-
		-	-	-	-	-	-	-	-	-	-	-	-	193280	-	-	-	-	-	-	-	-	-	-	-	-	-	-	-	-	-	-	-	-	-	-	-	-	-	-	-	-	-	-	-	-	-	-	-	-	-	-
		-	-	-	-	-	-	-	-	-	-	-	-	193279	-	-	-	-	-	-	-	-	-	-	-	-	-	-	-	-	-	-	-	-	-	-	-	-	-	-	-	-	-	-	-	-	-	-	-	-	-	-
		-	-	-	-	-	-	-	-	-	-	-	-	193269	-	-	CMN115C	-	-	-	-	-	-	-	-	-	-	-	-	-	-	-	10557	-	44888	-	-	-	-	-	-	-	-	-	-	-	-	-	-	-	-	-
		-	-	-	-	-	-	-	-	-	-	-	-	193268	-	-	-	-	-	-	-	-	-	-	-	-	-	-	-	-	-	-	-	-	-	-	-	-	-	-	-	-	-	-	-	-	-	-	-	-	-	-
		-	-	-	-	-	-	-	-	-	-	-	100271	193262	-	-	-	-	-	-	-	-	-	-	-	-	-	-	-	-	-	-	-	-	-	-	-	-	-	-	-	-	-	-	-	-	-	-	-	-	-	-
		-	+	-	-	-	-	GB13204-PA	-	9162	-	-	219532	193261	274300	-	-	OTTDARP00000020022	-	-	-	-	ENSGALP00000007694	-	OTTHUMP00000033405	-	109899	-	-	157648	-	-	-	-	-	173127	-	-	594655	-	-	-	-	SINFRUP00000135701	-	268817	-	-	19102	-	-	-
GAE1_CHLRE		-	+	+	-	-	-	GB11815-PA	AT4G10960.1	-	-	CE38295	95741	193254	389690	cgd4_2600	CMA041C	OTTDARP00000023985	DDB0217631	CG12030-PA	-	226.m00073	ENSGALP00000006431	-	OTTHUMP00000002991	LmjF33.2300	128420	18222	-	237812	NCU04442	Os05g51670.1	22913	GSPATP00000765001	46785	187084	-	-	815032	RO3G_05148.1	YBR019C	SPBC365.14c	-	SINFRUP00000136988	10.m00382	35532	-	42.m03458	57929	86507.m00070	Tb11.02.0330	UM06057.1
		-	+	-	-	-	-	GB17887-PA	AT3G03710.1	59314	-	CE39207	172563	193253	-	-	CMH146C	-	-	-	-	-	ENSGALP00000012877	-	NP_149100.1	-	-	34325	-	30443	-	Os07g07310.2	8234	-	20608	151398	136494	-	241943	-	-	-	GLEAN3_21797	SINFRUP00000129340	-	268644	-	-	57060	-	-	-
		-	-	-	-	-	-	-	-	-	-	-	-	193250	-	-	-	-	-	-	-	-	-	-	-	-	-	-	-	-	-	-	-	-	-	-	-	-	-	-	-	-	-	-	-	-	-	-	-	-	-	-
		-	-	-	-	-	-	-	-	-	-	-	-	193246	-	-	-	-	-	-	-	-	-	-	-	-	-	-	-	-	-	-	-	-	-	-	-	-	-	-	-	-	-	-	-	-	-	-	-	-	-	-
		-	-	-	-	-	-	-	-	-	-	-	-	193238	-	-	-	-	-	-	-	-	-	-	-	-	-	-	-	-	-	-	-	-	-	-	-	-	-	-	-	-	-	-	-	-	-	-	-	-	-	-

		-	-	-	-	-	-	-	-	-	-	-	-	193234	-	-	-	-	-	-	-	-	-	-	-	-	-	-	-	-	-	-	-	-	-	-	-	-	-	-	-	-	-	-	-	-	-	-	-	-	-	-
		-	+	-	-	-	-	GB20122-PA	-	62439	-	-	-	193232	-	cgd1_1520	-	-	-	-	-	-	ENSGALP00000008521	-	NP_005551.3	-	-	-	75241	70349	-	-	-	-	-	-	-	-	-	-	-	-	-	-	-	-	-	-	-	-	-	-
		-	-	-	-	-	-	-	-	-	-	-	-	193223	-	-	-	-	-	-	-	-	-	-	-	-	-	-	-	-	-	-	-	-	-	-	-	-	-	-	-	-	-	-	-	-	-	-	-	-	-	-
		-	-	-	-	-	-	-	-	-	-	-	-	193222	-	-	-	-	-	-	-	-	-	-	-	-	-	-	-	-	-	-	24053	-	-	-	-	-	-	-	-	-	-	-	-	-	-	-	-	-	-	-
	putative proline oxidase	-	-	-	-	-	-	-	-	-	-	-	-	193221	-	-	-	-	-	-	-	-	-	-	-	-	-	-	-	-	-	-	-	-	-	-	-	-	-	-	-	-	-	-	-	-	-	-	-	-	-	-
		-	-	-	-	-	-	-	-	-	-	-	-	193220	-	-	-	-	-	-	-	-	-	-	-	-	-	-	-	-	-	-	-	-	-	-	-	-	-	-	-	-	-	-	-	-	-	-	-	-	-	-
		-	-	-	-	-	-	-	-	67127	-	-	-	193218	-	-	-	-	-	-	-	-	-	-	-	-	-	-	-	-	-	-	-	-	-	-	-	-	-	-	-	-	-	-	-	-	-	-	-	-	-	-
		-	-	-	-	-	-	-	-	-	-	-	-	193217	-	-	-	-	-	-	-	-	-	-	-	-	-	-	-	-	-	-	-	-	-	-	-	-	-	-	-	-	-	-	-	-	-	-	-	-	-	-
		-	-	-	-	-	-	-	-	-	-	-	-	193216	-	-	-	-	-	-	-	-	-	-	-	-	-	-	-	-	-	-	-	-	-	-	-	-	-	-	-	-	-	-	-	-	-	-	-	-	-	-
		-	-	-	-	-	-	-	-	-	-	-	-	193213	-	-	-	-	-	-	-	-	-	-	-	-	-	-	-	-	-	-	-	-	-	-	-	-	-	-	-	-	-	-	-	-	-	-	-	-	-	-
		-	-	-	-	-	-	-	-	-	-	-	-	193211	-	-	-	-	-	-	-	-	-	-	-	-	-	-	-	-	-	-	-	-	-	-	-	-	-	-	-	-	-	-	-	-	-	-	-	-	-	-
MAPKKK13	MAP3K13; Mitogen Activated Protein Kinase Kinase Kinase Homolog 13	-	-	-	-	-	-	-	-	-	-	-	-	193209	-	-	-	-	-	-	-	-	-	-	-	-	-	-	-	-	-	-	-	-	-	-	-	-	-	-	-	-	-	-	-	-	-	-	-	-	-	-
		-	-	-	-	-	-	-	-	-	-	-	-	193208	-	-	-	-	-	-	-	-	-	-	-	-	-	-	-	-	-	-	-	-	-	-	-	-	-	-	-	-	-	-	-	-	-	-	-	-	-	-
		-	-	-	-	-	-	-	-	-	-	-	-	193207	-	-	-	-	-	-	-	-	-	-	-	-	-	-	-	-	-	-	-	GSPATP00027544001	-	-	-	-	-	-	-	-	-	-	-	-	-	-	-	-	-	-
		-	-	-	-	-	-	-	-	-	-	-	-	193206	-	-	-	-	-	-	-	-	-	-	-	-	-	-	-	-	-	-	-	-	-	-	-	-	-	-	-	-	-	-	-	-	-	-	-	-	-	-
		-	-	-	-	-	-	-	-	-	-	-	-	193203	-	-	-	-	-	-	-	-	-	-	-	-	-	-	-	-	-	-	-	-	-	-	-	-	-	-	-	-	-	-	-	-	-	-	-	-	-	-
		-	-	-	-	-	-	-	-	-	-	-	-	193202	-	-	-	-	-	-	-	-	-	-	-	-	-	-	-	-	-	-	-	-	-	-	-	-	-	-	-	-	-	-	-	-	-	-	-	-	-	-
CAX5	CAX family of Cation antiporters, membrane protein	-	-	-	-	-	-	-	-	-	-	-	-	193200	-	-	-	-	-	-	-	-	-	-	-	-	-	-	-	-	-	-	-	-	-	-	-	-	-	-	-	-	-	-	-	-	-	-	-	-	-	-
		-	-	-	-	-	-	-	-	-	-	-	-	193199	-	-	-	-	-	-	-	-	-	-	-	-	-	-	-	-	-	-	-	-	-	-	-	-	-	-	-	-	-	-	-	-	-	-	-	-	-	-
		-	+	-	-	-	-	GB16103-PA	AT1G72320.3	-	-	-	-	193198	-	-	-	-	DDB0217770	CG11123-PA	-	-	-	-	-	-	234897	36078	-	-	-	Os10g25110.1	31270	-	-	171461	143715	-	553831	RO3G_14886.1	YJL010C	-	-	SINFRUP00000155363	-	-	-	-	-	-	-	-
		-	+	-	-	-	-	-	AT4G20130.1	-	BDEG_06713	-	-	193195	-	-	-	-	-	-	-	-	-	-	-	-	-	-	-	-	NCU06658	-	35619	-	-	137034	-	-	-	-	-	-	-	-	-	-	-	-	-	-	-	-
		-	-	-	-	-	-	-	-	-	-	-	-	193194	-	-	-	-	-	-	-	-	-	-	-	-	-	-	-	-	-	-	-	-	-	-	-	-	-	-	-	-	-	-	-	-	-	-	-	-	-	-
FAP219	Flagellar Associated Protein	-	-	-	+	-	-	-	-	-	-	-	-	193182	-	-	-	-	-	-	-	-	-	-	-	-	-	-	-	-	-	-	-	-	-	-	-	-	-	-	-	-	-	-	-	-	-	-	-	-	-	-
		-	-	-	-	-	-	-	-	-	-	-	-	193176	-	-	-	-	-	-	-	-	-	-	-	-	-	-	-	-	-	-	-	-	-	-	-	-	-	-	-	-	-	-	-	-	-	-	-	-	-	-
RPA70b	Replication protein A 70 kDa DNA-binding subunit, single-strand DNA binding protein (SSB)	-	-	-	-	-	-	-	AT5G61000.1	-	-	-	-	193171	-	-	-	-	-	-	-	-	-	-	-	-	-	-	-	-	-	-	-	-	-	-	-	-	783062	-	-	-	-	-	-	-	-	-	-	-	-	-
CYC6	cytochrome c6, chloroplast precursor	-	-	-	-	-	-	-	-	23725	-	-	-	193170	-	-	CMV209C	-	-	-	-	-	-	-	-	-	-	-	-	-	-	-	-	-	44056	-	-	-	-	-	-	-	-	-	-	35934	-	-	-	-	-	-
		-	-	-	-	-	-	-	-	-	-	-	-	193168	-	-	-	-	-	-	-	-	-	-	-	-	-	-	-	-	-	-	-	-	-	-	-	-	-	-	-	-	-	-	-	-	-	-	-	-	-	-
		-	-	-	-	-	-	-	-	-	-	-	-	193167	-	-	-	-	-	-	-	-	-	-	-	-	-	-	-	-	-	-	-	-	-	-	-	-	-	-	-	-	-	-	-	-	-	-	-	-	-	-
		-	-	-	-	-	-	-	-	-	-	-	-	193165	-	-	-	-	-	-	-	-	-	-	-	-	-	-	-	-	-	-	-	-	-	-	-	-	-	-	-	-	-	-	-	-	-	-	-	-	-	-
		-	-	-	-	-	-	-	-	-	-	-	-	193155	-	-	-	-	-	-	-	-	-	-	-	-	-	-	-	-	-	-	-	-	-	-	-	-	-	-	-	-	-	-	-	-	-	-	-	-	-	-
FAP183	Flagellar Associated Protein	-	-	-	+	-	-	-	-	-	-	-	-	193154	-	-	-	-	-	-	-	-	-	-	-	-	-	-	-	-	-	-	-	-	-	-	-	-	-	-	-	-	-	-	-	-	-	-	-	-	-	-
		-	-	-	-	-	-	-	-	-	-	-	-	193151	-	-	-	-	-	-	-	-	-	-	-	-	-	-	-	-	-	-	-	-	-	-	-	-	-	-	-	-	-	-	-	-	-	-	-	-	-	-
		-	-	-	-	-	-	-	-	-	-	-	-	193150	-	-	-	-	-	-	-	-	-	-	-	-	-	-	-	-	-	-	-	-	-	-	-	-	-	-	-	-	-	-	-	-	-	-	-	-	-	-
		-	-	-	-	-	-	-	-	-	-	-	-	193149	-	-	-	-	-	-	-	-	-	-	-	-	-	-	-	-	-	-	-	-	-	-	-	-	-	-	-	-	-	-	-	-	-	-	-	-	-	-
		-	+	-	-	-	-	GB12080-PA	AT5G54900.1	28594	BDEG_02746	CE32131	-	193148	-	-	-	OTTDARP00000016420	DDB0183859	-	-	-	ENSGALP00000022466	-	OTTHUMP00000160047	-	92320	-	2097	20744	NCU00768	Os07g33330.1	37422	-	-	2573	113783	-	727110	RO3G_01606.1	YHR086W	SPAC17A2.09c	-	SINFRUP00000180877	-	-	-	57.m01807	32433	102962.m00004	-	UM01271.1
		-	+	-	-	-	-	-	AT2G04740.1	-	-	-	-	193146	243560	-	-	-	-	-	-	-	-	-	-	-	-	-	74902	-	NCU05044	Os06g21330.1	-	-	-	-	-	-	-	-	-	-	-	SINFRUP00000128854	-	-	-	-	-	-	-	-
		-	-	-	-	-	-	-	-	-	-	-	-	193144	-	-	-	-	-	-	-	-	-	-	-	-	-	-	-	-	-	-	-	-	-	-	-	-	-	-	-	-	-	-	-	-	-	-	-	-	-	-
		-	-	-	-	-	-	-	-	-	-	-	-	193143	-	-	-	-	-	-	-	-	-	-	-	-	-	-	-	-	-	-	-	-	-	-	-	-	-	-	-	-	-	-	-	-	-	-	-	-	-	-
		-	-	-	-	-	-	-	-	-	-	-	-	193137	-	-	-	-	-	-	-	-	-	-	-	-	-	-	-	-	-	-	-	-	-	-	-	-	-	-	-	-	-	-	-	-	-	-	-	-	-	-
		-	-	-	-	-	-	-	-	-	-	-	-	-	-	-	-	-	-	-	-	-	-	-	-	-	-	-	-	-	-	-	-	-	-	-	-	-	-	-	-	-	-	-	-	-	-	-	-	-	-	-
FAP63	Flagellar Associated Protein	-	-	-	+	-	-	-	-	-	-	-	-	193134	-	-	-	-	-	-	-	-	-	-	-	-	-	-	-	-	-	-	-	-	-	-	-	-	-	-	-	-	-	-	-	-	-	-	-	-	-	-
		-	-	-	-	-	-	-	-	-	-	-	-	193133	-	-	-	-	-	-	-	-	-	-	-	-	-	-	-	-	-	-	-	-	-	-	-	-	-	-	-	-	-	-	-	-	-	-	-	-	-	-
		-	-	-	-	-	-	-	-	-	-	-	-	193128	-	-	-	-	-	-	-	-	-	-	-	-	-	-	-	-	-	-	-	-	-	-	-	-	-	-	-	-	-	-	-	-	-	-	-	-	-	-
		-	-	-	-	-	-	-	-	-	-	-	-	193126	-	-	-	-	-	-	-	-	-	-	-	-	-	-	-	-	-	-	-	-	-	-	-	-	-	-	-	-	-	-	-	-	-	-	-	-	-	-
		-	-	-	-	-	-	-	-	-	-	-	-	193124	-	-	-	-	-	-	-	-	-	-	-	-	-	-	-	-	-	-	-	-	-	-	-	-	-	-	-	-	-	-	-	-	-	-	-	-	-	-
		-	-	-	-	-	-	-	-	-	-	-	-	193123	-	-	-	-	DDB0167562	-	-	-	-	-	-	-	-	-	-	-	-	-	11101	-	36109	-	-	-	-	-	-	-	-	-	-	-	-	-	-	-	-	-
	pentapeptide repeats; possible thylakoid lumen localization	-	+	-	-	-	-	-	-	-	-	-	-	193119	243525	-	-	OTTDARP00000023463	-	-	-	-	-	-	-	-	-	-	48585	-	-	-	6628	-	-	-	-	-	-	-	-	-	-	SINFRUP00000170106	-	-	-	-	-	-	-	-
		-	-	-	-	-	-	-	-	-	-	-	-	193116	-	-	-	-	-	-	-	-	-	-	-	-	-	-	-	-	-	-	-	-	-	-	-	-	-	-	-	-	-	-	-	-	-	-	-	-	-	-
		-	-	-	-	-	-	-	-	-	-	-	-	193109	-	-	-	-	-	-	-	-	-	-	-	-	-	-	-	-	-	-	-	-	-	-	-	-	-	-	-	-	-	-	-	-	-	-	-	-	-	-
		-	-	-	-	-	-	-	-	-	-	-	-	193106	-	-	-	-	-	-	-	-	-	-	-	-	-	-	-	-	-	-	-	-	-	-	-	-	-	-	-	-	-	-	-	-	-	-	-	-	-	-
STM2		-	-	-	-	-	-	-	-	-	-	-	-	193103	-	-	-	-	-	-	-	-	-	-	-	-	-	-	-	-	-	-	-	-	-	-	-	-	-	-	-	-	-	-	-	-	-	-	-	-	-	-
		-	-	-	-	-	-	-	-	-	-	-	-	193096	-	-	-	-	-	-	-	-	-	-	-	-	-	-	-	-	-	-	-	-	-	-	-	-	-	-	-	-	-	-	-	-	-	-	-	-	-	-
	expressed gene of unknown function	-	-	-	-	-	-	-	-	-	-	-	-	193090	-	-	-	-	-	-	-	-	-	-	-	-	-	-	-	-	-	-	-	-	-	-	-	-	-	-	-	-	-	-	-	-	-	-	-	-	-	-
		-	-	-	-	-	-	-	-	-	-	-	-	193088	-	-	-	-	-	-	-	-	-	-	-	-	-	-	-	128003	-	-	-	-	-	-	-	-	-	-	-	-	-	-	-	-	-	-	-	-	-	-
		-	+	-	-	-	-	-	AT1G14030.1	-	BDEG_03530	-	-	193086	-	-	CMJ178C	-	DDB0190540	-	-	-	ENSGALP00000018126	-	OTTHUMP00000067230	-	118649	-	-	-	-	Os09g24530.1	21349	GSPATP00020579001	-	156277	132933	-	421131	-	-	-	-	-	-	5135	-	-	-	-	-	-
		-	-	-	-	-	-	-	-	-	-	-	-	193085	-	-	-	-	-	-	-	-	-	-	-	-	-	-	-	-	-	-	-	-	-	-	-	-	-	-	-	-	-	-	-	-	-	-	-	-	-	-
		-	-	-	-	-	-	-	-	-	-	-	-	193079	-	-	-	-	-	-	-	-	-	-	-	-	-	-	-	-	-	-	-	-	-	-	-	-	-	-	-	-	-	-	-	-	-	-	-	-	-	-
		-	-	-	-	-	-	-	-	-	-	-	-	193075	-	-	-	-	-	-	-	-	-	-	-	-	-	-	-	-	-	-	-	-	-	-	-	-	-	-	-	-	-	-	-	-	-	-	-	-	-	-
		-	-	-	-	-	-	-	-	-	-	-	-	193074	-	-	-	-	-	-	-	-	-	-	-	-	-	-	-	-	-	-	-	-	-	-	-	-	-	-	-	-	-	-	-	-	-	-	-	-	-	-
		-	-	-	-	-	-	-	-	-	-	-	-	193072	-	-	-	-	-	-	-	-	-	-	-	-	-	-	-	-	-	-	-	-	-	-	-	-	-	-	-	-	-	-	-	-	-	-	-	-	-	-
		-	-	-	-	-	-	-	-	-	-	-	-	193064	-	-	-	-	-	-	-	-	-	-	-	-	-	-	-	-	-	-	-	-	-	-	-	-	-	-	-	-	-	-	-	-	-	-	-	-	-	-
		-	-	-	-	-	-	-	-	-	-	-	-	193060	-	-	-	-	-	-	-	-	-	-	-	-	-	-	-	-	-	-	-	-	-	-	-	-	-	-	-	-	-	-	-	-	-	-	-	-	-	-
		-	-	-	-	-	-	-	-	-	-	-	-	193059	-	-	-	-	-	-	-	-	-	-	-	-	-	-	-	-	-	-	-	-	-	-	-	-	-	-	-	-	-	-	-	-	-	-	-	-	-	-
		-	-	-	-	-	-	-	AT5G46840.2	-	-	-	-	193055	-	-	-	-	-	-	-	-	-	-	-	-	-	-	-	-	-	Os03g63730.1	30189	-	-	-	-	-	-	-	-	-	-	-	-	-	-	-	-	-	-	-
		-	-	-	-	-	-	-	-	-	-	-	-	193054	-	-	-	-	-	-	-	-	-	-	-	-	-	-	-	-	-	-	-	-	-	-	-	-	-	-	-	-	-	-	-	-	-	-	-	-	-	-
		-	-	-	-	-	-	-	-	-	-	-	-	193050	-	-	-	-	-	-	-	-	-	-	-	-	-	-	-	-	-	-	-	-	-	-	-	-	-	-	-	-	-	-	-	-	-	-	-	-	-	-
		-	+	+	-	-	-	-	AT4G11980.1	5776	-	CE18314	161037	193044	-	-	-	-	DDB0204862	-	-	185.m00093	ENSGALP00000022598	-	OTTHUMP00000019113	LmjF36.3660	204741	16883	-	-	NCU00548	Os06g03910.1	35058	GSPATP00018197001	-	-	-	-	-	-	YBR111C	SPBP35G2.12	-	-	8.m00384	262610	TA10150	74.m00461	-	-	Tb11.01.1570	UM01764.1
		-	-	-	-	-	-	-	-	-	-	-	-	193040	-	-	-	-	-	-	-	-	-	-	-	-	-	-	-	-	-	-	-	-	-	-	-	-	-	-	-	-	-	-	-	-	-	-	-	-	-	-
		-	-	-	-	-	-	-	-	-	-	-	-	193039	-	-	-	-	-	-	-	-	-	-	-	-	-	-	-	-	-	-	-	-	-	-	-	-	-	-	-	-	-	-	-	-	-	-	-	-	-	-
CPI		-	+	-	-	-	-	-	AT5G50375.1	23404	-	-	-	193038	-	-	-	-	DDB0202318	-	-	-	-	-	-	-	-	8620	81142	-	-	Os11g19700.1	17009	-	49447	206696	-	-	252617	-	-	-	-	-	-	7708	-	-	-	-	-	-
		-	-	-	-	-	-	-	-	-	-	-	-	193036	-	-	-	-	-	-	-	-	-	-	-	-	-	-	-	-	-	-	-	-	-	-	-	-	-	-	-	-	-	-	-	-	-	-	-	-	-	-
		-	-	-	-	-	-	-	-	68888	-	-	-	193033	-	-	-	-	-	-	-	-	-	-	-	-	-	-	-	-	-	-	-	-	-	-	-	-	231462	-	-	-	-	-	-	-	-	-	-	-	-	-
		-	-	-	-	-	-	-	-	-	-	-	-	193031	-	-	-	-	-	-	-	-	-	-	-	-	-	-	-	-	-	-	-	-	-	-	-	-	-	-	-	-	-	-	-	-	-	-	-	-	-	-
		-	-	-	-	-	-	-	-	-	-	-	-	193029	-	-	-	-	-	-	-	-	-	-	-	-	231852	11218	-	246103	-	-	-	-	-	-	-	-	-	-	-	-	-	-	-	-	-	-	-	-	-	-
ALA3	ATPase, phospholipid transporter	-	+	-	-	-	-	GB15437-PA	-	-	-	-	-	193025	-	-	-	-	-	-	-	-	-	-	-	LmjF13.1530	-	32498	-	-	-	-	-	-	-	-	-	-	-	-	-	-	-	-	-	-	-	42.m03340	-	-	Tb11.02.0850	-
SQD1		-	-	-	-	-	-	-	-	-	-	-	-	-	-	-	-	-	-	-	-	-	-	-	-	-	-	-	-	-	-	-	-	-	-	-	-	-	-	-	-	-	-	-	-	-	-	-	-	-	-	-
		-	-	-	-	-	-	-	-	-	-	-	-	193021	-	-	-	-	-	-	-	-	-	-	-	-	-	-	-	-	-	-	-	-	-	-	-	-	-	-	-	-	-	-	-	-	-	-	-	-	-	-
		-	+	-	-	-	-	GB12372-PA	-	37260	-	CE36022	182946	193019	283452	-	-	OTTDARP00000022576	DDB0187674	CG11537-PA	-	-	ENSGALP00000008535	-	OTTHUMP00000012456	-	228011	38869	-	182583	NCU08292	Os11g05390.1	-	-	45558	-	-	-	822713	RO3G_04707.1	-	-	-	SINFRUP00000144376	-	262480	-	212.m00014	28065	-	-	UM03554.1
		-	-	-	-	-	-	-	-	-	-	-	-	193018	-	-	-	-	-	-	-	-	-	-	-	-	-	-	-	-	-	-	-	-	-	-	-	-	-	-	-	-	-	-	-	-	-	-	-	-	-	-
TRP-2		-	-	-	-	-	-	-	-	71262	-	-	-	193017	-	-	-	-	-	-	-	-	-	-	-	-	-	-	-	-	-	-	-	-	-	-	131712	-	-	-	-	-	-	-	-	-	-	-	-	-	-	-
		-	-	-	-	-	-	-	-	-	-	-	-	193014	-	-	-	-	-	-	-	-	-	-	-	-	-	-	-	-	-	-	-	-	-	-	-	-	-	-	-	-	-	-	-	-	-	-	-	-	-	-
		-	-	-	-	-	-	-	-	-	-	-	169948	193011	-	-	-	-	-	-	-	-	-	-	-	-	232560	-	-	-	-	-	-	-	-	-	143318	-	-	-	-	-	-	-	-	-	-	-	30433	-	-	-
ELG20	exostosin-like glycosyltransferase	-	-	-	-	-	-	-	-	-	-	-	-	193010	-	-	-	-	-	-	-	-	-	-	-	-	-	-	-	-	-	-	-	-	-	-	-	-	-	-	-	-	-	-	-	-	-	-	1026	-	-	-
MCCA	methylcrotonoyl-CoA carboxylase subunit alpha, mitochondrial precursor	-	+	-	+	-	-	-	AT1G03090.2	71848	BDEG_08389	CE35517	161178	193008	207716	-	-	OTTDARP00000011423	DDB0187457	-	-	-	ENSGALP00000032512	-	OTTHUMP00000041176	LmjF31.3130	103911	21541	79559	162924	NCU00591	Os12g41250.2	-	GSPATP00001876001	843	176782	109683	PF14_0664	559591	RO3G_06576.1	-	-	GLEAN3_24071	SINFRUP00000144429	63.m00241	263480	-	-	18834	-	Tb927.8.6970	UM04382.1
		-	-	-	-	-	-	-	-	-	-	-	-	193006	-	-	-	-	-	-	-	-	-	-	-	-	-	-	-	-	-	-	-	-	-	-	-	-	-	-	-	-	-	-	-	-	-	-	-	-	-	-
		-	-	-	-	-	-	-	-	-	-	-	-	193005	-	-	-	-	-	-	-	-	-	-	-	-	-	-	-	-	-	-	33271	-	-	-	-	-	-	-	-	-	-	-	-	-	-	-	-	-	-	-
		-	-	-	-	-	-	-	-	-	-	-	-	193002	-	-	-	-	-	-	-	-	-	-	-	-	-	-	-	-	-	-	-	-	-	-	-	-	-	-	-	-	-	-	-	-	-	-	-	-	-	-
		-	-	-	-	-	-	-	-	-	-	-	-	192999	-	-	-	-	-	-	-	-	-	-	-	-	-	-	-	-	-	-	-	-	-	-	-	-	-	-	-	-	-	-	-	-	-	-	-	-	-	-
		-	-	-	-	-	-	-	-	-	-	-	-	192997	-	-	-	-	-	-	-	-	-	-	-	-	-	-	-	-	-	-	-	-	-	-	-	-	-	-	-	-	-	-	-	-	-	-	-	-	-	-
		-	-	-	-	-	-	-	-	-	-	-	-	192992	-	-	-	-	-	-	-	-	-	-	-	-	-	-	-	-	-	-	-	-	-	-	-	-	-	-	-	-	-	-	-	-	-	-	-	-	-	-
		-	+	-	-	-	-	-	-	-	BDEG_08080	-	-	192990	-	cgd1_1550	-	-	DDB0192000	-	-	1.m00674	-	-	-	LmjF35.4160	-	32701	80865	-	-	-	8527	-	41811	-	109343	MAL8P1.103	-	RO3G_10287.1	-	SPAC22H10.08	-	-	-	-	TA11040	65.m00027	-	-	Tb09.211.2100	-
PRL3		-	+	-	-	-	-	-	-	-	-	CE34797	178888	192988	246859	-	-	-	-	CG16995-PA	-	-	-	-	OTTHUMP00000168532	-	-	-	-	82002	-	Os12g43700.1	-	-	-	-	-	-	-	RO3G_04084.1	YJL079C	-	-	SINFRUP00000136612	-	-	-	-	-	-	-	UM01204.1
FAS7	fasciclin-like protein	-	-	-	+	-	-	-	-	-	-	-	-	192980	-	-	-	-	-	-	-	-	-	-	-	-	-	-	-	-	-	-	-	-	-	-	-	-	-	-	-	-	-	-	-	-	-	-	-	-	-	-
		-	-	-	-	-	-	-	-	-	-	-	-	192979	-	-	-	-	-	-	-	-	-	-	-	-	-	-	-	-	-	-	-	-	-	-	-	-	-	-	-	-	-	-	-	-	-	-	-	-	-	-
		-	-	-	-	-	-	-	-	-	-	-	-	192974	-	-	-	-	-	-	-	-	-	-	-	-	-	-	-	-	-	-	-	-	-	-	-	-	-	-	-	-	-	-	-	-	-	-	-	-	-	-
FAP236	Conserved Uncharacterized Flagellar Associated Protein	-	+	-	+	-	-	GB17761-PA	-	-	-	CE13447	117250	192970	245160	-	-	-	-	-	-	-	ENSGALP00000009860	-	-	-	232357	-	-	51828	-	-	-	-	-	-	-	PFI0460w	-	-	-	-	-	-	-	-	-	-	58739	-	-	-
		-	-	-	-	-	-	-	AT1G42960.1	-	-	-	-	192969	-	-	-	-	-	-	-	-	-	-	-	-	-	-	-	-	-	-	-	-	-	169954	-	-	819930	-	-	-	-	-	-	-	-	-	-	-	-	-
PRL8		-	+	-	-	-	-	GB14341-PA	-	-	-	-	-	192965	211796	-	-	-	-	-	-	-	-	-	-	-	-	-	67368	221063	-	Os01g28450.1	-	-	-	-	-	-	-	-	-	-	-	-	-	-	-	49.m00025	-	-	-	-
		-	-	-	-	-	-	-	-	-	-	-	-	192959	-	-	-	-	-	-	-	-	-	-	-	-	-	-	-	-	-	-	-	-	-	-	-	-	-	-	-	-	-	-	-	-	-	-	-	-	-	-
		-	-	-	-	-	-	-	-	-	-	-	-	192947	-	-	-	-	-	-	-	-	-	-	-	-	-	-	-	-	-	-	-	-	-	-	-	-	-	-	-	-	-	-	-	-	-	-	-	-	-	-
		-	-	-	-	-	-	-	-	-	-	-	-	192946	-	-	-	-	-	-	-	-	-	-	-	-	-	-	-	-	-	-	-	-	-	-	-	-	-	-	-	-	-	-	-	-	-	-	-	-	-	-
		-	-	-	-	-	-	-	-	-	-	-	-	192944	-	-	-	-	-	-	-	-	-	-	-	-	-	-	-	-	-	-	-	-	-	-	-	-	-	-	-	-	-	-	-	-	-	-	-	-	-	-
		-	-	-	-	-	-	-	-	-	-	-	-	192939	-	-	-	-	-	-	-	-	-	-	-	-	-	-	-	-	-	-	-	-	-	158207	-	-	590641	-	-	-	-	-	-	-	-	-	-	-	-	-
GOX18	putative glyoxal or galactose oxidase	-	+	-	-	-	-	-	-	-	-	-	-	192937	-	-	-	-	-	-	-	-	-	-	-	-	-	-	-	-	-	Os05g48520.1	-	-	49706	190327	-	-	-	RO3G_11035.1	-	-	-	-	-	-	-	-	-	-	-	UM02411.1
		-	-	-	-	-	-	-	-	-	-	-	-	192935	-	-	-	-	-	-	-	-	-	-	-	-	-	-	-	-	-	-	-	-	-	-	-	-	-	-	-	-	-	-	-	-	-	-	-	-	-	-
RPN8	26S proteasome regulatory subunit RPN8	-	+	+	-	-	-	GB18287-PA	AT3G11270.1	32049	BDEG_07618	CE18135	226208	192933	396734	cgd7_2900	CMT431C	-	DDB0205949	CG3416-PA	19068761	326.m00041	ENSGALP00000000999	GL50803_7896	OTTHUMP00000081414	LmjF32.0390	191571	37751	59054	161920	NCU01547	Os04g56646.1	5270	GSPATP00026763001	17259	193347	108965	PFI0630w	820395	RO3G_05875.1	YOR261C	SPCC1682.10	-	SINFRUP00000136251	24.m00265	40962	TA07380	59.m03441	23419	80746.m00307	Tb10.61.2180	UM01121.1
		-	-	-	-	-	-	-	-	-	-	-	-	192930	-	-	-	-	-	-	-	-	-	-	-	-	-	-	-	-	-	-	-	-	-	-	-	-	-	-	-	-	-	-	-	-	-	-	-	-	-	-
		-	+	-	-	-	-	GB14597-PA	AT5G15540.1	63990	-	CE36034	160616	192929	378732	-	-	OTTDARP00000019244	DDB0185354	CG17704-PE	-	-	ENSGALP00000005698	-	NP_056199.2	-	214826	-	61097	10114	NCU05250	Os07g01940.1	31065	-	50138	-	-	-	577738	-	-	SPAC31A2.05c	-	SINFRUP00000132715	-	21122	-	-	57843	-	-	UM05666.1
		-	-	-	-	-	-	-	-	-	-	-	-	192926	-	-	-	-	-	-	-	-	-	-	-	-	-	-	-	-	-	-	-	-	-	-	-	-	-	-	-	-	-	-	-	-	-	-	-	-	-	-
		-	+	-	-	-	-	GB10738-PA	-	-	-	-	213768	192924	295409	-	-	-	-	-	-	-	ENSGALP00000013340	-	OTTHUMP00000169399	-	160638	-	-	240973	-	-	18754	-	43473	172624	-	-	-	-	-	-	-	SINFRUP00000136299	-	9454	-	-	59192	-	-	-
		-	-	-	-	-	-	-	-	-	-	-	-	192922	-	-	-	-	-	-	-	-	-	-	-	-	-	-	-	-	-	-	-	-	-	-	-	-	-	-	-	-	-	-	-	-	-	-	-	-	-	-
		-	-	-	-	-	-	-	-	-	-	-	-	192918	-	-	-	-	-	-	-	-	-	-	-	-	-	-	-	-	-	-	-	-	-	-	-	-	-	-	-	-	-	-	-	-	-	-	-	-	-	-
		-	-	-	-	-	-	-	-	-	-	-	-	192916	-	-	-	-	-	-	-	-	-	-	-	-	-	-	-	-	-	-	-	-	-	-	-	-	-	-	-	-	-	-	-	-	-	-	-	-	-	-
		+	+	-	-	-	-	-	-	-	-	-	159316	192915	216502	-	-	-	-	-	-	-	ENSGALP00000015652	GL50803_23330	OTTHUMP00000035984	LmjF36.4080	194691	37188	63921	118058	-	-	-	GSPATP00018931001	-	86922	133070	-	-	-	-	-	-	-	270.m00027	-	-	-	51927	83472.m00512	Tb11.01.2120	-
		-	-	-	-	-	-	-	-	-	-	-	-	192914	-	-	-	-	-	-	-	-	-	-	-	-	-	-	-	-	-	-	-	-	-	-	-	-	-	-	-	-	-	-	-	-	-	-	-	-	-	-
		-	-	-	-	-	-	-	-	-	-	-	-	192912	-	-	-	-	-	-	-	-	-	-	-	-	-	-	-	-	-	-	-	-	-	-	-	-	-	-	-	-	-	-	-	4853	-	-	-	-	-	-
GAS28	hydroxyproline-rich glycoprotein; stress-induced	-	-	-	-	-	-	-	-	-	-	-	-	192908	-	-	-	-	-	-	-	-	-	-	-	-	-	-	-	-	-	-	-	-	-	-	-	-	-	-	-	-	-	-	-	-	-	-	-	-	-	-
		-	-	-	-	-	-	-	-	-	-	-	-	192907	-	-	-	-	-	-	-	-	-	-	-	-	-	-	-	-	-	-	-	-	-	-	-	-	-	-	-	-	-	-	-	-	-	-	-	-	-	-
		-	-	-	-	-	-	-	-	-	-	-	-	192903	-	-	-	-	-	-	-	-	-	-	-	-	-	-	-	-	-	-	-	-	-	-	-	-	-	-	-	-	-	-	-	-	-	-	-	-	-	-
HMGB3406	High mobility group protein	-	+	-	-	-	-	GB13747-PA	-	-	-	-	-	192899	-	-	-	-	-	CG12223-PE	19074377	-	-	-	-	LmjF29.0850	-	34324	-	-	-	-	-	-	-	121034	-	PFL0145c	-	-	-	-	-	-	-	-	TA04735	55.m10227	-	-	-	-
		-	-	-	-	-	-	-	-	-	-	-	-	192897	-	-	-	-	-	-	-	-	-	-	-	-	-	-	-	-	-	-	32508	-	-	-	-	-	-	-	-	-	-	-	96.m00141	-	-	-	-	-	-	-
		-	-	-	-	-	-	-	-	-	-	-	-	192894	-	-	-	-	-	-	-	-	-	-	-	-	-	-	-	-	-	-	-	-	-	-	-	-	-	-	-	-	-	-	-	-	-	-	-	-	-	-
		-	+	-	-	+	-	GB17418-PA	AT2G47600.1	28918	-	CE23275	128764	192884	270154	-	-	OTTDARP00000018696	-	CG17167-PB	-	-	ENSGALP00000032666	-	OTTHUMP00000042072	-	92127	29670	-	91794	-	Os11g43860.1	35614	-	17199	140132	128640	-	245784	-	-	-	GLEAN3_26183	SINFRUP00000155044	-	4973	-	-	53617	-	-	-
		-	-	-	-	-	-	-	-	-	-	-	-	192882	-	-	-	-	-	-	-	-	-	-	-	-	-	-	-	-	-	-	-	-	-	-	-	-	-	-	-	-	-	-	-	-	-	-	-	-	-	-
		-	-	-	-	-	-	-	-	-	-	-	-	192880	-	-	-	-	-	-	-	-	-	-	-	-	-	-	-	-	-	Os11g06810.1	-	-	-	-	-	-	732059	-	-	-	-	-	-	-	-	-	-	-	-	-
		-	-	-	-	-	-	-	-	-	-	-	-	192879	-	-	-	-	-	-	-	-	-	-	-	-	-	-	-	-	-	-	-	-	-	-	-	-	-	-	-	-	-	-	-	-	-	-	-	-	-	-
		-	-	-	-	-	-	-	-	-	-	-	-	192869	-	-	-	-	-	-	-	-	-	-	-	-	-	-	-	-	-	-	-	-	-	-	-	-	-	-	-	-	-	-	-	-	-	-	-	-	-	-
		-	-	-	-	-	-	-	-	-	-	-	-	192866	-	-	-	-	-	-	-	-	-	-	-	-	-	-	-	-	-	-	-	-	-	-	-	-	-	-	-	-	-	-	-	-	-	-	-	-	-	-
		-	-	-	-	-	-	-	-	-	-	-	-	192864	-	-	-	-	-	-	-	-	-	-	-	-	-	-	-	-	-	-	-	-	-	-	-	-	-	-	-	-	GLEAN3_18440	-	-	-	-	-	-	-	-	-
		-	-	-	-	-	-	-	-	-	-	-	-	192861	-	-	-	-	-	-	-	-	-	-	-	-	-	-	-	-	-	-	-	-	-	-	-	-	-	-	-	-	-	-	-	-	-	-	-	-	-	-
		-	-	-	-	-	-	-	-	-	-	-	-	192859	-	-	-	-	-	-	-	-	-	-	-	-	-	-	-	-	-	-	-	-	-	-	-	-	-	-	-	-	-	-	-	-	-	-	-	-	-	-
		-	-	-	-	-	-	-	-	-	-	-	-	192857	-	-	-	-	-	-	-	-	-	-	-	-	-	-	-	-	-	-	35597	-	-	-	-	-	-	-	-	-	-	-	-	-	-	-	-	-	-	-
		-	+	-	-	-	-	-	AT1G75280.1	-	-	-	-	192854	-	-	-	-	-	-	-	-	-	-	-	-	-	36236	72902	-	NCU06945	Os01g01660.1	-	-	31822	-	133755	-	830063	-	-	-	-	-	-	-	-	-	-	-	-	UM01441.1
		-	-	-	-	-	-	-	-	-	-	-	-	192853	-	-	-	-	-	-	-	-	-	-	-	-	-	-	-	-	-	-	-	-	-	-	-	-	-	-	-	-	-	-	-	-	-	-	-	-	-	-
FAP97	Flagellar Associated Protein	-	+	-	+	-	-	-	-	-	-	-	225719	192852	-	-	-	OTTDARP00000022615	-	-	-	-	ENSGALP00000017246	-	NP_065878.1	-	-	7037	-	246462	-	-	-	-	-	-	-	-	-	-	-	-	-	-	-	-	-	-	-	-	-	-
		-	-	-	-	-	-	-	-	-	-	-	-	192850	-	-	-	-	-	-	-	-	-	-	-	-	-	-	-	-	-	-	-	-	-	-	-	-	-	-	-	-	-	-	-	-	-	-	-	-	-	-
		-	+	-	-	-	-	GB14192-PA	AT5G64670.1	-	BDEG_02498	-	-	192847	-	-	CMC132C	-	DDB0185996	CG5219-PA	-	-	-	-	-	LmjF16.0010	-	2372	81542	94717	-	Os08g21840.1	5143	GSPATP00035112001	5769	17054	133205	-	269601	RO3G_07398.1	-	SPBC9B6.06	-	SINFRUP00000142497	-	17747	TA02775	-	21981	-	Tb927.5.3980	UM04231.1
		-	-	-	-	-	-	-	-	-	-	-	-	192839	-	-	-	-	-	-	-	-	-	-	-	-	-	-	-	-	-	-	-	-	-	-	-	-	-	-	-	-	-	-	-	-	-	-	-	-	-	-
		-	-	-	-	-	-	-	-	-	-	-	-	192837	-	-	-	-	-	-	-	-	-	-	-	-	167160	-	-	-	-	-	-	-	-	-	-	-	-	-	-	-	-	-	-	-	-	-	-	-	-	-
		-	-	-	-	-	-	-	-	-	-	-	-	192833	-	-	-	-	-	-	-	-	-	-	-	-	-	-	-	-	-	-	-	-	-	-	-	-	-	-	-	-	-	-	-	-	-	-	-	-	-	-
		-	-	-	-	-	-	-	-	-	-	-	-	192831	-	-	-	-	-	-	-	-	-	-	-	-	-	-	-	-	-	-	-	-	-	-	-	-	-	-	-	-	-	-	-	-	-	-	-	-	-	-
TIM21	TIM21, import inner membrane translocase subunit, mitochondrial	-	+	-	-	-	-	GB19658-PA	AT4G00026.1	-	-	CE25907	176959	192824	-	-	-	-	-	CG7382-PA	-	-	-	-	OTTHUMP00000163746	-	-	23273	-	-	NCU08810	Os03g14890.2	31499	-	-	172526	-	-	591721	-	-	-	-	-	-	-	-	-	-	-	-	-
C_1430012		-	-	-	-	-	-	-	-	-	-	-	-	192823	-	-	-	-	-	-	-	-	-	-	-	-	-	-	-	-	-	-	-	-	-	-	-	-	-	-	-	-	-	-	-	-	-	-	-	-	-	-
		-	+	-	-	-	-	GB19145-PA	AT2G07340.1	-	-	-	-	192821	241862	-	-	OTTDARP00000023951	DDB0187662	-	-	-	ENSGALP00000001394	-	-	-	236136	6895	72173	88977	NCU02970	Os02g26440.1	33928	GSPATP00003995001	-	224777	-	-	831873	RO3G_06254.1	-	-	-	SINFRUP00000150365	-	-	-	-	-	-	-	-
		-	-	-	-	-	-	-	-	-	-	-	-	192815	-	-	-	-	-	-	-	-	-	-	-	-	-	-	-	-	-	-	-	-	-	-	-	-	-	-	-	-	-	-	-	-	-	-	-	-	-	-
		-	+	-	-	-	-	GB12122-PA	-	19051	BDEG_07455	CE05809	177112	192814	287517	-	-	OTTDARP00000023094	DDB0186246	CG3663-PA	-	-	ENSGALP00000000216	-	NP_057132.2	LmjF12.0060	198478	23596	72006	165649	NCU00166	-	-	GSPATP00015228001	27696	158137	108159	-	-	RO3G_11419.1	-	-	-	SINFRUP00000161392	222.m00045	33024	-	-	20253	-	-	UM05757.1
		-	-	-	-	-	-	-	-	-	-	-	-	192813	-	-	-	-	-	-	-	-	-	-	-	-	-	-	-	-	-	-	-	-	-	-	-	-	-	-	-	-	-	-	-	-	-	-	-	-	-	-
		-	-	-	-	-	-	-	-	-	-	-	-	192811	-	-	-	-	-	-	-	-	ENSGALP00000008315	-	-	-	-	-	-	-	-	-	-	-	-	-	-	-	-	-	-	-	-	-	-	-	-	-	-	-	-	-
		-	-	-	+	-	-	-	-	-	-	-	-	192807	-	-	-	-	-	-	-	-	-	-	-	-	-	-	-	-	-	-	-	-	-	-	-	-	-	-	-	-	-	-	-	-	-	-	-	-	-	-
CCPR1	cytochrome c peroxidase	-	+	-	-	-	-	-	-	26696	BDEG_03308	-	-	192806	-	-	-	-	-	-	-	-	-	-	-	LmjF34.0070	-	16194	-	-	NCU03297	Os04g14680.1	28778	-	13174	-	108469	-	821619	RO3G_01594.1	YKR066C	-	-	-	10.m00322	37054	-	-	-	-	Tb09.211.3030	UM02377.1
		-	-	-	-	-	-	-	-	-	-	-	-	192803	-	-	-	-	-	-	-	-	-	-	-	-	-	-	-	-	-	-	-	-	-	-	-	-	-	-	-	-	-	-	-	-	-	-	-	-	-	-
		-	-	-	-	-	-	-	-	-	-	-	-	192802	-	-	-	-	-	-	-	-	-	-	-	-	-	-	-	-	-	-	-	-	43699	-	-	-	-	-	-	-	-	-	-	-	-	-	-	-	-	-
		-	-	-	-	-	-	-	-	-	-	-	-	192800	-	-	-	-	-	-	-	-	-	-	-	-	-	-	-	-	-	-	-	-	-	-	-	-	-	-	-	-	-	-	-	-	-	-	-	-	-	-
		-	-	-	-	-	-	-	-	-	-	-	-	192798	-	-	-	-	-	-	-	-	-	-	-	-	-	-	-	-	NCU03243	-	-	-	-	-	140932	-	-	-	YER019W	-	-	-	-	-	-	-	-	-	-	-
		-	-	-	-	-	-	-	-	-	-	-	-	192797	-	-	-	-	-	-	-	-	-	-	-	-	-	-	-	-	-	-	-	-	-	158713	-	-	-	-	-	-	-	-	-	-	-	-	-	-	-	-
		-	-	-	-	-	-	-	-	-	-	-	132480	192796	-	-	-	OTTDARP00000023072	-	-	-	-	-	-	-	-	-	-	-	-	-	-	-	-	-	-	-	-	-	-	-	-	-	SINFRUP00000139094	-	-	-	-	-	-	-	-

		-	-	-	-	-	-	-	-	-	-	-	-	192795	-	-	-	-	-	-	-	-	-	-	-	-	-	-	-	-	-	-	-	-	-	-	-	-	-	-	-	-	-	-	-	-	-	-	-	-	-	-
		-	-	-	-	-	-	-	-	-	-	-	-	192792	-	-	-	-	-	-	-	-	-	-	-	-	-	-	-	-	-	-	-	-	-	-	-	-	-	-	-	-	-	-	-	-	-	-	-	-	-	-
		-	-	-	-	-	-	-	-	-	-	-	-	192785	-	-	-	-	-	-	-	-	-	-	-	-	-	-	-	-	-	-	-	-	-	-	-	-	-	-	-	-	-	-	-	-	-	-	-	-	-	-
		-	-	-	-	-	-	-	-	-	-	-	-	192781	-	-	-	-	-	-	-	-	-	-	-	-	-	-	-	-	-	Os12g02094.1	-	-	-	-	-	-	-	-	-	-	GLEAN3_08303	-	-	-	-	-	-	-	-	-
		-	-	-	-	-	-	-	-	-	-	-	-	192778	-	-	-	-	-	-	-	-	-	-	-	-	-	-	-	-	-	-	-	-	-	-	-	-	-	-	-	-	-	-	-	-	-	-	-	-	-	-
		-	+	-	+	-	-	-	-	-	BDEG_04577	-	195904	192775	-	-	-	-	-	-	-	-	-	GL50803_13475	-	-	-	-	-	-	-	Os04g52460.1	-	GSPATP00021133001	35556	173340	-	-	824988	-	-	SPAC1F3.06c	-	-	-	-	-	49.m03138	-	83211.m00092	-	-
		-	-	-	-	-	-	-	-	-	-	-	-	192771	-	-	-	-	-	-	-	-	-	-	-	-	-	-	-	-	-	-	-	-	-	-	-	-	-	-	-	-	-	-	-	-	-	-	-	-	-	-
		-	-	-	-	-	-	-	-	-	-	-	-	192768	-	-	-	-	-	-	-	-	-	-	-	-	-	-	-	-	-	-	-	-	-	-	-	-	-	-	-	-	-	-	-	-	-	-	-	-	-	-
		-	-	-	-	-	-	-	-	-	-	-	-	192767	-	-	-	-	-	-	-	-	-	-	-	-	-	-	-	-	-	-	-	-	-	-	-	-	-	-	-	-	-	-	-	-	-	-	-	-	-	-
		-	-	-	-	-	-	-	-	-	-	-	-	192766	-	-	-	-	-	-	-	-	-	-	-	-	-	-	-	-	-	-	-	-	-	-	-	-	-	-	-	-	-	-	-	-	-	-	-	-	-	-
		+	+	-	+	-	-	GB12038-PA	-	-	BDEG_00885	-	190202	192763	240571	-	-	-	-	-	-	-	ENSGALP00000010542	GL50803_134442	OTTHUMP00000080600	LmjF33.1895	130281	-	52938	206132	-	-	-	GSPATP00016539001	-	-	158230	-	-	-	-	-	-	SINFRUP00000151150	67.m00161	20904	-	-	63536	86800.m00105	Tb11.02.0590	-
		-	-	-	-	-	-	-	AT5G13020.1	-	-	-	-	192759	-	-	-	-	-	-	-	-	-	-	-	-	-	-	-	-	-	Os09g10710.4	23456	-	-	-	-	-	413566	-	-	-	-	-	-	-	-	-	-	-	-	-
		-	-	-	-	-	-	-	-	-	-	-	-	192752	-	-	-	-	-	-	-	-	-	-	-	-	-	-	-	-	-	-	-	-	-	-	-	-	-	-	-	-	-	-	-	-	-	-	-	-	-	-
		-	-	-	-	-	-	-	-	-	-	-	-	192748	-	-	-	-	-	-	-	-	-	-	-	-	-	-	-	-	-	-	-	-	-	-	-	-	-	-	-	-	-	-	-	-	-	-	-	-	-	-
		-	-	-	-	-	-	-	-	-	-	-	-	192742	-	-	-	-	-	-	-	-	-	-	-	-	-	-	-	-	-	-	-	-	-	-	-	-	-	-	-	-	-	-	-	-	-	-	-	-	-	-
		-	-	-	-	-	-	-	-	-	-	-	-	192739	-	-	-	-	-	-	-	-	-	-	-	-	-	-	-	-	-	-	-	-	-	-	-	-	-	-	-	-	-	-	-	-	-	-	-	-	-	-
		-	-	-	-	-	-	-	-	-	-	-	-	192738	-	-	-	-	-	-	-	-	-	-	-	-	-	-	-	-	-	-	-	-	-	-	-	-	-	-	-	-	-	-	-	-	-	-	-	-	-	-
		-	-	-	-	-	-	-	-	-	-	-	-	192735	-	-	-	-	-	-	-	-	-	-	-	-	-	-	-	-	-	-	-	-	-	-	-	-	-	-	-	-	-	-	-	-	-	-	-	-	-	-
		-	-	-	-	-	-	-	-	-	-	-	-	192731	-	-	-	-	-	-	-	-	-	-	-	-	-	-	-	-	-	-	-	-	-	-	-	-	-	-	-	-	GLEAN3_24312	-	-	-	-	-	-	-	-	UM05068.1
		-	-	-	-	-	-	-	-	-	-	-	-	192728	-	-	-	-	-	-	-	-	-	-	-	-	-	-	-	-	-	-	-	-	-	-	-	-	-	-	-	-	-	-	-	-	-	-	-	-	-	-
		-	-	-	-	-	-	-	-	-	-	-	-	192727	-	-	-	-	-	-	-	-	-	-	-	-	-	-	-	-	-	-	-	-	-	-	-	-	-	-	-	-	-	-	-	-	-	-	-	-	-	-
THIC	Hydroxymethylpyrimidine phosphate synthase	-	+	-	-	-	-	-	AT2G29630.1	-	-	-	103986	192720	-	-	CMG171C	-	-	-	-	-	-	-	-	-	-	-	-	-	-	Os03g47610.8	-	-	38085	153830	-	-	172390	-	-	-	-	-	-	41733	-	-	-	-	-	-
		-	-	-	-	-	-	-	-	-	-	-	-	192719	-	-	-	-	-	-	-	-	-	-	-	-	-	-	-	-	-	-	-	-	-	-	-	-	-	-	-	-	-	-	-	-	-	-	-	-	-	-
		-	-	-	-	-	-	-	-	-	-	-	-	192718	-	-	-	-	-	-	-	-	-	-	-	-	-	-	-	-	-	-	-	-	-	-	-	-	-	-	-	-	-	-	-	-	-	-	-	-	-	-
		-	-	-	-	-	-	-	-	-	-	-	-	192712	-	-	-	-	-	-	-	-	-	-	-	-	-	-	-	-	-	-	-	-	-	-	-	-	-	-	-	-	-	-	-	-	-	-	-	-	-	-
		-	-	-	-	-	-	-	-	-	-	-	-	192711	-	-	-	-	-	-	-	-	-	-	-	-	-	-	-	-	-	-	-	-	-	-	-	-	-	-	-	-	-	-	-	-	-	-	-	-	-	-
FAP261	Flagellar Associated Protein	-	+	-	-	-	-	-	AT1G59453.1	66601	-	-	174103	192696	-	-	-	-	-	-	-	-	-	-	-	-	191501	-	72351	143091	-	Os02g21190.3	-	-	-	67112	-	-	553625	-	-	-	-	-	-	-	-	-	52557	-	-	-
		-	-	-	-	-	-	-	-	-	-	-	-	192692	-	-	-	-	-	-	-	-	-	-	-	-	-	-	-	-	-	-	-	-	-	-	-	-	-	-	-	-	-	-	-	-	-	-	-	-	-	-
		-	-	-	-	-	-	-	-	-	-	-	-	192689	-	-	-	-	-	-	-	-	-	-	-	-	-	-	-	-	-	-	-	-	-	-	-	-	-	-	-	-	-	-	-	-	-	-	-	-	-	-
		-	+	-	-	-	-	-	AT1G63680.1	-	-	-	-	192674	-	-	-	-	-	-	-	-	-	-	-	-	-	-	-	-	-	Os10g40130.1	29411	-	-	63092	-	-	751316	-	-	-	-	-	-	-	-	-	-	-	-	-
		-	-	-	-	-	-	-	-	-	-	-	-	192673	-	-	-	-	-	-	-	-	-	-	-	-	-	-	-	-	-	-	-	-	-	-	-	-	-	-	-	-	-	-	-	-	-	-	-	-	-	-
		-	-	-	-	-	-	-	-	-	-	-	-	192670	-	-	-	-	-	-	-	-	-	-	-	-	-	-	-	-	-	-	-	-	-	-	-	-	-	-	-	-	-	-	-	-	-	-	-	-	-	-
PAFA3401	Paf1 complex component	-	+	-	-	-	-	GB15101-PA	AT1G79730.1	-	BDEG_02960	-	117955	192669	272862	-	-	-	DDB0190242	CG2503-PA	-	-	-	-	OTTHUMP00000068329	-	115833	-	-	99040	-	Os08g06070.1	9574	-	-	43400	134208	-	553771	-	-	-	-	SINFRUP00000162918	-	-	-	-	33941	-	-	-
		-	-	-	-	-	-	-	-	-	-	-	-	192668	-	-	-	-	-	-	-	-	-	-	-	-	-	-	-	-	-	-	-	-	-	-	-	-	-	-	-	-	-	-	-	-	-	-	-	-	-	-
		-	-	-	-	-	-	-	-	-	-	-	-	192657	-	-	-	-	-	-	-	-	-	-	-	-	-	-	-	-	-	-	-	-	-	-	-	-	-	-	-	-	-	-	-	-	-	-	-	-	-	-
		-	-	-	-	-	-	-	-	-	-	-	-	192655	-	-	-	-	-	-	-	-	-	-	-	-	-	-	-	-	-	-	-	-	-	-	-	-	-	-	-	-	-	-	-	-	-	-	-	-	-	-
		-	-	-	-	-	-	-	-	-	-	-	-	192648	-	-	-	-	-	-	-	-	-	-	-	-	-	-	-	-	-	-	-	-	-	-	-	-	-	-	-	-	-	-	-	-	-	-	-	-	-	-
		-	+	+	-	-	-	GB14934-PA	AT3G04600.3	70088	BDEG_06531	CE35140	161193	192647	213719	cgd7_1490	CMG043C	-	DDB0190276	CG9735-PB	19069578	334.m00043	ENSGALP00000018294	GL50803_3032	NP_998810.1	LmjF29.0060	124007	32505	34972	217048	NCU06722	Os12g35570.1	17790	GSPATP00001942001	40901	118004	130369	PF13_0205	721365	RO3G_16975.1	YOL097C	SPAC2F7.13c	-	SINFRUP00000161838	63.m00211	43160	TA20460	80.m00063	50361	90204.m00027	Tb927.3.5580	UM03798.1
		-	+	-	-	-	-	-	-	-	-	-	-	192637	-	-	-	-	DDB0204205	-	-	-	-	-	-	-	-	-	-	-	-	Os02g44704.2	-	-	13700	-	-	-	814391	-	-	-	-	-	-	38317	-	-	-	-	-	-
		-	-	-	-	-	-	-	-	-	-	-	-	192634	-	-	-	-	-	-	-	-	-	-	-	-	-	-	-	-	-	-	-	-	-	-	-	-	-	-	-	-	-	-	-	-	-	-	-	-	-	-
		-	-	-	-	-	-	-	-	-	-	-	-	192633	-	-	-	-	-	-	-	-	-	-	-	-	-	-	-	-	-	-	-	-	-	-	-	-	-	-	-	-	-	-	-	-	-	-	-	-	-	-
		-	-	-	-	-	-	-	AT4G18260.1	-	-	-	-	192627	-	-	-	-	-	-	-	-	-	-	-	-	-	-	-	-	-	Os01g47620.1	-	-	-	59603	-	-	727839	-	-	-	-	-	-	-	-	-	-	-	-	-
HUS1	HUS1 DNA damage checkpoint protein.	-	+	-	-	-	-	-	AT1G52530.1	-	-	-	-	192621	-	-	-	-	DDB0186463	-	-	-	ENSGALP00000020325	-	-	-	185806	-	-	-	-	Os04g44620.2	33063	-	-	106047	-	-	597617	-	-	-	-	SINFRUP00000180264	-	-	-	-	-	-	-	-
		-	+	-	-	-	-	-	-	-	-	CE29081	-	192620	-	-	-	-	-	-	-	-	-	-	-	-	-	-	79010	-	-	-	-	-	45476	-	-	-	-	-	-	-	GLEAN3_01874	-	11.m00330	-	-	-	-	-	-	-
		-	-	-	-	-	-	-	-	-	-	-	-	192616	-	-	-	-	-	-	-	-	-	-	-	-	-	-	-	-	-	-	-	-	-	-	-	-	-	-	-	-	-	-	-	-	-	-	-	-	-	-
SMC6b	SMC6-like protein	-	-	-	-	-	-	-	-	-	-	-	-	192612	-	-	-	-	-	-	-	-	-	-	-	-	-	-	-	-	-	-	-	-	-	-	-	-	-	-	-	-	-	-	-	-	-	583.m00618	-	-	-	-
		-	-	-	-	-	-	-	-	-	-	-	-	192611	-	-	-	-	-	-	-	-	-	-	-	-	-	-	-	-	-	-	-	-	-	-	-	-	-	-	-	-	-	-	-	-	-	-	-	-	-	-
		-	-	-	-	-	-	-	-	-	-	-	-	192603	-	-	-	-	-	-	-	-	-	-	-	-	-	-	-	-	-	-	-	-	-	-	-	-	-	-	-	-	-	-	-	-	-	-	-	-	-	-
		-	-	-	-	-	-	-	-	-	-	-	-	192602	-	-	-	-	-	-	-	-	-	-	-	-	-	-	-	-	-	-	-	-	-	-	-	-	-	-	-	-	-	-	-	-	-	-	-	-	-	-
		-	-	-	-	-	-	-	-	-	-	-	-	192601	-	-	-	-	-	-	-	-	-	-	-	-	-	-	-	-	-	-	-	-	-	-	-	-	-	-	-	-	-	-	-	-	-	-	-	-	-	-
RBM8A	putative RNA binding motif protein 8 (RBM8A)	-	+	-	-	-	-	GB17273-PA	AT1G51510.1	-	BDEG_07076	CE01044	222447	192599	218437	-	CMT598C	-	DDB0218780	CG8781-PA	-	274.m00081	-	-	OTTHUMP00000015574	-	126420	27383	51342	91495	NCU03226	Os05g04850.1	17857	GSPATP00017571001	20716	119944	132792	PF14_0057	203151	RO3G_00643.1	-	SPAC23A1.09	GLEAN3_19673	SINFRUP00000138758	8.m00351	18841	TA18815	44.m00053	63736	-	-	-
GND1a	6-phosphogluconate dehydrogenase, decarboxylating, possible plastid form	-	-	-	-	-	-	-	-	-	-	-	-	192597	-	-	CML059C	-	-	-	-	-	-	-	-	-	-	-	-	-	NCU03100	-	-	-	-	189273	-	-	-	-	-	-	GLEAN3_24379	-	-	-	-	-	-	-	-	-
		-	-	-	-	-	-	-	-	-	-	-	-	192594	-	-	-	-	-	-	-	-	-	-	-	-	-	-	-	-	-	-	-	-	-	-	-	-	-	-	-	-	-	-	-	-	-	-	-	-	-	-
		-	-	-	-	-	-	-	-	-	-	-	-	192591	-	-	-	-	-	-	-	-	-	-	-	-	-	-	-	-	-	-	-	-	-	-	-	-	-	-	-	-	-	-	-	-	-	-	-	-	-	-
		-	-	-	-	-	-	-	-	-	-	-	-	192587	-	-	-	-	-	-	-	-	-	-	-	-	-	-	-	-	-	-	-	-	-	-	-	-	-	-	-	-	-	-	-	-	-	-	-	-	-	-
		-	-	-	-	-	-	-	AT1G73740.1	-	-	-	-	192581	-	-	-	-	-	-	-	-	-	-	-	-	-	-	-	-	-	Os01g33230.1	-	-	-	204518	-	-	423407	-	-	-	-	-	-	-	-	-	-	-	-	-
		-	-	-	-	-	-	-	-	-	-	-	-	192575	-	-	-	-	-	-	-	-	-	-	-	-	-	-	-	-	-	-	-	-	-	-	-	-	-	-	-	-	-	-	-	-	-	-	-	-	-	-
		-	-	-	-	-	-	-	-	-	-	-	-	192574	-	-	-	-	-	-	-	-	-	-	-	-	-	-	-	-	-	-	-	-	-	-	-	-	-	-	-	-	-	-	-	6743	-	-	-	-	-	-
		-	-	-	-	-	-	-	-	-	-	-	-	192569	-	-	-	-	-	-	-	-	-	-	-	-	-	-	-	-	-	-	-	-	-	-	-	-	-	-	-	-	-	-	-	-	-	-	-	-	-	-
		-	-	-	-	-	-	-	-	-	-	-	-	192566	-	-	-	-	-	-	-	-	-	-	-	-	-	-	-	-	-	-	-	-	-	-	-	-	-	-	-	-	-	-	-	-	-	-	-	-	-	-
DLC3	Flagellar outer dynein arm light chain 3	-	-	-	+	-	-	-	-	-	-	-	-	192564	-	-	-	-	-	CG18130-PA	-	-	-	-	OTTHUMP00000172994	-	-	-	-	-	-	-	-	-	-	-	-	-	-	-	-	-	-	-	-	-	-	-	22954	-	-	-
		-	+	+	-	-	-	-	AT4G15770.1	38980	BDEG_00693	CE08687	221286	192563	221122	cgd4_660	CMN108C	-	DDB0216882	-	19069318	63.m00174	ENSGALP00000000921	GL50803_6177	OTTHUMP00000080298	LmjF33.1350	197143	34642	76399	244907	NCU00105	Os07g47420.1	17302	GSPATP00008039001	13281	214679	109108	PF14_0635	768816	RO3G_09534.1	YPL211W	SPCC320.11c	-	SINFRUP00000143271	81.m00195	39003	TA08380	59.m03566	50432	82490.m00265	Tb10.389.1790	UM05683.1
PUF1	pumilio family (Puf) protein	-	+	+	-	-	-	GB15625-PA	AT3G16810.1	60553	BDEG_04091	CE01734	163846	192562	-	-	CML268C	-	DDB0187989	CG1685-PA	-	38.m00237	ENSGALP00000016527	GL50803_16602	NP_055693.4	LmjF25.2360	111730	506	78927	184407	NCU09380	Os03g61560.1	35364	-	47058	147581	134256	-	295997	RO3G_09462.1	YDR496C	SPCP1E11.11	-	SINFRUP00000155594	57.m00228	23354	TA14560	583.m05622	-	95415.m00086	Tb927.3.2470	-
		-	-	-	-	-	-	-	-	-	-	-	-	192560	-	-	-	-	-	-	-	-	-	-	-	-	-	-	-	-	-	-	33394	-	-	163643	-	-	-	-	-	-	-	-	-	-	-	-	-	-	-	-
DRP5A	Dynamin-related GTPase	-	+	-	-	-	-	-	AT1G53140.1	-	-	-	-	192551	-	-	-	-	DDB0216875	-	-	86.m00170	-	-	-	-	-	-	29250	-	-	Os01g54420.1	-	-	-	112955	-	-	568892	-	-	-	-	-	-	-	-	-	-	-	-	-
ALK	Aurora Like Kinase	-	-	-	-	-	-	-	-	-	-	-	-	192550	-	-	-	-	-	-	-	-	-	-	-	-	-	-	-	-	-	-	4957	-	-	-	-	-	-	-	-	-	-	-	-	-	-	-	-	-	-	-
		-	+	-	-	-	-	-	AT1G09575.1	-	-	CE27192	226862	192544	262469	-	-	-	DDB0186972	CG18769-PF	-	-	ENSGALP00000019885	-	NP_060388.1	-	203141	-	48567	227549	NCU08166	Os01g60110.1	24164	-	-	46529	128095	-	240126	-	-	-	-	SINFRUP00000163611	57.m00278	-	-	-	-	-	-	-
		-	-	-	-	-	-	-	-	-	-	-	-	192535	-	-	-	-	-	-	-	-	-	-	-	-	-	-	-	-	-	-	-	-	-	-	-	-	-	-	-	-	-	-	-	-	-	-	-	-	-	-
		-	-	-	-	-	-	-	-	-	-	-	-	192533	-	-	-	-	-	-	-	-	-	-	-	-	-	-	-	-	-	-	-	-	-	-	-	-	-	-	-	-	-	-	-	-	-	-	-	-	-	-
		-	-	-	-	-	-	-	-	-	-	-	-	192532	-	-	-	-	-	-	-	-	-	-	-	-	-	-	-	-	-	-	-	-	-	-	-	-	-	-	-	-	-	-	-	-	-	-	-	-	-	-
RPN2	26S proteasome regulatory subunit RPN2	-	+	+	-	-	-	GB19286-PA	AT1G04810.1	72853	BDEG_02138	CE31311	182474	192526	282556	cgd4_3950	CMQ283C	OTTDARP00000010845	DDB0187705	-	19068611	4.m00637	ENSGALP00000012405	GL50803_91643	OTTHUMP00000164295	LmjF28.1730	206376	32955	78721	193603	NCU09450	Os08g12820.1	20634	GSPATP00038321001	19300	105656	108910	PF14_0632	829819	RO3G_03530.1	YIL075C	SPBC17D11.07c	-	SINFRUP00000179862	50.m00190	24295	TA08355	55.m05030	64198	92880.m00133	Tb11.01.0960	UM04786.1
		-	-	-	+	-	-	-	-	-	-	-	-	192522	-	-	-	-	-	-	-	-	-	-	-	-	-	-	-	-	-	-	-	-	-	-	-	-	-	-	-	-	-	-	-	-	-	-	-	-	-	-
		-	-	-	-	-	-	-	-	-	-	-	-	192518	-	-	-	-	-	-	-	-	-	-	-	-	-	-	-	-	-	-	-	-	-	-	-	-	-	-	-	-	-	-	-	-	-	-	-	-	-	-
SHKA1	3-deoxy-D-arabino-heptulosonate 7-phosphate synthetase	-	+	-	-	-	-	-	AT4G39980.1	36370	BDEG_04759	-	144358	192517	-	-	CMQ120C	-	-	-	-	-	-	-	-	-	-	30543	-	-	NCU02785	Os08g37790.1	11450	GSPATP00002042001	24353	121478	109327	-	830232	RO3G_04249.1	-	-	-	-	64.m00224	2790	-	41.m00002	-	-	-	UM02010.1
		-	+	-	-	-	-	-	AT1G02870.1	65059	-	-	-	192516	-	-	-	-	-	-	-	-	ENSGALP00000005297	GL50803_8002	-	LmjF10.0860	214489	33025	45108	231966	-	Os05g30420.1	32502	-	-	172934	139244	-	824185	RO3G_02154.1	-	-	-	-	-	-	-	-	-	-	-	-
		-	-	-	-	-	-	-	-	-	-	-	-	192514	-	-	-	-	-	-	-	-	-	-	-	-	-	-	-	-	-	-	-	-	-	-	-	-	-	-	-	-	-	-	-	-	-	-	-	-	-	-
ATP12	Putative assembly factor 2 for F1 component of mitochondrial ATP synthase	-	+	-	-	-	-	GB19431-PA	AT5G40660.1	-	BDEG_05481	CE37421	114697	192510	287848	-	CMO239C	-	DDB0184519	CG8674-PA	-	-	ENSGALP00000007866	-	OTTHUMP00000183280	LmjF18.0350	110395	32882	-	114659	NCU00676	Os07g44940.1	21592	GSPATP00024635001	50559	226078	-	-	174190	RO3G_02047.1	YJL180C	SPAC9.12c	GLEAN3_06599	SINFRUP00000141758	181.m00067	22249	-	-	21486	-	-	UM01563.1
		-	-	-	-	-	-	-	-	-	-	-	-	192509	-	-	-	-	-	-	-	-	-	-	-	-	-	-	-	-	-	-	-	-	-	-	-	-	-	-	-	-	-	-	-	-	-	-	-	-	-	-
		-	-	-	-	-	-	-	-	-	-	-	-	192491	-	-	-	-	-	-	-	-	-	-	-	-	-	-	-	-	-	-	-	-	-	-	-	-	-	-	-	-	-	-	-	-	-	-	-	-	-	-
RBB3401	WD-40 repeat protein	-	+	+	-	-	-	GB15806-PA	AT2G19540.1	72521	BDEG_01729	CE27292	155831	192490	285367	cgd4_3360	CMB073C	-	DDB0183986	CG12792-PA	-	31.m00211	-	GL50803_14174	NP_113673.2	LmjF33.1910	111616	29376	33749	124120	NCU09521	Os11g03990.1	18061	GSPATP00034573001	47947	123753	156374	PF08_0065	217471	RO3G_03675.1	YMR131C	SPBC1711.07	-	SINFRUP00000129211	55.m00247	39022	TA14475	583.m00658	63433	95305.m00232	Tb11.02.0570	UM00802.1
	hypothetical protein	-	-	-	-	-	-	-	-	-	-	-	-	192489	-	-	-	-	-	-	-	-	-	-	-	-	-	-	-	-	-	-	-	-	-	-	-	-	-	-	-	-	-	-	-	-	-	-	-	-	-	-
	hypothetical protein	-	-	-	-	-	-	-	-	-	-	-	-	192488	-	-	-	-	-	-	-	-	-	-	-	-	-	-	-	-	-	-	-	-	-	-	-	-	-	-	-	-	-	-	-	-	-	-	-	-	-	-
PSAK	photosystem I reaction center subunit psaK, chloroplast precursor	-	+	-	-	-	-	-	AT1G30380.1	-	-	-	-	192478	-	-	-	-	-	-	-	-	-	-	-	-	-	-	-	-	-	Os07g05480.1	22023	-	-	62760	-	-	415807	-	-	-	-	-	-	-	-	-	-	-	-	-
LAH	Homologue of Lupus La protein	-	+	-	-	-	-	GB19284-PA	AT4G32720.2	-	-	CE08718	-	192476	-	cgd1_2310	CMR137C	-	DDB0204655	CG12058-PA	19171316	41.m00229	ENSGALP00000037720	GL50803_9803	OTTHUMP00000163067	LmjF21.0540	132027	34421	59316	236578	NCU08295	Os02g39700.1	30488	-	-	219047	142142	-	717953	RO3G_11303.1	YDL051W	SPAC57A10.10c	-	SINFRUP00000176118	-	-	-	-	-	93986.m00248	Tb10.70.5360	UM03724.1
		-	-	-	-	-	-	-	-	-	-	-	-	192470	-	-	-	-	-	-	-	-	-	-	-	-	-	-	-	-	-	-	-	-	-	-	-	-	-	-	-	-	-	-	-	-	-	-	-	-	-	-
		-	-	-	-	-	-	-	-	-	-	-	-	192467	-	-	-	-	-	-	-	-	-	-	-	-	-	-	-	-	-	-	-	-	-	-	-	-	-	-	-	-	-	-	-	-	-	-	-	-	-	-
		-	+	-	-	-	-	-	-	-	-	-	-	192466	-	-	-	-	-	CG33146-PA	-	3.m00563	-	-	-	-	-	-	-	-	-	-	-	-	-	-	-	-	-	RO3G_05663.1	-	-	-	SINFRUP00000177476	-	-	-	-	-	92321.m00068	-	-
		-	-	-	-	-	-	-	-	-	-	-	-	192462	-	-	-	-	-	-	-	-	-	-	-	-	-	-	-	-	-	-	-	-	-	-	-	-	-	-	-	-	-	-	-	-	-	-	-	-	-	-
		-	-	-	-	-	-	-	-	-	-	-	-	192452	-	-	-	-	-	-	-	-	-	-	-	-	-	-	-	-	-	-	-	-	-	-	-	-	-	-	-	-	-	-	-	-	-	-	-	-	-	-
		-	-	-	-	-	-	-	-	-	-	-	-	192448	-	-	-	-	-	-	-	-	-	-	-	-	-	-	-	-	-	-	-	-	-	-	-	-	-	-	-	-	-	-	-	-	-	-	-	-	-	-
		-	+	-	-	-	-	GB10083-PA	AT5G27490.1	26354	BDEG_04116	CE21475	178563	192447	264809	-	-	OTTDARP00000004452	DDB0204543	CG4645-PA	-	418.m00031	ENSGALP00000017456	-	NP_061855.1	LmjF30.0140	227472	-	29707	246530	NCU05276	Os01g42480.1	36681	GSPATP00022234001	44631	46689	134792	-	662766	RO3G_08534.1	-	SPAC227.06	GLEAN3_09734	SINFRUP00000150065	-	5790	-	-	57835	-	Tb927.6.1540	-
		-	-	-	-	-	-	-	-	-	-	-	-	192443	-	-	-	-	-	-	-	-	-	-	-	-	-	-	-	-	-	-	-	-	-	-	-	-	-	-	-	-	-	-	-	-	-	-	-	-	-	-
		+	+	-	-	-	-	-	-	-	-	-	207302	192442	212861	-	-	-	-	-	-	-	ENSGALP00000025719	-	OTTHUMP00000178110	-	117480	36264	56610	236343	-	-	-	GSPATP00023333001	-	-	-	-	-	-	-	-	-	-	114.m00114	-	-	-	52472	90316.m00126	Tb927.4.1690	-
RABL2A	RABL2a, small Rab-related GTPase	+	+	-	-	-	-	-	-	18664	BDEG_08668	-	179181	192441	297126	-	-	OTTDARP00000012587	-	-	-	-	ENSGALP00000015873	-	OTTHUMP00000045492	LmjF32.1840	171726	30574	60926	163982	-	-	-	GSPATP00015650001	-	-	111482	-	-	-	-	-	-	SINFRUP00000133276	226.m00042	8779	-	-	23051	81529.m00447	Tb11.01.6670	-
		-	+	-	-	-	-	-	-	21779	-	CE32416	175610	192430	390493	-	-	-	DDB0184264	-	-	-	ENSGALP00000011298	-	NP_037434.1	LmjF25.0680	232789	-	-	118107	-	-	-	-	34488	-	-	-	-	-	-	-	GLEAN3_11641	-	-	261433	-	-	33592	-	-	-
		-	-	-	-	-	-	-	-	-	-	-	-	192429	-	-	-	-	-	-	-	-	-	-	-	-	-	-	-	-	-	-	-	-	-	-	-	-	-	-	-	-	-	-	-	-	-	-	-	-	-	-
		-	-	-	-	-	-	-	-	-	-	-	-	192426	-	-	-	-	-	-	-	-	-	-	-	-	-	-	-	-	-	-	-	-	-	-	-	-	-	-	-	-	-	-	-	-	-	-	-	-	-	-
		-	-	-	-	-	-	-	-	-	-	-	-	192422	203731	-	CMT103C	-	-	-	-	-	-	-	-	-	-	-	-	-	-	-	-	-	-	-	-	-	-	-	-	-	-	-	-	-	-	-	-	-	-	-
FAP22	Flagellar Associated Protein Similar to Qilin (D. rerio)	+	+	-	+	-	-	GB11182-PA	-	2738	BDEG_02841	CE38264	165571	192420	272750	-	-	-	-	CG17599-PA	-	-	ENSGALP00000021808	GL50803_16707	OTTHUMP00000107638	LmjF25.1430	230213	27399	62998	244655	-	-	-	GSPATP00017664001	-	80743	140860	-	-	-	-	-	GLEAN3_11145	SINFRUP00000132534	410.m00008	5089	-	83.m01243	20699	82360.m00259	Tb927.3.1110	-
		-	-	-	-	-	-	-	-	-	-	-	-	192418	-	-	-	-	-	-	-	-	-	-	-	-	-	-	-	-	-	-	-	-	-	-	-	-	-	-	-	-	-	-	-	-	-	-	-	-	-	-
		-	+	-	-	-	-	-	AT1G72590.1	-	-	CE05157	197513	192416	286955	-	-	-	-	CG7840-PA	-	-	-	-	-	-	-	-	-	101187	-	Os04g48750.1	-	-	-	119780	-	-	759753	-	-	-	GLEAN3_13380	SINFRUP00000133466	-	-	-	-	53471	95599.m00088	-	-
		-	-	-	-	-	-	-	-	-	-	-	-	192415	-	-	-	-	-	-	-	-	-	-	-	-	-	-	-	-	-	-	-	-	-	-	-	-	-	-	-	-	-	-	-	-	-	-	-	-	-	-
		-	-	-	-	-	-	-	-	-	-	-	-	192406	-	-	-	-	-	-	-	-	-	-	-	-	-	-	-	-	-	-	-	-	44203	-	-	-	-	-	-	-	-	-	-	-	-	-	-	-	-	-
		-	-	-	-	-	-	-	-	-	-	-	-	192403	-	-	-	-	-	-	-	-	-	-	-	-	-	-	-	-	-	-	-	-	-	-	-	-	-	-	-	-	-	-	-	-	-	-	-	-	-	-
		-	-	-	-	-	-	-	-	-	-	-	-	192402	-	-	-	-	-	-	-	-	-	-	-	-	-	-	-	-	-	-	-	-	-	-	-	-	-	-	-	-	-	-	-	-	-	-	-	-	-	-
		-	+	-	-	-	-	-	AT5G26570.1	-	-	-	-	192398	-	-	-	-	-	-	-	-	-	-	-	-	157569	-	-	-	-	Os12g20150.1	18828	GSPATP00016527001	-	104427	-	-	664666	-	-	-	-	-	-	-	-	80.m02299	-	-	-	-
		-	-	-	-	-	-	-	-	-	-	-	-	192396	-	-	-	-	-	-	-	-	-	-	-	-	-	-	-	-	-	-	-	-	-	-	-	-	-	-	-	-	-	-	-	-	-	-	-	-	-	-
		-	-	-	-	-	-	-	-	-	-	-	-	192392	-	-	-	-	-	-	-	-	-	-	-	-	-	-	-	-	-	-	-	-	-	-	-	-	-	-	-	-	-	-	-	-	-	-	-	-	-	-
		-	-	-	-	-	-	-	-	-	-	-	-	192386	-	-	-	-	-	-	-	-	-	-	-	-	-	-	-	-	-	-	-	-	-	-	-	-	-	-	-	-	-	-	-	-	-	-	-	-	-	-
		-	-	-	-	-	-	-	-	-	-	-	-	192385	-	-	-	-	-	-	-	-	-	-	-	-	-	-	-	-	-	-	-	-	-	-	-	-	-	-	-	-	-	-	-	-	-	-	-	-	-	-
		-	-	-	-	-	-	-	-	-	-	-	-	192383	-	-	-	-	-	-	-	-	-	-	-	-	-	-	-	-	-	-	-	-	-	-	-	-	-	-	-	-	-	-	-	-	-	-	-	-	-	-
		-	-	-	-	-	-	-	-	-	-	-	-	192380	-	-	-	-	-	-	-	-	-	-	-	-	-	-	-	-	-	-	-	-	-	-	-	-	-	-	-	-	-	-	-	-	-	-	-	-	-	-
		-	-	-	-	-	-	-	-	-	-	-	-	192379	-	-	-	-	-	-	-	-	-	-	-	-	-	-	-	-	-	-	-	-	-	-	-	-	-	-	-	-	-	-	-	-	-	-	-	-	-	-
		-	-	-	-	-	-	-	-	-	-	-	-	192378	-	-	-	-	-	-	-	-	-	-	-	-	-	-	-	-	-	-	-	-	-	-	-	-	-	-	-	-	-	-	-	-	-	-	-	-	-	-
	expressed protein of unknown function	-	-	-	-	-	-	-	-	-	-	-	-	192375	-	-	-	-	-	-	-	-	-	-	-	-	-	-	-	-	-	-	-	-	-	-	-	-	-	-	-	-	-	-	-	-	-	-	-	-	-	-
		-	-	-	-	-	-	-	-	-	-	-	-	192371	-	-	-	-	-	-	-	-	-	-	-	-	-	-	-	-	-	-	-	-	-	-	-	-	-	-	-	-	-	-	-	262949	-	-	-	-	Tb11.01.5250	-
PCR1	pyrroline-5-carboxylate reductase	-	+	-	-	-	-	GB18352-PA	AT5G14800.1	59057	BDEG_04962	CE03511	166551	192364	377463	cgd6_3720	CMI158C	OTTDARP00000006622	DDB0190241	-	-	-	ENSGALP00000025936	-	NP_075566.1	LmjF13.1680	169622	27275	76810	235823	NCU06471	Os01g71990.1	13226	-	25379	138353	109147	MAL13P1.284	831811	RO3G_16325.1	YER023W	SPAPYUG7.05	GLEAN3_26583	SINFRUP00000131351	7.m00562	23275	-	46.m00029	27568	82366.m00095	Tb927.7.2440	UM00547.1
		-	+	-	-	-	-	GB11615-PA	-	-	BDEG_04833	CE15928	99966	192362	-	-	-	-	-	CG11474-PB	-	-	ENSGALP00000021133	-	OTTHUMP00000017433	-	178238	30731	-	163606	-	-	3233	-	-	-	-	-	-	RO3G_09753.1	-	SPCC1393.13	-	SINFRUP00000143426	-	-	-	-	51013	-	-	UM05521.1
		-	-	-	-	-	-	-	-	-	-	-	-	192361	-	-	-	-	-	-	-	-	-	-	-	-	-	-	-	-	-	-	-	-	-	-	-	-	-	-	-	-	-	-	-	-	-	-	-	-	-	-
		-	-	-	-	-	-	-	-	-	-	-	-	192356	-	-	-	-	-	-	-	-	-	-	-	-	-	-	-	-	-	-	-	-	-	-	-	-	-	-	-	-	-	-	-	-	-	-	-	-	-	-
		-	-	-	-	-	-	-	-	-	-	-	-	192354	-	-	-	-	-	-	-	-	-	-	-	-	-	-	-	-	-	-	-	-	-	-	-	-	-	-	-	-	-	-	-	-	-	-	-	-	-	-
CLC	Clathrin Light Chain	-	+	-	-	-	-	-	-	-	-	-	19313	192349	206119	-	-	-	-	-	-	-	ENSGALP00000005281	-	OTTHUMP00000021366	-	-	-	-	175828	-	-	-	-	-	-	-	-	-	RO3G_01814.1	-	-	-	SINFRUP00000169881	-	-	-	-	63622	-	-	-
IF1A	translation initiation factor 1A	-	+	-	-	-	-	GB15960-PA	AT2G40780.1	-	BDEG_04351	CE06668	172896	192348	291802	-	-	OTTDARP00000024020	DDB0184249	CG31957-PA	-	-	-	-	NP_115701.2	-	134275	38794	-	108065	NCU06553	Os03g02310.1	-	-	47235	220383	134116	-	581089	-	-	SPBC146.08c	-	SINFRUP00000147065	-	269049	-	-	59800	-	-	-
		-	-	-	-	-	-	-	-	-	-	-	-	192347	-	-	-	-	-	-	-	-	-	-	-	-	-	-	-	-	-	-	-	-	-	-	-	-	-	-	-	-	-	-	-	-	-	-	-	-	-	-
		-	-	-	-	-	-	-	-	-	-	-	-	192345	-	-	-	-	-	-	-	-	-	-	-	-	-	-	-	-	-	-	-	-	-	-	-	-	-	-	-	-	-	-	-	-	-	-	-	-	-	-
		-	-	-	-	-	-	-	-	-	-	-	-	192339	-	-	-	-	DDB0190726	-	-	-	-	-	-	-	-	-	-	-	-	-	-	-	-	-	-	-	-	-	-	-	-	-	-	-	-	-	-	-	-	-
		-	-	-	-	-	-	-	-	-	-	-	-	192338	-	-	-	-	-	-	-	-	-	-	-	-	-	-	-	-	-	-	33857	-	-	-	-	-	-	-	-	-	-	-	-	-	-	-	-	-	-	-
		-	-	-	-	-	-	-	-	-	-	-	-	192337	-	-	-	-	-	-	-	-	-	-	-	-	-	-	-	-	-	-	-	-	-	-	-	-	-	-	-	-	-	-	-	-	-	-	-	-	-	-
		-	-	-	-	-	-	-	-	-	-	-	-	192331	-	-	-	-	-	-	-	-	-	-	-	-	-	-	-	-	-	-	-	-	-	-	-	-	-	-	-	-	-	-	-	-	-	-	-	-	-	-
		-	-	-	-	-	-	-	-	-	-	-	-	192329	-	-	-	-	-	-	-	-	-	-	-	-	-	-	-	-	-	-	-	-	-	-	-	-	-	-	-	-	-	-	-	-	-	-	-	-	-	-
CHR3403		-	-	-	-	-	-	-	-	-	-	-	-	192323	-	-	-	-	-	-	-	-	-	-	-	-	-	-	-	-	-	Os05g25840.3	-	-	-	168107	-	-	-	-	-	-	-	-	-	-	-	-	-	-	-	-
		-	-	-	-	-	-	-	-	-	-	-	-	192321	-	-	-	-	-	-	-	-	-	-	-	-	-	-	-	-	-	-	-	-	-	-	-	-	-	-	-	-	-	-	-	-	-	-	-	-	-	-
		-	-	-	-	-	-	-	-	-	-	-	-	192312	-	-	-	-	-	-	-	-	-	-	-	-	-	-	-	-	-	-	-	-	-	-	-	-	-	-	-	-	-	-	-	-	-	-	-	-	-	-
		-	-	-	-	-	-	-	-	-	-	-	-	192311	-	-	-	-	-	-	-	-	-	-	-	-	-	-	-	-	-	-	-	-	-	-	-	-	-	-	-	-	-	-	-	-	-	-	-	-	-	-
AMT1	Ammonia channel	-	+	-	-	-	-	GB10552-PA	AT3G24290.1	10990	-	-	62344	192308	-	-	CMT526C	-	-	-	-	-	-	-	-	-	-	14204	-	173932	NCU03257	-	-	-	-	-	-	-	-	RO3G_12622.1	-	SPCPB1C11.01	-	-	-	-	-	-	-	-	-	-
		-	+	-	-	-	-	-	AT5G62960.1	-	-	-	-	192300	-	-	-	-	-	CG6149-PA	-	-	-	-	-	-	-	30817	-	-	-	Os07g46430.2	-	-	-	163353	136331	-	552585	-	-	-	-	-	-	-	-	-	-	-	-	-
FAP184	Conserved Uncharacterized Flagellar Associated Protein	+	+	-	+	-	-	-	-	-	BDEG_07420	-	228185	192295	251609	-	-	-	-	-	-	-	-	GL50803_22806	OTTHUMP00000155166	LmjF14.0210	168287	36697	2066	108600	-	-	-	GSPATP00003011001	-	67217	158512	-	-	-	-	-	-	SINFRUP00000176872	67.m00149	-	-	25.m01841	-	82174.m00119	Tb927.7.4510	-
		-	+	+	-	-	-	GB10045-PA	AT5G20520.1	13525	BDEG_07095	-	92523	192294	264185	cgd3_730	CMT218C	-	DDB0188509	CG18642-PA	19171321	72.m00175	ENSGALP00000027178	-	OTTHUMP00000018692	LmjF35.4020	129983	-	59950	227226	NCU03276	Os07g41730.1	-	GSPATP00010197001	10362	214723	140406	-	260017	RO3G_04470.1	YNL320W	SPBC32H8.03	-	SINFRUP00000159682	5.m00418	16777	-	52.m01670	62339	-	Tb09.211.2310	UM04699.1
		-	+	-	-	-	-	GB19388-PA	AT1G27330.1	-	-	CE31064	175858	192290	206180	cgd2_140	-	-	DDB0202595	CG32276-PB	-	-	-	-	OTTHUMP00000018334	-	193286	-	-	169659	-	Os12g32950.1	31481	-	-	205447	-	-	717321	-	-	-	-	SINFRUP00000143189	52.m00234	-	-	65.m02518	-	-	-	-
		-	-	-	-	-	-	-	-	-	-	-	-	192280	-	-	-	-	-	-	-	-	-	-	-	-	-	-	-	-	-	-	-	-	-	-	-	-	-	-	-	-	-	-	-	-	-	-	-	-	-	-
		-	-	-	-	-	-	-	-	-	-	-	-	192279	-	-	-	-	-	-	-	-	-	-	-	-	-	-	-	-	-	-	-	-	-	-	-	-	-	-	-	-	-	-	-	-	-	-	-	-	-	-
MFT2	putative MATE efflux family protein	-	-	-	-	-	-	-	-	-	-	-	-	192277	-	-	-	-	-	-	-	-	-	-	-	-	-	-	-	-	-	Os09g37610.1	-	-	-	-	-	-	-	-	-	-	-	-	-	-	-	-	-	-	-	-
		-	-	-	-	-	-	-	-	-	-	-	-	192267	-	-	-	-	-	-	-	-	-	-	-	-	-	-	-	-	-	-	-	-	-	-	-	-	-	-	-	-	-	-	-	-	-	-	-	-	-	-
		-	+	-	-	-	-	-	-	-	-	-	160904	192265	-	-	-	-	-	-	-	-	ENSGALP00000039480	-	OTTHUMP00000019816	LmjF31.1650	-	-	-	-	-	-	-	-	-	-	133958	-	-	-	-	-	-	SINFRUP00000163145	-	-	-	-	-	-	Tb927.4.4670	-
		-	-	-	-	-	-	-	-	-	-	-	-	192258	-	-	-	-	-	-	-	-	-	-	-	-	-	-	-	-	-	-	-	-	-	-	-	-	-	-	-	-	-	-	-	-	-	-	-	-	-	-
		-	-	-	-	-	-	-	-	-	-	-	-	192256	-	-	-	-	-	-	-	-	-	-	-	-	-	-	-	-	-	-	-	-	-	-	-	-	-	-	-	-	-	-	-	-	-	-	-	-	-	-
		-	-	-	-	-	-	-	-	-	-	-	-	192241	-	-	-	-	-	-	-	-	-	-	-	-	-	-	-	-	-	-	-	-	-	-	-	-	-	-	-	-	-	-	-	-	-	-	-	-	-	-
		-	-	-	-	-	-	-	-	-	-	-	-	192240	-	-	-	-	-	-	-	-	-	-	-	-	-	-	-	-	-	-	-	-	-	-	-	-	-	-	-	-	-	-	-	-	-	-	-	-	-	-
		-	-	-	-	-	-	-	-	-	-	-	-	192239	-	-	-	-	-	-	-	-	-	-	-	-	-	-	-	-	-	-	-	-	-	-	-	-	-	-	-	-	-	-	-	-	-	-	-	-	-	-
		-	-	-	-	-	-	-	-	-	-	-	-	192238	-	-	-	-	-	-	-	-	-	-	-	-	-	-	-	-	-	-	-	-	-	-	-	-	-	-	-	-	-	-	-	-	-	-	-	-	-	-
		-	-	-	-	-	-	-	-	-	-	-	-	192237	-	-	-	-	-	-	-	-	-	-	-	-	-	-	-	-	-	-	-	-	-	-	-	-	-	-	-	-	-	-	-	-	-	-	-	-	-	-
SIR2	ferredoxin-sulfite reductase, putative	-	-	-	-	-	-	-	-	-	-	-	-	192232	-	-	-	-	-	-	-	-	-	-	-	-	-	-	-	-	-	-	-	-	-	-	-	-	-	-	-	-	-	-	-	-	-	-	-	-	-	-
		-	-	-	-	-	-	-	-	-	-	-	-	192231	-	-	-	-	-	-	-	-	-	-	-	-	-	-	-	-	-	-	-	-	-	-	-	-	-	-	-	-	-	-	-	-	-	-	-	-	-	-
		-	-	-	-	-	-	-	-	-	-	-	-	192230	-	-	-	-	-	-	-	-	-	-	-	-	-	-	-	-	-	-	-	-	-	-	-	-	-	-	-	-	-	-	-	-	-	-	-	-	-	-
		-	-	-	-	-	-	-	-	-	-	-	-	192228	-	-	-	-	-	-	-	-	-	-	-	-	-	-	-	-	-	-	-	-	-	-	-	-	-	-	-	-	-	-	-	-	-	-	-	-	-	-
		-	-	-	-	-	-	-	-	-	-	-	-	192227	-	-	-	-	-	-	-	-	-	-	-	-	-	-	-	-	-	-	-	-	-	-	-	-	-	-	-	-	-	-	-	-	-	-	-	-	-	-

		-	-	-	-	-	-	-	-	-	-	-	-	192226	-	-	-	-	-	-	-	-	-	-	-	-	-	-	-	-	-	-	-	-	-	-	-	-	-	-	-	-	-	-	-	-	-	-	-	-	-	-
		-	-	-	-	-	-	-	-	-	-	-	-	192222	-	-	-	-	-	-	-	-	-	-	-	-	-	-	-	-	-	-	-	-	-	-	-	-	-	-	-	-	-	-	-	-	-	-	-	-	-	-
FAP29	Flagellar Associated Protein	-	+	-	-	-	-	-	AT1G19110.1	-	-	-	-	192221	250768	-	-	-	-	-	-	-	-	-	-	-	-	-	-	243833	-	Os07g42280.1	-	-	-	136279	-	-	785943	RO3G_12896.1	-	-	-	-	-	-	-	-	-	-	-	-
		-	-	-	-	-	-	-	-	-	-	-	-	192216	-	-	-	-	-	-	-	-	-	-	-	-	-	-	-	-	-	-	-	-	-	-	-	-	-	-	-	-	-	-	-	-	-	-	-	-	-	-
		-	+	-	-	-	-	GB14785-PA	AT3G50690.1	-	-	-	171884	192214	374725	cgd1_930	CMO169C	OTTDARP00000020705	DDB0204007	CG5784-PB	-	-	ENSGALP00000002398	GL50803_16951	OTTHUMP00000021759	-	138173	-	-	104042	-	Os07g41694.1	-	GSPATP00008369001	-	153718	-	-	593953	-	-	-	-	SINFRUP00000145578	33.m00277	-	-	59.m03592	-	-	-	-
		-	-	-	-	-	-	-	-	-	-	-	-	192211	-	-	-	-	-	-	-	-	-	-	-	-	-	-	-	-	-	-	-	-	-	-	-	-	-	-	-	-	-	-	-	-	-	-	-	-	-	-
		-	-	-	-	-	-	-	-	-	-	-	-	192210	-	-	-	-	-	-	-	-	-	-	-	-	-	-	-	-	-	-	-	-	-	-	-	-	-	-	-	-	-	-	-	-	-	-	-	-	-	-
		-	-	-	-	-	-	-	-	-	-	-	-	192209	-	-	-	-	-	-	-	-	-	-	-	-	-	-	-	-	-	-	-	-	-	-	-	-	-	-	-	-	-	-	-	-	-	-	-	-	-	-
		-	-	-	-	-	-	-	-	-	-	-	-	192207	-	-	-	-	-	-	-	-	-	-	-	-	-	-	-	-	-	-	-	-	-	-	-	-	-	-	-	-	-	-	-	-	-	-	-	-	-	-
IFT140	Intraflagellar transport particle protein IFT140	+	+	-	+	-	-	GB10317-PA	-	30803	BDEG_03067	CE39480	114177	192205	239096	-	-	OTTDARP00000001990	-	CG11838-PB	-	-	ENSGALP00000015148	GL50803_17251	OTTHUMP00000080879	LmjF32.0310	223604	17802	48798	122551	-	-	-	GSPATP00003644001	-	75973	134255	-	-	-	-	-	GLEAN3_20159	SINFRUP00000163188	18.m00435	-	-	33.m01346	60914	97007.m00130	Tb10.61.2260	-
		-	-	-	-	-	-	-	-	-	-	-	-	192202	-	-	-	-	-	-	-	-	-	-	-	-	-	-	-	-	-	-	-	-	-	-	-	-	-	-	-	-	-	-	-	-	-	-	-	-	-	-
FAP72	Flagellar Associated Protein	-	-	-	+	-	-	-	-	-	-	-	-	192200	-	-	-	-	-	-	-	-	-	-	-	-	-	-	-	-	-	-	-	-	-	-	-	-	-	-	-	-	-	-	-	-	-	-	-	-	-	-
		-	-	-	-	-	-	-	-	-	-	-	-	192195	-	-	-	-	-	-	-	-	-	-	-	-	-	-	-	-	-	-	-	-	-	-	-	-	-	-	-	-	-	-	-	-	-	-	-	-	-	-
		-	-	-	-	-	-	-	-	-	-	-	-	192194	295703	-	-	-	-	-	-	-	-	-	-	-	-	-	-	-	-	-	-	-	-	-	-	-	-	-	-	-	-	-	-	-	-	-	-	-	-	-
		-	-	-	-	-	-	-	-	-	-	-	-	192193	-	-	-	-	-	-	-	-	-	-	-	-	-	-	-	-	-	-	-	-	-	-	-	-	-	-	-	-	-	-	-	-	-	-	-	-	-	-
		-	-	-	-	-	-	-	-	-	-	-	-	192192	-	-	-	-	-	-	-	-	-	-	-	LmjF15.0540	-	-	-	213633	-	-	-	-	-	-	-	-	-	-	-	-	-	-	-	-	-	-	-	-	-	-
		-	-	-	-	-	-	-	-	-	-	-	-	192188	-	-	-	-	-	-	-	-	-	-	-	-	-	-	-	-	-	-	-	-	-	-	-	-	-	-	-	-	-	-	-	-	-	-	-	-	-	-
		-	-	-	-	-	-	-	-	-	-	-	-	192179	-	-	-	-	-	-	-	-	-	-	-	-	-	-	-	-	-	-	-	-	-	-	-	-	-	-	-	-	-	-	-	-	-	-	-	-	-	-
		-	-	-	-	-	-	-	-	-	-	-	-	192178	-	-	-	-	-	-	-	-	-	-	-	-	-	-	-	-	-	-	-	-	-	-	-	-	-	-	-	-	-	-	-	-	-	-	-	-	-	-
		-	-	-	-	-	-	-	-	-	-	-	-	192177	-	-	-	-	-	-	-	-	-	-	-	-	-	-	-	-	-	-	-	-	-	-	-	-	-	-	-	-	-	-	-	-	-	-	-	-	-	-
		-	-	-	-	-	-	-	-	-	-	-	-	192176	-	-	-	-	-	-	-	-	-	-	-	-	-	-	-	-	-	-	-	-	-	-	-	-	-	-	-	-	-	-	-	-	-	-	-	-	-	-
		-	-	-	-	-	-	-	-	-	-	-	-	192175	-	-	-	-	-	-	-	-	-	-	-	-	-	-	-	-	-	-	-	-	-	110625	-	-	-	-	-	-	-	-	-	-	-	-	-	-	-	-
MBO2	Move Backard Only 2	+	+	-	+	-	-	-	-	55076	BDEG_01977	-	228195	192170	294892	-	-	OTTDARP00000008559	-	-	-	-	ENSGALP00000013511	-	NP_065930.2	LmjF34.2480	121573	-	62959	158054	-	-	-	GSPATP00001410001	-	93259	108283	-	-	-	-	-	-	-	51.m00278	7910	-	-	25970	90372.m00042	Tb927.4.2140	-
		-	-	-	-	-	-	-	-	-	-	-	-	192167	-	-	-	-	-	-	-	-	-	-	-	-	-	-	-	-	-	-	-	-	-	-	-	-	-	-	-	-	-	-	-	-	-	-	-	-	-	-
		-	-	-	-	-	-	-	-	-	-	-	-	192166	-	-	-	-	-	-	-	-	-	-	-	-	-	-	-	-	-	-	-	-	-	-	-	-	-	-	-	-	-	-	-	-	-	-	-	-	-	-
		-	-	-	-	-	-	-	-	-	-	-	-	192165	-	-	-	-	-	-	-	-	-	-	-	-	-	-	-	-	-	-	-	-	-	-	-	-	-	-	-	-	-	-	-	-	-	-	-	-	-	-
NFA3401	Nucleosome assembly protein	-	+	+	-	-	-	GB14059-PA	AT2G19480.2	-	BDEG_07324	CE04306	163453	192162	288885	-	-	OTTDARP00000023140	DDB0190153	CG5330-PA	-	171.m00087	ENSGALP00000016623	-	OTTHUMP00000041622	-	103941	-	81952	236778	NCU01438	Os05g46230.2	4059	GSPATP00012503001	17779	225989	134487	PFL0185c	785327	RO3G_07999.1	YKR048C	SPCC364.06	GLEAN3_16631	SINFRUP00000152184	-	38073	TA04600	49.m00052	32481	-	Tb09.160.4240	UM05143.1
ASA7	F1Fo ATP synthase Associated 19.5 kDa protein, mitochondrial	-	-	-	-	-	-	-	-	-	-	-	-	192157	-	-	-	-	-	-	-	-	-	-	-	-	-	-	-	-	-	-	-	-	-	-	-	-	-	-	-	-	-	-	-	-	-	-	-	-	-	-
		-	-	-	-	-	-	-	-	-	-	-	-	-	-	-	-	-	-	-	-	-	-	-	-	-	-	-	-	-	-	-	-	-	-	-	-	-	-	-	-	-	-	-	-	-	-	-	-	-	-	-
		-	-	-	-	-	-	-	-	-	BDEG_01279	-	-	192150	-	-	-	-	-	-	-	-	-	-	OTTHUMP00000031691	-	-	-	-	-	-	-	-	-	-	-	137358	-	-	RO3G_03053.1	-	-	-	-	-	-	-	-	-	-	-	-
GCLP1	gamma carbonic anhydrase like protein 1	-	+	-	-	-	-	GB14831-PA	AT1G47260.1	34918	-	-	146607	192148	-	cgd5_3750	CMM052C	-	DDB0218053	-	-	203.m00115	-	-	-	-	203990	-	56279	154217	-	Os12g07220.1	-	-	35304	107913	139501	-	830061	-	-	-	-	-	10.m00418	22596	-	334.m00001	-	-	-	-
		-	+	-	-	-	-	-	AT3G48390.1	11348	-	-	170810	192147	251620	cgd2_2350	-	OTTDARP00000010814	-	CG10990-PA	-	-	ENSGALP00000014143	-	NP_663314.1	-	114600	8187	-	233387	-	Os08g02690.1	32879	-	50049	128768	138394	PF14_0546	666576	-	-	-	-	SINFRUP00000153483	-	264946	-	76.m00019	64126	-	-	-
ASA2	F1Fo ATP synthase Associated 45,5 kDa protein 2, mitochondrial	-	-	-	-	-	-	-	-	-	-	-	-	192142	-	-	-	-	-	-	-	-	-	-	-	-	-	-	-	-	-	-	-	-	-	-	-	-	-	-	-	-	-	-	-	-	-	-	-	-	-	-
ELG33	exostosin-like glycosyltransferase	-	+	-	-	-	-	-	-	-	-	-	-	192138	-	-	-	-	-	-	-	149.m00101	-	-	-	-	-	-	-	-	-	-	21436	GSPATP00022053001	-	-	139485	-	-	-	YLR336C	-	-	-	-	262041	-	-	-	-	-	-
		-	-	-	-	-	-	-	-	-	-	-	-	192130	-	-	-	-	-	-	-	-	-	-	-	-	-	-	-	-	-	-	-	-	-	-	-	-	-	-	-	-	-	-	-	-	-	-	-	-	-	-
		-	-	-	-	-	-	-	-	-	-	-	-	192126	-	-	-	-	-	-	-	-	-	-	-	-	-	-	-	-	-	-	-	-	-	-	-	-	-	-	-	-	-	-	-	-	-	-	-	-	-	-
		-	+	+	-	-	-	GB19570-PA	AT5G22330.1	39047	BDEG_07389	CE08426	21868	192123	208880	cgd7_2090	CMH022C	-	DDB0191832	CG4003-PA	19171386	144.m00093	ENSGALP00000009528	GL50803_9825	OTTHUMP00000172686	LmjF34.3500	201543	19122	56438	186514	NCU03482	Os07g08170.1	17022	GSPATP00004652001	19568	64922	108757	PF11_0071	293726	RO3G_13691.1	YDR190C	SPAPB8E5.09	GLEAN3_08115	SINFRUP00000127945	58.m00182	24527	TA10610	113.m00777	50758	88123.m00044	Tb927.4.1270	UM06201.1
		-	-	-	-	-	-	-	-	-	-	-	-	192115	-	-	-	-	-	-	-	-	-	-	-	-	-	-	-	-	-	-	-	-	-	-	-	-	-	-	-	-	-	-	-	-	-	-	-	-	-	-
		-	-	-	-	-	-	-	-	-	-	-	-	192114	-	-	-	-	-	-	-	-	-	-	-	-	-	-	-	-	-	-	-	-	-	-	-	-	-	-	-	-	-	-	-	-	-	-	-	-	-	-
		-	-	-	-	-	-	-	-	-	-	-	-	192113	-	-	-	-	-	-	-	-	-	-	-	-	-	-	-	-	-	-	-	-	-	-	-	-	-	-	-	-	-	-	-	-	-	-	-	-	-	-
		-	-	-	-	-	-	-	-	-	-	-	-	192111	-	-	-	-	-	-	-	-	-	-	-	-	-	-	-	-	-	Os03g34300.2	-	-	-	-	-	-	-	-	-	-	-	-	-	22223	-	-	-	-	-	-
		-	-	-	-	-	-	-	-	-	-	-	-	192108	-	-	-	-	-	-	-	-	-	-	-	-	-	-	-	-	-	-	-	-	-	-	-	-	-	-	-	-	-	-	-	-	-	-	-	-	-	-
		-	-	-	-	-	-	-	-	-	-	-	-	192100	-	-	-	-	-	-	-	-	-	-	-	-	-	-	-	-	-	-	-	-	-	-	-	-	-	-	-	-	-	-	-	-	-	-	-	-	-	-
TEF5	Rieske [2Fe-2S] domain, putative, chloroplast location proposed	-	+	-	-	-	-	-	AT1G71500.1	-	-	-	-	192099	-	-	-	-	-	-	-	-	-	-	-	-	-	-	-	-	-	Os11g13850.3	16884	-	-	46753	-	-	267982	-	-	-	-	-	-	-	-	-	-	-	-	-
		-	-	-	-	-	-	-	-	-	-	-	-	192098	-	-	-	-	-	-	-	-	-	-	-	-	-	-	-	-	-	-	-	-	-	-	-	-	-	-	-	-	-	-	-	-	-	-	-	-	-	-
NAR1;1		-	-	-	-	-	-	-	-	53005	-	-	-	192094	-	-	-	-	-	-	-	-	-	-	-	-	-	-	55063	-	-	-	-	-	13076	-	-	-	-	-	-	-	-	-	-	3393	-	-	-	-	-	-
		-	+	-	-	-	-	-	AT1G12940.1	-	-	-	-	192090	-	-	-	-	-	-	-	-	-	-	-	-	-	-	-	-	NCU07205	Os02g02190.1	24168	-	-	154919	-	-	-	-	-	-	-	-	-	-	-	-	-	-	-	-
NRT2;2		-	-	-	-	-	-	-	-	-	-	-	-	192088	-	-	-	-	-	-	-	-	-	-	-	-	-	-	-	61402	-	-	-	-	-	175301	140564	-	-	-	-	-	-	-	-	-	-	-	-	-	-	-
Nii1		-	+	-	-	-	-	-	AT2G15620.1	10808	-	-	201957	192085	-	-	-	-	-	-	-	-	-	-	-	-	-	-	-	224427	-	Os01g25484.2	26396	-	12902	193361	-	-	837131	-	-	-	-	-	-	262125	-	-	-	-	-	UM03848.1
MDHP	NADP-Malate Dehydrogenase	-	+	-	+	-	-	GB12615-PA	AT5G58330.1	-	-	-	-	192083	261983	-	CMP193C	-	-	-	-	-	-	-	-	-	-	-	-	-	-	Os08g44810.1	34202	-	-	93518	-	-	820232	-	-	-	-	-	2.m02347	-	-	-	-	-	-	-
		-	-	-	-	-	-	-	-	-	-	-	-	192075	-	-	-	-	-	-	-	-	-	-	-	-	-	-	-	-	-	-	-	-	-	-	-	-	-	-	-	-	-	-	-	-	-	-	-	-	-	-
		-	-	-	-	-	-	-	-	-	-	-	-	192060	-	-	-	-	-	-	-	-	-	-	-	-	-	-	-	-	-	-	-	-	-	-	-	-	-	-	-	-	-	-	-	-	-	-	-	-	-	-
		-	-	-	-	-	-	-	-	-	-	-	-	192056	-	-	-	-	-	-	-	-	-	-	-	-	-	-	-	-	-	-	-	-	-	-	-	-	-	-	-	-	-	-	-	-	-	-	-	-	-	-
		-	+	-	-	-	-	GB10909-PA	AT4G31580.2	-	-	CE29698	155905	192053	267437	-	-	OTTDARP00000018700	-	CG10203-PA	-	-	ENSGALP00000022386	-	OTTHUMP00000128261	-	98720	-	-	137721	NCU05800	Os02g54770.2	-	-	-	143810	-	-	583120	-	-	-	GLEAN3_16548	SINFRUP00000171556	18.m00379	-	-	27.m00859	-	-	-	-
		-	-	-	-	-	-	-	-	-	-	-	-	192051	-	-	-	-	-	-	-	-	-	-	-	-	-	-	-	-	-	-	-	-	-	-	-	-	-	-	-	-	-	-	-	-	-	-	-	-	-	-
HAM3401	Histone acetyltransferase	-	+	+	-	-	-	GB15903-PA	AT5G09740.1	34939	BDEG_00351	CE21225	138964	192045	297446	cgd6_2590	CML102C	OTTDARP00000020620	DDB0205993	CG6121-PA	19069317	48.m00190	-	GL50803_2851	OTTHUMP00000081253	LmjF14.0140	195151	28848	60479	167841	NCU05218	Os07g43360.1	34906	GSPATP00020067001	3062	115486	137858	PF11_0192	754644	RO3G_02589.1	YOR244W	SPAC637.12c	GLEAN3_08304	SINFRUP00000129410	29.m00290	36275	TA06835	641.m01473	55881	87481.m00126	Tb927.7.4560	UM05240.1
		-	-	-	-	-	-	-	-	-	-	-	-	192044	-	-	-	-	-	-	-	-	-	-	-	-	-	-	-	-	-	-	-	-	-	-	-	-	-	-	-	-	-	-	-	-	-	-	-	-	-	-
		-	-	-	-	-	-	-	-	-	-	-	-	192043	-	-	-	-	-	-	-	-	-	-	-	-	-	-	-	-	-	-	-	-	-	-	-	-	-	-	-	-	-	-	-	-	-	-	-	-	-	-
		-	-	-	-	-	-	-	-	-	-	-	-	192041	-	-	-	-	-	-	-	-	-	-	-	-	-	-	-	-	-	-	-	-	-	-	-	-	-	-	-	-	-	-	-	-	-	-	-	-	-	-
		-	-	-	-	-	-	-	-	-	-	-	-	192033	-	-	-	-	-	-	-	-	-	-	-	-	-	-	-	-	-	-	-	-	-	-	-	-	-	-	-	-	-	-	-	-	-	-	-	-	-	-
	matrix metalloproteinase-like protein	-	-	-	-	-	-	-	-	-	-	-	-	192029	-	-	-	-	-	-	-	-	-	-	-	-	-	-	-	-	-	-	-	-	-	-	-	-	-	-	-	-	-	-	-	-	-	-	-	-	-	-
		-	-	-	-	-	-	-	-	-	-	-	-	192024	-	-	-	-	-	-	-	-	-	-	-	-	-	-	-	-	-	-	-	-	-	-	-	-	-	-	-	-	-	-	-	-	-	-	-	-	-	-
		-	+	-	-	-	-	GB10999-PA	AT2G47210.1	-	BDEG_04164	CE29836	174434	192018	388569	cgd5_1120	CMN257C	OTTDARP00000020674	DDB0188089	CG11132-PA	-	-	ENSGALP00000016413	-	OTTHUMP00000008846	-	179116	39102	69970	162604	NCU04002	Os05g46330.1	32860	-	45548	119332	130750	PFF1385c	807470	-	YGR002C	SPAC9G1.13c	-	SINFRUP00000127270	37.m00314	-	TA09535	31.m00899	59326	-	-	UM00948.1
		-	-	-	-	-	-	-	-	-	-	-	-	192014	-	-	-	-	-	-	-	-	-	-	-	-	-	-	-	-	-	-	-	-	-	-	-	-	-	-	-	-	-	-	-	-	-	-	-	-	-	-
		-	-	-	-	-	-	-	-	-	-	-	-	192012	-	-	-	-	-	-	-	-	-	-	-	-	-	-	-	-	-	-	-	-	-	-	-	-	-	-	-	-	-	-	-	-	-	-	-	-	-	-
		-	-	-	-	-	-	-	-	-	-	-	-	192007	-	-	-	-	-	-	-	-	-	-	-	-	-	-	-	-	-	-	-	-	-	-	-	-	-	-	-	-	-	-	-	-	-	-	-	-	-	-
FAP239	Flagellar Associated Protein	-	+	-	+	-	-	-	-	66018	-	-	-	192005	-	-	-	-	-	-	-	-	-	-	-	-	117952	-	47385	112977	-	-	-	-	-	-	158165	-	-	-	-	-	-	-	-	-	-	-	-	-	-	-
		-	-	-	-	-	-	-	-	-	-	-	-	192003	-	-	-	-	-	-	-	-	-	-	-	-	-	-	-	-	-	-	-	-	-	-	-	-	-	-	-	-	-	-	-	-	-	-	-	-	-	-
		-	+	-	-	-	-	-	AT5G55570.1	-	-	-	-	191999	-	-	-	-	-	-	-	-	-	-	-	-	-	-	-	-	-	Os01g55670.1	35926	-	-	142011	-	-	272808	-	-	-	-	-	-	-	-	-	-	-	-	-
		-	-	-	-	-	-	-	-	-	-	-	-	191996	-	-	-	-	-	-	-	-	-	-	-	-	-	-	-	-	-	-	-	-	-	-	-	-	-	-	-	-	-	-	-	-	-	-	-	-	-	-
SHKH1	chorismate synthase, putative	-	+	-	-	-	-	-	AT1G48850.1	28035	BDEG_08669	-	141106	191993	-	-	CME064C	-	-	-	-	-	-	-	-	-	-	32779	-	223774	NCU05420	Os03g14990.1	23375	GSPATP00016892001	43429	178066	110337	PFF1105c	724971	RO3G_15462.1	YGL148W	SPCC1223.14	-	-	5.m00504	38964	-	20.m00001	-	-	-	UM01402.1
TEF10	Unknown function, chloroplast location proposed	-	+	-	-	-	-	-	AT1G67700.2	-	-	-	-	191988	-	-	CMJ126C	-	-	-	-	-	-	-	-	-	-	-	-	-	-	Os02g38820.1	30397	-	-	129970	-	-	726142	-	-	-	-	-	-	-	-	-	-	-	-	-
ARGC1	N-acetyl-gamma-glutamyl-phosphate reductase, chloroplast precursor	-	+	-	-	-	-	GB17340-PA	AT2G19940.2	72250	-	-	99177	191987	-	-	CMN048C	-	DDB0186887	-	-	-	-	-	-	-	-	-	-	-	NCU00567	Os03g42110.1	4532	-	36913	133210	139358	-	267747	RO3G_04935.1	YER069W	SPAC4G9.09c	-	-	-	21290	-	-	-	-	-	UM00945.1
		-	-	-	-	-	-	-	-	-	-	-	-	191980	-	-	-	-	-	-	-	-	-	-	-	-	-	-	-	-	-	-	-	-	-	-	-	-	-	-	-	-	-	-	-	-	-	-	-	-	-	-
		-	-	-	-	-	-	-	-	-	-	-	-	191972	-	-	-	-	-	-	-	-	-	-	-	-	-	-	-	-	-	-	-	-	-	-	-	-	-	-	-	-	-	-	-	-	-	-	-	-	-	-
		-	-	-	-	-	-	-	-	-	-	-	-	191971	-	-	-	-	-	-	-	-	-	-	-	-	-	-	-	-	-	-	-	-	-	-	-	-	-	-	-	-	-	-	-	-	-	-	-	-	-	-
		-	-	-	-	-	-	-	-	-	-	-	-	191969	-	-	-	-	-	-	-	-	-	-	-	-	-	-	-	239023	-	-	-	-	-	-	-	-	-	-	-	-	-	-	-	-	-	-	-	-	-	-
		-	+	-	-	-	-	-	AT3G53180.1	-	-	-	-	191968	-	-	-	-	DDB0168992	-	-	-	-	-	-	-	-	-	-	-	NCU04264	Os10g31820.2	-	-	-	199156	-	-	589509	-	-	-	-	-	-	-	-	-	-	-	-	-
		-	-	-	-	-	-	-	-	-	-	-	-	191964	-	-	-	-	-	-	-	-	-	-	-	-	-	-	-	-	-	-	-	-	-	-	-	-	-	-	-	-	-	-	-	-	-	-	-	-	-	-
FAP17	Flagellar Associated Protein of with ankyrin repeats	-	-	-	+	-	-	-	-	-	-	-	-	191962	-	-	-	-	-	-	-	-	-	-	-	-	-	39362	-	97653	-	-	-	-	-	-	-	-	-	-	-	-	-	-	-	-	-	-	-	-	-	-
		-	-	-	-	-	-	-	-	-	-	-	-	191960	-	-	-	-	-	-	-	-	-	-	-	-	-	-	-	-	-	-	-	-	-	-	-	-	-	-	-	-	-	-	-	-	-	-	-	-	-	-
		-	+	-	-	-	-	-	-	-	-	-	-	191958	-	-	-	-	-	-	-	-	-	-	-	-	-	5446	1957	-	-	-	-	-	36001	161943	137560	-	-	-	-	-	-	-	-	-	-	-	-	-	-	-
MRPS1	Putative mitochondrial ribosomal protein S1, imported to mitochondria	-	+	-	-	-	-	-	AT3G23700.1	-	-	-	149074	191951	-	-	-	-	-	-	-	-	-	-	-	-	-	-	-	-	-	Os03g62780.1	31480	-	-	147942	-	-	246437	-	-	-	-	-	-	-	TA19550	-	-	-	-	-
		-	-	-	-	-	-	-	-	-	-	-	-	191942	-	-	-	-	-	-	-	-	-	-	-	-	-	-	-	-	-	-	-	-	-	-	-	-	-	-	-	-	-	-	-	-	-	-	-	-	-	-
		-	-	-	-	-	-	-	-	-	-	-	-	191939	-	-	-	-	-	-	-	-	-	-	-	-	-	-	-	-	-	-	-	-	-	-	-	-	-	-	-	-	-	-	-	-	-	-	-	-	-	-
		-	-	-	-	-	-	-	-	-	-	-	-	191938	-	-	-	-	-	-	-	-	-	-	-	-	-	-	-	-	-	-	-	-	-	-	-	-	-	-	-	-	-	-	-	-	-	-	-	-	-	-
		-	-	-	-	-	-	-	-	-	-	-	-	191936	-	-	-	-	-	-	-	-	-	-	-	-	-	-	-	-	-	-	-	-	-	-	-	-	-	-	-	-	-	-	-	-	-	-	-	-	-	-
		-	-	-	-	-	-	-	-	-	-	-	-	191923	-	-	-	-	-	-	19171363	-	-	-	-	-	-	-	-	-	-	-	-	-	-	-	-	-	-	-	-	-	-	-	-	-	-	-	-	-	-	-
		-	-	-	-	-	-	-	-	-	-	-	-	191920	-	-	-	-	-	-	-	-	-	-	-	-	-	-	-	-	-	-	-	-	-	-	-	-	-	-	-	-	-	-	-	-	-	-	-	-	-	-
		-	-	-	-	-	-	-	-	-	-	-	-	191918	-	-	-	-	-	-	-	-	-	-	-	-	-	-	-	-	-	-	-	-	-	-	-	-	-	-	-	-	-	-	-	-	-	-	-	-	-	-
		-	-	-	-	-	-	-	-	-	-	-	-	191913	-	-	-	-	-	-	-	-	-	-	-	-	-	-	-	-	-	-	-	-	-	-	-	-	-	-	-	-	-	-	-	-	-	-	-	-	-	-
		-	-	-	-	-	-	-	-	-	-	-	-	191910	-	-	-	-	-	-	-	-	-	-	-	-	-	-	-	209853	-	Os11g36860.1	-	-	-	-	-	-	593623	RO3G_02687.1	-	-	-	-	-	-	-	-	-	-	-	-
UBA1	ubiquitin-activating enzyme E1	-	+	+	+	-	-	GB15854-PA	AT5G06460.1	54097	BDEG_04446	CE05449	162959	191903	204702	cgd4_2300	CMI034C	OTTDARP00000022145	DDB0190927	CG1782-PA	19168718	334.m00049	ENSGALP00000019178	GL50803_10661	OTTHUMP00000023197	LmjF23.0550	113559	33365	37722	129964	NCU04370	Os11g01510.3	351	GSPATP00021971001	54754	129081	108439	PFL1245w	815464	RO3G_16293.1	YKL210W	SPBC1604.21c	-	SINFRUP00000148258	188.m00079	268541	TA14590	80.m02267	25768	80459.m00170	Tb927.8.2640	UM06188.1
		-	-	-	-	-	-	-	-	-	-	-	-	191893	-	-	-	-	-	-	-	-	-	-	-	-	-	-	-	-	-	-	-	-	-	-	-	-	-	-	-	-	-	-	-	-	-	-	-	-	-	-
		-	+	-	-	-	-	-	-	70668	-	-	-	191891	-	-	-	-	-	-	-	-	-	-	-	-	173853	-	-	-	-	-	31422	-	45058	-	-	-	551794	-	-	-	-	-	-	-	-	-	-	-	-	-
		-	-	-	-	-	-	-	-	63903	-	-	197017	191889	-	-	-	-	-	-	-	-	-	-	-	-	-	-	-	-	-	-	-	-	-	165621	-	-	-	-	-	-	-	-	-	-	-	-	-	-	-	-
		-	-	-	-	-	-	-	-	-	-	-	-	191884	-	-	-	-	-	-	-	-	-	-	-	-	-	-	-	-	-	-	-	-	-	-	-	-	-	-	-	-	-	-	-	9860	-	-	-	-	-	-
FAP21	Flagellar Associated Protein	-	-	-	-	-	-	-	-	-	-	-	-	191879	-	-	-	-	-	-	-	-	-	-	-	-	-	-	-	-	-	-	-	-	-	-	-	-	-	-	-	-	-	-	-	-	-	-	-	-	-	-
FAP281	Flagellar Associated Coiled-Coil Protein	-	-	-	-	-	-	-	-	-	-	-	-	191876	-	-	-	-	-	-	-	-	-	-	-	-	-	-	-	-	-	-	-	-	-	-	-	-	-	-	-	-	-	-	-	-	-	-	-	-	-	-
FUT11	alpha 1,3 fucosyltransferase	-	+	-	-	-	-	GB17182-PA	AT3G19280.1	24165	-	CE32206	179280	191875	282649	-	-	-	DDB0218615	CG4435-PA	-	-	ENSGALP00000036990	-	NP_116053.3	LmjF02.0330	95386	-	74046	97787	-	Os08g36840.1	24247	-	46110	206064	-	-	201717	RO3G_04850.1	-	-	GLEAN3_26639	SINFRUP00000138199	-	5510	-	-	3761	-	-	-
		-	+	-	-	-	-	-	-	-	BDEG_00012	-	198457	191871	-	-	-	-	-	-	-	-	-	-	-	-	-	-	-	-	-	-	30709	-	32342	-	-	-	-	-	-	-	-	-	-	10524	-	-	-	-	-	-
		-	+	-	-	-	-	-	AT3G57420.1	68744	-	CE38377	204971	191870	-	-	-	-	-	-	-	-	-	-	-	-	230450	-	-	243874	-	-	24139	-	-	127388	-	-	-	-	-	-	-	-	-	-	-	-	-	-	-	-
		-	-	-	-	-	-	-	AT1G36320.1	-	-	-	-	191867	-	-	-	-	-	-	-	-	-	-	-	-	-	-	-	-	-	Os02g18940.1	-	-	-	112718	-	-	716554	-	-	-	-	-	-	-	-	-	-	-	-	-
		-	-	-	-	-	-	-	-	-	-	-	-	191866	-	-	-	-	-	-	-	-	-	-	-	-	-	-	-	-	-	-	-	-	-	-	-	-	-	-	-	-	-	-	-	-	-	-	-	-	-	-
		-	-	-	-	-	-	-	-	-	-	-	-	191865	-	-	-	-	-	-	-	-	-	-	-	-	-	-	-	-	-	-	-	-	-	-	-	-	-	-	-	-	-	-	-	-	-	-	-	-	-	-
ATPvD2		-	+	+	-	-	-	GB15226-PA	AT3G58730.1	32568	BDEG_08233	CE00209	183256	191863	285834	cgd5_530	CMD007C	OTTDARP00000019393	DDB0167892	CG8186-PA	19069265	156.m00109	ENSGALP00000015572	GL50803_3678	OTTHUMP00000028402	LmjF35.0700	233963	15580	74068	104847	NCU08035	Os04g55040.2	20232	GSPATP00024914001	51058	224180	141251	PF13_0227	641796	RO3G_08247.1	YEL051W	SPCC965.03	GLEAN3_12628	SINFRUP00000145973	140.m00061	39173	TA13470	74.m00445	63587	81616.m00168	Tb10.70.3600	UM04167.1
FAP294	Flagellar Associated Coiled-Coil Protein	-	-	-	-	-	-	-	-	-	-	-	-	191852	-	-	-	-	-	-	-	-	-	-	-	-	-	-	-	-	-	-	-	-	-	-	-	-	-	-	-	-	-	-	-	-	-	-	-	-	-	-
		-	-	-	-	-	-	-	-	-	-	-	-	191851	-	-	-	-	-	-	-	-	-	-	-	-	-	-	-	-	-	-	-	-	-	-	-	-	-	-	-	-	-	-	-	-	-	-	-	-	-	-
		-	-	-	-	-	-	-	-	-	-	-	-	191848	-	-	-	-	-	-	-	-	-	-	-	-	-	-	-	-	-	-	-	-	-	-	-	-	-	-	-	-	-	-	-	-	-	-	-	-	-	-
	related to gibberellin 20 oxidase	-	-	-	-	-	-	-	-	-	-	-	-	191845	-	-	-	-	-	-	-	-	-	-	-	-	-	-	-	-	NCU01364	-	-	-	-	-	-	-	-	-	-	-	-	-	-	-	-	-	-	-	-	-
		-	-	-	-	-	-	-	-	-	-	-	-	191843	-	-	-	-	-	-	-	-	-	-	-	-	-	-	-	-	-	-	-	-	-	-	-	-	-	-	-	-	-	-	-	-	-	-	-	-	-	-
		-	-	-	-	-	-	-	-	-	-	-	-	191838	-	-	-	-	-	-	-	-	-	-	-	-	-	-	-	-	-	-	-	-	-	-	-	-	-	-	-	-	-	-	-	-	-	-	-	-	-	-
COG2	Component of oligomeric golgi complex 2	-	+	-	-	-	-	GB18283-PA	AT4G24840.1	-	BDEG_05284	CE28817	184924	191834	-	-	-	OTTDARP00000001932	DDB0204051	-	-	-	ENSGALP00000018082	-	OTTHUMP00000035875	-	232605	37489	-	83212	-	Os03g63250.2	16275	-	44776	162850	129958	-	824748	-	-	-	GLEAN3_13719	SINFRUP00000180528	-	11664	-	-	26404	-	-	-
		-	+	-	-	-	-	-	AT4G14490.1	-	-	-	-	191829	-	-	-	-	-	-	-	-	-	-	-	-	-	-	-	-	-	-	-	-	45323	9094	140133	-	-	-	-	-	-	-	71.m00199	-	-	-	-	-	-	-
ASA8	putative F1Fo ATP synthase Associated 10,0 kDa protein, mitochondrial	-	-	-	-	-	-	-	-	-	-	-	-	191828	-	-	-	-	-	-	-	-	-	-	-	-	-	-	-	-	-	-	-	-	-	-	-	-	-	-	-	-	-	-	-	-	-	-	-	-	-	-
		-	-	-	-	-	-	-	-	-	-	-	-	191827	-	-	-	-	-	-	-	-	-	-	-	-	-	-	-	-	-	-	-	-	-	-	-	-	-	-	-	-	-	-	-	-	-	-	-	-	-	-
		-	-	-	-	-	-	-	-	-	-	-	-	191824	-	-	-	-	-	-	-	-	-	-	-	-	-	-	-	-	-	-	-	-	-	-	-	-	-	-	-	-	-	-	-	-	-	-	-	-	-	-
		-	-	-	-	-	-	-	-	-	-	-	-	191821	-	-	-	-	-	-	-	-	-	-	-	-	-	-	-	-	-	-	-	-	-	-	-	-	-	-	-	-	-	-	-	-	-	-	-	-	-	-
		-	+	-	-	-	-	-	AT1G29250.1	-	-	-	-	191820	-	-	-	-	-	-	-	-	-	-	-	LmjF13.0450	-	-	4975	-	-	Os01g07810.1	32828	-	-	233865	-	-	595398	-	-	-	-	-	-	-	-	-	-	-	Tb11.02.2030	-
		-	+	+	-	-	-	GB13746-PA	AT4G03120.1	-	BDEG_04014	CE09232	227784	191815	369901	cgd5_4060	-	-	DDB0205382	-	-	13.m00336	ENSGALP00000004369	-	OTTHUMP00000016238	-	104026	34150	66742	244745	NCU01341	Os02g16640.1	31135	GSPATP00019950001	45840	167707	142997	PF08_0084	712203	RO3G_16187.1	YLR298C	SPBP35G2.09	GLEAN3_20766	SINFRUP00000181957	45.m00264	21280	TA09515	541.m01233	9459	88115.m00029	-	UM05608.1
		-	-	-	-	-	-	-	-	-	-	-	-	191813	-	-	-	-	-	-	-	-	-	-	-	-	-	-	-	-	-	-	-	-	-	-	-	-	-	-	-	-	-	-	-	-	-	-	-	-	-	-
		-	-	-	-	-	-	-	-	-	-	-	-	191810	-	-	-	-	-	-	-	-	-	-	-	-	-	-	-	-	-	-	-	-	-	-	-	-	-	-	-	-	-	-	-	-	-	-	-	-	-	-
AMT5	Ammonia channel	-	-	-	-	-	-	-	-	-	-	-	-	191809	-	-	-	-	-	-	-	-	-	-	-	-	-	-	55677	-	-	-	-	GSPATP00027856001	-	-	-	-	-	-	-	-	-	-	-	-	-	-	-	-	-	UM04523.1
		-	-	-	-	-	-	-	-	-	-	-	-	191808	-	-	-	-	-	-	-	-	-	-	-	-	-	-	-	-	-	-	-	-	-	-	-	-	-	-	-	-	-	-	-	-	-	-	-	-	-	-
		-	-	-	-	-	-	-	-	-	-	-	-	191806	-	-	-	-	-	-	-	-	-	-	-	-	-	-	-	-	-	-	-	-	-	-	-	-	-	-	-	-	-	-	-	-	-	-	-	-	-	-
		-	-	-	-	-	-	-	-	-	-	-	-	191804	-	-	-	-	-	-	-	-	-	-	-	-	-	-	-	-	-	-	-	-	-	-	-	-	-	-	-	-	-	-	-	-	-	-	-	-	-	-
		-	-	-	-	-	-	-	-	-	-	-	-	191803	-	-	CMR506C	-	-	-	-	-	-	-	-	-	-	-	-	-	-	-	-	-	-	-	-	-	-	-	-	-	-	-	-	2502	-	-	-	-	-	-
		-	-	-	-	-	-	-	-	-	-	-	-	191800	-	-	-	-	-	-	-	-	-	-	-	-	-	-	-	-	-	-	-	-	-	-	-	-	-	-	-	-	-	-	-	-	-	-	-	-	-	-
		-	-	-	-	-	-	-	-	-	-	-	-	191797	-	-	-	-	-	-	-	-	-	-	-	-	-	-	-	-	-	-	-	-	-	-	-	-	-	-	-	-	-	-	-	-	-	-	-	-	-	-
FAP199	Flagellar Associated Protein	-	-	-	+	-	-	-	-	-	-	-	-	191796	-	-	-	-	-	-	-	-	-	-	-	-	-	-	-	-	-	-	-	-	-	-	-	-	-	-	-	-	-	-	-	-	-	-	-	-	-	-
		-	-	-	-	-	-	-	-	-	-	-	-	191786	224401	-	-	-	-	-	-	-	-	-	-	-	-	-	-	-	-	-	-	-	45226	159819	-	-	-	-	-	-	-	-	-	-	-	-	-	-	-	-
		-	-	-	-	-	-	-	-	-	-	-	-	191783	-	-	-	-	-	-	-	-	-	-	-	-	-	-	-	-	-	-	-	-	-	-	-	-	-	-	-	-	-	-	-	-	-	-	-	-	-	-
RPS5	Ribosomal protein S5, component of cytosolic 80S ribosome and 40S small subunit	-	+	+	-	-	-	GB11132-PA	AT2G37270.2	59875	BDEG_02360	CE06360	223251	191776	223496	cgd6_4320	CMT627C	OTTDARP00000021405	DDB0186804	CG8922-PA	19068734	117.m00154	-	GL50803_12981	OTTHUMP00000070889	LmjF11.0970	109561	37245	39045	160743	NCU09475	Os11g29190.2	26045	GSPATP00018009001	29097	106946	109358	PF07_0088	256059	RO3G_06981.1	YJR123W	SPAC328.10c	GLEAN3_22957	SINFRUP00000159144	358.m00012	29955	TA17380	49.m00006	37197	85859.m00394	Tb11.02.4170	UM00591.1
		-	-	-	-	-	-	-	-	-	-	-	-	-	-	-	-	-	-	-	-	-	-	-	-	-	-	-	-	-	-	-	-	-	-	-	-	-	-	-	-	-	-	-	-	-	-	-	-	-	-	-
		-	-	-	-	-	-	-	-	-	-	-	-	191764	-	-	-	-	-	-	-	-	-	-	-	-	-	-	-	-	-	-	-	-	-	-	-	-	-	-	-	-	-	-	-	-	-	-	-	-	-	-
CYCD2	D-type cyclin homologue	-	-	-	-	-	-	-	-	65889	-	-	-	191762	-	-	-	-	-	-	-	-	-	-	-	-	-	-	-	-	-	-	-	-	-	-	-	-	-	-	-	-	-	-	-	-	-	-	-	-	-	-
		-	-	-	-	-	-	-	-	-	-	-	-	191760	-	-	-	-	-	-	-	-	-	-	-	-	-	-	-	-	-	-	-	-	-	-	-	-	-	-	-	-	-	-	-	-	-	-	-	-	-	-
		-	-	-	-	-	-	-	-	-	-	-	-	191759	-	-	-	-	-	-	-	-	-	-	-	-	-	-	-	-	-	-	-	-	-	-	-	-	-	-	-	-	-	-	-	-	-	-	-	-	-	-
RPL32	Ribosomal protein L32, component of cytosolic 80S ribosome and 60S large subunit	-	+	+	-	-	-	GB10903-PB	AT4G18100.1	36203	BDEG_08152	CE03709	154331	191758	283522	cgd6_3340	CMJ289C	-	DDB0168588	-	19068851	9.m00403	-	GL50803_14049	OTTHUMP00000170276	LmjF21.1720	-	33951	39446	240738	-	Os09g32500.2	23350	PTETP500002001	21659	108328	109525	PFI0190w	746779	RO3G_16038.1	YBL092W	SPAC3H5.10	GLEAN3_03849	SINFRUP00000133774	6.m00440	24410	TA08390	57.m00005	36930	89123.m00093	Tb09.244.2590	UM00925.1
		-	-	-	-	-	-	-	AT5G45170.1	-	-	-	-	191755	-	-	-	-	-	-	-	-	-	-	-	-	-	-	-	-	-	Os12g43520.1	30274	-	-	-	-	-	575523	-	-	-	-	-	-	-	-	-	-	-	-	-
		-	-	-	-	-	-	-	-	-	-	-	-	191752	-	-	-	-	-	-	-	-	-	-	-	-	-	-	-	-	-	-	-	-	-	-	-	-	-	-	-	-	-	-	-	-	-	-	-	-	-	-
MGTE	divalent cation transporter	-	-	-	-	-	-	-	-	-	-	-	217249	191747	-	-	-	-	-	-	-	-	-	-	-	-	-	-	70652	-	-	-	-	-	42545	-	-	-	-	-	-	-	-	-	-	262046	-	-	-	-	-	-
		-	-	-	-	-	-	-	-	-	-	-	-	191746	-	-	-	-	-	-	-	-	-	-	-	-	-	-	-	-	-	-	-	-	-	-	-	-	-	-	-	-	-	-	-	-	-	-	-	-	-	-
		-	-	-	-	-	-	-	-	-	-	-	-	191743	-	-	-	-	DDB0167009	-	-	-	-	-	-	-	-	-	-	-	-	-	-	-	-	-	-	-	-	-	-	-	-	-	-	-	-	-	-	-	-	-
		-	-	-	-	-	-	-	-	-	-	-	-	191740	-	-	-	-	-	-	-	-	-	-	-	-	-	-	-	-	-	-	-	-	-	-	-	-	-	-	-	-	-	-	-	-	-	-	-	-	-	-
		-	-	-	-	-	-	-	-	-	-	-	-	191739	-	-	-	-	-	-	-	-	-	-	-	-	-	-	-	-	-	-	36205	-	-	-	-	-	-	-	-	-	-	-	-	-	-	-	-	-	-	-
		-	-	-	-	-	-	-	-	-	-	-	-	191738	-	-	-	-	-	-	-	-	-	-	-	-	-	-	-	-	-	-	-	-	-	-	-	-	-	-	-	-	-	-	-	-	-	-	-	-	-	-
		-	-	-	-	-	-	-	-	-	-	-	-	191737	-	-	-	-	-	-	-	-	-	-	-	-	-	-	-	-	-	-	-	-	-	-	-	-	-	-	-	-	-	-	-	-	-	-	-	-	-	-
		-	-	-	-	-	-	-	-	-	-	-	-	191731	-	-	-	-	-	-	-	-	-	-	-	-	-	-	-	-	-	-	-	-	-	-	-	-	-	-	-	-	-	-	-	-	-	-	-	-	-	-
		-	-	-	-	-	-	-	-	-	-	-	-	191728	-	-	-	-	-	-	-	-	-	-	-	-	-	-	-	-	-	-	-	-	-	-	-	-	-	-	-	-	-	-	-	-	-	-	-	-	-	-
		-	-	-	-	-	-	-	-	-	-	-	-	191725	-	-	CMN011C	-	-	-	-	-	-	-	-	-	-	-	-	-	-	-	22432	-	-	-	-	-	-	-	-	-	-	-	-	-	-	-	-	-	-	-
		-	-	-	-	-	-	-	-	-	-	-	-	191722	-	-	-	-	-	-	-	-	-	-	-	-	-	-	-	-	-	-	-	-	-	-	-	-	-	-	-	-	-	-	-	-	-	-	-	-	-	-
		-	-	-	-	-	-	-	-	-	-	-	-	191720	-	-	-	-	-	-	-	-	-	-	-	-	-	-	-	-	-	-	-	-	-	-	-	-	-	-	-	-	-	-	-	-	-	-	-	-	-	-
		-	-	-	-	-	-	-	-	-	-	-	-	191719	-	-	-	-	-	-	-	-	-	-	-	-	-	-	-	-	-	-	-	-	-	-	-	-	-	-	-	-	-	-	-	-	-	-	-	-	-	-
		-	-	-	-	-	-	-	-	-	-	-	-	191717	-	-	-	-	-	-	-	-	-	-	-	-	-	-	-	-	-	-	-	-	-	-	-	-	-	-	-	-	-	-	-	-	-	-	-	-	-	-
ALT1,AAT2	alanine aminotransferase	-	-	-	-	-	-	-	-	-	-	-	-	191703	-	-	-	-	-	-	-	-	-	-	-	-	-	-	-	-	-	-	-	-	-	-	-	-	-	-	-	-	-	-	-	-	-	-	-	-	-	-

LhcbM8		-	-	-	-	-	-	-	-	-	-	-	-	191695	-	-	-	-	-	-	-	-	-	-	-	-	-	-	-	-	-	-	-	-	-	-	-	-	-	-	-	-	-	-	-	-	-	-	-	-	-	-
		-	+	-	-	-	-	-	AT1G03350.1	-	-	CE27417	225535	191691	226218	-	-	OTTDARP00000020577	-	-	-	-	ENSGALP00000005376	-	OTTHUMP00000008867	-	157887	-	-	-	NCU03906	Os07g45310.1	-	-	-	172536	134047	-	565291	RO3G_11904.1	-	-	-	SINFRUP00000163549	-	-	-	-	58239	-	-	-
		-	-	-	-	-	-	-	-	-	-	-	-	191686	-	-	-	-	-	-	-	-	-	-	-	-	-	-	-	-	-	-	-	-	-	-	-	-	-	-	-	-	-	-	-	-	-	-	-	-	-	-
		-	-	-	-	-	-	-	-	-	BDEG_03639	-	-	191681	-	-	-	-	-	-	-	-	-	-	-	-	-	-	-	-	-	-	-	-	-	-	-	MAL13P1.25	-	-	-	SPAC3C7.09	-	-	-	-	-	-	-	-	-	-
		-	+	-	-	-	-	-	AT3G21865.1	-	BDEG_02024	-	-	191680	-	-	-	-	-	-	-	-	-	-	-	-	-	-	-	-	-	Os04g53690.1	-	-	-	177723	127058	PF11_0487	679507	-	-	-	-	-	-	-	-	-	-	-	-	-
FAP85	Flagellar Associated Protein	-	-	-	+	-	-	-	-	-	-	-	-	191679	-	-	-	-	-	-	-	-	-	-	-	-	-	-	-	-	-	-	-	-	-	-	-	-	-	-	-	-	-	-	-	-	-	-	-	-	-	-
		-	+	-	-	-	-	-	AT5G19050.1	-	-	-	-	191675	-	-	-	-	-	-	-	-	-	-	-	-	-	-	-	-	NCU00364	Os07g11060.1	-	-	-	220091	-	-	420512	-	-	-	-	-	-	-	-	69.m00139	-	-	-	-
CCD6	Similar to coiled-coil domain 6 protein	-	+	-	-	-	-	GB10097-PA	-	-	BDEG_06401	CE01652	171405	191672	-	-	CMR377C	OTTDARP00000020881	DDB0185818	CG6664-PD	-	-	ENSGALP00000005006	-	OTTHUMP00000019644	-	-	-	-	173973	-	-	33074	-	31991	-	-	-	-	RO3G_02052.1	-	-	-	SINFRUP00000140758	-	-	-	-	-	-	Tb927.8.3810	-
		-	-	-	-	-	-	-	-	-	-	-	-	191671	-	-	-	-	-	-	-	-	-	-	-	-	-	-	-	-	-	-	-	-	-	-	-	-	-	-	-	-	-	-	-	-	-	-	-	-	-	-
ICL1	isocitrate lyase	-	+	-	-	-	-	-	AT3G21720.1	-	BDEG_03049	CE32565	-	191668	-	-	-	-	DDB0217090	-	-	-	-	-	-	-	-	-	-	179675	NCU11238	Os07g34520.2	-	GSPATP00019454001	14401	103725	109161	-	589044	RO3G_03731.1	YER065C	SPBC1683.11c	-	-	251.m00042	35523	-	-	-	-	-	UM04285.1
		-	-	-	-	-	-	-	-	-	-	-	-	191666	-	-	-	-	-	-	-	-	-	-	-	-	-	-	-	-	-	-	31511	-	-	-	-	-	-	-	-	-	-	-	-	-	-	80.m02296	-	-	-	-
		-	-	-	-	-	-	-	-	-	-	-	-	191663	-	-	-	-	-	-	-	-	-	-	-	-	-	-	-	-	-	-	-	-	-	-	-	-	-	-	-	-	-	-	-	-	-	-	-	-	-	-
STA3	soluble starch synthase III , ADP-glucose alpha-1,4 glucane alpha-4-glucanotransferase	-	-	-	-	-	-	-	AT1G11720.1	-	-	-	-	191662	-	-	-	-	-	-	-	-	-	-	-	-	-	-	-	-	-	Os04g53310.1	29372	-	-	-	-	-	196527	-	-	-	-	-	-	-	-	-	-	-	-	-
		-	-	-	-	-	-	-	-	-	-	-	-	191660	-	-	-	-	-	-	-	-	-	-	-	-	-	-	-	-	-	-	-	-	-	-	-	-	-	-	-	-	-	-	-	-	-	-	-	-	-	-
		-	+	-	-	-	-	-	AT5G52220.1	-	BDEG_06090	-	-	191657	-	-	-	OTTDARP00000012884	-	-	-	-	ENSGALP00000000896	-	NP_001034779.1	-	155672	-	68986	217793	-	Os09g12780.1	-	-	-	19478	-	-	824914	-	-	-	-	-	-	-	-	-	-	-	-	-
		-	-	-	-	-	-	-	-	-	-	-	-	191656	-	-	-	-	-	-	-	-	-	-	-	-	-	-	-	-	-	-	-	-	-	-	-	-	-	-	-	-	-	-	-	-	-	-	-	-	-	-
		-	-	-	-	-	-	-	-	-	-	-	-	191655	-	-	-	-	-	-	-	-	-	-	-	-	-	-	-	-	-	-	-	-	-	-	-	-	-	-	-	-	-	-	-	-	-	-	-	-	-	-
CFA1		-	+	-	-	-	-	-	AT3G23510.1	11801	BDEG_03741	CE40383	105870	191650	-	-	CMP046C	-	DDB0185725	-	-	-	-	-	-	-	-	30477	-	168568	NCU01678	Os12g16650.1	536	-	29633	187778	108277	-	803768	RO3G_05852.1	-	-	-	-	-	12662	-	-	55813	-	-	UM04749.1
VPS5A	subunit of Retromer complex	-	+	-	+	-	-	GB15978-PA	AT5G06140.1	2963	BDEG_07106	CE07927	156986	191646	222075	-	-	OTTDARP00000021564	DDB0217073	CG2774-PA	-	-	ENSGALP00000008547	-	NP_003091.2	-	162699	18806	33048	134716	NCU04137	Os01g64280.1	16066	GSPATP00035982001	3137	215465	155641	-	825203	RO3G_06266.1	YOR069W	SPCPJ732.01	-	SINFRUP00000164009	-	262863	-	-	22154	-	Tb09.211.4240	UM05287.1
		-	+	-	-	-	-	-	AT5G44650.1	-	-	-	-	191642	-	-	-	-	-	-	-	-	-	-	-	-	-	-	-	-	-	Os01g58470.1	32303	-	-	142249	-	-	179527	-	-	-	-	-	-	-	-	-	-	-	-	-
		-	-	-	-	-	-	-	-	-	-	-	-	191640	-	-	-	-	-	-	-	-	-	-	-	-	-	-	-	-	-	-	-	-	-	-	-	-	-	-	-	-	-	-	-	-	-	-	-	-	-	-
		-	-	-	-	-	-	-	-	-	-	-	-	191638	-	-	-	-	-	-	-	-	-	-	-	-	-	30014	-	-	-	-	-	-	-	-	-	-	-	-	-	-	-	-	-	-	-	-	-	-	-	-
		-	-	-	-	-	-	-	-	-	-	-	-	191633	-	-	-	-	-	-	-	-	-	-	-	-	-	-	-	-	-	-	-	-	-	-	-	-	-	-	-	-	-	-	-	-	-	-	-	-	-	-
		-	-	-	-	-	-	-	-	-	-	-	-	191632	-	-	-	-	-	-	-	-	-	-	-	-	-	-	-	-	-	-	-	-	-	-	-	-	-	-	-	-	-	-	-	-	-	-	-	-	-	-
MAP1A	methionine aminopeptidase 1A	-	+	-	-	-	-	GB18314-PA	AT2G45240.1	59196	BDEG_06500	CE33852	184699	191628	-	cgd1_2700	CMT169C	OTTDARP00000008830	DDB0205763	-	-	-	ENSGALP00000019961	-	NP_055958.1	LmjF19.0550	228994	32734	-	245141	NCU06923	Os10g36470.2	16078	GSPATP00032184001	12595	63601	109564	PF10_0150	730835	RO3G_11458.1	YLR244C	SPBC3E7.10	-	SINFRUP00000183071	1.m01014	40044	TA07050	50.m00068	55901	-	Tb10.61.1210	-
		-	-	-	-	-	-	-	-	-	-	-	-	191623	-	-	-	-	-	-	-	-	-	-	-	-	-	-	-	-	-	-	-	-	-	-	-	-	-	-	-	-	-	-	-	-	-	-	-	-	-	-
		-	+	-	-	-	-	-	AT3G55850.2	10709	-	-	206365	191621	-	-	-	-	-	-	-	-	-	-	-	LmjF03.0930	-	27363	81873	-	NCU03948	Os01g72350.2	21577	GSPATP00026316001	-	138993	145568	-	414750	-	-	-	-	-	-	-	-	-	-	84414.m00050	-	UM03011.1
ASPS2	aspartyl-tRNA synthetase, probably cytosolic	-	+	+	-	-	-	GB12647-PA	AT4G31180.2	30982	BDEG_03125	CE00015	148058	191617	290174	cgd7_1540	CMJ218C	-	DDB0168814	CG3821-PA	19074329	262.m00059	ENSGALP00000020154	GL50803_10870	OTTHUMP00000162534	LmjF30.0460	118845	38870	82693	205625	NCU07082	Os02g46130.1	20243	GSPATP00014863001	42051	93042	108386	PFA0145c	652970	RO3G_04619.1	YLL018C	SPCC1223.07c	GLEAN3_17781	SINFRUP00000173490	85.m00147	268068	TA04845	20.m03726	56497	89154.m00303	Tb927.6.1880	UM04270.1
		-	-	-	-	-	-	-	-	-	-	-	-	191614	-	-	-	-	-	-	-	-	-	-	-	-	-	-	-	-	-	-	-	-	-	-	-	-	-	-	-	-	-	-	-	-	-	-	-	-	-	-
		-	-	-	-	-	-	-	-	-	-	-	-	191613	-	-	-	-	-	-	-	-	-	-	-	-	-	-	-	110975	-	-	-	-	-	-	-	-	-	-	-	-	-	-	-	-	-	-	-	-	-	-
		-	+	-	-	-	-	GB10289-PA	AT5G19750.1	29835	BDEG_01927	CE35017	92614	191611	294388	-	-	OTTDARP00000016299	DDB0188787	CG11077-PA	-	-	-	-	OTTHUMP00000122591	-	143853	23236	33383	193546	NCU02117	Os11g03670.1	31591	-	15341	161687	137879	-	818987	-	YLR251W	-	-	SINFRUP00000155101	9.m00331	33169	-	-	-	-	-	UM03207.1
		-	-	-	-	-	-	-	-	-	-	-	-	191608	-	-	-	-	-	-	-	-	-	-	-	-	-	-	-	-	-	-	-	-	-	-	-	-	-	-	-	-	-	-	-	-	-	-	-	-	-	-
AGD1	putative arogenate/prephenate dehydrogenase	-	+	-	-	-	-	-	AT1G15710.1	3833	-	-	-	191605	-	-	CMS326C	-	-	-	-	-	-	-	-	-	-	-	-	-	-	Os06g49520.1	4306	-	43288	24068	-	-	298870	RO3G_14282.1	-	SPCC1494.04c	-	-	-	33055	-	-	-	-	-	UM04182.1
		-	-	-	-	-	-	-	-	-	-	-	-	191599	-	-	-	-	-	-	-	-	-	-	-	-	-	-	-	-	-	-	-	-	-	-	-	-	-	-	-	-	-	-	-	-	-	-	-	-	-	-
		-	-	-	-	-	-	-	-	-	-	-	-	191598	-	-	-	-	-	-	-	-	-	-	-	-	-	-	-	-	-	-	-	-	-	-	-	-	-	-	-	-	-	-	-	-	-	-	-	-	-	-
		-	+	-	-	-	-	-	AT1G20925.1	-	-	-	-	191597	-	-	-	-	-	-	-	-	-	-	-	-	-	-	-	-	-	Os09g38130.2	-	GSPATP00021387001	-	131156	-	-	564590	-	YNL095C	SPAC5D6.04	-	-	-	-	-	-	-	-	-	UM03082.1
ATP5	Mitochondrial ATP synthase subunit 5, OSCP subunit	-	+	-	-	-	-	GB15629-PA	AT5G13450.1	27202	BDEG_03163	CE36139	150888	191596	226480	-	-	OTTDARP00000021528	-	-	-	-	ENSGALP00000025745	-	OTTHUMP00000067745	-	183079	18525	-	94346	NCU01606	Os06g43850.2	17250	-	44603	110216	108488	MAL13P1.47	814100	RO3G_09398.1	YDR298C	SPCC1840.06	-	SINFRUP00000152442	-	29359	TA06450	-	63937	-	-	UM06324.1
		-	+	-	-	-	-	-	AT3G23570.1	-	-	-	-	191592	-	-	CMK095C	-	-	-	-	-	-	-	-	-	-	-	72152	-	-	Os05g33100.1	-	-	-	146292	-	-	820918	-	-	SPBC30D10.14	-	-	-	-	-	-	-	-	-	-
		-	-	-	-	-	-	-	-	-	-	-	-	191591	-	-	-	-	-	-	-	-	-	-	-	-	-	-	-	-	-	-	-	-	-	-	-	-	-	-	-	-	-	-	-	-	-	-	-	-	-	-
		-	+	-	-	-	-	GB11573-PA	AT2G04750.1	71753	BDEG_07276	CE26598	148728	191590	210507	-	-	OTTDARP00000019724	DDB0205524	CG8649-PD	-	-	ENSGALP00000009526	-	NP_002289.1	-	203020	-	32936	217852	NCU03992	Os02g48740.1	-	PTETP5900009001	-	198118	127837	-	553250	RO3G_07605.1	YDR129C	SPBC1778.06c	GLEAN3_00091	SINFRUP00000129903	10.m00425	-	-	-	-	84191.m00017	-	UM04768.1
ELI1		-	-	-	-	-	-	-	-	-	-	-	-	191585	-	-	-	-	-	-	-	-	-	-	-	-	-	-	-	-	-	-	-	-	-	-	-	-	-	-	-	-	-	-	-	-	-	-	-	-	-	-
		-	+	-	-	-	-	-	-	-	-	CE26624	-	191584	-	-	CMD171C	-	DDB0186514	-	-	-	-	-	-	-	-	-	-	-	-	Os12g28270.1	-	-	-	76949	-	-	-	-	-	SPAC1F8.04c	-	-	-	-	-	-	-	-	-	-
PSBP2		-	+	-	-	-	-	-	AT2G28605.1	-	-	-	-	191582	-	-	CMT368C	-	-	-	-	-	-	-	-	-	-	-	-	-	-	Os04g35530.1	30331	-	-	221389	-	-	217280	-	-	-	-	-	-	2597	-	-	-	-	-	-
		-	-	-	-	-	-	-	-	-	-	-	-	191581	-	-	-	-	-	-	-	-	-	-	-	-	-	-	-	-	-	-	-	-	-	-	-	-	-	-	-	-	-	-	-	-	-	-	-	107722.m00014	-	-
		-	-	-	-	-	-	-	-	-	-	-	-	191579	-	-	-	-	-	-	-	-	-	-	-	-	-	-	-	-	-	-	-	-	-	-	-	-	-	-	-	-	-	-	-	-	-	-	-	-	-	-
		-	-	-	-	-	-	-	-	-	-	-	-	191578	-	-	-	-	-	-	-	-	-	-	-	-	-	-	-	-	-	-	-	-	-	-	-	-	-	-	-	-	-	-	-	-	-	-	-	-	-	-
		-	-	-	-	-	-	-	-	-	-	-	-	191577	-	-	-	-	-	-	-	-	-	-	-	-	-	-	-	-	-	-	-	-	-	-	-	-	-	-	-	-	-	-	-	-	-	-	-	-	-	-
		-	-	-	-	-	-	-	-	-	-	CE17241	-	191569	-	-	-	-	-	-	-	-	-	-	-	-	-	-	-	-	NCU07762	-	-	-	-	-	-	-	-	-	-	-	-	-	-	-	-	-	-	-	-	-
		-	-	-	-	-	-	-	-	-	-	-	-	191568	-	-	-	-	-	-	-	-	-	-	-	-	-	-	-	-	-	-	-	-	-	-	-	-	-	-	-	-	-	-	-	-	-	-	-	-	-	-
		-	-	-	-	-	-	-	-	-	-	-	-	191567	-	-	-	-	-	-	-	-	-	-	-	-	-	-	-	-	-	-	-	-	-	-	-	-	-	-	-	-	-	-	-	-	-	-	-	-	-	-
		-	-	-	-	-	-	-	-	-	-	-	-	191561	-	-	-	-	-	-	-	-	-	-	-	-	-	-	-	-	-	-	-	-	-	-	-	-	-	-	-	-	-	-	-	-	-	-	-	-	-	-
		-	-	-	-	-	-	-	-	-	-	-	-	191560	-	-	-	-	-	-	-	-	-	-	-	-	-	-	-	-	-	-	-	-	-	-	-	-	-	-	-	-	-	-	-	-	-	-	-	-	-	-
		-	+	-	-	-	-	-	-	-	-	CE37375	223883	191559	273107	-	-	-	-	-	-	-	ENSGALP00000039409	-	-	-	53545	13806	-	80747	NCU00553	-	-	-	-	-	-	-	-	RO3G_12149.1	-	-	-	-	-	-	-	-	-	-	-	-
		-	-	-	-	-	-	-	-	-	-	-	-	191557	-	-	-	-	-	-	-	-	-	-	-	-	-	-	-	-	-	-	-	-	-	-	-	-	-	-	-	-	-	-	-	-	-	-	-	-	-	-
		-	-	-	-	-	-	-	-	-	-	-	-	191556	-	-	-	-	-	-	-	-	-	-	-	-	-	-	-	-	-	-	-	-	-	-	-	-	-	-	-	-	-	-	-	-	-	-	-	-	-	-
		-	-	-	-	-	-	-	-	-	-	-	-	191544	-	-	-	-	-	-	-	-	-	-	-	-	-	-	-	-	-	-	-	-	-	-	-	-	-	-	-	-	-	-	-	-	-	-	-	-	-	-
MRPS13	Putative mitochondrial ribosomal protein S13, imported to mitochondria	-	-	-	-	-	-	-	-	-	-	-	-	191536	-	-	-	-	-	-	-	-	-	-	-	-	-	-	-	-	-	-	-	-	-	-	-	-	-	-	-	-	-	-	-	-	-	-	-	-	-	-
		-	-	-	-	-	-	-	-	-	-	-	-	191526	-	-	-	-	-	-	-	-	-	-	-	-	-	-	-	-	-	-	-	-	-	-	-	-	-	-	-	-	-	-	-	-	-	-	-	-	-	-
		-	-	-	-	-	-	-	-	-	-	-	-	191524	-	-	-	-	-	-	-	-	-	-	-	-	-	-	-	-	-	-	-	-	-	-	-	-	-	-	-	-	-	-	-	-	-	-	-	-	-	-
		-	-	-	-	-	-	-	-	-	-	-	221980	191521	-	-	-	-	-	-	-	-	ENSGALP00000008688	-	NP_001013682.2	-	-	-	-	-	-	-	-	-	-	44924	-	-	-	-	-	-	-	-	-	-	-	-	-	-	-	-
GMP1		-	+	-	-	-	-	GB12420-PA	AT2G39770.1	-	BDEG_08010	CE40443	154984	191520	207656	cgd2_1770	CML145C	-	DDB0187554	CG1129-PA	19069594	-	ENSGALP00000003934	GL50803_16598	NP_068806.1	LmjF23.0110	227532	12046	74057	162365	NCU11213	Os08g13930.2	-	GSPATP00002821001	-	198340	109074	PF14_0774	656143	RO3G_04395.1	YDL055C	SPCC1906.01	GLEAN3_00187	SINFRUP00000175904	88.m00165	-	TA14300	55.m04726	33563	93905.m00187	Tb927.8.2050	-
		-	+	-	+	-	-	GB12107-PA	AT5G04430.1	-	-	CE26206	173082	191517	392088	cgd7_720	-	OTTDARP00000018754	-	CG8144-PD	-	-	ENSGALP00000015995	-	OTTHUMP00000115468	-	123770	-	-	190891	-	Os02g57640.1	4453	-	40453	-	-	PF14_0151	822598	RO3G_06190.1	YBL032W	SPCC757.09c	GLEAN3_03114	SINFRUP00000132475	-	36929	-	37.m00781	28388	-	-	-
MITC14	mitochondrial substrate carrier protein	-	+	-	-	-	-	GB20046-PA	AT5G19760.1	31501	-	CE32102	166615	191516	204690	cgd1_600	CMC159C	-	DDB0185907	CG18418-PA	-	-	-	-	OTTHUMP00000183073	-	209667	18424	73453	239058	NCU10732	Os05g11780.1	19853	GSPATP00006881001	23908	197217	108637	PF08_0031	817666	RO3G_14366.1	-	SPAC139.02c	-	SINFRUP00000139879	115.m00128	26366	TA13260	59.m00061	50388	-	Tb10.389.0690	UM04060.1
		-	+	-	-	-	-	GB14827-PA	-	58891	BDEG_01016	-	173958	191514	295176	-	-	OTTDARP00000023978	DDB0184087	CG14353-PA	-	-	ENSGALP00000014235	GL50803_3531	OTTHUMP00000159961	LmjF19.1550	220887	38989	60369	195059	-	-	8259	GSPATP00017591001	-	56261	109574	PF13_0250	-	RO3G_12152.1	-	-	-	SINFRUP00000169071	12.m00295	261002	-	52.m01597	30155	82354.m00020	Tb10.61.0160	-
		-	+	-	-	-	-	-	AT3G23900.2	-	-	-	-	191512	-	-	-	-	-	CG8419-PA	-	3.m00554	ENSGALP00000023879	-	NP_079464.1	-	-	-	80016	238127	-	Os01g01150.3	35190	-	-	65813	-	-	827541	-	-	-	-	SINFRUP00000137101	-	-	-	-	-	-	-	-
		-	+	-	-	-	-	-	-	-	BDEG_07895	CE02530	91899	191511	-	-	CMC062C	-	DDB0204139	-	-	-	ENSGALP00000004058	-	NP_001091990.1	-	217236	-	73943	244837	NCU03908	-	-	GSPATP00012062001	-	164856	135451	-	-	RO3G_13162.1	YER004W	-	-	SINFRUP00000145716	23.m00305	-	-	-	55521	-	Tb927.8.670	UM01006.1
		-	-	-	-	-	-	-	-	-	-	-	-	191510	-	-	-	-	-	-	-	-	-	-	-	-	-	-	-	-	-	-	-	-	-	166355	-	-	-	-	-	-	-	-	-	-	-	-	-	-	-	-
		-	-	-	-	-	-	-	-	-	-	-	-	191500	-	-	-	-	-	-	-	-	-	-	-	-	-	-	-	-	-	-	-	-	-	-	-	-	-	-	-	-	-	-	-	-	-	-	-	-	-	-
		-	-	-	-	-	-	-	-	-	-	-	-	191493	-	-	-	-	-	-	-	-	-	-	-	-	-	-	-	-	-	-	-	-	-	-	-	-	-	-	-	-	-	-	-	-	-	-	-	-	-	-
SMT1		-	+	+	-	-	-	-	-	18319	BDEG_05622	CE18056	-	191486	-	cgd8_4260	CME004C	OTTDARP00000020159	DDB0186869	-	19069130	19.m00315	ENSGALP00000013729	-	OTTHUMP00000163740	LmjF08.0470	227264	28202	-	-	NCU09813	Os01g68950.3	-	GSPATP00007001001	27817	150469	135326	-	-	RO3G_01348.1	-	SPBC365.06	-	SINFRUP00000141134	44.m00197	38679	TA16970	57.m01794	33331	-	Tb927.5.3210	UM05863.1
		-	-	-	-	-	-	-	-	-	-	-	226708	191482	-	-	-	-	-	-	-	-	-	-	-	-	-	-	-	237029	-	-	-	-	-	-	-	-	-	RO3G_00553.1	-	-	-	-	-	-	-	-	61965	-	-	-
		-	-	-	-	-	-	-	-	-	-	-	-	191480	-	-	-	-	-	-	-	-	-	-	-	-	-	-	-	-	-	-	-	-	-	-	-	-	-	-	-	-	-	-	-	-	-	-	-	-	-	-
		-	-	-	-	-	-	-	-	-	-	-	-	191478	-	-	-	-	-	-	-	-	-	-	-	-	-	-	-	-	-	-	-	-	-	-	-	-	-	-	-	-	-	-	-	-	-	-	-	-	-	-
		-	-	-	-	-	-	-	-	-	-	-	-	191477	-	-	-	-	-	-	-	-	-	-	-	-	-	-	-	-	-	-	-	-	-	-	-	-	-	-	-	-	-	-	-	-	-	-	-	-	-	-
		-	-	-	-	-	-	-	-	-	-	-	-	191474	-	-	-	-	-	-	-	-	-	-	-	-	-	-	-	-	-	-	-	-	-	-	-	-	-	-	-	-	-	-	-	-	-	-	-	-	-	-
		-	-	-	-	-	-	-	-	-	-	-	-	191473	-	-	-	-	-	-	-	-	-	-	-	-	-	-	-	-	-	-	-	-	-	-	-	-	-	-	-	-	-	-	-	-	-	-	-	-	-	-
		-	-	-	-	-	-	-	-	-	-	-	-	191472	-	-	-	-	-	-	-	-	-	-	-	-	-	-	48199	-	-	-	-	-	-	-	-	-	-	-	-	-	-	-	-	-	-	-	-	-	-	-
		-	-	-	-	-	-	-	-	-	-	-	-	191467	-	-	-	-	-	-	-	-	-	-	-	-	-	-	-	-	-	-	-	-	-	-	-	-	-	-	-	-	-	-	-	-	-	-	-	-	-	-
		-	-	-	-	-	-	-	-	-	-	-	-	191466	-	-	-	-	-	-	-	-	-	-	-	-	-	-	79446	-	-	-	-	-	-	-	-	-	-	-	-	-	-	-	-	-	-	-	-	-	-	-
		-	-	-	-	-	-	-	-	-	-	-	-	191465	-	-	-	-	-	-	-	-	-	-	-	-	-	-	-	-	-	-	-	-	-	-	-	-	-	-	-	-	-	-	-	-	-	-	-	-	-	-
		-	-	-	-	-	-	-	-	-	-	-	-	191464	-	-	-	-	-	-	-	-	-	-	-	-	-	-	-	-	-	Os03g61760.1	36425	-	-	-	-	-	-	-	-	-	-	-	-	-	-	-	-	-	-	-
		-	-	-	-	-	-	-	-	-	-	-	-	191461	-	-	-	-	-	-	-	-	-	-	-	-	-	-	-	-	-	-	-	-	-	-	-	-	-	-	-	-	-	-	-	-	-	-	-	-	-	-
		-	+	-	-	-	-	-	AT4G33625.1	-	-	-	-	191454	-	-	-	-	-	-	-	-	-	-	-	-	-	-	-	-	-	Os07g02710.1	-	-	33025	47846	-	-	258135	-	-	-	-	-	-	-	-	-	-	-	-	-
		-	+	-	-	-	-	GB16118-PA	-	4152	BDEG_06159	-	159772	191453	208335	cgd4_2270	CMO249C	OTTDARP00000021547	-	CG30491-PA	-	188.m00088	ENSGALP00000015508	-	OTTHUMP00000115875	LmjF24.1810	197176	28464	46547	87569	NCU09735	-	31338	GSPATP00000448001	-	140064	109382	-	-	RO3G_10615.1	-	-	GLEAN3_27965	SINFRUP00000148734	12.m00532	3903	TA10925	55.m04886	37121	89008.m00044	Tb927.8.6420	-
		-	-	-	-	-	-	-	-	-	-	-	-	191447	-	-	-	-	-	-	-	-	-	-	-	-	-	-	-	-	-	-	-	-	-	-	-	-	-	-	-	-	-	-	-	-	-	-	-	-	-	-
		-	-	-	-	-	-	-	-	-	-	-	-	191446	-	-	-	-	-	-	-	-	-	-	-	-	-	-	-	-	-	-	-	-	-	-	-	-	-	-	-	-	-	-	-	-	-	-	-	-	-	-
		-	-	-	-	-	-	-	-	-	-	-	-	191445	-	-	-	-	-	-	-	-	-	-	-	-	-	-	-	-	-	-	-	-	-	-	-	-	-	-	-	-	-	-	-	-	-	-	-	-	-	-
		-	-	-	-	-	-	-	-	-	-	-	-	191443	-	-	-	-	-	-	-	-	-	-	-	-	-	-	-	-	-	-	-	-	-	-	-	-	-	-	-	-	-	-	-	-	-	-	-	-	-	-
		-	-	-	-	-	-	-	-	-	-	-	-	191441	-	-	-	-	-	-	-	-	-	-	-	-	-	-	-	-	-	-	-	-	-	-	-	-	-	-	-	-	-	-	-	-	-	-	-	-	-	-
		-	-	-	-	-	-	-	-	-	-	-	-	191435	-	-	-	-	-	-	-	-	-	-	-	-	-	-	-	-	-	-	-	-	-	-	-	-	-	-	-	-	-	-	-	-	-	-	-	-	-	-
		-	-	-	-	-	-	-	-	-	-	-	-	191433	-	-	-	-	-	-	-	-	-	-	-	-	-	-	-	-	-	-	-	-	-	-	-	-	-	-	-	-	-	-	-	-	-	-	-	-	-	-
COB1	S-adenosyl-L-methionine-dependent uroporphyrinogen III methyltransferase	-	+	-	-	-	-	-	AT5G40850.1	39455	-	-	-	191432	-	-	CMV039C	-	-	-	-	-	-	-	-	-	-	8192	-	156762	NCU00602	Os01g44050.1	4145	-	12925	15921	109158	-	271745	RO3G_09405.1	YKR069W	SPCC1739.06c	-	-	-	17623	-	-	-	-	-	UM03997.1
		-	-	-	-	-	-	-	-	-	-	-	-	191429	-	-	-	-	-	-	-	-	-	-	-	-	-	-	-	-	-	-	-	-	-	-	-	-	-	-	-	-	-	-	-	-	-	-	-	-	-	-
		-	-	-	-	-	-	-	-	-	-	-	-	191428	-	-	-	-	-	-	-	-	-	-	-	-	-	-	-	-	-	-	-	-	-	-	-	-	-	-	-	-	-	-	-	-	-	-	-	-	-	-
GSHR2	glutathione reductase	-	-	-	-	-	-	-	-	-	-	-	-	191427	-	-	-	-	-	-	-	-	-	-	-	-	-	-	-	-	-	-	-	-	-	-	-	-	-	-	-	-	-	-	-	-	-	-	-	-	-	-
		-	+	-	-	-	-	-	-	-	-	-	24332	191420	-	cgd4_4110	-	-	DDB0167928	-	19069197	-	-	-	-	-	-	-	-	-	NCU07558	-	-	-	-	-	-	MAL8P1.63	-	RO3G_02151.1	YDL001W	SPCC16C4.01	-	-	-	-	-	59.m03394	-	-	-	-
		-	-	-	-	-	-	-	-	-	-	-	-	191416	-	-	-	-	-	-	-	-	-	-	-	-	-	-	-	-	-	-	-	-	-	-	-	-	-	-	-	-	-	-	-	-	-	-	-	-	-	-
		-	+	-	-	-	-	-	AT4G23496.1	-	-	-	-	191409	-	-	-	-	-	-	-	-	-	-	-	-	-	-	-	246076	-	Os03g30430.2	23835	-	-	230662	141050	-	577765	-	-	-	-	-	-	-	-	-	-	-	-	-
		-	-	-	-	-	-	-	-	-	-	-	-	191408	-	-	-	-	-	-	-	-	-	-	-	-	-	-	-	-	-	-	-	-	-	-	-	-	-	-	-	-	-	-	-	-	TA10735	-	-	-	-	-
		-	-	-	-	-	-	-	-	-	-	-	-	191406	-	-	-	-	-	-	-	-	-	-	-	-	-	-	-	-	-	-	-	-	-	-	-	-	-	-	-	-	-	-	-	-	-	-	-	-	-	-
KDG4	diacylglycerol kinase	-	+	-	-	-	-	GB19002-PA	AT5G51290.1	-	-	CE18241	146129	191404	272303	-	-	OTTDARP00000008399	-	CG16708-PB	-	-	ENSGALP00000030103	-	OTTHUMP00000028554	-	168829	11991	-	84607	-	Os02g43906.1	-	-	-	74586	-	-	827488	-	-	-	GLEAN3_18769	SINFRUP00000158801	-	-	-	-	-	-	-	-
		-	-	-	-	-	-	-	-	-	-	-	-	191402	-	-	-	-	-	-	-	-	-	-	-	-	-	-	-	-	-	-	-	-	-	-	-	-	-	-	-	-	-	-	-	-	-	-	-	-	-	-
	Clathrin Assembly factor AP180	-	+	-	-	-	-	GB14848-PA	AT5G57200.1	-	BDEG_03429	CE27813	104543	191396	285148	-	CMB081C	OTTDARP00000023304	DDB0218102	CG2520-PA	-	-	ENSGALP00000025502	-	NP_055656.1	LmjF24.0020	107445	-	56097	129323	NCU02586	Os01g50010.1	24375	-	-	21255	-	-	564639	RO3G_08268.1	YGR241C	SPBC19F8.03c	-	SINFRUP00000153417	-	-	-	-	25611	-	Tb11.02.2410	UM05169.1
		-	-	-	-	-	-	-	-	-	-	-	-	191395	-	-	-	-	-	-	-	-	-	-	-	-	-	-	-	-	-	-	-	-	-	-	-	-	-	-	-	-	-	-	-	-	-	-	-	-	-	-
		-	-	-	-	-	-	-	-	-	-	-	-	191390	-	-	-	-	-	-	-	-	-	-	-	-	-	-	-	-	-	-	-	-	-	-	-	-	-	-	-	-	-	-	-	-	-	-	-	-	-	-
		-	-	-	-	-	-	-	-	-	-	-	-	191389	-	-	-	-	-	-	-	-	-	-	-	-	-	-	-	-	-	-	-	-	-	-	-	-	-	-	-	-	-	-	-	-	-	-	-	-	-	-
	SET domain containing protein, putative histone methyltransferase	-	+	-	-	-	-	GB10605-PA	AT1G77300.2	27977	BDEG_05644	CE37884	160470	191388	260608	cgd5_400	CMN313C	OTTDARP00000008771	DDB0189799	CG1716-PA	19171422	-	ENSGALP00000008825	GL50803_9130	OTTHUMP00000170947	-	227153	26994	3997	22303	NCU00269	Os02g34850.1	22195	GSPATP00003275001	16875	33525	155902	MAL13P1.122	204626	RO3G_14220.1	YJL168C	SPAC29B12.02c	GLEAN3_27218	SINFRUP00000145198	70.m00138	35510	TA09850	55.m04720	54228	-	-	UM02500.1
		-	-	-	-	-	-	-	-	-	-	-	-	191380	-	-	-	-	-	-	-	-	-	-	-	-	-	-	-	-	NCU05544	-	-	-	-	-	-	-	-	-	-	-	-	-	-	-	-	-	-	-	-	-
		-	-	-	-	-	-	-	-	-	-	-	-	191379	-	-	-	-	-	-	-	-	-	-	-	-	-	-	-	-	-	-	-	-	-	-	-	-	-	-	-	-	-	-	-	-	-	-	-	-	-	-
		-	-	-	-	-	-	-	-	-	-	-	-	191373	-	-	-	-	-	-	-	-	-	-	-	-	-	-	-	-	-	-	-	-	-	-	-	-	-	-	-	-	-	-	-	10722	-	-	-	-	-	-
		-	-	-	-	-	-	-	-	-	-	-	-	191371	-	-	-	-	-	-	-	-	-	-	-	-	-	-	-	-	-	-	-	-	-	-	-	-	-	-	-	-	-	-	-	-	-	-	-	-	-	-
		-	-	-	-	-	-	-	AT5G55500.1	65709	-	-	-	191368	-	-	-	-	-	-	-	-	-	-	-	-	-	-	-	-	-	-	-	-	-	-	133979	-	550155	-	-	-	-	-	-	-	-	-	-	-	-	-
	unknown function	-	-	-	-	-	-	-	-	-	-	-	-	191367	-	-	-	-	-	-	-	-	-	-	-	-	-	-	-	-	-	-	-	-	-	-	-	-	-	-	-	-	-	-	-	-	-	-	-	-	-	-
		-	-	-	-	-	-	-	-	-	-	-	-	191365	-	-	-	-	-	-	-	-	-	-	-	-	-	-	-	-	-	-	30367	-	-	-	-	-	-	-	-	-	-	-	-	-	-	-	-	-	-	-
FAP140	Flagellar Associated Protein	-	-	-	-	-	-	-	-	-	-	-	-	-	-	-	-	-	-	-	-	-	-	-	-	-	-	-	-	-	-	-	-	-	-	-	-	-	-	-	-	-	-	-	-	-	-	-	-	-	-	-
		-	-	-	-	-	-	-	-	-	-	-	-	191355	-	-	-	-	-	-	-	-	-	-	-	-	-	-	-	-	-	-	-	-	-	-	-	-	-	-	-	-	-	-	-	-	-	-	-	-	-	-
		-	+	-	-	-	-	GB14658-PA	AT1G33230.1	-	BDEG_00015	CE29983	179894	191353	254401	-	CMH277C	OTTDARP00000018671	DDB0188060	CG32795-PB	-	-	ENSGALP00000006756	-	OTTHUMP00000169548	-	239543	3096	71364	189126	-	Os02g01720.1	17365	-	-	131600	-	-	232802	-	-	-	GLEAN3_10671	SINFRUP00000165576	-	-	-	-	59815	-	-	-
		-	-	-	-	-	-	-	-	24123	-	-	-	191352	-	-	-	-	-	-	-	-	-	-	-	-	-	-	-	-	-	-	-	-	-	-	-	-	-	-	-	-	-	-	-	41690	-	-	-	-	-	-
		-	-	-	-	-	-	-	-	-	-	-	-	191351	-	-	-	-	-	-	-	-	-	-	-	-	-	-	-	-	-	-	-	-	-	-	-	-	-	-	-	-	-	-	-	-	-	-	-	-	-	-
		-	-	-	-	-	-	-	-	-	-	-	-	191346	-	-	-	-	-	-	-	-	-	-	-	-	-	-	-	-	-	-	-	-	-	-	-	-	-	-	-	-	-	-	-	-	-	-	-	-	-	-
		-	-	-	-	-	-	-	-	-	-	-	-	191344	-	-	-	-	-	-	-	-	-	-	-	-	-	-	-	-	-	-	-	-	-	-	-	-	-	-	-	-	-	-	-	-	-	-	-	-	-	-
		-	-	-	-	-	-	-	-	-	-	-	-	191343	-	-	-	-	-	-	-	-	-	-	-	-	-	-	-	-	-	-	-	-	-	-	-	-	-	-	-	-	-	-	-	-	-	-	-	-	-	-
PSBP5		-	+	-	-	-	-	-	AT3G05410.1	-	-	-	-	191340	-	-	CMN290C	-	-	-	-	-	-	-	-	-	-	-	-	-	-	Os01g70820.1	33605	-	49743	-	-	-	268592	-	-	-	-	-	-	-	-	-	-	-	-	-
		-	+	-	-	-	-	-	AT5G03555.1	-	-	-	-	191336	-	-	-	-	-	-	-	-	-	-	-	-	-	-	-	-	NCU07334	Os02g44680.1	28452	-	-	120934	-	-	589526	-	YIR028W	SPAC29B12.14c	-	-	-	-	-	-	-	-	-	UM03663.1
FAP114	Coiled-Coil Flagellar Associated Protein	-	-	-	+	-	-	-	-	-	-	-	-	191334	-	-	-	-	-	-	-	-	-	-	-	-	-	-	-	-	-	-	-	-	-	-	-	-	-	-	-	-	-	-	-	-	-	-	-	-	-	-
		-	-	-	-	-	-	-	-	-	-	-	-	191331	-	-	-	-	-	-	-	-	-	-	-	-	-	-	-	-	-	-	-	-	-	-	-	-	-	-	-	-	-	-	-	-	-	-	-	-	-	-
		-	-	-	-	-	-	-	-	-	-	-	-	191329	-	-	-	-	-	-	-	-	-	-	-	-	-	-	-	-	-	-	-	-	-	-	-	-	-	-	-	-	-	-	-	-	-	-	-	-	-	-
		-	-	-	-	-	-	-	-	-	-	-	-	191328	-	-	-	-	-	-	-	-	-	-	-	-	-	-	-	-	-	-	-	-	-	-	-	-	-	-	-	-	-	-	-	-	-	-	-	-	-	-
		-	-	-	-	-	-	-	-	-	-	-	-	191321	263185	-	-	-	-	-	-	-	-	-	-	-	-	-	-	-	-	-	-	-	-	-	-	-	-	-	-	-	-	-	-	-	-	-	-	-	-	-
		-	-	-	-	-	-	-	-	-	-	-	-	191320	-	-	-	-	-	-	-	-	-	-	-	-	-	-	-	-	-	-	-	-	-	-	-	-	-	-	-	-	-	-	-	-	-	-	-	-	-	-
GDIC1	Rab GDP dissociation inhibitor protein	-	+	+	-	-	-	GB13730-PA	AT5G09550.1	71109	BDEG_02932	CE14944	148575	191319	284296	cgd4_4080	CML223C	OTTDARP00000010502	DDB0189731	CG4422-PA	19068702	34.m00228	ENSGALP00000032695	GL50803_11495	OTTHUMP00000019010	LmjF29.2160	218952	17747	78215	194646	NCU05288	Os05g34540.1	14424	GSPATP00007065001	14827	218986	109350	PFL2060c	828557	RO3G_03976.1	YER136W	SPAC22H10.12c	GLEAN3_04785	SINFRUP00000159199	15.m00369	26136	TA17890	20.m00348	35792	84429.m00017	Tb927.3.4680	UM00756.1
		-	-	-	-	-	-	-	-	-	-	-	-	191315	-	-	-	-	-	-	-	-	-	-	-	-	-	-	-	-	-	-	-	-	-	-	-	-	-	-	-	-	-	-	-	-	-	-	-	-	-	-
		-	-	-	-	-	-	-	-	-	-	-	-	191310	-	-	-	-	-	-	-	-	-	-	-	-	-	-	-	-	-	-	-	-	-	-	-	-	-	-	-	-	-	-	-	-	-	-	-	-	-	-
FAP139	Flagellar Associated Protein	-	-	-	+	-	-	-	-	-	-	-	-	191309	-	-	-	-	-	-	-	-	-	-	-	-	-	-	-	-	-	-	-	-	-	-	-	-	-	-	-	-	-	-	-	-	-	-	-	-	-	-
		-	+	-	-	-	-	GB14864-PA	AT1G35470.2	-	BDEG_01438	CE20318	223242	191305	387434	-	CMP137C	-	DDB0184565	CG11763-PD	-	502.m00035	ENSGALP00000002189	-	OTTHUMP00000107905	-	122405	31450	-	219682	NCU01409	Os02g35070.1	-	GSPATP00013497001	45060	51208	143033	PF10_0140	287863	RO3G_11430.1	YGL227W	SPCC1259.12c	-	SINFRUP00000179373	18.m00362	-	TA07005	83.m01193	24556	-	-	UM05579.1
		-	-	-	-	-	-	-	-	-	-	-	-	191300	-	-	-	-	-	-	-	-	-	-	-	-	-	-	-	-	-	-	-	-	-	-	-	-	-	-	-	-	-	-	-	-	-	-	-	-	-	-
		-	-	-	-	-	-	-	AT4G16444.1	-	-	-	-	191298	-	-	-	-	-	-	-	-	-	-	-	-	-	-	-	-	-	Os06g09840.1	-	-	-	73478	-	-	650987	-	-	-	-	-	-	-	-	-	-	-	-	-
		-	-	-	-	-	-	-	-	-	-	-	-	191297	-	-	-	-	-	-	-	-	-	-	-	-	-	-	-	-	-	-	-	-	-	-	-	-	-	-	-	-	-	-	-	-	-	-	-	-	-	-
		-	+	-	-	-	-	-	-	8566	-	-	-	191293	-	-	-	-	-	-	-	-	-	-	-	-	-	26103	-	-	-	-	-	-	48196	-	136641	-	-	-	-	-	-	-	-	7558	-	-	-	-	-	-
		-	-	-	-	-	-	-	-	-	-	-	-	191283	-	-	-	-	-	-	-	-	-	-	-	-	-	-	-	-	-	-	-	-	-	-	-	-	-	-	-	-	-	-	-	-	-	-	-	-	-	-
		-	-	-	-	-	-	-	-	-	-	-	-	191281	-	-	-	-	-	-	-	-	-	-	-	-	-	-	-	-	-	-	-	-	-	-	-	-	-	-	-	-	-	-	-	-	-	-	-	-	-	-
		-	-	-	-	-	-	-	-	-	-	-	-	191277	-	-	-	-	-	-	-	-	-	-	-	-	-	-	-	-	-	-	31368	-	-	172609	-	-	-	-	-	-	-	-	-	-	-	-	-	-	-	-
		-	-	-	-	-	-	-	-	-	-	-	-	191276	-	-	-	-	-	-	-	-	-	-	-	-	-	-	-	-	-	-	-	-	-	-	-	-	-	-	-	-	-	-	-	-	-	-	-	-	-	-
		-	-	-	-	-	-	-	-	-	-	-	-	191270	-	-	-	-	-	-	-	-	-	-	-	-	-	-	-	-	-	-	-	-	-	-	-	-	-	-	-	-	-	-	-	-	-	-	-	-	-	-
NIFS1,NFS1	cysteine desulfurase, mitochondrial precursor	-	+	+	-	-	-	GB10777-PA	AT5G65720.1	18852	BDEG_06982	CE30405	155858	191269	203787	cgd4_3040	CMT234C	OTTDARP00000008454	DDB0205700	CG12264-PA	19069702	33.m00229	ENSGALP00000002382	GL50803_14519	OTTHUMP00000030799	LmjF27.1060	211436	38863	44858	174585	NCU04636	Os09g16910.3	27904	GSPATP00031355001	34119	88116	108984	MAL7P1.150	718085	RO3G_11728.1	YCL017C	SPBC21D10.11c	GLEAN3_24173	SINFRUP00000142693	6.m00432	240	TA19715	27.m00002	18881	88278.m00120	Tb11.55.0013	UM05776.1
		-	-	-	-	-	-	-	-	-	-	-	-	191268	-	-	-	-	-	-	-	-	-	-	-	-	-	-	-	-	-	-	-	-	-	-	-	-	-	-	-	-	-	-	-	-	-	-	-	-	-	-
		-	-	-	-	-	-	-	-	-	-	-	-	191267	-	-	-	-	-	-	-	-	-	-	-	-	-	-	-	-	-	-	-	-	-	-	-	-	-	-	-	-	-	-	-	-	-	-	-	-	-	-
		-	-	-	-	-	-	-	-	-	-	-	-	191263	-	-	-	-	-	-	-	-	-	-	-	-	-	-	-	-	-	-	-	-	-	-	-	-	-	-	-	-	-	-	-	-	-	-	-	-	-	-
		-	+	-	-	-	-	-	AT3G30390.2	19545	-	-	-	191256	-	-	-	-	-	-	-	-	-	-	-	-	-	-	-	-	NCU08066	Os06g16420.2	-	-	40731	197612	156480	-	412817	RO3G_02479.1	-	-	-	SINFRUP00000139062	-	-	-	-	-	-	-	-
		-	+	-	-	-	-	-	-	-	BDEG_00504	-	209554	191254	-	-	-	OTTDARP00000022659	-	CG3500-PA	-	-	-	-	OTTHUMP00000160121	-	119829	-	-	-	NCU00778	-	6209	-	-	36338	-	-	-	-	YHR181W	-	-	SINFRUP00000137680	-	-	-	-	-	-	-	-
		-	-	-	-	-	-	-	-	-	-	-	-	191253	-	-	-	-	-	-	-	-	-	-	-	-	-	-	-	-	-	-	-	-	-	-	-	-	-	-	-	-	-	-	-	-	-	-	-	-	-	-
		-	-	-	-	-	-	-	-	-	-	-	-	191249	-	-	-	-	-	-	-	-	-	-	-	-	-	-	-	-	-	-	-	-	-	-	-	-	-	-	-	-	-	-	-	-	-	-	-	-	-	-
		-	-	-	-	-	-	-	-	-	-	-	-	191248	-	-	-	-	-	-	-	-	-	-	-	-	-	-	-	-	-	-	10065	-	-	-	-	-	-	-	-	-	-	-	-	-	-	-	-	-	-	-
FAP1	Flagellar Associated Protein	-	-	-	+	-	-	-	-	-	-	-	-	191238	-	-	-	-	-	-	-	-	-	-	-	-	-	-	-	-	-	-	-	-	-	-	-	-	-	-	-	-	-	-	-	-	-	-	-	-	-	-
		-	-	-	-	-	-	-	-	-	-	-	-	191233	-	-	-	-	-	-	-	-	-	-	-	-	-	-	-	-	-	-	-	-	-	-	-	-	-	-	-	-	-	-	-	-	-	-	-	-	-	-
ELG12	exostosin-like glycosyltransferase	-	-	-	-	-	-	-	-	-	-	-	-	191232	-	-	-	-	-	-	-	-	-	-	-	-	-	-	-	-	-	-	9991	-	-	-	-	-	-	-	-	-	-	-	-	-	-	-	-	26340	-	-	-

		-	+	+	-	-	-	GB19798-PA	AT1G50510.1	23105	BDEG_07014	CE31247	196739	191230	279747	-	CMH116C	-	DDB0189786	CG2794-PA	-	270.m00051	ENSGALP00000020933	GL50803_15077	-	-	221393	27110	32917	192278	NCU03020	Os08g39420.2	35315	GSPATP00036280001	9211	108917	108728	-	172207	RO3G_13013.1	-	SPBC1861.05	-	SINFRUP00000139473	336.m00016	31193	-	-	19938	-	-	UM00024.1
		+	+	-	-	-	-	GB15531-PA	-	-	-	-	-	191228	-	-	-	-	-	CG11579-PE	-	-	-	-	-	LmjF32.0140	205205	37775	69247	-	-	-	-	-	-	73690	-	-	-	-	-	-	GLEAN3_09155	SINFRUP00000136043	-	-	-	-	-	97225.m00175	Tb10.61.2450	-
		-	-	-	-	-	-	-	-	-	-	-	-	191227	-	-	-	-	-	-	-	-	-	-	-	-	-	-	-	-	-	-	-	-	-	-	-	-	-	-	-	-	-	-	-	-	-	-	-	-	-	-
"Eli4,ELI4	Early light-inducible protein;	-	-	-	-	-	-	-	-	-	-	-	-	191226	-	-	-	-	-	-	-	-	-	-	-	-	-	-	-	-	-	-	-	-	-	-	-	-	-	-	-	-	-	-	-	-	-	-	-	-	-	-
		-	-	-	-	-	-	-	-	-	-	-	-	191217	-	-	-	-	-	-	-	-	-	-	-	-	-	-	-	-	-	-	-	-	-	-	-	-	-	-	-	-	-	-	-	-	-	-	-	-	-	-
SMP11	Sm protein-like	-	+	-	-	-	-	GB14039-PA	AT1G65700.2	23509	BDEG_03115	CE29146	206003	191216	241044	cgd7_5350	-	-	-	CG2021-PA	-	-	ENSGALP00000014820	-	OTTHUMP00000024532	-	186799	17908	-	236381	-	Os05g51650.1	28646	GSPATP00031295001	10752	167674	-	MAL8P1.9	641478	-	-	SPCC1840.10	-	SINFRUP00000137029	15.m00423	7311	TA04440	59.m06097	23559	89183.m00127	Tb927.3.1780	-
		-	+	-	-	-	-	GB16801-PA	AT1G27530.1	-	-	CE00114	181742	191210	288494	cgd8_3630	-	-	DDB0188229	CG8386-PA	-	-	-	-	OTTHUMP00000031885	LmjF15.1250	213091	21173	33148	214407	-	Os10g13800.2	-	GSPATP00012387001	-	195569	-	-	830255	-	-	-	-	SINFRUP00000138466	238.m00031	-	TA20810	31.m00087	35934	-	Tb09.160.4150	-
		-	+	+	-	-	-	GB18250-PA	AT1G16540.1	-	BDEG_02634	CE30560	166504	191208	291211	cgd2_30	-	-	DDB0217046	CG1692-PA	-	46.m00208	ENSGALP00000021432	GL50803_14200	OTTHUMP00000163413	-	235545	-	-	82242	NCU07805	Os06g45860.1	-	-	46641	118134	134525	-	719124	-	-	-	GLEAN3_01079	SINFRUP00000174940	-	-	-	145.m00351	20006	89789.m00070	-	UM03628.1
		-	+	-	-	-	-	-	-	39533	-	-	169384	191206	-	-	-	-	-	-	-	-	-	-	-	-	141723	-	-	188039	NCU04676	-	-	-	32813	-	-	-	-	-	-	-	-	-	-	-	-	-	-	-	-	UM00417.1
		-	-	-	-	-	-	-	-	-	-	-	-	191203	-	-	-	-	-	-	-	-	-	-	-	-	-	-	-	-	-	-	-	-	-	-	-	-	-	-	-	-	-	-	-	-	-	-	-	-	-	-
AGO3401	Argonaute protein	-	-	-	-	-	-	-	-	-	-	-	-	191193	-	-	-	-	-	-	-	-	-	-	-	-	-	-	-	-	-	-	-	-	-	-	-	-	-	-	-	-	-	-	-	-	-	-	-	-	-	-
		-	-	-	-	-	-	-	-	-	-	-	-	191191	-	cgd6_3750	-	-	-	-	-	-	-	-	-	-	-	-	-	-	-	-	-	-	-	-	-	-	-	-	-	SPAC27E2.01	-	-	-	-	-	50.m00029	-	-	-	-
		-	-	-	-	-	-	-	-	-	-	-	-	191186	-	-	-	-	-	-	-	-	-	-	-	-	-	-	-	-	-	-	-	-	-	-	-	-	-	-	-	-	-	-	-	-	-	-	-	-	-	-
		-	-	-	-	-	-	-	-	-	-	-	-	191184	-	-	-	-	-	-	-	-	-	-	-	-	-	-	-	-	-	-	-	-	-	-	-	-	-	-	-	-	-	-	-	-	-	-	-	-	-	-
FAP86	Flagellar Associated Protein	-	-	-	+	-	-	-	-	-	-	-	-	191183	-	-	-	-	-	-	-	-	-	-	-	-	-	-	-	-	-	-	-	-	-	-	-	-	-	-	-	-	-	-	-	-	-	-	-	-	-	-
		-	-	-	-	-	-	-	-	-	-	-	-	191179	-	-	-	-	-	-	-	-	-	-	-	-	-	-	-	-	-	-	-	-	-	170947	-	-	-	-	-	-	-	-	-	-	-	-	-	-	-	-
		-	-	-	-	-	-	-	-	-	-	-	-	191177	-	-	-	-	-	-	-	-	-	-	-	-	-	-	-	-	-	-	-	-	-	-	-	-	-	-	-	-	-	-	-	-	-	-	-	-	-	-
SGA3401,ASF1	Anti-silencing factor	-	+	+	-	-	-	GB10442-PA	AT1G66740.1	30361	BDEG_00122	CE39722	149983	191168	266270	cgd4_760	CMG143C	OTTDARP00000020046	DDB0186540	CG9383-PA	19069077	123.m00111	ENSGALP00000023972	GL50803_33433	NP_054753.1	LmjF20.0050	205091	33278	68398	178884	NCU09436	Os05g48030.2	28166	GSPATP00019708001	52450	200559	155659	PFL1180w	710363	RO3G_07784.1	YJL115W	SPCC663.05c	-	SINFRUP00000135588	50.m00199	35372	TA15120	25.m01849	29129	84270.m00659	Tb927.1.630	UM02415.1
MRCE1	mRNA Capping enzyme with dual 5' triphosphatase and guanylyltransferase activities	-	+	-	-	-	-	GB17572-PA	AT5G28210.1	-	-	CE05197	167250	191164	-	-	-	-	-	CG1810-PA	-	-	-	-	-	-	-	-	-	-	-	Os11g11070.2	15689	-	-	115854	-	-	735714	-	-	-	GLEAN3_08892	-	-	-	-	-	-	-	-	-
		-	-	-	-	-	-	-	-	-	-	-	-	191162	-	-	-	-	-	-	-	-	-	-	-	-	-	-	-	-	-	-	-	-	-	-	-	-	-	-	-	-	-	-	-	-	-	-	-	-	-	-
		-	-	-	-	-	-	-	-	-	-	-	-	191158	-	-	-	-	-	-	-	-	-	-	-	-	-	-	-	-	-	-	-	-	-	-	-	-	-	-	-	-	-	-	-	-	-	-	-	-	-	-
		-	-	-	-	-	-	-	-	-	-	-	-	191150	-	-	-	-	-	-	-	-	-	-	-	-	-	-	-	-	-	-	-	-	-	-	-	-	-	-	-	-	-	-	-	-	-	-	-	-	-	-
		-	-	-	-	-	-	-	-	-	-	-	-	191148	-	-	-	-	-	-	-	-	-	-	-	-	-	-	-	-	-	-	-	-	-	-	-	-	-	-	-	-	-	-	-	-	-	-	-	-	-	-
NUOP4	NADH:ubiquinone oxidoreductase (Complex I) 9 kDA subunit, mitochondrial	-	-	-	-	-	-	-	-	-	-	-	-	191146	-	-	-	-	-	-	-	-	-	-	-	-	-	-	-	-	-	-	-	-	-	-	-	-	-	-	-	-	-	-	-	-	-	-	-	-	-	-
		-	-	-	-	-	-	-	-	-	-	-	-	191144	-	-	-	-	-	-	-	-	-	-	-	-	-	-	-	-	-	-	-	-	-	-	-	-	-	-	-	-	-	-	-	-	-	-	-	-	-	-
TPT1	phosphate/phosphoenolpyruvate translocator, putative	-	+	-	-	-	-	-	AT5G33320.1	23976	-	-	147079	191135	-	-	CMN328C	-	-	-	-	-	-	-	OTTHUMP00000067630	-	102951	26899	-	162657	-	Os08g25624.1	-	-	54017	51520	-	-	252124	-	-	-	-	-	-	16075	-	-	-	-	-	-
		-	-	-	-	-	-	-	-	-	-	-	-	191132	-	-	-	-	-	-	-	-	-	-	-	-	-	-	-	-	-	-	-	-	-	-	-	-	-	-	-	-	-	-	-	-	-	-	-	-	-	-
		-	-	-	-	-	-	-	-	-	-	-	-	191125	-	-	-	-	-	-	-	-	-	-	-	-	-	-	-	-	-	-	32130	-	-	-	-	-	-	-	-	-	-	-	-	-	-	-	-	-	-	-
		-	-	-	-	-	-	-	-	-	-	-	-	191121	-	-	-	OTTDARP00000022239	-	-	-	-	ENSGALP00000010636	-	OTTHUMP00000018937	-	-	-	-	-	-	-	-	-	-	-	-	-	-	-	-	-	-	SINFRUP00000162539	-	-	-	-	-	-	-	-
		-	-	-	-	-	-	-	-	-	-	-	-	191119	-	-	-	-	-	-	-	-	-	-	-	-	-	-	-	-	-	-	-	-	-	-	143367	-	-	-	-	-	-	-	-	-	-	-	-	-	-	-
		-	-	-	-	-	-	-	-	-	-	-	-	191113	-	-	-	-	-	-	-	-	-	-	-	-	-	-	-	-	-	-	-	-	-	-	-	-	-	-	-	-	-	-	-	-	-	-	-	-	-	-
		-	-	-	-	-	-	-	-	-	-	-	-	191111	-	-	-	-	-	-	-	-	-	-	-	-	-	-	-	-	-	-	-	-	-	-	-	-	-	-	-	-	-	-	-	-	-	-	-	-	-	-
		-	-	-	-	-	-	-	-	-	-	-	-	191109	-	-	-	-	-	-	-	-	-	-	-	-	-	-	-	-	-	-	-	-	-	-	-	-	-	-	-	-	-	-	-	-	-	-	-	-	-	-
		-	-	-	-	-	-	-	-	-	-	-	-	191108	-	-	-	-	-	-	-	-	-	-	-	-	-	-	-	-	-	-	-	-	-	-	-	-	-	-	-	-	-	-	-	-	-	-	-	-	-	-
		-	-	-	-	-	-	-	-	-	-	-	-	191105	-	-	-	-	-	-	-	-	-	-	-	-	-	-	-	-	-	-	-	-	-	-	-	-	-	-	-	-	-	-	-	-	-	-	-	-	-	-
		-	-	-	-	-	-	-	-	-	-	-	-	191104	-	-	-	-	-	-	-	-	-	-	-	-	-	-	-	-	-	-	-	-	-	-	-	-	-	-	-	-	-	-	-	-	-	-	-	-	-	-
		-	-	-	-	-	-	-	-	-	-	-	-	191103	-	-	-	-	-	-	-	-	-	-	-	-	-	-	-	-	-	-	-	-	-	-	-	-	-	-	-	-	-	-	-	-	-	-	-	-	-	-
	putative plastidic phosphate/phosphoenolpyruvate translocator protein	-	+	-	-	-	-	GB19954-PA	-	-	BDEG_02705	CE37067	-	191102	-	-	CMK248C	OTTDARP00000017471	-	CG14971-PA	-	-	-	-	-	-	-	-	-	-	-	Os05g41480.2	4369	-	-	-	-	-	-	-	-	-	-	-	-	-	-	-	52532	-	-	-
		-	-	-	-	-	-	-	-	-	-	-	-	191101	-	-	-	-	-	-	-	-	-	-	-	-	-	-	-	-	-	-	-	-	-	-	-	-	-	-	-	-	-	-	-	-	-	-	-	-	-	-
CYP771A1	cytochrome P450, unkown function	-	-	-	-	-	-	-	-	-	-	-	-	191092	-	-	-	-	-	-	-	-	-	-	-	-	-	-	-	-	-	-	-	-	-	-	-	-	-	-	-	-	-	-	-	-	-	-	-	-	-	-
		-	-	-	-	-	-	-	-	-	-	-	-	191091	-	-	-	-	-	-	-	-	-	-	-	-	-	-	-	-	-	-	-	-	-	-	-	-	-	-	-	-	-	-	-	-	-	-	-	-	-	-
		-	-	-	-	-	-	-	-	-	-	-	-	191083	-	-	-	-	-	-	-	-	-	-	-	-	-	-	-	-	-	Os11g30180.1	-	-	-	-	-	-	-	-	-	-	-	-	13.m00483	-	-	-	-	-	-	-
		-	-	-	-	-	-	-	-	-	-	-	-	191079	-	-	-	-	-	-	-	-	-	-	OTTHUMP00000161840	-	-	-	-	-	-	-	-	-	-	-	-	-	-	-	-	-	-	-	-	-	-	-	-	-	-	-
		-	-	-	-	-	-	-	-	-	-	-	-	191074	-	-	-	-	-	-	-	-	-	-	-	-	-	-	-	-	-	-	-	-	-	-	-	-	-	-	-	-	-	-	-	-	-	-	-	-	-	-
		-	-	-	-	-	-	-	-	-	-	-	-	191073	-	-	-	-	-	-	-	-	-	-	-	-	-	-	-	-	-	-	-	-	-	-	-	-	-	-	-	-	-	-	-	-	-	-	-	-	-	-
		-	-	-	-	-	-	-	-	-	-	-	-	191072	-	-	-	-	-	-	-	-	-	-	-	-	-	-	-	-	-	-	-	-	-	-	-	-	-	-	-	-	-	-	-	-	-	-	-	-	-	-
		-	-	-	-	-	-	-	-	-	-	-	-	191070	-	-	-	-	-	-	-	-	-	-	-	-	-	-	-	-	-	-	-	-	-	-	-	-	-	-	-	-	-	-	-	-	-	-	-	-	-	-
		-	+	-	-	-	-	GB11435-PA	AT2G35320.1	-	-	-	218995	191069	261422	-	-	-	-	CG9554-PA	-	-	ENSGALP00000025135	-	NP_742108.1	-	124788	-	-	116873	-	Os06g02028.1	-	-	-	119866	-	-	796431	-	-	-	GLEAN3_13869	SINFRUP00000145923	-	-	-	-	-	-	-	-
		-	-	-	-	-	-	-	-	-	-	-	-	191068	-	-	-	-	-	-	-	-	-	-	-	-	-	-	-	-	-	-	-	-	-	-	-	-	-	-	-	-	-	-	-	-	-	-	-	-	-	-
		-	-	-	-	-	-	-	-	-	-	-	-	191061	-	-	-	-	-	-	-	-	-	-	-	-	-	15143	-	-	-	-	-	-	-	-	-	-	-	-	-	-	-	-	-	-	-	-	-	92553.m00049	-	-
		-	-	-	-	-	-	-	-	-	-	-	-	191060	-	-	-	-	-	-	-	-	-	-	-	-	-	-	-	-	-	-	-	-	-	131517	-	-	-	-	-	-	-	-	-	-	-	-	-	-	-	-
		-	-	-	-	-	-	-	-	-	-	-	-	191057	-	-	-	-	-	-	-	-	-	-	-	-	-	-	-	-	-	-	-	-	-	-	-	-	-	-	-	-	-	-	-	-	-	-	-	-	-	-
PAT2	phosphate acetyltransferase	-	+	-	-	-	-	-	-	-	-	-	-	191051	-	-	-	-	-	-	-	-	-	-	-	-	-	-	-	-	-	-	-	-	48580	158834	108393	-	584069	-	-	-	-	-	-	21434	-	-	-	-	-	-
UBI2		-	+	-	-	-	-	-	AT1G31340.1	-	-	-	-	191044	-	-	-	-	-	-	19068636	34.m00254	-	GL50803_7110	-	-	-	-	-	-	-	Os09g25320.1	-	-	-	189028	-	-	816196	-	-	-	-	-	-	40669	-	-	-	-	-	-
PPO	Protoporphyrinogen oxidase, chloroplast precursor	-	+	-	-	-	-	GB10177-PA	AT4G01690.1	70035	BDEG_00581	-	228548	191043	-	-	CMB025C	-	DDB0184179	-	-	-	-	-	OTTHUMP00000032238	-	180405	11214	-	207217	-	Os01g18320.1	28476	-	31109	178131	127485	PF10_0275	755736	RO3G_04971.1	-	SPAC1F5.07c	GLEAN3_27448	SINFRUP00000168864	13.m00329	264901	-	59.m03633	31028	-	-	-
		-	-	-	-	-	-	-	-	-	-	-	-	191037	-	-	-	-	-	-	-	-	-	-	-	-	-	-	-	-	-	-	-	-	-	-	-	-	-	-	-	-	-	-	-	-	-	-	-	-	-	-
		-	-	-	-	-	-	-	AT1G14330.1	-	-	-	-	191035	-	-	-	-	-	-	-	-	-	-	-	-	-	-	-	-	-	Os02g30210.2	-	-	-	-	-	-	-	-	-	-	-	-	-	-	-	-	-	-	-	-
	mitochondrial membrane protein	-	-	-	-	-	-	-	-	-	-	-	-	191034	-	-	-	-	-	-	-	-	-	-	-	-	-	-	-	-	-	-	-	-	-	-	-	-	-	-	-	-	-	-	-	-	-	-	-	-	-	-
		-	-	-	-	-	-	-	-	-	-	-	-	191027	-	-	-	-	-	-	-	-	-	-	-	-	-	-	-	-	-	-	-	-	-	-	-	-	-	-	-	-	-	-	-	-	-	-	-	-	-	-
NUP155	nuclear pore protein	-	+	-	-	-	-	GB14727-PA	AT1G14850.1	-	BDEG_03042	CE33869	198384	191024	274239	-	CMH179C	-	DDB0189286	CG4579-PA	-	-	ENSGALP00000038303	-	NP_004289.1	LmjF36.6890	234706	22425	61200	20064	NCU11296	Os06g20090.1	35197	-	-	-	143496	-	656976	RO3G_13054.1	YBL079W	SPAC890.06	-	SINFRUP00000170191	-	-	-	-	29198	-	-	-
FAP102	Flagellar Associated Protein	-	-	-	+	-	-	-	-	-	-	-	-	191022	-	-	-	-	-	-	-	-	-	-	-	-	-	-	-	-	-	-	-	-	-	-	-	-	-	-	-	-	-	-	-	-	-	-	-	-	-	-
FAP233	Flagellar Associated Protein	-	-	-	-	-	-	-	-	-	-	-	-	191010	-	-	-	-	-	-	-	-	-	-	-	-	-	-	-	-	-	-	-	-	-	-	-	-	-	-	-	-	-	-	-	-	-	-	-	-	-	-
		-	-	-	-	-	-	-	-	-	-	-	-	191008	-	-	-	-	-	-	-	-	-	-	-	-	-	-	-	-	-	-	-	-	-	-	-	-	-	-	-	-	-	-	-	-	-	-	-	-	-	-
		-	-	-	-	-	-	-	-	-	-	-	-	191006	-	-	-	-	-	-	-	-	-	-	-	-	-	-	-	-	-	-	-	-	-	-	-	-	-	-	-	-	-	-	-	-	-	-	-	-	-	-
		-	+	-	-	-	-	-	-	-	-	-	-	191002	-	-	CMN301C	-	DDB0185963	-	-	-	-	-	-	-	-	-	57680	-	-	-	-	GSPATP00038590001	-	-	158709	-	-	-	-	-	-	SINFRUP00000148543	78.m00191	-	-	80.m02224	-	81190.m00250	-	UM05498.1
		-	-	-	-	-	-	-	-	-	-	-	-	191001	-	-	-	-	-	-	-	-	-	-	-	-	-	-	-	-	-	-	-	-	-	-	-	-	-	-	-	-	-	-	-	-	-	-	-	-	-	-
		-	-	-	-	-	-	-	-	-	-	-	-	190995	-	-	-	-	-	-	-	-	-	-	-	-	-	-	-	-	-	-	-	-	-	-	-	-	-	-	-	-	-	-	-	-	-	-	-	-	-	-
		-	-	-	-	-	-	-	-	-	-	-	-	190994	-	-	-	-	-	-	-	-	-	-	-	-	-	-	-	-	-	-	-	-	-	-	-	-	-	-	-	-	-	-	-	-	-	-	-	-	-	-
		-	-	-	-	-	-	-	-	-	-	-	-	190992	-	-	-	-	-	-	-	-	-	-	-	-	-	-	-	-	-	-	-	-	-	-	-	-	-	-	-	-	-	-	-	-	-	-	-	-	-	-
		-	-	-	-	-	-	-	-	-	-	-	-	190991	-	-	-	-	-	-	-	-	-	-	-	-	-	-	-	-	-	-	-	-	-	-	-	-	-	-	-	-	-	-	-	-	-	-	-	-	-	-
		-	-	-	-	-	-	-	-	-	-	-	-	190985	-	-	-	-	-	-	-	-	-	-	-	-	-	-	-	-	-	-	-	-	-	-	-	-	-	-	-	-	-	-	-	-	-	-	-	-	-	-
ELG24	exostosin-like glycosyltransferase	-	-	-	-	-	-	-	-	-	-	-	-	190983	-	-	-	-	-	-	-	-	-	-	-	-	-	-	-	-	-	-	-	-	-	-	-	-	-	-	-	-	-	-	-	-	-	-	-	-	-	-
MCP1	molybdenum cofactor carrier protein	-	-	-	-	-	-	-	-	-	-	-	-	190981	-	-	-	-	-	-	-	-	-	-	-	-	-	23564	-	-	-	-	-	-	-	-	-	-	-	-	-	-	-	-	-	-	-	-	-	-	-	-
OAT1	O-acetyltransferase-related protein	-	+	-	-	-	-	GB15379-PA	AT1G29890.1	1346	-	-	145222	190979	-	-	-	OTTDARP00000015776	-	CG2938-PB	-	-	ENSGALP00000015703	-	OTTHUMP00000024974	-	63982	24903	-	70299	-	Os03g19970.1	20739	-	20024	226366	-	-	641544	RO3G_12385.1	-	-	GLEAN3_09151	SINFRUP00000144073	-	34795	-	-	-	-	-	-
FAP93	Flagellar Associated Protein	-	-	-	+	-	-	-	-	-	-	-	-	190974	-	-	-	-	-	-	-	-	-	-	-	-	-	-	-	-	-	-	-	-	-	-	-	-	-	-	-	-	-	-	-	-	-	-	-	-	-	-
		-	+	-	-	-	-	GB14969-PA	AT5G01430.1	58829	-	CE39501	222856	190973	297856	cgd2_190	CMI302C	OTTDARP00000007886	DDB0191661	CG32576-PB	-	-	ENSGALP00000029898	-	OTTHUMP00000166885	LmjF18.1620	159400	14085	31182	167406	NCU06396	Os03g11100.1	7494	GSPATP00010940001	16648	187613	108875	PFD0930w	741425	RO3G_06625.1	YMR292W	SPCC4B3.02c	GLEAN3_23058	SINFRUP00000167782	-	19662	-	86.m00374	61346	86904.m00058	Tb10.389.1310	-
GDH1		-	-	-	-	-	-	-	-	-	-	-	-	190969	-	-	-	-	-	-	-	-	-	-	-	-	-	-	1098	-	-	-	-	-	13951	-	108919	-	-	-	-	-	-	-	-	-	-	-	-	-	-	-
TEF21	Putative phosphoinositide phosphatase, mitochondrial localization assumed	-	+	+	-	-	-	GB16830-PA	AT5G66020.1	-	BDEG_04467	CE09807	220075	190968	240852	cgd4_80	CMB156C	OTTDARP00000015054	DDB0216876	CG9128-PA	19168741	107.m00109	ENSGALP00000019303	GL50803_8589	NP_054735.2	-	187933	24693	928	118891	NCU00896	Os02g34884.1	-	GSPATP00038474001	-	182250	157224	PF13_0285	558773	RO3G_10760.1	YKL212W	SPBC19F5.03	GLEAN3_14517	SINFRUP00000182948	34.m00240	262615	TA16590	583.m05734	24656	90756.m00160	-	UM00176.1
		-	-	-	-	-	-	-	-	-	-	-	-	190967	-	-	-	-	-	-	-	-	-	-	-	-	-	-	-	-	-	-	-	-	-	-	-	-	-	-	-	-	-	-	-	-	-	-	-	-	-	-
		-	+	-	-	-	-	-	AT2G16460.1	-	-	-	168302	190964	-	-	-	-	-	-	-	-	ENSGALP00000027835	-	NP_068597.2	-	163625	-	-	-	NCU02504	Os06g49890.1	-	-	-	130678	-	-	821141	RO3G_07337.1	YFL046W	SPAC2C4.09	-	SINFRUP00000132967	-	-	-	-	-	-	-	UM02107.1
		-	-	-	-	-	-	-	-	-	-	-	-	190954	-	-	-	-	-	-	-	-	-	-	-	-	-	-	-	-	-	-	-	-	-	-	-	-	-	-	-	-	-	-	-	-	-	-	-	-	-	-
		-	-	-	-	-	-	-	-	-	-	-	-	190949	-	-	-	-	-	-	-	-	-	-	-	-	-	-	-	-	-	-	-	-	-	-	-	-	-	-	-	-	-	-	-	-	-	-	-	-	-	-
		-	-	-	-	-	-	-	-	-	-	-	-	190948	-	-	-	-	-	-	-	-	-	-	-	-	-	-	-	-	-	-	-	-	-	-	-	-	-	-	-	-	-	-	-	-	-	-	-	-	-	-
		-	-	-	-	-	-	-	AT1G06730.1	-	-	-	-	190942	-	-	-	-	-	-	-	-	-	-	-	-	-	-	-	-	-	Os03g06880.1	34943	-	-	-	-	-	205730	-	-	-	-	-	-	-	-	-	-	-	-	-
		-	-	-	-	-	-	-	-	-	-	-	-	190939	-	-	-	-	-	-	-	-	-	-	-	-	-	-	-	-	-	-	-	-	-	-	-	-	-	-	-	-	-	-	-	-	-	-	-	-	-	-
FBB5		+	+	-	-	-	-	GB19146-PA	-	63920	BDEG_00462	-	204568	190937	285773	-	-	OTTDARP00000001964	-	-	-	-	ENSGALP00000027722	GL50803_8942	NP_116319.1	-	229887	-	72345	237431	-	-	-	GSPATP00011350001	-	-	135769	-	-	-	-	-	-	SINFRUP00000158972	81.m00122	-	-	-	52162	93857.m00376	-	-
		-	+	-	-	-	-	-	-	-	-	CE01925	-	190934	-	-	-	-	-	-	-	-	ENSGALP00000015837	-	NP_078880.1	LmjF27.2390	74812	-	-	248100	-	-	-	GSPATP00037155001	47492	-	-	-	811235	-	YBR155W	SPAC6B12.12	-	SINFRUP00000174746	-	5128	-	-	-	-	-	-
		-	-	-	-	-	-	-	-	-	-	-	-	190926	-	-	-	-	-	-	-	-	-	-	-	-	-	-	-	-	-	-	-	-	-	-	-	-	-	-	-	-	-	-	-	-	-	-	-	-	-	-
		-	+	-	-	-	-	-	AT3G57410.1	13277	BDEG_02730	CE06107	150579	190925	292267	-	-	OTTDARP00000022646	-	CG1106-PA	-	-	ENSGALP00000038047	-	OTTHUMP00000025505	-	221805	34323	44842	113215	-	Os03g24220.2	-	-	19089	117446	136639	-	819056	RO3G_05754.1	-	-	GLEAN3_00296	SINFRUP00000178365	-	22984	-	-	54421	93331.m00037	-	-
		-	-	-	-	-	-	-	-	-	-	-	-	190924	-	-	-	-	-	-	-	-	-	-	-	-	-	-	-	-	-	-	-	-	-	-	-	-	-	-	-	-	-	-	-	-	-	-	-	-	-	-
RER1	Retrieval of ER proteins-like	-	+	+	-	-	-	GB18994-PA	AT2G23310.1	70415	BDEG_05704	CE03349	225288	190919	291034	cgd2_2250	CMI263C	OTTDARP00000021077	DDB0184462	-	19173396	180.m00099	ENSGALP00000034539	GL50803_15413	OTTHUMP00000001502	LmjF22.0580	188487	33625	81308	185483	NCU06447	Os06g49460.1	21127	GSPATP00027855001	11248	206296	109284	PFI0150c	655092	RO3G_16849.1	YCL001W	SPAC22E12.05c	-	SINFRUP00000175053	42.m00258	36206	TA17635	80.m02317	7073	95400.m00120	Tb927.7.2510	UM03668.1
NUOB10	NADH:ubiquinone oxidoreductase (Complex I) 17.8 kD subunit, homolog to bovine PDSW subunit, mitochondrial	-	-	-	-	-	-	-	-	-	-	-	-	190916	-	-	-	-	-	-	-	-	-	-	-	-	-	-	-	-	-	-	-	-	-	216676	-	-	-	-	-	-	-	-	-	-	-	-	-	-	-	-
		-	-	-	-	-	-	-	-	-	-	-	-	190915	-	-	-	-	-	-	-	-	-	-	-	-	-	-	-	-	-	-	-	-	-	-	-	-	-	-	-	-	-	-	-	-	-	-	-	-	-	-
		-	-	-	-	-	-	-	-	-	-	-	-	190913	-	-	-	-	-	-	-	-	-	-	-	-	-	-	-	-	-	-	-	-	-	-	-	-	-	-	-	-	-	-	-	-	-	-	-	-	-	-
		-	-	-	-	-	-	-	-	-	-	-	-	190912	-	-	-	-	-	-	-	-	-	-	-	-	-	-	-	-	-	-	-	-	-	-	-	-	-	-	-	-	-	-	-	-	-	-	-	-	-	-
		-	-	-	-	-	-	-	-	-	-	-	-	190891	-	-	-	-	-	-	-	-	-	-	-	-	-	-	-	-	-	-	-	-	-	-	-	-	-	-	-	-	-	-	-	-	-	-	-	-	-	-
		-	-	-	-	-	-	-	-	-	-	-	-	190885	-	-	-	-	-	-	-	-	-	-	-	-	-	-	-	-	-	-	-	-	-	-	-	-	-	-	-	-	-	-	-	-	-	-	-	-	-	-
		-	+	-	-	-	-	-	AT3G61690.1	-	-	-	-	190884	-	-	-	-	-	-	-	-	-	GL50803_11638	-	LmjF18.1450	-	-	81677	-	-	Os03g11240.1	23249	-	-	234441	136186	-	422878	-	-	-	-	-	-	-	-	33.m01329	-	-	Tb10.389.1040	-
		-	-	-	-	-	-	-	-	-	-	-	-	190882	-	-	-	-	-	-	-	-	-	-	-	-	-	-	-	-	-	-	-	-	-	-	-	-	-	-	-	-	-	-	-	-	-	-	-	-	-	-
		-	-	-	-	-	-	-	-	-	-	-	201170	190879	-	-	-	-	-	-	-	-	-	-	-	-	-	-	-	-	-	-	-	-	-	-	-	-	-	-	-	-	-	-	-	-	-	-	-	-	-	-
		-	-	-	-	-	-	-	-	-	-	-	-	190872	-	-	-	-	-	-	-	-	-	-	-	-	-	-	-	-	-	-	-	-	-	-	-	-	-	-	-	-	-	-	-	-	-	-	-	-	-	-
		-	-	-	-	-	-	-	-	-	-	-	-	190871	-	-	-	-	-	-	-	-	-	-	-	-	-	-	-	-	-	-	-	-	-	-	-	-	-	-	-	-	-	-	-	-	-	-	-	-	-	-
LAO3		-	-	-	-	-	-	-	-	-	-	-	-	190870	-	-	-	-	-	-	-	-	-	-	-	-	-	-	-	-	-	-	-	-	-	-	-	-	-	-	-	-	-	-	-	-	-	-	-	-	-	-
		-	-	-	-	-	-	-	-	-	-	-	-	190866	-	-	-	-	-	-	-	-	-	-	-	-	-	-	-	-	-	-	-	-	-	-	-	-	-	-	-	-	-	-	-	-	-	-	-	-	-	-
		-	+	-	-	-	-	-	AT5G51040.1	16747	BDEG_02619	CE22606	-	190864	-	-	CMJ163C	-	DDB0192217	-	-	-	ENSGALP00000040533	-	NP_060311.1	LmjF30.1070	-	35418	-	-	NCU01343	Os11g32480.1	30351	GSPATP00012740001	43817	112512	157751	-	666780	RO3G_16323.1	YOL071W	SPAC12B10.06c	-	-	26.m00383	33219	-	-	-	-	Tb927.6.2510	UM00035.1
		-	-	-	-	-	-	-	-	-	-	-	-	190856	-	-	-	-	-	-	-	-	-	-	-	-	-	-	-	-	-	-	-	-	-	-	-	-	-	-	-	-	-	-	-	-	-	-	-	-	-	-
		-	-	-	-	-	-	-	-	-	-	-	-	190852	-	-	-	-	-	-	-	-	-	-	-	-	-	-	-	-	-	-	-	-	-	-	-	-	-	-	-	-	-	-	-	-	-	-	-	-	-	-
MIP1	aquaporin, glycerol transport activity	-	+	-	-	-	-	GB17478-PA	AT2G36830.1	29268	BDEG_05070	CE05345	25505	190851	293323	-	CMK067C	OTTDARP00000004598	DDB0205768	CG9023-PB	19069138	-	ENSGALP00000024367	-	OTTHUMP00000167433	LmjF34.3850	82572	29850	75649	185753	NCU08052	Os01g74450.1	-	GSPATP00023946001	44871	62169	140531	-	549212	RO3G_15947.1	YFL054C	SPAC977.17	GLEAN3_12095	SINFRUP00000127844	-	924	-	33.m00008	51804	-	Tb10.61.2650	UM00223.1
		-	-	-	-	-	-	-	-	-	-	-	-	190850	-	-	-	-	-	-	-	-	-	-	-	-	-	-	-	-	-	-	-	-	-	-	-	-	-	-	-	-	-	-	-	-	-	-	-	-	-	-
		-	-	-	-	-	-	-	-	-	-	-	-	190849	-	-	-	-	-	-	-	-	-	-	-	-	-	-	-	-	-	-	-	-	-	-	-	-	-	-	-	-	-	-	-	-	-	-	-	-	-	-
		-	-	-	-	-	-	-	-	-	-	-	-	190839	-	-	-	-	-	-	-	-	-	-	-	-	-	-	-	-	-	-	-	-	-	-	-	-	-	-	-	-	-	-	-	-	-	-	-	-	-	-
LHCBM2	light-harvesting protein of photosystem I;	-	-	-	-	-	-	-	-	-	-	-	-	190838	-	-	-	-	-	-	-	-	-	-	-	-	-	-	-	-	-	-	-	-	-	-	-	-	-	-	-	-	-	-	-	-	-	-	-	-	-	-
UBC7		-	-	-	-	-	-	-	-	-	-	-	-	190835	-	-	-	-	-	-	-	-	-	-	-	-	-	-	-	-	-	-	-	-	-	-	-	-	-	-	-	-	-	-	-	-	-	-	-	-	-	-
		-	-	-	-	-	-	-	-	-	-	-	-	190834	-	-	-	-	-	-	-	-	-	-	-	-	-	-	-	-	-	-	-	-	-	-	-	-	-	-	-	-	-	-	-	-	-	-	-	-	-	-
		-	-	-	-	-	-	-	-	-	-	-	-	190829	-	-	-	-	-	-	-	-	-	-	-	-	-	-	-	-	-	-	-	-	-	-	-	-	-	-	-	-	-	-	-	-	-	-	-	-	-	-
	putative methyltransferase	-	+	-	-	-	-	-	AT3G01660.1	-	-	-	-	190827	-	-	-	-	-	-	-	-	-	-	-	-	-	-	-	-	-	Os04g40530.1	16714	-	-	110754	-	-	174117	-	-	-	-	-	-	-	-	-	-	-	-	-
		-	-	-	-	-	-	-	-	-	-	-	-	190826	-	-	-	-	-	-	-	-	-	-	-	-	-	-	-	-	-	-	-	-	-	-	-	-	-	-	-	-	-	-	-	-	-	-	-	-	-	-
		-	+	+	-	-	-	GB13798-PA	AT3G46460.1	60286	BDEG_03821	CE00216	103291	190824	218134	cgd1_410	CMS503C	OTTDARP00000018206	DDB0168503	CG40045-PA	19068782	107.m00123	ENSGALP00000039649	GL50803_3171	OTTHUMP00000183055	LmjF32.0960	225382	36943	83191	167818	NCU03623	Os05g38550.2	17056	GSPATP00026002001	28433	110232	108483	PFI1030c	227541	RO3G_06175.1	YMR022W	SPBC1105.09	-	SINFRUP00000131210	46.m00166	3013	TA10690	20.m03747	20134	91117.m00136	Tb11.01.5790	UM02426.1
FAP125	Flagellar Associated Protein Similar to Kinesin	-	-	-	+	-	-	-	-	28941	-	-	-	190816	-	-	-	-	-	-	-	-	-	-	-	-	-	-	-	-	-	-	-	-	-	-	-	-	-	-	-	-	-	-	-	-	-	-	-	-	-	-
		-	+	-	-	-	-	-	AT3G01740.1	-	BDEG_03086	-	-	190813	-	-	-	-	-	-	-	-	-	-	OTTHUMP00000070829	-	-	-	-	240338	-	Os12g14080.2	7655	-	-	29109	-	-	549644	-	-	-	-	-	-	-	-	-	60330	-	-	-
		-	-	-	+	-	-	-	-	-	-	-	-	190810	-	-	-	-	-	-	-	-	-	-	-	-	-	-	-	-	-	-	-	-	-	-	-	-	-	-	-	-	-	-	-	-	-	-	-	-	-	-
		-	-	-	-	-	-	-	-	-	-	-	-	190806	-	-	-	-	-	-	-	-	-	-	-	-	-	-	-	-	-	-	-	-	-	-	-	-	-	-	-	-	-	-	-	-	-	-	-	-	-	-
		-	-	-	-	-	-	-	-	-	-	-	-	190804	-	-	-	-	-	-	-	-	-	-	-	-	-	-	-	-	-	-	-	-	-	-	-	-	-	-	-	-	-	-	-	-	-	-	-	-	-	-
		-	-	-	-	-	-	-	-	-	-	-	-	190801	-	-	-	-	-	-	-	-	-	-	-	-	-	-	-	-	-	-	-	-	-	-	-	-	-	-	-	-	-	-	-	-	-	-	-	-	-	-
		-	-	-	-	-	-	-	-	-	-	-	-	190800	-	-	-	-	-	-	-	-	-	-	-	-	-	-	-	-	-	-	-	-	-	-	-	-	-	-	-	-	-	-	-	-	-	-	-	-	-	-
		-	-	-	-	-	-	-	-	-	-	-	-	190798	-	-	-	-	-	-	-	-	-	-	-	-	-	-	-	-	-	-	-	-	-	-	-	-	-	-	-	-	-	-	-	-	-	-	-	-	-	-
RSP5	Radial spoke protein 5	-	-	-	+	-	-	-	-	-	-	-	-	190792	-	-	-	-	-	-	-	-	-	-	-	-	-	-	-	-	-	-	-	-	-	-	-	-	-	-	-	-	-	-	-	-	-	-	-	-	-	-
		-	-	-	-	-	-	-	-	-	-	-	-	190791	-	-	-	-	-	-	-	-	-	-	-	-	-	-	-	-	-	-	-	-	-	-	-	-	-	-	-	-	-	-	-	-	-	-	-	-	-	-
		-	-	-	-	-	-	-	-	-	-	-	-	190790	-	-	-	-	-	-	-	-	-	-	-	-	-	-	-	-	-	-	-	-	-	-	-	-	-	-	-	-	-	-	-	-	-	-	-	-	-	-
		-	-	-	-	-	-	-	-	-	-	-	-	190789	-	-	-	-	-	-	-	-	-	-	-	-	-	-	-	-	-	-	-	-	-	-	-	-	-	-	-	-	-	-	-	-	-	-	-	-	-	-
		-	+	+	-	-	-	GB15969-PA	AT2G45730.1	64086	BDEG_07679	CE35204	113381	190782	219719	-	-	-	DDB0204062	CG9596-PB	-	94.m00133	ENSGALP00000014919	GL50803_2323	OTTHUMP00000030210	LmjF36.5780	234974	23559	32088	119805	-	Os04g02150.2	17093	GSPATP00001289001	16688	145080	127192	PFI0625c	559822	RO3G_04763.1	YNL062C	SPBC800.08	GLEAN3_14020	SINFRUP00000179942	65.m00200	-	TA05665	74.m00434	59791	-	Tb10.6k15.1410	UM03768.1
		-	-	-	-	-	-	-	-	-	-	-	-	190775	-	-	-	-	-	-	-	-	-	-	-	-	-	-	-	-	-	-	-	-	-	-	-	-	-	-	-	-	-	-	-	-	-	-	-	-	-	-
		-	-	-	-	-	-	-	-	-	-	-	-	190773	-	-	-	-	-	-	-	-	-	-	-	-	-	-	-	-	-	-	-	-	-	-	-	-	-	-	-	-	-	-	-	-	-	-	-	-	-	-
		-	+	-	-	-	-	-	-	-	-	-	20984	190768	298200	-	-	-	DDB0187175	-	-	-	-	-	-	-	-	38315	-	241459	-	-	32056	-	-	132791	-	-	821174	-	-	-	-	-	-	-	-	-	-	-	-	-
	related to phosphoribulokinase/uridine kinase	-	+	-	-	-	-	-	AT1G80380.3	-	-	-	-	190767	-	-	CMK141C	-	-	-	-	-	-	-	-	-	-	-	-	-	NCU08827	Os01g48990.2	33357	GSPATP00014444001	-	111900	136559	-	829520	RO3G_07846.1	YGR205W	SPAC630.09c	-	-	4.m00623	-	-	-	-	-	-	UM01824.1
		-	-	-	-	-	-	-	-	-	-	-	-	190765	-	-	-	-	-	-	-	-	-	-	-	-	-	-	-	-	-	-	-	-	-	-	-	-	-	-	-	-	-	-	-	-	-	-	-	-	-	-
		-	-	-	-	-	-	-	-	-	-	-	-	190761	-	-	-	-	-	-	-	-	-	-	-	-	-	-	-	-	-	-	-	-	-	-	-	-	-	-	-	-	-	-	-	-	-	-	-	-	-	-
		-	-	-	-	-	-	-	-	-	-	-	-	190760	-	-	-	-	-	-	-	-	-	-	-	-	-	-	-	-	-	-	-	-	-	-	-	-	-	-	-	-	-	-	-	-	-	-	-	-	-	-
		-	-	-	-	-	-	-	-	-	-	-	-	190755	-	-	-	-	-	-	-	-	-	-	-	-	-	-	-	-	-	-	-	-	-	-	-	-	-	-	-	-	-	-	-	-	-	-	-	-	-	-
		-	-	-	-	-	-	-	-	-	-	-	-	190746	-	-	-	-	-	-	-	-	-	-	-	-	-	-	-	-	-	-	-	-	-	-	-	-	-	-	-	-	-	-	-	-	-	-	-	-	-	-
		-	-	-	-	-	-	-	-	-	-	-	-	190741	-	-	-	-	-	-	-	-	-	-	-	-	-	-	-	-	-	-	-	-	-	-	-	-	-	-	-	-	-	-	-	-	-	-	-	-	-	-
		-	-	-	-	-	-	-	-	-	-	-	-	190740	-	-	-	-	-	-	-	-	-	-	-	-	-	-	-	-	-	-	-	-	-	-	-	-	-	-	-	-	-	-	-	-	-	-	-	-	-	-
	putative organic anion transporter	-	-	-	-	-	-	-	-	65625	-	-	-	190739	-	-	-	-	-	-	-	-	-	-	-	-	-	-	-	-	-	-	-	-	-	-	-	-	-	-	-	-	-	-	-	-	-	-	-	-	-	-
		-	-	-	-	-	-	-	-	-	-	-	-	190737	-	-	-	-	-	-	-	-	-	-	-	-	-	-	-	-	-	-	-	-	-	-	-	-	-	-	-	-	-	-	-	-	-	-	-	-	-	-
RAD51	DNA recombination protein, RecA homologue.	-	+	+	-	-	-	GB17143-PA	AT5G20850.1	-	BDEG_03290	CE25285	114057	190735	218932	cgd5_410	CMR053C	OTTDARP00000006524	DDB0217219	-	19069607	63.m00157	ENSGALP00000032107	-	OTTHUMP00000175639	LmjF28.0550	-	-	-	-	NCU02741	Os11g40150.1	18636	GSPATP00036933001	-	-	108595	PF11_0087	589354	RO3G_10066.1	YER095W	SPAC644.14c	GLEAN3_09590	SINFRUP00000163886	11.m00336	261577	TA07350	59.m03654	-	87076.m00035	Tb11.01.0360	UM03290.1
		-	-	-	-	-	-	-	-	-	-	-	-	190733	-	-	-	-	-	-	-	-	-	-	-	-	-	-	-	-	-	-	-	-	-	-	-	-	-	-	-	-	-	-	-	-	-	-	-	-	-	-
		-	+	-	-	-	-	-	AT1G56700.3	28248	-	-	-	190730	-	-	CMD144C	-	DDB0189325	-	-	-	-	-	-	-	-	-	-	-	-	Os09g23280.1	-	-	43368	55238	-	-	671512	-	-	-	-	-	-	5692	-	-	-	-	-	-
		-	-	-	-	-	-	-	-	-	-	-	-	190724	-	-	-	-	-	-	-	-	-	-	-	-	-	-	-	-	-	-	-	-	-	-	-	-	-	-	-	-	-	-	-	-	-	-	-	-	-	-
FAP99	Flagellar Associated Protein	+	+	-	+	-	-	-	-	67374	BDEG_04195	-	191698	190721	280128	-	-	-	-	-	-	-	ENSGALP00000025252	-	XP_498961.1	-	188889	33293	50666	100119	-	-	-	GSPATP00025993001	-	234644	131524	-	-	-	-	-	-	SINFRUP00000180934	90.m00132	-	-	-	34497	-	Tb10.v4.0044	-
		-	-	-	-	-	-	-	-	-	-	-	-	190719	-	-	-	-	-	-	-	-	-	-	-	-	-	-	-	-	-	-	30229	-	-	72224	-	-	-	-	-	-	-	-	-	-	-	-	-	-	-	-
		-	-	-	-	-	-	-	-	-	-	-	-	190718	-	-	-	-	-	-	-	-	-	-	-	-	-	-	-	-	-	-	-	-	-	-	-	-	-	-	-	-	-	-	-	-	-	-	-	-	-	-
		-	-	-	-	-	-	-	-	-	-	-	-	190716	-	-	-	-	-	-	-	-	-	-	-	-	-	-	-	-	-	-	-	-	-	-	-	-	-	-	-	-	-	-	-	-	-	-	-	-	-	-
		+	+	-	-	-	-	GB15454-PA	-	-	BDEG_06518	CE00526	185094	190708	217903	-	-	-	-	CG14995-PD	-	-	ENSGALP00000010549	GL50803_16891	OTTHUMP00000109511	LmjF36.0710	119159	25224	74042	236584	-	-	-	GSPATP00004683001	-	-	-	-	-	-	-	-	-	SINFRUP00000137836	90.m00112	-	-	-	52163	86319.m00286	Tb10.70.2080	-
TIC22	22 kDa translocon at the inner membrane of chloroplasts	-	+	-	-	-	-	-	AT3G23710.1	-	-	-	-	190703	-	-	CMJ105C	-	-	-	-	-	-	-	-	-	-	-	-	-	-	Os06g11020.1	-	-	-	171249	-	-	816875	-	-	-	-	-	-	-	-	-	-	-	-	-
		-	-	-	-	-	-	-	-	-	-	-	-	190701	-	-	-	-	-	-	-	-	-	-	-	-	-	-	-	-	-	-	-	-	-	-	-	-	-	-	-	-	-	-	-	-	-	-	-	-	-	-
		-	-	-	-	-	-	-	-	-	-	-	-	190700	-	-	-	-	-	-	-	-	-	-	-	-	-	-	-	-	-	-	-	-	-	-	-	-	-	-	-	-	-	-	-	-	-	-	-	-	-	-

		-	-	-	-	-	-	-	-	-	-	-	-	190697	-	-	-	-	-	-	-	-	-	-	-	-	-	-	-	-	-	-	-	-	-	-	-	-	-	-	-	-	-	-	-	-	-	-	-	-	-	-
		-	-	-	-	-	-	-	-	-	-	-	-	190692	-	-	-	-	-	-	-	-	-	-	-	-	-	-	-	-	-	-	-	-	-	-	-	-	-	-	-	-	-	-	-	-	-	-	-	-	-	-
		-	-	-	-	-	-	-	-	-	-	-	-	190685	-	-	-	-	-	-	-	-	-	-	-	-	-	-	-	-	-	-	-	-	-	-	-	-	-	-	-	-	-	-	-	-	-	-	-	-	-	-
		+	+	-	+	-	-	GB18692-PA	-	-	-	-	-	190684	-	-	-	OTTDARP00000023599	-	CG8814-PA	-	-	ENSGALP00000012143	-	OTTHUMP00000019367	LmjF09.0750	-	-	-	220136	-	-	-	-	-	-	-	PF10_0015	-	-	-	-	-	SINFRUP00000171769	82.m00112	-	-	-	-	-	Tb927.7.6770	-
SPL7		-	+	-	-	-	-	GB16152-PA	-	-	-	CE30324	189098	190680	236940	cgd1_350	CMF163C	OTTDARP00000020756	-	CG8205-PE	-	-	ENSGALP00000005323	-	NP_060167.2	-	91728	34217	81544	30522	-	-	-	GSPATP00026956001	-	85834	-	PF10_0235	-	-	-	-	GLEAN3_14611	SINFRUP00000158224	3.m01740	-	TA18000	46.m01699	29141	-	Tb927.2.3880	-
		-	-	-	-	-	-	-	AT1G72230.1	-	-	-	-	190674	-	-	-	-	-	-	-	-	-	-	-	-	-	-	-	-	-	Os06g11490.1	-	-	-	65907	-	-	171892	-	-	-	-	-	-	-	-	-	-	-	-	-
		-	+	-	-	-	-	GB17057-PA	-	-	-	CE00825	-	190662	-	-	CMK211C	-	-	CG12224-PA	-	-	-	-	-	-	199898	13893	-	-	NCU05594	Os12g29760.2	-	-	-	-	-	-	815719	RO3G_17029.1	YMR041C	-	-	-	-	-	-	-	22637	-	-	UM02683.1
		-	+	-	-	-	-	-	-	-	-	-	151400	190657	-	-	-	-	DDB0188643	-	-	-	ENSGALP00000024777	-	OTTHUMP00000017823	-	-	-	-	125073	-	-	-	GSPATP00013195001	-	-	-	-	-	-	-	-	-	-	-	-	-	-	-	-	-	-
		-	-	-	-	-	-	-	-	-	-	-	-	190655	-	-	-	-	-	-	-	-	-	-	-	-	-	-	-	-	-	-	-	-	-	-	-	-	-	-	-	-	-	-	-	-	-	-	-	-	-	-
		-	-	-	-	-	-	-	-	-	-	-	-	190654	-	-	-	-	-	-	-	-	-	-	-	-	-	-	-	-	-	-	-	-	-	-	-	-	-	-	-	-	-	-	-	-	-	-	-	-	-	-
FAP94	Flagellar Associated Protein	-	-	-	+	-	-	-	-	-	BDEG_05685	-	-	190653	-	-	-	-	-	-	-	-	-	-	-	-	-	-	-	-	-	-	-	-	-	-	134571	-	-	-	-	-	-	-	-	-	-	-	-	-	-	-	-
TCR1-B	Tcr1 ORF2	-	-	-	-	-	-	-	-	-	-	-	-	190650	-	-	-	-	-	-	-	-	-	-	-	-	-	-	-	-	-	-	-	-	-	-	-	-	-	-	-	-	-	-	-	-	-	-	-	-	-	-
TCR1-A	Tcr1 ORF1	-	-	-	-	-	-	-	-	-	-	-	-	190649	-	-	-	-	-	-	-	-	-	-	-	-	-	-	-	-	-	-	-	-	-	-	-	-	-	-	-	-	-	-	-	-	-	-	-	-	-	-
FAP138	Flagellar Associated Protein	-	-	-	+	-	-	-	-	-	-	-	-	190644	-	-	-	-	-	-	-	-	-	-	-	-	-	-	-	-	-	-	-	-	-	-	-	-	-	-	-	-	-	-	-	-	-	-	-	-	-	-
		-	-	-	-	-	-	-	-	-	-	-	-	190643	-	-	-	-	-	-	-	-	-	-	-	-	-	-	-	-	-	-	-	-	-	-	-	-	-	-	-	-	-	-	141.m00082	-	-	-	-	-	-	-
IPB2	Importin Beta	-	+	-	-	-	-	GB18198-PA	AT5G19820.1	-	BDEG_00420	CE26971	179015	190633	236241	cgd7_3270	CMI255C	-	DDB0190979	CG1059-PA	-	-	ENSGALP00000027238	GL50803_15106	NP_002262.3	LmjF32.2150	184912	32626	36824	235624	NCU02357	Os07g38760.1	14349	GSPATP00035072001	23582	206059	139352	PFE1195w	555202	RO3G_11688.1	YMR308C	SPCC1840.03	GLEAN3_00842	SINFRUP00000165332	72.m00101	262326	TA21430	44.m02525	51271	94232.m00226	Tb11.01.7010	-
		-	-	-	-	-	-	-	-	-	-	-	-	190632	-	-	-	-	-	-	-	-	-	-	-	-	-	-	-	-	-	-	-	-	-	-	-	-	-	-	-	-	-	-	-	-	-	-	-	-	-	-
		-	-	-	-	-	-	-	-	-	-	-	-	190631	-	-	-	-	-	-	-	-	-	-	-	-	-	-	-	-	-	-	-	-	-	-	-	-	-	-	-	-	-	-	-	-	-	-	-	-	-	-
		-	-	-	-	-	-	-	-	-	-	-	-	190630	-	-	-	-	-	-	-	-	-	-	-	-	-	-	-	-	-	-	-	-	-	-	-	-	-	-	-	-	-	-	-	-	-	-	-	-	-	-
		-	-	-	-	-	-	-	-	-	-	-	-	190629	-	-	-	-	-	-	-	-	-	-	-	-	-	-	-	-	-	-	-	-	-	-	-	-	-	-	-	-	-	-	-	-	-	-	-	-	-	-
		-	-	-	-	-	-	-	-	-	-	-	-	190621	-	-	-	-	-	-	-	-	-	-	-	-	-	-	-	-	-	-	-	-	-	-	-	-	-	-	-	-	-	-	-	-	-	-	-	-	-	-
		-	+	+	-	-	-	GB16143-PA	AT3G12050.1	-	BDEG_04626	CE07817	163441	190619	-	cgd2_320	CMP323C	OTTDARP00000021148	DDB0190493	CG1416-PC	-	76.m00157	ENSGALP00000017014	GL50803_10423	OTTHUMP00000028484	LmjF18.0210	124038	32329	37564	118846	NCU04087	Os08g36150.1	15139	GSPATP00001591001	-	227834	-	PFC0360w	561237	RO3G_06940.1	YDR214W	SPBC1711.08	-	SINFRUP00000168646	15.m00404	-	-	49.m03383	59786	-	Tb10.61.3210	UM04823.1
FAP129	Flagellar Associated Protein	-	-	-	+	-	-	-	-	72505	-	-	-	190617	-	-	-	-	-	-	-	-	-	-	-	-	-	-	-	-	-	-	-	GSPATP00014590001	-	-	-	-	-	-	-	-	-	-	-	-	-	-	-	-	-	-
	Putative programmed cell death protein 6	-	+	-	-	-	-	GB19808-PA	AT3G10300.3	-	-	CE40264	151788	190610	287486	-	-	OTTDARP00000015807	DDB0189235	CG17765-PA	-	224.m00112	ENSGALP00000021816	GL50803_2933	OTTHUMP00000115318	LmjF13.1460	211477	35370	80960	236562	NCU02738	Os12g04240.1	23879	-	-	78816	-	-	679950	RO3G_07276.1	YGR058W	-	GLEAN3_22779	SINFRUP00000131090	-	-	-	-	28496	-	-	UM06140.1
		-	-	-	-	-	-	-	-	-	-	-	-	190605	-	-	-	-	-	-	-	-	-	-	-	-	-	-	51083	114326	NCU10658	-	-	-	35562	-	-	-	-	-	-	-	-	-	-	-	-	-	-	-	-	-
		-	-	-	-	-	-	-	-	-	-	-	-	190604	-	-	-	-	-	-	-	-	-	-	-	-	-	-	-	-	-	-	-	-	-	-	-	-	-	-	-	-	-	-	-	-	-	-	-	-	-	-
		-	+	-	-	-	-	GB12238-PA	AT4G01040.1	-	BDEG_04574	CE30126	174783	190601	223016	-	-	-	DDB0188880	CG8460-PA	-	250.m00078	ENSGALP00000011025	-	OTTHUMP00000164438	-	135362	1599	-	127721	-	Os01g43220.1	-	GSPATP00014649001	-	191448	130467	-	244897	RO3G_00916.1	-	-	GLEAN3_04590	SINFRUP00000130491	118.m00144	-	-	-	31888	81238.m00173	-	UM05290.1
FAP166	Flagellar Associated Protein	-	-	-	+	-	-	-	-	-	-	-	-	190600	-	-	-	-	-	-	-	-	-	-	-	-	-	-	-	-	-	-	-	-	-	-	-	-	-	-	-	-	-	-	-	-	-	-	-	-	-	-
	Putative DNA (cytosine-C5) methyltransferase	-	-	-	-	-	-	-	-	-	-	-	-	190599	-	-	-	-	-	-	-	-	-	-	-	-	-	-	-	-	-	-	-	-	-	-	-	-	-	-	-	-	-	-	-	-	-	-	-	-	-	-
		-	-	-	-	-	-	-	-	-	-	-	-	190598	-	-	-	-	-	-	-	-	-	-	-	-	-	-	-	-	-	-	-	-	-	-	-	-	-	-	-	-	-	-	-	-	-	-	-	-	-	-
		-	-	-	-	-	-	-	-	-	-	-	-	190597	-	-	-	-	-	-	-	-	-	-	-	-	-	-	-	-	-	-	-	-	-	-	-	-	-	-	-	-	-	-	-	-	-	-	-	-	-	-
		-	-	-	-	-	-	-	-	-	-	-	-	190596	-	-	-	-	-	-	-	-	-	-	-	-	-	-	-	-	-	-	-	-	-	-	-	-	-	-	-	-	-	-	-	-	-	-	-	-	-	-
		-	-	-	-	-	-	-	-	-	-	-	-	190591	-	-	-	-	-	-	-	-	-	-	-	-	-	-	-	-	-	-	-	-	-	-	-	-	-	-	-	-	-	-	-	-	-	-	-	-	-	-
		-	+	-	-	-	-	-	AT5G26610.2	-	BDEG_06498	CE28713	115905	190589	-	-	-	-	-	-	-	-	ENSGALP00000039929	-	OTTHUMP00000181876	-	-	25219	-	126194	NCU09973	Os04g02500.2	-	-	-	114282	139255	-	570654	RO3G_12147.1	-	-	-	SINFRUP00000138167	-	-	-	-	60856	-	-	-
		-	-	-	-	-	-	-	-	-	-	-	-	190587	-	-	-	-	-	-	-	-	-	-	-	-	154773	-	-	-	-	-	-	-	-	-	-	-	-	-	-	-	-	-	-	-	-	-	-	-	-	-	-
		-	-	-	-	-	-	-	-	-	-	-	-	190586	-	-	-	-	-	-	-	-	-	-	-	-	-	-	-	-	-	-	-	-	-	-	-	-	-	-	-	-	-	-	-	-	-	-	-	-	-	-
		-	-	-	-	-	-	-	-	-	-	-	-	190584	-	-	-	-	-	-	-	-	-	-	-	-	-	-	-	-	-	-	-	-	-	-	-	-	-	-	-	-	-	-	-	-	-	-	-	-	-	-
		-	-	-	-	-	-	-	-	-	-	-	-	190583	-	-	-	-	-	-	-	-	-	-	-	-	-	-	-	-	-	-	-	-	-	-	-	-	-	-	-	-	-	-	-	-	-	-	-	-	-	-
		-	-	-	-	-	-	-	-	-	-	-	-	190581	-	-	-	-	-	-	-	-	-	-	-	-	-	-	-	-	-	-	-	-	-	-	-	-	-	-	-	-	-	-	-	-	-	-	-	-	-	-
		-	-	-	-	-	-	-	-	-	-	-	-	190580	-	-	-	-	-	-	-	-	-	-	-	-	-	-	-	-	-	-	-	-	-	-	-	-	-	-	-	-	-	-	-	-	-	-	-	-	-	-
		-	+	-	-	-	-	GB18289-PA	AT1G78650.1	-	-	-	-	190579	-	-	-	-	-	-	-	-	ENSGALP00000027913	-	NP_006582.1	-	231845	23443	-	218204	-	Os01g10690.1	-	-	-	-	-	-	585498	-	-	-	-	-	-	-	-	-	52194	-	-	-
		-	-	-	-	-	-	-	-	-	-	-	-	190578	-	-	-	-	-	-	-	-	-	-	-	-	-	-	-	-	-	-	-	-	-	-	-	-	-	-	-	-	-	-	-	-	-	-	-	-	-	-
		-	-	-	-	-	-	-	-	-	-	-	-	190575	-	-	-	-	-	-	-	-	-	-	-	-	-	-	-	-	-	-	-	-	-	-	-	-	-	-	-	-	-	-	-	-	-	-	-	-	-	-
		-	-	-	-	-	-	-	-	-	-	-	-	190570	-	-	-	-	-	-	-	-	-	-	-	-	-	-	-	-	-	-	-	-	-	-	-	-	-	-	-	-	-	-	-	-	-	-	-	-	-	-
		-	-	-	-	-	-	-	-	-	-	-	-	190566	-	-	-	-	-	-	-	-	-	-	-	-	-	-	-	-	-	-	-	-	-	-	-	-	-	-	-	-	-	-	-	-	-	-	-	-	-	-
		-	+	-	-	-	-	GB13403-PA	AT3G17611.1	-	-	-	180450	190549	369221	-	-	-	-	-	-	-	ENSGALP00000007935	-	OTTHUMP00000164233	-	207912	-	-	248483	-	Os01g18100.1	-	-	-	205013	130205	-	657794	-	-	-	-	SINFRUP00000181829	-	-	-	-	53891	-	-	-
FAP278	Flagellar Associated Protein Similar to Adenosine Kinase	-	+	-	-	-	-	GB13063-PA	AT3G09820.1	52969	BDEG_00378	CE12564	170886	190547	-	cgd8_2370	CMK016C	OTTDARP00000021413	DDB0186795	CG11255-PB	-	-	ENSGALP00000008095	-	OTTHUMP00000019865	LmjF30.0880	210552	39088	58850	163941	NCU00414	Os04g43750.1	8807	GSPATP00023892001	51242	184505	108994	-	659896	RO3G_06141.1	YJR105W	SPCC338.14	GLEAN3_25307	SINFRUP00000164197	-	644	-	50.m00018	50549	-	Tb927.6.2300	UM00797.1
		-	-	-	-	-	-	-	-	-	-	-	-	190545	-	-	-	-	-	-	-	-	-	-	-	-	-	-	-	-	-	-	-	-	-	-	-	-	-	-	-	-	-	-	-	-	-	-	-	-	-	-
NUOB4	NADH:ubiquinone oxidoreductase (Complex I) 13 kDA subunit, homolog of bovine B15 subunit, mitochondrial	-	-	-	-	-	-	-	-	-	-	-	-	190543	-	-	-	-	-	-	-	-	-	-	-	-	-	-	-	-	-	-	-	-	-	-	-	-	-	-	-	-	-	-	-	-	-	-	-	-	-	-
		-	+	-	-	-	-	-	AT3G51520.1	-	-	-	-	190539	-	-	-	-	-	-	-	-	-	-	-	LmjF27.1590	-	-	-	-	-	Os02g48350.1	9124	-	-	145230	-	-	232754	-	-	-	-	-	62.m00172	37867	-	-	-	-	Tb11.18.0008	-
		-	-	-	-	-	-	-	-	-	-	-	-	190538	-	-	-	-	-	-	-	-	-	-	-	-	-	-	-	-	-	-	-	-	-	-	-	-	-	-	-	-	-	-	-	-	-	-	-	-	-	-
		-	-	-	-	-	-	-	-	-	BDEG_05346	-	-	190532	-	-	-	-	-	-	-	-	-	-	-	-	-	-	-	-	-	-	-	-	-	-	-	-	-	-	-	-	-	-	-	-	-	-	-	-	-	-
		-	-	-	-	-	-	-	-	-	-	-	-	190524	-	-	-	-	-	-	-	-	-	-	-	-	-	-	-	-	-	-	-	-	-	-	-	-	-	-	-	-	-	-	-	-	-	-	-	-	-	-
		-	-	-	-	-	-	-	-	-	-	-	-	190523	-	-	-	-	-	-	-	-	-	-	-	-	-	-	-	-	-	-	-	-	-	232071	-	-	-	-	-	-	-	-	-	-	-	-	-	-	-	-
		-	-	-	-	-	-	-	-	-	-	-	-	190521	-	-	-	-	-	-	-	-	-	-	-	-	-	-	-	-	-	-	-	-	-	-	-	-	-	-	-	-	-	-	-	-	-	-	-	-	-	-
		-	-	-	-	-	-	-	-	-	-	-	-	190520	-	-	-	-	-	-	-	-	-	-	-	-	-	-	-	-	-	-	-	-	-	-	-	-	-	-	-	-	-	-	-	-	-	-	-	-	-	-
		-	-	-	-	-	-	-	AT3G46450.2	-	-	-	-	190517	-	-	-	-	-	-	-	-	-	-	-	-	-	-	-	-	-	Os03g60610.3	-	-	-	121003	-	-	172756	-	-	-	-	-	-	-	-	-	-	-	-	-
		-	-	-	-	-	-	-	-	-	-	-	-	190514	-	-	-	-	-	-	-	-	-	-	-	-	-	-	-	-	-	-	35494	-	-	-	-	-	-	-	-	-	-	-	-	-	-	-	-	-	-	-
		-	-	-	-	-	-	-	-	-	-	-	-	190513	-	-	-	-	-	-	-	-	-	-	-	-	-	-	-	-	-	-	-	-	-	-	-	-	-	-	-	-	-	-	-	-	-	-	-	-	-	-
		-	-	-	-	-	-	-	-	-	-	-	-	190511	-	-	-	-	-	-	-	-	-	-	-	-	-	-	-	-	-	-	-	-	-	-	-	-	-	-	-	-	-	-	-	-	-	-	-	-	-	-
		-	+	-	-	-	-	-	-	19728	-	-	91755	190510	299001	-	CMQ301C	-	DDB0203635	-	-	-	-	-	-	-	159773	37421	-	-	NCU11179	Os09g23540.1	22199	-	-	-	109686	-	735178	-	YMR318C	-	-	-	-	-	-	-	60455	-	-	-
		-	-	-	-	-	-	-	-	-	-	-	-	190507	-	-	-	-	-	-	-	-	-	-	-	-	-	-	-	-	-	-	-	-	-	-	-	-	-	-	-	-	-	-	-	-	-	-	-	-	-	-
		-	+	-	-	-	-	GB18719-PA	AT4G37990.1	-	-	-	-	190503	-	cgd5_2670	-	-	-	-	-	-	-	-	-	LmjF23.0360	-	-	-	-	NCU04823	-	-	GSPATP00037594001	-	87951	109585	-	-	-	-	-	-	-	-	-	-	-	-	-	-	-	UM01861.1
		-	-	-	-	-	-	-	-	-	-	-	-	190502	-	-	-	-	-	-	-	-	-	-	-	-	-	-	-	-	-	-	-	-	-	-	-	-	-	-	-	-	-	-	-	-	-	-	-	-	-	-
		-	-	-	-	-	-	-	-	-	-	-	-	190500	-	-	-	-	-	-	-	-	-	-	-	-	-	-	-	-	-	-	35273	-	-	-	-	-	-	-	-	-	-	-	-	-	-	-	-	-	-	-
		-	-	-	-	-	-	-	-	-	-	-	-	190493	-	-	-	-	-	-	-	-	-	-	-	-	-	-	-	-	-	-	-	-	-	-	-	-	-	-	-	-	-	-	-	-	-	-	-	-	-	-
		-	-	-	-	-	-	-	-	-	-	-	-	190492	-	-	-	-	-	-	-	-	-	-	-	-	-	-	-	-	-	-	-	-	-	-	-	-	-	-	-	-	-	-	-	-	-	-	-	-	-	-
		-	-	-	-	-	-	-	-	-	-	-	-	190489	-	-	-	OTTDARP00000010320	-	-	-	-	-	-	-	-	-	-	-	-	-	-	-	-	-	-	-	-	-	-	-	-	-	-	-	-	-	-	-	-	-	-	-	-
		-	-	-	-	-	-	-	-	-	-	-	-	190484	-	-	-	-	-	-	-	-	-	-	-	-	-	-	-	-	-	-	-	-	-	-	-	-	-	-	-	-	-	-	-	-	-	-	-	-	-	-
		-	-	-	-	-	-	-	AT5G48340.1	-	-	-	-	190480	-	-	-	-	-	-	-	-	-	-	-	-	-	-	-	-	-	Os01g67170.1	-	-	-	86072	-	-	807710	-	-	-	-	-	-	-	-	-	-	-	-	-
		-	-	-	-	-	-	-	-	-	-	-	-	190469	-	-	-	-	-	-	-	-	-	-	-	-	-	-	-	-	-	-	-	-	-	-	-	-	-	-	-	-	-	-	-	-	-	-	-	-	-	-
		-	-	-	-	-	-	-	-	-	-	-	-	190468	-	-	-	-	-	-	-	-	-	-	-	-	-	-	-	-	-	-	-	-	-	-	-	-	-	-	-	-	-	-	-	-	-	-	-	-	-	-
		-	-	-	-	-	-	-	-	-	-	-	-	190461	-	-	-	-	-	-	-	-	-	-	-	-	-	-	-	-	-	-	-	-	-	-	-	-	-	-	-	-	-	-	-	-	-	-	-	-	-	-
RPOD	Chloroplast RNA polymerase sigma factor, chloroplast precursor	-	+	-	-	-	-	-	AT1G08540.1	53467	-	-	-	190458	-	-	CMK044C	-	-	-	-	-	-	-	-	-	-	-	-	-	-	Os11g26160.1	18021	-	17029	206687	-	PFF1015w	780378	-	-	-	-	-	-	33682	-	-	-	-	-	-
		-	-	-	-	-	-	-	-	-	-	-	-	190457	-	-	-	-	-	-	-	-	-	-	-	-	-	-	-	-	-	-	-	-	-	-	-	-	-	-	-	-	-	-	-	-	-	-	-	-	-	-
		-	-	-	-	-	-	-	-	-	-	-	-	190456	-	-	-	-	-	-	-	-	-	-	-	-	-	-	-	-	-	-	-	-	-	-	-	-	-	-	-	-	-	-	-	-	-	-	-	-	-	-
MDH6	putative NAD-dependent malate dehydrogenase	-	+	-	-	-	-	GB16464-PA	AT3G47520.1	-	-	CE09512	159451	190455	205744	cgd7_470	CMC188C	OTTDARP00000007888	-	-	-	-	ENSGALP00000003012	-	OTTHUMP00000161017	LmjF34.0140	225558	38523	-	248519	-	Os08g33720.1	8225	-	-	118897	109054	-	583880	RO3G_08319.1	-	-	GLEAN3_18624	SINFRUP00000136061	-	-	TA09590	641.m00168	34967	-	Tb10.70.5110	-
		-	-	-	-	-	-	-	-	-	-	-	-	190453	-	-	-	-	-	-	-	-	-	-	-	-	-	-	-	-	-	-	-	-	-	-	-	-	-	-	-	-	-	-	-	-	-	-	-	-	-	-
		-	-	-	-	-	-	-	-	-	-	-	-	190451	-	-	-	-	-	-	-	-	-	-	-	-	-	-	-	-	-	-	-	-	-	-	-	-	-	-	-	-	-	-	-	-	-	-	-	-	-	-
		-	-	-	-	-	-	-	-	-	-	-	-	190450	-	-	-	-	-	-	-	-	-	-	-	-	-	-	-	-	-	-	-	-	-	-	-	-	-	-	-	-	-	-	-	-	-	-	-	-	-	-
		-	+	-	-	-	-	-	AT4G27500.1	-	BDEG_02693	-	-	190448	-	-	CMA031C	-	-	-	-	-	-	-	-	-	236795	-	-	-	NCU05304	Os09g17730.3	-	-	-	172429	136356	-	579456	RO3G_13926.1	-	-	-	-	-	-	-	-	58379	-	Tb10.61.2670	UM00851.1
		-	+	-	-	-	-	GB16138-PA	AT4G17510.1	24113	BDEG_03761	CE20818	181658	190447	249685	cgd8_3200	CMQ026C	OTTDARP00000022363	DDB0205083	CG4265-PA	-	-	ENSGALP00000027305	-	OTTHUMP00000018509	LmjF25.0190	204019	35021	75524	107252	NCU06372	Os02g43760.1	22951	GSPATP00036561001	41928	232845	158844	PF14_0576	817181	RO3G_07474.1	YJR099W	SPAC27F1.03c	GLEAN3_25961	SINFRUP00000143073	141.m00107	263931	-	55.m05062	56125	87079.m00075	Tb11.03.0890	UM01905.1
PDH2	pyruvate dehydrogenase, E1 beta subunit, plastid precursor	-	+	-	-	-	-	-	AT1G30120.1	-	-	-	-	190446	-	-	CMV154C	-	-	-	-	-	-	-	-	-	-	-	-	-	-	Os12g42230.1	-	-	-	106829	-	PF14_0441	668506	-	-	-	-	-	-	-	-	59.m03618	-	-	-	-
FTRC	Ferredoxin Thioredoxin Reductase, catalytic chain, chloroplast precursor	-	+	-	-	-	-	-	AT2G04700.1	27097	-	-	-	190440	-	-	CMV226C	-	-	-	-	-	-	-	-	-	-	-	-	-	-	Os09g07570.1	29639	-	12171	175508	-	-	173755	-	-	-	-	-	-	37118	-	-	-	-	-	-
		-	+	-	-	-	-	-	AT3G61680.1	-	-	-	-	190433	-	-	-	OTTDARP00000022679	-	-	-	38.m00238	-	-	-	-	-	-	-	-	-	Os01g15000.2	36354	-	37579	42339	-	-	830453	-	-	-	-	-	-	-	-	-	-	-	-	UM05502.1
		-	+	-	-	-	-	GB14989-PA	AT5G01660.1	-	-	CE39523	172031	190424	262881	-	-	OTTDARP00000006884	-	CG3571-PB	-	-	ENSGALP00000003437	-	NP_277030.2	-	177864	-	70995	88501	-	-	23674	GSPATP00024750001	49881	65128	-	PF13_0238	197217	-	-	-	GLEAN3_03923	SINFRUP00000157952	11.m00532	5851	TA20610	55.m04978	50396	-	-	-
		-	-	-	-	-	-	-	-	-	-	-	-	190422	-	-	-	-	-	-	-	-	-	-	-	-	-	-	-	-	-	-	-	-	-	-	-	-	-	-	-	-	-	-	-	-	-	-	-	-	-	-
		-	-	-	-	-	-	-	-	64617	-	-	-	190417	-	-	-	-	-	-	-	-	-	-	-	-	-	-	-	-	-	-	32739	-	38958	-	-	-	-	-	-	-	-	-	-	23738	-	-	-	-	-	-
COPE1	Epsilon-COP	-	+	-	-	-	-	GB10258-PA	AT1G30630.1	28840	BDEG_06608	CE16047	225244	190416	289097	-	CMD014C	-	DDB0189670	CG9543-PA	-	-	ENSGALP00000004982	-	OTTHUMP00000067837	-	157712	18975	30177	227384	NCU11274	Os04g52270.1	23457	-	19093	175776	109221	-	823128	RO3G_12155.1	-	-	GLEAN3_19012	SINFRUP00000157001	-	39913	-	49.m00062	37307	91566.m00127	Tb11.01.6530	UM01353.1
LCI21	low-CO2-inducible protein	-	-	-	-	-	-	-	-	-	-	-	-	190415	-	-	-	-	-	-	-	-	-	-	-	-	-	-	-	-	-	-	-	-	-	-	-	-	-	-	-	-	-	-	-	-	-	-	-	-	-	-
		-	-	-	-	-	-	-	-	-	-	-	-	190414	-	-	-	-	-	-	-	-	-	-	-	-	-	-	-	-	-	-	-	-	-	-	-	-	-	-	-	-	-	-	-	-	-	-	-	-	-	-
		-	-	-	-	-	-	-	-	-	-	-	-	190412	-	-	-	-	-	-	-	-	-	-	-	-	-	-	-	-	-	-	-	-	-	-	-	-	-	-	-	-	-	-	-	-	-	-	-	-	-	-
DAL1	allantoinase	-	+	+	-	-	-	-	AT4G04955.1	14125	-	-	19397	190406	-	-	-	-	DDB0205262	CG6106-PA	-	189.m00093	-	-	-	LmjF16.0580	-	-	-	158591	NCU02296	Os04g58390.1	37567	GSPATP00020161001	35101	161879	-	-	831124	RO3G_06384.1	YIR027C	-	GLEAN3_09429	SINFRUP00000173969	11.m00504	-	-	-	-	-	Tb927.8.5630	-
		-	-	-	-	-	-	-	-	-	-	-	-	190404	-	-	-	-	-	-	-	-	-	-	-	-	-	-	-	-	-	-	-	-	-	-	-	-	-	-	-	-	-	-	-	-	-	-	-	-	-	-
		-	+	-	-	-	-	GB13061-PA	-	-	-	-	92244	190403	372973	-	-	-	-	CG12314-PA	-	-	-	-	OTTHUMP00000065516	-	155267	9955	-	131996	-	-	-	GSPATP00008523001	-	-	127295	-	-	RO3G_03210.1	-	-	-	SINFRUP00000161664	1190.m00002	-	-	-	-	-	-	-
		-	-	-	-	-	-	-	-	-	-	-	-	190400	-	-	-	-	-	-	-	-	-	-	-	-	-	-	-	-	-	-	-	-	-	-	-	-	-	-	-	-	-	-	-	-	-	-	-	-	-	-
		-	-	-	-	-	-	-	-	-	-	-	-	190399	-	-	-	-	-	-	-	-	-	-	-	-	-	-	-	-	-	-	-	-	-	-	-	-	-	-	-	-	-	-	-	-	-	-	-	-	-	-
ADCY36	adenylate cyclase	-	-	-	-	-	-	-	-	-	-	-	-	190398	-	-	-	-	-	-	-	-	-	-	-	-	-	-	-	-	-	-	-	GSPATP00008009001	-	-	-	-	-	RO3G_15565.1	-	-	-	-	-	-	-	-	-	-	-	-
		-	-	-	-	-	-	-	-	-	-	-	-	190396	-	-	-	-	-	-	-	-	-	-	-	-	-	-	-	-	-	-	-	-	-	-	-	-	-	-	-	-	-	-	-	-	-	-	-	-	-	-
		-	-	-	-	-	-	-	-	-	-	-	-	190395	-	-	-	-	-	-	-	-	-	-	-	-	-	-	-	-	-	-	-	-	-	-	-	-	-	-	-	-	-	-	-	-	-	-	-	-	-	-
		-	-	-	-	-	-	-	-	-	-	-	-	190393	-	-	-	-	-	-	-	-	-	-	-	-	-	-	-	-	-	-	-	-	-	-	-	-	-	-	-	-	-	-	-	-	-	-	-	-	-	-
		-	-	-	-	-	-	-	-	-	-	-	-	190392	-	-	-	-	-	-	-	-	-	-	-	-	-	-	-	-	-	-	-	-	-	-	-	-	-	-	-	-	-	-	-	-	-	-	-	-	-	-
		-	-	-	-	-	-	-	-	-	-	-	-	190390	-	-	-	-	-	-	-	-	-	-	-	-	-	-	-	-	-	-	-	-	-	-	-	-	-	-	-	-	-	-	-	-	-	-	-	-	-	-
		-	-	-	-	-	-	-	-	-	-	-	-	190388	-	-	-	-	-	-	-	-	-	-	-	-	-	-	-	-	-	-	-	-	-	-	-	-	-	-	-	-	-	-	-	-	-	-	-	-	-	-
MRP2	ABC transporter, Multidrug resistance associated protein, membrane protein	-	-	-	-	-	-	-	-	-	BDEG_06396	CE39102	-	190380	-	-	-	-	-	-	-	-	-	-	-	-	-	-	-	-	-	-	-	-	-	-	-	-	-	-	-	-	-	-	-	-	-	-	-	-	-	-
		-	-	-	-	-	-	-	-	-	-	-	-	190379	-	-	-	-	-	-	-	-	-	-	-	-	-	-	-	-	-	-	-	-	-	-	-	-	-	-	-	-	-	-	-	-	-	-	-	-	-	-
ATPvE		-	+	+	-	-	-	GB12913-PA	AT4G11150.1	19413	BDEG_04695	CE19362	166040	190371	231187	cgd8_360	CMQ010C	OTTDARP00000023512	DDB0167361	CG1088-PA	19170784	9.m00371	ENSGALP00000021250	GL50803_13603	OTTHUMP00000065606	LmjF36.3100	209321	37555	71627	237075	NCU07446	Os01g46980.1	8689	GSPATP00027382001	29711	223748	138060	PFI1670c	825432	RO3G_17130.1	YOR332W	SPAC11E3.07	GLEAN3_09588	SINFRUP00000130799	16.m00305	29117	TA17420	541.m02058	63784	83695.m00101	Tb11.01.1190	UM03951.1
PRPL19	Plastid ribosomal protein L19, imported to chloroplast, large ribosomal subunit	-	+	-	-	-	-	-	-	17219	-	-	-	190369	-	-	CMV108C	-	-	-	-	-	-	-	-	-	-	-	-	-	-	Os02g43600.1	22601	-	9259	-	141238	-	584247	RO3G_09417.1	-	-	-	SINFRUP00000135681	-	-	-	-	56974	-	-	UM06394.1
		-	-	-	-	-	-	-	-	-	-	-	-	190364	-	-	-	-	-	-	-	-	-	-	-	-	-	-	-	-	-	-	-	-	-	-	-	-	-	-	-	-	-	-	-	-	-	-	-	-	-	-
		-	-	-	-	-	-	-	-	-	-	-	-	190361	-	-	-	-	-	-	-	-	-	-	-	-	-	-	-	-	-	-	4289	-	-	161819	-	-	-	-	-	-	-	-	-	-	-	-	-	-	-	-
		-	-	-	-	-	-	-	-	-	-	-	-	190358	-	-	-	-	-	-	-	-	-	-	-	-	-	-	-	-	-	-	-	-	-	-	-	-	-	-	-	-	-	-	-	-	-	-	-	-	-	-
		-	-	-	-	-	-	-	-	-	-	-	-	190351	-	-	-	-	-	-	-	-	-	-	-	-	-	-	-	-	-	-	-	-	-	-	-	-	-	-	-	-	-	-	-	-	-	-	-	-	-	-
		-	-	-	-	-	-	-	-	-	-	-	-	190349	-	-	-	-	-	-	-	-	-	-	-	-	-	-	-	-	-	-	-	-	-	-	-	-	-	-	-	-	-	-	-	-	-	-	-	-	-	-
	flavoprotein monooxygenase	-	-	-	-	-	-	-	-	-	-	-	-	190341	-	-	-	-	-	-	-	-	-	-	-	-	-	-	-	-	NCU08315	-	-	-	-	-	-	-	-	-	-	-	-	-	-	-	-	-	-	-	-	UM05230.1
		-	-	-	-	-	-	-	-	-	-	-	-	190339	-	-	-	-	-	-	-	-	-	-	-	-	-	-	-	-	-	-	-	-	-	-	-	-	-	-	-	-	-	-	-	-	-	-	-	-	-	-
		-	-	-	-	-	-	-	-	-	-	-	-	190337	-	-	-	-	-	-	-	-	-	-	-	-	-	-	-	-	-	-	34649	-	-	-	-	-	-	-	-	-	-	-	-	-	-	-	-	-	-	-
		-	-	-	-	-	-	-	-	-	-	-	-	190332	-	-	-	-	-	-	-	-	-	-	-	-	-	-	-	-	-	-	-	-	-	-	-	-	-	-	-	-	-	-	-	-	-	-	-	-	-	-
		-	-	-	-	-	-	-	-	-	-	-	-	190330	-	-	-	-	-	-	-	-	-	-	-	-	-	-	-	-	-	-	-	-	-	-	-	-	-	-	-	-	-	-	-	-	-	-	-	-	-	-
		-	-	-	-	-	-	-	-	-	-	-	-	190325	-	-	-	-	-	-	-	-	-	-	-	-	-	-	-	-	-	-	-	-	-	-	-	-	-	-	-	-	-	-	-	-	-	-	-	-	-	-
		-	-	-	-	-	-	-	-	-	-	-	-	190324	-	-	-	-	-	-	-	-	-	-	-	-	-	-	-	-	-	-	-	-	-	-	-	-	-	-	-	-	-	-	-	-	-	-	-	-	-	-
		-	-	-	-	-	-	-	-	-	-	-	-	190323	-	-	-	-	-	-	-	-	-	-	-	-	-	-	-	-	-	-	-	-	-	-	-	-	-	-	-	-	-	-	-	-	-	-	-	-	-	-
		-	-	-	-	-	-	-	-	-	-	-	-	190320	-	-	-	-	-	-	-	-	-	-	-	-	-	-	-	-	-	-	-	-	-	-	-	-	-	-	-	-	-	-	-	-	-	-	-	-	-	-
		-	-	-	-	-	-	-	-	-	-	-	-	190319	-	-	-	-	-	-	-	-	-	-	-	-	-	-	-	-	-	-	-	-	-	-	-	-	-	-	-	-	-	-	-	-	-	-	-	-	-	-
		-	-	-	-	-	-	-	-	64600	-	-	-	190316	-	-	-	-	-	-	-	-	-	-	-	-	-	-	-	-	-	-	12628	-	-	-	-	-	-	-	-	-	-	-	-	23.m00359	-	-	-	-	-	-	-
		-	-	-	-	-	-	-	-	-	-	-	-	190315	-	-	CMS163C	-	-	-	-	-	-	-	-	-	-	-	-	-	-	-	-	-	-	-	-	PF14_0285	-	-	-	-	-	-	-	-	-	-	-	-	-	-	-
		-	-	-	-	-	-	-	-	-	-	-	-	190314	-	-	-	-	-	-	-	-	-	-	-	-	-	-	-	-	-	-	-	-	-	-	-	-	-	-	-	-	-	-	-	-	-	-	-	-	-	-
		-	+	-	-	-	-	-	AT2G21530.1	-	-	-	-	190311	-	-	-	-	-	-	-	-	-	GL50803_14347	-	-	143337	-	-	-	-	Os12g03070.2	34238	GSPATP00010748001	-	160333	-	-	821007	-	-	-	-	-	-	-	TA13320	-	-	-	-	-
		-	-	-	-	-	-	-	-	-	-	-	-	190305	-	-	-	-	-	-	-	-	-	-	-	-	-	-	-	-	-	-	-	-	-	-	-	-	-	-	-	-	-	-	-	-	-	-	-	-	-	-
		-	-	-	-	-	-	-	-	-	-	-	-	190302	-	-	-	-	-	-	-	-	-	-	-	-	-	-	-	-	-	-	-	-	-	-	-	-	-	-	-	-	-	-	-	-	-	-	-	-	-	-
		-	-	-	-	-	-	-	-	-	-	-	-	190299	-	-	-	-	-	-	-	-	-	-	-	-	-	-	-	-	-	-	-	-	-	-	-	-	-	-	-	-	-	-	-	-	-	-	-	-	-	-
		-	-	-	-	-	-	-	-	64738	-	-	-	190298	-	-	-	-	-	-	-	-	-	-	-	LmjF32.2480	-	-	-	-	-	-	-	-	-	-	158884	-	-	-	-	-	-	-	-	-	-	-	-	-	-	Tb11.01.7325	-
ARFE1	ARFE1, small Arf-related GTPase	-	+	-	-	-	-	GB15863-PA	AT2G24765.1	60231	BDEG_06231	CE02250	228707	190294	246537	-	-	-	-	CG6025-PA	-	175.m00105	ENSGALP00000019017	-	NP_001168.1	LmjF17.0070	228225	35363	60636	236810	NCU08989	Os08g15040.1	-	GSPATP00023591001	8659	108476	108343	PF10_0337	837483	-	-	-	-	SINFRUP00000139126	77.m00234	37988	-	55.m05015	51633	-	Tb927.7.6230	UM02680.1
ALA2	putative ATPase, aminophospholipid transporter	-	+	+	-	-	-	-	AT1G59820.1	53224	BDEG_06141	CE36241	176761	190292	249913	cgd3_2730	CMR306C	OTTDARP00000016204	DDB0190219	CG17034-PD	19074343	309.m00046	ENSGALP00000027589	-	NP_057613.4	-	225116	-	31746	10129	NCU00352	Os06g29380.1	17724	GSPATP00032129001	52368	205967	133459	PFL0950c	230718	RO3G_04222.1	YAL026C	SPBC887.12	GLEAN3_19141	SINFRUP00000182510	27.m00246	26702	-	50.m03192	25047	83472.m00461	-	UM04394.1
FAP126	Flagellar Associated Protein	-	-	-	-	-	-	-	-	-	-	-	-	-	-	-	-	-	-	-	-	-	-	-	-	-	-	-	-	-	-	-	-	-	-	-	-	-	-	-	-	-	-	-	-	-	-	-	-	-	-	-
		-	-	-	-	-	-	-	-	-	-	-	-	190290	-	-	-	-	-	-	-	-	-	-	-	-	-	-	-	-	-	-	-	-	-	-	-	-	-	-	-	-	-	-	-	-	-	-	-	-	-	-
		-	-	-	-	-	-	-	-	-	-	-	-	190288	-	-	-	-	-	-	-	-	-	-	-	-	-	-	-	-	-	-	-	-	-	164204	-	-	-	-	-	-	-	-	-	-	-	-	-	-	-	-
		-	-	-	-	-	-	-	-	-	-	-	-	190284	-	-	-	-	-	-	-	-	-	-	-	-	-	-	-	-	-	-	-	-	-	-	-	-	-	-	-	-	-	-	-	-	-	-	-	-	-	-
		-	-	-	-	-	-	-	-	-	-	-	-	190283	-	-	-	-	-	-	-	-	-	-	-	-	-	-	-	-	-	-	-	-	-	-	-	-	-	-	-	-	-	-	-	-	-	-	-	-	-	-
		-	+	-	-	-	-	-	AT1G65230.1	-	-	-	-	190282	-	-	-	-	-	-	-	-	-	-	-	-	-	-	-	-	-	Os01g68450.1	35649	-	-	132914	-	-	596964	-	-	-	-	-	-	-	-	-	-	-	-	-
		-	-	-	-	-	-	-	AT4G02980.1	-	-	-	-	190281	-	-	-	-	-	-	-	-	-	-	-	-	-	-	-	-	-	-	-	-	-	-	-	-	275956	-	-	-	-	-	-	-	-	-	-	-	-	-
		-	+	-	-	-	-	GB15142-PA	AT1G12640.1	-	-	CE04270	24585	190274	212415	-	-	OTTDARP00000010894	DDB0205522	CG18445-PA	-	149.m00109	ENSGALP00000023450	-	NP_005759.4	-	129765	-	33112	106212	NCU06194	Os02g45344.1	22506	-	20460	108531	128898	-	815112	RO3G_15694.1	YOR175C	SPBC16A3.10	-	SINFRUP00000177590	209.m00059	31566	-	-	-	-	-	UM01407.1
RPL7	Ribosomal protein L7, component of cytosolic 80S ribosome and 60S large subunit	-	+	+	-	-	-	GB10094-PA	AT2G44120.2	52193	BDEG_04918	CE11024	221278	190273	212434	cgd8_4350	CMO310C	OTTDARP00000005494	DDB0217817	CG4897-PA	19168720	350.m00050	ENSGALP00000025165	GL50803_19436	OTTHUMP00000166004	LmjF26.0170	221338	12901	36087	245394	NCU07829	Os08g13690.1	22760	GSPATP00037788001	22774	158886	108358	PFC0300c	831861	RO3G_16080.1	YGL076C	SPAC3H5.07	-	SINFRUP00000133890	115.m00079	27167	TA18030	583.m00012	36785	82511.m00152	Tb927.7.1740	UM03578.1
		-	-	-	-	-	-	-	-	-	-	-	-	190269	-	-	-	-	-	-	-	-	-	-	-	-	-	-	-	-	-	-	-	-	-	-	-	-	-	-	-	-	-	-	-	-	-	-	-	-	-	-
		-	-	-	-	-	-	-	-	-	-	-	-	190262	-	-	-	-	-	-	-	-	-	-	-	-	-	-	-	-	-	-	14295	-	-	-	-	-	-	-	-	-	-	-	-	-	-	-	-	-	-	-
		-	-	-	-	-	-	-	AT5G49830.1	-	-	-	-	190258	-	-	-	-	-	-	-	-	-	-	-	-	-	-	-	-	-	-	-	-	-	131734	-	-	-	-	-	-	-	-	-	-	-	-	-	-	-	-
		-	+	-	-	-	-	GB16659-PA	AT5G51410.2	-	BDEG_05269	CE34769	219978	190244	391361	cgd6_2550	-	-	DDB0206200	CG7564-PA	19069335	-	ENSGALP00000037522	-	OTTHUMP00000181675	-	56896	-	69221	137810	NCU05645	Os07g27300.3	24477	GSPATP00024772001	46402	170579	109009	PF14_0502	836384	RO3G_01896.1	YDL087C	SPCC16A11.13	GLEAN3_23529	SINFRUP00000170843	73.m00126	20799	TA13605	55.m00236	20548	81312.m00093	-	UM02646.1
		-	-	-	-	-	-	-	-	-	-	-	-	190241	-	-	-	-	-	-	-	-	-	-	-	-	-	-	-	-	-	-	-	-	-	-	-	-	-	-	-	-	-	-	-	-	-	-	-	-	-	-
		-	-	-	-	-	-	-	-	-	-	-	-	190235	-	-	-	-	-	-	-	-	-	-	-	-	-	-	-	-	-	Os04g32790.1	-	-	-	-	-	-	657483	-	-	-	-	-	-	-	-	-	-	-	-	-
SPC22	Signal Peptidase, 22 kDa subunit	-	+	+	-	-	-	GB14961-PA	AT5G27430.1	18385	BDEG_05900	CE00269	225712	190234	266289	cgd3_2680	CMO086C	-	DDB0189116	-	19171344	147.m00114	ENSGALP00000037390	-	NP_068747.1	-	-	37484	64781	94933	NCU06677	Os09g38370.1	36276	GSPATP00031627001	18533	110600	135382	PFI0215c	819523	-	YLR066W	SPAC56F8.11	-	SINFRUP00000149361	276.m00027	9852	TA08609	-	25476	95978.m00064	Tb927.5.1930	-
		-	-	-	-	-	-	-	-	-	-	-	-	190231	-	-	-	-	-	-	-	-	-	-	-	-	-	-	-	-	-	-	-	-	-	-	-	-	-	-	-	-	-	-	-	-	-	-	-	-	-	-
		-	-	-	-	-	-	-	AT3G17590.1	-	-	-	-	190230	-	-	-	-	-	-	-	-	-	-	-	-	-	-	-	-	-	Os02g49190.1	-	-	-	-	-	-	725577	-	-	-	-	-	29.m00182	-	-	-	-	-	-	-
		-	-	-	-	-	-	-	AT1G49970.1	-	-	-	-	190221	-	-	-	-	-	-	-	-	-	-	-	-	-	-	-	-	-	Os05g51450.1	6222	-	-	-	-	-	821289	-	-	-	-	-	-	-	-	-	-	-	-	-
		-	+	-	-	-	-	GB14950-PA	-	64211	-	CE18133	118653	190219	270387	-	-	OTTDARP00000001555	DDB0185430	CG15097-PB	-	-	ENSGALP00000015537	-	OTTHUMP00000163060	-	211219	-	68705	230546	-	Os01g70670.2	-	-	-	30287	-	-	-	-	-	-	GLEAN3_28676	SINFRUP00000132577	-	23483	-	-	-	83363.m00082	-	-
		-	-	-	-	-	-	-	-	-	-	-	-	190218	-	-	-	-	-	-	-	-	-	-	-	-	-	-	-	-	-	-	-	-	-	-	-	-	-	-	-	-	-	-	-	-	-	-	-	-	-	-
		-	-	-	-	-	-	-	-	-	-	-	-	190217	-	-	-	-	-	-	-	-	-	-	-	-	-	-	-	-	-	-	-	-	-	-	-	-	-	-	-	-	-	-	-	-	-	-	-	-	-	-
		-	-	-	-	-	-	-	-	-	-	-	-	190215	-	-	-	-	-	-	-	-	-	-	-	-	-	-	-	-	-	-	-	-	-	-	-	-	-	-	-	-	-	-	-	-	-	-	-	-	-	-

		-	-	-	-	-	-	-	-	-	-	-	-	190212	-	-	-	-	-	-	-	-	-	-	-	-	-	-	-	-	-	-	-	-	-	-	-	-	-	-	-	-	-	-	-	-	-	-	-	-	-	-
		-	-	-	-	-	-	-	-	-	-	-	-	190208	-	-	-	-	-	-	-	-	-	-	-	-	-	-	-	-	-	-	-	-	-	-	-	-	-	-	-	-	-	-	-	-	-	-	-	-	-	-
		-	-	-	-	-	-	-	-	-	-	-	-	190206	-	-	-	-	-	-	-	-	-	-	-	-	-	-	-	-	-	-	-	-	-	-	-	-	-	-	-	-	-	-	-	-	-	-	-	-	-	-
HSP22E	heat shock protein 22E	-	-	-	-	-	-	-	-	-	-	-	-	190200	-	-	-	-	-	-	-	-	-	-	-	-	-	34249	-	-	-	-	-	-	-	-	-	-	-	-	-	-	-	-	-	-	-	-	-	-	-	-
		-	-	-	-	-	-	-	-	-	-	-	-	190197	-	-	-	-	-	-	-	-	-	-	-	-	-	-	-	-	-	-	-	-	-	-	-	-	-	-	-	-	-	-	-	-	-	-	-	-	-	-
		-	-	-	-	-	-	-	-	-	-	-	-	190196	-	-	-	-	-	-	-	-	-	-	-	-	-	25886	-	-	-	-	16462	-	-	170513	-	-	-	-	-	-	-	-	-	-	-	-	-	-	-	-
FAP282	Flagellar Associated Coiled Coil Protein	-	-	-	-	-	-	-	-	-	-	-	-	190195	-	-	-	-	-	-	-	-	-	-	-	-	-	-	-	-	-	-	-	-	-	-	-	-	-	-	-	-	-	-	-	-	-	-	-	-	-	-
		-	-	-	-	-	-	-	-	-	-	-	-	190192	-	-	-	-	-	-	-	-	-	-	-	-	-	-	-	-	-	-	-	-	-	-	-	-	-	-	-	-	-	-	-	-	-	-	-	-	-	-
		-	-	-	-	-	-	-	-	-	-	-	-	190191	-	-	-	-	-	-	-	-	-	-	-	-	-	-	-	-	-	-	-	-	-	-	-	-	-	-	-	-	-	-	-	-	-	-	-	-	-	-
		-	-	-	-	-	-	-	-	-	-	-	-	190186	-	-	-	-	-	-	-	-	-	-	-	-	-	-	-	-	-	-	-	-	-	-	-	-	-	-	-	-	-	-	-	-	-	-	-	-	-	-
		-	-	-	-	-	-	-	-	-	-	-	-	190185	-	-	-	-	-	-	-	-	-	-	-	-	-	-	-	-	-	-	-	-	-	-	-	-	-	-	-	-	-	-	-	-	-	-	-	-	-	-
		-	+	-	-	-	-	GB10834-PA	AT1G03160.2	-	-	-	-	190184	-	-	-	-	DDB0187425	-	-	-	-	-	NP_284941.2	-	-	25320	73238	101013	-	Os05g32390.1	20016	-	-	149030	-	-	205434	-	-	-	-	-	-	-	-	-	-	-	-	-
		-	+	-	-	-	-	-	-	-	-	-	158003	190178	295564	cgd5_240	-	-	DDB0191976	-	-	-	-	-	-	-	232202	-	59663	-	-	-	33669	GSPATP00028659001	-	-	142532	-	-	RO3G_14815.1	-	-	-	-	27.m00276	-	-	65.m00026	63810	-	-	-
		-	+	+	-	-	-	GB11272-PA	AT5G12210.2	30070	-	CE24762	118195	190175	244163	cgd6_1750	CMT217C	OTTDARP00000022510	DDB0219584	CG18627-PA	19173456	505.m00018	ENSGALP00000018552	GL50803_17082	OTTHUMP00000011347	LmjF34.4030	165337	14854	59868	13474	NCU07509	Os08g40140.1	8811	GSPATP00024864001	28577	191544	156787	PFF0120w	806754	RO3G_06966.1	YPR176C	SPAC167.02	-	SINFRUP00000158725	89.m00153	268859	TA04900	583.m05739	53494	87414.m00280	Tb927.4.690	UM04276.1
		-	+	-	-	-	-	GB13326-PA	-	60889	BDEG_02412	-	-	190174	-	cgd4_3100	-	-	DDB0217781	-	-	-	ENSGALP00000004103	-	NP_060887.1	-	128523	32966	72900	137994	-	-	35526	GSPATP00031484001	43378	-	138267	MAL8P1.150	588866	RO3G_09281.1	-	-	GLEAN3_12084	-	189.m00052	24827	TA03520	59.m06067	61887	-	-	-
		-	-	-	-	-	-	-	-	-	-	-	-	190164	-	-	-	-	-	-	-	-	-	-	-	-	-	-	-	-	-	-	-	-	-	-	-	-	-	-	-	-	-	-	-	-	-	-	-	-	-	-
		-	-	-	-	-	-	-	-	-	-	-	-	190160	-	-	-	-	-	-	-	-	-	-	-	-	-	-	-	-	-	-	-	-	-	-	-	-	-	-	-	-	-	-	-	-	-	-	-	-	-	-
		-	+	-	-	-	-	-	AT5G43100.1	62422	-	-	-	190159	-	-	CMT203C	-	DDB0205773	-	-	-	-	-	-	-	-	-	-	-	-	Os07g40260.2	37724	GSPATP00014866001	31525	179925	144093	-	287275	-	-	-	-	-	-	-	-	49.m05696	-	-	-	-
		-	-	-	-	-	-	-	-	-	-	-	-	190156	-	-	-	-	-	-	-	-	-	-	-	-	-	-	-	-	-	-	-	-	-	-	-	-	-	-	-	-	-	-	-	-	-	-	-	-	-	-
		-	+	-	-	-	-	GB12967-PA	AT1G04790.1	-	BDEG_05871	-	219920	190155	299418	cgd6_1290	CMS095C	-	DDB0204786	CG9381-PB	19173466	-	ENSGALP00000027579	-	OTTHUMP00000161248	-	152917	-	46814	241157	-	Os04g43220.3	-	-	49280	-	136785	PFE1490c	554825	RO3G_14457.1	-	SPAP32A8.03c	-	SINFRUP00000147555	-	21140	TA14195	583.m00699	-	96895.m00058	Tb10.70.3100	-
		-	-	-	-	-	-	-	-	-	-	-	-	190154	-	-	-	-	-	-	-	-	-	-	-	-	-	-	-	-	-	-	-	-	-	-	-	-	-	-	-	-	-	-	-	-	-	-	-	-	-	-
ULP1	uncharacterized lumenal polypeptide	-	-	-	-	-	-	-	-	-	-	-	-	190151	-	-	-	-	-	-	-	-	-	-	-	-	-	-	-	-	-	-	31383	-	-	-	-	-	-	-	-	-	-	-	-	-	-	-	-	-	-	-
		-	+	-	-	-	-	-	AT1G75060.1	-	-	-	-	190150	-	-	-	-	-	-	-	-	ENSGALP00000006374	-	OTTHUMP00000160641	-	-	-	-	-	-	Os10g21460.1	-	-	-	200545	-	-	740257	-	-	-	-	SINFRUP00000131762	-	-	-	-	25386	-	-	-
ERD2A	KDEL Receptor A	-	+	+	-	-	-	GB11462-PA	AT3G25040.1	54782	BDEG_01241	CE01826	160167	190147	206937	cgd8_1110	CMR190C	OTTDARP00000020089	DDB0168686	CG5183-PC	19074408	186.m00118	ENSGALP00000019982	GL50803_4502	OTTHUMP00000069231	LmjF28.0630	106026	20669	60343	236409	NCU06771	Os11g28340.1	22716	GSPATP00008969001	42126	161051	108165	PF13_0280	577431	RO3G_16578.1	YBL040C	SPBP8B7.22	GLEAN3_22907	SINFRUP00000156542	136.m00087	34218	TA11365	55.m00006	21182	88399.m00055	Tb11.01.0410	UM04387.1
		-	+	-	-	-	-	-	-	-	-	-	183161	190146	-	-	-	-	DDB0204678	-	-	-	-	-	-	-	106220	-	64340	-	NCU11050	-	-	-	-	-	-	-	-	-	-	-	-	-	3.m01665	20739	-	-	-	-	-	-
DLC4	Flagellar outer dynein arm light chain 4	-	+	-	-	-	-	-	-	7786	-	-	-	190145	-	-	-	-	-	-	-	-	-	GL50803_7578	-	-	-	-	-	-	-	-	-	GSPATP00014268001	-	-	108841	-	-	-	-	-	-	-	200.m00044	33600	-	-	-	-	-	-
FAP225	Flagellar Associated Protein	-	-	-	+	-	-	-	-	-	-	-	-	190144	-	-	-	-	-	-	-	-	-	-	-	-	-	5632	-	-	-	-	-	-	-	-	-	-	-	-	-	-	-	-	-	-	-	-	54259	-	Tb11.01.3960	-
		-	-	-	-	-	-	-	-	-	-	-	-	190143	-	-	-	-	-	-	-	-	-	-	-	-	-	-	-	-	-	-	-	-	-	-	-	-	-	-	-	-	-	-	-	-	-	-	-	-	-	-
		-	+	-	-	-	-	GB18874-PA	AT5G51720.1	-	-	CE37932	148927	190138	229281	-	CMB155C	-	DDB0189485	-	-	-	ENSGALP00000004256	-	OTTHUMP00000019629	-	216266	15675	-	141604	-	Os07g28400.1	-	-	-	9234	142901	PFC0126c	570559	-	-	-	-	SINFRUP00000135160	-	20194	-	55.m08186	21706	-	-	-
		-	-	-	-	-	-	-	-	-	-	-	-	190133	-	-	-	-	-	-	-	-	-	-	-	-	-	-	-	-	-	-	-	-	-	-	-	-	-	-	-	-	-	-	-	-	-	-	-	-	-	-
		-	-	-	-	-	-	-	-	-	-	-	-	190128	-	-	-	-	-	-	-	-	-	-	-	-	-	-	82196	-	-	-	-	-	-	-	-	-	-	-	-	-	-	-	-	-	-	-	-	-	-	-
COX2b	Cytochrome c oxidase subunit II, protein IIb of split subunit.	-	+	-	-	-	-	-	ATMG00160.1	-	-	CE35351	204161	190125	-	-	CMW010C	-	-	-	-	-	ENSGALP00000034615	-	NP_536846.1	-	-	-	-	139879	-	-	-	-	-	157931	-	PF14_0288	283996	-	Q0250	SPMIT.11	-	-	-	-	TA02790	583.m00005	-	-	-	-
		-	-	-	-	-	-	-	-	-	-	-	-	190121	-	-	-	-	-	-	-	-	-	-	-	-	-	-	-	-	-	-	-	-	-	-	-	-	-	-	-	-	-	-	-	-	-	-	-	-	-	-
		-	-	-	-	-	-	-	AT3G27050.1	-	-	-	-	190119	-	-	-	-	-	-	-	-	-	-	-	-	-	-	-	-	-	Os11g29700.2	-	-	-	18502	-	-	641818	-	-	-	-	-	-	-	-	-	-	-	-	-
GTE3403	Ring3 protein	-	-	-	-	-	-	-	-	-	-	-	-	190116	-	-	-	-	-	-	-	-	-	-	-	-	-	-	-	-	-	-	-	-	-	-	-	-	-	-	-	-	-	-	-	-	-	-	-	-	-	-
		-	-	-	-	-	-	-	-	-	-	-	-	190113	-	-	-	-	-	-	-	-	-	-	-	-	-	-	-	-	-	-	-	-	-	-	-	-	-	-	-	-	-	-	-	-	-	-	-	-	-	-
		-	-	-	-	-	-	-	AT2G39910.1	-	-	-	-	190112	-	-	-	-	-	-	-	-	-	-	-	-	-	-	-	-	-	Os06g47900.1	-	-	-	170279	-	-	419433	-	-	-	-	-	-	-	-	-	-	-	-	-
		-	-	-	-	-	-	-	-	-	-	-	-	190111	-	-	-	-	-	-	-	-	-	-	-	-	-	-	-	-	-	-	-	-	-	-	-	-	-	-	-	-	-	-	-	-	-	-	-	-	-	-
		-	-	-	-	-	-	-	-	-	-	-	-	190106	-	-	-	-	-	-	-	-	-	-	-	-	-	-	-	-	-	-	-	-	-	-	-	-	-	-	-	-	-	-	-	-	-	-	-	-	-	-
		-	-	-	-	-	-	-	-	-	-	-	-	190103	-	-	-	-	-	-	-	-	-	-	-	-	-	-	-	-	-	-	-	-	-	-	-	-	-	-	-	-	-	-	-	-	-	-	-	-	-	-
		-	+	-	-	-	-	-	-	21273	-	-	202447	190098	-	-	-	-	-	-	-	-	-	-	-	-	159540	-	69475	-	NCU02543				-	-	-	-	-	-	-	-	-	-	-	11341	-	-	-	-	-	-
		-	-	-	-	-	-	-	-	-	-	-	-	190094	-	-	-	-	-	-	-	-	-	-	-	-	-	-	67271	-	-	-	-	-	-	173552	-	-	-	-	-	-	-	-	6.m00653	2294	-	-	-	-	-	-
		-	+	-	-	-	-	-	AT3G57810.3	-	-	-	-	190093	-	-	-	-	-	-	-	-	-	-	-	-	-	-	-	-	-	Os08g39560.1	32986	-	-	211921	-	-	583612	-	-	-	-	-	-	-	-	-	-	-	-	-
		-	+	-	-	-	-	-	-	-	-	-	-	190092	-	-	-	OTTDARP00000007534	-	-	-	-	ENSGALP00000013249	-	OTTHUMP00000019017	-	113635	-	-	248561	-	-	31476	-	-	-	-	-	-	-	-	-	-	-	-	-	-	-	-	-	-	UM03756.1
		-	-	-	-	-	-	-	-	-	-	-	-	190091	-	-	-	-	-	-	-	-	-	-	-	-	-	-	-	-	-	-	-	-	-	-	-	-	-	-	-	-	-	-	-	-	-	-	-	-	-	-
		-	-	-	-	-	-	-	-	-	-	-	-	190090	-	-	-	-	-	-	-	-	-	-	-	-	-	-	-	-	-	-	-	-	-	-	-	-	-	-	-	-	-	-	-	-	-	-	-	-	-	-
CAT2	catalase/peroxidase	-	+	-	-	-	-	-	-	-	-	-	-	190083	-	-	-	-	-	-	-	-	-	-	-	-	-	-	-	-	NCU05770	-	-	-	18572	-	123922	-	597360	-	-	-	-	-	-	11615	-	-	-	-	-	UM03399.1
	hypothetical protein	-	+	-	-	-	-	-	AT2G24860.1	-	-	-	-	190082	-	-	CMF104C	-	-	-	-	-	-	-	-	-	-	-	-	-	-	Os09g25590.1	-	-	40725	49086	-	-	562018	-	-	-	-	-	-	-	-	-	-	-	-	-
		-	-	-	-	-	-	-	-	-	-	-	-	190080	-	-	-	-	-	-	-	-	-	-	-	-	-	-	-	-	-	-	-	-	-	-	-	-	-	-	-	-	-	-	-	-	-	-	-	-	-	-
		-	-	-	-	-	-	-	-	-	-	-	-	190079	-	-	-	-	-	-	-	-	-	-	-	-	-	-	-	-	-	-	35395	-	-	-	-	-	-	-	-	-	-	-	-	-	-	-	-	-	-	-
FAP155	Flagellar Associated Protein	+	+	-	+	-	-	GB16696-PA	-	68098	BDEG_04469	-	177270	190077	236385	-	-	-	-	-	-	-	ENSGALP00000016511	-	NP_872345.1	-	212328	29891	68976	163817	-	-	-	GSPATP00017521001	-	-	128913	-	-	-	-	-	-	SINFRUP00000157841	108.m00189	-	-	-	50874	86827.m00490	Tb927.5.2270	-
		-	-	-	-	-	-	-	-	-	-	-	-	190074	-	-	-	-	-	-	-	-	-	-	-	-	-	-	-	-	-	-	-	-	-	-	-	-	-	-	-	-	-	-	-	-	-	-	-	-	-	-
PRL9		-	-	-	-	-	-	-	-	-	BDEG_08729	-	-	190072	-	-	-	-	-	-	-	-	-	-	NP_733758.1	-	160396	-	-	-	-	-	-	-	-	-	-	-	-	-	-	-	-	-	-	-	-	-	51643	-	-	-
		-	-	-	-	-	-	-	-	-	-	-	-	190071	-	-	-	-	-	-	-	-	-	-	-	-	-	-	-	-	-	-	-	-	-	-	-	-	-	-	-	-	-	-	-	-	-	-	-	-	-	-
		-	+	-	-	-	-	-	AT1G20080.1	-	-	-	-	190065	260095	-	-	OTTDARP00000002092	-	-	-	-	ENSGALP00000010556	-	NP_065779.1	-	-	38202	-	247335	-	Os02g25060.2	31947	-	50498	203762	-	-	803164	-	-	-	-	-	-	-	-	-	-	-	Tb10.61.3180	-
		-	-	-	-	-	-	-	-	-	-	-	-	190064	-	-	-	-	-	-	-	-	-	-	-	-	-	-	-	-	-	-	-	-	-	-	-	-	-	-	-	-	-	-	-	-	-	-	-	-	-	-
		-	-	-	-	-	-	-	-	-	-	-	-	190063	-	-	-	-	-	-	-	-	-	-	-	-	-	-	-	-	-	-	-	-	-	-	-	-	-	-	-	-	-	-	-	-	-	-	-	-	-	-
		-	+	-	-	-	-	-	-	61813	-	-	-	190062	-	-	-	-	DDB0218653	-	-	-	-	-	-	-	-	-	81161	222789	-	-	32718	-	-	-	131822	-	-	-	-	-	-	-	-	-	TA20782	-	-	-	-	-
		-	-	-	-	-	-	-	-	71644	-	-	99302	190061	-	-	-	-	-	-	-	-	-	-	-	-	-	-	-	-	-	-	11390	-	-	-	-	-	-	-	-	-	-	-	-	-	-	-	-	-	-	-
		-	-	-	-	-	-	-	-	-	-	-	-	190059	-	-	-	-	-	-	-	-	-	-	-	-	-	-	-	-	-	-	-	-	-	-	-	-	-	-	-	-	-	-	-	-	-	-	-	-	-	-
		-	-	-	-	-	-	-	-	-	-	-	-	190057	-	-	-	-	-	-	-	-	-	-	-	-	-	-	-	-	-	-	-	-	-	-	-	-	-	-	-	-	-	-	-	-	-	-	-	-	-	-
		-	-	-	-	-	-	-	-	-	-	-	-	190055	-	-	-	-	-	-	-	-	-	-	-	-	-	-	-	-	-	-	-	-	-	-	-	-	-	-	-	-	-	-	-	-	-	-	-	-	-	-
BBS7	Bardet-Biedl syndrome 7	+	+	-	-	-	-	GB18131-PA	-	22539	-	CE23024	117810	190054	224596	-	-	-	-	-	-	-	ENSGALP00000019359	-	OTTHUMP00000164091	LmjF29.0710	223884	31853	68114	103100	-	-	-	GSPATP00026091001	-	-	108146	-	-	-	-	-	GLEAN3_05312	SINFRUP00000142907	96.m00149	-	-	-	25051	-	Tb927.3.3640	-
		-	-	-	-	-	-	-	-	-	-	-	-	190053	-	-	-	-	-	-	-	-	-	-	-	-	-	-	-	-	-	-	-	-	-	-	-	-	-	-	-	-	-	-	-	-	-	-	-	-	-	-
		-	-	-	-	-	-	-	-	62277	-	-	-	190048	-	-	-	-	-	-	-	-	-	-	-	-	-	-	-	-	-	-	34657	-	-	170744	-	-	-	-	-	-	-	-	-	-	-	-	-	-	-	-
		-	-	-	-	-	-	-	-	-	-	-	-	190047	-	-	-	-	-	-	-	-	-	-	-	-	-	-	69303	-	-	-	-	-	44639	-	-	-	-	-	-	-	-	-	-	262506	-	-	-	-	-	-
		-	-	-	-	-	-	-	AT3G50685.1	-	-	-	-	190046	-	-	-	-	-	-	-	-	-	-	-	-	-	-	-	-	-	-	-	-	-	19071	-	-	219149	-	-	-	-	-	-	-	-	-	-	-	-	-
		-	+	+	-	-	-	GB15414-PA	AT5G11560.1	70759	BDEG_02717	CE27186	171523	190038	228585	-	-	-	DDB0217764	CG2943-PA	-	6.m00422	ENSGALP00000006311	-	OTTHUMP00000002581	LmjF34.4130	238345	30835	78106	234273	NCU00614	Os05g14170.1	20820	GSPATP00000688001	43900	184382	141803	-	802390	RO3G_15763.1	YCL045C	SPAC25H1.07	-	SINFRUP00000178221	21.m00262	24233	-	20.m03934	37902	-	Tb927.4.590	UM02448.1
		-	-	-	-	-	-	-	-	-	-	-	-	190036	-	-	-	-	-	-	-	-	-	-	-	-	-	-	-	-	-	-	-	-	-	-	-	-	-	-	-	-	-	-	-	-	-	-	-	-	-	-
		-	-	-	-	-	-	-	-	-	-	-	-	190034	-	-	-	-	-	-	-	-	-	-	-	-	-	-	-	-	-	-	-	-	-	-	-	-	-	-	-	-	-	-	-	-	-	-	-	-	-	-
		-	+	-	-	-	-	GB20088-PA	AT2G36305.1	19189	-	CE34031	19281	190033	-	-	-	-	DDB0184448	CG4852-PA	-	12.m00276	-	-	NP_005124.1	-	111054	2805	74579	218131	NCU11314	Os05g28950.1	-	-	7108	115539	136858	-	652951	-	YMR274C	SPAC1687.02	GLEAN3_03709	SINFRUP00000160497	-	269387	-	-	57211	87871.m00090	-	-
		-	-	-	-	-	-	-	-	-	-	-	-	190032	-	-	-	-	-	-	-	-	-	-	-	-	-	-	-	-	-	-	-	-	-	-	-	-	-	-	-	-	-	-	-	-	-	-	-	-	-	-
		-	-	-	-	-	-	-	-	-	-	-	-	190029	272191	-	-	-	-	-	-	-	-	-	-	-	-	-	-	-	-	-	-	-	-	-	-	-	-	-	-	-	-	-	-	-	-	-	-	-	-	-
		-	-	-	-	-	-	-	-	-	-	-	-	190027	-	-	-	-	-	-	-	-	-	-	-	-	-	-	-	-	-	-	-	-	-	-	-	-	-	-	-	-	-	-	-	-	-	-	-	-	-	-
		-	+	-	-	-	-	-	-	61014	BDEG_01839	-	-	190025	-	-	-	OTTDARP00000019269	-	-	-	9.m00438	-	-	-	LmjF36.4700	-	24578	-	245777	-	-	-	-	47285	-	-	-	-	-	-	-	-	SINFRUP00000178868	46.m00273	1479	-	-	-	-	Tb927.8.7450	-
		-	-	-	-	-	-	-	-	-	-	-	-	190024	-	-	-	-	-	-	-	-	-	-	-	-	-	-	-	-	-	-	-	-	-	-	-	-	-	-	-	-	-	-	-	-	-	-	-	-	-	-
		-	-	-	-	-	-	-	-	-	-	-	-	190023	-	-	-	-	-	-	-	-	-	-	-	-	-	-	-	-	-	-	-	-	-	-	-	-	-	-	-	-	-	-	-	-	-	-	-	-	-	-
		-	+	-	-	-	-	GB14336-PA	AT5G62910.1	-	BDEG_08498	CE27872	223184	190022	260876	cgd7_4960	CMM335C	OTTDARP00000014720	DDB0217593	CG31716-PG	-	-	ENSGALP00000019076	GL50803_8427	NP_001008226.1	-	117139	37886	37702	104011	NCU04073	Os02g37030.1	8862	-	10102	160398	111735	PFL1705w	661938	RO3G_05103.1	YER068W	SPAC16C9.04c	GLEAN3_20306	SINFRUP00000139476	-	17845	TA21145	49.m03145	51852	87875.m00079	-	UM02696.1
		-	-	-	-	-	-	-	-	-	-	-	-	190010	-	-	-	-	-	-	-	-	-	-	-	-	-	-	-	-	-	-	-	-	-	-	-	-	-	-	-	-	-	-	-	-	-	-	-	-	-	-
		-	-	-	-	-	-	-	-	-	-	-	-	190009	-	-	-	-	-	-	-	-	-	-	-	-	-	-	-	-	-	-	-	-	-	-	-	-	-	-	-	-	-	-	-	-	-	-	-	-	-	-
		-	-	-	-	-	-	-	AT2G46910.1	-	-	-	-	190008	-	-	-	-	-	-	-	-	-	-	-	-	-	-	-	-	-	-	36262	-	-	202760	-	-	552393	-	-	-	-	-	-	-	-	-	-	-	-	-
FAP85	Conserved Uncharacterized Flagellar Associated Protein, Similar to Notchless	-	+	-	+	-	-	-	-	31404	-	-	-	190007	-	-	-	-	DDB0191760	-	-	-	-	-	-	-	-	-	-	-	-	-	-	GSPATP00025202001	-	-	-	-	-	RO3G_01607.1	-	-	-	-	193.m00048	-	-	-	-	-	-	-
		-	-	-	-	-	-	-	-	-	-	-	-	189992	-	-	-	-	-	-	-	-	-	-	-	-	-	-	-	-	-	-	-	-	-	-	-	-	-	-	-	-	-	-	-	-	-	-	-	-	-	-
		-	-	-	-	-	-	-	-	-	-	-	-	189988	-	-	-	-	-	-	-	-	-	-	-	-	-	-	-	-	-	-	-	-	-	-	-	-	-	-	-	-	-	-	-	-	-	-	-	-	-	-
		-	-	-	-	-	-	-	-	-	-	-	-	189986	-	-	-	-	-	-	-	-	-	-	-	-	-	-	-	-	-	-	-	-	-	-	-	-	-	-	-	-	-	-	-	-	-	-	-	-	-	-
		-	-	-	-	-	-	-	-	-	-	-	-	189981	-	-	-	-	-	-	-	-	-	-	-	-	-	-	-	-	-	-	-	-	-	-	-	-	-	-	-	-	-	-	-	-	-	-	-	-	-	-
		-	-	-	-	-	-	-	-	-	-	-	-	189978	-	-	-	-	-	-	-	-	-	-	-	-	-	-	-	-	-	-	-	-	-	-	-	-	-	-	-	-	-	-	-	-	-	-	-	-	-	-
		-	-	-	-	-	-	-	-	-	-	-	-	189977	-	-	-	-	-	-	-	-	-	-	-	-	-	-	-	-	-	-	-	-	-	-	-	-	-	-	-	-	-	-	-	-	-	-	-	-	-	-
		-	-	-	-	-	-	-	-	-	-	-	-	189976	-	-	-	-	-	-	-	-	-	-	-	-	-	-	-	-	-	-	-	-	-	-	-	-	-	-	-	-	-	-	-	-	-	-	-	-	-	-
		-	-	-	-	-	-	-	-	-	-	-	-	189975	-	-	-	-	-	-	-	-	-	-	-	-	-	-	-	-	-	-	-	-	-	-	-	-	-	-	-	-	-	-	-	-	-	-	-	-	-	-
		-	+	-	-	-	-	-	AT1G55620.2	-	-	-	-	189974	-	-	CML267C	-	-	-	-	-	-	-	-	-	-	-	-	-	-	Os08g38980.1	32481	-	43785	116350	-	-	829973	-	-	-	-	-	-	262553	-	-	-	-	-	-
		-	-	-	-	-	-	-	-	-	-	-	-	189973	-	-	-	-	-	-	-	-	-	-	-	-	-	-	-	-	-	-	-	-	-	-	-	-	-	-	-	-	-	-	-	-	-	-	-	-	-	-
		-	+	-	-	-	-	GB12373-PA	-	-	-	-	-	189972	-	-	-	OTTDARP00000006993	-	-	-	-	ENSGALP00000015000	-	OTTHUMP00000035663	LmjF36.1350	-	-	-	-	-	-	-	-	-	188096	-	-	-	RO3G_04085.1	-	-	-	SINFRUP00000180578	103.m00103	-	-	-	-	-	Tb10.70.0230	-
		-	+	+	-	-	-	GB12994-PA	AT3G13440.1	6249	BDEG_03283	CE27815	164291	189967	285879	cgd4_3740	-	-	DDB0184184	CG9960-PA	19074311	303.m00071	ENSGALP00000025433	GL50803_1903	OTTHUMP00000096393	LmjF32.2090	118872	5849	32258	187131	NCU00504	Os03g60920.1	29075	GSPATP00005601001	5532	164820	156202	PF13_0016	179516	RO3G_10945.1	YDR140W	SPAC323.05c	-	SINFRUP00000132026	92.m00166	34058	TA09075	49.m03398	50516	83746.m00140	Tb11.01.6940	UM01979.1
		-	+	-	-	-	-	GB17974-PA	AT3G27640.1	-	BDEG_07801	CE12908	229258	189960	252722	-	-	OTTDARP00000004513	-	CG11295-PA	-	-	ENSGALP00000015973	-	NP_057532.1	-	138584	-	-	96181	NCU03668	Os03g49200.1	34764	-	-	196684	130850	-	752562	RO3G_03733.1	-	SPAC17H9.19c	-	SINFRUP00000133632	-	-	-	-	62034	-	-	UM01729.1
IRK2		-	-	-	-	-	-	-	-	-	-	-	-	189958	-	-	-	-	-	-	-	-	-	-	-	-	-	-	-	-	-	-	-	-	-	165096	-	-	-	-	-	-	-	-	-	-	-	-	-	-	-	-
		-	-	-	-	-	-	-	-	-	-	-	-	189957	-	-	-	-	-	-	-	-	-	-	-	-	-	-	-	-	-	-	-	-	-	-	-	-	-	-	-	-	-	-	-	-	-	-	-	-	-	-
		-	-	-	-	-	-	-	-	-	-	-	-	189956	-	-	-	-	-	-	-	-	-	-	-	-	-	-	-	-	-	-	-	-	-	-	-	-	-	-	-	-	-	-	-	-	-	-	-	-	-	-
		-	-	-	-	-	-	-	-	-	-	-	-	189953	-	-	-	-	-	-	-	-	-	-	-	-	-	-	-	-	-	-	-	-	-	-	-	-	-	-	-	-	-	-	-	-	-	-	-	-	-	-
		-	-	-	-	-	-	-	-	-	-	-	-	189950	-	-	-	-	-	-	-	-	-	-	-	-	-	-	-	-	-	-	-	-	-	-	-	-	-	-	-	-	-	-	-	-	-	-	-	-	-	-
		-	-	-	-	-	-	-	-	19900	-	-	-	189941	-	-	-	-	-	-	-	-	-	-	-	-	-	-	-	-	-	-	17171	-	-	159372	-	-	-	-	-	-	-	-	-	-	-	-	-	-	-	-
PDI2	Protein Disulfide Isomerase 2, PDI-D subfamily	-	-	-	-	-	-	-	AT2G47470.1	-	-	-	-	189940	-	-	-	-	-	-	-	-	-	-	-	-	-	-	29572	-	-	Os05g06430.2	-	-	-	-	-	-	-	-	-	-	-	-	-	-	-	-	-	84222.m00163	-	-
		-	-	-	-	-	-	-	AT3G62730.1	-	-	-	-	189937	-	-	-	-	-	-	-	-	-	-	-	-	-	-	-	-	-	Os04g33150.1	-	-	-	122983	-	-	642406	-	-	-	-	-	-	-	-	-	-	-	-	-
		-	-	-	-	-	-	-	-	-	-	-	-	189936	-	-	-	-	-	-	-	-	-	-	-	-	-	-	-	-	-	-	-	-	-	-	-	-	-	-	-	-	-	-	-	-	-	-	-	-	-	-
		-	-	-	-	-	-	-	-	-	-	-	-	189935	-	-	-	-	-	-	-	-	-	-	-	-	-	-	-	-	-	-	-	-	-	-	-	-	-	-	-	-	-	-	-	-	-	-	-	-	-	-
		-	-	-	-	-	-	-	-	-	-	-	-	189933	-	-	-	-	-	-	-	-	-	-	-	-	-	-	-	-	-	-	-	-	-	-	-	-	-	-	-	-	-	-	-	-	-	-	-	-	-	-
		-	-	-	-	-	-	-	-	-	-	-	-	189930	-	-	-	-	-	-	-	-	-	-	-	-	-	-	-	-	-	-	-	-	-	169411	109220	-	-	-	-	-	-	-	-	-	-	-	-	-	-	-
		-	-	-	-	-	-	-	-	-	-	-	-	189929	-	-	-	-	-	-	-	-	-	-	-	-	-	-	-	-	-	-	-	-	-	-	-	-	-	-	-	-	-	-	-	-	-	-	-	-	-	-
		-	-	-	-	-	-	-	-	-	-	-	-	189928	-	-	-	-	-	-	-	-	-	-	-	-	-	-	-	-	-	-	-	-	-	-	-	-	816895	-	-	-	-	-	-	-	-	-	-	-	-	-
		-	-	-	-	-	-	-	-	72817	-	-	-	189922	-	-	-	-	-	-	-	-	-	-	-	-	-	-	-	-	-	-	-	-	44473	-	133398	-	-	-	-	-	-	-	-	24552	-	-	-	-	-	-
FAP187	Conserved Uncharacterized Flagellar Associated Protein	-	+	-	+	-	-	-	-	-	BDEG_00568	-	187404	189917	287530	-	-	-	-	-	-	-	-	-	-	-	-	-	-	248770	-	-	-	-	-	232011	-	-	-	-	-	-	-	-	-	-	-	-	51347	86141.m00139	-	-
UGalT1_CHLRE		-	+	-	-	-	-	-	AT2G02810.1	-	BDEG_02720	-	220847	189914	290130	-	CMO220C	-	-	-	19068776	-	-	GL50803_15483	-	LmjF22.1010	-	38183	-	228065	NCU10721	Os06g39260.1	-	-	-	126784	-	-	653231	RO3G_05005.1	YPL244C	SPBC839.11c	GLEAN3_06031	SINFRUP00000161583	20.m00315	40489	TA16525	52.m00004	21659	-	Tb927.7.3070	UM03151.1
		-	-	-	-	-	-	-	-	-	-	-	-	189912	-	-	-	-	-	-	-	-	-	-	-	-	-	-	-	-	-	-	-	-	-	-	-	-	-	-	-	-	-	-	-	-	-	-	-	-	-	-
		-	-	-	-	-	-	-	-	-	-	-	-	189910	-	-	-	-	-	-	-	-	-	-	-	-	-	-	-	-	-	-	-	-	-	-	-	-	-	-	-	-	-	-	-	-	-	-	-	-	-	-
		-	+	-	-	-	-	-	AT4G37690.1	-	-	-	-	189909	-	-	-	-	-	-	-	-	-	-	-	-	-	-	76803	-	NCU09888	Os07g23494.1	-	-	33757	48067	-	-	663687	RO3G_12571.1	-	SPBC1289.13c	-	-	-	25537	-	-	-	-	-	-
		-	-	-	-	-	-	-	AT2G20940.1	-	-	-	-	189905	-	-	-	-	-	-	-	-	-	-	-	-	-	-	-	-	-	Os09g36982.1	-	-	-	-	-	-	200662	-	-	-	-	-	-	-	-	-	-	-	-	-
CSN2		-	+	+	-	-	-	GB17467-PA	AT2G26990.1	-	BDEG_06890	CE27562	151084	189898	215459	-	CMR159C	-	DDB0188387	CG9556-PA	-	86.m00166	ENSGALP00000038057	-	OTTHUMP00000176261	LmjF25.2210	181363	16111	60302	160233	NCU00593	Os01g17180.1	9469	GSPATP00030613001	28818	165552	142424	-	558178	RO3G_08398.1	-	SPAPB17E12.04c	-	SINFRUP00000133044	13.m00351	35771	-	-	30777	-	Tb927.3.2320	UM00817.1
PRPL34	Plastid ribosomal protein L34, imported to chloroplast, large ribosomal subunit	-	+	-	-	-	-	-	AT1G29070.1	-	-	-	-	189891	-	-	-	-	-	-	-	-	-	-	-	-	-	-	-	-	-	Os01g59060.1	17536	-	-	160895	-	-	235374	-	-	SPBC21C3.04c	-	-	-	-	-	-	-	-	-	-
		-	-	-	-	-	-	-	-	-	-	-	-	189889	-	-	-	-	-	-	-	-	-	-	-	-	-	-	-	-	-	-	-	-	-	-	-	-	-	-	-	-	-	-	-	-	-	-	-	-	-	-
		-	-	-	-	-	-	-	-	-	-	-	-	189883	-	-	-	-	-	-	-	-	-	-	-	-	-	-	-	-	-	-	-	-	-	-	-	-	-	-	-	-	-	-	-	-	-	-	-	-	-	-
		-	-	-	-	-	-	-	-	-	-	-	-	189880	-	-	-	-	-	-	-	-	-	-	-	-	-	-	-	-	-	-	-	-	-	-	-	-	-	-	-	-	-	-	-	-	-	-	-	-	-	-
HMGB3403	High mobility group protein	-	+	+	-	-	-	GB13118-PA	AT3G28730.1	72060	BDEG_02242	CE01414	172235	189879	214414	cgd7_5280	CMS034C	OTTDARP00000021667	DDB0188842	CG4817-PA	19069166	145.m00085	ENSGALP00000037946	-	NP_003137.1	-	207546	-	52473	110962	NCU11168	Os05g08970.1	36484	GSPATP00019702001	11409	21655	109072	PF14_0393	577589	RO3G_06992.1	YML069W	SPBC609.05	GLEAN3_25140	SINFRUP00000166989	3.m01985	262574	TA09185	55.m04934	64155	90417.m00146	-	UM04416.1
		-	-	-	-	-	-	-	-	-	-	-	-	189878	297087	-	-	-	-	-	-	-	-	-	-	-	-	-	-	-	-	-	-	-	-	-	-	-	-	-	-	-	-	-	-	-	-	-	-	-	-	-
TPIC	triose phosphate isomerase	-	-	-	-	-	-	-	-	-	-	-	-	-	-	-	-	-	-	-	-	-	-	-	-	-	-	-	-	-	-	-	-	-	-	-	-	-	-	-	-	-	-	-	-	-	-	-	-	-	-	-
COQ5D	ubiquinone/menaquinone biosynthesis methyltransferase	-	-	-	-	-	-	-	-	-	-	-	-	189872	-	-	-	-	-	-	-	-	-	-	-	-	-	-	-	-	-	-	-	-	-	-	-	-	-	-	-	-	-	-	-	-	-	-	-	-	-	-
		-	-	-	-	-	-	-	-	-	-	-	-	189870	-	-	-	-	-	-	-	-	-	-	-	-	-	-	-	-	-	-	-	-	-	-	-	-	-	-	-	-	-	-	-	-	-	-	-	-	-	-
		-	-	-	-	-	-	-	-	-	-	-	-	189866	-	-	-	-	-	-	-	-	-	-	-	-	-	-	-	-	-	-	-	-	-	-	-	-	-	-	-	-	-	-	-	-	-	-	-	-	-	-
		-	-	-	-	-	-	-	-	-	-	-	-	189858	-	-	-	-	-	-	-	-	-	-	-	-	-	-	-	-	-	-	-	-	-	-	-	-	-	-	-	-	-	-	-	-	-	-	-	-	-	-
		-	-	-	-	-	-	-	-	-	-	-	-	189857	-	-	-	-	-	-	-	-	-	-	-	-	-	-	-	-	-	-	-	-	-	-	-	-	-	-	-	-	-	-	-	-	-	-	-	-	-	-
		-	-	-	+	-	-	-	-	-	-	-	-	189856	-	-	-	-	-	-	-	-	-	-	-	-	-	-	-	-	-	-	-	-	-	-	-	-	-	-	-	-	-	-	-	-	-	-	-	-	-	-
		-	-	-	-	-	-	-	-	-	-	-	-	189849	-	-	-	-	-	-	-	-	-	-	-	-	-	-	-	-	-	-	-	-	-	-	-	-	-	-	-	-	-	-	-	-	-	-	-	-	-	-
		-	-	-	-	-	-	-	-	-	-	-	-	189836	-	-	-	-	-	-	-	-	-	-	-	-	-	-	-	-	-	-	-	-	-	-	-	-	-	-	-	-	-	-	-	-	-	-	-	-	-	-
		-	-	-	-	-	-	-	-	-	-	-	-	189835	-	-	-	-	-	-	-	-	-	-	-	-	-	-	-	-	-	-	-	-	-	-	-	-	-	-	-	-	-	-	-	-	-	-	-	-	-	-
		-	-	-	-	-	-	-	-	-	-	-	-	189834	-	-	-	-	-	-	-	-	-	-	-	-	-	-	-	-	-	-	-	-	-	-	-	-	-	-	-	-	-	-	-	-	-	-	-	-	-	-
RFK3		-	+	-	+	-	-	-	-	16279	-	-	-	189830	-	-	CMJ229C	-	-	-	-	193.m00065	-	-	-	LmjF35.3160	-	-	-	-	NCU08763	-	7347	-	12172	125797	-	-	-	-	-	-	-	-	-	-	-	-	-	-	Tb09.211.3420	-
		-	-	-	-	-	-	-	-	-	-	-	-	189825	-	-	-	-	-	-	-	-	-	-	-	-	-	-	-	-	-	-	-	-	-	-	-	-	-	-	-	-	-	-	-	-	-	-	-	-	-	-
		-	-	-	-	-	-	-	-	-	-	-	-	189821	-	-	-	-	-	-	-	-	-	-	-	-	-	-	-	-	-	-	-	-	-	-	-	-	-	-	-	-	-	-	-	-	-	-	-	-	-	-
		-	-	-	-	-	-	-	-	-	-	-	-	189820	-	-	-	-	-	-	-	-	-	-	-	-	-	-	-	-	-	-	-	-	-	-	-	-	-	-	-	-	-	-	-	-	-	-	-	-	-	-
		-	-	-	-	-	-	-	-	-	-	-	-	189819	-	-	-	-	-	-	-	-	-	-	-	-	-	-	-	-	-	-	-	-	-	-	-	-	-	-	-	-	-	-	-	-	-	-	-	-	-	-
		-	-	-	-	-	-	-	-	-	-	-	-	189809	-	-	-	-	-	-	-	-	-	-	-	-	-	-	-	-	-	-	-	-	-	-	-	-	-	-	-	-	-	-	-	-	-	-	-	-	-	-
		-	+	-	-	-	-	-	AT1G08030.1	-	-	-	-	189798	-	-	-	-	-	-	-	-	-	-	-	-	-	4844	-	-	-	Os06g06370.1	35351	-	-	65073	-	-	768390	-	-	-	-	-	-	-	-	-	-	-	-	-
		-	-	-	-	-	-	-	-	-	-	-	-	189797	-	-	-	-	-	-	-	-	-	-	-	-	-	-	-	-	-	-	-	-	-	-	-	-	-	-	-	-	-	-	-	-	-	-	-	-	-	-
		-	-	-	-	-	-	-	-	-	-	-	229136	189793	-	-	-	-	-	-	-	-	-	-	-	-	137224	-	-	-	-	-	-	-	-	-	156571	-	-	-	-	-	-	-	-	-	-	-	-	-	-	-
		-	-	-	-	-	-	-	-	-	-	-	-	189792	-	-	-	-	-	-	-	-	-	-	-	-	-	-	-	-	-	-	-	-	-	-	-	-	-	-	-	-	-	-	-	-	-	-	-	-	-	-
ADCY31	adenylate cyclase	-	-	-	-	-	-	-	-	-	-	-	-	189791	-	-	-	-	-	-	-	-	-	-	-	-	-	-	-	-	-	-	-	-	-	-	-	-	-	-	-	-	-	-	-	-	-	-	-	-	-	-
		-	+	+	-	-	-	GB13024-PA	AT3G15380.1	-	BDEG_01599	CE30970	153937	189789	260727	-	-	OTTDARP00000019798	DDB0188210	CG1311-PA	-	5.m00483	ENSGALP00000018540	-	OTTHUMP00000014867	LmjF14.0380	120296	18433	740	241158	NCU08895	Os04g58470.2	29832	GSPATP00005580001	45782	119604	141480	-	728407	RO3G_11730.1	YOR161C	-	GLEAN3_19227	SINFRUP00000168180	4.m00607	262743	-	31.m00865	53446	84985.m00055	Tb10.70.7750	UM00015.1
		-	-	-	-	-	-	-	-	-	-	-	-	189785	-	-	-	-	-	-	-	-	-	-	-	-	-	-	-	-	-	-	-	-	-	-	-	-	-	-	-	-	-	-	-	-	-	-	-	-	-	-
TIM22A	translocase of inner mitochondrial membrane 22 homolog	-	+	-	-	-	-	-	AT3G10110.1	8485	BDEG_08219	CE25820	138199	189783	295148	-	CMS209C	-	DDB0218527	-	-	-	ENSGALP00000006979	-	OTTHUMP00000180689	-	97403	30975	4182	160394	NCU03798	Os03g18500.2	32867	GSPATP00033637001	7114	18688	143292	PFF1330c	202436	-	YDL217C	SPBC25H2.04c	GLEAN3_21795	SINFRUP00000129414	-	268574	-	42.m00045	-	-	-	UM02563.1
		-	-	-	-	-	-	-	-	-	-	-	-	189781	-	-	-	-	-	-	-	-	-	-	-	-	-	-	-	-	-	-	-	-	-	-	-	-	-	-	-	-	-	-	-	-	-	-	-	-	-	-
		-	-	-	-	-	-	-	-	-	-	-	-	189780	-	-	-	-	-	-	-	-	-	-	-	-	-	-	-	-	-	-	-	-	-	-	-	-	-	-	-	-	-	-	-	-	-	-	-	-	-	-
HSP22D	heat shock protein 22D	-	-	-	-	-	-	-	-	-	-	-	-	189779	-	-	-	-	-	-	-	-	-	-	-	-	-	-	-	-	-	-	-	-	-	-	-	-	-	-	-	-	-	-	-	-	-	-	-	-	-	-
		-	+	-	-	-	-	GB19636-PA	AT1G73720.1	25560	BDEG_00552	CE15742	225878	189778	291748	cgd3_3070	-	-	DDB0205393	-	-	-	ENSGALP00000003130	-	OTTHUMP00000021186	-	198443	38210	70866	185519	-	Os01g21940.1	32673	GSPATP00038197001	43349	189716	140994	MAL13P1.54	823135	RO3G_11175.1	-	-	-	SINFRUP00000154648	70.m00171	262521	TA19400	80.m02289	52324	-	-	-
		-	-	-	-	-	-	-	-	-	-	-	-	189773	-	-	-	-	-	-	-	-	-	-	-	-	-	-	-	-	-	-	-	-	-	-	-	-	-	-	-	-	-	-	-	-	-	-	-	-	-	-
		-	+	-	-	-	-	-	-	71252	BDEG_04908	-	-	189770	-	-	-	-	DDB0201821	-	-	-	-	-	-	-	-	29211	-	-	-	-	-	-	45364	-	135679	-	-	-	-	-	-	-	-	2690	-	-	61807	-	-	-
GME1		-	+	-	-	-	-	-	AT5G28840.1	69812	-	-	-	189769	-	-	CMS308C	-	-	-	-	-	-	-	-	-	-	38874	-	-	-	Os11g37890.1	24671	-	45434	170239	-	PF10_0137	595429	-	-	-	-	-	-	41548	-	-	-	93514.m00027	Tb11.01.5560	-
		-	+	+	-	-	-	GB18573-PA	AT5G05920.1	-	BDEG_02373	CE19037	1610	189768	291895	cgd2_3930	CMA098C	-	DDB0186679	CG8005-PA	-	83.m00172	-	GL50803_15535	OTTHUMP00000083185	-	222351	15470	56201	172583	NCU05418	Os03g52970.3	18111	GSPATP00039607001	46999	130818	109344	PF14_0125	831005	RO3G_10161.1	YHR068W	SPBC1271.04c	GLEAN3_12301	SINFRUP00000156605	139.m00116	268986	TA13570	46.m00007	20912	92757.m00241	-	UM00071.1

		-	-	-	-	-	-	-	-	-	-	-	-	189766	-	-	-	-	-	-	-	-	-	-	-	-	-	-	-	-	-	-	-	-	-	-	-	-	-	-	-	-	-	-	-	-	-	-	-	-	-	-
		-	+	-	-	-	-	-	-	16582	-	-	-	189765	-	-	CMR172C	-	-	-	-	-	ENSGALP00000019357	-	OTTHUMP00000065594	LmjF35.4560	229680	-	-	169962	-	-	32665	GSPATP00035514001	-	-	-	-	-	-	-	-	-	-	-	-	-	-	53607	-	-	-
		-	-	-	-	-	-	-	-	-	-	-	-	189764	-	-	-	-	-	-	-	-	-	-	-	-	-	31096	-	-	-	-	-	-	-	132083	-	-	763077	-	-	-	-	-	-	-	-	-	-	-	-	-
		-	+	-	-	-	-	-	AT4G08170.2	-	-	-	99999	189763	-	-	-	OTTDARP00000005073	-	-	-	-	ENSGALP00000036796	-	OTTHUMP00000028357	-	127367	-	-	-	-	Os03g51610.1	-	-	-	102429	-	-	209085	-	-	-	-	-	-	-	-	-	-	-	-	-
		-	-	-	-	-	-	-	-	-	-	-	-	189762	-	-	-	-	-	-	-	-	-	-	-	-	-	-	-	-	-	-	-	-	-	-	-	-	-	-	-	-	-	-	-	-	-	-	-	-	-	-
		-	-	-	-	-	-	-	-	-	-	-	-	189761	-	-	-	-	-	-	-	-	-	-	-	-	-	-	-	-	-	-	-	-	-	-	-	-	-	-	-	-	-	-	-	-	-	-	-	-	-	-
		-	-	-	-	-	-	-	-	-	-	-	-	189758	-	-	-	-	-	-	-	-	-	-	-	-	-	-	-	-	-	-	-	-	-	-	-	-	-	-	-	-	-	-	-	-	-	-	-	-	-	-
	Putative quinoprotein	-	-	-	-	-	-	-	-	-	-	-	-	189756	291434	-	-	-	-	-	-	-	-	-	-	-	-	-	-	-	-	-	-	-	-	-	-	-	-	-	-	-	-	-	-	-	-	-	-	-	-	-
		-	-	-	-	-	-	-	-	-	-	-	-	189754	-	-	-	-	-	-	-	-	-	-	-	-	-	-	-	-	-	-	-	-	-	-	-	-	-	-	-	-	-	-	-	-	-	-	-	-	-	-
		-	-	-	-	-	-	-	-	-	-	-	-	189752	-	-	-	-	-	-	-	-	-	-	-	-	-	-	-	-	-	-	-	-	-	-	-	-	-	-	-	-	-	-	-	-	-	-	-	-	-	-
		-	+	+	-	-	-	GB10315-PA	AT3G59380.1	4335	BDEG_05042	CE18099	169970	189746	203696	cgd6_2510	-	-	DDB0218423	CG2976-PA	-	288.m00066	ENSGALP00000024792	GL50803_15820	OTTHUMP00000177445	-	201479	29023	2982	158576	NCU03632	Os09g33930.4	12665	GSPATP00009156001	45841	195400	115551	-	562248	RO3G_00627.1	YKL019W	SPAPB1A10.04c	-	SINFRUP00000147062	68.m00150	268577	TA17870	-	32965	95305.m00242	-	UM01488.1
		-	-	-	-	-	-	-	-	-	-	-	-	189742	-	-	-	-	-	-	-	-	-	-	-	-	-	-	-	-	-	-	-	-	-	-	-	-	-	-	-	-	-	-	-	-	-	-	-	-	-	-
		-	-	-	-	-	-	-	-	-	-	-	-	189733	-	-	-	-	-	-	-	-	-	-	-	-	-	-	-	-	-	-	-	-	-	-	-	-	-	-	-	-	-	-	-	-	-	-	-	-	-	-
		-	+	-	-	-	-	-	AT3G01920.1	61842	-	-	-	189732	-	-	CMC144C	-	-	-	-	-	-	-	-	-	-	-	-	-	-	Os08g41910.1	18816	-	-	216189	-	-	174486	-	-	-	-	-	-	-	-	-	-	-	-	-
FAP68	Flagellar Associated Protein	-	-	-	-	-	-	-	-	-	-	-	-	189731	-	-	-	-	-	-	-	-	-	-	-	-	-	-	-	-	-	-	-	-	-	-	-	-	-	-	-	-	-	-	-	-	-	-	-	-	-	-
POLD1	DNA polymerase delta subunit one.	-	+	+	-	-	-	GB17691-PA	AT5G63960.1	23037	BDEG_05039	CE09308	165849	189721	294018	cgd6_4410	CMN199C	OTTDARP00000019603	DDB0186487	CG5949-PA	19171290	53.m00199	-	GL50803_35094	OTTHUMP00000077835	LmjF33.1690	194456	27827	44642	233970	NCU01192	Os11g08330.1	31253	GSPATP00011433001	54677	159627	144243	PF10_0165	207140	RO3G_03762.1	YDL102W	SPBC336.04	GLEAN3_03651	SINFRUP00000158626	91.m00110	35189	TA06290	55.m04733	18979	81616.m00166	Tb927.2.1800	UM01458.1
		-	-	-	-	-	-	-	-	-	-	-	-	189720	-	-	-	-	-	-	-	-	-	-	-	-	-	-	-	-	-	-	-	-	-	-	-	-	-	-	-	-	-	-	-	-	-	-	-	-	-	-
		-	-	-	-	-	-	-	-	-	-	-	-	189719	-	-	-	-	-	-	-	-	-	-	-	-	-	-	-	-	-	-	-	-	-	-	-	-	-	-	-	-	-	-	-	-	-	-	-	-	-	-
		-	-	-	-	-	-	-	-	-	-	-	-	189718	-	-	-	-	-	-	-	-	-	-	-	-	-	-	-	-	-	-	-	-	-	-	-	-	-	-	-	-	-	-	-	-	-	-	-	-	-	-
		-	+	-	-	-	-	GB19119-PA	AT3G12760.1	33362	BDEG_02369	CE33527	112003	189714	296649	-	CMN116C	OTTDARP00000006608	DDB0185973	CG7427-PA	-	-	ENSGALP00000014300	-	OTTHUMP00000173758	LmjF34.4270	214717	33856	49668	-	NCU05716	Os06g12690.1	25391	GSPATP00004745001	-	-	157543	-	814035	RO3G_00990.1	YLR128W	SPBC839.03c	-	SINFRUP00000162932	97.m00184	25225	-	46.m03689	49550	-	Tb10.6k15.1330	UM01246.1
		-	-	-	-	-	-	-	-	-	-	-	-	189712	-	-	-	-	-	-	-	-	-	-	-	-	-	-	-	-	-	-	-	-	-	-	-	-	-	-	-	-	-	-	-	-	-	-	-	-	-	-
		-	-	-	-	-	-	-	-	-	-	-	-	189710	-	-	-	-	-	-	-	-	-	-	-	-	-	-	-	-	-	-	-	-	-	-	-	-	-	-	-	-	-	-	-	-	-	-	-	-	-	-
		-	-	-	-	-	-	-	-	-	-	-	-	189708	-	-	-	-	-	-	-	-	-	-	-	-	-	-	-	-	-	-	-	-	-	-	-	-	-	-	-	-	-	-	-	-	-	-	-	-	-	-
		-	-	-	-	-	-	-	-	-	-	-	-	189707	-	-	-	-	-	-	-	-	-	-	-	-	-	-	-	-	-	-	-	-	-	-	-	-	-	-	-	-	-	-	-	-	-	-	-	-	-	-
MFT5	MATE efflux family protein	-	+	-	-	-	-	-	-	-	-	-	221225	189706	-	-	-	-	-	-	-	-	-	-	OTTHUMP00000065793	-	-	-	-	-	-	-	30071	-	35296	183778	-	-	-	-	YHR032W	SPCC4B3.13	-	-	3.m01770	-	-	-	-	-	Tb10.389.1330	-
		-	-	-	-	-	-	-	-	-	-	-	-	189699	-	-	-	-	-	-	-	-	-	-	-	-	-	-	-	-	-	-	-	-	48164	-	-	-	-	-	-	-	-	-	-	7508	-	-	-	-	-	-
		-	-	-	-	-	-	-	-	-	-	-	-	189698	-	-	-	-	-	-	-	-	-	-	-	-	-	25902	-	-	-	-	-	-	-	-	-	-	-	-	-	-	-	-	-	-	-	-	-	-	-	-
		-	+	+	-	-	-	GB19864-PA	AT3G45740.1	12353	-	CE34906	20061	189693	272569	-	-	OTTDARP00000023578	DDB0185766	-	-	11.m00336	ENSGALP00000021239	GL50803_13890	OTTHUMP00000196150	LmjF35.4320	201919	-	62850	-	NCU05818	Os03g17590.1	-	GSPATP00010268001	-	153816	158662	-	765354	-	YKR070W	SPAC22A12.08c	-	SINFRUP00000148637	47.m00274	6800	-	-	-	-	Tb09.211.1880	UM04682.1
		-	-	-	-	-	-	-	-	-	-	-	-	189692	-	-	-	-	-	-	-	-	-	-	-	-	-	-	-	-	-	-	-	-	-	-	-	-	-	-	-	-	-	-	-	-	-	-	-	-	-	-
		-	-	-	-	-	-	-	AT1G69040.2	-	-	-	-	189688	-	-	-	-	-	-	-	-	-	-	-	-	-	-	-	-	-	Os03g29980.1	-	-	-	135380	-	-	723869	-	-	-	-	-	-	-	-	-	-	-	-	-
		-	-	-	+	-	-	-	AT4G22200.1	-	-	-	-	189686	-	-	-	-	-	-	-	-	-	-	-	-	-	-	-	-	-	Os06g14310.1	-	-	-	164636	-	-	802408	-	-	-	-	-	-	-	-	-	-	-	-	-
		-	-	-	-	-	-	-	-	-	-	-	-	189679	-	-	-	-	-	-	-	-	-	-	-	-	-	-	-	-	-	-	-	-	-	-	-	-	-	-	-	-	-	-	-	-	-	-	-	-	-	-
MSRA4	peptide methionine sulfoxide reductase	-	+	-	-	-	-	GB10196-PA	-	20277	-	-	178146	189675	289939	-	-	OTTDARP00000005938	DDB0217823	-	-	-	ENSGALP00000026815	-	OTTHUMP00000176999	-	232500	13036	-	-	-	-	29409	-	-	109244	-	-	-	-	YER042W	-	-	SINFRUP00000158962	-	-	-	-	-	-	-	UM01846.1
		-	-	-	-	-	-	-	-	-	-	-	-	189674	-	-	-	-	-	-	-	-	-	-	-	-	-	-	-	-	-	-	-	-	-	-	-	-	-	-	-	-	GLEAN3_10918	-	-	-	-	-	-	-	-	-
		-	-	-	-	-	-	-	-	-	-	-	-	189671	-	-	-	-	-	-	-	-	-	-	-	-	-	-	-	-	-	-	-	-	-	-	-	-	-	-	-	-	-	-	-	-	-	-	-	-	-	-
		-	-	-	-	-	-	-	-	-	-	-	-	189662	-	-	-	-	-	-	-	-	-	-	-	-	-	-	-	-	-	-	-	-	-	-	-	-	-	-	-	-	-	-	-	-	-	-	-	-	-	-
		-	-	-	-	-	-	-	-	-	-	-	-	189655	-	-	-	-	-	-	-	-	-	-	-	-	-	-	-	-	-	-	-	-	-	-	-	-	-	-	-	-	-	-	-	-	-	-	-	-	-	-
		-	-	-	-	-	-	-	-	-	-	-	-	189644	-	-	-	-	-	-	-	-	-	-	-	-	-	-	-	-	-	-	-	-	-	-	-	-	-	-	-	-	-	-	-	-	-	-	-	-	-	-
		-	-	-	-	-	-	-	AT1G33390.1	-	-	-	-	189639	-	-	-	-	-	-	-	-	-	-	-	-	-	-	-	-	-	-	17314	-	-	-	-	-	-	-	-	-	-	-	-	-	-	-	-	-	-	-
	hypothetical protein	-	-	-	-	-	-	-	-	-	-	-	-	189638	-	-	-	-	-	-	-	-	-	-	-	-	-	-	-	-	-	-	-	-	-	-	-	-	-	-	-	-	-	-	-	-	-	-	-	-	-	-
		-	-	-	-	-	-	-	-	-	-	-	-	189636	-	-	-	-	-	-	-	-	-	-	-	-	-	-	-	-	-	-	-	-	-	-	-	-	-	-	-	-	-	-	-	-	-	-	-	-	-	-
		-	-	-	-	-	-	-	-	-	-	-	-	189635	-	-	-	-	DDB0217740	-	-	-	-	-	-	-	-	-	-	-	-	-	-	-	-	-	-	-	-	-	-	-	-	-	-	-	-	-	-	-	-	-
FAP20	Flagellar Associated Protein	-	-	-	-	-	-	-	-	-	-	-	-	-	-	-	-	-	-	-	-	-	-	-	-	-	-	-	-	-	-	-	-	-	-	-	-	-	-	-	-	-	-	-	-	-	-	-	-	-	-	-
		-	-	-	-	-	-	-	-	-	-	-	-	189627	-	-	-	-	-	-	-	-	-	-	-	-	-	-	-	-	-	-	-	-	-	-	-	-	-	-	-	-	-	-	-	-	-	-	-	-	-	-
		-	-	-	-	-	-	-	-	-	-	-	-	189626	-	-	-	-	-	-	-	-	-	-	-	-	-	-	-	-	-	-	-	-	-	-	-	-	-	-	-	-	-	-	-	-	-	-	-	-	-	-
		-	-	-	-	-	-	-	-	-	-	-	-	189625	-	-	-	-	-	-	-	-	-	-	-	-	-	-	-	-	-	-	-	-	-	-	-	-	-	-	-	-	-	-	-	-	-	-	-	-	-	-
PTOX1	alternative oxidase, possibly chloroplast-localized	-	+	-	-	-	-	-	AT4G22260.1	14460	-	-	-	189624	-	-	CMI244C	-	-	-	-	-	-	-	-	-	-	-	-	-	-	Os04g57320.1	21990	-	41170	194301	-	-	274655	-	-	-	-	-	-	25462	-	-	-	-	-	-
		-	-	-	-	-	-	-	-	-	-	-	-	189621	-	-	-	-	-	-	-	-	-	-	-	-	-	-	-	-	-	-	-	-	-	-	-	-	-	-	-	-	-	-	-	-	-	-	-	-	-	-
		-	-	-	-	-	-	-	-	-	-	-	-	189618	-	-	-	-	-	-	-	-	-	-	-	-	-	-	-	-	-	-	-	-	-	-	-	-	-	-	-	-	-	-	-	-	-	-	-	-	-	-
		-	-	-	-	-	-	-	-	-	-	-	-	189615	-	-	-	-	-	-	-	-	-	-	-	-	-	-	-	-	-	-	-	-	-	-	-	-	-	-	-	-	-	-	-	-	-	-	-	-	-	-
		-	-	-	-	-	-	-	-	-	-	-	-	189613	-	-	-	-	-	-	-	-	-	-	-	-	-	-	-	-	-	-	-	-	-	-	-	-	-	-	-	-	-	-	-	-	-	-	-	-	-	-
		-	-	-	-	-	-	-	-	-	-	-	-	189609	-	-	-	-	-	-	-	-	-	-	-	-	-	-	-	-	-	-	-	-	-	-	-	-	-	-	-	-	-	-	-	-	-	-	-	-	-	-
		-	-	-	-	-	-	-	-	-	-	-	-	189606	-	-	-	-	-	-	-	-	-	-	-	-	-	-	-	-	-	-	-	-	-	-	-	-	-	-	-	-	-	-	-	-	-	-	-	-	-	-
		-	-	-	-	-	-	-	-	-	-	-	-	189605	-	-	-	-	-	-	-	-	-	-	-	-	-	-	-	-	-	-	-	-	-	-	-	-	-	-	-	-	-	-	-	-	-	-	-	-	-	-
		-	+	-	-	-	-	-	AT5G62640.2	-	-	-	-	189603	-	-	-	-	-	-	-	-	-	-	-	-	106827	-	-	-	-	Os07g09510.1	35489	-	-	-	130026	-	824729	-	-	SPCC663.11	-	-	-	-	-	59.m03530	-	-	-	-
		-	-	-	-	-	-	-	-	-	-	-	-	189600	-	-	-	-	-	-	-	-	-	-	-	-	-	-	-	-	NCU03639	-	-	-	-	-	-	-	-	-	-	-	-	-	32.m00181	-	-	-	-	-	-	-
		-	-	-	-	-	-	-	-	-	-	-	-	189599	-	-	-	-	-	-	-	-	-	-	-	-	-	-	-	-	-	-	-	-	-	-	-	-	-	-	-	-	-	-	-	-	-	-	-	-	-	-
		-	-	-	-	-	-	-	-	-	-	-	-	189597	-	-	-	-	-	-	-	-	-	-	-	-	-	-	-	-	-	-	-	-	-	-	-	-	-	-	-	-	-	-	-	-	-	-	-	-	-	-
		-	-	-	-	-	-	-	-	-	-	-	-	189596	-	-	-	-	-	-	-	-	-	-	-	-	-	-	-	-	-	-	-	-	-	-	-	-	-	-	-	-	-	-	-	-	-	-	-	-	-	-
		-	-	-	-	-	-	-	-	-	-	-	-	189595	-	-	-	-	-	-	-	-	-	-	-	-	-	-	-	-	-	-	-	-	-	-	-	-	-	-	-	-	-	-	-	-	-	-	-	-	-	-
		-	-	-	-	-	-	-	-	-	-	-	-	189591	-	-	-	-	-	-	-	-	-	-	-	-	-	-	-	-	-	-	-	-	-	-	-	-	-	-	-	-	-	-	-	-	-	-	-	-	-	-
		-	+	-	-	-	-	GB19424-PA	AT1G63980.2	-	BDEG_04033	-	-	189589	-	-	-	OTTDARP00000004619	-	CG11180-PA	-	-	ENSGALP00000026820	-	OTTHUMP00000177040	LmjF06.1020	-	39339	-	244746	NCU02528	Os03g03390.1	30355	-	34050	173421	-	-	554418	-	-	SPAC890.05	-	SINFRUP00000133862	-	21973	-	-	56912	-	Tb927.7.5640	-
		-	+	-	-	-	-	-	-	-	-	-	-	189588	-	-	-	-	DDB0202873	-	-	-	-	-	-	LmjF23.1460	-	-	70295	-	NCU01163	-	-	GSPATP00008911001	-	-	157709	-	-	RO3G_12580.1	YKL069W	-	-	-	73.m00190	-	-	-	-	86622.m00259	Tb927.5.1250	UM06048.1
RSP7	Radial spoke protein 7	-	-	-	-	-	-	-	-	-	-	-	-	189584	-	-	-	-	-	-	-	-	-	-	-	-	-	-	-	-	-	-	-	-	-	-	129937	-	-	-	-	-	-	-	-	-	-	-	-	-	-	-
		-	-	-	-	-	-	-	-	-	-	-	-	189583	-	-	-	-	-	-	-	-	-	-	-	-	-	-	-	-	-	-	-	-	-	-	-	-	-	-	-	-	-	-	-	-	-	-	-	-	-	-
		-	-	-	-	-	-	-	-	-	-	-	-	189582	-	-	-	-	-	-	-	-	-	-	-	-	-	-	-	-	-	-	-	-	-	-	-	-	-	-	-	-	-	-	-	-	-	-	-	-	-	-
		-	-	-	-	-	-	-	AT5G48440.2	-	-	-	-	189580	-	-	-	-	-	-	-	-	-	-	-	-	-	-	-	-	-	Os09g33900.1	-	-	-	121857	-	-	287331	-	-	-	-	-	-	-	-	-	-	-	-	-
		-	+	-	-	-	-	GB10991-PA	-	-	-	-	-	189576	298752	-	-	OTTDARP00000006144	-	CG5958-PA	-	-	ENSGALP00000024913	-	OTTHUMP00000177564	-	-	-	-	12337	-	-	-	-	-	-	-	-	-	-	-	-	-	SINFRUP00000131810	-	-	-	-	-	-	-	-
		-	-	-	-	-	-	-	-	-	-	-	-	189575	-	-	-	-	-	-	-	-	-	-	-	-	-	-	-	-	-	-	-	-	-	-	-	-	-	-	-	-	-	-	-	-	-	-	-	-	-	-
		-	-	-	-	-	-	-	-	-	-	-	-	189571	-	-	-	-	-	-	-	-	-	-	-	-	-	-	-	-	-	-	-	-	-	-	-	-	-	-	-	-	-	-	-	-	-	-	-	-	-	-
		-	-	-	-	-	-	-	AT5G24610.1	-	-	-	-	189570	-	-	-	-	-	-	-	-	-	-	-	-	-	-	-	-	-	Os10g36550.3	35875	-	-	-	-	-	732714	-	-	-	-	-	-	-	-	-	-	-	-	-
		-	+	+	-	-	-	GB12087-PA	-	-	-	CE31300	5356	189561	288767	-	-	-	DDB0188074	CG9752-PA	-	77.m00176	ENSGALP00000020530	-	OTTHUMP00000021562	LmjF36.1670	195975	-	31474	230591	NCU03527	Os07g39980.1	14953	GSPATP00027434001	16120	68232	158822	-	757112	RO3G_08101.1	-	SPAC589.05c	-	-	66.m00263	31819	-	-	29087	97190.m00040	-	-
		-	+	-	-	-	-	GB15448-PA	AT4G33945.1	64111	-	-	184330	189560	261179	-	-	-	-	CG5721-PA	-	-	ENSGALP00000004770	-	OTTHUMP00000067844	-	189885	22281	-	244842	-	Os02g52370.1	-	-	49181	94302	133152	-	837122	-	-	-	-	SINFRUP00000173118	-	10812	-	-	54752	-	-	-
		-	-	-	-	-	-	-	-	-	-	-	-	189559	-	-	-	-	-	-	-	-	-	-	-	-	-	-	-	-	-	-	-	-	-	-	-	-	-	-	-	-	-	-	-	-	-	-	-	-	-	-
		-	-	-	-	-	-	-	-	-	-	-	-	189557	-	-	-	-	-	-	-	-	-	-	-	-	-	-	-	-	-	-	-	-	-	-	-	-	-	-	-	-	-	-	-	-	-	-	-	-	-	-
		-	-	-	-	-	-	-	-	-	-	-	-	189556	-	-	-	-	-	-	-	-	-	-	-	-	-	-	-	-	-	-	-	-	-	-	-	-	-	-	-	-	-	-	-	-	-	-	-	-	-	-
		-	-	-	-	-	-	-	-	-	BDEG_03451	-	-	189555	-	-	-	-	-	-	-	-	-	-	-	-	-	-	-	79931	-	-	-	-	-	-	-	-	-	-	-	-	-	-	-	-	-	-	-	82578.m00636	-	-
		-	-	-	-	-	-	-	-	-	-	-	-	189554	-	-	-	-	-	-	-	-	-	-	-	-	-	-	-	-	-	-	-	-	-	-	-	-	-	-	-	-	-	-	-	-	-	-	-	-	-	-
CYP743A2	cytochrome P450, unkown function, in CYP711 clan	-	-	-	-	-	-	-	-	-	-	-	-	189550	-	-	-	-	-	-	-	-	-	-	-	-	-	-	-	-	-	-	-	-	-	-	-	-	-	-	-	-	-	-	-	-	-	-	-	-	-	-
		-	+	-	-	-	-	GB11923-PA	AT4G02450.1	23373	BDEG_05161	CE01436	175529	189547	267015	cgd6_1630	CMQ190C	-	DDB0189735	CG16817-PA	-	-	-	GL50803_10429	XP_001133623.1	LmjF34.0210	167297	32066	4677	-	NCU01792	Os08g27070.1	10521	GSPATP00005197001	42757	19585	128125	PF14_0510	205136	RO3G_03344.1	YKL117W	SPAC9E9.13	-	-	3.m01722	2160	TA13455	-	63793	96726.m00094	Tb10.70.5050	UM03938.1
		-	-	-	-	-	-	-	-	-	-	-	-	189529	-	-	-	-	-	-	-	-	-	-	-	-	-	-	-	-	-	-	-	-	-	-	-	-	-	-	-	-	-	-	-	-	-	-	-	-	-	-
		-	-	-	-	-	-	-	-	-	-	-	-	189524	-	-	-	-	-	-	-	-	-	-	-	-	-	-	-	-	-	-	-	-	-	-	-	-	-	-	-	-	-	-	-	-	-	-	-	-	-	-
MAPKKK5	Mitogen Activated Protein Kinase Kinase Kinase Homolog 5	-	-	-	-	-	-	-	-	-	-	-	-	189516	-	-	-	-	-	-	-	-	-	-	-	-	-	-	-	-	-	-	-	-	-	-	-	-	-	-	-	-	-	-	-	-	-	-	-	-	-	-
		-	-	-	-	-	-	-	-	-	-	-	-	189510	-	-	-	-	-	-	-	-	-	-	-	-	-	-	-	-	-	-	-	-	-	92644	-	-	-	-	-	-	-	-	-	-	-	-	-	-	-	-
		-	-	-	-	-	-	-	AT3G19220.1	-	-	-	-	189507	-	-	-	-	-	-	-	-	-	-	-	-	-	-	-	-	-	Os09g28480.1	-	-	-	169975	-	-	201689	-	-	-	-	-	-	-	-	-	-	-	-	-
		-	+	-	-	-	-	-	-	-	-	-	165907	189500	-	-	-	-	-	-	-	-	ENSGALP00000020059	-	-	LmjF19.0500	-	-	-	237264	-	-	-	-	-	95483	-	-	-	-	-	-	-	-	-	-	-	-	53654	-	-	-
		-	-	-	-	-	-	-	-	-	-	-	-	189496	-	-	-	-	-	-	-	-	-	-	-	-	-	-	-	-	-	-	-	-	-	-	-	-	-	-	-	-	-	-	-	-	-	-	-	-	-	-
		-	-	-	-	-	-	-	-	-	-	-	-	189491	-	-	-	-	-	-	-	-	-	-	-	-	-	-	-	-	-	-	-	-	-	-	-	-	-	-	-	-	-	-	-	-	-	-	-	-	-	-
		-	-	-	-	-	-	-	-	-	-	-	-	189490	-	-	-	-	-	-	-	-	-	-	-	-	-	-	-	-	-	-	-	-	-	-	-	-	-	-	-	-	-	-	-	-	-	-	-	-	-	-
		-	-	-	-	-	-	-	-	65507	-	-	-	189488	-	-	-	-	-	-	-	-	-	-	-	-	-	-	-	-	-	-	-	-	-	-	-	PFC0335c	-	-	-	-	-	-	62.m00211	-	-	-	-	-	-	-
		-	-	-	-	-	-	-	-	-	-	-	-	189485	-	-	-	-	-	-	-	-	-	-	-	-	-	-	-	-	-	-	-	-	-	-	-	-	-	-	-	-	-	-	-	-	-	-	-	-	-	-
		-	-	-	-	-	-	-	-	-	-	-	-	189482	-	-	-	-	-	-	-	-	-	-	-	-	-	-	-	-	-	-	-	GSPATP00007326001	-	-	-	-	-	-	-	-	-	-	-	-	-	-	-	-	-	-
		-	-	-	-	-	-	-	-	-	-	-	-	189481	-	-	-	-	-	-	-	-	-	-	-	-	-	-	-	-	-	-	-	-	-	-	-	-	-	-	-	-	-	-	-	-	-	-	-	-	-	-
		-	-	-	-	-	-	-	-	-	-	-	-	189480	-	-	-	-	-	-	-	-	-	-	-	-	-	-	-	-	-	-	-	-	-	-	-	-	-	-	-	-	-	-	-	-	-	-	-	-	-	-
		-	-	-	-	-	-	-	-	-	-	-	-	189478	-	-	-	-	-	-	-	-	-	-	-	-	-	-	-	-	-	-	-	-	-	-	-	-	-	-	-	-	-	-	-	-	-	-	-	-	-	-
		-	-	-	-	-	-	-	AT5G39260.1	-	-	-	-	189476	-	-	-	-	-	-	-	181.m00059	-	-	-	-	-	-	-	-	-	-	36246	-	-	81570	-	-	-	-	-	-	-	-	-	-	-	-	-	-	-	-
		-	-	-	-	-	-	-	-	-	-	-	-	189475	-	-	-	-	-	-	-	-	-	-	-	-	-	-	-	-	-	-	-	-	-	-	-	-	-	-	-	-	-	-	-	-	-	-	-	-	-	-
		-	-	-	-	-	-	-	-	-	-	-	-	189474	-	-	-	-	-	-	-	-	-	-	-	-	-	-	-	-	-	-	-	-	-	-	-	-	-	-	-	-	-	-	-	38351	-	-	-	-	-	-
		-	-	-	-	-	-	-	-	-	-	-	-	189473	-	-	-	-	-	-	-	-	-	-	-	-	-	-	-	-	-	-	-	-	-	-	-	-	-	-	-	-	-	-	-	-	-	-	-	-	-	-
		-	-	-	-	-	-	-	-	-	-	-	-	189472	-	-	-	-	-	-	-	-	-	-	-	-	-	-	-	-	-	-	-	-	-	-	-	-	-	-	-	-	-	-	-	-	-	-	-	-	-	-
		-	-	-	-	-	-	-	-	-	-	-	-	189471	-	-	-	-	-	-	-	-	-	-	-	-	-	-	-	-	-	-	-	-	-	-	-	-	-	-	-	-	-	-	-	-	-	-	-	-	-	-
		-	-	-	-	-	-	-	-	-	-	-	-	189470	-	-	-	-	-	-	-	-	-	-	-	-	-	-	-	-	-	-	-	-	-	-	-	-	-	-	-	-	-	-	-	-	-	-	-	-	-	-
		-	-	-	-	-	-	-	-	-	-	-	-	-	-	-	-	-	-	-	-	-	-	-	-	-	-	-	-	-	-	-	-	-	-	-	-	-	-	-	-	-	-	-	-	-	-	-	-	-	-	-
		-	-	-	-	-	-	-	-	-	-	-	-	189462	-	-	-	-	-	-	-	-	-	-	-	-	-	-	-	124191	-	-	-	-	-	-	-	-	-	-	-	-	-	-	-	-	-	-	-	-	-	-
		-	-	-	-	-	-	-	-	-	-	-	-	189455	-	-	-	-	-	-	-	-	-	-	-	-	-	-	-	-	-	-	-	-	-	-	-	-	-	-	-	-	-	-	-	-	-	-	-	-	-	-
		-	-	-	-	-	-	-	-	-	-	-	-	189454	-	-	-	-	-	-	-	-	-	-	-	-	-	-	-	-	-	-	-	-	-	-	-	-	-	-	-	-	-	-	-	-	-	-	-	-	-	-
		-	+	-	-	-	-	GB12707-PA	AT5G42950.1	17393	BDEG_00780	-	-	189453	-	-	-	OTTDARP00000022626	-	CG11148-PC	-	-	ENSGALP00000040520	-	NP_072096.2	-	171304	-	-	247227	NCU09657	Os07g04530.1	34920	-	43835	170788	127502	-	756442	RO3G_00766.1	YPL105C	SPAC4F10.13c	GLEAN3_25579	SINFRUP00000174076	-	-	-	-	57541	-	-	-
		-	-	-	-	-	-	-	-	-	BDEG_02352	-	-	189452	-	-	-	-	-	-	-	-	-	-	-	-	-	-	-	-	-	-	-	-	-	-	-	-	-	-	-	-	-	-	-	-	-	-	-	-	-	-
		-	-	-	-	-	-	-	-	-	-	-	-	189449	-	-	-	-	-	-	-	-	-	-	-	-	-	-	-	-	-	-	-	-	-	-	-	-	-	-	-	-	-	-	-	-	-	-	-	-	-	-
		-	-	-	-	-	-	-	-	-	-	-	-	189447	-	-	-	-	-	-	-	-	-	-	-	-	-	-	-	-	-	-	-	-	-	-	-	-	-	-	-	-	-	-	-	-	-	-	-	-	-	-
		-	+	-	-	-	-	-	-	-	-	-	226990	189445	294010	-	-	-	-	-	-	-	-	-	OTTHUMP00000183066	-	238557	-	-	219214	-	-	-	GSPATP00012586001	-	-	158827	-	-	-	-	-	-	-	-	-	-	-	-	-	-	-
		-	-	-	-	-	-	-	-	-	-	-	-	-	-	-	-	-	-	-	-	-	-	-	-	-	-	-	-	-	-	-	-	-	-	-	-	-	-	-	-	-	-	-	-	-	-	-	-	-	-	-
		-	-	-	-	-	-	-	-	-	-	-	-	189438	-	-	-	-	-	-	-	-	-	-	-	-	-	-	-	-	-	-	-	-	-	-	-	-	-	-	-	-	-	-	-	-	-	-	-	-	-	-
		-	-	-	-	-	-	-	-	-	-	-	-	189433	-	-	-	-	-	-	-	-	-	-	-	-	-	-	-	-	-	-	-	-	-	-	-	-	-	-	-	-	-	-	-	-	-	-	-	-	-	-
		-	-	-	-	-	-	-	-	-	-	-	-	189432	-	-	-	-	-	-	-	-	-	-	-	-	-	-	-	-	-	-	-	-	-	-	-	-	-	-	-	-	-	-	-	-	-	-	-	-	-	-
CCP1	low-CO2-inducible 36kD chloroplast envelope protein	-	+	-	-	-	-	-	-	-	-	CE32337	-	189430	-	-	-	-	-	-	-	-	ENSGALP00000018274	-	OTTHUMP00000028080	-	234443	17649	-	242628	-	Os10g42299.3	-	GSPATP00009330001	-	196448	157279	-	-	RO3G_10452.1	YPR058W	-	GLEAN3_02927	SINFRUP00000161841	88.m00112	31265	-	-	-	-	-	UM06303.1
		-	-	-	-	-	-	-	-	-	-	-	-	189429	-	-	-	-	-	-	-	-	-	-	-	-	-	-	-	-	-	-	-	-	-	-	-	-	-	-	-	-	-	-	-	-	-	-	-	-	-	-
		-	+	-	-	-	-	-	AT2G46560.1	-	BDEG_02883	-	-	189424	241064	-	-	-	-	-	-	220.m00059	ENSGALP00000036746	-	OTTHUMP00000159140	-	235301	-	-	205527	NCU01658	-	-	GSPATP00016886001	-	-	-	-	816638	-	-	-	-	SINFRUP00000169079	-	-	-	-	54433	-	-	UM00777.1
		-	-	-	-	-	-	-	-	-	-	-	-	189417	-	-	-	-	-	-	-	-	-	-	-	-	-	-	-	-	-	-	-	-	-	-	-	-	-	-	-	-	-	-	-	-	-	-	-	-	-	-
		-	-	-	-	-	-	-	-	-	-	-	-	189416	-	-	-	-	-	-	-	-	-	-	-	-	-	-	-	-	-	-	-	-	-	-	-	-	-	-	-	-	-	-	-	-	-	-	-	-	-	-
		-	-	-	-	-	-	-	-	-	-	-	-	189412	-	-	-	-	-	-	-	-	-	-	-	-	-	-	-	-	-	-	-	-	-	-	-	-	-	-	-	-	-	-	-	-	-	-	-	-	-	-
		-	-	-	-	-	-	-	-	-	-	-	-	189411	-	-	-	-	-	-	-	-	-	-	-	-	-	-	-	-	-	-	-	-	-	-	-	-	-	-	-	-	-	-	-	-	-	-	-	-	-	-
		-	-	-	-	-	-	-	-	-	-	-	-	189410	-	-	-	-	-	-	-	-	-	-	-	-	-	-	-	-	-	-	-	-	-	-	-	-	-	-	-	-	-	-	-	-	-	-	-	-	-	-
		-	+	-	-	-	-	-	AT1G17520.1	-	-	-	-	189408	-	cgd6_4510	-	-	-	-	-	-	-	-	NP_059523.1	-	164310	-	-	-	-	Os01g51154.3	24275	-	48573	27706	155908	-	582938	-	-	-	-	-	-	-	-	-	28428	-	-	UM02326.1
		-	-	-	-	-	-	-	-	-	-	-	-	189407	-	-	-	-	-	-	-	-	-	-	-	-	-	-	-	-	-	-	-	-	-	-	-	-	-	-	-	-	-	-	-	-	-	-	-	-	-	-
		-	+	-	-	-	-	-	AT5G45560.1	19712	-	-	-	189404	-	-	-	-	-	-	-	-	-	-	-	-	-	38896	-	-	-	Os10g31770.1	-	-	10508	151722	136693	-	799812	-	-	-	-	-	-	32430	-	-	-	-	-	-
		-	-	-	-	-	-	GB19549-PA	-	-	-	-	-	189403	-	-	-	-	-	-	-	-	-	-	-	-	-	-	-	-	-	-	-	-	-	-	-	-	-	-	-	-	-	-	-	-	-	-	-	-	-	-
		-	-	-	-	-	-	-	-	-	-	-	-	189401	-	-	-	-	-	-	-	-	-	-	-	-	-	-	-	-	-	-	-	-	-	-	-	-	-	-	-	-	-	-	-	-	-	-	-	-	-	-
		-	-	-	-	-	-	-	-	-	-	-	-	189399	-	-	-	-	-	-	-	-	-	-	-	-	-	-	-	-	-	-	-	-	-	-	-	-	-	-	-	-	-	-	-	-	-	-	-	-	-	-
		-	-	-	-	-	-	-	-	-	-	-	-	189397	-	-	-	-	-	-	-	-	-	-	-	-	-	-	-	-	-	-	-	-	-	-	-	-	-	-	-	-	-	-	-	-	-	-	-	-	-	-
		-	-	-	-	-	-	-	-	-	-	-	-	189395	-	-	-	-	-	-	-	-	-	-	-	-	-	-	-	-	-	-	-	-	-	-	142658	-	-	-	-	-	-	-	-	-	-	-	59686	-	-	-
		-	+	-	-	-	-	-	AT3G58830.1	-	BDEG_06241	-	-	189394	-	-	-	-	DDB0183828	-	-	-	-	-	-	-	-	25411	-	-	NCU01371	Os01g58390.1	-	GSPATP00025758001	6288	115411	-	-	551255	RO3G_05033.1	YHR100C	-	-	-	51.m00248	38164	-	-	-	-	-	UM01705.1
		-	-	-	-	-	-	-	-	-	-	-	-	189393	-	-	CMF039C	-	-	-	-	-	-	-	-	-	-	-	-	-	-	-	-	-	-	-	-	-	-	-	-	-	-	-	-	-	-	-	-	-	-	-
		-	-	-	-	-	-	-	-	-	-	-	-	189392	-	-	-	-	-	-	-	-	-	-	-	-	-	-	-	-	-	-	-	-	-	-	-	-	-	-	-	-	-	-	-	-	-	-	-	-	-	-
		-	-	-	-	-	-	-	-	-	-	-	86160	189391	-	-	-	-	-	-	-	-	-	-	-	-	-	-	-	-	-	-	-	-	-	-	-	-	-	-	-	-	-	-	-	-	-	-	-	-	-	-
		-	-	-	-	-	-	-	-	-	-	-	-	189385	-	-	-	-	-	-	-	-	-	-	-	-	-	-	-	-	-	-	-	-	-	-	-	-	-	-	-	-	-	-	-	-	-	-	-	-	-	-
		-	+	+	-	-	-	GB17571-PA	AT1G17690.1	62634	BDEG_02871	CE20249	118009	189378	271221	cgd7_250	CMH057C	OTTDARP00000021503	DDB0188327	CG3735-PA	-	104.m00165	ENSGALP00000016054	-	OTTHUMP00000107267	LmjF14.0410	138628	16374	78537	145697	NCU01022	Os05g22920.3	21992	-	10544	168559	138940	-	-	RO3G_01539.1	YIL091C	SPCC1827.01c	-	SINFRUP00000135566	102.m00142	36434	TA09740	59.m03547	57324	-	Tb927.7.4340	UM00743.1
		-	-	-	-	-	-	-	-	-	-	-	-	189377	-	-	-	-	-	-	-	-	-	-	-	-	-	-	-	-	-	-	-	-	-	-	-	-	-	-	-	-	-	-	-	-	-	-	-	-	-	-
		-	-	-	-	-	-	-	-	-	-	-	-	189375	-	-	-	-	-	-	-	-	-	-	-	-	-	-	-	-	-	Os01g01070.2	-	-	-	136490	-	-	657115	-	-	-	-	-	-	-	-	-	-	-	-	-
		-	-	-	-	-	-	-	-	-	-	-	-	189374	-	-	-	-	-	-	-	-	-	-	-	-	-	-	-	-	-	-	-	-	-	-	-	-	-	-	-	-	-	-	-	-	-	-	-	-	-	-
		-	+	-	-	-	-	GB16584-PA	AT3G24010.1	-	BDEG_06685	CE20286	225717	189373	228491	cgd7_1740	CMQ358C	OTTDARP00000001411	DDB0186000	CG9293-PB	19069360	-	ENSGALP00000023298	-	OTTHUMP00000018703	-	182053	25212	-	232401	NCU03461	Os03g04980.1	37042	GSPATP00010382001	-	224109	143511	-	712459	RO3G_06130.1	YHR090C	SPBC1709.11c	-	SINFRUP00000140908	3.m01708	-	-	-	50425	93664.m00039	-	UM01188.1
		-	-	-	-	-	-	-	-	-	-	-	-	189370	-	-	-	-	-	-	-	-	-	-	-	-	-	-	-	-	NCU06007	-	-	-	-	-	-	-	-	-	-	-	-	-	-	-	-	-	-	-	-	UM03430.1
RPB169aa		-	+	+	-	-	-	GB19341-PA	AT5G59180.1	59851	BDEG_00125	CE22465	177843	189366	297212	cgd2_2970	CMD026C	OTTDARP00000021673	DDB0218651	-	19069238	27.m00246	-	-	NP_002687.1	LmjF32.1280	212320	31260	74650	244053	NCU02661	Os05g33240.1	8786	GSPATP00000979001	35680	170987	119118	PF10_0269	707797	RO3G_03742.1	YDR404C	SPACUNK4.06c	GLEAN3_18081	SINFRUP00000147569	-	6383	TA19500	59.m00039	50378	84007.m00170	Tb11.01.6090	UM05334.1
		-	+	-	-	-	-	-	AT5G67290.1	29204	-	-	182566	189365	291626	-	-	-	-	-	-	-	ENSGALP00000001528	-	NP_060017.1	-	-	23194	-	-	NCU09535	Os07g06080.1	31934	-	40271	121387	-	-	558391	RO3G_02530.1	YHR009C	SPAC1F5.03c	-	-	-	38723	-	-	50587	-	-	UM05259.1
NDA5	putative type-II NADH dehydrogenase	-	+	-	-	-	-	-	AT5G08740.1	-	-	-	-	189359	-	-	CMM178C	-	-	-	-	-	-	-	-	-	-	-	-	-	-	Os06g11140.1	8383	-	-	133264	-	-	217341	-	-	-	-	-	-	38602	-	-	-	-	-	-
		-	-	-	-	-	-	-	-	-	-	-	-	189351	-	-	-	-	-	-	-	-	-	-	-	-	-	-	-	-	-	-	-	-	-	-	-	-	-	-	-	-	-	-	-	-	-	-	-	-	-	-
		-	-	-	-	-	-	-	-	-	-	-	-	189346	-	-	-	-	-	-	-	-	-	-	-	-	-	-	-	-	-	-	-	-	-	-	-	-	-	-	-	-	-	-	-	-	-	-	-	-	-	-
ETFA	putative electron transfer flavoprotein alpha-subunit , mitochondrial	-	+	-	-	-	-	GB16882-PA	AT1G50940.1	23943	BDEG_00603	CE24926	152823	189344	292461	-	CMS395C	-	DDB0189158	CG8996-PB	-	-	ENSGALP00000004607	-	OTTHUMP00000176507	LmjF28.1140	224100	21090	56308	181047	NCU08004	Os03g61920.1	-	GSPATP00016234001	12373	193847	108676	-	232479	RO3G_11016.1	YPR004C	SPAC27D7.06	GLEAN3_13838	SINFRUP00000138121	5.m00524	41216	-	23.m00312	63743	-	Tb11.02.5530	UM05754.1
		-	-	-	-	-	-	-	-	-	-	-	-	189342	-	-	-	-	-	-	-	-	-	-	-	-	-	-	-	-	-	-	-	-	-	-	-	-	-	-	-	-	-	-	-	-	-	-	-	-	-	-
	putative cellular apoptosis susceptibility protein	-	+	-	-	-	-	GB17150-PA	AT2G46520.1	10095	BDEG_01738	CE27001	165697	189325	294803	-	CMN027C	-	DDB0191603	CG13281-PA	-	-	ENSGALP00000007487	-	OTTHUMP00000043373	LmjF30.3390	205558	35084	79475	185581	NCU04104	Os01g13430.1	15127	-	21667	84750	134104	PFI1590c	664131	RO3G_06017.1	YGL238W	SPBC30B4.05	GLEAN3_19794	SINFRUP00000136811	-	-	TA08950	52.m01606	29181	-	Tb927.6.4740	UM03096.1
		-	+	-	-	-	-	GB19120-PA	-	-	-	CE33036	92293	189321	250081	-	-	OTTDARP00000019461	-	-	-	-	-	-	-	-	163822	-	46318	171792	-	Os06g46670.2	-	-	-	-	-	-	803968	-	-	-	GLEAN3_08910	SINFRUP00000171567	-	-	-	-	19383	-	-	-
		-	-	-	-	-	-	-	-	-	-	-	-	189316	-	-	-	-	-	-	-	-	-	-	-	-	-	-	-	-	-	-	-	-	-	-	-	-	-	-	-	-	-	-	-	-	-	-	-	-	-	-
		-	+	-	-	-	-	-	-	-	-	CE17654	-	189315	-	cgd6_4840	-	-	-	-	-	-	-	-	-	-	224835	-	-	-	-	-	-	-	48262	-	-	PFE0370c	549263	-	-	SPAC22E12.09c	-	-	-	-	-	50.m03259	-	-	-	-
		-	-	-	-	-	-	-	-	-	-	-	-	189308	-	-	-	-	-	-	-	-	-	-	-	-	-	-	-	-	-	-	-	-	-	-	-	-	-	-	-	-	-	-	-	-	-	-	-	-	-	-
		-	-	-	-	-	-	-	-	-	-	-	-	189307	-	-	-	-	-	-	-	-	-	-	-	-	-	-	-	-	-	-	-	-	-	-	-	-	-	-	-	-	-	-	-	3708	-	-	-	-	-	-
		-	-	-	-	-	-	-	-	-	-	-	-	189302	-	-	-	-	-	-	-	-	-	-	-	-	-	-	-	-	-	-	-	-	-	-	-	-	-	-	-	-	-	-	-	-	-	-	-	-	-	-

		-	+	-	-	-	-	-	AT1G67140.1	-	-	-	-	189299	-	-	-	-	DDB0204153	CG2747-PA	-	-	ENSGALP00000016191	-	-	-	-	-	-	-	-	Os06g30894.2	-	-	-	166787	-	-	758926	-	-	-	-	SINFRUP00000182466	-	-	-	-	-	-	-	-
		-	+	-	-	-	-	-	AT5G35690.1	-	BDEG_05414	-	-	189298	-	-	CMF024C	-	-	-	-	-	-	-	-	-	-	31838	-	-	NCU06782	Os08g39150.1	34260	-	48319	109461	131928	-	834957	RO3G_12736.1	-	SPCC1442.07c	-	-	-	21830	-	-	-	-	-	UM06399.1
		-	+	-	-	-	-	-	AT2G42770.1	-	-	-	-	189296	-	-	-	-	-	-	-	-	-	-	-	-	-	-	-	-	-	Os01g53060.1	15248	-	-	132231	-	-	828741	-	-	-	-	-	-	-	-	-	-	-	-	-
		-	-	-	-	-	-	-	-	-	-	-	-	189295	-	-	CMK277C	-	-	-	-	-	-	-	-	-	-	-	-	-	-	-	-	-	44224	-	-	-	-	-	-	-	-	-	-	2613	-	-	-	-	-	-
		-	-	-	-	-	-	-	-	-	-	-	-	189294	-	-	-	-	-	-	-	-	-	-	-	-	-	-	-	-	-	Os09g27150.1	4180	-	-	-	-	-	-	-	-	-	-	-	-	-	-	-	-	-	-	-
		-	+	-	-	-	-	-	-	3016	BDEG_06808	CE32064	123067	189289	206182	-	CMJ263C	OTTDARP00000023705	DDB0218826	CG13771-PA	-	-	ENSGALP00000035845	-	NP_001032248.1	LmjF29.1690	195153	-	81275	181769	-	-	-	GSPATP00025276001	-	-	-	PFB0835c	-	RO3G_02877.1	-	-	GLEAN3_22584	SINFRUP00000135407	39.m00209	-	-	57.m01740	-	-	-	UM06364.1
		-	-	-	-	-	-	-	-	-	-	-	-	189284	-	-	-	-	-	-	-	-	-	-	-	-	-	-	-	-	-	-	-	-	-	-	-	-	-	-	-	-	-	-	-	-	-	-	-	-	-	-
		-	-	-	-	-	-	-	-	-	-	-	-	189283	-	-	-	-	-	-	-	-	-	-	-	-	-	-	-	-	-	-	-	-	-	-	-	-	-	-	-	-	-	-	-	-	-	-	-	-	-	-
		-	-	-	-	-	-	-	-	-	-	-	-	189281	-	-	-	-	-	-	-	-	-	-	-	-	-	-	-	-	-	-	-	-	-	-	-	-	-	-	-	-	-	-	-	-	-	-	-	-	-	-
		-	-	-	-	-	-	-	-	-	-	-	-	189280	-	-	-	-	-	-	-	-	-	-	-	-	-	-	-	-	-	-	-	-	-	-	-	-	-	-	-	-	-	-	-	-	-	-	-	-	-	-
		-	-	-	-	-	-	-	-	-	-	-	-	189279	-	-	-	-	-	-	-	-	-	-	-	-	-	-	-	-	-	-	-	-	-	-	-	-	-	-	-	-	-	-	-	-	-	-	-	-	-	-
		-	-	-	-	-	-	-	-	-	-	-	-	189278	-	-	-	-	-	-	-	-	-	-	-	-	-	-	-	-	-	-	-	-	-	-	-	-	-	-	-	-	-	-	-	-	-	-	-	-	-	-
		-	-	-	-	-	-	-	-	-	-	-	-	189273	-	-	-	-	-	-	-	-	-	-	-	-	-	-	-	-	-	-	-	-	-	-	-	-	-	-	-	-	-	-	-	-	-	-	-	-	-	-
		-	-	-	-	-	-	-	-	-	-	-	-	189271	-	-	-	-	-	-	-	-	-	-	-	-	-	-	-	-	-	-	-	-	-	-	-	-	-	-	-	-	-	-	-	-	-	-	-	-	-	-
		-	-	-	-	-	-	-	-	-	-	-	-	189270	-	-	-	-	-	-	-	-	-	-	-	-	-	-	-	-	-	-	-	-	-	-	-	-	-	-	-	-	-	-	-	-	-	-	-	-	-	-
	plasma membrane calcium-transporting ATPase	-	-	-	+	-	-	-	-	-	-	-	-	189266	-	-	-	-	-	-	-	-	-	-	-	-	-	-	-	-	-	-	-	-	-	-	-	-	-	-	-	-	-	-	-	-	-	-	-	-	-	-
		-	-	-	-	-	-	-	-	-	-	-	-	189264	-	-	-	-	-	-	-	-	-	-	-	-	-	-	-	-	-	-	-	-	-	-	-	-	-	-	-	-	-	-	-	-	-	-	-	-	-	-
		-	-	-	-	-	-	-	-	-	-	-	-	189262	-	-	-	-	-	-	-	-	-	-	-	-	-	-	-	-	-	-	-	-	-	-	-	-	-	-	-	-	-	-	-	-	-	-	-	-	-	-
		-	-	-	-	-	-	-	-	-	-	-	-	189261	-	-	-	-	-	-	-	-	-	-	-	-	-	-	-	-	-	-	-	-	-	-	-	-	-	-	-	-	-	-	-	-	-	-	-	-	-	-
		-	-	-	-	-	-	-	-	-	-	-	-	189258	-	-	-	-	-	-	-	-	-	-	-	-	-	-	-	-	-	-	-	-	-	-	-	-	-	-	-	-	-	-	-	-	-	-	-	-	-	-
		-	+	-	-	-	-	GB15831-PA	AT3G26730.1	-	-	-	178155	189257	-	-	-	-	-	CG12099-PA	-	-	ENSGALP00000011554	-	OTTHUMP00000169457	-	228391	-	-	-	-	Os02g31150.1	35962	-	-	159136	-	-	246332	-	-	SPBP8B7.23	-	-	-	-	-	46.m01710	-	-	-	UM02623.1
		-	-	-	-	-	-	-	-	-	-	-	-	189256	-	-	-	-	-	-	-	-	-	-	-	-	-	-	-	-	-	-	-	-	-	-	-	-	-	-	-	-	-	-	-	-	-	-	-	-	-	-
		-	-	-	-	-	-	-	-	-	-	-	-	189235	-	-	-	-	-	-	-	-	-	-	-	-	-	-	-	-	-	-	-	-	-	-	-	-	-	-	-	-	-	-	-	-	-	-	-	-	-	-
	NUDIX hydrolase family protein	-	+	-	-	-	-	GB19047-PA	-	-	-	CE27911	161300	189231	214393	-	-	-	-	CG4098-PA	-	-	-	-	-	-	117098	-	-	-	-	-	-	-	32661	-	-	-	656751	-	-	-	-	-	-	5223	-	-	-	-	-	-
	ADP-ribose pyrophosphatase	-	-	-	-	-	-	-	-	-	-	-	-	189230	-	-	CMN245C	-	-	-	-	-	-	-	-	-	-	-	70971	-	-	-	-	GSPATP00012752001	-	-	-	-	-	-	-	-	-	-	3.m01662	-	-	-	-	-	-	-
	conserved protein of unknown function	-	-	-	-	-	-	-	-	-	-	-	65671	189217	-	-	-	-	-	-	-	-	-	-	-	-	-	22856	-	52158	-	-	-	-	-	-	-	-	-	-	-	-	-	-	-	-	-	-	52165	-	-	-
RBOL2	putative NADPH oxidase homolog, related to plant respiratory burst oxidase	-	+	-	-	-	-	GB13459-PA	AT4G25090.1	63827	BDEG_07125	CE28463	223007	189216	259986	-	CMG089C	OTTDARP00000020997	-	CG3131-PA	-	-	ENSGALP00000013077	-	OTTHUMP00000023657	LmjF30.1610	180688	37160	51918	28174	NCU10775	Os01g53294.1	-	-	54982	-	-	-	712105	-	YOL152W	-	GLEAN3_00513	SINFRUP00000163660	64.m00192	-	-	-	25818	-	Tb11.02.1990	UM02395.1
RNPH2	3'-5' Exoribonuclease PH component of the Exosome	-	+	+	-	-	-	-	AT3G07750.2	-	BDEG_06503	CE31921	159382	189214	210042	cgd8_340	-	-	DDB0206469	CG8395-PA	-	75.m00188	ENSGALP00000019366	-	NP_055819.1	-	188948	24698	30524	124306	-	Os12g21500.1	12954	-	-	191431	-	MAL13P1.204	834971	RO3G_03646.1	-	-	-	SINFRUP00000158981	-	-	TA06285	20.m05960	55798	81202.m00112	-	UM00460.1
		-	-	-	-	-	-	-	-	-	-	-	-	189210	-	-	-	-	-	-	-	-	-	-	-	-	-	-	-	-	-	-	-	-	-	-	-	-	-	-	-	-	-	-	-	-	-	-	-	-	-	-
		-	-	-	-	-	-	-	-	-	-	-	-	189206	-	-	-	-	-	-	-	-	-	-	-	-	-	-	-	-	-	-	-	-	-	-	-	-	-	-	-	-	-	-	-	-	-	-	-	-	-	-
		-	-	-	-	-	-	-	-	-	-	-	-	189204	-	-	-	-	-	-	-	-	-	-	-	-	-	-	-	-	-	-	-	-	-	-	-	-	-	-	-	-	-	-	-	-	-	-	-	-	-	-
		-	-	-	-	-	-	-	-	-	-	-	-	189197	-	-	-	-	-	-	-	-	-	-	-	-	-	-	50278	-	-	-	-	-	-	-	-	-	-	-	-	-	-	-	-	-	-	-	-	-	-	-
		-	-	-	-	-	-	-	-	-	-	-	-	189196	-	-	-	-	-	-	-	-	-	-	-	-	-	-	-	-	-	-	-	-	-	-	-	-	-	-	-	-	-	-	-	-	-	-	-	-	-	-
FAP146	Flagellar Associated Protein	+	+	-	+	-	-	-	-	4228	BDEG_01138	-	225144	189194	263699	-	-	-	-	-	-	-	ENSGALP00000007782	-	NP_115633.2	LmjF35.4390	123333	34740	29002	184605	-	-	-	GSPATP00027889001	-	73291	127876	-	-	-	-	-	-	-	34.m00257	-	-	583.m09186	57374	94984.m00182	Tb09.211.1790	-
		-	-	-	-	-	-	-	-	-	-	-	-	189189	-	-	-	-	-	-	-	-	-	-	-	-	-	-	-	-	-	-	23722	-	-	159883	135723	-	-	-	-	-	-	-	-	-	-	-	-	-	-	-
SBP1	sedoheptulose-1,7-bisphosphatase	-	+	-	-	-	-	-	AT3G55800.1	-	-	-	-	189186	-	-	CMI196C	-	-	-	-	-	-	-	-	-	-	-	-	-	NCU04483	Os04g16680.1	3614	-	9124	200440	-	-	820389	-	-	-	-	-	142.m00115	261823	-	46.m01668	-	-	Tb927.2.5800	-
FAP53	Flagellar Associated Protein	-	-	-	-	-	-	-	-	-	-	-	-	189183	-	-	-	-	-	-	-	-	-	-	-	-	-	-	-	-	-	-	-	-	-	-	-	-	-	-	-	-	-	-	-	-	-	-	-	-	-	-
		-	-	-	-	-	-	-	-	-	-	-	-	189182	-	-	-	-	-	-	-	-	-	-	-	-	-	-	-	-	-	-	-	-	-	-	-	-	-	-	-	-	-	-	-	-	-	-	-	-	-	-
		-	+	-	-	-	-	-	AT1G07010.1	18391	BDEG_04011	-	-	189179	-	cgd8_290	CMJ015C	-	-	-	-	-	-	-	-	LmjF29.0440	-	-	-	-	-	Os10g25430.1	32574	-	-	3965	-	PF14_0660	557612	RO3G_05581.1	-	SPCC1840.07c	-	-	-	264391	-	-	-	-	Tb927.6.750	UM03316.1
	Putative metacaspase type II	-	+	-	-	-	-	-	AT1G79320.1	-	-	-	-	189175	-	cgd4_4120	-	-	-	-	-	-	-	-	-	-	-	29825	-	-	-	Os01g58580.1	-	-	-	140308	-	-	567122	-	-	-	-	-	-	-	-	-	-	86587.m00089	-	-
		-	-	-	-	-	-	-	-	-	-	-	-	189172	-	-	-	-	-	-	-	-	-	-	-	-	-	-	-	-	-	Os02g03000.2	-	-	-	-	-	-	265643	-	-	-	-	-	-	-	-	-	-	-	-	-
		-	-	-	-	-	-	-	-	-	-	-	-	189171	-	-	-	-	-	-	-	-	-	-	-	-	-	-	-	-	-	-	-	-	-	-	-	-	-	-	-	-	-	-	-	-	-	-	-	-	-	-
		-	+	-	-	-	-	GB16615-PA	-	-	-	CE35686	21604	189165	286466	-	-	-	-	CG4239-PB	-	-	-	-	OTTHUMP00000021849	-	103317	-	-	-	-	-	-	-	-	-	-	-	-	-	-	-	-	SINFRUP00000132043	-	-	-	-	-	-	-	-
PHC19	cell wall protein pherophorin-C19	-	-	-	-	-	-	-	-	-	-	-	-	189163	-	-	-	-	-	-	-	-	-	-	-	-	-	-	-	-	-	-	-	-	-	-	-	-	-	-	-	-	-	-	-	-	-	-	-	-	-	-
		-	-	-	-	-	-	-	-	-	-	-	-	189162	-	-	-	-	-	-	-	-	-	-	-	-	-	-	-	-	-	-	-	-	-	-	-	-	-	-	-	-	-	-	-	-	-	-	-	-	-	-
	Putative chloroplast precursor of a protein of unknown function, not conserved	-	-	-	-	-	-	-	-	-	-	-	-	189153	-	-	-	-	-	-	-	-	-	-	-	-	-	-	-	-	-	-	-	-	-	-	-	-	-	-	-	-	-	-	-	-	-	-	-	-	-	-
	Conserved protein of unknown function	-	+	-	-	-	-	GB18894-PA	AT3G18165.1	-	BDEG_06261	-	171803	189152	297985	-	-	OTTDARP00000023164	-	-	-	-	ENSGALP00000003113	-	OTTHUMP00000013668	-	225373	33609	-	168072	-	Os01g16010.1	-	-	-	232960	132074	PFF0695w	574967	RO3G_09275.1	-	-	GLEAN3_04715	SINFRUP00000179542	-	-	-	-	50080	-	-	UM01682.1
		-	-	-	-	-	-	-	-	-	-	-	-	189148	-	-	-	-	-	-	-	-	-	-	-	-	-	-	-	-	-	-	-	-	-	-	-	-	-	-	-	-	-	-	-	-	-	-	-	-	-	-
LCI31	low-CO2-inducible protein	-	-	-	-	-	-	-	-	-	-	-	-	189142	-	-	-	-	-	-	-	-	-	-	-	-	-	-	-	-	-	Os09g36100.1	-	-	-	173852	-	-	-	-	-	-	-	-	-	-	-	-	-	-	-	-
		-	-	-	-	-	-	-	-	-	-	-	-	189138	-	-	-	-	-	-	-	-	-	-	-	-	-	-	79388	-	-	-	-	GSPATP00005771001	-	-	-	-	-	-	-	-	-	-	-	-	-	-	-	-	-	-
		-	-	-	-	-	-	-	-	-	-	-	-	189135	-	-	-	-	-	-	-	-	-	-	-	-	-	-	-	-	-	-	-	-	-	-	-	-	-	-	-	-	-	-	-	-	-	-	-	-	-	-
		-	-	-	-	-	-	-	-	-	-	-	-	-	-	-	-	-	-	-	-	-	-	-	-	-	-	-	-	-	-	-	-	-	-	-	-	-	-	-	-	-	-	-	-	-	-	-	-	-	-	-
		-	-	-	-	-	-	-	-	-	-	-	-	189127	-	-	-	-	-	-	-	-	-	-	-	-	-	-	-	-	-	-	-	-	-	-	-	-	-	-	-	-	-	-	-	-	-	-	-	-	-	-
		-	-	-	-	-	-	-	-	-	-	-	-	189126	-	-	-	-	-	-	-	-	-	-	-	-	-	-	-	-	-	-	-	-	-	-	-	-	-	-	-	-	-	-	-	-	-	-	-	-	-	-
	hypothetical protein	-	-	-	-	-	-	-	-	-	-	-	-	189125	-	-	-	-	-	-	-	-	-	-	-	-	-	-	-	-	-	-	-	-	-	-	-	-	-	-	-	-	-	-	-	-	-	-	-	-	-	-
		-	-	-	-	-	-	-	-	-	-	-	-	189123	-	-	-	-	-	-	-	-	-	-	-	-	-	-	-	-	-	-	-	-	-	-	-	-	-	-	-	-	-	-	-	-	-	-	-	-	-	-
		-	-	-	-	-	-	-	-	-	-	-	-	189122	-	-	-	-	-	-	-	-	-	-	-	-	-	-	-	-	-	-	-	-	-	-	-	-	-	-	-	-	-	-	-	12050	-	-	-	-	-	-
WNK1	WNK Protein Kinase Homolog 1	-	-	-	-	-	-	-	-	-	-	-	-	189121	-	-	-	-	-	-	-	-	-	-	-	-	-	-	-	-	-	-	-	-	-	-	-	-	-	-	-	-	-	-	-	-	-	-	-	-	-	-
		-	-	-	-	-	-	-	-	-	-	-	-	189120	-	-	-	-	-	-	-	-	-	-	-	-	-	-	-	-	-	-	-	-	-	-	-	-	-	-	-	-	-	-	-	-	-	-	-	-	-	-
		-	-	-	-	-	-	-	-	-	-	-	-	189117	-	-	-	-	-	-	-	-	-	-	-	-	-	-	-	-	-	Os05g05440.1	-	-	-	-	-	-	572763	-	-	-	-	-	-	-	-	-	-	-	-	-
		-	-	-	-	-	-	-	-	-	-	-	-	189113	-	-	-	-	-	-	-	-	-	-	-	-	-	-	-	-	-	-	-	-	-	-	-	-	-	-	-	-	-	-	-	-	-	-	-	-	-	-
		-	-	-	-	-	-	-	-	-	-	-	-	189111	-	-	-	-	-	-	-	-	-	-	-	-	-	-	-	-	-	-	-	-	-	-	128253	-	-	-	-	-	-	-	-	-	-	-	-	-	-	-
FAP59	Conserved Uncharacterized Flagellar Associated Protein	+	+	-	+	-	-	-	-	27516	BDEG_06140	-	178937	189109	210822	-	-	-	-	CG17387-PA	-	-	-	GL50803_95653	NP_852091.1	LmjF31.0230	233267	-	380	-	-	-	-	GSPATP00030401001	-	95235	129172	-	-	-	-	-	-	SINFRUP00000141864	19.m00324	-	-	38.m01031	-	87349.m00194	Tb927.4.4130	-
		-	-	-	-	-	-	-	-	-	-	-	-	189107	-	-	-	-	-	-	-	-	-	-	-	-	-	-	-	-	-	-	-	-	-	-	-	-	-	-	-	-	-	-	-	-	-	-	-	-	-	-
ANK29	protein of unknown function with ankyrin repeats	-	-	-	-	-	-	-	-	-	BDEG_02806	-	-	189100	-	-	-	-	-	-	-	131.m00135	-	-	-	-	-	-	-	-	-	-	-	-	-	-	-	PF10_0102	-	-	-	-	-	-	87.m00164	-	-	-	-	-	-	-
		-	-	-	-	-	-	-	-	-	-	-	-	189093	-	-	-	-	-	-	-	-	-	-	-	-	-	-	-	-	-	-	-	-	-	-	-	-	-	-	-	-	-	-	-	-	-	-	-	-	-	-
		-	-	-	-	-	-	-	-	-	-	-	-	189087	-	-	-	-	-	-	-	-	-	-	-	-	-	-	-	-	-	-	-	-	-	-	-	-	-	-	-	-	-	-	-	-	-	-	-	-	-	-
OFD1	Oral-facial-digital 1	-	-	-	-	-	-	-	-	-	-	-	-	189086	-	-	-	-	-	-	-	-	ENSGALP00000026704	-	-	-	64934	-	-	-	-	-	-	-	-	-	-	-	-	-	-	-	-	SINFRUP00000134932	-	-	-	-	-	-	-	-
ODA3	Outer dynein arm-docking complex subunit 1	-	-	-	+	-	-	-	-	-	-	-	-	189083	-	-	-	-	-	-	-	-	-	-	-	-	-	-	-	-	-	-	-	-	-	-	-	-	-	-	-	-	-	-	-	-	-	-	-	-	-	-
		-	-	-	-	-	-	-	-	71684	-	-	-	189079	-	-	-	-	-	-	-	-	-	-	-	-	-	-	-	-	-	-	-	-	-	-	-	-	-	-	-	-	-	-	-	-	-	-	-	-	-	-
GDPD5	glycerophosphoryl diester phosphodiesterase family protein	-	-	-	-	-	-	-	-	-	-	-	-	189078	-	-	-	-	-	-	-	-	-	-	-	-	-	-	-	-	-	-	-	-	32057	-	-	-	-	-	-	-	-	-	-	23858	-	-	-	-	-	-
FAP47	Flagellar Associated Protein	+	+	-	+	-	-	-	-	61240	-	-	225518	189076	287038	-	-	-	-	-	-	-	ENSGALP00000026181	GL50803_21048	OTTHUMP00000023133	LmjF22.1620	196276	-	-	131745	-	-	-	GSPATP00011857001	-	-	129703	-	-	-	-	-	-	-	62.m00148	-	-	-	56444	93440.m00583	Tb927.6.620	-
		-	-	-	-	-	-	-	-	-	-	-	-	189075	-	-	-	-	-	-	-	-	-	-	-	-	-	-	-	-	-	-	-	-	-	-	-	-	-	-	-	-	-	-	-	-	-	-	-	-	-	-
		-	-	-	-	-	-	-	-	-	-	-	-	189074	-	-	-	-	-	-	-	-	-	-	-	-	-	-	-	-	-	-	-	-	-	-	136731	-	-	-	-	-	-	-	-	-	-	-	-	-	Tb11.02.0340	-
		-	-	-	-	-	-	-	-	-	-	-	-	189072	-	-	-	-	-	-	-	-	-	-	-	-	-	-	-	-	-	-	-	-	-	-	-	-	-	-	-	-	-	-	-	-	-	-	-	-	-	-
		-	-	-	-	-	-	-	-	-	-	-	-	189069	-	-	-	-	-	-	-	-	-	-	-	-	-	-	-	-	-	-	-	-	-	-	-	-	-	-	-	-	-	-	-	-	-	-	-	-	-	-
		-	+	-	-	-	-	GB30165-PA	-	-	-	-	-	189062	-	-	-	-	-	-	-	-	ENSGALP00000034022	-	OTTHUMP00000185021	-	138000	24218	-	134376	-	-	-	-	-	-	-	-	-	-	-	-	GLEAN3_25266	-	-	-	-	-	-	-	-	-
		-	-	-	-	-	-	-	-	-	-	-	-	189059	-	-	-	-	-	-	-	-	-	-	-	-	-	-	-	-	-	-	-	-	-	-	-	-	-	-	-	-	-	-	-	-	-	-	-	-	-	-
		-	-	-	-	-	-	-	-	-	-	-	-	189058	-	-	-	-	-	-	-	-	-	-	-	-	-	-	-	-	-	-	-	-	-	-	-	-	-	-	-	-	-	-	-	-	-	-	-	-	-	-
		-	-	-	-	-	-	-	-	-	-	-	-	189056	-	-	-	-	-	-	-	-	-	-	-	-	-	-	-	-	-	-	-	-	-	-	-	-	-	-	-	-	-	-	-	-	-	-	-	-	-	-
LCI26	low-CO2-induced U-box protein	-	-	-	-	-	-	-	-	-	-	-	-	189052	-	-	-	-	-	-	-	-	-	-	-	-	-	-	-	-	-	-	-	-	-	-	-	-	-	-	-	-	-	-	-	-	-	-	-	-	-	-
		-	-	-	-	-	-	-	-	-	-	-	-	189051	-	-	-	-	-	-	-	-	-	-	-	-	-	-	-	-	-	-	-	-	-	-	-	-	-	-	-	-	-	-	-	-	-	-	-	-	-	-
PROB1	glutamate 5-kinase	-	-	-	-	-	-	GB15049-PB	-	-	-	-	-	189050	-	-	-	-	-	-	-	-	-	-	-	LmjF26.2710	-	-	-	-	-	-	19059	-	-	-	-	-	-	-	-	-	-	-	-	-	-	-	-	-	-	UM00215.1
		-	-	-	-	-	-	-	-	-	-	-	-	189047	-	-	-	-	-	-	-	-	-	-	-	-	-	-	-	-	-	-	-	-	-	-	-	-	-	-	-	-	-	-	-	-	-	-	-	-	-	-
		-	-	-	-	-	-	-	-	-	-	-	-	189041	-	-	-	-	-	-	-	-	-	-	-	-	-	-	-	-	-	-	-	-	-	121780	144400	-	-	-	-	-	-	SINFRUP00000157649	-	22313	-	-	-	-	-	-
		-	-	-	-	-	-	-	-	-	-	-	-	189040	-	-	-	-	-	-	-	-	-	-	-	-	-	-	-	-	-	-	-	-	-	-	-	-	-	-	-	-	-	-	-	-	-	-	-	-	-	-
		-	-	-	-	-	-	-	-	-	-	-	-	189039	-	-	-	-	DDB0187591	-	-	-	-	-	-	-	-	-	61782	-	-	-	-	-	-	-	-	-	-	-	-	-	-	-	-	-	-	-	-	-	-	-
		-	+	-	-	-	-	-	AT1G80950.1	-	-	-	36573	189038	388278	cgd5_1080	CMS008C	OTTDARP00000015110	-	CG32699-PA	-	-	ENSGALP00000021516	GL50803_15987	OTTHUMP00000176148	LmjF29.2410	217653	33064	-	-	-	Os05g28960.2	-	GSPATP00003417001	43099	43926	132558	PFI0695c	591825	RO3G_00654.1	YDL052C	-	GLEAN3_00052	SINFRUP00000149621	37.m00184	263660	-	42.m00024	52571	87719.m00175	Tb927.3.3360	UM06426.1
		-	-	-	-	-	-	-	-	-	-	-	-	189036	-	-	-	-	-	-	-	-	-	-	-	-	-	-	-	-	-	-	-	-	-	-	-	-	-	-	-	-	-	-	-	-	-	-	-	-	-	-
		-	-	-	+	-	-	-	-	-	-	-	-	189032	-	-	-	-	-	-	-	-	-	-	-	-	-	-	-	-	-	-	-	-	-	-	-	-	-	-	-	-	-	-	-	-	-	-	-	-	-	-
		-	-	-	-	-	-	-	-	-	-	-	-	189024	-	-	-	-	-	-	-	-	-	-	-	-	-	-	-	-	-	-	-	-	-	-	-	-	-	-	-	-	-	-	-	-	-	-	-	-	-	-
		-	-	-	-	-	-	-	-	-	-	-	199909	189019	-	-	-	-	-	-	-	-	-	-	-	-	-	-	-	-	-	-	-	-	-	-	-	-	-	-	-	-	-	-	-	-	-	-	-	-	-	-
	Tetraspanning membrane protein, SFT2-like	-	+	-	-	-	-	GB15153-PA	AT5G56020.1	6531	BDEG_03271	CE20520	152187	189017	208141	cgd6_630	-	-	-	CG33635-PA	19171342	-	-	-	-	-	192494	13176	-	89181	NCU02723	Os06g40630.1	-	GSPATP00035119001	42484	159124	139223	PF13_0124	744916	-	YBL102W	SPAC1527.02	-	-	164.m00064	10142	TA17700	641.m02539	57527	-	Tb927.3.2850	UM02349.1
SYP5	Qc-SNARE, Syn8/Syntaxin8-family	-	+	-	-	-	-	-	AT1G16240.3	-	BDEG_00163	-	162904	189016	265183	-	-	-	DDB0187948	CG4109-PA	-	-	ENSGALP00000001877	-	OTTHUMP00000183193	-	205331	39273	68071	-	NCU00242	Os02g02720.3	-	-	-	111312	-	-	818989	RO3G_01754.1	-	-	-	SINFRUP00000153255	-	-	-	-	-	-	-	-
		-	-	-	-	-	-	-	-	-	-	-	-	189008	-	-	-	-	-	-	-	-	-	-	-	-	-	-	-	-	-	-	-	-	-	-	-	-	-	-	-	-	-	-	-	-	-	-	-	-	-	-
		-	-	-	-	-	-	-	-	-	-	-	-	189006	-	-	-	-	-	CG8767-PA	-	-	-	-	-	-	-	-	-	-	-	-	-	-	-	-	-	-	-	-	-	-	-	-	-	-	-	-	-	-	-	-
		-	-	-	-	-	-	-	-	-	-	-	-	188997	-	-	-	-	-	-	-	-	-	-	-	-	-	-	-	-	-	-	-	-	-	-	-	-	-	-	-	-	-	-	-	-	-	-	-	-	-	-
NIFU3	Fe-S cluster assembly protein NifU	-	+	-	-	-	-	-	AT4G01940.1	-	-	-	-	188996	-	-	-	-	-	-	-	-	-	-	-	-	-	-	-	-	-	Os03g20010.1	19144	-	-	17883	-	-	245507	-	-	-	-	-	-	-	-	-	-	-	-	-
		-	-	-	-	-	-	-	-	-	-	-	-	188991	-	-	-	-	-	-	-	-	-	-	-	-	-	-	-	-	-	-	-	-	-	-	-	-	-	-	-	-	-	-	-	-	-	-	-	-	-	-
		-	-	-	-	-	-	-	-	-	-	-	-	188988	-	-	-	-	-	-	-	-	-	-	-	-	-	-	-	-	-	-	-	-	-	-	-	-	-	-	-	-	-	-	-	-	-	-	-	-	-	-
		-	-	-	-	-	-	-	-	-	-	-	-	188986	-	-	-	-	-	-	-	-	-	-	-	-	-	-	-	-	-	-	-	-	-	-	-	-	-	-	-	-	-	-	-	-	-	-	-	-	-	-
		-	-	-	-	-	-	-	-	-	-	-	-	188984	-	-	-	-	-	-	-	-	-	-	-	-	-	-	-	-	-	-	-	-	-	-	-	-	-	-	-	-	-	-	-	-	-	-	-	-	-	-
		-	-	-	-	-	-	-	-	-	-	-	-	188983	-	-	-	-	-	-	-	-	-	-	-	-	-	-	-	-	-	-	-	-	-	-	-	-	-	-	-	-	-	-	-	-	-	-	-	-	-	-
FAP293	Flagellar Associated Coiled-Coil Protein	-	-	-	-	-	-	-	-	-	-	-	-	188982	-	-	-	-	-	-	-	-	-	-	-	-	-	-	-	-	-	-	-	-	-	-	-	-	-	-	-	-	-	-	-	-	-	-	-	-	-	-
		-	+	-	-	-	-	-	AT5G27560.1	-	-	-	-	188978	-	-	-	-	-	-	-	-	-	-	-	-	-	-	-	-	-	Os06g05390.1	34012	-	44529	126332	-	-	570826	-	-	-	-	-	-	3506	-	-	-	-	-	-
MITC11	mitochondrial carrier protein	-	+	-	-	-	-	GB16715-PA	-	72253	BDEG_03131	CE28408	167531	188977	-	-	-	-	DDB0192006	CG4995-PB	-	-	ENSGALP00000011290	-	-	LmjF16.0200	-	38007	29180	210708	-	-	27812	-	-	-	130108	-	740022	-	-	-	GLEAN3_10293	-	-	-	-	-	59821	-	-	-
		-	-	-	-	-	-	-	-	-	-	-	-	188973	-	-	-	-	-	-	-	-	-	-	-	-	-	-	-	-	-	-	-	-	-	-	-	-	-	-	-	-	-	-	-	-	-	-	-	-	-	-
		-	-	-	-	-	-	-	-	-	-	-	-	188972	-	-	-	-	-	-	-	-	-	-	-	-	-	-	-	-	-	-	-	-	-	-	-	-	-	-	-	-	-	-	-	-	-	-	-	-	-	-
		-	-	-	-	-	-	-	-	-	-	-	-	188970	-	-	-	-	-	-	-	4.m00647	-	-	-	-	-	-	-	-	-	-	-	-	-	-	-	-	-	-	-	-	-	-	-	-	-	-	-	-	-	-
		-	-	-	-	-	-	-	-	-	-	-	-	188967	-	-	-	-	-	-	-	-	-	-	-	-	-	-	-	-	-	-	-	-	-	-	-	-	-	-	-	-	-	-	-	-	-	-	-	-	-	-
		-	+	-	-	-	-	-	-	29808	BDEG_00082	-	167098	188966	-	-	-	-	-	-	-	-	ENSGALP00000033213	-	OTTHUMP00000033540	-	-	-	-	-	-	-	14365	GSPATP00015007001	-	178834	-	-	200388	-	-	-	GLEAN3_22765	SINFRUP00000182129	-	-	-	-	-	-	-	-
FAP81	Flagellar Associated Protein	+	+	-	+	-	-	-	-	67097	BDEG_06675	-	224255	188960	260744	-	-	-	-	-	-	-	ENSGALP00000009344	-	NP_031363.2	-	238689	-	-	240209	-	-	-	GSPATP00017437001	-	76639	158307	-	-	-	-	-	-	SINFRUP00000152272	7.m00361	-	-	-	54907	-	-	-
FAP108	Flagellar Associated Protein	-	-	-	+	-	-	-	-	-	-	-	-	188958	-	-	-	-	-	-	-	-	-	-	-	-	-	-	-	-	-	-	-	-	-	-	-	-	-	-	-	-	-	-	-	-	-	-	-	-	-	-
		-	+	-	-	-	-	GB10430-PA	-	62531	-	CE34543	171745	188957	375964	-	-	-	DDB0167543	CG2924-PC	-	279.m00074	ENSGALP00000004723	-	OTTHUMP00000035378	-	234840	14240	81521	126045	-	-	33287	GSPATP00031850001	48512	168742	-	-	-	-	-	-	-	SINFRUP00000149644	-	-	-	-	21996	96423.m00238	-	-
		-	-	-	-	-	-	-	-	-	-	-	-	188952	-	-	-	-	-	-	-	-	-	-	-	-	-	-	-	-	-	-	-	-	-	-	-	-	-	-	-	-	-	-	-	-	-	-	-	-	-	-
		-	-	-	-	-	-	-	-	-	-	-	-	188950	-	-	-	-	-	-	-	-	-	-	-	-	-	-	-	-	-	-	-	-	-	-	-	-	-	-	-	-	-	-	-	-	-	-	-	-	-	-
PPRC2		-	-	-	-	-	-	-	AT4G34830.1	-	-	-	-	188949	-	-	-	-	-	-	-	-	-	-	-	-	-	-	-	-	-	Os10g10170.4	-	-	-	77778	-	-	195861	-	-	-	-	-	-	-	-	-	-	-	-	-
		-	-	-	-	-	-	-	-	-	-	-	-	188947	-	-	-	-	-	-	-	-	-	-	-	-	-	-	-	-	-	-	-	-	-	-	-	-	-	-	-	-	-	-	-	-	-	-	-	-	-	-
		-	-	-	-	-	-	-	-	61076	-	-	-	188945	-	-	-	-	-	-	-	-	ENSGALP00000005246	-	-	-	-	-	-	-	NCU00955	-	-	-	-	-	-	-	-	-	-	-	-	-	-	-	-	-	-	-	-	-
IF4A	Similar to Eukaryotic Initiation Factor 4A	-	+	+	-	-	-	-	AT3G13920.1	-	BDEG_07613	CE01341	170898	188942	208641	cgd1_880	CMK028C	OTTDARP00000016449	DDB0190216	CG9075-PD	-	3.m00565	ENSGALP00000038935	GL50803_10255	OTTHUMP00000173930	LmjF01.0770	207101	-	80026	236625	NCU07420	Os02g05330.1	28625	-	25743	233331	109443	PF14_0655	825619	RO3G_02301.1	YKR059W	SPAC1006.07	-	-	-	9716	TA11470	50.m05680	-	93465.m00063	Tb09.160.3270	UM05482.1
C_190071		-	-	-	-	-	-	-	-	-	-	-	-	188940	-	-	-	-	-	-	-	-	-	-	-	-	-	-	-	-	-	-	-	-	-	-	-	-	-	-	-	-	-	-	-	-	-	-	-	-	-	-
		-	-	-	-	-	-	-	-	-	-	-	-	188939	-	-	-	-	DDB0203302	-	-	-	-	-	-	-	-	-	-	-	-	-	-	-	-	-	128149	-	-	RO3G_06949.1	-	-	-	-	-	-	-	-	-	-	-	-
		-	-	-	-	-	-	-	-	24822	-	-	-	188937	-	-	-	-	-	-	-	-	-	-	-	-	-	-	-	-	-	-	-	-	-	-	-	-	-	-	-	-	-	-	-	-	-	-	-	-	-	-
		-	-	-	-	-	-	-	-	-	-	-	-	188936	-	-	-	-	-	-	-	-	-	-	-	-	-	-	-	-	-	-	-	-	-	-	-	-	-	-	-	-	-	-	-	-	-	-	-	-	-	-
TUG		-	+	+	-	-	-	GB18755-PA	AT3G61650.1	23795	BDEG_04849	CE00224	174186	188933	-	cgd7_1980	CMN304C	-	DDB0216898	CG17566-PA	19173393	55.m00198	ENSGALP00000004979	GL50803_114218	OTTHUMP00000181218	LmjF25.0960	237817	21602	56069	173802	NCU03954	Os05g06450.2	25029	GSPATP00001868001	44225	170153	109415	PF08_0125	755367	RO3G_11100.1	YLR212C	SPBC32F12.04	GLEAN3_20943	SINFRUP00000138209	5.m00548	29237	TA03470	42.m00121	49610	83992.m00196	Tb927.3.910	UM03803.1
		-	-	-	-	-	-	-	-	-	-	-	-	188931	-	-	-	-	-	-	-	-	-	-	-	-	-	-	-	-	-	-	-	-	-	-	-	-	-	-	-	-	-	-	-	-	-	-	-	-	-	-
		-	+	-	-	-	-	-	AT3G23790.1	-	-	-	-	188925	-	-	CMT459C	-	-	-	-	-	-	-	-	-	-	-	-	-	-	Os03g62850.2	-	-	12420	140960	-	-	174716	-	-	-	-	-	-	262925	-	-	-	-	-	-
		-	+	-	-	-	-	-	AT2G19640.2	-	-	-	-	188924	-	-	-	-	-	CG33548-PB	-	-	-	-	-	-	-	-	-	-	-	Os08g10470.2	-	-	-	4377	-	-	179471	RO3G_08888.1	-	SPCC1739.05	-	-	-	-	-	-	-	-	Tb10.70.3640	-
PIA1	N-acetylglucosaminyl-phosphatidylinositol de-N-acetylase-like protein	-	+	+	-	-	-	GB19790-PA	AT2G27340.4	55121	BDEG_00166	-	144788	188921	209304	cgd7_700	-	OTTDARP00000018838	DDB0191808	-	-	52.m00150	ENSGALP00000007144	-	OTTHUMP00000065644	LmjF09.0040	85239	-	33046	157870	NCU09510	Os04g58210.1	5600	GSPATP00019956001	34610	145059	122221	PFF1190c	421744	-	YMR281W	SPAPB2B4.01c	-	SINFRUP00000137631	56.m00256	8003	TA10880	55.m04741	-	-	Tb11.01.3900	UM00302.1
		-	-	-	-	-	-	-	-	-	-	-	-	188920	-	-	-	-	-	-	-	-	-	-	-	-	-	-	-	-	-	-	-	-	-	-	-	-	-	-	-	-	-	-	-	-	-	-	-	-	-	-
		-	+	-	-	-	-	-	AT4G16530.1	-	-	-	-	188919	-	-	-	-	-	-	-	-	-	-	-	-	-	-	-	-	-	Os10g36340.2	-	GSPATP00010227001	-	169315	-	PFC0605w	671245	-	-	-	-	-	125.m00099	-	TA07155	113.m00795	-	-	-	-
		-	-	-	-	-	-	-	-	-	-	-	-	188918	-	-	-	-	-	-	-	-	-	-	-	-	-	-	-	-	-	-	-	-	-	-	-	-	-	-	-	-	-	-	-	-	-	-	-	-	-	-
		-	-	-	-	-	-	-	-	-	-	-	-	188913	-	-	-	-	-	-	-	-	-	-	-	-	-	-	-	-	-	-	-	-	-	-	-	-	-	-	-	-	-	-	-	-	-	-	-	-	-	-
		-	+	-	-	-	-	GB19244-PA	-	-	BDEG_00093	-	221508	188912	-	-	-	-	-	-	-	-	-	-	NP_116200.1	-	-	-	63072	208393	-	-	-	-	-	169043	-	-	-	RO3G_07359.1	-	-	-	SINFRUP00000147049	-	-	-	-	-	84404.m00100	-	-
		-	-	-	-	-	-	-	-	-	-	-	-	188910	-	-	-	-	-	-	-	-	-	-	-	-	-	-	-	-	-	-	-	-	-	-	-	-	-	-	-	-	-	-	-	-	-	-	-	-	-	-
		-	-	-	-	-	-	-	-	-	-	-	-	188909	-	-	-	-	-	-	-	-	-	-	-	-	-	-	-	-	-	-	-	-	-	-	-	-	-	-	-	-	-	-	-	-	-	-	-	-	-	-
		-	-	-	-	-	-	-	-	-	-	-	-	188905	-	-	-	-	-	-	-	-	-	-	-	-	-	-	-	-	-	-	-	-	-	-	-	-	-	-	-	-	-	-	-	-	-	-	-	-	-	-
		-	-	-	-	-	-	-	-	-	-	-	-	188904	-	-	-	-	-	-	-	-	-	-	-	-	-	-	-	-	-	-	-	-	-	-	-	-	-	-	-	-	-	-	-	-	-	-	-	-	-	-
		-	-	-	-	-	-	-	-	-	-	-	-	188900	-	-	-	-	-	-	-	-	-	-	-	-	-	-	-	-	-	-	-	-	-	-	-	-	766891	-	-	-	-	-	-	-	-	-	-	-	-	-
		-	-	-	-	-	-	-	-	-	-	-	-	188899	-	-	-	-	-	-	-	-	-	-	-	-	-	-	-	-	-	-	-	-	-	-	-	-	-	-	-	-	-	-	-	-	-	-	-	-	-	-
		-	+	-	-	-	-	GB13164-PA	AT4G32630.1	61788	BDEG_00951	-	225407	188892	-	cgd5_140	-	OTTDARP00000022393	DDB0189354	CG3365-PB	-	82.m00165	-	GL50803_2834	OTTHUMP00000164242	LmjF36.3150	184772	-	-	92915	-	Os05g45840.2	23511	-	49833	166640	133668	-	730096	-	-	-	-	SINFRUP00000158849	-	24184	-	-	55214	91931.m00032	Tb11.01.1230	-
		-	-	-	-	-	-	-	-	-	-	-	-	188889	-	-	-	-	-	-	-	-	-	-	-	-	-	-	-	-	-	-	-	-	-	170354	-	-	-	-	-	-	-	-	-	-	-	-	-	-	-	-
		-	-	-	-	-	-	-	-	-	-	-	-	188887	-	-	-	-	-	-	-	-	-	-	-	-	-	-	-	90077	-	-	-	-	-	-	-	-	-	-	-	-	-	-	-	-	-	-	-	86485.m00546	-	-
		-	-	-	-	-	-	-	-	-	-	-	-	188886	-	-	-	-	-	-	-	-	-	-	-	-	-	-	-	-	-	-	-	-	-	-	-	-	-	-	-	-	-	-	-	-	-	-	-	-	-	-
FAP16	Conserved Uncharacterized Flagellar Associated Protein, Similar to Echinoderm Microtubule Binding Protein	-	-	-	+	-	-	-	-	-	-	-	-	188885	-	-	-	-	-	-	-	-	-	-	-	-	-	-	-	-	-	-	-	-	-	-	-	-	-	-	-	-	-	-	-	-	-	-	-	-	-	-
		-	-	-	-	-	-	-	-	-	-	-	-	188878	-	-	-	-	-	-	-	-	-	-	-	-	-	-	-	-	-	-	-	-	-	-	-	-	-	-	-	-	-	-	-	-	-	-	-	-	-	-
		-	-	-	-	-	-	-	-	-	-	-	-	188874	-	-	-	-	-	-	-	-	-	-	-	-	-	-	-	-	-	-	-	-	-	-	-	-	-	-	-	-	-	-	-	-	-	-	-	-	-	-
	PI-4-kinase-like	-	+	-	-	-	-	GB19870-PA	-	21311	BDEG_00054	CE39249	-	188872	-	cgd8_4500	CMI125C	-	DDB0187963	CG7004-PC	-	-	ENSGALP00000039753	-	OTTHUMP00000014283	LmjF34.3590	129853	34568	71866	20498	NCU10397	Os11g10420.1	21854	GSPATP00027213001	16364	147576	137537	PFE0485w	595372	-	YNL267W	SPAC22E12.16c	-	SINFRUP00000138339	18.m00398	31223	TA05835	86.m00397	21411	-	Tb927.4.1140	-
		-	-	-	-	-	-	-	-	-	-	-	-	188868	-	-	-	-	-	-	-	-	-	-	-	-	-	-	-	-	-	-	-	-	-	-	-	-	-	-	-	-	-	-	-	-	-	-	-	-	-	-
		-	-	-	-	-	-	-	-	-	-	-	-	188867	-	-	-	-	-	-	-	-	-	-	-	-	-	-	-	-	-	-	-	-	-	-	-	-	-	-	-	-	-	-	-	-	-	-	-	-	-	-
		-	-	-	-	-	-	-	-	-	-	-	-	188858	-	-	-	-	-	-	-	-	-	-	-	-	-	-	-	-	-	-	-	-	-	-	-	-	-	-	-	-	-	-	-	-	-	-	-	-	-	-
FAP290	Flagellar Associated Protein	-	+	-	-	-	-	-	AT4G26470.1	-	-	-	-	188857	-	-	-	-	-	-	-	-	-	-	-	-	-	-	-	-	-	Os02g10470.1	-	-	-	142899	141832	-	829970	-	-	-	-	-	-	-	-	-	-	-	-	-
		-	-	-	-	-	-	-	-	-	-	-	-	188853	-	-	-	-	-	-	-	-	-	-	-	-	-	-	-	-	-	-	-	-	-	-	-	-	-	-	-	-	-	-	-	-	-	-	-	-	-	-
		-	-	-	-	-	-	-	-	-	-	-	-	188849	-	-	-	-	-	-	-	-	-	-	-	-	-	-	-	-	-	-	-	-	-	-	-	-	-	-	-	-	-	-	-	-	-	-	-	-	-	-
		-	-	-	-	-	-	-	-	-	-	-	-	188847	-	-	-	-	-	-	-	-	-	-	-	-	-	-	-	-	-	-	-	-	-	-	-	-	-	-	-	-	-	-	-	-	-	-	-	-	-	-
	protein of unknown function with conserved domain COG3868	-	-	-	-	-	-	-	-	-	-	-	-	188845	-	-	-	-	-	-	-	-	-	-	163670	-	-	-	NCU05136	-	-	-	-	-	112963	-	-	-	-	-	-	-	55.m00188	-	-	-	-	-	-	-

		-	-	-	-	-	-	-	-	-	-	-	-	188843	-	-	-	-	-	-	-	-	-	-	-	-	-	-	-	-	-	-	-	-	-	-	-	-	-	-	-	-	-	-	-	-	-	-	-	-	-	-
		-	-	-	-	-	-	-	AT4G17070.1	-	-	-	-	188842	-	-	-	-	-	-	-	-	-	-	-	-	-	-	-	-	-	Os03g01090.1	-	-	-	-	-	-	589929	-	-	-	-	-	-	-	-	-	-	-	-	-
RPS4	Ribosomal protein S4, component of cytosolic 80S ribosome and 40S small subunit	-	+	+	-	-	-	GB19305-PA	AT2G17360.1	69604	BDEG_06287	CE24278	152725	188837	395147	cgd7_4760	CMH218C	OTTDARP00000020826	DDB0168933	CG11276-PB	19173414	331.m00054	ENSGALP00000007705	GL50803_11359	OTTHUMP00000023537	LmjF13.1230	210271	33368	82706	173646	NCU02181	Os02g01560.1	29689	GSPATP00017030001	17324	197071	132408	PF11_0065	257929	RO3G_14840.1	YJR145C	SPBC19F8.08	GLEAN3_22607	SINFRUP00000128667	12.m00363	27292	TA10515	25.m00221	37748	91860.m00077	Tb11.02.1090	UM02714.1
		-	-	-	-	-	-	-	-	-	-	-	-	188828	-	-	-	-	-	-	-	-	-	-	-	-	-	-	-	-	-	-	-	-	-	-	-	-	-	-	-	-	-	-	-	-	-	-	-	-	-	-
		-	-	-	-	-	-	-	-	-	-	-	-	188820	-	-	-	-	-	-	-	-	-	-	-	-	-	-	-	-	-	-	-	-	-	-	-	-	-	-	-	-	-	-	-	-	-	-	-	-	-	-
		-	-	-	-	-	-	-	-	-	-	-	-	188816	-	-	-	-	-	-	-	-	-	-	-	-	-	-	-	-	-	-	-	-	-	-	-	-	-	-	-	-	-	-	-	-	-	-	-	-	-	-
		-	-	-	-	-	-	-	-	-	-	-	-	188815	-	-	-	-	-	-	-	-	-	-	-	-	-	-	-	-	-	-	-	-	-	-	-	-	-	-	-	-	-	-	-	-	-	-	-	-	-	-
SUFE	cysteine desulfuration protein	-	+	-	-	-	-	-	AT4G26500.1	14776	-	-	-	188814	-	-	CMS131C	-	-	-	-	-	-	-	-	-	-	-	-	154215	-	Os09g09790.1	25212	-	7639	24633	-	PFB0270w	550633	-	-	-	-	-	-	36605	-	-	-	-	-	-
		-	-	-	-	-	-	-	-	-	-	-	-	188811	-	-	-	-	-	-	-	-	-	-	-	-	-	-	-	-	-	-	-	-	-	-	-	-	-	-	-	-	-	-	-	-	-	-	-	-	-	-
		-	-	-	-	-	-	-	-	-	-	-	-	188807	-	-	-	-	-	-	-	-	-	-	-	-	-	-	-	-	-	-	-	-	-	77522	-	-	-	-	-	-	-	-	-	-	-	-	-	-	-	-
		-	-	-	-	-	-	-	-	-	-	-	-	188806	-	-	-	-	-	-	-	-	-	-	-	-	-	-	-	-	-	-	-	-	-	-	-	-	-	-	-	-	-	-	-	-	-	-	-	-	-	-
		-	-	-	-	-	-	-	-	-	-	-	-	188805	-	-	-	-	-	-	-	-	-	-	-	-	-	-	-	-	-	-	-	-	-	-	-	-	-	-	-	-	-	-	-	-	-	-	-	-	-	-
		-	-	-	-	-	-	-	-	-	-	-	-	188804	-	-	-	-	-	-	-	-	-	-	-	-	-	-	-	-	-	-	-	-	-	-	-	-	-	-	-	-	-	-	-	-	-	-	-	-	-	-
		-	-	-	-	-	-	-	-	-	-	-	-	188803	-	-	-	-	-	-	-	-	-	-	-	-	-	-	-	-	-	-	-	-	-	-	-	-	-	-	-	-	-	-	-	-	-	-	-	-	-	-
		-	-	-	-	-	-	-	-	-	-	-	-	188795	-	-	-	-	-	-	-	-	-	-	-	-	-	-	-	-	-	-	-	-	-	-	-	-	-	-	-	-	-	-	-	-	-	-	-	-	-	-
		-	-	-	-	-	-	-	-	-	-	-	-	188787	-	-	-	-	-	-	-	-	-	-	-	-	-	-	-	-	-	-	-	-	-	-	-	-	-	-	-	-	-	-	-	-	-	-	-	-	-	-
RIR2A	ribonucleoside-diphosphate reductase small chain	-	+	-	-	-	-	GB13424-PA	AT3G27060.1	65685	BDEG_02254	CE00874	162025	188785	295696	cgd6_690	CML050C	OTTDARP00000005724	DDB0217423	CG8975-PA	19074323	-	ENSGALP00000025808	-	NP_001025.1	LmjF27.2050	178434	15279	56348	165579	NCU07887	Os06g03720.1	8886	GSPATP00027296001	17523	91828	109432	PF14_0053	742835	RO3G_12051.1	YJL026W	SPBC25D12.04	GLEAN3_24933	SINFRUP00000153067	20.m00438	32555	TA06715	23.m00001	-	-	Tb11.v4.0006	UM06368.1
		-	-	-	-	-	-	-	-	-	-	-	-	188782	-	-	-	-	-	-	-	-	-	-	-	-	-	-	-	-	-	-	-	-	-	-	-	-	-	-	-	-	-	-	-	-	-	-	-	-	-	-
		-	-	-	-	-	-	-	-	-	-	-	-	188780	-	-	-	-	-	-	-	-	-	-	-	-	-	-	-	-	-	-	-	-	-	-	-	-	-	-	-	-	-	-	-	-	-	-	-	-	-	-
		-	-	-	-	-	-	-	-	-	-	-	-	188778	-	-	-	-	-	-	-	-	-	-	-	-	-	-	-	-	-	-	-	-	-	-	-	-	-	-	-	-	-	-	-	-	-	-	-	-	-	-
		-	-	-	-	-	-	-	-	-	-	-	-	188768	-	-	-	-	-	-	-	-	-	-	-	-	-	-	-	-	-	-	-	-	-	-	-	-	-	-	-	-	-	-	-	-	-	-	-	-	-	-
OTC1		-	+	-	-	-	-	GB11109-PA	AT1G75330.1	33293	-	-	178077	188762	-	-	CML214C	-	-	-	-	-	ENSGALP00000026160	GL50803_10311	OTTHUMP00000024343	-	238192	11184	-	182282	NCU01667	Os02g47590.1	9328	-	30514	142289	155409	-	206001	RO3G_11443.1	YJL088W	SPAC4G9.10	GLEAN3_13209	SINFRUP00000176157	-	997	-	-	-	93088.m00129	-	UM03537.1
		-	-	-	-	-	-	-	-	-	-	-	-	188757	-	-	-	-	-	-	-	-	-	-	-	-	-	-	-	-	-	-	-	-	-	-	-	-	-	-	-	-	-	-	-	-	-	-	-	-	-	-
CDS1b		-	+	-	-	-	-	-	AT2G45150.3	-	-	-	-	188754	-	-	CMS056C	-	-	-	-	133.m00142	-	-	-	LmjF32.2870	-	-	70855	-	-	Os02g03460.1	16773	GSPATP00023276001	47250	134556	156385	-	-	-	-	-	-	-	78.m00188	30747	-	55.m05088	-	-	-	-
		-	-	-	-	-	-	-	-	-	-	-	-	188753	-	-	-	-	-	-	-	-	-	-	-	-	-	-	-	-	-	-	-	-	-	-	-	-	-	-	-	-	-	-	-	-	-	-	-	-	-	-
		-	-	-	-	-	-	-	-	-	-	-	-	188752	-	-	-	-	-	-	-	-	-	-	-	-	-	-	-	-	-	-	-	-	-	-	-	-	-	-	-	-	-	-	-	-	-	-	-	-	-	-
		-	-	-	-	-	-	-	-	-	-	-	-	188751	-	-	-	-	-	-	-	-	-	-	-	-	-	-	-	-	-	-	-	-	-	-	-	-	-	-	-	-	-	-	-	-	-	-	-	-	-	-
		-	-	-	-	-	-	-	-	-	-	-	-	188750	-	-	-	-	-	-	-	-	-	-	-	-	-	-	-	-	-	-	-	-	-	-	-	-	-	-	-	-	-	-	-	-	-	-	-	-	-	-
		-	-	-	-	-	-	-	-	-	-	-	-	188743	-	-	-	-	-	-	-	-	-	-	-	-	-	-	-	-	-	-	-	-	-	-	-	-	-	-	-	-	-	-	-	-	-	-	-	-	-	-
		-	-	-	-	-	-	-	-	-	-	-	-	188742	-	-	-	-	-	-	-	-	-	-	-	-	-	-	-	-	-	-	-	-	-	-	-	-	-	-	-	-	-	-	-	-	-	-	-	-	-	-
		-	-	-	-	-	-	-	-	-	-	-	-	188737	-	-	-	-	-	-	-	-	-	-	-	-	-	-	-	-	-	-	-	-	-	-	-	-	-	-	-	-	-	-	-	-	-	-	-	-	-	-
		-	-	-	-	-	-	-	-	-	-	-	-	188732	-	-	-	-	-	-	-	-	-	-	-	-	-	-	-	-	-	-	-	GSPATP00037150001	-	-	-	-	-	-	-	-	-	-	-	-	-	-	-	-	-	-
		-	+	-	-	-	-	-	AT4G21790.1	5107	-	-	-	188728	-	-	-	-	DDB0169013	-	-	-	-	-	-	-	-	-	-	-	-	Os03g36790.1	36997	-	-	222595	157086	-	832788	-	-	-	-	-	-	-	-	-	-	-	-	-
		-	-	-	-	-	-	-	-	-	-	-	-	188723	-	-	-	-	-	-	-	-	-	-	-	-	-	-	-	-	-	-	-	-	-	-	-	-	-	-	-	-	-	-	-	-	-	-	-	-	-	-
FAP271	Flagellar Associated Protein	-	+	-	-	-	-	-	-	-	-	-	164999	188722	-	-	-	-	-	-	-	-	-	-	OTTHUMP00000003335	-	118149	-	47401	191297	-	-	-	GSPATP00013718001	-	-	-	-	-	-	-	-	-	-	3.m01840	-	-	-	58238	-	-	-
		-	-	-	-	-	-	-	-	-	-	-	-	188721	-	-	-	-	-	-	-	-	-	-	-	-	-	-	-	-	-	-	-	-	-	-	-	-	-	-	-	-	-	-	-	-	-	-	-	-	-	-
		-	-	-	-	-	-	-	-	-	-	-	-	188712	-	-	-	-	-	-	-	-	-	-	-	-	-	-	-	-	-	-	-	-	-	-	-	-	-	-	-	-	-	-	-	-	-	-	-	-	-	-
		-	+	-	-	-	-	-	AT5G06550.1	26320	BDEG_00835	-	-	188711	-	-	-	-	DDB0190735	CG7200-PA	-	-	-	-	-	LmjF27.1320	-	1419	1858	-	NCU07419	Os11g36450.1	16032	-	46234	139383	138187	-	256136	-	-	-	-	-	-	14890	-	76.m01561	-	-	Tb11.46.0007	UM00799.1
		-	-	-	-	-	-	-	-	-	-	-	-	188710	-	-	-	-	-	-	-	-	-	-	-	-	-	-	-	-	-	-	-	-	-	-	-	-	-	-	-	-	-	-	-	-	-	-	-	-	-	-
		-	+	-	-	-	-	GB18406-PA	AT5G63440.2	-	BDEG_07388	CE28806	225003	188706	393318	-	-	-	-	-	-	-	ENSGALP00000014077	-	NP_071384.1	-	184723	-	-	168680	-	Os07g19470.1	24501	GSPATP00015214001	-	215900	-	-	823412	-	-	-	-	-	100.m00155	-	-	59.m06118	53219	-	-	-
		-	-	-	-	-	-	-	-	-	-	-	-	188695	-	-	-	-	-	-	-	-	-	-	-	-	-	-	-	-	-	-	-	-	-	-	-	-	-	-	-	-	-	-	-	-	-	-	-	-	-	-
		-	-	-	-	-	-	-	-	-	-	-	-	188694	-	-	-	-	-	-	-	-	-	-	-	-	-	-	-	-	-	-	-	-	-	-	-	-	-	-	-	-	-	-	-	-	-	-	-	-	-	-
		-	-	-	-	-	-	-	-	-	-	-	-	188692	-	-	-	-	-	-	-	-	-	-	-	-	-	-	-	-	-	-	-	-	-	-	-	-	-	-	-	-	-	-	-	-	-	-	-	-	-	-
		-	+	-	-	-	-	GB10290-PA	-	-	BDEG_07542	CE18697	183656	188689	271428	-	-	OTTDARP00000004011	DDB0219317	CG18659-PA	-	249.m00080	ENSGALP00000020939	-	NP_056504.3	LmjF35.2960	116963	-	61939	100263	-	-	36275	-	-	-	144560	-	-	RO3G_11350.1	-	-	-	SINFRUP00000132590	-	-	-	-	53305	92247.m00077	-	-
		-	-	-	-	-	-	-	-	-	-	-	-	188686	-	-	-	-	-	-	-	-	-	-	-	-	-	-	-	-	-	-	-	-	-	-	-	-	-	-	-	-	-	-	-	-	-	-	-	-	-	-
		-	-	-	-	-	-	-	-	-	-	-	-	188685	-	-	-	-	-	-	-	-	-	-	-	-	-	-	-	-	-	-	-	-	-	-	-	-	-	-	-	-	-	-	-	-	-	-	-	-	-	-
		-	-	-	-	-	-	-	-	-	-	-	-	188684	-	-	-	-	-	-	-	-	-	-	-	-	-	-	-	-	-	-	-	-	-	-	-	-	-	-	-	-	-	-	-	-	-	-	-	-	-	-
		-	-	-	-	-	-	-	-	-	-	-	-	188672	-	-	-	-	-	-	-	-	-	-	-	-	-	-	-	-	-	-	-	-	-	-	-	-	-	-	-	-	-	-	-	-	-	83.m01275	-	-	-	-
		-	+	-	-	-	-	GB15652-PA	AT3G15355.1	7275	-	CE37602	195632	188669	-	-	-	-	DDB0217536	CG6303-PA	-	-	ENSGALP00000017138	-	NP_057336.3	-	230508	22898	-	103523	-	Os09g12310.1	-	-	48883	155339	-	-	235877	-	-	-	GLEAN3_01262	SINFRUP00000165083	-	-	-	-	26979	-	-	UM00775.1
VTC1	Vacuolar Transport Chaperone-like	-	+	-	-	-	-	-	-	-	BDEG_08140	-	-	188668	-	-	-	-	DDB0187617	-	-	-	-	-	-	LmjF14.1040	-	-	-	-	NCU03883	-	-	-	15281	-	-	-	-	RO3G_11287.1	YER072W	SPBC21B10.04c	-	-	-	34498	-	125.m00072	-	-	Tb927.7.3900	UM05595.1
		-	-	-	-	-	-	-	AT5G11260.1	-	-	-	-	188667	-	-	-	-	DDB0205824	-	-	-	-	-	-	-	-	-	-	34679	-	-	-	-	-	-	-	-	-	-	-	-	-	-	-	-	-	-	-	-	-	-
		-	-	-	-	-	-	-	-	-	-	-	-	188662	-	-	-	-	-	-	-	-	-	-	-	-	-	-	-	-	-	-	-	-	-	-	-	-	-	-	-	-	-	-	-	-	-	-	-	-	-	-
MLT1	multi-eye protein	-	-	-	-	-	-	-	-	-	-	-	-	188661	-	-	-	-	-	-	-	-	-	-	-	-	-	-	-	-	-	-	-	-	-	-	-	-	-	-	-	-	-	-	-	-	-	-	-	-	-	-
		-	-	-	-	-	-	-	-	-	-	-	-	188658	-	-	-	-	-	-	-	-	-	-	-	-	-	-	-	-	-	-	-	-	-	-	-	-	-	-	-	-	-	-	-	-	-	-	-	-	-	-
		-	-	-	-	-	-	-	-	-	-	-	-	188654	-	-	-	-	-	-	-	-	-	-	-	-	-	-	-	-	-	-	-	-	-	-	-	-	-	-	-	-	-	-	-	-	-	-	-	-	-	-
		-	-	-	-	-	-	-	-	-	-	-	-	188649	-	-	-	-	-	-	-	-	-	-	-	-	-	-	-	-	-	Os02g51700.1	-	-	-	-	-	-	751689	-	-	-	-	-	-	-	-	-	-	-	-	-
EYE2	thioredoxin superfamily protein required for eyespot assembly	-	-	-	-	-	-	-	-	-	-	-	-	188648	-	-	-	-	-	-	-	-	-	-	-	-	-	-	-	-	-	-	-	-	-	-	-	-	-	-	-	-	-	-	-	-	-	-	-	-	-	-
		-	-	-	-	-	-	-	-	-	-	-	-	188646	-	-	-	-	-	-	-	-	-	-	-	-	-	-	-	-	-	-	-	-	-	104164	134708	-	-	-	-	-	-	-	-	-	-	-	-	-	-	-
		-	-	-	-	-	-	-	-	-	-	-	-	188643	-	-	-	-	-	-	-	-	-	-	-	-	-	-	-	-	-	-	-	-	-	-	-	-	-	-	-	-	-	-	-	-	-	-	-	-	-	-
		-	+	-	-	-	-	-	-	-	-	-	222011	188638	-	-	CMO029C	-	-	CG11784-PA	-	-	-	-	-	-	-	-	-	80855	-	-	34472	-	-	-	-	-	828962	-	YLL060C	-	-	-	164.m00105	261582	-	-	-	-	-	-
		-	-	-	-	-	-	-	-	-	-	-	-	188630	-	-	-	-	-	-	-	-	-	-	-	-	-	-	-	-	-	-	-	-	-	-	-	-	-	-	-	-	-	-	-	-	-	-	-	-	-	-
		-	-	-	-	-	-	-	-	-	-	-	-	188627	-	-	-	-	-	-	-	-	-	-	-	-	-	-	-	-	-	-	-	-	-	-	-	-	-	-	-	-	-	-	-	-	-	-	-	-	-	-
		-	-	-	-	-	-	-	-	-	-	-	-	188621	-	-	-	-	-	-	-	-	-	-	-	-	-	-	-	-	-	-	-	-	-	-	-	-	-	-	-	-	-	-	-	-	-	-	-	-	-	-
		-	-	-	-	-	-	-	-	-	-	-	-	188620	-	-	-	-	-	-	-	-	-	-	-	-	-	-	-	-	-	-	-	-	-	-	-	-	-	-	-	-	-	-	-	-	-	-	-	-	-	-
		-	-	-	-	-	-	-	-	-	-	-	-	-	-	-	-	-	-	-	-	-	-	-	-	-	-	-	-	-	-	-	-	-	-	-	-	-	-	-	-	-	-	-	-	-	-	-	-	-	-	-
		-	-	-	-	-	-	-	-	67084	-	-	-	188616	-	-	-	-	-	-	-	-	-	-	-	-	-	-	-	-	-	-	-	-	-	71504	-	-	-	-	-	-	-	-	-	-	-	-	-	-	Tb10.70.2530	-
ODA6	Flagellar outer dynein arm intermediate chain 2	-	+	-	+	-	-	GB12459-PA	-	34917	BDEG_01316	-	219457	188612	297913	cgd1_1540	-	-	DDB0215245	CG6053-PA	-	-	ENSGALP00000007149	GL50803_6939	NP_075462.2	LmjF32.1060	110559	39146	79232	186877	-	-	-	GSPATP00028934001	46969	-	108289	PF14_0243	-	RO3G_09754.1	-	-	GLEAN3_06699	SINFRUP00000138996	5.m00520	40529	-	52.m01629	20628	88271.m00391	Tb11.01.5910	-
CPN23	Chaperonin 23	-	+	-	-	-	-	-	-	-	-	-	-	188610	-	-	CMS188C	-	-	-	-	-	-	-	-	-	-	-	-	-	-	Os02g54060.1	21325	-	16537	115042	-	-	-	-	-	-	-	-	-	263181	-	59.m03713	-	93865.m00193	-	-
		-	-	-	-	-	-	-	-	-	-	-	-	188603	-	-	-	-	-	-	-	-	-	-	-	-	-	-	-	-	-	-	-	-	-	-	-	-	-	-	-	-	-	-	-	-	-	-	-	-	-	-
		-	+	-	-	-	-	-	AT5G16120.1	-	-	-	-	188602	-	-	CMS228C	-	DDB0169489	-	-	-	-	-	-	-	-	33890	66550	-	-	Os07g37840.1	23731	-	34489	117597	-	PFL2530w	225495	-	-	-	-	-	33.m00198	263538	-	541.m02064	-	-	-	-
		-	-	-	-	-	-	-	-	-	-	-	-	188598	-	-	-	-	-	-	-	-	-	-	-	-	-	-	-	-	-	-	-	-	-	-	-	-	-	-	-	-	-	-	-	-	-	-	-	-	-	-
		-	-	-	-	-	-	-	-	-	-	-	-	188595	-	-	-	-	-	-	-	-	-	-	-	-	-	-	-	-	-	-	-	-	-	-	-	-	-	-	-	-	-	-	-	-	-	-	-	-	-	-
MCS	2-C-methyl-D-erythritol 2,4-cyclodiphosphate synthase, putative chloroplast precursor	-	+	-	-	-	-	-	AT1G63970.1	21780	-	-	91535	188593	-	-	CMT435C	-	-	-	-	-	-	-	-	-	-	-	-	-	-	Os02g45660.1	6306	-	12330	150209	-	PFB0420w	413403	-	-	-	-	-	192.m00046	37585	TA04155	55.m04628	-	-	-	-
		-	-	-	-	-	-	-	-	-	-	-	-	188591	-	-	-	-	-	-	-	-	-	-	-	-	-	-	-	-	-	-	-	-	-	-	-	-	-	-	-	-	-	-	-	-	-	-	-	-	-	-
		-	+	-	-	-	-	GB16305-PA	AT1G29990.1	17056	BDEG_05455	CE05680	78564	188584	380446	cgd3_1200	-	OTTDARP00000023734	DDB0186836	CG7770-PA	-	-	-	GL50803_112870	OTTHUMP00000015282	LmjF05.1200	210667	38649	5238	248362	NCU06941	Os02g14860.1	23207	GSPATP00026924001	24783	191469	-	-	714512	RO3G_04931.1	YLR200W	SPAC3A11.13	-	-	6.m00517	11508	TA21500	50.m00044	53348	89461.m00063	Tb927.5.580	UM00870.1
	hypothetical conserved protein	-	+	-	-	-	-	-	-	61568	-	-	-	188583	215169	-	-	OTTDARP00000006165	-	-	-	-	-	-	OTTHUMP00000017103	LmjF35.4870	115288	-	-	-	-	-	-	GSPATP00005559001	-	-	132117	-	-	-	-	-	-	-	324.m00016	-	-	-	-	89789.m00072	-	-
		-	+	-	-	-	-	-	AT2G40620.1	-	-	-	-	188572	-	-	-	-	-	-	-	-	-	-	-	-	-	-	-	-	-	Os08g43090.1	-	-	-	45185	140472	-	411874	-	-	-	-	-	-	-	-	-	-	-	-	-
		-	-	-	-	-	-	-	-	-	-	-	-	-	-	-	-	-	-	-	-	-	-	-	-	-	-	-	-	-	-	-	-	-	-	-	-	-	-	-	-	-	-	-	-	-	-	-	-	-	-	-
		-	-	-	-	-	-	-	-	-	-	-	-	188566	-	-	-	-	-	-	-	-	-	-	-	-	-	-	-	-	-	-	-	-	-	-	-	-	-	-	-	-	-	-	-	-	-	-	-	-	-	-
TRM2A	tRNA (uracil-5-)-methyltransferase	-	+	-	-	-	-	-	AT3G21300.1	60367	-	-	-	188565	-	-	CMO215C	-	-	-	-	209.m00112	-	-	-	-	-	-	-	62479	NCU06780	Os01g09750.2	-	-	16911	213266	-	-	264925	-	-	-	-	-	-	36322	-	-	-	-	-	UM03063.1
RNB2	3'-5' Exoribonuclease II	-	+	-	-	-	-	-	AT5G02250.1	-	BDEG_07459	-	-	188559	-	-	-	-	-	-	-	-	-	-	-	-	-	-	-	-	-	Os01g62440.1	19329	-	43677	120849	-	-	819168	RO3G_14013.1	-	SPCC1322.01	-	-	-	21580	-	-	-	-	-	-
		-	-	-	-	-	-	-	-	-	-	-	-	188558	-	-	-	-	-	-	-	-	-	-	-	-	-	-	-	-	-	-	-	-	-	-	-	-	-	-	-	-	-	-	-	-	-	-	-	-	-	-
		-	-	-	-	-	-	-	-	-	-	-	-	188557	-	-	-	-	-	-	-	-	-	-	NP_002208.3	-	126343	-	-	-	-	-	-	-	-	-	-	-	-	-	-	-	GLEAN3_12005	-	-	-	-	-	53363	-	-	-
		-	-	-	-	-	-	-	-	-	-	-	-	188555	-	-	-	-	-	-	-	-	-	-	-	-	-	-	-	-	-	-	-	-	-	-	-	-	-	-	-	-	-	-	-	-	-	-	-	-	-	-
		-	-	-	-	-	-	-	-	-	-	-	-	188553	-	-	-	-	-	-	-	-	-	-	-	-	-	-	-	-	-	-	-	-	-	-	-	-	-	-	-	-	-	-	-	-	-	-	-	-	-	-
	hypothetical protein	-	-	-	-	-	-	-	-	-	-	-	-	188547	-	-	-	-	-	-	-	-	-	-	-	-	-	-	-	-	-	-	-	-	-	-	-	-	-	-	-	-	-	-	-	-	-	-	-	-	-	-
		-	-	-	-	-	-	-	-	-	-	-	-	188545	-	-	-	-	-	-	-	-	-	-	-	-	-	-	-	-	-	-	-	-	-	-	-	-	-	-	-	-	-	-	-	-	-	-	-	-	-	-
CHR3421		-	-	-	-	-	-	-	AT2G46020.2	-	-	-	-	188544	-	-	-	-	-	-	-	-	-	-	-	-	-	-	-	-	-	Os02g02290.1	-	-	-	160727	-	-	-	-	-	-	-	-	-	-	-	-	-	-	-	-
		-	-	-	-	-	-	-	-	-	-	-	-	188538	-	-	-	-	-	-	-	-	-	-	-	-	-	-	-	-	-	-	-	-	-	-	-	-	-	-	-	-	-	-	-	-	-	-	-	-	-	-
		-	-	-	-	-	-	-	-	-	-	-	-	188534	-	-	-	-	-	-	-	-	-	-	-	-	-	-	-	-	-	-	-	-	-	-	-	-	-	-	-	-	-	-	-	-	-	-	-	-	-	-
		-	+	-	-	-	-	-	-	-	BDEG_00111	-	-	188533	-	-	-	-	DDB0167375	-	-	253.m00082	-	-	-	-	-	-	80462	-	NCU00813	-	-	-	-	-	-	PF11_0055	-	RO3G_10695.1	-	-	-	-	-	-	TA12705	-	-	-	-	UM05352.1
		-	-	-	-	-	-	-	-	-	-	-	-	188532	-	-	-	-	-	-	-	-	-	-	-	-	-	-	-	-	-	-	-	-	-	-	-	-	-	-	-	-	-	-	-	-	-	-	-	-	-	-
		-	-	-	-	-	-	-	-	-	-	-	-	188531	-	-	-	-	-	-	-	-	-	-	-	-	-	-	-	-	-	-	-	-	-	-	-	-	-	-	-	-	-	-	-	-	-	-	-	-	-	-
		-	-	-	-	-	-	-	-	-	-	-	-	188530	-	-	-	-	-	-	-	-	-	-	-	-	-	-	-	-	-	-	-	-	-	-	-	-	-	-	-	-	-	-	-	-	-	-	-	-	-	-
		-	-	-	-	-	-	-	-	-	-	-	-	188528	-	-	-	-	-	-	-	-	-	-	-	-	-	-	-	-	-	-	-	-	-	-	-	-	-	-	-	-	-	-	-	-	-	-	-	-	-	-
		-	-	-	-	-	-	-	-	-	-	-	-	188524	-	-	-	-	-	-	-	-	-	-	-	-	-	-	-	-	-	-	-	-	-	-	-	-	-	-	-	-	-	-	-	-	-	-	-	-	-	-
		-	-	-	-	-	-	-	-	-	-	-	-	188522	-	-	-	-	-	-	-	-	-	-	-	-	-	-	-	-	-	-	-	-	-	-	-	-	-	-	-	-	-	-	-	-	-	-	-	-	-	-
		-	-	-	-	-	-	-	-	-	-	-	-	188520	-	-	-	-	-	-	-	-	-	-	-	-	-	-	-	-	-	-	-	-	-	-	-	-	-	-	-	-	-	-	-	-	-	-	-	-	-	-
		-	-	-	-	-	-	-	-	-	-	-	-	188519	-	-	-	-	-	-	-	-	-	-	-	-	-	-	-	-	-	-	-	-	-	-	-	-	-	-	-	-	-	-	-	-	-	-	-	-	-	-
	hypothetical protein	-	-	-	-	-	-	-	-	-	-	-	-	188513	-	-	-	-	-	-	-	-	-	-	-	-	-	-	-	-	-	-	-	-	-	-	-	-	-	-	-	-	-	-	-	-	-	-	-	-	-	-
		-	+	-	-	-	-	-	AT4G17150.1	2040	-	-	-	188508	-	cgd7_370	-	-	-	-	-	-	-	-	-	LmjF30.0400	-	-	2048	-	-	Os03g24450.1	-	GSPATP00014945001	14054	135289	143370	MAL8P1.38	768762	-	-	-	-	-	149.m00046	269163	-	59.m03580	-	96809.m00035	Tb927.6.1810	-
		-	-	-	-	-	-	-	-	-	-	-	-	188502	-	-	-	-	-	-	-	-	-	-	-	-	-	-	-	-	-	-	-	-	-	-	-	-	-	-	-	-	-	-	-	-	-	-	-	-	-	-
		-	-	-	-	-	-	-	-	-	-	-	-	188500	-	-	-	-	-	-	-	-	-	-	-	-	-	-	-	-	-	-	-	-	-	-	-	-	-	-	-	-	-	-	-	-	-	-	-	-	-	-
		-	-	-	-	-	-	-	-	61379	-	-	-	188496	-	-	-	-	-	-	-	-	-	-	-	-	-	-	-	-	-	-	36272	-	-	-	-	-	-	-	-	-	-	-	-	-	-	-	-	-	-	-
		-	-	-	-	-	-	-	-	-	-	-	-	188478	-	-	-	-	-	-	-	-	-	-	-	-	-	-	-	-	-	-	-	-	-	139285	-	-	576861	-	-	-	-	-	-	-	-	-	-	-	-	-
PST1	phosphoserine aminotransferase	-	+	+	-	-	-	GB19078-PA	AT4G35630.1	-	BDEG_02044	CE09710	173627	188476	292882	-	-	-	DDB0190692	-	-	30.m00263	ENSGALP00000024450	-	OTTHUMP00000021524	-	192615	18531	80405	206542	NCU01429	Os03g06200.1	25325	GSPATP00030058001	-	129784	-	-	208206	RO3G_03375.1	YOR184W	SPAC1F12.07	GLEAN3_20138	SINFRUP00000136191	45.m00243	14979	-	38.m00011	38236	83680.m00049	-	UM05531.1
		-	-	-	-	-	-	-	-	-	-	-	-	188474	-	-	-	-	-	-	-	-	-	-	-	-	-	-	-	-	-	-	-	-	-	-	-	-	-	-	-	-	-	-	-	-	-	-	-	-	-	-
		-	-	-	-	-	-	-	-	-	-	-	-	188470	-	-	-	-	-	-	-	-	-	-	-	-	-	-	-	-	-	-	-	-	-	-	-	-	-	-	-	-	-	-	-	-	-	-	-	-	-	-
		-	-	-	-	-	-	-	-	-	-	-	-	188468	-	-	-	-	-	-	-	-	-	-	-	-	-	-	-	-	-	-	-	-	-	-	-	-	-	-	-	-	-	-	-	-	-	-	-	-	-	-
		-	-	-	-	-	-	-	-	-	-	-	-	188467	286232	-	-	-	-	-	-	-	-	-	-	-	-	27601	-	-	-	-	-	-	-	-	-	-	-	-	-	-	-	-	-	-	-	-	-	-	-	-
		-	+	-	-	-	-	-	AT3G62810.1	-	-	-	-	188466	-	-	-	-	-	-	-	-	-	-	-	-	232124	-	-	247745	-	-	-	-	-	205054	-	-	834878	-	-	-	-	-	-	-	-	-	-	-	-	UM00665.1
		-	+	-	-	-	-	-	-	59716	-	-	-	188464	-	-	-	-	-	-	-	-	-	-	-	-	-	-	-	-	-	-	-	-	33535	163336	-	-	-	-	YDL244W	-	-	-	-	6708	-	-	-	-	-	-
		-	-	-	-	-	-	-	-	-	-	-	-	188457	-	-	-	-	-	-	-	-	-	-	-	-	-	-	-	-	-	-	-	-	-	-	-	-	-	-	-	-	-	-	-	-	-	-	-	-	-	-
		-	-	-	-	-	-	-	-	-	-	-	-	188455	-	-	-	-	-	-	-	-	-	-	-	-	-	-	-	-	-	-	-	-	-	-	-	-	-	-	-	-	-	-	-	-	-	-	-	-	-	-
		-	-	-	-	-	-	-	-	-	-	-	-	188454	-	-	-	-	-	-	-	-	-	-	-	-	-	-	-	-	-	-	-	-	-	-	-	-	-	-	-	-	-	-	-	-	-	-	-	-	-	-
		-	+	-	-	-	-	-	AT3G24050.1	-	BDEG_00393	-	-	188447	-	-	CMD180C	-	DDB0169282	-	-	-	-	-	-	-	-	-	-	-	-	Os10g32070.1	23721	-	-	34911	-	-	814881	RO3G_03309.1	-	SPCC16A11.14	-	-	-	-	-	-	-	-	-	UM04076.1
		-	-	-	-	-	-	-	-	-	-	-	-	188445	389780	-	-	-	-	-	-	-	-	-	-	-	-	-	-	-	-	-	-	-	-	-	-	-	-	-	-	-	-	-	-	-	-	-	-	-	-	-
		-	+	-	-	-	-	GB11458-PA	AT3G47630.2	-	BDEG_06406	CE28809	92307	188432	273799	-	CMR095C	-	DDB0217881	-	-	-	ENSGALP00000007893	-	OTTHUMP00000170246	-	217112	31806	64006	99622	NCU04429	Os11g34840.1	23898	GSPATP00009982001	49252	193881	141672	-	179583	RO3G_08364.1	YGR046W	SPBC1A4.06c	-	SINFRUP00000164067	35.m00322	-	-	583.m11391	4385	-	-	UM05612.1
		-	+	-	-	-	-	-	AT1G14300.1	-	BDEG_04146	-	84156	188430	-	-	-	-	DDB0169019	-	-	-	-	-	OTTHUMP00000178432	-	226261	32489	-	243614	-	Os10g26660.1	34348	-	48410	107769	140084	-	745075	RO3G_10948.1	YGR187C	SPAC26F1.12c	-	SINFRUP00000143656	111.m00104	-	-	-	19950	96660.m00060	-	-
TOM7	Translocase of outer membrane TOM7, mitochondrial	-	-	-	-	-	-	-	-	-	-	-	-	188428	-	-	-	-	-	-	-	-	-	-	-	-	-	-	-	-	-	-	-	-	-	-	-	-	-	-	-	-	-	-	-	-	-	-	-	-	-	-
		-	-	-	-	-	-	-	-	-	-	-	-	188423	-	-	-	-	-	-	-	-	-	-	-	-	-	-	-	-	-	-	-	-	-	-	-	-	-	-	-	-	-	-	-	-	-	-	-	-	-	-
		-	-	-	-	-	-	-	-	-	-	-	-	188421	-	-	-	-	-	-	-	-	-	-	-	-	-	-	-	-	-	-	-	-	-	-	-	-	-	-	-	-	-	-	-	-	-	-	-	-	-	-
		-	-	-	-	-	-	-	-	-	-	-	-	188420	-	-	-	-	-	-	-	-	-	-	-	-	-	-	-	-	-	-	-	-	-	-	-	-	-	-	-	-	-	-	-	-	-	-	-	-	-	-
		-	-	-	-	-	-	-	-	-	-	-	-	188416	-	-	-	-	-	-	-	-	-	-	-	-	-	-	-	-	-	-	-	-	-	-	-	-	-	-	-	-	-	-	-	-	-	-	-	-	-	-
		-	+	-	-	-	-	GB17757-PA	AT5G52980.1	-	-	-	191829	188413	-	-	-	-	-	-	-	-	ENSGALP00000038976	-	OTTHUMP00000180858	-	98317	-	-	-	-	Os02g13200.1	-	-	-	112214	-	-	712673	-	-	-	-	-	-	-	-	-	60361	-	-	-
		-	-	-	-	-	-	-	-	-	-	-	-	188412	-	-	-	-	-	-	-	-	-	-	-	-	-	-	-	-	-	-	-	-	-	-	-	-	-	-	-	-	-	-	-	-	-	-	-	-	-	-
RPT5	26S proteasome regulatory subunit	-	+	+	-	-	-	GB14678-PA	AT3G05530.1	70378	BDEG_06993	CE17915	225374	188404	298952	cgd2_1350	CMP294C	OTTDARP00000020733	DDB0186002	-	19069393	4.m00585	ENSGALP00000037708	GL50803_4365	NP_002795.2	LmjF22.0620	207066	35277	59859	244951	NCU04414	Os06g07630.1	8530	GSPATP00023663001	9090	144700	108207	PF11_0314	834472	RO3G_03248.1	YOR117W	SPAC3A11.12c	GLEAN3_03847	SINFRUP00000158943	10.m00552	516	TA21405	145.m00347	49668	81529.m00529	Tb927.7.2550	-
		-	-	-	-	-	-	-	-	-	-	-	-	188402	-	-	-	-	-	-	-	-	-	-	-	-	-	-	-	-	-	-	32855	-	48371	-	-	-	-	-	-	-	-	-	-	7292	-	-	-	-	-	-
GAT1	Glutamyl tRNA amidotransferase, subunit A	-	+	-	-	-	-	GB17721-PA	AT3G25660.1	11143	-	CE27621	114717	188401	295143	-	CMM318C	OTTDARP00000022808	DDB0187169	CG7900-PA	-	-	ENSGALP00000024707	-	OTTHUMP00000016923	LmjF16.1360	174160	25148	34997	96904	NCU00660	Os04g55050.1	22712	GSPATP00019396001	13890	220555	136763	PFD0780w	262031	RO3G_03393.1	YMR293C	SPBC646.03	GLEAN3_01311	SINFRUP00000138844	159.m00087	263203	TA11065	-	25932	-	Tb927.8.5080	UM04505.1
		-	+	-	-	-	-	GB20101-PB	-	-	BDEG_06075	CE08527	-	188400	-	cgd5_3050	-	-	-	CG4893-PA	-	-	-	GL50803_14748	-	-	-	-	-	-	-	-	-	-	-	-	-	-	-	-	-	-	-	-	-	-	TA07545	55.m08188	54943	-	-	-
		-	-	-	-	-	-	-	-	-	-	-	-	188399	-	-	-	-	-	-	-	-	-	-	-	-	-	-	-	-	-	-	-	-	-	-	-	-	-	-	-	-	-	-	-	-	-	-	-	-	-	-
		-	-	-	-	-	-	-	-	-	-	-	-	188398	-	-	-	-	-	-	-	-	-	-	-	-	-	-	-	-	-	-	-	-	-	-	-	-	-	-	-	-	-	-	-	-	-	-	-	-	-	-
		-	-	-	-	-	-	GB13983-PA	-	-	-	-	-	188395	-	-	-	-	-	-	-	-	-	-	-	-	-	-	-	-	-	-	-	-	-	-	-	-	-	-	-	-	-	-	-	-	-	-	-	-	-	-
		-	-	-	-	-	-	-	-	-	-	-	-	188389	-	-	-	-	-	-	-	-	-	-	-	-	-	-	-	-	-	-	-	-	-	-	-	-	-	-	-	-	-	-	-	-	-	-	-	-	-	-
CSP41a	chloroplast RNA binding protein	-	+	-	+	-	-	-	AT3G63140.1	27287	-	-	-	188387	-	-	-	-	-	-	-	-	-	-	-	-	-	-	-	-	-	Os07g11110.1	36792	-	-	105954	-	-	830112	-	-	-	-	-	-	21300	-	-	-	-	-	-
		-	-	-	-	-	-	-	-	-	-	-	-	-	-	-	-	-	-	-	-	-	-	-	-	-	-	-	-	-	-	-	-	-	-	-	-	-	-	-	-	-	-	-	-	-	-	-	-	-	-	-
		-	-	-	-	-	-	-	AT4G25800.2	-	-	-	-	188379	-	-	-	-	-	-	-	-	-	-	-	-	-	-	-	-	-	-	-	-	-	-	-	-	825902	-	-	-	-	-	-	-	-	-	-	-	-	-
		-	-	-	-	-	-	-	-	-	-	-	-	188378	-	-	-	-	-	-	-	-	-	-	-	-	-	-	-	-	-	-	-	-	-	-	-	-	-	-	-	-	-	-	-	-	-	-	-	-	-	-
GPX4	Glutathione peroxidase, possibly chloroplastic	-	+	-	-	-	-	-	-	-	-	-	172308	188373	-	-	-	-	-	-	-	-	-	-	-	-	86983	16837	-	-	-	-	-	-	-	-	-	-	-	-	-	-	-	-	159.m00055	-	-	-	63884	-	Tb927.7.1140	-
	expressed protein of unknown function	-	-	-	-	-	-	-	-	-	-	-	-	188372	-	-	-	-	-	-	-	-	-	-	-	-	-	-	-	-	-	-	-	-	-	-	-	-	-	-	-	-	-	-	-	-	-	-	-	-	-	-
		-	-	-	-	-	-	-	-	67879	-	-	-	188371	-	-	-	-	-	-	-	-	-	-	-	-	-	-	-	-	-	-	-	-	-	-	-	-	-	-	-	-	-	-	-	-	-	-	-	-	-	-
GPIT1	GPI (glycosyl phosphatidyl inositol) transamidase subunit	-	+	-	-	-	-	-	AT3G07140.1	-	-	-	-	188365	-	-	-	-	-	-	-	-	-	-	OTTHUMP00000031110	-	128945	-	-	-	-	Os11g28980.1	-	-	-	200018	-	-	-	-	-	-	GLEAN3_24540	-	-	-	-	-	-	-	-	-
		-	-	-	-	-	-	-	-	-	-	-	-	188363	-	-	-	-	-	-	-	-	-	-	-	-	-	-	-	-	-	-	-	-	-	-	-	-	-	-	-	-	-	-	-	-	-	-	-	-	-	-
		-	-	-	-	-	-	-	-	-	-	-	-	188358	-	-	-	-	-	-	-	-	-	-	-	LmjF32.1660	-	-	46181	-	-	-	-	-	-	-	138694	-	-	-	-	-	-	-	-	-	-	-	-	-	-	-
		-	-	-	-	-	-	-	-	-	-	-	-	188353	-	-	-	-	-	-	-	-	-	-	-	-	-	-	-	-	-	-	-	GSPATP00014922001	-	-	-	-	-	-	-	-	-	-	-	-	-	-	-	-	-	-
		-	-	-	-	-	-	-	-	-	-	-	-	188351	-	-	-	-	-	-	-	-	-	-	-	-	-	-	-	-	-	-	-	-	-	-	-	-	-	-	-	-	-	-	-	-	-	-	-	-	-	-
LSM2		-	+	+	-	-	-	GB13993-PA	AT1G03330.1	33162	BDEG_02288	CE37990	111414	188349	387478	cgd7_1070	CMB130C	OTTDARP00000020211	DDB0204666	CG10418-PA	19171428	61.m00208	-	GL50803_18713	OTTHUMP00000014843	-	222366	32429	44241	172574	NCU07382	Os08g05850.1	14803	GSPATP00001577001	10494	198696	-	PFE1020w	814695	-	YBL026W	SPCC1620.01c	GLEAN3_25813	SINFRUP00000182151	-	32314	TA07745	113.m00761	53171	84306.m00046	-	UM03799.1
		-	-	-	-	-	-	-	-	-	-	-	-	188340	-	-	-	-	-	-	-	-	-	-	-	-	-	-	-	-	-	-	-	-	-	-	-	-	-	-	-	-	-	-	-	-	-	-	-	-	-	-
		-	+	-	-	-	-	-	AT1G29700.1	-	-	-	-	188339	-	-	CMJ095C	-	-	-	-	-	-	-	-	-	-	-	-	-	-	Os12g44370.1	-	-	-	88056	-	-	820612	-	-	-	-	-	-	12095	-	-	-	-	-	-
CNK5	NIMA-related protein kinase homolog 5	-	+	-	-	-	-	-	-	-	-	-	-	188335	381783	-	-	OTTDARP00000020889	DDB0219417	-	-	-	-	-	-	-	-	-	-	79897	-	Os02g37830.1	-	GSPATP00028006001	-	-	133213	-	178651	-	-	-	-	-	15.m00300	-	-	38.m01045	-	-	-	-
		-	+	-	-	+	-	GB11967-PA	AT2G27900.2	32360	-	CE37754	184136	188334	270083	-	-	OTTDARP00000015719	DDB0186641	CG4996-PA	-	-	ENSGALP00000037320	-	NP_060137.2	-	140484	32036	77728	100869	-	Os10g39910.1	19111	-	-	153798	157082	-	199564	-	-	-	-	SINFRUP00000182806	-	22308	-	-	54017	83711.m00120	-	-
		-	-	-	-	-	-	-	-	-	-	-	-	188332	-	-	-	-	-	-	-	-	-	-	-	-	-	-	-	-	-	-	-	-	-	-	-	-	-	-	-	-	-	-	-	-	-	-	-	-	-	-
		-	-	-	-	-	-	-	-	-	-	-	-	188331	-	-	-	-	-	-	-	-	-	-	-	-	-	-	-	-	-	-	-	-	-	-	-	-	-	-	-	-	-	-	-	-	-	-	-	-	-	-
		-	-	-	-	-	-	-	-	-	-	-	-	188329	-	-	-	-	-	-	-	-	-	-	-	-	-	-	-	-	-	-	-	-	-	-	-	-	-	-	-	-	-	-	-	-	-	-	-	-	-	-
		-	-	-	-	-	-	-	-	-	-	-	-	188328	-	-	-	-	-	-	-	-	-	-	-	-	-	-	-	-	-	-	37353	-	-	-	-	-	-	-	-	-	-	-	-	-	-	-	-	-	-	-
		-	-	-	-	-	-	-	-	-	-	-	-	188324	-	-	-	-	-	-	-	-	-	-	-	-	-	-	-	-	-	-	-	-	-	-	-	-	-	-	-	-	-	-	-	-	-	-	-	-	-	-

		-	-	-	-	-	-	-	-	-	-	-	-	188323	-	-	-	-	-	-	-	-	-	-	-	-	-	-	-	-	-	-	-	-	-	-	140021	-	-	-	-	-	-	-	-	-	-	-	-	-	-	-
		-	-	-	-	-	-	-	-	-	-	-	-	188322	-	-	-	-	-	-	-	-	-	-	-	-	-	-	-	-	-	-	-	-	-	-	-	-	-	-	-	-	-	-	-	-	-	-	-	-	-	-
		-	-	-	-	-	-	-	-	-	-	-	-	188317	-	-	-	-	-	-	-	-	-	-	-	-	-	-	-	-	-	-	-	-	-	168308	-	-	-	-	-	-	-	-	-	-	-	-	-	-	-	-
		-	-	-	-	-	-	-	-	-	-	-	-	188311	-	-	-	-	-	-	-	-	-	-	-	-	-	-	-	-	-	-	-	-	-	-	-	-	-	-	-	-	-	-	-	-	-	-	-	-	-	-
MAA7		-	+	-	-	-	-	-	AT5G54810.1	25486	BDEG_06124	-	60710	188307	-	cgd5_4560	CMJ191C	-	-	-	-	-	-	-	-	-	-	26458	80373	152185	NCU10283	Os08g04180.1	18488	-	41702	177951	108915	-	773968	RO3G_05101.1	YGL026C	SPAC19A8.15	-	-	-	25605	-	-	-	-	-	UM04584.1
		-	-	-	-	-	-	-	-	-	-	-	-	-	-	-	-	-	-	-	-	-	-	-	-	-	-	-	-	-	-	-	-	-	-	-	-	-	-	-	-	-	-	-	-	-	-	-	-	-	-	-
		-	-	-	-	-	-	-	AT4G25070.1	-	-	-	-	188303	-	-	-	-	-	-	-	-	-	-	-	-	-	-	-	-	-	Os01g70320.1	-	-	-	141085	-	-	708819	-	-	-	-	-	-	-	-	-	-	-	-	-
		-	-	-	-	-	-	-	AT4G31080.1	-	-	-	-	188302	-	-	-	-	-	-	-	-	-	-	-	-	-	-	-	-	-	Os02g41990.1	-	-	-	140902	-	-	-	-	-	-	-	-	-	-	-	-	-	-	-	-
		-	-	-	-	-	-	-	-	-	-	-	-	188301	-	-	-	-	-	-	-	-	-	-	-	-	-	-	-	-	-	-	-	-	-	-	-	-	-	-	-	-	-	-	-	-	-	-	-	-	-	-
HDA3405	Histone deacetylase	-	-	-	-	-	-	-	-	-	-	-	-	188299	-	-	-	-	-	-	-	-	-	-	-	-	-	-	-	-	-	-	-	-	-	-	-	-	-	-	-	-	-	-	-	-	-	-	-	-	-	-
		-	-	-	-	-	-	-	-	-	-	-	-	188296	-	cgd7_760	-	-	-	-	-	-	-	-	-	-	-	-	-	-	-	-	-	-	-	-	-	-	-	-	-	-	-	-	-	-	-	-	-	-	-	-
		-	+	-	-	-	-	-	-	-	-	-	-	188293	270856	-	-	-	-	-	-	-	-	-	-	-	229612	30870	-	168814	-	-	-	-	-	-	-	-	-	-	-	-	-	-	-	-	-	-	61593	-	-	-
		-	-	-	-	-	-	-	-	-	-	-	-	188292	-	-	-	-	-	-	-	-	-	-	-	-	-	-	-	-	-	-	-	-	-	-	-	-	-	-	-	-	-	-	-	-	-	-	-	-	-	-
		-	-	-	-	-	-	-	AT5G12960.1	-	-	-	-	188291	-	-	-	-	-	-	-	-	-	-	-	-	-	-	-	-	-	Os02g10190.1	-	-	-	126648	-	-	814847	-	-	-	-	-	-	-	-	-	-	-	-	-
		-	+	-	-	-	-	GB12839-PA	AT3G54710.1	-	BDEG_01348	-	-	188290	-	-	-	-	-	-	-	-	ENSGALP00000022344	-	-	-	-	-	-	-	-	Os04g10650.1	13749	-	42660	-	-	-	831622	-	-	-	-	SINFRUP00000130732	-	-	-	-	-	-	-	-
		-	-	-	-	-	-	-	-	-	-	-	-	188289	-	-	-	-	-	-	-	-	-	-	-	-	-	-	-	-	-	-	-	-	-	-	-	-	-	-	-	-	-	-	-	-	-	-	-	-	-	-
TEF7	Unknown function, chloroplast location proposed	-	-	-	-	-	-	-	-	-	-	-	-	188287	-	-	-	-	-	-	-	-	-	-	-	-	-	-	-	-	-	-	-	-	-	-	-	-	-	-	-	-	-	-	-	-	-	-	-	-	-	-
		-	-	-	-	-	-	-	-	-	-	-	-	188284	-	-	-	-	-	-	-	-	-	-	-	-	-	-	-	-	-	-	-	-	-	-	-	-	-	-	-	-	-	-	-	-	-	-	-	-	-	-
		-	-	-	-	-	-	-	-	-	-	-	-	188281	-	-	-	-	-	-	-	-	-	-	-	-	-	-	-	-	-	-	-	-	-	-	-	-	-	-	-	-	-	-	-	-	-	-	-	-	-	-
		-	-	-	-	-	-	-	-	-	-	-	-	188280	-	-	-	-	-	-	-	-	-	-	-	-	-	-	-	-	-	-	-	-	-	-	-	-	-	-	-	-	-	-	-	-	-	-	-	-	-	-
		-	-	-	-	-	-	-	-	-	-	-	-	188275	-	-	-	-	-	-	-	-	-	-	-	-	-	-	-	-	-	-	-	-	-	-	-	-	-	-	-	-	-	-	-	-	-	-	-	-	-	-
		-	-	-	-	-	-	-	-	-	-	-	-	188274	-	-	-	-	-	-	-	-	-	-	-	-	-	-	-	-	-	-	-	-	-	-	-	-	-	-	-	-	-	-	-	-	-	-	-	-	-	-
		-	-	-	-	-	-	-	-	-	-	-	-	188270	-	-	-	-	-	-	-	-	-	-	-	-	-	-	-	-	-	-	-	-	-	-	-	-	-	-	-	-	-	-	-	-	-	-	-	-	-	-
		-	+	-	-	-	-	GB13236-PA	-	-	-	-	176851	188264	291806	-	-	-	-	-	-	-	-	-	OTTHUMP00000063220	-	133798	39196	-	-	-	-	-	-	-	-	-	-	-	-	-	-	-	-	-	-	-	-	-	-	-	-
		-	-	-	-	-	-	-	-	-	-	-	-	188263	-	-	-	-	-	-	-	-	-	-	-	-	-	-	-	-	-	-	-	-	-	-	-	-	-	-	-	-	-	-	-	-	-	-	-	-	-	-
		-	-	-	-	-	-	-	-	-	-	-	-	188262	-	-	-	-	-	-	-	-	-	-	-	-	-	-	-	-	-	-	-	-	-	-	-	-	-	-	-	-	-	-	-	-	-	-	-	-	-	-
		-	-	-	-	-	-	-	-	-	-	-	-	188260	-	-	-	-	-	-	-	-	-	-	-	-	-	-	-	-	-	-	-	-	-	-	-	-	-	-	-	-	-	-	-	-	-	-	-	-	-	-
		-	-	-	-	-	-	-	-	-	-	-	-	188259	-	-	-	-	-	-	-	-	-	-	-	-	-	-	-	-	-	-	-	-	-	-	-	-	-	-	-	-	-	-	-	-	-	-	-	-	-	-
		-	+	-	-	-	-	-	-	29797	-	CE33319	-	188251	-	-	-	-	-	-	-	-	-	-	-	-	-	-	-	-	-	-	8981	-	-	-	141681	-	-	RO3G_03872.1	YJL097W	-	-	-	-	-	-	-	-	-	-	-
GTE3404	Ring3 protein	-	-	-	-	-	-	-	-	-	-	CE37638	-	188250	-	-	-	-	-	-	-	-	ENSGALP00000039884	-	OTTHUMP00000014752	-	-	-	-	-	-	-	-	-	-	159394	-	-	-	-	-	-	-	-	-	-	-	-	-	-	-	-
		-	-	-	-	-	-	-	-	-	-	-	-	188248	-	-	-	-	-	-	-	-	-	-	-	-	-	-	-	-	-	-	-	-	-	-	-	-	-	-	-	-	-	-	-	-	-	-	-	-	-	-
FAP69	Conserved Uncharacterized Flagellar Associated Protein	+	+	-	+	-	-	-	-	-	BDEG_04464	-	176397	188246	289269	-	-	-	-	-	-	-	ENSGALP00000014669	-	OTTHUMP00000067737	-	223522	9503	62358	240826	-	-	-	GSPATP00018576001	-	-	142476	-	-	-	-	-	-	-	106.m00143	-	-	-	-	-	-	-
		-	-	-	-	-	-	-	-	-	-	-	-	188241	-	-	CMC090C	-	-	-	-	-	-	-	-	-	-	-	-	-	NCU06296	-	-	-	-	-	-	-	-	-	-	-	-	-	-	-	-	-	-	-	-	UM03429.1
		-	-	-	-	-	-	-	-	-	-	-	-	188239	-	-	-	-	-	-	-	-	-	-	-	-	-	-	-	-	-	-	-	-	-	-	-	-	-	-	-	-	-	-	-	-	-	-	-	-	-	-
		-	-	-	-	-	-	-	AT1G63900.1	-	-	-	-	188238	-	-	-	-	-	-	-	-	-	-	-	-	-	-	-	-	-	Os07g45350.3	-	-	-	175369	-	-	246109	-	-	-	-	-	-	-	-	-	-	-	-	-
		-	-	-	-	-	-	-	-	-	-	-	-	188236	-	-	-	-	DDB0217068	-	-	-	-	-	-	-	-	-	48033	-	-	-	-	-	-	-	-	-	-	-	-	-	-	-	-	-	-	-	-	-	-	-
		-	-	-	-	-	-	-	-	-	-	-	-	188231	-	-	-	-	-	-	-	-	-	-	-	-	-	-	-	-	-	-	-	-	-	-	-	-	-	-	-	-	-	-	-	-	-	-	-	-	-	-
		-	-	-	-	-	-	-	-	-	-	-	-	188226	-	-	-	-	-	-	-	-	-	-	-	-	-	-	-	-	-	-	-	-	-	-	-	-	-	-	-	-	-	-	-	-	-	-	-	-	-	-
		-	+	+	-	-	-	GB19708-PA	-	-	BDEG_08154	CE28261	218029	188223	270008	cgd6_960	CMH090C	-	DDB0187313	CG8431-PA	19173375	223.m00079	ENSGALP00000010361	GL50803_5867	OTTHUMP00000012668	LmjF12.0250	237933	223	82919	159064	NCU05642	-	23545	GSPATP00036898001	26346	160662	134891	PF10_0149	-	RO3G_05648.1	YNL247W	SPAC29E6.06c	-	SINFRUP00000153504	74.m00138	1499	TA03175	145.m00322	51757	89419.m00160	Tb927.6.950	UM02613.1
CR008	CR008 protein	-	-	-	-	-	-	-	-	-	-	-	-	188222	-	-	-	-	-	-	-	-	-	-	-	-	-	-	-	-	-	-	32518	-	-	168353	-	-	-	-	-	-	-	-	-	-	-	-	-	-	-	-
		-	-	-	-	-	-	-	-	-	-	-	-	188218	-	-	-	-	-	-	-	-	-	-	-	-	-	-	-	-	-	-	-	-	-	165093	-	-	-	-	-	-	-	-	-	4922	-	-	-	81864.m00399	-	-
		-	-	-	-	-	-	-	-	-	-	-	-	188210	-	-	-	-	-	-	-	-	-	-	-	-	-	-	-	-	-	-	-	-	-	-	-	-	-	-	-	-	-	-	-	-	-	-	-	-	-	-
SEC61a	SEC61-alpha subunit of ER-translocon	-	+	+	-	-	-	GB11642-PA	AT2G34250.2	70223	BDEG_00233	CE14954	21026	188207	289360	cgd7_950	CMK192C	OTTDARP00000020338	DDB0206262	CG9539-PA	19171259	467.m00033	ENSGALP00000022602	GL50803_5744	OTTHUMP00000019101	LmjF11.1050	194715	38396	82968	190623	NCU08897	Os09g17840.1	21584	GSPATP00036723001	41031	215299	109010	MAL13P1.231	828572	RO3G_11125.1	YLR378C	SPBC354.02c	-	SINFRUP00000145654	67.m00257	25355	TA17425	59.m00005	63808	97114.m00175	Tb11.02.4100	UM05188.1
FAP123	Flagellar Associated Protein	-	-	-	+	-	-	-	-	68120	-	-	-	188205	-	-	-	-	-	-	-	-	-	-	-	-	-	-	-	-	-	-	-	-	-	-	-	-	-	-	-	-	-	-	-	-	-	-	-	-	-	-
		-	-	-	-	-	-	-	-	-	-	-	-	188200	-	-	-	-	-	-	-	-	-	-	-	-	-	-	-	-	-	-	-	-	-	-	-	-	-	-	-	-	-	-	-	-	-	-	-	-	-	-
PTOX2	alternative oxidase, possibly chloroplast-localized	-	-	-	-	-	-	-	-	-	-	-	-	188199	-	-	-	-	-	-	-	-	-	-	-	-	-	-	-	-	-	-	-	-	-	-	-	-	-	-	-	-	-	-	-	-	-	-	-	-	-	-
PRMT3401	Protein arginine N-methyltransferase	-	+	+	-	-	-	GB10121-PA	AT4G29510.1	22815	BDEG_06673	CE23297	173477	188198	248658	cgd8_4760	CMD031C	OTTDARP00000020425	DDB0183976	CG6554-PA	-	54.m00193	ENSGALP00000021646	-	NP_001527.3	LmjF12.1270	177209	37585	80851	179399	NCU07459	Os09g19560.1	-	GSPATP00025365001	17184	56390	108352	PF14_0242	418476	RO3G_15619.1	YBR034C	SPAC890.07c	GLEAN3_01244	SINFRUP00000147234	18.m00403	3690	TA15115	38.m00018	49708	95299.m00081	Tb927.1.4690	UM05900.1
BLD2		+	+	-	-	-	-	GB15835-PA	-	2189	BDEG_05632	-	218781	188195	299098	-	-	OTTDARP00000003871	-	-	-	-	ENSGALP00000024172	GL50803_6336	OTTHUMP00000017033	LmjF21.1010	224456	29416	44774	89613	-	-	-	GSPATP00038073001	-	99422	118214	PF14_0725	-	-	-	-	GLEAN3_02663	SINFRUP00000142299	8.m00446	-	-	-	50572	82471.m00124	Tb10.70.6950	-
		-	-	-	-	-	-	-	-	-	-	-	-	188191	-	-	-	-	-	-	-	-	-	-	-	-	134727	-	-	-	-	-	-	-	-	-	-	-	-	-	-	-	-	-	-	-	-	-	-	-	-	-
		-	-	-	-	-	-	-	-	65121	-	-	-	188189	-	-	CMS086C	-	-	-	-	-	-	-	-	-	-	-	-	-	-	-	-	-	-	-	-	-	-	RO3G_06789.1	-	-	-	-	-	-	-	-	-	-	-	-
		-	-	-	-	-	-	-	-	-	-	-	-	188181	-	-	-	-	-	CG5700-PB	-	-	-	-	-	-	-	-	-	-	-	-	-	-	-	-	-	-	-	-	-	-	-	-	-	-	-	-	-	-	-	-
FAP111	Conserved Uncharacterized Flagellar Associated Protein	+	+	-	+	-	-	GB17830-PA	-	7168	-	-	164006	188180	204976	-	-	-	-	-	-	-	-	GL50803_7296	NP_660352.1	-	87093	35695	4690	136528	-	-	-	GSPATP00015380001	-	-	133032	PF14_0651	-	-	-	-	-	-	30.m00292	-	-	50.m05675	26078	-	-	-
		-	-	-	-	-	-	-	-	-	-	-	-	188179	-	-	-	-	-	-	-	-	-	-	-	-	-	-	-	-	-	-	-	-	-	-	-	-	-	-	-	-	-	-	-	-	-	-	-	-	-	-
		-	+	-	-	-	-	-	AT3G23080.1	-	-	CE36098	157995	188173	273854	-	-	-	-	CG6565-PA	-	-	-	-	NP_064536.2	-	-	-	-	35024	-	Os02g03230.1	37404	-	-	155288	-	-	230902	-	-	-	GLEAN3_20679	SINFRUP00000170376	-	-	-	-	58714	-	-	-
		-	+	-	-	-	-	GB11103-PA	-	7566	-	-	155053	188169	-	-	CMS231C	-	-	CG2789-PA	-	-	ENSGALP00000022870	-	OTTHUMP00000028891	-	210696	16397	-	167042	-	-	32816	-	49987	190625	-	-	-	-	-	-	GLEAN3_13092	SINFRUP00000150790	-	2880	-	-	37351	-	-	-
		-	-	-	-	-	-	-	-	-	-	-	-	188167	-	-	-	-	-	-	-	-	-	-	-	-	-	-	-	-	-	-	-	-	-	-	-	-	-	-	-	-	-	-	-	-	-	-	-	-	-	-
		-	+	-	-	-	-	-	AT1G71730.1	-	-	-	221466	188159	-	-	-	-	-	-	-	-	-	-	-	LmjF36.1510	230306	-	-	-	-	Os05g08920.1	-	-	-	49326	-	-	749099	RO3G_06523.1	-	-	-	-	-	-	-	-	-	-	-	-
		-	-	-	-	-	-	-	-	-	-	-	-	188157	-	-	-	-	-	-	-	-	-	-	-	-	-	-	-	-	-	-	-	-	-	-	-	-	-	-	-	-	-	-	-	-	-	-	-	-	-	-
		-	+	-	-	-	-	GB11107-PA	AT2G02560.2	-	BDEG_07930	CE20378	19001	188156	219170	-	-	OTTDARP00000007720	DDB0167539	CG5366-PA	-	60.m00147	ENSGALP00000013701	-	OTTHUMP00000168390	-	130688	34257	58949	128552	NCU08875	Os02g07120.1	31683	-	46608	97881	127449	-	827477	RO3G_11610.1	-	-	GLEAN3_25585	SINFRUP00000137036	-	262874	-	-	51005	-	-	UM03477.1
		-	-	-	-	-	-	-	-	-	-	-	-	188151	-	-	-	-	-	-	-	-	-	-	-	-	-	-	-	-	-	-	-	-	-	-	-	-	-	-	-	-	-	-	-	-	-	-	-	-	-	-
		-	+	-	-	-	-	GB14436-PA	AT5G50720.1	25684	BDEG_07291	CE24624	156801	188150	298618	cgd1_1830	CMT630C	OTTDARP00000021123	-	CG30193-PF	19170864	-	ENSGALP00000037103	-	OTTHUMP00000195052	-	149167	16940	33108	107629	NCU05521	Os01g52780.1	34540	GSPATP00018446001	43671	227472	156427	PFC0730w	177877	RO3G_03850.1	YPR028W	SPCC830.08c	-	SINFRUP00000164924	205.m00042	262530	TA13960	55.m04670	37376	93686.m00049	-	UM06393.1
CAM1	calmodulin	-	+	+	+	-	-	GB15633-PA	AT5G21274.1	36419	BDEG_04693	CE13902	157141	188144	225820	cgd2_810	CME034C	OTTDARP00000004546	DDB0205842	CG8472-PB	19168781	151.m00100	ENSGALP00000016260	GL50803_5333	OTTHUMP00000159070	LmjF09.0910	216228	33928	55564	239788	NCU04120	Os07g48780.1	24922	GSPATP00015825001	23794	185037	109328	PF14_0323	580637	RO3G_11905.1	YBR109C	SPAC3A12.14	GLEAN3_05032	SINFRUP00000130883	89.m00187	41291	TA14735	50.m03285	37105	81616.m00154	Tb11.01.4624	UM03910.1
NUOS6	NADH:ubiquinone oxidoreductase (Complex I) 16.3 kDA subunit, homolog to bovine 13A subunit mitochondrial	-	+	-	-	-	-	-	AT3G03070.1	-	-	-	-	188142	-	-	CMN320C	-	DDB0189366	-	-	-	-	-	-	-	-	-	-	-	-	Os08g06430.1	31701	GSPATP00006358001	-	116971	-	-	596933	-	-	-	-	-	-	-	-	-	17646	-	-	-
		-	+	-	-	-	-	GB13027-PA	AT5G37370.1	37711	BDEG_07287	CE26040	220262	188141	274646	-	-	OTTDARP00000020501	DDB0187924	CG1622-PA	-	54.m00225	ENSGALP00000003060	-	NP_060531.1	-	95079	33792	66683	199548	-	Os05g08600.2	10602	GSPATP00034895001	45533	177795	138456	PF14_0070	836549	RO3G_16225.1	-	-	-	SINFRUP00000130956	146.m00074	8500	TA09950	76.m01593	-	-	-	-
		-	-	-	-	-	-	-	-	-	-	-	-	188136	-	-	-	-	-	-	-	-	-	-	-	-	-	-	-	-	-	-	-	-	-	-	-	-	-	-	-	-	-	-	-	-	-	-	-	-	-	-
		-	-	-	-	-	-	-	-	-	-	-	-	188134	-	-	-	-	-	-	-	-	-	-	-	-	-	-	-	-	-	-	-	-	-	-	-	-	-	-	-	-	-	-	-	-	-	-	-	-	-	-
		-	-	-	-	-	-	-	-	-	-	-	-	188133	-	-	-	-	-	-	-	-	-	-	-	-	-	-	-	-	-	-	-	-	-	-	-	-	-	-	-	-	-	-	-	-	-	-	-	-	-	-
		-	-	-	-	-	-	-	-	-	-	-	-	188132	-	-	-	-	-	-	-	-	-	-	-	-	-	-	-	-	-	-	-	-	-	-	-	-	-	-	-	-	-	-	-	-	-	-	-	-	-	-
CHR3401	Putative chromodomain-helicase-DNA-binding protein	-	+	+	-	-	-	GB14981-PA	AT2G13370.1	-	BDEG_03472	CE32454	167753	188131	269794	cgd1_3670	CMQ215C	OTTDARP00000013790	DDB0185877	CG3733-PA	19068436	48.m00200	ENSGALP00000011246	GL50803_112978	OTTHUMP00000194776	-	218699	689	29521	50041	NCU03060	Os07g46590.1	-	GSPATP00005981001	-	132084	142676	PF10_0232	172227	RO3G_02376.1	YER164W	SPAC3G6.01	GLEAN3_23296	SINFRUP00000160883	16.m00319	-	TA06105	55.m04750	54568	82761.m00451	-	UM05086.1
LCI24	low-CO2-inducible membrane protein	-	-	-	-	-	-	-	-	-	-	-	-	188129	-	-	-	-	-	-	-	-	-	-	-	-	-	-	-	-	-	-	-	-	-	-	-	-	-	-	-	-	-	-	-	-	-	-	-	-	-	-
		-	-	-	-	-	-	-	-	-	-	-	-	188127	-	-	-	-	-	-	-	-	-	-	-	-	-	-	-	-	-	-	-	-	-	-	-	-	-	-	-	-	-	-	-	-	-	-	-	-	-	-
		-	-	-	-	-	-	-	-	-	-	-	-	188126	-	-	-	OTTDARP00000017653	-	-	-	-	-	-	-	-	-	-	-	-	-	-	-	-	-	-	-	-	-	-	-	-	-	-	-	-	-	-	-	-	-	-
		-	-	-	-	-	-	-	-	-	-	-	-	188125	-	-	-	-	-	-	-	-	-	-	-	-	-	-	-	-	-	-	-	-	-	-	-	-	-	-	-	-	-	-	-	-	-	-	-	-	-	-
		-	-	-	-	-	-	-	-	-	-	-	-	188124	-	-	-	-	-	-	-	-	-	-	-	-	-	-	-	-	-	-	-	-	-	-	-	-	-	-	-	-	-	-	-	-	-	-	-	-	-	-
		-	-	-	-	-	-	-	-	-	-	-	-	188122	296714	-	-	-	-	-	-	-	-	-	-	-	-	-	-	-	-	-	-	-	-	-	-	-	-	-	-	-	-	-	-	-	-	-	-	-	-	-
	Myo-inositol-1-phosphate synthase	-	+	+	-	-	-	GB11572-PA	AT2G22240.1	28436	BDEG_03425	CE31076	156627	188120	-	-	CMR036C	-	DDB0186536	CG11143-PA	-	57.m00178	-	GL50803_17579	OTTHUMP00000067818	LmjF14.1360	225722	31130	36195	229609	NCU06666	Os03g09250.1	15645	GSPATP00029489001	45120	223663	108192	PFE0585c	831371	RO3G_04151.1	YJL153C	-	GLEAN3_27561	SINFRUP00000181622	64.m00256	36788	-	-	-	94275.m00081	Tb10.6k15.3600	UM05549.1
SIR3		-	-	-	-	-	-	-	-	-	-	-	-	-	-	-	-	-	-	-	-	-	-	-	-	-	-	-	-	-	-	-	-	-	-	-	-	-	-	-	-	-	-	-	-	-	-	-	-	-	-	-
FBB6		-	+	-	-	-	-	-	-	-	-	-	-	188114	295118	-	-	-	-	-	-	-	ENSGALP00000027638	-	NP_055437.2	-	119348	-	-	92763	-	-	-	-	-	-	-	-	-	-	-	-	GLEAN3_09848	-	-	-	-	-	32651	-	-	-
		-	+	-	-	-	-	-	-	-	-	-	110378	188109	291598	-	-	-	-	CG4751-PA	-	-	-	-	OTTHUMP00000066635	-	140609	-	-	22327	-	-	33576	-	-	-	-	-	-	RO3G_16177.1	-	-	-	SINFRUP00000144759	-	-	-	-	54406	-	-	-
		-	-	-	-	-	-	-	-	-	-	-	-	188108	-	-	-	-	-	-	-	-	-	-	-	-	-	-	-	-	-	-	-	-	-	-	-	-	-	-	-	-	-	-	-	-	-	-	-	-	-	-
		-	-	-	-	-	-	-	-	-	-	-	-	188106	-	-	-	-	-	-	-	-	-	-	-	-	-	-	-	-	-	-	-	-	-	-	-	-	-	-	-	-	-	-	-	-	-	-	-	-	-	-
		-	-	-	-	-	-	-	-	-	-	-	-	188105	-	-	-	-	-	-	-	-	-	-	-	-	-	-	-	-	-	-	-	-	-	-	-	-	-	-	-	-	-	-	-	-	-	-	-	-	-	-
		-	-	-	-	-	-	-	-	-	-	-	-	188101	-	-	-	-	-	-	-	-	-	-	-	-	-	-	-	-	-	-	-	-	-	-	-	-	-	-	-	-	-	-	-	-	-	-	-	-	-	-
		-	-	-	-	-	-	-	-	-	-	-	-	188099	-	-	-	-	-	-	-	-	-	-	-	-	-	-	-	-	-	-	-	-	-	-	-	-	-	-	-	-	-	-	-	-	-	-	-	-	-	-
		-	-	-	-	-	-	-	-	-	-	-	-	188095	-	-	-	-	-	-	-	-	-	-	-	-	-	-	-	-	-	-	-	-	-	-	-	-	-	-	-	-	-	-	-	-	-	-	-	-	-	-
		-	-	-	-	-	-	-	-	-	-	-	-	188091	-	-	-	-	-	-	-	-	-	-	-	-	-	-	-	-	-	-	-	-	-	-	-	-	-	-	-	-	-	-	-	-	-	-	-	-	-	-
SCL27	putative SR protein factor involved in mRNA splicing	-	+	-	-	-	-	-	AT5G18810.1	17068	BDEG_00508	-	124300	188086	257770	cgd7_940	CMO246C	-	-	-	-	-	ENSGALP00000006566	-	XP_941333.1	-	109032	3751	-	81449	-	Os02g15310.2	7793	-	12366	141868	136528	PFE0160c	420699	RO3G_16588.1	-	-	GLEAN3_06900	SINFRUP00000150947	-	23196	-	-	-	85889.m00409	-	-
	cytochrome b-561 like protein	-	+	-	-	-	-	GB11498-PA	AT1G14730.1	-	-	-	-	188082	-	-	-	OTTDARP00000022299	-	CG1275-PA	-	-	ENSGALP00000015541	-	OTTHUMP00000171271	-	236962	-	-	-	-	-	-	-	-	221256	129715	-	-	-	-	-	GLEAN3_12183	-	-	-	-	-	-	-	-	-
		-	+	-	-	-	-	GB30019-PB	AT1G56500.1	-	-	-	52631	188073	-	-	CMM305C	-	-	CG12547-PA	-	-	ENSGALP00000032451	-	OTTHUMP00000020518	-	119776	-	-	233976	-	Os03g19760.2	-	-	-	128572	-	-	240352	-	-	-	-	SINFRUP00000128220	-	-	-	-	11855	-	-	-
		-	-	-	-	-	-	-	-	-	-	-	-	188070	-	-	-	-	-	-	-	-	-	-	-	-	-	-	-	-	-	-	-	-	-	-	-	-	-	-	-	-	-	-	-	-	-	-	-	-	-	-
IF6A	eukaryotic translation initiation factor 6	-	+	+	-	-	-	GB11906-PA	AT3G55620.1	5779	BDEG_06645	CE17565	156578	188065	284015	cgd4_3130	CMT351C	OTTDARP00000002095	DDB0203843	CG17611-PA	19068797	83.m00186	ENSGALP00000002796	GL50803_14606	OTTHUMP00000030734	LmjF36.0890	206392	20305	81588	177965	NCU09004	Os07g44620.2	22707	GSPATP00023809001	24493	173032	108957	PF13_0178	719158	RO3G_13353.1	YPR016C	SPCC1919.09	-	SINFRUP00000146339	119.m00105	29782	TA12585	641.m01501	18785	87873.m00078	Tb10.70.1770	UM06443.1
GST	GST, glutathione S-transferase, mitochondrial	-	+	-	-	-	-	GB18045-PB	AT2G30860.1	67824	BDEG_08491	-	146821	188060	284915	-	CMR110C	OTTDARP00000021672	DDB0215230	CG17522-PA	-	-	-	-	-	-	189800	32442	44250	237669	NCU04109	Os01g27360.1	-	-	15764	183533	145350	-	678517	-	YNL229C	SPCC965.07c	-	-	43.m00274	8861	-	145.m00003	-	-	-	UM06325.1
		-	-	-	-	-	-	-	-	-	-	-	-	188059	-	-	-	-	-	-	-	-	-	-	-	-	-	-	-	-	-	-	-	-	-	-	-	-	-	-	-	-	-	-	-	-	-	-	-	-	-	-
		-	+	-	-	-	-	-	AT1G04900.1	-	BDEG_02645	-	-	188058	-	-	CMD137C	-	DDB0206168	-	-	-	-	-	-	LmjF22.1340	-	-	30894	-	NCU02565	Os01g68390.1	-	GSPATP00031154001	33037	143192	143931	-	707212	RO3G_00587.1	-	-	-	-	51.m00241	-	-	-	-	-	Tb927.7.3340	-
FAP189	Conserved Uncharacterized Coiled-Coil Flagellar Associated Protein	-	+	-	+	-	-	GB14268-PA	-	30784	BDEG_02624	-	-	188056	215861	-	-	-	-	-	-	82.m00159	ENSGALP00000013696	GL50803_3409	OTTHUMP00000020442	LmjF36.5020	-	823	-	-	-	-	535	GSPATP00001265001	-	-	129837	PFF1285w	-	-	YDL058W	-	-	SINFRUP00000145526	2.m02357	-	-	55.m05112	-	96546.m00064	Tb11.01.2670	-
		-	-	-	-	-	-	-	-	-	-	-	-	188054	-	-	-	-	-	-	-	-	-	-	-	-	-	-	-	-	-	-	-	-	-	-	-	-	-	-	-	-	-	-	-	-	-	-	-	-	-	-
		-	-	-	-	-	-	-	-	-	-	-	-	188052	-	-	-	-	-	-	-	-	-	-	-	-	-	-	-	-	-	-	-	-	-	-	-	-	-	-	-	-	-	-	-	-	-	-	-	-	-	-
		-	-	-	-	-	-	-	-	-	-	-	203517	188051	-	-	-	-	-	-	-	-	-	-	-	-	-	-	-	-	-	-	-	-	-	-	-	-	-	-	-	-	-	-	-	-	-	-	-	-	-	-
		-	+	-	-	-	-	GB11672-PA	AT3G05090.2	-	-	CE31502	223811	188044	369532	-	CMJ077C	OTTDARP00000020507	DDB0217972	-	-	-	ENSGALP00000030646	-	OTTHUMP00000170690	-	195432	29733	49758	185249	NCU01569	Os12g06810.2	13747	-	-	212890	-	-	827334	RO3G_12460.1	-	-	-	SINFRUP00000137883	-	-	-	-	22317	-	-	-
		-	-	-	-	-	-	-	-	-	-	-	-	188043	-	-	-	-	-	-	-	-	-	-	-	-	-	-	-	-	-	-	-	-	-	-	-	-	-	-	-	-	-	-	-	-	-	-	-	-	-	-
		-	-	-	-	-	-	-	-	-	-	-	-	188042	-	-	-	-	-	-	-	-	-	-	-	-	-	-	-	-	-	-	-	-	-	-	-	-	-	-	-	-	-	-	-	-	-	-	-	-	-	-
		-	-	-	-	-	-	-	-	-	-	-	-	188040	-	-	-	-	-	-	-	-	-	-	-	-	-	-	-	-	-	-	-	-	-	-	-	-	-	-	-	-	-	-	-	-	-	-	-	-	-	-
		-	-	-	-	-	-	-	-	-	-	-	-	188035	-	-	-	-	-	-	-	-	-	-	-	-	-	-	-	-	-	-	-	-	-	-	-	-	-	-	-	-	-	-	-	-	-	-	-	-	-	-
		-	-	-	-	-	-	-	-	-	-	-	-	188034	-	-	-	-	-	-	-	-	-	-	-	-	-	-	-	-	-	-	-	-	-	-	-	-	-	-	-	-	-	-	-	-	-	-	-	-	-	-
		-	-	-	-	-	-	-	-	-	-	-	-	188033	-	-	-	-	-	-	-	-	-	-	-	-	-	-	-	-	-	-	-	-	-	-	-	-	-	-	-	-	-	-	-	-	-	-	-	-	-	-
		-	-	-	-	-	-	-	-	-	-	-	-	188027	-	-	-	-	-	-	-	-	-	-	-	-	-	-	-	-	-	-	-	-	-	-	-	-	-	-	-	-	-	-	-	-	-	-	-	-	-	-
		-	+	-	-	-	-	-	-	14350	-	-	-	188026	207790	-	-	-	-	-	-	-	-	GL50803_4577	-	-	75717	24169	-	72121	-	-	-	GSPATP00006069001	-	-	-	-	-	-	-	-	-	-	43.m00292	-	-	-	-	-	-	-
		-	+	-	-	-	-	-	AT2G39000.1	-	-	-	-	188025	-	-	CMF120C	-	DDB0187407	-	-	-	-	-	-	-	-	-	-	-	-	-	15489	-	46134	-	-	-	-	-	-	-	-	-	-	-	-	-	-	-	-	-
		-	-	-	-	-	-	-	-	-	-	-	-	188024	-	-	-	-	-	-	-	-	-	-	-	-	-	-	-	-	-	-	-	-	-	-	-	-	-	-	-	-	-	-	-	-	-	-	-	-	-	-
		-	-	-	-	-	-	-	-	-	-	-	-	188017	-	-	-	-	-	-	-	-	-	-	-	-	-	-	-	-	-	-	-	-	-	-	-	-	-	-	-	-	-	-	-	-	-	-	-	-	-	-
		-	-	-	-	-	-	-	-	-	-	-	-	188013	-	-	-	-	-	-	-	-	-	-	-	-	-	-	-	-	-	-	-	-	-	-	-	-	-	-	-	-	-	-	-	-	-	-	-	-	-	-
		-	-	-	-	-	-	-	-	-	-	-	-	188012	-	-	-	-	-	-	-	-	-	-	-	-	-	-	-	-	-	-	-	-	-	-	-	-	-	-	-	-	-	-	-	-	-	-	-	-	-	-
		-	-	-	-	-	-	-	-	-	-	-	-	188011	-	-	-	-	-	-	-	-	-	-	-	-	-	-	-	-	-	-	-	-	-	-	-	-	-	-	-	-	-	-	-	-	-	-	-	-	-	-
		-	-	-	-	-	-	-	-	-	-	-	-	188008	-	-	-	-	-	-	-	-	-	-	-	-	-	-	-	-	-	-	-	-	-	-	-	-	-	-	-	-	-	-	-	-	-	-	-	-	-	-
		-	+	-	-	-	-	-	-	62461	-	-	-	188007	-	-	-	-	-	-	-	-	-	-	-	-	-	-	-	-	-	-	31644	GSPATP00013922001	34205	-	130450	-	-	-	-	-	-	-	12.m00519	-	-	-	-	-	-	-
FKB20-2	peptidyl-prolyl cis-trans isomerase, FKBP-type	-	+	-	-	-	-	-	AT3G60370.1	-	-	-	-	188000	-	-	-	-	-	-	-	-	-	-	-	-	-	-	-	-	-	Os07g30800.1	25583	-	-	124948	-	-	781385	-	-	-	-	-	-	-	-	-	-	-	-	-
VTI1	Qb-SNARE, VTI1-family	-	+	+	-	-	-	GB15918-PA	AT5G39510.1	67575	BDEG_03743	CE28871	152399	187998	392951	cgd3_180	CMJ056C	OTTDARP00000021549	DDB0219803	CG3279-PA	-	200.m00075	ENSGALP00000014415	-	OTTHUMP00000020493	LmjF23.1750	141904	38961	81391	132347	NCU05959	Os01g51120.1	32285	GSPATP00002940001	40521	201435	129917	PFL1740w	825574	RO3G_09316.1	YMR197C	SPBC3B9.10	-	SINFRUP00000148942	150.m00099	264899	TA20420	65.m01154	60180	93986.m00172	Tb927.8.3470	UM05645.1
		-	+	-	-	-	-	GB12526-PA	AT3G10370.1	72359	BDEG_05301	CE14180	176445	187997	246161	-	CML209C	-	DDB0185769	CG8256-PA	19069338	-	ENSGALP00000020459	-	OTTHUMP00000162788	LmjF28.0240	238523	-	80825	246617	NCU05454	Os04g14790.1	27797	GSPATP00027986001	1129	115350	109316	PFC0275w	822583	RO3G_08855.1	YIL155C	SPCC1223.03c	GLEAN3_05863	SINFRUP00000148733	47.m00255	41505	TA17925	55.m05083	64277	-	Tb11.02.5280	UM01619.1
ACP2		-	+	-	-	-	-	-	AT3G05020.1	-	-	-	-	187994	-	-	CMV132C	-	-	-	-	-	-	-	-	-	-	-	-	-	-	Os11g31900.2	-	-	9709	211342	-	PFB0385w	417581	-	-	-	-	-	-	-	-	55.m00019	-	-	-	-
		-	+	-	-	-	-	-	-	-	-	-	-	187991	-	-	-	-	-	-	-	-	ENSGALP00000026557	-	-	-	143089	10631	-	-	-	Os02g36520.1	-	-	-	159974	139440	-	-	-	-	-	-	SINFRUP00000139293	-	24961	-	-	-	-	-	-
		-	-	-	-	-	-	-	-	-	-	-	-	187987	-	-	-	-	-	-	-	-	-	-	-	-	-	-	-	-	-	-	-	-	-	-	-	-	-	-	-	-	-	-	-	-	-	-	-	-	-	-
		-	-	-	-	-	-	-	-	-	-	-	-	187986	-	-	-	-	-	-	-	-	-	-	-	-	-	-	-	-	-	-	-	-	-	-	-	-	-	-	-	-	-	-	-	-	-	-	-	-	-	-
		-	+	+	-	-	-	GB14596-PA	-	11569	BDEG_06168	CE20529	167074	187983	-	-	-	-	DDB0168569	CG6428-PA	-	10.m00347	ENSGALP00000018854	GL50803_14547	XP_935652.1	LmjF15.0390	-	-	1759	83566	NCU03768	-	36264	GSPATP00024733001	20905	-	132865	-	-	RO3G_00950.1	-	-	-	SINFRUP00000139664	58.m00275	7120	-	80.m02169	30040	-	-	UM01007.1
		-	-	-	-	-	-	-	-	-	-	-	-	187982	-	-	-	-	-	-	-	-	-	-	-	-	-	-	-	-	-	-	-	-	-	-	-	-	-	-	-	-	-	-	-	-	-	-	-	-	-	-
		-	+	-	-	-	-	-	AT2G27100.1	-	-	CE33369	-	187979	388681	-	-	OTTDARP00000015948	-	CG7843-PC	-	-	-	-	-	-	180663	-	-	239938	-	Os08g40560.2	37036	-	-	133793	-	-	679868	RO3G_10853.1	-	-	GLEAN3_00171	SINFRUP00000173756	-	-	-	-	-	-	-	-
		-	-	-	-	-	-	-	-	-	-	-	-	187976	-	-	-	-	-	-	-	-	-	-	-	-	-	-	-	-	-	-	-	-	44454	-	-	-	-	-	-	-	-	-	-	-	-	-	-	-	-	-
		-	-	-	-	-	-	-	-	-	-	-	-	187972	-	-	-	-	-	-	-	-	-	-	-	-	-	-	-	-	-	-	-	-	-	-	-	-	-	-	-	-	-	-	-	-	-	-	-	-	-	-
		-	-	-	-	-	-	-	-	-	-	-	-	187971	-	-	-	-	-	-	-	-	-	-	-	-	-	-	-	-	-	-	-	-	-	-	-	-	-	-	-	-	-	-	-	-	-	-	-	-	-	-
		-	-	-	-	-	-	-	AT4G33690.1	-	-	-	-	187970	-	-	-	-	-	-	-	-	-	-	-	-	-	-	-	-	-	Os08g09300.2	-	-	-	211137	-	-	767748	-	-	-	-	-	-	-	-	-	-	-	-	-
		-	-	-	-	-	-	-	AT3G07560.1	-	-	-	-	187965	-	-	-	-	-	-	-	-	-	-	-	-	-	-	-	-	-	Os07g05810.1	-	-	-	166717	-	-	679642	-	-	-	-	-	-	-	-	-	-	-	-	-
		-	-	-	-	-	-	-	-	-	-	-	-	187964	-	-	-	-	-	-	-	-	-	-	-	-	-	-	-	-	-	-	-	-	-	-	-	-	-	-	-	-	-	-	-	-	-	-	-	-	-	-
		-	-	-	-	-	-	-	-	-	-	-	-	187963	-	-	-	-	-	-	-	-	-	-	-	-	-	-	-	-	-	-	-	-	-	-	-	-	-	-	-	-	-	-	-	-	-	-	-	-	-	-
		-	+	-	-	-	-	-	-	-	-	-	157516	187959	-	-	-	-	-	CG10444-PA	-	-	ENSGALP00000033081	-	-	-	177946	-	-	-	-	-	24300	-	-	-	-	-	-	-	-	-	-	-	-	-	-	-	-	-	-	-
		-	-	-	-	-	-	-	-	-	-	-	-	187957	-	-	-	-	-	-	-	-	-	-	-	-	-	-	-	-	-	-	-	-	-	-	-	-	-	-	-	-	-	-	-	-	-	-	-	-	-	-
		-	-	-	-	-	-	-	-	-	-	-	-	187956	-	-	-	-	-	-	-	-	-	-	-	-	-	-	-	-	-	-	-	-	-	-	-	-	-	-	-	-	-	-	-	-	-	-	-	-	-	-
		-	-	-	-	-	-	-	-	-	-	-	-	187951	277461	-	-	-	-	-	-	-	-	-	-	-	-	-	72249	-	-	-	14863	-	-	-	-	-	560914	-	-	-	-	-	-	-	-	-	-	-	-	-
		-	-	-	-	-	-	-	-	-	-	-	-	187941	-	-	-	-	-	-	-	-	-	-	-	-	-	-	-	-	-	-	-	-	-	-	-	-	-	-	-	-	-	-	-	-	-	-	-	-	-	-
HMGB3407	ARID domain containing HMGB protein	-	+	-	-	+	-	GB19258-PA	AT1G76110.1	-	BDEG_02606	CE31068	64285	187927	267606	-	-	OTTDARP00000018864	-	CG5403-PB	-	-	ENSGALP00000002095	-	NP_689854.2	-	131492	-	53708	42971	-	Os09g37250.1	-	GSPATP00004674001	-	51717	-	-	206566	-	-	-	GLEAN3_25601	SINFRUP00000152561	21.m00216	-	-	-	54817	-	-	-
		-	-	-	-	-	-	-	-	-	-	-	-	187924	-	-	-	-	-	-	-	-	-	-	-	-	-	-	-	-	-	-	-	-	-	-	-	-	-	-	-	-	-	-	-	-	-	-	-	-	-	-
		-	-	-	-	-	-	-	-	-	-	-	-	187917	-	-	-	-	-	-	-	-	-	-	-	-	-	-	-	-	-	-	-	-	-	-	-	-	-	-	-	-	-	-	-	-	-	-	-	-	-	-
		-	-	-	-	-	-	-	-	-	-	-	-	187914	-	-	-	-	-	-	-	-	-	-	-	-	-	-	-	-	-	-	-	-	-	-	-	-	-	-	-	-	-	-	-	-	-	-	-	-	-	-
		-	+	-	-	-	-	-	AT1G44920.1	-	-	-	-	187910	-	-	-	-	-	-	-	-	-	-	-	-	-	-	-	-	-	Os03g51930.2	32848	-	45766	6357	-	-	816442	-	-	-	-	-	-	5830	-	-	-	-	-	-
		-	-	-	-	-	-	-	-	-	-	-	-	187907	-	-	-	-	-	-	-	-	-	-	-	-	-	-	-	-	-	-	-	-	-	-	-	-	-	-	-	-	-	-	-	-	-	-	-	-	-	-
		-	-	-	-	-	-	-	-	-	-	-	-	187906	-	-	-	-	-	-	-	-	-	-	-	-	-	-	-	-	-	-	-	-	-	-	-	-	-	-	-	-	-	-	-	-	-	-	-	-	-	-
		-	-	-	-	-	-	-	-	-	-	-	-	187905	-	-	-	-	-	-	-	-	-	-	-	-	-	-	-	-	-	-	-	-	-	-	128494	-	-	-	-	-	-	-	-	-	-	-	-	-	-	-
		-	-	-	-	-	-	-	-	-	-	-	-	187904	-	-	-	-	-	-	-	-	-	-	-	-	-	-	-	-	-	-	-	-	-	-	-	-	-	-	-	-	-	-	-	-	-	-	-	-	-	-
ISU1	iron-sulfur cluster assembly scaffold protein	-	+	+	-	-	-	GB13375-PA	AT4G22220.1	60318	BDEG_00407	CE16643	220819	187899	206163	cgd6_1050	CMR318C	OTTDARP00000016311	DDB0185317	CG9836-PA	19068452	21.m00283	ENSGALP00000007760	GL50803_15196	NP_998760.1	LmjF35.3620	210679	30103	32298	227033	NCU05778	Os05g49300.1	15203	GSPATP00011514001	28374	48714	109414	PF14_0518	834330	RO3G_15308.1	YPL135W	SPAC227.13c	GLEAN3_10311	SINFRUP00000155113	97.m00123	36099	TA19345	46.m00021	53451	95287.m00238	Tb09.211.2830	UM05632.1
STA1	ADP-glucose pyrophosphorylase large subunit	-	-	-	-	-	-	-	-	-	-	-	-	-	-	-	-	-	-	-	-	-	-	-	-	-	-	-	-	-	-	-	-	-	-	-	-	-	-	-	-	-	-	-	-	-	-	-	-	-	-	-
		-	+	-	-	-	-	-	AT1G10030.1	-	BDEG_02757	-	165270	187890	-	-	-	-	DDB0188866	-	-	-	-	-	OTTHUMP00000028165	-	222610	-	31075	-	NCU04461	Os12g43670.1	-	-	-	109549	-	-	830270	RO3G_05441.1	YER044C	SPBC337.09	-	SINFRUP00000152671	-	-	-	-	57153	-	-	UM01789.1
HTA3404	Histone H2A variant	-	+	-	-	-	-	GB12991-PA	AT1G52740.1	59843	-	CE07426	170341	187884	224180	cgd5_940	CMR457C	OTTDARP00000016046	DDB0187128	-	-	-	ENSGALP00000022676	-	OTTHUMP00000161678	LmjF17.0280	156627	-	5289	180812	NCU05347	Os10g28230.1	8126	GSPATP00011487001	28445	181052	-	PFC0920w	737128	RO3G_16041.1	YOL012C	SPBC11B10.10c	GLEAN3_24890	SINFRUP00000139912	11.m00370	268117	TA14155	145.m00002	35819	-	Tb927.7.6360	UM00469.1
COX5b	Cytochrome c oxidase subunit IV, 13 kD	-	-	-	-	-	-	-	-	-	-	-	-	187882	-	-	-	-	-	-	-	-	-	-	OTTHUMP00000161109	-	-	-	-	116927	-	-	30323	-	-	-	-	-	-	-	-	-	-	SINFRUP00000135634	-	-	-	-	-	-	-	-
		-	-	-	-	-	-	-	-	-	-	-	-	187881	-	-	-	-	-	-	-	-	-	-	-	-	-	-	-	-	-	-	-	-	-	-	-	-	-	-	-	-	-	-	-	-	-	-	-	-	-	-
[truncated: 1,192,532 more chars]
