# Supplementary material for: Conservation of ciliary proteins in plants with no cilia
Source: BMC Plant Biol. 2011 Dec 30;11:185. doi: 10.1186/1471-2229-11-185 (PMC3268115; doi:10.1186/1471-2229-11-185)
Supplement: Additional file 4 — Protein information and sequence identification for CCP proteins. [file 1471-2229-11-185-S4.PDF]

Additional file 4 - CCP proteins

Short	Description	In proteomes	Apime	Arath	Auran	Batde	Caeel	Capsp	Chlre	Cioin	Crypa	Cyame	Danre	Dicdi	Drome	Enccu	Enthi	Galga	Giala	Homsa	Leima	Lotgi	Monbr	Naegr	Nemve	Neucr	Orysa	Ostta	Parte	Phatr	Phypa	Physo	Plafa	Poptr	Rhior	Sacce	Schpo	Strpu	Takru	Tetth	Thaps	Thean	Toxgo	Triad	Triva	Trybr	Ustma
CCP1	Putative Katanin p60 catalytic subunit	-	-	AT2G34560.2	25901	-	-	183389	98650	289753	-	-	OTTDARP00000023144	-	-	-	-	ENSGALP00000002864	-	XP_001129888.1	LmjF13.0960	208512	33720	36870	120426	-	Os01g55260.1	-	GSPATP00012036001	-	209093	127339	-	280297	-	-	-	-	-	45.m00146	38770	-	-	54346	86141.m00115	Tb11.02.1370	-
	Metallocarboxypeptidase/ zinc ion binding	-	GB11727-PA	AT5G42320.1	-	BDEG_05427	CE31658	227948	177379	226733	-	-	OTTDARP00000022757	-	CG18417-PA	-	-	ENSGALP00000013044	-	OTTHUMP00000024880	-	159841	23753	-	87987	-	Os02g02710.1	11914	GSPATP00032829001	-	44208	-	-	818859	-	-	-	GLEAN3_08959	SINFRUP00000155189	44.m00202	-	-	-	59794	-	-	-
WNK2	Hypothetical protein	-	GB16910-PB	AT5G41990.1	26387	BDEG_03110	CE37332	-	162667	253799	-	-	OTTDARP00000011134	-	CG7177-PA	-	-	ENSGALP00000004717	-	OTTHUMP00000181227	-	52941	32672	31865	-	-	Os05g01780.1	18914	-	-	1987	136369	-	252319	-	-	-	GLEAN3_22200	-	75.m00159	35929	-	-	4059	-	-	-
PF15	Putative Katanin p80 subunit	-	GB14488-PA	AT5G23430.2	33836	BDEG_03581	-	177482	80954	294910	-	-	-	-	CG13956-PA	-	-	ENSGALP00000039652	-	OTTHUMP00000080849	-	191659	22808	72175	133468	-	Os04g58130.1	-	GSPATP00011153001	-	21908	158533	-	803047	-	-	-	GLEAN3_14392	SINFRUP00000182752	62.m00215	-	-	-	63553	-	-	-
HMGB3407	ARID domain containing HMGB protein	-	GB19258-PA	AT1G76110.1	-	BDEG_02606	CE31068	64285	187927	267606	-	-	OTTDARP00000018864	-	CG5403-PB	-	-	ENSGALP00000002095	-	NP_689854.2	-	131492	-	53708	42971	-	Os09g37250.1	-	GSPATP00004674001	-	51717	-	-	206566	-	-	-	GLEAN3_25601	SINFRUP00000152561	21.m00216	-	-	-	54817	-	-	-
BUG22	Basal body protein of unknown function	+	GB13434-PA	AT3G12300.1	27836	BDEG_03225	CE33367	19508	32173	297236	-	-	-	-	CG5343-PA	-	-	ENSGALP00000001318	GL50803_104866	OTTHUMP00000164726	LmjF21.0380	191567	39112	81674	190658	-	Os09g33570.1	-	GSPATP00016658001	-	168952	109615	PFE0415w	712694	-	-	-	-	SINFRUP00000165463	45.m00266	1564	-	583.m09163	23937	94489.m00084	Tb10.70.5560	-
CCP2	Predicted ubiquitin-fold modifier 1 precursor	-	GB10591-PA	AT1G77710.1	-	-	CE00449	156758	195751	298942	cgd1_2790	-	OTTDARP00000020061	-	-	-	-	ENSGALP00000035687	-	OTTHUMP00000018797	LmjF16.1065	123041	-	60149	87714	-	Os01g73140.1	-	GSPATP00024294001	-	170305	124155	-	816297	-	-	-	-	-	16.m00326	-	-	583.m05409	63410	-	Tb927.8.5380	-
FASS1	FASS1/ tonneau 2 protein phosphatase type 2B	-	GB10687-PA	AT5G18580.1	31049	BDEG_05703	-	161182	116578	203725	cgd6_4380	-	-	-	-	-	-	ENSGALP00000016315	GL50803_17157	OTTHUMP00000027933	LmjF29.1930	110627	1300	64738	161077	-	Os05g05710.1	-	GSPATP00007128001	-	89210	-	-	811180	-	-	-	-	SINFRUP00000139586	86.m00165	-	-	-	57146	81869.m00184	Tb927.3.4470	-
FUSED	Hypothetical protein	-	GB10754-PA	AT1G50240.2	5353	-	-	150354	104702	-	-	-	OTTDARP00000018054	DDB0189678	CG6551-PA	-	-	ENSGALP00000018527	GL50803_17368	OTTHUMP00000164097	LmjF13.0440	153051	29411	34462	136852	-	Os12g24550.1	21800	GSPATP00004229001	-	125145	-	-	780405	-	-	-	GLEAN3_08747	SINFRUP00000169870	337.m00012	-	-	-	28806	96252.m00300	Tb11.02.2050	-
FAP267	Flagellar Associated Protein	-	GB17456-PA	AT1G77550.1	70560	BDEG_04764	CE38330	134404	100760	218738	-	-	-	-	-	-	-	ENSGALP00000010418	-	NP_001008409.1	LmjF18.0370	214448	1395	-	243308	-	Os03g08140.1	3214	GSPATP00030537001	-	127213	131864	PFE0700c	780940	-	YBR094W	-	GLEAN3_00277	SINFRUP00000140522	128.m00077	-	-	65.m01201	23726	97035.m00024	Tb10.61.3050	-
IBR1	Indole-3-butyric acid response 1	-	GB11191-PA	AT4G05530.1	29097	-	CE19894	157637	137062	237803	-	-	-	-	CG10672-PA	-	-	ENSGALP00000022323	GL50803_14626	OTTHUMP00000164690	-	211528	28844	-	130952	NCU03693	Os09g04730.1	-	GSPATP00018029001	-	206369	-	-	727523	-	-	-	GLEAN3_01934	SINFRUP00000181310	1.m00808	-	-	-	37528	90569.m00146	-	-
GPX3	Glutathione peroxidase, possibly mitochondrial	-	GB18955-PA	AT2G25080.1	7667	-	CE17231	-	137012	-	-	-	OTTDARP00000023112	-	CG12013-PD	-	-	ENSGALP00000022024	-	NP_001034937.1	-	208098	27890	30206	93209	NCU09534	Os04g46960.2	17179	GSPATP00004297001	-	186759	133117	PFL0595c	709364	-	-	-	-	SINFRUP00000136183	-	-	-	57.m00002	9015	88600.m00006	-	-
	Hypothetical protein	-	GB10046-PA	AT3G51040.3	15834	-	CE40258	157271	129827	-	-	-	-	-	CG8372-PB	-	3.m00628	ENSGALP00000001407	GL50803_3636	NP_115501.2	LmjF27.0900	164035	-	74306	179278	-	Os03g58520.1	-	GSPATP00014839001	-	118562	108582	PFE0240w	655385	-	-	-	-	SINFRUP00000145525	126.m00143	38651	-	-	57530	86827.m00376	Tb10.70.1250	-
	Hypothetical protein	-	GB14371-PA	AT3G54020.1	72578	-	CE04642	148915	127014	252549	-	-	OTTDARP00000024158	-	CG32380-PB	-	-	ENSGALP00000005856	-	OTTHUMP00000035396	LmjF35.4990	221916	-	48624	184324	-	Os05g21180.2	-	-	-	143973	144078	-	735668	-	-	-	-	SINFRUP00000164184	-	23224	TA10795	-	51176	81379.m00081	Tb09.211.1000	-
	Hypothetical protein	-	GB17418-PA	AT2G47600.1	28918	-	CE23275	128764	192884	270154	-	-	OTTDARP00000018696	-	CG17167-PB	-	-	ENSGALP00000032666	-	OTTHUMP00000042072	-	92127	29670	-	91794	-	Os11g43860.1	35614	-	17199	140132	128640	-	245784	-	-	-	GLEAN3_26183	SINFRUP00000155044	-	4973	-	-	53617	-	-	-
	Hypothetical protein	-	GB11967-PA	AT2G27900.2	32360	-	CE37754	184136	188334	270083	-	-	OTTDARP00000015719	DDB0186641	CG4996-PA	-	-	ENSGALP00000037320	-	NP_060137.2	-	140484	32036	77728	100869	-	Os10g39910.1	19111	-	-	153798	157082	-	199564	-	-	-	-	SINFRUP00000182806	-	22308	-	-	54017	83711.m00120	-	-
FKB62	Peptidyl-prolyl cis-trans isomerase, FKBP-type	-	GB13770-PA	AT3G25230.1	36393	-	CE15929	173491	34008	294630	-	CMH207C	OTTDARP00000001824	-	CG4535-PA	-	-	ENSGALP00000029868	-	OTTHUMP00000166201	LmjF19.1530	102242	21434	78715	183679	-	Os08g41390.2	29158	-	-	134160	121523	-	711545	-	-	-	GLEAN3_20251	SINFRUP00000129987	-	-	-	-	51478	-	Tb10.61.0180	-
MTP5	CDF transporter, membrane protein	-	GB15962-PA	AT1G51610.1	-	BDEG_05873	CE40222	183911	149470	286905	-	-	-	-	CG8632-PB	-	-	ENSGALP00000022999	-	OTTHUMP00000125233	-	165600	9689	56908	207889	-	Os04g23180.1	14643	GSPATP00027394001	13994	138991	134440	-	228460	-	-	-	-	SINFRUP00000132093	-	7978	-	-	25311	-	-	-
CCP3	Flagellar Associated Protein	-	GB17012-PA	AT2G25240.1	-	-	CE37003	20380	149241	387818	-	-	OTTDARP00000005067	-	CG9453-PG	-	232.m00066	ENSGALP00000015400	GL50803_4653	OTTHUMP00000173474	-	195168	-	74653	186693	-	Os01g16200.1	-	-	-	117874	-	-	677784	-	-	-	GLEAN3_13377	SINFRUP00000137160	70.m00159	264633	-	50.m03103	63332	-	-	-
HAC3401	CREB-binding protein	-	GB30193-PB	AT1G79000.1	38626	-	CE21117	224799	145462	223735	-	-	OTTDARP00000011858	-	CG15319-PB	-	-	ENSGALP00000019537	-	OTTHUMP00000096019	-	136183	9670	81728	106064	-	Os02g04490.1	16654	-	45764	10222	143063	-	828090	-	-	-	GLEAN3_19024	SINFRUP00000173359	-	24331	-	-	60973	-	-	-
TEF18	Glutathione S-transferase-related protein	-	GB17749-PA	AT5G42150.1	31067	-	CE06303	228714	142756	-	-	CMM110C	OTTDARP00000020909	-	CG4086-PA	-	-	ENSGALP00000007619	-	NP_945176.1	LmjF14.1480	180368	8222	29414	29018	-	Os04g17050.1	31220	-	-	45591	130578	-	569734	-	-	-	-	SINFRUP00000148451	-	6816	-	540.m00198	63654	-	Tb927.7.3500	-
