# Supplementary material for: Conservation of ciliary proteins in plants with no cilia
Source: BMC Plant Biol. 2011 Dec 30;11:185. doi: 10.1186/1471-2229-11-185 (PMC3268115; doi:10.1186/1471-2229-11-185)
Supplement: Additional file 5 — mRNA Expression data for Arabidopsis thaliana mined from and used in analysis of CCP protein functions. [file 1471-2229-11-185-S5.PDF]

Additional file - 5 mRNA expression

Name	CCP1 AT2G34560	BUG22 AT3G12300	CCP2 AT1G77710	AT3G51040	AT5G18580	AT1G50240	AT1G77550	AT3G25230	AT5G42150	AT5G42320
**Mature pollen Wt 6 wk**	1.5	-0.354772504	0.184097684	0.339796949	0.59067857	0.43544521	-0.079602372	0.011407434	0	0.066634115
**Whole plant seedling, green parts Wt 21 days**	0.254442101	-0.020273872	-0.14424177	-0.014118842	0.083063894	0.259084094	0.112413897	0.271890457	0.117265142	0.009368662
**Floral sepals Wt 21+ days**	0.21788315	-0.04564254	-0.141865942	0.131081643	0.155051254	0.280064663	0.039672135	0	-0.325712605	0.124117667
**Cauline leaves Wt 21+ days**	0.688919272	-0.147306794	-0.115452741	-0.036253811	0.070599005	0.485597368	0.12729997	0.367215481	0.094200384	0.032879939
**Seeds**	0.071721382	-0.182105853	0.000629843	-0.036369071	0.088190467	0	0.246678671	-0.020055405	0.190349493	0.377063161
**Flowers stage 15 Wt 21+ days**	0.128014688	0.036250237	-0.152675875	0.065574825	0.073271586	0.019673008	0	0.152532104	-0.070080617	0.152722492
**Stem, 2nd internode Wt 21+ days**	0.139200655	-0.06160213	-0.160341048	-0.179519472	-0.116450378	0.20531329	0.246610233	0.25344302	0.147482817	0
**Flowers stage 12 Wt 21+ days**	-0.133907022	0.135172927	0.067289402	0.079464092	-0.072893283	-0.515412282	-0.209928182	-0.015263037	-0.096117134	-0.063930581
**Stem, 1st node Wt 21+ days**	0	0.197887445	-0.207881348	-0.048191185	-0.138557271	-0.216740667	0.017573577	0.086610538	0.052873754	-0.080725878
**Siliques w/ seeds**	-0.042597457	0.064706937	-0.056589116	0	-0.081948025	-0.534448749	-0.088597474	0.001942757	0.089499915	0.047742455
**Floral pedicels Wt 21+ days**	-0.088783305	0.013075839	0.120004153	0.03248957	0	-0.243512988	0.029861424	0.067232414	0.06513208	-0.035256845
**Floral stamens Wt 21+ days**	0.189408407	-0.007131279	0.048604463	-0.004490789	0.138332905	0.073770616	0.243197921	-0.047323086	0.063043437	-0.354854879
**Root Wt 21 days**	-0.030312225	0.017349956	-0.202388985	-0.164708024	0.049814475	-0.030624248	-0.144872169	-0.070751871	-0.252425406	-0.088431236
**Floral petals Wt 21+ days**	-0.315557693	0.015614565	0.042080959	-0.02785464	-0.114425421	0.10069771	-0.033482198	-0.501570365	-0.158998699	-0.268994267
**Floral carpels Wt 21+ days**	-0.190479814	0	0	0.021841811	-0.195821926	-0.802089002	-0.447818749	-0.561858882	-0.200012842	0.008990603
**Whole plant, vegetative rosette Wt 21 days**	-0.071000013	-0.024131817	0.113194172	0.055346286	-0.031782535	-0.82417966	-0.511640227	-0.135726402	-0.171533321	-0.075787384
**Inflorescence (after bolting) Wt 21 days**	-0.114543727	0.108685126	0.126459589	0.097325836	-0.251779739	-1.162676194	-0.615282642	-0.36326016	-0.277528913	-0.134866849

AT5G41990	AT5G23430	AT1G76110	AT4G05530	AT2G25080	AT2G47600	AT2G27900	AT1G51610	CCP3 AT2G25240	AT1G79000
-0.936441108	0.01897472	-0.774629685	0	1.218274159	-0.593790299	0.376175692	1.098487179	-0.262456635	-0.081828154
0	0.174532439	-0.185213102	0.352682464	-0.547705958	0.276766701	0.05422427	0.073618435	0	-0.063365376
0.000148075	0.177685551	0	-0.088872413	-0.522904812	-0.001412383	0	-0.111403996	0.009331557	0.068052821
0.082946025	0.367617912	-0.352479314	-0.157281781	-0.482297253	-0.024870786	-0.097586829	0.040004431	-0.010118863	-0.183838814
-0.191073443	0.170773859	0.397565251	-0.021374545	0.192998019	0.021921381	0.176572331	-0.04858767	-0.139949459	0.025727021
-0.158646474	0.172565891	0.264609807	-0.056660479	-0.088142998	-0.060894057	-0.018663658	0.049696829	0.027275595	-0.016704801
-0.156487907	-0.200749544	-0.08453618	0.073527548	-0.045426165	-0.158506924	-0.111313177	0.084319309	-0.008700352	-0.116717405
0.045044044	-0.015182709	0.527881326	-0.003245522	0	-0.11217557	0.066465289	0	0.021430359	0.079432113
-0.220958082	-0.074282084	0.331970658	0.128210532	0.222048355	-0.070823961	-0.117732609	-0.204545935	0.021125885	0
0.022301759	0.124976835	-0.124353275	0.011451699	-0.183594907	0	0.267078629	0.006019862	0.020473253	0.279351843
0.127645511	-0.02854326	-0.264507996	0.180412061	-0.172533025	0.031635656	-0.157815519	-0.193448445	-0.028718425	0.053873357
-0.475093339	0.071068249	0.062419081	-0.217658361	0.663981251	-0.775777067	-0.025603209	0.232174509	-0.006911756	0.016670094
-0.425274417	-0.074012678	1.1363566	0.04927903	0.506404225	0.167586101	0.056442935	0.223340098	-0.560034692	-0.081467356
-0.006291268	-0.175988815	1.104496458	-0.093154197	0.20214092	0.166538479	-0.102862096	-0.057597088	0.010861597	-0.130652499
0.160157197	-0.105224516	0.803150554	-0.072368041	0.445851594	0.285651757	-0.080085796	-0.031296369	0.018395179	0.04228917
0.35866609	0	-0.34940623	0.209295227	-0.014386102	0.232291157	0.061797257	-0.20143657	-0.034589237	0.033778966
0.107975846	-0.268708959	-0.009250767	0.030748889	0.478808924	0.248023872	0.039579062	-0.10668079	0.019028917	-0.03373907

Name	Werewolf AT5G14750	TMM AT1G80080	LFY AT5G61850	RSL4 AT1G27740	UBC1 AT5G19180	FT AT1G65480	Tapetum1 AT3G42960
**Mature pollen Wt 6 wk**	-0.793777001	0	-0.211742952	-0.619545341	0.280537723	0	-0.250992627
**Whole plant seedling, green parts Wt 21 days**	0.036315445	-0.010667225	-0.018937316	0.035104697	0.007151207	-0.189994978	-0.174602264
**Floral sepals Wt 21+ days**	0.041813984	0.349283209	0.009882933	0.006415213	-0.046147863	-1.152413996	0.013106364
**Cauline leaves Wt 21+ days**	0	0.404155306	0	0	0.052883883	-1.377088071	-0.00068192
**Seeds**	-0.508377222	-0.823865739	-0.039857743	-0.169965969	0.285889063	-0.541320692	-0.097463478
**Flowers stage 15 Wt 21+ days**	0.024771376	-0.34076171	0.024498225	0.061562708	0.056217181	-1.201067752	0.04924607
**Stem, 2nd internode Wt 21+ days**	-0.243987122	0.48375225	0.000589034	0.02319447	0.117297537	0.277986505	0
**Flowers stage 12 Wt 21+ days**	0.147097756	-0.538185897	-0.013503071	0.075188899	-0.073195459	-0.037661099	0.02065815
**Stem, 1st node Wt 21+ days**	0.075720135	0.574802135	0.019431373	0.029648345	0.14118075	0.380795999	0.029792977
**Siliques w/ seeds**	-0.540416142	-0.378184127	0.025010602	0.02629161	0.070379392	-1.5	-0.031581272
**Floral pedicels Wt 21+ days**	-0.081245329	0.279358056	-0.015505109	0.027202609	0	-0.248923482	-0.025073849
**Floral stamens Wt 21+ days**	0.001176929	0.560410497	-1.25615456	-0.016857502	-0.029683483	0.412808183	0.043834879
**Root Wt 21 days**	-0.67905258	0.587678039	0.017884947	-0.677483499	-0.096558824	0.545562678	0.015192558
**Floral petals Wt 21+ days**	0.02278928	0.508510156	0.010817321	-0.087131155	-0.084043933	0.3594779	-0.024835529
**Floral carpels Wt 21+ days**	-0.062303509	-0.962476994	0.018539714	-0.020178512	-0.155153097	0.249925187	0.017386116
**Whole plant, vegetative rosette Wt 21 days**	-0.001581792	-1.234766537	-0.024090988	-0.044666523	-0.110330328	0.431680444	-0.008525926
**Inflorescence (after bolting) Wt 21 days**	0.011211904	-0.829167337	-1.5	-0.013677285	-0.238354207	0.394866542	0.002616155
